# Study title: A systematic review of RCTs to examine the risk of adverse cardiovascular events with nicotine use

**DOI:** 10.3389/fcvm.2023.1111673

**Published:** 2023-03-21

**Authors:** Mimi M. Kim, Isabella Steffensen, Red Thaddeus D. Miguel, Tanja Babic, Aubrey D. Johnson, Julien Carlone, Ryan Potts, Christopher S. Junker

**Affiliations:** ^1^RAI Services Company, Reynolds American Inc., Winston-Salem, NC, United States; ^2^Thera-Business, Kanata, ON, Canada; ^3^BAT, London, United Kingdom

**Keywords:** systematic review and meta-analysis, nicotine, adverse event (AE), cardiovascular, PRISMA (Preferred Reporting Items for Systematic Reviews and Meta-Analysis)

## Abstract

Associations between cigarette smoking and increased risk of cardiovascular disease are well established. However, it is unclear whether the association is mediated by exposure to nicotine and/or to other constituents in cigarette smoke. The objective of this systematic review and meta-analysis of randomized control trials (RCTs) was to identify any potential associations between exposure to nicotine and the risk of clinically diagnosed adverse cardiovascular events in adult current users and nonusers of tobacco products. Among 1,996 results, 42 studies, comparing nicotine and non-nicotine groups, were included and were both qualitatively and quantitatively synthesized across the outcomes of arrhythmia, nonfatal myocardial infarction, nonfatal stroke, and cardiovascular death. The majority of studies evaluating nonfatal myocardial infarction, nonfatal stroke, and cardiovascular death reported no events that occurred in either the nicotine or non-nicotine control groups. Among the studies that reported events, rates of adverse events were similarly low between both groups. Consistent with findings from previous systematic reviews and meta-analyses, pooled data showed that rates for arrhythmia, nonfatal myocardial infarction, nonfatal stroke, and cardiovascular death were not significantly different between nicotine and non-nicotine groups. The overall quality of the body of evidence for each of the four outcomes of interest was graded as “moderate,” limited only by the imprecision of results. The findings of this systematic review and meta-analysis indicate that, with moderate certainty, there are no significant associations between the use of nicotine and the risk of clinically diagnosed adverse cardiovascular events—specifically, arrhythmia, nonfatal myocardial infarction, nonfatal stroke, and cardiovascular death.

## Introduction

Cardiovascular disease (CVD) is a leading cause of death and morbidity worldwide (1–3). According to the Global Burden of Disease study, CVD accounted for 18.6 million deaths worldwide (1, 4) in 2019, as well as 359 million years of life lost (YLLs), 34.4 million years lived with disability (YLDs), and 393 million disability-adjusted life years (DALYs) (5). Data between 2010 and 2019 indicate an increase in the prevalence and burden of CVD, driven in large part by population growth and an aging population (4, 5).

An association between cigarette smoking and increased risk of CVD is well-established, but it is not clear whether this association is mediated by exposure to nicotine and/or to other constituents found in cigarette smoke (6). Both laboratory and clinical studies in humans have shown that nicotine stimulates the sympathetic nervous system, resulting in transient increases in heart rate and blood pressure, as well as changes in other cardiovascular parameters (7–13). However, there is evidence to suggest that these effects are more pronounced in response to exposure to components related to the use of combustible tobacco products than to nicotine alone (6, 10, 14). Further, there is evidence to suggest that reductions in heart rate occur when cigarette smokers quit, even when abstinence is maintained through the use of nicotine replacement therapy (NRT) (15, 16). Collectively, the evidence suggests that although nicotine administration alone may result in acute increases in blood pressure, heart rate, and biomarkers of cardiovascular risk in humans, these increases appear to be lower than those associated with combustible tobacco product use. Further, it is unclear whether the transient cardiovascular effects observed with nicotine administration alone can lead to an increased risk of adverse cardiovascular events.

### Adverse cardiovascular events and cardiovascular disease

CVD is not a single disease, but rather a collection of afflictions of the cardiovascular system (17). Concurrently, the development of CVD is multifactorial and can involve dynamic etiologies with complex progressions. As such, the vast determinants of the disease allow for a vast number of key metrics to be measured in studying CVD. A few examples include behaviors that are associated with an increased risk of CVD, changes in cardiovascular parameters and disease progression through the serial monitoring of laboratory and imaging metrics. However, not only are these metrics not always feasible to carry out in a clinical trial, but they also fail to directly inform actual disease outcomes. Thus, CVD clinical trials often investigate the safety and efficacy impact of interventions using clinical outcomes. Specifically, clinical endpoints of cardiovascular mortality, myocardial infarction, and stroke, have been suggested by the FDA as endpoints in evaluating therapies’ impact on CVD (18).

### The current evidence base

Two systematic reviews and/or meta-analyses have examined the potential association between nicotine exposure and the incidence of serious adverse cardiovascular events. The first by Lee et al. (19) found that compared with non-nicotine controls, no significant effect of NRT use was observed for the risk of acute myocardial infarction or cardiovascular mortality. The second, a network meta-analysis by Mills et al. (20), found a statistically significant association for the risk of any cardiovascular adverse event (AE) between NRT and both bupropion and placebo, but not varenicline. However, symptoms such as pounding heart and heart palpitations were included as outcomes, which on their own have unknown clinical significance and call into question the relevance of the results. When limiting analyses to the risk of major adverse cardiovascular events (MACE), the authors found no significant associations with NRT.

## Objectives

The objective of this systematic review and meta-analysis of RCTs was to identify any potential associations between the exposure of nicotine (compared to no nicotine exposure) and the risk of clinically diagnosed adverse cardiovascular events in a sample of adult current users and nonusers of tobacco products at baseline. The outcomes of interest evaluated separately were arrhythmia, nonfatal myocardial infarction, nonfatal stroke, and cardiovascular death. The exposure of nicotine referred to nicotine *via* tobacco leaf free oral nicotine (e.g., portioned oral nicotine pouches) or NRT products (e.g., nicotine patch).

## Methods

### Overview

This review’s protocol was registered with the PROSPERO international prospective register of systematic reviews on July 2, 2021 (PROSPERO 2021 CRD42021258686) and assesses the Key Question (KQ): “Is there an association between nicotine and the risk of adverse clinically diagnosed cardiovascular events in adult current users and nonusers of tobacco?”

This review adhered to standards of systematic review methodology as defined by the Preferred Reporting Items for Systematic Reviews and Meta-Analyses (PRISMA) guidelines (21) and the AMSTAR 2 (A MeaSurement Tool to Assess systematic Reviews 2) critical appraisal tool (22).

Meta-analyses using a random effects model and applying the inverse variance method (23) were conducted to calculate the risk ratios (RR) with a 95% confidence interval (CI). Heterogeneity was assessed using I^2^ statistic and interpreted based on the I^2^ thresholds suggested by The Cochrane Collaboration (24). Where possible, a funnel plot was developed to test for the risk of publication bias (25). A subgroup analysis for each of the four outcomes by the duration of nicotine exposure was conducted. Additionally, a subgroup analysis of arrhythmia by type of arrhythmia was conducted. Two sensitivity analyses were planned *a priori* to detect whether pooled results were sensitive to the removal of (1) studies judged to be at high risk of bias, and (2) studies that did not report the collection of AE data as an outcome of interest in either their protocol or methodology. Data were analyzed through Review Manager Version 5.3 (26).

## Literature search

**Figure 1:**
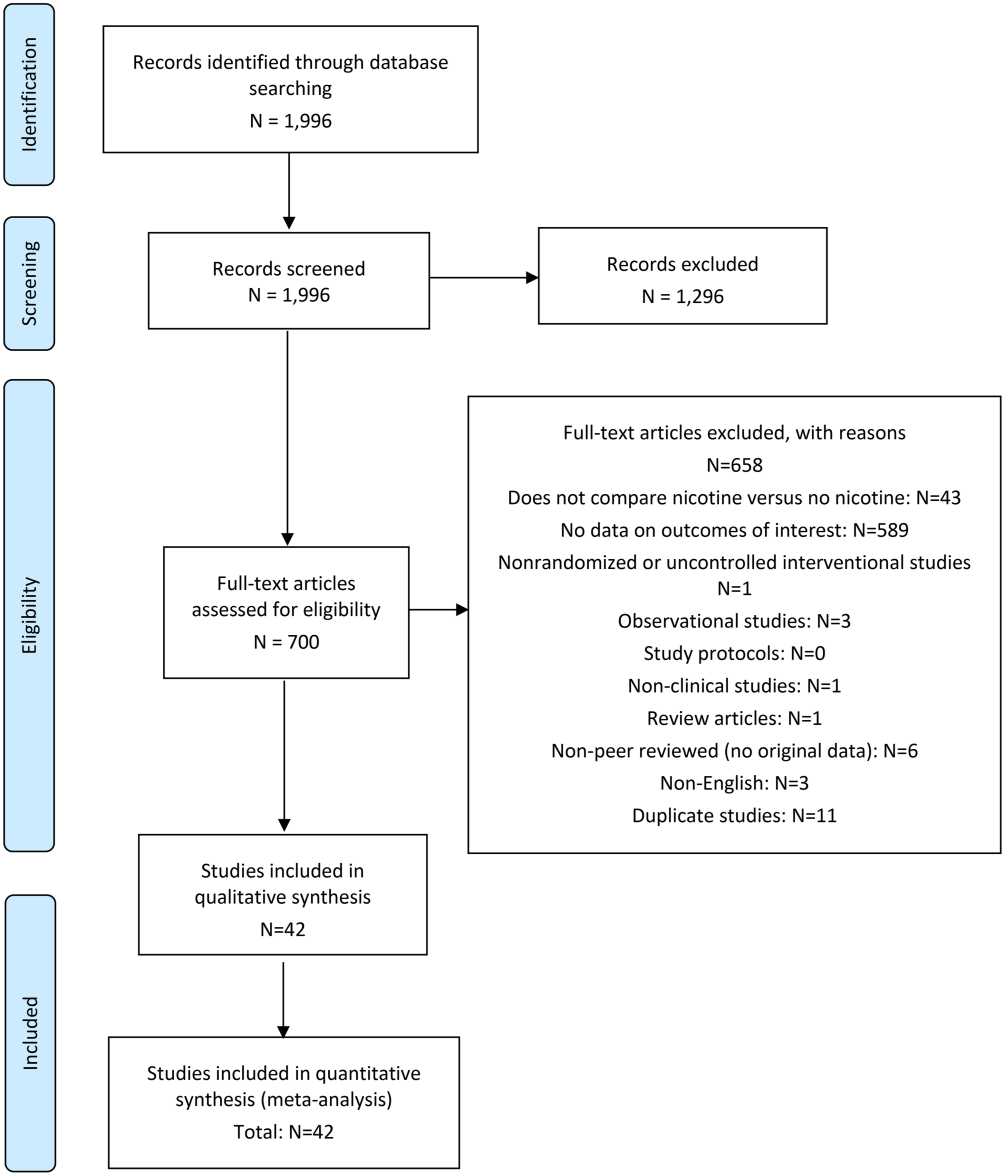
PRISMA flow diagram.

The literature search was conducted by an information specialist who has credentials as a health sciences librarian and is qualified in conducting systematic literature searches. Search terms were developed using keywords associated with tobacco leaf free nicotine or NRT products nicotine and their various modes of administration. The search strategy included the use of synonyms of search terms, truncation, wild card symbols, Boolean logic, proximity operators, and limits, in order to focus the search on the most relevant clinical literature. Results are presented shown in Figure 1.

The following online databases were searched for relevant articles published from inception to 10 June 2021: PUBMED/MEDLINE, Embase, and Cochrane Database of Systematic Reviews (included as part of the Embase search). The full literature search strategy can be found in Appendix A.

Other methods used for identifying relevant research included: a grey literature search; searching of bibliographies of included studies and relevant published systematic reviews and meta-analyses; searching of trial registries; and contacting experts in the field.

## Eligibility criteria

The PICOS (**P**opulation or participants and conditions of interest, **I**nterventions or exposures, **C**omparisons or control groups, **O**utcomes of interest, and **S**tudy designs) review method was used, as it is an objective, non-biased, systematic review method. The following inclusion criteria were applied:

**Table:** 

**P**opulation or participants and conditions of interest	Adults who are current users or nonusers (includes never or former users) of tobacco products
**I**nterventions or exposures	Nicotine
**C**omparisons or control groups	No nicotine
**O**utcomes of interest	Clinical diagnoses of an adverse cardiovascular event (i.e., arrhythmia, nonfatal myocardial infarction, nonfatal stroke, cardiovascular death) during the treatment period.
**S**tudy designs	Randomized controlled trials (RCTs)

The intervention was tobacco leaf free oral nicotine (also referred to as modern oral products) or NRT products, hereafter referred to as “nicotine.” Consequently, the intervention was not nicotine administered in the form of other tobacco products, such as cigarettes, electronic cigarettes, or smokeless tobacco. The effects of nicotine and poly- or dual-use with tobacco products or switching between nicotine and tobacco products, were outside of the scope of this systematic review and meta-analysis. Additionally, in order to be included, the nicotine intervention had to be actively prescribed, not simply offered or recommended. For example, studies evaluating tobacco cessation interventions that included the option of NRT products were not included.

Control groups were required to have a regimen that did not administer nicotine in any form. For example, studies whose placebo arm included products with low levels of nicotine were excluded. Further, as the criteria for the control was strictly “no nicotine,” this allowed for comparator groups of other active treatments to be included (e.g., varenicline or bupropion).

Outcomes were required to be clinical diagnoses of either arrhythmia, nonfatal myocardial infarction, nonfatal stroke, or cardiovascular death. Consistent with the accepted definition of serious AEs (27), studies reporting that no serious AEs occurred were considered to have had no occurrence of nonfatal myocardial infarction, nonfatal stroke, or cardiovascular death. To meet this review’s inclusion criteria, outcomes were also required to have occurred during the treatment period, i.e., an adverse cardiovascular event that occurred during a non-treatment follow-up period of a study was not considered an outcome for this systematic review. Where the temporality of the occurrence of outcomes was unclear, authors were contacted for additional detail; if the temporality of outcomes could not be confirmed, they were excluded.

## Exclusion criteria

The following studies were excluded from the systematic review:

1. RCTs that did not include nicotine versus no nicotine intervention.
2. RCTs that did not provide data on an outcome of interest (i.e., arrhythmia, nonfatal myocardial infarction, nonfatal stroke, cardiovascular death) during the treatment period.
3. Non-randomized controlled or uncontrolled interventional studies.
4. Observational studies (e.g., cohort, case–control, and cross-sectional studies).
5. Registered protocols.
6. Non-clinical, non-human studies, such as laboratory research, animal studies, or *in vitro* or *ex vivo* studies.
7. Review articles, systematic reviews, and meta-analyses.
8. Letters to the editors, opinions, editorials, press releases, manufacturers’ advertisements, and other non-peer reviewed publications, unless the publication contained original data from RCTs.
9. Articles in which the abstract and full text were non-English.
10. Duplicate articles or articles with the exact same study outcome data as another published article.

## Review methods

### Study selection process

Articles were initially screened at the title/abstract level. Full text articles were obtained for any articles that could not be excluded based on the title/abstract alone. Each article was independently screened by two reviewers, according to the inclusion criteria. Any discrepancies regarding studies that were included/excluded by reviewers were discussed and resolved in a meeting between reviewers, and a joint decision was made on whether the article should be included or excluded. Any disagreements that could not be resolved between the reviewers were decided by a third clinical reviewer at Thera-Business Inc. Reasons for excluding an article were documented.

### Data extraction

Data were independently extracted by one research associate from Thera-Business Inc. and checked by a second research associate from Thera-Business Inc. Discrepancies were identified and resolved through discussion and included a third team member, when necessary. Data extraction forms were hosted on DistillerSR®. For each study, information regarding the study characteristics and outcomes—as defined in the PICOS—were extracted. The extracted information included: location and setting; trial name; registration/protocol; funding; study design; eligibility criteria; recruitment strategy; intervention(s) and control(s); study start/end date; study population and subject characteristics; and study outcomes. Supplementary materials and clinical trial registries, where applicable, were reviewed for relevant data and extracted accordingly. As required, study authors were contacted for clarification pertaining to the data extracted.

### Risk of bias assessment

The included studies were individually assessed for risk of bias using the Cochrane ‘Risk of Bias’ tool, evaluating for risks of bias from the following sources: randomized sequence generation (selection bias); allocation concealment (selection bias); blinding of participants and personnel (performance bias); blinding of outcome assessment (detection bias); incomplete outcome data (attrition bias); selective reporting (reporting bias); and other bias (28). Each of these potential sources of bias were graded as either “low,” “high,” or “unclear” risk; subsequently, an overall risk of bias grade was given for each study.

Risk of bias assessments were independently performed by two research associates from Thera-Business Inc.; any discrepancies were discussed and resolved in a meeting between reviewers, and a joint decision was made. Any disagreements that could not be resolved between the reviewers were decided by a third clinical reviewer at Thera-Business Inc. Overall risk of bias was determined for each study as follows: “low” overall risk of bias if the study was judged to be at “low” risk across all domains evaluated; “high” overall risk of bias if the study was rated as “high” risk in at least one domain; and “unclear” overall risk if at least one bias domain was assessed as “unclear” risk, and no bias domains were assessed as “high” risk.

### Strength of evidence

To grade the confidence in the overall conclusions for each outcome, a systematic, objective, and transparent assessment of the overall strength of evidence (SOE) was performed (29). Following the standards of Cochrane methodology, this assessment was performed using the Grading of Recommendations Assessment, Development, and Evaluation (GRADE) system of rating quality of evidence; GRADE is among the most widely adopted tools for grading the quality of evidence and strength of recommendations in systematic reviews (30).

The GRADE system begins by assessing RCTs as high-quality evidence; thereafter, the SOE evaluation is assessed using five domains: (1) study limitations, (2) consistency of effect, (3) imprecision, (4) indirectness, and (5) publication bias. Depending on the assessment, the study’s quality of evidence for each outcome may be downgraded. Ultimately, the quality of evidence falls into one of four categories from high to very low (see Table 1).

**Table 1:** Strength of evidence grades and interpretations (31).

Grade	Interpretation
High	We are very confident that the true effect lies close to that of the estimate of the effect.
Moderate	We are moderately confident in the effect estimate: the true effect is likely to be close to the estimate of the effect, but there is a possibility that it is substantially different.
Low	Our confidence in the effect estimate is limited: the true effect may be substantially different from the estimate of the effect.
Very low	We have very little confidence in the effect estimate: the true effect is likely to be substantially different from the estimate of the effect.

The SOE evaluation was independently performed by one research associate from Thera-Business Inc. and checked by a second research associate from Thera-Business Inc. The final SOE judgment was necessarily qualitative but reflected a sound, reasoned weighing of domain ratings.

## Meta-analysis

Where studies had used the same intervention and comparator, with the same outcome measure, the results were pooled using a random effects meta-analysis. The inverse variance method (23) was used to calculate the RR with a 95% CI. Outcomes measured were the presence or absence of an event occurring, therefore, continuous data were not used in the analysis. For missing data, a conservative approach was taken by utilizing the randomized sample. Where indicated that the randomized sample was different from the sample that initiated their allocated intervention or control, the latter sample was prioritized in order to increase the certainty regarding the association between allocation and outcome.

A subgroup analysis for the outcome of arrhythmia by type of arrhythmia was conducted; there were no possible sub-group analyses by types of nonfatal myocardial infarction, nonfatal stroke, and cardiovascular death. Further, a subgroup analysis for the each of the four outcomes by duration of nicotine exposure was conducted, grouping studies with a duration of nicotine treatment of 12 weeks or more and of less than 12 weeks.

Where 10 or more studies provided estimates pooled in the meta-analysis, a funnel plot was developed to test for the risk of publication bias (25). Heterogeneity was assessed using I^2^ statistic, and the level of heterogeneity was interpreted based on the I^2^ thresholds suggested by The Cochrane Collaboration (24):

- 0% to 40%: may not be important;
- 30% to 60%: may represent moderate heterogeneity;
- 50% to 90%: may represent substantial heterogeneity;
- 75% to 100%: considerable heterogeneity.

Results were presented through forest plots developed using RevMan version 5.3. The main analyses were presented alphabetically, while sensitivity analyses were presented by descending magnitude to organize the studies with estimated effect sizes together.

## Sensitivity analyses

Two sensitivity analyses were planned prior to the implementation of the meta-analyses and were conducted for all outcomes. Specifically, sensitivity analyses were conducted to detect whether pooled results were sensitive to the removal of (1) studies judged to be at high risk of bias, and (2) studies that did not report systematically collecting AE data, defined as studies that did not report the collection of AE data as an outcome of interest in either their protocol or methodology.

## Protocol deviations

An update to the protocol was approved by the sponsor on August 18, 2021, and registered with PROSPERO on October 13, 2021, reflecting the following changes:

- The review question and objectives were amended to reflect the inclusion of both current users and nonusers of tobacco, as defined in the PICOS. Originally, the review question and objectives did not reflect users of tobacco products.
- The exclusion criteria were expanded to reflect the comprehensive list of exclusion criteria required during screening. Specifically, two criteria were added to the protocol: “RCTs that do not include a nicotine versus no nicotine intervention,” and “Protocols.”

## Results

A total of 1,996 articles were retrieved from the specified databases, of which 1,954 were excluded based on pre-defined criteria included in the registered protocol—resulting in 42 studies eligible for inclusion in the review. All 42 studies were included in both the qualitative and quantitative syntheses of evidence: 11 studies for occurrence of arrhythmia; 32 studies for occurrence of nonfatal myocardial infarction; 29 studies for occurrence of nonfatal stroke; and 33 studies for occurrence of cardiovascular death.

### Characteristics of included studies

Appendix E contains the complete study and sample characteristics for each of the included studies.

#### Study designs

All 42 included studies were RCTs, the majority of which (26 studies) were two-armed trials (32–57). Seven included studies were three-armed trials, each consisting of one nicotine intervention group and two non-nicotine control groups (58–64). A further seven studies were four-armed trials: four studies had two nicotine intervention groups and two non-nicotine control groups (65–68); two studies had one nicotine intervention group and three non-nicotine control groups (69, 70); and one study had three nicotine intervention groups and one non-nicotine control group (71). One study was a five-armed trial, with four nicotine intervention groups and one non-nicotine control group (72). Finally, one study had a crossover design, such that all subjects received both a nicotine intervention and a non-nicotine control, with a washout period of more than 7 days between treatments (73).

#### Study evaluations

The evaluations of interest in two-thirds of the included studies (*n* = 28) were related to cigarette smoking abstinence (32, 35–38, 40, 43–48, 51–53, 56–64, 66–68, 72); a further two studies evaluated smokeless tobacco abstinence as their primary outcome (34, 71). Five studies were safety analyses whose primary outcomes of interest related to the safety of various NRTs (33, 39, 42, 55, 69). Two studies evaluated the efficacy of nicotine as a treatment for ulcerative colitis as their primary outcome (49, 54). The primary outcomes of interest of the remaining five studies were as follows: one study evaluated cigarette smoking relapse (65); one study evaluated cigarette cravings (50); one study evaluated the efficacy of nicotine chewing gum in the prevention of postoperative ileus after colorectal surgery (41); one study evaluated the effects of nicotine on rectal sensation, rectal compliance, and anorectal sphincter function (73); and one study used nicotine as a cholinergic agonist in subjects with schizotypy to assist with assessing the utility of biomarkers (70).

#### Outcomes measures

With regards to outcome measures reported among the 42 included studies, 11 studies reported data on arrhythmia (33, 39, 49, 54, 56, 60, 61, 64, 66, 69, 73), 32 studies reported data on nonfatal myocardial infarction (32–41, 43–48, 50, 51, 53, 54, 56, 60–63, 65, 67–72), 29 studies reported data on nonfatal stroke (32–34, 36–38, 40, 42–47, 50, 51, 53, 54, 56, 58, 60–63, 65, 67–71), and 33 studies reported data on cardiovascular death (32–38, 40–47, 50–59, 61, 62, 65, 67–71) (see Table 2).

**Table 2:** Number of studies according to outcome measures.

Measure	Number of studies	List of studies
Arrhythmia	11	Benowitz et al., 2018; de Jong et al., 2018; Joseph et al., 1996; Kavin and Shivey, 1995; Lerman et al., 2015; Lewis et al., 1998; Sandborn et al., 1997; Shiffman et al., 2009; Thomas et al., 1995; Uyar et al., 2007; Wallström et al., 2000
Nonfatal myocardial infarction	32	Aubin et al., 2008; Benowitz et al., 2018; Covey et al., 2007; de Jong et al., 2018; Ebbert et al., 2013; Ebbert et al., 2007; Foulds et al., 1993; Gilbert et al., 2020; Hughes et al., 2003; Joseph et al., 1996; Koychev et al., 2011; Kralikova et al., 2009; Lambrichts et al., 2017; Lerman et al., 2015; Lewis et al., 1998; Myung et al., 2007; Oncken et al., 2014; The Preloading Investigators, 2018; Ramon et al., 2014; Rohsenow et al., 2017; Rungruanghiranya et al., 2008; Rungruanghiranya et al., 2012; Sachs et al., 1993; Shiffman and Ferguson, 2008; Shiffman et al., 2006; Stein et al., 2013; Sun et al., 2009; Thomas et al., 1995; Tønnesen et al., 1999; Tuisku et al., 2016; Wallström et al., 2000; Xiao et al., 2020
Nonfatal stroke	29	Aubin et al., 2008; Benowitz et al., 2018; Chen et al., 2020; Covey et al., 2007; de Jong et al., 2018; Ebbert et al., 2013; Ebbert et al., 2007; Gilbert et al., 2020; Hughes et al., 2003; Koychev et al., 2011; Kralikova et al., 2009; Lerman et al., 2015; Lewis et al., 1998; Møller et al., 2002; Myung et al., 2007; Oncken et al., 2014; The Preloading Investigators, 2018; Ramon et al., 2014; Rohsenow et al., 2017; Rungruanghiranya et al., 2008; Rungruanghiranya et al., 2012; Shiffman and Ferguson, 2008; Shiffman et al., 2006; Stein et al., 2013; Sun et al., 2009; Thomas et al., 1995; Tuisku et al., 2016; Wallström et al., 2000; Xiao et al., 2020
Cardiovascular death	33	Aubin et al., 2008; Benowitz et al., 2018; Chen et al., 2020; Covey et al., 2007; de Jong et al., 2018; Ebbert et al., 2013; Ebbert et al., 2007; Etter et al., 2002; Foulds et al., 1993; Gilbert et al., 2020; Hays et al., 1999; Hughes et al., 2003; Koychev et al., 2011; Kralikova et al., 2009; Lambrichts et al., 2017; Lewis et al., 1998; Møller et al., 2002; Myung et al., 2007; Oncken et al., 2014; The Preloading Investigators, 2018; Ramon et al., 2014; Rohsenow et al., 2017; Rungruanghiranya et al., 2008; Rungruanghiranya et al., 2012; Shiffman and Ferguson, 2008; Shiffman et al., 2006; Stapleton and Sutherland, 2011; Sun et al., 2009; Thomas et al., 1995; Thomsen et al., 2010; Tuisku et al., 2016; Wallström et al., 2000; Xiao et al., 2020

#### Publication dates

**Figure 2:**
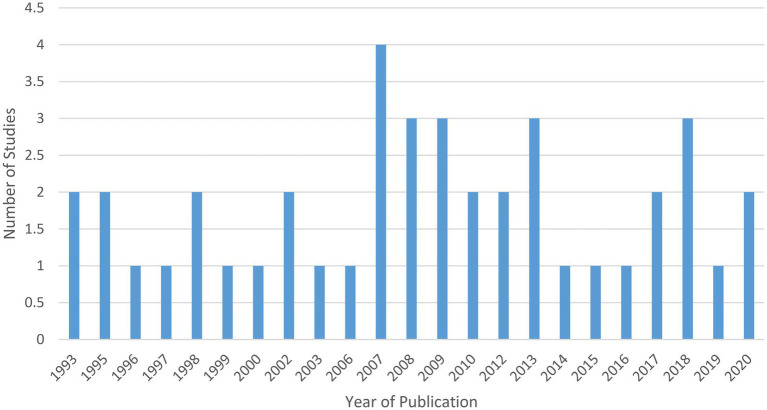
Included studies by publication year.

Of the 42 included studies: nine studies were published between 2016 and 2020 (33, 36, 38, 41, 45, 58, 67–69); seven studies were published between 2011 and 2015 (34, 44, 47, 60, 62, 63, 70); 13 studies were published between 2006 and 2010 (32, 40, 43, 46, 50–53, 55, 64–66, 71); three were published between 2001 and 2005 (37, 42, 59); and 10 were published in 2000 or earlier (35, 39, 48, 49, 54, 56, 57, 61, 72, 73) (see Figure 2).

#### Study locations

The highest proportion of studies were conducted in North America (*n* = 19), with the rest of the studies coming from Europe (*n* = 15), Asia (*n* = 5), Eurasia (*n* = 1), or multiple regions (*n* = 2).

**Figure 3:**
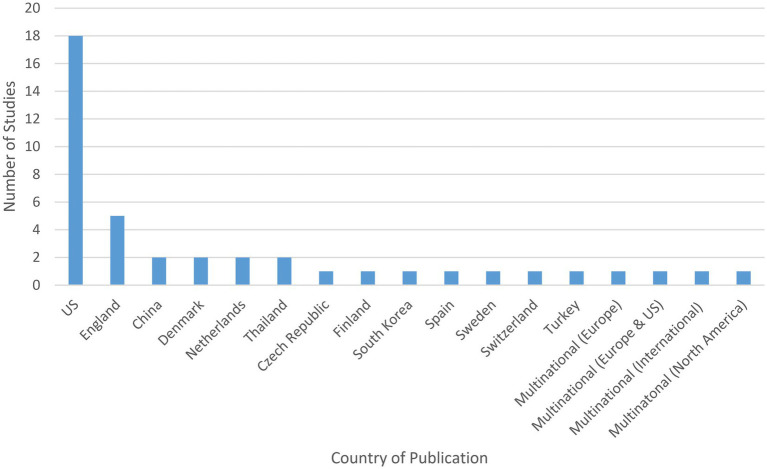
Study site of included studies.

According to country, the highest proportion of studies were conducted in the United States (US) (*n* = 18) (34, 36, 37, 39, 45, 48–51, 57, 58, 61–63, 65, 66, 71, 73), followed by England (*n* = 5) (35, 38, 52, 54, 70) (see Figure 3). Other countries included: China (*n* = 2) (53, 68); Denmark (*n* = 2) (42, 55); Netherlands (*n* = 2) (33, 41); Thailand (*n* = 2) (46, 47); Czech Republic (*n* = 1) (40); Finland (*n* = 1) (67); South Korea (*n* = 1) (43); Spain (*n* = 1) (44); Sweden (*n* = 1) (56); Switzerland (*n* = 1) (59); and Turkey (*n* = 1) (64). Four studies were multinational, one of which was conducted in Europe (Austria, Belgium, Denmark, Finland, France, Germany, Greece, Ireland, Italy, Netherlands, Norway, Portugal, Spain, Sweden, Switzerland, and UK) (72), one of which was conducted in Europe and the US (Belgium, France, Netherlands, UK, and US) (32), one of which was conducted internationally (Argentina, Australia, Brazil, Bulgaria, Canada, Chile, Denmark, Finland, Germany, Mexico, New Zealand, Russian Federation, Slovakia, South Africa, Spain, and US) (69), and one of which was conducted in North America (Canada and US) (60).

#### Study sample sizes

Total sample sizes among the included studies ranged from 11 subjects (73) to 8,058 subjects (69), for a total of 27,794 subjects included across all 42 studies. Of these, a total of 12,545 subjects were allocated to a nicotine trial arm, and 15,260 subjects were allocated to a non-nicotine trial arm. Eleven subjects from the one included crossover study received both a nicotine intervention and a non-nicotine control, and as such are reflected in both aforementioned counts (73).

#### Study population

Table 3 presents a summary of study population characteristics, to include general health status and tobacco use characteristics among the study populations, where applicable.

**Table 3:** Study population health and tobacco use characteristics among the included studies.

Study	Study population health status	Study population tobacco use status at baseline	Tobacco abstinence rates during the study
Aubin et al., 2008	Unspecified	Current cigarette smokers	*Continuous abstinence (eCO ≤ 10 ppm) for the last 4 weeks of treatment*Nicotine patch group: 43.2% (*n* = 160)Varenicline group: 55.9% (*n* = 210)
			Benowitz et al., 2018	Unspecified	Current cigarette smokers	*Continuous abstinence rates from Weeks 9 to 12*Nicotine patch group: 23.4%Varenicline group: 33.5%Bupropion group: 22.6%Placebo group: 12.5%
						Chen et al., 2020	Unspecified	Current cigarette smokers	*7-day PPA at end of treatment period*NRT group: 20.0%Varenicline group: 25.5%Placebo group: 8.8%
Covey et al., 2007	Unspecified	Current cigarette smokers							NR
de Jong et al., 2018	Critically ill, mechanically-ventilated subjects	Current cigarette smokers							NR
Ebbert et al., 2013	Subjects “were in good general health”	Current smokeless tobacco users							*7-day PPA at the end of treatment (confirmed by urinary anabasine < 2 ng/ml)*Nicotine patch group: 44% (*n* = 11)Placebo patch group: 22% (*n* = 6)Prolonged abstinence*Nicotine patch group: 44% (*n* = 11)Placebo patch group: 22% (*n* = 6)*Defined as meeting criteria for 7-day PPA and also reporting no tobacco use for 7 consecutive days, nor at least once each week, on 2 consecutive weeks, since 2 weeks following their target quit date.
			Ebbert et al., 2007	Subjects “in good general health”	Current smokeless tobacco users	*Continuous tobacco abstinence at Week 8*21 mg nicotine patch group: 40% (*n* = 4)42 mg nicotine patch group: 55% (*n* = 6)63 mg nicotine patch group: 70% (*n* = 7)Placebo group: 45% (*n* = 5)*Self-reported 7-day PPA at Week 8*21 mg nicotine patch group: 40% (*n* = 4)42 mg nicotine patch group: 73% (*n* = 8)63 mg nicotine patch group: 70% (*n* = 7)Placebo group: 73% (*n* = 8)
						Etter et al., 2002	Unspecified	Current cigarette smokers	*Prevalence of past 7-day abstinence (no puff of tobacco) at end of treatment period*Nicotine group: 5.3%Placebo group: 2.2%Control group: 4.1%*Prevalence of past 4-week abstinence (no puff of tobacco) at end of treatment period*Nicotine group: 4.2%Placebo group: 1.9%Control group: 3.9%
									Foulds et al., 1993	General hospital outpatients	Current cigarette smokers	*Continuous abstinence (eCO < 10 ppm) from Week 6 to 12*Nicotine patch group: 13.7% (*n* = 17)Placebo patch group: 10.5% (*n* = 13)
												Gilbert et al., 2020	Unspecified	Non-tobacco and light tobacco smokers	eCO concentration*Nicotine patch group: <5 ppmPlacebo patch group: <5 ppm*Exact eCO values NR; however, no subjects in either group reported an eCO of ≥5 ppm throughout the treatment period.
															Hays et al., 1999	Unspecified	Current cigarette smokers	*Prevalence of biochemically-verified 7-day PPA at Week 6*Nicotine (blind) group: 16.8%Nicotine (open-label) group: 19.0%Placebo group: 9.6%
									Hughes et al., 2003	History of alcohol dependence	Current cigarette smokers							*Adjusted prolonged smoking abstinence rates (eCO < 10 ppm on all visits after the first 2 weeks) at Week 16*Nicotine patch group: 28% (95% CI 15–46)Placebo patch group: 11% (95% CI 4–24)
																		Joseph et al., 1996	History of ≥1 CVD	Current cigarette smokers	*Continuous 8-week abstinence (eCO ≤ 10 ppm) at Week 14 (%)*Nicotine group: 21%Placebo group: 9%
																					Kavin and Shivey, 1995	Healthy subjects	Former cigarette smokers (quit cigarette smoking ≥1 month prior to study)	NA (former cigarette smokers)
Koychev et al., 2011	Healthy subjects scoring high or average on a schizotypal personality measure	Nonsmokers							NA (nonsmokers)											
Kralikova et al., 2009	Unspecified	Current cigarette smokers							*Sustained abstinence rate (not a single cigarette smoked and eCO < 10 ppm at each visit)*From Week 6 to Month 4:NRT group: 20.1% (*n* = 42)Placebo group: 8.6% (*n* = 9)From Month 6 to Month 12:NRT group: 18.7% (*n* = 39)Placebo group: 8.6% (*n* = 9)
						Lambrichts et al., 2017	Subjects who underwent colorectal surgery	Unspecified	NR
			Lerman et al., 2015	Unspecified	Current cigarette smokers	*Rate of 7-day PPA (eCO ≤ 8 ppm) at end of treatment period*“Varenicline was more efficacious than nicotine patch in normal metabolizers (OR 2.17, 95% CI 1.38–3.42; *p* = 0·001), but not in slow metabolizers (OR 1.13, 0.74–1.71; p = 0·56).”
						Lewis et al., 1998	Hospitalized subjects	Current cigarette smokers	*Self-reported 7-day PPA at end of treatment period*Nicotine patch group: 30.6% (*n* = 19)Placebo patch group: 17.7% (*n* = 11)Minimal care group: NR
			Møller et al., 2002	Subjects scheduled for primary elective hip or knee alloplasty	Current cigarette smokers				NR
			Myung et al., 2007	“In good health”	Current cigarette smokers				*Self-reported point prevalence at Month 3*Nicotine patch group: 23.7% (*n* = 14)Placebo group: 16.9% (*n* = 10)
									Oncken et al., 2014	Medically stable (i.e., no serious or unstable medical or psychiatric condition for at least 6 months)	Current cigarette smokers	*Abstinence rate (eCO ≤ 10 ppm) during the last 4 weeks of treatment*Nicotine patch + topiramate group: 37% (*n* = 7)Placebo tablet group: 5% (*n* = 1)Topiramate group: 26% (*n* = 5)
						The Preloading Investigators, 2018	Unspecified	Current cigarette smokers				NA (subjects continued to smoke cigarettes during treatment period, i.e., preloading period)
Ramon et al., 2014	Unspecified	Current cigarette smokers	*Continuous abstinence from Week 2-Week 12 with eCO concentrations of < 10 ppm at 12 weeks*Nicotine patch group: 39.1% (*n* = 66)Placebo patch group: 31.8% (*n* = 54)								
			Rohsenow et al., 2017	Substance use disorder	Current cigarette smokers	*Confirmed 7-day smoking abstinence (eCO ≤ 4 ppm and salivary cotinine ≤ 15 ng/ml) at 3 months*Nicotine patch group: 3% (*n* = 2)Varenicline group: 13% (*n* = 10)					
						Rungruanghiranya et al., 2008	Unspecified	Current cigarette smokers	*Total abstinence (eCO ≤ 10 ppm) at end-of-treatment*Nicotine gum group: 50.0% (*n* = 10)Placebo gum group: 9.0% (*n* = 2)
Rungruanghiranya et al., 2012	Unspecified	Current cigarette smokers							*Continuous abstinence rate (eCO ≤ 10 ppm) from Week 9 to 12*Nicotine gum group: 66.0% (*n* = 35)Fresh lime group: 61.7% (*n* = 29)
			Sachs et al., 1993	Healthy smokers	Current cigarette smokers				*Continuous abstinence (eCO ≤ 9 ppm at each visit) Week 2 to 12*Nicotine patch group: 45% (*n* = 51)Placebo patch group: 26% (*n* = 28)
						Sandborn et al., 1997	Ulcerative colitis	Nonsmokers or former cigarette smokers	NA (nonsmokers or former cigarette smokers)
Shiffman and Ferguson, 2008	Unspecified	Current cigarette smokers				NR
Shiffman et al., 2009	Unspecified	Current cigarette smokers	*Continuous abstinence (not even a puff) up to Day 28*Nicotine (2 mg and 4 mg combined) group: 10.3%Control (2 mg and 4 mg placebo combined) group: 3.9%
			Shiffman et al., 2006	Subjects “In good health”	Current cigarette smokers	*7-day PPA at Week 5*Nicotine patch group: 52% (*n* = 98)Placebo patch group: 26% (*n* = 35)
						Stapleton and Sutherland, 2011	Subjects “in good general health”	Current cigarette smokers	*Abstinence (eCO < 10 ppm) from Week 3 to 12*Nicotine nasal spray group: 15.4% (*n* = 78)Placebo group: 6.7% (*n* = 17)
Stein et al., 2013	Methadone-maintained (opiate-dependent) subjects	Current cigarette smokers							*7-day PPA (eCO < 8 ppm) immediately prior to Month 6 assessment*NRT group: 8.3% (*n* = 11)Varenicline group: 3.7% (*n* = 5)Varenicline-placebo group: 2.2% (*n* = 1)*Continuous abstinence from day 14 through the 6-month assessment*			
			NRT group: 1.5% (*n* = 2)Varenicline group: 1.5% (*n* = 2)Varenicline-placebo group: 0.0% (*n* = 0)					
			Sun et al., 2009	Unspecified	Current cigarette smokers	*Self-reported abstinence at end-of-treatment period*Nicotine tablet group: 52.0%Placebo tablet group: 19.0%
						Thomas et al., 1995	Subjects with ulcerative colitis	Nonsmokers or former cigarette smokers	NA (nonsmokers or former cigarette smokers)
						Thomsen et al., 2010	Subjects with breast cancer	Current cigarette smokers	*Continuous abstinence from 2 days before to 10 days after surgery*NRT group: 28.0% (*n* = 16)Control group: 11.0% (*n* = 7)
			Tønnesen et al., 1999	Healthy cigarette smokers	Current cigarette smokers				*Continuous abstinence (eCO < 10 ppm) at Week 8*Nicotine (25 mg long duration) group: 36.9% (*n* = 264)Nicotine (25 mg short duration) group: 40.8% (*n* = 292)Nicotine (15 mg long duration) group: 32.5% (*n* = 232)Nicotine (15 mg short duration) group: 29.2% (*n* = 209)Placebo group: 20.7% (*n* = 148)
									Tuisku et al., 2016	Unspecified	Current cigarette smokers	*Self-reported abstinence (not having smoked for about 1 week) at Week 4*Total: 33.0% (*n* = 96)10 mg nicotine patch group: 26.6% (*n* = 25)15 mg nicotine patch group: 19.6% (*n* = 10)Placebo group: 19.8% (*n* = 17)Varenicline group: 73.3% (*n* = 44)*Self-reported abstinence at Week 12*Total: 23.0% (*n* = 67)10 mg nicotine patch group: 23.4% (*n* = 22)15 mg nicotine patch group: 15.7% (*n* = 8)Placebo group: 17.4% (*n* = 15)Varenicline group: 36.7% (*n* = 22)
						Uyar et al., 2007	Unspecified	Current cigarette smokers				*Abstinence rate (measure NR) at Week 4*Nicotine patch group: 36.0%Bupropion group:46.0%No treatment group: 22.5%*Abstinence rate at Week 8*Nicotine patch group: 28.0%Bupropion group: 38.0%No treatment group: 16.1%
												Wallström et al., 2000	Healthy cigarette smokers	Current cigarette smokers	*Continuous abstinence (eCO < 10 ppm) from Week 2 to Month 6*Nicotine group: 33%Placebo group: 18%
Xiao et al., 2020	Unspecified	Current cigarette smokers													*28-day continuous abstinence (eCO ≤ 10 ppm) at Week 6*2 mg nicotine lozenge group: 24.48% (*n* = 59)4 mg nicotine lozenge group: 30.83% (*n* = 37)2 mg placebo lozenge group: 21.49% (*n* = 52)4 mg placebo group: 20.17% (*n* = 24)*7-day PPA rate at Week 24*2 mg nicotine lozenge group: 41.5%2 mg placebo group: 43.1%4 mg nicotine lozenge group: 50.0%4 mg placebo group: 43.7%			

CI, confidence interval; CVD, cardiovascular disease; eCO, expired/exhaled carbon monoxide; mg, milligrams; NA, not applicable; ng/ml, nanograms per milliliter; NR, not reported; NRT, nicotine replacement therapy; OR, odds ratio; PPA, point prevalence abstinence; ppm, parts per million.

#### Health status

Ten included studies obtained their samples from a population of volunteers with an indication of good general health (34, 43, 48, 51, 52, 56, 62, 71–73), while 13 included studies had populations with varying medical conditions or an indication of adverse general health, including: breast cancer (55); history of one or more CVDs (39); critical illness (i.e., mechanically-ventilated subjects) (33); history of alcohol dependence (37); opiate dependency (receiving methadone-maintenance treatment) (63); undergoing primary elective hip or knee alloplasty (42); substance use disorder (45); ulcerative colitis (in two studies) (49, 54); recent colorectal surgery (41); hospitalization (61); subjects from a hospital outpatient clinic (35); and otherwise healthy volunteers scoring high or average on a schizotypal personality measure (70). Nineteen included studies did not specify or give any indication of the health of their study populations (32, 36, 38, 40, 44, 46, 47, 50, 53, 57–60, 64–69).

#### Tobacco use characteristics

Thirty-seven of the 42 included studies reported a study population of current tobacco product users. The vast majority of these studies (*n* = 34) were among current cigarette smokers (32, 33, 35, 37–40, 42–48, 50–53, 55–69, 72), while two studies were among current smokeless tobacco users (34, 71). One study included both nonsmokers and light cigarette smokers (36). Additionally, one included study did not specify tobacco use status in its study population (41).

Among the 37 studies reporting a study population of current tobacco product users, 32 reported tobacco abstinence as an outcome: 30 reported cigarette smoking abstinence (32, 35–37, 39, 40, 43–48, 51–53, 55–64, 66–69, 72), and two reported smokeless tobacco abstinence (34, 71). One additional study evaluated cigarette smoking abstinence among current cigarette smokers; however, because abstinence was only measured 4 weeks following the last nicotine exposure period, these abstinence data were not considered relevant to this systematic review.

Tobacco abstinence rates among studies were generally low, with only about one-fifth of studies (*n* = 6) reporting rates greater than 50% in any of their trial arms (32, 47, 51, 53, 67, 71). Of these, only one study reported abstinence rates greater than 50% across all trial arms (expired carbon monoxide [eCO]-verified continuous abstinence in final 3 weeks of treatment: nicotine gum group, 66%; fresh lime group, 62%) (47). More than a quarter of studies (*n* = 9) reported rates of less than 25% across all trial arms (35, 39, 40, 45, 52, 57, 59, 63, 66).

Among the different nicotine intervention groups, the highest rate of tobacco abstinence was 73% 7-day point prevalence abstinence (PPA) at the end of the 8-week treatment period for subjects using smokeless tobacco (71). The highest rate of cigarette smoking abstinence was 66% eCO-verified continuous abstinence in the final 3 weeks of treatment (47). The lowest rate of tobacco abstinence was 1.5% continuous cigarette smoking abstinence from Day 14 through to the end of the 6-month treatment period (63).

Among the placebo groups, the highest rate of tobacco abstinence was 73% 7-day PPA at the end of the 8-week treatment period for subjects using smokeless tobacco (71). The highest rate of cigarette smoking abstinence was 44% 7-day PPA at the end of the 24-week treatment period (68). The lowest abstinence rate was 0% continuous cigarette smoking abstinence from Day 14 through to the end of the 6-month treatment period (63).

Among control groups receiving varenicline, the rate of tobacco abstinence was 73% self-reported cigarette smoking abstinence of “about 1 week” at Week 4 of an 8-week treatment (67). The lowest rate of tobacco abstinence was 1.5% continuous cigarette smoking abstinence from Day 14 through to the end of the 6-month treatment period (63). Among control groups receiving bupropion, the rate of tobacco abstinence was 46% cigarette smoking abstinence [measure not reported (NR)] at Week 4 of a 6-week treatment period (64). The lowest abstinence rate was 23% continuous cigarette smoking abstinence from Weeks 9 to 12 (i.e., to end of treatment) (69). The one study that included a control group receiving topiramate reported an eCO-confirmed cigarette smoking abstinence rate of 26% during the last 4 weeks of treatment (62).

Among control groups the highest rate of tobacco abstinence was 22% (measure NR) at Week 4 of a 6-week treatment period (64). The lowest rate of tobacco abstinence was 3.9% 4-week continuous abstinence at the end of treatment (59).

### Treatment regimens

#### Treatments evaluated

The most common evaluation across the 42 included studies was comparing nicotine patch with placebo patch, which was evaluated in 22 studies (52%) (33–39, 43–45, 48–51, 54, 57, 60, 61, 67, 69, 71, 72). This was followed by comparing nicotine gum with placebo gum, which was done in five studies (12%) (41, 46, 66, 68, 73). A full list of the evaluations conducted in the evidence base is provided in Table 4.

**Table 4:** Interventions evaluated among the included studies.

Interventions evaluated	Number of studies	List of studies
Nicotine patch versus placebo patch	22	Benowitz et al., 2018; de Jong et al., 2018; Ebbert et al., 2007; Ebbert et al., 2013; Foulds et al., 1993; Gilbert et al., 2020; Hays et al., 1999; Hughes et al., 2003; Joseph et al., 1996; Lerman et al., 2015; Lewis et al., 1998; Myung et al., 2007; The Preloading Investigators, 2018; Ramon et al., 2014; Rohsenow et al., 2017; Sachs et al., 1993; Sandborn et al., 1997; Shiffman and Ferguson, 2008; Shiffman et al., 2006; Thomas et al., 1995; Tønnesen et al., 1999; Tuisku et al., 2016
Nicotine gum versus placebo gum	5	Kavin and Shivey, 1995; Lambrichts et al., 2017; Rungruanghiranya et al., 2008; Shiffman et al., 2009; Xiao et al., 2020
Nicotine patch versus varenicline	4	Aubin et al., 2008; Benowitz et al., 2018; Lerman et al., 2015; Tuisku et al., 2016
NRT^a^ versus placebo NRT^a^	4	Chen et al., 2020; Etter et al., 2002; Kralikova et al., 2009; Stein et al., 2013
Nicotine patch versus bupropion	2	Benowitz et al., 2018; Uyar et al., 2007
Nicotine patch versus no treatment	2	Lewis et al., 1998; Uyar et al., 2007
Nicotine tablet versus placebo tablet	2	Sun et al., 2009; Wallström et al., 2000
NRT^a^ and counselling versus no treatment	2	Møller et al., 2002; Thomsen et al., 2010
NRT^a^ versus no treatment	1	Etter et al., 2002
Nicotine gum versus fresh lime	1	Rungruanghiranya et al., 2012
Nicotine gum versus bupropion^b^	1	Covey et al., 2007
Nicotine nasal spray versus placebo nasal spray	1	Stapleton and Sutherland, 2011
Nicotine patch versus amisulpride versus risperidone^c^	1	Koychev et al., 2011
Nicotine patch versus topiramate^d^	1	Oncken et al., 2014

NRT, nicotine replacement therapy.

a Any combination of two or more NRTs, to include patches, inhalers, gum, and/or lozenges.

b Covey et al. (2007) evaluated nicotine gum and bupropion across four arms: nicotine gum and bupropion (combined); nicotine gum; bupropion; and placebo.

c Koychev et al. (2011) evaluated nicotine patch, amisulpride, and risperidone across four arms: nicotine patch; amisulpride; risperidone; and placebo.

d Oncken et al. (2014) evaluated nicotine patch and topiramate across three arms: nicotine patch and topiramate (combined); topiramate; and placebo.

#### Duration of treatment periods

Duration of nicotine treatment period (the period in which nicotine was administered, irrespective of the overall treatment duration) among the included studies ranged from 1 hour to 6 months, with a median treatment duration of 10 weeks (see Table 5). Fifteen of the 42 included studies had a nicotine treatment duration of 12 weeks or longer (35, 40, 45–48, 52, 54, 56, 58, 59, 63, 65, 68, 72).

**Table 5:** Treatment protocol and adherence rates among included studies.

Study	Treatment period	Type and strength of intervention(s)	Type and strength of control(s)	Treatment adherence
Aubin et al., 2008	10 weeks (NRT); 12 weeks (Varenicline)	Nicotine patches (21 mg patch for 6 weeks, 14 mg patch for 2 weeks, 7 mg patch for 2 weeks).	Varenicline (0.5 mg once daily for 3 days, 0.5 mg twice daily for 4 days, and 1 mg varenicline twice daily for following 11 weeks)	NR
Benowitz et al., 2018	11 weeks (nicotine patch); 12 weeks (varenicline and bupropion)	21 mg nicotine patch daily with taper, placebo bupropion, and placebo varenicline. All three treatments used daily.	Varenicline group*: 1 mg varenicline twice daily, placebo bupropion, and placebo nicotine patch; Bupropion group*: 150 mg bupropion twice daily, placebo nicotine patch, and placebo varenicline; Placebo group*: Placebo nicotine patch, placebo varenicline, and placebo bupropion*All three treatments used daily	*Mean number of days of treatment exposure (assessed by patch/pill count) throughout treatment period*	NRT group: 73.7 ± 23.6 daysVarenicline group: 74.4 ± 23.1 daysBupropion group: 73.7 ± 23.8 daysPlacebo group: 73.6 ± 23.6 days
			Chen et al., 2020	13 weeks	Nicotine patches (21 mg patches for 8 weeks, 14 mg patches for 2 weeks, and 7 mg patches for 2 weeks) and 13 weeks’ worth of 2 mg or 4 mg nicotine lozenge for use as needed.	Varenicline group: 0.5 mg once daily for 3 days, 0.5 mg twice daily for 4 days, and 1 mg varenicline twice daily for following 11 weeks; Placebo group: placebo nicotine patches or placebo lozenges or placebo varenicline regimen	*Treatment adherence at end of treatment period*NRT group: 65% (95% CI, 61–69)Varenicline group: 66% (95% CI, 61–70)Placebo group: 62% (95% CI, 58–67)
							Covey et al., 2007	16 weeks^a^	Nicotine gum+bupropion group: 2 mg nicotine gum and bupropion (300 mg daily); Nicotine gum + placebo pill group: 2 mg nicotine gum and placebo pills	Placebo gum+bupropion group: placebo gum and bupropion pills (300 mg daily); Placebo gum+placebo pill group: Placebo gum and placebo pill	45% of subjects randomized to maintenance treatment used the nicotine or placebo gum; mean number of weeks of gum use was 6.5 ± 5.5 (rate per group NR).
							de Jong et al., 2018	Until ICU discharge, or 30 days postoperatively	14 mg nicotine patches; 21 mg nicotine patches.	Placebo patches	NR
							Ebbert et al., 2013	8 weeks^b^	Nicotine patches (two 21 mg patches daily for 6 weeks, one 21 mg patch daily for the following 2 weeks).	Placebo patches	*Median medication adherence (time point NR)*Nicotine patch group: 93%Placebo patch group: 61%
Ebbert et al., 2007	8 weeks	21 mg NRT group*: 21 mg patch and two placebo patches daily; 42 mg NRT group*: Two 21 mg patches and one placebo patch daily; 63 mg NRT group*: three 21 mg nicotine patches* Subjects received three patches on weeks 1 to 4, two on weeks 5 and 6, and one on weeks 7 and 8.	Three placebo patches. Subjects received three patches on weeks 1 to 4, two on weeks 5 and 6, and one on weeks 7 and 8.	NR						
		Etter et al., 2002			6 months	25 mg nicotine patch, 4 mg nicotine gum or 10 mg nicotine inhaler. Subjects could switch between products or use several products at the same time					Placebo group*: Placebo patches, placebo gums or placebo inhalers; Control group: NA	*Current use of treatment at Month 6*Nicotine group: 36.1% daily; 25.2% occasionally (non-daily); 32.9% never			
							*Subjects could switch between products or use several products at the same time	Placebo group: 25.3% daily; 30.4% occasionally, (non-daily); 44.3% neverControl group: 2.4% daily; 2.6% occasionally (non-daily); 95.0% never				
Foulds et al., 1993	12 weeks		Nicotine patches (30 cm^2^ patches containing 0.83 mg nicotine per cm^2^)	Placebo patches			*Patch use at Week 12*Nicotine patch group: 29%Placebo patch group: 16%
							Gilbert et al., 2020	15 days	7 mg nicotine patches	Placebo patches	NR
		Hays et al., 1999			6 weeks	Nicotine group: 22 mg patch once daily	Placebo patches	*Product dispensed (assessed as a surrogate for patch compliance)*Nicotine group: 4.8 ± 2.1 boxesPlacebo group: 5.1 ± 2.1 boxes
Hughes et al., 2003	10 weeks^c^		Nicotine patches (21 mg patch once daily for 6 weeks, 14 mg patch once daily for 2 weeks, 7 mg nicotine patch once daily for 2 weeks, and placebo patch for 2 weeks)	Placebo patches				During treatment period, 77% of subjects wore the patch on all days that they did not smoke (rate per group NR)
Joseph et al., 1996	10 weeks		Nicotine patches (21 mg patch for 6 weeks, 14 mg patch for 2 weeks, and 7 mg patch for 2 weeks)	Placebo patches				*Subjects wearing patches at Week 6*Nicotine patch group: 73%Placebo patch group: 56%
		Kavin and Shivey, 1995			1 h (two rounds of 30 min)	2 mg nicotine gum	Placebo (1 mg of biologically inactivated nicotine) gum	NR
		Koychev et al., 2011			4.5 h (tests performed 4.5 h after patch application; 1.5 h after capsule administration).	7 mg nicotine patch and placebo capsule	Amisulpride group: Placebo patch and 400 mg amisulpride capsule; Risperidone group: Placebo patch and 2 mg risperidone capsule; Placebo group: Placebo patch and placebo capsule	NR
Kralikova et al., 2009	6 months	10 mg nicotine inhaler or 4 mg nicotine gum	Placebo inhaler or placebo gum	*Daily patch use at Month 9* (i.e.*, after 6 months treatment plus 3 months voluntary tapering*)NRT group: 45%Placebo group: 39%
				Lambrichts et al., 2017	Until the first passage of feces and tolerance of solid food for >24 h	2 mg nicotine gum	Placebo gum	*Compliance at Postoperative Day 3*Nicotine gum group: 15% (*n* = 3) as per protocol; 65% (*n* = 13) less than protocol; 0% (*n* = 0) more than protocol; 20% (*n* = 4) NRPlacebo gum group: 15% (*n* = 3) as per protocol; 55% (*n* = 11) less than protocol; 0% (*n* = 0) more than protocol; 30% (*n* = 6) NR
								Lerman et al., 2015	11 weeks (nicotine patch); 12 weeks (varenicline)	Nicotine patches (21 mg patch for 6 weeks, 14 mg patch for 2 weeks, 7 mg patch for 3 weeks, and placebo pills for 12 weeks)	Placebo patch+varenicline: 0.5 mg varenicline once daily for 3 days, 0.5 mg varenicline twice daily for 4 days, and 1.0 mg varenicline for 11 weeks, and placebo patches; Placebo patch+placebo pill: Placebo patch and placebo pill	On average, 62% of subjects used ≥80% the pill dose recommended; 63% of subjects used ≥80% the patches recommended (rate per group NR; time point NR)
Lewis et al., 1998	6 weeks	Nicotine patches (22 mg patches for 3 weeks and 11 mg nicotine patches for 3 weeks)	Placebo patch group: Placebo patches for 6 weeks; Minimal care group: NA					*Treatment compliance rate at Week 1*Nicotine patch group: 70% (*n* = 40)Placebo group: 60% (*n* = 34)Minimal care group: NR
				Møller et al., 2002	6 to 8 weeks prior to surgery and 10 days after surgery	Unspecified nicotine dosage	NA	NR
				Myung et al., 2007	6 weeks	Nicotine patches (57 mg patch (delivered 21 mg) for 2 weeks, 38 mg patch (delivered 14 mg) for 2 weeks, and 19 mg patch (delivered 7 mg) for 2 weeks)	Placebo patches for 6 weeks	NR
				Oncken et al., 2014	8 weeks (nicotine patch); 10 weeks (topiramate)	Nicotine patches (21 mg patch for 7 weeks, 14 mg patch for 3 days, and 7 mg patch for 4 days) and topiramate (25 mg once daily for 1 week, 25 mg twice daily for 1 week, 50 mg twice daily for 1 week, 75 mg twice daily for 1 week, 100 mg twice daily for 5 weeks, and 30% reduction in dose every 3 days for 1 week)	Placebo tablet group: Placebo tablets; Topiramate group: 25 mg once daily for 1 week, 25 mg twice daily for 1 week, 50 mg twice daily for 1 week, 75 mg twice daily for 1 week, 100 mg twice daily for 5 weeks, and 30% reduction in dose every 3 days for 1 week	*Treatment adherence rate (based on pill counts at each treatment visit)*Nicotine patch + topiramate group: 93% ± 10.1%Placebo tablet group: 93.8% ± 8.0%Topiramate group: 89.4% ± 14.0%
The Preloading Investigators, 2018	4 weeks	21 mg nicotine patch	NA					“Three quarters of participants used the patch daily during the first week and four fifths did so in the subsequent weeks.” (rate per group NR)
Ramon et al., 2014	11 weeks (nicotine patch); 12 weeks (varenicline)	21 mg nicotine patch for 11 weeks and varenicline (0.5 mg daily for 3 days, 0.5 mg twice daily for 4 days, 1 mg twice daily for 11 weeks)	Placebo patches for 11 weeks and varenicline (0.5 mg daily for 3 days, 0.5 mg twice daily for 4 days, 1 mg twice daily for 11 weeks)					Nicotine patch group: One subject did not use the nicotine patch; one subject did not use varenicline between Weeks 4 and 12; three subjects did not use varenicline between Weeks 8 and 12.Placebo patch group: Two subjects did not use the placebo patch; two subjects did not use varenicline between Weeks 8 and 12.
								Rohsenow et al., 2017	12 weeks (nicotine patch); 13 weeks (varenicline)	Placebo capsules and nicotine patches (21 mg for 4 weeks, 14 mg for 4 weeks, and 7 mg for 4 weeks)	Placebo patches and varenicline (0.5 mg once daily for 3 days, 0.5 mg twice daily for 4 days, 1 mg varenicline twice daily for 11 weeks)	*Treatment products used during treatment period*Nicotine patch group: subjects used 43.3% ± 41.0% of nicotine patches and 42.4% ± 39.7% of placebo capsules.Varenicline group: Subjects used 40.0% ± 37.5% of placebo patches and 37.4% ± 35.1% of varenicline capsules.
Rungruanghiranya et al., 2008	12 weeks	2 mg nicotine gum; 4 mg nicotine gum	Placebo gum	NR							
Rungruanghiranya et al., 2012	12 weeks	2 mg nicotine gum; 4 mg nicotine gum	NA	NR							
Sachs et al., 1993	18 weeks	Nicotine patches (30 cm^2^ patches for 12 weeks, 20 cm^2^ patches for 3 weeks, 10 cm^2^ patches for 3 weeks)	Placebo patches	Nicotine patch group: 65% at Week 12 (end of full treatment); 60% at Week 18 (end of tapering)Placebo patch group: 54% at Week 12 (end of full treatment); 61% at Week 18 (end of tapering)
				Sandborn et al., 1997	4 weeks	Nicotine patches (11 mg patch for 7 days and 21 mg patch for 21 days)	Non-nicotine placebo patches	Subjects who wore patches as directed on at least 90% of the study days:Nicotine patch group: 97% (*n* = 30)Placebo patch group: 97% (*n* = 32)
Shiffman and Ferguson, 2008	2 weeks	21 mg nicotine patch	Placebo patches					NR
Shiffman et al., 2009	8 weeks^d^	2 mg nicotine gum; 4 mg nicotine gum	Placebo gum					NR
Shiffman et al., 2006	5 weeks	Nicotine patches (two patches (21 mg and 14 mg) for a total daily dose of 35 mg for 3 weeks, and one 21 mg patch and one placebo patch for 2 weeks)	Placebo patches	Patches were applied on 99.3% of days (rate per group NR)
Stapleton and Sutherland, 2011	12 weeks	1 mg single dose nicotine nasal spray; 28 mg daily maximum	Placebo nasal spray	NR
Stein et al., 2013	24 weeks	21 mg or 42 mg nicotine patch and 4 mg nicotine gum (4-week supply)	Varenicline (0.5 mg once daily for 3 days, 0.5 mg twice daily for 4 days, and 1 mg twice daily for 23 weeks)	*Adherence to treatment during 7 days immediately prior to Month 6 assessment*NRT group: 48.8%Varenicline group: 34.2%Varenicline-placebo group: 34.4%
				Sun et al., 2009	8 weeks^e^	2 mg nicotine tablets; 20 tablet daily maximum	3 μg capsaicin placebo tablets	NR
				Thomas et al., 1995	6 months	15 mg nicotine patch	Placebo patches	NR
				Thomsen et al., 2010	Perioperative period (3 to 7 days preoperative; 10 days postoperative)	NRT administered in accordance to preference and dependency	NA	NR
Tønnesen et al., 1999	26 weeks^f^	Nicotine (25 mg long duration) group: Nicotine patches (25 mg nicotine for 22 weeks, 15 mg patches for 2 weeks, and 10 mg patches for 2 weeks); Nicotine (25 mg short duration) group: Nicotine patches (25 mg nicotine patches for 8 weeks, 15 mg patches for 2 weeks, 10 mg patches for 2 weeks, and 0 mg patches for 14 weeks); Nicotine (15 mg long duration) group: Nicotine patches (15 mg nicotine patches for 22 weeks, 10 mg patches for 4 weeks); Nicotine (15 mg short duration) group: Nicotine patches (15 mg patches for 8 weeks, 10 mg patches for 4 weeks, and 0 mg patches for 14 weeks)	Placebo patches	*Subjects using patches daily at Week 26*Nicotine (25 mg long duration) group: 52% (*n* = 291)Nicotine (25 mg short duration) group: 47% (*n* = 290)Nicotine (15 mg long duration) group: 53% (*n* = 274)Nicotine (15 mg short duration) group:45% (*n* = 222)Placebo group: 51% (*n* = 186)
				Tuisku et al., 2016	8 weeks (nicotine patch); 12 weeks (varenicline)	10 mg nicotine patch; 15 mg nicotine patch	Placebo patch group: Placebo patches; Varenicline group: 0.5 mg for 3 days, 1 mg for 4 days; 2 mg for 12 weeks	*Percent of subjects who used treatment for over 2 weeks (percent who completed treatment)*10 mg nicotine patch group: 51.1% (21.3%)15 mg nicotine patch group: 37.3% (9.8%)Varenicline group: 76.7% (20.0%)Placebo group: 36.1% (10.5%)
								Uyar et al., 2007	6 weeks (nicotine patch); 3 days plus 6 weeks (bupropion)	Nicotine patches (21 mg patch for 2 weeks, 14 mg patch for 2 weeks, 7 mg patch for 2 weeks)	Bupropion (150 mg for the first 3 days, 300 mg for 6 weeks)	*Treatment compliance at Week 6*Nicotine patch group: 22%Bupropion group: 40%No treatment group: NR
												Wallström et al., 2000	6 months	4 mg nicotine sublingual tablets; 40 tablets daily maximum	3 μg capsaicin placebo tablets	*Rate of daily use of treatment at Week 6 (mean daily consumption), stratified by dependency level*Highly dependent group: nicotine group, 83% (22 ± 8 tablets); placebo group 77% (22 ± 9 tablets)Low dependent group: nicotine group, 89% (11 ± 4 tablets); placebo group, 70% (12 ± 6 tablets)
																Xiao et al., 2020	24 weeks	2 mg nicotine lozenge; 4 mg nicotine lozenge	Placebo lozenges	Safety analysis included subjects in good compliance with study protocol; three subjects were excluded from safety analysis due to non-use of study drugs (treatment group NR).

μg, microgram; CI, confidence interval; cm^2^, square centimeters; eCO, exhaled/expired carbon monoxide; hr(s), hours; ICU, intensive care unit; mg, milligrams; NA, not applicable; ng/ml = nanograms per milliliter; NR, not reported; NRT, nicotine replacement therapy; PPA, point prevalence abstinence; ppm, parts per million.

a Covey et al. (2007) was preceded by an open-label treatment, consisting of 7 weeks nicotine and 8 weeks bupropion; not considered in the analysis.

b Ebbert et al. (2013) administered nicotine patches during a 2-day of inpatient stay, prior to 8-week treatment period.

c Hughes et al. (2003) reported a 12-week treatment period; however, the final 2 weeks of treatment was a placebo patch in both groups, i.e., the effective treatment period for nicotine administration was considered as 10 weeks.

d Shiffman et al. (2009) administered nicotine gum for up to 8 weeks initially (or until 24-h abstinence was achieved); for those who achieved 24-h abstinence, up to an additional 12 weeks of nicotine gum was administered; however, AE data only reported for first 8 weeks.

e Sun et al. (2009) was a 12-week study; however, no nicotine tablets were administered in the final 4 weeks.

f Tønnesen et al. (1999) included both short- and long-duration trial arms, in which active nicotine patches were administered for either 12 or 26 weeks, respectively. Outcomes of interest were not reported by treatment duration, but by strength of nicotine.

#### Study interventions

The strength of nicotine administered varied considerably across the 42 included studies (see Table 5). Two-thirds of the included studies (*n* = 28) administered nicotine *via* nicotine patch, with 16 of these studies including a tapering of nicotine strength over the treatment period (32, 34, 37, 39, 43, 45, 48, 51, 58, 60–62, 64, 69, 72). Nicotine patch strength among these studies ranged from an initial strength of 21 mg to 63 mg, and a final strength of 7 mg to 21 mg. Conversely, one study began with a lower daily nicotine patch strength (11 mg) for the first week, increasing to 21 mg for the subsequent 3 weeks (49). Two of the included studies supplemented their nicotine patch intervention with nicotine gum: one study that administered either 21 mg or 42 mg nicotine patches also provided subjects with 4 mg nicotine gum to use in conjunction with their respective treatments (63); and one study that administered a tapering nicotine patch regimen (21 mg patches for 8 weeks, 14 mg patches for 2 weeks, and 7 mg patches for 2 weeks) also provided subjects with either 2 mg or 4 mg nicotine lozenges for use, as needed (58). Among the 12 studies that administered a fixed strength nicotine patch regimen, daily nicotine patch strength ranged from 7 mg (36, 70) to 63 mg (71). Across all 28 studies administering nicotine patches, 21 mg was the most common nicotine strength, used in 18 studies: 12 studies in which 21 mg was the starting strength in a tapered treatment regimen (32, 34, 37, 39, 43, 45, 51, 58, 60, 62, 64, 69), and six studies with a fixed strength regimen throughout the treatment period (33, 38, 44, 50, 63, 71).

Among the remaining nicotine interventions, six studies administered nicotine as a nicotine gum, with a strength of either 2 mg (used in six studies) (41, 46, 47, 65, 66, 73) or 4 mg (used in three studies) (46, 47, 66). Three studies administered nicotine as a nicotine tablet or lozenge, with a strength of either 2 mg (in two studies) (53, 68) or 4 mg (in two studies) (56, 68). One study administered nicotine nasal spray as its treatment, using a strength of 1 mg per dose (dose was two sprays, one per nostril), for *ad libitum* use throughout the 12-week treatment period (52). Two included studies offered subjects a choice of nicotine product: one study provided subjects with the choice of either 4 mg nicotine gum or 10 mg inhaler to use throughout the treatment period (40), while one study offered subjects three products— a 25 mg nicotine patch, a 4 mg nicotine gum, and a 10 mg nicotine inhaler—which they could use concurrently or alternate between uses throughout the treatment period (59). Lastly, two studies did not specify the mode of delivery or strength of nicotine administered, defining their treatment as personalized NRT in accordance with subjects’ preferences and levels of nicotine dependency (42, 55).

Among the control groups receiving an active treatment (see Table 5), eight studies used varenicline, with an initial dose of 0.5 mg daily, titrating up to 2 mg daily (32, 44, 45, 58, 60, 63, 67, 69). Three studies used bupropion with an initial dose of 150 mg daily, titrating up to 300 mg daily (64, 65, 69). One study with three control arms included a group in which 400 mg amisulpride was administered, as well as a group in which 2 mg risperidone was administered, both as single doses in a 1-day clinical trial (70). One 10-week study administered topiramate in one of its control arms, starting with a 25 mg daily dose and titrating up to 200 mg daily, before decreasing the dosage by 30% every 3 days in the final week (62).

#### Adherence to treatments

Of the 42 included studies, 26 studies (62%) reported data on treatment adherence (see Table 5). Treatment adherence rates varied considerably across the studies, ranging from 15% in both the intervention and control groups (41) to 99% overall (rate per group not reported) (51). Eight studies reported a treatment adherence rate of 75% or higher, with all eight studies evaluating the adherence rate through to the end of the treatment period (37, 38, 44, 49, 51, 56, 62, 69). One additional study reported a high treatment adherence rate in its nicotine patch arm (93%), but a moderate treatment adherence rate in its placebo patch arm (61%) (34). Five studies reported treatment adherence rates of 50% to 74% (39, 48, 58, 60, 61), of which two studies only reported the treatment adherence rate at time points within the treatment period—Joseph et al. (1996) reported the number of subjects who wore patches at Week 6 of a 10-week trial (39), while Lewis et al. (1998) reported the treatment adherence rate at Week 1 of a 6-week trial (61). One study, a five-armed RCT, reported that the treatment adherence rates at the end of the treatment period ranged from 45% to 53% across the study arms (72). Eight studies reported treatment adherence rates below 50% across all groups at the end treatment period (35, 40, 41, 45, 59, 63–65). An additional three studies reported unclear measures of adherence: one study reported mean number of boxes of product dispensed in each study arm (assessed as a surrogate for patch compliance) (57); one study stated that its safety analysis only included subjects “in good compliance with study protocol” (68); and, one study—a four-armed trial—reported the percent of subjects who used treatment for over 2 weeks during an 8-week trial, for which the values ranged from 36% to 77% across the study arms (67). The remaining 16 studies did not report on treatment adherence (32, 33, 36, 42, 43, 46, 47, 50, 52–55, 66, 70, 71, 73).

## Risk of bias assessment

Overall risk of bias grades for the 42 included studies were as follows: seven studies (17%) were graded as having a “low” risk of bias (33, 40, 52, 58, 60, 68, 69), 14 studies (33%) were graded as having a “high” risk of bias (32, 34, 35, 38, 45, 47, 54, 55, 59, 62, 63, 65, 67, 72), and 21 studies (50%) were graded as having an “unclear” risk of bias (36, 39, 41–44, 46, 48–51, 53, 56, 57, 61, 64, 66, 70, 71, 73, 74).The complete risk of bias assessments for each study are provided in Appendix F.

Across all the included studies, the risk of selection bias (random sequence generation) was generally low, with this domain having the highest proportion of “low” risk grades (36 of 42 studies) among all the evaluated domains (32–36, 38–47, 49–62, 65–69, 71, 72). Of the remaining studies, no studies were graded as “high” risk, and six studies were graded as having an “unclear” risk (37, 48, 63, 64, 70, 73). Similarly, the risk for selection bias (allocation concealment) was generally low, with 27 studies being graded as “low” risk for this domain (33–35, 38, 40–45, 49, 51, 52, 55, 57–61, 63, 65, 67–72), no studies being graded as “high” risk, and 15 studies being graded as having an “unclear” risk (32, 36, 37, 39, 46–48, 50, 53, 54, 56, 62, 64, 66, 73). Performance bias (blinding of participants and personnel) had the largest proportion of “high” risk of bias grades among all the domains evaluated, with seven studies graded as “high” risk (32, 38, 47, 55, 59, 62, 67); of the remaining 35 studies, 19 were graded as “low” risk (33–36, 40, 42, 45, 49, 52, 54, 58, 60, 61, 63, 65, 68–70, 72), and 16 were graded as “unclear” risk (37, 39, 41, 43, 44, 46, 48, 50, 51, 53, 56, 57, 64, 66, 71, 73). In terms of detection bias (blinding of outcome assessment) only two studies were graded as “high” risk (62, 67); of the remaining 40 studies, 20 were graded as “low” risk (33, 34, 38, 40, 42, 45, 49, 52, 55, 57–61, 63, 65, 68–70, 72), and 20 were graded as “unclear” risk (32, 35, 36, 39, 41, 43, 44, 46–48, 50, 51, 53, 54, 56, 64, 66, 71, 73, 74). Attrition bias (incomplete outcome data) was graded as “high” risk in five studies (34, 35, 54, 62, 72), “unclear” risk in seven studies (37, 39, 53, 56, 61, 64, 66), and “low” risk in 30 studies (32, 33, 36, 38, 40–52, 55, 57–60, 63, 65, 67–71, 73). Reporting bias (selective reporting) had the highest proportion of “unclear” risk of bias grades (21 studies) (35, 37, 41–43, 46, 48–51, 53, 54, 56, 57, 59, 61, 64, 66, 70, 71, 73); of the remaining 21 studies, 15 were graded as “low” risk (33, 36, 38–40, 44, 47, 52, 55, 58, 60, 62, 68, 69, 72) and six were graded as “high” risk (32, 34, 45, 63, 65, 67). No additional sources of bias were identified.

## Synthesis of results

### Arrhythmia

Across the 11 studies reporting on arrhythmia during the treatment period (*n* = 13,869), the occurrence of arrhythmia was generally low and similar between nicotine (*n* = 4,721) and non-nicotine groups (*n* = 9,148) (33, 39, 49, 54, 56, 60, 61, 64, 66, 69, 73). The number of arrhythmias reported was higher in the nicotine group compared with the non-nicotine group in six studies (39, 49, 54, 56, 69, 73), lower in the nicotine group in three studies (33, 60, 64), and the same in both groups in two studies (61, 66). The type of arrhythmia reported varied across studies and included: atrial fibrillation in two studies (56, 60); bradycardia in one study (54); tachycardia in three studies (49, 64, 73); and serious cardiac arrhythmia in one study (69). The type of arrhythmia was not specified in the remaining four studies.

Seven of the studies provided information on the study population’s health status, two of which were among healthy subjects (56, 73), and five of which were among study populations with varying indicators of adverse health: critically-ill, mechanically-ventilated subjects admitted to the medical-surgical ICU (33), subjects with a history of one or more CVDs (39), hospitalized subjects (61), or subjects with ulcerative colitis (49, 54).

Three studies were among a population of either former cigarette smokers (73), or a combination of nonsmokers and former cigarette smokers (49, 54). The remaining eight studies were all among current cigarette smokers. Seven of these studies reported cigarette smoking abstinence, which was generally low across studies, never reaching 50% in any one treatment group. Cigarette smoking abstinence among nicotine groups ranged from 10% self-reported continuous abstinence (66) to 36% abstinence (measure not reported) (64). Among non-nicotine controls, cigarette smoking abstinence ranged from 3.9% self-reported continuous abstinence (66) to 46% abstinence (measure not reported) (64).

Ten studies evaluated nicotine versus placebo (33, 39, 49, 54, 56, 60, 61, 66, 69, 73). Additionally, nicotine versus varenicline was evaluated in two studies (60, 69), nicotine versus bupropion in two studies (64, 69), and nicotine versus no treatment in two studies (61, 64). The nicotine treatment period duration ranged from 1 h (73) to 6 months (54), with a median treatment duration of 10 weeks. Treatment adherence rates were reported in seven of the 11 studies and varied considerably. Adherence ranged from 22% (64) to 97% (49) among nicotine groups, and from 40% (64) to 97% (49) among non-nicotine groups.

Among the 11 studies reporting occurrence of arrhythmia, three were graded as “low” risk of bias, one was graded as “high” risk of bias, and seven were graded as “unclear” risk of bias.

#### Main analysis for arrhythmia (overall)

All 11 studies, with a total of 13,869 subjects (4,721 nicotine subjects and 9,148 non-nicotine control subjects) met the criteria for inclusion in the meta-analysis (33, 39, 49, 54, 56, 60, 61, 64, 66, 69, 73). RRs could not be estimated from one study of 124 subjects (62 subjects in both the nicotine and non-nicotine groups), as no arrhythmia events occurred in either study group. Among the remaining 10 studies with recorded events of arrhythmia, pooled data showed that the rates of arrhythmia were not statistically significantly different between the nicotine and non-nicotine control groups (RR 1.20; 95% CI 0.59–2.42). Statistical heterogeneity observed by the model was moderate (I^2^ 50%).

**Figure 4:**
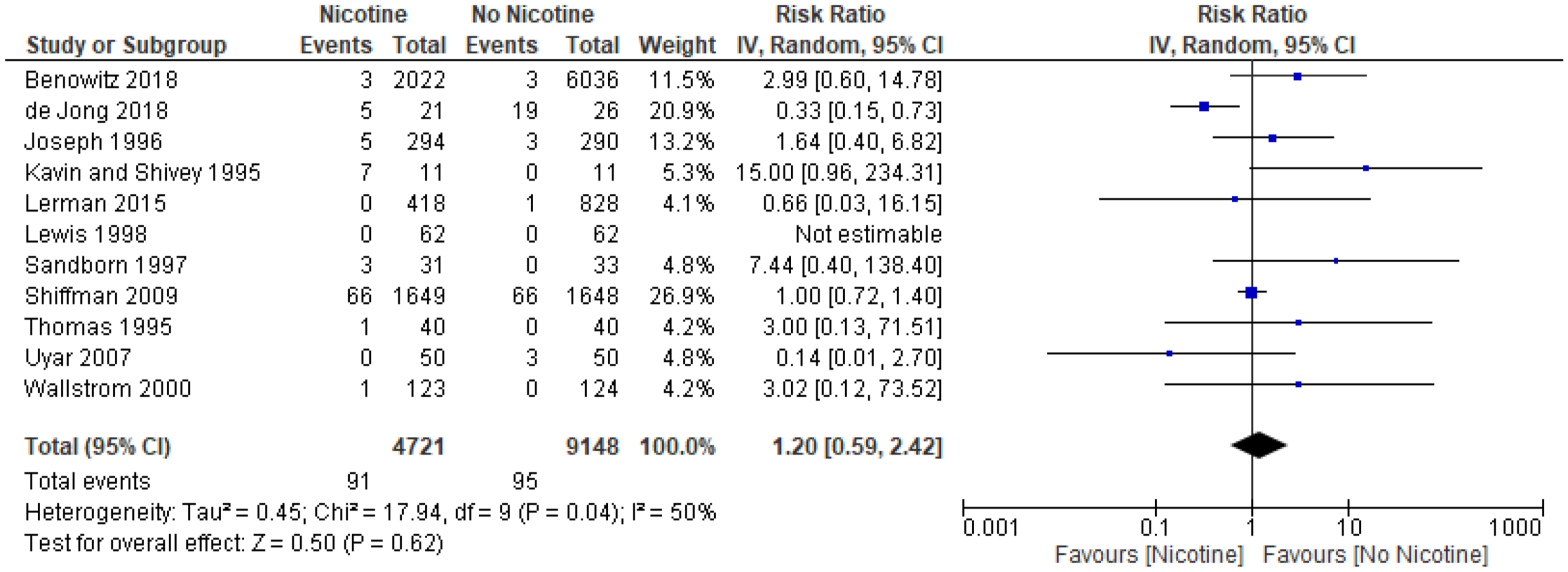
Forest plot, meta-analysis for arrhythmia (overall).

The forest plot for the overall meta-analysis of arrhythmia is presented in Figure 4.

### Nonfatal myocardial infarction

Thirty-two studies reported data on the occurrence of nonfatal myocardial infarction during the treatment period (*n* = 20,945; *n* = 9,323 in the nicotine groups and *n* = 11,622 in the non-nicotine control groups) (32–41, 43–48, 50, 51, 53, 54, 56, 60–63, 65, 67–72). Overall, the number of nonfatal myocardial infarctions reported was low. In 23 studies, with a pooled sample size of 5,291 subjects (2,454 in the nicotine group and 2,837 subjects in the non-nicotine control groups), no myocardial infarctions occurred (34, 36, 37, 40, 41, 43–48, 50, 51, 53, 54, 56, 60–62, 67, 68, 70, 71). Among the remaining nine studies in which nonfatal myocardial infarctions did occur, five studies reported a higher number of myocardial infarctions in the nicotine group than in the non-nicotine control group (32, 38, 63, 65, 72), while three studies reported a lower number of nonfatal myocardial infarctions in the nicotine group (33, 35, 39). In one study with a four-arm design, the number of nonfatal myocardial infarctions in the nicotine group was the same or lower than in the non-nicotine control group (69).

Eighteen of the 32 studies included for reporting data on nonfatal myocardial infarction provided information on their study population’s health status. Half of these studies were among healthy subjects (34, 43, 48, 51, 56, 62, 70–72). The other half described their study populations as having some indication of adverse health—critically-ill, mechanically-ventilated subjects (33), subjects with a history of one or more CVDs (39), hospitalized subjects (61), general hospital outpatients (35), subjects with ulcerative colitis (54), subjects who underwent colorectal surgery (41), subjects with a history of alcohol dependence (37), subjects with substance use disorder (45), and methadone-maintained (opiate-dependent) subjects (63).

The majority of studies (28 of 32 studies) reporting on nonfatal myocardial infarction were among study populations of current tobacco users: 26 studies were among current cigarette smokers (32, 33, 35, 37–40, 43–48, 50, 51, 53, 56, 60–63, 65, 67–69, 72), and two studies were among current smokeless tobacco users (34, 71). Among the four remaining studies, one study was among nonsmokers and light cigarette smokers (36), one study was among nonsmokers (70), one study was among nonsmokers or former cigarette smokers (54), and one study did not specify a tobacco use status among its study population (41). Twenty-five of the 32 studies reported abstinence from tobacco as an outcome measure, which was generally low: among nicotine groups, abstinence rates ranged from 1.5% continuous cigarette smoking abstinence (from Day 14 through to 6-month assessment) (63) to 73% 7-day PPA of smokeless tobacco use at the end of 8-week treatment period (71). Abstinence rates among the non-nicotine control groups ranged from 0% continuous cigarette smoking abstinence (from Day 14 through to 6-month assessment) (63) to 73% self-reported abstinence (not having smoked for about 1 week) at Week 4 of an 8-week treatment period (67). In 17 of the 25 studies reporting tobacco abstinence, abstinence rates did not exceed 50% in any one group (34, 35, 37, 39, 40, 43–46, 48, 56, 61–63, 68, 69, 72).

Twenty-nine studies evaluating nonfatal myocardial infarction evaluated nicotine versus placebo (33–41, 43–46, 48, 50, 51, 53, 54, 56, 60–63, 67–72). Five studies evaluated nicotine versus varenicline (32, 60, 63, 67, 69), and two studies evaluated nicotine versus bupropion (65, 69). Additionally, a range of other non-nicotine controls were compared to nicotine, each reported in one study: topiramate (62); amisulpride (70); risperidone (70); fresh lime (47); and no treatment (61). The duration of treatment periods ranged from 4.5 h (70) to 26 weeks (72), with a median treatment duration of 10 weeks. Treatment adherence, reported in 21 of the 32 studies evaluating nonfatal myocardial infarction, varied considerably. The highest adherence rate reported was 99% across all treatment groups (rates per treatment group were not reported) (51). Per treatment group, reported rates of adherence ranged from 10% (67) to 93% (34, 62) among nicotine groups, and from 10% (67) to 94% (62) among non-nicotine control groups.

Among the 32 studies reporting on nonfatal myocardial infarction, five studies were graded as “low” risk of bias (33, 40, 60, 68, 69), 12 were graded as “high” risk (32, 34, 35, 38, 45, 47, 54, 62, 63, 65, 67, 75), and 15 were graded as “unclear” risk (36, 37, 39, 41, 43, 44, 46, 48, 50, 51, 53, 56, 61, 70, 71).

One study reporting on nonfatal myocardial infarctions provided additional detail on individual events. The Preloading Investigators (2008) (38) evaluated a nicotine patch (21 mg daily) administered before a target quit date, and reported one case of nonfatal myocardial infarction in the nicotine patch group. The subject had stopped the medication prematurely 2 days before the myocardial infarction. No nonfatal myocardial infarctions occurred in the non-nicotine (i.e., no treatment) control group.

#### Main analysis for nonfatal myocardial infarction (overall)

All 32 studies with a total of 20,945 subjects (9,323 subjects in the nicotine groups and 11,622 subjects in the non-nicotine control groups) met the criteria for inclusion in the meta-analysis. RRs could not be estimated for the 23 studies in which no nonfatal myocardial infarctions occurred in either the nicotine or the non-nicotine control groups (34, 36, 37, 40, 41, 43–48, 50, 51, 53, 54, 56, 60–62, 67, 68, 70, 71).

**Figure 5:**
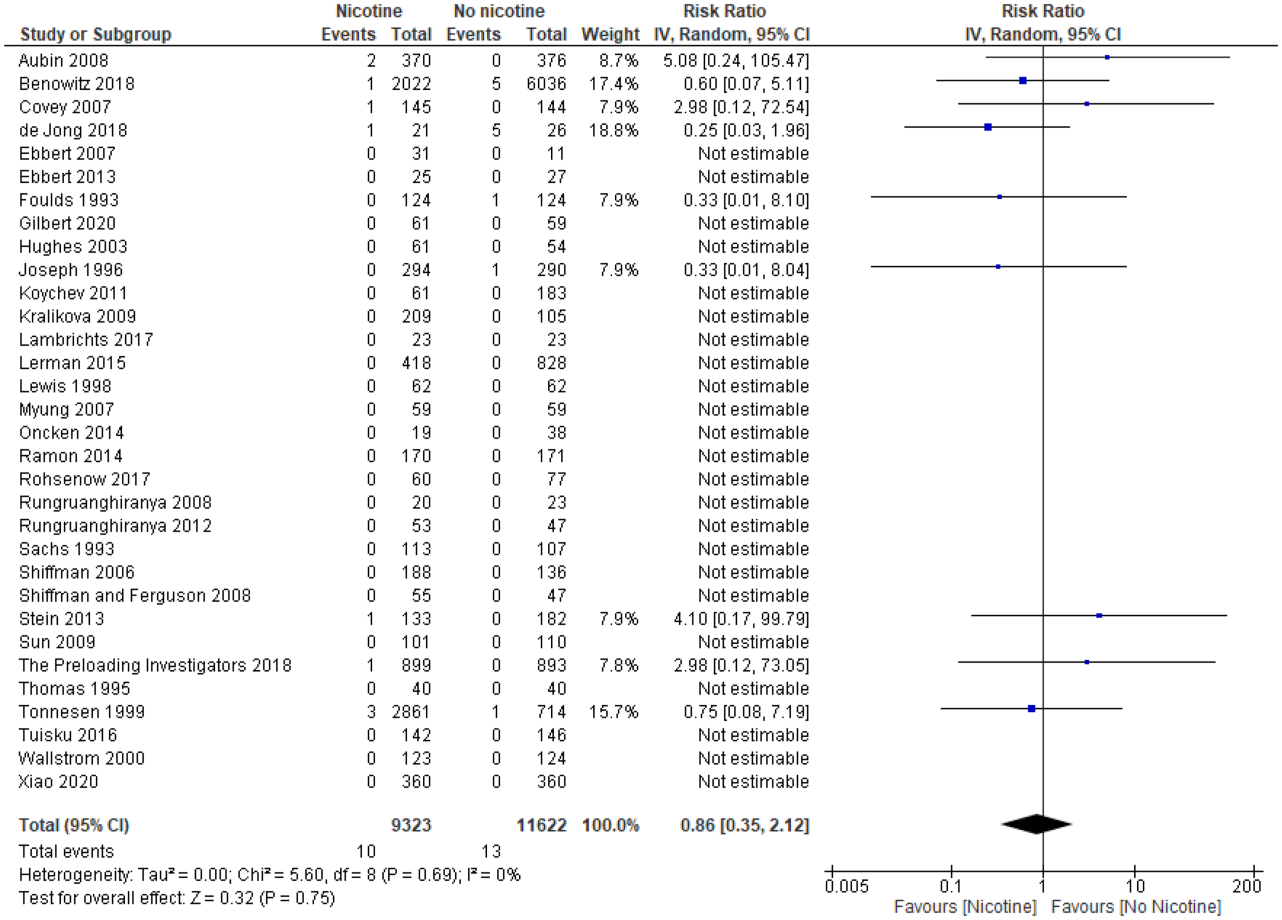
Forest plot, meta-analysis for nonfatal myocardial infarction.

Among the remaining nine studies reporting at least one nonfatal myocardial infarction, pooled data showed that the rates of nonfatal myocardial infarction associated with nicotine compared with non-nicotine controls were not statistically significantly different (RR 0.86; 95% CI 0.35–2.12). No statistical heterogeneity was observed by the model (I^2^ 0%). The forest plot for the overall meta-analysis of nonfatal myocardial infarction is presented in Figure 5.

### Nonfatal stroke

Twenty-nine studies reported data on the occurrence of nonfatal stroke during the treatment period (*n* = 17,213; *n* = 6,247 in the nicotine groups and *n* = 10,966 in the non-nicotine control groups) (32–34, 36–38, 40, 42–47, 50, 51, 53, 54, 56, 58, 60–63, 65, 67–71). Overall, the number of nonfatal strokes reported was low. In 26 studies, with a pooled sample size of 14,831 subjects (5,345 in the nicotine group and 9,486 subjects in the non-nicotine control group), no nonfatal strokes occurred at all (32–34, 36–38, 42–47, 50, 51, 53, 54, 56, 61–63, 65, 67–71). Among the remaining three studies with observed events, none reported more than two nonfatal strokes, either overall or in any one treatment group.

Fourteen of 29 studies reporting on nonfatal stroke provided information on their study population’s health status. Half of these studies were among healthy subjects (34, 43, 51, 56, 62, 70, 71). The other half described their study populations as having some indication of adverse health—critically ill, mechanically-ventilated subjects, subjects scheduled for primary elective hip or knee surgery, hospitalized subjects, subjects with ulcerative colitis (54), subjects with a history of alcohol dependence, opiate dependent and methadone-maintained subjects, and subjects with a substance abuse disorder.

The majority of studies (26 of 29) reporting on nonfatal stroke were among study populations of current tobacco users: 24 studies were among current cigarette smokers (32, 33, 37, 38, 40, 42–47, 50, 51, 53, 56, 58, 60–63, 65, 67–69), two studies were among current smokeless tobacco users (34, 71), two studies were of nonsmokers (54, 70), and one study was of nonsmokers and light cigarette smokers (36).

Across the studies reporting on nonfatal stroke, tobacco abstinence rates varied, ranging from 3% eCO verified 7-day PPA (45) to 73% self-reported 7-day PPA (71) in the nicotine groups, and from 2.2% (eCO-verified) 7-day PPA (63) to 73% self-reported 7-day PPA in the non-nicotine control groups.

Twenty four studies reporting on nonfatal stroke evaluated nicotine versus placebo (32, 33, 37, 38, 40, 42–47, 50, 51, 53, 56, 58, 60–63, 65, 67–69). Nicotine was evaluated versus varenicline in seven studies (32, 45, 58, 60, 63, 67, 69), and versus bupropion in two studies (65, 69). Other control treatments, evaluated by one study each, included topiramate (62), amisulpride or risperidone (70), counseling (38), fresh lime (47), and no treatment (42). The duration of treatment periods ranged from 4.5 h (70) to 6 months (40), with a median treatment duration of 10 weeks. Treatment adherence rates, reported in 17 of the 29 studies evaluating nonfatal stroke, varied considerably. Adherence ranged from 10% (67) to 99% (44) among the nicotine groups, and from 36% (67) to 99% (44) among the non-nicotine control groups.

Among the 29 studies reporting on nonfatal stroke, six studies were graded as “low” risk of bias (33, 40, 58, 60, 68, 69), 10 were graded as “high” risk of bias (32, 34, 38, 45, 47, 54, 62, 63, 65, 67), and 13 were graded as “unclear” risk of bias (36, 37, 42–44, 46, 50, 51, 53, 56, 61, 70, 71).

One of the 29 studies, two studies provided further details on nonfatal strokes that occurred during their study. In Lerman et al. (60) two nonfatal strokes occurred, both in the placebo patch plus placebo pill group: one event occurred at Week 11 in a subject who reported to be abstinent from cigarette smoking at the time of the event; and, one event occurred at Week 8 in a subject reporting to be smoking 15 cigarettes per day at the time of the event. In Kralikova et al. (40), one nonfatal stroke—diagnosed as cerebrovascular disorder—occurred in a 52 year old female subject in the placebo group, who had been smoking cigarettes for 27 years, and reported smoking 33 cigarettes per day at baseline.

#### Main analysis for nonfatal stroke (overall)

All 29 studies (17,213 subjects; 6,247 subjects in nicotine groups and 10,966 subjects in non-nicotine control groups) met the criteria for inclusion in the meta-analysis. In 26 of the 29 studies, RRs could not be estimated, since no nonfatal strokes occurred in either the nicotine or the non-nicotine control groups (32–34, 36–38, 42–47, 50, 51, 53, 54, 56, 61–63, 65, 67–71).

Among the three studies in which nonfatal strokes did occur, pooled data showed that the rates of nonfatal stroke were not statistically significantly different between nicotine and non-nicotine controls (RR: 0.30; 95% CI 0.05–1.80). No statistical heterogeneity was observed by the model (I^2^ 0%).

**Figure 6:**
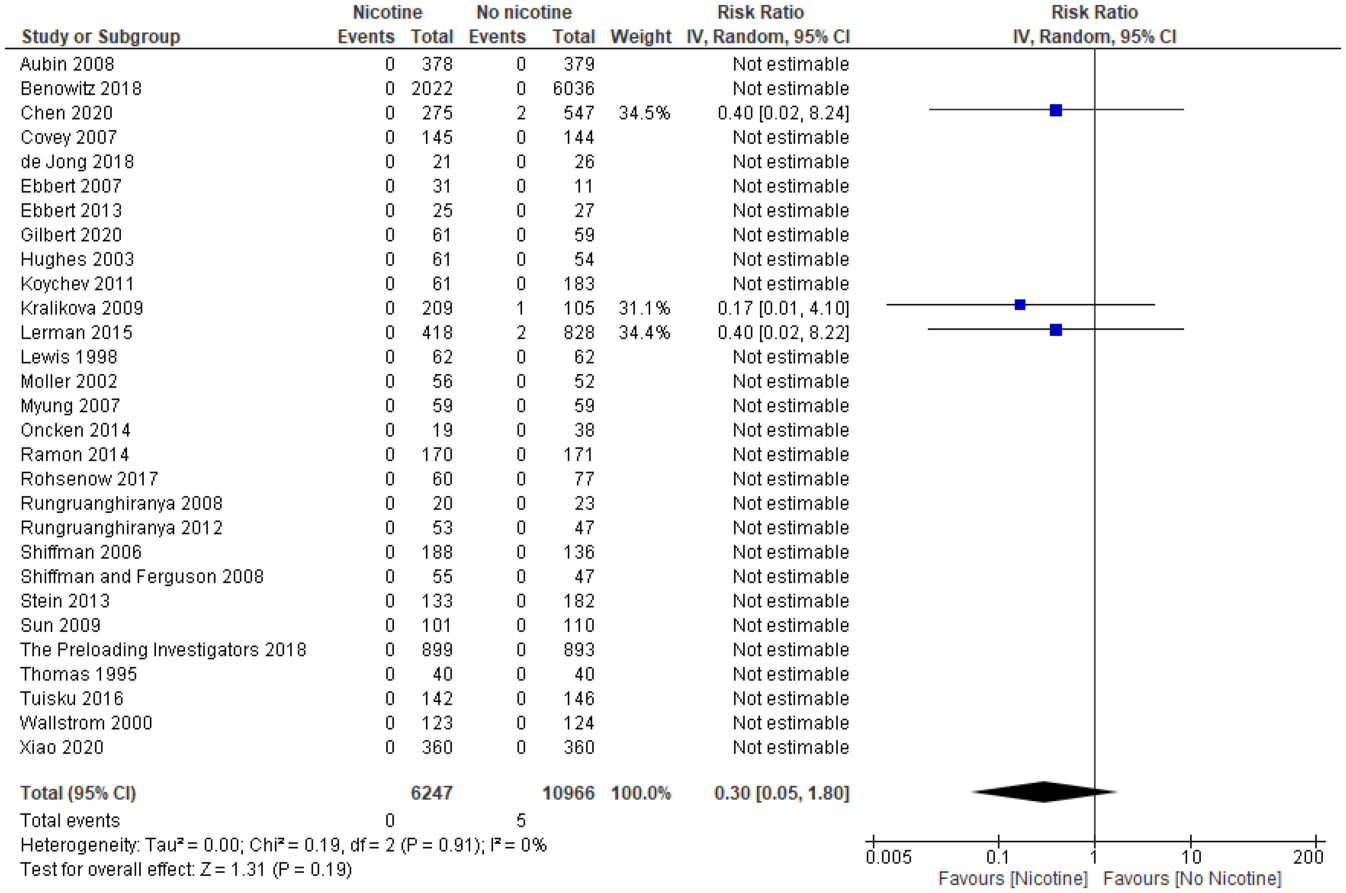
Forest plot, meta-analysis for nonfatal stroke.

The forest plot for the overall meta-analysis of nonfatal stroke is presented in Figure 6.

### Cardiovascular death

Thirty-three studies reported data on the occurrence of cardiovascular deaths during the treatment period (*n* = 18,382; *n* = 6,985 in the nicotine groups and *n* = 11,397 in the non-nicotine control groups) (32–38, 40–47, 50–59, 61, 62, 65, 67–71). Across all 33 studies, the occurrence of cardiovascular deaths was low. No cardiovascular deaths were observed in 29 studies with a pooled sample size of 7,936 subjects (4,102 subjects in the nicotine groups and 3,834 subjects in the non-nicotine control groups). Among the remaining four studies, none reported more than two cardiovascular deaths overall, and none reported more than one cardiovascular death in any one treatment group.

Seventeen of the 33 studies reporting data on cardiovascular deaths provided information on their study population’s health status; eight studies were among healthy subjects (34, 43, 51, 52, 56, 62, 70, 71), and the remaining nine studies described their study populations as having some indication of adverse health— critically ill, mechanically-ventilated subjects, hospital outpatients, hospitalized subjects, subjects scheduled for primary elective hip or knee surgery, subjects who underwent colorectal surgery, and subjects who had breast cancer, ulcerative colitis (54), history of alcohol dependence, or substance use disorder.

The majority of studies (27 of 33 studies) reporting on cardiovascular deaths were among current cigarette smokers (32, 33, 35–38, 40, 42–47, 50–53, 55–59, 61, 62, 65, 67–69). Of the remaining six studies, two were among nonsmokers or former smokers (54, 70), two were among current smokeless tobacco users (34, 71), one was among nonsmokers and light cigarette smokers, and one study did not specify the smoking status (41). Tobacco abstinence rates were reported by 26 of the 33 studies reporting on cardiovascular deaths. Abstinence rates among nicotine groups ranged from 3% 7-day abstinence (confirmed with eCO level ≤ 4 ppm and salivary cotinine level ≤ 15 ng/ml) at 3 months (45) to 73% 7-day PPA (71). Abstinence rates among nicotine groups did not exceed 50% in 22 of 26 studies. Among non-nicotine control groups, abstinence rates ranged from 2.2% self-reported 7-day abstinence (59) to 73% self-reported abstinence (not having smoked for about 1 week) (67). Abstinence rates among non-nicotine groups did not exceed 50% in 23 of 26 studies.

Twenty-seven studies reporting on cardiovascular deaths evaluated nicotine versus placebo (32, 33, 35–38, 40, 42–44, 46, 47, 50–53, 55–59, 61, 62, 65, 67–69). Four studies evaluated nicotine versus varenicline (32, 45, 58, 67), three studies evaluated nicotine versus no treatment (42, 55, 59), and two studies evaluated nicotine versus bupropion (14, 65). Additionally, a range of other non-nicotine controls were compared to nicotine, each reported in one study: topiramate (62), amisulpride or risperidone (70), fresh lime (47), placebo-topiramate (62), and counseling (38). The duration of treatment period ranged from 4.5 h (70) to 6 months (54, 59), with a median treatment duration of approximately 9 weeks. Treatment adherence was reported by 19 of 33 studies reporting data on cardiovascular deaths, and ranged considerably across studies: from 29% (35) to 99% (44) in the nicotine groups, and from 15% (41) to 99% (44) in non-nicotine control groups.

Among the 33 studies reporting data on cardiovascular deaths, six studies were graded as “low” risk of bias (33, 40, 52, 58, 68, 69), 12 were graded as “high” risk of bias (32, 34, 35, 38, 45, 47, 54, 55, 59, 62, 65, 67), and 15 were graded as “unclear” risk of bias (36, 37, 41–44, 46, 50, 51, 53, 56, 57, 61, 70, 71).

Of the 33 studies, two studies provided further details on the events of cardiovascular deaths. The study by Benowitz et al. (69) reported two cardiovascular deaths: the first, a White male aged 52 years, died on Day 77 of treatment with bupropion. The subject’s wife reported that he experienced intense pain in his chest and in both arms, and died on a public road with no medical assistance. The death certificate listed the cause as “non-traumatic cardiorespiratory arrest.” The second, a Black female aged 42 years, died on Day 60 of treatment with placebo. The subject was found dead; an autopsy determined the cause to be bilateral pulmonary thromboemboli, and toxicology showed cocaine abuse. The study by Hays et al. (57) reported one cardiovascular death in their nicotine patch group, as a result of myocardial infarction. The subject had failed to return to the study after Week 3, and had reported continued cigarette smoking at each follow-up that had been attended; family members also reported that nicotine patches were not being used by the subject, and that the subject had continued smoking cigarettes.

#### Main analysis for cardiovascular death (overall)

**Figure 7:**
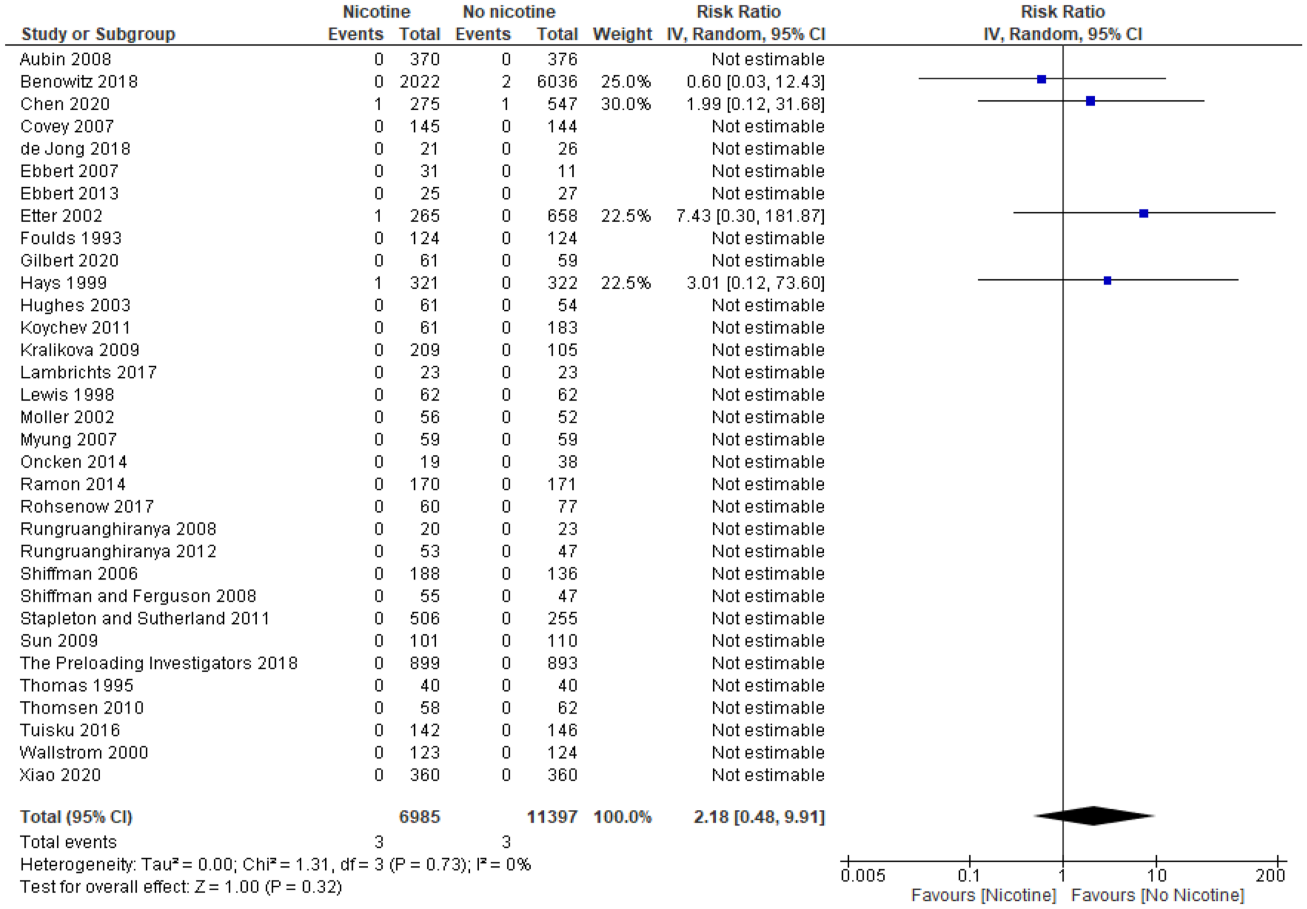
Forest plot, meta-analysis for cardiovascular death.

All 33 studies with a pooled sample of 18,382 subjects (6,985 subjects in the nicotine group and 11,397 subjects in the non-nicotine control group) met the criteria for inclusion in the meta-analysis. For 29 of the 33 studies, RRs could not be estimated as there were no cardiovascular deaths recorded in either the nicotine or the no-nicotine control groups. Among the remaining four studies, pooled data showed that the rates of cardiovascular death were not statistically significantly different between nicotine and non-nicotine control groups (RR 2.18; 95% CI 0.48–9.91; see Figure 7). No statistical heterogeneity was observed by the model (I^2^ 0%).

## Strength of evidence

The overall quality of the body of evidence was assessed and graded as “high,” “moderate,” “low,” or “very low” using the GRADE system. Table 6 provides the quality of evidence for the outcome measures used in the current review to examine the association between nicotine and the risk of adverse cardiovascular events. The bodies of evidence for all four outcomes—arrhythmia, nonfatal myocardial infarction, nonfatal stroke, and cardiovascular death—were all graded as “moderate,” having been downgraded for imprecision. For all four outcomes, this was due to the low number of adverse cardiovascular events reported in individual studies, and the variation in effects, as evidenced by the wide CIs (see Figures 4, 5, 6 and 7).

**Table 6:** SOE domain scores according to the GRADE system

	Study limitations	Consistency of effect	Imprecision	Indirectness	Publication bias	Overall
Arrhythmia	No serious limitations	No serious inconsistency	Serious imprecision	No serious indirectness	No publication bias	Moderate
Nonfatal Myocardial infarction	No serious limitations	No serious inconsistency	Serious imprecision	No serious indirectness	Undetermined^a^	Moderate
Nonfatal Stroke	No serious limitations	No serious inconsistency	Serious imprecision	No serious indirectness	Undetermined^a^	Moderate
Cardiovascular Death	No serious limitations	No serious inconsistency	Serious imprecision	No serious indirectness	Undetermined^a^	Moderate

GRADE, Grading of Recommendations Assessment, Development, and Evaluation; SOE, strength of evidence.

a Publication bias could not be assessed due to the inclusion of less than 10 studies whose effect measures could be estimated.

## Discussion and conclusions

The majority of studies evaluating nonfatal myocardial infarction, nonfatal stroke, and cardiovascular death reported no events that occurred in either the nicotine or non-nicotine control groups. Among the studies that did report occurrence of the events, rates of AEs were similarly low between the nicotine and non-nicotine control groups.

Consistent with findings from previous systematic reviews and/or meta-analyses (19, 76), pooled data showed that rates for all four outcomes—arrhythmia, nonfatal myocardial infarction, nonfatal stroke, and cardiovascular death— were not significantly different between nicotine and non-nicotine controls. Further, findings were consistently nonsignificant across all sensitivity analyses for each outcome, all subgroup analyses by duration of nicotine exposure for each outcome, and a subgroup analysis of arrhythmia by subtype. No statistical heterogeneity was observed by any of the models analyzing nonfatal myocardial infarction, nonfatal stroke, and cardiovascular death; the moderate to substantial statistical heterogeneity observed for arrhythmia overall, for two of its subtypes, for its subgroup analysis by exposure duration, and for its sensitivity analyses did not appear to affect the results and may have been the result of the broad definitions of interventional and controls adopted for this review. Where evaluation was possible—specifically, for arrhythmia—there was no indication of publication bias.

The overall quality of the body of evidence for each of the four outcomes of interest was graded as “moderate,” limited only by the imprecision of results. This was due to the low rates of occurrence and the correspondingly wide confidence intervals observed for each outcome and is consistent with previous systematic reviews (19, 20, 76). The strength of each evidence base was not limited by risk of bias, given that the sensitivity analyses which excluded “high” risk of bias studies did not change the overall findings. Additionally, although a high number of studies for each outcome measure included studies graded as having “unclear” risk of bias, this did not affect the strength of evidence evaluation, since risk of bias cannot be inferred from a lack of information. Further, the sensitivity analyses that excluded studies not reporting the systematic collection of AE data provided some indication that studies with an unclear degree of methodological rigor in their collecting and reporting of AE data did not impact the findings.

The current systematic review is limited by a shortage of studies among the evidence base that evaluated adverse cardiovascular events as primary outcomes, poor treatment adherence across the included studies, and short duration of trials. Few studies evaluated safety as their primary objective, and among these there was a lack of specific cardiovascular AEs evaluated as primary outcomes of interest. Thus, the reporting of outcomes often lacked detail on individual AEs and information regarding systematic collection of AE data. Further, where reported, treatment adherence varied considerably across studies, ranging from 15% to 99%. Hence, the rates of adverse cardiovascular events attributable to not only nicotine, but also to other active controls—such as varenicline and bupropion—may have been affected by the level of treatment adherence. This may introduce bias by differentially influencing rates of adverse cardiovascular events reported by the studies. Lastly, the short duration of the intervention or the short follow up period may have influenced the outcomes reported. However, although CVD is a chronic and multifaceted disease, which could allow for many risk factors prior to intervention to influence the outcomes, the randomization of the studies should have addressed both known and unknown risk factors between groups. Moreover, as stated in the methodology, the main aim of this systematic review and meta-analysis was to investigate adverse cardiovascular events; consequently, the nature of the outcome is acute. The systematic review and meta-analysis was not investigating progression of the disease, or changes in cardiovascular parameters that may have led to the adverse cardiovascular events.

Despite the methodological rigor of this systematic review, some limitations resulting from inherent limitations in the evidence base, should be noted. First, the predominance of tobacco cessation studies among the included studies could have led to an imbalance in the rate of cigarette smoking—a known risk factor for CVD, as well as an additional source of nicotine administration—across groups. Indeed, abstinence was generally low among cessation studies. Additionally, the broad definition of both intervention and control allowed for a range of treatments. More specifically, for the intervention, broad definitions included various routes and strengths of nicotine. Controls varied across placebo, no treatment, and other non-nicotine interventions, such as varenicline and bupropion, among others. Although current evidence suggests no elevated risk of adverse cardiovascular events with bupropion (77, 78), the evidence base pertaining to varenicline is less clear (77, 79–81). Hence, it is possible that active treatments as controls may have differential associations with adverse cardiovascular events that could confound the analyses in this review.

This systematic review exhibits numerous key strengths. Having been benchmarked against the AMSTAR-2 critical appraisal tool, the current systematic review was conducted with a high degree of methodological rigor. The search strategy was defined to allow for all RCTs administering nicotine and, importantly, was not restricted by search terms related specifically to the review’s outcome measures. This comprehensive approach ensured that the literature search was not limited to studies that reported adverse cardiovascular events as primary outcomes of interest. Secondly, the current systematic review restricted the research design of included studies to RCTs, which are considered the gold standard and are better-suited to examine causal associations due to their minimization of biases (82, 83). Subsequently, through a well-defined PICOS that only allowed for studies evaluating precisely defined, clinically diagnosed, adverse cardiovascular events occurring during the exposure period among strict nicotine and non-nicotine groups, this review was able to distill the evidence base to those studies directly evaluating the association between exposure to nicotine alone and adverse cardiovascular events. Additionally, the strict adherence to PRISMA guidelines ensured a high degree of transparency in reporting. Lastly, the meta-analyses benefited from the inclusion of all studies for each of the outcomes, allowing for greater analytical power, potentially higher precision and reliability, and a more straightforward interpretation.

The findings of this systematic review and meta-analysis indicate that, with moderate certainty, there are no significant associations between the use of nicotine and the risk of clinically diagnosed adverse cardiovascular events—specifically, arrhythmia, nonfatal myocardial infarction, nonfatal stroke, and cardiovascular death. Future studies evaluating adverse cardiovascular events as primary outcomes, with larger sample sizes and longer treatment periods, would be needed to provide stronger evidence for such an association. However, given the operative costs of RCTs, designing trials to address these limitations may be challenging. Further, although the ideal study design would include only never tobacco users exposed to either nicotine or a non-nicotine control, providing long-term administration of NRTs to never tobacco users is not feasible.

## Author contributions

MK conceived the study. MK, IS, RM, TB, AJ, and JC collected and analyzed project data. MK, IS, RM, RP, and CJ defined the study design, selection of measures, interpretation of data, and co-wrote the manuscript. All authors have read and approved the final article.

## Funding

All study activities were executed by providers external to RAI Services Company (Thera-Business), who were financially compensated for services according to contractual terms with RAI Services Company. RAI Services Company is a wholly owned subsidiary of Reynolds American Inc., whose operating companies manufacture and market tobacco products. The conception, analysis, and writing for this manuscript was a collaboration between Thera-Business and RAI Services Company.

## Conflict of interest

MK and CJ serve as full-time employee of RAI Services Company, a wholly owned subsidiary of Reynolds American Inc. RP is a full-time employee of BAT (Investments) Limited.

All study activities were executed by providers external to RAI Services Company (Thera-Business), who were financially compensated for services according to contractual terms with RAI Services Company. RAI Services Company is a wholly owned subsidiary of Reynolds American Inc., whose operating companies manufacture and market tobacco products. The conception, analysis, and writing for this manuscript was a collaboration between Thera-Business and RAI Services Company.

## Publisher’s note

All claims expressed in this article are solely those of the authors and do not necessarily represent those of their affiliated organizations, or those of the publisher, the editors and the reviewers. Any product that may be evaluated in this article, or claim that may be made by its manufacturer, is not guaranteed or endorsed by the publisher.

## Abbreviations

μg: Microgram
AE: Adverse Event
AMSTAR: A MeaSurement Tool to Assess systematic Reviews
CI: Confidence interval
cm^2^: Square centimeters
cpd: Cigarettes per day
CVD: Cardiovascular disease
DALY: Disability-adjusted life years
EAGLES: Evaluating Adverse Events in a Global Smoking Cessation Study
eCO: Expired/exhaled carbon monoxide
GRADE: Grading of Recommendations Assessment, Development, and Evaluation
HR: Hazard ratio
ICU: Intensive care unit
MACE: Major adverse cardiovascular event
MeSH: Medical subject headings
mg: Milligram
NA: Not applicable
ng/ml: Nanograms per milliliter
NR: Not reported
NRT: Nicotine replacement therapy
OR: Odds ratio
PICOS: Population or participants and conditions of interest, interventions or exposures, comparisons or control groups, outcomes of interest, and study designs
PPA: Point prevalence abstinence
ppm: Parts per million
PRISMA: Preferred Reporting Items for Systematic Reviews and Meta-Analyses
RCT: Randomized controlled trial
RR: Relative risk
SD: Standard deviation
SOE: Strength of evidence
UK: United Kingdom
US: United States
WHO: World Health Organization
YLD: Years lived with disability
YLL: Years of life lost
