# Supplementary material for: Study title: A systematic review of RCTs to examine the risk of adverse cardiovascular events with nicotine use
Source: Front Cardiovasc Med. 2023 Mar 21;10:1111673. doi: 10.3389/fcvm.2023.1111673 (PMC10071010; doi:10.3389/fcvm.2023.1111673)
Supplement: Supplementary file 1 [file Data_Sheet_1.docx]

# APPENDICES

## Literature Search Strategy

Database(s): **Embase**1974 to 2021 June 10**, Ovid MEDLINE(R) ALL**1946 to June 10, 2021

Search Strategy:

| **Set #** | **Searches** | **Results** |
| --- | --- | --- |
| S1 | ((AB,TI((nicotine) near/3 (lozenge* or patch* or gum or "nasal spray" or inhale* or tablet* OR sublingual OR therapy)))) | 12479 |
| S2 | AB,TI(random*) | 2909528 |
| S3 | S2 AND S1 | 2047 |
| S4 | (S3) and (la.exact("English")) | 1996 |

## Literature Search Output: Articles Reviewed at the Full-Text Level (n=700)

| **Refid** | **Bibliography** |
| --- | --- |
| 5 | Hsueh,​ Kuang-Chieh,​ Tang,​ Pei-Ling,​ McRobbie,​ Hayden. Effectiveness of Varenicline Versus Combination Nicotine Replacement Therapy for Smoking Cessation: One-Year Outcomes in a Smoking Cessation Clinic in Taiwan. Nicotine & tobacco research : official journal of the Society for Research on Nicotine and Tobacco. 2021;23:1094.  **Abstract:** INTRODUCTIONVarenicline and combination nicotine replacement treatment (cNRT) have been recommended as the most effective pharmacotherapies,​ with equal abstinence rate for smoking cessation in a network meta-analysis of randomized trials,​ but data from real-world long-term follow-up studies are rare. This study aimed to compare the 12-month sustained abstinence rates of smokers using varenicline versus cNRT in their quit attempt.METHODSA total of 3569 smokers were recruited via the Department of Family Medicine outpatient department at Kaohsiung Veteran General Hospital between June 2013 and March 2019. Participants received counseling from a physician and chose either varenicline (N = 2870) or cNRT (N = 699) for smoking cessation. Both varenicline and cNRT users could receive a free 8-week supply and eight clinic visits over 90 days. Participants were followed-up by telephone at 12,​ 24,​ and 52 weeks from first visit. The primary outcome measure of the study was self-reported sustained abstinence up to 52 weeks.RESULTSVarenicline users had a significantly higher sustained abstinence rate at weeks 12-52,​ adjusted for baseline variables (15.2% vs 10.3%,​ p = .001; adjusted odds ratio = 1.47,​ 95% confidence interval: 1.05-2.05). Other significant predictors of 52 weeks sustained abstinence were being male,​ having a higher income,​ attending more clinical visits,​ and have lower nicotine dependence.CONCLUSIONVarenicline appears to have higher sustained abstinence rates to 52 weeks compared with cNRT,​ in a smoking cessation clinic where smokers can choose their medication option.IMPLICATIONSNetwork meta-analysis of randomized trials suggests that varenicline and cNRT are similarly effective for smoking cessation. This study shows that 1-year sustained abstinence rates were significantly higher among smokers using varenicline,​ compared with smokers using cNRT,​ when used as part of a structured smoking cessation program. These findings are highly relevant to policy makers and service providers to help determine provision of smoking cessation treatment. |
| 11 | Crouser, Elliott D, Smith, Rachel M, Culver, Daniel A, Julian, Mark W, Martin, Karen, Baran, Joanne, Diaz, Christopher, Erdal, Barbaros Selnur, Hade, Erinn M. A Pilot Randomized Trial of Transdermal Nicotine for Pulmonary Sarcoidosis. Chest. 2021; 203:A1329.  **Abstract:** BACKGROUNDTobacco smoking is associated with a reduced risk of developing sarcoidosis,​ and we previously reported that nicotine normalizes immune responses to environmental antigens in patients with active pulmonary sarcoidosis. The effects of nicotine on pulmonary sarcoidosis progression is unknown.RESEARCH QUESTIONIs nicotine treatment well-tolerated and will it improve lung function in patients with active pulmonary sarcoidosis?STUDY DESIGN AND METHODSWith local IRB approval,​ we conducted a randomized,​ double-blind,​ controlled pilot trial of daily nicotine transdermal patch treatment (21 mg daily) or placebo patch treatment for 24 weeks. The Ohio State University Wexner Medical Center and Cleveland Clinic enrolled 50 consecutive adult subjects 18 years of age and older with active pulmonary sarcoidosis,​ based on symptoms (dyspnea,​ cough) and objective radiographic evidence of infiltrates consistent with non-fibrotic lung disease. We compared each treatment group at 26 weeks based on repeated measures of forced vital capacity (FVC),​ forced expiratory volume in one second (FEV1),​ quantitative lung texture score (LTS) based on CT texture analysis,​ Fatigue Assessment Score (FAS),​ St George Respiratory Questionnaire (SGRQ),​ Sarcoidosis Assessment Tool (SAT).RESULTSNicotine treatment was associated with a clinically significant,​ approximately 2.1% (70 ml),​ improvement in FVC from baseline to 26 weeks. FVC decreased by a similar amount (2.2%) in the placebo group,​ with a net increase of 140 ml (95% CI: 10,​ 260) when comparing nicotine versus placebo treatment groups at 26 weeks. FEV1 and FAS improved marginally in the nicotine-treated group,​ compared to those on placebo. No improvement was observed in LTS,​ FAS,​ SGRQ or SAT. There were no reported serious adverse events or evidence of nicotine addiction.INTERPRETATIONNicotine treatment was well-tolerated in patients with active pulmonary sarcoidosis,​ and the preliminary findings of this pilot study suggest that it may reduce disease progression,​ based on FVC. |
| 14 | Vega,​ Jennifer N,​ Albert,​ Kimberly M,​ Mayer,​ Ingrid A,​ Taylor,​ Warren D,​ Newhouse,​ Paul A. Subjective cognition and mood in persistent chemotherapy-related cognitive impairment. Journal of cancer survivorship : research and practice. 2021. Epub ahead of print.  **Abstract:** PURPOSEPersistent chemotherapy-related cognitive impairment (CRCI) is commonly reported following cancer treatment and negatively affects quality of life. While past research has focused on potential pathophysiological mechanisms underlying this relationship,​ the role of psychological factors,​ such as mood,​ stress,​ and anxiety,​ in the development of persistent CRCI has received less attention. As an additional analysis of data from a trial investigating the effects of transdermal nicotine patches on cognitive performance in patients with persistent CRCI,​ we examined whether change in mood was associated with changes in subjective and objective cognitive functioning.METHODSParticipants were randomized to either placebo (n = 11) or transdermal nicotine (n = 11) for 6 weeks,​ followed by 2 weeks of treatment withdrawal for a total of 8 weeks. Participants were assessed using behavioral,​ subjective,​ and objective measures of cognitive functioning and mood at five visits before,​ during,​ and after treatment.RESULTSAlthough we did not detect an effect of treatment assignment on mood,​ over the course of the study,​ we observed a significant improvement on measures of mood that correlated with improvement in subjective and objective cognitive performance.CONCLUSIONSWe observed improvement in objective and subjective cognitive performance measures. These changes were associated with improvement in subsyndromal mood symptoms,​ likely resulting from participation in the trial itself.IMPLICATIONS FOR CANCER SURVIVORSThese results suggest that women with persistent CRCI may benefit from support and validation of their cognitive complaints,​ cognitive rehabilitation/therapies into their post-cancer care.TRIAL REGISTRATIONThe study was registered with clinicaltrials.gov (trial registration: NCT02312943). |
| 18 | Jackson,​ Christopher D,​ Carter,​ Jocelyn,​ Kansagara,​ Devan. E-Cigarettes Versus Nicotine Replacement Therapy for Smoking Cessation : Hajek P,​ Phillips-waller A,​ Przulj D,​ et al. A Randomized Trial of E-Cigarettes versus Nicotine-Replacement Therapy. N Engl J Med. 2019;380(7):629-637. Journal of general internal medicine. 2021;36:1481.  **Abstract:** |
| 21 | Cook,​ Jessica W,​ Baker,​ Timothy B,​ Fiore,​ Michael C,​ Collins,​ Linda M,​ Piper,​ Megan E,​ Schlam,​ Tanya R,​ Bolt,​ Daniel M,​ Smith,​ Stevens S,​ Zwaga,​ Deejay,​ Jorenby,​ Douglas E,​ Mermelstein,​ Robin. Evaluating four motivation-phase intervention components for use with primary care patients unwilling to quit smoking: a randomized factorial experiment. Addiction (Abingdon,​ England). 2021. Epub ahead of print.  **Abstract:** AIMSTo assess the effectiveness of intervention components designed to increase quit attempts and promote abstinence in patients initially unwilling to quit smoking.DESIGNA four-factor,​ randomized factorial experiment.SETTINGSixteen primary care clinics in southern Wisconsin.PARTICIPANTSA total of 577 adults who smoke (60% women,​ 80% White) recruited during primary care visits who were currently willing to reduce their smoking but unwilling to try to quit. Interventions Four factors contrasted intervention components administered over a 1-year period: (i) nicotine mini-lozenge versus none; (ii) reduction counseling versus none; (iii) behavioral activation (BA) counseling versus none; and (iv) motivational 5Rs counseling versus none. Participants could request cessation treatment at any time.MEASUREMENTSThe primary outcome was 7-day point-prevalence abstinence at 52 weeks post enrollment; secondary outcomes were point-prevalence abstinence at 26 weeks and making a quit attempt by weeks 26 and 52.FINDINGSNo abstinence main effects were found but a mini-lozenge × reduction counseling × BA interaction was found at 52 weeks; P = 0.03. Unpacking this interaction showed that the mini-lozenge alone produced the highest abstinence rate (16.7%); combining it with reduction counseling produced an especially low abstinence rate (4.1%). Reduction counseling decreased the likelihood of making a quit attempt by 52 weeks relative to no reduction counseling (P = 0.01).CONCLUSIONSNicotine mini-lozenges may increase smoking abstinence in people initially unwilling to quit smoking,​ but their effectiveness declines when used with smoking reduction counseling or other behavioral interventions. Reduction counseling decreases the likelihood of making a quit attempt in people initially unwilling to quit smoking. |
| 24 | Beard, Emma, Jackson, Sarah E, Anthenelli, Robert M, Benowitz, Neal L, Aubin, Lisa St, McRae, Thomas, Lawrence, David, Russ, Cristina, Krishen, Alok, Evins, A Eden, West, Robert. Estimation of risk of neuropsychiatric adverse events from varenicline,bupropion and nicotine patch versus placebo: secondary analysis of results from the EAGLES trial using Bayes factors. Addiction (Abingdon,England). 2021; 116(10):2816.  **Abstract:** BACKGROUND AND AIMSAnalysed using classical frequentist hypothesis testing with alpha set to 0.05,​ the Evaluating Adverse Events in a Global Smoking Cessation Study (EAGLES) did not find enough evidence to reject the hypothesis of no difference in neuropsychiatric adverse events (NPSAEs) attributable to varenicline,​ bupropion,​ or nicotine patch compared with placebo. This might be because the null hypothesis was true or because the data were insensitive. The present study aimed to test the hypothesis more directly using Bayes factors.DESIGNEAGLES was a randomised,​ double-blind,​ triple-dummy,​ controlled trial.SETTINGGlobal (16 countries across five continents),​ between November 2011 and January 2015.PARTICIPANTSParticipants were smokers with (n = 4116) and without (n = 4028) psychiatric disorders.INTERVENTIONSVarenicline (1 mg twice daily),​ bupropion (150 mg twice daily),​ nicotine patch (21 mg once daily with taper) and matched placebos.MEASUREMENTSThe outcomes included: (i) a composite measure of moderate/severe NPSAEs; and (ii) a composite measure of severe NPSAEs. The relative evidence for there being no difference in NPSAEs versus data insensitivity for the medications was calculated in the full and sub-samples using Bayes factors and corresponding robustness regions.FINDINGSFor all but two comparisons,​ Bayes factors were <1/3,​ indicating moderate to strong evidence for no difference in risk of NPSAEs between active medications and placebo (Bayes factor = 0.02-0.23). In the psychiatric cohort versus placebo,​ the data were suggestive,​ but not conclusive of no increase in NPSAEs with varenicline (Bayes factor = 0.52) and bupropion (Bayes factor = 0.71). Here,​ the robustness regions ruled out a ≥7% and ≥8% risk increase with varenicline and bupropion,​ respectively.CONCLUSIONSSecondary analysis of the Evaluating Adverse Events in a Global Smoking Cessation Study trial using Bayes factors provides moderate to strong evidence that use of varenicline,​ bupropion or nicotine patches for smoking cessation does not increase the risk of neuropsychiatric adverse events relative to use of placebo in smokers without a history of psychiatric disorder. For smokers with a history of psychiatric disorder the evidence also points to no increased risk but with less confidence. |
| 27 | Kim,​ Nayoung,​ McCarthy,​ Danielle E,​ Piper,​ Megan E,​ Baker,​ Timothy B. Comparative effects of varenicline or combination nicotine replacement therapy versus patch monotherapy on candidate mediators of early abstinence in a smoking cessation attempt. Addiction (Abingdon,​ England). 2021;116:926.  **Abstract:** BACKGROUND AND AIMSThe phase-based model of smoking cessation treatment suggests that treatment needs may vary across phases (e.g. pre-cessation,​ cessation). This study tested the comparative effects of varenicline and combination nicotine replacement therapy (C-NRT) relative to nicotine patch monotherapy on pre-cessation and cessation phase candidate withdrawal,​ expectancy and motivation mediators; relations between mediators and abstinence; and indirect effects of enhanced treatments on abstinence via candidate mediators.DESIGNSecondary mediation analysis of data from the open-label,​ randomized Wisconsin Smokers' Health Study 2,​ a comparative effectiveness trial of varenicline or C-NRT,​ versus patch monotherapy,​ in adults who smoked,​ recruited via media and community outreach.SETTINGResearch clinics in Madison and Milwaukee,​ Wisconsin,​ USA.PARTICIPANTSA total of 1051 daily smokers motivated to quit smoking (52.5% female; mean age = 48.1,​ standard deviation = 11.6).INTERVENTIONSTwelve weeks of varenicline (n = 407) or 12 weeks of combination nicotine patch and nicotine lozenge therapy (n = 421),​ both compared with 12 weeks of patch control condition (n = 230),​ with individual smoking cessation counseling.MEASUREMENTSThe primary abstinence outcome was biochemically verified 7-day point-prevalence abstinence 4 weeks post-target quit day (TQD). Candidate mediators (craving,​ positive smoking expectancies,​ withdrawal symptoms,​ and quitting motivation) were assessed via ecological momentary assessment from 1 week prior (pre-cessation phase) to 4 weeks after (cessation phase) the TQD.FINDINGSPre-cessation and cessation mean levels and slopes of craving [adjusted odds ratio (aOR) = 0.34-0.79],​ smoking expectancies (aOR = 0.46-0.79) and quitting motivation (aOR = 1.35-7.21) significantly predicted 4-week post-TQD abstinence (P < 0.05). Significant varenicline mediation occurred via greater suppression in pre-cessation craving [mediated effect (ab) = 0.09,​ standard error (SE) = 0.03,​ 95% confidence interval (CI) = 0.04-0.14] and smoking expectancies (ab = 0.06,​ SE = 0.02,​ 95% CI = 0.02-0.12). C-NRT mediation occurred via greater reduction in pre-post-TQD changes in craving (ab = 0.04,​ SE = 0.02,​ 95% CI = 0.01-0.08) and expectancies (ab = 0.03,​ SE = 0.02,​ 95% CI = 0.001-0.07),​ relative to patch monotherapy.CONCLUSIONAmong adult smokers seeking to quit,​ varenicline seems to work through its effects on suppression of craving and smoking expectancies pre-cessation while combination nicotine replacement therapy mediation seems to work through cessation-related reduction in craving and smoking expectancies changes. |
| 30 | Brett,​ Emma I,​ Chavarria,​ Jesus,​ Liu,​ Melissa,​ Hedeker,​ Donald,​ King,​ Andrea C. Effects of a brief motivational smoking intervention in non-treatment seeking disadvantaged Black smokers. Journal of consulting and clinical psychology. 2021;89:241.  **Abstract:** Objective: While the U.S. adult smoking rate has declined,​ Black smokers disproportionately face more barriers to accessing brief effective tobacco cessation treatments compared with other racial groups. This study developed and tested the effects of a novel,​ evidence-based,​ brief smoking intervention culturally targeted for disadvantaged Black smokers (ClinicalTrials.gov ID: NCT04460417). Method: In this randomized controlled trial,​ primarily low-income Black non-treatment-seeking smokers (N = 204,​ 51% female) were randomized to enhanced care (EC) or treatment as usual (TAU). The EC group received a 30-min session with personal feedback on smoking,​ education on health outcomes and tobacco advertising targeting Black smokers,​ and nicotine replacement therapy (NRT) starter kits. TAU included provision of self-help materials. Primary outcome was motivation to change smoking behavior,​ and secondary outcomes included NRT knowledge and use,​ quit attempts,​ and number of cigarettes smoked per day. Data were collected at baseline,​ 1- and 6-month follow-ups. Results: Compared with TAU,​ EC increased motivation to change (p = .02),​ accuracy in NRT knowledge,​ (p < .001),​ NRT use (p = .01),​ and likelihood of making a serious quit attempt as well as reduced cigarettes smoked per day (p < .01) through 6-month follow-up. Conclusions: A brief motivational intervention for Black non-treatment-seeking smokers increased motivation to change smoking and resulted in improvements in NRT knowledge,​ use,​ and quit-relevant behaviors. Findings support cultural-targeting and provision of NRT to enhance motivation in Black smokers to reduce cultural and institutional barriers to tobacco cessation. (PsycInfo Database Record (c) 2021 APA,​ all rights reserved). |
| 32 | Correa, John B, Lawrence, David, McKenna, Benjamin S, Gaznick, Natassia, Saccone, Phillip A, Dubrava, Sarah, Doran, Neal, Anthenelli, Robert M. Psychiatric co-morbidity and multi-morbidity in the EAGLES trial: Descriptive correlates and associations with neuropsychiatric adverse events, treatment adherence, and smoking cessation. Nicotine & tobacco research: official journal of the Society for Research on Nicotine and Tobacco. 2021; 23(10):1646.  **Abstract:** INTRODUCTIONPsychiatric and substance use disorders represent barriers to smoking cessation. We sought to identify correlates of psychiatric co-morbidity (2 diagnoses) and multi-morbidity (3+ diagnoses) among smokers attempting to quit and to evaluate whether these conditions predicted neuropsychiatric adverse events (NPSAEs),​ treatment adherence,​ or cessation efficacy (CE).METHODData were collected from November 2011 to January 2015 across sixteen countries and reflect the psychiatric cohort of the EAGLES trial. Participants were randomly assigned to receive varenicline,​ bupropion,​ nicotine replacement therapy,​ or placebo for 12 weeks and were followed for an additional 12 weeks post-treatment. NPSAE outcomes reflected sixteen moderate-to-severe neuropsychiatric symptom categories,​ and CE outcomes included continuous abstinence at weeks 9-12 and weeks 9-24.RESULTSOf the 4103 participants included,​ 36.2% were diagnosed with multiple psychiatric conditions (20.9% co-morbidity,​ 15.3% multi-morbidity). Psychiatric co- and multi-morbidity were associated with several baseline factors,​ including male gender,​ non-white race/ethnicity,​ more previous quit attempts,​ and more severe mental health symptoms. The incidence of moderate-to-severe NPSAEs was significantly higher (p<0.01) in participants with multi-morbidity (11.9%) than those with co-morbidity (5.1%) or primary diagnosis only (4.6%). There were no significant (ps>0.05) main effects or interactions with treatment condition for diagnostic grouping on treatment adherence or CE outcomes.CONCLUSIONSWhile having multiple psychiatric diagnoses increased risk of developing moderate-to-severe NPSAEs during a quit attempt,​ neither co- nor multi-morbidity were associated with treatment adherence or odds of quitting. These findings reassure providers to advise smokers with multiple stable psychiatric conditions to consider using FDA-approved medications when trying to quit. |
| 35 | Fernandes,​ Thiago P,​ Almeida,​ Natalia L,​ Silva,​ Gabriella M,​ Santos,​ Natanael A. Nicotine gum enhances visual processing in healthy nonsmokers. Brain imaging and behavior. 2021. Epub ahead of print.  **Abstract:** OBJECTIVEThe main purpose of this study was to investigate the isolated effects of nicotine on visual processing,​ namely contrast processing.METHODSThirteen participants,​ aged 18-40 years,​ were enrolled in this double blind,​ randomized and pilot controlled trial involving nicotine gum administration (placebo,​ 2-mg and 4-mg doses). The participants' instruction was to detect the location of vertical gratings (0.2; 1.0; 3.3; 5.7; 8.8; 13.2 and 15.9 cycles per degree) when it was presented either left or right on the monitor screen. A repeated multivariate analysis of variance was conducted to analyse the results for the visual processing tasks. Bayesian analyses were also carried out considering maximum robustness to avoid bias.RESULTSThe findings that nicotine gum administration resulted in better contrast discrimination when compared to placebo gum (p < .001). More specifically,​ the 4-mg resulted in better visual sensitivity when compared to the 2-mg (p < .01) and the placebo (p < .001) gum. Demographic data were not related to the outcomes.CONCLUSIONSThese data bring the need for support the findings. If proved,​ it is possible that nicotine,​ in small doses,​ can have a potential therapeutic use for those populations with low vision.TRIAL REGISTRATION NUMBERRBR-46tjy3. |
| 42 | Sahr, Michelle, Kelsh, Shelby, Blower, Noah, Sohn, Minji. Pilot Study of Electronic Nicotine Delivery Systems (ENDS) Cessation Methods. Pharmacy (Basel,Switzerland). 2021; 9(1):21.  **Abstract:** Currently,​ 7.6% of the U.S. young adults aged 18-24 years old use e-cigarettes. This study piloted three methods of Electronic Nicotine Delivery Systems (ENDS) cessation by measuring cessation rates,​ motivational techniques that contributed to cessation success,​ and participants' changes after decreasing vape use. Participants were randomized into three study arms (nicotine replacement therapy (NRT) + behavioral support,​ vape-taper + behavioral support,​ self-guided) in a 1:1:1 ratio. All participants were invited to attend nine in-person or phone appointments over the 6-month study period. At 12 weeks,​ 3 of 7 (42.9%) participants in the NRT + behavioral support arm,​ 6 of 8 (75%) vape-taper + behavioral support arm,​ and 7 of 9 (77.8%) self-guided arm self-reported being vape-free and nicotine-free. At 6 months,​ 3 of 7 (42.9%) participants in the NRT + behavioral support arm,​ 6 of 8 (75%) vape-taper + behavioral support arm,​ and 4 of 9 (44.4%) self-guided arm self-reported being vape-free and nicotine-free. A challenge to quitting and remain quit is social pressures,​ but participants identified self-control and establishing new habits to be the best methods to overcome the desire to vape. Participants who received behavioral support and a vape-taper plan from pharmacists were more likely to be vape-free and nicotine-free at 6 months. |
| 53 | Nollen,​ Nicole L,​ Ahluwalia,​ Jasjit S,​ Sanderson Cox,​ Lisa,​ Okuyemi,​ Kolawole,​ Lawrence,​ David,​ Samuels,​ Larry,​ Benowitz,​ Neal L. Assessment of Racial Differences in Pharmacotherapy Efficacy for Smoking Cessation: Secondary Analysis of the EAGLES Randomized Clinical Trial. JAMA network open. 2021;4:e2032053.  **Abstract:** ImportanceUnderstanding Black vs White differences in pharmacotherapy efficacy and the underlying reasons is critically important to reducing tobacco-related health disparities.ObjectiveTo compare pharmacotherapy efficacy and examine variables to explain Black vs White differences in smoking abstinence.Design,​ Setting,​ and ParticipantsThis study is a secondary analysis of the Evaluating Adverse Events in a Global Smoking Cessation Study (EAGLES) double-blind,​ placebo-controlled,​ randomized clinical trial,​ which took place at clinical trial centers,​ academic centers,​ and outpatient clinics in 29 states in the US. US Black and White smokers who smoked 10 or more cigarettes per day with and without psychiatric comorbidity were enrolled between November 2011 and January 2015. Data analysis was performed from July 2019 to January 2020.InterventionsParticipants were randomized (1:1:1:1) in a double-blind,​ triple-dummy,​ placebo- and active-controlled (nicotine patch) trial of varenicline and bupropion for 12 weeks with follow-up through week 24.Main Outcomes and MeasuresBiochemically verified continuous cigarette abstinence rate (CAR) from weeks 9 to 24. Baseline,​ postbaseline treatment,​ and safety characteristics were examined as variables to explain race differences in abstinence.ResultsOf the 1065 Black smokers enrolled,​ 255 were randomized to receive varenicline,​ 259 received bupropion,​ 286 received nicotine replacement therapy (NRT [ie,​ nicotine patch]),​ and 265 received placebo. Among the 3044 White smokers enrolled,​ 778 were randomized to receive varenicline,​ 769 received bupropion,​ 738 received NRT,​ and 759 received placebo. Participants were predominantly female (614 Black [57.7%] and 1786 White [58.7%] women) and heavy smokers (mean [SD] cigarettes per day,​ 18.2 [7.9] for Black and 20.0 [7.5] for White smokers),​ with a mean (SD) age of 47.2 (11.2) years for Black and 46.5 (12.7) years for White participants. Treatment and race were associated with CAR for weeks 9 to 24. The CAR was 4.9% lower for Black vs White participants (odds ratio [OR],​ 0.53; 95% CI,​ 0.41-0.69; P < .001); differences were found across all treatments. Pooling psychiatric and nonpsychiatric cohorts,​ varenicline (OR,​ 2.63; 95% CI,​ 1.90-3.63; P < .001),​ bupropion (OR,​ 1.75; 95% CI,​ 1.25-2.46; P = .001),​ and NRT (OR,​ 1.52; 95% CI,​ 1.07-2.16; P = .02) had greater efficacy than placebo for White participants. Only varenicline (OR,​ 2.63; 95% CI,​ 1.26-5.48; P = .01) had greater efficacy than placebo for Black participants. Baseline,​ postbaseline,​ and safety characteristics differed by race,​ but these variables did not eliminate the association of race with CAR. Black participants had 49% reduced odds of CAR for weeks 9 to 24 compared with White participants in the adjusted model (OR,​ 0.51; 95% CI,​ 0.39-0.66; P < .001).Conclusions and RelevanceBlack and White smokers achieved the highest rate of abstinence while taking varenicline,​ suggesting that it is the best first-line therapy for these groups. However,​ Black smokers were less responsive to all therapies,​ including placebo. Understanding variables (eg,​ socioeconomic or biological) beyond those may lead to improved treatment outcomes for Black smokers.Trial RegistrationClinicalTrials.gov Identifier: NCT01456936. |
| 54 | Smaily,​ Hussein,​ Khalaf,​ Michel,​ Melkane,​ Antoine E,​ Helou,​ Diane,​ Richa,​ Tony,​ Khoury,​ Clement,​ Azoury,​ Fares,​ Farha,​ Georges,​ Haddad,​ Amine,​ Matar,​ Nayla. Smoking cessation intervention for patients with head and neck cancer: A prospective randomized controlled trial. American journal of otolaryngology. 2021;42:102832.  **Abstract:** AIMSTo evaluate the effectiveness of a brief smoking cessation intervention in head and neck cancer patients (HNCP).STUDY DESIGNA prospective randomized controlled trial that randomly assigns participants in two groups: a usual care group (UCG),​ and a standardized intervention group (SIG).MATERIAL AND METHODSPatients with a confirmed diagnosis of head and neck squamous cell carcinoma (HNSCC) and who are active smokers were prospectively approached by one of 4 trained Ear-nose-throat (ENT) residents. Participants were randomized into a UCG,​ and a SIG consisting of a brief perioperative smoking cessation intervention based on National Institute of Health (NIH) "5A's" model along with an informative motivational document and nicotine patch therapy (NPT) offered for 8 weeks in gradually decreasing doses.OBJECTIVEThe evaluation of abstinence at 3,​ 6 and 12 months after enrollment.RESULTS56 subjects were randomized into the UCG (N = 29,​ 52%),​ and the SIG (N = 27,​ 48%). The overall smoking cessation rates were not statistically different between the two groups; we observed at 3 months cessation rates of 57.1% vs. 57.7% (p = 0.96); at 6 months,​ 42.9% vs. 24% (p = 0.148); and at 12 months,​ 33.3% vs. 20.8% (p = 0.318),​ for the UCG and the SIG respectively.CONCLUSIONThis study failed to show the effectiveness of a combined brief smoking cessation approach led by and ENT resident in HNSCC patients. A multifaceted approach addressing different pharmacological treatments,​ factors contributing to smoking maintenance,​ mainly alcohol dependence and mood disturbances and dealing with relapse risks through close face-to-face or phone call follow-ups may have better outcomes and should be evaluated in upcoming trials. |
| 56 | Fernandes,​ Thiago P,​ Butler,​ Pamela D,​ Rodrigues,​ Stephanye J,​ Silva,​ Gabriella M,​ Anchieta,​ Marcos V,​ Souto,​ Jandirlly J S,​ Gomes,​ Giulliana H V,​ Almeida,​ Natalia L,​ Santos,​ Natanael A. Short-term effects of nicotine gum on facial detection in healthy nonsmokers: a pilot randomized controlled trial. Journal of addictive diseases. 2021;39:15.  **Abstract:** Objective: The main purpose of this study was to investigate short-term effects of nicotine gum on facial detection. Methods: Fourteen participants (mean age = 26.8 years,​ SD = 2.5 years; eight males) were enrolled in this pilot randomized controlled trial of nicotine gum administration (placebo,​ 2-mg and 4-mg doses). The participants were instructed to detect the location of a face when it was presented in a face/nonface pair on the screen. A repeated multivariate analysis of variance was conducted to analyze the results for reaction time and discrimination index. Demographics were used to explore significant association on facial detection. Bayesian analyses were also carried out considering maximum robustness to avoid bias. Results: The results indicated that the 2-mg dose resulted in faster reaction time and better discrimination than the 4-mg dose (p < 0.001). The 4-mg dose resulted in slower reaction time and lower discrimination index compared to both placebo (p < 0.01) and 2-mg doses (p < 0.001). Demographic data were not related to the outcomes. Conclusions: The results indicate that nicotine improved facial detection,​ but only at low doses (i.e.,​ 2-mg),​ following a U-shaped curve. We trust future studies will continue to advance this research field,​ and if further work supports these preliminary findings,​ nicotine can act as therapeutic target in populations such as those with low vision. |
| 58 | Silvestri, Nathaniel J, Dahne, Jennifer, Wahlquist, Amy E, Toll, Benjamin, Carpenter, Matthew J. Does Medication Sampling Improve Compliance with Brief Advice? Results from a Pragmatic Randomized Clinical Trial. Journal of smoking cessation. 2021; 2021:6638872.  **Abstract:** IntroductionThe 5As model is a standard component of most guidelines for tobacco treatment. Unfortunately,​ provider adherence to this model is modest.AimsProviding physicians with adjunctive tools to adhere to 5As guidelines may serve as a catalyst for brief advice delivery.MethodsThis was a secondary data analysis of a cluster randomized clinical trial assessing the uptake and impact of free nicotine replacement therapy (NRT) sampling versus standard care in primary care. Patients reported receipt of separate elements of the 5As model,​ assessed one month following a baseline visit. Analyses compared patients who recalled receipt of brief advice among those who received NRT vs. standard care. Additional analyses examined demographic predictors of receiving brief advice.Results/FindingsMedication sampling did not improve compliance with ask,​ advise,​ or assess. Receipt of "assistance" was significantly higher among NRT recipients (70%) (p ≤ 0.0001). The NRT sampling group was more likely to have received all components (p = 0.004). As age increased,​ being asked (p = 0.006),​ advised (p = 0.05),​ and assessed (p = 0.003) decreased. Non-Whites reported higher rates of assessment (p = 0.02).ConclusionsProvision of NRT sampling increased provider compliance with some elements of the brief advice model,​ thus enhancing the impact of cessation advice within primary care.Trial RegistrationThis trial is registered with ClinicalTrials.gov NCT02096029. |
| 59 | Evins,​ A Eden,​ West,​ Robert,​ Benowitz,​ Neal L,​ Russ,​ Cristina,​ Lawrence,​ David,​ McRae,​ Thomas,​ Maravic,​ Melissa Culhane,​ Heffner,​ Jaimee L,​ Anthenelli,​ Robert M. Efficacy and Safety of Pharmacotherapeutic Smoking Cessation Aids in Schizophrenia Spectrum Disorders: Subgroup Analysis of EAGLES. Psychiatric services (Washington,​ D.C.). 2021;72:7.  **Abstract:** OBJECTIVEThis study aimed to evaluate the efficacy and safety of varenicline,​ bupropion,​ and nicotine replacement therapy (NRT) among smokers with schizophrenia spectrum disorders in post hoc analyses of Evaluating Adverse Events in a Global Smoking Cessation Study data.METHODSSmokers with schizophrenia spectrum disorder (N=390) and without a psychiatric illness (control group,​ N=4,​028) were randomly assigned to receive varenicline,​ bupropion,​ NRT patch,​ or placebo for 12 weeks. Outcomes included abstinence rates during treatment and follow-up,​ number needed to treat (NNT) for abstinence,​ incidence of neuropsychiatric adverse events (NPSAEs),​ and temporal relationship between NPSAEs and abstinence status.RESULTSSmokers with schizophrenia smoked more and had greater dependence and fewer prior trials of cessation pharmacotherapy at baseline. At each time point,​ smokers with schizophrenia assigned to varenicline had significantly greater odds of abstinence compared with their matched placebo group,​ with NNT comparable to the control group. Bupropion and NRT increased odds of abstinence; confidence intervals (CIs) included 1 for some comparisons,​ and NNT for smokers with schizophrenia was greater than for the control group. No treatment was associated with significantly more NPSAEs,​ compared with placebo,​ in either cohort. The estimated NPSAE rate was 5% (95% CI=3.0-7.7) for smokers with schizophrenia and 1% (95% CI=0.6-2.1) for the control group. Over one-third of NPSAEs occurred during partial or full abstinence,​ suggesting a multifactorial nature.CONCLUSIONSFor smokers with schizophrenia,​ varenicline led to significantly higher abstinence rates,​ and NNT was comparable to the control group. A significant proportion of NPSAEs occurred during early abstinence. No treatment significantly increased NPSAE prevalence. |
| 64 | Chen,​ Li-Shiun,​ Baker,​ Timothy B,​ Miller,​ J Philip,​ Bray,​ Michael,​ Smock,​ Nina,​ Chen,​ Jingling,​ Stoneking,​ Faith,​ Culverhouse,​ Robert C,​ Saccone,​ Nancy L,​ Amos,​ Christopher I,​ Carney,​ Robert M,​ Jorenby,​ Douglas E,​ Bierut,​ Laura J. Genetic Variant in CHRNA5 and Response to Varenicline and Combination Nicotine Replacement in a Randomized Placebo-Controlled Trial. Clinical pharmacology and therapeutics. 2020;108:1315.  **Abstract:** It is unclear if genetic variants affect smoking cessation treatment response. This study tested whether variants in the cholinergic receptor nicotinic alpha 5 subunit (CHRNA5) predict response to smoking cessation medication by directly comparing the two most effective smoking cessation pharmacotherapies. In this genotype-stratified randomized,​ double-blind,​ placebo-controlled clinical trial (May 2015-August 2019 in St Louis,​ Missouri),​ smokers were randomized by genotype in blocks of six (1:1:1 ratio) to three conditions: 12 weeks of placebo (n = 273),​ combination nicotine patch and lozenge (combination nicotine replacement therapy,​ cNRT,​ n = 275),​ or varenicline (n = 274). All participants received counseling and were followed for 12 months. The primary end point was biochemically verified 7-day point prevalence abstinence at the end of treatment (EOT,​ week 12). Trial registration and eligibility criteria are on clinicaltrials.gov (https://clinicaltrials.gov/) (NCT02351167). We conducted the genetic analyses separately for 516 European ancestry (EA) smokers and 306 non-EA smokers (including 270 African American smokers). In African American smokers,​ there was a genotype-by-treatment interaction for EOT abstinence (χ2 = 10.7,​ degrees of freedom = 2. P = 0.0049): specifically,​ cNRT was more effective in smokers with rs16969968 GG genotype than was placebo,​ while varenicline was more effective in smokers of GA/AA genotypes. In EA ancestry smokers,​ there was no significant genotype-by-treatment interaction. In the whole sample,​ although both were effective at EOT,​ only varenicline,​ and not cNRT,​ was significantly effective relative to placebo at 6-month follow-up. Importantly,​ this study suggests that genetic information can further enhance smoking cessation treatment effectiveness. |
| 67 | Goldenson,​ Nicholas I,​ Buchhalter,​ August R,​ Augustson,​ Erik M,​ Rubinstein,​ Mark L,​ Van Hoof,​ Dennis,​ Henningfield,​ Jack E. Abuse liability assessment of the JUUL system in two nicotine concentrations compared to combustible cigarette,​ nicotine gum and comparator electronic nicotine delivery system. Drug and alcohol dependence. 2020;217:108441.  **Abstract:** BACKGROUNDTo assess the abuse liability of the JUUL System (JS) in 5.0 % (59 mg/mL) and 3.0 % (35 mg/mL) nicotine concentrations.METHODSAdult smokers (N = 146; 45.9 % female; mean age = 41.29 years) were randomized to one of four study flavor arms and then to a within-subjects cross-over sequence for five test product categories: (1) JS 5.0 % nicotine concentration; (2) JS 3.0 % nicotine; (3) usual brand (UB) cigarette; (4) 4 mg mint nicotine gum; (5) comparator ENDS (VUSE Alto 5.0 % nicotine). Products were tested by ad libitum use (5 min for ENDS and cigarette; 30 min for gum); nicotine pharmacokinetic (PK) parameters and subjective effects were assessed following use.RESULTSMaximum plasma nicotine concentration (Cmax-BL),​ rate of plasma nicotine rise and total nicotine exposure (AUC0-60-BL) of UB cigarette were significantly greater than all other test products. The comparator ENDS was significantly greater than 5.0 % and 3.0 % JS and nicotine gum on Cmax-BL,​ rate of plasma nicotine rise,​ and AUC0-60-BL; Cmax-BL of JS 5.0 % was significantly greater than JS 3.0 % and nicotine gum. Product liking and satisfying effects were significantly highest for the UB cigarette; JS products and comparator ENDS did not significantly differ and were rated higher than nicotine gum on most subjective measures.CONCLUSIONSThese results suggest that the abuse liability of both 5.0 % and 3.0 % JS is: (1) substantially lower than UB cigarette; (2) somewhat lower than comparator ENDS; and (3) higher than nicotine gum. Additionally,​ the abuse liability of JS 5.0 % is somewhat higher than JS 3.0 %. |
| 68 | McRobbie,​ Hayden J,​ Phillips-Waller,​ Anna,​ El Zerbi,​ Catherine,​ McNeill,​ Ann,​ Hajek,​ Peter,​ Pesola,​ Francesca,​ Balmford,​ James,​ Ferguson,​ Stuart G,​ Li,​ Lin,​ Lewis,​ Sarah,​ Courtney,​ Ryan J,​ Gartner,​ Coral,​ Bauld,​ Linda,​ Borland,​ Ron. Nicotine replacement treatment,​ e-cigarettes and an online behavioural intervention to reduce relapse in recent ex-smokers: a multinational four-arm RCT. Health technology assessment (Winchester,​ England). 2020;24:1.  **Abstract:** BACKGROUNDRelapse remains an unresolved issue in smoking cessation. Extended stop smoking medication use can help,​ but uptake is low and several behavioural relapse prevention interventions have been found to be ineffective. However,​ opportunistic 'emergency' use of fast-acting nicotine replacement treatment or electronic cigarettes may be more attractive and effective,​ and an online behavioural Structured Planning and Prompting Protocol has shown promise. The present trial aimed to evaluate the clinical effectiveness and cost-effectiveness of these two interventions.DESIGNA randomised controlled trial.SETTINGEnglish stop smoking services and Australian quitlines,​ Australian social media and St Vincent's Hospital Melbourne,​ Fitzroy,​ VIC.PARTICIPANTSEx-smokers abstinent for at least 4 weeks,​ with some participants in Australia also recruited from 1 week post quit date. The planned sample size was 1400,​ but the trial was curtailed when 235 participants were recruited.INTERVENTIONSParticipants were randomised in permuted blocks of random sizes to (1) oral nicotine replacement treatment/electronic cigarettes to use if at risk of relapse,​ plus static text messages (n = 60),​ (2) the Structured Planning and Prompting Protocol and interactive text messages (n = 57),​ (3) oral nicotine replacement treatment/electronic cigarettes plus the Structured Planning and Prompting Protocol with interactive text messages (n = 58) or (4) usual care plus static text messages (n = 59).OUTCOME MEASURESOwing to delays in study set-up and recruitment issues,​ the study was curtailed and the primary outcome was revised. The original objective was to determine whether or not the two interventions,​ together or separately,​ reduced relapse rates at 12 months compared with usual care. The revised primary objective was to determine whether or not number of interventions received (i.e. none,​ one or two) affects relapse rate at 6 months (not biochemically validated because of study curtailment). Relapse was defined as smoking on at least 7 consecutive days,​ or any smoking in the last month at final follow-up for both the original and curtailed outcomes. Participants with missing outcome data were included as smokers. Secondary outcomes included sustained abstinence (i.e. no more than five cigarettes smoked over the 6 months),​ nicotine product preferences (e.g. electronic cigarettes or nicotine replacement treatment) and Structured Planning and Prompting Protocol coping strategies used. Two substudies assessed reactions to interventions quantitatively and qualitatively. The trial statistician remained blinded until analysis was complete.RESULTSThe 6-month relapse rates were 60.0%,​ 43.5% and 49.2% in the usual-care arm,​ one-intervention arm and the two-intervention arm,​ respectively (p = 0.11). Sustained abstinence rates were 41.7%,​ 54.8% and 50.9%,​ respectively (p = 0.17). Electronic cigarettes were chosen more frequently than nicotine replacement treatment in Australia (71.1% vs. 29.0%; p = 0.001),​ but not in England (54.0% vs. 46.0%; p = 0.57). Of participants allocated to nicotine products,​ 23.1% were using them daily at 6 months. The online intervention received positive ratings from 63% of participants at 6 months,​ but the majority of participants (72%) completed one assessment only. Coping strategies taught in the Structured Planning and Prompting Protocol were used with similar frequency in all study arms,​ suggesting that these are strategies people had already acquired. Only one participant used the interactive texting,​ and interactive and static messages received virtually identical ratings.LIMITATIONSThe inability to recruit sufficient participants resulted in a lack of power to detect clinically relevant differences. Self-reported abstinence was not biochemically validated in the curtailed trial,​ and the ecological momentary assessment substudy was perceived by some as an intervention.CONCLUSIONSRecruiting recent ex-smokers into an interventional study proved problematic. Both interventions were well received and safe. Combining the interventions did not surpass the effects of each intervention alone. There was a trend in favour of single interventions reducing relapse,​ but it did not reach significance and there are reasons to interpret the trend with caution.FUTURE WORKFurther studies of both interventions are warranted,​ using simpler study designs.TRIAL REGISTRATIONCurrent Controlled Trials ISRCTN11111428.FUNDINGThis project was funded by the National Institute for Health Research (NIHR) Health Technology Assessment programme and will be published in full in Health Technology Assessment; Vol. 24,​ No. 68. See the NIHR Journals Library website for further project information. Funding was also provided by the National Health and Medical Research Council,​ Canberra,​ ACT,​ Australia (NHMRC APP1095880). Public Health England provided the funds to purchase the nicotine products in England. |
| 74 | Mahabee-Gittens, E Melinda, Ammerman, Robert T, Khoury, Jane C, Tabangin, Meredith E, Ding, Lili, Merianos, Ashley L, Stone, Lara, Gordon, Judith S. A Parental Smoking Cessation Intervention in the Pediatric Emergency Setting: A Randomized Trial. International journal of environmental research and public health. 2020; 17(21):8151.  **Abstract:** We examined the efficacy of a pediatric emergency visit-based screening,​ brief intervention,​ and referral to treatment (SBIRT) condition compared to a control condition (Healthy Habits Control,​ HHC) to help parental smokers quit smoking. We enrolled 750 parental smokers who presented to the pediatric emergency setting with their child into a two-group randomized controlled clinical trial. SBIRT participants received brief cessation coaching,​ quitting resources,​ and up to 12-weeks of nicotine replacement therapy (NRT). HHC participants received healthy lifestyle coaching and resources. The primary outcome was point-prevalence tobacco abstinence at six weeks (T1) and six months (T2). The mean (SD) age of parents was 31.8 (7.7) years,​ and 86.8% were female,​ 52.7% were Black,​ and 64.6% had an income of ≤,​000. Overall abstinence rates were not statistically significant with 4.2% in both groups at T1 and 12.9% and 8.3% in the SBIRT and HHC groups,​ respectively,​ at T2. There were statistically significant differences in SBIRT versus HHC participants on the median (IQR) reduction of daily cigarettes smoked at T1 from baseline (-2 [-5,​ 0] versus 0 [-4,​ 0],​ p = 0.0008),​at T2 from baseline (-4 [-9,​ -1] vs. -2 [-5,​ 0],​ p = 0.0006),​ and on the mean (SD) number of quit attempts at T2 from baseline (1.25 (6.5) vs. 0.02 (4.71),​ p = 0.02). Self-reported quitting rates were higher in SBIRT parents who received NRT (83.3% vs. 50.9%,​ p = 0.04). The novel use of the pediatric emergency visit to conduct cessation interventions helped parents quit smoking. The near equivalent abstinence rates in both the SBIRT and HHC groups may be due to underlying parental concern about their child's health. Cessation interventions in this setting may result in adult and pediatric public health benefits. |
| 75 | Shiffman,​ Saul,​ Ferguson,​ Stuart G,​ Mao,​ Jason,​ Scholl,​ Sarah M,​ Hedeker,​ Donald,​ Tindle,​ Hilary A. Effectiveness of nicotine gum in preventing lapses in the face of temptation to smoke among non-daily smokers: a secondary analysis. Addiction (Abingdon,​ England). 2020;115:2123.  **Abstract:** BACKGROUND AND AIMSNon-daily smokers (NDS) comprise a large fraction of US smokers. Despite little or no dependence,​ as typically assessed,​ intermittent smokers (ITS) have difficulty quitting smoking. A randomized clinical trial comparing the effect of nicotine gum with placebo on quitting smoking in non-daily smokers did not find an effect on overall abstinence. We undertook an analysis to assess whether using nicotine gum versus placebo when tempted to smoke could reduce incidence of lapses in those situations.DESIGNWithin a 6-week randomized,​ placebo-controlled clinical trial of nicotine gum,​ analyses contrasted the outcome of temptation episodes where gum was or was not used.SETTINGSmoking cessation research clinic in Pittsburgh,​ PA,​ USA.PARTICIPANTSA total of 255 adult ITS (131 nicotine gum,​ 124 placebo) seeking help for smoking cessation.INTERVENTIONNicotine gum (2 mg) versus placebo for up to 8 weeks,​ with as-needed dosing instructions.MEASUREMENTSOutcome was lapsing in temptation episodes,​ as reported by participants via ecological momentary assessment (EMA). Propensity scores predicting gum use from situational factors (e.g. mood,​ social setting,​ smoking cues) served as a control variable.FINDINGSParticipants reported 2713 temptation episodes,​ 46.0% (1248) of which resulted in smoking (lapsing). There was a significant gum use × active treatment interaction (P = 0.0009). Using nicotine gum decreased the odds of lapsing by 55% compared with using placebo [odds ratio (OR) = 0.45; 0.22-0.94]; when gum was not used,​ the assigned gum condition made no significant difference (OR = 1.53; 0.78-3.01; Bayes factor = 0.14). The nicotine effect was not reliably different when participants were trying to achieve abstinence versus when trying to maintain abstinence (OR = 0.44; 0.10,​ 2.03; P = 0.294; Bayes factor = 0.11),​ for men and women (OR = 1.68; 0.58,​ 4.87; P = 0.343; Bayes factor = 0.10),​ or for participants with some or no dependence (OR = 0.88; 0.30,​ 2.59; P = 0.811; Bayes factor = 0.06).CONCLUSIONSWhen used in response to temptation to smoke,​ 2 mg nicotine gum can help to prevent lapses among non-daily smokers. |
| 78 | Landim,​ F S,​ Laureano Filho,​ J R,​ Nascimento,​ J,​ do Egito Vasconcelos,​ B C. Effectiveness of nicotine patch for the control of pain,​ oedema,​ and trismus following third molar surgery: a randomized clinical trial. International journal of oral and maxillofacial surgery. 2020;49:1508.  **Abstract:** The aim of this study was to evaluate the effectiveness of a nicotine patch for the control of pain,​ oedema,​ and trismus following lower third molar surgery. A prospective,​ randomized,​ triple-blind,​ split-mouth trial was performed involving 20 patients who underwent two surgical procedures at different times. A patch containing 14mg nicotine was used in the experimental group,​ whereas a patch without nicotine (placebo) was used in the control group. The nicotine patch was effective at controlling pain after 4hours and 8hours (P= 0.023 and P= 0.005,​ respectively). The nicotine patch also had a significant effect on the control of oedema at 24 hours (P= 0.002),​ 48 hours (P= 0.001),​ and 72 hours (P= 0.005) following the intervention. Postoperative mouth opening was significantly greater among the patients who received the nicotine patch after 72 hours and 7 days. The number of rescue analgesics required was lower (P= 0.026) and the level of satisfaction was significantly higher (P= 0.008) when the patch was used,​ although higher levels of nausea were found in the nicotine group (P= 0.031 at 30 minutes,​ P= 0.008 at 4 hours). The nicotine patch was effective at controlling pain,​ oedema,​ and trismus following third molar surgery. |
| 94 | Cheung,​ Yee Tak Derek,​ Cheung Li,​ William Ho,​ Wang,​ Man Ping,​ Lam,​ Tai Hing. Delivery of a Nicotine Replacement Therapy Sample at Outdoor Smoking Hotspots for Promoting Quit Attempts: A Pilot Randomized Controlled Trial. Nicotine & tobacco research : official journal of the Society for Research on Nicotine and Tobacco. 2020;22:1468.  **Abstract:** INTRODUCTIONOutdoor smoking hotspots are convenient venues for promoting smoking cessation. This randomized controlled trial aimed to obtain proof-of-concept evidence of the feasibility and preliminary effectiveness on quit attempts of delivering a 1-week free nicotine replacement therapy sample (NRTS) to smokers.METHODSThis pilot parallel,​ single-blinded,​ two-group (1:1) randomized controlled trial proactively recruited adult smokers in outdoor smoking hotspots in Hong Kong. Smokers consuming at least 10 cigarettes per day and fit for NRT use were individually randomized to receive either a 1-week NRT gum/patch and brief advice lasting 10 minutes (NRTS,​ n = 50),​ or receive only brief advice (control,​ n = 50). The primary outcomes were any self-reported quit attempts (stop smoking for at least 24 hours) at 1- and 3-month telephone follow-up. Risk ratios from log-binomial regression models were used to assess the associations.RESULTSThe NRTS increased quit attempts at 1-month (14% vs. 10%; adjusted risk ratio = 1.25,​ 95% CI = 0.43 to 3.61) and 3-month follow-up (26% vs. 12%; adjusted risk ratio = 2.17,​ 95% CI = 0.89 to 5.27),​ but the differences were not significant. Trial participation rate was about 81.3%. Around 54% of the intervention group participants used the NRT sample by the first month. The NRT users reported generally positive feedback about the usefulness of NRT sample for smoking cessation. Major factors of not using NRT included bad gum taste and their perception that NRT was not useful.CONCLUSIONSDelivering NRTS to smokers in outdoor smoking hotspots was feasible and efficacious in increasing NRT use. Additional post-recruitment support to sustain the use of NRT and cessation services is needed.IMPLICATIONSOur study supported that smokers at outdoor smoking hotspots can be approached for a brief smoking cessation intervention including an onsite delivery of NRTS. Delivering NRTS and a brief advice on using NRT to these smokers was feasible and efficacious to increase NRT use. A larger trial on the benefits on quit attempts and long-term abstinence is warranted. |
| 96 | Kruse,​ Gina R,​ Park,​ Elyse R,​ Chang,​ Yuchiao,​ Haberer,​ Jessica E,​ Abroms,​ Lorien C,​ Shahid,​ Naysha N,​ Howard,​ Sydney,​ Haas,​ Jennifer S,​ Rigotti,​ Nancy A. Proactively Offered Text Messages and Mailed Nicotine Replacement Therapy for Smokers in Primary Care Practices: A Pilot Randomized Trial. Nicotine & tobacco research : official journal of the Society for Research on Nicotine and Tobacco. 2020;22:1509.  **Abstract:** INTRODUCTIONProactive,​ population health cessation programs can guide efforts to reach smokers outside of the clinic to encourage quit attempts and treatment use.AIMS AND METHODSThis study aimed to measure trial feasibility and preliminary effects of a proactive intervention offering text messages (TM) and/or mailed nicotine replacement therapy (NRT) to smokers in primary care clinics. From 2017 to 2019 we performed a pilot randomized trial comparing brief telephone advice (control: BA),​ TM,​ 2 weeks of mailed NRT,​ or both interventions (TM + NRT). Patients were identified using electronic health records and contacted proactively by telephone to assess interest in the study. We compared quit attempts,​ treatment use,​ and cessation in the intervention arms with BA.RESULTSOf 986 patients contacted,​ 153 (16%) enrolled (mean age 53 years,​ 57% female,​ 76% white,​ 11% black,​ 8% Hispanic,​ 52% insured by Medicaid) and 144 (94%) completed the 12-week assessment. On average,​ patients in the TM arms received 159 messages (99.4% sent,​ 0.6% failed),​ sent 19 messages,​ and stayed in the program for 61 days. In all groups,​ a majority of patients reported quit attempts (BA 67% vs. TM 86% [p = .07],​ NRT 81% [p = .18],​ TM + NRT 79% [p = .21]) and NRT use (BA 51% vs. NRT 83% [p = .007],​ TM 65% [p = .25],​ TM + NRT 76% [p = .03]). Effect estimates for reported 7-day abstinence were BA 10% versus TM 26% (p = .09),​ NRT 28% (p = .06),​ and TM + NRT 23% (p = .14).CONCLUSIONSProactively offering TM or mailed nicotine medications was feasible among primary care smokers and a promising approach to promote quit attempts and short-term abstinence.IMPLICATIONSProactive intervention programs to promote quit attempts outside of office visits among smokers enrolled in primary care practices are needed. TM have potential to engage smokers not planning to quit or to support smokers to make a planned quit attempt. This pilot study demonstrates the feasibility of testing a proactive treatment model including TM and/or mailed NRT to promote quit attempts,​ treatment use,​ and cessation among nontreatment-seeking smokers in primary care.CLINICALTRIALS.GOV IDENTIFIERNCT03174158. |
| 103 | Nollen,​ Nicole L,​ Cox,​ Lisa Sanderson,​ Mayo,​ Matthew S,​ Ellerbeck,​ Edward F,​ Ahluwalia,​ Jasjit S. Counseling alone or in combination with nicotine replacement therapy for treatment of black non-daily smokers: a randomized trial. Addiction (Abingdon,​ England). 2020;115:1547.  **Abstract:** BACKGROUND AND AIMSOne-third of US tobacco users are non-daily smokers (NDS). Black NDS have strikingly high levels of nicotine and carcinogen exposure. No smoking cessation studies have been conducted with this high-risk group. This study compared the effectiveness in black NDS of smoking cessation counseling alone or in combination with the participant's choice of nicotine replacement therapy.DESIGNTwo-arm parallel-group individually randomized clinical trial (allocation ratio of 2 : 1 intervention to control) SETTING: Academic medical and federally qualified health centers in three US cities.PARTICIPANTSNon-Hispanic black adult NDS receiving counseling with nicotine replacement therapy (C + NRT,​ n = 185) or counseling alone (C,​ n = 93).INTERVENTIONSTwelve weeks of in-person and telephone smoking cessation counseling in combination with nicotine replacement therapy (NRT; C + NRT) or counseling alone (C). All participants received five sessions of counseling; those randomized to C + NRT received their choice of nicotine gum,​ patch and/or lozenge after a 9-day product trial period. The target quit day was set at 2 weeks post-baseline for both groups.MEASUREMENTSPrimary outcome was biochemically verified 30-day abstinence at week 12. Secondary outcomes were change in nicotine and carcinogen exposure [4-(methynitrosamino)-1-(3) pyridyle-1-butanol; NNAL] and tobacco consumption patterns.FINDINGSAbstinence was 11.4% in C + NRT and 8.6% in C [odds ratio (OR) = 1.4,​ 95% confidence interval (CI) = 0.6,​ 3.2,​ P = 0.48]. Both groups experienced significant reduction in NNAL (C + NRT: 53% reduction,​ C: 50% reduction,​ within-group P < 0.0001) but non-significant changes in cotinine (P = 0.69). C + NRT reported more days abstinent (P < 0.001) and fewer total cigarettes (P = 0.002) compared with C. There was no evidence of compensation with other tobacco products.CONCLUSIONSAmong black non-daily smokers in the United States,​ there was no difference in abstinence between nicotine replacement therapy (NRT) and counseling alone. NRT led to greater increase in days abstinent and reduction in cigarettes,​ with no evidence of compensation from other sources of nicotine. |
| 108 | Carpenter,​ Matthew J,​ Wahlquist,​ Amy E,​ Dahne,​ Jennifer,​ Gray,​ Kevin M,​ Garrett-Mayer,​ Elizabeth,​ Cummings,​ K Michael,​ Davis,​ Robert,​ Egan,​ Brent M. Nicotine replacement therapy sampling for smoking cessation within primary care: results from a pragmatic cluster randomized clinical trial. Addiction (Abingdon,​ England). 2020;115:1358.  **Abstract:** BACKGROUND AND AIMSWithin the context of busy clinical settings,​ health-care providers need practical,​ evidence-based options to engage smokers in quitting. Sampling of nicotine replacement therapy [i.e. provision of nicotine replacement therapy (NRT starter kits)] is a brief,​ pragmatic strategy to address this need. We aimed to compare the effects of NRT sampling plus standard care (SC),​ relative to SC alone,​ provided by primary care providers during routine clinic visits.DESIGNCluster-randomized clinical trial.SETTINGTwenty-two primary care clinics in South Carolina,​ USA.PARTICIPANTSAdult smokers [n = 1245; 61% female,​ mean age = 50.7,​ standard deviation (SD) = 13.5] both motivated and unmotivated to quit,​ seen during routine clinical visit. Interventions were provider-delivered SC (n = 652,​ 12 clinics) cessation advice or SC + a 2-week supply of both nicotine patch and lozenge,​ with minimal instructions on use (n = 593; 10 clinics).MEASUREMENTSThe primary outcome was 7-day point prevalence smoking abstinence at 6-month follow-up,​ using intent-to-treat. Additional outcomes included NRT use and quit attempts,​ assessed at 1,​ 3 and 6 months following baseline.FINDINGSSeven-day point prevalence abstinence rates were significantly higher in the NRT sampling group throughout follow-up,​ including at 6 months [12 versus 8%,​ odds ratio (OR) = 1.5,​ 95% confidence interval (CI) = 1.0-2.4]. NRT sampling increased prevalence of any use of NRT (65 versus 25%,​ OR = 5.8,​ 95% CI = 4.3-7.7),​ with higher prevalence of use at 6 months (25 versus 14%,​ OR = 2.0,​ 95% CI = 1.5-2.7). NRT sampling increased the rate of quit attempts in the initial month (24 versus 18%,​ OR = 1.5,​ 95% CI = 1.0-2.3) but had no significant effect on overall rate of quit attempts (48 versus 45%,​ OR = 1.2,​ 95% CI = 0.8-1.7).CONCLUSIONProviding smokers with a free 2-week starter kit of nicotine replacement therapy increased quit attempts,​ use of stop smoking medications and smoking abstinence compared with standard care in a primary care setting. |
| 110 | Kaye,​ Jesse T,​ Johnson,​ Adrienne L,​ Baker,​ Timothy B,​ Piper,​ Megan E,​ Cook,​ Jessica W. Searching for Personalized Medicine for Binge Drinking Smokers: Smoking Cessation Using Varenicline,​ Nicotine Patch,​ or Combination Nicotine Replacement Therapy. Journal of studies on alcohol and drugs. 2020;81:426.  **Abstract:** OBJECTIVEHeavy drinking is common among smokers and is associated with especially poor health outcomes. Varenicline may affect mechanisms and clinical outcomes that are relevant for both smoking cessation and alcohol use. The current study examines whether varenicline,​ relative to nicotine replacement therapy,​ yields better smoking cessation outcomes among binge drinking smokers.METHODSecondary data analyses of a comparative effectiveness randomized controlled trial of three smoking cessation pharmacotherapies (12 weeks of varenicline,​ nicotine patch,​ or nicotine patch and lozenge) paired with six counseling sessions were conducted. Adult daily cigarette smokers (N = 1,​078,​ 52% female) reported patterns of alcohol use,​ cigarette craving,​ and alcohol-related cigarette craving at baseline and over 4 weeks after quitting. Smoking cessation outcome was 7-day biochemically confirmed point-prevalence abstinence.RESULTSBinge drinkers had higher relapse rates than moderate drinkers at 4-week post-target quit day but not at the end of treatment or long-term follow up (12 and 26 weeks). Varenicline did not yield superior smoking cessation outcomes among binge drinkers,​ nor did it affect alcohol use early in the quit attempt. Varenicline did produce relatively large reductions in alcohol-related cigarette craving and overall cigarette craving during the first 4 weeks after quitting.CONCLUSIONSVarenicline did not yield higher smoking abstinence rates or reduce alcohol use among binge drinkers. Varenicline did reduce alcohol-related cigarette craving but this did not translate to meaningful differences in smoking abstinence. Varenicline's effects on smoking abstinence do not appear to vary significantly as a function of drinking status. |
| 122 | Wang,​ Kainan S,​ Zegel,​ Maya,​ Molokotos,​ Elena,​ Moran,​ Lauren V,​ Olson,​ David P,​ Pizzagalli,​ Diego A,​ Janes,​ Amy C. The acute effects of nicotine on corticostriatal responses to distinct phases of reward processing. Neuropsychopharmacology : official publication of the American College of Neuropsychopharmacology. 2020;45:1207.  **Abstract:** Nicotine enhances the reinforcement of non-drug rewards by increasing nucleus accumbens (NAcc) reactivity to anticipatory cues. This anticipatory effect is selective as no clear evidence has emerged showing that nicotine acutely changes reward receipt reactivity. However,​ repeated rewarding experiences shift peak brain reactivity from hedonic reward outcome to the motivational anticipatory cue yielding more habitual cue-induced behavior. Given nicotine's influence on NAcc reactivity and connectivity,​ it is plausible that nicotine acutely induces this shift and alters NAcc functional connectivity during reward processing. To evaluate this currently untested hypothesis,​ a randomized crossover design was used in which healthy non-smokers were administered placebo and nicotine (2-mg lozenge). Brain activation to monetary reward anticipation and outcome was evaluated with functional magnetic resonance imaging. Relative to placebo,​ nicotine induced more NAcc reactivity to reward anticipation. Greater NAcc activation during anticipation was significantly associated with lower NAcc activation to outcome. During outcome,​ nicotine reduced NAcc functional connectivity with cortical regions including the anterior cingulate cortex,​ orbitofrontal cortex,​ and insula. These regions showed the same negative relationship between reward anticipation and outcome as noted in the NAcc. The current findings significantly improve our understanding of how nicotine changes corticostriatal circuit function and communication during distinct phases of reward processing and critically show that these alterations happen acutely following a single dose. The implications of this work explain nicotinic modulation of general reward function,​ which offer insights into the initial drive to smoke and the subsequent difficulty in cessation. |
| 123 | Chai,​ Stephanie H,​ Leventhal,​ Adam M,​ Kirkpatrick,​ Matthew G,​ Eisenlohr-Moul,​ Tory A,​ Rapkin,​ Andrea J,​ D'Orazio,​ Lina,​ Pang,​ Raina D. Effectiveness of transdermal nicotine patch in premenopausal female smokers is moderated by within-subject severity of negative affect and physical symptoms. Psychopharmacology. 2020;237:1737.  **Abstract:** RATIONALENicotine patches may be less effective in female compared with male smokers. However,​ it is unknown if negative affect and physical symptoms influence transdermal nicotine patch-related effects on smoking behaviors.METHODSEighty-one acutely tobacco-abstinent premenopausal female smokers attended three counter-balanced experimental sessions across the menstrual cycle (early follicular,​ late follicular,​ and mid-luteal) and were randomized to patch condition (nicotine [21 mg] vs. placebo [0 mg] transdermal patch). Negative affect and physical symptoms were assessed prior to patch administration. The patch was removed 5 h post-administration,​ and participants completed a smoking reinstatement task. Multilevel linear models tested associations of patch condition,​ negative affect and physical symptoms,​ and their interaction on smoking behavior.RESULTSThere was a significant patch condition × Negative Affect and Pain symptoms interaction on the number of cigarettes smoked (p < 0.05). When Negative Affect and Pain were lower-than-usual,​ females administered a nicotine patch smoked significantly fewer cigarettes than females administered a placebo patch (p < .05),​ but there were no significant patch differences when Negative Affect and Pain were higher-than-usual. There was also a significant patch condition × Negative Affect interaction on time delay. The effects of patch condition on time delay to smoking were greater during sessions in which Negative Affect was higher-than-usual.CONCLUSIONSResults suggest that among female smokers transdermal nicotine patch effectiveness may interact with negative affect and pain. Understanding and considering female-specific factors that may impact the efficacy of one of the most commonly used cessation medications is important for improving smoking cessation in female smokers. |
| 129 | Bernstein,​ Steven L.,​ Dziura,​ James,​ Weiss,​ June,​ Harper-Brooks,​ Avis,​ Miller,​ Ted,​ Vickerman,​ Katrina,​ Grau,​ Lauretta E.,​ Pantalon,​ Michael V.,​ Abroms,​ Lorien,​ Collins,​ Linda,​ Toll,​ Benjamin. Successful tobacco dependence treatment in the emergency department: A randomized trial using the multiphase optimization strategy. Academic Emergency Medicine. 2020;27:S52.  **Abstract:** Background and Objectives: Tobacco dependence treatment begun in the hospital emergency department (ED) is effective. Treatment interventions typically involves multiple components,​ making it difficult to identify specific components that are effective or estimate interactions between components. The Multiphase Optimization Strategy (MOST) allows investigators to identify these effects. Methods: We conducted a full-factorial,​ 2x2x2x2 (16-condition) optimization trial in a busy hospital ED of 4 tobacco dependence components: a brief negotiation interview (BNI),​ delivered by a research assistant; 6 weeks of nicotine replacement therapy (NRT),​ in the form of patches and gum,​ with the first dose delivered in the ED; automatic referral to a telephone quitline; and enrollment in SmokefreeTXT,​ a free short-messaging service text program developed by the National Cancer Institute. We modified SmokefreeTXT slightly,​ by eliminating the 2-week module of pre-quit date messages,​ retaining the 6 weeks of post-quit messages and adding several ED-specific messages. Study data were analyzed with a novel mixed methods design to assess clinical efficacy,​ cost effectiveness,​ and qualitative participant feedback. The primary endpoint was tobacco abstinence at 3 months,​ verified by participants' exhaled carbon monoxide. Results: Between February 2017 and May 2019,​ we enrolled 1056 adult smokers visiting the ED. Biochemically confirmed abstinence rates at 3 months for each component vs. control were: BNI,​ 13.5% vs. 8.9% (P=0.02); NRT,​ 14.4% vs. 8.0% P=0.001); quitline,​ 12.4% vs. 10.1% (P=0.24); SmokefreeTXT,​ 11.6% vs. 10.8% (P=0.70). There were no statistically significant interactions among components. Economic and qualitative data,​ presented elsewhere,​ support the cost effectiveness and feasibility of these components. Conclusion: The BNI and NRT were efficacious in this MOST trial,​ which is the first to identify components of ED-initiated tobacco dependence treatment that are individually effective. Future work will focus on enhancing scalability,​ by testing provider-delivered BNIs,​ offering NRT prescriptions rather than distribution of 6 weeks of NRT,​ and disseminating a toolkit for ED treatment. |
| 130 | Cunningham,​ John A,​ Kushnir,​ Vladyslav,​ Selby,​ Peter,​ Zawertailo,​ Laurie,​ Tyndale,​ Rachel F,​ Leatherdale,​ Scott T,​ Schell,​ Christina. Five-Year Follow-up of a Randomized Clinical Trial Testing Mailed Nicotine Patches to Promote Tobacco Cessation. JAMA internal medicine. 2020;180:792.  **Abstract:** |
| 131 | Zakiniaeiz, Yasmin, Liu, Heather, Gao, Hong, Najafzadeh, Soheila, Ropchan, Jim, Nabulsi, Nabeel, Huang, Yiyun, Cosgrove, Kelly, Morris, Evan. Nicotine patch reduces striatal smoking-induced dopamine release compared to placebo patch. Journal of Nuclear Medicine. 2020; 61(S1):1566.  **Abstract:** Introduction: Tobacco smoking and combustible product-related deaths greatly exceed those from alcohol,​ firearms,​ AIDS,​ and all other drugs of abuse including opioids,​ combined. Although all available treatments for smoking cessation have only limited success rates,​ currently,​ the first line pharmacological treatment for tobacco smoking is nicotine replacement therapy (NRT),​ i.e. nicotine patch. NRT acts directly at the nicotinic-acetylcholine receptor on dopamine (DA) terminals to release DA in the striatum-which encodes reward and habit formation. To better understand treatment efficacy,​ we used a naturalistic experimental system combined with a kinetic model designed to characterize smoking-induced DA release,​ in vivo. The goals of this study were (1) to examine the strength of cigarette smoking-induced striatal DA release in tobacco smokers under nicotine patch and placebo patch conditions and,​ (2) to relate the spatial extent of DA release to smoking behavior-nicotine dependence. We hypothesized that (1) nicotine patch would reduce the spatial extent of DA release in the striatum compared to placebo patch,​ and (2) number of smoking pack years would be associated with the spatial extent of DA response in the striatum. Methods: Twenty-eight tobacco smokers (13 female) received a nicotine patch (21mg,​ daily) for 1-week and a placebo patch for 1-week in a randomized,​ counter-balanced order. Following 1-week under each condition and then overnight abstinence,​ smokers participated in two 90-minute [11C]raclopride PET scans and smoked a cigarette while lying in the scanner. We used lp-ntPET,​ a model of tracer uptake containing a timevarying term to identify highly localized DA transients in PET data on a voxel-by-voxel basis. lp-ntPET was fitted to PET TACs at each voxel in the pre-commissural striatum. DA responses were retained only for voxels if the inclusion of the time-varying term improved the fit. Each frame was smoothed with a 3D Gaussian filter before fitting. “Probability of activation” maps were generated,​ summed by condition and divided by the number of group members. Smoking pack years were calculated by multiplying the number of cigarette packs smoked per day by the number of years smoked. Participants were divided into low and high number of pack years groups using a median split analysis. Probability of activation maps for low pack years and high pack years were made for exploratory analysis. Results: Nicotine patch reduced the spatial extent of DA release and the probability of DA activation following cigarette smoking compared to smoking following placebo patch (Figure 1A). The high number of pack years group had higher spatial extent and probability of DA activation in the striatum during the placebo patch condition compared to the low number of pack years group (Figure 1B). Conclusions: Consistent with our hypotheses,​ the nicotine patch reduced the strength of cigarette-induced striatal DA response compared to placebo patch,​ suggesting a potential mechanism for the effect of nicotine patch treatment on the rewarding response of cigarette smoking. Number of pack years was associated with the strength of the striatal DA response in both reward-related and habit-formation hubs of the striatum. These preliminary findings suggest that pack years of tobacco smoking contribute to DA response and that our method might be able to parse out group differences in smoking behavior characteristics. |
| 132 | Reid,​ Carol,​ Fenech,​ Mary,​ Jones,​ Lee,​ Salehi,​ Nasim. Nurse practitioner interventions for smokers with chronic hepatitis C. Journal of the American Association of Nurse Practitioners. 2020;32:380.  **Abstract:** BACKGROUNDSmoking is a grossly overlooked risk factor for people with chronic hepatitis C with regard to disease progression. It is unclear whether current smoking cessation interventions are effective for this population.PURPOSEThe purpose was to evaluate the effectiveness of a telephone counseling and nicotine replacement therapy (NRT) intervention for smokers with chronic hepatitis C to quit or reduce rates of smoking.METHODSA randomized controlled trial was conducted with participants randomized and stratified according to heaviness of smoking. Ninety-two eligible adults who smoked cigarettes and attended hepatology outpatient clinics were recruited. The intervention included NRT and telephone counseling compared with telephone counseling alone. Data collection occurred from December 2010 to November 2011. Data were collected at baseline,​ 6,​ and 12 weeks to assess smoking cessation. Change scores were analyzed using analysis of variance to examine the differences between smoking interventions.RESULTSAt 6 weeks,​ both control and intervention groups had quit or reduced the number of cigarettes smoked daily. However,​ over 12 weeks,​ the intervention group showed sustained quitting or reduced smoking,​ with 5.8 (confidence interval [CI]: 2.4,​ 9.3) fewer cigarettes smoked per day from baseline. The control group maintained an average reduction of 1.6 (CI: -1.9,​ 5.2) fewer cigarettes per day.IMPLICATIONS FOR PRACTICENicotine replacement therapy and individualized telephone counseling interventions increase the prospects of smoking cessation. Interventions such as these,​ introduced at routine clinic appointments in the outpatients' setting,​ by a nurse practitioner (hepatology) showed clinically important results for smoking cessation in this population. |
| 133 | Gilbert,​ David G,​ Rabinovich,​ Norka E,​ McDaniel,​ Justin T. Nicotine patch for cannabis withdrawal symptom relief: a randomized controlled trial. Psychopharmacology. 2020;237:1507.  **Abstract:** RATIONALEGiven that tetrahydrocannabinol (THC) and nicotine have similar effects on negative affect (NA),​ we hypothesized that a 7-mg nicotine patch (NP) would reduce NA-related cannabis (CAN) withdrawal symptoms in cannabis-dependent (CD) individuals who were not nicotine dependent.OBJECTIVEWe sought to determine whether NP reduces NA across 15 days of CAN abstinence in two groups: non-tobacco smokers (NTS) and light tobacco smokers (LTS).METHODSCD participants (N = 127; aged 18-35) who used CAN at least 5 times/week for the past 12 + months were randomized to (1) NP or (2) a placebo patch (PP) and received 0 for sustained biochemically verified CAN abstinence. Of those randomly assigned,​ 52 of 63 NP,​ and 56 of 64 PP maintained biochemically verified CAN abstinence and 51 NP and 50 PP participants complied with all aspects of the study. Affect and other withdrawal symptoms were measured every 48 h across 15 days of CAN abstinence.RESULTSAfter controlling for age,​ tobacco use,​ baseline THC concentration,​ and baseline measurements of the dependent variable,​ NP reduced NA symptoms across the 15-day treatment relative to PP. Differences in NA and CAN withdrawal symptoms were not moderated by tobacco user status.CONCLUSIONSThe findings provide the first evidence that NP may be able to attenuate NA-related withdrawal symptoms in individuals with cannabis use disorder who are not heavy users of tobacco or nicotine.CLINICAL TRIALS REGISTRYNCT01400243 http://www.clinicaltrials.gov. |
| 143 | Nides,​ Mitchell,​ Danielsson,​ Tobias,​ Saunders,​ Frederick,​ Perfekt,​ Roland,​ Kapikian,​ Roxanne,​ Solla,​ Janice,​ Leischow,​ Scott J,​ Myers,​ Andrew. Efficacy and Safety of a Nicotine Mouth Spray for Smoking Cessation: A Randomized,​ Multicenter,​ Controlled Study in a Naturalistic Setting. Nicotine & tobacco research : official journal of the Society for Research on Nicotine and Tobacco. 2020;22:339.  **Abstract:** BACKGROUNDNicotine replacement therapy (NRT) has been demonstrated to be an effective pharmacological treatment for smoking cessation,​ and most types of NRT have been approved as over-the-counter (OTC) medications. In an effort to create a fast-acting,​ flexible,​ and discreet NRT,​ a nicotine mouth spray (NMS) has been developed. This study was designed to assess the efficacy and safety of NMS in a naturalistic setting in the United States.METHODSThis was a multicenter,​ randomized,​ double-blind,​ placebo-controlled,​ parallel-group,​ 26-week study in 1198 smokers motivated to quit. The study was designed to resemble an OTC environment,​ and thus included limited intervention,​ limited motivational screening,​ and no behavioral support. The primary efficacy endpoint was carbon monoxide-verified,​ self-reported continuous abstinence from smoking from week 2 until week 6. The safety of NMS was assessed by measuring vital signs,​ visual mouth inspection,​ and collection of subject-reported adverse events (AEs).RESULTSThe percentage of subjects with carbon monoxide-verified continuous abstinence from week 2 to week 6 was statistically significantly greater in the NMS group compared with the placebo group (5.0% vs. 2.5%,​ p = .021). Statistically significant treatment effects for the NMS were maintained throughout the 26-week period. The study medications were generally well tolerated. The severity of AEs was similar for both treatment groups,​ and most AEs were of mild or moderate severity.CONCLUSIONSThese study results demonstrate that the NMS is an effective and safe smoking cessation option for smokers motivated to quit,​ even in a naturalistic setting and without behavioral support.IMPLICATIONSThis study demonstrated the safety,​ efficacy,​ and acceptability of an NMS in an OTC environment with no behavioral counseling or support. It provides an additional option for smokers motivated to quit.TRIAL REGISTRATIONClinicalTrials.gov (number NCT02355665). |
| 146 | Shiffman,​ Saul,​ Scholl,​ Sarah M,​ Mao,​ Jason,​ Ferguson,​ Stuart G,​ Hedeker,​ Donald,​ Primack,​ Brian,​ Tindle,​ Hilary A. Using Nicotine Gum to Assist Nondaily Smokers in Quitting: A Randomized Clinical Trial. Nicotine & tobacco research : official journal of the Society for Research on Nicotine and Tobacco. 2020;22:390.  **Abstract:** INTRODUCTIONNon-daily intermittent smokers (ITS) comprise 30% of US adult smokers. ITS smoke for nicotine and have trouble quitting,​ but tend to smoke in particular situations. This study tested the effect of nicotine gum,​ used to prevent or react to situational temptations,​ for helping ITS quit.METHODSITS (smoking 4-27 days/month) seeking help quitting were randomized to 2 mg nicotine gum (n = 181) or placebo (n = 188),​ to be used to anticipate or react to temptations to smoke,​ for 8 weeks. Participants received up to six sessions of behavioral counseling. The primary outcome was 6-month biochemically verified continuous abstinence; analyses also examined 14-day point-prevalence abstinence at multiple time points,​ and used event-history analyses to assess progression to abstinence,​ lapsing,​ and relapsing. Analyses adjusted for group differences in age and baseline smoking,​ and considered several potential moderators of treatment effects.RESULTSNicotine gum did not significantly improve outcomes on any measure. Biochemically verified 6-month continuous abstinence rates were 7.2% for active gum and 5.3% for placebo (AOR = 1.39,​ 0.58-3.29,​ p > .25). ITS with any degree of dependence (Fagerstrom Test of Nicotine Dependence scores >0) showed poorer outcomes on multiple endpoints,​ and did more poorly on active gum on some outcomes. Gum use was low,​ starting at 1 gum per day on average and declining over time.CONCLUSIONSNicotine gum (2 mg),​ used intermittently,​ did not improve cessation rates among ITS,​ including those demonstrating some degree of dependence.IMPLICATIONSNicotine replacement has been extensively tested with daily smokers,​ especially those who smoke relatively heavily. Nondaily smoking is now common,​ creating a need for treatment for ITS. Despite evidence that ITS' smoking is motivated by nicotine-seeking,​ a theoretically and empirically derived situational approach to using acute nicotine replacement was not successful at helping ITS quit. Gum use was low; whether higher or more frequent dosing is needed,​ or whether an entirely different approach is needed,​ is not clear. Effective treatment options are needed for ITS,​ especially those with some degree of dependence. |
| 152 | Ayers,​ Catherine R,​ Heffner,​ Jaimee L,​ Russ,​ Cristina,​ Lawrence,​ David,​ McRae,​ Thomas,​ Evins,​ A Eden,​ Anthenelli,​ Robert M. Efficacy and safety of pharmacotherapies for smoking cessation in anxiety disorders: Subgroup analysis of the randomized,​ active- and placebo-controlled EAGLES trial. Depression and anxiety. 2020;37:247.  **Abstract:** BACKGROUNDSmoking rates are high in adults with anxiety disorders (ADs),​ yet little is known about the safety and efficacy of smoking-cessation pharmacotherapies in this group.METHODSPost hoc analyses in 712 smokers with AD (posttraumatic stress disorder [PTSD],​ n = 192; generalized anxiety disorder [GAD],​ n = 243; panic disorder [PD],​ n = 277) and in a nonpsychiatric cohort (NPC; n = 4,​028). Participants were randomly assigned to varenicline,​ bupropion,​ nicotine-replacement therapy (NRT),​ or placebo plus weekly smoking-cessation counseling for 12 weeks,​ with 12 weeks follow-up. General linear models were used to test the effects of treatment group,​ cohort,​ and their interaction on neuropsychiatric adverse events (NPSAEs),​ and continuous abstinence weeks 9-12 (treatment) and 9-24 (follow-up).RESULTSNPSAE incidence for PTSD (6.9%),​ GAD (5.4%),​ and PD (6.2%) was higher versus NPC (2.1%),​ regardless of treatment. Across all treatments,​ smokers with PTSD (odds ratio [OR] = 0.58),​ GAD (OR = 0.72),​ and PD (OR = 0.53) had lower continuous abstinence rates weeks 9-12 (CAR9-12) versus NPC. Varenicline demonstrated superior efficacy to placebo in smokers with GAD and PD,​ respectively (OR = 4.53; 95% confidence interval [CI] = 1.20-17.10; and OR = 8.49; 95% CI = 1.57-45.78); NRT was superior to placebo in smokers with PD (OR = 7.42; 95% CI = 1.37-40.35). While there was no statistically significant effect of any treatment on CAR9-12 for smokers with PTSD,​ varenicline improved 7-day point prevalence abstinence at end of treatment in this subcohort.CONCLUSIONIndividuals with ADs were more likely than those without psychiatric illness to experience moderate to severe NPSAEs during smoking-cessation attempts,​ regardless of treatment. While the study was not powered to evaluate abstinence outcomes with these subgroups of smokers with ADs,​ varenicline provided significant benefit for cessation in those with GAD and PD,​ while NRT provided significant benefit for those with PD. |
| 156 | Pham,​ Carol Q,​ Kapolowicz,​ Michelle R,​ Metherate,​ Raju,​ Zeng,​ Fan-Gang. Nicotine enhances auditory processing in healthy and normal-hearing young adult nonsmokers. Psychopharmacology. 2020;237:833.  **Abstract:** RATIONALEElectrophysiological studies show that systemic nicotine narrows frequency receptive fields and increases gain in neural responses to characteristic frequency stimuli. We postulated that nicotine enhances related auditory processing in humans.OBJECTIVESThe main hypothesis was that nicotine improves auditory performance. A secondary hypothesis was that the degree of nicotine-induced improvement depends on the individual's baseline performance.METHODSYoung (18-27 years old),​ normal-hearing nonsmokers received nicotine (Nicorette gum,​ 6mg) or placebo gum in a single-blind,​ randomized,​ crossover design. Subjects performed four experiments involving tone-in-noise detection,​ temporal gap detection,​ spectral ripple discrimination,​ and selective auditory attention before and after treatment. The perceptual differences between posttreatment nicotine and placebo conditions were measured and analyzed as a function of the pre-treatment baseline performance.RESULTSNicotine significantly improved performance in the more difficult tasks of tone-in-noise detection and selective attention (effect size = - 0.3) but had no effect on relatively easier tasks of temporal gap detection and spectral ripple discrimination. The two tasks showing significant nicotine effects further showed no baseline-dependent improvement.CONCLUSIONSNicotine improves auditory performance in difficult listening situations. The present results support future investigation of nicotine effects in clinical populations with auditory processing deficits or reduced cholinergic activation. |
| 158 | Webb,​ A R,​ Coward,​ L,​ Soh,​ L,​ Waugh,​ L,​ Parsons,​ L,​ Lynch,​ M,​ Stokan,​ L-A,​ Borland,​ R. Smoking cessation in elective surgical patients offered free nicotine patches at listing: a pilot study. Anaesthesia. 2020;75:171.  **Abstract:** Free nicotine patches may promote pre-operative smoking cessation. Smokers (≥ 10 cigarettes.day-1 ) awaiting non-urgent surgery were randomly assigned (3:1) to an offer of free nicotine patches or a control group who were not offered free nicotine patches. The suggested regimen lasted 5 weeks,​ with patch strength decreasing incrementally after 3 and 4 weeks. The primary outcome was smoking abstinence for ≥ 4 weeks,​ as self-reported by participants on the day of surgery,​ including,​ where possible,​ corroboration using exhaled carbon monoxide testing. Out of 600 included smokers,​ 447 (74.5%) were randomly assigned to an offer of pre-operative nicotine patches,​ with 175 (39.1%) of these accepting the offer and 56 (12.5%) using patches for ≥ 3 weeks. Out of 396 participants offered nicotine patches who were included for analysis,​ 36 (9.1%) quit smoking for ≥ 4 weeks before surgery as compared with 8 (5.9%) controls,​ OR 1.5 [95%CI 0.7-3.2],​ p = 0.300. Sixty-three (15.9%) quit smoking for 24 h before surgery as compared with 15 (11.1%) controls,​ OR 1.4 [95%CI 0.8-2.4],​ p = 0.200. Participants offered nicotine patches were more likely to engage in a cessation attempt lasting more than 24 h,​ 46 (11.6%) vs. 5 (3.7%),​ OR 3.4 [95%CI 1.8-8.8],​ p = 0.010. Out of 78 participants who quit smoking by the day of surgery and were followed up at 6 months,​ 46 (59%) had relapsed. Offering free nicotine patches stimulated interest in quitting compared with controls,​ but our protocol had limited effectiveness. |
| 161 | Schlam,​ Tanya R,​ Baker,​ Timothy B,​ Smith,​ Stevens S,​ Cook,​ Jessica W,​ Piper,​ Megan E. Anxiety Sensitivity and Distress Tolerance in Smokers: Relations With Tobacco Dependence,​ Withdrawal,​ and Quitting Success†. Nicotine & tobacco research : official journal of the Society for Research on Nicotine and Tobacco. 2020;22:58.  **Abstract:** INTRODUCTIONThis study examined relations of two affective vulnerabilities,​ high anxiety sensitivity (AS) and low distress tolerance (DT),​ with tobacco dependence,​ withdrawal,​ smoking cessation,​ and pharmacotherapy response.METHODSSmokers interested in quitting (N = 1067; 52.2% female,​ 28.1% African American) were randomized to 12 weeks of nicotine patch,​ nicotine patch plus nicotine lozenge,​ or varenicline. Baseline questionnaires assessed AS,​ DT,​ negative affect,​ anxiety,​ and dependence. Withdrawal was assessed the first-week post-quit via ecological momentary assessment.RESULTSDT,​ but not AS,​ predicted biochemically confirmed point-prevalence abstinence at multiple endpoints: weeks 4,​ 12,​ 26,​ and 52 post-quit (ps < .05); relations remained after controlling for pharmacotherapy treatment,​ AS,​ baseline negative affect,​ anxiety,​ and anxiety disorder history (ps < .05). Additional exploratory analyses examining week 4 abstinence showed DT predicted abstinence (p = .004) even after controlling for baseline dependence,​ post-quit withdrawal (craving and negative affect),​ and treatment. DT moderated treatment effects on abstinence in exploratory analyses (interaction p = .025); those with high DT were especially likely to be abstinent at week 4 with patch plus lozenge versus patch alone.CONCLUSIONSDT,​ but not AS,​ predicted abstinence over 1 year post-quit (higher DT was associated with higher quit rates),​ with little overlap with other affective measures. DT also predicted early abstinence independent of dependence and withdrawal symptoms. Results suggest low DT may play a meaningful role in motivation to use tobacco and constitute an additional affective risk factor for tobacco cessation failure beyond negative affect or clinical affective disorders.IMPLICATIONSPeople in a stop-smoking study who reported a greater ability to tolerate distress were more likely to quit smoking and remain smoke-free 1 year later. Smokers with high DT were more likely to be smoke-free 4 weeks after their target quit day if they received nicotine patch plus nicotine lozenge rather than nicotine patch alone.TRIAL REGISTRATIONNCT01553084. |
| 163 | Riley,​ Shannon,​ Anand,​ Anju,​ Stanbrook,​ Matthew. Twitter discussions from a respirology journal club: A randomized trial of e-cigarettes versus nicotine-replacement therapy. Canadian Journal of Respiratory,​ Critical Care,​ and Sleep Medicine. 2020;4:64.  **Abstract:** |
| 166 | Nollen, Nicole L., Ahluwalia, Jasjit S., Sanderson Cox, Lisa, Okuyemi, Kolawole, Lawrence, David, Samuels, Larry, Benowitz, Neal L.. Assessment of Racial Differences in Pharmacotherapy Efficacy for Smoking Cessation: Secondary Analysis of the EAGLES Randomized Clinical Trial. JAMA Network Open. 2020; 4(1): e2032053.  **Abstract:** Importance: Understanding Black vs White differences in pharmacotherapy efficacy and the underlying reasons is critically important to reducing tobacco-related health disparities. Objective: To compare pharmacotherapy efficacy and examine variables to explain Black vs White differences in smoking abstinence. Design,​ Setting,​ and Participants: This study is a secondary analysis of the Evaluating Adverse Events in a Global Smoking Cessation Study (EAGLES) double-blind,​ placebo-controlled,​ randomized clinical trial,​ which took place at clinical trial centers,​ academic centers,​ and outpatient clinics in 29 states in the US. US Black and White smokers who smoked 10 or more cigarettes per day with and without psychiatric comorbidity were enrolled between November 2011 and January 2015. Data analysis was performed from July 2019 to January 2020. Interventions: Participants were randomized (1:1:1:1) in a double-blind,​ triple-dummy,​ placebo- and active-controlled (nicotine patch) trial of varenicline and bupropion for 12 weeks with follow-up through week 24. Main Outcomes and Measures: Biochemically verified continuous cigarette abstinence rate (CAR) from weeks 9 to 24. Baseline,​ postbaseline treatment,​ and safety characteristics were examined as variables to explain race differences in abstinence. Results: Of the 1065 Black smokers enrolled,​ 255 were randomized to receive varenicline,​ 259 received bupropion,​ 286 received nicotine replacement therapy (NRT [ie,​ nicotine patch]),​ and 265 received placebo. Among the 3044 White smokers enrolled,​ 778 were randomized to receive varenicline,​ 769 received bupropion,​ 738 received NRT,​ and 759 received placebo. Participants were predominantly female (614 Black [57.7%] and 1786 White [58.7%] women) and heavy smokers (mean [SD] cigarettes per day,​ 18.2 [7.9] for Black and 20.0 [7.5] for White smokers),​ with a mean (SD) age of 47.2 (11.2) years for Black and 46.5 (12.7) years for White participants. Treatment and race were associated with CAR for weeks 9 to 24. The CAR was 4.9% lower for Black vs White participants (odds ratio [OR],​ 0.53; 95% CI,​ 0.41-0.69; P <.001); differences were found across all treatments. Pooling psychiatric and nonpsychiatric cohorts,​ varenicline (OR,​ 2.63; 95% CI,​ 1.90-3.63; P <.001),​ bupropion (OR,​ 1.75; 95% CI,​ 1.25-2.46; P =.001),​ and NRT (OR,​ 1.52; 95% CI,​ 1.07-2.16; P =.02) had greater efficacy than placebo for White participants. Only varenicline (OR,​ 2.63; 95% CI,​ 1.26-5.48; P =.01) had greater efficacy than placebo for Black participants. Baseline,​ postbaseline,​ and safety characteristics differed by race,​ but these variables did not eliminate the association of race with CAR. Black participants had 49% reduced odds of CAR for weeks 9 to 24 compared with White participants in the adjusted model (OR,​ 0.51; 95% CI,​ 0.39-0.66; P <.001). Conclusions and Relevance: Black and White smokers achieved the highest rate of abstinence while taking varenicline,​ suggesting that it is the best first-line therapy for these groups. However,​ Black smokers were less responsive to all therapies,​ including placebo. Understanding variables (eg,​ socioeconomic or biological) beyond those may lead to improved treatment outcomes for Black smokers. Trial Registration: ClinicalTrials.gov Identifier: NCT01456936. |
| 167 | Xiao,​ Dan,​ Kotler,​ Mitchell,​ Kang,​ Jian,​ Wang,​ Chen. A Multicenter,​ Randomized,​ Double-blind,​ Parallel,​ Placebo-controlled Clinical Study to Evaluate the Efficacy and Safety of a Nicotine Mint Lozenge (2 and 4 mg) in Smoking Cessation. Journal of addiction medicine. 2020;14:69.  **Abstract:** OBJECTIVETo evaluate the efficacy in smoking cessation and safety of 2 and 4 mg nicotine mint lozenges in Chinese adult smokers.METHODSThis was a multicenter,​ randomized,​ stratified,​ double-blind,​ placebo-controlled,​ parallel-group study. The low-dependence stratum included 483 smokers (241 randomized to active 2 mg nicotine lozenge and 242 to placebo lozenge). The high-dependence stratum included 240 smokers (120 randomized to active 4 mg nicotine lozenge and 120 to placebo lozenge). The primary endpoint was successful smoking cessation at 6 weeks postquit,​ defined as continuous abstinence from smoking for the 28-day period up to and including the 6-week visit (verified by CO measurement). Cochran-Mantel-Haenszel tests were performed to compare quit rates between active nicotine and placebo separately for the high-dependence and low-dependence strata.RESULTSThe primary analysis showed that in the low-dependence (2 mg) stratum,​ 59 subjects (24.5%) of 241 in the active nicotine group and 52 subjects (21.5%) of 242 in the placebo group were successful quitters (P = .3851). In the high-dependence (4 mg) stratum,​ 37 subjects (30.8%) of 120 in the active nicotine group and 24 subjects (20.2%) of 119 in the placebo group were successful quitters (P = .0565).CONCLUSIONSThe 4 mg nicotine lozenge provided a directionally significant improvement in smoking cessation rates compared with placebo in Chinese adult smokers with high nicotine dependence for the primary endpoint. The 2 mg nicotine lozenge provided higher,​ but nonsignificant,​ smoking cessation rates than placebo. Both nicotine lozenges were generally well tolerated in Chinese adult smokers. |
| 170 | Palmisano,​ Alexandra N,​ Astur,​ Robert S. Nicotine Facilitation of Conditioned Place Preference to Food Reward in Humans. Substance use & misuse. 2020;55:2156.  **Abstract:** BACKGROUNDNicotine has recently been shown to enhance the motivational value of non-nicotine stimuli in nonhumans. To investigate whether nicotine also enhances reward in humans,​ we used a virtual translation of the conditioned place preference (CPP) paradigm to examine nicotine's reward-enhancing effects using a low-dose 2 mg nicotine lozenge targeted to a mild use population. Methods: Sixty-eight nicotine-using undergraduates were randomly assigned to receive either a 2 mg nicotine or placebo lozenge prior to conditioning. During each of six,​ three-minute conditioning sessions,​ participants were confined to one of two VR rooms. In one room,​ they received real chocolate M&Ms,​ whereas no M&Ms were administered in the other room. Following conditioning,​ a three-minute free-access test session occurred during which participants had unrestricted access to both rooms without reward. Results: Individuals who received nicotine demonstrated a CPP by spending significantly more time in the room previously paired with M&Ms compared to the unrewarded room (p = 0.04). Those who received placebo did not demonstrate a CPP (p > 0.05). Moreover,​ we observed no significant differences between treatment groups in terms of the amount of time spent in each virtual room. Conclusion: While nicotine seems to facilitate CPP expression for a virtual environment previously paired with chocolate food rewards,​ further characterization of the mechanism by which this occurs is needed. |
| 174 | Zarghami,​ Mehran,​ Taghizadeh,​ Fatemeh,​ Sharifpour,​ Ali,​ Alipour,​ Abbas. Efficacy of guided self-change for smoking cessation in chronic obstructive pulmonary disease patients: A randomized controlled clinical trial. Tobacco induced diseases. 2019;17:90.  **Abstract:** INTRODUCTIONThe aim of this study was to examine the efficacy of guided self-change (GSC),​ nicotine replacement therapy (NRT),​ and their combination,​ on smoking cessation among patients with COPD.METHODSA total of 60 participants were randomly assigned to three groups for GSC (n=20),​ nicotine replacement therapy (NRT) (n=20) or their combination (n=20),​ from December 2016 to November 2017. The quality of life (QoL) questionnaire,​ clinical assessment test (CAT) and exhaled carbon monoxide (CO),​ were measured at baseline and post-treatment.RESULTSAt 6,​ 12,​ and 29 weeks,​ the abstinence rate in the NRT group was 5.3%,​ 15.8% and 21.1%,​ in the GSC group 21.1%,​ 31.6% and 47.4%,​ and in the combined group 36.8%,​ 36.8% and 47.4%,​ respectively. The exhaled CO in the NRT group was greater than the GSC group,​ however this difference was not statistically significant (3.4; 95% CI: -0.24-7.0; p=0.067),​ CO levels in the combined group were less than the GSC group,​ while this difference was also not significant (-0.75; 95% CI : -4.2-2.7; p=0.68). CAT and QoL recovery in the GSC and combined groups were higher than in the NRT group (9.2; 95% CI: 5.0-13.4; p=0.001) and (-4.5; 95% C: -8.1- -0.6; p=0.02),​ respectively. However,​ differences between combined and GSC groups were not significant (p=0.24 and p=0.41,​ respectively). There was a statistically significant difference between the abstinence rate in the GSC or combined group and the NRT group (p=0.001). The GEE model showed that GSC reduced the odds of smoking compared with the NRT group (interaction group effect) (OR=0.31,​ 95% CI: 0.022-0.545; p=0.001).CONCLUSIONSIn our context among COPD patients,​ GSC was more effective in decreasing smoking than NRT alone. Moreover,​ the recovery of exhaled carbon monoxide,​ CAT and QoL in GSC was more than in the NRT group. Moreover,​ since GSC was as effective as GSC plus NRT,​ the effectiveness of the combination method for smoking cessation in COPD patients may be attributed to GSC.Clinical trial registration details: IRCT201609271457N11; www.irct.ir. |
| 175 | Thao,​ Viengneesee,​ Nyman,​ John A,​ Nelson,​ David B,​ Joseph,​ Anne M,​ Clothier,​ Barbara,​ Hammett,​ Patrick J,​ Fu,​ Steven S. Cost-effectiveness of population-level proactive tobacco cessation outreach among socio-economically disadvantaged smokers: evaluation of a randomized control trial. Addiction (Abingdon,​ England). 2019;114:2206.  **Abstract:** AIMSTo estimate the cost-effectiveness at population-level of the OPT-IN proactive tobacco cessation outreach program for adult smokers enrolled in publicly funded health insurance plans for low-income persons (e.g. Medicaid).DESIGNCost-effectiveness analysis using a state transition model based on data from the Offering Proactive Treatment Intervention (OPT-IN) randomized control trial.SETTINGThe trial was conducted in Minnesota,​ USA,​ and the economic analysis was conducted from the Medicaid program perspective.PARTICIPANTSData were used from 2406 smokers who were randomized into the intervention or comparator groups.INTERVENTION AND COMPARATORThe intervention was comprised of proactive outreach (mailed invitation and telephone calls) and free cessation treatment (nicotine replacement therapy and intensive telephone counseling). The comparator was usual care,​ which comprised access to a primary care physician,​ insurance coverage of Food and Drug Administration (FDA)-approved smoking cessation medications and the state's telephone quitline.MEASUREMENTSSmoking status,​ quality of life and health-care use at varying times,​ including at baseline and 1 year.FINDINGSThe OPT-IN program cost an average of per participant greater than the comparator. One year after randomization,​ the population-level,​ 6-month prolonged smoking abstinence rate was 16.5% in the proactive outreach intervention group and 12.1% in the usual care group (P < 0.05). The model projected that the proactive outreach intervention added in life-time cost and generated 0.005 additional quality-adjusted life-years (QALYs),​ with an expected incremental cost-effectiveness ratio of 31 per QALY. Probabilistic sensitivity analysis found that the proactive outreach intervention would be cost-effective against a willingness-to-pay threshold of  000/QALY approximately 68% of the time.CONCLUSIONSPopulation-level proactive tobacco treatment with personal telephone outreach was effective in achieving higher population-level quit rates and was cost-effective at various willingness-to-pay thresholds,​ compared with usual care (i.e. reactive treatment). Taken together with prior research,​ population-level proactive tobacco cessation outreach programs are judged to be highly cost-effective over the long term. |
| 177 | Gilbert,​ David G,​ Rabinovich,​ Norka E,​ Gilbert-Matuskowitz,​ Elizabeth A,​ Klein,​ Keith P,​ Pergadia,​ Michele L. Smoking abstinence symptoms across 67 days compared with randomized controls-Moderation by nicotine replacement therapy,​ bupropion,​ and negative-affect traits. Experimental and clinical psychopharmacology. 2019;27:536.  **Abstract:** Accurate knowledge of negative affect (NA)-related smoking abstinence symptoms (SAS) severity and duration and their moderation by pharmacotherapy and NA-related personality traits is critical for efficacious treatments given that elevated state and trait NA are predictors of relapse. However,​ SAS severity,​ duration,​ and moderation are not well characterized. To date,​ the longest randomized controlled trial (RCT) of NA-related SAS using randomized delayed-quit smoking controls only examined symptoms across 45 days,​ despite clinical evidence that SAS may last longer. The present RCT assessed SAS across 67 days in dependent smokers (N = 95) who were randomized either to quit or to delay quitting for the course of the trial. The quit group was further randomized to receive either nicotine replacement therapy (NRT),​ bupropion (BUP),​ or placebo. Abstinence-related increases in anger-irritability,​ depressive,​ anxiety,​ and general NA symptoms did not resolve relative to the delayed quit group (DQG) levels across the 67 days in any of the 3 quit groups,​ though craving fell to below DQG and prequit levels. While NRT attenuated Day 3 SAS relative to BUP and placebo,​ BUP and NRT generally did not reduce SAS. High scores on trait measures of NA/neuroticism predicted greater increases in and duration of NA-related SAS,​ potentially indicating that smoking abstinence unmasks affective symptoms. Positive affect was not impacted by abstinence or treatment. The results support the views that (a) prequit baseline values are not a valid index of NA SAS recovery,​ and (b) on average,​ NA-related SAS take longer than 67 days to resolve. (PsycINFO Database Record (c) 2019 APA,​ all rights reserved). |
| 178 | Perry,​ Robin N,​ Schlagintweit,​ Hera E,​ Darredeau,​ Christine,​ Helmick,​ Carl,​ Newman,​ Aaron J,​ Good,​ Kimberley P,​ Barrett,​ Sean P. The impacts of actual and perceived nicotine administration on insula functional connectivity with the anterior cingulate cortex and nucleus accumbens. Journal of psychopharmacology (Oxford,​ England). 2019;33:1600.  **Abstract:** BACKGROUNDChanges in resting state functional connectivity between the insula and dorsal anterior cingulate cortex as well as between the insula and nucleus accumbens have been linked to nicotine withdrawal and/or administration. However,​ because many of nicotine's effects in humans appear to depend,​ at least in part,​ on the belief that nicotine has been administered,​ the relative contribution of nicotine's pharmacological actions to such effects requires clarification.AIMSThe purpose of this study was to examine the impacts of perceived and actual nicotine administration on neural responses.METHODSTwenty-six smokers were randomly assigned to receive either a nicotine inhaler (4 mg deliverable) or a nicotine-free inhaler across two sessions. Inhaler content instructions (told nicotine vs told nicotine-free) differed across sessions. Resting state functional connectivity between sub-regions of the insula and the dorsal anterior cingulate cortex and nucleus accumbens was measured using magnetic resonance imaging before and after inhaler administration.RESULTSBoth actual and perceived nicotine administration independently altered resting state functional connectivity between the anterior insula and the dorsal anterior cingulate cortex,​ with actual administration being associated with decreased resting state functional connectivity,​ and perceived administration with increased resting state functional connectivity. Actual nicotine administration also contralaterally reduced resting state functional connectivity between the anterior insula and nucleus accumbens,​ while reductions in resting state functional connectivity between the mid-insula and right nucleus accumbens were observed when nicotine was administered unexpectedly. Changes in resting state functional connectivity associated with actual or perceived nicotine administration were unrelated to changes in subjective withdrawal and craving. Changes in withdrawal and craving were however independently associated with resting state functional connectivity between the nucleus accumbens and insula.CONCLUSIONSOur findings highlight the importance of considering non-pharmacological factors when examining drug mechanisms of action. |
| 180 | Fischell,​ Sarah Aronson,​ Ross,​ Thomas,​ Salmeron,​ Betty Jo,​ Stein,​ Elliot. Transcranial direct current stimulation applied to the left dorsolateral prefrontal cortex in smokers modifies cognitive circuits implicated in the nicotine withdrawal syndrome. Neuropsychopharmacology. 2019;44:523.  **Abstract:** Background: Symptoms of nicotine withdrawal remain a major impediment for smokers trying to quit; most quit attempts fail within the first week of abstinence. The Nicotine Withdrawal Syndrome (NWS) is characterized by both cognitive (reduced attention,​ working memory,​ "WM") and affective (irritability,​ anxiety) disturbances. Studies employing functional magnetic resonance imaging (fMRI) have identified reductions in strength of the Executive Control Network (ECN) and related nodes-and increases in strength of the Default Mode Network (DMN) and related nodes-as associated with NWS cognitive deficits,​ while hyperactivity of the amygdala and related circuits have been associated with NWS affective dysfunction. Transcranial Direct Current Stimulation (tDCS) has the potential to modify these neuronal circuits by producing a subthreshold conductive current through the scalp and into the brain. Two potential targets for tDCS as a smoking cessation aid are the dorsolateral prefrontal cortex (dlPFC),​ a node of the ECN,​ and the ventromedial prefrontal cortex (vmPFC),​ a node of the DMN. We hypothesized that functional activity in cognitive control networks,​ and downstream amygdala circuits,​ would be modified by acute application of tDCS to the left (L) dlPFC and right (R) vmPFC. Methods: 15 smokers (in 12-hours nicotine abstinence,​ crossed between nicotine and placebo patch) and 28 matched nonsmokers served in a randomized,​ sham-controlled,​ double-blind,​ crossover design with 3 conditions of 25 min,​ 2mA tDCS: anodal L-dlPFC + cathodal R-vmPFC ("An-dlPFC"); polarity reversed ("CatdlPFC"); and sham. Tasks probed relevant cognitive constructs (error monitoring: parametric Flanker task; WM: N-back task; emotional reactivity: matching faces task),​ and brain activity measured with simultaneous fMRI (3T Siemens Prisma; NIDA-IRP,​ Baltimore,​ MD; 2017-2019). We generated two statistical models to test tDCS effects on two aspects of smoking addiction: Trait,​ the between-subjects factor of nonsmokers vs. sated smokers; and State,​ the within-smokers factor of nicotine withdrawal vs. nicotine sated. Behavioral outcomes included task accuracy,​ response time,​ and d-prime sensitivity. Images were preprocessed with BIDS-app fmriprep,​ 1st level and group processed in AFNI,​ with statistical testing completed in R. We measured BOLD signal to detect tDCS effects on a priori regions of interest (ROIs) for each task,​ Bonferroni-corrected for number of ROIs: N-back,​ 9 ECN-related ROIs; Flanker,​ 3 salience processing ROIs; Emotion,​ 6 amygdala sub-regions. We further conducted an exploratory,​ familywise error corrected (FWE,​ α < 0.01,​ p-voxelwise = 0.001),​ whole-brain search for effects of tDCS on each task. Results: Behavior: Sated smokers were more accurate than Nonsmokers (p = 0.03),​ and had a lower criterion to detect signal (p = 0.03),​ on the N-back task. Within smokers,​ nicotine deprivation induced slower response times (p < 0.05,​ N-back,​ Flanker,​ and Matching),​ reduced accuracy (p < 0.05,​ N-back and Matching),​ higher signal criterion and response omission rate (Nback,​ p < 0.05). We did not observe tDCS effects on task behavior. ROI analysis: An-dlPFC tDCS increased right anterior cingulate cortex (ACC) activity in the smoker group to a greater degree than the nonsmoker group (p = 0.02) across all difficulty levels of Flanker. Whole-brain analysis: We observed that An-dlPFC tDCS strengthened the deactivation of 14 DMN associated regions (including hippocampal and parahippocampal gyri,​ mid-cingulate gyrus,​ precuneus,​ and temporal regions) across all subjects on the N-back Trait model. In the State model,​ An-dlPFC tDCS reduced activity in the same DMN-related regions,​ however the effect was more prominent in the nicotine sated vs. withdrawal state. Conclusions: Single session acute tDCS enhanced the deactivation of DMN nodes during a WM task,​ and enhanced ACC activity during an error monitoring task. While all subjects were sensitive to tDCS effects on DMN,​ smokers were more sensitive to tDCS effects on ACC. Further,​ smokers were more sensitive to tDCS effects on DMN in the nicotine-sated state,​ in which subjects were generally more attentive to task and therefore potentially more receptive to an adjuvant modifier,​ than during withdrawal,​ when subjects demonstrated reduced task engagement (slower responses,​ reduced accuracies) and attention (more omissions). Thus,​ the cognitive circuit dysregulation associated with NWS,​ and often alleviated by nicotine replacement therapy,​ may be further modifiable by "adjuvant" anodal,​ excitatory tDCS applied to LdlPFC. Use of tDCS as a complement to standard therapy has been successfully tested in depression,​ in which combined tDCS + antidepressant medication improved outcomes more than either alone. The present data support the possible use of tDCS as a complementary therapy to other,​ standard treatments for nicotine addiction,​ especially the dysregulated cognitive processes seen during the NWS. This work was supported by the NIDA-IRP. |
| 190 | McCarthy,​ Danielle E,​ Versella,​ Mark V. Quitting Failure and Success With and Without Using Medication: Latent Classes of Abstinence and Adherence to Nicotine Monotherapy,​ Combination Therapy,​ and Varenicline. Nicotine & tobacco research : official journal of the Society for Research on Nicotine and Tobacco. 2019;21:1488.  **Abstract:** INTRODUCTIONNonadherence to pharmacotherapies complicates studies of comparative pharmacotherapy effectiveness. Modeling adherence and abstinence simultaneously may facilitate analysis of both treatment acceptability and effectiveness.METHODSSecondary analyses of a three-arm randomized comparative trial of nicotine patch,​ varenicline,​ and combination nicotine patch and lozenge among adult daily smokers (N = 1086) were conducted. Adherence rates collected via interactive voice response systems during the first 27 days of quitting were compared across treatment conditions. Repeated measures latent class analyses of adherence and abstinence in 3-day parcels through 27 days of a quit attempt were conducted with treatment,​ demographic,​ and smoking history covariates.RESULTSAdherence varied across treatments and was lowest for nicotine lozenge use in combination nicotine replacement therapy (NRT). Five latent classes that differed significantly in 6-month abstinence rates were retained,​ including three subgroups of adherent participants varying in treatment response and two nonadherent groups varying in abstinence probabilities. Nonadherence was more likely among those receiving varenicline and combination NRT,​ relative to patch monotherapy. Varenicline and combination NRT did not promote abstinence among adherent latent classes but did promote abstinence among those partially adherent,​ relative to patch alone. Combination therapy attenuated increased risk of treatment disengagement with more years smoking. Minority smokers,​ those high in dependence,​ and those with shorter past abstinence were at increased risk for low-adherence and low-abstinence latent classes.CONCLUSIONSVarenicline and combination nicotine patch and lozenge are less likely to be used as directed and may not increase first-month abstinence better than patch alone when taken adherently.IMPLICATIONSThis secondary analysis of adherence and abstinence in a comparative effectiveness trial shows that adherence is highest for the nicotine patch,​ next highest for varenicline,​ and lowest for combination nicotine patch and lozenge therapy due to low lozenge use. Distinct latent classes were found that varied in both first-month abstinence and adherence. Varenicline and combination NRT may not enhance abstinence over patch alone among smokers who take medication adherently. Adherent use of medication especially benefits those who are low in dependence and have positive quitting histories; it is less beneficial to at-risk smokers and members of racial minorities. |
| 201 | Vega,​ Jennifer N,​ Albert,​ Kimberly M,​ Mayer,​ Ingrid A,​ Taylor,​ Warren D,​ Newhouse,​ Paul A. Nicotinic treatment of post-chemotherapy subjective cognitive impairment: a pilot study. Journal of cancer survivorship : research and practice. 2019;13:673.  **Abstract:** PURPOSEPersistent chemotherapy-related cognitive impairment (pCRCI) is commonly reported following cancer treatment and negatively affects quality of life; however,​ there is currently no pharmacological treatment indicated for pCRCI. This pilot study obtained preliminary data regarding the use of transdermal nicotine patches as a therapeutic strategy for women with pCRCI to (1) reduce subjective cognitive complaints and (2) enhance objective cognitive performance in breast,​ colon,​ lymphoma,​ or ovarian cancer survivors with pCRCI.METHODSParticipants were randomized to either placebo (n = 11) or transdermal nicotine (n = 11) for 6 weeks,​ followed by 2 weeks of treatment withdrawal for a total of 8 weeks. Participants were assessed using both subjective and objective measures of cognitive functioning at five visits before,​ during,​ and after treatment.RESULTSOver the course of the study,​ women in both groups improved substantially in severity of self-reported cognitive complaints measured by Functional Assessment of Cancer Therapy-Cognitive Function Perceived Cognitive Impairments regardless of treatment arm. Additionally,​ objective cognitive performance measures improved in both groups; however,​ there was no significant difference in improvement between groups.CONCLUSIONSDue to a large placebo response,​ we were unable to determine if a drug effect was present. However,​ we did observe substantial improvement in self-reported cognitive symptoms,​ likely resulting from factors related to participation in the trial rather than specific drug treatment effects.TRIAL REGISTRATIONThe study was registered with clinicaltrials.gov (trial registration: NCT02312943).IMPLICATIONS FOR CANCER SURVIVORSThese results suggest that women with pCRCI can exhibit improvement in subjective cognition,​ with attention paid to symptoms and close follow-up over a short period of time. |
| 204 | Hahn,​ Britta,​ Shrieves,​ Megan E,​ Yuille,​ Marie B,​ Buchanan,​ Robert W,​ Wells,​ Ashleigh K. Nicotine effects on cognitive remediation training outcome in people with schizophrenia: A pilot study. Psychiatry research. 2019;280:112498.  **Abstract:** Cognitive remediation training can alleviate cognitive impairment associated with schizophrenia,​ but the impact is limited by small effect sizes. The present study aimed at augmenting training effects by administering nicotine prior to training sessions. Twenty-five people with schizophrenia were enrolled in a 10-week,​ 5 days/week,​ computerized cognitive training regimen. Participants were randomized to two treatment groups: nicotine or placebo. Every Monday and Thursday,​ the nicotine group received a nicotine lozenge before the training,​ and the placebo group a placebo lozenge. Outcome measurements were conducted on a no-lozenge day in weeks 0,​ 4,​ 7,​ and 10,​ and at 4-week follow-up. The MATRICS Consensus Cognitive Battery composite score improved over time,​ but there was no group difference in this effect. A significant group difference emerged over time in the reasoning/problem solving sub-domain: the placebo group improved but not the nicotine group,​ suggesting that nicotine exposure negatively impacted training benefits on executive control processes. There were no effects on psychiatric symptoms. However,​ significant improvements were seen across groups on the Quality of Life Scale and the Cognitive Assessment Interview,​ measuring real-life functional outcome. In conclusion,​ the present study failed to find evidence that nicotine exposure during cognitive remediation training may potentiate training benefits. |
| 209 | Claire,​ Ravinder,​ Coleman,​ Tim,​ Leonardi-Bee,​ Jo,​ Berlin,​ Ivan. Saliva cotinine concentrations in pregnant women who smoke and use nicotine patches. Addiction (Abingdon,​ England). 2019;114:1651.  **Abstract:** BACKGROUND AND AIMSDue to concerns about increased exposure to nicotine,​ pregnant women using nicotine replacement therapy (NRT) to stop smoking are usually advised to stop using NRT if they relapse to smoking. This study investigated whether this is justified. We compared changes in saliva cotinine from baseline to 2 weeks post-target quit date pregnant smokers who relapsed to smoking and continued to use their patches having been assigned to use nicotine patches or placebo.DESIGN AND SETTINGControlled pre-post design stratified by intervention condition from the 'Study of Nicotine Patch in Pregnancy',​ a randomized,​ placebo-controlled trial.PARTICIPANTSA sample of 268 pregnant women,​ assigned placebo (n = 122) or nicotine (n = 146) patches,​ who returned for further supplies of patches and who reported any smoking in the week prior to a visit at 2 weeks after their target quit date.MEASUREMENTSSaliva cotinine concentrations were measured at baseline and 2 weeks after participants' target quit dates. Any smoking in the previous week was assessed by self-report,​ validated by expired air carbon monoxide (CO).FINDINGSThere was no change in saliva cotinine concentrations between baseline and 2 weeks post-target quit date in saliva cotinine concentration in the nicotine patch group [ratio of geometric means = 0.94,​ 95% confidence interval (CI) = 0.83 to 1.07; P = 0.37,​ Bayes factor = 0.15]. However,​ there was a reduction in reported number of cigarettes smoked/day (mean difference -6,​ 95% CIs -7 to -5,​ P < 0.001) and in CO concentrations (mean difference -3.0 parts per million,​ 95% CIs -4.2 to -1.9,​ P < 0.001). These changes were not significantly different from changes in the placebo group except for cigarette consumption,​ which reduced more in the nicotine group (P = 0.046).CONCLUSIONSIn women trying to stop smoking with the aid of a nicotine patch but having smoked at 2 weeks post-target quit,​ their nicotine concentration did not change from baseline,​ but they reported smoking fewer cigarettes and had lower carbon monoxide concentrations. |
| 214 | Dai, Siyu, Chan, Kate Ching Ching. Effectiveness of a smoking reduction intervention programme for smoking parents of paediatric patients in Hong Kong. European Respiratory Journal. 2019; 54(S63):2864.  **Abstract:** Background: Parental smoking is a major source of environmental tobacco smoke exposure in children. Encounter of these smoking parents through medical care of the paediatric patients serves good opportunities to intervene. Methods: This is an on-going randomized controlled trial. Smoking parents recruited from the paediatric in-patient and out-patient units of Prince of Wales Hospital were allocated randomly into the intervention or control group. Intervention group received monthly counselling on smoking reduction and nicotine replacement therapy,​ while control group received standard advice on smoking cessation. Our primary outcome was successful smoking reduction rate at week-24,​ which was defined as a self-reported reduction of daily cigarette consumption by 50% or more compared with baseline. Results: 166 smoking parents (mean age: 37.6±7.6 yrs.; male: 86.7%) were included in this preliminary analysis. The baseline mean number of cigarettes smoked per day was 14.9±8.3. Using intention-to-treat analysis,​ the successful smoking reduction rate in the intervention group (44.0%) was higher than the control group (15.9%). The intervention was shown to be effective both in the univariate analysis (OR=4.18,​ 95% CI: 2.01-8.69,​ P<0.001) and in the multivariate analysis (AOR=2.61,​ 95% CI: 1.07-6.40,​ P=0.03). The adjusted confounders included demographic characteristics,​ parental baseline smoking condition and nicotine dependence level,​ and the paediatric patients' disease severity. Conclusions: In this preliminary analysis,​ our designed intervention was shown to be effective in smoking reduction of smoking parents of paediatric patients in Hong Kong. |
| 216 | Heffner,​ Jaimee L,​ Evins,​ A Eden,​ Russ,​ Cristina,​ Lawrence,​ David,​ Ayers,​ Catherine R,​ McRae,​ Thomas,​ Aubin,​ Lisa St,​ Krishen,​ Alok,​ West,​ Robert,​ Anthenelli,​ Robert M. Safety and efficacy of first-line smoking cessation pharmacotherapies in bipolar disorders: Subgroup analysis of a randomized clinical trial. Journal of affective disorders. 2019;256:267.  **Abstract:** OBJECTIVESPost hoc analyses of EAGLES data to examine safety and efficacy of first-line smoking cessation pharmacotherapies in smokers with bipolar disorders (BD).METHODSSmokers with BD I/II (n = 285; 81.4% with BD I) and a comparison nonpsychiatric cohort (NPC; n = 2794) were randomly assigned to varenicline,​ bupropion,​ nicotine replacement therapy (NRT),​ or placebo for 12 weeks,​ plus weekly counseling. Primary outcomes were occurrence of moderate to severe neuropsychiatric adverse events (NPSAEs) and Weeks 9-12 biochemically-confirmed continuous abstinence (CA) rates.RESULTSFor BD smokers,​ NPSAE risk differences versus placebo were: varenicline,​ 6.17 (95% CI: -7.84 to 20.18); bupropion,​ 4.09 (-8.82 to 16.99); NRT,​ -0.56 (-12.34 to 11.22). ORs for Weeks 9-12 CA,​ comparing active medication to placebo among BD smokers were: varenicline,​ 2.61 (0.68-9.95); bupropion,​ 1.29 (0.31-5.37),​ NRT,​ 0.71 (0.14-3.74). Pooling across treatments,​ NPSAE occurrence was higher (10.7% versus 2.3%; P < 0.001) and CA rates were lower (22.8% versus 13.3%; P = 0.008) in BD than NPC.LIMITATIONSStudy not powered to detect differences in safety and efficacy in the BD subcohort; generalizability limited to stably treated BD without current substance use disorders.CONCLUSIONSSmokers with BD had higher risk of NPSAEs and were less likely to quit overall than NPC smokers. Among smokers with BD,​ NPSAE risk difference estimates for active treatments versus placebo ranged from 1% lower to 6% higher. Efficacy of varenicline in smokers with BD was similar to EAGLES main outcomes; bupropion and NRT effect sizes were descriptively lower. Varenicline may be a tolerable and effective cessation treatment for smokers with BD.TRIAL REGISTRATIONClinicalTrials.gov identifier (https://clinicaltrials.gov/): NCT01456936. |
| 223 | O'Reilly,​ Christian,​ Chapotot,​ Florian,​ Pittau,​ Francesca,​ Mella,​ Nathalie,​ Picard,​ Fabienne. Nicotine increases sleep spindle activity. Journal of sleep research. 2019;28:e12800.  **Abstract:** Studies have shown that both nicotine and sleep spindles are associated with enhanced memorisation. Further,​ a few recent studies have shown how cholinergic input through nicotinic and muscarinic receptors can trigger or modulate sleep processes in general,​ and sleep spindles in particular. To better understand the interaction between nicotine and sleep spindles,​ we compared in a single blind randomised study the characteristics of sleep spindles in 10 healthy participants recorded for 2 nights,​ one with a nicotine patch and one with a sham patch. We investigated differences in sleep spindle duration,​ amplitude,​ intra-spindle oscillation frequency and density (i.e. spindles per min). We found that under nicotine,​ spindles are more numerous (average increase: 0.057 spindles per min; 95% confidence interval: [0.025-0.089]; p = .0004),​ have higher amplitude (average amplification: 0.260 μV; confidence interval: [0.119-0.402]; p = .0032) and last longer (average lengthening: 0.025 s; confidence interval: [0.017-0.032]; p = 2.7e-11). These results suggest that nicotine can increase spindle activity by acting on nicotinic acetylcholine receptors,​ and offer an attractive hypothesis for common mechanisms that may support memorisation improvements previously reported to be associated with nicotine and sleep spindles. |
| 225 | Iyen,​ Barbara,​ Vaz,​ Luis R,​ Taggar,​ Jaspal,​ Cooper,​ Sue,​ Lewis,​ Sarah,​ Coleman,​ Tim. Is the apparently protective effect of maternal nicotine replacement therapy (NRT) used in pregnancy on infant development explained by smoking cessation?: secondary analyses of a randomised controlled trial. BMJ open. 2019;9:e024923.  **Abstract:** OBJECTIVETo investigate relationships between maternal smoking status in pregnancy and infant development. The largest randomised controlled trial of nicotine replacement therapy (NRT) for smoking cessation in pregnancy,​ the smoking,​ nicotine and pregnancy (SNAP) trial,​ found that at 1 month after randomisation,​ smoking cessation rates were doubled in the NRT group compared with the placebo group. At delivery,​ there was no significant difference in cessation rates between groups. Surprisingly,​ infants born to women randomised to NRT were more likely to have unimpaired development at 2 years. We hypothesised that this apparently protective effect was due to smoking cessation caused by NRT and so,​ investigate this relationship using the same cohort.DESIGNSecondary analysis of a randomised controlled trial.SETTINGSeven antenatal hospitals in the Midlands and North-West England.PARTICIPANTSEight hundred and eighty-four pregnant smokers randomised to receive either NRT patches or visually-identical placebo in the SNAP trial. Participants' smoking behaviour were recorded at randomisation,​ 1 month after their target quit date and at delivery.METHODSUsing logistic regression models,​ we investigated associations between participants' smoking measures and infant development (assessed using the Ages and Stages questionnaire) at 2 years.MAIN OUTCOME MEASURES2 year infant development.RESULTSDevelopmental impairment was reported for 12.7% of study 2 year olds. Maternal heaviness of smoking at randomisation (OR: 1.26,​ 95% CI: 0.82 to 1.96,​ p=0.091),​ validated smoking abstinence recorded at 1 month after a quit date (OR: 1.02,​ 95% CI: 0.60 to 1.74,​ p=0.914) and validated smoking abstinence recorded at both 1 month after a quit date and at the end of pregnancy (OR: 1.52,​ 95% CI: 0.81 to 2.85,​ p=0.795) were not independently associated with infant developmental impairment at 2 years.CONCLUSIONWe found no evidence that NRT treatment improved infants' developmental outcomes through smoking cessation.TRIAL REGISTRATION NUMBERCTA03057/0002/001-0001; Post-results. |
| 226 | Nakagata, Takashi, Fukao, Kosuke, Kobayashi, Hiroyuki, Katamoto, Shizuo, Naito, Hisashi. The Effects of Transdermal Nicotine Patches on the Cardiorespiratory and Lactate Responses During Exercise from Light to Moderate Intensity: Implications for Exercise Prescription during Smoking Cessation. Medicina (Kaunas,Lithuania). 2019; 55(7):348.  **Abstract:** Background and objectives: Exercise can help ease withdrawal symptoms of smokers. However,​ there is little information about the physiological responses,​ such as cardiorespiratory and lactate (La) responses,​ during exercise from light to moderate intensity combined with transdermal nicotine patches (TNPs) in smokers. This study aimed to investigate the effect of TNPs on the cardiorespiratory and La responses during exercise at light to moderate intensity. Materials and Methods: Fourteen young men (8 non-smokers,​ 6 current smokers) aged 20 to 26 years participated in this study. They performed an incremental graded submaximal exercise test using an electromagnetic cycle ergometer set from 30 to 210 W with (TNP condition) or without a TNP (control condition) in a random order. The TNP was applied to the left arm 8-10 h prior to starting the exercise to achieve the peak level of blood nicotine concentration. Heart rate (HR),​ rate of perceived exertion (RPE),​ oxygen consumption (VO2),​ ventilation (VE),​ and blood La at rest and during exercise were measured and analyzed. Results: The HR at rest was significantly higher in the TNP condition than in the control condition (TNP; 74.7 ± 13.8 bpm,​ control; 65.3 ± 10.8 bpm,​ p < 0.001). There was no interaction (condition × exercise intensity) between any of the variables,​ and VO2,​ VE,​ RPE,​ and La during exercise were not significantly different between the conditions. However,​ HR during exercise was 6.7 bpm higher on average in the TNP condition. Conclusions: The HR during exercise was greater at light to moderate intensity with a TNP. Our study results will guide clinicians or health professionals when prescribing exercise programs combined with TNPs for healthy young smokers. |
| 231 | Anthenelli,​ Robert M,​ Gaffney,​ Michael,​ Benowitz,​ Neal L,​ West,​ Robert,​ McRae,​ Thomas,​ Russ,​ Cristina,​ Lawrence,​ David,​ St Aubin,​ Lisa,​ Krishen,​ Alok,​ Evins,​ A Eden. Predictors of Neuropsychiatric Adverse Events with Smoking Cessation Medications in the Randomized Controlled EAGLES Trial. Journal of general internal medicine. 2019;34:862.  **Abstract:** BACKGROUNDPre-treatment factors that increase smokers' risk of experiencing neuropsychiatric adverse events (NPSAEs) when quitting smoking are unknown.OBJECTIVETo identify baseline smoker characteristics beyond the history of mental illness that predict which participants were more likely to experience moderate to severe NPSAEs in EAGLES.DESIGNA prospective correlational cohort study in the context of a multinational,​ multicenter,​ double-blind,​ randomized trial.PARTICIPANTSSmokers without (N = 3984; NPC)/with (N = 4050; PC) histories of,​ or current clinically stable,​ psychiatric disorders including mood (N = 2882; 71%),​ anxiety (N = 782; 19%),​ and psychotic (N = 386; 10%) disorders.INTERVENTIONSBupropion,​ 150 mg twice daily,​ or varenicline,​ 1 mg twice daily,​ versus active control (nicotine patch,​ 21 mg/day with taper) and placebo for 12 weeks with 12-week non-treatment follow-up.MAIN MEASURESPrimary safety outcome was the incidence of a composite measure of moderate/severe NPSAEs. Associations among baseline demographic/clinical characteristics and the primary safety endpoint were analyzed post hoc via generalized linear regression.KEY RESULTSThe incidence of moderate to severe NPSAEs was higher among smokers in the PC (238/4050; 5.9%) than in the NPC (84/3984; 2.1%). Three baseline characteristics predicted increased risk for experiencing clinically significant NPSAEs when quitting regardless of carrying a psychiatric diagnosis: current symptoms of anxiety (for every ~ 4-unit increase in HADS anxiety score,​ the absolute risk of occurrence of the NPSAE endpoint increased by 1% in both PC and NPC); prior history of suicidal ideation and/or behavior (PC,​ 4.4% increase; P = 0.001; NPC,​ 4.1% increase; P = 0.02),​ and being of White race (versus Black: PC,​ 2.9% ± 0.9 [SE] increase; P = 0.002; and NPC,​ 3.4% ± 0.8 [SE] increase; P = 0.001). Among smokers with psychiatric disorders,​ younger age,​ female sex,​ history of substance use disorders,​ and proxy measures of nicotine dependence or psychiatric illness severity also predicted greater risk. There were no significant interactions between these characteristics and treatment. Smokers with unstable psychiatric disorders or with current,​ active substance abuse were excluded from the study.CONCLUSIONSIrrespective of cessation pharmacotherapy use,​ smokers attempting to quit were more likely to experience moderate to severe NPSAEs if they reported current anxiety or prior suicidal ideation at baseline and were White. In smokers with a psychiatric history,​ female sex,​ younger age,​ and greater severity of nicotine dependence were also predictive.TRIAL REGISTRATIONClinicalTrials.gov Identifier: NCT01456936. |
| 235 | Kaye,​ J.T.,​ Piper,​ M.E.,​ Baker,​ T.B.,​ Cook,​ J.W.. Searching for personalized medicine for heavy drinking smokers: smoking cessation using varenicline,​ nicotine patch,​ or combination nicotine replacement therapy. Alcoholism: Clinical and Experimental Research. 2019;43:247A.  **Abstract:** Purpose: Heavy drinking smokers are at risk for a wide array of health concerns and are less likely to successfully quit smoking relative tomoderate drinkers. Preliminary evidence suggests that varenicline may be particularly useful in aiding heavy drinking smokers to quit smoking and reduce alcohol use. We performed secondary data analysis of a large comparative effectiveness trial of smoking cessation pharmacotherapies to: 1) evaluate the relations between baseline alcohol use patterns (heavy drinker,​ moderate drinker,​ and non/infrequent drinker) and point-prevalence abstinence at 4-,​ 12-,​ and 26-weeks,​ and 2) explore whether different pharmacotherapies are especially effective for heavy drinkers. Methods: Smokers motivated to quit cigarettes (N = 1086) were randomized to receive varenicline,​ nicotine patch,​ or combination nicotine replacement therapy (C-NRT; nicotine patch + nicotine lozenge) in a 12-week open-label smoking cessation trial. Results: Results showed that 27% of participants were classified as heavy drinkers (>1 binge/ month in past year),​ 39% as moderate drinkers (>1 day/month in past year but did not meet criteria for heavy drinking),​ and 34%as non/infrequent drinkers (< 1/month in past year). Heavy drinkers reported lower 4-week abstinence rates (29%) compared to moderate drinkers (40%; OR = 1.5,​ 95%CI = 1.1 2.1),​ but these differences did not persist over time. There was not a significant interaction between alcohol use and treatment at any time point,​ suggesting that treatments were not differentially effective overall as a function of baseline alcohol use,​ p's >.28. Focused follow up tests did not find varenicline to be more effective for smoking cessation than NRT and C-NRT for heavy drinking smokers in particular. Conclusions: Contrary to expectations,​ this large comparative effectiveness trial did not indicate that varenicline leads to superior smoking cessation outcomes for heavy drinking smokers relative to NRT/C-NRT. Despite growing interest in varenicline as a potential targeted treatment for heavy drinking smokers,​ this study did not provide support this avenue of personalized medicine. |
| 243 | Hahn,​ Britta,​ McComas,​ Megan,​ Marie,​ Yuille,​ Robert,​ Buchanan,​ Ashleigh,​ Wells. Effects of Nicotine on Cognitive Remediation Training in Schizophrenia. Biological Psychiatry. 2019;85:S192.  **Abstract:** Background: Cognitive remediation training can alleviate cognitive deficits associated with schizophrenia,​ but its impact is limited by small effect sizes. We aimed at enhancing the beneficial effects of the training challenges by administering nicotine prior to some of the training sessions. Nicotine-induced facilitation of sensory processing,​ alertness/attention,​ and learning/memory was expected to promote training benefits. Methods: Twenty-five people with schizophrenia completed a 10-week,​ 5 days/week,​ computerized auditory and visual cognitive training regimen. Every Monday and Thursday,​ participants randomized to the nicotine group received a nicotine polacrilex lozenge (2 or 4 mg,​ depending on smoking status) prior to the training,​ and participants in the placebo group a placebo lozenge. Outcome measures were taken on a no-lozenge day at baseline and every 3-4 weeks thereafter. Results: The MATRICS Consensus Cognitive Battery (MCCB) composite score improved over time,​ but there were no group differences in this effect. When exploring the seven MCCB sub-domains,​ only the Reasoning/Problem Solving domain displayed a Group x Time interaction (P=0.003); the placebo group improved over time,​ but not the nicotine group,​ suggesting that intermittent nicotine exposure negatively impacted training benefits on higher-order cognitive processes. Psychiatric symptoms did not change from before to after the training intervention in either group. However,​ significant improvements from pre- to post-intervention were seen on the Quality of Life Scale (P<0.05) and the Cognitive Assessment Interview (P<0.001),​ both measuring real-life functional outcome. These effects did not differ between treatment groups. Conclusions: In conclusion,​ there was no evidence that nicotine exposure during cognitive remediation training may potentiate training benefits. Supported By: R21 MH095824 (B. Hahn) Keywords: Schizophrenia,​ Cognitive Deficits,​ Cognitive Remediation Training,​ Nicotine,​ Clinical Trial |
| 253 | Kruse,​ Gina R.,​ Park,​ Elyse,​ Chang,​ Yuchiao,​ Haberer,​ Jessica,​ Abroms,​ Lorien,​ Shahid,​ Naysha N.,​ Howard,​ Sydney,​ Haas,​ Jennifer,​ Rigotti,​ Nancy A.. A proactive text messaging intervention with medication adherence support tailored to primary care populations: A pilot randomized trial of getready2quit. Journal of General Internal Medicine. 2019;34:S114.  **Abstract:** Background: Text messaging is an effective aid to help with smoking cessation,​ but it is unclear how to integrate it effectively into primary care settings. We developed and tested an automated,​ proactive,​ text messaging program with 2 novel uses: 1) to enhance motivation for smokers not ready to quit and 2) to promote adherence to nicotine replacement therapy (NRT). We present feasibility and efficacy Results for a 12-week pilot randomized controlled trial (RCT). Methods: We used proactive telephone calls to recruit adult daily smokers from 10/2017-10/2018 for a RCT. Randomization was stratified by clinic and smoker's plan to quit within 30 days. Enrollees were assigned to 1 of 4 groups: brief advice about treatment options and local services (BA),​ brief advice + text messages (TM),​ brief advice + 2-weeks of NRT by mail (NRT),​ or brief advice + TM + NRT (TM+NRT). Patients were surveyed by phone or email at 1-,​ 2-,​ 6-and 12-weeks. Results: We approached 1,​544 potentially eligible patients,​ we reached 988 (64%). Of these,​ 527 (53%) declined,​ 32 (3%) dropped out before randomization,​ 276 (28%) were ineligible and 153 (15%) of those reached were randomized. Of these,​ 54% were women,​ mean age 53 years,​ 76% white,​ 12% African-American,​ 6% Hispanic,​ 5% other races,​ 54% on Medicaid,​ mean 15 cigarettes/day and 75% smoked < 30 minutes from awakening. Overall,​ 31% screened positive for alcohol use disorder (single-item measure),​ 13% for substance use (single-item measure),​ 35% for depression (PHQ-2) and 55% for anxiety (GAD-2). Acceptance of treatments offered (BA,​ TM,​ and/or NRT) was 100%. Retention at the 12-week follow-up was 92% (n=140). A large majority of patients self-reported 1 quit attempt(s) (1oclinical outcome),​ with no differences by group compared to BA (71%),​ TM (89%,​ p=0.13),​ NRT (82%,​ p=0.40),​ TM+NRT (81%,​ p=0.41). Past 7-day abstinence at 12-weeks was reported twice as often in the NRT,​ TM,​ and TM+NRT groups,​ though differences were not significant compared to BA (11%),​ TM (29%,​ p=0.13),​ NRT (30%,​ p=0.07),​ and TM+NRT (22%,​ p=0.35). Groups offered NRT by mail were more likely to report NRT use compared to BA (51%),​ TM (63%,​ p=0.47),​ NRT (82%,​ p=0.011),​ and TM+NRT (76%,​ p=0.049). There was no difference in days of NRT use over the first 2 weeks between NRT only (4.6 days of NRT use) or NRT+TM (3.0 days,​ p=0.13). Conclusions: This pilot RCT demonstrated feasibility as we recruited,​ treated,​ and retained smokers from a primary care network. Our uptake of 15% is comparable to other proactive outreach interventions for smokers. A proactive offer to have NRT mailed to patients' homes increased NRT use,​ even though many patients already had access to low-cost NRT from Medicaid. Adherence messages in the texting program did not increase NRT use. Proactively-delivered smoking cessation interventions that are offered outside office visits are feasible and hold promise for reducing smoking rates and promoting health in primary care populations. |
| 258 | Engle,​ Jessica L,​ Mermelstein,​ Robin,​ Baker,​ Timothy B,​ Smith,​ Stevens S,​ Schlam,​ Tanya R,​ Piper,​ Megan E,​ Jorenby,​ Douglas E,​ Collins,​ Linda M,​ Cook,​ Jessica W. Effects of motivation phase intervention components on quit attempts in smokers unwilling to quit: A factorial experiment. Drug and alcohol dependence. 2019;197:149.  **Abstract:** BACKGROUNDSmoking reduction treatment is a promising approach to increase abstinence amongst smokers initially unwilling to quit. However,​ little is known about which reduction treatment elements increase quit attempts and the uptake of cessation treatment amongst such smokers.METHODSThis study is a secondary analysis of a 4-factor randomized factorial experiment conducted amongst primary care patients (N = 517) presenting for regular healthcare visits in Southern Wisconsin who were unwilling to quit smoking but willing to cut down. We evaluated the main and interactive effects of Motivation-phase intervention components on whether participants: 1) made a quit attempt (intentional abstinence ≥24 h) by 6- and 26-weeks post-study enrollment and,​ 2) used cessation treatment. We also evaluated the relations of quit attempts with abstinence. The four intervention components evaluated were: 1) Nicotine Patch vs. None; 2) Nicotine Gum vs. None; 3) Motivational Interviewing (MI) vs. None; and 4) Behavioral Reduction Counseling (BR) vs. None. Intervention components were administered over 6 weeks,​ with an option to repeat treatment; participants could request cessation treatment at any point.RESULTSNicotine gum significantly increased the likelihood of making a quit attempt by 6 weeks (23% vs. 15% without gum; p < .05). Conversely,​ nicotine patch reduced quit attempts when used with BR. Patch also discouraged use of cessation treatment (15.8% vs. 23% without patch; p < .05). Aided vs. unaided quit attempts produced abstinence in 42% vs. 10% of participants,​ respectively.CONCLUSIONNicotine gum is a promising Motivation-phase intervention that may spur quit attempts amongst smokers initially unwilling to quit. |
| 259 | Mündel,​ Toby,​ Houltham,​ Stuart D,​ Barnes,​ Matthew J,​ Stannard,​ Stephen R. Nicotine Supplementation Does Not Influence Performance of a 1h Cycling Time-Trial in Trained Males. Frontiers in physiology. 2019;10:292.  **Abstract:** The use of nicotine amongst professional and elite athletes is high,​ with anecdotal evidence indicating increased prevalence amongst cycling sports. However,​ previous investigations into its effects on performance have not used high-validity or -reliability protocols nor trained cyclists. Therefore,​ the present study determined whether nicotine administration proved ergogenic during a ∼1 h self-paced cycling time-trial (TT). Ten well-trained male cyclists (34 ± 9 years; 71 ± 8 kg; O2max: 71 ± 6 ml ⋅ kg-1 ⋅ min-1) completed three work-dependent TT following ∼30 min administration of 2 mg nicotine gum (GUM),​ ∼10 h administration of 7 mg ⋅ 24 h-1 nicotine patch (PAT) or color- and flavor-matched placebos (PLA) in a randomized,​ crossover,​ and double blind design. Measures of nicotine's primary metabolite (cotinine),​ core body temperature,​ heart rate,​ blood biochemistry (pH,​ HCO3 -,​ La-) and Borg's rating of perceived exertion (RPE) accompanied performance measures of time and power output. Plasma concentrations of cotinine were highest for PAT,​ followed by GUM,​ then PLA,​ respectively (p < 0.01). GUM and PAT resulted in no significant improvement in performance time compared to PLA (62.9 ± 4.1 min,​ 62.6 ± 4.5 min,​ and 63.3 ± 4.1 min,​ respectively; p = 0.73),​ with mean power outputs of 264 ± 31,​ 265 ± 32,​ and 263 ± 33 W,​ respectively (p = 0.74). Core body temperature was similar between trials (p = 0.33) whilst HR averaged 170 ± 10,​ 170 ± 11,​ and 171 ± 11 beats ⋅ min-1 (p = 0.60) for GUM,​ PAT,​ and PLA,​ respectively. There were no differences between trials for any blood biochemistry (all p > 0.46) or RPE with mean values of 16.7 ± 0.9,​ 16.8 ± 0.7,​ and 16.8 ± 0.8 (p = 0.89) for GUM,​ PAT,​ and PLA,​ respectively. In conclusion: (i) nicotine administration,​ whether via gum or transdermal patch,​ did not exert an ergogenic or ergolytic effect on self-paced cycling performance of ∼1 h; (ii) systemic delivery of nicotine was greatest when using a transdermal patch; and (iii) nicotine administration did not alter any of the psycho-physiological measures observed. |
| 263 | Oncken,​ Cheryl,​ Dornelas,​ Ellen A,​ Kuo,​ Chia-Ling,​ Sankey,​ Heather Z,​ Kranzler,​ Henry R,​ Mead,​ Erin L,​ Thurlow,​ Ms Sheila D. Randomized Trial of Nicotine Inhaler for Pregnant Smokers. American journal of obstetrics & gynecology MFM. 2019;1:10.  **Abstract:** BackgroundSmoking during pregnancy is a serious public health problem in need of better treatments. Nicotine replacement treatment (NRT) (patch or gum) has not been shown in randomized placebo-controlled trials to be efficacious for smoking cessation during pregnancy. However,​ the nicotine inhaler may have advantages over other NRTs as it replicates some of the sensory effects of smoking.ObjectiveThe purpose of the study was examine the efficacy and safety of the nicotine inhaler for smoking cessation during pregnancy. We hypothesized that the nicotine inhaler compared to placebo would increase quit rates and reduce smoking during treatment and at the end of pregnancy,​ result in a higher birth weight and gestational age in the offspring,​ and reduce the incidence of preterm birth and low birth weight infants.Study DesignWe conducted a randomized,​ double-blind,​ placebo-controlled trial of the nicotine inhaler for smoking cessation during pregnancy. Pregnant women who smoked ≥5 cigarettes daily received behavioral counseling and random assignment to a 6-week treatment with nicotine or placebo inhaler,​ followed by a 6-week taper period. Throughout treatment,​ we assessed tobacco exposure biomarkers,​ cessation rates,​ and adverse events. We also obtained information on birth outcomes. The primary outcome was smoking cessation at 32-34 weeks gestation; secondary outcomes were smoking reduction,​ birth weight and gestational age,​ and the incidence of preterm birth or low birth weight infants. We compared treatment groups on these measures using t-tests,​ Fisher's exact tests,​ and multivariate linear and logistic regression.ResultsParticipants in the placebo (n=67) and nicotine (n=70) groups were comparable on baseline characteristics,​ though women in the placebo group reported a higher motivation to quit (p=0.016). Biochemically-validated smoking cessation rates were similar with nicotine and placebo (after 6 weeks of treatment: 4% (3/70) vs. 3% (2/67),​ respectively,​ p< 0.99,​ and at 32-34 weeks gestation: 10% (7/70) vs. 18% (12/67),​ respectively,​ p=0.220). Cigarettes per day (CPD) decreased over time in both groups (p< 0.001),​ with the nicotine inhaler group having a greater decrease than the placebo group two (p=0.022) and six weeks after the quit date (p=0.042),​ but not at 32-34 weeks gestation (p=0.108). Serum cotinine levels,​ birth weight,​ gestational age and reductions in carbon monoxide did not differ by group. However,​ the incidence of preterm delivery was higher in the placebo than the nicotine group: 15% (10/67) vs. 4% (3/67),​ respectively,​ p=0.030). The incidence of delivering a low birth weight infant was also higher in the placebo than the nicotine group: 15% (10/67) vs. 6% (4/67),​ respectively,​ p=0.035,​ but not after adjusting for preterm delivery p=0.268.ConclusionsAlthough the nicotine inhaler group did not have a higher quit rate during pregnancy than the placebo group,​ the outcome of preterm delivery occurred less frequently in the nicotine group. |
| 264 | Evins,​ A Eden,​ Benowitz,​ Neal L,​ West,​ Robert,​ Russ,​ Cristina,​ McRae,​ Thomas,​ Lawrence,​ David,​ Krishen,​ Alok,​ St Aubin,​ Lisa,​ Maravic,​ Melissa Culhane,​ Anthenelli,​ Robert M. Neuropsychiatric Safety and Efficacy of Varenicline,​ Bupropion,​ and Nicotine Patch in Smokers With Psychotic,​ Anxiety,​ and Mood Disorders in the EAGLES Trial. Journal of clinical psychopharmacology. 2019;39:108.  **Abstract:** BACKGROUNDNeuropsychiatric safety and relative efficacy of varenicline,​ bupropion,​ and transdermal nicotine patch (NRT) in those with psychiatric disorders are of interest.METHODSWe performed secondary analyses of safety and efficacy outcomes by psychiatric diagnosis in EAGLES (Evaluating Adverse Events in a Global Smoking Cessation Study),​ a 12-week,​ randomized,​ double-blind,​ triple-dummy,​ placebo- and active (NRT)-controlled trial of varenicline and bupropion with 12-week follow-up,​ in a subset population,​ n = 4092,​ with a primary psychotic (n = 390),​ anxiety (n = 792),​ or mood (n = 2910) disorder. Primary end-point parameters were incidence of prespecified moderate and severe neuropsychiatric adverse events (NPSAEs) and weeks 9 to 12 continuous abstinence rates (9-12CAR).RESULTSThe observed NPSAE incidence across treatments was 5.1% to 6.3% in those with a psychotic disorder,​ 4.6% to 8.0% in those with an anxiety disorder,​ and 4.6% to 6.8% in those with a mood disorder. Neither varenicline nor bupropion was associated with significantly increased NPSAEs relative to NRT or placebo in the psychiatric cohort or any psychiatric diagnostic subcohort. There was a significant effect of treatment on 9-12CAR (P < 0.0001) and no significant treatment-by-diagnostic subcohort interaction (P = 0.24). Abstinence rates with varenicline were superior to bupropion,​ NRT,​ and placebo,​ and abstinence with bupropion and NRT was superior to placebo. Within-diagnostic subcohort comparisons of treatment efficacy yielded estimated odds ratios for 9-12CAR versus placebo of greater than 3.00 for varenicline,​ greater than 1.90 for bupropion,​ and greater than 1.80 for NRT for all diagnostic groups.CONCLUSIONSVarenicline,​ bupropion,​ and nicotine patch are well tolerated and effective in adults with psychotic,​ anxiety,​ and mood disorders. The relative effectiveness of varenicline,​ bupropion,​ and NRT versus placebo did not vary across psychiatric diagnoses. |
| 265 | Robinson,​ Jason D,​ Li,​ Liang,​ Chen,​ Minxing,​ Lerman,​ Caryn,​ Tyndale,​ Rachel F,​ Schnoll,​ Robert A,​ Hawk,​ Larry W,​ George,​ Tony P,​ Benowitz,​ Neal L,​ Cinciripini,​ Paul M. Evaluating the temporal relationships between withdrawal symptoms and smoking relapse. Psychology of addictive behaviors : journal of the Society of Psychologists in Addictive Behaviors. 2019;33:105.  **Abstract:** Smokers attempting to quit often attribute smoking relapse to negative affect,​ craving,​ and other nicotine withdrawal symptoms. In addition,​ there is evidence that smoking relapse can increase these symptoms,​ particularly negative affect. To address this issue,​ we analyzed data from an 11-week smoking cessation clinical trial in which smokers (n = 1,​246) were randomized to receive either nicotine replacement therapy (NRT),​ varenicline,​ or placebo,​ combined with behavioral counseling. Using cross-lagged analyses,​ we examined the temporal bidirectional relationships between self-reported measures of affect,​ craving,​ and composite withdrawal symptoms and biochemically verified smoking abstinence. The relative strength of these temporal relationships was examined by comparing the explained variances of the models. The results showed that higher negative affect,​ craving,​ and composite withdrawal symptoms increased the likelihood of subsequent smoking relapse,​ and that smoking relapse led to subsequent increases in these same symptoms. A comparison of the explained variances found symptom predicting subsequent relapse models to be stronger than those where relapse predicted subsequent symptoms. Although the explained variance findings generally support a negative reinforcement conceptualization of nicotine dependence,​ the bidirectional relationship between symptoms and smoking relapse suggests that struggling with quitting smoking leads to significant negative affect,​ craving,​ and other withdrawal symptoms that do not quickly resolve. These findings highlight the importance of addressing specific symptoms within the context of smoking cessation. (PsycINFO Database Record (c) 2019 APA,​ all rights reserved). |
| 267 | Aung,​ Myo Nyein,​ Yuasa,​ Motoyuki,​ Moolphate,​ Saiyud,​ Lorga,​ Thaworn,​ Yokokawa,​ Hirohide,​ Fukuda,​ Hiroshi,​ Kitajima,​ Tsutomu,​ Tanimura,​ Susumu,​ Hiratsuka,​ Yoshimune,​ Ono,​ Koichi,​ Thinuan,​ Payom,​ Minematsu,​ Kazuo,​ Deerojanawong,​ Jitladda,​ Suya,​ Yaoyanee,​ Marui,​ Eiji. Effectiveness of a new multi-component smoking cessation service package for patients with hypertension and diabetes in northern Thailand: a randomized controlled trial (ESCAPE study). Substance abuse treatment,​ prevention,​ and policy. 2019;14:10.  **Abstract:** BACKGROUNDSmoking cessation is an achievable behavioral change,​ which reduces the risks of cardiovascular diseases,​ cancers and tobacco-related diseases. There is a need for an effective smoking cessation service for low and middle income country settings where the smoking rate is generally very high whilst a cessation service is not usually accessible. This study devised a new smoking cessation service package and assessed its effectiveness in the primary health care setting of northern Thailand.METHODSThis randomized controlled trial was centered at Maetha district hospital,​ Lampang province,​ Thailand,​ and its network of mobile non-communicable disease clinics at seven primary care units. A total of 319 eligible patients who consented to participate in the study,​ were randomly allocated to an intervention arm (160) and a control arm (159),​ applying block randomization. The multi-component intervention service consisted of: (1) regular patient motivation by the same nurse over a 3-month period; (2) a monthly piCO+ Smokerlyzer test for 3 months; (3) continual assistance from a trained family member,​ using a smoking-cessation- diary; and (4) optional nicotine replacement chewing gum therapy. The control group received the routine service comprising of brief counseling and casual follow-up. Smoking cessation,​ confirmed by six months of abstinence and the piCo+ Smokerlyzer breath test,​ was compared between the two services after a year follow-up. The trial is registered as an international current control trial at the ISRCTN registry. ISRCTN89315117.RESULTSThe median age of the participants was 64 years,​ with females constituting 28.84%. Most of the participants smoke hand-rolled cigarettes (85%). The intervention arm participants achieved a significantly higher smoking cessation rate than the control arm 25.62% vs 11.32%,​ with an adjusted odd ratio of 2.95 and 95% confidence interval 1.55-5.61.CONCLUSIONIn relation to accessing smoking cessation services within the primary health care setting,​ participants who received the evidence-based intervention package were about three times more likely to succeed in giving up smoking than those who received the routine service. Utilizing community resources as major intervention components,​ the evidence from this trial may provide a useful and scalable smoking cessation intervention for low and middle income countries.TRIAL REGISTRATIONCurrent controlled trials ISRCTN89315117 . WHO international clinical trial identifier number: U1111-1145-6916; 3/2013. |
| 272 | DiFrancisco-Donoghue,​ Joanne,​ Jung,​ Min-Kyung,​ Leder,​ Adena. Nicotine Gum as a Therapeutic Approach for Low Blood Pressure in Parkinson's Disease: A Randomized Pilot Study. Nicotine & tobacco research : official journal of the Society for Research on Nicotine and Tobacco. 2019;21:253.  **Abstract:** IntroductionOne cause for low blood pressure (BP) in Parkinson's disease (PD) is denervation of the sympathetic nervous system and reduced levels of norepinephrine. Nicotine increases heart rate and BP acutely by causing sympathetic stimulation. The absorption rate of nicotine gum is relatively quick and absorbed at a constant rate. Our objective was to evaluate how nicotine gum affects acute low BP in PD.MethodsTen subjects (age 69.3 ± 8.8) completed this double blind,​ placebo controlled,​ cross-over design trial using nicotine gum (4 mg) and placebo gum on two separate days. The gum was administered for 30 min. BP was recorded every 10 min for 90 min.ResultsOn the nicotine gum treatment day,​ the baseline systolic BP was 94.8 (standard deviation [SD] = 4.4),​ and it increased in a parabolic pattern to be 115.8 (SD = 11.2) in 20 min,​ 124.2 (SD = 9.3) in 40 min,​ and 133.2 (SD = 13.1) in 60 min reaching the highest value,​ and then decreased to be 121.6 (SD = 10.4) in 90 min. On the placebo day,​ the baseline systolic BP 95.2 (SD = 3.0) didn't show an outstanding change with the mean systolic BP values from 93.0 to 95.7 (SD from 2.1 to 3.7) at all time points.ConclusionsOur data suggests that 4 mg of nicotine gum can increase systolic BP within 10 min of administration. It is strongly warranted that further research should pursue the use of nicotine gum as an intervention to treat acute episodes of low BP in individuals with PD.ImplicationsMore than 50% of Parkinson's disease (PD) patients have low blood pressure (BP) that fluctuates throughout the day and decreases quality of life. This study found an increase in systolic blood pressure within 10 min of administering nicotine gum to Parkinson's subjects with low BP. Their BP remained elevated for 90 min. Nicotine gum gets absorbed rapidly and may act as a therapeutic novel approach to individuals whose daily lives are interrupted with low BP. |
| 283 | Hajek,​ Peter,​ Lewis,​ Sarah,​ Munafo,​ Marcus,​ Lindson,​ Nicola,​ Coleman,​ Tim,​ Aveyard,​ Paul. Mediators of the effect of nicotine pre-treatment on quitting smoking. Addiction (Abingdon,​ England). 2018;113:2280.  **Abstract:** BACKGROUND AND AIMSUsing smoking cessation medications for several weeks prior to quitting smoking facilitates quitting success,​ but how it does so is not clear. Candidate theories are that pre-cessation medication enhances self-efficacy,​ facilitates medication adherence post-quit,​ induces aversion to smoking,​ reduces reward from smoking or reduces the drive to smoke. We investigated these pathways using data from a large trial of nicotine pre-loading,​ using mediation analysis.DESIGNRandomized controlled trial of nicotine pre-loading. Potential mediators were assessed at baseline and 1 week into the pre-loading (3 weeks prior to quitting). In addition to this,​ urges to smoke in abstainers were assessed 1 week after the target quit date.SETTINGEngland.PARTICIPANTSA total of 1792 smokers who wanted to quit attending specialist smoking cessation services in England were enrolled between 13 August 2012 and 10 March 2015.INTERVENTION AND COMPARATORParticipants were randomized to either standard smoking cessation medications accompanied by behavioural support or the same treatment supplemented by nicotine 'pre-loading',​ i.e. 4 weeks of 21 mg nicotine patch use prior to quitting.MEASUREMENTSThe primary outcome,​ selected for its proximity in time to potential mediators,​ was biochemically validated abstinence from smoking at 4 weeks post-target quit date. Potential mediators included the Modified Cigarette Evaluation Questionnaire,​ with subscales assessing satisfaction,​ reward,​ craving and aversion; ratings of strength and frequency of urges to smoke; the Mood and Physical Symptoms Scale assessing cigarette withdrawal symptoms; two items from the Nicotine Dependence Syndrome Scale assessing smoking stereotypy; self-reported reduction in cigarettes per day and in carbon monoxide (CO) reading; post-target quit day (TQD) medication adherence; self-efficacy; nausea.FINDINGSPre-loading reduced urges to smoke at 3 weeks pre-quit (P < 0.001) and exhaled CO concentrations (P < 0.001),​ and also urges to smoke post-quit in abstainers (P = 0.001). At 3 weeks pre-quit,​ it also reduced cigarette consumption,​ enjoyment of and satisfaction from smoking and smoking reward and increased nausea,​ aversion (all P < 0.001) and smoking stereotypy (P = 0.003). Only the first three variables,​ however (reduced smoke intake and reduced urges to smoke pre- and post-quit),​ mediated abstinence from smoking at 4 weeks and only the latter two mediated abstinence at 6 months (indirect mediating effects P < 0.05).CONCLUSIONSNicotine pre-loading appears to facilitate smoking abstinence by reducing urges to smoke and smoke intake before quitting and urges to smoke after quitting. |
| 285 | de Jong,​ Ben,​ Schuppers,​ Anne Sophie,​ Kruisdijk-Gerritsen,​ Arriette,​ Arbouw,​ Maurits Erwin Leo,​ van den Oever,​ Hubertus Laurentius Antonius,​ van Zanten,​ Arthur R H. The safety and efficacy of nicotine replacement therapy in the intensive care unit: a randomised controlled pilot study. Annals of intensive care. 2018;8:70.  **Abstract:** BACKGROUNDStudies evaluating nicotine replacement therapy (NRT) to prevent nicotine withdrawal symptoms in ICU patients have yielded conflicting results. We performed a randomised controlled double-blind pilot study to assess the safety and efficacy of NRT in critically ill patients. Mechanically ventilated patients admitted to two medical-surgical intensive care units and smoking more than 10 cigarettes per day before ICU admission were enrolled in this study. Participants were randomised to transdermal NRT (14 or 21 mg per day) or placebo until ICU discharge or day 30. Smoking status was confirmed by the biomarkers serum cotinine and urinary NNAL. The primary endpoint was 30-day mortality. Among secondary endpoints and post hoc endpoints,​ 90-day mortality,​ safety,​ time spent without delirium,​ sedation and coma,​ and patient destination at day 30 were addressed.RESULTSWe enrolled 47 patients. No differences were found between NRT and control group patients concerning 30-day mortality (9.5 vs. 7.7%,​ p = 0.84) and 90-day mortality (14.3 vs. 19.2%,​ p = 0.67). The number of serious adverse events was comparable between groups (NRT: 4,​ control: 11,​ p = 0.13). At day 20,​ average time alive without delirium,​ sedation and coma was 16.6 days among NRT patients versus 12.6 days among control patients (p = 0.03). At day 30,​ more NRT group patients were discharged from the ICU or hospital compared with controls (p = 0.03).CONCLUSIONSNRT did not affect mortality or the number of (serious) adverse events compared with placebo. Time alive without delirium,​ sedation and coma at day 20 in NRT patients was longer than in control patients. An adequately powered randomised controlled trial to further study safety and efficacy of NRT in ICU patients seems feasible and is warranted. Trial registration ClinicalTrials.gov,​ number NCT01362959,​ registered 1 June 2011. |
| 289 | Wells,​ Quinn S,​ Freiberg,​ Matthew S,​ Greevy,​ Robert A,​ Jr,​ Tyndale,​ Rachel F,​ Kundu,​ Suman,​ Duncan,​ Meredith S,​ King,​ Stephen,​ Abney,​ Lesa,​ Scoville,​ Elizabeth,​ Beaulieu,​ Dawn B,​ Gatskie,​ Vanessa,​ Tindle,​ Hilary A. Nicotine Metabolism-informed Care for Smoking Cessation: A Pilot Precision RCT. Nicotine & tobacco research : official journal of the Society for Research on Nicotine and Tobacco. 2018;20:1489.  **Abstract:** IntroductionVarenicline doubles cessation over nicotine replacement therapy (NRT) patch for "normal,​" but not "slow,​" nicotine metabolizers,​ as assessed by the nicotine metabolite ratio (NMR). Metabolism-informed care (MIC) could improve outcomes by matching normal metabolizers with non-nicotine medication (e.g.,​ varenicline) and slow metabolizers with NRT patch.MethodsWe conducted a feasibility randomized controlled trial of MIC versus guideline based care (GBC) among 81 outpatient adult daily smokers with medical comorbidity. Participants reported perceptions of MIC,​ underwent blood draw for NMR,​ and received expert cessation counseling. For MIC participants,​ medication selection was informed by NMR result (normal (≥0.31) vs. slow (< 0.31)). The primary outcome was MIC feasibility,​ reflected by attitudes toward MIC and by match rates between NMR and medication. Secondary endpoints (cessation confidence,​ medication use,​ smoking status) were assessed over 6 months to inform future studies.ResultsParticipants were median age 53 years,​ 46% female,​ 28% black,​ and ~90% endorsed MIC. Despite high varenicline prescription rates (~60%) in both arms,​ NMR-medication matching was higher in MIC (84%) versus GBC (58%) participants (p=0.02); unadjusted odds ratio (OR) 3.67,​ 95% confidence interval [1.33,​ 11.00; p-value=0.02]. Secondary endpoints were similar at 1,​ 3,​ and 6 months.ConclusionsMIC,​ an NMR-based precision approach to smoking cessation,​ was acceptable to 90% of smokers and improved NMR-medication match rates more than 3-fold compared to GBC,​ even with generally high use of varenicline. These data support the feasibility of MIC,​ which could maximize efficacy of smoking cessation medication while minimizing side effects and cost.ImplicationsAmong treatment-seeking daily smokers with medical comorbidity,​ most viewed metabolism-informed care (MIC),​ guided by the nicotine metabolism ratio (NMR),​ favorably,​ and were willing to accept MIC-guided medication. Compared to GBC participants (58%),​ more MIC participants (84%) were prescribed NMR-matched medication (i.e.,​ normal metabolizers received varenicline; slow metabolizers received NRT patch). MIC increased the odds of optimized matching between NMR and medication more than 3-fold over GBC. Because the number needed to treat (NNT) to help one normal metabolizer quit smoking is only 4.9 for varenicline versus 26 for patch,​ broad implementation of MIC will improve drug efficacy in normal metabolizers as well as minimize side effects in slow metabolizers. |
| 290 | Renwick,​ Charlotte,​ Wu,​ Qi,​ Breton,​ Magdalena Opazo,​ Thorley,​ Rebecca,​ Britton,​ John,​ Lewis,​ Sarah,​ Ratschen,​ Elena,​ Parrott,​ Steve. Cost-effectiveness of a complex intervention to reduce children's exposure to second-hand smoke in the home. BMC public health. 2018;18:1252.  **Abstract:** BACKGROUNDSecond-hand smoke (SHS) causes numerous health problems in children such as asthma,​ respiratory tract infections and sudden infant death syndrome. The home is the main source of exposure to SHS for children,​ particularly for young children. We estimated the cost-effectiveness of a complex intervention designed to reduce SHS exposure of children whose primary caregiver feels unable or unwilling to quit smoking.METHODSA cost-effectiveness analysis was carried out alongside an open-label,​ parallel,​ randomised controlled trial in deprived communities in Nottingham,​ England. A complex intervention combining behavioural support,​ nicotine replacement therapy and personalised feedback on home air quality was compared with usual care. A total number of 205 households were recruited,​ where the main caregivers were aged 18 and over,​ with a child aged under five years living in their household reporting smoking inside their home. Analyses for this study were undertaken from the National Health Service/Personal Social Services perspective. All costs were estimated in UK pounds (£) at 2013/14 prices. The primary outcome was the incremental cost-effectiveness of change in air quality in the home,​ measured as average 16-24 h levels of particulate matter of < 2.5 μm diameter (PM2.5),​ between baseline and 12 weeks. Secondary outcomes included incremental cost per quitter,​ quit attempts and cigarette consumption in the home. A non-parametric bootstrap re-sampling technique was employed to explore uncertainty around the calculated incremental cost-effectiveness ratios.RESULTSThe complex intervention achieved reduced PM2.5 by 21.6 μg/m3 (95% CI: 5.4 to 37.9),​ with an incremental cost of £283 (95% CI: £254-£313),​ relative to usual care. The incremental cost-effectiveness ratio was £131 (bootstrapped 95% CI: £72-£467) per additional 10μg/m3 reduction in PM2.5,​ or £71 (bootstrapped 95% CI: -£57-£309) per additional quitter.CONCLUSIONSThis trial targeted a socio-economically disadvantaged population that has been neglected within the literature. The complex intervention was more costly but more effective in reducing PM2.5 compared with the usual care. It offers huge potential to reduce children's' tobacco-related harm by reducing exposure to SHS in the home. The intervention is considered cost-effective if the decision maker is willing to pay £131 per additional 10μg/m3 of PM2.5 reduction.TRIAL REGISTRATIONThe Smoke Free Homes trial was registered with isrctn.com on 29 January 2013 with the identifier ISRCTN81701383 . |
| 296 | Zhang,​ K.M.,​ Clyde,​ M.,​ Pipe,​ A.,​ Reid,​ R.,​ Els,​ C.,​ Tulloch,​ H.E.. Do women and men differ in baseline smoking characteristics and quit rates folllowing treatment with smoking cessation medications? A secondary analysis of the flex study. Journal of Cardiopulmonary Rehabilitation and Prevention. 2018;38:E21.  **Abstract:** Background: Tobacco accounts for 33% of all deaths from cardiovascular disease. Women are more susceptible to health consequences of smoking and are less likely to be successful at smoking cessation than men. Little is known about how men and women differ in smoking characteristics and abstinence using standard nicotine replacement therapy (NRT),​ combined formulations of NRT or varenicline (VR). Objective: This study examined sex differences in withdrawal symptoms and smoking abstinence at 10,​ 22,​ and 52 weeks post target quit date. Sex differences in baseline smoking characteristics (cigarettes smoked per day; cumulative years smoked; number of previous quit attempts; nicotine dependence; motivation to quit; and confidence to quit) were also investigated. Methods: Smokers (N = 737; female = 46.6%) were randomly assigned to either 1) NRT (10 weeks of 21 mg daily maximum patches); 2) NRT+ (35 mg daily maximum patches and gum or inhaler for up to 22 weeks); or 3) VR (1 mg VR twice daily for up to 24 weeks) group. All participants received six 15-minute smoking cessation counseling sessions. Smoking abstinence was measured by carbon monoxide confirmed continuous abstinence rates (CAR) from weeks 5-10; 5-22,​ and 5-52. Demographic information,​ smoking history,​ withdrawal symptoms,​ and nicotine dependence were assessed using validated questionnaires. Results: Women reported higher number of years smoked (F (1,​ 722) = 4.48,​ p = .035),​ and lower number of cigarettes/day (F (1,​ 723) = 5.82,​ p = .016) at baseline than men. Women also endorsed more withdrawal symptoms at 10 weeks (F = 1,​ 443) = 4.86,​ p = .028),​ but no sex differences were found at 22 and 52 weeks. No sex differences were found in confidence,​ motivation,​ number of quit attempts and nicotine dependence (ps >.31). The odds of women achieving cessation rates were not statistically different from men at 10-weeks (odds ratio (OR) = 1.11,​ 95% confidence interval (CI) = .64 to 1.92),​ 22 weeks (OR = 1.15,​ CI = .56 to 2.37) and 52 weeks (OR = 1.18,​ CI = .50 to 2.79). No statistically significant interactions between sex and treatment group were found,​ suggesting that both sexes were equally likely to quit smoking in the NRT+ and VR groups as compared to the NRT group across time points. Conclusion: Women experienced more short-term withdrawal symptoms than men. No sex differences were found in efficacy of different treatment groups,​ suggesting that smoking cessation medications may help attenuate sex disparities in smoking quit rates. |
| 300 | Oertel,​ W.,​ Müller,​ H.,​ Schade-Brittinger,​ C.,​ Kamp,​ C.,​ Balthasar,​ K.,​ Articus,​ K.,​ Brinkman,​ M.,​ Venuto,​ C.,​ Unger,​ M.,​ Eggert,​ K.,​ Vadasz,​ D.,​ Kieburtz,​ K.,​ Boyd,​ J.. The NIC-PD-study-A randomized,​ placebo-controlled,​ double-blind,​ multi-centre trial to assess the disease-modifying potential of transdermal nicotine in early Parkinson's disease in Germany and N. America. Movement Disorders. 2018;33:S159.  **Abstract:** Objective: To evaluate the disease modifying potential of transdermal (TD) nicotine in early Parkinson's disease (PD). For this explanatory purpose,​ the primary endpoint was the change in the total UPDRS (I-III) score between baseline and 60 weeks (52 weeks treatment,​ 8 weeks washout). Background: Epidemiological studies have shown an inverse relation between PD and tobacco consumption. in vitro investigations suggest nicotine exerts multiple effects that may slow neurodegeneration in PD. The German and N. American Parkinson Study Groups sought to investigate the disease-modifying effect of TD nicotine on the progression of early PD. Methods: PD subjects within 18 months of diagnosis,​ Hoehn & Yahr stage ≤2,​ not requiring dopaminergic therapy,​ with or without stable (≥8 weeks) MAOBI therapy were randomized 1:1 to TD nicotine (up to 28 mg/day) or placebo. Differences in total UPDRS change from baseline to 60 weeks and 52 weeks (secondary pragmatic purpose) between groups were estimated using the Hodges-Lehmann (HL) method and tested using the exact 2-sided stratified Mann-Whitney-Wilcoxon test in the intent-to-treat-population. Results: 163 subjects were randomized (61 at 11 US centers; 102 at 13 Germany centers),​ 1 excluded before treatment started. Baseline traits were similar in both arms [Table 1]. N=96 completed per protocol with 101 analyzed for the primary endpoint: mean worsening of total UPDRS was 3.5 in placebo (N=54) vs 6.0 in the nicotine arm (N=47),​ (HL difference with 95% CI: 3 [0,​ 6],​ p=0.0560). Drop out was almost exclusive due to early stop of treatment or major deviations from required assessments. N=138 were analyzed at 52 weeks with worsening of 5.4 in placebo (N=74) vs 9.1 in the nicotine arm (N=64),​ (4 [1,​ 7],​ p=0.0100). Cutaneous adverse effects were most common [Table 2]. 29% (23 placebo,​ 24 nicotine) initiated symptomatic therapy. Conclusions: TD nicotine does not slow progression in early PD. In contrast to the hypothesis,​ nicotine may exert an accelerating effect as evidenced by worsened UPDRS scores compared to placebo. |
| 305 | Dahne,​ Jennifer,​ Wahlquist,​ Amy E,​ Boatright,​ Amy S,​ Garrett-Mayer,​ Elizabeth,​ Fleming,​ Douglas O,​ Davis,​ Robert,​ Egan,​ Brent,​ Carpenter,​ Matthew J. Nicotine replacement therapy sampling via primary care: Methods from a pragmatic cluster randomized clinical trial. Contemporary clinical trials. 2018;72:1.  **Abstract:** BACKGROUNDPrimary care is the most important point of healthcare contact for smokers. Brief physician advice to quit,​ based on the 5As/AAR model,​ offers some efficacy but is inconsistently administered and has limited population impact. Nicotine replacement therapy (NRT) sampling,​ defined as provision of a brief NRT starter kit,​ when added to the 5As/AAR,​ is well-suited to primary care because it is simple,​ brief,​ and can be provided to all smokers. This article describes the design and methods of an ongoing comparative effectiveness trial testing standard care vs. standard care + NRT sampling within primary care.METHODSSmokers were recruited directly from primary care practices between July 2014 and December 2017 within an established network of South Carolina clinics. Interventions were delivered randomly by clinic personnel,​ and phone-based follow-ups were centrally coordinated by research staff to track outcomes through six months post-intervention. Primary study aims are to examine the impact of NRT sampling on smoking,​ inclusive of cessation,​ quit attempts,​ and uptake of evidence-based treatment.RESULTSTwenty-two clinics were recruited. Across clinics,​ patient census ranged from 985 to 10,​957 and number of providers ranged from 1 to 63. Average patient age across clinics was 52.9 years and smoking prevalence across ranged from 10.6% to 28.5%.CONCLUSIONImproving the effectiveness and reach of brief interventions within primary care could have a considerable impact on population quit rates. We consider the advantages and disadvantages of key methodological decisions relevant to the design of future primary care-based cessation trials. |
| 307 | Clyde,​ Matthew,​ Pipe,​ Andrew,​ Els,​ Charl,​ Reid,​ Robert,​ Fu,​ Angel,​ Clark,​ Alexa,​ Tulloch,​ Heather. Nicotine metabolite ratio and smoking outcomes using nicotine replacement therapy and varenicline among smokers with and without psychiatric illness. Journal of psychopharmacology (Oxford,​ England). 2018;32:979.  **Abstract:** INTRODUCTIONIt has been suggested that the effectiveness of nicotine replacement smoking cessation pharmacotherapy may be enhanced by assessing rates of nicotine metabolism using the nicotine metabolite ratio - which reflects differences in the activity of the CYP2A6 hepatic enzyme - and titrating doses appropriately. To date,​ supporting evidence is equivocal,​ with little information regarding the assessment and effectiveness of the nicotine metabolite ratio among smokers with psychiatric conditions.METHODSThe nicotine metabolite ratio of 499 smokers from the FLEX trial was determined using urine samples obtained at baseline. They were randomized to receive either: standard transdermal nicotine (nicotine replacement therapy); extended nicotine replacement therapy + adjunct nicotine agent; or varenicline. Primary cessation outcomes were seven-day point prevalence at 5,​ 10,​ 22,​ and 52 weeks post-target quit date,​ comparing across treatment and psychiatric status. Our principal analysis employed logistic regression (outcome: abstinence),​ using slow metabolizers as the reference category.RESULTSNo differences were observed by nicotine metabolite ratio classification (slow,​ moderate,​ fast) with respect to any demographic or smoking-related variables. Nicotine metabolite ratio class did not predict smoking cessation in either the overall sample,​ or by treatment condition at any time-point (week 52 moderate metabolizers: odds ratio 1.34,​ 95% confidence interval (0.68-2.63),​ p=0.394; fast metabolizers: odds ratio 1.04 (0.56-1.91),​ p=0. 906).CONCLUSIONOur results did not find any associations between nicotine metabolite ratio and cessation outcomes among smokers using nicotine replacement therapy or varenicline with and without lifetime psychiatric conditions. |
| 308 | Liberman,​ Keliane,​ Van Schuerbeek,​ Peter,​ Herremans,​ Sarah,​ Meysman,​ Marc,​ De Mey,​ Johan,​ Buls,​ Nico. The effect of nicotine patches on craving in the brain: A functional MRI study on heavy smokers. Medicine. 2018;97:e12415.  **Abstract:** BACKGROUNDSmoking is a common phenomenon and kills over 6 million people every year. Many smokers try to quit smoking by using nicotine replacement therapy (NRT). Most of the time,​ relapse occurs in less than six months after finishing the program of NRT. We performed a single blinded study in which our aim was to figure out what the effect of the nicotine patch is on craving in the brain of smokers deprived from smoking.METHODSFive heavy smokers (Fagerström Test for Nicotine Dependence ≥4) underwent a functional magnetic resonance imaging (fMRI) in 4 random conditions: smoking (S); smoking deprivation (SD); SD combined with a NP (SD + NP); SD combined with a placebo patch (SD + PP). Visual stimulation provoked craving in block design by randomly displaying images of smoking related scenes. After image preprocessing,​ a fixed-effect analysis was performed to compare average group activations. The Questionnaire for Smoking Urges (QSU) was obtained before and after each scan.RESULTSThe fMRI results showed higher activation in areas involved in craving in S compared with SD + NP,​ SD + PP,​ and SD. In the SD + NP,​ limbic circuit and attention area were higher activated compared with SD and SD + PP. The SD + PP and SD showed higher activation in the frontal cortex and limbic system compared with S and SD + NP. Nonsmokers showed higher limbic activation compared with SD.The QSU increased significantly after the fMRI experiment in S (P = .036).The SD had higher QSU scores compared with the S before (P = .002),​ and also after (P = .022) the fMRI experiment. The NP showed lower scores than the SD before the experiment (P = .046).CONCLUSIONThe fMRI experiment revealed lower activity in areas associated with attention when subjects were nicotine deprived (SD + PP and SD). Areas involved with craving showed less activity when nicotine is present (S and SD + NP). The QSU showed a significant difference between SD and when nicotine is present (S and SD + NP). |
| 309 | Krishnan,​ Nandita,​ Gittelsohn,​ Joel,​ Ross,​ Alexandra,​ Elf,​ Jessica,​ Chon,​ Sandy,​ Niaura,​ Raymond,​ Martinson,​ Neil,​ Golub,​ Jonathan E. Qualitative Exploration of a Smoking Cessation Trial for People Living With HIV in South Africa. Nicotine & tobacco research : official journal of the Society for Research on Nicotine and Tobacco. 2018;20:1117.  **Abstract:** IntroductionIn South Africa,​ people living with HIV have a high prevalence of smoking,​ which undermines the beneficial effects of antiretroviral therapy. However,​ little is known about barriers to smoking cessation and what interventions work for people living with HIV in this setting.MethodsA randomized trial comparing intensive anti-smoking counseling versus counseling and nicotine replacement therapy was recently concluded in Klerksdorp,​ South Africa. In a post-trial follow-up,​ 23 in-depth interviews with patients and one focus group discussion with counselors from the trial were conducted. A codebook was developed and codes were applied to the transcripts,​ which were analyzed using a thematic analysis.ResultsBarriers at the economic,​ social/interpersonal,​ and individual levels induced stress,​ which hindered smoking cessation. Economic stressors included unemployment and poverty. Social or interpersonal stressors were lack of social support for quitting smoking and lack of social support due to having HIV. Individual stressors were traumatic life events. Alcohol was used to cope with stress and frequently co-occurred with smoking. Managing cravings was a barrier unrelated to stress. Participants proposed income and employment opportunities,​ group counseling,​ and more frequent counseling as solutions to address stressors at different levels. Nicotine replacement therapy was helpful to mitigate cravings.ConclusionsFuture smoking cessation interventions need to target barriers at multiple levels. Increasing the supply and duration of nicotine replacement therapy may increase its effectiveness. Other behavioral approaches such as group counseling or peer counseling could hold promise in this setting but need to be tested for efficacy through randomized controlled trials.ImplicationsTo our knowledge,​ this is the first qualitative study examining barriers to smoking cessation for people living with HIV in South Africa. Smoking is highly prevalent among people with HIV in South Africa and cessation interventions are urgently needed. A better understanding of barriers to smoking cessation that people with HIV face will lead to the development of contextually appropriate interventions. This study also provides feedback on interventions from a recently concluded smoking cessation randomized trial and will help guide the design of future smoking cessation trials. |
| 310 | Cooperman,​ Nina A,​ Lu,​ Shou-En,​ Richter,​ Kimber P,​ Bernstein,​ Steven L,​ Williams,​ Jill M. Pilot Study of a Tailored Smoking Cessation Intervention for Individuals in Treatment for Opioid Dependence. Nicotine & tobacco research : official journal of the Society for Research on Nicotine and Tobacco. 2018;20:1152.  **Abstract:** IntroductionOver 85% of opioid-dependent individuals in methadone treatment smoke cigarettes; however,​ smoking cessation interventions are minimally effective in this population. To better help opioid-dependent individuals quit smoking,​ we developed and pilot-tested an intervention,​ based in the Information-Motivation-Behavioral Skills (IMB) model of behavior change,​ which could be tailored to address individual barriers to smoking cessation in this population.MethodsWe randomized participants (n = 83) in methadone treatment to the eight-session,​ IMB model-based,​ intervention plus nicotine replacement therapy (intervention,​ n = 41) or a facilitated referral to the state Quitline (control,​ n = 42). All participants completed assessments at baseline,​ 3 months,​ and 6 months.ResultsIntervention participants completed a median of five sessions (interquartile range [IQR] 3-8) and had significantly higher intervention satisfaction than control participants. Intervention participants reported smoking significantly fewer cigarettes per day at 3 months (median [IQR] = 6 [4-15]) and 6 months (median [IQR] = 8 [4-14]) as compared control participants at 3 months (median [IQR] = 10 [5-20]) and 6 months (median [IQR] = 10 [6-20]). Fifty-six percent of the intervention group and 41% of the control group a made a quit attempt during the study (p = .16). At 3 months,​ 7% (n = 3) of intervention participants and none of the control participants were abstinent from smoking (p = .23). At 6 months,​ 2% of participants in both groups were abstinent. Twenty-four percent and 10% of the intervention and control group participants,​ respectively,​ reported 20 or more smoke-free days (p = .43).ConclusionsAn IMB model-based smoking cessation intervention for opioid-dependent smokers is feasible and acceptable in methadone treatment and may help methadone maintained smokers cut down on their smoking.ImplicationsThis is the first study of a tailored,​ IMB Model-based,​ smoking cessation intervention for opioid dependent smokers. Results showed that opioid dependent smokers are willing and able to participate in an IMB model-based smoking cessation intervention,​ and this intervention may help this population cut down on their smoking. Also,​ the Quitline seems less feasible and acceptable for this population than a face-to-face intervention. Further research is needed to determine how to integrate smoking cessation treatment into methadone programs and how to improve interventions so that treatment gains can lead to long-term abstinence in this population. |
| 312 | West,​ Robert,​ Evins,​ A Eden,​ Benowitz,​ Neal L,​ Russ,​ Cristina,​ McRae,​ Thomas,​ Lawrence,​ David,​ St Aubin,​ Lisa,​ Krishen,​ Alok,​ Maravic,​ Melissa C,​ Anthenelli,​ Robert M. Factors associated with the efficacy of smoking cessation treatments and predictors of smoking abstinence in EAGLES. Addiction (Abingdon,​ England). 2018;113:1507.  **Abstract:** AIMSTo assess (1) how far the efficacies of front-line smoking cessation pharmacotherapies vary as a function of smoker characteristics and (2) associations between these characteristics and success of smoking cessation attempts.DESIGNProspective correlational study in the context of a double-blind randomized trial. The outcome was regressed individually onto each covariate after adjusting for treatment,​ and then a forward stepwise model constructed. Treatment moderator effects of covariates were tested by treatment × covariate interactions.SETTINGHealth service facilities in multiple countries.PARTICIPANTSData came from 8120 smokers willing to make a quit attempt,​ randomized to varenicline,​ bupropion,​ nicotine replacement therapy (NRT) or placebo in Evaluating Adverse Events in a Global Smoking Cessation Study (EAGLES) between 30 November 2011 and 13 January 2015.MEASUREMENTSSmoker characteristics measured at baseline were country,​ psychiatric history,​ sex,​ age,​ body mass index (BMI),​ ethnic group,​ life-time suicidal ideation/behaviour,​ anxiety,​ depression,​ aggression,​ psychotropic medication,​ history of alcohol/substance use disorder,​ age of starting smoking,​ cigarette dependence [Fagerström Test for Cigarette Dependence (FTCD)] and prior use of study medicines. Outcome was biochemically confirmed continuous abstinence at weeks 9-24 from start of treatment.FINDINGSNo statistically significant treatment × covariate interactions were found. Odds of success were associated independently positively with age [odds ratio (OR) = 1.01; 95% confidence interval (CI) = 1.00,​ 1.01],​ BMI (1.01; 95% CI = 1.00,​ 1.02) and age of starting smoking (1.03; 95% CI = 1.02,​ 1.04). Odds were associated independently negatively with US (versus non-US) study site (0.53; 95% CI = 0.46,​ 0.61),​ black (versus white) ethnic group (0.57; 95% CI = 0.45,​ 0.72),​ mood disorder (0.85; 95% CI = 0.73,​ 0.99),​ anxiety disorder (0.71; 95% CI = 0.55,​ 0.90) and psychotic disorder (0.73; 95% CI = 0.50,​ 1.07),​ taking psychotropic medication (0.81; 95% CI = 0.68,​ 0.95),​ FTCD (0.89; 95% CI = 0.87,​ 0.92) and previous use of NRT (0.78; 95% CI = 0.67,​ 0.91).CONCLUSIONSWhile a range of smoker characteristics-including psychiatric history,​ cigarette dependence and prior use of nicotine replacement therapy (NRT)-are associated with lower cessation rates,​ they do not substantially influence the efficacy of varenicline,​ bupropion or NRT. |
| 316 | Aveyard,​ Paul,​ Lindson,​ Nicola,​ Tearne,​ Sarah,​ Adams,​ Rachel,​ Ahmed,​ Khaled,​ Alekna,​ Rhona,​ Banting,​ Miriam,​ Healy,​ Mike,​ Khan,​ Shahnaz,​ Rai,​ Gurmail,​ Wood,​ Carmen,​ Anderson,​ Emma C,​ Ataya-Williams,​ Alia,​ Attwood,​ Angela,​ Easey,​ Kayleigh,​ Fluharty,​ Megan,​ Freuler,​ Therese,​ Hurse,​ Megan,​ Khouja,​ Jasmine,​ Lacey,​ Lindsey,​ Munafò,​ Marcus,​ Lycett,​ Deborah,​ McEwen,​ Andy,​ Coleman,​ Tim,​ Dickinson,​ Anne,​ Lewis,​ Sarah,​ Orton,​ Sophie,​ Perdue,​ Johanna,​ Randall,​ Clare,​ Anderson,​ Rebecca,​ Bisal,​ Natalie,​ Hajek,​ Peter,​ Homsey,​ Celine,​ McRobbie,​ Hayden J,​ Myers-Smith,​ Katherine,​ Phillips,​ Anna,​ Przulj,​ Dunja,​ Li,​ Jinshuo,​ Coyle,​ Doug,​ Coyle,​ Katherine,​ Pokhrel,​ Subhash. Nicotine preloading for smoking cessation: the Preloading RCT. Health technology assessment (Winchester,​ England). 2018;22:1.  **Abstract:** BACKGROUNDNicotine preloading means using nicotine replacement therapy prior to a quit date while smoking normally. The aim is to reduce the drive to smoke,​ thereby reducing cravings for smoking after quit day,​ which are the main cause of early relapse. A prior systematic review showed inconclusive and heterogeneous evidence that preloading was effective and little evidence of the mechanism of action,​ with no cost-effectiveness data.OBJECTIVESTo assess (1) the effectiveness,​ safety and tolerability of nicotine preloading in a routine NHS setting relative to usual care,​ (2) the mechanisms of the action of preloading and (3) the cost-effectiveness of preloading.DESIGNOpen-label randomised controlled trial with examination of mediation and a cost-effectiveness analysis.SETTINGNHS smoking cessation clinics.PARTICIPANTSPeople seeking help to stop smoking.INTERVENTIONSNicotine preloading comprised wearing a 21 mg/24 hour nicotine patch for 4 weeks prior to quit date. In addition,​ minimal behavioural support was provided to explain the intervention rationale and to support adherence. In the comparator group,​ participants received equivalent behavioural support. Randomisation was stratified by centre and concealed from investigators.MAIN OUTCOME MEASURESThe primary outcome was 6-month prolonged abstinence assessed using the Russell Standard. The secondary outcomes were 4-week and 12-month abstinence. Adverse events (AEs) were assessed from baseline to 1 week after quit day. In a planned analysis,​ we adjusted for the use of varenicline (Champix®; Pfizer Inc.,​ New York,​ NY,​ USA) as post-cessation medication. Cost-effectiveness analysis took a health-service perspective. The within-trial analysis assessed health-service costs during the 13 months of trial enrolment relative to the previous 6 months comparing trial arms. The base case was based on multiple imputation for missing cost data. We modelled long-term health outcomes of smoking-related diseases using the European-study on Quantifying Utility of Investment in Protection from Tobacco (EQUIPT) model.RESULTSIn total,​ 1792 people were eligible and were enrolled in the study,​ with 893 randomised to the control group and 899 randomised to the intervention group. In the intervention group,​ 49 (5.5%) people discontinued preloading prematurely and most others used it daily. The primary outcome,​ biochemically validated 6-month abstinence,​ was achieved by 157 (17.5%) people in the intervention group and 129 (14.4%) people in the control group,​ a difference of 3.02 percentage points [95% confidence interval (CI) -0.37 to 6.41 percentage points; odds ratio (OR) 1.25,​ 95% CI 0.97 to 1.62; p = 0.081]. Adjusted for use of post-quit day varenicline,​ the OR was 1.34 (95% CI 1.03 to 1.73; p = 0.028). Secondary abstinence outcomes were similar. The OR for the occurrence of serious AEs was 1.12 (95% CI 0.42 to 3.03). Moderate-severity nausea occurred in an additional 4% of the preloading group compared with the control group. There was evidence that reduced urges to smoke and reduced smoke inhalation mediated the effect of preloading on abstinence. The incremental cost-effectiveness ratio at the 6-month follow-up for preloading relative to control was £710 (95% CI -£13,​674 to £23,​205),​ but preloading was dominant at 12 months and in the long term,​ with an 80% probability that it is cost saving.LIMITATIONSThe open-label design could partially account for the mediation results. Outcome assessment could not be blinded but was biochemically verified.CONCLUSIONSUse of nicotine-patch preloading for 4 weeks prior to attempting to stop smoking can increase the proportion of people who stop successfully,​ but its benefit is undermined because it reduces the use of varenicline after preloading. If this latter effect could be overcome,​ then nicotine preloading appears to improve health and reduce health-service costs in the long term. Future work should determine how to ensure that people using nicotine preloading opt to use varenicline as cessation medication.TRIAL REGISTRATIONCurrent Controlled Trials ISRCTN33031001.FUNDINGThis project was funded by the NIHR Health Technology Assessment programme and will be published in full in Health Technology Assessment; Vol. 22,​ No. 41. See the NIHR Journals Library website for further project information. |
| 317 | Schuster,​ Randi Melissa,​ Pachas,​ Gladys N,​ Stoeckel,​ Luke,​ Cather,​ Corinne,​ Nadal,​ Mireya,​ Mischoulon,​ David,​ Schoenfeld,​ David A,​ Zhang,​ Haiyue,​ Ulysse,​ Christine,​ Dodds,​ Elisabeth B,​ Sobolewski,​ Sara,​ Hudziak,​ Vicenta,​ Hanly,​ Ailish,​ Fava,​ Maurizio,​ Evins,​ A Eden. Phase IIb Trial of an α7 Nicotinic Receptor Partial Agonist With and Without Nicotine Patch for Withdrawal-Associated Cognitive Deficits and Tobacco Abstinence. Journal of clinical psychopharmacology. 2018;38:307.  **Abstract:** PURPOSE/BACKGROUNDThe objective of this study was to determine whether a novel α7 nicotinic acetylcholine receptor partial agonist improves cognition during nicotine withdrawal and improves abstinence rates. To do so,​ the effect of the α7 nicotinic acetylcholine receptor partial agonist,​ encenicline,​ on cognition and abstinence was evaluated when given as monotherapy and when combined with transdermal nicotine patch (nicotine replacement therapy [NRT]).METHODSAdult daily smokers,​ n = 160,​ who were motivated to quit smoking completed cognitive testing at satiated baseline and after overnight abstinence and then were randomized to receive a 12-week trial of encenicline 1 mg twice daily or identical placebo the day of the overnight abstinent cognitive testing. In the first 6 weeks of the 12-week encenicline administration,​ participants were also randomized to 6 weeks of NRT patch or placebo patch. Primary outcomes were cognition during abstinence and 7-day point-prevalence abstinence at week 12.RESULTSNo beneficial effects of encenicline were observed on cognition or abstinence when compared with placebo or when combined with NRT compared with placebo capsule + NRT. Of the 4 conditions,​ abstinence rates were lowest among those assigned to encenicline alone.CONCLUSIONSBeneficial effects of NRT were observed on cognitive and abstinence outcomes when combined with encenicline compared with encenicline plus placebo patch. Addition of NRT to encenicline improved odds of abstinence approximately 3-fold compared with encenicline plus placebo patch. We conclude that encenicline,​ 1 mg/d,​ did not improve abstinence-associated cognitive impairment or abstinence rates as monotherapy or adjunctive therapy to NRT patch. |
| 318 | Dedert,​ Eric A,​ Dennis,​ Paul A,​ Calhoun,​ Patrick S,​ Dennis,​ Michelle F,​ Beckham,​ Jean C. A Randomized Clinical Trial of Nicotine Preloading for Smoking Cessation in People with Posttraumatic Stress Disorder. Journal of dual diagnosis. 2018;14:148.  **Abstract:** OBJECTIVEThe aim of this research was to determine whether augmenting standard smoking cessation treatment by wearing an active nicotine patch before the smoking quit date improves rates of smoking cessation in individuals with posttraumatic stress disorder (PTSD) and to explore mechanisms of treatment response such as decreased cigarette craving and symptom relief from smoking.METHODSThis was a double-blind parallel randomized controlled trial in 81 people with PTSD who smoked cigarettes. Participants were recruited from Veterans Affairs outpatient clinics and flyers in the community. Participants provided ecological momentary assessments (EMAs) of PTSD symptoms,​ smoking withdrawal symptoms,​ and cravings before and after smoking a cigarette during one week of ad lib smoking and then three weeks of either a nicotine patch (n = 37) or placebo patch (n = 44) preceding the quit date. All participants received standard pharmacotherapy and behavioral treatment for smoking cessation after the quit date. To test the efficacy of nicotine patch preloading for engaging proposed treatment targets during the pre-quit phases,​ we used multilevel models to compare post-smoking changes in symptoms and cravings during the preloading phases to post-smoking changes reported during the ad lib smoking phase.RESULTSThere was no significant difference in quit rates across the two conditions on the primary outcome of seven-day point prevalence smoking abstinence bioverified with breath carbon monoxide at six weeks post-quit date. In a multivariable multilevel model pre- to post-cigarette changes in PTSD symptom clusters,​ smoking withdrawal symptoms,​ and cravings,​ there was a significant interaction between treatment phase and condition. Relative to participants in the placebo condition,​ participants in the nicotine patch condition experienced diminished relief from PTSD reexperiencing symptoms,​ smoking withdrawal symptoms,​ and cigarette craving after smoking a cigarette.CONCLUSIONSRelative to placebo patch preloading,​ nicotine patch preloading diminished the reinforcing effects of smoking cigarettes. However,​ the low quit rates in both conditions suggest that nicotine patch preloading is not a sufficiently intensive treatment for achieving smoking cessation in people with PTSD.TRIAL REGISTRATIONclinicaltrials.gov: NCT00625131. |
| 319 | Zarghami,​ Mehran,​ Taghizadeh,​ Fatemeh,​ Sharifpour,​ Ali,​ Alipour,​ Abbas. Efficacy of Smoking Cessation on Stress,​ Anxiety,​ and Depression in Smokers with Chronic Obstructive Pulmonary Disease: A Randomized Controlled Clinical Trial. Addiction & health. 2018;10:137.  **Abstract:** BackgroundStress,​ anxiety,​ and depression have been reported as very common comorbidities in smokers with chronic obstructive pulmonary disease (COPD). This study was aimed to investigate the effectiveness of smoking cessation on stress,​ anxiety,​ and depression in smokers with COPD.MethodsThree block-randomized controlled trial groups with a block size of 6 and 9 including guided self-change (GSC) (n = 19),​ nicotine replacement therapy (NRT) (n = 19),​ and combined GSC-NRT (n = 19) with a follow-up of 29 weeks were considered in this research. Participants included elderly adult smokers with COPD. The patients carried out 5 weekly GSC counseling sessions and NRT for smoking cessation. Transtheoretical Model (TTM) questionnaire,​ Fagerstrom Test for Nicotine Dependence (FTND),​ Depression Anxiety Stress Scale (DASS),​ the Beck Depression Inventory-II (BDI-II),​ and Hospital Anxiety and Depression Scale (HADS) as well as the exhaled carbon monoxide (CO) were evaluated over the baseline and 12 and 29 weeks following treatments.FindingsCOPD participants with mean of 23 daily cigarette smoking completed the current study. The odds ratio (OR) of smoking cessation in GSC and GSC-NRT groups decreased more than NRT group. In addition,​ DASS,​ FTND,​ and the exhaled CO in GSC and GSC-NRT groups showed a better performance compared with the NRT group.ConclusionThe results showed that GSC and combined GSC-NRT therapy were significantly more effective than NRT alone. Also,​ the findings showed that GSC,​ NRT,​ and combined GSC-NRT were effective on stress,​ depression,​ and anxiety decreasing in smoking cessation. It seems that reducing smoking is associated with recovery in stress,​ anxiety,​ and depression in smokers with COPD. |
| 323 | Martins Filho,​ Euclides Dias,​ Vasconcelos,​ César Freire DE Melo,​ Oliveira,​ Fernando DE Santa Cruz,​ Pereira,​ Adriano DA Fonseca,​ Ferraz,​ Álvaro Antônio Bandeira. Evaluation of nicotine patch in pain control of patients undergoing laparoscopic cholecystectomy. Revista do Colegio Brasileiro de Cirurgioes. 2018;45:e1756.  **Abstract:** OBJECTIVEto analyze the effects of nicotine patch on pain control,​ occurrence of nausea and its hemodynamic repercussions in laparoscopic cholecystectomy procedures.METHODSwe conducted an analytical,​ prospective,​ randomized,​ triple-blinded,​ clinical study between January and July 2017. The sample consisted of 17 patients who underwent laparoscopic cholecystectomy for the treatment of cholelithiasis. Nine patients used nicotine patch,​ and eight,​ placebo patch. The studied variables were pain,​ nausea,​ patient satisfaction,​ blood pressure,​ heart rate,​ oximetry and morphine rescue.RESULTStaking into account the pain and nausea parameters,​ there was no statistically significant difference between the groups (p>0.05). Also,​ the evaluation of rescue medication,​ both opioids and prokinetics,​ did not show any significant statistical difference between the groups. Among the hemodynamic parameters,​ there was only one statistically significant difference in the analysis of oxygen saturation and systolic blood pressure (SBP) six hours after surgery: the mean oxygen saturation was higher in the Test group (97.89 x 95.88) and the mean SBP was higher in the Control group (123.89 x 110.0).CONCLUSIONalthough pain levels were lower for nicotine within 24 hours,​ the action of nicotine and the need for rescue opioids in pain control were not statistically significant between the groups and at the time intervals studied. There was no clinical repercussion in the hemodynamic parameters. |
| 326 | . Effects on abstinence of nicotine patch treatment before quitting smoking: parallel,​ two arm,​ pragmatic randomised trial. BMJ (Clinical research ed.). 2018;361:k2164.  **Abstract:** OBJECTIVETo examine the effectiveness of a nicotine patch worn for four weeks before a quit attempt.DESIGNRandomised controlled open label trial.SETTINGPrimary care and smoking cessation clinics in England,​ 2012-15.PARTICIPANTS1792 adults who were daily smokers with tobacco dependence. 899 were allocated to the preloading arm and 893 to the control arm.INTERVENTIONSParticipants were randomised 1:1,​ using concealed randomly permuted blocks stratified by centre,​ to either standard smoking cessation pharmacotherapy and behavioural support or the same treatment supplemented by four weeks of 21 mg nicotine patch use before quitting: "preloading."MAIN OUTCOME MEASURESThe primary outcome was biochemically confirmed prolonged abstinence at six months. Secondary outcomes were prolonged abstinence at four weeks and 12 months.RESULTSBiochemically validated abstinence at six months was achieved by 157/899 (17.5%) participants in the preloading arm and 129/893 (14.4%) in the control arm: difference 3.0% (95% confidence interval -0.4% to 6.4%),​ odds ratio 1.25 (95% confidence interval 0.97 to 1.62),​ P=0.08 in the primary analysis. There was an imbalance between arms in the frequency of varenicline use as post-cessation treatment,​ and planned adjustment for this gave an odds ratio for the effect of preloading of 1.34 (95% confidence interval 1.03 to 1.73),​ P=0.03: difference 3.8% (0.4% to 7.2%). At four weeks,​ the difference in prolonged abstinence unadjusted for varenicline use was odds ratio 1.21 (1.00 to 1.48),​ difference 4.3% (0.0% to 8.7%),​ P=0.05,​ and adjusted for varenicline use was 1.32 (1.08 to 1.62) P=0.007. At 12 months the odds ratio was 1.28 (0.97 to 1.69),​ difference 2.7% (-0.4% to 5.8%),​ P=0.09 unadjusted for varenicline use and after adjustment was 1.36 (1.02 to 1.80) P=0.04. 5.9% of participants discontinued preloading owing to intolerance. Gastrointestinal symptoms-chiefly nausea-occurred in 4.0% (2.2% to 5.9%) more people in the preloading arm than control arm. Eight serious adverse events occurred in the preloading arm and eight in the control arm (odds ratio 0.99,​ 0.36 to 2.75).CONCLUSIONSEvidence was insufficient to confidently show that nicotine preloading increases subsequent smoking abstinence. The beneficial effect seems to have been masked by a concurrent reduction in the use of varenicline in people using nicotine preloading,​ and future studies should explore ways to mitigate this unintended effect.TRIAL REGISTRATIONCurrent Controlled Trials ISRCTN33031001. |
| 327 | Sharma,​ Surendra Kumar,​ Mohan,​ Alladi,​ Singh,​ Achintya Dinesh,​ Mishra,​ Hridesh,​ Jhanjee,​ Sonali,​ Pandey,​ Ravindra Mohan,​ Singh,​ Binit Kumar,​ Sharma,​ Rohini,​ Pallipamu,​ Prakash Babu,​ Pai,​ Madhukar,​ Dheda,​ Keertan. Impact of nicotine replacement therapy as an adjunct to anti-tuberculosis treatment and behaviour change counselling in newly diagnosed pulmonary tuberculosis patients: an open-label,​ randomised controlled trial. Scientific reports. 2018;8:8828.  **Abstract:** We evaluated the impact of intensive smoking cessation activities as an adjunct to anti-tuberculosis treatment on patient-related treatment outcomes. In this open-label,​ randomised controlled trial,​ self-reporting smokers with pulmonary tuberculosis who initiated standard anti-tuberculosis treatment were randomised to either nicotine replacement therapy and behaviour change counselling (n = 400) or counselling alone (n = 400) provided at baseline and two follow-up visits. The primary outcomes were change in TBscore at 24-weeks and culture conversion at 8-weeks. Biochemical smoking quit rates defined as serum cotinine levels <10 ng/mL and/or exhaled carbon monoxide levels <6 ppm (47·8% vs 32·4%,​ p-value =< 0·001) and self-reported quit rates (69.3% vs 38·7%,​ p-value =< 0·001) were significantly higher in the intervention arm at 24-weeks. Though the TBscores at 24 weeks (95% CI) were lower in the intervention arm [2·07 (1·98,​ 2·17) versus 2.12 (2·02,​ 2·21)],​ the difference was not clinically meaningful. Patients in the control arm required treatment extension more often than intervention arm (6·4% vs 2·6%,​ p-value = 0·02). Combining nicotine replacement therapy with behaviour change counselling resulted in significantly higher quit rates and lower cotinine levels,​ however,​ impact on patient-related (TBscore) or microbiological outcomes (culture conversion) were not seen. |
| 328 | Rasmussen,​ Scott,​ Horkan,​ Kathleen Halabuk,​ Kotler,​ Mitchell. Pharmacokinetic Evaluation of Two Nicotine Patches in Smokers. Clinical pharmacology in drug development. 2018;7:506.  **Abstract:** Smoking continues to be a major preventable cause of early mortality worldwide,​ and nicotine replacement therapy has been demonstrated to increase rates of abstinence among smokers attempting to quit. Nicotine transdermal systems (also known as nicotine patches) attach to the skin via an adhesive layer composed of a mixture of different-molecular-weight polyisobutylenes (PIBs) in a specific ratio. This randomized,​ single-dose,​ 2-treatment,​ crossover pharmacokinetic (PK) trial assessed the bioequivalence of nicotine patches including a replacement PIB adhesive (test) compared with the PIB adhesive historically used on marketed patches (reference). The test and reference patches were bioequivalent,​ as determined by the PK parameters of Cmax and AUC0-t . In addition,​ the parameters Tmax and t1/2 did not significantly differ between the 2 patches,​ supporting the bioequivalence finding from the primary analysis. The tolerability profiles of the patches containing the replacement and previously used PIB adhesives were similar; application-site adverse events did not significantly differ between test and reference patches. Overall,​ these data establish the bioequivalence of the nicotine patch with the replacement PIB adhesive formulation and the previously utilized PIB adhesive formulation. |
| 332 | Christiansen,​ Bruce A,​ Carbin,​ Julianne,​ TerBeek,​ Erin,​ Fiore,​ Michael C. Helping Smokers with Severe Mental Illness Who Do Not Want to Quit. Substance use & misuse. 2018;53:949.  **Abstract:** BACKGROUNDPeople with a severe and persistent mental illness are far more likely to smoke than others. While a large portion would like to quit,​ they are less likely to make quit attempts and succeed.OBJECTIVEThis study used an Randomized Controlled Trial (RCT) to test an intervention designed to increase engagement in cessation treatment,​ quit attempts,​ and quitting in smokers who did not want to quit in the next 30 days. It also compared these smokers with those who were motivated to quit in the next 30 days.METHODSParticipants (N = 222),​ were smokers with significant mental illness receiving intensive outpatient care from Wisconsin Community Support Programs who were not interested in quitting in the next 30 days. They were randomly assigned to either an intervention group or an attention control group. The intervention,​ administered during four weekly sessions,​ included a motivational element,​ components designed to prepare the smoker for a quit attempt,​ and pre-quit nicotine patch. Additionally,​ 48 smokers motivated to quit in the next 30 days served as a comparison group.RESULTSCompared to control participants,​ smokers receiving the intervention were more likely to be abstinent at the three month follow-up (biochemically verified,​ intent to treat,​ 8.5% vs. 1.0%,​ respectively,​ p = .01). They were also more likely to accept four more quitting preparation sessions (intent to treat,​ 50.8% vs 29.2%,​ respectively,​ p < .001) but were not more likely to call a telephone tobacco quit line. Conclusion/Importance: Brief motivational interventions increased engagement in cessation treatment and abstinence among smokers with signification mental illness. |
| 338 | Benowitz,​ Neal L,​ Pipe,​ Andrew,​ West,​ Robert,​ Hays,​ J Taylor,​ Tonstad,​ Serena,​ McRae,​ Thomas,​ Lawrence,​ David,​ St Aubin,​ Lisa,​ Anthenelli,​ Robert M. Cardiovascular Safety of Varenicline,​ Bupropion,​ and Nicotine Patch in Smokers: A Randomized Clinical Trial. JAMA internal medicine. 2018;178:622.  **Abstract:** ImportanceQuitting smoking is enhanced by the use of pharmacotherapies,​ but concerns have been raised regarding the cardiovascular safety of such medications.ObjectiveTo compare the relative cardiovascular safety risk of smoking cessation treatments.Design,​ Setting,​ and ParticipantsA double-blind,​ randomized,​ triple-dummy,​ placebo- and active-controlled trial (Evaluating Adverse Events in a Global Smoking Cessation Study [EAGLES]) and its nontreatment extension trial was conducted at 140 multinational centers. Smokers,​ with or without established psychiatric diagnoses,​ who received at least 1 dose of study medication (n = 8058),​ as well as a subset of those who completed 12 weeks of treatment plus 12 weeks of follow up and agreed to be followed up for an additional 28 weeks (n = 4595),​ were included.InterventionsVarenicline,​ 1 mg twice daily; bupropion hydrochloride,​ 150 mg twice daily; and nicotine replacement therapy,​ 21-mg/d patch with tapering.Main Outcomes and MeasuresThe primary end point was the time to development of a major adverse cardiovascular event (MACE: cardiovascular death,​ nonfatal myocardial infarction,​ or nonfatal stroke) during treatment; secondary end points were the occurrence of MACE and other pertinent cardiovascular events (MACE+: MACE or new-onset or worsening peripheral vascular disease requiring intervention,​ coronary revascularization,​ or hospitalization for unstable angina).ResultsOf the 8058 participants,​ 3553 (44.1%) were male (mean [SD] age,​ 46.5 [12.3] years). The incidence of cardiovascular events during treatment and follow-up was low (<0.5% for MACE; <0.8% for MACE+) and did not differ significantly by treatment. No significant treatment differences were observed in time to cardiovascular events,​ blood pressure,​ or heart rate. There was no significant difference in time to onset of MACE for either varenicline or bupropion treatment vs placebo (varenicline: hazard ratio,​ 0.29; 95% CI,​ 0.05-1.68 and bupropion: hazard ratio,​ 0.50; 95% CI,​ 0.10-2.50).Conclusions and RelevanceNo evidence that the use of smoking cessation pharmacotherapies increased the risk of serious cardiovascular adverse events during or after treatment was observed. The findings of EAGLES and its extension trial provide further evidence that smoking cessation medications do not increase the risk of serious cardiovascular events in the general population of smokers.Trial Registrationclinicaltrials.gov Identifier: NCT01574703. |
| 339 | Park, Elyse R., Perez, Giselle Katiria, Regan, Susan, Muzikanksy, Alona, Rigotti, Nancy, Levy, Douglas E., Temel, Jennifer S., Cooley, Mary E., Partridge, Ann H., Pirl, William F., Irwin, Kelly, Friedman, Emily R., Borderud, Sarah, Hyland, Kelly, Rabin, Julia, Sprunck, Kim, Kwon, Diana, Ostroff, Jamie S.. Integrating tobacco treatment into cancer care: A first snapshot of RCT findings. Journal of Clinical Oncology. 2018; 36(S15):6505.  **Abstract:** Background: Despite ASCO recommendations that tobacco use be assessed and managed,​ most cancer patients who smoke do not receive tobacco treatment. Evidence-based tobacco treatment has not yet been integrated into routine oncology care,​ and the optimal tobacco treatment strategy in this context is unknown. Methods: We conducted a two-arm,​ two-site RCT to compare sustained counseling plus medication (Intervention Group; IG) to standard tobacco counseling (comparison group; CG) to assist newly diagnosed cancer patients to quit smoking. Both treatment groups received 4 weekly telephone-delivered motivational counseling sessions. The IG additionally received 4 biweekly plus 3 monthly counseling sessions (total 11) and 12-weeks of free FDAapproved cessation medication (nicotine replacement therapy (NRT; patch/lozenge),​ varenicline,​ or bupropion). Eligibility criteria included a recent cancer diagnosis (breast,​ GI/GU,​ gyn,​ head & neck,​ lymphoma,​ lung,​ melanoma),​ cigarette use in the past 30 days,​ and English/Spanish speaking. The primary outcome was 6- month biochemically verified abstinence. Results: 303 (70% of confirmed eligibles) patients were enrolled and randomized to a treatment group. Participants were 56% female; 82% white non- Hispanic and 10% black; mean age = 58.3 (sd = 9.7); 40% had a nonsmoking related tumor. 86% completed the 6-month surveys. 80% of IG patients used a smoking cessation medication,​ among which 83% selected NRT. Using intention-to-treat,​ 6-month quit rates were 33% in the IG group vs. 19% in the CG group (p < .02). Using intention-to-treat,​ 57% of IG patients were adherent to sustained counseling (≥7 sessions),​ which was associated with increased 6- month quit rates (p < .0001). Cost per patient was ,​273 (IG) vs. 8 (CG). Conclusions: Among newly-diagnosed cancer patients,​ a treatment program of sustained telephone-delivered counseling and free medication produced a higher 6-month quit rate vs. a briefer counseling program. The cost-per-quit compared favorably to other cessation interventions. Findings provide strong support for the benefit of sustained tobacco treatment and a model for effective implementation of tobacco treatment into oncology care settings nationwide. |
| 340 | Vickerman,​ Katrina A,​ Keller,​ Paula A,​ Deprey,​ Mona,​ Lachter,​ Randi B,​ Jenssen,​ Jacalyn,​ Dreher,​ Marietta. Never Quit Trying: Reengaging Tobacco Users in Statewide Cessation Services. Journal of public health management and practice : JPHMP. 2018;24:e25.  **Abstract:** CONTEXTTobacco dependence is well established as a chronic condition typically requiring numerous quit attempts. Tobacco users are unlikely to return to the same cessation program on their own.OBJECTIVEThis program evaluation examined the effectiveness of using multiple outreach methods to reengage tobacco users in a statewide cessation program at varying time points after their initial program enrollment.DESIGNParticipants were randomized to receive or not receive reengagement outreach. We conducted outreach via phone,​ e-mail,​ and/or text (based on methods participants agreed to receive) at 1,​ 2,​ or 3 months post-initial engagement. Participants were offered the opportunity to reenroll in QUITPLAN Services.SETTINGMinnesota's QUITPLAN Services PARTICIPANTS:: A total of 3020 tobacco users who enrolled in Minnesota's QUITPLAN Services and either received a 2-week starter kit of nicotine replacement therapy or completed 0-1 QUITPLAN Helpline calls.MAIN OUTCOME MEASURESWe explored group differences in the odds of reengagement (defined as enrolling in a phone cessation program or selecting 2 or more of nicotine replacement therapy starter kit,​ text messaging,​ e-mail program,​ or print materials),​ contributors to reengagement,​ and costs.RESULTS14.7% in the Reengagement Outreach (RO) group and 3.4% in the Comparison (no outreach) group reengaged. The majority (71%) reengaged during phone outreach. There were no significant differences in reengagement rates by follow-up time period (1,​ 2,​ or 3 months). Cost per reengagement was 6,​ mostly due to one-time setup costs; scaling to 10 000 tobacco users would cost approximately 1 per reengagement.CONCLUSIONSConducting proactive outreach through state-funded quitlines is an effective approach to reengaging tobacco users,​ yielding a 5-fold greater odds of reengagement compared with no outreach. Since most costs were for initial setup,​ the cost per reengagement would decrease as the outreach population size increases. Such outreach has the potential to foster treatment utilization and quit attempts. |
| 341 | Hall,​ Sharon M,​ Humfleet,​ Gary L,​ Gasper,​ James J,​ Delucchi,​ Kevin L,​ Hersh,​ David F,​ Guydish,​ Joseph R. Cigarette Smoking Cessation Intervention for Buprenorphine Treatment Patients. Nicotine & tobacco research : official journal of the Society for Research on Nicotine and Tobacco. 2018;20:628.  **Abstract:** IntroductionPatients receiving medication assisted therapy (MAT) for opioid use disorder have high cigarette smoking rates. Cigarette smoking interventions have had limited success. We evaluated an intervention to increase cigarette abstinence rates in patients receiving buprenorphine-assisted therapy.MethodsCigarette smokers (N = 175; 78% male; 69% Caucasian; 20% Hispanic),​ recruited from a buprenorphine clinic were randomly assigned to either an extended innovative system intervention (E-ISI) or to Standard Treatment Control (STC). The E-ISI combined motivational intervention with extended treatment (long-term nicotine replacement therapy ,​ varenicline,​ and extended cognitive behavioral therapy). STC received written information about quit-lines,​ medication,​ and resources. Assessments were held at baseline and 3,​ 6,​ 12,​ and 18 months. Seven-day biochemically verified point-prevalence cigarette abstinence was the primary outcome measure.ResultsFifty-four percent of E-ISI participants entered the extended treatment intervention; E-ISI and STC differed at 3 months on abstinence status but not at months 6,​ 12,​ and 18. E-ISI participants were more likely to attempt to quit,​ to have a goal of complete abstinence,​ and to be in a more advanced stage of change than STC participants. A higher number of cigarettes smoked and the use of cannabis in the previous 30 days predicted continued smoking.ConclusionsThe E-ISI was successful in increasing motivation to quit smoking but did not result in long-term abstinence. The failure of treatments that have been efficacious in the general population to produce abstinence in patients receiving MAT of opioid use disorder suggests that harm reduction and other innovative interventions should be explored.ImplicationsThis study demonstrates that an intervention combining motivational interviewing with an extended treatment protocol can increase cigarette quit attempts,​ enhance cigarette abstinence goals,​ and further movement through stages of change about quitting smoking in patients receiving MAT for opioid use disorder who smoke cigarettes. The intervention did not increase abstinence rates over those observed in a standard treatment control,​ however. The latter finding supports those of earlier investigators who also failed to find efficacy for smoking cessation in this population and who also used interventions effective in the general population. This pattern of findings suggests that patients with opioid use disorder can be motivated to change smoking behavior,​ but alternative and innovative approaches to cigarette smoking treatment should be studied. |
| 342 | Graham,​ Amanda L,​ Papandonatos,​ George D,​ Cha,​ Sarah,​ Erar,​ Bahar,​ Amato,​ Michael S. Improving Adherence to Smoking Cessation Treatment: Smoking Outcomes in a Web-based Randomized Trial. Annals of behavioral medicine : a publication of the Society of Behavioral Medicine. 2018;52:331.  **Abstract:** BackgroundPartial adherence in Internet smoking cessation interventions presents treatment and evaluation challenges. Increasing adherence may improve outcomes.PurposeTo present smoking outcomes from an Internet randomized trial of two strategies to encourage adherence to tobacco dependence treatment components: (i) a social network (SN) strategy to integrate smokers into an online community and (ii) free nicotine replacement therapy (NRT). In addition to intent-to-treat analyses,​ we used novel statistical methods to distinguish the impact of treatment assignment from treatment utilization.MethodsA total of 5,​290 current smokers on a cessation website (WEB) were randomized to WEB,​ WEB + SN,​ WEB + NRT,​ or WEB + SN + NRT. The main outcome was 30-day point prevalence abstinence at 3 and 9 months post-randomization. Adherence measures included self-reported medication use (meds),​ and website metrics of skills training (sk) and community use (comm). Inverse Probability of Retention Weighting and Inverse Probability of Treatment Weighting jointly addressed dropout and treatment selection. Propensity weights were used to calculate Average Treatment effects on the Treated.ResultsTreatment assignment analyses showed no effects on abstinence for either adherence strategy. Abstinence rates were 25.7%-32.2% among participants that used all three treatment components (sk+comm +meds).Treatment utilization analyses revealed that among such participants,​ sk+comm+meds yielded large percentage point increases in 3-month abstinence rates over sk alone across arms: WEB = 20.6 (95% CI = 10.8,​ 30.4),​ WEB + SN = 19.2 (95% CI = 11.1,​ 27.3),​ WEB + NRT = 13.1 (95% CI = 4.1,​ 22.0),​ and WEB + SN + NRT = 20.0 (95% CI = 12.2,​ 27.7).ConclusionsNovel propensity weighting approaches can serve as a model for establishing efficacy of Internet interventions and yield important insights about mechanisms.Clinical Trials.govNCT01544153. |
| 344 | Wang,​ Ying-Ying,​ Liu,​ Zhao,​ Wu,​ Yuan,​ Yang,​ Li,​ Guo,​ Lang-Tao,​ Zhang,​ Hao-Bin,​ Yang,​ Jin-Sheng. Efficacy of Acupuncture Is Noninferior to Nicotine Replacement Therapy for Tobacco Cessation: Results of a Prospective,​ Randomized,​ Active-Controlled Open-Label Trial. Chest. 2018;153:680.  **Abstract:** BACKGROUNDWe designed and conducted this multicenter randomized active-controlled open-label trial to evaluate the efficacy of acupuncture,​ auricular point pressing,​ and nicotine replacement therapy (NRT) on tobacco cessation in the Chinese population.METHODSThis randomized controlled trial was conducted in seven hospitals in China between October 2013 and February 2016. Eligible participants were recruited and randomly assigned to receive acupuncture or auricular point pressing or NRT via a central randomization system with a 1:1:1 ratio. All treatment was given for a total of 8 weeks,​ and follow-up visit was at 16 weeks. The primary outcome measure was carbon monoxide (CO)-confirmed 24-h point abstinence rate (<10 parts per million),​ 24 weeks after quit day.RESULTSA total of 300 participants were recruited and 195 participants finished,​ with a dropout rate of 35.00%. Two cases of adverse events in the acupuncture group and 2 cases in the NRT group were observed. The CO-confirmed 24-h point abstinence rate was 43.00% at 24 weeks in the acupuncture group,​ which was similar to 44.00% in the NRT group (P > .05),​ but significantly higher than the 30.00% in the auricular point group (P < .05). At 24 weeks,​ the Fagerstrom Nicotine Dependence Test and the Minnesota Nicotine Withdrawal Scale scores in the acupuncture group were significantly lower than those in the auricular point group and in the NRT group (P < .05). Kaplan-Meier analysis showed the time to relapse for acupuncture (44.12 days) was insignificantly longer than NRT (41.18 days),​ but significantly longer than auricular point pressing (29.53 days).CONCLUSIONSWe found acupuncture was safe and a possible treatment for tobacco cessation,​ but it requires further study to establish its role.TRIAL REGISTRYChinese Clinical Trial Registry; No.: ChiCTR-TRC-13003544; URL: http://www.chictr.org.cn/abouten.aspx. |
| 348 | Sukhija,​ Manpreet,​ Srivastava,​ Reena,​ Kaushik,​ Aditya. Pharmacokinetic characterization of three novel 4-mg nicotine lozenges . International journal of clinical pharmacology and therapeutics. 2018;56:113.  **Abstract:** OBJECTIVENicotine replacement therapy (NRT) increases the probability of smoking cessation. This study was conducted to determine if three prototype 4-mg nicotine lozenges produced locally in India were bioequivalent to a globally marketed reference product,​ Nicorette® 4-mg nicotine lozenge.MATERIALS AND METHODSHealthy adult smokers (N = 39) were treated with three prototype 4-mg nicotine lozenges in comparison with a reference 4-mg lozenge in this single-center,​ randomized,​ open-label,​ single-dose,​ 4-way crossover study. Pharmacokinetic sampling was obtained to test for bioequivalence using maximal plasma concentration (Cmax) and extent of absorption (AUC0-t). Secondarily,​ AUC;0-∞,​ time to maximal plasma concentration (tmax),​ half-life (T1/2),​ elimination rate constant (Kel),​ and safety of the prototype lozenges versus the reference lozenge were compared.RESULTSEach prototype 4-mg nicotine lozenge was found to be bioequivalent to the reference 4-mg nicotine lozenge based on the ratio of geometric means and 90% confidence intervals for Cmax,​ AUC0-t,​ and AUC;0-∞. Although tmax; was significantly longer for prototype III,​ all four lozenges achieved maximum plasma nicotine concentrations at a median of 1.5 hours. The safety profiles of the three prototype 4-mg lozenges did not differ from that of the 4-mg reference product.CONCLUSIONEach prototype 4-mg nicotine lozenge was bioequivalent to the reference 4-mg nicotine lozenge and was well tolerated. Furthermore,​ as these bioequivalent prototypes differed in in-vitro dissolution profiles,​ these data suggest that performance from the in -vitro method deployed is not a firm predictor of pharmacokinetic behavior. . |
| 354 | Ratschen,​ Elena,​ Thorley,​ Rebecca,​ Jones,​ Laura,​ Opazo Breton,​ Magdalena,​ Cook,​ Juliette,​ McNeill,​ Ann,​ Britton,​ John,​ Coleman,​ Tim,​ Lewis,​ Sarah. A randomised controlled trial of a complex intervention to reduce children's exposure to secondhand smoke in the home. Tobacco control. 2018;27:155.  **Abstract:** OBJECTIVESExposing children to secondhand tobacco smoke (SHS) causes significant harm and occurs predominantly through smoking by caregivers in the family home. We report a trial of a complex intervention designed to reduce secondhand smoke exposure of children whose primary caregiver feels unable or unwilling to quit smoking.DESIGNAn open-label,​ parallel,​ randomised controlled trial.SETTINGDeprived communities in Nottingham City and County,​ England PARTICIPANTS: Caregivers resident in Nottingham City and County in England who were at least 18 years old,​ the main caregiver of a child aged under 5 years living in their household,​ and reported that they were smoking tobacco inside their home.INTERVENTIONSWe compared a complex intervention combining personalised feedback on home air quality,​ behavioural support and nicotine replacement therapy for temporary abstinence with usual care.MAIN OUTCOMESThe primary outcome was change in air quality in the home,​ measured as average 16-24  hours levels of particulate matter of  < 2.5  µm diameter (PM2.5),​ between baseline and 12 weeks. Secondary outcomes included changes in maximum PM2.5,​ proportion of time PM2.5 exceeded WHO recommended levels of maximum exposure of 25  µg/mg3,​ child salivary cotinine,​ caregivers' cigarette consumption,​ nicotine dependence,​ determination to stop smoking,​ quit attempts and quitting altogether during the intervention.RESULTSArithmetic mean PM2.5 decreased significantly more (by 35.2 %; 95%  CI 12.7% to 51.9 %) in intervention than in usual care households,​ as did the proportion of time PM2.5 exceeded 25  µg/mg3,​ child salivary cotinine concentrations,​ caregivers' cigarette consumption in the home,​ nicotine dependence,​ determination to quit and likelihood of having made a quit attempt.CONCLUSIONSBy reducing exposure to SHS in the homes of children who live with smokers unable or unwilling to quit,​ this intervention offers huge potential to reduce children's' tobacco-related harm.TRIAL REGISTRATION NUMBERISRCTN81701383.This trial was funded by the UK National Institute for Health Research (NIHR): RP-PG-0608-10020. |
| 368 | Kalkhoran,​ Sara,​ Inman,​ Elizabeth,​ Kulesa Kelley,​ Jennifer H.,​ Ashburner,​ Jeffrey M.,​ Rigotti,​ Nancy A.. Proactive population health strategy to offer tobacco dependence treatment to smokersin aprima-ry care practice network. Journal of General Internal Medicine. 2018;33:312.  **Abstract:** Background: Health care systems typically provide tobacco cessation treatment at office visits. Offering treatment outside of office visits is a population-based strategy that could expand treatment reach and support clinicians' office-based efforts. Using electronic health record (EHR) data,​ a system can identify current smokers and proactively reach out to offer tobacco treatment. How best to engage and deliver tobacco treatment to smokers outside the office is not clear. Methods: A 3-arm pragmatic randomized controlled trial based in a primary care network explored the feasibility and reach of a proactive outreach effort and compared 2 strategies for delivering tobacco cessation treatment vs. usual care (UC). Current smokers with primary care providers (PCPs) at 5 community health centers in MAwere identified via the EHR and proactively recruited and consented using automated interactive voice response phone calls. Two intervention groups that offered proactive tobacco counseling and medications in different ways were compared to UC. Group 1: A health center-based Tobacco Care Coordinator provided brief counseling,​ coordinated medications with PCPs,​ and linked a smoker to additional care (in-person,​ phone call or text). Group 2: Smokers were transferred directly to a community-based Quitline for counseling and a free sample of nicotine replacement therapy. The primary outcome was the proportion of smokers with evidence-based cessation treatment documented in EHR or Quitline records in the 6 months after enrollment. We compared a pooled intervention group (Group 1 + Group 2) to UC. Secondary analyses compared treatment use in Group 1 vs. Group 2. Exploratory outcomes assessed self-reported past-30 day cigarette abstinence at 6 months. Chi-square tests were used for analyses. Results: Between 4/2016-2/2017,​ automated calls were made to 5,​225 smokers,​ of whom 640 (12%) answered the call. 234 (4.5%) were eligible,​ consented,​ and were randomly assigned to Group 1 (n=79),​ Group 2 (n=79),​ or UC (n=76). Groups were comparable in baseline demographic and tobacco use characteristics. At 6-month follow-up,​ the pooled intervention group vs. UC had a higher documented rate of any smoking cessation treatment (63% vs. 34%,​ p< 0.001),​ any counseling (47% vs. 9%,​ p< 0.001) and any cessation medication prescription (53% vs. 30%,​ p=0.001). Group 1 participants received more cessation treatment than Group 2 (76% vs 51%,​ p=0.001). There was no significant difference in self-reported past-30-day cigarette abstinence among the 3 groups (14.5%,​ Group 1; 14.3%,​ Group 2; and 8.8%,​ UC). Conclusions: A population health strategy of outreach to smokers using automated phone calls was feasible but had modest reach. Both interventions outperformed UC in providing tobacco cessation treatment to smokers who responded to the outreach,​ and the internal coordinator model outperformed the external quitline referral. Future work should aim to improve population reach and test the effect on smoking cessation rates. |
| 372 | Cooperman,​ Nina A.,​ Lu,​ Shou-En,​ Richter,​ Kimber P.,​ Bernstein,​ Steven L.,​ Williams,​ Jill M.. Pilot study of atailored smoking cessation intervention for individuals intreatment for opioid dependence. Nicotine and Tobacco Research. 2018;20:1152.  **Abstract:** Introduction: Over 85% of opioid-dependent individuals in methadone treatment smoke cigarettes; however,​ smoking cessation interventions are minimally effective in this population.To better help opioid-dependent individuals quit smoking,​ we developed and pilot-tested an intervention,​ based in the Information-Motivation-Behavioral Skills (IMB) model of behavior change,​ which could be tailored to address individual barriers to smoking cessation in this population. Methods: We randomized participants (n = 83) in methadone treatment to the eight-session,​ IMB model-based,​ intervention plus nicotine replacement therapy (intervention,​ n = 41) or a facilitated referral to the state Quitline (control,​ n = 42). All participants completed assessments at baseline,​ 3 months,​ and 6 months. Results: Intervention participants completed a median of five sessions (interquartile range [IQR] 3-8) and had significantly higher intervention satisfaction than control participants. Intervention participants reported smoking significantly fewer cigarettes per day at 3 months (median [IQR] = 6 [4-15]) and 6 months (median [IQR] = 8 [4-14]) as compared control participants at 3 months (median [IQR] = 10 [5-20]) and 6 months (median [IQR] = 10 [6-20]). Fifty-six percent of the intervention group and 41% of the control group a made a quit attempt during the study (p = .16). At 3 months,​ 7% (n = 3) of intervention participants and none of the control participants were abstinent from smoking (p = .23). At 6 months,​ 2% of participants in both groups were abstinent.Twenty-four percent and 10% of the intervention and control group participants,​ respectively,​ reported 20 or more smoke-free days (p = .43). Conclusions: An IMB model-based smoking cessation intervention for opioid-dependent smokers is feasible and acceptable in methadone treatment and may help methadone maintained smokers cut down on their smoking. Implications: This is the first study of a tailored,​ IMB Model-based,​ smoking cessation intervention for opioid dependent smokers. Results showed that opioid dependent smokers are willing and able to participate in an IMB model-based smoking cessation intervention,​ and this intervention may help this population cut down on their smoking. Also,​ the Quitline seems less feasible and acceptable for this population than a face-to-face intervention. Further research is needed to determine how to integrate smoking cessation treatment into methadone programs and how to improve interventions so that treatment gains can lead to long-term abstinence in this population. |
| 373 | Noor,​ F.,​ Koegelenberg,​ C.F.N.,​ Esterhuizen,​ T.M.,​ Irusen,​ E.M.. Predictors of treatment success in smoking cessation with varenicline combined with nicotine replacement therapy v. Varenicline alone. South African Medical Journal. 2018;108:45.  **Abstract:** Background. Identification of the predictors of treatment success in smoking cessation may help healthcare workers to improve the effectiveness of attempts at quitting. Objective. To identify the predictors of success in a randomised controlled trial comparing varenicline alone or in combination with nicotine replacement therapy (NRT). Methods. A post-hoc analysis of the data of 435 subjects who participated in a 24-week,​ multicentre trial in South Africa was performed. Logistic regression was used to analyse the effect of age,​ sex,​ age at smoking initiation,​ daily cigarette consumption,​ nicotine dependence,​ and reinforcement assessment on abstinence rates at 12 and 24 weeks. Point prevalence and continuous abstinence rates were self-reported and confirmed biochemically with exhaled carbon monoxide readings. Results. The significant predictors of continuous abstinence at 12 and 24 weeks on multivariate analysis were lower daily cigarette consumption (odds ratio (OR) 1.86,​ 95% confidence interval (CI) 1.21 - 2.87,​ p=0.005 and OR 1.83,​ 95% CI 1.12 - 2.98,​ p=0.02,​ respectively) and older age (OR 1.52,​ 95% CI 1.00 - 2.31,​ p=0.049 and OR 1.79,​ 95% CI 1.13 - 2.84,​ p=0.01,​ respectively). There was no difference in the predictors of success in the univariate analysis,​ except that older age predicted point prevalence abstinence at 12 weeks (OR 1.47,​ 95% CI 1.00 - 2.15,​ p=0.049). The findings were inconclusive for an association between abstinence and lower nicotine dependence,​ older age at smoking initiation and positive reinforcement. Conclusion. Older age and lower daily cigarette consumption are associated with a higher likelihood of abstinence in patients using varenicline,​ regardless of the addition of NRT. |
| 374 | Aveyard, Paul. Effects on abstinence of nicotine patch treatment before quitting smoking: Parallel, two arm, pragmatic randomised trial: The preloading investigators. The BMJ. 2018; 361:k2164.  **Abstract:** Objective To examine the effectiveness of a nicotine patch worn for four weeks before a quit attempt. Design Randomised controlled open label trial. Setting Primary care and smoking cessation clinics in England,​ 2012-15. Participants 1792 adults who were daily smokers with tobacco dependence. 899 were allocated to the preloading arm and 893 to the control arm. Interventions Participants were randomised 1:1,​ using concealed randomly permuted blocks stratified by centre,​ to either standard smoking cessation pharmacotherapy and behavioural support or the same treatment supplemented by four weeks of 21 mg nicotine patch use before quitting: "preloading." Main outcome measures The primary outcome was biochemically confirmed prolonged abstinence at six months. Secondary outcomes were prolonged abstinence at four weeks and 12 months. Results Biochemically validated abstinence at six months was achieved by 157/899 (17.5%) participants in the preloading arm and 129/893 (14.4%) in the control arm: difference 3.0% (95% confidence interval -0.4% to 6.4%),​ odds ratio 1.25 (95% confidence interval 0.97 to 1.62),​ P=0.08 in the primary analysis. There was an imbalance between arms in the frequency of varenicline use as post-cessation treatment,​ and planned adjustment for this gave an odds ratio for the effect of preloading of 1.34 (95% confidence interval 1.03 to 1.73),​ P=0.03: difference 3.8% (0.4% to 7.2%). At four weeks,​ the difference in prolonged abstinence unadjusted for varenicline use was odds ratio 1.21 (1.00 to 1.48),​ difference 4.3% (0.0% to 8.7%),​ P=0.05,​ and adjusted for varenicline use was 1.32 (1.08 to 1.62) P=0.007. At 12 months the odds ratio was 1.28 (0.97 to 1.69),​ difference 2.7% (-0.4% to 5.8%),​ P=0.09 unadjusted for varenicline use and after adjustment was 1.36 (1.02 to 1.80) P=0.04. 5.9% of participants discontinued preloading owing to intolerance. Gastrointestinal symptoms - chiefly nausea - occurred in 4.0% (2.2% to 5.9%) more people in the preloading arm than control arm. Eight serious adverse events occurred in the preloading arm and eight in the control arm (odds ratio 0.99,​ 0.36 to 2.75). Conclusions Evidence was insufficient to confidently show that nicotine preloading increases subsequent smoking abstinence. The beneficial effect seems to have been masked by a concurrent reduction in the use of varenicline in people using nicotine preloading,​ and future studies should explore ways to mitigate this unintended effect. Trial registration Current Controlled Trials ISRCTN33031001. |
| 377 | Murray,​ R.L.,​ Thorley,​ R.,​ Lewis,​ S.,​ Breton,​ M. Opazo,​ Nyakutsikwa,​ B.,​ Cheema,​ K.,​ Ablewhite,​ J.,​ Britton,​ J.. Systematic delivery of a smoking cessation intervention after discharge from a secondary care setting. Tobacco Induced Diseases. 2018;16:198.  **Abstract:** Background: Hospital admission provides an ideal opportunity to promote smoking cessation. Clinical guidelines recommend delivery of cessation interventions for all admissions but no research to date has focused on supporting smokers who successfully abstain from smoking whilst in hospital to maintain abstinence after discharge. We therefore designed a trial to test the effectiveness of an intensive home support intervention to maintain abstinence from smoking for newly abstinent smokers leaving hospital Methods: Participants were recruited from 18 medical wards in one large teaching hospital in the UK and were individually randomised to either intervention or usual care using concealed allocation. During inpatient stay both treatment groups received smoking cessation support as recommended in NICE PH48 guidance. At discharge,​ patients randomised to usual care were offered a referral to a community Stop Smoking Service (SSS). Patients randomised to the intervention group were offered a home visit (or telephone call if a home visit is refused),​ as soon as practicable after discharge and typically within 48 hours,​ to deliver a multi-component intervention which included personalised behavioural support and nicotine replacement therapies (NRT). All smokers were asked to give consent to be contacted at fourweeks and three-months after discharge to assess smoking status and use of cessation support. Results: 404 patients were randomised in total. 200 to intervention and 204 to usual care. Primary outcome data were available at four-weeks for 149 and 134 patients,​ respectively. Preliminary results demonstrate no statistically significant difference in quit rates between treatment groups at either 4 (21.00% intervention,​ 19.12% usual care) or 12 weeks (19.50% Intervention and 16.18% Usual Care) following discharge. Conclusions: Adding a home visit to deliver additional smoking cessation support to patients who have remained abstinent during their hospital stay and treated according to NICE PH48 guidance does not increase quit rates. |
| 381 | Mündel,​ Toby,​ Machal,​ Marine,​ Cochrane,​ Darryl J,​ Barnes,​ Matthew J. A Randomised,​ Placebo-Controlled,​ Crossover Study Investigating the Effects of Nicotine Gum on Strength,​ Power and Anaerobic Performance in Nicotine-Naïve,​ Active Males. Sports medicine - open. 2017;3:5.  **Abstract:** BACKGROUNDNicotine use amongst athletes is high and increasing,​ especially team sports,​ yet the limited previous studies investigating the performance consequences of this behaviour have not examined the effects of the principal active ingredient,​ nicotine,​ per se. Therefore,​ we determined whether nicotine gum affected muscular and anaerobic performance.METHODSNine active males (24 ± 3 years) completed three trials in a random order in which 20 min prior to testing they chewed 2 mg (NIC-2),​ 4 mg (NIC-4) nicotine or flavour-matched placebo (PLA) gum. Peak and average peak isometric,​ concentric and eccentric leg extensor torque was measured followed by vertical counter-movement jump height and a 30-s Wingate test. Heart rate was measured whilst capillary blood samples determined pH,​ HCO3- and venous blood confirmed the presence of nicotine.RESULTSNicotine was confirmed by the presence of its major metabolite,​ cotinine and participants reported no side effects with nicotine. Peak and average peak isometric and eccentric torque was significantly affected (NIC-2 > PLA; p < 0.05) whilst peak (NIC-2 > PLA; p < 0.05) but not average peak (p > 0.05) concentric torque was different between trials. Counter-movement jump height was similar across trials (p > 0.05). Anaerobic capacity during the Wingate remained similar across trials (p > 0.05); however,​ pacing strategy (peak power and rate of fatigue) was different during NIC-2 than PLA. pH was affected by nicotine (NIC-2 > PLA; p < 0.05) and was reduced following the Wingate in all trials. HCO3- showed similar responses across trials (p > 0.05) although it was also reduced following the Wingate (p < 0.05),​ whilst heart rate was significantly affected (NIC-2/NIC-4 > PLA; p < 0.05).CONCLUSIONSChewing low-dose (2 mg) nicotine gum 20 min prior to exercise significantly improved leg extensor torque but did not affect counter-movement jump height or Wingate performance compared to a placebo,​ whilst there were minimal effects of the 4 mg nicotine gum on the performance parameters measured. |
| 387 | Bekiroglu,​ Korkut,​ Russell,​ Michael A,​ Lagoa,​ Constantino M,​ Lanza,​ Stephanie T,​ Piper,​ Megan E. Evaluating the effect of smoking cessation treatment on a complex dynamical system. Drug and alcohol dependence. 2017;180:215.  **Abstract:** OBJECTIVETo understand the dynamic relations among tobacco withdrawal symptoms to inform the development of effective smoking cessation treatments. Dynamical system models from control engineering are introduced and utilized to evaluate complex treatment effects. We demonstrate how dynamical models can be used to examine how distinct withdrawal-related processes are related over time and how treatment influences these relations.METHODIntensive longitudinal data from a randomized placebo-controlled smoking cessation trial (N=1504) are used to estimate a dynamical model of withdrawal-related processes including momentary craving,​ negative affect,​ quitting self-efficacy,​ and cessation fatigue for each of six treatment conditions (nicotine patch,​ nicotine lozenge,​ bupropion,​ patch + lozenge,​ bupropion + lozenge,​ and placebo).RESULTSEstimation and simulation results show that (1) withdrawal measurements are interrelated over time,​ (2) nicotine patch + nicotine lozenge showed reduced cessation fatigue and enhanced self-efficacy in the long-term while bupropion + nicotine lozenge was more effective at reducing negative affect and craving,​ and (3) although nicotine patch + nicotine lozenge had a better initial effect on cessation fatigue and self-efficacy,​ nicotine lozenge had a stronger effect on negative affect and nicotine patch had a stronger impact on craving.CONCLUSIONSThis approach can be used to provide new evidence illustrating (a) the total impact of treatment conditions (via steady state values) and (b) the total initial impact (via rate of initial change values) on smoking-related outcomes for separate treatment conditions,​ noting that the conditions that produce the largest change may be different than the conditions that produce the fastest change. |
| 391 | Rohsenow,​ Damaris J,​ Tidey,​ Jennifer W,​ Martin,​ Rosemarie A,​ Colby,​ Suzanne M,​ Swift,​ Robert M,​ Leggio,​ Lorenzo,​ Monti,​ Peter M. Varenicline versus nicotine patch with brief advice for smokers with substance use disorders with or without depression: effects on smoking,​ substance use and depressive symptoms. Addiction (Abingdon,​ England). 2017;112:1808.  **Abstract:** AIMSVarenicline was compared with transdermal nicotine (NRT) for smokers with current substance use disorders (SUD) for effects on 3-month smoking abstinence (primary outcome) and,​ secondarily,​ on 3- and 6 month abstinence while adjusting for medication adherence,​ and on additional smoking and substance use outcomes. Moderation by major depressive disorder history (MDD) and adherence were investigated.DESIGNDouble-blind double-placebo-controlled randomized design,​ stratifying by MDD,​ gender and nicotine dependence,​ with 3 and 6 months follow-up.SETTINGUniversity offices in Rhode Island,​ USA.PARTICIPANTSAdult smokers (n = 137),​ in SUD treatment,​ substance abstinent <12 months (n = 77 varenicline,​ 60 NRT).INTERVENTION AND COMPARATORTwelve weeks of varenicline (2 mg/day,​ after 1-week dose run-up) or NRT (21 mg/day decreasing to 7 mg/day).MEASUREMENTSPrimary: point-prevalence smoking abstinence (7-day,​ confirmed) at 3 months. Secondary: point-prevalence abstinence at 6 months,​ quantity and frequency of smoking and substance use at 3 and 6 months,​ and within-treatment abstinence,​ medication adherence and depressive symptoms. Smoking outcome analyses were repeated controlling for adherence and investigating adherence as a moderator.FINDINGSEffects on 3-month abstinence were P < 0.065 without a covariate (Bayes factor 3.35,​ supporting the effect strongly) and differed significantly when controlling for baseline smoking [varenicline: 13%,​ NRT: 3%; odds ratio (OR) = 4.81,​ 95% confidence interval (CI) 1.00,​ 23.13,​ P < 0.05]. The threefold difference at 6 months was not significant. Medication effect on abstinence across time was significant (P < 0.05) covarying adherence and baseline smoking (OR = 6.40,​ 95% CI = 1.00,​ 40.93). Medication differences in 3-month abstinence occurred among participants with ≥ 77% adherence (P < 0.02). No significant medication effects on heavy drinking,​ drug use or depressive symptoms were found.CONCLUSIONSVarenicline appears to improve the chances of achieving at least 3 months of smoking abstinence in smokers with substance use disorders trying to stop,​ compared with transdermal nicotine patches,​ the effect being independent of history of depressive disorder. |
| 399 | Hansen,​ Eva Ø,​ Arendt-Nielsen,​ Lars,​ Boudreau,​ Shellie A. A Comparison of Oral Sensory Effects of Three TRPA1 Agonists in Young Adult Smokers and Non-smokers. Frontiers in physiology. 2017;8:663.  **Abstract:** This study profiled intra-oral somatosensory and vasomotor responses to three different transient receptor potential (TRP) channels,​ subfamily A,​ member 1 (TRPA1) agonists (menthol,​ nicotine,​ and cinnamaldehyde) in smoking and non-smoking young adults. Healthy non-smokers (N = 30) and otherwise healthy smokers (N = 25) participated in a randomized,​ double-blinded,​ cross-over study consisting of three experimental sessions in which they received menthol (30 mg),​ nicotine (4 mg),​ or cinnamaldehyde (25 mg) chewing gum. Throughout a standardized 10 min chewing regime,​ burning,​ cooling,​ and irritation intensities,​ and location were recorded. In addition,​ blood pressure,​ heart rate and intra-oral temperature were assessed before,​ during,​ and after chewing. Basal intra-oral temperature was lower in smokers (35.2°C ± 1.58) as compared to non-smokers (35.9°C ± 1.61) [F(1,​ 52) = 8.5,​ P = 0.005,​ post hoc,​ p = 0.005]. However,​ the increase in temperature,​ heart rate,​ and blood pressure in response to chewing menthol,​ nicotine,​ and cinnamaldehyde gums were similar between smokers and non-smokers. Although smoking status did not influence the intensity of burning,​ cooling,​ and irritation,​ smokers did report nicotine burn more often (92%) than non-smokers (63%) [[Formula: see text] = 6.208,​ P = 0.013]. Reports of nicotine burn consistently occurred at the back of the throat and cinnamaldehyde burn on the tongue. The cooling sensation of menthol was more widely distributed in the mouth of non-smokers as compared to smokers. Smoking alters thermoregulation,​ somatosensory,​ and possibly TRPA1 receptor responsiveness and suggests that accumulated exposure of nicotine by way of cigarette smoke alters oral sensory and vasomotor sensitivity. |
| 402 | Lambrichts,​ D.P.V.,​ Boersema,​ G.S.A.,​ Tas,​ B.,​ Wu,​ Z.,​ Vrijland,​ W.W.,​ Kleinrensink,​ G.J.,​ Jeekel,​ J.J.,​ Lange,​ J.F.,​ Menon,​ A.G.. Nicotine chewing gum for the prevention of postoperative ileus after colorectal surgery-A multicentre,​ double-blind,​ randomised,​ controlled pilot study. European Surgical Research. 2017;58:35.  **Abstract:** Background: When postoperative ileus is not resolved after 5 days or recurs after apparent resolution,​ prolonged POI (PPOI) is diagnosed. PPOI increases discomfort,​ morbidity and hospitalization length and is mainly caused by an inflammatory response following intestinal manipulation. This can be weakened by targeting the cholinergic anti-inflammatory pathway,​ with nicotine as essential regulator. Chewing gum,​ already known to stimulate gastrointestinal motility itself,​ combined with nicotine is hypothesized to improve gastrointestinal recovery and prevent PPOI. This pilot study is the first to assess effectiveness and safety of nicotine gum in colorectal surgery. Material and Methods: Patients undergoing elective oncological colorectal surgery were enrolled in this double-blind,​ controlled trial and randomly assigned to a treatment protocol with normal or nicotine gum (2 mg). Patient reported outcomes (PROMS),​ clinical characteristics and blood samples were collected. Primary endpoint was defined as time to first passage of faeces and toleration of solid food for at least 24 h. Results: Forty patients were enrolled (20 vs. 20). In both groups 30% of patients developed PPOI. Median time to primary endpoint (4.50 [3.00-7.25] vs. 3.50 days [3.00-4.25],​ p = 0.398) and median length of stay (5.50 [4.00-8.50] vs. 4.50 days [4.00-6.00],​ p = 0.738) did not differ significantly between normal and nicotine gum. There were no differences in PROMS,​ inflammatory parameters and postoperative complications. Conclusion: We proved nicotine gum to be safe but ineffective in improving gastrointestinal recovery and prevention of PPOI after colorectal surgery. Other dosages and administration routes of nicotine should be tested in future research. |
| 403 | Schuppers, A., De Jong, B., Van Den Oever, H., Arbouw, M., Kruisdijk-Gerritsen, A., Van Zanten, A.. Nicotine replacement therapy in the intensive care unit: A randomized controlled pilot study. Intensive Care Medicine Experimental. 2017; 5(S2):0437.  **Abstract:** INTRODUCTION. Delirium and agitation are common in mechanically ventilated patients admitted to the intensive care unit (ICU). Retrospective data have shown that smoking,​ or the acute abstinence of smoking,​ is an additional risk factor for agitation. A safe and effective method to treat nicotine withdrawal symptoms in outpatient and hospitalized smoking adults is transdermal nicotine replacement therapy (NRT). Previous studies on the use of transdermal NRT in critically ill smokers admitted to the ICU have produced conflicting results.The main goal of this pilot study was to investigate the feasibility of a phase III trial,​ by conducting a test run of a randomized controlled trial. We aimed to assess the safety and efficacy of transdermal NRT in mechanically ventilated smokers admitted to the ICU. METHODS. In this two-centre,​ randomized,​ placebo-controlled pilot study,​ mechanically ventilated smokers admitted to the ICU were included. Subjects received either a nicotine patch (14 or 21 mg/day) or a placebo patch daily until ICU discharge or for a maximum of 30 days. The primary outcome was 30-day mortality. Secondary outcomes included 90-day mortality and number of (serious) adverse events. In a post hoc analysis,​ we defined a composite outcome parameter as time spent alive without sedation and delirium in the first 30 days. RESULTS. The study was stopped after the inclusion of 47 patients. The two groups were comparable with respect to baseline characteristics. No differences were found in 30-day mortality and 90-day mortality between NRT and control group (2/21 vs. 2/26; p = 0.843 and 3/21 vs. 5/26; p = 0.665). There was no difference in the number of adverse and serious adverse events,​ between NRT and control patients (102 vs. 177; p = 0.096,​ and 5 vs. 11; p = 0.251). Patients in the NRT group had spent more time alive without sedation and delirium during the first 10 days (160 hours (96-216) vs. 88 hours (20-210); p = 0.043) and during the first 20 days (400 hours (316-448) vs. 304 hours (110-432); p = 0.033) of treatment. CONCLUSION. Transdermal NRT in mechanically ventilated smokers had no effect on 30- and 90-day mortality,​ although our study was underpowered to establish such differences. Furthermore,​ the number of (serious) adverse events between the two groups was comparable. Finally,​ patients with NRT spent more time alive without sedation and delirium during the first 10 and 20 days. We suggest that future trials should focus on a possible effect of NRT on predefined delirium endpoints,​ particularly in the second and third weeks of ICU stay. The results of this pilot study showed that,​ with some modifications of the trial design,​ a randomized controlled phase III study to assess the safety and efficacy of transdermal NRT in mechanically ventilated smokers admitted to the ICU is feasible. (Figure Presented). |
| 410 | Malaithong,​ Wanwipha,​ Munjupong,​ Sithapan. Efficacy of a transdermal nicotine patch in pain relief after arthroscopic shoulder surgery: A randomized controlled trial. Journal of the Medical Association of Thailand. 2017;100:901.  **Abstract:** Background: Perioperative nicotine administration was suggested to reduce pain scores and opioid consumption in visceral pain control. However,​ there is no evidence to support the analgesic effect of nicotine administration in postoperative somatic pain. Objective: To study the efficacy of transdermal nicotine patches (TNP) in postoperative somatic pain relief by assessing numerical rating scale (NRS) scores and opioid consumption. Material and Method: A prospective,​ double-blind,​ placebo-controlled study was conducted in 46 patients,​ who received general anesthesia for elective arthroscopic shoulder surgery. All participants were randomly allocated to receive a patch of 7 mg nicotine or placebo before induction of anesthesia and remaining for 24 hours after surgery. Participants and assessors were blinded to allocation. Average pain score and intravenous morphine patient-controlled analgesia (PCA) consumption were assessed at 1 hour and 24 hours postoperatively. Results: There was no significant difference in mean NRS and average opioid consumption at 1 hour and 24 hours postoperatively between controlled and treatment group. However,​ the significant reduction in average NRS from baseline at 1 hour and 24 hours postoperatively were found in both groups (p<0.001). Conclusion: Administration of a 7 mg TNP did not significantly reduce in pain scores and postoperative opioid consumption compared with transdermal placebo during perioperative elective arthroscopic shoulder surgery. |
| 411 | Schlagintweit,​ Hera E,​ Campbell,​ Niamh K,​ Barrett,​ Sean P. Quit Intentions Moderate Subjective and Physiological Responses to Acute Nicotine Replacement Therapy Administration in Dependent Smokers. Nicotine & tobacco research : official journal of the Society for Research on Nicotine and Tobacco. 2017;19:922.  **Abstract:** IntroductionThis study assessed the impact of expectancy and administration components of acute nicotine inhaler use on craving,​ heart rate,​ and smoking behavior in smokers with varying intentions to quit.Methods47 dependent smokers that differed in self-reported intention to quit (no intention to quit during the next month N = 26 vs. intention to initiate a quit attempt within 2 weeks N = 21) were randomly administered a 4 mg nicotine or nicotine-free inhaler across two sessions. Instructions regarding the inhaler's nicotine content (expect nicotine vs. expect nicotine-free; nicotine expectancy) and flavor (mint vs. citrus) varied across sessions. Craving and heart rate were assessed before and after inhaler administration (two-second inhalations every 10 seconds over 20 minutes). Next,​ participants were offered an opportunity to self-administer puffs of their preferred tobacco brand during an hour-long progressive ratio task.ResultsAcross participants,​ nicotine expectancy independently reduced withdrawal related craving (p = .018),​ but no comparable effects of nicotine administration were evident. In quitting motivated smokers,​ nicotine expectancy and administration interacted to reduce intention to smoke (p = .040),​ while nicotine expectancy (p = .047) and administration (p = .025) independently reduced intention to smoke in quitting unmotivated smokers. Blunted heart rate reactivity to nicotine administration was observed in quitting motivated relative to unmotivated smokers (p = .042); however,​ neither expectancy nor administration impacted smoking behavior in either group (p values > .25).ConclusionsFindings indicate that participant quitting intentions moderate acute nicotine replacement therapy responses. In quitting motivated smokers,​ a combination of pharmacological and psychological factors may be necessary for nicotine replacement therapy to impact craving.ImplicationsFindings from this study demonstrate that motivations to quit smoking moderate subjective and physiological responses to acute nicotine administration and expectancy in dependent cigarette smokers. Quitting motivated smokers showed blunted heart rate reactivity to nicotine administration,​ suggesting that they may be less sensitive to the rewarding aspects of nicotine consumption. Nicotine administration and expectancy were found to interact to reduce craving in quitting motivated but not in unmotivated smokers,​ suggesting that pharmacological and psychological factors may be necessary for nicotine replacement therapy to impact craving in smokers who plan to quit. |
| 413 | Murphy,​ Cara M,​ MacKillop,​ James,​ Martin,​ Rosemarie A,​ Tidey,​ Jennifer W,​ Colby,​ Suzanne M,​ Rohsenow,​ Damaris J. Effects of varenicline versus transdermal nicotine replacement therapy on cigarette demand on quit day in individuals with substance use disorders. Psychopharmacology. 2017;234:2443.  **Abstract:** RATIONALECigarette demand is a behavioral economic measure of the relative value of cigarettes. Decreasing the value of cigarette reinforcement may help with quitting smoking.OBJECTIVESThis study aimed to evaluate the effects of initial use of varenicline (VAR) versus nicotine replacement therapy (NRT) on demand for cigarettes on quit day among smokers with substance use disorders (SUD) and to determine whether reduced demand was associated with subsequent abstinence from smoking at 1 and 3 months.METHODSParticipants (N = 110) were randomized to double-blind,​ double-placebo conditions: VAR with placebo NRT or NRT with placebo capsules. The cigarette purchase task (CPT) was used to assess demand for cigarettes at baseline and on quit day,​ following a 1-week medication dose run-up/placebo capsule lead-in and first day use of the patch.RESULTSDemand for cigarettes decreased from baseline to quit day without significant differences between medications. Reductions in CPT intensity (number of cigarettes that would be smoked if they were free) and CPT breakpoint (lowest price at which no cigarettes would be purchased) predicted greater likelihood of abstaining on quit day. Reduced intensity predicted length of abstinence at 1 and 3 months while reduced breakpoint predicted only 1 month length of abstinence.CONCLUSIONSInitial therapeutic doses of VAR and NRT resulted in similar reductions in cigarette reinforcement. Larger initial reductions in demand on quit day were associated with early success with abstaining from cigarettes. Behavioral economic approaches may be useful for identifying individuals who benefit less from pharmacotherapy and may need additional treatment resources.TRIAL REGISTRATIONhttps://clinicaltrials.gov/ct2/show/NCT00756275. |
| 415 | Kushnir,​ Vladyslav,​ Sproule,​ Beth A,​ Cunningham,​ John A. Impact of large-scale distribution and subsequent use of free nicotine patches on primary care physician interaction. BMC public health. 2017;18:4.  **Abstract:** BACKGROUNDLarge-scale distribution efforts of free nicotine replacement therapy (NRT) have been documented to be cost-effective interventions for increasing smoking quit rates. However,​ despite nearly a dozen studies evaluating their effectiveness,​ none have examined whether free NRT provision promotes further primary care help-seeking and the impact that it may have on cessation efforts.METHODSIn the context of a randomized controlled trial,​ a secondary analysis was conducted on 1000 adult regular smokers randomized to be mailed a 5-week supply of nicotine patches or to a no intervention control group. Recipients and users of free nicotine patches at an 8 week follow-up were successfully case matched to controls based on age,​ gender,​ baseline level of nicotine dependence and intent to quit (n = 201 per group). Differences in physician interaction between the two groups were evaluated at both 8 week and 6 month follow-ups. The impact of physician interaction on self-reported smoking abstinence at each follow-up was also examined.RESULTSAlthough no differences in physician interaction were noted between groups at the 8 week follow-up,​ at the 6 month follow-up,​ nicotine patch users reported greater frequency of discussing smoking with their physician (43.9%),​ as compared to the control group (30.3%) (p = 0.011). Across both groups,​ over 90% of those that discussed smoking with a physician were encouraged to quit and approximately 70% were provided with additional support. Separate ANOVAs revealed no significant impact of physician interaction on cessation (p > 0.05),​ regardless of group or follow-up period,​ however,​ at the 6 month follow-up,​ nicotine patch users who discussed cessation with a physician had made serious quit attempts at significantly greater rates (72.6%),​ compared to controls (49.1%) (p = 0.007).CONCLUSIONSIrrespective of group,​ the majority of smokers in the present study did not discuss cessation with their physician. Recipients and users of nicotine patches however,​ were more likely to discuss smoking with their physician,​ suggesting that the provision of free NRT particularly to those who are likely to use it may facilitate opportunities for benefits beyond the direct pharmacological effects of the medication.TRIAL REGISTRATIONclinicaltrials.gov ,​ NCT01429129 . Registered: 2 September 2011. |
| 417 | Ikonomidis,​ Ignatios,​ Marinou,​ Margarita,​ Vlastos,​ Dimitrios,​ Kourea,​ Kallirhoe,​ Andreadou,​ Ioanna,​ Liarakos,​ Nikolaos,​ Triantafyllidi,​ Helen,​ Pavlidis,​ George,​ Tsougos,​ Elias,​ Parissis,​ John,​ Lekakis,​ John. Effects of varenicline and nicotine replacement therapy on arterial elasticity,​ endothelial glycocalyx and oxidative stress during a 3-month smoking cessation program. Atherosclerosis. 2017;262:123.  **Abstract:** BACKGROUND AND AIMSThe effects of medically-aided smoking cessation on vascular function and oxidative stress are not fully clarified.METHODSOne hundred eighty-eight current smokers were randomized to varenicline or nicotine replacement treatment (NRT) for a 3-month period. We assessed: (a) augmentation index (Aix) and pulse wave velocity (PWV); (b) perfusion boundary region (PBR) of sublingual microvasculature (range:5-25 μm),​ an index of the endothelial glycocalyx thickness,​ using Sideview,​ Darkfield imaging; (c) the exhaled CO; and (d) the malondialdehyde (MDA) and protein carbonyls (PC) plasma levels,​ as markers of oxidative stress,​ at baseline and after 3 and 12 months.RESULTSAfter 3 months of treatment,​ CO,​ MDA,​ PC and Aix were decreased in all subjects (median CO: 25 vs. 6 ppm,​ MDA: 0.81 vs. 0.63 nmol/L,​ PC: 0.102,​ vs. 0.093 nmol/mg protein,​ Aix: 13% vs. 9%,​ p < 0.05) while PWV remained unchanged. Endothelial glycocalyx integrity showed a greater improvement in the varenicline than the NRT treatment (PBR range 5-9 μm: 1.07 ± 0.02 vs. 1.17 ± 0.02 μm,​ p = 0.03) in parallel with the greater CO reduction (5 vs. 7 ppm,​ p = 0.02). At 1-year follow-up,​ MDA,​ PC,​ Aix and PBR at 5-25 μm range were further improved in subjects who abstained from smoking (n = 84 out of 188),​ while the above markers and PWV deteriorated in relapsed smokers (p < 0.05).CONCLUSIONSA smoking cessation program using varenicline or NRT for 3 months resulted in a decrease of CO,​ oxidative stress,​ arterial stiffness and restored endothelial glycocalyx. These effects were more evident after varenicline treatment,​ likely because of a greater CO reduction,​ and were maintained after 1 year only in subjects who abstained from smoking. |
| 419 | Lu,​ Wenying,​ Chappell,​ Kate,​ Walters,​ Julia A E,​ Jacobson,​ Glenn A,​ Patel,​ Rahul,​ Schüz,​ Natalie,​ Ferguson,​ Stuart G. The effect of varenicline and nicotine patch on smoking rate and satisfaction with smoking: an examination of the mechanism of action of two pre-quit pharmacotherapies. Psychopharmacology. 2017;234:1969.  **Abstract:** OBJECTIVESIn recent years,​ there has been growing research interest in using nicotine replacement medications to aid smoking reduction prior to a quit attempt. Gaining a better understanding of how treatments influence smoking reduction may allow for better tailoring of treatments and,​ ultimately,​ better cessation outcomes. The objective of the current study was to test the effects of the pre-quit use of varenicline and nicotine patch on smoking rate and satisfaction with smoking.METHODSAll participants were required to attend up to five study visit sections. Participants (n = 213) who were interested in quitting were randomised (open-label) to receive either pre-quit patch or varenicline (both treatments started 2 weeks prior to an assigned quit day,​ followed by 10 weeks post-quit) or standard patch (10 weeks starting from an assigned quit day). Participants used modified smartphones to monitor their smoking in real time for 4 weeks.RESULTSParticipants in the two pre-quit treatment groups reported significant reductions in both their satisfaction with smoking (p < 0.001) and smoking rate (p < 0.001) from baseline to the end of pre-quit period; participants in the standard patch group did not. The observed reduction of smoking rate was associated with the satisfaction with smoking (p < 0.01),​ although the mediation effect of satisfaction was small.CONCLUSIONSPre-quit treatment caused reductions in satisfaction with smoking and smoking rate. Satisfaction was associated with changes in smoking rate,​ but the relationship was weak. As such,​ monitoring reductions in satisfaction do not appear to be a viable method of evaluating responsiveness to treatment. |
| 427 | Lesage,​ Elise,​ Aronson,​ Sarah E,​ Sutherland,​ Matthew T,​ Ross,​ Thomas J,​ Salmeron,​ Betty Jo,​ Stein,​ Elliot A. Neural Signatures of Cognitive Flexibility and Reward Sensitivity Following Nicotinic Receptor Stimulation in Dependent Smokers: A Randomized Trial. JAMA psychiatry. 2017;74:632.  **Abstract:** ImportanceWithdrawal from nicotine is an important contributor to smoking relapse. Understanding how reward-based decision making is affected by abstinence and by pharmacotherapies such as nicotine replacement therapy and varenicline tartrate may aid cessation treatment.ObjectiveTo independently assess the effects of nicotine dependence and stimulation of the nicotinic acetylcholine receptor on the ability to interpret valence information (reward sensitivity) and subsequently alter behavior as reward contingencies change (cognitive flexibility) in a probabilistic reversal learning task.Design,​ Setting,​ and ParticipantsNicotine-dependent smokers and nonsmokers completed a probabilistic reversal learning task during acquisition of functional magnetic resonance imaging (fMRI) in a 2-drug,​ double-blind placebo-controlled crossover design conducted from January 21,​ 2009,​ to September 29,​ 2011. Smokers were abstinent from cigarette smoking for 12 hours for all sessions. In a fully Latin square fashion,​ participants in both groups underwent MRI twice while receiving varenicline and twice while receiving a placebo pill,​ wearing either a nicotine or a placebo patch. Imaging analysis was performed from June 15,​ 2015,​ to August 10,​ 2016.Main Outcome and MeasuresA well-established computational model captured effects of smoking status and administration of nicotine and varenicline on probabilistic reversal learning choice behavior. Neural effects of smoking status,​ nicotine,​ and varenicline were tested for on MRI contrasts that captured reward sensitivity and cognitive flexibility.ResultsThe study included 24 nicotine-dependent smokers (12 women and 12 men; mean [SD] age,​ 35.8 [9.9] years) and 20 nonsmokers (10 women and 10 men; mean [SD] age,​ 30.4 [7.2] years). Computational modeling indicated that abstinent smokers were biased toward response shifting and that their decisions were less sensitive to the available evidence,​ suggesting increased impulsivity during withdrawal. These behavioral impairments were mitigated with nicotine and varenicline. Similarly,​ decreased mesocorticolimbic activity associated with cognitive flexibility in abstinent smokers was restored to the level of nonsmokers following stimulation of nicotinic acetylcholine receptors (familywise error-corrected P < .05). Conversely,​ neural signatures of decreased reward sensitivity in smokers (vs nonsmokers; familywise error-corrected P < .05) in the dorsal striatum and anterior cingulate cortex were not mitigated by nicotine or varenicline.Conclusions and RelevanceThere was a double dissociation between the effects of chronic nicotine dependence on neural representations of reward sensitivity and acute effects of stimulation of nicotinic acetylcholine receptors on behavioral and neural signatures of cognitive flexibility in smokers. These chronic and acute pharmacologic effects were observed in overlapping mesocorticolimbic regions,​ suggesting that available pharmacotherapies may alleviate deficits in the same circuitry for certain mental computations but not for others.Trial Registrationclinicaltrials.gov Identifier: NCT00830739. |
| 428 | Cook,​ Jessica W,​ Lanza,​ Stephanie T,​ Chu,​ Wanghuan,​ Baker,​ Timothy B,​ Piper,​ Megan E. Anhedonia: Its Dynamic Relations With Craving,​ Negative Affect,​ and Treatment During a Quit Smoking Attempt. Nicotine & tobacco research : official journal of the Society for Research on Nicotine and Tobacco. 2017;19:703.  **Abstract:** IntroductionResearch shows that abstinence from tobacco leads to a withdrawal-related decrement in responsivity to nondrug rewards (ie,​ anhedonia). However,​ it remains unclear how anhedonia relates to other key withdrawal symptoms and withdrawal-related constructs over time. We analyzed ecological momentary assessment data to examine whether a decrement in response to rewards during a 10-day period following quitting shows a pattern of associations with other variables (ie,​ treatment,​ tobacco dependence,​ negative affect,​ and craving) that is consistent with anhedonia being a tobacco withdrawal symptom.MethodsAs part of a randomized controlled trial of smoking cessation therapies,​ 1122 adults (58% female) were assigned to: placebo (n = 131),​ bupropion (alone or with nicotine lozenge; n = 401),​ or nicotine replacement therapy (NRT; lozenge,​ patch,​ both; n = 590). Participants completed 4 ecological momentary assessments per day for 10 days postquit,​ resulting in 22 575 assessments.ResultsTime-varying effect modeling showed that anhedonia was significantly greater among those high in dependence relative to lower dependent smokers out to day 9 postquit. The placebo group showed elevated anhedonia immediately postquit,​ which fell to levels similar to the treatment groups by day 7. NRT effectively reduced anhedonia and its time-varying association with craving early in the quit attempt. The positive association between negative affect and anhedonia was moderate and stable over time for both active treatment groups.ConclusionsThese results provide additional support that anhedonia following quitting smoking is a manifestation of the tobacco withdrawal syndrome.ImplicationsThis study supported the hypothesis that diminished responsivity to nondrug rewards (ie,​ anhedonia) is a symptom of the tobacco withdrawal syndrome. Results showed that anhedonia: (1) was significantly associated with dependence,​ especially during the early postquit period when withdrawal was at its peak intensity; (2) showed significant time-varying associations with other withdrawal symptoms,​ especially craving; and (3) was significantly suppressed by agonist administration as was its association with craving over time. |
| 444 | Goodney,​ Philip P,​ Spangler,​ Emily L,​ Newhall,​ Karina,​ Brooke,​ Benjamin S,​ Schanzer,​ Andres,​ Tan,​ Tze-Woei,​ Beck,​ Adam W,​ Hallett,​ John H,​ MacKenzie,​ Todd A,​ Edelen,​ Maria O,​ Hoel,​ Andrew W,​ Rigotti,​ Nancy A,​ Farber,​ Alik. Feasibility and pilot efficacy of a brief smoking cessation intervention delivered by vascular surgeons in the Vascular Physician Offer and Report (VAPOR) Trial. Journal of vascular surgery. 2017;65:1152.  **Abstract:** BACKGROUNDThis study determined the feasibility and potential efficacy of an evidence-based standardized smoking cessation intervention delivered by vascular surgeons to smokers with peripheral arterial disease.METHODSWe performed a cluster-randomized trial of current adult smokers referred to eight vascular surgery practices from September 1,​ 2014,​ to July 31,​ 2015. A three-component smoking cessation intervention (physician advice,​ nicotine replacement therapy,​ and telephone-based quitline referral) was compared with usual care. The primary outcome was smoking cessation for 7 days,​ assessed 3 months after the intervention. Secondary outcomes were patients' nicotine dependence and health expectancies of smoking assessed using Patient Reported Outcomes Measurement Information System (PROMIS; RAND Corporation,​ Santa Monica,​ Calif).RESULTSWe enrolled 156 patients (65 in four intervention practices,​ 91 in four control practices),​ and 141 (90.3%) completed follow-up. Patients in the intervention and control practices were similar in age (mean,​ 61 years),​ sex (68% male),​ cigarettes per day (mean,​ 14),​ and prior quit attempts (77%). All three components of the intervention were delivered to 75% of patients in intervention practices vs to 7% of patients at control practices (P < .001). At 3 months,​ 23 of 57 patients (40.3%) in the intervention group quit smoking (23 of 56 patients quit who completed follow-up,​ plus 1 death included in the analysis in the denominator as a smoker),​ and 26 of 84 patients (30.9%) In the control group quit smoking (26 patients of 84 who completed follow-up,​ including 2 deaths included in the denominator as smokers). This difference (40.3% quit rate in intervention,​ 31% quit rate in control; P = .250) was not statistically significant in crude analyses (P = .250) or analyses adjusted for clustering (P = .470). Multivariable analysis showed factors associated with smoking cessation were receipt of physician advice (odds ratio for cessation,​ 1.96; 95% confidence interval,​ 1.28-3.02; P < .002) and nicotine replacement therapy (odds ratio,​ 1.92; 95% confidence interval,​ 1.43-2.56; P < .001).CONCLUSIONSImplementation of a brief,​ surgeon-delivered smoking cessation intervention is feasible for patients with peripheral arterial disease. A larger trial will be necessary to determine whether this is effective for smoking cessation. |
| 449 | Graham,​ Amanda L,​ Papandonatos,​ George D,​ Cha,​ Sarah,​ Erar,​ Bahar,​ Amato,​ Michael S,​ Cobb,​ Nathan K,​ Niaura,​ Raymond S,​ Abrams,​ David B. Improving Adherence to Smoking Cessation Treatment: Intervention Effects in a Web-Based Randomized Trial. Nicotine & tobacco research : official journal of the Society for Research on Nicotine and Tobacco. 2017;19:324.  **Abstract:** BackgroundWeb-based smoking cessation interventions can deliver evidence-based treatments to a wide swath of the population,​ but effectiveness is often limited by insufficient adherence to proven treatment components. This study evaluated the impact of a social network (SN) intervention and free nicotine replacement therapy (NRT) on adherence to evidence-based components of smoking cessation treatment in the context of a Web-based intervention.MethodsA sample of adult U.S. smokers (N = 5290) was recruited via BecomeAnEX.org,​ a free smoking cessation Web site. Smokers were randomized to one of four arms: (1) an interactive,​ evidence-based smoking cessation Web site (WEB) alone; (2) WEB in conjunction with an SN intervention designed to integrate participants into the online community (WEB+SN); (3) WEB plus free NRT (WEB+NRT); and (4) the combination of all treatments (WEB+SN+NRT). Adherence outcomes assessed at 3-month follow-up were as follows: Web site utilization metrics,​ use of skills training components,​ intratreatment social support,​ and pharmacotherapy use.ResultsWEB+SN+NRT outperformed all others on Web site utilization metrics,​ use of practical counseling tools,​ intratreatment social support,​ and NRT use. It was the only intervention to promote the sending of private messages and the viewing of community pages over WEB alone. Both social network arms outperformed WEB on most metrics of online community engagement. Both NRT arms showed higher medication use compared to WEB alone.ConclusionsThis study demonstrated the effectiveness of two approaches for improving adherence to evidence-based components of smoking cessation treatment. Integrated approaches to medication provision and social network engagement can enhance adherence to components known to improve cessation.ImplicationsThis study demonstrated that an integrated approach to medication provision and social network integration,​ when delivered through an online program,​ can enhance adherence across all three recommended components of an evidence-based smoking cessation program (skills training,​ social support,​ and pharmacotherapy use). Nicotine replacement therapy-when provided as part of an integrated program-increases adherence to other program elements,​ which in turn augment its own therapeutic effects. An explicit focus on approaches to improve treatment adherence is an important first step to identifying leverage points for optimizing intervention effectiveness. |
| 451 | Ben Taleb,​ Ziyad,​ Ward,​ Kenneth D,​ Asfar,​ Taghrid,​ Jaber,​ Rana,​ Bahelah,​ Raed,​ Maziak,​ Wasim. Smoking Cessation and Changes in Body Mass Index: Findings From the First Randomized Cessation Trial in a Low-Income Country Setting. Nicotine & tobacco research : official journal of the Society for Research on Nicotine and Tobacco. 2017;19:351.  **Abstract:** BackgroundIn high-income countries,​ quitting cigarette smoking is associated with weight gain,​ which can reduce motivation to abstain. Whether smoking cessation is associated with weight gain in a low-income country context has never been investigated. We aimed to determine the post-cessation changes in body mass index (BMI) and its predictors among smokers who received a smoking cessation intervention in a low-income country setting.MethodsWe performed post hoc analyses of data from 269 smokers who participated in a two-group,​ parallel-arm,​ double-blind,​ placebo-controlled randomized trial of combined nicotine replacement therapy (NRT) and behavioral counseling in primary care clinics in Aleppo,​ Syria. We used generalized estimating equation modeling to identify predictors of changes in BMI at 6 weeks and 6- and 12-month follow-ups after quit date.ResultsThe mean pre-cessation BMI of the sample was 27.9kg/m2 (SD = 5.2). Over 12 months of follow-up,​ BMI of smoking abstainers averaged 1.8 BMI units (approximately 4.8kg) greater than non-abstainers (p = .012). Throughout the study,​ greater BMI was associated with being female (p = .048),​ reporting smoking to control weight (p < .001) and having previously failed to quit due to weight gain (p = .036).ConclusionSimilar to findings from high-income countries,​ smoking cessation in Syria is associated with weight gain,​ particularly among women and those who have weight concerns prior to quitting. This group of smokers may benefit from tailored cessation interventions with integrated body weight management elements that take into consideration the prevailing local and cultural influences on diet and levels of physical activity.ImplicationsThis study provides the first evidence regarding post-cessation changes in BMI among smokers who attempt to quit in a low-income country setting. Our findings advance knowledge regarding post-cessation weight gain and offers insight for researchers and clinicians to identify smokers at higher risk of post-cessation weight gain. This information will help in delivering interventions that take into account the prevailing cultural influence on diet and physical activity and will ultimately help in designing future tailored cessation programs in Syria and other low-income countries with similar cultural background and level of development. |
| 458 | Piper,​ Megan E,​ Cook,​ Jessica W,​ Schlam,​ Tanya R,​ Smith,​ Stevens S,​ Bolt,​ Daniel M,​ Collins,​ Linda M,​ Mermelstein,​ Robin,​ Fiore,​ Michael C,​ Baker,​ Timothy B. Toward precision smoking cessation treatment II: Proximal effects of smoking cessation intervention components on putative mechanisms of action. Drug and alcohol dependence. 2017;171:50.  **Abstract:** BACKGROUNDUnderstanding how smoking cessation treatments exert their effects can inform treatment development and use. Factorial designs allow researchers to examine whether multiple intervention components affect hypothesized change mechanisms,​ and whether the affected mechanisms are related to cessation.METHODSThis is a secondary data analysis of smokers recruited during primary care visits (N=637,​ 55% women,​ 87% white) who were motivated to quit. Participants in this fractional factorial experiment were randomized to one level of each of six intervention factors: Prequit Nicotine Patch vs None,​ Prequit Nicotine Gum vs None,​ Preparation Counseling vs None,​ Intensive In-Person Counseling vs Minimal,​ Intensive Phone Counseling vs Minimal,​ and 16 vs 8 Weeks of Combination Nicotine Replacement (nicotine patch+nicotine gum). Data on putative mechanisms (e.g.,​ medication use,​ withdrawal,​ self-efficacy) and smoking status were gathered using daily assessments and during follow-up assessment calls.RESULTSSome intervention components influenced hypothesized mechanisms. Prequit Gum and Patch each reduced prequit smoking and enhanced prequit coping and self-efficacy. In-Person Counseling increased prequit motivation to quit,​ postquit self-efficacy,​ and postquit perceived intratreatment support. Withdrawal reduction and reduced prequit smoking produced the strongest effects on cessation. The significant effect of combining Prequit Gum and In-Person Counseling on 26-week abstinence was mediated by increased prequit self-efficacy.CONCLUSIONSThis factorial experiment identified which putative treatment mechanisms were influenced by discrete intervention components and which mechanisms influenced cessation. Such information supports the combined use of prequit nicotine gum and intensive in-person counseling as cessation interventions that operate via increased prequit self-efficacy. |
| 460 | Mamey,​ Mary Rose,​ Burns,​ Leonard,​ Barbosa-Leiker,​ Celestina,​ Parks,​ Craig,​ McPherson,​ Sterling. Parallel growth modeling to better understand co-addiction: A randomized clinical trial of tobacco smoking and stimulant use. Drug and Alcohol Dependence. 2017;171:e127.  **Abstract:** Aims: To demonstrate the usefulness of a parallel latent growth curve model in co-addiction treatment research. Using data from a combined treatment study,​ cigarette smoking (target) and stimulant use (SU; secondary target) were modeled to determine whether (1) initial levels of smoking and SU were related to each other and/or change over time; (2) change in smoking and SU were related to each other and/or initial levels; and (3) treatment was related to change over time. Methods: Secondary data analyses were performed on participants (n = 528) who took part in a 10-week RCT. The placebo group received one 10-min counseling session 1×/week (treatment as usual; TAU) to address SU. The treatment group received TAU and smoking cessation treatment (SCT),​ which included bupropion,​ smoking cessation counseling,​ nicotine inhaler,​ and contingency management. Smoking was measured using carbon monoxide (CO; >8ppm indicated CO+),​ and SU was measured using biochemical urinalysis (UA). Results: The parallel LGCM showed that there was a significant relationship (r =-.130; p < 0.05) between the initial statuses of the disorders: those with CO+ at baseline were also more likely to test UA+ at baseline. There were no other significant relationships. There was a significant treatment effect on change (β = .523; p < 0.05) in CO levels,​ but no significant effect on change SU,​ where those in the treatment group had more of a decrease in CO levels than those in the placebo group. Conclusions: The relationship between cigarette smoking and SU remains high,​ though the two are rarely treated simultaneously. While these findings are in line with the original findings reported,​ it is important for treatment researchers to understand and better optimize how treatment of one disorder can have an impact on secondary targeted disorders. |
| 471 | Brody,​ Arthur L,​ Zorick,​ Todd,​ Hubert,​ Robert,​ Hellemann,​ Gerhard S,​ Balali,​ Shabnam,​ Kawasaki,​ Sarah S,​ Garcia,​ Lizette Y,​ Enoki,​ Ryutaro,​ Abraham,​ Paul,​ Young,​ Paulina,​ McCreary,​ Charles. Combination Extended Smoking Cessation Treatment Plus Home Visits for Smokers With Schizophrenia: A Randomized Controlled Trial. Nicotine & tobacco research : official journal of the Society for Research on Nicotine and Tobacco. 2017;19:68.  **Abstract:** INTRODUCTIONThe majority of people with schizophrenia have a diagnosis of tobacco dependence during their lifetime. A major obstacle to reducing the burden of cigarette smoking in this population is that these smokers have lower quit rates when undergoing standard treatment compared to smokers with no mental illness. We sought to determine if combination extended treatment (COMB-EXT) and home visits (HV) would lead to improved outcomes in smokers with schizophrenia.METHODSThirty-four cigarette smokers with schizophrenia completed either COMB-EXT with HV,​ COMB-EXT without HV,​ or treatment as usual (TAU) (random assignment). COMB-EXT consisted of group cognitive-behavioral therapy (CBT),​ bupropion,​ nicotine patch,​ and nicotine lozenge,​ which were initiated within 2 weeks and continued for 26 weekly visits. HV consisted of biweekly visits to the home with assessment of secondhand smoke (SHS) exposure and brief behavioral therapy with participants and others in the home environment. TAU consisted of group CBT plus serial single or combination medication trials as per standard care.RESULTSSmokers with schizophrenia who received COMB-EXT (with or without HV) had greater reductions in cigarettes per day than those treated with TAU (both ps < .01). In addition,​ 7-day point prevalence abstinence rates for the three groups were 45%,​ 20%,​ and 8%,​ respectively,​ which was significantly higher for COMB-EXT plus HV than TAU (χ2(1) = 4.8,​ p = .03). Groups did not differ significantly in the number of adverse events,​ and HV were easily scheduled.CONCLUSIONCOMB-EXT improves outcomes for smokers with schizophrenia. HV appeared to provide additional benefit for smoking cessation in this treatment-resistant population.IMPLICATIONSThe clear benefit found here of rapidly initiated,​ combination,​ extended treatment over TAU suggests that aggressive and extended treatment should be considered in clinical practice for smokers with schizophrenia. Furthermore,​ HV to address SHS exposure showed initial promise for assisting smokers with schizophrenia in maintaining abstinence,​ indicating that this intervention may be worthy of future research. |
| 474 | Johns,​ D.. Randomised controlled trial comparing nicotine replacement therapy (nrt) and counselling on smoking cessation in patients prone to lung cancer using bed font micro-smokerlyzer. Supportive Care in Cancer. 2017;25:S102.  **Abstract:** Introduction Tobacco use is the greatest preventable morbidity and mortality. Cessation interventions requires pharmacotherapy and behavioural supportive care. Objectives To determine whether a brief counselling or counselling intervention given with NRT,​ is more effective than usual care in promoting smoking cessation. Methods Patients who were prone to lung cancer were randomised to receive either usual care (no additional advice at admission),​ counselling alone (20 minute intervention with written materials),​ or NRT plus counselling (counselling intervention with a 6 week course of NRT). Inclusion Criteria-Previous lung disease,​ a family history of lung cancer,​ Past cancer treatment,​ Lowered immunity,​ Previous smoking related cancers,​ Exposure to certain chemicals and radon gas. Continuous and point prevalence abstinence from smoking (validated by exhaled carbon monoxide <10 ppm) was measured at 3 and 12 months,​ and self-reported reduction in cigarette consumption in smokers was assessed at 3 and 12 months. Results 300 smokers were enrolled. Abstinence was higher in the NRT plus counselling group (n=100) than in the counselling alone (n=100) or usual care (n=100) groups. The difference between the groups was significant for validated point prevalence abstinence at 3 months (65%,​33%,​ 27% respectively,​ p=0.045) and at 12 months (27%,​ 16%,​ 14%,​ p=0.03). There was no significant difference between counselling alone and usual care,​ or in reduction in cigarette consumption between the treatment groups. The NRT preferred was Nicotine transdermal patch 43%,​nicotine gum 27%,​ Nicotine inhalator 12%,​ Nicotine sublingual tablet 11%,​ Nicotine nasal spray 7% Conclusions NRT given with brief counselling to patients prone to lung cancer is an effective smoking cessation intervention. |
| 476 | Rozario,​ H.P.. Nicotine dependence assessment using Fagerstrom test and Nicotine Replacement therapy (NRT) recommendation techniques for smoking cessation among Paniya tribes. Annals of Oncology. 2016;27:ix187.  **Abstract:** Background: Tobacco is a highly addictive substance. It is estimated that 1.9 billion people currently smoke worldwide. Tobacco kills one in two users. It is responsible for the death of 1 in 10 adults,​ with 4.9 million deaths occurring worldwide each year. Tribes are a special population with high prevalence of smoking. Most people who try to stop smoking do so unassisted,​ and many return to smoking within a few months. There is now substantial evidence that pharmacotherapy,​ such as nicotine replacement therapy (NRT),​ can significantly increase an individual's chances of stopping. Indeed,​ it is widely recommended that pharmacotherapy be incorporated into any quit attempt when not contraindicated. Methods: Objectives: To investigate the effectiveness of nicotine replacement therapy (NRT) for smoking cessation among Paniya tribes. Design: Randomised controlled trial. Participants and setting: 200 Paniya tribal smokers from South India,​ aged 18 years and older with a Fagerstrom score of 1 and above were included in the study. Interventions: The scoring of the tribal patients was done using Fagerstrom test for nicotine dependence. The NRT recommendation chart was used to give the appropriate intervention according to the scoring criteria. Main outcome measures: Self-reported abstinence assessed by questionnaires at 1,​ 2,​ 3 and 6 months. Results: Of the 200 patients approached,​ 165 (82.5%) agreed to participate; five of these were later excluded. Among the 160 tried NRT during the study period. At 30-day follow-up,​ 82 (51.25%) who had used NRT planned to continue using them,​ it reduced to 74,​52 and 30 at the end of 2,​ 3 and 6 months respectively. Average cigarette consumption decreased from 15.6 per person/d to 7.6 over the study period (P<.001). Conclusions: Pre existing traditions and customs,​ superadded with illiteracy in Paniya tribes have resulted in reduction in the success rate of NRT therapy. However the therapy have paved the way in reduction of daily tobacco use. |
| 478 | Bernstein,​ Steven L,​ Weiss,​ June-Marie,​ Toll,​ Benjamin,​ Zbikowski,​ Susan M. Association Between Utilization of Quitline Services and Probability of Tobacco Abstinence in Low-Income Smokers. Journal of substance abuse treatment. 2016;71:58.  **Abstract:** INTRODUCTIONQuitlines (QL) are an effective means for smoking cessation,​ but a paucity of data exist examining the dose-response relationship between use of QL services and quit rates,​ especially among low-income smokers. The purpose of this study was to study the relationship between tobacco abstinence and use of QL services among low-income smokers.METHODSSecondary analysis of a randomized trial of every- or some-day smokers aged 18 years or older visiting an urban emergency department. Inclusion criteria included self-pay or Medicaid insurance,​ as a proxy for low-income and low socioeconomic status. Intervention participants received a motivational interview,​ 6 weeks of nicotine patches and gum,​ a referral faxed to the state-sponsored QL,​ a booster call,​ and a quitline brochure. Control participants received the brochure. Smoking status was assessed by phone at 1 and 3 months,​ with confirmation via exhaled carbon monoxide testing at 3 months for those reporting abstinence. QL usage was obtained by utilization data from the QL database.RESULTSOf 778 subjects,​ 197 (25.3%) reported any use of QL services at 3 months. Participants were trichotomized: no QL usage,​ 1 call only,​ and >1 call (583,​ 99,​ and 98 participants,​ respectively). Quit rates at 3 months in these no,​ low-,​ and high-use groups were,​ respectively,​ 7.2%,​ 9.1%,​ and 15.3% (P=0.03). Participants who used the QL had a median of 28 total minutes of telephone contact.CONCLUSIONAmong low-income smokers,​ greater use of QL services is associated with higher abstinence. Whether this resulted from a direct effect of the QL,​ or greater motivation among smokers using QL services cannot be determined from these data. |
| 485 | Moreno-Coutiño,​ Ana,​ Pérez-López,​ Alejandro,​ Gallegos,​ Luis Villalobos. Predictors of retention in a multicomponent treatment for smokers. Revista de Psiquiatria Clinica. 2016;43:134.  **Abstract:** Background: There is a lack of knowledge about factors that promote or hinder retention of smokers in treatment. Objective: The aim of this study was the identification of variables that predict retention of smokers who received a multicomponent treatment against smoking. Method: Participants (n = 79) simultaneously received pharmacological and psychological treatment,​ including an intervention phase prior to the date of smoking cessation. They were evaluated periodically in their abstinence,​ depressive and anxious symptoms,​ and were randomly assigned to three treatment conditions (nicotine patch,​ bupropion or nicotine patch + bupropion). Eighteen variables were grouped into four categories (demographic,​ consumption pattern,​ mood and treatment). Data were analyzed using student’s t test and X2,​ for inclusion into a multivariate logistic regression model. Results: Results indicate that age of onset of regular tobacco consumption,​ secondary education and bupropion pharmacological treatment are significant in relation to the retention of smokers to smoking treatment. Discussion: The reported “age of onset” correlates with treatment retention (OR = 1.545,​ 95% CI = 1.175-2.032). This variable has not previously been reported in the literature,​ and taking it into account in the design of prevention and treatment for smoking could increase their success. |
| 486 | Taylor,​ Gemma M.J.,​ Taylor,​ Amy E.,​ Thomas,​ Kyla H.,​ Jones,​ Tim,​ Martin,​ Richard M.,​ Munafò,​ Marcus R.,​ Windmeijer,​ Frank,​ Davies,​ Neil M.. Effectiveness of varenicline versus nicotine replacement therapy on long-term smoking cessation in primary care: A prospective,​ cohort study of electronic medical records. The Lancet. 2016;388:107.  **Abstract:** Background No studies have investigated the eff ectiveness of varenicline versus nicotine replacement therapy (NRT) on long-term (>24 months) smoking cessation in primary care,​ or whether its effectiveness is altered by socioeconomic status. We aimed to estimate the long-term eff ectiveness of varenicline on smoking cessation,​ and to determine whether the eff ectiveness of varenicline diff ers by socioeconomic status. Methods We conducted a prospective cohort study of electronic medical records within the Clinical Practice Research Datalink (CPRD),​ using three diff erent analytical methods: multivariable logistic regression,​ propensity score matching,​ and instrumental variable analyses. Our sample comprised 220136 patients who were prescribed either NRT (n=149 526) or varenicline (70 610) between Sept 1,​ 2006,​ and Sept 30,​ 2015,​ who attended 654 general practices in the UK. Primary outcome was smoking cessation at 2 year follow-up. The outcome and covariates were defi ned with validated code lists and algorithms. Socioeconomic status was defi ned with the Index of Multiple Deprivation (IMD). We used multiple imputation to impute missing values of body-mass index (13•6%) and IMD (0•1%). Findings Patients prescribed varenicline were more likely than those prescribed NRT to successfully quit smoking after 2 years (odds ratio 1•26,​ 95% CI 1•23 to 1•29; p<0•0001); results from the propensity score matching were similar. The association persisted up to 4 years' follow-up. Instrumental variable analysis indicated that,​ for every 100 patients treated with varenicline rather than NRT,​ an additional 4•99 patients (95% CI 3•01 to 6•98,​ p<0•0001) would be expected to quit up to 2 years after treatment. On average patients had 2•6 smoking cessation prescriptions in the 2 years after first prescription. We found little evidence that the eff ectiveness of varenicline diff ered by socioeconomic status. Interpretation Patients prescribed varenicline in primary care were less likely to smoke after 2 years than were those prescribed NRT. This is the largest study to date,​ to our knowledge,​ to investigate the effectiveness of varenicline for smoking cessation when used in real-world primary care. Although our results could be subject to residual confounding,​ they are consistent with results from a network meta-analysis and large randomised controlled trial,​ and together could be used to update clinical guidelines on the use of varenicline for smoking cessation. |
| 489 | Cummins,​ Sharon E,​ Gamst,​ Anthony C,​ Brandstein,​ Kendra,​ Seymann,​ Gregory B,​ Klonoff-Cohen,​ Hillary,​ Kirby,​ Carrie A,​ Tong,​ Elisa K,​ Chaplin,​ Edward,​ Tedeschi,​ Gary J,​ Zhu,​ Shu-Hong. Helping Hospitalized Smokers: A Factorial RCT of Nicotine Patches and Counseling. American journal of preventive medicine. 2016;51:578.  **Abstract:** INTRODUCTIONMost smokers abstain from smoking during hospitalization but relapse upon discharge. This study tests the effectiveness of two proven treatments (i.e.,​ nicotine patches and telephone counseling) in helping these patients stay quit after discharge from the hospital,​ and assesses a model of hospital-quitline partnership.STUDY DESIGNThis study had a 2×2 factorial design in which participants were stratified by recruitment site and smoking rate and randomly assigned to usual care,​ nicotine patches only,​ counseling only,​ or patches plus counseling. They were evaluated at 2 and 6 months post-randomization.SETTING/PARTICIPANTSA total of 1,​270 hospitalized adult smokers were recruited from August 2011 to November 2013 from five hospitals within three healthcare systems.INTERVENTIONParticipants in the patch condition were provided 8 weeks of nicotine patches at discharge (or were mailed them post-discharge). Quitline staff started proactively calling participants in the counseling condition 3 days post-discharge to provide standard quitline counseling.MAIN OUTCOME MEASURESThe primary outcome measure was self-reported 30-day abstinence at 6 months using an intention-to-treat analysis. Data were analyzed from September 2015 to May 2016.RESULTSThe 30-day abstinence rate at 6 months was 22.8% for the nicotine patch condition and 18.3% for the no-patch condition (p=0.051). Nearly all participants (99%) in the patch condition were provided nicotine patches,​ although 36% were sent post-discharge. The abstinence rates were 20.0% and 21.1% for counseling and no counseling conditions,​ respectively (p=0.651). Fewer than half of the participants in the counseling condition (47%) received counseling (mean follow-up sessions,​ 3.6).CONCLUSIONSProvision of nicotine patches proved feasible,​ although their effectiveness in helping discharged patients stay quit was not significant. Telephone counseling was not effective,​ in large part because of low rates of engagement. Future interventions will need to be more immediate to be effective.TRIAL REGISTRATIONThis study is registered at www.clinicaltrials.gov NCT01289275. |
| 494 | Janum,​ Susanne,​ Nielsen,​ Signe T,​ Werner,​ Mads U,​ Mehlsen,​ Jesper,​ Kehlet,​ Henrik,​ Møller,​ Kirsten. Pain perception in healthy volunteers: effect of repeated exposure to experimental systemic inflammation. Innate immunity. 2016;22:546.  **Abstract:** We aimed to study the relationship between pain perception and cytokine release during systemic inflammation. We present a randomized crossover trial in healthy volunteers (n = 17) in 37 individual trials. Systemic inflammation was induced by an i.v. bolus of Escherichia coli LPS (2 ng/kg) on two separate trial days,​ with or without a nicotine patch applied 10 h previously. Pain perception at baseline,​ and 2 and 6 h after LPS was assessed by pressure algometry and tonic heat stimulation at an increasing temperature (45-48℃) during both trials. Compared with baseline,​ pain pressure threshold was reduced 2 and 6 h after LPS,​ while heat pain perception was accentuated at all testing temperatures after 2 but not 6 h. The magnitude of changes in pain perception did not correlate to cytokine release. No effect of transdermal nicotine or training status was observed. In conclusion,​ LPS administration in healthy human volunteers leads to reduction in pain pressure threshold and an increase in pain perception to heat stimuli,​ supporting a relationship between acute systemic inflammation and pain perception. |
| 498 | Vaz,​ Luis R,​ Aveyard,​ Paul,​ Cooper,​ Sue,​ Leonardi-Bee,​ Jo,​ Coleman,​ Tim. The Association Between Treatment Adherence to Nicotine Patches and Smoking Cessation in Pregnancy: A Secondary Analysis of a Randomized Controlled Trial. Nicotine & tobacco research : official journal of the Society for Research on Nicotine and Tobacco. 2016;18:1952.  **Abstract:** INTRODUCTIONIn nonpregnant "quitters,​" adherence to nicotine replacement therapy (NRT) increases smoking cessation. We investigated relationships between adherence to placebo or NRT patches and cessation in pregnancy,​ including an assessment of reverse causation and whether any adherence: cessation relationship is moderated when using nicotine or placebo patches.METHODSUsing data from 1050 pregnant trial participants,​ regression models investigated associations between maternal characteristics,​ adherence and smoking cessation.RESULTSAdherence during the first month was associated with lower baseline cotinine concentrations (β -0.08,​ 95% confidence interval [CI] -0.15 to -0.01) and randomization to NRT (β 2.59,​ 95% CI 1.50 to 3.68). Adherence during both treatment months was associated with being randomized to NRT (β 0.51,​ 95% CI 0.29 to 0.72) and inversely associated with higher nicotine dependence. Adherence with either NRT or placebo was associated with cessation at 1 month (odds ratio [OR] 1.11,​ 95% CI 1.08 to 1.13) and delivery (OR 1.06,​ 95% CI 1.03 to 1.09),​ but no such association was observed in the subgroup where reverse causation was not possible. Amongst all women,​ greater adherence to nicotine patches was associated with increased cessation (OR 2.47,​ 95% CI 1.32 to 4.63) but greater adherence to placebo was not (OR 0.98,​ 95% CI: 0.44 to 2.18).CONCLUSIONWomen who were more adherent to NRT were more likely to achieve abstinence; more nicotine dependent women probably showed lower adherence to NRT because they relapsed to smoking more quickly. The interaction between nicotine-containing patches and adherence for cessation suggests that the association between adherence with nicotine patches and cessation may be partly causal.IMPLICATIONSThis study used placebo randomized controlled trial data to investigate both associations between women's characteristics and adherence to NRT patch treatment,​ and the relationship between adherence to NRT patch treatment and odds of cessation in pregnant quitters. Greater adherence was seen with NRT patches,​ and greater adherence with NRT patches increased the odds of smoking cessation. A likely explanation for findings is that NRT patches,​ if used sufficiently,​ may be effective for at least some pregnant women who try to stop smoking. Trials testing interventions which encourage women's adherence to higher dose NRT are indicated. |
| 507 | Kushnir,​ Vladyslav,​ Sproule,​ Beth A,​ Zawertailo,​ Laurie,​ Selby,​ Peter,​ Tyndale,​ Rachel F,​ Leatherdale,​ Scott T,​ Cunningham,​ John A. Impact of self-reported lifetime depression or anxiety on effectiveness of mass distribution of nicotine patches. Tobacco control. 2016;26:526.  **Abstract:** BACKGROUNDLarge-scale public health initiatives providing free nicotine replacement therapy have been shown to increase smoking cessation rates; however,​ their effectiveness among the highly prevalent population of smokers with depression and anxiety disorders has not been explored. The aim of this study was to investigate the influence of lifetime history of depression or anxiety on smoking cessation success following the free distribution of nicotine patches.METHODIn the context of a randomised controlled trial,​ a secondary analysis was conducted on 1000 adult regular smokers randomised to be mailed a 5-week supply of nicotine patches or to a no intervention control group. Participants were divided into subgroups based on the presence of self-reported lifetime diagnosis of depression and anxiety.RESULTSIrrespective of self-reported lifetime history of depression or anxiety,​ odds of self-reported cessation at 6 months were significantly greater among groups receiving nicotine patches compared to no intervention control (no history of depression or anxiety: OR 2.20; 95% CI 1.05 to 4.63; history of depression or anxiety present: OR 3.90; 95% CI 1.28 to 11.88). Among nicotine patch recipients only,​ quit outcomes did not differ between those with and without self-reported lifetime depression or anxiety in models unadjusted and adjusted for differences in demographic and smoking characteristics.CONCLUSIONSThe mass distribution of free nicotine patches (without behavioural support) is effective among smokers with or without lifetime history of depression or anxiety alike,​ providing further support for the adoption of similar initiatives as a means of promoting tobacco cessation on a population level.TRIAL REGISTRATION NUMBERNCT01429129,​ Post-results. |
| 511 | Dennis,​ Paul A,​ Kimbrel,​ Nathan A,​ Dedert,​ Eric A,​ Beckham,​ Jean C,​ Dennis,​ Michelle F,​ Calhoun,​ Patrick S. Supplemental nicotine preloading for smoking cessation in posttraumatic stress disorder: Results from a randomized controlled trial. Addictive behaviors. 2016;59:24.  **Abstract:** BACKGROUNDIndividuals with posttraumatic stress disorder (PTSD) are more likely to smoke and more likely to relapse following a quit attempt than individuals without PTSD. Thus,​ there is a significant need to study promising interventions that might improve quit rates for smokers with PTSD. One such intervention,​ supplemental nicotine patch-preloading,​ entails the use of nicotine replacement therapy prior to quitting. Objective The objective of this study was to conduct a randomized controlled trial of the efficacy of supplemental nicotine patch-preloading among smokers with PTSD. We hypothesized that,​ relative to participants in the placebo condition,​ participants in the nicotine patch-preloading condition would: (1) smoke less and experience reduced craving for cigarettes during the nicotine patch-preloading phase; (2) experience less smoking-associated relief from PTSD symptoms and negative affect during the preloading phase; and (3) exhibit greater latency to lapse,​ and higher short- and long-term abstinence rates.METHODSSixty-three smokers with PTSD were randomized to either nicotine or placebo patch for three weeks prior to their quit date. Ecological momentary assessment was used to assess craving,​ smoking,​ PTSD symptoms,​ and negative affect during the preloading period.RESULTSNicotine patch-preloading failed to reduce smoking or craving during the preloading phase,​ nor was it associated with less smoking-associated relief from PTSD symptoms and negative affect. Moreover,​ no differences were observed between the treatment conditions for time to lapse,​ 6-week abstinence,​ or 6-month abstinence.CONCLUSIONSThe findings from the present research suggest that supplemental nicotine patch-preloading is unlikely to substantially enhance quit rates among smokers with PTSD. |
| 514 | Ikonomidis,​ I.,​ Kourea,​ K.,​ Vlastos,​ D.,​ Marinou,​ M.,​ Vlachos,​ S.,​ Varoudi,​ M.,​ Tympas,​ K.,​ Liarakos,​ N.,​ Andreadou,​ I.,​ Triantafyllidi,​ H.,​ Pavlidis,​ G.,​ Tsougos,​ E.,​ Lekakis,​ J.. Varenicline vs. Nicotine replacement therapy: A prospective study of changes in arterial stiffness,​ endothelial glycocalyx and oxidative stress in smokers during 1 year follow-up. European Heart Journal. 2016;37:553.  **Abstract:** Background: Smoking serves as a major cardiovascular disease risk factor. We studied the changes in arterial stiffness,​ glycocalyx integrity,​ and oxidative stress status in adults who attended a smoking cessation program. Methods: 166 current smokers (mean age 50 years) with no diagnosed cardiovascular disease participated in the study. We measured a) the aortic PWV and augmentation index by Arteriograph and Complior; b) the perfusion boundary region of the sublingual arterial microvessels using Sideview,​ Darkfield imaging (Microscan,​ Glycocheck) a marker of endothelial glycocalyx thickness; c) the exhaled CO level (parts per million -ppm) as a surrogate marker of smoking status; and d) the malondialdehyde (MDA) and protein carbonyls (PC) plasma levels,​ as biomarkers of oxidative stress at baseline,​ and after 3 and 12 months of intervention. Patients were randomized to Varenicline (n=72,​ 44%) or nicotine replacement therapy (NRT; n=94,​ 56%) Results: CO levels were significantly decreased during the entire intervention time span (median CO: 30 with IQR=50-25,​ 5 with IQR=3-9 and 6 with IQR= 3- 13,​ at baseline,​ 3 and 12 months respectively) with varenicline resulting a greater decrease at 3 months of treatment compared to NRT (p=0.04). Oxidative stress was progressively attenuated (median MDA: 0.81 with IQR 0.45- 1.33 and 0.63 with IQR 0.47- 2.1 at baseline and 12 months respectively; median PC: 0.102 with IQR 0.073- 0.133 and 0.071 with IQR 0.065- 0.130 at baseline and 12 months respectively p<0.05). At 12 months,​ PWV was also reduced from 10±1 to 8.5±1.2 m/sec,​ p<0.05. The reduction of MDA and PC was significantly associated with the concomitant reduction of PWV (p=0.04 and 0.008 respectively). At 3 months of intervention,​ glycocalyx integrity showed a greater improvement in the varenicline than in NRT arm (2.1±0.5 vs. 1.9±0.5,​ p=0.04 for interaction of the type of treatment) probably due to the greater CO reduction (p=0.04) and was moderately correlated with CO (rho= 0.33,​ p=0.01),​ PWV (rho=0.42,​ p=0.001),​ and augmentation index (rho= 0.30,​ p=0.01). Conclusions: A 12 month smoking cessation program,​ using either varenicline or NRT,​ resulted in a decrease of CO levels,​ and oxidative stress during 1-year follow-up. These were associated with endothelial integrity restoration and improved arterial elastic properties though these effects were evident earlier in the varenicline compared to the nicotine replacement therapy. |
| 518 | Geiser,​ Christian,​ Griffin,​ Daniel,​ Shiffman,​ Saul. Using Multigroup-Multiphase Latent State-Trait Models to Study Treatment-Induced Changes in Intra-Individual State Variability: An Application to Smokers' Affect. Frontiers in psychology. 2016;7:1043.  **Abstract:** Sometimes,​ researchers are interested in whether an intervention,​ experimental manipulation,​ or other treatment causes changes in intra-individual state variability. The authors show how multigroup-multiphase latent state-trait (MG-MP-LST) models can be used to examine treatment effects with regard to both mean differences and differences in state variability. The approach is illustrated based on a randomized controlled trial in which N = 338 smokers were randomly assigned to nicotine replacement therapy (NRT) vs. placebo prior to quitting smoking. We found that post quitting,​ smokers in both the NRT and placebo group had significantly reduced intra-individual affect state variability with respect to the affect items calm and content relative to the pre-quitting phase. This reduction in state variability did not differ between the NRT and placebo groups,​ indicating that quitting smoking may lead to a stabilization of individuals' affect states regardless of whether or not individuals receive NRT. |
| 519 | Bernstein,​ Steven L,​ Rosner,​ June,​ Toll,​ Benjamin. A Multicomponent Intervention Including Texting to Promote Tobacco Abstinence in Emergency Department Smokers: A Pilot Study. Academic emergency medicine : official journal of the Society for Academic Emergency Medicine. 2016;23:803.  **Abstract:** BACKGROUNDEmergency department (ED) patients commonly smoke. Current treatment approaches use motivational interviewing,​ which is effective,​ but resource-intensive. Mobile health approaches may be more feasible and generalizable.OBJECTIVEThe objective was to assess the feasibility of an ED-initiated program of tobacco dependence treatment that employs text messaging.METHODSSmokers age 18 or older were randomized to intervention or control arms. Control subjects received a brochure describing the state smokers' quitline. Intervention subjects received the brochure,​ 4 weeks of nicotine patches and gum (with the initial dose administered in the ED),​ a referral to the quitline,​ and enrollment in SmokefreeTXT,​ a free SMS-messaging service. SmokefreeTXT delivered 28 days of messages,​ two to five messages/day. Some messages ask subjects to provide data on mood or craving. Follow-up was conducted by phone call.RESULTSSixty subjects were enrolled in May 2014. Of all subjects,​ 33 (55%) were nonwhite; 78% were insured by Medicaid. All intervention subjects used the texting program,​ with 24/30 (80%) using the program for all 28 days. At 1 month,​ 14/30 subjects (47%) in the intervention arm reported abstinence versus 3/30 (10%) in the control arm (p = 0.003). At 3 months,​ the abstinence rates in the intervention and control arms were,​ respectively,​ 9/30 (30%) and 4/30 (13%; p = 0.21). Subjects responding to more assessments of mood or craving were more likely to report abstinence at 1 month.CONCLUSIONA texting program,​ combined with pharmacotherapy and a quitline referral,​ is feasible and may promote tobacco abstinence in ED smokers. A larger trial is planned to assess these results. |
| 521 | Tuisku,​ Anna,​ Salmela,​ Merita,​ Nieminen,​ Pentti,​ Toljamo,​ Tuula. Varenicline and Nicotine Patch Therapies in Young Adults Motivated to Quit Smoking: A Randomized,​ Placebo-controlled,​ Prospective Study. Basic & clinical pharmacology & toxicology. 2016;119:78.  **Abstract:** This study compares the nicotine patch to placebo in young adult light smokers,​ and the nicotine patch to varenicline in heavy smokers. Volunteer daily smokers were recruited into a randomized,​ placebo-controlled study via community media,​ colleges and the army (aged 18-26 years). Those subjects with light tobacco dependence were randomized to (i) placebo patch (n = 86) and (ii) nicotine patch 10 mg/16 hr for 8 weeks (n = 94),​ and those with stronger dependence to (iii) nicotine patch 15 mg/16 hr for 8 weeks (n = 51) and (iv) varenicline for 12 weeks (n = 60). The primary outcome variable was self-reported smoking abstinence at week 12. Secondary outcome variables were self-reported smoking abstinence at weeks 4 and 26,​ and self-reported abstinence verified by saliva cotinine level at week 12. The prevalence of self-reported smoking abstinence did not differ statistically significantly in light smokers during the follow-up (week 4: 19.8% for placebo patch and 26.6% for nicotine patch 10 mg/16 hr; week 12: 17.4% versus 23.4%; week 26: 15.1% versus 20.2%),​ but the groups of heavy smokers differed significantly for 12 weeks (week 4: 19.6% for nicotine patch 15 mg/16 hr and 73.3% for varenicline,​ p < 0.001; week 12: 15.7% versus 36.7%,​ p = 0.018). This statistically significant difference did not endure for the entire follow-up (week 26: 9.8% versus 18.3%,​ p = 0.280). However,​ saliva cotinine verified abstinence at week 12 did not support self-reported abstinence. Varenicline may be more effective than the nicotine patch as a smoking cessation pharmacotherapy among young adult heavy smokers in the short-term. |
| 523 | Esmat,​ Ibrahim M.,​ Kassim,​ Dina Y.. Comparative study between transdermal nicotine and melatonin patches on postoperative pain relief after laparoscopic cholecystectomy,​ a double-blind,​ placebo-controlled trial. Egyptian Journal of Anaesthesia. 2016;32:299.  **Abstract:** Background This study evaluated the efficacy of transdermal nicotine (TDN) delivery system (15 mg/16 h) or transdermal melatonin (TDM) delivery system (7 mg) 2 h preoperatively for acute postoperative pain after laparoscopic cholecystectomy compared to placebo group (C). Methods Sixty female non-smoker patients,​ aged 18–50 years and ASA I and II undergoing elective laparoscopic cholecystectomy under general anesthesia were included in this randomized controlled double-blind study. Patients were randomly divided into 3 groups 20 each,​ and C group patients received transdermal placebo patch,​ TDN group (15 mg/16 h) and TDM group (7 mg/8 h). Assessment of postoperative pain,​ sedation,​ hemodynamic variables such as HR and MAP,​ postoperative monitoring of arterial SpO2 and side effects (e.g. nausea,​ vomiting,​ pruritus,​ respiratory depression and hemodynamic instability) were done 30 min,​ 1,​ 2,​ 6 and 12 h postoperatively. Postoperative Patient's and Surgeons’ satisfaction,​ Intraoperative bleeding and plasma cortisol (μg/dl) 2 h postoperatively were also assessed. Results There was a significant reduction in the VAS score,​ total pethidine requirements (mg) and significantly higher patient's satisfaction in TDN and TDM groups when compared with the C group postoperatively. The sedation score and surgeons’ satisfaction were significantly higher associated with a significant decrease in MAP and Intraoperative bleeding in TDM group compared to C and TDN groups postoperatively. Significant nausea and vomiting in TDN group and significant sedation in TDM group were recorded. Conclusion The use of preoperative TDN (15 mg/16 h) or TDM (7 mg/8 h) was an effective and a safe adjuvant for acute pain after surgery. |
| 524 | Ben Taleb,​ Ziyad,​ Ward,​ Kenneth D,​ Asfar,​ Taghrid,​ Jaber,​ Rana,​ Auf,​ Rehab,​ Maziak,​ Wasim. Predictors of nicotine withdrawal symptoms: findings from the first randomized smoking cessation trial in a low-income country setting. International journal of public health. 2016;61:701.  **Abstract:** OBJECTIVESTo identify predictors of nicotine withdrawal symptoms among smokers who participated in a randomized cessation trial in a low-income country.METHODSWe analyzed data from 269 smokers who participated in a randomized,​ placebo-controlled smoking cessation trial conducted in primary healthcare in Aleppo,​ Syria. All participants received behavioral counseling and were randomized to receive either 6 weeks of nicotine or placebo patch and were followed for one year.RESULTSThroughout the study,​ lower total withdrawal score was associated with greater education (p = 0.044),​ older age of smoking initiation (p = 0.017),​ lower nicotine dependence (p = 0.024),​ higher confidence in ability to quit (p = 0.020),​ lower reported depression (p < 0.001),​ higher adherence to patch (p = 0.026),​ belief of receiving nicotine patches rather than placebo (p = 0.011),​ and waterpipe use (p = 0.047).CONCLUSIONSLower nicotine dependence,​ greater educational attainment,​ higher confidence in ability to quit and waterpipe use predict lower withdrawal severity. Waterpipe smoking may serve as a barrier to smoking cessation efforts in countries where its use is highly prevalent. Further,​ expectancies about the effects of pharmacotherapy appear to mediate the experience of nicotine withdrawal. |
| 525 | Anthenelli,​ Robert M,​ Benowitz,​ Neal L,​ West,​ Robert,​ St Aubin,​ Lisa,​ McRae,​ Thomas,​ Lawrence,​ David,​ Ascher,​ John,​ Russ,​ Cristina,​ Krishen,​ Alok,​ Evins,​ A Eden. Neuropsychiatric safety and efficacy of varenicline,​ bupropion,​ and nicotine patch in smokers with and without psychiatric disorders (EAGLES): a double-blind,​ randomised,​ placebo-controlled clinical trial. Lancet (London,​ England). 2016;387:2507.  **Abstract:** BACKGROUNDSubstantial concerns have been raised about the neuropsychiatric safety of the smoking cessation medications varenicline and bupropion. Their efficacy relative to nicotine patch largely relies on indirect comparisons,​ and there is limited information on safety and efficacy in smokers with psychiatric disorders. We compared the relative neuropsychiatric safety risk and efficacy of varenicline and bupropion with nicotine patch and placebo in smokers with and without psychiatric disorders.METHODSWe did a randomised,​ double-blind,​ triple-dummy,​ placebo-controlled and active-controlled (nicotine patch; 21 mg per day with taper) trial of varenicline (1 mg twice a day) and bupropion (150 mg twice a day) for 12 weeks with 12-week non-treatment follow-up done at 140 centres (clinical trial centres,​ academic centres,​ and outpatient clinics) in 16 countries between Nov 30,​ 2011,​ and Jan 13,​ 2015. Participants were motivated-to-quit smokers with and without psychiatric disorders who received brief cessation counselling at each visit. Randomisation was computer generated (1:1:1:1 ratio). Participants,​ investigators,​ and research personnel were masked to treatment assignments. The primary endpoint was the incidence of a composite measure of moderate and severe neuropsychiatric adverse events. The main efficacy endpoint was biochemically confirmed continuous abstinence for weeks 9-12. All participants randomly assigned were included in the efficacy analysis and those who received treatment were included in the safety analysis. The trial is registered at ClinicalTrials.gov (number NCT01456936) and is now closed.FINDINGS8144 participants were randomly assigned,​ 4116 to the psychiatric cohort (4074 included in the safety analysis) and 4028 to the non-psychiatric cohort (3984 included in the safety analysis). In the non-psychiatric cohort,​ 13 (1·3%) of 990 participants reported moderate and severe neuropsychiatric adverse events in the varenicline group,​ 22 (2·2%) of 989 in the bupropion group,​ 25 (2·5%) of 1006 in the nicotine patch group,​ and 24 (2·4%) of 999 in the placebo group. The varenicline-placebo and bupropion-placebo risk differences (RDs) for moderate and severe neuropsychiatric adverse events were -1·28 (95% CI -2·40 to -0·15) and -0·08 (-1·37 to 1·21),​ respectively; the RDs for comparisons with nicotine patch were -1·07 (-2·21 to 0·08) and 0·13 (-1·19 to 1·45),​ respectively. In the psychiatric cohort,​ moderate and severe neuropsychiatric adverse events were reported in 67 (6·5%) of 1026 participants in the varenicline group,​ 68 (6·7%) of 1017 in the bupropion group,​ 53 (5·2%) of 1016 in the nicotine patch group,​ and 50 (4·9%) of 1015 in the placebo group. The varenicline-placebo and bupropion-placebo RDs were 1·59 (95% CI -0·42 to 3·59) and 1·78 (-0·24 to 3·81),​ respectively; the RDs versus nicotine patch were 1·22 (-0·81 to 3·25) and 1·42 (-0·63 to 3·46),​ respectively. Varenicline-treated participants achieved higher abstinence rates than those on placebo (odds ratio [OR] 3·61,​ 95% CI 3·07 to 4·24),​ nicotine patch (1·68,​ 1·46 to 1·93),​ and bupropion (1·75,​ 1·52 to 2·01). Those on bupropion and nicotine patch achieved higher abstinence rates than those on placebo (OR 2·07 [1·75 to 2·45] and 2·15 [1·82 to 2·54],​ respectively). Across cohorts,​ the most frequent adverse events by treatment group were nausea (varenicline,​ 25% [511 of 2016 participants]),​ insomnia (bupropion,​ 12% [245 of 2006 participants]),​ abnormal dreams (nicotine patch,​ 12% [251 of 2022 participants]),​ and headache (placebo,​ 10% [199 of 2014 participants]). Efficacy treatment comparison did not differ by cohort.INTERPRETATIONThe study did not show a significant increase in neuropsychiatric adverse events attributable to varenicline or bupropion relative to nicotine patch or placebo. Varenicline was more effective than placebo,​ nicotine patch,​ and bupropion in helping smokers achieve abstinence,​ whereas bupropion and nicotine patch were more effective than placebo.FUNDINGPfizer and GlaxoSmithKline. |
| 527 | Tulloch,​ Heather E,​ Pipe,​ Andrew L,​ Els,​ Charl,​ Clyde,​ Matthew J,​ Reid,​ Robert D. Flexible,​ dual-form nicotine replacement therapy or varenicline in comparison with nicotine patch for smoking cessation: a randomized controlled trial. BMC medicine. 2016;14:80.  **Abstract:** BACKGROUNDExtended use of combined pharmacotherapies to treat tobacco dependence may increase smoking abstinence; few studies have examined their effectiveness. The objective of this study was to evaluate smoking abstinence with standard nicotine patch (NRT),​ extended use of combined formulations of nicotine replacement therapy (NRT+),​ or varenicline (VR).METHODSA total of 737 smokers,​ including those with medical and psychiatric comorbidities,​ were randomly assigned to one of the above three treatment conditions. The NRT group received 10 weeks of patches (21 mg daily maximum); the NRT+ group received patches (35 mg daily maximum) and gum or inhaler for up to 22 weeks; and the VR group received 1 mg twice daily for up to 24 weeks (22 weeks post target quit date). All participants also received six standardized 15-minute smoking cessation counseling sessions by nurses experienced in tobacco dependence treatment. The primary outcome was carbon monoxide-confirmed continuous abstinence rates (CAR) from weeks 5-52. Secondary outcomes were: CAR from weeks 5-10 and 5-22,​ and carbon monoxide-confirmed 7-day point prevalence (7PP) at weeks 10,​ 22,​ and 52. Adjusted and unadjusted logistic regression analyses were conducted using intention-to-treat procedures.RESULTSThe CARs for weeks 5-52 were 10.0 %,​ 12.4 %,​ and 15.3 % in the NRT,​ NRT+,​ and VR groups,​ respectively; no group differences were observed. Results with 7PP showed that VR was superior to NRT at week 52 (odds ratio (OR),​ 1.84; 97.5 % Confidence Interval (CI),​ 1.04-3.26) in the adjusted intention-to-treat analysis. Those in the VR group had higher CAR at weeks 5-22 (OR,​ 2.01; CI,​ 1.20-3.36) than those in the NRT group. Results with 7PP revealed that both NRT+ (OR,​ 1.72; CI,​ 1.04-2.85) and VR (OR,​ 1.96; CI,​ 1.20-3.23) were more effective than NRT at 22 weeks. As compared to NRT monotherapy,​ NRT+ and VR produced significant increases in CAR for weeks 5-10 (OR,​ 1.52; CI,​ 1.00-2.30 and OR,​ 1.58; CI,​ 1.04-2.39,​ respectively); results were similar,​ but somewhat stronger,​ when 7PP was used at 10 weeks (OR,​ 1.57; CI,​ 1.03-2.41 and OR,​ 1.79; CI,​ 1.17-2.73,​ respectively). All medications were well tolerated,​ but participants in the VR group experienced more fatigue,​ digestive symptoms (e.g.,​ nausea,​ diarrhea),​ and sleep-related concerns (e.g.,​ abnormal dreams,​ insomnia),​ but less dermatologic symptoms than those in the NRT or NRT+ groups. The frequency of serious adverse events did not differ between groups.CONCLUSIONSFlexible and combination NRT and varenicline enhance success in the early phases of quitting. Varenicline improves abstinence in the medium term; however,​ there is no clear evidence that either varenicline or flexible,​ dual-form NRT increase quit rates in the long-term when compared to NRT monotherapy.TRIAL REGISTRATIONClinicalTrials.gov Identifier: NCT01623505 ; Retrospectively registered on July 13,​ 2011. |
| 528 | Rabin,​ Rachel A,​ Ashare,​ Rebecca L,​ Schnoll,​ Robert A,​ Cinciripini,​ Paul M,​ Hawk,​ Larry W,​ Jr,​ Lerman,​ Caryn,​ Tyndale,​ Rachel F,​ George,​ Tony P. Does cannabis use moderate smoking cessation outcomes in treatment-seeking tobacco smokers? Analysis from a large multi-center trial. The American journal on addictions. 2016;25:291.  **Abstract:** BACKGROUND AND OBJECTIVETobacco and cannabis are frequently used in combination and cannabis co-use may lead to poor tobacco cessation outcomes. Therefore,​ it is important to explore if cannabis co-use is associated with a reduced likelihood of achieving successful tobacco abstinence among treatment-seeking tobacco smokers. The present study examined whether current cannabis use moderated tobacco cessation outcomes after 12 weeks of pharmacological treatment (varenicline vs. nicotine patch vs. placebo) with adjunctive behavioral counseling.METHODSTreatment-seeking tobacco smokers (N = 1,​246) were enrolled in an intent-to-treat study,​ of which 220 were current cannabis users. Individuals were randomly assigned to 12 weeks of placebo (placebo pill plus placebo patch),​ nicotine patch (active patch plus placebo pill),​ or varenicline (active pill plus placebo patch),​ plus behavioral counseling. The primary endpoint was biochemically verified 7-day point prevalence abstinence at the end of treatment.RESULTSControlling for rate of nicotine metabolism,​ treatment arm,​ age,​ sex,​ alcohol,​ and level of nicotine dependence,​ cannabis users were as successful at achieving biochemically verified 7-day point prevalence abstinence compared to tobacco-only smokers.CONCLUSIONS AND SCIENTIFIC SIGNIFICANCEFindings suggest that cannabis use does not hinder the ability to quit tobacco smoking. Future tobacco cessation studies should employ prospective,​ longitudinal designs investigating cannabis co-use over time and at different severity levels. (Am J Addict 2016;25:291-296). |
| 529 | Prapavessis,​ Harry,​ De Jesus,​ Stefanie,​ Fitzgeorge,​ Lindsay,​ Faulkner,​ Guy,​ Maddison,​ Ralph,​ Batten,​ Sandra. Exercise to Enhance Smoking Cessation: the Getting Physical on Cigarette Randomized Control Trial. Annals of behavioral medicine : a publication of the Society of Behavioral Medicine. 2016;50:358.  **Abstract:** BACKGROUNDExercise has been proposed as a useful smoking cessation aid.PURPOSEThe purpose of the present study is to determine the effect of an exercise-aided smoking cessation intervention program,​ with built-in maintenance components,​ on post-intervention 14-,​ 26- and 56-week cessation rates.METHODFemale cigarette smokers (n = 413) participating in a supervised exercise and nicotine replacement therapy (NRT) smoking cessation program were randomized to one of four conditions: exercise + smoking cessation maintenance,​ exercise maintenance + contact control,​ smoking cessation maintenance + contact control or contact control. The primary outcome was continuous smoking abstinence.RESULTSAbstinence differences were found between the exercise and equal contact non-exercise maintenance groups at weeks 14 (57 vs 43 %),​ 26 (27 vs 21 %) and 56 (26 vs 23.5 %),​ respectively. Only the week 14 difference approached significance,​ p = 0.08.CONCLUSIONSAn exercise-aided NRT smoking cessation program with built-in maintenance components enhances post-intervention cessation rates at week 14 but not at weeks 26 and 56. |
| 532 | Schoenberg,​ Nancy E,​ Studts,​ Christina R,​ Shelton,​ Brent J,​ Liu,​ Meng,​ Clayton,​ Richard,​ Bispo,​ Jordan Baeker,​ Fields,​ Nell,​ Dignan,​ Mark,​ Cooper,​ Thomas. A randomized controlled trial of a faith-placed,​ lay health advisor delivered smoking cessation intervention for rural residents. Preventive medicine reports. 2016;3:317.  **Abstract:** INTRODUCTIONRural US residents smoke at higher rates than urban or suburban residents. We report results from a community-based smoking cessation intervention in Appalachian Kentucky.STUDY DESIGNSingle-blind,​ group-randomized trial with outcome measurements at baseline,​ 17 weeks and 43 weeks.SETTING/PARTICIPANTSThis faith-placed CBPR project was located in six counties of rural Appalachian Kentucky. A total of 590 individual participants clustered in 28 churches were enrolled in the study.INTERVENTIONLocal lay health advisors delivered the 12-week Cooper/Clayton Method to Stop Smoking program,​ leveraging sociocultural factors to improve the cultural salience of the program for Appalachian smokers. Participants met with an interventionist for one 90 min group session once per week incorporating didactic information,​ group discussion,​ and nicotine replacement therapy.MAIN OUTCOME MEASURESThe primary outcome was self-reported smoking status. Secondary outcomes included Fagerström nicotine dependence,​ self-efficacy,​ and decisional balance.RESULTSWith post-intervention data from 92% of participants,​ those in intervention group churches (N = 383) had 13.6 times higher odds of reporting quitting smoking one month post-intervention than participants in attention control group churches (N = 154,​ p < 0.0001). In addition,​ although only 3.2% of attention control group participants reported quitting during the control period,​ 15.4% of attention control participants reported quitting smoking after receiving the intervention. A significant dose effect of the 12-session Cooper/Clayton Method was detected: for each additional session completed,​ the odds of quitting smoking increased by 26%.CONCLUSIONSThe Cooper/Clayton Method,​ delivered in rural Appalachian churches by lay health advisors,​ has strong potential to reduce smoking rates and improve individuals' health. |
| 537 | Arendt Nielsen,​ Thomas,​ Nielsen,​ Bruno Provstgaard,​ Wang,​ Kelun,​ Arendt-Nielsen,​ Lars,​ Boudreau,​ Shellie A. Psychophysical and Vasomotor Responses of the Oral Tissues: A Nicotine Dose-Response and Menthol Interaction Study. Nicotine & tobacco research : official journal of the Society for Research on Nicotine and Tobacco. 2016;18:596.  **Abstract:** INTRODUCTIONThis study implemented an intra-oral test-platform to assess the sensory,​ psychophysical,​ and vasomotor responses to nicotine and menthol,​ alone or in combination.METHODSTwo double-blinded,​ placebo-controlled,​ randomized,​ cross-over studies,​ including healthy nonsmoking participants were performed. Study I: A dose-response relationship (N = 20) between 0,​ 2,​ and 4 mg nicotine gum. Study II: An interaction response (N = 22) to 30 mg menthol and 4 mg nicotine alone or in combination. Heart rate,​ blood pressure,​ tactile and thermosensory thresholds,​ intra-oral blood flow and temperature,​ pain/irritation intensities/locations,​ McGill Pain Questionnaire,​ and taste experience were assessed before,​ during or after the completion of a standardized chewing regime.RESULTSA dose-response elevation in heart rate was attenuated when nicotine was combined with menthol. Blood flow,​ temperature,​ and warm-detection thresholds,​ as assessed on the tongue,​ similarly increased for all gums. Pain intensity and taste experiences were similar between nicotine doses. Nicotine attenuated the sweet,​ cooling,​ and freshening sensation of menthol. Within the first 4 minutes,​ menthol reduced the intensity but not the area of nicotine-induced pain and irritation. The 4-mg nicotine dose led to a continued increase in the intensity and area of irritation in the throat post-chewing. Moreover,​ one-half of participants responded to menthol as an irritant,​ and these individuals demonstrated larger areas of nicotine-induced irritation in the throat post-chewing.CONCLUSIONSThe intra-oral test platform provides a basis to optimize the assessment of nicotine-related taste and sensory experiences and can be used in future studies for profiling nicotine gum. |
| 539 | McRobbie,​ Hayden,​ Przulj,​ Dunja,​ Smith,​ Katherine Myers,​ Cornwall,​ Danielle. Complementing the Standard Multicomponent Treatment for Smokers With Denicotinized Cigarettes: A Randomized Trial. Nicotine & tobacco research : official journal of the Society for Research on Nicotine and Tobacco. 2016;18:1134.  **Abstract:** INTRODUCTIONStandard treatments (STs) for smoking cessation typically combine pharmacotherapy and behavioral support but do not address the sensory and behavioral aspects of smoking which may play a role in maintaining smoking behavior. Replacing such sensations temporarily after cessation may enhance treatment efficacy. We hypothesized that denicotinized cigarettes (DNCs),​ which have a very low nicotine content but provide these sensory and behavioral stimuli,​ could help alleviate urges to smoke and tobacco withdrawal symptoms and in turn enhance the efficacy of ST.METHODSTwo hundred smokers seeking treatment received nine weekly behavioral support sessions and pharmacotherapy (100 used varenicline,​ 100 used nicotine replacement therapy). They were randomized on the target quit day to receive 280 DNCs (used ad libitum over 2 weeks in addition to ST) or ST alone.RESULTSUrge-to-smoke frequency (2.61 vs. 2.96,​ P = .03) but not strength (2.85 vs. 3.10,​ P = .20) in the first week of abstinence was significantly lower in DNC users versus ST alone. There were no differences in composite withdrawal scores between groups. Abstinence was significantly higher among DNC users versus ST alone at 1 (OR = 2.07; 95% CI: 1.63% to 3.70%) and 4 weeks (OR = 1.83; 95% CI: 1.05% to 3.21%),​ but not at 12 weeks (OR = 1.42; 95% CI: 0.79% to 2.55%). DNC use was a significant predictor of abstinence at 1 and 4 weeks (OR = 2.63; 95% CI: 1.40% to 4.93% and OR = 2.38; 95% CI: 1.26% to 4.46%),​ but not at 12 weeks.CONCLUSIONSAdding DNCs to ST has the potential to assist smokers early in their quit attempt,​ but research is needed to determine how best to utilize DNCs in treatment. |
| 541 | Fu,​ Steven S,​ van Ryn,​ Michelle,​ Nelson,​ David,​ Burgess,​ Diana J,​ Thomas,​ Janet L,​ Saul,​ Jessie,​ Clothier,​ Barbara,​ Nyman,​ John A,​ Hammett,​ Patrick,​ Joseph,​ Anne M. Proactive tobacco treatment offering free nicotine replacement therapy and telephone counselling for socioeconomically disadvantaged smokers: a randomised clinical trial. Thorax. 2016;71:446.  **Abstract:** BACKGROUNDEvidenced-based tobacco cessation treatments are underused,​ especially by socioeconomically disadvantaged smokers. This contributes to widening socioeconomic disparities in tobacco-related morbidity and mortality.METHODSThe Offering Proactive Treatment Intervention trial tested the effects of a proactive outreach tobacco treatment intervention on population-level smoking abstinence and tobacco treatment use among a population-based sample of socioeconomically disadvantaged smokers. Current smokers (n=2406),​ regardless of interest in quitting,​ who were enrolled in the Minnesota Health Care Programs,​ the state's publicly funded healthcare programmes for low-income populations,​ were randomly assigned to proactive outreach or usual care. The intervention comprised proactive outreach (tailored mailings and telephone calls) and free cessation treatment (nicotine replacement therapy and intensive,​ telephone counselling). Usual care comprised access to a primary care physician,​ insurance coverage of Food and Drug Administration-approved smoking cessation medications,​ and the state's telephone quitline. The primary outcome was self-reported 6-month prolonged smoking abstinence at 1 year and was assessed by follow-up survey.FINDINGSThe proactive intervention group had a higher prolonged abstinence rate at 1 year than usual care (16.5% vs 12.1%,​ OR 1.47,​ 95% CI 1.12 to 1.93). The effect of the proactive intervention on prolonged abstinence persisted in selection models accounting for non-response. In analysis of secondary outcomes,​ use of evidence-based tobacco cessation treatments were significantly greater among proactive outreach participants compared with usual care,​ particularly combination counselling and medications (17.4% vs 3.6%,​ OR 5.69,​ 95% CI 3.85 to 8.40).INTERPRETATIONPopulation-based proactive tobacco treatment increases engagement in evidence-based treatment and is effective in long-term smoking cessation among socioeconomically disadvantaged smokers. Findings suggest that dissemination of population-based proactive treatment approaches is an effective strategy to reduce the prevalence of smoking and socioeconomic disparities in tobacco use.TRIAL REGISTRATION NUMBERNCT01123967. |
| 544 | Eugen-Olsen,​ Jesper,​ Ladelund,​ Steen,​ Sørensen,​ Lars Tue. Plasma suPAR is lowered by smoking cessation: a randomized controlled study. European journal of clinical investigation. 2016;46:305.  **Abstract:** BACKGROUNDSoluble urokinase plasminogen activator receptor (suPAR) is a stable inflammatory biomarker. In patients,​ suPAR is a marker of disease presence,​ severity and prognosis. In the general population,​ suPAR is predictive of disease development,​ such as diabetes and cardiovascular disease and,​ in smokers,​ predictive of long-term lung cancer development. Whether smoking cessation impacts the suPAR level is unknown.MATERIALS AND METHODSForty-eight smokers were randomized into three groups of 16: (i) continued to smoke 20 cigarettes per day,​ (ii) refrained from smoking and used transdermal nicotine patches and (iii) refrained from smoking and used placebo patches. Nonsmokers were included for comparison. suPAR and C-reactive protein (CRP) levels were measured by ELISA.RESULTSAt baseline,​ the suPAR level was significantly higher in the 48 smokers (median 3·2 ng mL,​ IQR (2·5-3·9)) than in 46 never smokers (1·9 ng/mL (1·7-2·2)). In smokers randomized to smoking cessation,​ suPAR levels after 4 weeks of stopping were decreased and no longer significantly different from the never smokers values. SuPAR decreased in both those who received a placebo as well as nicotine patch. Interestingly,​ those with the highest suPAR level at time of smoking were also those with the highest level of suPAR after smoking cessation. In contrast,​ smoking or smoking cessation had no influence on CRP levels.CONCLUSIONOur study suggests that the suPAR level may aid to personalize the risk of smoking by identifying those smokers with the highest risk of developing disease and who may have the most benefit of smoking cessation. |
| 545 | Berndt,​ Nadine,​ Bolman,​ Catherine,​ Lechner,​ Lilian,​ Max,​ Wendy,​ Mudde,​ Aart,​ de Vries,​ Hein,​ Evers,​ Silvia. Economic evaluation of a telephone- and face-to-face-delivered counseling intervention for smoking cessation in patients with coronary heart disease. The European journal of health economics : HEPAC : health economics in prevention and care. 2016;17:269.  **Abstract:** OBJECTIVEThis study examined the cost-effectiveness and cost-utility of two smoking cessation counseling interventions differing in their modality for patients diagnosed with coronary heart disease from a societal perspective.METHODSIn a randomized controlled trial conducted in Dutch hospital wards,​ cardiac patients who smoked prior to admission were allocated to usual care (n = 245),​ telephone counseling (n = 223) or face-to-face counseling (n = 157). The counseling interventions lasted for 3 months and were complemented by nicotine patches. Baseline histories were obtained,​ and interviews took place 6 months after hospitalization to assess self-reported smoking status and quality adjusted life years (QALYs). Incremental cost-effectiveness ratios per quitter and cost-utility ratios per QALY were calculated and presented in acceptability curves. Uncertainty was accounted for by sensitivity analysis.RESULTSUsing continued abstinence as the outcome measure showed that telephone counseling had the highest probability of being cost-effective. Face-to-to-face counseling was also more cost-effective than usual care. No significant improvements and differences in QALYs between the three conditions were found. Varying costs and effect estimations revealed that the results of the primary analyses were robust.CONCLUSIONSAssuming a willingness-to-pay of €20,​000 per abstinent patient,​ telephone counseling would be a highly cost-effective smoking cessation intervention assisting cardiac patients to quit. However,​ the lack of consensus concerning the willingness-to-pay per quitter impedes drawing firm conclusions. Moreover,​ studies with extended follow-up periods are needed to capture late relapses and possible differences in QALYs. |
| 554 | Tingen, Martha S., Andrews, Jeannette O., Heath, Janie, Williams, Lovoria B., Schroeder, Carsten, Dainer, Paul, Khleif, Samir N., Waller, Jennifer L.. Tailored parental cessation delivered concurrently with tobacco prevention in children enrolled in urban and rural southern elementary schools. Cancer Epidemiology Biomarkers and Prevention. 2016;25(3):B62.  **Abstract:** Purpose: Socioeconomically disadvantaged populations have higher tobacco use rates resulting in more disparate cancer outcomes. The effectiveness of tailored cessation interventions in parent/caregiver smokers delivered within an elementary school setting while their child is concurrently enrolled in a smoking prevention program has been minimally explored. Procedures/Methods: During a randomized controlled trial (RCT) that included children and one parent/caregiver per child,​ parent/caregiver smokers were offered cessation in years 1 and 2 (if relapsed/refused in year 1) at their child's respective school or local community setting. Parent/caregiver smokers in the intervention group received tailored cessation including eight individual face-to-face motivational interviewing sessions with a matched gender and/or racial/ethnically similar counselor and eight weeks of nicotine replacement therapy. The control parents/caregivers who smoked received information for contacting the state toll-free Tobacco Quit Line and tailored (based on race/ethnicity) written materials to assist with cessation. Children in the intervention arm received concurrently a tobacco prevention curriculum,​ Botvin's LifeSkills Training,​ over the same time period in years 1 & 2. The control group children received the standard health education curriculum for public school systems. This study reports on the effectiveness of the parent/caregiver cessation intervention including outcomes of selfreported smoking status (percentages) and the biological measure,​ salivary cotinine (ng/ml),​ both obtained at baseline,​ end of treatment (EOT)/year 2,​ and in year 4 follow-up. Results/Summary: Parent/Caregiver smokers (n=110) comprised 24.6% of the enrolled (n=447) parent/caregiver sample. The parent/caregiver smokers were predominantly female (81%),​ mean age 37.2 yrs. (SD 11.1); 37% Black; 54% had < a high school education; 54% earned < ,​000 annually; and 6 1 % were covered by Medicaid and/or Medicare. Using repeated measures,​ binomial generalized estimating equation or mixed models,​ differences over time between parents/caregivers in the intervention and control groups were found for quitting smoking and cotinine levels. Parents/caregivers in the intervention group [EOT: 6.5% (SE=5.7%),​ Yr.4: 40.6%(SE=5.7%)] showed a larger increase in quitting smoking over time than the control group [EOT: 0.0%(SE=6.5%),​ Yr.4: 13.2%(SE=6.4%)] (F=4.82,​ p=0.0306). For cotinine (F=5.72,​ p=0.0039),​ the intervention group showed a decrease from baseline (239.9,​[SE=1.3]) to EOT (99.3,​[SE=1.4]) and then maintenance through Yr.4 (109.6,​[SE=1.4]),​ while the control group showed increases from baseline (221.1,​[SE=1.4]) to EOT (239.0,​[SE=1.4]) to Yr.4 (325.8,​ [SE=14]). Conclusions: This study provides beginning evidence that tailored cessation offered to parents/caregivers in their child's school setting while their child is enrolled in tobacco prevention classes may be an avenue for more robust success in quitting smoking. Such a strategy may serve as an initial step for improving public health and for decreasing tobacco-related disparities and associated poor health outcomes among disadvantaged populations. By improving parent's/caregiver's success and maintenance of tobacco cessation,​ a joint program may reinforce the child's resistance to the lure of tobacco products and may also potentially decrease his or her secondhand smoke exposure within the home. |
| 559 | Unrod,​ Marina,​ Simmons,​ Vani N,​ Sutton,​ Steven K,​ Cummings,​ K Michael,​ Celestino,​ Paula,​ Craig,​ Benjamin M,​ Lee,​ Ji-Hyun,​ Meltzer,​ Lauren R,​ Brandon,​ Thomas H. Relapse-Prevention Booklets as an Adjunct to a Tobacco Quitline: A Randomized Controlled Effectiveness Trial. Nicotine & tobacco research : official journal of the Society for Research on Nicotine and Tobacco. 2016;18:298.  **Abstract:** INTRODUCTIONRelapse prevention (RP) remains a major challenge to smoking cessation. Previous research found that a set of self-help RP booklets significantly reduced smoking relapse. This study tested the effectiveness of RP booklets when added to the existing services of a telephone quitline.METHODSQuitline callers (N = 3458) were enrolled after their 2-week quitline follow-up call and randomized to one of three interventions: (1) Usual Care: standard intervention provided by the quitline,​ including brief counseling and nicotine replacement therapy; (2) Repeated Mailings (RM): eight Forever Free RP booklets sent to participants over 12 months; and (3) Massed Mailings: all eight Forever Free RP booklets sent upon enrollment. Follow-ups were conducted at 6-month intervals,​ through 24 months. The primary outcome measure was 7-day-point-prevalence-abstinence.RESULTSOverall abstinence rates were 61.0% at baseline,​ and 41.9%,​ 42.7%,​ 44.0%,​ and 45.9% at the 6-,​ 12-,​ 18- and 24-month follow-ups,​ respectively. Although RM produced higher abstinence rates,​ the differences did not reach significance for the full sample. Post-hoc analyses of at-risk subgroups revealed that among participants with high nicotine dependence (n = 1593),​ the addition of RM materials increased the abstinence rate at 12 months (42.2% vs. 35.2%; OR = 1.38; 95% CI = 1.03% to 1.85%; P = .031) and 24 months (45% vs. 38.8%; OR = 1.31; 95% CI = 1.01% to 1.73%; P = .046).CONCLUSIONSSending self-help RP materials to all quitline callers appears to provide little benefit to deterring relapse. However,​ selectively sending RP booklets to callers explicitly seeking assistance for RP and those identified as highly dependent on nicotine might still prove to be worthwhile. |
| 564 | Cunningham,​ John A,​ Kushnir,​ Vladyslav,​ Selby,​ Peter,​ Tyndale,​ Rachel F,​ Zawertailo,​ Laurie,​ Leatherdale,​ Scott T. Effect of Mailing Nicotine Patches on Tobacco Cessation Among Adult Smokers: A Randomized Clinical Trial. JAMA internal medicine. 2016;176:184.  **Abstract:** IMPORTANCEThe efficacy of nicotine replacement therapy (NRT) is well demonstrated in clinical trials in which NRT is accompanied by behavioral support. Epidemiologic data,​ however,​ indicate that people using NRT are no more likely to successfully quit smoking than those who do not use NRT.OBJECTIVETo evaluate the effect of mailing nicotine patches to smokers without behavioral support on quit success rates.DESIGN,​ SETTING,​ AND PARTICIPANTSA single-blinded,​ 2-group randomized clinical trial of adult smokers recruited across Canada by random-digit dialing of home and cell telephone numbers from June 4,​ 2012,​ through June 26,​ 2014. Follow-up was completed on January 5,​ 2015,​ and data were analyzed from May 24,​ 2015,​ through July 6,​ 2015. A total of 2093 individuals who smoked more than 10 cigarettes per day were interviewed at baseline and asked if they would be hypothetically interested in receiving nicotine patches by mail to quit smoking. Those who were interested and deemed eligible to participate (no contraindications to NRT) were randomized to the experimental group to be mailed a 5-week supply of nicotine patches or to a control group. Telephone follow-ups were conducted at 8 weeks and 6 months.INTERVENTIONSParticipants in the experimental group were sent a 5-week course of nicotine patches by expedited postal mail (3 weeks of step 1 [21 mg of nicotine],​ 1 week of step 2 [14 mg of nicotine],​ 1 week of step 3 [7 mg of nicotine],​ no behavioral support provided). Participants randomized to the control group were not offered the nicotine patches or any other intervention.MAIN OUTCOMES AND MEASURESThe primary outcome was 30-day smoking abstinence at 6 months.RESULTSOf the 2093 participants who were interviewed as part of the baseline survey (76.5% response rate),​ 1000 were found eligible for the trial and randomized to a group. Analyses were conducted on 500 participants in the experimental group (mean [SD] age,​ 48.0 [12.8] years; 255 female [51.0%]) and 499 in the control group (mean [SD] age,​ 49.7 [12.7] years; 256 female [51.3%]). Self-reported abstinence rates were significantly higher among participants who were sent nicotine patches compared with the control group (30-day abstinence: 38 [7.6%] of 500 vs 15 [3.0%] of 499; odds ratio,​ 2.65; 95% CI,​ 1.44-4.89; P = .002). Usable saliva samples were returned by only 50.9% of the participants. Biochemically validated abstinence at 6 months was found in 14 (2.8%) of 500 participants in the experimental group vs 5 (1.0%) of 499 in the control group (odds ratio,​ 2.85; 95% CI,​ 1.02-7.96; P = .046).CONCLUSIONS AND RELEVANCEThe trial provides evidence of the effectiveness of mailed nicotine patches without behavioral support to promote tobacco cessation. The strength of these findings is tempered by the lack of biochemical validation for all participants.TRIAL REGISTRATIONclinicaltrials.gov Identifier: NCT01429129. |
| 565 | Raja,​ Mitali,​ Saha,​ Sabyasachi,​ Krishna-Reddy,​ Vamsi,​ Mohd,​ Shafaat,​ Narang,​ Ridhi,​ Sood,​ Poonam. Effectiveness of oral health education versus nicotine replacement therapy for tobacco cessation- a parallel randomized clinical trial. Journal of clinical and experimental dentistry. 2016;8:e64.  **Abstract:** BACKGROUNDIndia has millions of tobacco users. It is the leading cause of deaths due to oral cancer and hence needs effective strategies to curb it. Hence the aim of present study was to evaluate and compare the effectiveness of Oral Health Education (OHE) and Nicotine Replacement Therapy (NRT) in tobacco cessation.MATERIAL AND METHODSThe clinical trial consisted of Manohar Lal Kapoor (MLK) factory workers (n= 40) giving history of tobacco consumption (smoking/smokeless) within past 30 days. They were randomized into OHE (n=20) and NRT (n=20) groups. Baseline evaluation (demographic,​ smoking/ smokeless behaviour) was done. Fagerstrom test was used for Nicotine Dependence (FTND) and to assess nicotine addiction level. Follow up was done at an interval of 1week,​ 2 weeks,​ 1 month,​ 2 months and 3 months to assess the reduction in the mean FTND score. "Nano-CheckTM Rapid Nicotine test" was used for the qualitative detection of cotinine in human urine. Appropriate statistical analysis was performed (Paired and Unpaired t test).RESULTSIn both OHE and NRT group there was a significant reduction (p< 0.00001) in mean Fagerstrom score at every follow up but when both the groups were compared mean Fagerstrom score reduction was more in NRT than OHE at all time interval though it was not statistically significant (p>0.05).CONCLUSIONSNRT is better than OHE when both the groups were compared. However,​ it was found that any intervention given to tobacco users either NRT or OHE is helpful for the patients in the process of quitting tobacco.KEY WORDSTobacco cessation,​ nicotine replacement therapy,​ oral health education,​ fagerstrom test,​ urine cotinine. |
| 567 | Baker,​ Timothy B,​ Piper,​ Megan E,​ Stein,​ James H,​ Smith,​ Stevens S,​ Bolt,​ Daniel M,​ Fraser,​ David L,​ Fiore,​ Michael C. Effects of Nicotine Patch vs Varenicline vs Combination Nicotine Replacement Therapy on Smoking Cessation at 26 Weeks: A Randomized Clinical Trial. JAMA. 2016;315:371.  **Abstract:** IMPORTANCESmoking cessation medications are routinely used in health care; it is vital to identify medications that most effectively treat this leading cause of preventable mortality.OBJECTIVETo compare the efficacies of varenicline,​ combination nicotine replacement therapy (C-NRT),​ and the nicotine patch for 26-week quit rates.DESIGN,​ SETTING,​ AND PARTICIPANTSThree-group randomized intention-to-treat clinical trial occurring from May 2012 to November 2015 among smokers recruited in the Madison,​ Wisconsin,​ and Milwaukee,​ Wisconsin,​ communities; 65.5% of smokers offered the study (2687/4102) refused participation prior to randomization.INTERVENTIONSParticipants were randomized to one of three 12-week open-label smoking cessation pharmacotherapy groups: (1) nicotine patch only (n = 241); (2) varenicline only (including 1 prequit week; n = 424); and (3) C-NRT (nicotine patch + nicotine lozenge; n = 421). Six counseling sessions were offered.MAIN OUTCOMES AND MEASURESThe primary outcome was carbon monoxide-confirmed self-reported 7-day point-prevalence abstinence at 26 weeks. Secondary outcomes were carbon monoxide-confirmed self-reported initial abstinence,​ prolonged abstinence at 26 weeks,​ and point-prevalence abstinence at weeks 4,​ 12,​ and 52.RESULTSAmong 1086 smokers randomized (52% women; 67% white; mean age,​ 48 years; mean of 17 cigarettes smoked per day),​ 917 (84%) provided 12-month follow-up data. Treatments did not differ on any abstinence outcome measure at 26 or 52 weeks,​ including point-prevalence abstinence at 26 weeks (nicotine patch,​ 22.8% [55/241]; varenicline,​ 23.6% [100/424]; and C-NRT,​ 26.8% [113/421]) or at 52 weeks (nicotine patch,​ 20.8% [50/241]; varenicline,​ 19.1% [81/424]; and C-NRT,​ 20.2% [85/421]). At 26 weeks,​ the risk differences for abstinence were,​ for patch vs varenicline,​ -0.76% (95% CI,​ -7.4% to 5.9%); for patch vs C-NRT,​ -4.0% (95% CI,​ -10.8% to 2.8%); and for varenicline vs C-NRT,​ -3.3% (95% CI,​ -9.1% to 2.6%). All medications were well tolerated,​ but varenicline produced more frequent adverse events than did the nicotine patch for vivid dreams,​ insomnia,​ nausea,​ constipation,​ sleepiness,​ and indigestion.CONCLUSIONS AND RELEVANCEAmong adults motivated to quit smoking,​ 12 weeks of open-label treatment with nicotine patch,​ varenicline,​ or C-NRT produced no significant differences in biochemically confirmed rates of smoking abstinence at 26 weeks. The results raise questions about the relative effectiveness of intense smoking pharmacotherapies.TRIAL REGISTRATIONclinicaltrials.gov Identifier: NCT01553084. |
| 568 | Cook,​ Jessica W,​ Collins,​ Linda M,​ Fiore,​ Michael C,​ Smith,​ Stevens S,​ Fraser,​ David,​ Bolt,​ Daniel M,​ Baker,​ Timothy B,​ Piper,​ Megan E,​ Schlam,​ Tanya R,​ Jorenby,​ Douglas,​ Loh,​ Wei-Yin,​ Mermelstein,​ Robin. Comparative effectiveness of motivation phase intervention components for use with smokers unwilling to quit: a factorial screening experiment. Addiction (Abingdon,​ England). 2016;111:117.  **Abstract:** AIMSTo screen promising intervention components designed to reduce smoking and promote abstinence in smokers initially unwilling to quit.DESIGNA balanced,​ four-factor,​ randomized factorial experiment.SETTINGEleven primary care clinics in southern Wisconsin,​ USA.PARTICIPANTSA total of 517 adult smokers (63.4% women,​ 91.1% white) recruited during primary care visits who were willing to reduce their smoking but not quit.INTERVENTIONSFour factors contrasted intervention components designed to reduce smoking and promote abstinence: (1) nicotine patch versus none; (2) nicotine gum versus none; (3) motivational interviewing (MI) versus none; and (4) behavioral reduction counseling (BR) versus none. Participants could request cessation treatment at any point during the study.MEASUREMENTSThe primary outcome was percentage change in cigarettes smoked per day at 26 weeks post-study enrollment; the secondary outcomes were percentage change at 12 weeks and point-prevalence abstinence at 12 and 26 weeks post-study enrollment.FINDINGSThere were few main effects,​ but a significant four-way interaction at 26 weeks post-study enrollment (P = 0.01,​ β = 0.12) revealed relatively large smoking reductions by two component combinations: nicotine gum combined with BR and BR combined with MI. Further,​ BR improved 12-week abstinence rates (P = 0.04),​ and nicotine gum,​ when used without MI,​ increased 26-week abstinence after a subsequent aided quit attempt (P = 0.01).CONCLUSIONSMotivation-phase nicotine gum and behavioral reduction counseling are promising intervention components for smokers who are initially unwilling to quit. |
| 569 | Piper,​ Megan E,​ Fiore,​ Michael C,​ Smith,​ Stevens S,​ Fraser,​ David,​ Bolt,​ Daniel M,​ Collins,​ Linda M,​ Mermelstein,​ Robin,​ Schlam,​ Tanya R,​ Cook,​ Jessica W,​ Jorenby,​ Douglas E,​ Loh,​ Wei-Yin,​ Baker,​ Timothy B. Identifying effective intervention components for smoking cessation: a factorial screening experiment. Addiction (Abingdon,​ England). 2016;111:129.  **Abstract:** AIMSTo identify promising intervention components intended to help smokers to attain and maintain abstinence in their quit smoking attempts.DESIGNA fully crossed,​ six-factor randomized fractional factorial experiment.SETTINGEleven primary care clinics in southern Wisconsin,​ USA.PARTICIPANTSA total of 637 adult smokers (55% women,​ 88% white) motivated to quit smoking who visited primary care clinics.INTERVENTIONSSix intervention components designed to prepare smokers to quit,​ and achieve and maintain abstinence (i.e. for the preparation,​ cessation and maintenance phases of smoking treatment): (1) preparation nicotine patch versus none; (2) preparation nicotine gum versus none; (3) preparation counseling versus none; (4) intensive cessation in-person counseling versus minimal; (5) intensive cessation telephone counseling versus minimal; and (6) 16 versus 8 weeks of combination nicotine replacement therapy (nicotine patch  +  nicotine gum).MEASUREMENTSSeven-day self-reported point-prevalence abstinence at 16 weeks.FINDINGSPreparation counseling significantly improved week 16 abstinence rates (P = .04),​ while both forms of preparation nicotine replacement therapy interacted synergistically with intensive cessation in-person counseling (P < 0.05). Conversely,​ intensive cessation phone counseling and intensive cessation in-person counseling interacted antagonistically (P < 0.05)-these components produced higher abstinence rates by themselves than in combination.CONCLUSIONSPreparation counseling and the combination of intensive cessation in-person counseling with preparation nicotine gum or patch are promising intervention components for smoking and should be evaluated as an integrated treatment package. |
| 570 | Schlam,​ Tanya R,​ Fiore,​ Michael C,​ Smith,​ Stevens S,​ Fraser,​ David,​ Bolt,​ Daniel M,​ Collins,​ Linda M,​ Mermelstein,​ Robin,​ Piper,​ Megan E,​ Cook,​ Jessica W,​ Jorenby,​ Douglas E,​ Loh,​ Wei-Yin,​ Baker,​ Timothy B. Comparative effectiveness of intervention components for producing long-term abstinence from smoking: a factorial screening experiment. Addiction (Abingdon,​ England). 2016;111:142.  **Abstract:** AIMSTo identify promising intervention components that help smokers attain and maintain abstinence during a quit attempt.DESIGNA 2 × 2 × 2 × 2 × 2 randomized factorial experiment.SETTINGEleven primary care clinics in Wisconsin,​ USA.PARTICIPANTSA total of 544 smokers (59% women,​ 86% white) recruited during primary care visits and motivated to quit.INTERVENTIONSFive intervention components designed to help smokers attain and maintain abstinence: (1) extended medication (26 versus 8 weeks of nicotine patch + nicotine gum); (2) maintenance (phone) counseling versus none; (3) medication adherence counseling versus none; (4) automated (medication) adherence calls versus none; and (5) electronic medication monitoring with feedback and counseling versus electronic medication monitoring alone.MEASUREMENTSThe primary outcome was 7-day self-reported point-prevalence abstinence 1 year after the target quit day.FINDINGSOnly extended medication produced a main effect. Twenty-six versus 8 weeks of medication improved point-prevalence abstinence rates (43 versus 34% at 6 months; 34 versus 27% at 1 year; P = 0.01 for both). There were four interaction effects at 1 year,​ showing that an intervention component's effectiveness depended upon the components with which it was combined.CONCLUSIONSTwenty-six weeks of nicotine patch + nicotine gum (versus 8 weeks) and maintenance counseling provided by phone are promising intervention components for the cessation and maintenance phases of smoking treatment. |
| 571 | Xiao,​ Dan,​ Kang,​ Jian,​ Kotler,​ Mitchell,​ Wang,​ Chen. A multicenter randomized,​ double-blind,​ parallel,​ placebo-controlled clinical study to evaluate the efficacy and safety of nicotine mint lozenge (2 mg and 4 mg) in smoking cessation. Chest. 2016;149:A589.  **Abstract:** PURPOSE: To evaluate the efficacy in smoking cessation and safety of 2mg and 4mg Nicotine Mint Lozenges in Chinese adult smokers. METHODS: This was a multi-centre,​ randomized,​ stratified,​ double-blind,​ placebo-controlled,​ parallel-group study. The low-dependence stratum had 483 smokers randomized (241 to active 2mg nicotine lozenge and 242 to placebo lozenge). The high-dependent stratum had 240 smokers randomized (120 to active 4mg nicotine lozenge and 120 to placebo lozenge). The primary endpoint was successful smoking cessation at 6 weeks post-quit; this was defined as continuous abstinence from smoking for the 28-day period up to and including the 6-week visit (verified by carbon monoxide (CO) measurement). Cochran-Mantel-Haenszel (CMH) tests were performed to compare quit rates between active nicotine and placebo separately for the high and low dependence strata. RESULTS: The primary analysis showed that in the low-dependence (2mg) stratum 59 subjects (24.48%) out of 241 in the active nicotine group and 52 subjects (21.49%) out of 242 in the placebo group were successful quitters (P=0.3851). In the high-dependence (4mg) stratum,​ 37 subjects (30.8%) out of 120 in the active nicotine group and 24 subjects (20.2%) out of 119 in the placebo group were successful quitters (P=0.0565). CONCLUSIONS: The 4mg nicotine lozenge showed improved smoking cessation rates in high-dependence Chinese adult smokers with directional significance for the primary end point. The 2mg nicotine lozenge showed higher smoking cessation rates than placebo but the differences were not statistically significant. Both nicotine lozenges were safe and well tolerated in Chinese adult smokers. CLINICAL IMPLICATIONS: Tobacco use is responsible for the death of nearly six million people each year globally - more than tuberculosis,​ HIV/AIDS,​ and malaria combined. China is the biggest tobacco producer,​ biggest tobacco consumer,​ and biggest victim of tobacco-related diseases. Globally,​ about one-third smokers are Chinese. More than one million Chinese die from smoking-related diseases each year. Concerning tobacco dependence as a chronic disease,​ effective medications are helpful for addictive smokers quit smoking. The efficacy and safety data of nicotine lozenges of Chinese smokers in this abstract provided a new picture in tobacco treatment area in China. |
| 580 | Wilcox,​ Charles,​ Oskooilar,​ Nader,​ Guevarra,​ Kimberly,​ -Linh Tong,​ My,​ Grosz,​ Daniel,​ Morrissey,​ Judy,​ Henry,​ Mellissa,​ De Francisco,​ Don. A double-blind,​ active-and placebo-controlled evaluation of the neuropsychiatric safety and efficacy of varenicline and bupropion for smoking cessation in subjects with (Pre-Existing) psychiatric disorders: An objective blinded analysis. Neuropsychopharmacology. 2015;40:S260.  **Abstract:** Background: Neuropsychiatric Symptoms (NPS) have been reported and highly publicized with respect to both Chantixs (varenicline) and Zybant (bupropion). Further complicating any cause-and-effect inferences is the fact that anxiety and/or depressed mood may be symptoms of nicotine withdrawal. To investigate the neuropsychiatric profiles of varenicline and bupropion,​ as compared with placebo,​ we enrolled smokers with a prior history of a psychiatric disorder,​ who were motivated to stop smoking. Methods: Our Southern California-based research center,​ along with approximately 200 other research sites in multiple countries,​ enrolled a total of 8,​000 subjects in this Phase-IV,​ double-blind,​ Nicotine Replacement Therapy (NRT)-and placebo-controlled study. We are reporting (only) on data generated by and statistically analyzed at Pharmacology Research Institute (PRI). There was a 3-to-14 day screening phase into which 51 subjects were entered and six were excluded. Forty-five subjects who met all of the entry criteria were randomly assigned on a 1:1:1:1 ratio to varenicline,​ bupropion,​ NRT patch or placebo. The duration of the active treatment phase was 12 weeks,​ followed by a non-treatment follow-up phase for an additional 12 weeks. There were weekly visits up to and including Week 6 and then bi-weekly visits between Weeks 6 and 12. On weeks with no scheduled clinic visits,​ telephone contact visits were performed to collect the smoking status of participants. All subjects set a target quit date (TQD) to coincide with their Week 1 visit,​ study Day 8. Brief (10-minute) smoking cessation counseling sessions,​ consistent with the Agency for Healthcare Research and Quality (AHRQ),​ were incorporated into each clinic visit. The primary objective was to evaluate the neuropsychiatric profiles of varenicline and bupropion as compared with placebo,​ including any neuropsychiatric adverse experiences (NAEs) at endpoint. The primary efficacy endpoint was 4-weeks carbon monoxide (CO)-confirmed abstinence from Weeks 9-12. The secondary efficacy endpoint was CO-confirmed continuous abstinence from Weeks 9 through 24. Bivariate summary statistical analyses for continuous variables in successful quitters versus non-quitters were performed. The non-parametric Wilcoxon rank sum test was used to compute the p values for this comparison. The p values for comparing categorical variables in quitters and non-quitters [also ignoring time to quit] were computed using Fisher's exact test (2 x 2) or the chi-square test. Multivariate analyses using both Cox proportional hazard regression model,​ looking at quit rate per week,​ and a regression tree were used to simultaneously evaluate fifteen (15) potential predictors. Inclusion Criteria (partial)Male and female cigarette smokers,​ 18-75 years of age Smoked at least 10 cigarettes per day during the past year Exhaled carbon monoxide (CO)410 ppm at screening visit Current and/or past Axis I and/or II diagnosis using DSM-IV-TR criteria based on clinical assessment and confirmed by SCID Results: The “top line” results indicated that only one (1) subject had a neuropsychiatric adverse experience (NAE),​ panic attack. She began the study with a primary diagnosis of Generalized Anxiety Disorder (GAD) and Social Phobia,​ under the care of a therapist. At approximately “Week 9,​” she began taking Abilifys (aripiprazole) [15 mg./day],​ missing study medication doses and 'losing' her NRT patches. She was discontinued from the study at Week 12 [end of active treatment] at which time she had successfully stopped smoking. Smoking cessation (e.g.,​ quit) rates were such that 15 of the 42 evaluable subjects,​ or 35.7%,​ were categorized as successful quitters utilizing the protocol-specified definition of CO values of less than ten parts per million [o 10 ppm]. Notwithstanding the very small sample size(s),​ statistical analyses of previously observed and reported predictors of successful cessation,​ again demonstrated statistically significant results,​ including the average number of cigarettes per day (p <.05) and the number of lifetime quit attempts (p <.05). Similarly,​ there was also a statistical trend with respect to the age one started smoking (p<.16) and Body Mass Index (BMI) (p <.07) as having potential predictive utility with respect to successful cessation. Conclusions: Our objective “still blinded-to-treatment assignment” analyses of neuropsychiatric adverse events,​ all involving subjects with a prior and/or current diagnosis of a psychiatric disorder,​ indicated that only one subject had a relatively mild NAE,​ which may have pertained more to her non-compliance than study drug-related causality. At her completion in the active treatment phase of the trial,​ she had successfully stopped smoking. Previously reported positive baseline indicators of successful smoking cessation were replicated and re-verified vis-à-vis our analyses. |
| 588 | Rohsenow,​ Damaris,​ Tidey,​ Jennifer W.,​ Martin,​ Rosemarie A.,​ Colby,​ Suzanne,​ Monti,​ Peter M.. Varenicline versus nicotine patch plus brief advice for sober smokers in substance treatment. Drug and Alcohol Dependence. 2015;156:e191.  **Abstract:** Aims: Smokers with substance use disorders (SUD) have great difficulty quitting smoking during their first year of sobriety. Varenicline (VAR) is the most effective medication for smoking cessation,​ has few contraindications,​ targets nicotine receptors selectively,​ and reduces alcohol use,​ so may be best in this population. The aim was to investigate effects of treatment with varenicline versus nicotine replacement (NRT),​ both combined with brief advice adapted for sobriety concerns,​ for smokers in treatment for SUD. Methods: Smokers abstinent <12 months in any SUD treatment (n = 137) from the community were randomized to 12 wks varenicline vs. nicotine patch,​ double-placebo,​ plus 8 sessions brief advice adapted to address sobriety concerns. Randomization was stratified by depression diagnosis and gender. Smoking point-prevalence (7-day) abstinence was assessed during the 12 weeks and at 3,​ 6 and 12 months after treatment start using self-report and cotinine confirmation of past 7 days point-prevalence abstinence. Results: Within treatment,​ 12% had complete abstinence at 8 weeks in each condition (ns). At 3 months,​ 13% with VAR and 3% with NRT had point-prevalence abstinence (p < .05) and at 6 months 9% with VAR and 3% with NRT had point-prevalence abstinence (p = .18). At 6 months,​ heavy drinking was reported by 29% in VAR and 16% in placebo,​ and drug use by 29% in VAR and 32% in placebo (all non-significant). Conclusions: Varenicline with brief advice greatly increased the odds of smoking abstinence at 3 months and did not harm SUD recovery. While varenicline resulted in 3 times as much abstinence as placebo at 6 months,​ the results were no longer significant. Thus,​ varenicline has utility in this population. |
| 589 | Vaz,​ Luis R,​ Coleman,​ Tim,​ Cooper,​ Sue,​ Aveyard,​ Paul,​ Leonardi-Bee,​ Jo. The Nicotine Metabolite Ratio in Pregnancy Measured by trans-3'-Hydroxycotinine to Cotinine Ratio: Characteristics and Relationship With Smoking Cessation. Nicotine & tobacco research : official journal of the Society for Research on Nicotine and Tobacco. 2015;17:1318.  **Abstract:** INTRODUCTIONNicotine replacement therapy (NRT) helps nonpregnant smokers quit,​ but there is no evidence that standard dose NRT is effective in pregnancy. As nicotine metabolism increases in pregnancy,​ this could reduce NRT efficacy. Using the ratio of trans-3'-hydroxycotinine to cotinine,​ the nicotine metabolite ratio (NMR),​ we investigated relationships between the rate of nicotine metabolism,​ maternal characteristics and smoking cessation in pregnant women recruited to a randomized controlled trial of NRT.METHODSData from 1,​050 pregnant smokers in the Smoking,​ Nicotine and Pregnancy trial who were of 12-24 weeks gestation had exhaled carbon monoxide readings of ≥8 ppm at recruitment and who were randomized to NRT or placebo patches were used. Linear and logistic regression investigated associations between maternal characteristics and NMR and also between NMR and subsequent validated cessation from smoking.RESULTSSix hundred and sixty-two women (63%) provided blood samples for NMR estimation. Higher NMR was associated with increased cigarette consumption prior to pregnancy. At 1 month (odds ratio [OR] = 0.87; 95% CI = 0.76-0.99; p = .043) and delivery (OR = 0.79; 95% CI = 0.66-0.95; p = .010),​ there was a significant negative association between a 0.1 unit increase in NMR and odds of achieving cessation after adjusting for possible confounders. There was no evidence for an interaction between a 0.1 unit increase in NMR and treatment assignment on the odds of cessation at 1 month post-quit date (p = .556).CONCLUSIONPregnant women who metabolize nicotine more rapidly are less likely to achieve cessation when they try to quit smoking. There is no evidence that NRT is more effective in women who metabolize nicotine more slowly. |
| 593 | Jalali,​ Farzad,​ Afshari,​ Reza,​ Babaei,​ Ali,​ Abasspour,​ Hassan,​ Vahedian-Shahroodi,​ Mohammad. Comparing Motivational Interviewing-Based Treatment and its combination with Nicotine Replacement Therapy on smoking cessation in prisoners: a randomized controlled clinical trial. Electronic physician. 2015;7:1318.  **Abstract:** BACKGROUNDThe prevalence of smoking is much higher in prisoners than it is in the general population. Prisoners who smoke cause many health problems for themselves and other prisoners. Therefore,​ we should help them stop smoking.OBJECTIVETo compare the effects of motivational interviewing-based (MI-based) treatment and its combination with nicotine replacement therapy (NRT) on smoking cessation in prisoners at Mashhad Central Prison.METHODSThe study was designed as a double-blind,​ randomized,​ controlled clinical trial,​ and it began in February 2013 and ended in February 2014. Two hundred and thirteen prisoners met the inclusion criteria and were enrolled in the study. They were divided randomly into three groups,​ i.e.,​ MI-based treatment,​ MI with NRT,​ and the control group,​ which didn't receive any therapy. The outcome measures were reported after intervention and at a 90-day follow-up,​ and changes in the CO levels in expired air and nicotine dependency were measured.RESULTSThe average age of the subjects was 37.59 ± 8.76,​ and their mean duration of imprisonment was 3.3 ± 1.90 years. They smoked an average of 21.84 ± 8.72 cigarettes per day. Analysis of the concentration of CO in expired air in the pre-test,​ post-test,​ and at the follow-up for the three groups showed that the variations in the mean CO concentrations in the MI group and the MI with NRT group at the pre-test and at the post-test were statistically significant (p < 0.001),​ but no significant changes occurred between the post-test and the follow-up (p > 0.050). In addition,​ the results indicated that CO concentration in expired air in the MI with NRT group was statistically significant,​ with better efficacy of smoking cessation,​ compared with control group and the MI group after the follow-up (p = 0.02).CONCLUSIONSMotivational interviewing combined with NRT for smoking cessation is more effective than MI alone,​ and it resulted in a significant decrease in the CO concentration in expired air at the 90-day follow-up. |
| 595 | Chandrashekar,​ M,​ Sattar,​ F A,​ Bondade,​ S,​ Kumar,​ K Kiran. A comparative study of different modalities of treatment in nicotine dependence syndrome. Asian journal of psychiatry. 2015;17:29.  **Abstract:** UNLABELLEDThere are different modalities for management of Nicotine dependence,​ but it is still inconclusive which is the best modality for the treatment of Nicotine dependence syndrome (NDS). In this background the present study was carried out to assess the efficacy and to compare different modalities for the treatment of NDS.METHODSPatients diagnosed as NDS as per ICD-10 were taken up for study. These patients were administered proforma to elicit sociodemographic details,​ Fagerstrom test for Nicotine Dependence,​ Questionnaire of Smoking Urges-Brief and breath analysis was done using carbon monoxide meter. Assessment was done at base line and at weekly follow-ups for 12 weeks. Patients were divided into six groups randomly. Group A received BUP at a dose of 150mg/day for 3 days; subsequently increased to 300mg/day,​ Group B: for initial 6 weeks Nicotine gum of 4mg every 1-2 hourly was used and next 6 weeks every 2-4 hourly was used,​ Group C: BI,​ Group D: BI+BUP,​ Group E: BI+NRT,​ Group F received BUP+NRT+BI.RESULTSThe quit rates at end of the study were BUP-30%,​ NRT-26.66%,​ BI-23.33%,​ BI+BUP-43.33%,​ BI+NRT-33.33%,​ and BI+BUP+NRT-50%. BI+BUP+NRT had 2-3 times more quit rates than the individual modality treatment group.CONCLUSIONThere was no statistically significant difference between the study groups,​ but there was clinical difference in quit rates. Among the groups BI+BUP+NRT had higher quit rates compared to other groups. Combination modalities yield better quit rates than individual modalities. |
| 599 | Cosci,​ Fiammetta,​ Anna Aldi,​ Giulia,​ Nardi,​ Antonio Egidio. Does smoking abstinence influence distress tolerance? An experimental study comparing the response to a breath-holding test of smokers under tobacco withdrawal and under nicotine replacement therapy. Psychiatry research. 2015;229:89.  **Abstract:** Distress tolerance has been operationalized as task persistence in stressful behavioral laboratory tasks. According to the distress tolerance perspective,​ how an individual responds to discomfort/distress predicts early smoking lapses. This theory seems weakly supported by experimental studies since they are limited in number,​ show inconsistent results,​ do not include control conditions. We tested the response to a stressful task in smokers under abstinence and under no abstinence to verify if tobacco abstinence reduces task persistence,​ thus distress tolerance. A placebo-controlled,​ double-blind,​ randomized,​ cross-over design was used. Twenty smokers underwent a breath holding test after the administration of nicotine on one test day and a placebo on another test day. Physiological and psychological variables were assessed at baseline and directly before and after each challenge. Abstinence induced a statistically significant shorter breath holding duration relative to the nicotine condition. No different response to the breath holding test was observed when nicotine and placebo conditions were compared. No response to the breath holding test was found when pre- and post-test values of heart rate,​ blood pressure,​ Visual Analogue Scale for fear or discomfort were compared. In brief,​ tobacco abstinence reduces breath holding duration but breath holding test does not influence discomfort. |
| 601 | Karip,​ Bora,​ Metin,​ Keskin,​ Yalin,​ Iscan,​ Emre,​ Balik. Nicotine gum chewing for postoperative ileus after colorectal surgery. Colorectal Disease. 2015;17:29.  **Abstract:** Aim: Our aim was to evaluate the effects of different sham feeding methods on postoperative ileus after rectum cancer surgery. Method: 68 rectum cancer patients who underwent low anterior resection with an ileostomy were randomized into 4 groups: control (n = 16),​ ice cream (n = 18),​ gum (n = 17) and nicotine gum (n = 17). Data collected including patients' demographics,​ smoking habbits,​ constipation scores. Amount of perioperative bleeding and fluid administration,​ drain placement,​ time of the first sense of stoma motility,​ stool passage from ileostomy and return of appetite were recorded in each group and compared. Results: Constipation scores,​ smoking conditions,​ intraoperative bleeding,​ fluid administration and drain placement were similar between groups (P > 0,​05). First sense of stoma motility was shorter in nicotine group than control and gum groups (P=0,​004 and 0,​046). Average time to first stool passage and first appetite were found shortest in the nicotine gum group. In paired comparisons stool passage time was significantly shorter in the nicotine than control group (P = 0,​011). Return of appetite was found shorter in nicotine group when compared to gum group (P = 0.036). Conclusion: Nicotine gum chewing seems to be an effective method to resolve postoperative ileus after rectal cancer surgery. |
| 602 | Chen,​ Li-Shiun,​ Baker,​ Timothy B,​ Jorenby,​ Douglas,​ Piper,​ Megan,​ Saccone,​ Nancy,​ Johnson,​ Eric,​ Breslau,​ Naomi,​ Hatsukami,​ Dorothy,​ Carney,​ Robert M,​ Bierut,​ Laura J. Genetic variation (CHRNA5),​ medication (combination nicotine replacement therapy vs. varenicline),​ and smoking cessation. Drug and alcohol dependence. 2015;154:278.  **Abstract:** OBJECTIVERecent evidence suggests that the efficacy of smoking cessation pharmacotherapy can vary across patients based on their genotypes. This study tests whether the coding variant rs16969968 in the CHRNA5 nicotinic receptor gene predicts the effects of combination nicotine replacement therapy (cNRT) and varenicline on treatment outcomes.METHODIn two randomized smoking cessation trials comparing cNRT vs. placebo,​ and varenicline vs. placebo,​ we used logistic regression to model associations between CHRNA5 rs16969968 and abstinence at end of treatment.RESULTSFor abstinence at end of treatment,​ there was an interaction between cNRT and rs16969968 (X(2)=8.15,​ df=2,​ omnibus-p=0.017 for the interaction); individuals with the high-risk AA genotype were more likely to benefit from cNRT. In contrast,​ varenicline increased abstinence,​ but its effect did not vary with CHRNA5. However,​ the genetic effects differed between the placebo control groups across two trials (wald=3.94,​ df=1,​ p=0.047),​ this non-replication can alter the interpretation of pharmacogenetic findings.CONCLUSIONSResults from two complementary smoking cessation trials demonstrate inconsistent genetic results in the placebo arms. This evidence highlights the need to compare the most effective pharmacotherapies with the same placebo control to establish pharmacogenetic evidence to aid decisions on medication choice for patients trying to quit smoking. |
| 603 | Guillot, Casey R, Stone, Matthew D, Geary, Bree A, Kirkpatrick, Matthew G, Tidey, Jennifer W, Cook, Jessica W, Leventhal, Adam M. Pharmacological, sensorimotor, and expectancy effects on tobacco withdrawal: a preliminary study. Human psychopharmacology. 2015;30:364.  **Abstract:** OBJECTIVEResearch designs for parsing the mechanisms underlying tobacco withdrawal are scant. This study introduced a novel research design that simultaneously manipulated three tobacco withdrawal mechanisms: pharmacological (nicotine dissipation), sensorimotor (elimination of the smoking ritual), and expectancy (activation of beliefs regarding the effects of nicotine deprivation), permitting examination of the effects of each mechanism while holding the other two mechanisms constant.METHODSFollowing overnight abstinence, 32 regular cigarette smokers were randomized in a 2 (expectancy: told patch contains nicotine versus told placebo patch) × 2 (drug: receive 21-mg transdermal nicotine patch versus receive placebo patch) × 2 (sensorimotor: smoke very low nicotine content cigarettes versus no smoking) full factorial between-subjects design. Participants repeatedly completed measures of craving, affect, and anticipated pleasure from and desire for rewarding experiences, followed by a smoking lapse analog task.RESULTSReceiving nicotine (versus placebo) increased positive affect and anticipated pleasure from and desire for reward. Expecting nicotine (versus placebo) reduced negative affect and increased smoking delay. Sensorimotor stimulation from smoking (versus no smoking) reduced smoking urge and behavior.CONCLUSIONResults provided initial validation of this novel three-mechanism design. This design can be used in the future to advance understanding and treatment of tobacco withdrawal. |
| 607 | Impey, Danielle, El-Marj, Nicole, Parks, Andrea, Choueiry, Joelle, Fisher, Derek, Knott, Verner J. Mismatch negativity in tobacco-naïve cannabis users and its alteration with acute nicotine administration. Pharmacology, biochemistry, and behavior. 2015;136:73.  **Abstract:** Chronic cannabis use may interact with factors, such as age of onset of cannabis use, family history, and genetic factors, to elicit schizophrenia (SZ)-like symptoms, including sensory and cognitive deficits. However, evidence of a relationship between cannabis use and cognitive impairment is confounded by concomitant use of tobacco. The objective of this study was to compare tobacco-naïve cannabis users with individuals without a history of tobacco/cannabis use on the auditory mismatch negativity (MMN) event-related potential (ERP), a neural measure of auditory deviance detection which is diminished in SZ. An exploratory arm of the study, conducted within a randomized, double-blind, placebo controlled design, examined the acute effects of nicotine gum (6mg) on MMN in cannabis users. MMN was recorded in response to 5 deviant stimuli within an optimal MMN paradigm in 44 healthy, non-tobacco smoking volunteers aged 18-26. Cannabis users (n=21) started smoking cannabis prior to age 17, at least 1 joint per month. To examine the effects of chronicity, users were grouped into relatively heavy long-term (HLT; n=11) users and light short-term (LST; n=10) users. Impaired deviance detection was shown in cannabis users vs. nonusers as reflected by a smaller MMN to duration deviants. Chronicity of use was also associated with MMN alterations, as HLTs displayed a reduced duration and gap MMN vs. LSTs. Compared with placebo, nicotine treatment enhanced select MMN deviants in cannabis user subgroups. As deficits associated with early and persistent cannabis use are similar to those seen in SZ, these dose-dependant disturbances in early sensory processing with cannabis use may be one cognitive pathway which mediates an increased risk for SZ in vulnerable youth, and be influenced by concurrent cigarette smoking behavior. |
| 616 | Bernstein, Steven L, D'Onofrio, Gail, Rosner, June, O'Malley, Stephanie, Makuch, Robert, Busch, Susan, Pantalon, Michael V, Toll, Benjamin. Successful Tobacco Dependence Treatment in Low-Income Emergency Department Patients: A Randomized Trial. Annals of emergency medicine. 2015;66:140.  **Abstract:** STUDY OBJECTIVETobacco use is common among emergency department (ED) patients, many of whom have low income. Our objective is to study the efficacy of an intervention incorporating motivational interviewing, nicotine replacement, and quitline referral for adult smokers in an ED.METHODSThis was a 2-arm randomized clinical trial conducted from October 2010 to December 2012 in a northeastern urban US ED with 90,000 visits per year. Eligible subjects were aged 18 years or older, smoked, and were self-pay or had Medicaid insurance. Intervention subjects received a motivational interview by a trained research assistant, 6 weeks' worth of nicotine patches and gum initiated in the ED, a faxed referral to the state smokers' quitline, a booster call, and a brochure. Control subjects received the brochure, which provided quitline information. The primary outcome was biochemically confirmed tobacco abstinence at 3 months. Secondary endpoints included quitline use.RESULTSOf 778 enrolled subjects, 774 (99.5%) were alive at 3 months. The prevalence of biochemically confirmed abstinence was 12.2% (47/386) in the intervention arm versus 4.9% (19/388) in the control arm, for a difference in quit rates of 7.3% (95% confidence interval 3.2% to 11.5%). In multivariable logistic modeling controlling for age, sex, and race or ethnicity, study subjects remained more likely to be abstinent than controls (odds ratio 2.72; 95% confidence interval 1.55 to 4.75).CONCLUSIONAn intensive intervention improved tobacco abstinence rates in low-income ED smokers. Because approximately 20 million smokers, many of whom have low income, visit US EDs annually, these results suggest that ED-initiated treatment may be an effective technique to treat this group of smokers. |
| 618 | Ben Taleb, Ziyad, Ward, Kenneth D, Asfar, Taghrid, Bahelah, Raed, Maziak, Wasim. Predictors of adherence to pharmacological and behavioral treatment in a cessation trial among smokers in Aleppo, Syria. Drug and alcohol dependence. 2015;153:167.  **Abstract:** INTRODUCTIONThe development of evidence-based smoking cessation programs is in its infancy in developing countries, which continue to bear the main brunt of the tobacco epidemic. Adherence to treatment recommendations is an important determinant of the success of smoking cessation programs, but little is known about factors influencing adherence to either pharmacological or behavioral treatment in developing countries settings. Our study represents the first attempt to examine the predictors of adherence to cessation treatment in a low-income developing country.METHODSPredictors of adherence to pharmacological and behavioral treatment were identified by analyzing data from a multi-site, two-group, parallel-arm, double-blind, randomized, placebo-controlled smoking cessation trial in primary care clinics in Aleppo, Syria. Participants received 3 in-person behavioral counseling sessions plus 5 brief follow-up phone counseling sessions, and were randomized to either 6 weeks of nicotine or placebo patch.RESULTSOf the 269 participants, 68% adhered to pharmacological treatment, while 70% adhered to behavioral counseling. In logistic regression modeling, lower adherence to pharmacological and behavioral treatment was associated with higher daily smoking at baseline, greater withdrawal symptoms, and perception of receiving placebo instead of active nicotine patch. Women showed lower adherence than men to behavioral treatment, while being assigned to placebo condition and baseline waterpipe use were associated with lower adherence to pharmacological treatment.CONCLUSIONAdherence to cessation treatment for cigarette smokers in low-income countries such as Syria may benefit from integrated cessation components that provide intensive treatment for subjects with higher nicotine dependence, and address concurrent waterpipe use at all stages. |
| 622 | McCarthy, Danielle E, Ebssa, Lemma, Witkiewitz, Katie, Shiffman, Saul. Paths to tobacco abstinence: A repeated-measures latent class analysis. Journal of consulting and clinical psychology. 2015;83:696.  **Abstract:** OBJECTIVEKnowledge of smoking change processes may be enhanced by identifying pathways to stable abstinence. We sought to identify latent classes of smokers based on their day-to-day smoking status in the first weeks of a cessation attempt. We examined treatment effects on class membership and compared classes on baseline individual differences and 6-month abstinence rates.METHODIn this secondary analysis of a double-blind randomized placebo-controlled clinical trial (N = 1,433) of 5 smoking cessation pharmacotherapies (nicotine patch, nicotine lozenge, bupropion SR, patch and lozenge, or bupropion SR and lozenge), we conducted repeated-measures latent class analysis of daily smoking status (any smoking vs. none) for the first 27 days of a quit attempt. Treatment and covariate relations with latent class membership were examined. Distal outcome analysis compared confirmed 6-month abstinence rates among the latent classes.RESULTSA 5-class solution was selected. Three-quarters of smokers were in stable smoking or abstinent classes, but 25% were in classes with unstable abstinence probabilities over time. Active treatment (compared to placebo), and particularly the patch and lozenge combination, promoted early quitting. Latent classes differed in 6-month abstinence rates and on several baseline variables, including nicotine dependence, quitting history, self-efficacy, sleep disturbance, and minority status.CONCLUSIONSRepeated-measures latent class analysis identified latent classes of smoking change patterns affected by treatment, related to known risk factors, and predictive of distal outcomes. Tracking behavior early in a change attempt may identify prognostic patterns of change and facilitate adaptive treatment planning. |
| 633 | Kroemer, Nils B., Wuttig, Franziska, Bidlingmaier, Martin, Zimmermann, Ulrich S., Smolka, Michael N.. Nicotine enhances modulation of food-cue reactivity by leptin and ghrelin in the ventromedial prefrontal cortex. Addiction Biology. 2015;20:832.  **Abstract:** Endocrine signals such as ghrelin and leptin are known to modulate the mesocorticolimbic dopaminergic system and, consequently, show associations with food and drug reward. In animal models, nicotine was demonstrated to reduce body weight by attenuating food intake and effects of leptin and ghrelin are partly modulated by nicotinic acetylcholine receptors which hint at potential interactions. However, the neuropharmacological modulation of endocrine signals by nicotine in healthy humans remains to be tested experimentally. We used functional magnetic resonance imaging to investigate food-cue reactivity after an overnight fast and following a caloric load (oral glucose tolerance test, OGTT) in 26 healthy normal-weight never-smokers. Moreover, we administered either nicotine (2mg) or placebo gums using a randomized cross-over design and assessed blood plasma levels of ghrelin and leptin. During fasting, nicotine administration decreased correlations with ghrelin levels in the mesocorticolimbic system whereas correlations with leptin were increased. After the OGTT, nicotine increased the modulatory effects of ghrelin and leptin on food-cue reactivity, particularly in the ventromedial prefrontal cortex (vmPFC) and the amygdala. Critically, this led to an indirect modulation of the behavioral 'appetizer effect' (i.e. cue-induced increases in subjective appetite) by homeostatic feedback signals via food-cue reactivity in vmPFC. We conclude that nicotine enhances the effect of ghrelin and leptin in the valuation and relevance network which might, in turn, reduce appetite. This highlights that amplifying the impact of homeostatic signals such as ghrelin and leptin in normal-weight individuals might hint at a mechanism contributing to nicotine's anorexic potential. Nicotine reduces body weight by attenuating food intake, but little is known about potential interactions with homeostatic signals such as leptin and ghrelin that regulate ingestive behavior. Following nicotine administration versus placebo, we investigated food-cue reactivity using functional magnetic resonance imaging. Nicotine enhanced the modulatory effects of leptin and ghrelin on food-cue reactivity, particularly in ventromedial prefrontal cortex and amygdala. We conclude that enhanced modulation of food-cue reactivity by homeostatic signals could contribute to the anorexic potential of nicotine. |
| 634 | Bowers, H, Smith, D, de la Salle, S, Choueiry, J, Impey, D, Philippe, T, Dort, H, Millar, A, Daigle, M, Albert, P R, Beaudoin, A, Knott, V. COMT polymorphism modulates the resting-state EEG alpha oscillatory response to acute nicotine in male non-smokers. Genes, brain, and behavior. 2015;14:466.  **Abstract:** Performance improvements in cognitive tasks requiring executive functions are evident with nicotinic acetylcholine receptor (nAChR) agonists, and activation of the underlying neural circuitry supporting these cognitive effects is thought to involve dopamine neurotransmission. As individual difference in response to nicotine may be related to a functional polymorphism in the gene encoding catechol-O-methyltransferase (COMT), an enzyme that strongly influences cortical dopamine metabolism, this study examined the modulatory effects of the COMT Val158Met polymorphism on the neural response to acute nicotine as measured with resting-state electroencephalographic (EEG) oscillations. In a sample of 62 healthy non-smoking adult males, a single dose (6 mg) of nicotine gum administered in a randomized, double-blind, placebo-controlled design was shown to affect α oscillatory activity, increasing power of upper α oscillations in frontocentral regions of Met/Met homozygotes and in parietal/occipital regions of Val/Met heterozygotes. Peak α frequency was also found to be faster with nicotine (vs. placebo) treatment in Val/Met heterozygotes, who exhibited a slower α frequency compared to Val/Val homozygotes. The data tentatively suggest that interindividual differences in brain α oscillations and their response to nicotinic agonist treatment are influenced by genetic mechanisms involving COMT. |
| 638 | Gray, Kevin M, McClure, Erin A, Baker, Nathaniel L, Hartwell, Karen J, Carpenter, Matthew J, Saladin, Michael E. An exploratory short-term double-blind randomized trial of varenicline versus nicotine patch for smoking cessation in women. Addiction (Abingdon, England). 2015;110:1027.  **Abstract:** AIMSWithin a parent study examining ovarian hormone effects on smoking cessation in women, we conducted an exploratory short-term trial of varenicline versus transdermal nicotine patch.DESIGNDouble-blind double-dummy randomized trial.SETTINGSingle-site out-patient research clinic in the United States.PARTICIPANTSFemale smokers, ages 18-45 years and averaging ≥10 cigarettes per day for at least 6 months (n=140).INTERVENTIONSParticipants were randomized to receive a 4-week course of (a) varenicline tablets and placebo patches (n = 67) or (b) placebo tablets and nicotine patches (n=73). Two brief cessation counseling sessions were provided for all participants.MEASUREMENTSThe outcome of primary clinical interest was 2-week end-of-treatment abstinence. Secondary outcomes included 1- and 4-week end-of treatment abstinence and abstinence at a post-treatment follow-up visit occurring 4 weeks after treatment conclusion. Breath carbon monoxide (≤ 10 parts per million) was used to confirm biochemically self-reported abstinence.FINDINGSTwo-week end-of-treatment abstinence was achieved by 37.3% (25 of 67) of varenicline participants and by 17.8% (13 of 73) of nicotine patch participants [odds ratio (OR) = 2.7, 95% confidence interval (CI)=1.3-6.0, P=0.011]. One-week (44.8 versus 20.6%, OR=3.1, 95% CI=1.5-6.6, P=0.003) and 4-week (22.4 versus 9.6%, OR=2.7, 95% CI=1.0-7.2, P=0.043) end-of-treatment abstinence similarly favored varenicline, although post-treatment follow-up Russell Standard abstinence was not significantly different between groups (23.9 versus 13.7%, OR=2.0, 95% CI=0.8-4.7, P=0.126).CONCLUSIONIn an exploratory 4-week head-to-head trial in female smokers, varenicline, compared with nicotine patch, more than doubled the odds of end-of-treatment abstinence, although this diminished somewhat at post-treatment follow-up. |
| 641 | Essex, Holly N, Parrott, Steve, Wu, Qi, Li, Jinshuo, Cooper, Sue, Coleman, Tim. Cost-Effectiveness of Nicotine Patches for Smoking Cessation in Pregnancy: A Placebo Randomized Controlled Trial (SNAP). Nicotine & tobacco research : official journal of the Society for Research on Nicotine and Tobacco. 2015;17:636.  **Abstract:** INTRODUCTIONSmoking during pregnancy is the most important, preventable cause of adverse pregnancy outcomes including miscarriage, premature birth, and low birth weight with huge financial costs to the National Health Service. However, there are very few published economic evaluations of smoking cessation interventions in pregnancy and previous studies are predominantly U.S.-based and do not present incremental cost-effectiveness ratios (ICER). A number of studies have demonstrated cost-effectiveness of nicotine replacement therapy (NRT) in the general population, but this has yet to be tested among pregnant smokers.METHODSA cost-effectiveness analysis was undertaken alongside the smoking, nicotine, and pregnancy trial to compare NRT patches plus behavioral support to behavioral support alone, for pregnant women who smoked.RESULTSAt delivery, biochemically verified quit rates were slightly higher at 9.4% in the NRT group compared to 7.6% in the control group (odds ratio = 1.26, 95% CI = 0.82-1.96), at an increased cost of around £90 per participant. Higher costs in the NRT group were mainly attributable to the cost of NRT patches (mean = £46.07). The incremental cost-effectiveness ratio associated with NRT was £4,926 per quitter and a sensitivity analysis including only singleton births yielded an ICER of £4,156 per quitter. However, wide confidence intervals indicated a high level of uncertainty.CONCLUSIONSWithout a specific willingness to pay threshold, and due to high levels of statistical uncertainty, it is hard to determine the cost-effectiveness of NRT in this population. Furthermore, future research should address compliance issues, as these may dilute any potential effects of NRT, thus reducing the cost-effectiveness. |
| 644 | Smucny, Jason, Olincy, Ann, Eichman, Lindsay S, Tregellas, Jason R. Neuronal effects of nicotine during auditory selective attention. Psychopharmacology. 2015;232:2017.  **Abstract:** RATIONALEAlthough the attention-enhancing effects of nicotine have been behaviorally and neurophysiologically well-documented, its localized functional effects during selective attention are poorly understood.OBJECTIVESIn this study, we examined the neuronal effects of nicotine during auditory selective attention in healthy human nonsmokers. We hypothesized to observe significant effects of nicotine in attention-associated brain areas, driven by nicotine-induced increases in activity as a function of increasing task demands.METHODSA single-blind, prospective, randomized crossover design was used to examine neuronal response associated with a go/no-go task after 7 mg nicotine or placebo patch administration in 20 individuals who underwent functional magnetic resonance imaging at 3T. The task design included two levels of difficulty (ordered vs. random stimuli) and two levels of auditory distraction (silence vs. noise).RESULTSSignificant treatment × difficulty × distraction interaction effects on neuronal response were observed in the hippocampus, ventral parietal cortex, and anterior cingulate. In contrast to our hypothesis, U and inverted U-shaped dependencies were observed between the effects of nicotine on response and task demands, depending on the brain area.CONCLUSIONSThese results suggest that nicotine may differentially affect neuronal response depending on task conditions. These results have important theoretical implications for understanding how cholinergic tone may influence the neurobiology of selective attention. |
| 648 | Bernstein, Steven L., Rosner, June, Toll, Benjamin. Texting to promote tobacco abstinence in emergency department smokers: A pilot study. Academic Emergency Medicine. 2015;22:S74.  **Abstract:** Background: ED patients have a high smoking prevalence. Current treatment approaches use motivational interviewing, which is effective, but resource-intensive. Mobile health approaches may be more generalizable. Objectives: To assess the feasibility of an ED-initiated program of tobacco dependence treatment that employs text messaging. Methods: Smokers age 18 or older were randomized to intervention or control arms. Control subjects received a brochure describing the state smokers' quitline. Intervention subjects received the brochure, four weeks of nicotine patches and gum, with the initial dose administered in the ED, a faxed referral to the quitline, and enrollment in SmokeFreeTxt, a free SMS-messaging service developed by the National Cancer Institute. SmokeFreeTxt delivered 28 days of messages, 2-5 messages/day. Message content adapts principles of cognitive behavioral therapy. Some messages ask subjects to provide data on mood or craving. Subjects received at enrollment; texting subjects received an additional to offset potential costs to cellphone plans. Follow-up was by self-reported phone call. Subjects lost to follow-up were considered to be smoking. Data were analyzed with Microsoft Excel and SPSS 19.0. Results: 60 subjects were enrolled in May 2014. Of all subjects, 30 (50%) were female, 27 (45%) were white, 33 (55%) nonwhite, mean age 40 years (SD 11), and insurance coverage of Medicaid, self-pay, or other, respectively, of 78%, 8%, 14%. All intervention subjects used the texting program, with 24/30 (80%) using the program for all 28 days; 6 subjects opted out at some point. At one month, 14/30 subjects (47%) in the intervention arm reported tobacco abstinence, vs. 3/30 (10%) in the control arm (P=0.003). At three months, the self-reported abstinence rates in the intervention and control arms was, respectively, 9/30 (30%) and 4/30 (13%) (P=0.21). Conclusion: A texting program, combined with pharmacotherapy and a quitline referral, shows promise to promote tobacco abstinence in ED smokers. These data will provide an effect size to power a larger randomized clinical trial. |
| 657 | Saladin, Michael E, McClure, Erin A, Baker, Nathaniel L, Carpenter, Matthew J, Ramakrishnan, Viswanathan, Hartwell, Karen J, Gray, Kevin M. Increasing progesterone levels are associated with smoking abstinence among free-cycling women smokers who receive brief pharmacotherapy. Nicotine & tobacco research : official journal of the Society for Research on Nicotine and Tobacco. 2015;17:398.  **Abstract:** INTRODUCTIONPreclinical and human laboratory research suggests that (a) progesterone may decrease drug reward, craving, and smoking behavior, and (b) estradiol may enhance drug reward and smoking behavior. A modest majority of treatment research examining the relationship between menstrual cycle phase and outcomes suggests that the luteal menstrual phase, with its uniquely higher progesterone levels, is associated with better cessation outcomes. However, no studies to date have examined the effects of naturally occurring variation in progesterone and estradiol levels on medication-assisted smoking cessation. The present study sought to fill this notable gap in the treatment literature.METHODSWeekly plasma progesterone and estradiol levels were obtained from nicotine-dependent female smokers enrolled in a 4-week cessation trial. Participants (N = 108) were randomized to receive a 4-week course of either varenicline (VAR) tablets and placebo patches or placebo tablets and nicotine patches. Plasma samples were obtained 1 week before their cessation attempt and weekly during medication administration. Abstinence was assessed weekly.RESULTSWeekly hormone data replicated commonly observed menstrual cycle patterns of progesterone and estradiol levels. Importantly, increases in progesterone level were associated with a 23% increase in the odds for being abstinent within each week of treatment. This effect was driven primarily by nicotine patch-treated versus VAR-treated females.CONCLUSIONSThis study was the first to identify an association between progesterone level (increasing) and abstinence outcomes in free-cycling women smokers who participated in a medication-based treatment. Furthermore, the potential benefits of progesterone may vary across different pharmacotherapies. Implications of these findings for smoking cessation intervention are discussed. |
| 661 | O'Brien, Brigid, Knight-West, Oliver, Walker, Natalie, Parag, Varsha, Bullen, Christopher. E-cigarettes versus NRT for smoking reduction or cessation in people with mental illness: secondary analysis of data from the ASCEND trial. Tobacco induced diseases. 2015;13:5.  **Abstract:** BACKGROUNDPeople with mental illness have higher rates of smoking than the general population and are at greater risk of smoking-related death and disability. In smokers from the general population, electronic cigarettes (e-cigarettes) have been shown to have a similar effect on quit rates as nicotine replacement therapy, but little is known about their effect in smokers with mental illness.METHODSSecondary analysis of data from the ASCEND trial involving 657 dependent adult smokers motivated to quit, randomised to 16 mg nicotine e-cigarette, 21 mg nicotine patch, or 0 mg nicotine e-cigarette, with minimal behavioural support. Using self-reported medication use and the Anatomical Therapeutic Chemical Classification System, we identified 86 participants with mental illness and analysed their cessation and smoking reduction outcomes.RESULTSFor e-cigarettes alone, and all interventions pooled, there was no statistically significant difference in biochemically verified quit rates at six months between participants with and without mental illness, nor in smoking reduction, adverse events, treatment compliance, or acceptability. Rates of relapse to smoking were higher in participants with mental illness. Among this group, differences between treatments were not statistically significant for cessation (patch 14% [5/35], 16 mg e-cigarette 5% [2/39], 0 mg e-cigarette 0% [0/12], p = 0.245), adverse events or relapse rates. However, e-cigarette users had higher levels of smoking reduction, treatment compliance, and acceptability.CONCLUSIONSThe use of e-cigarettes for quitting appears to be equally effective, safe, and acceptable for people with and without mental illness. For people with mental illness, e-cigarettes may be as effective and safe as patches, yet more acceptable, and associated with greater smoking reduction.TRIAL REGISTRATIONAustralian New Zealand Clinical trials Registry, number: ACTRN12610000866000. |
| 662 | Minami, Haruka, Kahler, Christopher W., Bloom, Erika L., Strong, David R., Abrantes, Ana M., Zywiak, William H., Price, Lawrence H., Brown, Richard A.. Effects of depression history and sex on the efficacy of sequential versus standard Fluoxetine for smoking cessation in elevated depressive symptom smokers. Addictive Disorders and their Treatment. 2015;14:29.  **Abstract:** Objective: The potential benefits of antidepressant pharmacotherapy with fluoxetine for smoking cessation among vulnerable subpopulations of depressed smokers have not been well explored. This study examined whether the efficacy of a sequential course of fluoxetine for smoking cessation differed as a function of depression history (none vs. single vs. recurrent episodes) and sex. Methods: Data were from a randomized controlled trial that evaluated the efficacy of sequential fluoxetine treatment (SEQ-FLUOX; 16 wk, starting 8 wk prequit) in comparison with standard fluoxetine treatment (ST-FLUOX; 10 wk, starting 2 wk prequit) and transdermal nicotine patch only (TNP) for 216 smokers with elevated depressive symptoms. Results: Cox regression analyses revealed significant moderating effects of depression history (recurrent vs. single), but not sex, on the efficacy of SEQ-FLUOX versus ST-FLUOX on latency to relapse. Among smokers with recurrent major depressive disorder (MDD) episodes, those receiving SEQ-FLUOX were slower to relapse, compared with those receiving ST-FLUOX, but not relative to TNP. No such treatment difference was observed in smokers with no MDD history or a single past MDD episode. Furthermore, those with recurrent MDD receiving SEQ-FLUOX reported significantly lower levels of depressive symptoms over 26 weeks after quitting, compared with those receiving ST-FLUOX, but not relative to TNP. Conclusions: Findings suggest the possible specific benefits of treating elevated depressive symptom smokers with recurrent MDD using fluoxetine in a sequential manner (vs. standard). However, given the small sample size, the reliability of this difference is unknown. Replication with a larger sample of recurrent MDD history is needed. © |
| 665 | Danaher, Brian G., Severson, Herbert H., Crowley, Ryann, van Meter, Nora, Tyler, Milagra S., Widdop, Chris, Lichtenstein, Edward, Ebbert, Jon O.. Randomized controlled trial examining the adjunctive use of nicotine lozenges with MyLastDip: An eHealth smokeless tobacco cessation intervention. Internet Interventions. 2015;2:69.  **Abstract:** Introduction: Promising Web-based interventions for smokeless tobacco cessation have emerged. We describe a randomized controlled trial (RCT) testing the relative benefits of adding the nicotine lozenge as an adjunct to the MyLastDip Web-based smokeless tobacco cessation intervention. Methods: 407 smokeless tobacco users who wanted to quit were recruited, screened online, and randomly assigned to one of two conditions: (a) the interactive MyLastDip Web-based intervention (Web Only; n = 202), or (b) the website plus the offer of nicotine lozenges (Web. +. Lozenge; n = 205). MyLastDip program content is grouped according to three sequential frames: preparing to quit, quitting, and staying quit. If a participant reported a lapse then the program would provide tailored content on lessons learned and starting over ("retooling"). The primary outcome was 7-day point prevalence tobacco abstinence measured at follow-up assessments that occurred 3. months and 6. months post-enrollment. Results: Assessment completion rates were 71.5% at 3. months, 72.9% at 6. months, and 65.1% for both 3 and 6. months, and did not differ by condition. Using Intent to Treat analyses, the Web. +. Lozenge condition was associated with a significantly higher 7-day point prevalence tobacco abstinence rate than the Web Only condition at 3. months (43.4% vs. 29.7%, p = .004), at the combined 3 and 6. month assessment of repeated point prevalence (35.6% vs. 23.3%, p = .007), but not at 6. months (44.4% vs. 35.1%, p = .057). Similar results were obtained for smokeless tobacco abstinence. Participants reported being satisfied with their programs and the Web. +. Lozenge condition participants visited the MyLastDip program more often (p<. .001). A composite engagement measure of the number and duration of program visits was positively related to 6-month tobacco abstinence (p = .009). Conclusions: Consistent with previous research, the MyLastDip Web-based tobacco cessation intervention encouraged long-term levels of tobacco and smokeless tobacco abstinence. The addition of nicotine lozenges significantly improved both participant engagement and self-reported 7-day point prevalence tobacco abstinence at 3. months and when considering 3- and 6-month repeated point prevalence tobacco abstinence. |
| 666 | Severson, Herbert H, Danaher, Brian G, Ebbert, Jon O, van Meter, Nora, Lichtenstein, Edward, Widdop, Chris, Crowley, Ryann, Akers, Laura, Seeley, John R. Randomized trial of nicotine lozenges and phone counseling for smokeless tobacco cessation. Nicotine & tobacco research : official journal of the Society for Research on Nicotine and Tobacco. 2015;17:309.  **Abstract:** INTRODUCTIONRelatively few treatment programs have been developed specifically for smokeless tobacco (ST) users who want to quit. Their results suggest that self-help materials, telephone counseling, and nicotine lozenges are efficacious. This study provides the first direct examination of the separate and combined effects of telephone counseling and lozenges.METHODSWe recruited ST users online (N = 1067) and randomly assigned them to 1 of 3 conditions: (a) a lozenge group (n = 356), who were mailed 4-mg nicotine lozenges; (b) a coach calls group (n = 354), who were offered 3 coaching phone calls; or (c) a lozenge + coach calls group (N = 357), who received both lozenges and coaching calls. Additionally, all participants were mailed self-help materials. Self-reported tobacco abstinence was assessed at 3 and 6 months after randomization.RESULTSComplete-case and intention-to-treat (ITT) analyses for all tobacco abstinence were performed at 3 months, 6 months, and both 3 and 6 months (repeated point prevalence). ITT analyses revealed a highly similar result: the lozenge + coach calls condition was significantly more successful in encouraging tobacco abstinence than either the lozenge group or the coach calls group, which did not differ.CONCLUSIONSCombining nicotine lozenges and phone counseling significantly increased tobacco abstinence rates compared with either intervention alone, whereas coach calls and lozenges were equivalent. The study confirms the high tobacco abstinence rates for self-help ST cessation interventions and offers guidance to providing tobacco treatment to ST users. |
| 673 | Haas, Jennifer S, Linder, Jeffrey A, Park, Elyse R, Gonzalez, Irina, Rigotti, Nancy A, Klinger, Elissa V, Kontos, Emily Z, Zaslavsky, Alan M, Brawarsky, Phyllis, Marinacci, Lucas X, St Hubert, Stella, Fleegler, Eric W, Williams, David R. Proactive tobacco cessation outreach to smokers of low socioeconomic status: a randomized clinical trial. JAMA internal medicine. 2015;175:218.  **Abstract:** IMPORTANCEWidening socioeconomic disparities in mortality in the United States are largely explained by slower declines in tobacco use among smokers of low socioeconomic status (SES) than among those of higher SES, which points to the need for targeted tobacco cessation interventions. Documentation of smoking status in electronic health records (EHRs) provides the tools for health systems to proactively offer tobacco treatment to socioeconomically disadvantaged smokers.OBJECTIVETo evaluate a proactive tobacco cessation strategy that addresses sociocontextual mediators of tobacco use for low-SES smokers.DESIGN, SETTING, AND PARTICIPANTSThis prospective, randomized clinical trial included low-SES adult smokers who described their race and/or ethnicity as black, Hispanic, or white and received primary care at 1 of 13 practices in the greater Boston area (intervention group, n = 399; control group, n = 308).INTERVENTIONSWe analyzed EHRs to identify potentially eligible participants and then used interactive voice response (IVR) techniques to reach out to them. Consenting patients were randomized to either receive usual care from their own health care team or enter an intervention program that included (1) telephone-based motivational counseling, (2) free nicotine replacement therapy (NRT) for 6 weeks, (3) access to community-based referrals to address sociocontextual mediators of tobacco use, and (4) integration of all these components into their normal health care through the EHR system.MAIN OUTCOMES AND MEASURESSelf-reported past-7-day tobacco abstinence 9 months after randomization ("quitting"), assessed by automated caller or blinded study staff.RESULTSThe intervention group had a higher quit rate than the usual care group (17.8% vs 8.1%; odds ratio, 2.5; 95% CI, 1.5-4.0; number needed to treat, 10). We examined whether use of intervention components was associated with quitting among individuals in the intervention group: individuals who participated in the telephone counseling were more likely to quit than those who did not (21.2% vs 10.4%; P < .001). There was no difference in quitting by use of NRT. Quitting did not differ by a request for a community referral, but individuals who used their referral were more likely to quit than those who did not (43.6% vs 15.3%; P < .001).CONCLUSIONS AND RELEVANCEProactive, IVR-facilitated outreach enables engagement with low-SES smokers. Providing counseling, NRT, and access to community-based resources to address sociocontextual mediators among smokers reached in this setting is effective.TRIAL REGISTRATIONclinicaltrials.gov Identifier: NCT01156610. |
| 674 | Cook, Jessica W, Piper, Megan E, Leventhal, Adam M, Schlam, Tanya R, Fiore, Michael C, Baker, Timothy B. Anhedonia as a component of the tobacco withdrawal syndrome. Journal of abnormal psychology. 2015;124:215.  **Abstract:** Animal research suggests that anhedonia is a tobacco withdrawal symptom, but this topic has not been addressed definitively in research with humans. This research sought to determine whether anhedonia is (a) an element of the tobacco withdrawal syndrome in humans and (b) an impediment to successful tobacco cessation. Data were from 1,175 smokers (58.3% women; 85.5% White) participating in a randomized double-blind, placebo-controlled trial of smoking cessation pharmacotherapies. Ecological momentary assessments for 5 days before and 10 days after the target quit day were used to assess anhedonia and other established withdrawal symptoms. Consistent with drug withdrawal, anhedonia showed an inverted-U pattern of change in response to tobacco cessation and was associated with the severity of other withdrawal symptoms and tobacco dependence. Postquit anhedonia was associated with decreased latency to relapse (hazard ratio = 1.09, 95% confidence interval [CI] [1.02, 1.17]) and with lower 8-week point-prevalence abstinence (odds ratio = .91, 95% CI [.86, .97])-relations that remained significant when other withdrawal symptoms were included as predictors. Finally, nicotine replacement therapy nearly fully suppressed the increase in abstinence-related anhedonia (β = -.66, p < .001), suggesting agonist suppression of withdrawal. Results suggest that anhedonia is a unique and motivationally significant element of the tobacco withdrawal syndrome in humans. These results have implications for defining and assessing tobacco use disorder and for understanding and treating tobacco addiction. |
| 675 | Bhatia, S., Waller, J.L., Tingen, M.. Examining the effectiveness of a multi-level intervention on parental self-efficacy for delivering an anti-tobacco socialization message in a predominantly African American population. Journal of Investigative Medicine. 2015;63:461.  **Abstract:** Purpose of Study: Tobacco use kills 480,000 people every year. Recent studies have shown that higher parental self-efficacy in delivering an anti-tobacco socialization message can reduce teen smoking by 28%. However, self-efficacy in a minority population has not been thoroughly investigated and intervention programs tailored to minorities are lacking. The purpose of this study was to investigate the effect of a new tobacco cessation intervention on parental self-efficacy in a largely African American population. Methods Used: The study participants included 4th graders (n=329) and at least one parent or guardian (n=344) from both rural and urban settings. The participants were randomly assigned to control or intervention groups. The control group received a traditional health class for children and general health information for parents, including self-help information and contact information for the state toll-free Quit-Line for parent smokers. The intervention group received school and home-based interventions aimed at promoting the development of healthy behaviors, specifically tobacco prevention, as well as parental self-efficacy. Smokers in the intervention group also received nicotine replacement therapy and tailored motivational interviewing via telephone or face-to-face for 8 weeks. Parental-self efficacy was measured at pre and post intervention, using a 7- point Likert scale. Summary of Results: It was found that parental self-efficacy scores in the intervention group increased significantly (pre=63.1, post=65.1, p=0.0037), while there was no increase in the control group (pre=65.7, post=65.0, p=0.4399). Conclusions: These results suggest that a multi-level approach, which targets the family, may be effective in increasing parental self-efficacy and therefore help to reduce tobacco initiation in the homes of African Americans. |
| 677 | Lerman, Caryn, Schnoll, Robert A, Hawk, Larry W, Jr, Cinciripini, Paul, George, Tony P, Wileyto, E Paul, Swan, Gary E, Benowitz, Neal L, Heitjan, Daniel F, Tyndale, Rachel F. Use of the nicotine metabolite ratio as a genetically informed biomarker of response to nicotine patch or varenicline for smoking cessation: a randomised, double-blind placebo-controlled trial. The Lancet. Respiratory medicine. 2015;3:131.  **Abstract:** BACKGROUNDSubstantial variability exists in therapeutic response and adverse effects with pharmacotherapies for tobacco dependence. Biomarkers to optimise treatment choice for individual smokers might improve treatment outcomes. We tested whether a genetically informed biomarker of nicotine clearance, the nicotine metabolite ratio (NMR; 3'-hydroxycotinine:cotinine), predicts response to nicotine patch or varenicline for smoking cessation.METHODSWe undertook NMR-stratified multicentre, randomised, placebo-controlled clinical trial from Nov 16, 2010, to Sept 12, 2014, at four sites. Smokers seeking treatment were randomly assigned by baseline NMR status and study site, in blocks of 12 patients (1:1:1 ratio), to 11 weeks of placebo (placebo pill plus placebo patch), nicotine patch (active patch plus placebo pill), or varenicline (active pill plus placebo patch), plus behavioural counselling. Participants and investigators were masked to group allocation and NMR status. An intention-to-treat analysis was done. Participants were followed up for 12 months after the target quit date. The primary endpoint was biochemically verified 7 day point prevalence abstinence at the end of treatment to estimate the pharmacological effect of treatment by NMR. The trial is registered at ClinicalTrials.gov, number NCT01314001.FINDINGS1246 participants (662 slow metabolisers of nicotine, 584 normal metabolisers of nicotine) were enrolled and randomly assigned to the three interventions (408 placebo, 418 nicotine patch, 420 varenicline). At end of treatment, varenicline was more efficacious than nicotine patch in normal metabolisers (OR 2·17, 95% CI 1·38-3·42; p=0·001), but not in slow metabolisers (OR 1·13, 0·74-1·71; p=0·56). In the longitudinal model including all timepoints, the NMR-by-treatment interaction was significant (ratio of odds ratios [ORR] 1·96, 95% CI 1·11-3·46; p=0·02). An NMR-by-treatment interaction showed that slow (vs normal) metabolisers reported greater overall side-effect severity with varenicline versus placebo (β=-1·06, 95% CI -2·08 to -0·03; p=0·044).INTERPRETATIONTreating normal metabolisers with varenicline and slow metabolisers with nicotine patch could optimise quit rates while minimising side-effects.FUNDINGNational Institutes of Health, Canadian Institutes of Health Research, Abramson Cancer Center, Centre for Addiction and Mental Health Foundation, and Pennsylvania Department of Health. |
| 681 | Vorel, Stanislav R., Mahoney, Melissa, Bisaga, A.. Influence of nicotine replacement therapy on heart rate variability and relapse. Drug and Alcohol Dependence. 2015;146:e12.  **Abstract:** Aims: Research has shown nicotine replacement therapy (NRT) to increase heart rate variability (HRV) in smokers and increased HRV has been shown to negatively predict relapse. The present study investigates the relationship between HRV and relapse across multiple doses of NRT. Methods: Participants were 24 tobacco smokers (17 male, 7 female) who were not seeking treatment. After overnight abstinence, they received NRT dose (0 mg, 7 mg, 21 mg, 42 mg) in a randomized double-blind fashion with each receiving 3 of 4 possible doses on different days, 1 week apart. No smoking was allowed during the study day. Time domain HRV variability was measured using a three-channel ECG during presentations of cigarette cues (a lit cigarette), which occurred during 1-hour intervals at 7 time points. Relapse was measured as the amount of money a subject would pay to smoke at the end of the research day. Participants who paid more could smoke sooner. Additionally, we measured nicotine levels at the end of each research day and breath CO levels the following afternoon. Results: In the 0, 7 and 21mg NRT conditions, HRV was shown to significantly increase over time, F(2, 23) = 4.616, p = .021. No significant time effect was found in the 42mg NRT condition. During cue presentation, HRV decreased dose-dependently across all NRT conditions, F(3,21) = 7.64, p = .001. Relapse rates decreased dose dependently F(2,21) = 4.042, p = .01 with relapse rates lowest at the 42mgdose. There was a significant correlation between decrease in HRV and relapse (r = .29, p < .05). Relapse was also negatively correlated with nicotine levels (r =-.25, p < .05). End of day nicotine levels were negatively correlated with CO measures (r =-.285) the following day. Conclusions: NRT may increase HRV in smokers during smoking cessation, but lower levels of HRV may be found with higher NRT doses and higher NRT doses may lead to lower relapse. Our preliminary results challenge the common notion that increased HRV is protective against relapse. |
| 686 | O'Brien, Brigid, Knight-West, Oliver, Walker, Natalie, Parag, Varsha, Bullen, Christopher. E-cigarettes versus NRT for smoking reduction or cessation in people with mental illness: Secondary analysis of data from the ASCEND trial Dr Maciej L. Goniewicz. Tobacco Induced Diseases. 2015; 13(1):5.  **Abstract:** Background: People with mental illness have higher rates of smoking than the general population and are at greater risk of smoking-related death and disability. In smokers from the general population, electronic cigarettes (e-cigarettes) have been shown to have a similar effect on quit rates as nicotine replacement therapy, but little is known about their effect in smokers with mental illness. Methods: Secondary analysis of data from the ASCEND trial involving 657 dependent adult smokers motivated to quit, randomised to 16 mg nicotine e-cigarette, 21 mg nicotine patch, or 0 mg nicotine e-cigarette, with minimal behavioural support. Using self-reported medication use and the Anatomical Therapeutic Chemical Classification System, we identified 86 participants with mental illness and analysed their cessation and smoking reduction outcomes. Results: For e-cigarettes alone, and all interventions pooled, there was no statistically significant difference in biochemically verified quit rates at six months between participants with and without mental illness, nor in smoking reduction, adverse events, treatment compliance, or acceptability. Rates of relapse to smoking were higher in participants with mental illness. Among this group, differences between treatments were not statistically significant for cessation (patch 14% [5/35], 16 mg e-cigarette 5% [2/39], 0 mg e-cigarette 0% [0/12], p∈=∈0.245), adverse events or relapse rates. However, e-cigarette users had higher levels of smoking reduction, treatment compliance, and acceptability. Conclusions: The use of e-cigarettes for quitting appears to be equally effective, safe, and acceptable for people with and without mental illness. For people with mental illness, e-cigarettes may be as effective and safe as patches, yet more acceptable, and associated with greater smoking reduction. Trial registration: Australian New Zealand Clinical trials Registry, number: ACTRN12610000866000. |
| 687 | Walker, Natalie, Howe, Colin, Glover, Marewa, McRobbie, Hayden, Barnes, Joanne, Nosa, Vili, Parag, Varsha, Bassett, Bruce, Bullen, Christopher. Cytisine versus nicotine for smoking cessation. The New England journal of medicine. 2014;371:2353.  **Abstract:** BACKGROUNDPlacebo-controlled trials indicate that cytisine, a partial agonist that binds the nicotinic acetylcholine receptor and is used for smoking cessation, almost doubles the chances of quitting at 6 months. We investigated whether cytisine was at least as effective as nicotine-replacement therapy in helping smokers to quit.METHODSWe conducted a pragmatic, open-label, noninferiority trial in New Zealand in which 1310 adult daily smokers who were motivated to quit and called the national quitline were randomly assigned in a 1:1 ratio to receive cytisine for 25 days or nicotine-replacement therapy for 8 weeks. Cytisine was provided by mail, free of charge, and nicotine-replacement therapy was provided through vouchers for low-cost patches along with gum or lozenges. Low-intensity, telephone-delivered behavioral support was provided to both groups through the quitline. The primary outcome was self-reported continuous abstinence at 1 month.RESULTSAt 1 month, continuous abstinence from smoking was reported for 40% of participants receiving cytisine (264 of 655) and 31% of participants receiving nicotine-replacement therapy (203 of 655), for a difference of 9.3 percentage points (95% confidence interval, 4.2 to 14.5). The effectiveness of cytisine for continuous abstinence was superior to that of nicotine-replacement therapy at 1 week, 2 months, and 6 months. In a prespecified subgroup analysis of the primary outcome, cytisine was superior to nicotine-replacement therapy among women and noninferior among men. Self-reported adverse events over 6 months occurred more frequently in the cytisine group (288 events among 204 participants) than in the group receiving nicotine-replacement therapy (174 events among 134 participants); adverse events were primarily nausea and vomiting and sleep disorders.CONCLUSIONSWhen combined with brief behavioral support, cytisine was found to be superior to nicotine-replacement therapy in helping smokers quit smoking, but it was associated with a higher frequency of self-reported adverse events. (Funded by the Health Research Council of New Zealand; Australian New Zealand Clinical Trials Registry number, ACTRN12610000590066.). |
| 691 | Smith, Stevens S, Fiore, Michael C, Baker, Timothy B. Smoking cessation in smokers who smoke menthol and non-menthol cigarettes. Addiction (Abingdon, England). 2014;109:2107.  **Abstract:** AIMSTo assess the relations of menthol cigarette use with measures of cessation success in a large comparative effectiveness trial (CET).DESIGNParticipants were randomized to one of six medication treatment conditions in a randomized double-blind, placebo-controlled clinical trial. All participants received six individual counseling sessions.SETTINGCommunity-based smokers in two communities in Wisconsin, USA.PARTICIPANTSA total of 1504 adult smokers who smoked at least 10 cigarettes per day during the past 6 months and reported being motivated to quit smoking. The analysis sample comprised 1439 participants: 814 white non-menthol smokers, 439 white menthol smokers and 186 African American (AA) menthol smokers. There were too few AA non-menthol smokers (n = 16) to be included in the analyses.INTERVENTIONSNicotine lozenge, nicotine patch, bupropion sustained release, nicotine patch + nicotine lozenge, bupropion + nicotine lozenge and placebo.MEASUREMENTSBiochemically confirmed 7-day point-prevalence abstinence assessed at 4, 8 and 26 weeks post-quit.FINDINGSIn longitudinal abstinence analyses (generalized estimating equations) controlling for cessation treatment, menthol smoking was associated with reduced likelihood of smoking cessation success relative to non-menthol smoking [model-based estimates of abstinence = 31 versus 38%, respectively; odds ratio (OR) = 0.71, 95% confidence interval (CI) = 0.59, 0.86]. In addition, among menthol smokers, AA women were at especially high risk of cessation failure relative to white women (estimated abstinence = 17 versus 35%, respectively; OR = 2.63, 95% CI = 1.75, 3.96; estimated abstinence rates for AA males and white males were both 30%, OR = 1.06, 95% CI = 0.60, 1.66).CONCLUSIONIn the United States, smoking menthol cigarettes appears to be associated with reduced cessation success compared with non-menthol smoking, especially in African American females. |
| 697 | Fraser, D, Kobinsky, K, Smith, S S, Kramer, J, Theobald, W E, Baker, T B. Five population-based interventions for smoking cessation: a MOST trial. Translational behavioral medicine. 2014;4:382.  **Abstract:** Little is known about the relative, additive, and interactive effects of different population-based treatments for smoking cessation. The goal of this study was to evaluate the main and interactive effects of five different smoking interventions. Using the multiphase optimization strategy (MOST), 1,034 smokers who entered a Web site for smokers (smokefree.gov) were randomly assigned to the "on" and "off" conditions of five smoking cessation interventions: the National Cancer Institute's (NCI) Web site (www.smokefree.gov vs a "lite" Web site), telephone quitline counseling (vs none), a smoking cessation brochure (vs a lite brochure), motivational e-mail messages (vs none), and mini-lozenge nicotine replacement therapy (NRT vs none). Analyses showed that the NCI Web site and NRT both increased abstinence; however, the former increased abstinence significantly only when it was not used with the e-mail messaging intervention (messaging decreased Web site use). The other interventions showed little evidence of effectiveness. There was evidence that mailed nicotine mini-lozenges and the NCI Web site (www.smokefree.gov) provide benefit as population-based smoking interventions. |
| 705 | de Dios, Marcel A, Anderson, Bradley J, Caviness, Celeste M, Stein, Michael D. Early quit days among methadone-maintained smokers in a smoking cessation trial. Nicotine & tobacco research : official journal of the Society for Research on Nicotine and Tobacco. 2014;16:1463.  **Abstract:** INTRODUCTIONMethadone maintenance treatment (MMT) patients have an exceedingly high prevalence of tobacco use, and interventions that have been specifically developed for this vulnerable subpopulation have struggled to attain even modest rates of cessation. A significant barrier has been an inability to initiate a quit attempt early in the treatment process and adherence to treatment.METHODSThis study examined the extent to which self-efficacy, medication adherence, and other demographic and smoking variables predicted an early quit day in a sample of MMT smokers (n = 315) enrolled in a smoking cessation pharmacotherapy trial. Using logistic regression, we estimated the association of having an early quit day-24hr without smoking during the first month of treatment.RESULTSOnly 35.2% of participants reported a successful early quit day. The likelihood of an early quit day increased significantly (odds ratio [OR] = 1.39, 95% CI = 1.04-1.86, p < .05) with education level and if a quit attempt was made in the past year (OR = 2.27, 95% CI = 1.33-3.87, p < .01). Compared to the placebo arm, those randomized to either nicotine replacement therapy (OR = 3.25, 95% CI = 1.30-8.10, p < .01) or varenicline (OR = 3.16, 95% CI = 1.26-7.92) were significantly more likely to have an early quit day. The likelihood of an early quit day was also positively associated with adherence to the medication protocol (OR = 2.05, 95% CI = 1.52-2.76).CONCLUSIONSDifficulty in achieving an early quit attempt may help explain the very low cessation rates found in studies of MMT smokers. |
| 707 | Ramon, Josep M, Morchon, Sergio, Baena, Antoni, Masuet-Aumatell, Cristina. Combining varenicline and nicotine patches: a randomized controlled trial study in smoking cessation. BMC medicine. 2014;12:172.  **Abstract:** BACKGROUNDSome smokers may benefit from a therapy that combines different nicotine replacement therapies (NRT) or drugs with different mechanisms of action.The aim of this study was to determine the efficacy of the combined therapy of varenicline and nicotine patches versus varenicline monotherapy.METHODSThree hundred forty-one smokers who smoked 20 or more cigarettes per day were recruited from a smoking cessation clinic between February 2012 and June 2013. The participants were randomized to receive a varenicline plus nicotine patch of 21 mg every 24 hours (170) or varenicline plus a placebo patch (171). All of the smokers received a standard 12-week course of varenicline and an 11-week course of either the placebo patch or the active patch after the target quit day. Both groups received behavioral support. The primary outcome was continuous abstinence for weeks 2 through 12 confirmed by exhaled levels of carbon monoxide. Post hoc subgroup analyses were performed to evaluate the treatment effects for a specific endpoint in subgroups of smokers.RESULTSThe combination of the nicotine patch with varenicline was not associated with higher rates of continuous abstinence at 12 weeks (39.1% versus 31.8%; odds ratio (OR) 1.24; 95% confidence interval (CI) 0.8 to 2.6) and 24 weeks (32.8% versus 28.2%; OR 1.17; 95% CI 0.4 to 1.9). When participants were analyzed by subgroups according to cigarette consumption, the abstinence rates among smokers who smoked more than 29 cigarettes per day at 12 weeks (OR 1.39; 95% CI 1.2 to 2.5) and 24 weeks (OR 1.46; 95% CI 1.2 to 2.8) were significantly higher in the combination group. Other post hoc analyses based on level of dependence and previous quit attempts did not show subgroup differences. No differences between the groups for the reported adverse events were observed (χ2 value 0.07; P 0.79).CONCLUSIONSThe combination of varenicline with the nicotine patch does not improve abstinence rates at 12 and 24 weeks compared with varenicline used as monotherapy when all smokers were analyzed as a whole, independent of consumption level.TRIAL REGISTRATIONThis study is registered at clinicaltrial.gov (NCT01538394). |
| 714 | Kawachi, E., Imaizumi, S., Takata, K., Abe, S., Matsuo, Y., Yahiro, E., Uehara, Y., Zhang, B., Miura, S., Saku, K.. Effect of smoking cessation on HDL function. European Heart Journal. 2014;35:902.  **Abstract:** Background: Smoking cessation reduces cardiovascular disease (CVD) and improves health outcomes. Although the oxidative modification of HDL by smoking could reduce the functionality of HDL, the impact of smoking cessation on HDL function has not been studied. Purpose: We studied the effect of smoking cessation on HDL function. Methods: Thirty-two smokers were randomly treated with varenicline or nicotine patch for 24 weeks. The plasma lipid profile, malondialdehyde level, and the cholesterol efflux capacity and anti-oxidative capacity of HDL were measured before and after treatment. Results: The rate of smoking cessation was 78.6%. There was a significant correlation between efflux capacity and body mass index (BMI) (r= -0.44, p=0.01). After the study period, BMI and plasma uric acid were significantly increased (p<0.001, p<0.05, respectively) and plasma malondialdehyde, pulse rate, hemoglobin, systolic blood pressure and the carbon monoxide (CO) level were significantly decreased in the successful smoking cessation group (p<0.05, p<0.05, p<0.001, p<0.05, p<0.001, respectively). The change in the cholesterol efflux capacity was inversely associated with the reduction of CO (r= -0.45, p=0.02). Although there was no difference in HDL subfractions as analyzed by capillary isotachophoresis between the successful and unsuccessful smoking cessation groups, there were significant differences in the anti-oxidative and cholesterol efflux capacity of HDL between the groups. Conclusion: These results indicate that cigarette smoking reduces HDL function and smoking cessation leads to the improvement of HDL functionality, which may be one mechanism by which smoking cessation has beneficial effects against CVD. |
| 715 | Knott, Verner, Impey, Danielle, Philippe, Tristan, Smith, Dylan, Choueiry, Joelle, de la Salle, Sara, Dort, Heather. Modulation of auditory deviance detection by acute nicotine is baseline and deviant dependent in healthy nonsmokers: a mismatch negativity study. Human psychopharmacology. 2014;29:446.  **Abstract:** OBJECTIVECognitive enhancement resulting from nicotinic acetylcholine receptor stimulation may be evidenced by increased efficiency of the auditory-frontal cortex network of auditory discrimination, which is impaired in schizophrenia, a cognitive disorder associated with excessive tobacco use. Investigating automatic (preattentive) detection of acoustic change with the mismatch negativity (MMN) brain event-related potential in response to nicotine in individuals with varying baseline levels of auditory discrimination may provide useful insight into the cholinergic regulation of this neural network and its potential amelioration with novel nicotinic agents.METHODSSixty healthy, non-smoking male volunteers were presented with an 'optimal' multi-feature MMN paradigm in a randomized, placebo controlled double-blind design with 6 mg of nicotine gum.RESULTSParticipants with low, medium, and high baseline amplitudes responded differently to nicotine (vs. placebo), and nicotine response was feature specific. Whereas MMN in individuals with high amplitudes was diminished by nicotine, MMN increased in those with low amplitudes. Nicotine effects were not shown in medium amplitude participants.CONCLUSIONSThese findings provide preliminary support for the role of nicotinic neurotransmission in sensory memory processing of auditory change and suggest that nicotinic receptor modulation can both enhance and diminish change detection, depending on baseline MMN and its eliciting stimulus feature. |
| 720 | Cooper, Sue, Taggar, Jaspal, Lewis, Sarah, Marlow, Neil, Dickinson, Anne, Whitemore, Rachel, Coleman, Tim. Effect of nicotine patches in pregnancy on infant and maternal outcomes at 2 years: follow-up from the randomised, double-blind, placebo-controlled SNAP trial. The Lancet. Respiratory medicine. 2014;2:728.  **Abstract:** BACKGROUNDThe SNAP (Smoking and Nicotine in Pregnancy) trial compared nicotine replacement therapy (NRT) patches with placebo in pregnant smokers; although NRT doubled cessation rates in the first 4 weeks, by delivery no differences in maternal smoking or birth outcomes were noted. As a result, NRT used in standard doses during pregnancy is considered ineffective for smoking cessation. Subsequent effects of NRT on the children of treated mothers are unknown because no trials have investigated the effect of gestational NRT use beyond birth. To assess whether NRT use in pregnancy might cause harm to infants, we aimed to compare effects of NRT and placebo on infant development 2 years after delivery.METHODS1050 pregnant smokers aged 16-45 years, at 12-24 weeks' gestation, and smoking at least five cigarettes per day were recruited from seven hospitals in England between May 1, 2007, and Feb 26, 2010, and followed up until their infants were 2 years old. Participants were randomly assigned (1:1) to receive up to 8-weeks treatment with NRT (15 mg/16 h transdermal patches) or identically packaged and visually matched placebo patches (all patches manufactured by and purchased at market rate from United Pharmaceuticals, Amman, Jordan), issued as two 4-week supplies (521 for NRT group, 529 for placebo group) [Corrected]. Randomisation was stratified by site with participants, health-care professionals, and research staff masked to treatment allocation. The primary results for participants and infants at delivery were published in 2012; we present results from the trial cohort 2 years after birth. After delivery, questionnaires were posted to participants and, if there was no response, to family physicians. The primary outcome at 2 years was infants' survival without developmental impairment (ie, no disability or problems with behaviour or development). Treatment groups were compared on an intention-to-treat basis. The trial is registered with Controlled-Trials.com, number ISRCTN07249128.FINDINGSQuestionnaires were returned at 2 years for 891 (88%) of 1010 live singleton births (445 of (88%) 503 given NRT and 446 (88%) of 507 given placebo). Because of missing data, developmental outcomes, including four infant deaths, were documented for 888 of (88%) 1010 singleton infants; 445 (88%) of 503 infants in NRT group and 443 (87%) of 507 infants in placebo. In the NRT group, 323 (73%) of 445 infants had no impairment compared with 290 (65%) of 443 infants in the placebo group (odds ratio [OR] 1.40, 95% CI 1.05-1.86, p=0.023). At 2 years, 15 (3%) of 521 mothers in the NRT group and nine (2%) of 529 mothers in the placebo groups self-reported prolonged smoking abstinence since a quit date set in pregnancy (OR 1.71, 95% CI 0.74-3.94, p=0.20). Adverse events were not collected after delivery, but previously reported adverse pregnancy and birth outcomes were similar in the two groups.INTERPRETATIONInfants born to women who used NRT for smoking cessation in pregnancy were more likely to have unimpaired development. NRT had no effect on prolonged abstinence from smoking but did cause a temporary doubling of smoking cessation shortly after randomisation during pregnancy, which could explain findings. If findings are confirmed by subsequent research, this has potential implications for the management of smoking in pregnancy.FUNDINGNational Institute for Health Research Health Technology Assessment Programme. |
| 723 | Cooper, Sue, Lewis, Sarah, Thornton, James G, Marlow, Neil, Watts, Kim, Britton, John, Grainge, Matthew J, Taggar, Jaspal, Essex, Holly, Parrott, Steve, Dickinson, Anne, Whitemore, Rachel, Coleman, Tim. The SNAP trial: a randomised placebo-controlled trial of nicotine replacement therapy in pregnancy--clinical effectiveness and safety until 2 years after delivery, with economic evaluation. Health technology assessment (Winchester, England). 2014;18:1.  **Abstract:** BACKGROUNDSmoking during pregnancy causes many adverse pregnancy and birth outcomes. Nicotine replacement therapy (NRT) is effective for cessation outside pregnancy but efficacy and safety in pregnancy are unknown. We hypothesised that NRT would increase smoking cessation in pregnancy without adversely affecting infants.OBJECTIVESTo compare (1) at delivery, the clinical effectiveness and cost-effectiveness for achieving biochemically validated smoking cessation of NRT patches with placebo patches in pregnancy and (2) in infants at 2 years of age, the effects of maternal NRT patch use with placebo patch use in pregnancy on behaviour, development and disability.DESIGNRandomised, placebo-controlled, parallel-group trial and economic evaluation with follow-up at 4 weeks after randomisation, delivery and until infants were 2 years old. Randomisation was stratified by centre and a computer-generated sequence was used to allocate participants using a 1 : 1 ratio. Participants, site pharmacies and all study staff were blind to treatment allocation.SETTINGSeven antenatal hospitals in the Midlands and north-west England.PARTICIPANTSWomen between 12 and 24 weeks' gestation who smoked ≥ 10 cigarettes a day before and ≥ 5 during pregnancy, with an exhaled carbon monoxide (CO) reading of ≥ 8 parts per million (p.p.m.).INTERVENTIONSNRT patches (15 mg per 16 hours) or matched placebo as an 8-week course issued in two equal batches. A second batch was dispensed at 4 weeks to those abstinent from smoking.PARTICIPANTSself-reported, prolonged abstinence from smoking between a quit date and childbirth, validated at delivery by CO measurement and/or salivary cotinine (COT) (primary outcome). Infants, at 2 years: absence of impairment, defined as no disability or problems with behaviour and development. Economic: cost per 'quitter'.RESULTSOne thousand and fifty women enrolled (521 NRT, 529 placebo). There were 1010 live singleton births and 12 participants had live twins, while there were 14 fetal deaths and no birth data for 14 participants. Numbers of adverse pregnancy and birth outcomes were similar in trial groups, except for a greater number of caesarean deliveries in the NRT group. Smoking: all participants were included in the intention-to-treat (ITT) analyses; those lost to follow-up (7% for primary outcome) were assumed to be smoking. At 1 month after randomisation, the validated cessation rate was higher in the NRT group {21.3% vs. 11.7%, odds ratio [OR], [95% confidence interval (CI)] for cessation with NRT, 2.05 [1.46 to 2.88]}. At delivery, there was no difference between groups' smoking cessation rates: 9.4% in the NRT and 7.6% in the placebo group [OR (95% CI), 1.26 (0.82 to 1.96)]. Infants: at 2 years, analyses were based on data from 888 out of 1010 (87.9%) singleton infants (including four postnatal infant deaths) [445/503 (88.5%) NRT, 443/507 (87.4%) placebo] and used multiple imputation. In the NRT group, 72.6% (323/445) had no impairment compared with 65.5% (290/443) in placebo (OR 1.40, 95% CI 1.05 to 1.86). The incremental cost-effectiveness ratio for NRT use was £4156 per quitter (£4926 including twins), but there was substantial uncertainty around these estimates.CONCLUSIONSNicotine replacement therapy patches had no enduring, significant effect on smoking in pregnancy; however, 2-year-olds born to women who used NRT were more likely to have survived without any developmental impairment. Further studies should investigate the clinical effectiveness and safety of higher doses of NRT.TRIAL REGISTRATIONCurrent Controlled Trials ISRCTN07249128.FUNDINGThis project was funded by the NIHR Health Technology Assessment programme and will be published in full in Health Technology Assessment; Vol. 18, No. 54. See the NIHR Journals Library programme website for further project information. |
| 724 | Schlagintweit, Hera E, Good, Kimberley P, Barrett, Sean P. The impact of nicotine lozenges and stimulus expectancies on cigarette craving. Journal of psychopharmacology (Oxford, England). 2014;28:773.  **Abstract:** Reduced craving associated with nicotine replacement therapy use is frequently attributed to the effects of nicotine pharmacology, however non-pharmacological factors may also play a role. This study examined the impact of nicotine pharmacology and non-pharmacological components of an acute nicotine lozenge (4 mg) on cigarette craving, mood and heart rate in 70 daily smokers (36 male). Smoking-related stimuli were used to assess cue-induced craving. Participants were randomly assigned to one of four conditions in a balanced placebo design where half the participants were provided deceptive information regarding the nicotine content of a lozenge. Subjective ratings of craving and mood were collected and heart rate was assessed before and after neutral and smoking cues. Nicotine expectancy reduced withdrawal-related craving (p = 0.006) regardless of actual nicotine administration while combined nicotine expectancy and administration reduced intentions to smoke (p = 0.046) relative to each of the other conditions. Exposure to smoking-related stimuli increased cigarette craving (p ≤ 0.001) and negative affect (p ≤ 0.001) regardless of expectancy or pharmacology. Following the smoking cue, women reported a greater increase in withdrawal-related craving than men (p = 0.027). Findings suggest that both pharmacological and non-pharmacological components of nicotine lozenge administration contribute to its acute effects on craving, yet neither appears effective in preventing craving triggered by exposure to environmental smoking stimuli. |
| 728 | Koegelenberg, Coenraad F N, Noor, Firdows, Bateman, Eric D, van Zyl-Smit, Richard N, Bruning, Axel, O'Brien, John A, Smith, Clifford, Abdool-Gaffar, Mohamed S, Emanuel, Shaunagh, Esterhuizen, Tonya M, Irusen, Elvis M. Efficacy of varenicline combined with nicotine replacement therapy vs varenicline alone for smoking cessation: a randomized clinical trial. JAMA. 2014;312:155.  **Abstract:** IMPORTANCEBehavioral approaches and pharmacotherapy are of proven benefit in assisting smokers to quit, but it is unclear whether combining nicotine replacement therapy (NRT) with varenicline to improve abstinence is effective and safe.OBJECTIVETo evaluate the efficacy and safety of combining varenicline and a nicotine patch vs varenicline alone in smoking cessation.DESIGN, SETTING, AND PARTICIPANTSRandomized, blinded, placebo-controlled clinical trial with a 12-week treatment period and a further 12-week follow-up conducted in 7 centers in South Africa from April 2011 to October 2012. Four hundred forty-six generally healthy smokers were randomized (1:1); 435 were included in the efficacy and safety analyses.INTERVENTIONSNicotine or placebo patch treatment began 2 weeks before a target quit date (TQD) and continued for a further 12 weeks. Varenicline was begun 1 week prior to TQD, continued for a further 12 weeks, and tapered off during week 13.MAIN OUTCOMES AND MEASURESTobacco abstinence was established and confirmed by exhaled carbon monoxide measurements at TQD and at intervals thereafter up to 24 weeks. The primary end point was the 4-week exhaled carbon monoxide-confirmed continuous abstinence rate for weeks 9 through 12 of treatment, ie, the proportion of participants able to maintain complete abstinence from smoking for the last 4 weeks of treatment, as assessed using multiple imputation analysis. Secondary end points included point prevalence abstinence at 6 months, continuous abstinence rate from weeks 9 through 24, and adverse events. Multiple imputation also was used to address loss to follow-up.RESULTSThe combination treatment was associated with a higher continuous abstinence rate at 12 weeks (55.4% vs 40.9%; odds ratio [OR], 1.85; 95% CI, 1.19-2.89; P = .007) and 24 weeks (49.0% vs 32.6%; OR, 1.98; 95% CI, 1.25-3.14; P = .004) and point prevalence abstinence rate at 6 months (65.1% vs 46.7%; OR, 2.13; 95% CI, 1.32-3.43; P = .002). In the combination treatment group, there was a numerically greater incidence of nausea, sleep disturbance, skin reactions, constipation, and depression, with only skin reactions reaching statistical significance (14.4% vs 7.8%; P = .03); the varenicline-alone group experienced more abnormal dreams and headaches.CONCLUSIONS AND RELEVANCEVarenicline in combination with NRT was more effective than varenicline alone at achieving tobacco abstinence at 12 weeks (end of treatment) and at 6 months. Further studies are needed to assess long-term efficacy and safety.TRIAL REGISTRATIONclinicaltrials.gov Identifier: NCT01444131. |
| 733 | Ostroff, Jamie S, Burkhalter, Jack E, Cinciripini, Paul M, Li, Yuelin, Shiyko, Mariya P, Lam, Cho Y, Hay, Jennifer L, Dhingra, Lara K, Lord-Bessen, Jennifer, Holland, Susan M, Manna, Ruth. Randomized trial of a presurgical scheduled reduced smoking intervention for patients newly diagnosed with cancer. Health psychology : official journal of the Division of Health Psychology, American Psychological Association. 2014;33:737.  **Abstract:** OBJECTIVECancer patients who smoke are advised to quit smoking to reduce treatment complications and future cancer risk. This study's main objective was to evaluate the efficacy of a novel, presurgical cessation intervention in newly diagnosed cancer patients scheduled for surgical hospitalization.METHODWe conducted a parallel-arm, randomized controlled trial comparing the efficacy of our hospital-based, tobacco cessation "best practices" treatment model (BP; cessation counseling and nicotine replacement therapy) with BP enhanced by a behavioral tapering regimen (scheduled reduced smoking; BP + SRS) administered by a handheld computer before hospitalization for surgery. Cessation outcomes were short (hospital admission and 3 months) and longer-term (6 months) biochemically verified smoking abstinence. We hypothesized that BP + SRS would be superior to BP alone. One hundred eighty-five smokers were enrolled.RESULTSOverall, 7-day-point prevalence, confirmed abstinence rates at 6 months for BP alone (32%) and BP + SRS (32%) were high; however, no main effect of treatment was observed. Patients who were older and diagnosed with lung cancer were more likely to quit smoking.CONCLUSIONCompared to best practices for treating tobacco dependence, a presurgical, scheduled reduced smoking intervention did not improve abstinence rates among newly diagnosed cancer patients.reserved). |
| 734 | Pilhatsch, Maximilian, Scheuing, Hannah, Kroemer, Nils, Kobiella, Andrea, Bidlingmaier, Martin, Farger, Gisbert, Smolka, Michael N, Zimmermann, Ulrich S. Nicotine administration in healthy non-smokers reduces appetite but does not alter plasma ghrelin. Human psychopharmacology. 2014;29:384.  **Abstract:** OBJECTIVEWe studied whether suppressed secretion of the orexigenic peptide ghrelin might be involved in the anorexigenic effects of nicotine.METHODSFifty healthy non-smokers chewed gums containing 2 mg nicotine, or no nicotine in a double-blind randomised crossover design in two independent studies.RESULTSPlasma nonacylated ghrelin was not significantly affected by nicotine after 30 and 60 min. Increased blood pressure and decreased appetite ratings confirmed a biological nicotine effect.CONCLUSIONSThese results do not support a key role of peripheral ghrelin secretion in weight changes related to smoking or smoking cessation, but do not rule out that central nervous system ghrelin is involved. |
| 735 | Brody, Arthur L, Mukhin, Alexey G, Mamoun, Michael S, Luu, Trinh, Neary, Meaghan, Liang, Lidia, Shieh, Jennifer, Sugar, Catherine A, Rose, Jed E, Mandelkern, Mark A. Brain nicotinic acetylcholine receptor availability and response to smoking cessation treatment: a randomized trial. JAMA psychiatry. 2014;71:797.  **Abstract:** IMPORTANCECigarette smoking leads to upregulation of nicotinic acetylcholine receptors (nAChRs) in the human brain, including the common α4β2* nAChR subtype. While subjective aspects of tobacco dependence have been extensively examined as predictors of quitting smoking with treatment, no studies to our knowledge have yet reported the relationship between the extent of pretreatment upregulation of nAChRs and smoking cessation.OBJECTIVETo determine whether the degree of nAChR upregulation in smokers predicts quitting with a standard course of treatment.DESIGN, SETTING, AND PARTICIPANTSEighty-one tobacco-dependent cigarette smokers (volunteer sample) underwent positron emission tomographic (PET) scanning of the brain with the radiotracer 2-FA followed by 10 weeks of double-blind, placebo-controlled treatment with nicotine patch (random assignment). Pretreatment specific binding volume of distribution (VS/fP) on PET images (a value that is proportional to α4β2* nAChR availability) was determined for 8 brain regions of interest, and participant-reported ratings of nicotine dependence, craving, and self-efficacy were collected. Relationships between these pretreatment measures, treatment type, and outcome were then determined. The study took place at academic PET and clinical research centers.MAIN OUTCOMES AND MEASURESPosttreatment quit status after treatment, defined as a participant report of 7 or more days of continuous abstinence and an exhaled carbon monoxide level of 3 ppm or less.RESULTSSmokers with lower pretreatment VS/fP values (a potential marker of less severe nAChR upregulation) across all brain regions studied were more likely to quit smoking (multivariate analysis of covariance, F8,69 = 4.5; P < .001), regardless of treatment group assignment. Furthermore, pretreatment average VS/fP values provided additional predictive power for likelihood of quitting beyond the self-report measures (stepwise binary logistic regression, likelihood ratio χ21 = 19.8; P < .001).CONCLUSIONS AND RELEVANCESmokers with less upregulation of available α4β2* nAChRs have a greater likelihood of quitting with treatment than smokers with more upregulation. In addition, the biological marker studied here provided additional predictive power beyond subjectively rated measures known to be associated with smoking cessation outcome. While the costly, time-consuming PET procedure used here is not likely to be used clinically, simpler methods for examining α4β2* nAChR upregulation could be tested and applied in the future to help determine which smokers need more intensive and/or lengthier treatment.TRIAL REGISTRATIONclinicaltrials.gov Identifier: NCT01526005. |
| 736 | Xu, Xiaomeng, Clark, Uraina S, David, Sean P, Mulligan, Richard C, Knopik, Valerie S, McGeary, John, MacKillop, James, McCaffery, Jeanne, Niaura, Raymond S, Sweet, Lawrence H. Effects of nicotine deprivation and replacement on BOLD-fMRI response to smoking cues as a function of DRD4 VNTR genotype. Nicotine & tobacco research : official journal of the Society for Research on Nicotine and Tobacco. 2014;16:939.  **Abstract:** INTRODUCTIONReactivity to smoking cues is an important factor in the motivation to smoke and has been associated with the dopamine receptor 4 variable number tandem repeat (DRD4 exon III VNTR) polymorphism. However, little is known about the associated neural mechanisms.METHODSNon-treatment-seeking Caucasian smokers completed overnight abstinence and viewed smoking and neutral cues during 2 separate functional magnetic resonance imaging scans while wearing either a nicotine or placebo patch (order randomized) and were genotyped for the DRD4 VNTR. We conducted mixed-effects repeated-measures analyses of variance (within-subject factor: nicotine or placebo patch; between-subject factor: DRD4 long [L: ≥ 1 copy of ≥ 7 repeats] or short [S: 2 copies ≤ 6 repeats] genotype) of 6 a priori regions of interest.RESULTSRelative to neutral cues, smoking cues elicited greater activity in bilateral ventral striatum and left amygdala during nicotine replacement and deactivation in these regions during nicotine deprivation. A patch × DRD4 interaction was observed in the left amygdala, an area associated with appetitive reinforcement and relapse risk, such that S allele carriers demonstrated greater activation on active patch than on placebo patch.CONCLUSIONSBrain systems associated with reward salience may become primed and overreactive at nicotine replacement doses intended for the first step of smoking cessation and may become inhibited during nicotine withdrawal in DRD4 S but not in DRD4 L carriers. These findings are consistent with the role of these regions in drug reinforcement and suggest a differential influence of nicotine replacement on amygdala activation in the association of incentive salience with smoking stimuli across DRD4 genotypes. |
| 737 | Jardin, Bianca F, Cropsey, Karen L, Wahlquist, Amy E, Gray, Kevin M, Silvestri, Gerard A, Cummings, K Michael, Carpenter, Matthew J. Evaluating the effect of access to free medication to quit smoking: a clinical trial testing the role of motivation. Nicotine & tobacco research : official journal of the Society for Research on Nicotine and Tobacco. 2014;16:992.  **Abstract:** INTRODUCTIONAlthough the majority of smokers are ambivalent about quitting, few treatments specifically target smokers lacking motivation to quit in the near future. Most existing interventions are instead predicated on the belief that active treatments should only be distributed to smokers interested in quitting, a largely untested assumption.METHODSIn the current clinical trial (N = 157), motivated smokers wanting to quit in the next 30 days were given a 2-week nicotine replacement therapy (NRT) sample and a referral to a quitline (Group MNQ), while unmotivated smokers were randomized to receive the same treatment (Group UNQ) or a quitline referral only (Group UQ). Participants were tracked via telephone for 3 months to assess quitting behaviors and smoking reduction.RESULTSGroups significantly differed across all comparisons with regard to incidence of any quit attempt (MNQ: 77%, UNQ: 40%, UQ: 18%, p < .05) and any 24-hr quit attempts (62%, 32%, 16%, p < .05). Clinically meaningful differences emerged in the rates of floating (19%, 17%, 6%) and point prevalence abstinence (17%, 15%, 5%). Compared to participants in Group UQ (11%), a greater proportion of participants in Group MNQ (48%, p = .01) and Group UNQ (31%, p = .01) reduced their daily cigarette consumption by at least half. Proxy measures of cessation readiness (e.g., motivation) favored participants receiving active forms of treatment.CONCLUSIONSProviding NRT samples engaged both motivated and unmotivated smokers into the quitting process and produced positive changes in smoking outcomes. This suggests that motivation should not be considered a necessary precondition to receiving treatment. |
| 738 | Knott, Verner, Choueiry, Joelle, Dort, Heather, Smith, Dylan, Impey, Danielle, de la Salle, Sara, Philippe, Tristan. Baseline-dependent modulating effects of nicotine on voluntary and involuntary attention measured with brain event-related P3 potentials. Pharmacology, biochemistry, and behavior. 2014;122:107.  **Abstract:** Cholinergic stimulation produces cognitive effects that vary across individuals, and stimulus/task conditions. As of yet, the role of individual differences in moderating the effects of the nicotinic acetylcholine receptor agonist nicotine on specific attentional functions and their neural and behavioral correlates is not fully understood. In this randomized, double-blind, placebo-controlled study of 64 healthy non-smokers, we address the contribution of baseline-dependence to inter-individual variability in response to nicotine gum (6 mg) assessed with event-related brain potential (ERP) indices of involuntary (the anteriorly distributed P3a) and voluntary (the posteriorly distributed P3b) attention derived from an active 3-stimulus auditory oddball paradigm involving listening to standard and novel stimuli and detection and response to target stimuli. Nicotine enhanced the amplitude of P3a elicited during the processing of novel stimuli but only in individuals with relatively low baseline P3a amplitudes. Exhibiting an inverted-U nicotine response profile, target P3b and standard N1 amplitudes were increased and decreased in participants with low and high baseline amplitudes, respectively. In all, the findings corroborate the involvement of nicotinic mechanisms in attention, generally acting to increase attentional capacity in relatively low attentional functioning (reduced baseline ERPs) individuals, while having negative or detrimental effects in those with medium/high attentional levels (increased baseline ERPs), and in a manner that is differentially expressed during bottom-up (involuntary) attentional capture and top-down (voluntary) attentional allocation. |
| 739 | Ferguson, Stuart G, Shiffman, Saul. Effect of high-dose nicotine patch on craving and negative affect leading up to lapse episodes. Psychopharmacology. 2014;231:2595.  **Abstract:** RATIONALENicotine patches have been reliably demonstrated to improve smoking cessation outcomes but most users still lapse, and then relapse, during treatment. While patch has been shown to alleviate background cravings, its effects on cue-induced cravings - which have been linked to the occurrence of lapse events - are poorly understood.OBJECTIVESHere we investigate the effect of nicotine patch on the intensity of craving and negative affect experienced during the hours immediately preceding lapse episodes.METHODSParticipants were 185 smokers who had quit in the context of a randomized, double-blind trial of high-dose (35 mg) nicotine patch and who lapsed at least once during the first 5 weeks of treatment. Participants used electronic diaries to monitor their smoking, affect, and craving during their cessation attempt.RESULTS AND CONCLUSIONSThe data suggest that developments on the lapse day - either external events or changes in internal states - caused craving and negative affect to rise, cumulating in the lapse. Nicotine is known to lower background craving and negative affect, but the difference between patch and placebo appeared to dissipate in the hours immediately preceding lapse episodes. Understanding the process by which these symptoms "spike" prior to a lapse - and developing treatments to counter it - are worthy research endeavors. |
| 740 | Hsueh, Kuang-Chieh, Hsueh, Shu-Chun, Chou, Ming-Yueh, Pan, Lee-Fei, Tu, Ming-Shium, McEwen, Andy, West, Robert. Varenicline versus transdermal nicotine patch: a 3-year follow-up in a smoking cessation clinic in Taiwan. Psychopharmacology. 2014;231:2819.  **Abstract:** RATIONALEA network meta-analysis of randomized trials and real-world comparative studies strongly suggest that varenicline is more effective in aiding smoking cessation than single form nicotine replacement therapy (NRT). Modeling the health benefits attributable to this difference relies on extrapolation to lifetime cessation, but to date, follow-up has only extended to 12 months. Longer term follow-up data are helpful in checking these assumptions.OBJECTIVESThis study aimed to compare the sustained abstinence rates of smokers using varenicline versus nicotine patch in their quit attempt up to 36 months.METHODFive hundred eighty-seven smokers were recruited at Kaohsiung Veteran General Hospital between Feb 2006 and Aug 2009. Participants received counseling from a physician and received either varenicline (N=296) or the nicotine patch (N=291) for smoking cessation. Both varenicline and nicotine patch users could receive their medications for a maximum of 8 weeks. Participants were followed up by telephone at 3, 6, 12, and 36 months from the first visit. The primary outcome measure was self-reported sustained abstinence up to 36 months. Measures were also taken of smoking characteristics, cigarette dependence, and sociodemographic characteristics.RESULTSMultiple logistic regression of 36-month sustained abstinence on to medication adjusting for other baseline variables showed a significant advantage for varenicline, OR=7.94 (95 % CI 1.87-33.74).CONCLUSIONAn 8-week course of varenicline appears to yield higher abstinence rate up to 3 years than a similar length course of nicotine transdermal patch in routine clinical practice where behavioral support is available. |
| 755 | Bernstein, Steven L., Rosner, June, O'Malley, Stephanie, D'Onofrio, Gail, Makuch, Robert, Busch, Susan, Toll, Benjamin. Successful tobacco dependence treatment achieved via pharmacotherapy and motivational interviewing in low-income emergency department patients. Academic Emergency Medicine. 2014;21:S59.  **Abstract:** Background: Tobacco use is common in patients who visit EDs, many of whom are low-income and have limited access to care. Prior research studying the efficacy of ED-initiated tobacco dependence treatment has shown mixed results. Objectives: To study the efficacy of an intervention incorporating motivational interviewing, multimodal nicotine replacement, and active quitline referral for adult smokers in an ED. Methods: A two-arm randomized clinical trial of subjects age 18 years or older who smoke and were self-pay or had Medicaid insurance was conducted in a busy urban ED in the northeastern United States. Intervention arm subjects received a motivational interview delivered by a trained research assistant, 6 weeks of nicotine patches and gum, a referral to the state smokers' quitline, a booster call 3 days later, and a brochure. Control arm subjects received only the brochure. Smoking status was assessed by telephone self-report at 1 and 3 months. The primary outcome, at 3 months, included in-person confirmation via exhaled carbon monoxide testing for subjects selfreporting tobacco abstinence. Subjects lost to follow-up were considered to be smoking. Results: From October 2010-December 2012, 778 subjects were enrolled, of whom 774 were alive at 3 months.Demographics included 407 (52.2%) female, median age 41 years, 305 (39.1%) white/non- Hispanic, 308 (39.5%) black/non-Hispanic, 159 (20.2%) Hispanic ethnicity; median daily cigarette use was 11 (IQR 8-20). Treatment arms had comparable baseline characteristics. At 3 months, the prevalence of biochemically confirmed abstinence was 12.2% (47/386) in the intervention arm vs. 4.9% (19/388) in the control arm (P<0.001). The proportions of subjects using quitline services in the intervention and control arms were, respectively, 32.0% (124/386) and 18.7% (73/388) (P<0.0001). Conclusion: An intensive multicomponent intervention was more efficacious than usual care in treating tobacco dependence in adult ED smokers. Intervention arm subjects were more likely to use nicotine replacement products and engage with a quitline. One-year follow-up is ongoing. |
| 756 | Bernstein, Steven L., Zbikowski, Susan, Rosner, June, O'Malley, Stephanie, D'Onofrio, Gail, Makuch, Robert, Busch, Susan, Toll, Benjamin. Association between intensity of quitline services and probability of smoking abstinence. Academic Emergency Medicine. 2014;21:S239.  **Abstract:** Background: Quitlines are effective and cost-effective for smoking cessation. There is a paucity of research regarding the “effective dose” of quitline services needed to induce tobacco abstinence. Objectives: To study the relationship between tobacco abstinence and intensity of use of quitline services among low-income adult smokers enrolled in a clinical trial. Methods: Secondary analysis of a two-arm randomized clinical trial of subjects age 18 years or older affirming every- or some-day smoking visiting a busy urban emergency department in the northeastern US. Inclusion criteria included self-pay or Medicaid insurance. Intervention arm subjects received a motivational interview delivered by a trained research assistant, 6 weeks of nicotine patches and gum, a referral to the state smokers' quitline, a nurse-delivered booster call 3 days later, and a cessation brochure. Control arm subjects received the brochure. Smoking status was assessed by phone at 1 and 3 months, with inperson confirmation via exhaled carbon monoxide testing at 3 months for those reporting tobacco abstinence. At 3 months, smokers were asked about use of quitline services. Quitline data included numbers of phone sessions and call duration. Subjects lost to follow-up were considered to be smoking. Results: Of 770 subjects with data at 3 months, 197 (25.3%) reported any use of quitline services. Subjects were trichotomized: no quitline usage, 1 call only, and >1 call. The biochemically confirmed quit rates at 3 months in these low, medium, and high-use groups were, respectively, 5.7%, 14.1%, and 19.4% (583, 99, and 98 subjects, P<0.0001). Subjects who used the quitline had a median of 28 total minutes of telephone contact. Subjects were also trichotomized by minutes of quitline contact: 0, 1-27, and 28-143 minutes, with respective quit rates of 5.7%, 13.3%, and 20.2% (583, 98, and 99 subjects, P<0.0001). Conclusion: Greater intensity of use of quitline services is associated with higher quit rates. The direction of the association is unclear, however. The quitline appears to have helped subjects quit, although quitline-using subjects may be more motivated to quit. |
| 763 | Winhusen, Theresa M, Brigham, Gregory S, Kropp, Frankie, Lindblad, Robert, Gardin, John G, 2nd, Penn, Pat, Hodgkins, Candace, Kelly, Thomas M, Douaihy, Antoine, McCann, Michael, Love, Lee D, DeGravelles, Eliot, Bachrach, Ken, Sonne, Susan C, Hiott, Bob, Haynes, Louise, Sharma, Gaurav, Lewis, Daniel F, VanVeldhuisen, Paul, Theobald, Jeff, Ghitza, Udi. A randomized trial of concurrent smoking-cessation and substance use disorder treatment in stimulant-dependent smokers. The Journal of clinical psychiatry. 2014;75:336.  **Abstract:** OBJECTIVETo evaluate the impact of concurrent treatments for substance use disorder and nicotine-dependence for stimulant-dependent patients.METHODA randomized, 10-week trial with follow-up at 3 and 6 months after smoking quit date conducted at 12 substance use disorder treatment programs between February 2010 and July 2012. Adults meeting DSM-IV-TR criteria for cocaine and/or methamphetamine dependence and interested in quitting smoking were randomized to treatment as usual (n = 271) or treatment as usual with smoking-cessation treatment (n = 267). All participants received treatment as usual for substance use disorder treatment. Participants assigned to treatment as usual with concurrent smoking-cessation treatment received weekly individual smoking cessation counseling and extended-release bupropion (300 mg/d) during weeks 1-10. During post-quit treatment (weeks 4-10), participants assigned to treatment as usual with smoking-cessation treatment received a nicotine inhaler and contingency management for smoking abstinence. Weekly proportion of stimulant-abstinent participants during the treatment phase, as assessed by urine drug screens and self-report, was the primary outcome. Secondary measures included other substance/nicotine use outcomes and treatment attendance.RESULTSThere were no significant treatment effects on stimulant-use outcomes, as measured by the primary outcome and stimulant-free days, on drug-abstinence, or on attendance. Participants assigned to treatment as usual with smoking-cessation treatment, relative to those assigned to treatment as usual, had significantly better outcomes for drug-free days at 6-month follow-up (χ(2)(1) = 4.09, P <.05), with a decrease in drug-free days from baseline of -1.3% in treatment as usual with smoking-cessation treatment and of -7.6% in treatment as usual. Participants receiving treatment as usual with smoking-cessation treatment, relative to those receiving treatment as usual, had significantly better outcomes on smoking point-prevalence abstinence (25.5% vs 2.2%; χ(2)(1) = 44.69, P < .001; OR =18.2).CONCLUSIONSThese results suggest that providing smoking-cessation treatment to illicit stimulant-dependent patients in outpatient substance use disorder treatment will not worsen, and may enhance, abstinence from nonnicotine substance use.TRIAL REGISTRATIONClinicalTrials.gov identifier: NCT01077024. |
| 769 | Davis, James M, Goldberg, Simon B, Anderson, Maggie C, Manley, Alison R, Smith, Stevens S, Baker, Timothy B. Randomized trial on mindfulness training for smokers targeted to a disadvantaged population. Substance use & misuse. 2014;49:571.  **Abstract:** We report the results of a randomized trial comparing a novel smoking cessation treatment Mindfulness Training for Smokers (MTS) to a usual care therapy (Controls), which included the availability of a tobacco quit line and nicotine patches. Data were collected from 196 low socioeconomic status smokers in 2010-2011 in Madison, Wisconsin. Participants were randomized to either MTS or a telephonic quit line. The primary outcome was 6-month smoking abstinence measured by carbon monoxide breath testing and Time-Line Follow-Back. Among treatment initiators (randomized participants who participated in the intervention), abstinence rates were significantly different between the MTS (38.7%) and control (20.6%, p = .05) groups. Study limitations are also discussed. Results suggest that further study is warranted. |
| 772 | Berlin, Ivan, Grangé, Gilles, Jacob, Nelly, Tanguy, Marie-Laure. Nicotine patches in pregnant smokers: randomised, placebo controlled, multicentre trial of efficacy. BMJ (Clinical research ed.). 2014;348:g1622.  **Abstract:** OBJECTIVETo determine the efficacy of 16 hour nicotine patches among pregnant smokers, with the dose individually adjusted according to saliva cotinine levels (potential range 10-30 mg/day).DESIGNRandomised, double blind, placebo controlled, parallel group, multicentre trial (Study of Nicotine Patch in Pregnancy, SNIPP) between October 2007 and January 2013.SETTING23 maternity wards in France.PARTICIPANTS476 pregnant smokers aged more than 18 years and between 12 and 20 weeks' gestation, who smoked at least five cigarettes a day. After exclusions, 402 women were randomised: 203 to nicotine patches and 199 to placebo patches. Data were available on 192 live births in each group.INTERVENTIONSNicotine and identical placebo patches were administered from quit day up to the time of delivery. Doses were adjusted to saliva cotinine levels when smoking to yield a substitution rate of 100%. Participants were assessed monthly and received behavioural smoking cessation support.MAIN OUTCOME MEASURESThe primary outcomes were complete abstinence (self report confirmed by carbon monoxide level in expired air ≤ 8 ppm) from quit date to delivery, and birth weight. The secondary outcomes were point prevalence of abstinence, time to lapse (a few puffs) or relapse, and delivery and birth characteristics. All data were analysed on an intention to treat basis.RESULTSComplete abstinence was achieved by 5.5% (n=11) of women in the nicotine patch group and 5.1% (n=10) in the placebo patch group (odds ratio 1.08, 95% confidence interval 0.45 to 2.60). The median time to the first cigarette smoked after target quit day was 15 days in both groups (interquartile range 13-18 in the nicotine patch group, 13-20 in the placebo patch group). The point prevalence abstinence ranged from 8% to 12.5% in the nicotine patch group and 8% to 9.5% in the placebo patch group without statistically significant differences. The nicotine substitution rate did not differ from 100%, and the self reported median compliance rate was 85% (interquartile range 56-99%) in the nicotine patch group and 83% (56-95%) in the placebo patch group, assessed at 1016 visits. The mean birth weight was 3065 g (SE 44 g) in the nicotine patch group and 3015 g (SE 44 g) in the placebo patch group (P=0.41). Diastolic blood pressure was significantly higher in the nicotine patch group than in the placebo patch group. The frequency of serious adverse events was similar between the groups, although more non-serious adverse reactions, mainly of skin, occurred in the nicotine patch group.CONCLUSIONThe nicotine patch did not increase either smoking cessation rates or birth weights despite adjustment of nicotine dose to match levels attained when smoking, and higher than usual doses.TRIAL REGISTRATIONClinicalTrials.gov NCT00507975. |
| 773 | Ghanem, Maha. Evaluation of intensive outpatient antismoking counselling program in a low-income country. Chest. 2014;145(S3):617A.  **Abstract:** PURPOSE: A number of non-pharmacological approaches to smoking cessation have been developed with varying degree of success. Intensive anti-smoking counselling remains to be evaluated in countries with limited budget for nicotine replacement therapy; hence this study aimed to define the role of intensive antismoking counselling in taking patients off tobacco in a lowincome country. METHODS: Two hundreds fifty five male smokers, aged 16 50 years were randomly classified into two groups: the control group [Group I, 130 smokers] that was subjected to standard clinical practice and short (1 to 3 minutes) sporadic sessions of unstructured medical antismoking advice, and the intervention group [Group II, 125 smokers] who were subjected to intensive anti-smoking counselling program with three follow up visits for reinforcement of abstinence. Main measurement: Self-reported tobacco abstinence at 6 months. Other measurements: personal details, medical and smoking history; date of first giving up and symptoms of tobacco withdrawal. RESULTS: The rate of continuous abstinence at 6 months was 22.8% for the intervention versus 6.7% for the control group (p<0.000). In subgroup analyses, effectiveness of the intervention did not vary substantially with age, tobacco dependence, number of cigarettes smoked per day, number of years of tobacco consumption, degree of desire to quit smoking, time spent with smokers, subjective health, and presence of tobacco related symptoms. CONCLUSIONS: Intensive out-patient anti-smoking counselling is an effective approach for smoking cessation in low income countries. |
| 775 | Buller, David B, Halperin, Abigail, Severson, Herbert H, Borland, Ron, Slater, Michael D, Bettinghaus, Erwin P, Tinkelman, David, Cutter, Gary R, Woodall, William Gill. Effect of nicotine replacement therapy on quitting by young adults in a trial comparing cessation services. Journal of public health management and practice : JPHMP. 2014;20:E7.  **Abstract:** CONTEXTYoung adult smokers have the highest smoking prevalence among all US age groups but are least likely to use evidence-based cessation counseling or medication to quit.OBJECTIVEUse and effectiveness of nicotine patch were explored in a randomized trial evaluating smoking cessation interventions with this population.PARTICIPANTSSmokers aged 18 to 30 (n = 3094) were recruited through online and off-line methods and from telephone quit lines and analyzed.DESIGNSmokers were enrolled in a pretest-posttest trial, and randomized to 1 of 3 cessation services.SETTINGTrial delivering counseling services by self-help booklet, telephone quit lines, or online expert system in the 48 continental United States.INTERVENTIONSmokers could request a free 2-week course of nicotine replacement therapy (NRT) patches from the project.MAIN OUTCOME MEASUREFollow-up surveys at 12 and 26 weeks assessed smoking abstinence, use of NRT, counseling, and other cessation medications, and smoking-related variables.RESULTSOverall, 69.0% of smokers reported using NRT (M = 3.2 weeks) at 12 weeks and 74.8% (M = 3.3 weeks) at 26 weeks. More smokers who were sent the free nicotine patches (n = 1695; 54.8%) reported using NRT than those who did not receive them (12 weeks: 84.3% vs 41.9%, P < .001; 26 weeks: 87.6% vs 51.1%, P < .001). The use of NRT was associated with greater smoking abstinence at 12 weeks (P < .001) and 26 weeks (P < .05), especially if used for more than 2 weeks (P < .001). Smokers assigned to a self-help booklet or cessation Web site and heavier smokers were most likely to use NRT (P < .05), whereas those reporting marijuana use and binge drinking used NRT less (P < .05).CONCLUSIONSMany young adults were willing to try NRT, and it appeared to help them quit in the context of community-based cessation services. Strategies should be developed to make NRT available to this age group and support them in using it to prevent lifelong smoking. |
| 776 | Oncken, Cheryl, Arias, Albert J, Feinn, Richard, Litt, Mark, Covault, Jonathan, Sofuoglu, Mehmet, Kranzler, Henry R. Topiramate for smoking cessation: a randomized, placebo-controlled pilot study. Nicotine & tobacco research : official journal of the Society for Research on Nicotine and Tobacco. 2014;16:288.  **Abstract:** INTRODUCTIONTopiramate (TOP) blocks glutamate receptors and facilitates GABA (γ-aminobutyric acid) neurotransmission, effects that may facilitate smoking cessation. We compared the effects of behavioral counseling combined with (a) TOP, (b) TOP/nicotine patch (TOP/NIC), or (c) placebo (PLC) for smoking cessation.METHODSWe conducted a 10-week randomized trial in which subjects and research personnel were blinded to TOP versus PLC but not to the TOP/NIC patch condition. In groups receiving TOP, the medication dosage was titrated gradually up to 200 mg/day. The smoking quit date (QD) was scheduled after 2 weeks of medication treatment. NIC (21 mg) was started on the QD in subjects randomized to the TOP/NIC condition. The main outcome measure was the end-of-treatment, 4-week continuous abstinence rate (CAR; biochemically confirmed).RESULTSFifty-seven subjects were randomized to treatment. The 4-week CAR was 1 of 19 (5%) in the PLC group, 5 of 19 (26%) in the TOP group, and 7 of 19 (37%) in the TOP/NIC group (p = .056). Pairwise comparisons showed a difference between TOP/NIC and PLC (p = .042) and a nonsignificant difference between TOP and PLC (p = .18). The PLC group gained 0.37 lb/week, the TOP group lost 0.41 lb/week, and the TOP/NIC group lost 0.07 lb/week (p = .004). Pairwise comparisons showed a difference between TOP and PLC (p < .001) and between TOP/NIC and PLC groups (p = .035). Paresthesia was more frequent in subjects on TOP than PLC (p = .011).CONCLUSIONSTOP, alone or in combination with the NIC, resulted in a numerically higher quit rate than PLC and decreased weight. A larger, PLC-controlled trial is needed to confirm these findings. |
| 778 | Jaehne, A, Unbehaun, T, Feige, B, Herr, S, Appel, A, Riemann, D. The influence of 8 and 16 mg nicotine patches on sleep in healthy non-smokers. Pharmacopsychiatry. 2014;47:73.  **Abstract:** AIM AND METHODSThe purpose of this study was to determine whether sleep changes are a consequence of nicotine presence or withdrawal during the night, we examined 66 healthy non-smokers (33 males, 33 females, age: 20-25 years) after an adaptation night in a sleep laboratory setting. Subjects were randomized to receive placebo or either 8 or 16 mg nicotine patches during the day or during the night in a double blind, parallel group design.RESULTSThe 16 mg nicotine patch applied during the night caused a reduced sleep period time and sleep efficiency as well as an increased wake time. A reduced REM-sleep latency and subjective sleep quality rating were found in subjects receiving nicotine during the night. Arousals, apneas and periodic leg movements were not affected by nicotine.DISCUSSIONThis study documents insomnia-like sleep changes in healthy non-smokers caused by nicotine in a dose-dependent manner. There was no evidence for sleep-related withdrawal symptoms after 13 h of nicotine application. |
| 781 | Barnett, Paul G, Wong, Wynnie, Jeffers, Abra, Munoz, Ricardo, Humfleet, Gary, Hall, Sharon. Cost-effectiveness of extended cessation treatment for older smokers. Addiction (Abingdon, England). 2014;109:314.  **Abstract:** AIMSWe examined the cost-effectiveness of extended smoking cessation treatment in older smokers.DESIGNParticipants who completed a 12-week smoking cessation program were factorial randomized to extended cognitive behavioral treatment and extended nicotine replacement therapy.SETTINGA free-standing smoking cessation clinic.PARTICIPANTSA total of 402 smokers aged 50 years and older were recruited from the community.MEASUREMENTSThe trial measured biochemically verified abstinence from cigarettes after 2 years and the quantity of smoking cessation services utilized. Trial findings were combined with literature on changes in smoking status and the age- and gender-adjusted effect of smoking on health-care cost, mortality and quality of life over the long term in a Markov model of cost-effectiveness over a lifetime horizon.FINDINGSThe addition of extended cognitive behavioral therapy added in smoking cessation services cost [P = 0.012, confidence interval (CI) = -212]. At the end of follow-up, cigarette abstinence rates were 50.0% with extended cognitive behavioral therapy and 37.2% without this therapy (P < 0.05, odds ratio 1.69, CI 1.18-2.54). The model-based incremental cost-effectiveness ratio was 24 per quality-adjusted life year (QALY). Probabilistic sensitivity analysis found that the additional 7 in lifetime cost of the intervention had a 95% confidence interval of -1 to 2081; the 0.15 additional QALYs had a confidence interval of 0.035-0.280, and that the intervention was cost-effective against a  000/QALY acceptance criterion in 99.6% of the replicates. Extended nicotine replacement therapy was not cost-effective.CONCLUSIONSAdding extended cognitive behavior therapy to standard cessation treatment was cost-effective. Further intensification of treatment may be warranted. |
| 782 | Hasan, Faysal M, Zagarins, Sofija E, Pischke, Karen M, Saiyed, Shamila, Bettencourt, Ann Marie, Beal, Laura, Macys, Diane, Aurora, Sanjay, McCleary, Nancy. Hypnotherapy is more effective than nicotine replacement therapy for smoking cessation: results of a randomized controlled trial. Complementary therapies in medicine. 2014;22:1.  **Abstract:** BACKGROUNDThe efficacy of pharmacotherapy for smoking cessation is well documented. However, due to relapse rates and side effects, hypnotherapy is gaining attention as an alternative treatment option. The aim of this one-center randomized study was to compare the efficacy of hypnotherapy alone, as well as hypnotherapy with nicotine replacement therapy (NRT), to conventional NRT in patients hospitalized with a cardiac or pulmonary illness.METHODSWe evaluated self-reported and biochemically verified 7-day prevalence smoking abstinence rates at 12 and 26 weeks post-hospitalization. Patients (n=164) were randomized into one of three counseling-based treatment groups: NRT for 30 days (NRT; n=41), a 90-min hypnotherapy session (H; n=39), and NRT with hypnotherapy (HNRT; n=37). Treatment groups were compared to a "self-quit" group of 35 patients who refused intervention.RESULTSHypnotherapy patients were more likely than NRT patients to be nonsmokers at 12 weeks (43.9% vs. 28.2%; p=0.14) and 26 weeks after hospitalization (36.6% vs. 18.0%; p=0.06). Smoking abstinence rates in the HNRT group were similar to the H group. There was no difference in smoking abstinence rates at 26 weeks between "self quit" and participants in any of the treatment groups. In multivariable regression analysis adjusting for diagnosis and demographic characteristics, H and HNRT were over three times more likely than NRT participants to abstain at 26-weeks post-discharge (RR=3.6; p=0.03 and RR=3.2; p=0.04, respectively).CONCLUSIONHypnotherapy is more effective than NRT in improving smoking abstinence in patients hospitalized for a smoking-related illness, and could be an asset to post-discharge smoking cessation programs. |
| 787 | Heydari, G, Talischi, F, Batmanghelidj, E, Pajooh, M R, Boroomand, A, Zamani, M, Salehi, A, Maddah, S. Dual addictions, parallel treatments: nicotine replacement therapy for patients receiving methadone treatment in the Islamic Republic of Iran. Eastern Mediterranean health journal = La revue de sante de la Mediterranee orientale = al-Majallah al-sihhiyah li-sharq al-mutawassit. 2014;19 Suppl 3:S25.  **Abstract:** This parallel, randomized, open-ended clinical trial tested the impact of nicotine replacement pharmacotherapy during the course of methadone treatment among opiate abusers. A total of 424 men entered the study at 4 drug treatment centres in Tehran, Islamic Republic of Iran. The intervention group received a 6-week regimen of nicotine replacement pharmacotherapy at no charge. After 6 months, 211 persons (99.5%) in the control group continued to smoke and 1 person (0.5%) had quit. In the intervention group, 117 (55.1%) persons smoked, 15 (7.1%) persons had quit and 80 (37.7%) had reduced by more than 50% the number of cigarettes they smoked at the start of the study (P < 0.0001). The findings suggest that the use of nicotine replacement pharmacology in tandem with methadone maintenance treatment can lead to dramatically improved efficacy for treatment of dual addictions. |
| 791 | Chen, Li-Shiun, Bloom, A Joseph, Baker, Timothy B, Smith, Stevens S, Piper, Megan E, Martinez, Maribel, Saccone, Nancy, Hatsukami, Dorothy, Goate, Alison, Bierut, Laura. Pharmacotherapy effects on smoking cessation vary with nicotine metabolism gene (CYP2A6). Addiction (Abingdon, England). 2014;109:128.  **Abstract:** BACKGROUND AND AIMSEvidence suggests that both the nicotinic receptor α5 subunit (CHRNA5) and Cytochrome P450 2A6 (CYP2A6) genotypes influence smoking cessation success and response to pharmacotherapy. We examine the effect of CYP2A6 genotype on smoking cessation success and response to cessation pharmacotherapy, and combine these effects with those of CHRNA5 genotypes.DESIGNPlacebo-controlled randomized smoking cessation trial.SETTINGAmbulatory care facility in Wisconsin, USA.PARTICIPANTSSmokers (n = 709) of European ancestry were randomized to placebo, bupropion, nicotine replacement therapy or combined bupropion and nicotine replacement therapy.MEASUREMENTSSurvival analysis was used to model time to relapse using nicotine metabolism derived from CYP2A6 genotype-based estimates. Slow metabolism is defined as the lowest quartile of estimated metabolic function.FINDINGSCYP2A6-defined nicotine metabolic function moderated the effect of smoking cessation pharmacotherapy on smoking relapse over 90 days [hazard ratio (HR) = 2.81, 95% confidence interval (CI) = 1.32-5.99, P = 0.0075], with pharmacotherapy significantly slowing relapse in fast (HR = 0.39, 95% CI = 0.28-0.55, P = 1.97 × 10(-8)), but not slow metabolizers (HR = 1.09, 95% CI = 0.55-2.17, P = 0.80). Further, only the effect of nicotine replacement, and not bupropion, varies with CYP2A6-defined metabolic function. The effect of nicotine replacement on continuous abstinence is moderated by the combined genetic risks from CYP2A6 and CHRNA5 (Wald = 7.44, d.f. = 1, P = 0.0064).CONCLUSIONSNicotine replacement therapy is effective among individuals with fast, but not slow, CYP2A6-defined nicotine metabolism. The effect of bupropion on relapse likelihood is unlikely to be affected by nicotine metabolism as estimated from CYP2A6 genotype. The variation in treatment responses among smokers with genes may guide future personalized smoking cessation interventions. |
| 792 | Koegelenberg, C.F., Noor, F., Bolliger, C.T., Bateman, E.D., Van Zyl-Smit, R.N., O'Brien, J.A., Bruning, A., Smith, C., Abdool-Gaffar, M.S., Emanuel, S., Irusen, E.M.. A 24-week multicentre, randomised, double-blind study to evaluate the efficacy and safety of varenicline in combination with nicotine replacement therapy vs. varenicline alone for smoking cessation. American Journal of Respiratory and Critical Care Medicine. 2014; 189:A1083.  **Abstract:** Introduction: Tobacco use is an important preventable cause of morbidity and mortality from respiratory, cardiovascular, and metabolic diseases, and cancer. Tobacco dependence is difficult to treat. Together with behavioral support, pharmacotherapy is a proven aid to smoking cessation. There is growing interest in combining nicotine replacement therapy (NRT) and varenicline to improve treatment outcomes, but evidence is limited. The aim of this study was to evaluate the efficacy and safety of combining varenicline and a nicotine patch versus varenicline alone in smoking cessation. Secondary endpoints were sustained abstinence from cigarette smoking and safety of these treatments assessed at 24 weeks. Methods: This multicenter study involved 446 patients seeking help with smoking cessation and was conducted at 7 sites in South Africa [NCT01444131]. Patients were randomized (1:1) to varenicline plus placebo patch or varenicline plus nicotine patch (15 mg for 16 hours). Patches were commenced two weeks prior to and varenicline up-titrated one week prior to a target quit date (TQD), and both were continued for 12 weeks. Smoking status was established (and confirmed by exhaled CO measurements) at the TQD, and at 1, 12 and 24 weeks thereafter. Participants lost to follow-up were analyzed as continuing smokers. Side-effects and withdrawal symptoms were documented. Results: Patents randomized to varenicline plus nicotine patch (n = 222) were well matched with those randomized to varenicline plus placebo (n=224) with regards to age (46.6+/-11.8 vs. 46.4+/-11.9 years, p=0.639), sex (87 vs. 84 males, p=0.791), dependency as measured by the Fagerström Test (4.3+/-1.3 vs. 4.5+/-1.4, p=0.976) and smoking history (21.3+/14.8 vs. 21.4 +/-14.1 pack years, p=0.976). The combination treatment was associated with a significantly superior abstinence rate compared to varenicline plus placebo at 12 weeks post TQD (44.1% vs. 32.6%, p=0.016) and 6 months post TQD (39.2% vs. 24.6%, p=0.001) respectively [table]. The most commonly reported side effects included disturbed sleep and/or abnormal dreams (26.9%), nausea (25.3%), headaches (8.7%) and mild skin reactions (10.7%). Skin reaction was the only adverse event more frequent in the nicotine group (14.0% vs. 7.5%, p = 0.043). There were no reports of suicidal ideation. Conclusion: Varenicline in combination with nicotine replacement was more effective than Varenicline alone at achieving abstinence at 12 weeks and six months. The combination therapy was safe and well tolerated. (Table Presented). |
| 793 | VanNostrand, K., Fisher, K., Khan, B.A.. Smoking cessation after surviving critical illness-is it feasible?-Authors experience with implementation and recruitment. American Journal of Respiratory and Critical Care Medicine. 2014; 189:A4482.  **Abstract:** INTRODUCTION Smoking contributes directly and indirectly to critical illnesses requiring admission to the intensive care unit (ICU). Few studies have investigated smoking cessation programs in critical care survivors. We hypothesize that implementation of a comprehensive, intensive stepwise smoking cessation intervention for critical care survivors would be feasible. METHODS We designed a prospective, randomized smoking cessation study of critical care survivors in the ICU of a county hospital system. Electronic medical records of all ICU admissions were screened at the time of transfer. Eligible subjects were approached by a member of the research team. Consenting subjects were randomly assigned to either the comprehensive, intensive smoking cessation group or usual care. The smoking cessation intervention consisted of an individualized meeting with a trained smoking cessation counselor prior to hospital discharge and scheduled post-discharge telephone calls. Usual care was determined by the primary care team. Both groups completed questionnaires to assess quality of life, depression, anxiety, nicotine addiction and dependence prior to discharge. Nicotine replacement therapy (NRT) was recommended for all subjects with high or severe nicotine dependence. RESULTS A total of 172 subjects were screened from July 15, 2013 through November 1, 2013 and 162 subjects were excluded. Most were excluded because of non-smoking status, active substance abuse and/or psychiatric illness. Ten subjects were eligible and 4 agreed to participate. All consenting subjects were female with a mean age of 61.5 years. Baseline smoking data showed a mean of 48.75 pack-years and 50% were interested in quitting. No subjects reported smoking cessation counseling during hospitalization prior to enrollment. NRT was recommended and prescribed for 3 subjects. Subjects had moderate to severe anxiety scores, mild to moderately-severe depression and moderate to low quality of life scores at baseline. CONCLUSION Our study hopes to address the feasibility of smoking cessation after ICU survival. To date we have found challenges with recruitment. This may be related to an increased number of patients with substance abuse or psychiatric illness. Some eligible subjects declined to participate because of previous failed attempts and not wanting to quit. Occasionally, the time between transfer and discharge is short. Subjects often feel tired and are distracted by discharge planning. Therefore, completion of multiple questionnaires and counseling can be an exhausting process for recovering subjects. Moving forward, we may need to consider enrollment earlier in the hospital course to help with both recruitment and subject with adequate time to participate in this study. |
| 794 | Pei, Hao, Zhang, Ling-Jie, Zeng, Li-Ming, Yu, Feng. Effect of preoperative smoking intervention on postoperative complications of total hip replacement. Chinese Journal of Evidence-Based Medicine. 2014;14:399.  **Abstract:** Objective To investigate the effect of preoperative smoking intervention on the incidence of postoperative complication in patients undergoing total hip replacement. Methods Patients undergoing elective total hip replacement were randomly (block randomization) assigned to either smoking intervention group or control group 4 weeks before scheduled hip replacement. Smoking intervention group received counseling and nicotine replacement therapy and achieved smoking cessation or at least 50% smoking reduction while the control group received standard care with little or no information about the risk of smoking or smoking cessation counselling. Anesthesia was done in accordance with the practice of our hospital with either general anesthesia or combined spinal-epidural anesthesia (CSEA). An assessor who was masked to the intervention recorded the incidence of cardiopulmonary, renal, neurological, or surgical complications and duration of hospital admittance and treatment in intensive care unit (ICU) of two groups of patients. Results A total of 120 patients were finally included (60 cases were in each group, all male, ASA I-III, aged 65-82 years, weighting 42-75 kg). A total of 56 patients in the smoking intervention group and 57 in the control group were analyzed finally. The results showed that, the smoking intervention group was lower than the control group in the incidences of post-operative complications (12/56 vs. 39/57, P=0.000), postoperative wound-related complications (3/56 vs. 21/57, P=0.000), incision infection (1/56 vs. 13/57, P=0.001), and the requirement of secondary surgery (1/56 vs. 9/57, P=0.022) and wound debridement (1/56 vs. 8/57, P=0.040). The median length of stay in the smoking intervention group was significantly less than that of the control group (14 days vs. 26 days, P=0.000). Conclusion An effective smoking intervention 4 weeks before surgery reduces incidence of postoperative complications. © 2014 Editorial Board of Chin J Evid-based Med. |
| 795 | Takata, Kohei, Imaizumi, Satoshi, Kawachi, Emi, Suematsu, Yasunori, Shimizu, Tomohiko, Abe, Satomi, Matsuo, Yoshino, Tsukahara, Hitomi, Noda, Keita, Yahiro, Eiji, Zhang, Bo, Uehara, Yoshinari, Miura, Shin-Ichiro, Saku, Keijiro. Impact of cigarette smoking cessation on high-density lipoprotein functionality: – VN-SEESAW-HDL –. Circulation Journal. 2014;78:2955.  **Abstract:** Background: Smoking cessation reduces the risk of cardiovascular disease (CVD) and improves clinical outcomes in public health. We studied the effect of smoking cessation on high-density lipoprotein (HDL) functionality.Methods and Results: We randomly treated 32 smokers with varenicline or a transdermal nicotine patch as part of a 12-week smoking cessation program (The VN-SEESAW Study). The plasma lipid profiles, plasma and HDL malondialdehyde (MDA) levels, HDL subfractions as analyzed by capillary isotachophoresis, cholesterol efflux capacity, and antiinflammatory activity of HDL were measured before and after the anti-smoking intervention. After smoking cessation, HDL-C, apoA-I levels and HDL subfractions were not significantly different from the respective baseline values. However, cholesterol efflux capacity and the HDL inflammatory index (HII) were significantly improved after smoking cessation. The changes in both parameters (%Δ cholesterol efflux capacity and ΔHII) were also significantly improved in the successful smoking cessation group compared with the unsuccessful group. The changes in cholesterol efflux capacity and HII also correlated with those in end-expiratory CO concentration and MDA in HDL, respectively.Conclusions: Our findings indicate that smoking cessation leads to improved HDL functionality, increased cholesterol efflux capacity and decreased HII, without changing HDL-C or apoA-I levels or HDL subfractions. This may be one of the mechanisms by which smoking cessation improves the risk of CVD. |
| 803 | Kotz, Daniel, Brown, Jamie, West, Robert. Effectiveness of varenicline versus nicotine replacement therapy for smoking cessation with minimal professional support: evidence from an English population study. Psychopharmacology. 2014;231:37.  **Abstract:** RATIONALELimited evidence from randomised controlled trials suggests that varenicline might be more effective than nicotine replacement therapy (NRT) in achieving abstinence from smoking. The comparative effectiveness of varenicline when prescribed under routine circumstances and in the general population has not been tested.OBJECTIVESTo compare the abstinence rates of smokers trying to stop having used varenicline vs. NRT on prescription (Rx) when provided with minimal professional support in the general population while adjusting for key potential confounders.METHODSA large survey of a representative sample of the English population. Participants were 1,579 adults who smoked within the previous 12 months and made at least one quit attempt with varenicline or NRT Rx in their most recent quit attempt. The main outcome measure was self-reported abstinence up to the time of the survey, adjusted for key potential confounders including urges to smoke. A sensitivity analysis was conducted in subsamples in which the quit attempt started up to 6 months or more than 6 months ago.RESULTSThe adjusted odds of abstinence in users of varenicline were 1.76 (95 % CI = 1.22-2.53) times higher compared with users of NRT Rx. However, there was no detectable difference in the subsample of smokers who started their quit attempt more than 6 months ago (adjusted OR = 1.03, 95 %CI = 0.54-1.96).CONCLUSIONSVarenicline use with minimal professional support in the general population of smokers appears more effective than NRT Rx in achieving short-term abstinence. However, this effect may disappear in the long-term. Research is needed to confirm this and establish what may underlie it. |
| 807 | Stapleton, John, West, Robert, Hajek, Peter, Wheeler, Jenny, Vangeli, Eleni, Abdi, Zeinab, O'Gara, Colin, McRobbie, Hayden, Humphrey, Kirsty, Ali, Rachel, Strang, John, Sutherland, Gay. Randomized trial of nicotine replacement therapy (NRT), bupropion and NRT plus bupropion for smoking cessation: effectiveness in clinical practice. Addiction (Abingdon, England). 2013;108:2193.  **Abstract:** BACKGROUND AND AIMSBupropion was introduced for smoking cessation following a pivotal trial showing that it gave improved efficacy over the nicotine patch and also suggesting combination treatment was beneficial. We tested in clinical practice for an effectiveness difference between bupropion and nicotine replacement therapy (NRT), whether the combination improves effectiveness and whether either treatment might be more beneficial for certain subgroups of smokers.DESIGNOpen-label randomized controlled trial with 6-month follow-up.SETTINGFour UK National Health Service (NHS) smoking cessation clinics.PARTICIPANTSSmokers (n = 1071) received seven weekly behavioural support sessions and were randomized to an NRT product of their choice (n = 418), bupropion (n = 409) or NRT plus bupropion (n = 244).MEASURESThe primary outcome was self-reported cessation over 6 months, with biochemical verification at 1 and 6 months. Also measured were baseline demographics, health history, smoking characteristics and unwanted events during treatment.FINDINGSAbstinence rates for bupropion (27.9%) and NRT (24.2%) were not significantly different (odds ratio = 1.21, 95% confidence interval = 0.883-1.67), and the combination rate (24.2%) was similar to that for either treatment alone. There was some evidence that the relative effectiveness of bupropion and NRT differed according to depression (χ(2) = 2.86, P = 0.091), with bupropion appearing more beneficial than NRT in those with a history of depression (29.8 versus 18.5%). Several unwanted symptoms were more common with bupropion.CONCLUSIONThere is no difference in smoking cessation effectiveness among bupropion, nicotine replacement therapy and their combination when used with behavioural support in clinical practice. There is some evidence that bupropion is more beneficial than nicotine replacement therapy for smokers with a history of depression. |
| 808 | McClure, Erin A, Saladin, Michael E, Baker, Nathaniel L, Carpenter, Matthew J, Gray, Kevin M. Smoking topography and abstinence in adult female smokers. Addictive behaviors. 2013;38:2833.  **Abstract:** Preliminary evidence, within both adults and adolescents, suggests that the intensity with which cigarettes are smoked (i.e., smoking topography) is predictive of success during a cessation attempt. These reports have also shown topography to be superior compared to other variables, such as cigarettes per day, in the prediction of abstinence. The possibility that gender may influence this predictive relationship has not been evaluated but may be clinically useful in tailoring gender-specific interventions. Within the context of a clinical trial for smoking cessation among women, adult daily smokers completed a laboratory session that included a 1-hour ad libitum smoking period in which measures of topography were collected (N=135). Participants were then randomized to active medication (nicotine patch vs. varenicline) and abstinence was monitored for 4weeks. Among all smoking topography measures and all abstinence outcomes, a moderate association was found between longer puff duration and greater puff volume and continued smoking during the active 4-week treatment phase, but only within the nicotine patch group. Based on the weak topography-abstinence relationship among female smokers found in the current study, future studies should focus on explicit gender comparisons to examine if these associations are specific to or more robust in male smokers. |
| 809 | Cosci, Fiammetta, Bertoli, Giuly, Abrams, Kenneth. Effects of nicotine withdrawal on panic-like response to breath holding: a placebo-controlled, double-blind, crossover patch study. Depression and anxiety. 2013;30:1217.  **Abstract:** BACKGROUNDCigarette smoking may increase the likelihood of developing panic disorder. Periods of nicotine withdrawal, in particular, may promote panic in individuals high in anxiety sensitivity. We examined the importance of nicotine withdrawal in the occurrence of smoking and panic.METHODSWe utilized a placebo-controlled, double-blind, randomized, crossover design. Fifty smokers underwent a breath-holding (BH) challenge after the transdermal administration of nicotine on one test day and a placebo on another test day. Physiological and psychological variables were assessed at baseline as well as directly before and after the challenges.RESULTSNicotine abstinence induced a decrease in heart rate and systolic blood pressure (BP) before the BH procedure (heart rate: 78.80 ± 11.43 under nicotine, 70.88 ± 10.83 under placebo; systolic BP: 124.90 ± 11.34 under nicotine, 121.18 ± 13.44 under placebo) and shorter BH duration relative to the nicotine patch condition. Nicotine abstinence did not, though, increase fear reactivity to the challenge.CONCLUSIONSThe findings for heart rate and BP are consistent with the stimulant properties of nicotine. The reduced capacity to maintain apnea under placebo might be due to carbon dioxide (CO2 ) hypersensitivity during periods of nicotine abstinence. The negative findings regarding fear reactivity might be due to BH being a relatively weak anxiogen. Future researchers are encouraged to employ CO2 -inhalation procedures to study the relationship between nicotine withdrawal and panic. |
| 810 | Stein, M D, Caviness, C M, Kurth, M E, Audet, D, Olson, J, Anderson, B J. Varenicline for smoking cessation among methadone-maintained smokers: a randomized clinical trial. Drug and alcohol dependence. 2013;133:486.  **Abstract:** BACKGROUNDWith smoking rates far exceeding the general population, methadone-maintained (MMT) opiate-dependent smokers experience high rates of tobacco-related health consequences. Previous treatment studies have used nicotine replacement and produced low quit rates.METHODSWe test, using a three-group randomized design, the efficacy of varenicline versus placebo, in comparison with nicotine replacement therapy (NRT) that combines nicotine patch prescription plus ad libitum nicotine rescue, for smoking cessation. We recruited methadone-maintained smokers from nine treatment centers in southern New England and provided six months of treatment, and a minimal behavioral intervention at baseline (NCI's 5A's). Outcomes included carbon monoxide (CO) confirmed 7-day point smoking cessation prevalence at 6 months and self-reported change in mean cigarettes per day.RESULTSThe 315 participants had a mean age of 40, with 50% male and 79% non-Hispanic White, smoked an average of 19.6 (± 10.4) cigarettes/day, and had a mean daily methadone dose of 109 mg. Intent-to-treat analyses, with missing considered to be smoking, showed the rate of CO-confirmed 7-day abstinence at 6-months was 5.4% overall, with varenicline 3.7% compared to placebo 2.2%, and NRT 8.3% (p>.05). Adherence rates during the 7-days immediately prior to 6-month assessment were 34.2% in varenicline, 34.4% in placebo, and 48.8% in NRT. Between baseline and 6-months there was an overall self-reported mean reduction of 8.3 cigarettes/day.CONCLUSIONVarenicline did not increase quit rates over placebo. Smoking cessation rates in methadone-maintained smokers are low and novel treatment strategies are required. |
| 811 | Liu, Xiaoyu, Li, Runze, Lanza, Stephanie T, Vasilenko, Sara A, Piper, Megan. Understanding the role of cessation fatigue in the smoking cessation process. Drug and alcohol dependence. 2013;133:548.  **Abstract:** BACKGROUNDTo understand the dynamic process of cessation fatigue (i.e., the tiredness of trying to quit smoking) with respect to its average trend, effect on relapse, time-varying relations with craving and negative affect, and differences among genders and treatment groups.METHODRandomized placebo-controlled clinical trial. Participants received either placebo, monotherapy (bupropion SR, nicotine patch, nicotine lozenge), or combined pharmacotherapy (bupropion SR+nicotine lozenge, nicotine patch+nicotine lozenge). Data were collected from 1504 daily smokers who were motivated to quit smoking. The participants completed baseline assessments and ecological momentary assessments for 2 weeks post-quit.RESULTSCessation fatigue reduced the likelihood of 6-month post-quit abstinence (OR=0.97, 95% CI (0.95, 0.99)), and was positively associated with craving and negative affect. After controlling for these two factors, average cessation fatigue increased over time. Compared to men, women experienced greater fatigue (t=-10.69, p<0.0001) and a stronger relation between fatigue and craving (t=-8.80, p<0.0001). The relationship between fatigue and negative affect was significantly stronger in men (t=5.73, p<0.0001). Cessation fatigue was significantly reduced by combined pharmacotherapy (t=-13.4, p<0.0001), as well as monotherapy (t=-6.2, p<0.0001).CONCLUSIONSCessation fatigue was closely related to craving, negative affect, and cessation outcomes. Women reported greater cessation fatigue than men. Current treatments appeared to reduce fatigue and weaken its relations with craving and negative affect. |
| 812 | Allen, Alicia M, Kleppinger, Alison, Lando, Harry, Oncken, Cheryl. Effect of nicotine patch on energy intake and weight gain in postmenopausal women during smoking cessation. Eating behaviors. 2013;14:420.  **Abstract:** INTRODUCTIONPost-cessation weight gain is a commonly cited barrier to smoking cessation. Some evidence suggests that nicotine replacement therapy may limit post-cessation weight gain by reducing energy intake. This project aims to assess differential changes in energy intake and body weight during smoking cessation in a sample of postmenopausal women randomized to receive 21 mg nicotine or placebo patch for 12 weeks.METHODSPostmenopausal women who smoked ≥10 cigarettes/day were enrolled in this double-blind randomized placebo-controlled study. Total energy intake (via four-day food diaries), body mass index (BMI; kg/m(2)), cigarettes/day and smoking status (self-report verified by exhaled carbon monoxide) were assessed at three time points: 2 weeks prior to quit date, 12 weeks after quit date, and 12 months after smoking cessation treatment.RESULTSParticipants (n = 119) were, on average, 55.8 ± 6.7 years old with a baseline BMI of 27.0 ± 5.2 and average cigarette/day was 21.1 ± 8.6. At Week 12, participants randomized to nicotine patch increased their mean caloric intake by 146.4 ± 547.7 kcal/day whereas those on placebo patch decreased their caloric intake by 175.3 ± 463.2 (f-value = 10.1, p-value = 0.002). Despite the differences in caloric intake, body weight remained similar between groups.CONCLUSIONSThe results of this study indicate that nicotine patch may increase energy intake during treatment, and does not prevent post-cessation weight gain in postmenopausal smokers. Additional research is needed to replicate these findings and assess whether different forms of nicotine replacement therapy influence caloric intake and post-cessation weight gain in postmenopausal smokers. |
| 813 | Hawkins, Kirsten A, Cougle, Jesse R. The effects of nicotine on intrusive memories in nonsmokers. Experimental and clinical psychopharmacology. 2013;21:434.  **Abstract:** Correlational research suggests that smoking increases risk of posttraumatic stress disorder (PTSD), though such research by nature cannot rule out third variable explanations for this relationship. The present study used an analogue trauma film design to experimentally test the effects of nicotine on the occurrence of intrusive memories. Fifty-four healthy nonsmokers were randomly assigned to ingest either a nicotine or placebo lozenge before viewing a film depicting motor vehicle accidents. Participants recorded intrusive memories immediately after the film and for a week via diary. Participants in the nicotine condition reported significantly more intrusive memories immediately after watching the film, yet no group differences emerged on intrusions or intrusion-related distress reported during the following week. Among participants low in dispositional rumination, those who had ingested a nicotine lozenge reported more intrusions in the subsequent week than those in the placebo condition. These findings provide novel experimental evidence for the role of nicotine in increasing risk of PTSD and suggest that nicotine may contribute to trauma-related rumination but not heightened reactivity to trauma cues. |
| 814 | Brody, Arthur, Mukhin, Alexey, Mamoun, Michael, Luu, Trinh, Neary, Meaghan, Liang, Lidia, Shieh, Jennifer, Sugar, Catherine A., Rose, Jed, Mandelkern, Mark. Nicotinic acetylcholine receptor density as a predictor of quitting smoking with treatment. Neuropsychopharmacology. 2013;38:S140.  **Abstract:** Background: Up-regulation of nicotinic acetylcholine receptors (nAChRs), including the common α4β2∗ nAChR subtype, is one of the most well-established effects of smoking on the human brain. While subjective aspects of tobacco dependence have been extensively examined as predictors of quitting smoking with treatment, we are not aware of studies determining the relationship between pretreatment up-regulation of nAChRs and smoking cessation with a standard course of treatment. Methods: 81 tobacco-dependent smokers underwent positron emission tomography (PET) scanning with the radiotracer 2-FA (for labeling α4β2∗ nAChRs) followed by double-blind treatment with either active nicotine (n=41) or placebo (n=40) patch for 10 weeks (random assignment). In addition to PET scanning, participants also completed the Fagerstrom Test for Nicotine Dependence, Urge to Smoke (craving) scale, and a self-efficacy rating scale prior to treatment, in order to examine well-known predictors of treatment response. Quit status was defined as a participant report of > 1 week of smoking abstinence and an exhaled carbon monoxide level of o 3 parts per million at the final study treatment visit. For the primary study analysis, an overall multivariate analysis of covariance (MANCOVA) was performed using total binding volume of distribution (VT/fP) values for eight regions of interest (ROIs) as the measures of interest, treatment subgroup (placebo or nicotine patch) and quit status as factors, and age as a nuisance covariate (since prior research indicates that nAChR densities decline with age). Follow-up ANCOVAS were performed for the ROIs separately with the same variables as for the overall MANCOVA. For descriptive purposes, mean α4β2∗ nAChR densities for quitters and non-quitters were compared to available values from non-smoking controls in a previous study, and the percentage up-regulation of α4β2∗ nAChR densities for both groups was calculated. In order to determine which pre-treatment factors best predicted quit status, binary logistic regression was also used, as in prior studies, with quit status as the outcome variable and pre-treatment PET VT/fP data (mean of all ROIs), severity of nicotine dependence (FTND score), subjective craving (mean UTS score on a scale of 0 to 6), and self-efficacy ratings (on a scale of 0 to 100) as the predictor variables. Statistical tests were performed using PASW/SPSS Statistics version 21.0 (SPSS, Inc., Chicago, IL). Results: 20 of the 81 participants met full criteria for having quit smoking at the end of treatment, with a higher percentage of participants treated with active patch having quit smoking than those treated with placebo patch (34.1 versus 15.0%, Chi-Square test, p o 0.05). The overall MANCOVA examining the relationship between pre-treatment α4β2∗ nAChR density and quit status revealed a significant main effect of pre-treatment VT/fP values on quit status (F [8,69]=3.9, P=0.001), resulting from quitters having lower pre-treatment VT/fP values than non-quitters. All ROIs had significant associations with quit status (Fs [1,80]=11.4 to 18.8, Ps=<0.0005 to 0.001), indicating that the relationship between pre-treatment nAChR density and quitting with treatment was not region specific. The interaction between treatment type and quit status was not significant (F [8,69]=0.3, n.s.), indicating that the relationship between pre-treatment nAChR density and quit status was not dependent on treatment type. For the brainstem and prefrontal cortex, quitters had 14 and 21% up-regulation of nAChRs (respectively) compared to previously scanned non-smoking controls, while non-quitters had 42 and 52% up-regulation in these regions. For the logistic regression analysis, the overall test of the ability to use pre-treatment variables to predict quitting was highly significant (Chi-square=30.3, df=4, p o 0.0005). Low mean pre-treatment pre-treatment PET VT/fP values (9.6 versus 14.9; p o 0.0005), low mean craving (UTS) scores (2.2. versus 3.4; p=0.003), and high self-efficacy scores (60 versus 46; p=0.02) were all associated with quitting, while low nicotine dependence (FTND) score had the expected directional relationship, but did not reach statistical significance (4.0 versus 4.6; P=0.25). Conclusions: Cigarette smokers with less severe up-regulation of brain α4β2∗ nAChR density have an improved chance of quitting smoking with treatment than smokers with more severe up-regulation of these receptors. This finding was present in smokers treated with nicotine and placebo patch, and is consistent with our prior preliminary evaluation of smaller groups of smokers treated with cognitive-behavioral therapy, bupropion HCl, or pill placebo. Furthermore, this study supports the use of other measures (severity of nicotine dependence, level of craving, and self-efficacy) in the prediction of treatment response, but the association between the biological marker measured here (up-regulation of α4β2∗ nAChRs) and quit status was numerically more highly significant than the associations between subjectively- rated symptoms and quit status. |
| 816 | Ebbert, Jon O, Croghan, Ivana T, Schroeder, Darrell R, Hurt, Richard D. A randomized phase II clinical trial of high-dose nicotine patch therapy for smokeless tobacco users. Nicotine & tobacco research : official journal of the Society for Research on Nicotine and Tobacco. 2013;15:2037.  **Abstract:** INTRODUCTIONNicotine patch therapy has not been shown to be efficacious for increasing long-term (≥6 months) tobacco abstinence rates among smokeless tobacco (ST) users. Higher doses of nicotine patch therapy may be needed to increase tobacco abstinence rates in this population of tobacco users.METHODSWe randomized ST users who used ≥3 cans/pouches per week to either 8 weeks of high-dose nicotine patch therapy (42mg/day) or matching placebo patch. Subjects were followed for 6 months after randomization.RESULTSFifty-two subjects were randomized. Compared with placebo, high-dose nicotine patch therapy was associated with significantly higher prolonged tobacco abstinence at end-of-treatment (44% vs. 22%, odds ratio [OR] = 2.7, p = .050) and 3 months (40% vs. 19%, OR = 2.9, p = .047). High-dose nicotine patch therapy was associated with significant weight gain attenuation among tobacco abstinence subjects at 3 months (p = .013) and 6 months (p = .018). Compared with placebo, high-dose nicotine patch therapy was associated with nonsignificantly lower nicotine withdrawal scores. Adverse events were not significantly increased with high-dose nicotine patch therapy.CONCLUSIONSHigh-dose nicotine patch therapy is safe and increases short-term tobacco abstinence rates among ST users who use ≥3 cans/pouches per week. High-dose nicotine patch therapy is associated with significant long-term attenuation of weight gain. Future studies to investigate the long-term efficacy of high-dose nicotine patch therapy and the comparative efficacy of this approach compared with standard nicotine patch doses for ST users seems warranted. |
| 819 | Bullen, Christopher, Howe, Colin, Laugesen, Murray, McRobbie, Hayden, Parag, Varsha, Williman, Jonathan, Walker, Natalie. Electronic cigarettes for smoking cessation: a randomised controlled trial. Lancet (London, England). 2013;382:1629.  **Abstract:** BACKGROUNDElectronic cigarettes (e-cigarettes) can deliver nicotine and mitigate tobacco withdrawal and are used by many smokers to assist quit attempts. We investigated whether e-cigarettes are more effective than nicotine patches at helping smokers to quit.METHODSWe did this pragmatic randomised-controlled superiority trial in Auckland, New Zealand, between Sept 6, 2011, and July 5, 2013. Adult (≥18 years) smokers wanting to quit were randomised (with computerised block randomisation, block size nine, stratified by ethnicity [Māori; Pacific; or non-Māori, non-Pacific], sex [men or women], and level of nicotine dependence [>5 or ≤5 Fagerström test for nicotine dependence]) in a 4:4:1 ratio to 16 mg nicotine e-cigarettes, nicotine patches (21 mg patch, one daily), or placebo e-cigarettes (no nicotine), from 1 week before until 12 weeks after quit day, with low intensity behavioural support via voluntary telephone counselling. The primary outcome was biochemically verified continuous abstinence at 6 months (exhaled breath carbon monoxide measurement <10 ppm). Primary analysis was by intention to treat. This trial is registered with the Australian New Zealand Clinical Trials Registry, number ACTRN12610000866000.FINDINGS657 people were randomised (289 to nicotine e-cigarettes, 295 to patches, and 73 to placebo e-cigarettes) and were included in the intention-to-treat analysis. At 6 months, verified abstinence was 7·3% (21 of 289) with nicotine e-cigarettes, 5·8% (17 of 295) with patches, and 4·1% (three of 73) with placebo e-cigarettes (risk difference for nicotine e-cigarette vs patches 1·51 [95% CI -2·49 to 5·51]; for nicotine e-cigarettes vs placebo e-cigarettes 3·16 [95% CI -2·29 to 8·61]). Achievement of abstinence was substantially lower than we anticipated for the power calculation, thus we had insufficient statistical power to conclude superiority of nicotine e-cigarettes to patches or to placebo e-cigarettes. We identified no significant differences in adverse events, with 137 events in the nicotine e-cigarettes group, 119 events in the patches group, and 36 events in the placebo e-cigarettes group. We noted no evidence of an association between adverse events and study product.INTERPRETATIONE-cigarettes, with or without nicotine, were modestly effective at helping smokers to quit, with similar achievement of abstinence as with nicotine patches, and few adverse events. Uncertainty exists about the place of e-cigarettes in tobacco control, and more research is urgently needed to clearly establish their overall benefits and harms at both individual and population levels.FUNDINGHealth Research Council of New Zealand. |
| 825 | Cropsey, Karen L, Hendricks, Peter S, Jardin, Bianca, Clark, C Brendan, Katiyar, Nandan, Willig, James, Mugavero, Michael, Raper, James L, Saag, Michael, Carpenter, Matthew J. A pilot study of screening, brief intervention, and referral for treatment (SBIRT) in non-treatment seeking smokers with HIV. Addictive behaviors. 2013;38:2541.  **Abstract:** INTRODUCTIONPLHIV have higher rates of smoking and lower motivation to quit smoking; thus to impact smoking rates, cessation interventions need to be acceptable to a wider range of PLHIV smokers as well as feasible to implement in a busy clinical setting. The purpose of this study was to evaluate the acceptability, feasibility, and effects of a Screening, Brief Intervention, and Referral for Treatment (SBIRT) model in an HIV/AIDS clinic among a sample of PLHIV.METHODSPLHIV smokers (N=40) were randomized at baseline, irrespective of their self-reported discrete smoking cessation motivation status, to receive either 8-weeks of combination nicotine replacement therapy (NRT) in conjunction with brief counseling (SBIRT framework) (n=23) or usual care (n=17). Smoking outcome measures included cigarettes smoked per day, nicotine dependence, smoking urge, and smoking withdrawal symptoms.RESULTSThe SBIRT intervention appeared to be acceptable and feasible, and produced medium to large reductions in cigarettes smoked per day, physical nicotine dependence, smoking urge, and smoking withdrawal symptoms, even for smokers not ready to quit within 6months.CONCLUSIONSFindings provide preliminary support for the integration of an SBIRT model in an HIV/AIDS clinic setting to screen and provide active treatment to all smokers, regardless of readiness to quit smoking. Given the high prevalence and incredible health burden of continued smoking in this population, identifying brief and effective interventions that are easily translated into clinical practice represents an enormous challenge that if met, will yield significant improvements to overall patient outcomes. |
| 832 | Pathak, Vikas, Rendon, Iliana Samara Hurtado, Lupu, Roxana, Tactuk, Nancy, Olutade, Toyosi, Durham, Carolyn, Stumacher, Richard. Outcome of nicotine replacement therapy in patients admitted to ICU: a randomized controlled double-blind prospective pilot study. Respiratory care. 2013;58:1625.  **Abstract:** BACKGROUNDThe effect of nicotine withdrawal in smokers admitted to the ICU is not well understood, so the role of nicotine replacement therapy (NRT) in those patients is controversial.OBJECTIVETo determine whether NRT in ICU patients affects the need for sedatives/analgesics, ventilator days, and ICU stay.METHODSIn a 20-bed ICU, 40 subjects were randomized to either a 21 mg nicotine patch or a placebo nicotine patch daily until either ICU discharge, transfer to a medical floor, or 10 weeks in the ICU. We collected data on sedatives/analgesics use during ICU stay and use and duration of mechanical ventilation .RESULTSThere were 27 male and 13 female subjects. The mean age was 57.4 y in the intervention group and 52.5 y in the control group. The mean Acute Physiology and Chronic Health Evaluation II score was 14.3 in the intervention group and 13.8 in the control group. The mean ICU stay was 4.5 d in the intervention group and 7 d in the control group. The mean number of days on ventilator was 1.9 in the intervention group and 3.5 in the control group. The number of days on sedation/analgesia was less in the intervention group than in the control group.CONCLUSIONSAlthough ICU stay and ventilator days decreased numerically in this pilot study, statistically there was no beneficial effect from NRT. (International Standard Randomised Controlled Trial Register ISRCTN66928309). |
| 833 | Kochunov, Peter, Du, Xiaoming, Moran, Lauren V, Sampath, Hemalatha, Wijtenburg, S Andrea, Yang, Yihong, Rowland, Laura M, Stein, Elliot A, Hong, L Elliot. Acute nicotine administration effects on fractional anisotropy of cerebral white matter and associated attention performance. Frontiers in pharmacology. 2013;4:117.  **Abstract:** INTRODUCTIONNicotinic acetylcholine receptors are present in the cerebral white matter (WM). We hypothesized that WM response to nicotine can be detected by diffusion tensor imaging (DTI); and that such responses may be associated with nicotine-led cognitive enhancement in sustained attention.METHODSA randomized, nicotine-placebo patch, crossover, double-blind clinical trial in two non-overlapping cohorts of smokers was used to test the hypothesis. The discovery cohort consisted of 39 subjects (N = 20/19 controls/schizophrenic patients, age = 36.8 ± 10.1 years) and the replication cohorts consisted of 38 healthy smokers (31.7 ± 10.5 years). WM integrity was measured by fractional anisotropy (FA) values for the whole brain and nine preselected WM tracts using tract-based-spatial-statistics.RESULTSNicotine significantly enhanced FA values for the genu of corpus callosum compared with placebo (ΔFAgenu) (p = 0.01) in smokers with low recent smoking exposure as measured by low average cotinine level. This finding was replicated in the second cohort (p = 0.02). ΔFAgenu values explained 22% of variance in performance of a sustained attention task during the nicotine session (p = 0.006). However, this effect was limited to schizophrenia patients (r = 0.62 and 0.09; p = 0.003 and 0.7 for patients and controls, respectively).CONCLUSIONAcute pharmacological influence of nicotine patch on WM integrity appeared present, but was dependent on nicotine intake from recent smoking. Change in the WM integrity in the genu of corpus callosum was associated with a significant proportion of variability of nicotine-led changes in sustained attention/working memory of the smokers. Further studies will be necessary to understand biophysical underpinning of the nicotine-related changes in FA. |
| 834 | Lee, Susan M, Landry, Jennifer, Jones, Philip M, Buhrmann, Ozzie, Morley-Forster, Patricia. The effectiveness of a perioperative smoking cessation program: a randomized clinical trial. Anesthesia and analgesia. 2013;117:605.  **Abstract:** BACKGROUNDCigarette smoking by surgical patients is associated with increased complications, particularly perioperative respiratory problems and poor wound healing. In this study, we sought to determine whether a pragmatic perioperative smoking cessation intervention designed for a busy preadmission clinic would be successful in reducing smoking rates and intraoperative and immediate postoperative complications.METHODSThis randomized controlled trial was conducted at a university-affiliated hospital in London, Ontario, Canada. Patients seen in the preadmission clinic at least 3 weeks preoperatively were randomized to either the control group (84 patients) or the intervention group (84 patients). The control group received no specific smoking cessation intervention. The intervention group received (1) brief counseling by the preadmission nurse, (2) brochures on smoking cessation, (3) referral to the Canadian Cancer Society's Smokers' Helpline, and (4) a free 6-week supply of transdermal nicotine replacement therapy. All outcome assessors and caregivers on the operative day were blinded to group assignment. The primary outcome was the rate of smoking cessation as confirmed by exhaled carbon monoxide breath test. Secondary outcomes included perioperative complications and smoking status at 30 days postoperatively.RESULTSBetween October 2010 and April 2012, 168 patients were recruited into the study. Smoking cessation occurred in 12 patients (14.3%) in the intervention group as compared with 3 patients (3.6%) in the control group (relative risk 4.0; 95% confidence interval [CI], 1.2-13.7; P = 0.03). The overall rate of combined intraoperative and immediate postoperative complications was not significantly different between intervention and control groups (13.1% and 16.7%, respectively; relative risk 0.79; 95% CI, 0.38-1.63; P = 0.67). At follow-up 30 days postoperatively, smoking cessation was reported in 22 patients (28.6%) in the intervention group compared with 8 patients (11%) in controls (relative risk 2.6; 95% CI, 1.2-5.5; P = 0.008).CONCLUSIONSOne of the objections to widespread use of smoking cessation interventions in the preadmission clinic is that it is too labor-intensive. The results of this study show that a smoking cessation intervention, designed to minimize additional use of physician or nursing time, results in decreased smoking rates on the day of surgery and promotes abstinence 30 days postoperatively. |
| 835 | Bullen, Christopher, Howe, Colin, Laugesen, Murray, McRobbie, Hayden, Parag, Varsha, Williman, Jonathan, Walker, Natalie. Do electronic cigarettes help smokers quit? Results from a randomized controlled trial. European Respiratory Journal. 2013; 42(S57):1047.  **Abstract:** Introduction. Users of electronic cigarettes ('e-cigarettes') inhale a propylene glycol and/or glycerol mist generated by an electrically-powered vaporizer, usually containing nicotine. Smokers report using them to quit; several withdrawal studies suggest potential as cessation aids but, until now, no adequately-powered randomized controlled cessation trial has been conducted to establish if they assist smokers to quit. Aims and objectives. To investigate if e-cigarettes (16mg nicotine) increase quit rates at 6 months after the quit date, compared to 21mg nicotine patches and to placebo (0mg nicotine) e-cigarettes. Methods. Three-arm randomized controlled trial; 657 smokers aged 18 years and over from Auckland, New Zealand, wanting to quit, were allocated to e-cigarettes with nicotine cartridges or placebo cartridges, or nicotine patches. The primary outcome is continuous abstinence verified by exhaled carbon monoxide. Participants randomized to both e-cigarette arms were blind to nicotine content, instructed to use them for 1 week before and 12 weeks after quit day; those randomized to patches used them daily for an equivalent period. All were offered behavioral support. Results. Of 682 participants registered, 657 were randomized to 16mg nicotine e-cigarette cartridges (n=292), placebo cartridges (n=73) and 21mg patches (n=292). Last participant 6 month follow up will be in July 2013. Quitting results will be presented. Conclusions. This trial will provide robust data on 6-month cessation efficacy of e-cigarettes, with important implications for policies and regulation of these novel devices. |
| 837 | Brown, Jamie, Hajek, Peter, McRobbie, Hayden, Locker, Jo, Gillison, Fiona, McEwen, Andy, Beard, Emma, West, Robert. Cigarette craving and withdrawal symptoms during temporary abstinence and the effect of nicotine gum. Psychopharmacology. 2013;229:209.  **Abstract:** RATIONALEIt is widely believed that nicotine withdrawal symptoms appear within a few hours of stopping smoking, but few data exist documenting their emergence in naturalistic settings. In several countries, nicotine replacement products are licensed for relief of withdrawal symptoms during temporary abstinence, but again, there are no data supporting this from naturalistic settings.OBJECTIVESTo examine the emergence of cigarette craving and withdrawal symptoms during temporary abstinence in a naturalistic setting while using either nicotine or placebo gum.METHODSDouble-blind, randomised, placebo-controlled study in which 132 dependent smokers abstained for 6 h with the assistance of either nicotine (2 mg, n = 42 or 4 mg, n = 24) or placebo (n = 66) gum while travelling on a non-smoking train. Outcome measures were ratings of craving and mood withdrawal symptoms prior to treatment and at regular intervals during abstinence.RESULTSIn a multivariate analysis of all symptoms, there was no interaction between treatment and time [F(21,110) = 1.28, p = 0.20, η²(p)= 0.20] nor an effect of treatment [F(7,124) = 0.45, p = 0.87, η²(p)=  0.03]. There was an effect of time [F(21,110) = 11.59, p < 0.001, η²(p)= 0.69) and univariate analyses revealed that the majority of symptoms increased linearly throughout the period of abstinence with detectable onsets typically between the first 60 and 180 min of abstinence.CONCLUSIONSSmokers who temporarily abstain in naturalistic settings experience craving and withdrawal symptoms that emerge linearly over the first 6 h of abstinence. Changes in craving and several mood withdrawal symptoms can be detected within the first 3 h. Nicotine gum may not have an acute effect on the development of these symptoms. |
| 843 | Rose, Jed E, Behm, Frédérique M. Adapting smoking cessation treatment according to initial response to precessation nicotine patch. The American journal of psychiatry. 2013;170:860.  **Abstract:** OBJECTIVE The authors evaluated an adaptive smoking cessation treatment strategy in which nicotine patch treatment was initiated before a quit date, and then, depending on initial therapeutic response, either the nicotine patch was continued or alternative pharmacotherapies were provided. METHOD The study was a double-blind, parallel-arm adaptive treatment trial. A total of 606 cigarette smokers started open-label nicotine patch treatment 2 weeks before the quit date. Those whose ad lib smoking did not decrease by &gt;50% after 1 week were randomly assigned to one of three double-blind treatments: nicotine patch alone (control condition); "rescue" treatment with bupropion augmentation of the patch; or rescue treatment with varenicline alone. Participants whose precessation smoking decreased &gt;50% but who lapsed after the quit date were also randomly assigned to the two rescue treatments or to nicotine patch alone. Logistic regression analyses compared each rescue treatment against the control condition in terms of abstinence at the end of treatment (weeks 8-11) and at 6 months. RESULTS Smokers who did not respond adequately to precessation nicotine patch benefited from bupropion augmentation; abstinence rates at end of treatment were 16% with nicotine patch alone and 28% with bupropion augmentation (odds ratio=2.04, 95% CI=1.03-4.01). Switching to varenicline produced less robust effects, but point abstinence at 6 months was 6.6% with the patch alone and 16.5% with a switch to varenicline (odds ratio=2.80, 95% CI=1.11-7.06). Postquit adaptive changes in treatment had no significant effects on any abstinence outcome. CONCLUSIONS It is possible to rescue a significant portion of smokers who would have failed to achieve abstinence if left on nicotine patch alone by identifying these smokers before their quit date and implementing adaptive changes in treatment. |
| 846 | Petrovsky, Nadine, Ettinger, Ulrich, Quednow, Boris B, Landsberg, Martin W, Drees, Judith, Lennertz, Leonhard, Frommann, Ingo, Heilmann, Katharina, Sträter, Birgitta, Kessler, Henrik, Dahmen, Norbert, Mössner, Rainald, Maier, Wolfgang, Wagner, Michael. Nicotine enhances antisaccade performance in schizophrenia patients and healthy controls. The international journal of neuropsychopharmacology. 2013;16:1473.  **Abstract:** Nicotine has been proposed to be a cognitive enhancer, particularly in schizophrenia patients. So far, the published studies of nicotine effects on antisaccade performance in schizophrenia patients only tested participants who were deprived smokers. Thus, we aimed to test both smoking and non-smoking patients as well as healthy controls in order to extend previous findings. Moreover, we employed a paradigm using standard and delayed trials. We hypothesized that, if nicotine is a genuine cognitive enhancer, its administration would improve antisaccade performance both in smoking and non-smoking participants. A total of 22 patients with schizophrenia (12 smokers and 10 non-smokers) and 26 controls (14 smokers and 12 non-smokers) completed the study. The effects of a nicotine patch (14 mg for smokers, 7 mg for non-smokers) on antisaccade performance were tested in a randomized, double-blind, placebo-controlled, cross-over trial. Schizophrenia patients made significantly more antisaccade errors than controls (p = 0.03). Both patients and controls made fewer antisaccade errors in the delayed trials than in the standard trials (p < 0.0001). Nicotine significantly reduced antisaccade error rate in the standard trials, but not in the delayed trials (p = 0.02). Smoking status did not influence the nicotine effect on antisaccade error rate (p = 0.10) indicating an equal procognitive effect of nicotine in smokers and non-smokers. Overall the present findings indicate that beneficial effects of nicotine on antisaccade performance are not confined to smoking schizophrenia patients. Instead, the findings likely represent genuine nicotine-induced enhancement of cognitive performance. |
| 849 | El-Mohandes, Ayman A E, Windsor, Richard, Tan, Sylvia, Perry, David C, Gantz, Marie G, Kiely, Michele. A randomized clinical trial of trans-dermal nicotine replacement in pregnant African-American smokers. Maternal and child health journal. 2013;17:897.  **Abstract:** We compared acceptability, adherence and efficacy of trans-dermal nicotine patches and cognitive behavioral therapy (Group 1) to cognitive behavioral therapy alone (Group 2) in minority pregnant smokers. This is a randomized controlled trial. 52 women were recruited during pregnancy with a mean gestational age 18.5 ± 5.0 weeks and followed through delivery. Randomization was by site and initial cotinine levels. Interventionists and interviewers were blinded to group assignment. Two different nicotine replacement therapy dosing regiments were administered according to the baseline salivary cotinine level. A process evaluation model summarized patient adherence. The main outcome measure was self-report of cessation since last visit, confirmed by exhaled carbon monoxide. Analyses of categorical and continuous measures were conducted as well as linear trend tests of salivary cotinine levels. Women lost to follow-up were considered treatment failures. Participants were on average 27.5 ± 5.4 years old, 81 % were single, 69 % unemployed and 96 % were Medicaid eligible. A process evaluation indicated patients in both groups were adherent to scheduled program procedures through Visit 4, but not for Visits 5 and 6. Confirmed quit rates were: at visit 3, 23 (Group 1) and 0 % (Group 2) (p = 0.02); at visits 4 and 5, no difference; at visit 6, 19 (Group 1) and 0 % (Group 2) (p = 0.05). Group 1 delivered infants with a mean gestational age of 39.4 weeks versus 38.4 weeks in Group 2 (p = 0.02). 73 % (52/71) of the eligible smokers agreed to participate and 65 % (17/26) of Group 1 completed the protocol (i.e. attended 6 visits). A comparison of Group 1 and 2 quit rates confirmed a non-significant difference. |
| 850 | Duffy, Sonia A, Fowler, Karen E, Flanagan, Petra S, Ronis, David L, Ewing, Lee A, Waltje, Andrea H. The development of the tobacco tactics website. JMIR research protocols. 2013;2:e22.  **Abstract:** BACKGROUNDWeb-based cessation interventions have been shown to reduce tobacco use, be more efficacious than self-help booklets, be more efficacious if they provide tailored messages, and enhance quit rates in conjunction with nicotine replacement therapy.OBJECTIVEThe objective of this study was to usability test and pilot test the Tobacco Tactics website for veterans.METHODSBoth formative and summative evaluations were used across three small successive studies to develop and test the Tobacco Tactics website for veterans, which was based on a prior face-to-face smoking cessation intervention. Once the website was developed, the research team and Web developers usability tested the website with 5 veteran smokers and former smokers. Feedback from the veterans was collected as they navigated each webpage, then used to revise the website. In pilot study 1, 9 veteran smokers were provided access to the website, and given a baseline and 30-day follow-up survey. In pilot study 2, 18 veteran smokers, who were also motivated to quit smoking, were recruited and randomized to either the Tobacco Tactics website plus nicotine replacement therapy or to the 1-800-QUIT-NOW telephone line.RESULTSAs a result of usability testing, more than 27 modifications were made to improve the website. In pilot study 1, 50% (3/6) veterans who entered the website had cut down on the number of cigarettes and 83% (5/6) found the website enjoyable, easy to read, easy to navigate, and would recommend the website to others. In pilot study 2, which included only smokers motivated to quit and also offered nicotine replacement therapy, seven-day point prevalence abstinence at 30-day follow-up was 40% (4/10) in the intervention group compared to 13% (1/8) in the control group.CONCLUSIONSThese preliminary results are promising and suggest the need for wider-scale testing of the Tobacco Tactics website for veterans. |
| 851 | Rasmussen, Louise, Ladelund, Steen, Brünner, Nils, Jørgensen, Lars N, Nielsen, Hans J, Sørensen, Lars T. Tissue inhibitor of metalloproteinases-1 as a biological marker in colorectal cancer: influence of smoking on plasma levels?. The International journal of biological markers. 2013;28:226.  **Abstract:** BACKGROUNDAt present plasma tissue inhibitor of metalloproteinases-1 (TIMP-1) is undergoing validation as a biological marker in colorectal cancer (CRC). The clinical implementation of plasma TIMP-1 in prognosis, prediction, screening and monitoring CRC requires robust information as to the influence of preanalytical factors, including inter- and intrapersonal biological variations. The aim of the present study was to evaluate the possible effects of smoking on the level of TIMP-1 in plasma from healthy subjects.MATERIALS AND METHODSForty-six never-smokers and 48 daily smokers participated in the 13-week study. Smokers were randomized into 3 groups of 16 subjects each: one group continued to smoke, a second group refrained from smoking and used a transdermal nicotine patch, and a third group refrained from smoking and used placebo patches. Plasma TIMP-1 levels were determined using ELISA.RESULTSNo significant differences in TIMP-1 levels could be shown between the smoking group, the 2 different abstinent smoking groups and the never-smoking group.CONCLUSIONSmoking does not appear to have any influence on TIMP-1 levels in plasma collected from healthy subjects. |
| 857 | Weimer, Katja, Horing, Björn, Stürmer, Julian, Klosterhalfen, Sibylle, Zipfel, Stephan, Enck, Paul. Nicotine stimulus expectancy differentially affects reaction time in healthy nonsmokers and smokers depending on sex: a pilot study. Experimental and clinical psychopharmacology. 2013;21:181.  **Abstract:** Effects of nicotine on neurocognitive performance have been shown but are influenced by nonpharmacological expectancies in smokers, whereas there is little knowledge about expectancy effects in nonsmokers. A half balanced placebo design provides no drug but only placebo and tests the effects of expectations elicited by the information that nicotine was given. Sixty-four healthy participants balanced for smoking status and sex were told that a chewing gum may contain either nicotine or is a placebo in a double-blinded and randomized fashion. One hour later and immediately before neurocognitive function testing (Parametric Go/No-Go task) they were informed--balanced for smoking status and sex--that they belong to the nicotine or to the placebo group. Reaction times of Go responses (RT) and the number of false No-Go responses were analyzed. A significant interaction of all three factors (information, smoking status, sex) was found, indicating that the information to have received nicotine compared with placebo shortened RTs in female smokers but increased it in female nonsmokers, whereas results in men are in part reversed. No effects on No-Go errors were found, and beliefs about nicotine effects had no influence on results. Therefore, the known effects of nicotine on RTs could be influenced by stimulus expectancy not only in smokers but also in nonsmokers. Furthermore, previous results on sex-specific responsiveness to nicotine instructions are supported. |
| 860 | Hajek, Peter, Smith, Katie Myers, Dhanji, Al-Rehan, McRobbie, Hayden. Is a combination of varenicline and nicotine patch more effective in helping smokers quit than varenicline alone? A randomised controlled trial. BMC medicine. 2013;11:140.  **Abstract:** BACKGROUNDNicotine replacement therapy (NRT) and varenicline are both effective in helping smokers quit. There is growing interest in combining the two treatments to improve treatment outcomes, but no experimental data exist on whether this is efficacious. This double-blind randomised controlled trial was designed to evaluate whether adding nicotine patches to varenicline improves withdrawal relief and short-term abstinence rates.METHODS117 participants seeking help to stop smoking were randomly allocated to varenicline plus placebo patch or varenicline plus nicotine patch (15 mg/16 hour). Varenicline use commenced one week prior to the target quit date (TQD), patch use started on the TQD. Ratings of urges to smoke and cigarette withdrawal symptoms were collected weekly over 4 weeks post-TQD. Medication use and smoking status were established at 1, 4 and 12 weeks. Participants lost to follow-up were included as continuing smokers.RESULTS92% of participants used both medications during the first week after the TQD. The combination treatment generated no increase in nausea or other adverse effects. It had no overall effect on urges to smoke or on other withdrawal symptoms. The combination treatment did not improve biochemically validated abstinence rates at 1 week and 4 weeks post-TQD (69% vs 59%, p=0.28 and 60% vs 59%, p=0.91, in the nicotine patch and placebo patch arm, respectively), or self reported abstinence rates at 12 weeks (36% vs. 29%, p=0.39, NS).CONCLUSIONSThe efficacy of varenicline was not enhanced by the addition of nicotine patches, although further trials would be useful to exclude the possibility of type II error.TRIAL REGISTRATIONClinicaltrials.govREGISTRATION NUMBERNCT01184664. |
| 862 | Ebbert, Jon O, Severson, Herbert H, Croghan, Ivana T, Danaher, Brian G, Schroeder, Darrell R. Comparative effectiveness of the nicotine lozenge and tobacco-free snuff for smokeless tobacco reduction. Addictive behaviors. 2013;38:2140.  **Abstract:** Long-term smokeless tobacco (ST) use is associated with cardiovascular disease and cancer, but not all ST users want to quit. Previous studies have evaluated the effectiveness of nicotine lozenges and tobacco-free snuff for reducing ST use among ST users not ready to quit, but no comparative effectiveness trials of these two products have been conducted. We conducted a multicenter, randomized clinical pilot study evaluating the comparative effectiveness of the 4-mg nicotine lozenge and tobacco-free snuff for reducing ST use and increasing tobacco abstinence among ST users with no intention of quitting in the next 30 days. Participants received 8 weeks of treatment and behavioral counseling on tobacco reduction strategies with follow-up to 26 weeks. We randomized 81 participants (40 nicotine lozenges, 41 tobacco-free snuff). No significant differences in reduction were observed between the two groups at weeks 8, 12, and 26. No significant differences were observed between groups in nicotine withdrawal or tobacco craving. However, both groups significantly reduced (p<.001) ST use in cans/week and dips/day from baseline which was sustained through the end-of-study. The observed biochemically-confirmed abstinence rates at week 26 were similar between groups (12% vs. 12%, one-tailed p=.615). The 4-mg nicotine lozenge and the tobacco-free snuff both appear to be effective and comparable for reducing ST use among ST users not ready to quit in the next 30 days. |
| 866 | Perkins, Kenneth A, Karelitz, Joshua L, Jao, Nancy C. Optimal carbon monoxide criteria to confirm 24-hr smoking abstinence. Nicotine & tobacco research : official journal of the Society for Research on Nicotine and Tobacco. 2013;15:978.  **Abstract:** INTRODUCTIONSmoking cessation is typically verified biochemically by expired-air carbon monoxide (CO) levels below 9 ppm (i.e., ≤8 ppm), but this CO criterion may lead many who have smoked within 24hr to be misclassified as abstinent.METHODSAdult dependent smokers (N = 261; 124 men, 137 women) prospectively recorded each cigarette smoked and provided CO on five consecutive days during each of two short-term attempts to quit smoking. Participants were those recruited for crossover tests of the effects of placebo versus medication (nicotine patch or varenicline) on the ability to initiate 24-hr abstinence. All had either a high or low interest in permanently quitting smoking soon (within 3 months) and were randomized to the presence or absence of daily () monetary reinforcement of abstinence.RESULTSTotal accuracy of sensitivity to detect smoking (83%) plus specificity to verify abstinence (87%) was optimal at a CO criterion for abstinence below 5 ppm (≤4 ppm), compared with below 9 ppm (sensitivity of 60%, specificity of 97%). Overall CO detection of sensitivity and specificity was higher in those with high versus low quit interest, but reinforcement of abstinence made no difference.CONCLUSIONSResults indicate a CO criterion half that used in most clinical research may optimally validate 24-hr cessation and reduce misclassification of smokers as "abstinent." |
| 867 | Impey, D, Chique-Alfonzo, M, Shah, D, Fisher, D J, Knott, V J. Effects of nicotine on visuospatial attentional orienting in non-smokers. Pharmacology, biochemistry, and behavior. 2013;106:1.  **Abstract:** Nicotine is a highly addictive substance, suggested to be in part due to its cognitive enhancing effects in the attentional domain. Improvements in stimulus selection with nicotine have been reported but its effects on visual-spatial selective attention are unclear. This study utilized event-related potentials (ERPs) to examine the acute effects of nicotine on selective attention in non-smokers performing a Posner-type visuo-spatial task. The attentional processing of visual-spatial locations is reflected in the P1 ERP component, which represents earlier stages of visual analysis. 24 non-smokers received nicotine gum (6 mg) in a randomized, double-blind, placebo-controlled, repeated measures design. Behavioral performance and ERPs were assessed in response to target locations preceded by valid, invalid and neutral cues. Nicotine did not affect behavioral performance indices. P1 amplitudes were greater in valid and neutral cue trials compared to invalid cue trials and acute nicotine administration (vs. placebo) was found to increase P1 amplitudes in the right hemisphere, particularly in valid cue trials. In addition, in high symptomatic subjects (as indexed by greater increases in heart rate post-administration), nicotine (vs. placebo) produced greater P1 amplitudes in valid cue trials. The study concludes that nicotine enhanced visuospatial selective attention with regards to early visual encoding and analysis. These results demonstrate support in general for the attentional effects of nicotine and nicotinic agonists and they specifically extend these effects to include orienting of visual-spatial attention. |
| 869 | Negoescu, R.M., Bunea, M., Ungurean, C.. Smoking cessation: NRT or educated volition? Best, both of them. European Journal of Preventive Cardiology. 2013;20:S90.  **Abstract:** Background: Some recent studies doubt effectiveness of NRT (nicotine replacement therapy) as compared with more conventional smoking cessation strategies. We investigated whether NRT may contribute to quitting in adults motivated by Quit&Win (Q&W) - a contest challenging smokers to quit during the month of May to qualify for monetary prizes. Methods: Within the CINDI-Pucioasa 2000 prevention project encompassing about 600 people, 50 healthy subjects (ss) aged 39.7+/-11.1 (SD) years, 32 males (M) and 18 females (F), were randomly recruited for the NRT treatment; a part of them were also participating to a Q&W contest. They smoked daily since at least 1 year and 58%, 26% and 16% of them ranked Fagerstrom 7, 8 and 9 grades respectively. They have been given NRT in the chewing gum formulation cf. to the instructions of the producer during the 4 weeks and results were collected using a phone interview or a GP's office visit. The controls were 50 ss (32 M and 18 F) participating to Q&W in Bucharest, aged 32.8+/-11.45 y, smoking 10-19 or over 20 cigarettes/day as per 42% and 54% respectively. They did not use NRT during the contest and were similarly questioned afterwhere. In a further selection we matched 32 ss from every group cf. to criteria: same gender,+/-5 years of age,+/-5 cigarettes smoked daily. Results: Between Pucioasa (NRT) and Bucharest (no NRT) groups of 50 ss there were highly significant differences in quitting: 46.0% vs 30.0%, P=.000075, chi square (15.7) test. Between gender-, age- and tobacco consumption -matched groups of 32 ss there were a significant trend to quit in favour of the NRT group: 53.1% vs 37.5%, P=.069, chi square=3.30. Among nonsuccessful ss, 81.0% of NRT group vs 28.57% of no-NRT 50 group (P=.000057, chi square=16.31) succeeded to diminish smoking with 10 or more cigarettes/day. There were no significant differences as for withdrawal symptoms. In the NRT group 52% did not claim adverse reactions, while 48% signalled mild nausea, palpitations and mastication pain. 60% of quitting people assigned their success 75 to 100% to the NRT treatment. While smoking in both Pucioasa (40%) and Bucharest (59%) was over the national prevalence at that time (circa 30%), the more alert and aggressive ambiance in the latter could make quitting more difficult; on the other hand only a part of Pucioasa ss were supra-motivated by Q&W enrolment. Conclusion: A case-control study conducted on two moderate-size samples of adult smokers, partly or totally pre-motivated by a conventional ceasing campaign, suggests that NRT may have favourably influenced the success rate. |
| 871 | Ghiasi, Faramarz, Farhang, Sara, Farnam, Alireza, Safikhanlou, Salman. The short term effect of nicotine abstinence on visuospatial working memory in smoking patients with schizophrenia. Nordic journal of psychiatry. 2013;67:104.  **Abstract:** BACKGROUNDPatients with schizophrenia suffer from wide range of deficits in neurocognitive functions of the brain, including visuospatial working memory.AIMSThis study aims at evaluating the effect of short-term smoking abstinence on different components of visuospatial working memory in smoker patients with schizophrenia as well as possible reversal effect of a nicotine patch.METHODSIn this trial, 45 male smoker patients with schizophrenia (Razi Hospital, Tabriz, Iran, 2010) were randomly divided into three groups. One group experienced a short time (overnight) smoking abstinence; one group used a single dose of nicotine patch (21 mg) after an overnight smoking abstinence and one group with no intervention or restrain on smoking was considered as control. The function of visuospatial working memory was tested by the brief visuospatial memory test-revised (BVMT-R) at the baseline and after the intervention.RESULTSThe three groups were matched regarding age, educational level and the initial elements of cognitive performance. Between-the-group analysis showed that patients with an overnight smoking abstinence had a significant decrease in percent retained score and an increase in recognition biases compared to patients using nicotine patch and controls. No significant changes were observed in patints using nicotine patch or controls.CONCLUSIONSSmoking abstinence results in visuospatial disabilities in male smoker patients with schizophrenia, including delayed recall and recognition biases. |
| 872 | Weimer, Katja, Horing, Björn, Walentin, Susanne, Winkler, Simone, Klosterhalfen, Sibylle, Zipfel, Stephan, Enck, Paul. Nicotine stimulus expectancy but not nicotine affects cognitive performance in healthy smokers and non-smokers. Psychosomatic Medicine. 2013;75:A.  **Abstract:** Background: Effects of nicotine on cognitive performance have been shown but are influenced by non-pharmacological expectancies in smokers, while there is little knowledge about expectancy effects in non-smokers. Sex differences in nicotine expectancy effects and in placebo effects in general are in part conflicting. Methods: Healthy non-smokers (n=46) and minimally deprived smokers (n=50) (25.2±3.7 years, 49% women) were randomly assigned to 4 groups in a doubleblind balanced placebo cross-over design. They came to the lab twice at least 5 days apart and received a nicotine or placebo patch according to study design: 1) nicotine/placebo, 2) placebo/nicotine, 3) nicotine/nicotine, or 4) placebo/placebo, but expected to receive nicotine and placebo patches in random order. They completed a parametric go/no-go task to assess reaction time (RTT, in ms), sustained attention (% correct trials, PCTT), and response inhibition (% correct inhibition trials, PCIT) in three levels of difficulty. Expectations about nicotine effects were assessed on visual analog scales (VAS; response expectancy) and they were asked which patch they belief to receive (stimulus expectancy). Results: Smokers expected more often to receive nicotine than non-smokers on day1 (65% vs. 50%), and their expectancies about concentration and vigilance were significantly more positive than non-smokers' (p<.05) whereas both groups exhibited low expectations for an effect of nicotine on reaction times. Both nonsmokers and smokers failed to correctly assess the patch they had received on both days. Participants who expected nicotine were faster at all levels on day1 (RTT; p<.05) but not on day2, irrespective of patch, smoking status, or sex, whereas PCTT and PCIT were only randomly affected by these factors. Conclusion: Non-smokers' and non-deprived smokers' reaction times in a go/no-go task are affected by stimulus expectancy only but not by response expectancies or by nicotine itself. Furthermore, there were no sex differences in expectancy or nicotine effects. Results have implications for research on the establishment and maintenance of smoking behavior, and on clinical trials using nicotine for enhancement of cognitive deficits. |
| 875 | Smith, Stevens S, Keller, Paula A, Kobinsky, Kate H, Baker, Timothy B, Fraser, David L, Bush, Terry, Magnusson, Brooke, Zbikowski, Susan M, McAfee, Timothy A, Fiore, Michael C. Enhancing tobacco quitline effectiveness: identifying a superior pharmacotherapy adjuvant. Nicotine & tobacco research : official journal of the Society for Research on Nicotine and Tobacco. 2013;15:718.  **Abstract:** INTRODUCTIONTelephone tobacco quitlines are effective and are widely used, with more than 500,000 U.S. callers in 2010. This study investigated the clinical effectiveness and cost-effectiveness of 3 different quitline enhancements: combination nicotine replacement therapy (NRT), longer duration of NRT, and counseling to increase NRT adherence.METHODSIn this study, 987 quitline callers were randomized to a combination of quitline treatments in a 2 × 2 × 2 factorial design: NRT duration (2 vs. 6 weeks), NRT type (nicotine patch only vs. patch plus nicotine gum), and standard 4-call counseling (SC) versus SC plus medication adherence counseling (MAC). The primary outcome was 7-day point-prevalence abstinence (PPA) at 6 months postquit in intention-to-treat (ITT) analyses.RESULTSCombination NRT for 6 weeks yielded the highest 6-month PPA rate (51.6%) compared with 2 weeks of nicotine patch (38.4%), odds ratios [OR] = 1.71 (95% confidence interval [CI]:1.20-2.45). A similar result was found for 2 weeks of combination NRT (48.2%), OR = 1.49 (95% CI: 1.04-2.14) but not for 6 weeks of nicotine patch alone (46.2%), OR = 1.38 (95% CI: 0.96-1.97). The MAC intervention effect was nonsignificant. Cost analyses showed that the 2-week combination NRT group had the lowest cost per quit (2 vs. 4 for 2-week patch only, 5 for 6-week patch only, and 5 for 6-week combination NRT).CONCLUSIONSCombination NRT for 2 or 6 weeks increased 6-month abstinence rates by 10% and 13%, respectively, over rates produced by 2 weeks of nicotine patch when offered with quitline counseling. A 10% improvement would potentially yield an additional 50,000 quitters annually, assuming 500,000 callers to U.S. quitlines per year. |
| 878 | Koychev, Ivan, McMullen, Katrina, Lees, Jane, Dadhiwala, Rukiya, Grayson, Lois, Perry, Charlotte, Schmechtig, Anne, Walters, James, Craig, Kevin J., Dawson, Gerard R., Dourish, Colin T., Ettinger, Ulrich, Wilkinson, Lawrence, Williams, Steve, Deakin, John Francis William, Barkus, Emma. Differential effect of amisulpride on cognition in schizotypy: Validation of models for the early identification of cognitive enhancing agents. The Lancet. 2013;381:S59.  **Abstract:** Background The cognitive impairment associated with schizophrenia is a major target for drug development but none of the drugs designed to address this problem has shown consistent efficacy. The most common causes for failure in the registration trials could be preempted by testing novel agents in milder forms of the disorder, such as schizotypy and assessing the effect with validated biomarkers. In this study we aimed to test this approach by comparing the effects of risperidone, amisulpride, and nicotine in a double-blind placebo-controlled three-centre study on the cognitive performance in schizotypy. Methods We recruited healthy volunteers who scored high and average on the Schizotypal Personality Questionnaire (122 in each group). 244 participants were randomised to an acute dose of risperidone (n=62, capsule plus placebo patch), amisulpride (n=62, capsule plus placebo patch), nicotine (n=61, placebo capsule plus nicotine patch), or placebo (n=59, placebo capsule plus placebo patch) and proceeded to complete cognitive tests (n-back, verbal fluency, and spatial working memory tasks). Primary outcome measures were percentage correct, errors of commission, and response latencies (n-back), number of correct words and category transitions (verbal fluency), and between-search and within-search errors (spatial working memory tasks). This trial has not been registered. Findings We found evidence for worse performance in the high schizotypy group on the n-back and verbal fluency tasks (p<0.01, η2=0.059 and p<0.01, η2=0.064, respectively). Amisulpride had a differential effect on the two groups in respect to these two tasks (p=0.02 and p=0.04, respectively): it improved performance in the high schizotypy group but impaired the controls. By contrast, risperidone impaired both groups while nicotine had a beneficial effect for the low baseline performers. There was a statistically significant increase in the reaction time latency in the n-back task in the risperidone arm compared with all other arms. Interpretation We attribute the effects of amisulpride to its presynaptic dopamine enhancing action at low doses. The deterioration of performance with risperidone on the other hand was probably due to sedation while nicotine showed signs of general vigilance improvement. Our findings confirm that cognitive abnormalities in schizotypy are replicable in multisite studies and responsive to drug challenges. Importantly, these results underline the importance of dopamine agonism in the pathophysiology of the cognitive deficits in the schizophrenia spectrum disorders. |
| 880 | Ward, Kenneth D, Asfar, Taghrid, Al Ali, Radwan, Rastam, Samer, Weg, Mark W Vander, Eissenberg, Thomas, Maziak, Wasim. Randomized trial of the effectiveness of combined behavioral/pharmacological smoking cessation treatment in Syrian primary care clinics. Addiction (Abingdon, England). 2013;108:394.  **Abstract:** AIMSEffectiveness of nicotine replacement therapy (NRT) for smoking cessation has not been evaluated in low income countries, such as Syria, where it is expensive and not widely available. We evaluated whether nicotine patch boosts smoking cessation rates when used in conjunction with behavioral support in primary care clinics in Aleppo, Syria.DESIGNTwo arm, parallel group, randomized, placebo controlled, double-blinded multi-site trial.SETTINGFour primary care clinics in Aleppo, Syria.PARTICIPANTS Two hundred and sixty-nine adult primary care patients received behavioral cessation counseling from a trained primary care physician and were randomized to receive six weeks of treatment with nicotine versus placebo patch.MEASUREMENTSPrimary end-points were prolonged abstinence (no smoking after a 2-week grace period) at end of treatment, and 6 and 12 months post-quit day, assessed by self-report and exhaled carbon monoxide levels of <10 p.p.m.FINDINGSTreatment adherence was excellent and nicotine patch produced expected reductions in urges to smoke and withdrawal symptoms, but no treatment effect was observed. The proportion of patients in the nicotine and placebo groups with prolonged abstinence was 21.6% and 20.0%, respectively, at end of treatment, 13.4% and 14.1% at 6 months, and 12.7% and 11.9% at 12 months.CONCLUSIONS Nicotine patches may not be effective in helping smokers in low-income countries to stop when given as an adjunct to behavioural support. |
| 881 | Rose, Emma Jane, Ross, Thomas J, Salmeron, Betty Jo, Lee, Mary, Shakleya, Diaa M, Huestis, Marilyn A, Stein, Elliot A. Acute nicotine differentially impacts anticipatory valence- and magnitude-related striatal activity. Biological psychiatry. 2013;73:280.  **Abstract:** BACKGROUNDDopaminergic activity plays a role in mediating the rewarding aspects of abused drugs, including nicotine. Nicotine modulates the reinforcing properties of other motivational stimuli, yet the mechanisms of this interaction are poorly understood. This study aimed to ascertain the impact of nicotine exposure on neuronal activity associated with reinforcing outcomes in dependent smokers.METHODSSmokers (n = 28) and control subjects (n = 28) underwent functional imaging during performance of a monetary incentive delay task. Using a randomized, counterbalanced design, smokers completed scanning after placement of a nicotine or placebo patch; nonsmokers were scanned twice without nicotine manipulation. In regions along dopaminergic pathway trajectories, we considered event-related activity for valence (reward/gain vs. punishment/loss), magnitude (small, medium, large), and outcome (successful vs. unsuccessful).RESULTSBoth nicotine and placebo patch conditions were associated with reduced activity in regions supporting anticipatory valence, including ventral striatum. In contrast, relative to controls, acute nicotine increased activity in dorsal striatum for anticipated magnitude. Across conditions, anticipatory valence-related activity in the striatum was negatively associated with plasma nicotine concentration, whereas the number of cigarettes daily correlated negatively with loss anticipation activity in the medial prefrontal cortex only during abstinence.CONCLUSIONSThese data suggest a partial dissociation in the state- and trait-specific effects of smoking and nicotine exposure on magnitude- and valence-dependent anticipatory activity within discrete reward processing brain regions. Such variability may help explain, in part, nicotine's impact on the reinforcing properties of nondrug stimuli and speak to the continued motivation to smoke and cessation difficulty. |
| 882 | Julian, Mark W, Shao, Guohong, Schlesinger, Larry S, Huang, Qin, Cosmar, David G, Bhatt, Nitin Y, Culver, Daniel A, Baughman, Robert P, Wood, Karen L, Crouser, Elliott D. Nicotine treatment improves Toll-like receptor 2 and Toll-like receptor 9 responsiveness in active pulmonary sarcoidosis. Chest. 2013;143:461.  **Abstract:** BACKGROUNDNew evidence links nicotine to the regulation of T cell-mediated inflammation via a 7 nicotinic cholinergic receptor activation, and chronic nicotine exposure (smoking) reduces the incidence of granulomatous diseases. We sought to determine whether nicotine treatment was well tolerated while effectively normalizing immune responses in patients with active pulmonary sarcoidosis.METHODSConsenting adults with symptomatic sarcoidosis (n 5 13) were randomly assigned to receive 12 weeks of nicotine treatment plus conventional therapy or conventional therapy alone. Obtained blood cells were evaluated for their responsiveness to selected Toll-like receptor (TLR) and nucleotide oligomerization domain-like receptor ligands and T cell surface marker expression before and after nicotine treatment. Asymptomatic patients (n 5 6) and disease-free subjects (n 5 6) served as comparative control subjects. Adverse events were monitored for the duration of the study.RESULTSCompared with the asymptomatic group, symptomatic patients had impaired peripheral responses to TLR2, TLR4, and TLR9 ligands (anergy) and reduced peripheral populations of CD4 1 FoxP3 1 regulatory T cells (Tregs). Nicotine treatment was associated with restoration of TLR2 and TLR9 responsiveness, and expansion of Tregs, including the CD4 1 CD25 2 FoxP3 1 phenotype. There were no serious adverse events or signs of nicotine dependency.CONCLUSIONSNicotine treatment in active pulmonary sarcoidosis was well tolerated and restored peripheral immune responsiveness to TLR2 and TLR9 agonists and expansion of FoxP3 1 Tregs, including a specific “preactivated” (CD25 2 ) phenotype. The immune phenotype of patients with symptomatic sarcoidosis treated with nicotine closely resembled that of asymptomatic patients, supporting the notion that nicotine treatment may be beneficial in this patient population. |
| 886 | Shiffman, Saul, Sembower, Mark A, Rohay, Jeffrey M, Gitchell, Joe G, Garvey, Arthur J. Assigning dose of nicotine gum by time to first cigarette. Nicotine & tobacco research : official journal of the Society for Research on Nicotine and Tobacco. 2013;15:407.  **Abstract:** INTRODUCTIONNicotine gum, a proven effective aid to cessation, comes in two doses: 2-mg and 4-mg. Assignment to the higher dose has traditionally been based on daily cigarette consumption. This paper evaluates efficacy of the gum when the 4-mg dose is assigned based on time to first cigarette (TTFC) being ≤ 30 min.METHODSIn a secondary analysis of a randomized, double-blind, placebo-controlled trial that allocated smokers randomly to placebo, 2-mg, or 4-mg gum (Garvey, A. J., Kinnunen, T., Nordstrom, B. L., Utman, C. H., Doherty, K., Rosner, B., et al. (2000). Effects of nicotine gum dose by level of nicotine dependence. Nicotine & Tobacco Research, 2, 53-63. doi:10.1080/14622200050011303), we evaluated outcomes when 4-mg gum was given to subjects with TTFC ≤ 30 (n = 158, placebo n = 159).RESULTSActive treatment doubled or tripled abstinence rates versus placebo. This also held among smokers with a history of treatment failure. The effect of 4-mg gum was significant among light smokers (<25 CPD) with TTFC ≤ 30; 2-mg gum was not.CONCLUSIONThis analysis suggests that assigning dose of nicotine gum based on TTFC is an effective and appropriate means of dose allocation. |
| 889 | Bergen, Andrew W, Javitz, Harold S, Krasnow, Ruth, Nishita, Denise, Michel, Martha, Conti, David V, Liu, Jinghua, Lee, Won, Edlund, Christopher K, Hall, Sharon, Kwok, Pui-Yan, Benowitz, Neal L, Baker, Timothy B, Tyndale, Rachel F, Lerman, Caryn, Swan, Gary E. Nicotinic acetylcholine receptor variation and response to smoking cessation therapies. Pharmacogenetics and genomics. 2013;23:94.  **Abstract:** OBJECTIVETo evaluate the association of nicotinic acetylcholine receptor (nAChR) single nucleotide polymorphism (SNP) with 7-day point prevalence abstinence (abstinence) in randomized clinical trials of smoking cessation therapies in individuals grouped by pharmacotherapy randomization to inform the development of personalized smoking cessation therapy.MATERIALS AND METHODSWe quantified association of four SNPs at three nAChRs with abstinence in eight randomized clinical trials. Participants were 2633 outpatient treatment-seeking, self-identified European ancestry individuals smoking at least 10 cigarettes/day, recruited through advertisement, prescribed pharmacotherapy, and provided with behavioral therapy. Interventions included nicotine replacement therapy (NRT), bupropion, varenicline, placebo (PLA), or combined NRT and bupropion, and five modes of group and individual behavioral therapy. Outcome measures tested in multivariate logistic regression were end of treatment and 6 month (6MO) abstinence, with demographic, behavioral, and genetic covariates.RESULTS'Risk' alleles previously associated with smoking heaviness were significantly (P<0.05) associated with reduced abstinence in the PLA pharmacotherapy group (PG) at 6MO [for rs588765, odds ratio (95% confidence interval) 0.41 (0.17-0.99)], and at end of treatment and at 6MO [for rs1051730, 0.42 (0.19-0.93) and 0.31 (0.12-0.80)], and with increased abstinence in the NRT PG at 6MO [for rs588765, 2.07 (1.11-3.87) and for rs1051730, 2.54 (1.29-4.99)]. We observed significant heterogeneity in rs1051730 effects (F=2.48, P=0.021) between PGs.CONCLUSIONchr15q25.1 nAChR SNP risk alleles for smoking heaviness significantly increase relapse with PLA treatment and significantly increase abstinence with NRT. These SNP-PG associations require replication in independent samples for validation, and testing in larger sample sizes to evaluate whether similar effects occur in other PGs. |
| 899 | Heydari, G., Talischi, F., Batmanghelidj, E., Pajooh, M.R., Boroomand, A., Zamani, M., Salehi, A., Maddah, S.. Dual addictions, parallel treatments: Nicotine replacement therapy for patients receiving methadone treatment in the Islamic Republic of Iran. Eastern Mediterranean Health Journal. 2013;19:25.  **Abstract:** This parallel, randomized, open-ended clinical trial tested the impact of nicotine replacement pharmacotherapy during the course of methadone treatment among opiate abusers. A total of 424 men entered the study at 4 drug treatment centres in Tehran, Islamic Republic of Iran. The intervention group received a 6-week regimen of nicotine replacement pharmacotherapy at no charge. After 6 months, 211 persons (99.5%) in the control group continued to smoke and 1 person (0.5%) had quit. In the intervention group, 117 (55.1%) persons smoked, 15 (7.1%) persons had quit and 80 (37.7%) had reduced by more than 50% the number of cigarettes they smoked at the start of the study (P < 0.0001). The findings suggest that the use of nicotine replacement pharmacology in tandem with methadone maintenance treatment can lead to dramatically improved efficacy for treatment of dual addictions. |
| 902 | Zincir, Selma Bozkurt, Zincir, Serkan, Kaymak, Esra, Sunbul, Esra Aydin. Comparison of the effectiveness of varenicline, extended-release bupropion and nicotine replacement therapy on the success and the maintenance of a smoking cessation program. Klinik Psikofarmakoloji Bulteni. 2013;23:224.  **Abstract:** Objectives: The most effective approach for individuals who decide to stop tobacco smoking is behavioral training complemented with motivational support and pharmacological therapy. This study aims to compare the effects of varenicline, extended-release bupropion and nicotine replacement therapy (NRT) on the success and the maintenance of a smoking cessation program. Methods: 300 consecutive patients from a smoking cessation clinic, whose nicotine dependence was confirmed, were evaluated in a randomized manner. 251 patients completed the study. In this study, we used the Fagerström nicotine dependence test, a semistructured clinic interview and the Hospital Anxiety and Depression scale (HAD) for clinical follow-up. Carbonmonoxide (CO) levels of the patients were measured with a carboxymeter. Patients had used the determined randomised pharmacological treatment option for 12 weeks. Results: There were no significant differences between the therapy groups according to age, gender and education levels (p > 0.05). Smoking cessation rates were 57.1% in the bupropion group (n= 77), 54.8% in the NRT group (n=73) and 72.3% in the varenicline group (n=101). Fagerstöm scores and the number of cigarettes consumed per day in the NRT group were significantly lower than those of the other therapy groups (p =0.002). There was a significant decrease compared with the initial measurements in the HAD-A 12-week scores in the bupropion and NRT groups (p=0.001 and p=0.003, respectively). Conclusions: According to our findings, 61.8% of all patients participating in this study continued the smoking cessation behavior during the 12 week followup period. It seems that varenicline is better than the other pharmacological therapy modalities for smoking cessation programs. The lower anxiety scores in the bupropion and NRT groups might have increased their success rates. |
| 906 | Fisher, Derek J, Knobelsdorf, Amy, Jaworska, Natalia, Daniels, Richelle, Knott, Verner J. Effects of nicotine on electroencephalographic (EEG) and behavioural measures of visual working memory in non-smokers during a dual-task paradigm. Pharmacology, biochemistry, and behavior. 2013;103:494.  **Abstract:** Research in smokers has shown that nicotine may have the ability to improve certain aspects of cognitive performance, including working memory and attention, processes which implicate frontal and frontal-parietal brain networks. There is limited research on the cognitive effects of nicotine and their associated neural underpinnings in non-smokers. This study examined the effects of acute nicotine on a working memory task alone or combined with a visual detection task (single- and dual-task conditions) using electroencephalographic (EEG) recordings and behavioural performance measures. Twenty non-smokers (13 females; 7 males) received nicotine gum (6 mg) in a double-blind, randomized, placebo-controlled, repeated measures design. Spectral EEG, together with response speed and accuracy measures, were obtained while participants completed a series of N-Back tasks under single- and dual-task conditions. Nicotine failed to exert any significant effects on performance measures, however, EEG changes were observed, primarily in frontal recordings, which varied with memory load, task condition and hemisphere. These findings, discussed in relation to previous studies in smokers, support the notion that nicotine may modulate central executive systems and contribute to smoking behaviour. |
| 909 | Sharifirad, Gholam Reza, Eslami, Ahmad Ali, Charkazi, Abdurrahman, Mostafavi, Firozeh, Shahnazi, Hossein. The effect of individual counseling, line follow-up, and free nicotine replacement therapy on smoking cessation in the samples of Iranian smokers: Examination of transtheoretical model. Journal of research in medical sciences : the official journal of Isfahan University of Medical Sciences. 2012;17:1128.  **Abstract:** BACKGROUNDAccording to transtheoretical model (TTM), Stage matched interventions are more effective in quitting. The objective of current study was to investigate the effect of individual counseling, line follow-up, and free nicotine replacement therapy (NRT) on smoking cessation in smokers who are in preparation stage of smoking.MATERIALS AND METHODSIn a randomized clinical trial design, through sending the short message system, potential participants in preparation stage of smoking were recruited and divided into control (n = 60)and treatment (n = 50) groups. The treatment group received an in-person counseling, line follow-up, and free NRT. TTM variables trend; pros and cons of smoking, behavioral and experiential processes, temptation, were assessed at baseline, 3 and 6 months follow-up along with point prevalence and continuous abstinence.RESULTSContinuous abstinence at 6-month follow-up were 3.3% (n = 2) in control group and 46% (n = 23) in the treatment group (x (2) = 34.041, P < 0.001). Time Χ group analyses indicated that except cons of smoking (P > 0.05), all TTM constructs had significantly changed; temptation (F = 36.864, P < 0.001), pros (F = 12.172, P < 0.001), experiential processes (F = 3.377, P < 0.001), and behavioral processes (F = 11.131, P < 0.001).CONCLUSIONInterventions based on TTM variables increased the quite rate in prepared and motivated people. Our findings suggest that interventions through individual counseling along with free NRT and line follow-up in people who prepare for quitting are beneficial for our country. |
| 910 | Rungruanghiranya, Suthat, Ekpanyaskul, Chatchai, Sakulisariyaporn, Chanin, Watcharanat, Prapada, Akkalakulawas, Kunyanit. Efficacy of fresh lime for smoking cessation. Journal of the Medical Association of Thailand = Chotmaihet thangphaet. 2012;95 Suppl 12:S76.  **Abstract:** OBJECTIVETo determine the efficacy of fresh lime as a smoking cessation aid compared with nicotine gum.MATERIAL AND METHODA randomized, controlled trial was conducted between March 2009 and September 2009. Only regular smokers aged 18 or older who were willing to quit were randomized to receive either fresh lime (n = 47) or nicotine gum (n = 53). Smokers were excluded if they were using other smoking cessation aids, allergic to citrus, or had dental problems. Exhaled carbon monoxide (CO)-confirmed continuous abstinence rate (CAR) during week 9-12 was measured as the primary outcomes. To grade the severity of craving, a 100-mm visual analogue scale (VAS) was used.RESULTSThere was no significant difference in CO-confirmed CAR between the fresh lime group and the nicotine gum group during weeks 9-12 (61.7% vs. 66.0%; p = 0.65), although 7-day point prevalence abstinence at week 4 of the fresh lime users was statistically significant lower than those using nicotine gum (38.3% vs. 58.5%; p = 0.04). Cravings did not differ significantly between the groups, although fresh lime users tend to report more cravings intensity.CONCLUSIONFresh lime can be used effectively as a smoking cessation aid, although not as good as nicotine gum in reducing cravings. |
| 912 | Kochunov, Peter, Stein, Elliot A., Hong, Elliot. Acute nicotine administration improves white matter integrity and associated attention performance. Neuropsychopharmacology. 2012;38:S134.  **Abstract:** Background: Ligand binding studies demonstrate that, in addition to being expressed on synaptic terminals, nicotinic acetylcholine receptors (nAChRs) are expressed in the cerebral white matter (WM). We tested the hypothesis that an acute nicotine pharmacological challenge will alter WM integrity measured, as change in the fractional anisotropy (FA) and that this change is predictive of cognitive effects of nicotine. Methods: A randomized, nicotine patch vs. placebo patch, crossover, double-blind clinical trial. Adult smokers (N=39, 20 controls and 19 schizophrenic patients) were evaluated by diffusion tensor imaging under nicotine vs. placebo patches. Findings of significant nicotine-related FA change were tested in a replication cohort of healthy adult smokers (N=38). Average FA values for the whole brain and nine preselected WM tracts were calculated using tract-based-spatial-statistics. Nicotine-related FA changes were examined in relationship to behavioral performance of a sustained attention/working memory task. Results: Nicotine-placebo difference in FA values for the genu of corpus callosum (DFAgenu) was significantly associated with average cotinine level (R2 change=35%, p=0.001), a measure of nicotine metabolites from primarily recent smoking; a similar finding was observed in the replication cohort (R2 change=13%, p=0.02). Smokers with lower serum cotinine levels were more likely to show a positive DFAgenu change in response to nicotine administration. Further, increased FA in the genu from placebo to nicotine patch explained 22% of variance in performance of a rapid visual sustained attention task during the nicotine session (p=0.006). Conclusions: We observed and replicated an acute pharmacological influence of nicotine on WM that appeared in part contingent upon recent nicotine intake and metabolism from recent smoking. Furthermore, the DFAgenu was significantly and positively correlated with nicotine-related improvement in sustained attention/ working memory. The biophysical underpinning of the nicotine-related changes in FA signal is unknown, but could be due to stimulation of axonal, non-synaptic nAchRs that leads to alterations in local axonal extra cellular environment and improvement in cerebral connectivity and cognitive performance. |
| 914 | Lee, W, Bergen, A W, Swan, G E, Li, D, Liu, J, Thomas, P, Tyndale, R F, Benowitz, N L, Lerman, C, Conti, D V. Gender-stratified gene and gene-treatment interactions in smoking cessation. The pharmacogenomics journal. 2012;12:521.  **Abstract:** We conducted gender-stratified analyses on a systems-based candidate gene study of 53 regions involved in nicotinic response and the brain-reward pathway in two randomized clinical trials of smoking cessation treatments (placebo, bupropion, transdermal and nasal spray nicotine replacement therapy). We adjusted P-values for multiple correlated tests, and used a Bonferroni-corrected α-level of 5 × 10(-4) to determine system-wide significance. Four single-nucleotide polymorphisms (rs12021667, rs12027267, rs6702335, rs12039988; r2 > 0.98) in erythrocyte membrane protein band 4.1 (EPB41) had a significant male-specific marginal association with smoking abstinence (odds ratio (OR) = 0.5; 95% confidence interval (CI): 0.3-0.6) at end of treatment (adjusted P < 6 × 10(-5)). rs806365 in cannabinoid receptor 1 (CNR1) had a significant male-specific gene-treatment interaction at 6-month follow-up (adjusted P = 3.9 × 10(-5)); within males using nasal spray, rs806365 was associated with a decrease in odds of abstinence (OR = 0.04; 95% CI: 0.01-0.2). While the role of CNR1 in substance abuse has been well studied, we report EPB41 for the first time in the nicotine literature. |
| 916 | Moran, Lauren V, Sampath, Hemalatha, Stein, Elliot A, Hong, L Elliot. Insular and anterior cingulate circuits in smokers with schizophrenia. Schizophrenia research. 2012;142:223.  **Abstract:** Schizophrenia (SZ) is associated with high rates of smoking. We previously found that resting state functional connectivity (rsFC) between the dorsal anterior cingulate (dACC) and striatum is independently associated with nicotine addiction and psychiatric illness. Since the insula is implicated in nicotine dependence, we hypothesized that SZ smokers will have greater dysfunction in smoking-related insular and dACC circuits than normal control smokers (NC) independent of smoking severity, consistent with an inherent disease-related weakening of smoking-related circuits. Nicotine challenge was used to demonstrate that decreased rsFC in identified circuits reflects addiction trait and is not affected by pharmacological state. Twenty-four NC smokers and 20 smokers with SZ matched on nicotine addiction severity participated in a resting state fMRI study and were scanned during two separate sessions while receiving a placebo or nicotine patch, in a randomized, cross-over design. Using individualized, anatomically defined anterior and posterior insula and dACC as regions of interest (ROI), whole brain rsFC was performed using each ROI as a seed. Significant negative correlations between smoking severity and rsFC between insula, dACC and striatum were found for both groups. Furthermore, smokers with SZ demonstrated additive reductions in circuit strength between the dACC and insula compared to NC smokers independent of smoking severity. Nicotine challenge did not significantly alter rsFC in insula-dACC-striatal circuits. Reduced rsFC strength between the insula, dACC and striatum is associated with nicotine addiction severity in both non-psychiatrically ill and in SZ smokers. Decreased insula-dACC rsFC may index overlapping circuitry associated with smoking and SZ. |
| 922 | Fenech, Mary A., Reid, Carol. A smoking cessation intervention for people with chronic hepatitis C: A randomised controlled trial. Hepatology. 2012;56:229A.  **Abstract:** Purpose: The purpose of this research was to test the validity of current practice in smoking cessation for the general population i.e., a telephone counselling and nicotine replacement therapy (NRT) intervention and its applicability to people with chronic hepatitis-C. Method: A randomised controlled trial was conducted. Ninety-two smokers with chronic hepatitis-C were randomly assigned into intervention group (telephone counselling from Quitline and NRT) and control group (telephone counselling from Quitline). Consenting adults with Hepatitis-C, who smoke cigarettes and attended the Nurse Practitioner hepatology clinics at the hospital were recruited. Data collection tools included a socio-demographics questionnaire and Fagerstrom Test for Nicotine Dependence. All statistical data were analysed using SPSS. Nominal data was expressed as frequencies and percentages. Fisher's exact test and logistic regression were used to compare differences between groups. Ordinal and numeric data was described by means and standard deviations. ANOVA and t-tests were used to show relationships between 2 or more groups. Results: A telephone counselling and NRT intervention resulted in a reduction in the number of cigarettes smoked per day after 6 weeks. No difference was found between the groups at 6 weeks; the control group showed an average reduction of 5.3 (CI:1.6, 9.0) cigarettes per day and the intervention group a reduction of 6 (CI:2.6, 9.5) (t= -0.3, p=0.751) cigarettes per day. Over the 12 week period the intervention group showed a sustained reduction of smoking with participants reporting they smoked 7.7 (CI: 3.6, 11.7) cigarettes less per day. At 12 weeks it was shown that the telephone counselling group maintained an average of 1.3 (CI:-2.6, 5.2) cigarette reduction from baseline. This result was significantly lower than the intervention group who maintained a 7.7 (CI: 3.6, 11.7) reduction of cigarettes per day (t=-2.3, p=0.026). Conclusion: The results showed that a telephone counselling and nicotine replacement therapy intervention from the nurse practitioner, hepatology significantly reduced smoking in patients with chronic hepatitis-C compared to those who received a telephone counselling intervention only. It was also shown that those who received the counselling and NRT intervention had a sustained reduction over the 12 weeks. These results further informed a nurse practitioner model of care for approaches to smoking cessation for this vulnerable and marginalised patient group. |
| 927 | Knott, Verner, Shah, Dhrasti, Millar, Anne, McIntosh, Judy, Fisher, Derek, Blais, Crystal, Ilivitsky, Vadim. Nicotine, Auditory Sensory Memory, and sustained Attention in a Human Ketamine Model of Schizophrenia: Moderating Influence of a Hallucinatory Trait. Frontiers in pharmacology. 2012;3:172.  **Abstract:** BACKGROUNDThe procognitive actions of the nicotinic acetylcholine receptor (nAChR) agonist nicotine are believed, in part, to motivate the excessive cigarette smoking in schizophrenia, a disorder associated with deficits in multiple cognitive domains, including low-level auditory sensory processes and higher-order attention-dependent operations.OBJECTIVESAs N-methyl-d-aspartate receptor (NMDAR) hypofunction has been shown to contribute to these cognitive impairments, the primary aims of this healthy volunteer study were to: (a) to shed light on the separate and interactive roles of nAChR and NMDAR systems in the modulation of auditory sensory memory (and sustained attention), as indexed by the auditory event-related brain potential - mismatch negativity (MMN), and (b) to examine how these effects are moderated by a predisposition to auditory hallucinations/delusions (HD).METHODSIn a randomized, double-blind, placebo-controlled design involving a low intravenous dose of ketamine (0.04 mg/kg) and a 4 mg dose of nicotine gum, MMN, and performance on a rapid visual information processing (RVIP) task of sustained attention were examined in 24 healthy controls psychometrically stratified as being lower (L-HD, n = 12) or higher (H-HD) for HD propensity.RESULTSKetamine significantly slowed MMN, and reduced MMN in H-HD, with amplitude attenuation being blocked by the co-administration of nicotine. Nicotine significantly enhanced response speed [reaction time (RT)] and accuracy (increased % hits and d' and reduced false alarms) on the RVIP, with improved performance accuracy being prevented when nicotine was administered with ketamine. Both % hits and d', as well as RT were poorer in H-HD (vs. L-HD) and while hit rate and d' was increased by nicotine in H-HD, RT was slowed by ketamine in L-HD.CONCLUSIONSNicotine alleviated ketamine-induced sensory memory impairment and improved attention, particularly in individuals prone to HD. |
| 936 | Heydari, Gholamreza, Tafti, Saeid Fallah. Quit smoking with Champix: Parallel, randomised clinical trial of efficacy for the first time in Iran. European Respiratory Journal. 2012; 40(S56):1378.  **Abstract:** Introduction: Smoking cessation programs were first introduced in Iran in 1997 by the National Research Institute of Tuberculosis and Lung Diseases and up to now, various nicotine replacement therapies have been prescribed. The aim of this study was to evaluate effectiveness of Varenicline for tobacco cessation in this country and compare it with other measures. Materials and Methods: This was a randomized parallel clinical study during 2009-2010. Participants were divided into three parallel groups randomly. The first group received brief counseling on cessation. The second group received nicotine patches 15 mg/daily for 8 weeks and the third group was prescribed Varenicline one 0.5 mg pill daily for the first 3 days, followed by 0.5 mg twice a day for 4 days and subsequently 1 mg twice daily for 8 weeks. Results: The study had 272 participants including 160 men (58.8%). Ninety one people were in the first group, 92 individuals in the second and 89 in the third group. At the end of the first month, 128 people from total (47.1%) succeeded in quitting; this included 17 individuals (18.7%) in the first group, 60 (65.2%) in the second group and 51 (57.3%) in the third group (P=0.000). Follow up at 6 month and a year showed 111 people of total (40.8%) and 58 individual (21.3%) remained smoke free which included 12 (13.2%) and 6 (6.6%) in the first group, 47 (51.1%) and 23 (25%) in the second group and 52 (58.4%) and 29(32.6%) in the third group respectively (P=0.000). Conclusion: Drug treatment can improve success in quitting several fold. Success with Varenicline is slightly better from nicotine replacement treatment. |
| 937 | Tønnesen, Philip, Lauri, Hans, Perfekt, Roland, Mann, Karl, Batra, Anil. Efficacy of a nicotine mouth spray in smoking cessation: a randomised, double-blind trial. The European respiratory journal. 2012;40:548.  **Abstract:** A nicotine mouth spray has advantages over other acute forms of nicotine replacement therapy, such as a faster uptake of nicotine and faster relief of craving. This multicentre, randomised (2:1), double-blind, placebo-controlled efficacy and safety study evaluated self-reported, carbon monoxide-verified continuous abstinence from smoking from week 2 until weeks 6, 24, and 52 in 479 smokers (≥ 1 cigarette per day) who were treated with either active (n=318) or placebo (n=161) spray for 12 weeks and low-intensity counselling at three smoking cessation clinics in Denmark and Germany. Active treatment yielded significantly higher continuous abstinence rates than placebo from week 2 until week 6 (26.1% versus 16.1%; relative success rate (RR) 1.62, 95% CI 1.09-2.41), week 24 (15.7% versus 6.8%; RR 2.30, 95% CI 1.23-4.30), and week 52 (13.8% versus 5.6%; RR 2.48, 95% CI 1.24-4.94). Most adverse events were mild to moderate, and 9.1% of subjects on active spray withdrew due to adverse events, compared to 7.5% on placebo. The overall rate of treatment-related adverse events was 87.4% with active spray versus 71.4% with placebo spray. Nicotine mouth spray delivered significantly higher 6-, 24- and 52-week continuous abstinence rates than placebo. |
| 938 | Berlin, Ivan, Hunneyball, Ian M, Greiling, Doris, Jones, Stephen P, Fuder, Hermann, Stahl, Hans-Detlev. A selective reversible monoamine oxidase B inhibitor in smoking cessation: effects on its own and in association with transdermal nicotine patch. Psychopharmacology. 2012;223:89.  **Abstract:** RATIONALEMonoamine oxidase B (MAO-B) activity is reduced in smokers. A MAO-B inhibitor alone or co-administered with nicotine may mimic the effects of smoking and be a candidate drug for smoking cessation.OBJECTIVEThis study aims to determine the efficacy and safety of EVT302, a selective reversible MAO-B inhibitor, alone and on top of nicotine patch (NP) in smoking cessation.METHODSThis was a randomised, double blind, placebo-controlled phase II, multicentre trial. Smokers (≥10 cigarettes/day) received either EVT302 (N = 145) or placebo (N = 145), or EVT302 (N = 61) or placebo (N = 61) on top of open label NP 21 mg/day for 8 weeks. The main comparison was between EVT302 and placebo without NP. The primary outcome measure was end-of-treatment 4-week continuous abstinence rate (CAR).SECONDARY OUTCOME MEASURESpoint prevalence abstinence rate, saliva cotinine concentrations in the groups without NP, urge to smoke, nicotine withdrawal symptoms and assessment of subjective effects of cigarettes.RESULTSThe 4-week CAR was 15.2 % in the placebo, 17.2 % in the EVT302, 26.8 % in the NP + placebo and 32.8 % in the NP + EVT302 groups, respectively. There was no difference between EVT302 and placebo either alone (adjusted OR: 1.45, 95 % CI: 0.65-3.26) or when co-administered with NP. No statistically significant difference occurred for the secondary outcome measures.CONCLUSIONSThe selective, reversible MAO-B inhibitor EVT302 was not superior to placebo in helping smokers quit, in line with data with selegiline and confirms that MAO-B inhibitors are not effective in smoking cessation. Co-administration of NP does not provide a supplementary benefit. |
| 943 | Dawkins, Lynne, Turner, John, Hasna, Surrayyah, Soar, Kirstie. The electronic-cigarette: effects on desire to smoke, withdrawal symptoms and cognition. Addictive behaviors. 2012;37:970.  **Abstract:** Electronic cigarettes (e-cigarettes) are battery operated devices that deliver nicotine via inhaled vapour. Few studies have evaluated acute effects on craving and mood, and none have explored effects on cognition. This study aimed to explore the effects of the White Super e-cigarette on desire to smoke, nicotine withdrawal symptoms, attention and working memory. Eighty-six smokers were randomly allocated to either: 18 mg nicotine e-cigarette (nicotine), 0mg e-cigarette (placebo), or just hold the e-cigarette (just hold) conditions. Participants rated their desire to smoke and withdrawal symptoms at baseline (T1), and five (T2) and twenty (T3) minutes after using the e-cigarette ad libitum for 5 min. A subset of participants completed the Letter Cancellation and Brown-Peterson Working Memory Tasks. After 20 min, compared with the just hold group, desire to smoke and some aspects of nicotine withdrawal were significantly reduced in the nicotine and placebo group; the nicotine e-cigarette was superior to placebo in males but not in females. The nicotine e-cigarette also improved working memory performance compared with placebo at the longer interference intervals. There was no effect of nicotine on Letter Cancellation performance. To conclude, the White Super e-cigarette alleviated desire to smoke and withdrawal symptoms 20 min after use although the nicotine content was more important for males. This study also demonstrated for the first time that the nicotine e-cigarette can enhance working memory performance. Further evaluation of the cognitive effects of the e-cigarette and its efficacy as a cessation tool is merited. |
| 950 | Eades, Sandra J, Sanson-Fisher, Rob W, Wenitong, Mark, Panaretto, Katie, D'Este, Catherine, Gilligan, Conor, Stewart, Jessica. An intensive smoking intervention for pregnant Aboriginal and Torres Strait Islander women: a randomised controlled trial. The Medical journal of Australia. 2012;197:42.  **Abstract:** OBJECTIVETo determine the effectiveness of an intensive quit-smoking intervention on smoking rates at 36 weeks' gestation among pregnant Aboriginal and Torres Strait Islander women.DESIGNRandomised controlled trial.SETTING AND PARTICIPANTSPregnant Aboriginal and Torres Strait Islander women (n = 263) attending their first antenatal visit at one of three Aboriginal community-controlled health services between June 2005 and December 2009.INTERVENTIONA general practitioner and other health care workers delivered tailored advice and support to quit smoking to women at their first antenatal visit, using evidence-based communication skills and engaging the woman's partner and other adults in supporting the quit attempts. Nicotine replacement therapy was offered after two failed attempts to quit. The control ("usual care") group received advice to quit smoking and further support and advice by the GP at scheduled antenatal visits.MAIN OUTCOME MEASURESelf-reported smoking status (validated with a urine cotinine measurement) between 36 weeks' gestation and delivery.RESULTSParticipants in the intervention group (n = 148) and usual care group (n = 115) were similar in baseline characteristics, except that there were more women who had recently quit smoking in the intervention group than the control group. At 36 weeks, there was no significant difference between smoking rates in the intervention group (89%) and the usual care group (95%) (risk ratio for smoking in the intervention group relative to usual care group, 0.93 [95% CI, 0.86-1.08]; P = 0.212). Smoking rates in the two groups remained similar when baseline recent quitters were excluded from the analysis.CONCLUSIONAn intensive quit-smoking intervention was no more effective than usual care in assisting pregnant Aboriginal and Torres Strait Islander women to quit smoking during pregnancy. Contamination of the intervention across groups, or the nature of the intervention itself, may have contributed to this result.TRIAL REGISTRATIONAustralian New Zealand Clinical Trials Registry ACTRN12609000929202. |
| 951 | Ferguson, Stuart G, Gitchell, Joseph G, Shiffman, Saul. Continuing to wear nicotine patches after smoking lapses promotes recovery of abstinence. Addiction (Abingdon, England). 2012;107:1349.  **Abstract:** AIMS  Smokers who lapse during a cessation attempt are at particularly high risk of relapse, so interventions to help smokers recover from lapses are urgently needed. Two recent studies have suggested continuing to use nicotine patches following a lapse may be a beneficial relapse prevention strategy. However, to date no study that uses approved doses of nicotine patches under real-world conditions has tested this hypothesis.DESIGN AND SETTING  Clinical trial conducted across eight US study sites.PARTICIPANTS AND MEASUREMENTS  Using data from 509 subjects (240 active; 269 placebo) who lapsed during weeks 3-5 of treatment in a randomized, double-blind placebo-controlled trial of 21-mg nicotine patches, we examined whether active nicotine patch use improved the chances of recovering abstinence (7-day point-prevalence) at weeks 6 and 10.FINDINGS  Active patch use (versus placebo) increased the likelihood of recovery from a lapse both at 6 weeks [8.3% versus 0.8%; relative risk (RR) = 11.0, P < 0.001] and at 10 weeks (9.6% versus 2.6%; RR = 3.7, P < 0.001).CONCLUSIONS  Continuing treatment to aid smoking cessation with active patches promotes recovery from lapses. Smokers should be encouraged to persist with patch treatment if they lapse to smoking. |
| 954 | Locatelli, Isabella, Clair, Carole, Collet, Tinh-Hai, Rodondi, Nicolas, Cornuz, Jacques. The interaction between gender and amount of smoking in the prediction of one-year weight gain after smoking cessation. Journal of General Internal Medicine. 2012;27:S333.  **Abstract:** BACKGROUND: Smoking cessation is associated with weight gain. We aimed to determine the amount of weight gain and its predictors among primary care smokers after 1 year of smoking cessation. METHODS: We merged two randomized controlled studies (RCTs) assessing new interventions helping smokers to quit. The first RCT (n= 477) evaluated a program of moderate intensity physical activity; the second (n=536) assessed the effect of carotid plaque screening by ultrasound. Both RCTs studied the intervention effect on smoking cessation rates after one year compared to a control program of intensive counseling and nicotine replacement therapies. The similarities between both RCTs and the identical control programs allowed us to merge the two studies, regardless of the individual assignment to the RCT-specific control/intervention group. The analysis included all subjects that made at least one quit attempt during the study (n=810). We used a multivariable linear regression model to test the effect on the mean weight gain at one year for the following potential predictors and all their interactions: age (dichotomized at the median, 47 years), sex, education level, number of cigarettes at baseline and duration of smoking abstinence. We applied a backward procedure to select the best model (p<0.05). RESULTS: At baseline, subjects (mean age 46.9 years, 43.7% women) smoked a median of 22 cigarettes per day, ranging from 8 to 70, and median smoking abstinence was 18 weeks during follow-up. After 1 year, the mean weight gain was 2.6 kilograms (kg, 5.7 lbs) (3.6% of the initial weight). Among smokers that were abstinent more than 18 weeks, the mean weight gain was 3.8 kg (5.3% of the initial weight). Age, sex and number of cigarettes at baseline were significantly associated with weight gain at one year. Older subjects (>47 years) gained 0.7 kg less than younger (p=0.001). We found an interaction between sex and the number of cigarettes smoked at baseline (p=0.003): men gained more weight than women when smoking <30 cigarettes per day; the reverse was observed for heavy smokers (Figure). According to this model, a woman younger than 47 and smoking more than 30 cigarettes/day could gain up to 5.5 kg on average one year after smoking cessation (9% of her initial weight). A man of the same age group and smoking the same amount would expect a mean weight gain of 4.5 kg (6% of his initial weight). CONCLUSIONS: Age, sex and number of cigarettes at baseline predicted 1-year weight gain among smokers who quit, and a significant interaction between sex and number of cigarettes smoked was found. Young women smoking heavily at baseline were those with the highest risk of weight gain after quitting and should be followed closely by their physicians during abstinence periods. (Figure Presented). |
| 961 | Barrett, Sean P, Darredeau, Christine. The acute effects of nicotine on the subjective and behavioural responses to denicotinized tobacco in dependent smokers. Behavioural pharmacology. 2012;23:221.  **Abstract:** Both nicotine and various non-nicotine smoking factors are believed to contribute to tobacco addiction but their relative roles remain incompletely understood. This study aimed to help clarify these roles by examining acute interactions between nicotine and denicotinized tobacco (DT). During two randomized blinded sessions, the effects of a quick-release 4 mg nicotine lozenge (NL) versus placebo lozenge (PL) on the subjective and behavioural responses to DT were examined in 27 (14 men) dependent, daily smokers. Participants were administered NL or PL for 30 min before receiving one initial DT cigarette. Participants could then earn additional DT cigarette puffs over the following 60 min. Subjective state was assessed using the Questionnaire of Smoking Urges-Brief and visual analogue scales at baseline, postlozenge and postinitial DT cigarette. Relative to PL, NL was associated with increased alertness as well as with reduced levels of DT self-administration (P<0.01). The administration of a single DT cigarette was followed by a reduction in craving under both lozenge conditions (P<0.001), an effect that was significantly greater in women (P<0.01). Moreover, DT administration was associated with increased ratings of 'pleasant', 'satisfied', 'stimulated' and 'relaxed', as well as with decreased ratings of 'anxious' (P's<0.01), independent of lozenge condition. The findings suggest that both nicotine and non-nicotine smoking factors may make important contributions towards the addictive properties of tobacco. |
| 962 | Lee, Susan M., Jones, Philip, Morley-Forster, Patricia K., Landry, Jennifer, Buhrmann, Ozzie. Effectiveness of a perioperative smoking cessation program: An RCT. Canadian Journal of Anesthesia. 2012; 59:92.  **Abstract:** Introduction: Cigarette smoking by surgical patients is associated with increased complications(1), particularly perioperative respiratory problems and poor wound-healing(2). Facing surgery is a “teachable moment” that may motivate patients to engage in permanent smoking cessation(3). Perioperative smoking cessation interventions have been shown to be effective in European populations in reducing perioperative smoking(4) and perioperative complications(5,6). The purpose of this study was to determine if a perioperative smoking cessation intervention designed for a busy preadmission clinic would be successful in reducing smoking rates and intraoperative and immediate postoperative complications in a Canadian setting. The primary outcome is the rate of smoking cessation as confirmed by exhaled carbon monoxide breath test. Secondary outcomes include perioperative complications and unanticipated hospital admission. Methods: This randomized controlled trial was conducted at a university-affiliated hospital. Following approval of the research protocol by the local research ethics board, adult smokers were identified in the preadmission clinic at least 3 weeks preoperatively. After informed consent, baseline data were collected, including selfreported smoking status, exhaled carbon monoxide level and modified Fagerstrom test for nicotine dependency(7). Patients were randomized to either intervention or control groups. The control group received no specific smoking cessation intervention. The intervention group received 1) brief counselling by the preadmission nurse, 2) brochures on smoking cessation, 3) referral to the Canadian Cancer Society's Smokers' Helpline, and 4) a free 6-week supply of transdermal nicotine replacement therapy. All caregivers on the operative day were blinded to group assignment. Blinded observers collected self-reported smoking status, exhaled CO level, and using a standardized data sheet, obtained information on perioperative complications from the anesthesiologist and PACU nurse. Patients were called 30-days postoperatively for self-reported smoking status and postoperative complications. Results: Between October 2010 and January 2012, 153 patients were recruited into the study, representing 91% recruitment. Data were not unblinded at the time of this abstract submission pending full recruitment to the study, which is expected to be complete by March 2012. Aggregate data to date show that 46.4% of the 138 analyzed patients either achieved smoking cessation (8.7%) or reduction (37.7%) on the day of surgery. Intraoperative or postoperative complications occurred in 11.6% patients and 2.9% had an unanticipated hospital admission. Discussion: One of the objections to more widespread use of smoking cessation interventions in the preadmission clinic is that it is too labour-intensive. The results of this study will show whether a smoking cessation intervention, designed to minimize additional use of physician or nursing time, introduced 3 weeks preoperatively results in decreased smoking rates on the day of surgery. Examination of perioperative complications may aid in determining whether a three week period is sufficient to demonstrate improvement in clinical outcomes. |
| 968 | Heydari, Gholamreza. Quit smoking with champix: Parallel, randomised clinical trial of efficacy for the first time in Iran. Circulation. 2012;125:e895.  **Abstract:** Introduction: Tobacco consumption like other habit forming substances is difficult to let go. Treatment includes a variety of methods including brief clinical counseling and with medication. Smoking cessation programs were first introduced in Iran in 1997 and up to now, various nicotine replacement therapies have been prescribed. Objectives: The aim of this study was to evaluate effectiveness of Varenicline for tobacco cessation. Methods: This was a randomized parallel clinical study at tobacco cessation clinics during 2009-2010. Participants were smokers willing to quit visiting the first time a smoking cessation clinic in Tehran divided into three parallel groups randomly. The first group received brief counseling on cessation. The second group received nicotine patches 15 mg/daily for 8 weeks and the third group was prescribed Varenicline one 0.5 mg pill daily for the first 3 days, followed by 0.5 mg twice a day for 4 days and subsequently 1 mg twice daily for 8 weeks. Results: The study had 272 participants. 91 individuals were in the first group, 92 in the second and 89 in the third group. At the end of the first month, 128 people from total (47.1%) succeeded in quitting; this included 17 individuals (18.7%) in the first group, 60 (65.2%) in the second group and 51 (57.3%) in the third group (P=0.000). Follow up at one year showed 58 individual (21.3%) remained smoke free which included 6 (6.6%) in the first group, 23 (25%) in the second group and 29(32.6%) in the third group (P=0.000). Conclusion: Drug treatment can improve success in quitting several fold. Success with Varenicline is slightly better but not significantly different from nicotine replacement treatment. |
| 969 | Carmody, Timothy P, Delucchi, Kevin, Duncan, Carol L, Banys, Peter, Simon, Joel A, Solkowitz, Sharon N, Huggins, Joy, Lee, Sharon K, Hall, Sharon M. Intensive intervention for alcohol-dependent smokers in early recovery: a randomized trial. Drug and alcohol dependence. 2012;122:186.  **Abstract:** INTRODUCTIONThe purpose of this study was to investigate the efficacy of an intensive tobacco cessation intervention for alcohol-dependent smokers in early recovery.METHODSA total of 162 alcohol-dependent smokers were randomized to either intensive intervention for smoking cessation or usual care. The intensive intervention consisted of 16 sessions of individual cognitive behavior therapy (CBT) and combination nicotine replacement therapy that lasted 26 weeks. Usual care involved referral to a free-standing smoking cessation program that provided smoking cessation counseling of varying duration and guideline-concordant medications. The primary cessation outcome was verified 7-day point prevalence abstinence (PPA) at 12, 26, 38, and 52 weeks.RESULTSAt 12 and 26 weeks, the verified 7-day point-prevalence quit rate was significantly higher for the intensive intervention group than for the usual care group (both p=0.03). However, the quit rates for the two treatment groups were not significantly different at 38 or 52 weeks. Verified 30-day alcohol abstinence rates were not significantly different for the two treatment groups at any of the follow-up assessments.CONCLUSIONSThe intensive smoking cessation intervention yielded a higher short-term smoking quit rate without jeopardizing sobriety. A chronic care model might facilitate maintenance of smoking cessation during the first year of alcohol treatment and perhaps for longer periods of time. It is hoped that studies such as this will inform the development of more effective interventions for concurrent alcohol and tobacco use disorders. |
| 970 | Marine Gambaryan, M.H., Kalinina, A.M., Grigoryan, T.A., Vigodin, V.A.. Intervention strategies for smoking cessation in primary health care in Russia: Opportunities and challenges. European Journal of Preventive Cardiology. 2012;19:S38.  **Abstract:** Smoking is big public health problem in Russia with highest prevalence of smokers and smoking related mortality in European Region. Since 3 years Russia is implementing FCTC and National Policy against tobacco use for years 2010-1015, launched 2010. One of the biggest challenges is that Health Care System is not prepared enough for effective tobacco control (TC). The aim of this study is to assess opportunities, challenges for and effectiveness of different smoking intervention strategies in various settings in pulmonary hypertension C to define algorithms for effective TC. Pilot studies in 2 different settings undertaken: 1) randomized control trial for smoking cessation (SC) interventions in cardiologist's practice of municipal outpatient clinic was conducted in 60 patients. Smoking cessation intensive advice combined with nicotine replacement therapy and without nicotine replacement therapy was offered to intervention and control groups, 2) prospective study to assess the impact of workplace health promotion programs (WHPP) on SC rates was conducted in 348 health professionals at 4 municipal outpatient clinics in 12-months period. Average age of patients of outpatient clinic was 49+/-12; 75,9% had reported arterial hypertension (AH), 27,6%-myocardial infarction, 45% high blood cholesterol (CH) levels, 45%-chronic obstructive pulmonary disease. At the first visit 77.6% of the patients were diagnosed with high blood cholesterol, and 94.8%-with chronic obstructive pulmonary disease GOLD stage II and IIImeaning 51% and 58.2% respectively were never diagnosed with the disease before (p < 0,05). By the end of the treatment smoking cessation rate in the intervention group was 46.7% compared to 3.3% of controls (p<0,001). Significant increase of lung function parameter was observed in intervention group compared with the controls (p<0,01). A decrease in stress levels was observed in 32% in intervention group and 20% in controls (p=0.07); among quitters the stress levels did not change in 50% and decreased in 35.7% cases. Among participants of WHPP 44% had AH, 61% were overweight, 69.3% had high CH level, and 30.7% were regular smokers. After 12-months in settings where intensive intervention was applied (group HE interventions+HP handouts) a significant decrease of 17% of smoking rates was reached, in the reference settings (only handouts given) no significant changes observed. Prevention and early detection of smoking related diseases require systematic identification of smokers and provision of smoking cessation assistance, implemented at all levels of pulmonary hypertensionC. Workplaces are ideal settings for HP interventions, where SC may be implemented as part of the program. Intensive and assisted SC interventions significantly increase the success rates. |
| 972 | Ferketich, Amy K, Otterson, Gregory A, King, Mark, Hall, Nathan, Browning, Kristine K, Wewers, Mary Ellen. A pilot test of a combined tobacco dependence treatment and lung cancer screening program. Lung cancer (Amsterdam, Netherlands). 2012;76:211.  **Abstract:** Lung cancer screening with computed tomography has demonstrated a significant reduction in mortality. While these findings are important for the lung cancer research field, the most important risk factor for lung cancer, i.e. smoking, should not be ignored. We performed a pilot study to examine the feasibility of delivering a program that included both tobacco dependence treatment and lung cancer screening. The objectives of this study were to: (1) estimate the proportion of smokers who complied with a 12-week treatment protocol that included both tobacco dependence treatment and lung cancer screening, (2) obtain preliminary estimates of abstinence and quit attempts at 4 and 6 months, and (3) obtain preliminary estimates of the cognitive social health information processing (C-SHIP) constructs and how they change following the intervention. In this randomized pilot study, 18 volunteers completed a 12-week protocol: half received the tobacco dependence treatment program before a CT scan (BCT) and the other received the CT scan first, followed by the treatment program (ACT). The treatment protocol included both nurse-delivered telephone counseling and either nicotine replacement therapy or varenicline. Only one person did not complete all follow-up evaluations. At 4 months post enrollment, the carbon monoxide confirmed quit rates were 33.3% in the BCT arm and 22.2% in the ACT arm (27.8% overall), and all but one had made a 24-h attempt to quit. At 6 months the confirmed abstinence decreased to 22.1% in the BCT arm and 11.1% in the ACT arm (16.7% overall), and 72.2% of participants had made a 24-h quit attempt. These preliminary results suggest that it might be better to deliver treatment before the screening test. Future randomized trials with a larger sample size are needed to confirm these findings. |
| 975 | Ferguson, Janet, Docherty, Graeme, Bauld, Linda, Lewis, Sarah, Lorgelly, Paula, Boyd, Kathleen Anne, McEwen, Andy, Coleman, Tim. Effect of offering different levels of support and free nicotine replacement therapy via an English national telephone quitline: randomised controlled trial. BMJ (Clinical research ed.). 2012;344:e1696.  **Abstract:** OBJECTIVETo compare the effects of free nicotine replacement therapy or proactive telephone counselling in addition to standard smoking cessation support offered through a telephone quitline.DESIGNParallel group, 2 × 2 factorial, randomised controlled trial.SETTINGNational quitline, England.PARTICIPANTS2591 non-pregnant smokers aged 16 or more residing in England who called the quitline between February 2009 and February 2010 and agreed to set a quit date: 648 were each randomised to standard support, proactive support, or proactive support with nicotine replacement therapy, and 647 were randomised to standard support with nicotine replacement therapy.INTERVENTIONSTwo interventions were offered in addition to standard support: six weeks' nicotine replacement therapy, provided free, and proactive counselling sessions (repeat telephone calls from, and interaction with, cessation advisors).MAIN OUTCOME MEASURESThe primary outcome was self reported smoking cessation for six or more months after the quit date. The secondary outcome was cessation validated by exhaled carbon monoxide measured at six or more months.RESULTSAt six months, 17.7% (n = 229) of those offered nicotine replacement therapy reported smoking cessation compared with 20.1% (n = 261) not offered such therapy (odds ratio 0.85, 95% confidence interval 0.70 to 1.04), and 18.2% (n = 236) offered proactive counselling reported smoking cessation compared with 19.6% (n = 254) offered standard support (0.91, 0.75 to 1.11). Data validated by carbon monoxide readings changed the findings for nicotine replacement therapy only, with smoking cessation validated in 6.6% (85/1295) of those offered nicotine replacement therapy compared with 9.4% (122/1296) not offered such therapy (0.67, 0.50 to 0.90).CONCLUSIONSOffering free nicotine replacement therapy or additional (proactive) counselling to standard helpline support had no additional effect on smoking cessation.TRIAL REGISTRATIONClinicalTrials.gov NCT00775944. |
| 978 | Schnoll, Robert A, Wileyto, E Paul, Lerman, Caryn. Extended duration therapy with transdermal nicotine may attenuate weight gain following smoking cessation. Addictive behaviors. 2012;37:565.  **Abstract:** AIMPeople who quit smoking often gain 11-12 lb, on average, which can frequently lead to a relapse to smoking. This study evaluated whether extended vs. standard duration treatment with nicotine patch helps those able to quit smoking to reduce cessation-induced weight gain and explored nicotine patch adherence as a mediator of treatment effects.DESIGN AND SETTINGWe examined data from a completed randomized placebo-controlled clinical trial of extended (24 weeks) vs. standard (8 weeks plus 16 weeks of placebo) transdermal nicotine patch therapy. Changes in measured weight over 24 weeks were compared across the two treatment arms, controlling for gender, baseline smoking rate, and previous weight. Adherence to patch use was assessed using self-report of daily use over 24 weeks.PARTICIPANTS139 clinical trial participants who were confirmed to be abstinent at weeks 8 and 24.FINDINGSCompared to participants who received 8 weeks of nicotine patch therapy, participants who received 24 weeks of treatment showed significantly less weight gain from pre-treatment to week 24 (β=-4.76, 95% CI: -7.68 to -1.84, p=.002) and significantly less weight gain from week 8 to week 24 (β=-2.31, 95% CI: -4.39 to -0.23, p=.03). Extended treatment increased patch adherence which, in turn, reduced weight gain; patch adherence accounted for 20% of the effect of treatment arm on weight gain.CONCLUSIONCompared to 8 weeks of transdermal nicotine therapy, 24 weeks of patch treatment may help to reduce the weight gain that is typical among smokers who are able to achieve abstinence from tobacco use. Extended treatment increased nicotine patch adherence which, in turn, reduced weight gain. |
| 979 | Brunner Frandsen, Nicole, Sørensen, Margit, Hyldahl, Tanja Kirstine, Henriksen, Rikke Mitzi, Bak, Søren. Smoking cessation intervention after ischemic stroke or transient ischemic attack. A randomized controlled pilot trial. Nicotine & tobacco research : official journal of the Society for Research on Nicotine and Tobacco. 2012;14:443.  **Abstract:** BACKGROUNDSmoking cessation is widely recommended for secondary stroke prevention. However, little is known about the efficacy of smoking cessation intervention after stroke or transient ischemic attack (TIA).METHODSNinety-four smokers under age 76, admitted with ischemic stroke or TIA were randomized to minimal smoking cessation intervention or intensive smoking cessation intervention. All patients attended a 30-min individual counseling by the study nurse. Patients randomized to intensive smoking cessation intervention also participated in a 5-session outpatient smoking cessation program by an authorized smoking cessation instructor, a 30-min outpatient visit after 6 weeks, and 5 telephone counseling sessions by the study nurse. Free samples of nicotine replacement therapy were offered as part of the intensive smoking cessation program. Smoking cessation rates at 6 months were determined by self-report and verified by measurement of exhaled carbon monoxide (CO). Fewer patients than expected were recruited, which renders this report a pilot study.RESULTSThe 6-month self-reported smoking cessation rate was 37.8% in the minimal intervention group and 42.9% in the intensive intervention group. Smoking cessation rates verified by exhaled CO levels in the minimal intervention group and the intensive intervention group were 28.9% and 32.7%, respectively. No difference was found between the two groups (χ(2) = 0.16, p = .69).CONCLUSIONSOverall smoking cessation rates were moderate and comparable to the results from other studies. Intensive smoking cessation intervention was not superior to short smoking cessation intervention. Thus, other factors than intensity of smoking cessation intervention might influence the smoking cessation rates after stroke or TIA. |
| 983 | Singh, Prabhat K., Gautam, Sujeet K., Gupta, Devendra, Agarwal, Anil, Singh, Uttam. Evaluation of trans-dermal nicotine patch for attenuation of venous cannulation pain. British Journal of Anaesthesia. 2012;108:ii350.  **Abstract:** Introduction:Venous cannulation pain, the first painful experience by the patient in the operation room is often discomforting and stressful for both patients and health care professionals. Various pharmacological and nonpharmacological measures have been tried to minimize venous cannulation pain with variable success. Objective: The present study was planned to evaluate the efficacy of trans-dermal nicotine patch for attenuating venous cannulation pain. Methods: Sixty adults (16-60 yrs), ASA physical status I and II, of either sex, undergoing laparoscopic cholecystectomy, were included in this prospective, randomized, double blind and placebo controlled clinical study. Patients were divided into 2 groups of 30 each. Control group: received placebo patch; Nicotine group: received trans-dermal nicotine patch. The patches were applied at the proposed venous cannulation site 1 hr prior to cannulation with 20 G IV cannula; venous cannulation pain was assessed on a visual analogue scale (VAS) of 0-100 mm (01/4no pain, 1001/4worst possible pain). Data were analyzed using student's T test, chi square test, Mann Whitney U test and Fisher's exact test. P,0.05 was considered significant. Results: The incidence of venous cannulation pain in the nicotine group (63%) was significantly lower as compared to the control group (100%; P,0.01). Severity of venous cannulation pain [median VAS (inter-quartile range)] was also significantly reduced in the nicotine group 20 (30) as compared to the control group 50 (30) (P,0.001). Incidence of side effects were similar among the groups (P.0.05). Conclusion: Application of trans-dermal nicotine patch at the venous cannulation site 1 h before venous cannulation decreases both the incidence and severity of venous cannulation pain. |
| 987 | Coleman, Tim, Cooper, Sue, Thornton, James G, Grainge, Matthew J, Watts, Kim, Britton, John, Lewis, Sarah. A randomized trial of nicotine-replacement therapy patches in pregnancy. The New England journal of medicine. 2012;366:808.  **Abstract:** BACKGROUNDNicotine-replacement therapy is effective for smoking cessation outside pregnancy and its use is widely recommended during pregnancy. We investigated the efficacy and safety of nicotine patches during pregnancy.METHODSWe recruited participants from seven hospitals in England who were 16 to 50 years of age with pregnancies of 12 to 24 weeks' gestation and who smoked five or more cigarettes per day. Participants received behavioral cessation support and were randomly assigned to 8 weeks of treatment with active nicotine patches (15 mg per 16 hours) or matched placebo patches. The primary outcome was abstinence from the date of smoking cessation until delivery, as validated by measurement of exhaled carbon monoxide or salivary cotinine. Safety was assessed by monitoring for adverse pregnancy and birth outcomes.RESULTSOf 1050 participants, 521 were randomly assigned to nicotine-replacement therapy and 529 to placebo. There was no significant difference in the rate of abstinence from the quit date until delivery between the nicotine-replacement and placebo groups (9.4% and 7.6%, respectively; unadjusted odds ratio with nicotine-replacement therapy, 1.26; 95% confidence interval, 0.82 to 1.96), although the rate was higher at 1 month in the nicotine-replacement group than in the placebo group (21.3% vs. 11.7%). Compliance was low; only 7.2% of women assigned to nicotine-replacement therapy and 2.8% assigned to placebo used patches for more than 1 month. Rates of adverse pregnancy and birth outcomes were similar in the two groups.CONCLUSIONSAdding a nicotine patch (15 mg per 16 hours) to behavioral cessation support for women who smoked during pregnancy did not significantly increase the rate of abstinence from smoking until delivery or the risk of adverse pregnancy or birth outcomes. However, low compliance rates substantially limited the assessment of safety. (Funded by the National Institute for Health Research Health Technology Assessment Programme; Current Controlled Trials number, ISRCTN07249128.). |
| 989 | Heydari, G, Talischi, F, Tafti, S F, Masjedi, M R. Quitting smoking with varenicline: parallel, randomised efficacy trial in Iran. The international journal of tuberculosis and lung disease : the official journal of the International Union against Tuberculosis and Lung Disease. 2012;16:268.  **Abstract:** BACKGROUNDSmoking cessation programmes were first introduced in Iran in 1997. To date a number of types of nicotine replacement therapy have been prescribed.OBJECTIVETo evaluate the effectiveness of varenicline for tobacco cessation.METHODSThis was a randomised parallel clinical study conducted in 2010. Participants were smokers willing to quit who were visiting a smoking cessation clinic for the first time and were randomly divided into three groups: all three groups received brief counselling on cessation, Group 2 received nicotine patches and Group 3 was prescribed varenicline for 8 weeks.RESULTSThere were 272 participants in the study: 91 in Group 1, 92 in Group 2 and 89 in Group 3. At the end of the first month, 128 of the 272 subjects (47.1%) succeeded in quitting: 17 (18.7%) in Group 1, 60 (65.2%) in Group 2 and 51 (57.3%) in Group 3 (P = 0.000). At follow-up after 12 months, 58 subjects (21.3%) remained smoke-free, of whom 6 (6.6%) were in Group 1, 23 (25%) in Group 2 and 29 (32.6%) in Group 3 (P = 0.000).CONCLUSIONIn the study, varenicline treatment was slightly more effective than but not significantly different from nicotine replacement therapy. |
| 991 | Bolt, Daniel M, Piper, Megan E, Theobald, Wendy E, Baker, Timothy B. Why two smoking cessation agents work better than one: role of craving suppression. Journal of consulting and clinical psychology. 2012;80:54.  **Abstract:** OBJECTIVEThis research examined why smokers receiving combination medication for smoking cessation are more likely to quit smoking than are those who receive either single agent (monotherapy) or placebo.METHODData were collected from 1,504 current smokers (58.2% women, 83.9% White; mean age = 44.67 years, SD = 11.08) participating in a cessation clinical trial who were randomized to 1 of 6 cessation pharmacotherapy conditions (placebo, nicotine patch, nicotine lozenge, bupropion, nicotine patch + nicotine lozenge, and bupropion + nicotine lozenge). Participants completed ecological momentary assessments 4 times a day, concerning 5 hypothesized mediators (negative affect, positive affect, craving, smoking expectations, and withdrawal) of pharmacotherapy effects. Medications were provided for 8-12 weeks post-quit along with 6 individual counseling sessions. Mediational paths were estimated via a novel Bayesian approach with estimation of multiple mediator models.RESULTSBiochemically confirmed 8-week abstinence was the outcome variable, with the monotherapy and combination pharmacotherapy composites producing 45% (n = 689) and 54% (n = 478) abstinence rates, respectively. The univariate models suggested that the combination treatments produced higher abstinence rates than the monotherapies because of greater suppression of withdrawal, craving, and smoking expectations. However, multiple mediator models showed that the suppression of craving on the quit day produced the strongest mediational effects and could account for the mediational effects of other tested variables.CONCLUSIONSuppression of craving on the quit day significantly mediates the clinical effects of monotherapies and combination smoking pharmacotherapies, and the higher abstinence rates for combination therapy versus monotherapies appear primarily due to greater craving suppression. |
| 992 | Newhouse, P, Kellar, K, Aisen, P, White, H, Wesnes, K, Coderre, E, Pfaff, A, Wilkins, H, Howard, D, Levin, E D. Nicotine treatment of mild cognitive impairment: a 6-month double-blind pilot clinical trial. Neurology. 2012;78:91.  **Abstract:** OBJECTIVETo preliminarily assess the safety and efficacy of transdermal nicotine therapy on cognitive performance and clinical status in subjects with mild cognitive impairment (MCI).METHODSNonsmoking subjects with amnestic MCI were randomized to transdermal nicotine (15 mg per day or placebo) for 6 months. Primary outcome variables were attentional improvement assessed with Connors Continuous Performance Test (CPT), clinical improvement as measured by clinical global impression, and safety measures. Secondary measures included computerized cognitive testing and patient and observer ratings.RESULTSOf 74 subjects enrolled, 39 were randomized to nicotine and 35 to placebo. 67 subjects completed (34 nicotine, 33 placebo). The primary cognitive outcome measure (CPT) showed a significant nicotine-induced improvement. There was no statistically significant effect on clinician-rated global improvement. The secondary outcome measures showed significant nicotine-associated improvements in attention, memory, and psychomotor speed, and improvements were seen in patient/informant ratings of cognitive impairment. Safety and tolerability for transdermal nicotine were excellent.CONCLUSIONThis study demonstrated that transdermal nicotine can be safely administered to nonsmoking subjects with MCI over 6 months with improvement in primary and secondary cognitive measures of attention, memory, and mental processing, but not in ratings of clinician-rated global impression. We conclude that this initial study provides evidence for nicotine-induced cognitive improvement in subjects with MCI; however, whether these effects are clinically important will require larger studies.CLASSIFICATION OF EVIDENCEThis study provides Class I evidence that 6 months of transdermal nicotine (15 mg/day) improves cognitive test performance, but not clinical global impression of change, in nonsmoking subjects with amnestic MCI. |
| 995 | Pai, Anuradha, Prasad, Shesha. Attempting tobacco cessation--an oral physician's perspective. Asian Pacific journal of cancer prevention : APJCP. 2012;13:4973.  **Abstract:** INTRODUCTIONTobacco use is a global health care problem. Repetitive exposure to nicotine produces neuroadaptation resulting in nicotine dependence. Smoking is associated with a range of diseases, causing high levels of morbidity and mortality and is one of the leading causes of preventable deaths, with more than 4.6 million smokers worldwide dying each year from smoking related illnesses. Stopping smoking has major health benefits. Quitting at any age provides both short and long term benefits.MATERIALS AND METHODS45 patients attending the outpatient department at the Oxford Dental College, Bangalore, were randomly allocated to three groups of interventions namely placebo, counseling and nicotine replacement therapy (NRT). Initially each one was assessed for carbon monoxide levels using a breath analyser (pico smokerlyser bedfont UK). They were followed up for six months and the carbon monoxide levels were again assessed using the same instrument. The paired t test was used to compare the results before and after the intervention.RESULTSThe scores before the initiation of intervention and after treatment were compared and all three interventions were found to be statistically significant after six months. It was noticed that patients with very low or low dependence followed by high dependence had good response in the placebo group (68% and 47.6% respectively), in the counseling group maximum response was seen in the medium followed by the very low group (61% and 59% respectively), and maximum response was seen in very high followed by the very low group with NRT (78.7% and 60.5% respectively).CONCLUSIONThe inference that can be drawn from the present study is that non-invasive, non pharmacological methods like placebo and counseling are effective in low to medium groups, and NRT is effective with higher nicotine dependence. |
| 996 | Knott, V.J., Millar, A.M., McIntosh, J.F., Shah, D.K., Fisher, D.J., Blais, C.M., Ilivitsky, V., Horn, E.. Neurochemical modulation of auditory processing in healthy controls stratified for low and high auditory hallucinatory experiences. European Psychiatry. 2012; 27:1.  **Abstract:** Introduction: Contributing to poor global functioning, auditory hallucinations (AH) also interfere with elementary cognitive processes, including auditory discrimination. This is evidenced in schizophrenic (SZ) hallucinators (vs. non-hallucinators) by a greater reduction of the MMN, an auditory event-related brain potential (ERP) generated in part by NMDA receptor activity and normalized with nicotinic (nACh) agonist treatment. Objectives: To increase our understanding of NMDA-nACh interactions with auditory processing, using healthy young adults varying in their degree of experience with AH, thereby reducing the confounding influence of illness chronicity and medication associated with the study of SZ patients. Aims: To investigate MMN differences between low and high AH subjects during separate and combined administration of ketamine, an NMDA antagonist, and nicotine, an nACh agonist. Methods: In 40 healthy controls, all rated for AH with the Bell Object Relations and Reality Testing Inventory, MMN to frequency deviants was assessed in a randomized, placebo-controlled crossover design involving the separate and combined administration of a intravenous sub-psychotomimetic dose of ketamine (0.04 mg/kg) and a dose of nicotine gum (4 mg). Results: In high AH subjects, ketamine reduced MMN, with the resulting amplitude being smaller than that of low AH subjects. This ketamine-induced MMN reduction was evident only with placebo gum; furthermore combined nicotine-ketamine treatment acted to increase MMN in high scorers. Conclusions: AH in otherwise healthy individuals is associated with heightened sensitivity to NMDA receptor blockade, the effects of which are moderated by nicotinic neurotransmission. Both neurotransmitters may interact to moderate auditory processing and AH in SZ. |
| 997 | Zincir, Selma Bozkurt, Kaymak, Esra, Sünbül, Esra Aydin, Zincir, Nihat. Comparison of the effects of varenicline, bupropion and nicotine replacement therapy on smoking cessation program. Klinik Psikofarmakoloji Bulteni. 2012;22:S35.  **Abstract:** Objective: The most effective aproach to individuals who wants to and decided to stop cigarettes smoking is behavior training with the application of motivational support and pharmacological therapy. This study aims to compare the effects of varenicline, slow-release bupropion, nicotine replacement therapy (NRT) on the success and the maintenance of smoking cessation program. Method: 300 consecutive patient from smoking cessation clinic whose nicotine dependence were confirmed were evaluated in a randomized manner. 262 patients completed the study. In this study, we used Fagerström nicotine dependence test, semistructured clinic interview and hospital anxiety and depression scale (HAD) for clinical follow-up. Carbonmonoxyde (CO) levels of the patients were measured with carboxymeter. Patients had used the determined randomised pharmacological treatment option for 12 weeks. Results: There were no significant difference between the therapy groups according to age, gender and education levels (p>0.05). Fagerstöm scores and the number of cigarettes consumed per day in NRT group were significantly lower than the other therapy groups (p<0.05). There were a significant decrease compared with the initial measurements in HAD- A 12 week scores in bupropion and NRT groups (p<0.05). Smoking cessation rates were 57.1% in bupropion group (n=77), 54.8% in NRT group (n=73) , 72.3% in varenicline group (n=101) and 50% in bupropion + NRT group (n=10). Conclusion: According to the our findings, 61.8% of all patients participating in this study continued the smoking cessation behavior during the 12 week follow-up period. It seems that varenicline is better than the other pharmacological therapy modalities for smoking cessation programs. The lower anxiety scores in bupropion and NRT groups might have increased their success rates. |
| 1002 | Cabioǧlu, Mehmet Tuǧrul, Kutlu, Ruhuşen, Albayrak, Canan, Marakoǧlu, Kamile, Ergene, Neyhan. Changes in the levels of serum beta endorphin, serotonin, adrenaline, noradrenaline and dopamine during smoking cessation by electroacupuncture and nicotine patch. Turkiye Klinikleri Cardiovascular Sciences. 2012;24:95.  **Abstract:** Objective: The purpose of this study is to investigate the changes in the levels of serum beta endorphin, serotonin, adrenaline, noradrenaline and dopamine during smoking cessation with acupuncture and nicotine patch. Material and Methods: This study consists of 42 volunteer persons who applied to quit smoking to Outpatient Family Medicine Clinic of Meram Medical Faculty. The participants were divided randomly into two groups as: 1- Electroacupuncture (EA) group (n=21) and 2- Nicotine patch group (n=21). EA was performed once a day for a period of 30 minutes in the first three days and then once every other day for 30 days. Nicotine patch was applied on the clean, hairless parts of the skin in several steps; for example, 21, 14, and 7 mg for a period of two months. The serum beta endorphin, serotonin, adrenaline, nor adrenaline, dopamine levels were measured before and after acupuncture and nicotine patch therapy. Results: When we compare serum serotonin, adrenaline, noradrenaline, dopamine and beta-endorphin levels on the 1st and 30th days in the EA group, we determined that beta endorphin (p=0.000), serotonin (p=0.009), adrenaline (p=0.007) and noradrenaline (p=0.007) levels increased but dopamine (p=0.898) levels did not change. In the nicotine patch group, serum serotonin (p=0.045) levels increased but adrenaline (p=0.318), beta endorphin (p=0.463), noradrenaline (p=0.099) and dopamine (p=0.655) levels did not change. When we compared two groups at the end of 30th day, we found that the serum noradrenaline (p<0.000), dopamin (p<0.028) levels were statistically higher in EA group than nicotine patch group. Conclusion: We think that EA and nicotin patch application makes it easy to quit smoking for those trying to give up smoking with psychological support. There was no statistically difference in the rates of smoking cessation in both groups. Therefore, both electroacupuncture and nicotin patch application are effective to stop smoking. Copyright © 2012 by Türkiye Klinikleri. |
| 1006 | Sutherland, Matthew T., Carroll, Allison J., Jo Salmeron, Betty, Ross, Thomas J., Stein, Elliot A.. Insula functional connectivity with default-mode network is modulated by varenicline and nicotine in abstinent smokers. Neuropsychopharmacology. 2011;36:S367.  **Abstract:** Background: The insula appears to play a critical role in nicotine addiction. Emerging evidence from intrinsic network connectivity studies also implicates insula in the modulation of dynamic activity between competitively interacting brain networks associated with internally-oriented cognitive operations (default-mode network: DMN) and externally-oriented attentional processes (executive control network: ECN). Altered network dynamics may underlie cognitive impairment and affective dysregulation often noted during smoking abstinence. Varenicline, via actions on nicotinic acetylcholine receptors, is thought to reduce smoking behaviors by ameliorating abstinence-induced effects during a quit attempt, while also dampening nicotine-induced effects during a smoking lapse. We exploit this theorized “dual action” profile to examine the impact of varenicline, both in the presence and absence of nicotine, on insula's resting-state functional connectivity (rsFC) with other brain regions. We hypothesized: 1) abstinence-induced alterations on insula-centered neural circuits would be ameliorated by both nicotine and varenicline; and 2) nicotine would have reduced impact in the presence of varenicline. Methods: Abstinent smokers (412 h; N=24) completed six fMRI assessments on separate days in a randomized, double-blinded, placebo-controlled, two-drug crossover study. At three different points during a varenicline administration regime (PILL factor: pre-pill, placebo, varenicline), participants underwent imaging on 2 occasions, once each with a transdermal nicotine or placebo patch (PATCH factor). Each participant underwent varenicline and placebo pill administration for B17 days and completed nicotine and placebo patch scans at the end of each medication period. fMRI data were collected during 8-min “resting” scans in which participants were instructed to simply relax with eyes-closed. For each smoker and session, rsFC was assessed by correlating each voxel's time course with a reference time course from a defined “seed” region. A left-mid insula seed (3024 mmł; Talairach: -40, 6, 4) was employed that had been indentified in a previous analysis exploring amygdala-centered rsFC. The impact of varenicline and nicotine on insula's rsFC with other regions was assessed in a whole-brain PILLPATCH interaction analysis (pcorrected o 0.05). Results: This PILLPATCH analysis identified several regions whose rsFC with insula was subject to pharmacological manipulations, including: ventromedial prefrontal cortex (vmPFC), dorsomedial PFC (dmPFC), posterior cingulate cortex (PCC), bilateral hippocampus, and left amygdala. Focusing on the vmPFC region of interest (ROI), rsFC with insula was greatest following abstinence and modulated by study medications in a manner consistent with varenicline's dual action profile. Specifically, nicotine (vs. placebo) decreased insula-vmPFC rsFC only in the absence of varenicline (pre-pill: p=0.003, placebo-pill: p<0.001; active-pill: p=0.27). No nicotine-induced reduction was observed under active-pill conditions as varenicline: 1) acting as a partial agonist in nicotine's absence, was associated with a modest rsFC decrease (placebopatch: p=0.024); and 2) acting as an antagonist in nicotine's presence, was associated with a rsFC increase (nicotine-patch: p<0.001). Similar interaction patterns were observed for all ROIs. Additionally, to investigate the relationship between nicotine withdrawal and insula's rsFC strength with other regions, an exploratory whole-brain correlation analysis was performed using self-reported withdrawal symptoms from each smoker and session to predict the corresponding session's connectivity values. Greater rsFC between insula and vmPFC, dmPFC, and PCC covaried with increased withdrawal in regions overlapping those showing druginduced effects. Discussion: Varenicline and nicotine decreased abstinence-induced alterations in rsFC between insula and constituent regions of the canonical DMN. These data support proposals derived from recent intrinsic network connectivity research that insula influences moment-to-moment information processing by toggling activity between large scale brain networks. During nicotine withdrawal, the insula, serving an interoceptive monitoring role, may bias processing towards the DMN network and away from the ECN, a shift that may underlie objectively assessed cognitive impairments during abstinence. |
| 1010 | Joseph, Anne M, Fu, Steven S, Lindgren, Bruce, Rothman, Alexander J, Kodl, Molly, Lando, Harry, Doyle, Brandon, Hatsukami, Dorothy. Chronic disease management for tobacco dependence: a randomized, controlled trial. Archives of internal medicine. 2011;171:1894.  **Abstract:** BACKGROUNDTobacco dependence disorder is a chronic relapsing condition, yet treatment is delivered in discrete episodes of care that yield disappointing long-term quit rates.METHODSWe conducted a randomized controlled trial from June 1, 2004, through May 31, 2009, to compare telephone-based chronic disease management (1 year; longitudinal care [LC]) with evidence-based treatment (8 weeks; usual care [UC]) for tobacco dependence. A total of 443 smokers each received 5 telephone counseling calls and nicotine replacement therapy by mail for 4 weeks. They were then randomized to UC (2 additional calls) or LC (continued counseling and nicotine replacement therapy for an additional 48 weeks). Longitudinal care targeted repeat quit attempts and interim smoking reduction for relapsers. The primary outcome was 6 months of prolonged abstinence measured at 18 months of follow-up.RESULTSAt 18 months, 30.2% of LC participants reported 6 months of abstinence from smoking, compared with 23.5% in UC (unadjusted, P = .13). Multivariate analysis showed that LC (adjusted odds ratio, 1.74; 95% CI, 1.08-2.80), quit attempts in past year (1.75; 1.06-2.89), baseline cigarettes per day (0.95; 0.92-0.99), and smoking in the 14- to 21-day interval post-quit (0.23; 0.14-0.38) predicted prolonged abstinence at 18 months. The LC participants who did not quit reduced smoking more than UC participants (significant only at 12 months). The LC participants received more counseling calls than UC participants (mean, 16.5 vs 5.8 calls; P < .001), longer total duration of counseling (283 vs 117 minutes; P < .001), and more nicotine replacement therapy (4.7 vs 2.4 boxes of patches; P < .001).CONCLUSIONA chronic disease management approach increases both short- and long-term abstinence from smoking.TRIAL REGISTRATIONclinicaltrials.gov Identifier: NCT00309296. |
| 1011 | Carpenter, Matthew J, Hughes, John R, Gray, Kevin M, Wahlquist, Amy E, Saladin, Michael E, Alberg, Anthony J. Nicotine therapy sampling to induce quit attempts among smokers unmotivated to quit: a randomized clinical trial. Archives of internal medicine. 2011;171:1901.  **Abstract:** BACKGROUNDRates of smoking cessation have not changed in a decade, accentuating the need for novel approaches to prompt quit attempts.METHODSWithin a nationwide randomized clinical trial (N = 849) to induce further quit attempts and cessation, smokers currently unmotivated to quit were randomized to a practice quit attempt (PQA) alone or to nicotine replacement therapy (hereafter referred to as nicotine therapy), sampling within the context of a PQA. Following a 6-week intervention period, participants were followed up for 6 months to assess outcomes. The PQA intervention was designed to increase motivation, confidence, and coping skills. The combination of a PQA plus nicotine therapy sampling added samples of nicotine lozenges to enhance attitudes toward pharmacotherapy and to promote the use of additional cessation resources. Primary outcomes included the incidence of any ever occurring self-defined quit attempt and 24-hour quit attempt. Secondary measures included 7-day point prevalence abstinence at any time during the study (ie, floating abstinence) and at the final follow-up assessment.RESULTSCompared with PQA intervention, nicotine therapy sampling was associated with a significantly higher incidence of any quit attempt (49% vs 40%; relative risk [RR], 1.2; 95% CI, 1.1-1.4) and any 24-hour quit attempt (43% vs 34%; 1.3; 1.1-1.5). Nicotine therapy sampling was marginally more likely to promote floating abstinence (19% vs 15%; RR, 1.3; 95% CI, 1.0-1.7); 6-month point prevalence abstinence rates were no different between groups (16% vs 14%; 1.2; 0.9-1.6).CONCLUSIONNicotine therapy sampling during a PQA represents a novel strategy to motivate smokers to make a quit attempt.TRIAL REGISTRATIONclinicaltrials.gov Identifier: NCT00706979. |
| 1017 | Grainge, Matthew, Coleman, Tim, Cooper, Sue, Thornton, Jim, Watts, Kim, Britton, John, Lewis, Sarah. Double-blind, placebo-controlled, randomised trial of nicotine replacement therapy patches in pregnancy. Journal of Perinatal Medicine. 2011; 39(S1):A172.  **Abstract:** Nicotine replacement therapy (NRT) is effective for smoking cessation outside pregnancy, but it is unclear whether NRT will be effective in pregnant women as they metabolise nicotine and cotinine faster. Despite this lack of evidence, health professionals in the United Kingdom (UK) routinely offer this treatment to pregnant smokers. This double-blind, placebo controlled randomised controlled trial investigated whether or not 15mg/16hr nicotine patches, used in addition to behavioural smoking cessation support, are effective and safe when used for smoking cessation in pregnancy. METHODOLOGY: We recruited women smoking >5 daily cigarettes from seven hospitals in the UK as they attended for antenatal ultrasound scans. Enrolled women all received behavioural cessation support and were randomised to eight weeks' treatment with active or placebo patches. Further behavioural support was provided by trial research midwives and local cessation services. The primary outcome was prolonged smoking cessation between a quit date set within 2 weeks of randomisation and delivery, validated by either or both of exhaled air carbon monoxide and saliva cotinine. Birth outcomes were monitored to investigate potential fetal harm. RESULTS AND DISCUSSION: 1050 women were recruited between 2007 and 2010, with 521 receiving NRT and 529 receiving placebo. Participants were recruited at a mean (SD) of 16.2 (3.5) weeks gestation and were highly nicotine-addicted, with 33.6% reporting smoking their first daily cigarette within 5 minutes of waking. There was no evidence that NRT had any impact on prolonged cessation; 49 (9.4%) in the NRT group were validated as not smoking compared with 40 (7.6%) who received placebo, unadjusted OR (95% CI) 1.26 (0.82-1.96). Birth outcomes were broadly similar in both groups. CONCLUSIONS: Adding a 15mg/16hr nicotine patch to behavioural cessation support provided to pregnant women is not effective for smoking cessation and has neither a beneficial nor negative impact on birth outcomes. |
| 1018 | Wardle, Margaret C, Munafò, Marcus R, de Wit, Harriet. Effect of social stress during acute nicotine abstinence. Psychopharmacology. 2011;218:39.  **Abstract:** RATIONALERelapse to smoking is often precipitated by stress, yet little is known about the effects of nicotine withdrawal on responses to acute stress, or whether nicotine replacement reverses withdrawal-induced changes in stress response.OBJECTIVESThe aim of the present study is to use an effective social stressor, the Trier Social Stress Test (TSST), to study subjective, cardiovascular and hormonal responses to stress during withdrawal, and examine whether nicotine replacement moderates responses to stress during withdrawal.METHODSForty-nine current regular smokers were randomly assigned to smoke as normal (SM), 12-h abstention with placebo patch (PL), or 12-h abstention with nicotine patch (NIC). They participated in a single session using the TSST, during which subjective affect, heart rate (HR), mean arterial blood pressure (MAP) and salivary cortisol were measured.RESULTSThe TSST produced expected increases in subjective negative affect, HR, MAP, and cortisol. Groups did not differ in subjective or cardiovascular responses, but the PL group exhibited larger stress-induced increase in cortisol than the other groups.CONCLUSIONSThe increased cortisol response might indicate a greater hormonal stress response during nicotine withdrawal. Alternatively, considering that cortisol also provides negative feedback to the stress system, and blunted cortisol responses are predictive of smoking relapse, the lower cortisol responses in the NIC and SM groups might indicate chronic dysregulation of the stress system. In this case, restoration of cortisol response by nicotine treatment to the lower levels seen during regular smoking may actually represent an undesired side effect of nicotine replacement. |
| 1030 | Reid, Malcolm S, Jiang, Huiping, Fallon, Bryan, Sonne, Susan, Rinaldi, Paul, Turrigiano, Eva, Arfken, Cynthia, Robinson, James, Rotrosen, John, Nunes, Edward V. Smoking cessation treatment among patients in community-based substance abuse rehabilitation programs: exploring predictors of outcome as clues toward treatment improvement. The American journal of drug and alcohol abuse. 2011;37:472.  **Abstract:** BACKGROUNDPredictors of smoking cessation (SC) treatment outcome were explored in a multisite clinical trial of SC treatment at community-based, outpatient, substance abuse rehabilitation programs affiliated with the National Drug Abuse Treatment Clinical Trials Network.OBJECTIVESTo explore baseline demographic and clinical predictors of abstinence during treatment.METHODSCigarette smokers from five methadone maintenance programs and two drug and alcohol dependence treatment programs were randomly assigned to SC treatment as an adjunct to substance abuse treatment as usual or to substance abuse treatment as usual. SC treatment consisted of group counseling (weeks 1-8) plus transdermal nicotine patch treatment (21 mg/day, weeks 1-6; 14 mg/day, weeks 7-8). Demographic and clinical predictors of smoking abstinence were evaluated among those patients assigned to the active SC condition (N = 153) using logistic regression.RESULTSAbstinence during treatment was positively associated with younger age, Hispanic or Caucasian (as opposed to African American) ethnicity/race, employment or student status, fewer cigarettes per day at baseline, lower severity of the primary substance problem at baseline, and higher methadone doses (among the subsample in methadone treatment).CONCLUSIONS AND SCIENTIFIC SIGNIFICANCEDuring future efforts to improve SC treatments among drug- and alcohol-dependent patients, consideration should be given to adequate treatment to reduce the severity of the primary drug or alcohol problem, tailoring treatments for patients with greater severity of smoking and of the primary substance problem, and culturally sensitive interventions. Analysis of predictors of outcome may be a useful tool for treatment development. |
| 1031 | Knott, Verner J, Millar, Anne M, McIntosh, Judy F, Shah, Dhrasti K, Fisher, Derek J, Blais, Crystal M, Ilivitsky, Vadim, Horn, Ed. Separate and combined effects of low dose ketamine and nicotine on behavioural and neural correlates of sustained attention. Biological psychology. 2011;88:83.  **Abstract:** Given the cognitive-promoting properties of the nicotinic acetylcholinergic receptor (nAChR) agonist, nicotine, the increased prevalence of smoke-inhaled nicotine in schizophrenia has been interpreted as an attempt to self-correct cognitive deficits, which have been particularly pronounced in the attentional domain. As glutamatergic abnormalities have been implicated in these attentional deficiencies, this study attempted to shed light on the separate and interactive roles of the N-methyl-d-aspartate receptor (NMDAR) and nAChR systems in the modulation of attention by investigating, in healthy volunteers, the separate and combined effects of nicotine and the NMDAR antagonist ketamine on neural and behavioural responses in a sustained attention task. In a randomized, double-blind, placebo controlled study, performance and the P300 event-related brain potential (ERP) in a visual information processing (RVIP) task were examined in 20 smokers and 20 non-smokers (both male and female). Assessment involved intravenous injection of a low subperceptual bolus dose (.04mg/kg) of ketamine or placebo, which was accompanied by acute treatment with nicotine (4mg) or placebo gum. Nicotine-enhanced attentional processing was most evident in nonsmokers, with both performance accuracy and P300 amplitude measures. Ketamine's detrimental effects on these behavioural and electrophysiologic measures were negatively moderated by acute nicotine, the synergistic effects being expressed differently in smokers and nonsmokers. These findings support the view that acute alterations and individual differences in nAChR function can moderate even subtle glutamatergic-driven cognitive deficiencies in schizophrenia and can be important therapeutic targets for treating cognitive impairments in schizophrenia. |
| 1035 | Mobascher, A., Warbrick, T., Brinkmeyer, J., Musso, F., Stoecker, T., Shah, N.J., Vossel, S., Winterer, G.. Nicotine effects on attention in schizophrenia: A simultaneous EEG-fMRI study. European Neuropsychopharmacology. 2011;21:S515.  **Abstract:** Background: Schizophrenia is associated with cognitive deficits and alterations in task-induced brain activity in several cognitive domains such as attention and working memory. Nicotine dependence is more frequent in schizophrenic patients than in the general population [1]. According to the “self-medication hypothesis” patients with schizophrenia consume nicotine to counteract schizophrenia-associated cognitive deficits. However there is sparse literature on the effects of nicotine on task-induced brain activation in patients suffering from schizophrenia. It was the purpose of the present study to investigate the acute effects of nicotine on the attention network as revealed by EEG (electroencephalography) P300-informed fMRI (functional magnetic resonance imaging) in schizophrenic smokers compared to healthy smoking control subjects. Methods: We performed a randomized, double-blind, placebo-controlled cross-over nicotine (1 mg nasal spray) challenge with simultaneous EEG-fMRI recording. We investigated 16 smoking patients suffering from schizophrenia and 19 smoking controls (Fagerström test for nicotine dependence/FTND score >4) during a visual choice reaction time task similar to an oddball task. EEG data were analyzed using the software BrainVision Analyzer 2 (Brain Products, Germany); fMRI data were analyzed using the software package FSL (FMRIB's Software Library, www.fmrib.ox.ac.uk/fsl). In addition to “conventional” fMRI analysis of task-related brain activity, single trial peak amplitudes of the EEG P300 event-related potential were included as regressors in the general linear model (GLM) analysis of the fMRI data [2]. Results: Only when applying the EEG P300-informed fMRI BOLD model in schizophrenic smokers we found higher cluster-corrected brain activation (Z>2.3; p < 0.05) in the nicotine condition compared to placebo (nicotine > placebo). Activated regions comprised a fronto-parietal network that has been implicated in attention such as prefrontal areas and the anterior cingulate cortex. In contrast no significant nicotine effects compared to placebo on the fMRI BOLD (blood oxygen-level dependent) signal were observed in schizophrenic smokers in the conventional fMRI analysis (no EEG considered). In smoking controls neither model (P300-informed or conventional) revealed significant nicotine effects. Conclusions: To our knowledge this is the first study addressing the effects of nicotine on task-induced brain activity in schizophrenic smokers that applied a simultaneous EEG-fMRI approach. Our data suggest that EEG-informed fMRI may be suited to study drug effects on cognitive processes. These effects may be subtle - like in the case of nicotine - and may not be captured when solely averaged task-induced fMRI BOLD activation without additional single trial information is considered. The sensitivity of EEG single trial amplitude-informed fMRI may be higher compared to conventional analyses of the fMRI BOLD signal. Moreover the EEG-informed analysis can emphasize certain aspects of task-induced brain activity such as neuronal sub-processes that are reflected by P300 amplitude. In view of the relationship between P300 amplitude, cognitive performance and schizophrenia our data suggest that positive effects of nicotine on cognition (as measured by a nicotine-induced increase in the P300 amplitude-informed fMRI BOLD signal) may be pronounced in patients with schizophrenia. This interpretation would be consistent with the self-medication hypothesis. |
| 1036 | Valencia, Borja, Ortega, Francisco, Vellisco, Arturo, Márquez-Martín, Eduardo, Campos, Jose Luis López, Rodríguez, Ana María, Ferrer, Marta, Cejudo, Pilar, Barrot, Emilia. Effectiveness of a cognitive orientation program with and without nicotine replacement therapy in stopping smoking in hospitalised patients. European Respiratory Journal. 2011; 38(S55):4224.  **Abstract:** Introduction: We analysed the effectiveness of a high intensity behavioural-cognitive intervention compared to minimal intervention started during a hospital stay, to see if the combination of nicotine replacement therapy (NRT) can increase the quitting rate at 12 months of follow up. Method: 2560 active smokers were studied during their hospital stay. Of these, 717 smokers refused to enter the study and after a minimal intervention they were asked if we could telephone them after one year to ask if they still smoked. The remaining 1843 smokers who received high intensity cognitive therapy were randomised to receive or not receive NRT. The follow up after discharge was carried out by outpatient visits or with telephone sessions. Results: At one year of follow up, 7% of those who declined to enter the study had stopped smoking compared to 27% of those who entered the study (p=0.001). There were significant differences between the group that only had behavioural therapy (21% stopped) compared to the group that also had NRT (33% stopped; p=0.002). In this latter group there were significant differences (p=0.03) between those who had follow up in clinics (39% stopped) compared to those who were followed up telephone sessions (30%). In the multivariate analysis, the predictors of quitting at 12 months were: to have used NRT (OR 12.2; 95% CI, 5.2-32; p=0.002) and a higher score in the Richmond Test (OR 10.1; 95% CI, 3.9-24.2; p=0.01). Conclusions: A cognitive type intervention started on smokers when admitted to hospital increases quitting rates at 12 months, compared to a minimal intervention, and these rates increase even more significantly if NRT is added. |
| 1038 | Williams, David M, Dunsiger, Shira, Whiteley, Jessica A, Ussher, Michael H, Ciccolo, Joseph T, Jennings, Ernestine G. Acute effects of moderate intensity aerobic exercise on affective withdrawal symptoms and cravings among women smokers. Addictive behaviors. 2011;36:894.  **Abstract:** A growing number of laboratory studies have shown that acute bouts of aerobic exercise favorably impact affect and cravings among smokers. However, randomized trials have generally shown exercise to have no favorable effect on smoking cessation or withdrawal symptoms during quit attempts. The purpose of the present study was to explore this apparent contradiction by assessing acute changes in affect and cravings immediately prior to and following each exercise and contact control session during an eight-week smoking cessation trial. Sixty previously low-active, healthy, female smokers were randomized to an eight-week program consisting of brief baseline smoking cessation counseling and the nicotine patch plus either three sessions/week of moderate intensity aerobic exercise or contact control. Findings revealed a favorable impact of exercise on acute changes in positive activated affect (i.e., energy), negative deactivated affect (i.e., tiredness), and cigarette cravings relative to contact control. However, effects dissipated from session to session. Results suggest that aerobic exercise has potential as a smoking cessation treatment, but that it must be engaged in frequently and consistently over time in order to derive benefits. Thus, it is not surprising that previous randomized controlled trials-in which adherence to exercise programs has generally been poor-have been unsuccessful in showing effects of aerobic exercise on smoking cessation outcomes. |
| 1039 | Fukada, Tomoko, Iwakiri, Hiroko, Ozaki, Makoto. A randomised double-blind crossover trial of the potential analgesic effect of a transdermal nicotine patch in non-smokers based on objective and subjective assessment. European journal of anaesthesiology. 2011;28:592.  **Abstract:** OBJECTIVEThe results of studies of the analgesic effect of nicotine in humans are complex because these studies have included smokers with variable smoking histories. We investigated whether the use of a 17.5 mg transdermal nicotine (TDN) patch decreased the magnitude of pressure pain on the hands of healthy non-smoking volunteers.DESIGNThis was a randomised double-blind crossover trial. A TDN patch or placebo (drug-free bandage) was applied randomly on the anterior chest of non-smoking volunteers 1 h before the experiments. We measured minimum perceived current and pain threshold on the right hand and then evaluated the magnitude of pressure pain by using the Pain Vision PS-2100 (Nipro Co., Osaka, Japan) which helps in objective quantitative assessment of pain magnitude. After estimating minimum perceived current, pressure pain was produced using a combination of 100-g discs and a rod. The rod and the discs weighing 0 (no disc), 200 (two discs), 400 (four discs), 200 (two discs) and 0 g (no disc) were placed consecutively in this order on the right hand and pain threshold was measured. At the same time, volunteers were asked to rate pain on a numerical rating scale (NRS). Minimum perceived current is the current at which the volunteer perceives the first sensation on applying gradually increasing pulsed current. Pain threshold is the compatible electrode current at which the volunteer feels the intensity of pressure pain. Pain degree is calculated as (pain threshold-minimum perceived current)/minimum perceived current × 100.PARTICIPANTSForty non-smoking volunteers were enrolled in this study.RESULTSNo significant differences between groups were observed in minimum perceived current, pain threshold, pain degree or NRS. Of the volunteers who received the nicotine patch, four became anorexic and nauseated and two required anti-emetics.CONCLUSIONThe nicotine patch had no analgesic effect in non-smoking volunteers. |
| 1050 | Bernstein, Steven L, Bijur, Polly, Cooperman, Nina, Jearld, Saba, Arnsten, Julia H, Moadel, Alyson, Gallagher, E John. A randomized trial of a multicomponent cessation strategy for emergency department smokers. Academic emergency medicine : official journal of the Society for Academic Emergency Medicine. 2011;18:575.  **Abstract:** OBJECTIVESThe objective was to determine the efficacy of an emergency department (ED)-based smoking cessation intervention.METHODSThis study was a randomized trial conducted from January 2006 to September 2007 at an urban ED that treats 90,000 adults per year. Discharged adults who smoked at least 10 cigarettes per day were randomized to 1) usual care, receiving a smoking cessation brochure; or 2) enhanced care, receiving the brochure, a motivational interview (MI), nicotine patches, and a phone call at 3 days. Interventions were performed by a peer educator trained in tobacco treatment. Blinded follow-up was performed at 3 months.RESULTSA total of 338 subjects were enrolled, mean (±SD) age was 40.2 (±12.0) years, 51.8% were female, and 56.5% were either self-pay or Medicaid. Demographic and clinical variables were comparable between groups. Enhanced and usual care arms showed similar cessation rates at 3 months (14.7% vs. 13.2%, respectively). The proportion of subjects making a quit attempt (69.2% vs. 66.5%) and decrease in daily cigarette use (five vs. one; all p > 0.05) were also similar. In logistic modeling, factors associated with quitting included any tobacco-related International Classification of Diseases, ninth revision (ICD-9), code for the ED visit (odds ratio [OR]= 3.42, 95% confidence interval [CI] = 1.61 to 7.26) or subject belief that the ED visit was tobacco-related (OR = 2.47, 95% CI = 1.17 to 5.21). Conversely, subjects who reported having a preexisting tobacco-related illness were less likely to quit (OR = 0.22, 95% CI = 0.10 to 0.50).CONCLUSIONSThe primary endpoint was negative, reflecting a higher-than-expected quit rate in the control group. Subjects whose ED visit was tobacco-related, based either on physician diagnosis or subject perception, were more than twice as likely to quit. These data suggest that even low-intensity screening and referral may prompt substantial numbers of ED smokers to quit or attempt to quit. |
| 1051 | Chan, Sophia S C, Leung, Doris Y P, Abdullah, Abu S M, Wong, Vivian T, Hedley, Anthony J, Lam, Tai-Hing. A randomized controlled trial of a smoking reduction plus nicotine replacement therapy intervention for smokers not willing to quit smoking. Addiction (Abingdon, England). 2011;106:1155.  **Abstract:** AIMSTo examine the effectiveness of smoking reduction counselling plus free nicotine replacement therapy (NRT) for smokers not willing to quit.DESIGN, SETTING AND PARTICIPANTSA total of 1154 Chinese adult smokers not willing to quit but who were interested in reducing smoking were allocated randomly to three arms. Intervention group A1 (n=479) received face-to-face counselling on smoking reduction and adherence to NRT at baseline, 1 week and 4 weeks with 4 weeks of free NRT. Group A2 (n=449) received the same intervention, but without the adherence intervention. Control group B (n=226) received simple cessation advice at baseline.MEASUREMENTSSelf-reported 7-day point prevalence of tobacco abstinence and reduction of cigarette consumption (≥50%) at 6 months and continuous use of NRT for 4 weeks at 3 months.FINDINGSUsing intention-to-treat analysis, compared to control group B, the intervention groups (A1+A2) had achieved higher 6-month tobacco abstinence (17.0% versus 10.2%, P=0.01) and reduction rates (50.9% versus 25.7%, P<0.001). There was no significant difference in the 4-week NRT adherence rate at 3 months, but group A1 achieved a higher abstinence rate than group A2 at 6 months (20.9% versus 12.9%; P=0.001).CONCLUSIONSIn smokers with no immediate plans to quit, smoking reduction programmes with behavioural support and nicotine replacement therapy are more effective than brief advice to quit. Current guidelines recommend advice to quit on medical grounds as the best clinical intervention in this group of smokers, but smoking reduction programmes offer an alternative and effective option. |
| 1052 | Walker, Natalie, Howe, Colin, Bullen, Chris, Grigg, Michele, Glover, Marewa, McRobbie, Hayden, Laugesen, Murray, Jiang, Joy, Chen, Mei-Hua, Whittaker, Robyn, Rodgers, Anthony. Does improved access and greater choice of nicotine replacement therapy affect smoking cessation success? Findings from a randomized controlled trial. Addiction (Abingdon, England). 2011;106:1176.  **Abstract:** AIMSTo determine the effect of offering smokers who want to quit easy access to nicotine replacement therapy (NRT), a period of familiarization and choice of product on smoking abstinence at 6 months.DESIGNSingle-blind, randomized controlled trial.SETTINGNew Zealand.PARTICIPANTSA total of 1410 adult smokers who called the national Quitline for quitting support were randomized to usual Quitline care or a box containing different NRT products (patch, gum, inhaler, sublingual tablet, oral pouch) to try for a week prior to quitting, and then to choose one or two of these products for 8 weeks' use.MEASUREMENTSThe primary outcome was 7-day point prevalence smoking abstinence 6 months after quit day. Secondary outcomes included continuous abstinence, cigarette consumption, withdrawal, NRT choice and serious adverse events at 1 and 3 weeks and 3 and 6 months.FINDINGSNo differences in 6-month quit rates (7-day point prevalence or continuous abstinence) were observed between the groups. However, smokers allocated to the intervention group were more likely to have quit smoking at 3 months [self-reported point prevalence, relative risk (RR)=1.17, 95% confidence interval (CI): 1.02, 1.35, P=0.03], had a longer time to relapse (median 70 days versus 28 days, P<0.01) and used significantly more NRT. The selection box concept was highly acceptable to users, with the patch and inhaler combination the most popular choice (34%).CONCLUSIONSIn terms of smoking abstinence at 6 months, offering smokers who want to quit free access to a wide range of nicotine replacement therapy, including a 1-week period of familiarization and choice of up to two products, appears no different to offering reduced cost and choice of nicotine replacement therapy, with no familiarization period. This trial is registered with the Australasian Clinical Trials Network Number: ACTRN 12606000451505. |
| 1056 | Richardson, Elizabeth J., Richards, J. Scott, Stewart, Christopher C., Ness, Timothy J.. Effects of nicotine on spinal cord injury related pain: A randomized, double-blind, placebo-controlled crossover trial. Topics in Spinal Cord Injury Rehabilitation. 2011;16:31.  **Abstract:** Objective: Previous case reports and neurobiological studies implicate the potential effect of nicotine on spinal cord injury (SCI)-related pain. The present study is an extension of a previous pilot trial among persons with SCI in order to clarify the nicotine-pain relationship. Design: Randomized, double-blind placebo-controlled crossover trial using nicotine and placebo gum. Participants/methods: A total of 103pain sites from 42 smokers and nonsmokers with SCI were evaluated across trials. There were 101 pain sites included in final analysis and were classified as musculoskeletal (n = 42), neuropathic (n = 39), and mixed (n = 20). Pain ratings were measured via a numeric rating scale at 30 and 90 minutes during each trial. Results: In the placebo arm, main effects for smoking and pain type emerged but no interaction for placebo dose. In the nicotine arm, there was a significant pain type by smoking interaction. On average, smokers appeared to experience an increase in mixed and neuropathic pain but no change in musculoskeletal pain, while nonsmokers experienced a decrease in mixed pain only with increasing nicotine dosage. Conclusions: Should this study be replicated, it implies potentially different treatment strategies for smokers and nonsmokers who experience SCI-related mixed and neuropathic forms of pain. |
| 1058 | Rigotti, Nancy A., Bitton, Asaf, Kelley, Jennifer K., Hoeppner, Bettina B., Levy, Douglas E., Mort, Elizabeth. Supplementing office-based care with a populationbased direct-to-smoker outreach intervention offering free treatment to smokers in a community health center: A randomized controlled trial. Journal of General Internal Medicine. 2011;26:S33.  **Abstract:** BACKGROUND: Tobacco remains the leading U.S. preventable cause of death. Treating tobacco use is among the most cost-effective actions in health care. Brief office-based interventions offered to all smokers are effective but clinicians do not reliably offer them. We hypothesized that tobacco treatment use and quit rates could be increased by offering treatment directly to all smokers, apart from office visits. We tested the effectiveness of a population-based direct-to-smoker (DTS) outreach programoffering free tobacco treatment to smokers in ahealthcare system. METHODS: A randomized controlled trial of 590 smokers at 1 community health center compared usual care (n=177) to usual care plus DTS outreach (n=413). The DTS group was sent 3 monthly letters offering free phone consultation with a tobacco coordinator who offered fax-referral to the state telephone quitline and up to 8 weeks of free nicotine patches (NRT). Outcomes, assessed at 3 month follow-up, were the percent of smokers who used any tobacco treatment (counseling or meds), used NRT, used counseling, and self-reported 7-day and 30-day point prevalence tobacco abstinence. RESULTS: 43 (10.4%) of 413 smokers in the DTS group accepted the treatment offer; 42 (98%) requested NRT and 30 (70%) were referred to counseling. At 3-month follow-up, in an intention-to-treat analysis adjusted by logistic regression for age, sex, race, insurance, and history of diabetes and/or coronary heart disease, a higher proportion of the DTS group, compared to controls, had used NRT (11.6% vs 3.9%, OR 3.47; 95%CI 1.52-7.92, p=.003), used any tobacco treatment (14.5% vs 7.3%, OR 1.95, 95%CI 1.04-3.65, p=.036), and reported tobacco abstinence for the past 7 days (5.3% vs. 1.1%, OR 5.35, 95%CI 1.23- 22.32, p=.026) and past 30 days (4.1% vs. 0.6%, OR 8.25, 95%CI 1.08- 63.01, p=.042). The treatment offer did not increase use of counseling (1.1% vs 1.7%, p=NS) or non-NRT medication use (3.6% vs 3.9%, p=NS). Estimated marginal cost per 7-day quit at 3 months was 6. CONCLUSION: In a real-world effectiveness study, a population-based direct-to-smoker outreach offering free tobacco treatment to smokers in a community health center is a feasible, cost-effective way to increase the reach of treatment (primarily pharmacotherapy) and to increase short-term quit rates in the population. |
| 1061 | Yagoubian, Benjamin, Akkara, Joseph, Afzali, Payam, Alfi, David M, Olson, Luke, Conell-Price, Jessamyn, Yeh, Janet, Eisig, Sidney B, Flood, Pamela. Nicotine nasal spray as an adjuvant analgesic for third molar surgery. Journal of oral and maxillofacial surgery : official journal of the American Association of Oral and Maxillofacial Surgeons. 2011;69:1316.  **Abstract:** PURPOSETo determine the efficacy of preoperatively administered nicotine nasal spray (3 mg) for analgesia after third molar (TM) surgery.MATERIALS AND METHODSA single-center, prospective, randomized, double-blind, crossover trial was conducted. The study population consisted of 20 nonsmoking patients referred to the Department of Oral and Maxillofacial Surgery of Columbia University College of Dental Medicine for extraction of all 4 TMs. Each patient received nicotine nasal spray or placebo spray before TM surgery. At a subsequent visit the contralateral TMs were removed with prior administration of the alternate treatment. For an hour postoperatively, subjects reported information on pain and nausea, and hemodynamic variables were recorded at 15-minute intervals. Telephone follow-up was recorded for 5 days postoperatively, where patients reported information on pain, nausea, and use of hydrocodone/acetaminophen as rescue analgesia.RESULTSNicotine treatment was associated with a highly significant decrease in pain reported during the 5 days after TM surgery. There was no difference in the amount of hydrocodone/acetaminophen used or amount of nausea reported. There was a small but significant increase in heart rate after nicotine treatment compared with placebo during the first hour after surgery. There was no difference in blood pressure between groups.CONCLUSIONPain is well controlled by hydrocodone/acetaminophen in most patients after TM surgery. However, there is significant variability in pain reported. Nicotinic agonists represent a new class of analgesic that can be considered for patients who are expected to have significant opioid-resistant pain after TM surgery. Caution should be used with patients in whom a small increase in heart rate would be deleterious. |
| 1065 | Stapleton, John A, Sutherland, Gay. Treating heavy smokers in primary care with the nicotine nasal spray: randomized placebo-controlled trial. Addiction (Abingdon, England). 2011;106:824.  **Abstract:** AIMSOf six established nicotine replacement therapy (NRT) formulations, only the gum and patch have been tested without specialist clinic support in placebo-controlled trials. We aimed to broaden the evidence base by examining if the nicotine nasal spray (NNS) could be effective with only brief support in general practice.DESIGNRandomized placebo-controlled trial.SETTINGTwenty-seven English general practices.PARTICIPANTSA total of 761 heavy smokers received brief support and 12 weeks of treatment with NNS (506) or placebo (255).MEASUREMENTSThe primary outcome was biochemically verified complete abstinence from smoking throughout weeks 3-12.FINDINGSNNS compared with placebo more than doubled the number who successfully stopped smoking [15.4% versus 6.7%, odds ratio (OR) = 2.6, 95% confidence interval (CI) = 1.5-4.4]. Many participants reported minor irritant adverse symptoms. NNS was particularly effective among those who were more highly dependent on nicotine (OR = 6.17, 95% CI = 2.13-17.9). Of those who failed to stop during the first week (417, 54.8%), only one (0.2%) achieved later success.CONCLUSIONSNNS is effective when given in primary care. The benefit was lower than in a specialist clinic but similar to that with the nicotine patch in primary care. Unlike most other NRT formulations, bupropion or varenicline, NNS was especially helpful for more dependent smokers. Continuing treatment of those initially failing was not beneficial. An initial 1-week prescription to those more dependent on nicotine is likely to be the most cost-effective NNS treatment protocol. These results should offer support to the effectiveness of the other NRT formulations untested in this setting. |
| 1067 | Allen, Michael H, Debanné, Martin, Lazignac, Coralie, Adam, Eric, Dickinson, L Miriam, Damsa, Cristian. Effect of nicotine replacement therapy on agitation in smokers with schizophrenia: a double-blind, randomized, placebo-controlled study. The American journal of psychiatry. 2011;168:395.  **Abstract:** OBJECTIVEThe authors conducted a randomized, placebo-controlled study of nicotine replacement therapy for the reduction of agitation and aggression in smokers with schizophrenia.METHODParticipants were 40 smokers 18-65 years of age admitted to a psychiatric emergency service with a diagnosis of schizophrenia confirmed by the Mini International Neuropsychiatric Interview. Patients were screened for agitation with the excited component subscale of the Positive and Negative Syndrome Scale (PANSS) and for nicotine dependence with the Fagerström Test for Nicotine Dependence. A score of at least 14 on the PANSS excited component subscale and at least 6 on the Fagerström test were required for study eligibility. Participants in the nicotine replacement group received a 21-mg nicotine transdermal patch, and those in the placebo group were treated with a placebo patch. Participants received usual care with antipsychotics. The Agitated Behavior Scale and other agitation measures were administered at baseline and again at 4 and 24 hours.RESULTSAt baseline, participants were at least moderately agitated, and 28% reported aggressive behavior in the previous week. The mean Agitated Behavior Scale score for the nicotine replacement group was 33% lower at 4 hours and 23% lower at 24 hours than for the placebo group. Participants with lower levels of nicotine dependence responded better than those with higher levels of dependence.CONCLUSIONSThe drug-placebo difference in this study was similar to that obtained in trials of parenteral antipsychotics in similar populations. This finding suggests that in patients with schizophrenia, smoking status should be included in the assessment of agitation and nicotine replacement included in the treatment of those who are smokers. |
| 1070 | Hilberink, Sander R, Jacobs, Johanna E, Breteler, Marinus H M, de Vries, Hein, Grol, Richard P T M. General practice counseling for patients with chronic obstructive pulmonary disease to quit smoking: impact after 1 year of two complex interventions. Patient education and counseling. 2011;83:120.  **Abstract:** OBJECTIVETo evaluate two counseling programs in general practice to help smokers with chronic obstructive pulmonary disease (COPD) to quit smoking.METHODSCluster randomized controlled trial including 68 general practices (667 patients) using a randomly assigned intervention program with counseling and advice about nicotine replacement therapy (and additional bupropion-SR in one of the programs) or usual care. Usual care consisted of periodic regular check-ups and COPD information. The main outcome measure was biochemically verified point prevalence at 12 months.RESULTSThe two intervention groups were treated as one in the analysis because they were equally effective. The intervention resulted in a significantly self-reported higher success rate (14.5%) compared to usual care (7.4%); odds ratio=2.1, 95% confidence interval=1.1-4.1. Biochemically verified quit rates were 7.5% (intervention) and 3.4% (usual care); odds ratio=2.3, 95% confidence interval=0.9-6.0.CONCLUSIONThe program doubled the cessation rates (statistically nonsignificant). Too few participants used the additional bupropion-SR to prove its effectiveness.PRACTICE IMPLICATIONSThe protocols can be used for COPD patients in general practice, but expectations should be modest. If quitting is unsuccessful, a stepped care approach should be considered. |
| 1072 | Ferguson, Stuart G, Shiffman, Saul, Rohay, Jeffrey M, Gitchell, Joseph G, Garvey, Arthur J. Effect of compliance with nicotine gum dosing on weight gained during a quit attempt. Addiction (Abingdon, England). 2011;106:651.  **Abstract:** AIMUsing nicotine gum can reduce the amount of weight gained when quitting. Here we examine the relationship between weight gain and use of adequate amounts of gum. To mitigate the confounders associated with correlational analyses, we contrast the effects of active gum and placebo, and analyze outcomes prospectively.DESIGN AND SETTINGRandomized double-blind placebo-controlled trial of nicotine gum. Participants were instructed to use nine to 15 pieces of gum/day for the first 2 months of treatment.PARTICIPANTSParticipants (n = 103) were randomized to either active (2 mg or 4 mg) or placebo gum.MEASUREMENTSWe examined the effect on weight gain of the interaction between treatment (active versus placebo) and daily gum use [≥9 pieces/day (compliant use) versus < 9 pieces/day].FINDINGSAfter 30 days of abstinence, smokers treated with active gum had not gained significantly less weight than those on placebo (1.1 kg versus 1.6 kg, P = 0.175). However, a significant compliance-treatment interaction was observed (P = 0.005): active gum users who used ≥9 pieces/day during the first 14 days of treatment had gained less weight at follow-up (0.6 kg versus 1.6 kg for those who used <9 pieces/day, P = 0.017), but participants randomized to the placebo group saw no such benefit from compliant use. A similar compliance-treatment interaction (P = 0.046) was also observed when the effect of compliance was examined within active treatment (2 mg versus 4 mg).CONCLUSIONSWhen smokers are quitting, those who use more pieces of nicotine gum experience less weight gain in the first 30 days. This relationship is not seen for smokers on placebo gum. |
| 1073 | Czarnetzki, Christoph, Schiffer, Eduardo, Lysakowski, Christopher, Haller, Guy, Bertrand, Daniel, Tramèr, Martin R. Transcutaneous nicotine does not prevent postoperative nausea and vomiting: a randomized controlled trial. British journal of clinical pharmacology. 2011;71:383.  **Abstract:** AIMSThere is empirical evidence that smokers are less likely to suffer from postoperative nausea and vomiting (PONV). We sought to investigate whether transcutaneus nicotine prevents PONV.METHODSNon-smokers receiving general anaesthesia for surgery were randomly allocated to Nicotinell Patch 10cm(2) (TTS 10), containing 17.5mg of nicotine (average delivery rate, 7mg 24h(-1) ) or matching placebo patch. Patches were applied 1h before surgery and were left in situ until 24h after surgery (or until the first PONV symptoms occurred).RESULTSWe randomized 90 patients (45 nicotine, 45 placebo). In the post-anaesthetic care unit, the incidence of nausea was 22.2% with nicotine and 24.4% with placebo (P= 0.80), and the incidence of vomiting was 20.0% with nicotine and 17.8% with placebo (P= 0.78). Cumulative 24h incidence of nausea was 42.2% with nicotine and 40.0% with placebo (P= 0.83), and of vomiting was 31.1% with nicotine and 28.9% with placebo (P= 0.81). PONV episodes tended to occur earlier in the nicotine group. Postoperative headache occurred in 17.8% of patients treated with nicotine and in 15.6% with placebo (P= 0.49). More patients receiving nicotine reported a low quality of sleep during the first postoperative night (26.7% vs. 6.8% with placebo; P= 0.01).CONCLUSIONSNon-smokers receiving a prophylactic nicotine patch had a similar incidence of PONV during the first 24h and tended to develop PONV symptoms earlier compared with controls. They had a significantly increased risk of insomnia during the first postoperative night. |
| 1074 | Koychev, I., McMullen, K., Lees, J., Grayson, L., Schmechtig, A., Dourish, C., Wilkinson, L., Williams, S., Deakin, J.F.W., Barkus, E.. A validation of cognitive biomarkers for the early identification of cognitive enhancing agents in schizotypy. European Neuropsychopharmacology. 2011;21:S86.  **Abstract:** Introduction: The cognitive impairment associated with schizophrenia is a major target for drug development but none of the drugs designed to address this problem have shown consistent efficacy. The most common causes for failure in the registration trials could be preempted by testing the novel agents in milder forms of the disorder, such as schizotypy and assessing the effect using validated biomarkers [1]. In this study we aimed to test this approach by comparing the acute effects of risperidone, amisulpride and nicotine in a double-blind placebo-controlled three-centre study on the cognitive performance in schizotypy. We hypothesized that the neuropsychological abnormalities associated with schizotypy will be reversed by the acute antipsychotic and nicotine challenges. Methods: We recruited healthy volunteers that scored high (HS group) or average (AS group) on the Schizotypal Personality Questionnaire (SPQ) (122 in each group). The participants were randomized to an acute dose of risperidone (2 mg), amisulpride (400 mg), nicotine patch (7 mg) or placebo and proceeded to complete cognitive tests, including the currently reported N-back, Verbal Fluency (VF) and Spatial Working Memory (SWM) tasks. Results: We found evidence for overall worse performance in the placebo-treated HS group relative to AS on the N-back and VF tasks (F(1,46) = 6.628, p = 0.013 and F(1,46) =6.063, p = 0.018 respectively). Amisulpride exerted differential effect on the two schizotypy groups in respect to their cognitive performance: in the N-back it impaired the AS group but improved performance in HS. Similarly, in the VF task it reduced the word production in the AS group but did not affect tHS (F(6,376) = 2.023, p = 0.062). A similar trend was found for the SWM but it did not reach significance. In contrast, risperidone impaired both groups (comparisons vs. placebo: p = 0.06 in the N-back and p = 0.01 in the VF), while nicotine had beneficial effect for the low baseline performers (comparison vs. placebo in the N-back: p = 0.024). The latencies of the reaction times in the N-back task were significantly increased in the risperidone arm of the study in both schizotypy groups (F(3,189) =7.804, p < 0.001). Conclusions: We attribute the effects of amisulpride to its presynaptic dopamine enhancing action at low doses [2]. This is based on reports that increase in dopamine neurotransmission is beneficial for people and primates with low baseline cognitive performance but impairs those with optimal performance. The deterioration of performance with risperidone on the other hand was probably due to sedation (increased reaction times on the N-back task) while nicotine showed signs of general vigilance improvement. In summary, the reported findings confirm that cognitive abnormalities in schizotypy are replicable in multi-site studies and responsive to drug challenges. Importantly, these results underline the importance of dopamine agonism in the pathophysiology of the cognitive deficits in the schizophrenia spectrum disorders. |
| 1075 | Moran, Lauren V., Sampath, Hemalatha, Yang, Yihong, Ross, Thomas J., Jo Salmeron, Betty, Buchholz, Brittany, Thaker, Gunvant K., Stein, Elliot A., Hong, Elliot. Insula, dorsal anterior cingulate and ventral striatum resting state functional connectivity network in nicotine administration, nicotine addiction and schizophrenia. Schizophrenia Bulletin. 2011;37:147.  **Abstract:** Background: People with schizophrenia (SZ) have unusually high rates of smoking. Damage to the insula alters smoking behavior. Decreased resting state functional connectivity (rsFC) in a dorsal anterior cingulate (dACC) to ventral striatum (VS) circuit is associated with nicotine addiction. We hypothesized that insula and dACC circuits may jointly contribute to nic-otine addiction in normal control smokers (NC) and smokers with SZ. Methods: Using a double-blind, placebo-control, cross-over design, 19 smokers with SZ and 19 NC underwent resting state fMRI during randomized placebo vs. nicotine patch conditions. We manually partitioned left and right insula (lIns, rIns) and dACC (ldACC, rdACC) as region of interest (ROI) “seeds” and generated whole-brain rsFC with each ROI. We studied the contribution of each ROI's rsFC circuit to nicotine addiction severity as measured by the Fagerstrom Test for Nicotine Dependence (FTND). Findings are based on pcorrected <.05 [puncorrected <.001; cluster >324 mm3] for each seed-based rsFC whole brain analysis. Results: FTND scores did not differ between SZ and NC subjects (P = .62). Regression analysis identified 7 rsFC circuits that were significantly associated with FTND after Bonferroni correction (P <.004; see table). Mixed effect ANOVA revealed that more severe addiction was associated with reduced rsFC in all 7 circuits. We found no significant drug effect (nicotine vs. placebo). Furthermore, ldACC to right and left VS and rIns to left somatosensory cortex showed significantly reduced rsFC in SZ compared with NC. Stepwise regression revealed that 2 of the 7 circuits (ldACC-right VS and rIns-bilateral dACC) combined explained 43% of the variance in FTND (P <.001). Conclusion: Reduced rsFC in an insula-dACC-VS network model explains a substantial portion of the variance of nicotine addiction. Decreased rsFC in ldACC-VS and rIns-somatosensory cortex circuits may index an overlapping circuitry for smoking and SZ. |
| 1076 | Hong, L Elliot, Schroeder, Matthew, Ross, Thomas J, Buchholz, Brittany, Salmeron, Betty Jo, Wonodi, Ikwunga, Thaker, Gunvant K, Stein, Elliot A. Nicotine enhances but does not normalize visual sustained attention and the associated brain network in schizophrenia. Schizophrenia bulletin. 2011;37:416.  **Abstract:** Sustained attention abnormality in schizophrenia is usually refractory to available treatment. Nicotine can transiently improve sustained attention in schizophrenia patients, although its neural mechanisms are unknown. Understanding the neural basis of this effect may lead to new treatment strategies for this cognitive deficit. Twenty schizophrenia patients and 24 healthy comparison smokers participated in a double-blind, placebo-controlled, crossover, randomized functional magnetic resonance imaging study comparing nicotine vs placebo patch on sustained attention, using the rapid visual information-processing task. Schizophrenia patients had impaired visual sustained attention accuracy and processing speed (all P's <.001) and showed significantly reduced activation in the frontal-parietal-cingulate-thalamic attention network compared with healthy comparison subjects. Nicotine administration enhanced accuracy and processing speed compared with placebo (all P's ≤.006), with no drug × diagnosis interactions. However, schizophrenia patients' task performance remained impaired during the nicotine condition, even when compared with healthy comparison subjects in the placebo condition (all P's ≤.01). Nicotine exerted no significant reversal of the impaired attention network associated with schizophrenia. Activations in brain regions associated with nicotine-induced behavioral improvement were not significantly different between patients and comparison subjects. Thus, nicotine transiently enhanced sustained attention similarly in schizophrenia patients and in healthy comparison smokers. The neural mechanisms for this nicotinic effect in schizophrenia appear similar to those for healthy comparison subjects. However, nicotine, at least in a single sustained dose, does not normalize impaired sustained attention and its associated brain network in schizophrenia. These findings provide guidance for developing new treatment strategies for the sustained attention deficit in schizophrenia. |
| 1081 | Piper, Megan E, Cook, Jessica W, Schlam, Tanya R, Jorenby, Douglas E, Baker, Timothy B. Anxiety diagnoses in smokers seeking cessation treatment: relations with tobacco dependence, withdrawal, outcome and response to treatment. Addiction (Abingdon, England). 2011;106:418.  **Abstract:** AIMSTo understand the relations among anxiety disorders and tobacco dependence, withdrawal symptoms, response to smoking cessation pharmacotherapy and ability to quit smoking.DESIGNRandomized placebo-controlled clinical trial. Participants received six 10-minute individual counseling sessions and either: placebo, bupropion SR, nicotine patch, nicotine lozenge, bupropion SR + nicotine lozenge or nicotine patch + nicotine lozenge.SETTINGTwo urban research sites.PARTICIPANTSData were collected from 1504 daily smokers (>9 cigarettes per day) who were motivated to quit smoking and did not report current diagnoses of schizophrenia or psychosis or bupropion use.MEASUREMENTSParticipants completed baseline assessments, the Composite International Diagnostic Interview and ecological momentary assessments for 2 weeks.FINDINGSA structured clinical interview identified participants who ever met criteria for a panic attack (n = 455), social anxiety (n = 199) or generalized anxiety disorder (n = 99), and those who qualified for no anxiety diagnosis (n = 891). Smokers with anxiety disorders reported higher levels of nicotine dependence and pre-quit withdrawal symptoms. Those ever meeting criteria for panic attacks or social anxiety disorder showed greater quit-day negative affect. Smokers ever meeting criteria for anxiety disorders were less likely to be abstinent at 8 weeks and 6 months post-quit and showed no benefit from single-agent or combination-agent pharmacotherapies.CONCLUSIONSAnxiety diagnoses were common among treatment-seeking smokers and were related to increased motivation to smoke, elevated withdrawal, lack of response to pharmacotherapy and impaired ability to quit smoking. These findings could guide treatment assignment algorithms and treatment development for smokers with anxiety diagnoses. |
| 1083 | Knott, Verner, Heenan, Adam, Shah, Dhrasti, Bolton, Kiley, Fisher, Derek, Villeneuve, Crystal. Electrophysiological evidence of nicotine's distracter-filtering properties in non-smokers. Journal of psychopharmacology (Oxford, England). 2011;25:239.  **Abstract:** Nicotine-enhanced attentional functions are purported to underlie improvements in behavioral performance in cognitive tasks but it is unclear as to whether these effects involve selective attention or attentional control under conditions of distraction. Behavioral and event-related potential measures were used to examine the effects of nicotine on distractibility in 21 non-smokers who were instructed to ignore task-irrelevant auditory stimuli while they performed a visual discrimination task. In a randomized, double-blind, placebo-controlled cross-over design, nicotine gum (6 mg) shortened overall reaction times but failed to prevent increased response slowing and errors caused by deviant sounds. Relative to placebo, nicotine did not modulate the early pre-attentive detection of deviants as reflected in the mismatch negativity but it attenuated the amplitude of the deviant-elicited P3a, an event-related potential component indexing the involuntary shifting of attention. Nicotine also enhanced attentional re-focusing back on to task-relevant stimuli following distraction as evidenced by an increased amplitude of the re-orienting negativity. These findings and the behavioral-electrophysiological dissociation seen with nicotine are discussed in relation to theories of attention and smoking motivation. |
| 1088 | Ortega, Francisco, Vellisco, Arturo, Márquez, Eduardo, López-Campos, José Luis, Rodríguez, Ana, de los Ángeles Sánchez, María, Barrot, Emilia, Cejudo, Pilar. Effectiveness of a cognitive orientation program with and without nicotine replacement therapy in stopping smoking in hospitalised patients. Archivos de bronconeumologia. 2011;47:3.  **Abstract:** INTRODUCTIONWe analysed the effectiveness of a high intensity behavioural-cognitive intervention compared to minimal intervention started during a hospital stay, to see if the combination of nicotine replacement therapy (NRT) can increase the quitting rate at 12 months of follow up.METHODA total of 2560 active smokers were studied during their hospital stay. Of these, 717 smokers refused to enter the study and after a minimal intervention they were asked if we could telephone them after one year to ask if they still smoked. The remaining 1843 smokers who received high intensity cognitive therapy were randomised to receive or not receive NRT. The follow up after discharge was carried out by outpatient visits or with telephone sessions.RESULTSAt one year of follow up, 7% of those who declined to enter the study had stopped smoking compared to 27% of those who entered the study (p<0.001). There were significant differences between the group that only had behavioural therapy (21% stopped) compared to the group that also had NRT (33% stopped; p=0.002). In this latter group there were significant differences (p=0.03) between those who had follow up in clinics (39% stopped) compared to those who were followed up telephone sessions (30%). In the multivariate analysis, the predictors of quitting at 12 months were: to have used NRT (OR 12.2; 95% CI, 5.2-32; p=0.002) and a higher score in the Richmond Test (OR 10.1; 95% CI, 3.9-24.2; p=0.01).CONCLUSIONSA cognitive type intervention started on smokers when admitted to hospital increases quitting rates at 12 months, compared to a minimal intervention, and these rates increase even more significantly if NRT is added. |
| 1089 | Barrett, Sean P, Campbell, Mallory L, Temporale, Kirsten, Good, Kim Berley P. The acute effect of Swedish-style snus on cigarette craving and self-administration in male and female smokers. Human psychopharmacology. 2011;26:58.  **Abstract:** INTRODUCTIONSwedish-style snus (SS) has recently garnered controversy for its proposed use as a smoking cessation aid and/or harm reduction tool. However, to date, little work has been done to evaluate the extent to which SS affects cigarette cravings and smoking behavior under double-blind controlled conditions.METHODSDuring four double-blind placebo-controlled randomized sessions, 15 smokers (8 male) administered SS, placebo (nicotine/tobacco-free) snus (PS), a nicotine-containing lozenge (NL), or a placebo lozenge (PL) for 30 min and assessed their effects using Visual Analogue Scales and the Brief Questionnaire of Smoking Urges. They could then self-administer their usual brand of cigarettes using a progressive ratio task over the next 60 min. Following the completion of their final session, 11 participants (6 male) ranked each of the products used in the study in terms of their preferences.RESULTSRelative to the other products, SS was associated with a decreased intention to smoke as well as a delayed onset of cigarette smoking in men but not women. However, SS administration was also associated with increased feelings of frustration and irritability relative to NL and SS was ranked as being the least preferred product used in the study.DISCUSSIONFindings suggest that SS is effective in acutely suppressing craving and smoking in at least some smokers, but that its acceptability may be limited. |
| 1095 | Arias, Albert J., Feinn, Richard, Kranzler, Henry R., Oncken, Cheryl. Allelic association of the GluR5 kainate receptor subunit gene GRIK1 with adverse effects of topiramate. Neuropsychopharmacology. 2010;35:S114.  **Abstract:** Background: Topiramate is efficacious in the treatment of alcohol dependence, with evidence that it may also be useful in the treatment of nicotine and cocaine dependence, but adverse effects substantially limit its use. Based on an allelic association of GRIK1 with alcohol dependence (Kranzler et al. 2007), Ray et al., (2009) found a (nominally) significant association between a single nucleotide polymorphism (SNP) rs28322407 in GRIK1 and severity of adverse effects in a study of non-treatment seeking alcoholics. We sought to replicate that finding in a sample of individuals treated with topiramate for smoking cessation. Methods: Pharmacogenetic analysis was performed on N=57 subjects participating a 10-week smoking cessation trial. Subjects were randomized to one of three treatment groups: placebo, topiramate, or topiramate plus nictotine patch. The dosage of topiramate was titrated over 4 weeks to a maximum of 200 mg/day, and the topiramate plus patch group received a standard nicotine patch taper starting with 21 mg/day on the quit date (after 2 weeks on topiramate). Adverse effects were assessed at each visit via a standard self-report questionnaire. Subjects were genotyped for rs28322407 and rs2186305, which was also previously associated to alcohol dependence (Kranzler et al. 2007). Results: The topiramate/nicotine patch group reported significantly more adverse effects than placebo (F=5.27, p=.006). However, there was no evidence of association to adverse effects. Rs28322407 showed no main effect of genotype (F=.14, p=.240) and no interaction between topiramate and genotype ((F=.012, df=1, p=.90). Rs186305 (F=6.64, df=1, p=.013) showed a main effect, but no evidence of a genotype by topiramate interaction (F=.46, df=1, p=.50). Discussion: We found no significant association to the severity of adverse effects for the GRIK1 SNPs examined in this trial of topiramate for smoking cessation. Pharmacogenetic analysis of topiramate adverse effects in larger samples is needed to evaluate the validity of the prior report by Ray et al. (2009) since such effects could help to optimize and personalize topiramate treatment for addictive disorders. |
| 1102 | Darredeau, Christine, Barrett, Sean P. The role of nicotine content information in smokers' subjective responses to nicotine and placebo inhalers. Human psychopharmacology. 2010;25:577.  **Abstract:** OBJECTIVEA growing body of evidence suggests that non-pharmacological factors may play an important role in smoking cessation outcomes using nicotine replacement therapies. This study examined the role of information about nicotine content in smokers' subjective responses to nicotine and placebo inhalers, using the four conditions of the balanced-placebo design in a mixed within/between-subjects design.METHODSTwenty-four adult smokers (12 male) completed two laboratory sessions following overnight abstinence from smoking. Participants were randomly assigned to receive either nicotine inhalers or placebo inhalers in both sessions but were told that they received a nicotine-containing inhaler in one session and a nicotine-free inhaler in the other. In each session participants completed subjective assessments before and after inhaler administration using visual analogue scales and the Brief Questionnaire of Smoking Urges.RESULTSWhile neither nicotine content nor information about it significantly affected cigarette craving associated with withdrawal relief, participants reported a greater reduction in craving associated with intention to smoke when told the inhalers contained nicotine than when told the inhalers were nicotine-free, regardless of actual nicotine content.CONCLUSIONSFindings suggest that psychological factors play an important role in smokers' subjective responses to nicotine inhalers, the effects of which cannot be solely attributed to the direct pharmacological effects of nicotine. |
| 1104 | Thomsen, Thordis, Tønnesen, Hanne, Okholm, Mette, Kroman, Niels, Maibom, Annerikke, Sauerberg, Marie-Louise, Møller, Ann M. Brief smoking cessation intervention in relation to breast cancer surgery: a randomized controlled trial. Nicotine & tobacco research : official journal of the Society for Research on Nicotine and Tobacco. 2010;12:1118.  **Abstract:** BACKGROUNDSmokers are more prone to develop postoperative complications. Smoking cessation intervention beginning 4-8 weeks prior to surgery improves the postoperative outcome. Cancer patients, however, often undergo surgery less than 4 weeks after diagnosis. The primary objective of this study was therefore to examine if a brief smoking cessation intervention shortly before breast cancer surgery would influence postoperative complications and smoking cessation.METHODSA randomized controlled multicentre trial with blinded outcome assessment conducted at 3 hospitals in Denmark. One hundred and thirty patients were randomly assigned to brief smoking intervention (n = 65) or standard care (n = 65). The intervention followed the principles of motivational interviewing and included personalized nicotine replacement therapy aimed at supporting smoking cessation from 2 days before to 10 days after surgery.RESULTSThe overall postoperative complication rate (including seroma requiring aspiration) was 61% in both groups risk ratio (RR) 1.00 (95% CI 0.75-1.33). The wound complication rate was 44% versus 45%. The effect on perioperative smoking cessation was modest, 28% intervention versus 11% control group patients, RR 2.49 (95% CI 1.10-5.60). There was no effect on smoking cessation at 12 months, 13% versus 9%.CONCLUSIONSBrief smoking intervention administered shortly before breast cancer surgery modestly increased self-reported perioperative smoking cessation without having any clinical impact on postoperative complications. The study adds to the body of evidence indicating that brief intervention has no clinical importance for surgical patients in regard to postoperative morbidity. Future studies should be designed to determine the optimal time of smoking cessation before surgery. |
| 1105 | Sørensen, Lars Tue, Toft, Birgitte G, Rygaard, Jørgen, Ladelund, Steen, Paddon, Maria, James, Tim, Taylor, Richard, Gottrup, Finn. Effect of smoking, smoking cessation, and nicotine patch on wound dimension, vitamin C, and systemic markers of collagen metabolism. Surgery. 2010;148:982.  **Abstract:** BACKGROUNDPostoperative wound disruption and tissue-destructive disorders are more frequent in smokers than in nonsmokers. Impaired wound healing and altered connective tissue turnover are suggested mechanisms, but exact details remain unknown.METHODSFull-thickness, 5-mm punch biopsy wounds were made lateral to the sacrum in 48 smokers and were randomized double-blinded to continuous smoking, abstinence with transdermal nicotine patch (TNP), or abstinence with placebo patch and 30 never smokers. At 1, 4, 8, and 12 weeks, the wounds were excised and fixed for wound measurement, and blood was collected for measurement of vitamin C, procollagen I N-propeptide (PINP), matrix metalloproteinase 8 (MMP), MMP-9, neutrophils, and eosinophils.RESULTSOne week after wounding, smokers' wounds were 3.1 ± 0.1 mm (mean, standard error of the mean) wide and were 1.3 ± 0.1 mm deep compared with the never smokers' wounds, measuring 3.7 ± 0.1 mm wide and 1.5 ± 0.1 mm deep (P < .01, respectively). Abstinent smokers' wounds were 3.3 ± 0.1 mm wide (NS) and were 1.4 ± 0.1 mm deep (P = .02 compared with smokers). In smokers, vitamin C and PINP were 50.5 ± 9.0 μmol/L and were 52.7 ± 6.6 ng/mL, respectively, compared with 68.8 ± 14.5 μmolL and 64.7 ± 4.7 ng/mL in never smokers (P < .001 and P = .07). Both increased significantly after smoking cessation. Plasma MMP-8 and MMP-9 were correlated with neutrophil blood count, which significantly was affected by smoking status. No effect of TNP was found.CONCLUSIONSmokers have smaller, more superficial wounds and lesser blood levels of vitamin C and PINP. Smoking cessation resulted in increased wound depth, vitamin C, and PINP as well as a decreased neutrophil blood count. These findings suggest that wound contraction and collagen metabolism are affected by a smoking-induced alteration in vitamin C turnover and by a change in inflammatory cell response. |
| 1106 | Reid, R.D., Armstrong, A., McDonnell, L., Aitken, D.A., Robert, L., LaRue, A., Laroche, M., Pipe, A.L.. Varenicline versus transdermal nicotine patch for smoking cessation in patients with coronary heart disease: A pilot randomized trial. Canadian Journal of Cardiology. 2010;26:53D.  **Abstract:** BACKGROUND: Smoking cessation is vitally important for patients with CHD who smoke. Ideally, interventions should be commenced at the time of hospitalization. Nicotine replacement therapy (NRT) and varenicline are two first-line smoking cessation medications proven to increase the likelihood of smoking cessation in non-hospitalized smokers. To date there are no published studies reporting the comparative effectiveness of these medications in smokers hospitalized with CHD. A pilot randomized trial was conducted to estimate effect size for a larger trial comparing the effects of varenicline versus NRT in smokers hospitalized with CHD. CONCLUSION: A pilot randomized trial was conducted to estimate the effect size of varenicline versus NRT on cessation outcomes in smokers hospitalized with CHD. At the end of treatment, there was a potentially clinically important difference in cessation rates (13.4%) favouring varenicline compared to NRT. The comparative efficacy of these two medications needs to be examined in an appropriately powered clinical trial examining longer-term outcomes. |
| 1109 | Dulude, Louise, Labelle, Alain, Knott, Verner J. Acute nicotine alteration of sensory memory impairment in smokers with schizophrenia. Journal of clinical psychopharmacology. 2010;30:541.  **Abstract:** CONTEXTPatients with schizophrenia have a high rate of cigarette smoking and also exhibit profound deficits in sensory processing, which may in part be ameliorated by the acute actions of smoke-inhaled nicotine. The mismatch negativity (MMN), a preattentive event-related potential index of auditory sensory memory, is diminished in schizophrenia. The MMN is increased in healthy controls with acute nicotine.OBJECTIVETo utilize the MMN to compare auditory sensory memory in minimally tobacco-deprived (3 hours) patients and matched tobacco-deprived smoking controls and to assess the effects of acute nicotine on MMN-indexed sensory memory processing in the patients.DESIGNEvent-related potentials were recorded in 2 auditory oddball paradigms, one involving tone frequency changes (frequency MMN) and one involving tone duration changes (duration MMN). Controls were assessed once under nontreatment conditions, and patients were assessed twice under randomized double-blind treatment conditions involving placebo and nicotine (8 mg) gum.SETTINGOutpatient mental health center.PATIENTSTwelve smokers with schizophrenia and twelve control smokers.RESULTSCompared with the controls, the patients showed reduced frequency-MMN (P < 0.001) and duration-MMN (P < 0.04) amplitudes. In addition to prolonging peak latency in duration MMN (P < 0.01), nicotine, relative to placebo, increased the amplitude of the patients' duration MMN (P < 0.01), but not their frequency MMN, to a level comparable with that seen in the controls.CONCLUSIONSThese preliminary findings demonstrate for the first time that acute nicotine can normalize temporal aspects of sensory memory processing in patients with schizophrenia, an effect that may be mediated by activation of α7 nicotinic acetylcholine receptors, the function of which is diminished in schizophrenia. These ameliorating actions of nicotine may have implications for understanding the close relationship between tobacco smoking and schizophrenia and for developing nicotinic pharmacotherapies to alleviate sensory memory impairments in schizophrenia. |
| 1110 | Okuyemi, Kolawole S, Thomas, Janet L, Warren, Jennifer, Guo, Hongfei, Ahluwalia, Jasjit S. Relationship between smoking reduction and cessation among light smokers. Nicotine & tobacco research : official journal of the Society for Research on Nicotine and Tobacco. 2010;12:1005.  **Abstract:** INTRODUCTIONTo determine the smoking reduction patterns of light smokers (≤ 10 CPD) and whether reduction predicts future cessation.METHODSStudy is a secondary analysis of data that were derived from a 2 × 2 randomized study that assessed the efficacy of nicotine gum (vs. placebo) and counseling (motivational interviewing vs. health education) for smoking cessation among 755 light smokers. Participants were categorized into three groups based on self-reported CPD smoked at time of study enrollment compared with CPD smoked a year prior to enrollment. That is, (a) those who reduced number of cigarettes per day (CPD), (b) those who smoked the same number of CPD, and (c) those who increased their number of CPD. Sociodemographic and smoking characteristics were assessed at enrollment as well as cotinine-verified 7-day smoking abstinence rates at the Week 26 follow-up assessment. A stepwise logistic regression model to predict the probability abstinence at 26-week follow-up was also performed.RESULTSCompared with a year prior to enrollment, 43.7% of participants reduced, 35.2% smoked the same, and 21.2% increased their CPD. Compared with those who smoked the same or increased their CPD, those who had reduced their CPD were older, more likely to be males, smoked fewer CPD at enrollment, initiated smoking at a younger age, and less likely to be nicotine dependent. Adjusted logistic regression showed that those who had reduced their smoking prior to enrollment were more likely to quit at Week 26 (odds ratio [OR] = 1.77; 95% CI = 1.062-2.957; p = .029).DISCUSSIONFindings suggest that reducing number of CPD smoked prior to enrolling in a clinical trial is a positive predictor of abstinence. Therefore, encouraging smoking reduction prior to attempting cessation may enhance cessation outcomes for light smokers. |
| 1116 | Okuyemi, Kolawole S, Zheng, Hui, Guo, Hongfei, Ahluwalia, Jasjit S. Predictors of adherence to nicotine gum and counseling among African-American light smokers. Journal of general internal medicine. 2010;25:969.  **Abstract:** BACKGROUNDA high proportion of African-American smokers are light smokers, and they experience low smoking cessation rates and disproportionately high tobacco-related morbidity; yet no studies have examined tobacco treatment adherence in this group.OBJECTIVESTo determine the predictors of adherence to nicotine gum and counseling among African-American light smokers (defined as smoking < or =10 cigarettes/day), and the effects of adherence on smoking cessation.DESIGNData were from a 2 x 2 randomized, placebo-controlled smoking cessation trial of nicotine gum (2 mg versus placebo) and counseling (motivational interviewing versus health education).PARTICIPANTSSeven hundred fifty-five African-American light smokers at a community-based clinic.MEASUREMENTSDemographic and health-related information, smoking behaviors, psychosocial variables, adherence to nicotine gum and counseling, and cotinine-verified 7-day abstinence from smoking at week-26 follow-up.RESULTSA logistic regression model showed that having a higher body mass index (OR = 1.03, 95% CI = 1.01 to 1.05), more quit attempts in the past year (OR = 1.04, 95% CI = 1.01 to 1.07), higher baseline exhaled carbon monoxide (OR = 1.22, 95% CI = 1.01 to 1.48), and higher perceived stress (OR = 1.12, 95% CI = 1.03 to 1.22) increased the likelihood of adherence to nicotine gum. Being a high school graduate was a predictor of adherence to counseling (OR = 1.58, 95% CI = 1.02 to 2.44). Surprisingly, being adherent to nicotine gum significantly reduced the odds of smoking cessation (OR = 0.50, CI = 0.28 to 0.87). On the other hand, adherence to counseling dramatically increased the likelihood of smoking cessation (OR = 3.32, CI = 1.36 to 8.08).CONCLUSIONSIndividual risk factors may influence adherence to nicotine gum and counseling. Improving psychological interventions and promoting adherence to counseling may increase overall smoking cessation success among African-American light smokers. |
| 1117 | Unbehaun, T., Jaehne, A., Feige, B., Riemann, D.. Nicotine and sleep - studying smokers and healthy non-smokers during nicotine consumption and withdrawal. Journal of Sleep Research. 2010;19:107.  **Abstract:** Objectives: Sleep disorders, especially insomnia, can increase the risk for depression and substance dependency through heightened stress levels. In this study we examined the impact of nicotine on polysomnographically (PSG) recorded sleep in healthy, good sleeping nonsmokers. In a next step we investigated PSG in smoking subjects during a period of smoking, during withdrawal after discontinuation of smoking and three months later during abstinence from nicotine. We hypothesized that the amount of sleep disturbance observed during the cessation period might be predictive for early relapse into smoking. Methods: Sixty six healthy good sleepers (20-25 years., 33 females), matched for age and gender, were randomized to placebo or either 8 or 16 mg nicotine patch (daytime or night time application) in a double- blind, parallel group design. All subjects had an adaptation night followed by the experimental night. In the second study, 80 smokers will have two nights of polysomnographic examination during smoking, immediately after quitting and 3 months after cessation. Data from 40 smokers have been collected up to now. Results: Depending on dosage, nicotine administration caused increased sleep onset latency, decreased slow wave sleep, sleep efficiency and a decreased frequency of leg movements in sleep. Sleep was only slightly disturbed in smokers during nicotine cessation compared to the smoking state. Subjects who relapsed during the 3 months follow-up presented higher initial depressive symptoms (though on a subclinical level) and higher scores on a scale of nicotine dependency compared to abstainers; relapsers also reported stronger withdrawal symptoms and more pronounced impairments through subjective sleep disturbances during cessation. Conclusions: The preliminary data analysis shows a disturbance of polysomnographically measured sleep quality caused by nicotine consumption in healthy non-smoking good sleepers. Ex-smokers who relapse after initial successful smoking cessation seem to be characterized by increased depressive symptoms and stronger nicotine dependency before giving up smoking. Impairments of sleep during the withdrawal phase may contribute to earlier relapse into smoking behaviours. Sleep may serve as a model to better understand the effects of nicotine on the human brain and changes of sleep during nicotine cessation maybe indicative of relapse tendencies. |
| 1118 | Bailey, Steffani R, Fong, Dalea M, Bryson, Susan W, Fortmann, Stephen P, Killen, Joel D. Perceived drug assignment and treatment outcome in smokers given nicotine patch therapy. Journal of substance abuse treatment. 2010;39:150.  **Abstract:** This study assessed the relationship between treatment outcome and perceived drug assignment in smokers (nicotine patch [NP] or placebo) using abstinence and relapse status. Smokers (N = 424) were randomly assigned to receive either NP or placebo as part of a study that examined the effects of combining NP with self-help programs. Beliefs about drug assignment, assessed at the 12-month follow-up, were obtained from 384 participants. Beliefs were related to abstinence at the 2-month, p < .05, and 6-month follow-ups, p < .05, for the NP group, but not the placebo. Beliefs were not related to abstinence at 12 months for either group. Survival analysis assessing relapse revealed that beliefs were related to relapse status, regardless of actual group assignment. Our results suggest that there is a relationship between perceived drug assignment and treatment outcome. Future studies using multiple treatment outcome measures and assessments of beliefs over time are warranted. |
| 1123 | Kras, M., Stough, C., Scholey, A., Kure, C., Camfield, D.. Hypericum perforatum, nicotine patches and combination hypericum perforatum/nicotine patches for smoking cessation. European Neuropsychopharmacology. 2010;20:S608.  **Abstract:** Purpose: There are several treatments available for smoking cessation. However current treatments are only mildly efficacious and have side effects [1]. Research suggests that Hypericum Perforatum Extract (Ze117) influences the dopamine reward pathways [2], which may help reduce nicotine addiction. The current study examined the efficacy of 3 treatments over 10 weeks. 1. Ze117 (a high quality standardized HP extract manufactured by Zeller (Switzerland) containing 1.375g HP herb and 500mcg hypericin); 2. NicabateCQ Nicotine Replacement Therapy (NRT) using a recommended three-stage step down program of: 21 mg nicotine p/day (weeks 1-6), 14 mg nicotine p/day (weeks 7-8) and 7 mg nicotine p/day (weeks 9-10); 3. a combination of Ze117/ NRT. Methods: Sixty smokers (>10 cigarettes/day) aged 18-60 yrs were randomly allocated to receive Ze117, NRT, or combination Ze117/NRT. Smoking status was assessed using CO levels and self-reported number of cigarettes smoked over the course of the study. Secondary measures of anxiety (STAI), DSM-IV nicotine dependence, Fagerstrom Smoking Tolerance Scale and personality (NEO PI) were also recorded. Participants completed all measures four weeks prior to quitting (run-in baseline), on their quit date (baseline), and six and ten weeks post-quit.(Table presented) Results: Means and standard deviations are presented in Table 1. An analysis of CO levels using the Bedfont Smokerlyzer revealed no statistical difference in the number of smokers versus non-smokers at either the quite date (Kolmogorov-Smirnov Z = 0.116, p>0.05) or at the end of the treatment date, week 10 (Kolmogorov-Smirnov Z = 0.116, p>0.05). There was also no statistical difference in the change of the mean number of cigarettes smoked per week in any of the three conditions from quit date to 10 weeks of treatment. This indicates that the three conditions are equally effective in reducing the number of cigarettes smoked across the smoking cessation trial. There were no statistically significant differences in the secondary measures between the three treatment conditions. Conclusions: Although still regarded as a pilot study (N = 20 participants per arm), the results indicate the equivalence of all three treatments in the management of smoking cessation. NRT shows relatively poor efficacy for smoking cessation with many smokers unable to quite even after prolonged use of NRT [1]. Given the significant health effects of smoking, alternative treatments for smoking cessation are urgently required. The results of this study, although preliminary provide a strong framework for a large randomized double blind study to confirm the use of Ze117 for smoking cessation in addition to NRT. |
| 1126 | Ebbert, Jon O, Edmonds, Amanda, Luo, Xianghua, Jensen, Joni, Hatsukami, Dorothy K. Smokeless tobacco reduction with the nicotine lozenge and behavioral intervention. Nicotine & tobacco research : official journal of the Society for Research on Nicotine and Tobacco. 2010;12:823.  **Abstract:** INTRODUCTIONStudies have evaluated smoking reduction with nicotine replacement therapy to reduce tobacco exposure and facilitate abstinence among cigarette smokers, but none have evaluated a reduction approach in smokeless tobacco (ST) users.METHODSWe conducted an open-label pilot study to determine if the 4-mg nicotine lozenge with a behavioral intervention could facilitate ST use reduction among ST users compared with a behavioral intervention alone. Eligible subjects were ST users not interested in quitting.RESULTSOne hundred and two subjects were randomized. Both interventions were associated with significant decreases in ST use and toxicant exposure and with increased abstinence, quit attempts, and duration of abstinence. However, no significant differences were observed between groups for these outcomes.DISCUSSIONA behavioral intervention with or without the nicotine lozenge may be effective for decreasing both ST use and toxicant exposure and for increasing tobacco abstinence, quit attempts, and duration of abstinence. The use of reduction strategies for ST users not interested in quitting deserves further evaluation as an intervention strategy. |
| 1134 | McRobbie, Hayden, Thornley, Simon, Bullen, Chris, Lin, Ruey-Bin, Senior, Hugh, Laugesen, Murray, Whittaker, Robyn, Hajek, Peter. A randomized trial of the effects of two novel nicotine replacement therapies on tobacco withdrawal symptoms and user satisfaction. Addiction (Abingdon, England). 2010;105:1290.  **Abstract:** AIMSTo determine effects on craving, user satisfaction, and consumption patterns of two new nicotine replacement therapies (NRT) used for eight hours after overnight tobacco abstinence.DESIGNIn a within-subject, cross-over trial participants were randomly assigned Zonnic nicotine mouth spray (1 mg/spray), Zonnic nicotine lozenge (2.5 mg), Nicorette gum (4 mg) and placebo lozenge on each of four study days.SETTINGUniversity research unit.PARTICIPANTSForty-seven dependent adult smokers.MEASUREMENTSParticipants rated their urges to smoke, irritability, concentration and restlessness before and during the first hour of product use on a 100-point scale. A subsample of 11 participants provided blood samples for nicotine analysis.FINDINGSAll active products reduced craving significantly more than placebo (mean reductions of 28.6, 25.8, 24.7 and 8.9 points for mouth spray, gum, lozenge and placebo). Mouth spray relieved craving faster than placebo and gum with significant reductions within five minutes of use (mean differences of -14.5 (95% CI: -23.0 to -6.0) and -10.6 (95% CI: -19.1 to -2.1) with placebo and gum respectively. Mouth spray produced a faster time to maximum plasma nicotine concentration (14.5 minutes, 95% CI: 8.0 to 21.0) compared to the lozenge (30.3 minutes, 95% CI: 21.1 to 39.5) and gum (45.8 minutes, 95% CI: 36.2 to 55.4). Maximum concentrations of blood nicotine were higher with mouth spray (10.0 ng/ml) and lozenge (10.8 ng/ml) compared to gum (7.8 ng/ml). Both lozenge and mouth spray were well tolerated.CONCLUSIONSThe mouth spray and lozenge are at least as effective as 4 mg nicotine gum in relieving craving suggesting that they are likely to be effective in aiding smoking cessation. The mouth spray may be particularly useful for acute craving relief. |
| 1135 | Gordon, Judith S, Andrews, Judy A, Albert, David A, Crews, Karen M, Payne, Thomas J, Severson, Herbert H. Tobacco cessation via public dental clinics: results of a randomized trial. American journal of public health. 2010;100:1307.  **Abstract:** OBJECTIVESWe sought to compare the effectiveness of a dental practitioner advice and brief counseling intervention to quit tobacco use versus usual care for patients in community health centers on tobacco cessation, reduction in tobacco use, number of quit attempts, and change in readiness to quit.METHODSWe randomized 14 federally funded community health center dental clinics that serve diverse racial/ethnic groups in 3 states (Mississippi, New York, and Oregon) to the intervention (brief advice and assistance, including nicotine replacement therapy) or usual care group.RESULTSWe enrolled 2549 smokers. Participants in the intervention group reported significantly higher abstinence rates at the 7.5-month follow-up, for both point prevalence (F(1,12) = 6.84; P < .05) and prolonged abstinence (F(1,12) = 14.62; P < .01) than did those in the usual care group.CONCLUSIONSThe results of our study suggest the viability and effectiveness of tobacco cessation services delivered to low-income smokers via their dental health care practitioner in community health centers. Tobacco cessation services delivered in public dental clinics have the potential to improve the health and well-being of millions of Americans. |
| 1136 | Mumenthaler, Martin S, Benowitz, Neal L, Taylor, Joy L, Friedman, Leah, Noda, Art, Yesavage, Jerome A. Nicotine deprivation and pilot performance during simulated flight. Aviation, space, and environmental medicine. 2010;81:660.  **Abstract:** OBJECTIVESMost airlines enforce no-smoking policies, potentially causing flight performance decrements in pilots who are smokers. We tested the hypotheses that nicotine withdrawal affects aircraft pilot performance within 12 h of smoking cessation and that chewing nicotine gum leads to significant relief of these withdrawal effects.METHODSThere were 29 pilots, regular smokers, who were tested in a Frasca 141 flight simulator on two 13-h test days, each including three 75-min flights (0 hr, 6 hr, 12 hr) in a randomized, controlled trial. On the first day (baseline), all pilots smoked one cigarette per hour. On the second day, pilots were randomly assigned to one of four groups: (1) nicotine cigarettes; (2) nicotine gum; (3) placebo gum; (4) no cigarettes/no gum. Flight Summary Scores (FSS) were compared between groups with repeated measures ANOVAs.RESULTSNo statistically significant differences in overall simulator flight performance were revealed between pilots who smoked cigarettes and pilots who were not allowed to smoke cigarettes or chew nicotine gum, but there was a trend for pilots who were not allowed to smoke to perform worse. However, pilots who chewed placebo gum performed significantly worse during the 6-h (FSS = -0.03) as well as during the 12-h flight (FSS = -0.08) than pilots who chewed nicotine gum (FSS = 0.15 / 0.30, respectively).CONCLUSIONSResults suggest that nicotine withdrawal effects can impair aircraft pilot performance within 12 h of smoking cessation and that during smoking abstinence chewing one stick of 4-mg nicotine gum per hour may lead to significantly better overall flight performance compared to chewing placebo gum. |
| 1138 | Ferguson, Stuart G, Shiffman, Saul. Effect of high-dose nicotine patch on the characteristics of lapse episodes. Health psychology : official journal of the Division of Health Psychology, American Psychological Association. 2010;29:358.  **Abstract:** OBJECTIVENicotine patch improves treatment outcomes, but lapses are still common. To understand the process of relapse on patch, we investigated differences in the antecedents (withdrawal, setting, triggers) of lapse episodes experienced on high-dose (35 mg) nicotine patches versus placebo.DESIGNParticipants were smokers who lapsed during a randomized, double-blind trial of active patches (n = 100) versus placebo (n = 85). Participants used electronic diaries to monitor their smoking, affect, and activities in real time for 5 weeks during their cessation attempt.RESULTSWe analyzed 490 lapse episodes (active: 266; placebo: 224). Lapses on nicotine patch were characterized by significantly lower positive affect and higher negative affect than placebo lapses. Participants treated with high-dose patch were also significantly more likely to lapse in situations involving little or no craving. Situational antecedents of lapses on patch resembled those on placebo.CONCLUSIONThe results suggest that treatment with patch may set a higher threshold for affective stimuli to provoke lapses, but does not change the proximal cues that trigger lapses. This suggests that behavioral relapse-prevention strategies developed for unmedicated smokers should also apply to those treated with nicotine patch. |
| 1142 | Tsukahara, H., Noda, K., Uehara, Y., Saku, K.. A randomized open trial of varenicline versus nicotine patch in adult smokers: Efficacy, safety and withdrawal symptoms (the VN-seesaw study). Atherosclerosis Supplements. 2010;11:151.  **Abstract:** We conducted a randomized controlled trial of varenicline versus nicotine patch in adult smokers, and compared efficacy, safety and withdrawal symptoms. Thirty-two adult smokers were randomly divided into a varenicline group (VG, n = 16, varenicline, 0.5-2 mg daily) and a nicotine patch group (NG, n = 16, 52.5-7 mg nicotine daily) for 12 weeks and 8 weeks, respectively. The primary endpoints were the 12- and 24-week smoking-abstinence rates, withdrawal symptoms and safety. No significant difference in abstinence rates was observed between the two groups over weeks 9-12, and weeks 9-24. The total withdrawal symptom score in VG was higher than that in NG at 2 weeks (14.8±3.5 vs. 12.2±2.4, p = 0.083): in detail, the frequencies of an inability to concentrate at 2, 4, and 8 weeks (P = 0.034, 0.070, 0.10, respectively), wakeful nights at 2 weeks (p = 0.003), and a lack of self-composure at 4 weeks (p = 0.146) were higher in VG than in NG. Adverse side effects associated with a gastro-intestinal disorder were observed in 14 cases and 1 case, and skin allergy was seen in 0 and 9 cases, respectively. BW and BMI were increased after 12 weeks in both groups, while systolic blood pressure tended to decrease in NP and LDL-C and HDL-C did not change after 12 weeks. Urinary 8-OHdG and 8-isoprostane decreased in NG group. We concluded that the selection of treatment should depend on the patient's request based on the desired acuteness of cessation of smoking, gastro-intestinal problems, and skin allergy. |
| 1151 | Ebbert, Jon O, Severson, Herbert H, Croghan, Ivana T, Danaher, Brian G, Schroeder, Darrell R. A pilot study of mailed nicotine lozenges with assisted self-help for the treatment of smokeless tobacco users. Addictive behaviors. 2010;35:522.  **Abstract:** Smokeless tobacco (ST) is associated with adverse health consequences yet treatment resources for ST are not widely available. Cost-effective behavioral interventions incorporating self-help materials and counseling calls have been demonstrated to reduce ST use rates and can be easily disseminated, but the feasibility and effectiveness of incorporating pharmacotherapy into this approach have not been evaluated. We conducted a clinical pilot study randomizing 60 patients to 12 weeks of the 4-mg nicotine lozenge or placebo delivered through the mail. All subjects received an assisted self-help intervention (ASH) with telephone support. At the end of the medication phase, lozenges were being used by 63% of subjects in the 4-mg nicotine lozenge group and 43% in placebo. The nicotine lozenge decreased composite withdrawal symptoms and adverse events were minimal. No significant differences were observed in abstinence rates between the two groups at 3 or 6 months. We conclude that the mailing of nicotine lozenges to ST users is a feasible and safe strategy the efficacy of which needs to be evaluated. |
| 1153 | Brunner-Frandsen, N.S., Bak, S.. Smoking cessation intervention after stroke or transient ischemic attack. A randomised controlled trial. Cerebrovascular Diseases. 2010;29:323.  **Abstract:** Background: Smoking cessation is widely recommended for secondary. However, little is known about the efficacy of smoking cessation advise after stroke or transient ischemic attack (TIA). Methods: Ninety-four current smokers under age 76 admitted with acute ischemic stroke or TIA were randomised to smoking cessation counselling or an intensive smoking cessation programme. The smoking cessation counselling consisted of a 30 minutes individual counselling by the study nurse. The intensive smoking cessation programme included the smoking cessation counselling by the study nurse, a 5 session smoking cessation programme by an authorized smoking cessation instructor plus a 30 minutes out-patient visit after 6 weeks and 5 telephone counselling sessions by the study nurse. Free samples of nicotine replacement therapy were delivered as part of the intensive smoking cessation programme. Smoking cessation rates at 6 months were determined by self-report and verified by measurement of exhaled carbon monoxide (CO). Results: The 6 months self-reported smoking cessation rate was 0.38 in the counselling group and 0.43 in the intensive programme group. The smoking cessation rates verified by exhaled CO levels in the counselling group and the intensive programme group were 0.29 and 0.33 respectively. No difference was found between the two groups (χ2=0.16, P=0.69). |
| 1156 | Marton, J.P., Zou, K.H., Russ, C., Willke, R.J.. Comparison of patient reported outcomes based on the minnesota nicotine withdrawal scale (MNWS) using abstinence profiles in treatments with varenicline and transdermal nicotine patch (NRT). Value in Health. 2010;13:A202.  **Abstract:** OBJECTIVES: To conduct a post-hoc analysis of the time-courses of MNWS item or domain scores (MNWS scores) and weekly point prevalence of abstinence (PVR) during the treatment phase of a previously-published (Aubin et al, 2008) randomized open-label clinical trial of varenicline (N = 376) vs. NRT (N = 370). METHODS: Current cigarette smokers, motivated to quit smoking, participated in the trial and completed the MNWS instrument. Descriptive statistics (mean ± standard error) of the MNWS scores from weeks 2 to 7 were computed. Time-course comparisons stratified by PVR were performed, with weekly responders defined by PVR = 0 and nonresponders otherwise. Multivariate repeated-measures mixed-effects regression was conducted for each MNWS domain score as the outcome variable. Covariates included baseline, treatment, patient characteristics, smoking history and PVR. Statistical significance was reached when two-sided p ≤ 0.05. RESULTS: The mean baseline MNWS scores of varenicline vs. NRT were comparable. By PVR, varenicline significantly reduced the mean urge to smoke vs. NRT in weeks 2, 3, 4 and 5 (0.45 ± 0.10; 0.28 ± 0.09; 0.27 ± 0.10; 0.27 ± 0.09, respectively; all p < 0.01) among responders, and in weeks 2 to 6 (0.59 ± 0.13; 0.44 ± 0.14; 0.46 ± 0.16; 0.52 ± 0.18; 0.38 ± 0.17; all p < 0.03) among non-responders. The mean negative affect scores were significantly lower in weeks 2 to 5 and 7 (0.31 ± 0.07; 0.20 ± 0.06; 0.18 ± 0.07; 0.23 ± 0.07; 0.13 ± 0.06; all p < 0.03) among responders and in week 2 (0.22 ± 0.11; p < 0.04) among non-responders. Additionally, restlessness was significantly reduced in weeks 2 to 5 (0.45 ± 0.10; 0.33 ± 0.09; 0.22 ± 0.09; 0.22 ± 0.08; all p < 0.02) among responders and in week 2 (0.42 ± 0.14; p < 0.003) among non-responders, and similarly the reduction of increased appetite (0.38 ± 0.18; p < 0.05) among nonresponders in week 7. CONCLUSIONS: Overall, lower mean patient-reported MNWS scores associated with symptoms of tobacco withdrawal were observed for varenicline than for NRT, reaching statistical significance, particularly among abstainers. Differences in the dynamics of treatment effects along with concomitant abstinence status warrant further bivariate analyses. |
| 1158 | Tsukahara, Hitomi, Noda, Keita, Saku, Keijiro. A randomized controlled open comparative trial of varenicline vs nicotine patch in adult smokers: efficacy, safety and withdrawal symptoms (the VN-SEESAW study). Circulation journal : official journal of the Japanese Circulation Society. 2010;74:771.  **Abstract:** BACKGROUNDIt has been suggested that anti-smoking therapy gives encouraging results, but this has not been verified by well-randomized study protocols. The present study was a randomized controlled trial of varenicline vs nicotine patch in adult smokers for comparison of efficacy, safety and withdrawal symptoms.METHODS AND RESULTSThe 32 adult smokers were randomly divided into a varenicline group (VG, n=16) and a nicotine patch group (NG, n=16). The primary endpoints were the 12- and 24-week smoking-abstinence rates, safety and withdrawal symptoms including stress. No significant difference in abstinence rates was observed between the 2 groups over weeks 9-12 (71.4% vs 78.6% in the VG and NG, respectively), and weeks 9-24 (64.3% vs 71.4%, respectively). The frequencies of inability to concentrate at 2, 4, and 8 weeks, and wakeful nights at 2 weeks were higher in the VG than in the NG. Adverse side-effects associated with a gastrointestinal disorder occurred in 14 cases and 1 case in the VG and NG, respectively, and skin allergy was seen in 0 and 9 cases, respectively.CONCLUSIONSThe selection of treatment depends on the balance of desired acuteness of cessation of smoking and side-effects, such as psychiatric and gastrointestinal problems or skin allergy. |
| 1160 | Knott, V, Millar, A, Fisher, D, Albert, P. Effects of nicotine on the amplitude and gating of the auditory P50 and its influence by dopamine D2 receptor gene polymorphism. Neuroscience. 2010;166:145.  **Abstract:** Evidence of normalized auditory P50 suppression with acute nicotine in schizophrenia has supported the contention that elevated smoking rates in this disorder may be an attempt to correct a nicotinic receptor pathophysiology that may underly impaired sensory gating in these patients. There is very little information regarding the neurochemical or genetic pathways through which nicotine regulates P50 amplitude and its suppression in human studies. In a randomized, double-blind, placebo-controlled design with 24 non-smokers, this study examined the influence of TaqIA dopamine D2 receptor gene polymorphisms on P50 and its inhibition during nicotine gum (6 mg) administration. Within a paired click (S(1)-S(2)) paradigm, placebo treated A1(+) and A1(-) allele groups differed with respect to P50 amplitude and gating. While nicotine (relative to placebo) attenuated S(1) P50 amplitude in A1(+) allele carriers, in the A1(-) carriers it increased S(2) P50 amplitude and increased P50 gating as indexed by an augmented gating difference wave (GDW). These findings suggest that nicotine exerts mixed gating properties in healthy nicotine naive volunteers and that dopamine functions to alter both P50 and its gating as well as their response to acute nicotine agonist treatment. |
| 1162 | Sonne, Susan C, Nunes, Edward V, Jiang, Huiping, Tyson, Clare, Rotrosen, John, Reid, Malcolm S. The relationship between depression and smoking cessation outcomes in treatment-seeking substance abusers. The American journal on addictions. 2010;19:111.  **Abstract:** The National Drug Abuse Treatment Clinical Trials Network (CTN) recently completed a randomized, open label trial comparing treatment as usual (TAU) combined with nicotine patches plus cognitive behavioral group counseling for smoking cessation (n = 153) to TAU alone (n = 72) for patients enrolled in treatment programs for drug or alcohol dependence, who were interested in quitting smoking. This report is a secondary analysis evaluating the effect of depressive symptomatology (n = 70) or history of depression (n = 110) on smoking cessation outcomes. A significant association was seen between measures of depression and difficulty quitting cigarettes. Specifically, there was a greater probability for smoking abstinence for those with lower baseline Beck Depression Inventory II (BDI-II) scores. These data suggest that evaluation and treatment of depressive symptoms may play an important role in improving smoking cessation outcomes. (Am J Addict 2010;00:1-8). |
| 1163 | Barrett, Sean P. The effects of nicotine, denicotinized tobacco, and nicotine-containing tobacco on cigarette craving, withdrawal, and self-administration in male and female smokers. Behavioural pharmacology. 2010;21:144.  **Abstract:** The effects of the acute administration of nicotine [through nicotine inhalers (NI) and placebo inhalers (PI)], nicotine-containing tobacco (NT), and denicotinized tobacco (DT), on smokers' subjective responses and motivation to smoke were examined in 22 smokers (12 male, 10 female; 11 low dependent, 11 high dependent). During four randomized blinded sessions, participants self-administered NI, PI, NT, or DT, and assessed their effects using Visual Analogue Scales and the Brief Questionnaire of Smoking Urges. They could then self-administer their preferred brand of cigarettes using a progressive ratio task. NT and DT were each associated with increased satisfaction and relaxation as well as decreased craving relative to the inhalers and NT increased ratings of stimulation relative to each of the other products. Both NT and DT delayed the onset of preferred tobacco self-administration relative to NI and PI but only NT reduced the total amount self-administered. Sex differences were evident in the effects of DT on withdrawal-related cravings with women experiencing greater DT-induced craving relief than men. Findings suggest that DT is effective in acutely reducing many smoking abstinence symptoms, especially in women, but a combination of nicotine and non-nicotine tobacco ingredients may be necessary to suppress smoking behavior. |
| 1166 | Bock, Beth C, Hudmon, Karen S, Christian, James, Graham, Amanda L, Bock, Frederick R. A tailored intervention to support pharmacy-based counseling for smoking cessation. Nicotine & tobacco research : official journal of the Society for Research on Nicotine and Tobacco. 2010;12:217.  **Abstract:** INTRODUCTIONPharmacists are uniquely positioned within the community to provide smoking cessation counseling to their patients. However, pharmacists experience significant barriers to providing counseling, including limited time, reimbursement, and training in counseling techniques. We tested a computer-driven software system, "Exper_Quit" (EQ), that provided individually tailored interventions to patients who smoke and matching tailored reports for pharmacists to help guide cessation counseling.METHODSA two-phase design was used to recruit an observation-only group (OBS; n = 100), followed by participants (n = 200) randomly assigned to receive either EQ-assisted pharmacist counseling or EQ plus 8 weeks of nicotine transdermal patch (EQ+). Both treatment groups were scheduled to receive two follow-up counseling calls from pharmacists.RESULTSMost participants in the EQ and EQ+ groups reported receiving counseling from a pharmacist, including follow-up calls, while none of the OBS participants reported speaking with the pharmacist about cessation. At 6 months, fewer OBS participants reported a quit attempt (42%) compared with EQ (76%) or EQ+ (65%) participants (p < .02). At 6 months, 7-day point-prevalence abstinence was 28% and 15% among the EQ+ and EQ groups, respectively, compared with 8% among OBS participants (p < .01), and EQ+ participants were twice as likely to be quit than were EQ participants (p < .01).DISCUSSIONA tailored software system can facilitate the delivery of smoking cessation counseling to pharmacy patients. Results suggest that EQ was successful in increasing (a) the delivery of cessation counseling, (b) quit attempts, and (c) quit rates. Pharmacists can play an important role in the effective delivery of smoking cessation counseling. |
| 1168 | Sørensen, Lars Tue, Toft, Birgitte, Rygaard, Jørgen, Ladelund, Steen, Teisner, Børge, Gottrup, Finn. Smoking attenuates wound inflammation and proliferation while smoking cessation restores inflammation but not proliferation. Wound repair and regeneration : official publication of the Wound Healing Society [and] the European Tissue Repair Society. 2010;18:186.  **Abstract:** Full-thickness 5 mm punch biopsy wounds were made lateral to the sacrum in 48 smokers and 30 never smokers. After 1 week, the wounds were excised and fixed. The smokers were then randomized to continuous smoking or abstinence with a transdermal nicotine patch or a placebo patch. The sequence of wounding and excision was repeated after 4, 8, and 12 weeks. All excised tissue was stained with hematoxylin-eosin and immunohistochemically for macrophages (CD68), procollagen 1 N-terminal propeptide (PINP) in fibroblasts, and endothelial cells (CD31). The cellularity was assessed and scored by two independent histopathologists, and for the analysis, proportional odds models and random effect models for repeated measurements were applied. Macrophages and PINP-stained fibroblasts were reduced in the smokers' wounds (0.28 [0.14-0.58] [OR, 95%CI]; p=0.01 and 0.37[0.19-0.70]; p<0.01, respectively, when compared with never smokers' wounds). Inflammation scores were marginally affected. Following smoking cessation, inflammatory cell infiltration and macrophages in the wounds increased. PINP-stained fibroblasts were unaffected. Neovascularization was not affected by smoking or abstinence. Wound inflammation and fibroblast proliferation were attenuated in smokers, suggesting delayed healing. Abstinence from smoking restores inflammation, but does not affect proliferation. These findings suggest a pathophysiologic mechanism for postoperative wound infection and dehiscence in smokers and why smoking cessation appears to reduce wound infection but not dehiscence. |
| 1170 | Fisher, Derek J, Scott, Terri Lynne, Shah, Dhrasti K, Prise, Stephanie, Thompson, Mackenzie, Knott, Verner J. Light up and see: enhancement of the visual mismatch negativity (vMMN) by nicotine. Brain research. 2010;1313:162.  **Abstract:** Both smoking and nicotine can facilitate cognitive efficiency in humans, however the exact mechanism underlying this improvement in cognitive performance is unclear. Nicotine-related improvements in visual task performance may stem from facilitation of the identification and encoding of rare deviant stimuli at early sensory levels. Visual processes at these early levels are thought to be indexed by the visual mismatch negativity (vMMN), an event-related potential (ERP) measure of pre-conscious deviant detection. In order to contribute to our understanding of the neural mechanisms underlying nicotinic modulated cognition, the current study investigated the acute effects of nicotine on vMMN in a non-smoking sample. Twenty-seven volunteers (7 males, 20 females) were treated with nicotine gum (6 mg) in a double-blind randomized, placebo-controlled repeated measures design. ERPs (vMMN; visual N100 and P200) and motor indices of performance were extracted from an intermodal task, requiring participants to attend selectively to auditory targets presented within concurrent, non-overlapping oddball sequences of visual standard and deviant stimuli. Behavioural performance was unaffected by nicotine, however nicotine was found to enhance vMMN and P200 amplitude. The findings are discussed in relation to attentional and neurobiological theories of nicotine dependence and of cognition in general. |
| 1173 | Hukkanen, Janne, Jacob Iii, Peyton, Peng, Margaret, Dempsey, Delia, Benowitz, Neal L. Effects of nicotine on cytochrome P450 2A6 and 2E1 activities. British journal of clinical pharmacology. 2010;69:152.  **Abstract:** AIMSSmoking slows the metabolism of nicotine and accelerates the metabolism of chlorzoxazone, which are probe reactions for cytochrome P450 2A6 (CYP2A6) and CYP2E1 activities, respectively. We aimed to determine the role of nicotine in these metabolic effects of cigarette smoking.METHODSThe study had a single-blind, randomized, crossover two-arm design. Twelve healthy smokers were given two transdermal patches with 42-mg nicotine a day or placebo patches, each for 10 days. The subjects abstained from smoking during the study arms. Oral chlorzoxazone was given on day 7 and deuterium-labelled nicotine-d(2) and cotinine-d(4) infusion on day 8.RESULTSThere was no significant influence of transdermal nicotine administration on pharmacokinetic parameters of nicotine-d(2) or on the formation of cotinine-d(2). Nicotine decreased the volume of distribution (62.6 vs. 67.7 l, 95% confidence interval of the difference -9.7, -0.6, P= 0.047) of infused cotinine-d(4). There were no significant differences in disposition kinetics of chlorzoxazone between the treatments.CONCLUSIONSCYP2A6 and CYP2E1 activities are not affected by nicotine. The tobacco smoke constituents responsible for the reduced CYP2A6 and increased CYP2E1 activities remain unknown. |
| 1177 | Croghan, Ivana T., Hurt, Richard D., Ebbert, Jon O., Croghan, Gary A., Polk Jr., Octavius D., Stella, Philip J., Novotny, Paul J., Sloan, Jeff, Loprinzi, Charles L.. Racial differences in smoking abstinence rates in a multicenter, randomized, open-label trial in the United States. Journal of Public Health. 2010;18:59.  **Abstract:** Background: This study evaluates differences in smoking abstinence between white and minority smokers using pharmaceutical aids. Methods: This is an analysis of data from a multi-center, randomized, clinical trial conducted in the United States. Of the 1,684 subjects randomized to one of three medications (nicotine inhaler, bupropion, or a combination of both), 60% were women and 10% were minority races. Results: Factors associated with a decreased likelihood of smoking at 12 weeks were older age (OR = 0.971, p< 0.0001), being married (OR = 0.678, p= 0.0029), using bupropion SR (OR = 0.480, p∈<∈0.0001), and using combination therapy (OR = 0.328, p∈<∈0.0001). Factors associated with an increased likelihood of smoking were higher tobacco dependence scores (OR = 1.244, p < 0.0001), prior quit attempts (OR = 1.812, p=0.004), and being a minority (OR = 1.849, p=0.0083). Compared to white smokers, minority smokers were significantly older at time of study entry (46 vs. 42 years, p< 0.0001), less likely to be married (35% vs. 59%, p< 0.0001), older at smoking initiation (21 vs. 19 years of age, p< 0.0001), and had a lower abstinence rate (16% vs. 26%, p=0.0065). Conclusion: Regardless of the treatment used, minority smokers in the US have lower smoking abstinence after treatment for tobacco dependence. Future research should focus on the improvement in treatment strategies for minority smokers. |
| 1184 | West, Robert, May, Sylvia, McEwen, Andy, McRobbie, Hayden, Hajek, Peter, Vangeli, Eleni. A randomised trial of glucose tablets to aid smoking cessation. Psychopharmacology. 2010;207:631.  **Abstract:** RATIONALEOral glucose has been found to decrease tobacco craving among abstaining smokers. One study has demonstrated an effect of glucose on short-term abstinence. There is a need to examine any long-term benefit of glucose on abstinence.OBJECTIVESTo assess whether glucose tablets improve 6-month continuous abstinence rates compared with low-calorie placebo tablets.METHODSSmokers attempting to stop (n = 928) were randomised to receive glucose or sorbitol (placebo) in a double-blind placebo-controlled trial. All participants received group-based psychological support, and approximately half (n = 474) received nicotine replacement therapy (NRT), buproprion, or both. Smokers were seen weekly for 5 weeks and used tablets ad libitum, with a recommended minimum of 12 per day. Participants were recruited through general practitioner referral, word of mouth, and advertising. The participants were 38% male, smoked an average of 23.5 cigarettes per day, and had a mean age of 44 years. There were no significant pretreatment differences between groups. The primary outcome measure was continuous, CO-verified abstinence from the target quit date for 6 months.RESULTSNo significant effect of glucose tablets on abstinence was found (14.6% vs 13.4% abstinence in the glucose and placebo groups, respectively). However, there was a significant interaction with a glucose effect observed in smokers also receiving other medication (18.2% vs 12.6%, p < 0.05) but not otherwise (10.7% vs 14.3% ; p < 0.05 for the interaction).CONCLUSIONSNo significant effect of glucose tablets over and above sweet tasting tablets could be detected overall, but the possibility of an effect as an adjunct to NRT or bupropion merits further investigation. |
| 1186 | Erden, Veysel, Başaranoǧlu, Gökçen, Erkalp, Kerem, Yangin, Zehra, Delatioǧlu, Hamdi, Teker, M. Gökhan, Saitoǧlu, Leyla. The effect of nicotine treatment on propofol injection pain. Trakya Universitesi Tip Fakultesi Dergisi. 2010;27:18.  **Abstract:** Objectives: Smokers had higher risk than nonsmokers for pain. The incidence of pain on injection of propofol has been reported to be 70%. We explored the relationship between smoking habits and propofol injection pain, whether nicotine replacement has an influence on intensity or incidence of pain associated with propofol in smokers. Patients and Methods: Two hundred patients ASA (American Society of Anesthesiologists) physical status 1, undergoing elective surgical procedures were studied. Hundred (50-50) patients who used cigarette were included in smoker group and nicotine group. Hundred (50-50) patients who never used tobacco were included in nonsmoker (NS) group and NS+nicotine group. Prior to surgery, the anesthesiologist gave a nicotine gum to nicotine group which was randomized in smokers and nonsmokers. The severity of propofol injection pain was evaluated using four point scale (none, mild, moderate, and severe). Results: Incidence of pain was higher in the smoker group compared with the nonsmoker, nicotine and NS+nicotine groups. The intensity of mild pain was higher in the smoker group compared with the nonsmoker group. The intensity of moderate+severe pain was lower in the NS+nicotine group compared with the nonsmoker and smoker and nicotine groups. Conclusion: The incidence of propofol injection pain and intensity of mild pain increased in smokers deprived of nicotine. Nicotine replacement in these patients reduced the incidence of propofol injection pain similar to nonsmokers. When nonsmokers were given nicotine, intensity of moderate+severe pain decreased. These results suggest that smokers' awareness of pain increases and nicotine has an analgesic effect. © Medical Journal of Trakya University. Published by Ekin Medical Publishing. All rights reserved. |
| 1187 | Smith, Stevens S, McCarthy, Danielle E, Japuntich, Sandra J, Christiansen, Bruce, Piper, Megan E, Jorenby, Douglas E, Fraser, David L, Fiore, Michael C, Baker, Timothy B, Jackson, Thomas C. Comparative effectiveness of 5 smoking cessation pharmacotherapies in primary care clinics. Archives of internal medicine. 2009;169:2148.  **Abstract:** BACKGROUNDRandomized efficacy clinical trials conducted in research settings may not accurately reflect the benefits of tobacco dependence treatments when used in real-world clinical settings. Effectiveness trials (eg, in primary care settings) are needed to estimate the benefits of cessation treatments in real-world use.METHODSA total of 1346 primary care patients attending routine appointments were recruited by medical assistants in 12 primary care clinics. Patients were randomly assigned to 5 active pharmacotherapies: 3 monotherapies (nicotine patch, nicotine lozenge, and bupropion hydrochloride sustained release [SR]) and 2 combination therapies (patch + lozenge and bupropion SR + lozenge). Patients were referred to a telephone quit line for cessation counseling. Primary outcomes included 7-day point prevalence abstinence at 1 week, 8 weeks, and 6 months after quitting and number of days to relapse.RESULTSAmong 7128 eligible smokers (> or =10 cigarettes per day) attending routine primary care appointments, 1346 (18.9%) were enrolled in the study. Six-month abstinence rates for the 5 active pharmacotherapies were the following: bupropion SR, 16.8%; lozenge, 19.9%; patch, 17.7%; patch + lozenge, 26.9%; and bupropion SR + lozenge, 29.9%. Bupropion SR + lozenge was superior to all of the monotherapies (odds ratio, 0.46-0.56); patch + lozenge was superior to patch and bupropion monotherapies (odds ratio, 0.56 and 0.54, respectively).CONCLUSIONSOne in 5 smokers attending a routine primary care appointment was willing to make a serious quit attempt that included evidence-based counseling and medication. In this comparative effectiveness study of 5 tobacco dependence treatments, combination pharmacotherapy significantly increased abstinence compared with monotherapies. Provision of free cessation medications plus quit line counseling arranged in the primary care setting holds promise for assisting large numbers of smokers to quit. Trial Registration clinicaltrials.gov Identifier: NCT00296647. |
| 1188 | Olson, Luke C, Hong, Daewha, Conell-Price, Jessamyn S, Cheng, Sean, Flood, Pamela. A transdermal nicotine patch is not effective for postoperative pain management in smokers: a pilot dose-ranging study. Anesthesia and analgesia. 2009;109:1987.  **Abstract:** BACKGROUNDNicotine has an antinociceptive effect in animal models. The analgesic effect in humans has been examined, but studies have had mixed results. A proposed etiology is variability in chronic nicotine exposure because of differences in tobacco smoking rates and second-hand smoke exposure. In this study, we examined the postoperative analgesic effect of a transdermal nicotine patch in smokers in a parallel design to a previous study in nonsmokers.METHODSWe conducted a randomized, double-blind, prospective, placebo-controlled trial of 28 patients undergoing abdominal or pelvic surgery who required patient-controlled analgesia and an overnight hospital stay. Before anesthetic induction, a transdermal nicotine patch was applied (0, 5, 10, or 15 mg). The primary outcome variable was postoperative pain reported over the first hour and over the next 5 days using a standard numerical rating scale. Secondary outcome variables were pain medication use, hemodynamic values, nausea, and sedation.RESULTSPatients treated with nicotine reported higher pain scores than those treated with placebo over the first hour after surgery (P < 0.01, average numerical rating scale increase = 0.67) and there was no difference between groups in the subsequent 5 days (P > 0.05). There was no significant dose effect. Diastolic blood pressure in the first hour was higher in the placebo group compared with the nicotine-treated group (P < 0.01, average increase = 11 mm Hg). There was no difference in nausea or sedation.CONCLUSIONSTransdermal nicotine, 5-15 mg, failed to relieve postoperative pain or reduce opioid use in smokers. |
| 1189 | Bolin, Kristian, Wilson, Koo, Benhaddi, Hicham, de Nigris, Enrico, Marbaix, Sophie, Mork, Ann-Christin, Aubin, Henri-Jean. Cost-effectiveness of varenicline compared with nicotine patches for smoking cessation--results from four European countries. European journal of public health. 2009;19:650.  **Abstract:** BACKGROUNDThe aim of this study was to evaluate and compare the cost-effectiveness of varenicline with nicotine replacement therapy (NRT) for smoking cessation in four European countries (Belgium, France, Sweden and the UK).METHODSMarkov simulations, using the Benefits of Smoking Cessation on Outcomes (BENESCO) model, were performed. We simulated the incidence of four smoking-related morbidities: lung cancer, chronic obstructive pulmonary disease, coronary heart disease and stroke. The model computes quality-adjusted life-years gained and incremental cost-effectiveness ratios. Incremental cost-utility ratios were calculated, adopting a lifetime perspective. Efficacy data were obtained from a randomized open-label trial: Week 52 continuous abstinence rates were 26.1% for varenicline and 20.3% for NRT.RESULTSThe analyses imply that for countries analysed, smoking cessation using varenicline versus NRT was associated with reduced smoking-related morbidity and mortality. The number of morbidities avoided, per 1000 smokers attempting to quit, ranged from 9.7 in Belgium to 6.5 in the UK. The number of quality-adjusted life-years gained, per 1000 smokers, was 23 (Belgium); 19.5 (France); 29.9 (Sweden); and 23.7 (UK). In all base-case simulations (except France), varenicline dominated (more effective and cost saving) NRT regarding costs per quality-adjusted life-year gained; for France the incremental cost-effectiveness ratio was 2803.CONCLUSIONThis cost-effectiveness analysis demonstrated that since varenicline treatment was more effective, the result was increased healthcare cost savings in Belgium, Sweden and the UK. Our results suggest that funding varenicline as a smoking cessation aid is justifiable from a healthcare resource allocation perspective. |
| 1191 | Ebbert, Jon O, Severson, Herbert H, Croghan, Ivana T, Danaher, Brian G, Schroeder, Darrell R. A randomized clinical trial of nicotine lozenge for smokeless tobacco use. Nicotine & tobacco research : official journal of the Society for Research on Nicotine and Tobacco. 2009;11:1415.  **Abstract:** INTRODUCTIONSmokeless tobacco (ST) use is associated with adverse health consequences, and effective treatments are needed. Pilot data suggest that 4-mg nicotine lozenge decreases tobacco craving and nicotine withdrawal symptoms among ST users.METHODSWe conducted a randomized, placebo-controlled multicenter clinical trial to evaluate the efficacy of 12 weeks of 4-mg nicotine lozenge for ST use.RESULTSWe randomized 270 participants (136 active lozenge, 134 placebo). No significant differences were observed between the groups in biochemically confirmed all tobacco abstinence rates at Week 12 (36% lozenge vs. 27.6% placebo; odds ratio [OR] 1.5, 95% CI 0.7-2.1; p = .138). However, the 4-mg nicotine lozenge increased self-reported all tobacco abstinence (44.1% vs. 29.1%; OR 1.9, 95% CI 1.2-3.2; p = .011) and self-reported ST abstinence (50.7% vs. 34.3%; OR 2.0, 95% CI 1.2-3.2; p = .013) compared with placebo at the end of treatment (Week 12). Following target quit date (TQD), nicotine withdrawal symptoms decreased significantly with time (time effect = -.022 per day, SE = .003; p < .001) and was significantly lower for the active lozenge (treatment effect = -.213, SE = .071; p = .003). Tobacco craving also decreased significantly following TQD (time effect = -.071, SE = .006; p < .001) and was lower for the active nicotine lozenge (treatment effect = -.452, SE = .164; p = .006).DISCUSSIONThe 4-mg nicotine lozenge increased self-reported but not biochemically confirmed tobacco abstinence rates at 3 months. The use of the 4-mg nicotine lozenge is associated with decreased nicotine withdrawal symptoms and tobacco craving. |
| 1194 | Kralikova, Eva, Kozak, Jiri T, Rasmussen, Thomas, Gustavsson, Gunnar, Le Houezec, Jacques. Smoking cessation or reduction with nicotine replacement therapy: a placebo-controlled double blind trial with nicotine gum and inhaler. BMC public health. 2009;9:433.  **Abstract:** BACKGROUNDEven with effective smoking cessation medications, many smokers are unable to abruptly stop using tobacco. This finding has increased interest in smoking reduction as an interim step towards complete cessation.METHODSThis multi-center, double-blind placebo-controlled study evaluated the efficacy and safety of nicotine 4 mg gum or nicotine 10 mg inhaler in helping smokers (N = 314) to reduce or quit smoking. It included smokers willing to control their smoking, and participants could set individual goals, to reduce or quit. The study was placebo-controlled, randomized in a ratio of 2:1 (Active:Placebo), and subjects could choose inhaler or gum after randomization. Outcome was short-term (from Week 6 to Month 4) and long-term (from Month 6 to Month 12) abstinence or reduction. Abstinence was defined as not a single cigarette smoked and expired CO readings of <10 ppm. Smoking reduction was defined as a reduction in number of cigarettes per day by 50% or more versus baseline, verified by a lower-than-baseline CO reading at each visit during the same periods.RESULTSSignificantly more smokers managed to quit in the Active group than in the Placebo group. Sustained abstinence rates at 4 months were 42/209 (20.1%) subjects in the Active group and 9/105 (8.6%) subjects in the Placebo group (p = 0.009). Sustained abstinence rates at 12 months were 39/209 (18.7%) and 9/105 (8.6%), respectively (p = 0.019). Smoking reduction did not differ between the groups, either at short-term or long-term. Twelve-month reduction results were 17.2% vs. 18.1%, respectively. No serious adverse events were reported.CONCLUSIONIn conclusion, treatment with 10 mg nicotine inhaler or 4 mg nicotine chewing gum resulted in a significantly higher abstinence rate than placebo. In addition a large number of smokers managed to reduce their cigarette consumption by more than 50% compared to baseline. |
| 1196 | Piper, Megan E, Smith, Stevens S, Schlam, Tanya R, Fiore, Michael C, Jorenby, Douglas E, Fraser, David, Baker, Timothy B. A randomized placebo-controlled clinical trial of 5 smoking cessation pharmacotherapies. Archives of general psychiatry. 2009;66:1253.  **Abstract:** CONTEXTLittle direct evidence exists on the relative efficacies of different smoking cessation pharmacotherapies, yet such evidence is needed to make informed decisions about their clinical use.OBJECTIVETo assess the relative efficacies of 5 smoking cessation pharmacotherapy interventions using placebo-controlled, head-to-head comparisons.DESIGNA randomized, double-blind, placebo-controlled clinical trial.SETTINGTwo urban research sites.PATIENTSOne thousand five hundred four adults who smoked at least 10 cigarettes per day during the past 6 months and reported being motivated to quit smoking. Participants were excluded if they reported using any form of tobacco other than cigarettes; current use of bupropion; having a current psychosis or schizophrenia diagnosis; or having medical contraindications for any of the study medications.INTERVENTIONSParticipants were randomized to 1 of 6 treatment conditions: nicotine lozenge, nicotine patch, sustained-release bupropion, nicotine patch plus nicotine lozenge, bupropion plus nicotine lozenge, or placebo. In addition, all participants received 6 individual counseling sessions.MAIN OUTCOME MEASURESBiochemically confirmed 7-day point-prevalence abstinence assessed at 1 week after the quit date (postquit), end of treatment (8 weeks postquit), and 6 months postquit. Other outcomes were initial cessation, number of days to lapse, number of days to relapse, and latency to relapse after the first lapse.RESULTSAll pharmacotherapies differed from placebo when examined without protection for multiple comparisons (odds ratios, 1.63-2.34). With such protection, only the nicotine patch plus nicotine lozenge (odds ratio, 2.34, P < .001) produced significantly higher abstinence rates at 6-month postquit than did placebo.CONCLUSIONWhile the nicotine lozenge, bupropion, and bupropion plus lozenge produced effects that were comparable with those reported in previous research, the nicotine patch plus lozenge produced the greatest benefit relative to placebo for smoking cessation. |
| 1206 | Ferguson, Stuart G, Gitchell, Joseph G, Shiffman, Saul, Sembower, Mark A. Prediction of abstinence at 10 weeks based on smoking status at 2 weeks during a quit attempt: secondary analysis of two parallel, 10-week, randomized, double-blind, placebo-controlled clinical trials of 21-mg nicotine patch in adult smokers. Clinical therapeutics. 2009;31:1957.  **Abstract:** BACKGROUNDSmoking cessation outcomes are calculated as the probability of abstinence at follow-up among all enrolled smokers, but it is also useful to estimate the probability of success for those who experienced success or failure at earlier time points.OBJECTIVESThe primary aims were to estimate the probability of maintaining abstinence through week 10 among subjects who were abstinent at week 2, and to assess the effect of active treatment with a nicotine patch. We also examined outcomes at week 6 and, among subjects who smoked during the first 2 weeks of treatment, the probability of reestablishing abstinence later, as well as the effect of active treatment on this outcome.METHODSWe analyzed pooled data from 2 previously published, parallel, double-blind studies in which subjects were initially randomized to receive an active nicotine patch (starting at 21 mg) or a placebo patch. Subjects randomized to active treatment followed a double-blind step-down dosing regimen: 24-h/21-mg patches for the first 6 weeks of treatment, followed by 14- and 7-mg patches for successive 2-week periods. Biochemically verified abstinence (exhaled carbon monoxide <or=8 ppm) was assessed during laboratory visits.RESULTSMost subjects were white (94.8%) and female (61.6%), with a mean (SD) age of 43.1 (10.2) years. Subjects had been smoking for a mean of 24.5 (10.2) years and smoked a mean of 30.6 (10.4) cigarettes per day. In both the nicotine (n = 249) and placebo (n = 253) groups, all subjects who were abstinent during the first 2 weeks had a high probability of maintaining abstinence through week 10, but subjects treated with the active patch were significantly more likely to remain abstinent (active: 79.8% [67/84], placebo: 52.6% [20/38]; relative risk [RR] = 1.52 [95% CI, 1.10-2.09]). Also, at week 6, subjects receiving active treatment had a greater chance of remaining abstinent (active: 94.3% [82/87], placebo: 78.0% [32/41]; RR = 1.21 [95% CI, 1.02-1.43]). To assess the effect of treatment on recovery from smoking lapses, we examined the probability of abstinence during week 10 among subjects who smoked during the first 2 weeks of treatment. Among them, treatment was associated with a greater probability of later success: 31.4% (50/159) of those treated with the active patch and 12.5% (26/208) of those receiving placebo were abstinent at week 10 (RR = 2.52 [95% CI, 1.64-3.85]). Similar results were observed at week 6 (49.4% [80/162] vs 21.2% [45/212]; RR = 2.33 [95% CI, 1.72-3.15]).CONCLUSIONSMore than two thirds (71.3%) of subjects who were abstinent 2 weeks into a quit attempt maintained that abstinence through the end of 10 weeks of treatment. Use of a nicotine patch was significantly associated with maintaining abstinence and with recovering abstinence after an early lapse. |
| 1211 | Adamopoulos, Dionysios, Argacha, Jean-François, Gujic, Marko, Preumont, Nicolas, Degaute, Jean-Paul, van de Borne, Philippe. Acute effects of nicotine on arterial stiffness and wave reflection in healthy young non-smokers. Clinical and experimental pharmacology & physiology. 2009;36:784.  **Abstract:** 1. Recently, we have demonstrated that cigarette smoke exposure proportionally increases plasma nicotine levels and arterial wave reflection to the aorta. However, the exact contribution of nicotine to the smoke-induced enhancement of wave reflection and the potential underlying mechanisms have not been fully investigated. 2. The present study was a prospective study in 15 healthy male non-smokers. All received a placebo and a 2 mg nicotine tablet, according to a randomized double-blind cross-over study design. Each subject underwent repeated measurements at baseline and for 1 h after nicotine or placebo intake, using carotid-femoral pulse wave velocity (PWV) to assess arterial compliance. Concurrently, aortic pressures and the augmentation index were evaluated using applanation tonometry. 3. Plasma nicotine concentrations achieved 1 h after intake of the nicotine tablet reached comparable levels to those achieved after 1 h exposure to passive smoke (3.6 +/- 0.4 vs 3.2 +/- 0.4 ng/mL, respectively; P = 0.4). 4. Nicotine enhanced arterial wave reflection to the aorta, as assessed by the augmentation index corrected for heart rate (4.2 +/- 1.3 vs-0.7 +/- 0.8% with placebo; P = 0.001). In addition, a progressive increase in carotid-femoral PWV was noted after nicotine administration (0.3 +/- 0.1 vs-0.02 +/- 0.1 m/s with placebo; P = 0.04). This remained significant even after adjustment for changes in mean blood pressure and heart rate (P = 0.01). 5. Plasma nicotine concentrations comparable to those achieved after exposure to passive smoke enhance arterial wave reflection to the aorta. This is accompanied by an increase in carotid-femoral PWV, denoting a deterioration of arterial compliance by nicotine. |
| 1220 | Hall, Sharon M, Humfleet, Gary L, Muñoz, Ricardo F, Reus, Victor I, Robbins, Julie A, Prochaska, Judith J. Extended treatment of older cigarette smokers. Addiction (Abingdon, England). 2009;104:1043.  **Abstract:** AIMSTobacco dependence treatments achieve abstinence rates of 25-30% at 1 year. Low rates may reflect failure to conceptualize tobacco dependence as a chronic disorder. The aims of the present study were to determine the efficacy of extended cognitive behavioral and pharmacological interventions in smokers > or = 50 years of age, and to determine if gender differences in efficacy existed.DESIGNOpen randomized clinical trial.SETTINGA free-standing, smoking treatment research clinic.PARTICIPANTSA total of 402 smokers of > or = 10 cigarettes per day, all 50 years of age or older.INTERVENTIONParticipants completed a 12-week treatment that included group counseling, nicotine replacement therapy (NRT) and bupropion. Participants, independent of smoking status, were then assigned randomly to follow-up conditions: (i) standard treatment (ST; no further treatment); (ii) extended NRT (E-NRT; 40 weeks of nicotine gum availability); (iii) extended cognitive behavioral therapy (E-CBT; 11 cognitive behavioral sessions over a 40-week period); or (iv) E-CBT plus E-NRT (E-combined; 11 cognitive behavioral sessions plus 40 weeks nicotine gum availability).MEASUREMENTSPrimary outcome variable was 7-day point prevalence cigarette abstinence verified biochemically at weeks 24, 52, 64 and 104.FINDINGSThe most clinically important findings were significant main effects for treatment condition, time and the treatment x time interaction. The E-CBT condition produced high cigarette abstinence rates that were maintained throughout the 2-year study period [(week 24 (58%), 52 (55%), 64 (55%) and 104 (55%)], and was significantly more effective than E-NRT and ST across that period. No other treatment condition was significantly different to ST. No effects for gender were found.CONCLUSIONSExtended cognitive behavioral treatments can produce high and stable cigarette abstinence rates for both men and women. NRT does not add to the efficacy of extended CBT, and may hamper its efficacy. Research is needed to determine if these results can be replicated in a sample with a greater range of ages, and improved upon with the addition of medications other than NRT. |
| 1226 | Elkader, Alexander K, Brands, Bruna, Selby, Peter, Sproule, Beth A. Methadone-nicotine interactions in methadone maintenance treatment patients. Journal of clinical psychopharmacology. 2009;29:231.  **Abstract:** Smoking is highly prevalent (85%-98%) in methadone maintenance treatment (MMT) patients. Methadone has been shown to increase cigarette smoking in a dose-dependent manner, whereas smoking/nicotine has been shown to increase methadone self-administration and reinforcing properties. The objective of this study was to evaluate methadone-nicotine interactions in MMT patients during trough and peak methadone effect conditions. Subjective effects of nicotine (administered by cigarette smoking, 4 mg of nicotine gum and placebo gum) and methadone and their combination were assessed in 40 regularly smoking, stabilized MMT patients using a randomized, placebo-controlled, within-subject study design. Subjects responded to a battery of subjective assessments before and after nicotine administration both before methadone administration (cycles 1 and 2) and 3 hours after methadone administration (cycles 3 and 4). There was a main effect of methadone on the decrease of opioid withdrawal scores (P < 0.001), and cigarette smoking enhanced this effect (day x methadone interaction, P = 0.031). Both nicotine and methadone had main effects on the decrease of nicotine withdrawal scores (P < 0.001 and P = 0.001, respectively); this was associated with the cigarette day (day x nicotine interaction, P = 0.003, and day x methadone interaction, P = 0.004). Nicotine plasma levels were highest on the cigarette smoking day (P < 0.001). Methadone and nicotine shared main effects on the increase of ratings of euphoria and drug liking and on the decrease of restlessness, irritability, and depression. The overall results may help to explain high smoking rates in the MMT population and may account for reports of increased positive effects of methadone when the drugs are taken together. |
| 1227 | Thornley, Simon, McRobbie, Hayden, Lin, Ruey-Bin, Bullen, Chris, Hajek, Peter, Laugesen, Murray, Senior, Hugh, Whittaker, Robyn. A single-blind, randomized, crossover trial of the effects of a nicotine pouch on the relief of tobacco withdrawal symptoms and user satisfaction. Nicotine & tobacco research : official journal of the Society for Research on Nicotine and Tobacco. 2009;11:715.  **Abstract:** INTRODUCTIONWe compared the effects of a 4-mg oral nicotine pouch (Zonnic pouch), with nicotine chewing gum and placebo pouch, on withdrawal discomfort after overnight tobacco abstinence. We also assessed participants' preferences, satisfaction, and consumption patterns.METHODSThis was a randomized, placebo-controlled, three-way crossover study of 30 adult smokers. After overnight tobacco abstinence, subjects reported on a Visual Analog Scale (VAS; 0-100) tobacco withdrawal symptoms (craving, irritability, difficulty concentrating, and restlessness) before use and during the first hour after first product use. They then used the product throughout the study day and in the evening reported product usefulness, temporary abstinence success, and satisfaction.RESULTSIn a multivariate analysis, area under the curve (craving vs. time) was reduced by 23 points 60 min after taking the study medication in the nicotine pouch group, compared with 15- and 8-point decreases in the gum and placebo groups, respectively. The difference in craving ratings between the pouch and placebo was significant (p = .002). Nicotine pouch reduced irritability more than gum (difference = 9.86; p = .01). For pouch users, the odds ratio for temporary tobacco abstinence (21.5 hr) during study days (compared with gum) was 2.8 (95% CI = 0.8-8.1). Compared with the gum, the pouch was rated as significantly more "helpful to stop smoking" (difference = 20.6; 95% CI = 2.4-38.9) and "pleasant to use" (difference = 17.3; 95% CI = 2.6-32.0).DISCUSSIONThe Zonnic pouch appears to be as effective at relieving craving as nicotine gum and was subjectively favored over the gum. These results suggest that the pouch will be a helpful addition to the range of existing nicotine replacement treatments. |
| 1229 | Gilbert, David G, Zuo, Yantao, Rabinovich, Norka E, Riise, Hege, Needham, Rachel, Huggenvik, Jodi I. Neurotransmission-related genetic polymorphisms, negative affectivity traits, and gender predict tobacco abstinence symptoms across 44 days with and without nicotine patch. Journal of abnormal psychology. 2009;118:322.  **Abstract:** Genetic and personality trait moderators of tobacco abstinence-symptom trajectories were assessed in a highly controlled study. Based on evidence suggesting their importance in stress reactivity and smoking, moderators studied were serotonin transporter gene (5-HTTLPR) and dopamine D2 receptor gene (DRD2) polymorphisms and personality traits related to negative affect (NA). Smokers were randomly assigned to quit smoking with nicotine or placebo patches. Financial incentives resulted in 80% verified abstinence across the 44-day study. Individuals with 1 or 2 short alleles of 5-HTTLPR (S carriers) experienced larger increases in NA symptoms than did those without a short allele. Nicotine replacement therapy (NRT) alleviated anxiety only in S carriers. NRT reduced NA to a greater extent in DRD2 A1 carriers than in A2A2 individuals during the 1st 2 weeks of treatment (when on the 21-mg patch); however, A1 carriers experienced a renewal of NA symptoms when switched to the 7-mg patch and when off the patch, while A2A2 individuals continued to benefit from NRT. The results suggest that the effects of genotype and treatment may vary across different durations of abstinence, treatment doses, and genotypes. |
| 1231 | Knott, Verner J, Bolton, Kiley, Heenan, Adam, Shah, Dhrasti, Fisher, Derek J, Villeneuve, Crystal. Effects of acute nicotine on event-related potential and performance indices of auditory distraction in nonsmokers. Nicotine & tobacco research : official journal of the Society for Research on Nicotine and Tobacco. 2009;11:519.  **Abstract:** INTRODUCTIONAlthough nicotine has been purported to enhance attentional processes, this has been evidenced mostly in tasks of sustained attention, and its effects on selective attention and attentional control under conditions of distraction are less convincing.METHODSThis study investigated the effects of nicotine on distractibility in 21 (11 males) nonsmokers with event-related potentials (ERPs) and behavioral performance measures extracted from an auditory discrimination task requiring a choice reaction time response to short- and long-duration tones, with and without imbedded deviants. Administered in a randomized, double-blind, placebo-controlled crossover design, nicotine gum (6 mg) failed to counter deviant-elicited behavioral distraction characterized by longer reaction times and increased response errors.RESULTSOf the deviant-elicited ERP components, nicotine did not alter the P3a-indexed attentional switching to the deviant, but in females, it tended to diminish the automatic processing of the deviant as shown by a smaller mismatch negativity component, and it attenuated attentional reorienting following deviant-elicited distraction, as reflected by a reduced reorienting negativity ERP component.DISCUSSIONResults are discussed in relation to attentional models of nicotine and with respect to future research directions. |
| 1232 | Perkins, Kenneth A, Grottenthaler, Amy, Ciccocioppo, Melinda M, Conklin, Cynthia A, Sayette, Michael A, Wilson, Annette S. Mood, nicotine, and dose expectancy effects on acute responses to nicotine spray. Nicotine & tobacco research : official journal of the Society for Research on Nicotine and Tobacco. 2009;11:540.  **Abstract:** INTRODUCTIONWe recently showed effects of nicotine dose and nicotine expectancy on some responses to cigarette smoking, with generally no influence of induced mood on these effects. The present study extended this line of research to Nicotrol nasal spray, to determine whether formulation (spray vs. smoking) alters responses.METHODSSmokers abstained overnight before each of two virtually identical sessions, involving negative or positive mood induction. They were randomized to one of five groups, four comprising the 2 x 2 balanced placebo design, varying actual and expected dose of nicotine in the nasal spray, and the fifth group a no-spray control. Dependent measures included self-reported affect, craving, withdrawal, and spray ratings of "liking" and "how much nicotine." Analyses were limited to those whose nicotine expectancies were manipulated successfully (N = 48).RESULTSThe following results matched those from our smoking study: expecting nicotine increased liking; expected, but not actual, nicotine dose increased dose perception; neither actual nor expected nicotine dose had much influence on affect or withdrawal; and mood had no influence on these effects. However, both actual and expected nicotine dose decreased craving in response to spray, contrary to our prior study with smoking.DISCUSSIONFormulation made little difference in some effects of nicotine and expectancies, but other effects differed by formulation. Some of these findings, particularly for craving reduction, may have implications for enhancing the acute therapeutic effects of nasal spray and, perhaps, other medications in smokers trying to maintain abstinence after quitting. |
| 1233 | Sørensen, Lars Tue, Zillmer, Rikke, Agren, Magnus, Ladelund, Steen, Karlsmark, Tonny, Gottrup, Finn. Effect of smoking, abstention, and nicotine patch on epidermal healing and collagenase in skin transudate. Wound repair and regeneration : official publication of the Wound Healing Society [and] the European Tissue Repair Society. 2009;17:347.  **Abstract:** Delayed wound healing may explain postoperative tissue and wound dehiscence in smokers, but the effects of smoking and smoking cessation on the cellular mechanisms remain unclear. Suction blisters were raised in 48 smokers and 30 never smokers. The fluid was retrieved and the epidermal roof was excised. Transepidermal water loss (TEWL) was measured after 2, 4, and 7 days. Then, the smokers were randomized to continuous smoking or abstinence with a transdermal nicotine patch or a placebo by concealed allocation. The sequence was repeated after 4, 8, and 12 weeks in all smokers and abstainers and in 6 never smokers. Matrix metalloproteinase (MMP)-8 and MMP-1 levels in suction blister fluid were assessed by an enzyme-linked immunosorbent assay. Random-effects models for repeated measurements were applied and p< or =0.05 was considered significant. One week after wounding the TEWL was 17.20 (14.47-19.92) g/cm(2) hour (mean, 95% CI) in smokers and 13.89 (9.46-18.33) in never smokers (p<0.01). In abstinent smokers TEWL was 18.95 (15.20-22.70)(p<0.01, when compared with smokers). In smokers, MMP-8 was 36.4 (24.3-48.5) ng/mL (mean, 95% CI) and 15.2 (1.4-30.2) ng/mL in never smokers (p<0.01). Abstinent smokers' MMP-8 level was 21.2 ng/mL (6.6-43.0) (p=0.02, when compared with smokers). MMP-1 was unaffected by smoking and abstention. Transdermal nicotine patch did not affect any parameter. We conclude that smoking attenuates epidermal healing and may enhance extracellular matrix degradation. Three months of abstinence from smoking does not restore epidermal healing, whereas 4 weeks of abstinence normalizes suction blister MMP-8 levels. These findings suggest sustained impaired wound healing in smokers and potential reversibility of extracellular matrix degradation. |
| 1240 | Davenport, Paul W, Vovk, Andrea, Duke, Rita K, Bolser, Donald C, Robertson, Erin. The urge-to-cough and cough motor response modulation by the central effects of nicotine. Pulmonary pharmacology & therapeutics. 2009;22:82.  **Abstract:** The urge-to-cough is a respiratory sensation that precedes the cough motor response. Since affective state modulates the perception of respiratory sensations such as dyspnoea, we wanted to test whether nicotine, an anxiolytic, would modulate the urge-to-cough and hence, the cough motor response. We hypothesized that withdrawal from and administration of nicotine in smoking subjects would modulate their anxiety levels, urge-to-cough and cough motor response to capsaicin stimulation. Twenty smoking (SM) adults (8F, 12M; 22+/-3 years; 2.9+/-2.0 pack years) and matched non-smoking (NS) controls (22+/-2 years) were presented with randomized concentrations of capsaicin (0-200 microM) before and after nicotine (SM only) gum and/or placebo (SM and NS) gum. Subjects rated their urge-to-cough using a Borg scale at the end of each capsaicin presentation. Cough number and cough motor pattern were determined from airflow tracings. Subjects completed State-Trait Anxiety Inventory (STAI) questionnaires before and after gum administration. SM subjects that withdrew from cigarette smoking for 12 h exhibited an increase in anxiety scores, a greater number of coughs and higher urge-to-cough ratings compared to NS subjects. Administration of nicotine gum reduced anxiety scores, cough number and urge-to-cough ratings to match the NS subjects. There was no effect of placebo gum on any of the measured parameters in the SM and NS groups. The results from this study suggest that modulation of the central neural state with nicotine withdrawal and administration in young smoking adults is associated with a change in anxiety levels which in turn modulates the perceptual and motor response to irritant cough stimulants. |
| 1242 | Newhouse, Paul, Levin, Edward, Kellar, Kenneth, Coderre, Emily, Wilkins, Heather, Aisen, Paul, White, Heidi, Wesnes, Keith. Transdermal nicotine treatment of Mild Cognitive Impairment (MCI): A multi-center pilot study. American Journal of Geriatric Psychiatry. 2009;17:A102.  **Abstract:** Background: Interest in nicotinic treatment of Alzheimer's disease (AD) developed following recognition of the loss of nicotinic receptors and that short-term administration of nicotine improved cognitive performance in AD. However, Mild Cognitive Impairment (MCI) may also be an appropriate target for nicotinic treatment. We initiated a 1 year multicenter pilot trial and hypothesized that nicotine treatment would produce improvements in cognitive performance and clinical ratings while being well tolerated. Methods: 74 non-smoking subjects from 3 sites who met criteria for amnestic MCI were randomized to receive either double-blind transdermal nicotine or placebo patch for the first 6 months of the study (and open label nicotine patch for the second 6 months of the study, not reported here). Subjects randomized to nicotine had their dose blindly titrated from 5 mg to 15 mg per day over 3 weeks. For the blind phase, subjects were cognitively assessed at baseline, 91, and 182 days utilizing computerized assessment (Cognitive Drug Research battery), Conners continuous performance task (CPT), non-computer verbal and visual tasks, and clinical global impression of change (CGIC). Results: For cognitive performance, preliminary analysis showed that nicotine treatment produced significant (p<.05) improvements in CPT accuracy and reaction time standard error and delayed word recall accuracy, speed of memory, and choice reaction time accuracy from the CDR battery. Several other cognitive measures including quality of episodic memory and digit symbol substitution task showed strong trends (p<.1) in favor of nicotine treatment. Immediate recall on the paragraph recall task favored placebo. The CGIC results showed that a greater proportion of patients on nicotine were rated as improved compared to the placebo group (23% nicotine vs. 9% placebo) (p = 0.15). Subject self-ratings of cognitive impairment and alertness showed a strong trend (p<.07) in favor of nicotine. Analysis by APOE status showed evidence for greater positive nicotine effects in E4- vs E4+ subjects in CGI ratings and performance measures. Adverse events were mild and evenly split between nicotine and placebo treatment. Predictably, weight showed a small but significant decline on nicotine (p = 0.02) within the first 3 months and nicotine significantly (p = .02) reduced systolic blood pressure compared to placebo. Conclusions: Transdermal nicotine treatment of MCI showed significant improvements in measures of attention, memory and speed with several other measures also showing strong trends towards improvement. The CGIC scores favored nicotine. Treatment with nicotine in this nonsmoking older population was well tolerated and did not produce clinically meaningful worsening of vital signs. We conclude that transdermal nicotine treatment has significant promise as a symptomatic treatment for amnestic MCI and deserves further exploration in a larger trial. Supported by NIA R01 AG022462. |
| 1243 | Sadr Azodi, O, Lindström, D, Adami, J, Tønnesen, H, Nåsell, H, Gilljam, H, Wladis, A. The efficacy of a smoking cessation programme in patients undergoing elective surgery: a randomised clinical trial. Anaesthesia. 2009;64:259.  **Abstract:** It is known that smokers constitute an important risk group of patients undergoing surgery. It is unknown how smoking cessation intervention initiated 4 weeks prior to elective surgery affects the probability of permanent cessation. We randomly assigned 117 patients, scheduled to undergo elective orthopaedic and general surgery, to smoking cessation intervention and control group. The intervention group underwent a programme initiated, on average, 4 weeks prior to surgery with weekly meetings or telephone counselling and were provided with free nicotine replacement therapy (NRT). The control group received standard care. As a result, 20/55 (36%) patients the intervention group vs 1/62 (2%) in the control group became completely abstinent throughout the peri-operative period (p < 0.001). After 1 year, those in the intervention group was most likely to be abstinent (18/55 (33%) vs 9/62 (15%) of the controls (p = 0.03). Level of nicotine dependence and obesity seemed to be a predictor of long-term abstinence (p = 0.02). |
| 1244 | Salize, Hans Joachim, Merkel, Silke, Reinhard, Iris, Twardella, Dorothee, Mann, Karl, Brenner, Hermann. Cost-effective primary care-based strategies to improve smoking cessation: more value for money. Archives of internal medicine. 2009;169:230.  **Abstract:** BACKGROUNDEvidence from cost-effective smoking cessation programs is scarce. This study determined the cost-effectiveness of 3 smoking cessation strategies as provided by general practitioners (GPs) in Germany.METHODSIn a cluster-randomized smoking cessation trial, rates and intervention costs for 577 smoking patients of 82 GPs were followed up for 12 months. Three smoking cessation treatments were tested: (1) GP training plus GP remuneration for each abstinent patient, (2) GP training plus cost-free nicotine replacement medication and/or bupropion hydrochloride for the patient, and (3) a combination of both strategies. Smoking abstinence at 12 months was the primary outcome used to calculate incremental cost-effectiveness ratios and net monetary benefits.RESULTSIntervention 1 was not effective compared with treatment as usual (TAU). Interventions 2 and 3 each proved to be cost-effective compared separately with TAU. When applying a 95% level of certainty of cost-effectiveness against TAU, euro 9.80 or euro 6.96, respectively, had to be paid for each additional 1% of patients abstinent at 12 months (maximum willingness to pay). That means that in intervention 2, euro 92.12 per patient in the program must be invested to gain 1 additional quitter (as opposed to euro 39.10 paid per patient during the trial). In intervention 2, the cost was euro 82.82, as opposed to euro 50.04. Neither of these 2 cost-effective treatments proved to be superior to the other. The cost-effectiveness of both treatments was stable against TAU in sensitivity analyses. (The exchange rate from October 1, 2003, was used; euro1 = .17.)CONCLUSIONSBoth treatments have a high potential to reduce smoking-related morbidity at a low cost. It is highly recommended that they be implemented as a routine service offered by GPs because in many countries, health insurance plans currently do not fund nicotine replacement therapy. |
| 1246 | Rigotti, Nancy A, Gonzales, David, Dale, Lowell C, Lawrence, Daniel, Chang, Yuchiao. A randomized controlled trial of adding the nicotine patch to rimonabant for smoking cessation: efficacy, safety and weight gain. Addiction (Abingdon, England). 2009;104:266.  **Abstract:** AIMSBecause smoking cessation rates might be improved by combining drugs and by reducing post-cessation weight gain, we tested the smoking cessation efficacy, safety and effect on body weight of adding the nicotine patch to rimonabant, a cannabanoid type-1 receptor antagonist that reduces body weight.DESIGNRandomized double-blind placebo-controlled trial.SETTINGFifteen US research centers.PARTICIPANTSA total of 755 smokers (> OR = 15 cigarettes/day). Intervention Rimonabant (20 mg daily) was given open-label for 9 weeks. The 735 participants completing week 1 were randomized at day 8 (target quit day) to add a nicotine patch (n = 369) or placebo patch (n = 366) for 10 weeks (21 mg daily for 8 weeks plus a 2-week taper). Participants received weekly smoking counseling and were followed for 24 weeks.MEASUREMENTSBiochemically validated 4-week continuous abstinence at end-of-treatment (weeks 6-9; primary end-point); 7-day point prevalence abstinence at weeks 9 and 24; sustained abstinence (weeks 6-24); change in body weight; and adverse events.FINDINGSRimonabant plus nicotine patch was superior to rimonabant plus placebo in validated continuous abstinence at weeks 6-9 (39.0% versus 21.3%; odds ratio 2.36, 95% confidence interval: 1.71-2.37; P < 0.01) and in all other efficacy measures. Mean end-of-treatment weight gain among quitters did not differ between groups (0.04 kg for combination versus 0.49 kg for rimonabant only, P = 0.15) and was similar in weight-concerned smokers. Serious adverse event rates did not differ between groups. Depression- and anxiety-related adverse events occurred in 32 (4.2%) and 44 (5.8%) subjects, respectively; eight (1.1%) and nine (1.2%) subjects stopped the drug due to depression and anxiety, respectively.CONCLUSIONSAdding a nicotine patch to rimonabant was well tolerated and increased smoking cessation rates over rimonabant alone. There was little post-cessation weight gain in either group, even among weight-concerned smokers, during drug treatment. |
| 1247 | Shiffman, Saul, Ferguson, Stuart G, Strahs, Kenneth R. Quitting by gradual smoking reduction using nicotine gum: a randomized controlled trial. American journal of preventive medicine. 2009;36:96.  **Abstract:** BACKGROUNDMany smokers express a desire to quit smoking by gradually reducing the number of cigarettes they smoke until they stop completely. This study tested the efficacy of nicotine gum in facilitating cessation through gradual reduction.DESIGNThis was a multi-center, placebo-controlled, double-blind RCT of 2- and 4-mg nicotine gum versus placebo.SETTING/PARTICIPANTS3297 smokers who were interested in quitting gradually.INTERVENTIONSubjects were instructed to gradually reduce their smoking while increasing their gum use over the course of up to 8 weeks. Once they had achieved initial abstinence (no smoking for 24 hours), gum was to be used in accordance with the current FDA-approved directions for cessation. The study was conducted under over-the-counter conditions, with no counseling provided. Continuous abstinence was assessed after 28 days and 6 months. Secondary measures of smoking reduction were also assessed. Analyses were conducted in 1999-2000 and 2007-2008.MAIN OUTCOME MEASURESSmokers on active gum were significantly more likely to achieve initial cessation (2 mg: OR=1.42; 4 mg: OR=1.90); 28-day continuous abstinence (2 mg: OR=2.01; 4 mg: OR=4.66); and continuous abstinence at 6 months (2 mg: OR=1.80; 4 mg: OR=5.96). During the reduction phase, active gum aided smoking reduction, and participants who reduced their smoking were more likely to achieve abstinence.CONCLUSIONSThese findings demonstrate that smokers who wish to quit smoking by gradual reduction can increase their success by using nicotine gum to facilitate reduction and cessation. |
| 1252 | Cosci, Fiammetta, Corlando, Annalisa, Fornai, Edo, Pistelli, Francesco, Paoletti, Paolo, Carrozzi, Laura. Nicotine dependence, psychological distress and personality traits as possible predictors of smoking cessation. Results of a double-blind study with nicotine patch. Addictive behaviors. 2009;34:28.  **Abstract:** AIMNicotine replacement therapy (NRT) is an effective treatment for smokers who want to quit, however, the rates of successful quitting can be improved even more. In this context, nicotine dependence (assessed via the Fagerström Tolerance Questionnaire, FTQ), psychological distress (measured via the Symptom Rating Test, SRT), and personality traits (evaluated via the Adult Eysenck Personality Inventory, AEPI) were evaluated as possible predictors of smoking cessation.RESULTSA total of 297 cigarette smokers were followed for one year as part of a NRT double-blind, parallel group, randomized trial. Baseline nicotine dependence (weeks 12 and 26: p<0.05), AEPI neuroticism (weeks 12 and 52: p<0.05), and AEPI psychoticism (weeks 12 and 52: p<0.05) scores significantly influenced the outcome of smoking cessation during one-year of follow-up. An increase in psychological distress during follow-up was associated with a lower probability of quitting smoking (p=0.000).CONCLUSIONSNicotine dependence, neuroticism, psychoticism and, over time, psychological distress were the main factors influencing the long-term outcome (i.e., up to 12 months) of smoking cessation under NRT. |
| 1253 | Sun, Hong-Qiang, Guo, Song, Chen, Da-Fang, Jiang, Zuo-Ning, Liu, Yu, Di, Xiao-Lan, Yang, Fu-De, Zhang, Xiang-Yang, Kosten, Thomas R, Lu, Lin. Family support and employment as predictors of smoking cessation success: a randomized, double-blind, placebo-controlled trial of nicotine sublingual tablets in chinese smokers. The American journal of drug and alcohol abuse. 2009;35:183.  **Abstract:** BACKGROUNDThe purpose of this study is to assess social support and demographic factors that influence the success of smoking cessation aided with sublingual nicotine tablets in a Han Chinese population.METHODSWe randomly allocated 211 Beijing residents who smoked >or= 10 cigarettes a day for at least 1 year into a double-blind, placebo-controlled 3-month randomized smoking cessation trial using sublingual nicotine replacement therapy (NRT). Self-reports of sustained smoking cessation were verified during the study by expired carbon monoxide concentrations and urine-cotinine concentrations. Logistic regression analysis used an intent to treat sample for sociodemographic associations with abstinence and reduction in smoking.RESULTSThe abstinence rates at the end of treatment for NRT vs. placebo were 52 % vs .19%, and smoking reduction (reduced to at least 50% of baseline) rates for NRT vs. placebo were 43% vs .15% for a total response rate with NRT of 95% for either stopping completely or reducing smoking by 50%. The only factor strongly associated with successful smoking cessation after 3 months of sublingual NRT was being married (adjusted odds ratio 2.18; 95%confidence interval 1.10-4.33). Smoking association, on the other hand, was associated with being married and with employment as a white collar worker (2.24; 1.03 to 4.86).CONCLUSIONSThese findings suggest the need for a more in-depth examination of the impact of being married and employment as a white collar worker (rather than manual laborer) in order to develop better targeted interventions for improving smoking cessation interventions. |
| 1255 | Martins, Luíz Cláudio, Ferreira-Melo, Sílvia Elaine, Sabha, Maricene, Coelho, Otávio Rizzi, Yugar-Toledo, Juan Carlos, Quinaglia, Thiago, Moreira, Marcos Mello, Coca, Antonio, Moreno Júnior, Heitor. Acute effects of pharmacotherapies in blood pressure in normotensive moderate smokers. Blood pressure. 2009;18:255.  **Abstract:** This study was designed to evaluate the changes in arterial blood pressure (BP) and heart rate (HR) in moderate smokers during smoking abstinence after 7 days of treatment with bupropion alone, transdermal nicotine or bupropion combined with transdermal nicotine. Twenty-four healthy moderate smokers (12 female/12 male; 40+/-7 years) were evaluated randomly on five occasions and their systolic, diastolic, mean arterial blood pressure (MAP) and HR were measured by a Finapres device for 10 h, immediately after smoking interruption. All of the 24 smokers participated on five protocols during 7 days: control group (C) - no drugs; placebo group (PL); bupropion group (BUP) 150-300 mg; transdermal nicotine group (TN) - 21 mg; and BUP+TN-nicotine patch. Concomitantly, the subjects were evaluated by ABPM (ambulatory BP monitoring). All of BP parameters monitored shown significant statistical differences in the BUP, TN and BUP+TN groups compared with the controls (p<0.05), when measured by Finapres. The HR remained unaltered in all of the groups. No significant differences were seen in the BP or HR during the 24-h ABPM. These findings indicate that in moderate smokers, bupropion, transdermal nicotine or bupropion associated with transdermal nicotine caused an elevation in the BP after acute smoking interruption. |
| 1260 | Glasgow, Russell E, Gaglio, Bridget, Estabrooks, Paul A, Marcus, Alfred C, Ritzwoller, Debra P, Smith, Tammy L, Levinson, Arnold H, Sukhanova, Anna, O'Donnell, Colin, Ferro, Erica F, France, Eric K. Long-term results of a smoking reduction program. Medical care. 2009;47:115.  **Abstract:** INTRODUCTIONThere have been few comprehensive evaluations of smoking reduction, especially in health care delivery systems, and little is known about its cost, maintenance of reduced smoking, or robustness across patient subgroups.METHODSA generally representative sample of 320 adult smokers from an HMO scheduled for outpatient surgery or a diagnostic procedure was randomized to enhanced usual care or a theory-based smoking reduction intervention that combined telephone counseling and tailored newsletters. Outcomes included cigarettes smoked, carbon monoxide levels, and costs.RESULTSBoth intervention and control conditions continued to improve from 3- to 12-month assessments. Between-condition differences using intent-to-treat analyses on both self-report and carbon monoxide measures were nonsignificant by the 12-month follow-up (25% vs. 19% achieved 50% or greater reductions in cigarettes smoked). The intervention was implemented consistently despite logistical constraints and was generally robust across patient characteristics (eg, education, ethnicity, health literacy, dependence).CONCLUSIONSIn the absence of nicotine replacement therapy, the long-term effects of this smoking reduction intervention seem modest and nonsignificant. Future research is indicated to enhance intervention effects and conduct more comprehensive economic analyses of program variations. |
| 1262 | Colrain, I.M., Javitz, H.S., Baker, F.C., Krasnow, R., Swan, G.E.. The impact of smoking cessation and pharmacotherapy on sleep assessed across the first 10 days after quitting. Sleep. 2009;32:A364.  **Abstract:** Introduction: Subjective sleep disruption is a prominent symptom of smoking cessation. Polysomnography (PSG) studies tracking the period following smoking cessation are rare. We report data from a randomized, double-blind placebo controlled trial of nicotine replacement therapy (NRT) and bupropion (BP) treatment for smoking cessation with repeated assessments over the first 10 days following quitting. Methods: 27 smokers (15 men, 40.9 ± 13.9 years) underwent full night PSG on a baseline night (B) (still smoking) and the first night following quitting (Q1). 25 smokers had an additional night 3-5 days post quit (Q5) and 16 a fourth night 7-10 (Q10) days post quit. BP and NRT were administered as per current clinical practice guidelines. Data at Q1, Q5 and Q10 were analyzed using a regression model with a constant assuming no difference from B and terms for, NRT and BP, controlling for age, sex, smoking intensity and baseline Epworth Sleepiness Scale score Results: Independent of treatment, sleep efficiency (SE%) was reduced at Q1 (-4.2 ±1.6 %, p =0.016) but not at Q5 or Q10. Similarly, wakeful- ness after sleep onset and percentage of stage 1 sleep (S1%) showed significant deterioration at Q1 but not at Q5 or Q10. Number of wake periods was increased at Q1 (5.3 ± 1.6, p= 0.004) and at Q5 (3.2 ± 1.1, p= 0.007) but was no longer significantly elevated at Q10. SE% showed additional effects of BP at Q5 and NRT at Q10 with continued deterioration with active medications. S1% showed continued deterioration with active NRT at Q10. Conclusion: Smoking cessation leads to a negative impact on sleep continuity on the first night of quitting smoking with rapid recovery in the direction of baseline within 5 to 10 days post-quit. There is some evidence that active NRT and BP may prolong the sleep disturbance. |
| 1265 | Joyce, Geoffrey F, Niaura, Raymond, Maglione, Margaret, Mongoven, Jennifer, Larson-Rotter, Carrie, Coan, James, Lapin, Pauline, Morton, Sally. The effectiveness of covering smoking cessation services for medicare beneficiaries. Health services research. 2008;43:2106.  **Abstract:** OBJECTIVETo examine whether reimbursement for Provider Counseling, Pharmacotherapies, and a telephone Quitline increase smoking cessation relative to Usual Care.STUDY DESIGNRandomized comparison trial testing the effectiveness of four smoking cessation benefits.SETTINGSeven states that best represented the national population in terms of the proportion of those > or = 65 years of age and smoking rate.PARTICIPANTSThere were 7,354 seniors voluntarily enrolled in the Medicare Stop Smoking Program and they were followed-up for 12 months.INTERVENTION(S)(1) Usual Care, (2) reimbursement for Provider Counseling, (3) reimbursement for Provider Counseling with Pharmacotherapy, and (4) telephone counseling Quitline with nicotine patch.MAIN OUTCOME MEASURESeven-day self-reported cessation at 6- and 12-month follow-ups.PRINCIPAL FINDINGSUnadjusted quit rates assuming missing data=smoking were 10.2 percent (9.0-11.5), 14.1 percent (11.7-16.5), 15.8 percent (14.4-17.2), and 19.3 percent (17.4-21.2) at 12 months for the Usual Care, Provider Counseling, Provider Counseling + Pharmacotherapy, and Quitline arms, respectively. Results were robust to sociodemographics, smoking history, motivation, health status, and survey nonresponse. The additional cost per quitter (relative to Usual Care) ranged from several hundred dollars to ,450.CONCLUSIONSA telephone Quitline in conjunction with low-cost Pharmacotherapy was the most effective means of reducing smoking in the elderly. |
| 1267 | Wong, Candice C, Tsoh, Janice Y, Tong, Elisa K, Hom, Fred B, Cooper, Bruce, Chow, Edward A. The Chinese community smoking cessation project: a community sensitive intervention trial. Journal of community health. 2008;33:363.  **Abstract:** This paper describes (1) the design, methods and baseline data of the first smoking cessation clinical trial for Chinese Americans with medical conditions - Chinese Community Smoking Cessation Project (CCSCP); (2) the collaborative process between researchers and the Chinese community; and (3) the barriers and facilitators of implementing the study. CCSCP was a culturally tailored, randomized, smoking cessation trial testing the efficacy of an intensive (physician advice, in-person counseling with nicotine replacement therapy, 5 telephone calls) compared to a minimal (physician advice and self-help manual) intervention. The study applied a community-sensitive research method involving community members in all phases of the research process in San Francisco Bay Area during 2001-2007. CCSCP recruited 464 smokers from health care facilities (79%) located in Chinese neighborhoods and through Chinese language media (21%). Baseline assessments and interventions were conducted in-person using translated and tested questionnaire and intervention materials. The majority of the participants were men (91%) with a mean age of 58.3 years, foreign born (98%), with less than high school education (58%), spoke no English (42%) and in non-skilled or semi-skilled occupations (60%) with <,000 household income (51%). Participants smoked regularly on an average 38.6 +/- 17 years, smoked 9.1 +/- 8 cigarettes per day and 85% smoked daily. Cultural tailoring of recruitment methods and intervention design led to successful enrollment and retention of participants, overcoming barriers faced by the participants. Community sensitive collaborative process facilitated implementation of study protocol in community health care settings. |
| 1272 | Rungruanghiranya, Suthat, Ekpanyaskul, Chatchai, Hattapornsawan, Yongyos, Tundulawessa, Yongyuth. Effect of nicotine polyestex gum on smoking cessation and quality of life. Journal of the Medical Association of Thailand = Chotmaihet thangphaet. 2008;91:1656.  **Abstract:** OBJECTIVETo determine the effectiveness and safety of the novel nicotine polyestex gum for smoking cessation, along with its impact on the quality of life (QOL).MATERIAL AND METHODA double-blind, placebo-controlled, randomized clinical trial was conducted on 43 smokers. All of them received either nicotine gum or placebo. Only those who could quit completely and continuously by the end of 3 months were considered total abstinence. QOL was also measured using WHO questionnaires.RESULTSTreatment with nicotine polyestex gum resulted in significantly greater abstinence rate at 3 months compared with placebo (50% vs. 9%; p = 0.003). Adverse events were modest and not encountered more often than those seen in the placebo group. QOL at 3-months improved in both groups, but there was no statistically significant difference between the groups.CONCLUSIONNicotine polyestex gum is effective and safe for smoking cessation. It is also associated with significant improvement in the QOL. |
| 1277 | Cropsey, Karen, Eldridge, Gloria, Weaver, Michael, Villalobos, Gabriela, Stitzer, Maxine, Best, Al. Smoking cessation intervention for female prisoners: addressing an urgent public health need. American journal of public health. 2008;98:1894.  **Abstract:** OBJECTIVESWe tested the efficacy of a combined pharmacologic and behavioral smoking cessation intervention among women in a state prison in the southern United States.METHODSThe study design was a randomized controlled trial with a 6-month waitlist control group. The intervention was a 10-week group intervention combined with nicotine replacement therapy. Two hundred and fifty participants received the intervention, and 289 were in the control group. Assessments occurred at baseline; end of treatment; 3, 6, and 12 months after treatment; and at weekly sessions for participants in the intervention group.RESULTSThe intervention was efficacious compared with the waitlist control group. Point prevalence quit rates for the intervention group were 18% at end of treatment, 17% at 3-month follow-up, 14% at 6-month follow-up, and 12% at 12-month follow-up, quit rates that are consistent with outcomes from community smoking-cessation interventions.CONCLUSIONSFemale prisoners are interested in smoking cessation interventions and achieved point-prevalence quit rates similar to community samples. Augmenting tobacco control policies in prison with smoking cessation interventions has the potential to address a significant public health need. |
| 1278 | Shiffman, Saul, Sweeney, Christine T, Ferguson, Stuart G, Sembower, Mark A, Gitchell, Joseph G. Relationship between adherence to daily nicotine patch use and treatment efficacy: secondary analysis of a 10-week randomized, double-blind, placebo-controlled clinical trial simulating over-the-counter use in adult smokers. Clinical therapeutics. 2008;30:1852.  **Abstract:** BACKGROUNDIt has been reported that the efficacy of acute forms of nicotine replacement therapy, such as nicotine gum and lozenges, improves when sufficient quantities of medication are used.OBJECTIVEThis analysis examined whether adherence with daily nicotine patch wear was associated with improved rates of smoking abstinence.METHODSThis was a secondary analysis of data from a double-blind study in which subjects were randomized to receive either an active nicotine patch or a placebo patch under simulated over-the-counter conditions. Subjects were asked to complete a daily diary on their patch use and smoking. Logistic regression, controlling for smoking in the first 3 weeks of treatment, was used to evaluate the likelihood of abstinence at 6 weeks as a function of treatment assignment (active vs placebo) and adherence (ie, patch wear for >or=20 of the first 21 days of treatment). The relationship between reported adverse events and adherence was also examined.RESULTSThis analysis involved data from 371 subjects, 204 using the active patch and 167 using the placebo patch. The study population was mainly white (87.3%), had a mean age of 42.8 years, a mean weight of 77.3 kg, had been smoking for a mean of 24.4 years, and smoked a mean of 25.2 cigarettes per day. Two hundred fifty-three subjects were classified as adherent. Rates of adherence did not differ significantly between the active and placebo groups (139 [68.1%] and 114 [68.3%], respectively). The likelihood of experiencing an adverse event did not differ significantly between adherent and nonadherent subjects in either group. Among active patch users, 61.5% of nonadherent subjects experienced an adverse event, compared with 59.7% of adherent subjects; among placebo patch users, the corresponding proportions were 41.5% and 43.9%. Among active patch users, the odds of abstinence at 6 weeks were more than 3 times greater for adherent versus nonadherent subjects (53.2% vs 21.5%, respectively; adjusted odds ratio [OR] = 3.25; 95% CI, 1.30-8.09; P = 0.011); no benefit of adherence over nonadherence was seen among users of the placebo patch (16.7% vs 15.1%; adjusted OR = 0.60; 95% CI, 0.16-2.31). The interaction between treatment group and adherence was statistically significant (P = 0.022).CONCLUSIONUnder conditions simulating over-the-counter use, adherence to daily nicotine patch wear within the first 3 weeks of treatment was associated with an improved likelihood of achieving smoking abstinence at 6 weeks. |
| 1279 | Shiffman, Saul, Ferguson, Stuart G. The effect of a nicotine patch on cigarette craving over the course of the day: results from two randomized clinical trials. Current medical research and opinion. 2008;24:2795.  **Abstract:** OBJECTIVESThe objective of this analysis was to assess the efficacy of a 21 mg/24-h nicotine patch for the reduction of craving throughout the waking day, compared both to placebo, and to a 15 mg/16-h patch differing pharmacokinetic profile over the day. The primary end-point was craving during the evening hours, because previous research suggested that smoking relapse was particularly likely at that time.RESEARCH DESIGN AND METHODSData were drawn from two similar randomized clinical trials among nicotine-dependent smokers who were quitting smoking: Study 1 compared the 21 mg/24-h patch to a placebo patch, while Study 2 compared the 21 mg/24-h patch to a 15 mg/16-h nicotine patch. In both studies, subjects (Study 1: n = 102; Study 2: n = 244) were prompted by an electronic diary to rate their craving multiple times per day during a 1 week baseline period, and for up to 2 weeks after quitting. For analysis, the day was divided into five blocks: morning (up to 10:59 a.m.), mid-day (11:00 a.m.-1:59 p.m.), afternoon (2:00 p.m.-4:59 p.m.), evening (5:00 p.m.-8:59 p.m.), and late night (9:00 p.m. onwards). The individual craving ratings were divided into three intervals based on time since quitting: Days 1-3, 4-7, and 8-14.RESULTSThe 21 mg/24-h nicotine patch resulted in significantly lower craving during all post-quit intervals, at each time of day, both compared to placebo (Study 1), and compared to the 15 mg/16-h nicotine patch (Study 2). Study 2 saw a significant treatment by interval interaction: in later time intervals, the difference in craving experience between 24- and 16-h patch conditions shrunk--while remaining significantly different--as overall levels of craving experienced by subjects in the two groups dropped. Adverse events reported in both studies tended to be mild and transient, consistent with the well characterized adverse event profile of nicotine patches.CONCLUSIONSStudy 1 demonstrated that a 21 mg/24-h patch was effective in reducing craving throughout the day, including the evening period when relapse risk is heightened. A further study comparing the 21 mg/24-h patch to a 15 mg/16-h nicotine patch found that craving was significantly lower at all times of day for smokers using the 21 mg/24-h patch. The studies were limited in that craving was only monitored for the first 2 weeks of quitting (when craving is most prominent), and cannot elucidate the impact of patch use on craving outside of this time. Also, there was substantial attrition of the sample over time, partly due to relapse in all conditions. |
| 1282 | Oncken, Cheryl, Dornelas, Ellen, Greene, John, Sankey, Heather, Glasmann, Allen, Feinn, Richard, Kranzler, Henry R. Nicotine gum for pregnant smokers: a randomized controlled trial. Obstetrics and gynecology. 2008;112:859.  **Abstract:** OBJECTIVETo estimate the safety and efficacy of treatment with 2-mg nicotine gum for smoking cessation during pregnancy.METHODSPregnant women who smoked daily received individualized behavioral counseling and random assignment to a 6-week treatment with 2-mg nicotine gum or placebo followed by a 6-week taper period. Women who did not quit smoking were instructed to reduce the number of cigarettes smoked by substituting with gum. Measures of tobacco exposure were obtained throughout the study.RESULTSParticipants in the nicotine (n = 100) and placebo (n = 94) groups were comparable in age, race/ethnicity, and smoking history. Biochemically validated smoking-cessation rates were not significantly higher with nicotine gum compared with placebo (after 6 weeks of treatment: 13% compared with 9.6%, P=.45; at 32-34 weeks of gestation: 18% compared with 14.9%, P=.56). Using a completer analysis, nicotine gum significantly reduced the number of cigarettes smoked per day (nicotine gum: -5.7 [standard deviation (SD)=6.0]; placebo: -3.5 [SD=5.7], P=.035), and cotinine concentration (nicotine gum: -249 ng/mL [SD=397]; placebo: -112 ng/mL [SD=333]; P=.04). Birth weights were significantly greater with nicotine gum compared with placebo (3,287 g [SD=566] and 2,950 g [SD=653], respectively, P<.001). Gestational age was also greater with nicotine-replacement therapy than with placebo (38.9 weeks [SD=1.7] and 38.0 weeks [SD=3.3], respectively; P=.014).CONCLUSIONAlthough nicotine gum did not increase quit rates, use of nicotine gum increased birth weight and gestational age, two key parameters in predicting neonatal wellbeing. |
| 1283 | Habib, Ashraf S, White, William D, El Gasim, Magdi A, Saleh, Gamal, Polascik, Thomas J, Moul, Judd W, Gan, Tong J. Transdermal nicotine for analgesia after radical retropubic prostatectomy. Anesthesia and analgesia. 2008;107:999.  **Abstract:** BACKGROUNDPrevious animal and human studies suggested that nicotine might have an antinociceptive effect. We hypothesized that the preoperative application of a 7 mg nicotine patch would result in reduced postoperative analgesic requirements in patients undergoing radical retropubic prostatectomy (RRP) under general anesthesia.METHODSNonsmokers undergoing RRP under general anesthesia were enrolled in this prospective, double-blind, placebo-controlled study. Patients were randomly assigned to receive a patch of 7 mg nicotine or placebo applied behind the ear 30-60 min before induction of anesthesia. The anesthetic technique was standardized. Postoperative analgesia was provided with a standardized morphine patient-controlled analgesia and 6 hourly ketorolac 15 mg IV. Data were collected in the postanesthesia care unit and at 6, 12, and 24 h after surgery.RESULTSNinety patients were included in the analysis: 44 in the nicotine group and 46 in the placebo group. The groups did not differ significantly with respect to age, height, weight, ASA class, length of surgery, or amounts of intraoperative fentanyl received. The nicotine group showed significantly lower cumulative morphine consumption at 24 h (mean +/- sd): 33.3 +/- 30.8 mg vs 44.7 +/- 26.4 mg (P = 0.0059, time x treatment P = 0.0031). However, the repeated measures tests found no difference in amount of pain reported on coughing or at rest, either as treatment effects or in interaction with time. In post hoc comparisons, there was no significant difference in amount of pain reported on coughing or at rest at any of the times assessed. There were also no significant differences between the groups in the incidence of postoperative nausea and vomiting or the need for rescue antiemetics. However, the maximum nausea verbal rating scale score was higher in the nicotine than in the placebo group (median, 25th to 75th percentiles = 4, 0-6 vs 0, 0-6, P = 0.0158). There was a significant negative correlation between the 24 h plasma nicotine levels and postoperative morphine consumption in the postanesthesia care unit (P = 0.049), as well as at 6, 12, and 24 h (P = 0.002).CONCLUSIONThe preoperative application of a 7 mg nicotine patch resulted in a significant reduction in opioid consumption in patients undergoing RRP under general anesthesia. Despite this reduction in opioid use, there was no reduction in pain scores or postoperative nausea and vomiting with the use of transdermal nicotine. |
| 1284 | Hong, Daewha, Conell-Price, Jessamyn, Cheng, Sean, Flood, Pamela. Transdermal nicotine patch for postoperative pain management: a pilot dose-ranging study. Anesthesia and analgesia. 2008;107:1005.  **Abstract:** BACKGROUNDNicotine has been shown to be antinociceptive in the postoperative period in animal studies. Human studies with nasal nicotine sprays have had mixed results, possibly due to variability in pharmacokinetics and potential patient variables such as exposure to nicotine in tobacco smokers. In this pilot study, we examined the analgesic effect of a transdermal nicotine patch applied before surgery in nonsmokers.METHODSWe conducted a randomized, double-blind, prospective placebo-controlled trial of 40 subjects, undergoing general surgery that required postoperative patient-controlled analgesia and an overnight hospital admission. Immediately before surgery, a transdermal nicotine patch containing 0, 5, 10, or 15 mg was applied. The primary outcome variable was pain report using a numerical rating scale (NRS) in the first hour after surgery and over the next 5 days. Secondary outcomes were pain medication use, hemodynamic values, nausea, and sedation.RESULTSPatients treated with nicotine reported lower pain scores when compared with those treated with placebo during the first hour after surgery (P = 0.003, average NRS decrease = 1.4, 95% CI = 0.3-2.6) and for 5 days after surgery (P = 0.03, average NRS decrease = 1.0, 95% CI = 0.1-1.9). There was no increased benefit of nicotine with doses larger than 5 mg. There was a trend suggesting decreased pain medicine use, increased nausea, decreased tachycardia, and slightly decreased systolic blood pressure in the nicotine groups, but these values did not reach significance.CONCLUSIONSTransdermal nicotine, 5-15 mg, reduced postoperative pain scores but failed to decrease the need for opioid analgesics or opioid-related side effects after general surgical procedures. |
| 1285 | Turan, Alparslan, White, Paul F, Koyuncu, Onur, Karamanliodlu, Beyhan, Kaya, Gaye, Apfel, Christian C. Transdermal nicotine patch failed to improve postoperative pain management. Anesthesia and analgesia. 2008;107:1011.  **Abstract:** BACKGROUNDA single 3 mg intranasal dose of nicotine has been reported to have analgesic properties. We designed placebo-controlled study to test the hypothesis that transdermal nicotine (TDN) administered over a 3-day period would decrease postoperative pain and opioid analgesic usage and improve the recovery process after lower abdominal surgery.METHODSNinety-seven patients undergoing abdominal hysterectomy procedures were randomly assigned to one of two treatment groups: (1) control group received inert (sham) patches 1 h before and for 2 days after surgery, or the (2) nicotine group received TDN 30 (21 mg nicotine) patches 1 h before induction of anesthesia and for two additional days after surgery. The anesthetic technique was identical in both groups, and the postoperative assessments included verbal rating scales for pain and sedation, IV patient-controlled analgesia morphine usage, quality of recovery assessment, recovery of bowel function, resumption of normal activities, and patient satisfaction with their pain management. Follow-up evaluations were performed at 1 and 3 mo after the operation to assess late recovery events.RESULTSPostoperative patient-controlled analgesia morphine usage and pain scores while supine or sitting up, intraoperative fentanyl use, oral analgesic consumption, return of bowel sounds, and passage of flatus did not differ between the two groups. Although ambulation and hospitalization times, as well as quality of recovery scores, did not differ, resumption of oral intake was delayed in the nicotine group. Discharge eligibility scores were higher in the nicotine group at 48 and 72 h compared with the control group, but the time to return to work was 19 days in both treatment groups.CONCLUSIONSPerioperative administration of a high-dose TDN patch did not improve postoperative pain control or decrease the analgesic requirement after pelvic gynecological surgery. Despite delayed resumption of oral intake, more patients in the nicotine group were ready for discharge at 48 and 72 h after surgery. However, times to resuming activities of daily living were similar in both groups. |
| 1288 | Goedert, J J, Scoppio, B M, Pfeiffer, R, Neve, L, Federici, A B, Long, L R, Dolan, B M, Brambati, M, Bellinvia, M, Lauria, C, Preiss, L, Boneschi, V, Whitby, D, Brambilla, L. Treatment of classic Kaposi sarcoma with a nicotine dermal patch: a phase II clinical trial. Journal of the European Academy of Dermatology and Venereology : JEADV. 2008;22:1101.  **Abstract:** BACKGROUNDKaposi sarcoma (KS), a malignancy of dermal endothelial cells that is caused by human herpesvirus 8 (HHV8) infection, is sensitive to perturbations of immunity. Nicotine might be effective against KS because of its immunologic and vascular effects and because smoking is associated with a low risk of KS.OBJECTIVE AND STUDY DESIGNWe conducted a masked, randomized phase 2 clinical trial of transdermal nicotine and placebo patches to assess the safety and efficacy of nicotine against classic KS (cKS).SUBJECTS AND METHODSThree cKS lesions, predominantly nodules, in each of 24 non-smoking patients were randomly assigned to 15 weeks continuous treatment with nicotine patch (escalated to 7 mg), identical masked placebo patch or no patch. Changes in lesion area and elevation from baseline through six follow-up visits, by direct measurement and by two independent readers using digital photographs of the lesions, were compared using non-parametric and regression methods. Changes in longitudinal levels of HHV8 antibodies and DNA in blood cells were similarly assessed.RESULTSThere were no systemic or serious adverse events, and compliance was good. One patient resumed smoking and discontinued patches, and two patients withdrew at week 12 for unrelated indications. Six (29%) of the remaining 21 suspended use of patches to relieve local skin irritation; four of these six completed the trial at reduced dose. Treatment assignment was not associated with significant or consistent changes in cKS lesion area or elevation, HHV8 viral load or antibodies.CONCLUSIONTransdermal nicotine and placebo patches caused no serious toxicities but had no demonstrable effect on nodular cKS lesions or HHV8 levels. |
| 1292 | Shiffman, Saul. Effect of nicotine lozenges on affective smoking withdrawal symptoms: secondary analysis of a randomized, double-blind, placebo-controlled clinical trial. Clinical therapeutics. 2008;30:1461.  **Abstract:** BACKGROUNDThe suggested mechanism for the effects of nicotine replacement medications such as nicotine lozenges on smoking abstinence is reduction in the withdrawal symptoms of emotional distress and craving (the subjective desire to smoke).OBJECTIVESThis study assessed the effect of nicotine lozenges on affective withdrawal symptoms (collectively termed emotional distress) and craving over 6 weeks of treatment and the role of emotional distress and craving in mediating the effect of the lozenges on smoking abstinence.METHODSThis was a secondary analysis of data from a randomized, double-blind, placebo-controlled clinical trial of nicotine lozenges. High-dependence smokers (those who smoked their first cigarette of the day within 30 minutes of waking) were assigned to receive the 4-mg lozenge; low-dependence smokers (those who smoked their first cigarette of the day >30 minutes after waking) were assigned to receive the 2-mg lozenge. Participants were randomized to receive active or placebo lozenges within these dose and dependence strata. Smokers were to rate their withdrawal symptoms daily during the baseline week (while still smoking) and for 6 weeks after starting treatment. Study analyses included the effect of the active lozenge on affective symptoms (ie, anxiety; anger, irritability, or frustration; difficulty concentrating; restlessness; and depressed mood) during weeks 1 through 6 in high- and low-dependence smokers; the prospective associations between these symptoms and craving and subsequent abstinence; and the mediating influence of these symptoms on the lozenge's effect on abstinence. The analyses included smokers who provided symptom data for the baseline period and for at least week after the initiation of treatment.RESULTSOf 1,818 smokers enrolled in the original study, this analysis included data from 1,144. The population was predominantly white, had a mean age ranging ranging from 40.65 to 46.01 years, and included slightly more women than men. The 2-mg lozenge did not have consistently significant effects on the withdrawal symptoms of emotional distress among low-dependence smokers; however, in high-dependence smokers, the 4-mg dose was associated with significant reductions versus placebo in overall emotional distress symptoms through week 4 (P < 0.001-P = 0.025), all individual symptoms through week 3 (P < 0.001-P = 0.035), and irritability and anxiety through week 4 (P = 0.002-P = 0.049). In the low-dependence group, the 2-mg lozenge was associated with significant reductions versus placebo in craving through week 3 (P = 0.012-P = 0.033), whereas in the high-dependence group, the 4-mg lozenge was associated with significant reductions in craving in each of the first 6 weeks (P < 0.001-P = 0.028). Among high-dependence smokers, both week-1 and week-2 emotional distress scores were associated with a return to smoking by week 6 (P < 0.001); among low dependence smokers, the association applied only to week-2 symptoms (P = 0.017). Week-1 and week-2 craving was associated with a return to smoking at week 6 in both groups (P < 0.001-P = 0.001). Emotional distress modestly and inconsistently mediated the effects of the lozenges, accounting for 3% to 13% of the treatment effects, whereas craving more strongly (though incompletely) mediated the treatment effects, particularly among high-dependence smokers, in whom it accounted for 29% to 39% of the treatment effects.CONCLUSIONSIn high-dependence smokers, the 4-mg nicotine lozenge significantly reduced all affective withdrawal symptoms through the first 4 weeks of treatment. Lozenge-related decreases in craving partially mediated the effect of treatment on abstinence, particularly in high-dependence smokers. |
| 1293 | Hong, L Elliot, Wonodi, Ikwunga, Lewis, Jada, Thaker, Gunvant K. Nicotine effect on prepulse inhibition and prepulse facilitation in schizophrenia patients. Neuropsychopharmacology : official publication of the American College of Neuropsychopharmacology. 2008;33:2167.  **Abstract:** Acoustic prepulse inhibition (PPI) is considered an important biomarker in animal studies of psychosis and a number of psychiatric conditions. Nicotine has been shown to improve acoustic PPI in some animal strains and in humans. However, there is little data on effects of nicotine on acoustic PPI in schizophrenia patients using a double-blind, placebo-controlled study design. The primary aim of the current study was to test the effect of nicotine nasal spray on acoustic PPI in schizophrenia patients. The secondary aim was to test nicotine effect on prepulse facilitation (PPF). The study included 18 schizophrenia patient smokers and 12 healthy control smokers, tested in a double-blind, placebo-controlled, crossover, randomized design immediately after nicotine or saline placebo nasal sprays. PPI was tested using 120 ms prepulse-pulse interval. PPF was tested using 4500 ms prepulse-pulse interval. The results showed a significant main effect of drug on PPI in that nicotine improved PPI compared to placebo (p=0.008) with no drug by diagnosis interaction (p=0.90). Improvement in PPI in response to nicotine was significantly correlated with the baseline severity of clinical symptoms (r=0.59, p=0.02) in patients. There was no significant drug or drug by diagnosis interaction for the 4500 ms prepulse-pulse interval condition. However, nicotine improved inhibition in a subgroup of subjects exhibiting PPF (p=0.002). In conclusion, the findings confirmed that nicotine transiently improves acoustic PPI in schizophrenia patients. Additionally, schizophrenia patients with more clinical symptoms may have benefited more from nicotinic effect on PPI. |
| 1295 | Aubin, H-J, Bobak, A, Britton, J R, Oncken, C, Billing, C B, Jr, Gong, J, Williams, K E, Reeves, K R. Varenicline versus transdermal nicotine patch for smoking cessation: results from a randomised open-label trial. Thorax. 2008;63:717.  **Abstract:** BACKGROUNDVarenicline, a new treatment for smoking cessation, has demonstrated significantly greater efficacy over placebo and sustained release bupropion (bupropion SR). A study was undertaken to compare a 12-week standard regimen of varenicline with a 10-week standard regimen of transdermal nicotine replacement therapy (NRT) for smoking cessation.METHODSIn this 52-week, open-label, randomised, multicentre, phase 3 trial conducted in Belgium, France, The Netherlands, UK and USA, participants were randomly assigned (1:1) to receive varenicline uptitrated to 1 mg twice daily for 12 weeks or transdermal NRT (21 mg/day reducing to 7 mg/day) for 10 weeks. Non-treatment follow-up continued to week 52. The primary outcome was the biochemically confirmed (exhaled carbon monoxide < or = 10 ppm) self-reported continuous abstinence rate (CAR) for the last 4 weeks of the treatment period in participants who had taken at least one dose of treatment. Secondary outcomes included CAR from the last 4 weeks of treatment through weeks 24 and 52, and measures of craving, withdrawal and smoking satisfaction.RESULTSA total of 376 and 370 participants assigned to varenicline and NRT, respectively, were eligible for analysis. The CAR for the last 4 weeks of treatment was significantly greater for varenicline (55.9%) than NRT (43.2%; OR 1.70, 95% CI 1.26 to 2.28, p<0.001). The week 52 CAR (NRT, weeks 8-52; varenicline, weeks 9-52) was 26.1% for varenicline and 20.3% for NRT (OR 1.40, 95% CI 0.99 to 1.99, p = 0.056). Varenicline significantly reduced craving (p<0.001), withdrawal symptoms (p<0.001) and smoking satisfaction (p<0.001) compared with NRT. The most frequent adverse event was nausea (varenicline, 37.2%; NRT, 9.7%).CONCLUSIONSThe outcomes of this trial established that abstinence from smoking was greater and craving, withdrawal symptoms and smoking satisfaction were less at the end of treatment with varenicline than with transdermal NRT.TRIAL REGISTRATION NUMBERNCT00143325. |
| 1303 | Reid, Malcolm S, Fallon, Bryan, Sonne, Susan, Flammino, Frank, Nunes, Edward V, Jiang, Huiping, Kourniotis, Eva, Lima, Jennifer, Brady, Ron, Burgess, Cynthia, Arfken, Cynthia, Pihlgren, Eric, Giordano, Louis, Starosta, Aron, Robinson, James, Rotrosen, John. Smoking cessation treatment in community-based substance abuse rehabilitation programs. Journal of substance abuse treatment. 2008;35:68.  **Abstract:** Nicotine dependence is highly prevalent among drug- and alcohol-dependent patients. A multisite clinical trial of smoking cessation (SC) treatment was performed at outpatient community-based substance abuse rehabilitation programs affiliated with the National Drug Abuse Treatment, Clinical Trials Network. Cigarette smokers (N=225) from five methadone maintenance programs and two drug and alcohol dependence treatment programs were randomly assigned in a 2:1 ratio to receive either (1) SC treatment as an adjunct to substance abuse treatment-as-usual (TAU) or (2) substance abuse TAU. Smoking cessation treatment consisted of 1 week of group counseling before the target quit date and 8 weeks of group counseling plus transdermal nicotine patch treatment (21 mg/day for Weeks 1-6 and 14 mg/day for Weeks 7 and 8) after the target quit date. Smoking abstinence rates in SC, 10%-11% during treatment and 5%-6% at the 13- and 26-week follow-up visits, were significantly better than those in TAU during treatment (p< .01). In addition, SC was associated with significantly greater reductions as compared with TAU in cigarettes smoked per day (75% reduction, p< .001), exhaled carbon monoxide levels (p< .001), cigarette craving (p< .05), and nicotine withdrawal (p< .05). Smoking cessation did not differ from TAU on rates of retention in substance abuse treatment, abstinence from primary substance of abuse, and craving for primary substance of abuse. Compliance with SC treatment, moderate at best, was positively associated with smoking abstinence rates. Smoking cessation treatment resulted in significant reductions in daily smoking and modest smoking abstinence rates without having an adverse impact on substance abuse rehabilitation when given concurrently with outpatient substance abuse treatment. Substance abuse treatment programs should not hesitate to implement SC for established patients. |
| 1306 | Sherman, Scott E, Aldana, Ileana, Estrada, Maribel, York, Laura. Comparing the tolerability and effectiveness of two treatment regimens in a smoking clinic. Military medicine. 2008;173:550.  **Abstract:** This study compares the effectiveness and tolerability of bupropion versus bupropion plus nicotine patch for smoking cessation in a routine clinical setting. Patients at the Sepulveda Veterans Health Administration Smoking Cessation Clinic completed a baseline survey and received counseling over 2 months, along with bupropion or bupropion plus nicotine patch. Of the 227 patients randomized to treatment, 112 (49%) received bupropion only and 115 (51%) received the combination therapy. At least one side effect was noted in 55% of bupropion patients and 70% of combination therapy patients; treatment regimens were changed in 7% and 14%, respectively. Abstinence rates at 2 months were 26% for the bupropion group and 37% for the combination therapy group (p = 0.1), and at 6 months were 42% versus 35%, respectively (p = 0.4). Although 6-month abstinence rates were derived from patient self-report and should be interpreted with caution, these results suggest that most patients referred to the clinic are able to take these medications. There was no difference in the rate of switching treatments, or in long-term abstinence rates. |
| 1309 | Barnett, Paul G, Wong, Wynnie, Hall, Sharon. The cost-effectiveness of a smoking cessation program for out-patients in treatment for depression. Addiction (Abingdon, England). 2008;103:834.  **Abstract:** AIMSSmoking cessation programs are highly cost-effective. The cost-effectiveness of programs for psychiatric patients may be affected by differences in cost, efficacy, survival and quality of life. We evaluated cost-effectiveness of a program for smokers being treated for depression.DESIGNA randomized trial compared brief contact to a stepped smoking cessation program in 322 cigarette smoking mental health out-patients. We determined the intervention's direct cost. Because smoking cessation may affect short-term use of mental health care, we used administrative databases and self-report to find mental health-care cost.FINDINGSThe 163 individuals randomized to stepped care received an average of 6 of smoking cessation services, including 1 for computer-mediated assessments of readiness to quit, and 4 for counseling, nicotine replacement therapy and bupropion. The cessation program was used by 53 participants. Total cost of smoking cessation and mental health services was 05 in the stepped care group and 73 in the brief-contact care group (not significantly different). After 18 months of follow-up, the stepped care group had 5.5% greater abstinence from smoking. Smoking cessation services cost 04 per successful quit. Cessation services and mental health care cost was 496 per successful quit.CONCLUSIONIf smoking cessation yields 1.2 years additional life, the cessation services cost 70 per life-year, and cessation services and mental health care cost 80 per life-year. Even if quitting does not increase survival of depressed individuals as well as in other smokers, the stepped care intervention is likely to be regarded as cost-effective. |
| 1313 | Takahashi, Hidehiko, Fujimura, Yota, Hayashi, Mika, Takano, Harumasa, Kato, Motoichiro, Okubo, Yoshiro, Kanno, Iwao, Ito, Hiroshi, Suhara, Tetsuya. Enhanced dopamine release by nicotine in cigarette smokers: a double-blind, randomized, placebo-controlled pilot study. The international journal of neuropsychopharmacology. 2008;11:413.  **Abstract:** Previous studies of smoking on dopamine release in humans were investigated only in smokers. Using nicotine gum, we examined the effect of nicotine on dopamine release in smokers and non-smokers and its relation to the degree of nicotine dependence. Smokers and non-smokers participated in a double-blind, randomized, placebo-controlled cross-over study. They participated in two PET measurements with [11C]raclopride, in which they received either nicotine or placebo. Changes in [11C]raclopride non-displaceable binding potential (BPND) following nicotine administration were quantified. Smokers showed significant decrease in BP in the striatum following nicotine administration, but non-smokers did not show such a decrease. The BPND difference between the two scanning sessions was correlated with the degree of nicotine dependence. The BPND difference might reflect enhanced dopamine release in smokers and the reinforced effect of nicotine. These data suggest the feasibility of our gum method as well as the importance of the degree of dependence in future studies of the nicotine effect on the dopamine system. |
| 1315 | Harte, Christopher B, Meston, Cindy M. The inhibitory effects of nicotine on physiological sexual arousal in nonsmoking women: results from a randomized, double-blind, placebo-controlled, cross-over trial. The journal of sexual medicine. 2008;5:1184.  **Abstract:** INTRODUCTIONExtensive research suggests that long-term cigarette smoking is an independent risk factor for the introduction of sexual dysfunction in men. However, results of limited data investigating this relationship in women are mixed. No studies have examined the acute effects of tobacco or nicotine on physiological sexual response in women. Controlled experimental studies examining acute effects of isolated nicotine intake on female physiological sexual responses are necessary in order to help elucidate tobacco's potential role in the development and/or maintenance of sexual impairment in women.AIMTo examine whether isolated nicotine intake acutely affects sexual arousal responses in nonsmoking women.METHODSTwenty-five sexually functional women (mean age = 20 years) each with less than 100 direct exposures to nicotine completed two counterbalanced conditions in which they were randomized to received either nicotine gum (6 mg) or placebo gum, both administered double-blind and matched for appearance, taste, and consistency, approximately 40 minutes prior to viewing an erotic film.MAIN OUTCOME MEASURESPhysiological (changes in vaginal pulse amplitude via vaginal photoplethysmography) and subjective (continuous self-report) sexual responses to erotic stimuli were examined, as well as changes in mood.RESULTSNicotine significantly reduced genital responses to the erotic films (P = 0.05), corresponding to a 30% attenuation in physiological sexual arousal. This occurred in 11 of 18 women with valid physiological assessments. Nicotine had no significant effect on continuous self-report ratings of sexual arousal (P = 0.45), or on mood (all Ps > 0.05).CONCLUSIONSAcute nicotine intake significantly attenuates physiological sexual arousal in healthy nonsmoking women. Our findings provide support to the hypothesis that nicotine may be the primary pharmacological agent responsible for genital hemodynamic disruption, thereby facilitating a cascade of biochemical and vascular events which may impair normal sexual arousal responses. |
| 1317 | Pan, Alan X, de la Peña, Amparo, Yeo, Kwee P, Chan, Clark, Loh, Mei T, Wise, Stephen D, Silverman, Bernard L, Muchmore, Douglas B. Effects of smoking cessation, acute re-exposure and nicotine replacement in smokers on AIR inhaled insulin pharmacokinetics and glucodynamics. British journal of clinical pharmacology. 2008;65:480.  **Abstract:** WHAT IS ALREADY KNOWN ABOUT THIS SUBJECT* Only one other study (Becker et al.) has reported on the influence of smoking cessation and smoking resumption on inhaled insulin pharmacokinetics and glucodynamics, concluding that the rapid changes associated with smoking resumption carry the risk for hypoglycaemia and thus should not be used by active smokers.WHAT THIS STUDY ADDS* This is the first euglycaemic clamp study on the impact of smoking cessation, acute smoking re-exposure and nicotine replacement on AIR((R)) inhaled insulin pharmacokinetics and glucodynamics. * We demonstrate clinically and statistically significant shifts in glucodynamic response to acute re-exposure to a single cigarette, leading us to conclude that active smokers should be advised against inhaled insulin therapy until smoking abstinence is stable. * Additionally, these results are also the first to demonstrate an apparent independent effect of nicotine replacement therapy on insulin exposure and glucodynamic response.AIMSTo explore the effects of smoking cessation and acute smoking re-exposure on the pharmacokinetic (PK) and glucodynamic (GD) profiles of AIR inhaled insulin (AIR Insulin) with or without nicotine replacement therapy (NRT).METHODSNondiabetic smokers (n = 24) with normal pulmonary function completed a two-phase (four-period), open-label, randomized euglycaemic clamp study. During the initial study phase, subjects underwent glucose clamps following AIR Insulin dosing, shortly after smoking, 8-12 h after smoking, or following subcutaneous insulin lispro shortly after smoking. AIR Insulin PK and GD were again assessed during and after a 4-week smoking-cessation period with or without NRT. In the last study period, subjects smoked one cigarette shortly before final AIR Insulin dosing and glucose clamp, to study the effect of acute smoking re-exposure on inhaled insulin PK and GD.RESULTSCompared with the preceding active smoking phase, the administration of AIR Insulin in nondiabetic subjects undergoing a 4-week period of smoking abstinence resulted in a decrease in PK and GD of approximately 25% (P = 0.008 for both), an effect which was greater in subjects using NRT. Following rechallenge with a single cigarette (without NRT), GD response to AIR Insulin increased significantly (P = 0.006) towards precessation levels, relative to smoking abstinence. In subjects using NRT, however, the increase in GD was less pronounced.CONCLUSIONSmoking, smoking cessation and acute re-exposure with a single cigarette are associated with clinically significant alterations in AIR Insulin pharmacokinetics and glucodynamics. AIR Insulin should not be used by smokers or those at risk for recidivism. |
| 1319 | David, S P, Munafò, M R, Murphy, M F G, Proctor, M, Walton, R T, Johnstone, E C. Genetic variation in the dopamine D4 receptor (DRD4) gene and smoking cessation: follow-up of a randomised clinical trial of transdermal nicotine patch. The pharmacogenomics journal. 2008;8:122.  **Abstract:** Smokers of European ancestry (n=720) who participated in a double-blind, randomised, placebo-controlled trial of transdermal nicotine replacement therapy, were genotyped for two functional polymorphisms (variable number of tandem repeats (VNTR) and a C to T transition at position -521 (C-521T)) in the dopamine D4 receptor gene (DRD4) gene. Logistic regression models of abstinence at 12- and 26-week follow-ups were carried out separately for each polymorphism. For the DRD4 VNTR models, the main effect of treatment was significant at both 12-week (P=0.001) and 26-week (P=0.006) follow-ups, indicating an increased likelihood of successful cessation on active nicotine replacement therapy transdermal patch relative to placebo. The main effect of DRD4 VNTR genotype was associated with abstinence at 12-week follow-up (P=0.034), with possession of one or more copies of the long allele associated with reduced likelihood of cessation (17 vs 23%), but this effect was not observed at 26-week follow-up. For the DRD4 C-521T models, no main effect or interaction terms involving genotype were retained in the models at either 12- or 26-week follow-up. These data are consistent with observations from studies of the DRD2 gene that genetic variants related to relatively decreased dopaminergic tone in the mesocorticolimbic system are associated with increased risk for relapse to smoking following a cessation attempt. |
| 1323 | Sofuoglu, Mehmet, Yoo, Sonah, Hill, Kevin P, Mooney, Marc. Self-administration of intravenous nicotine in male and female cigarette smokers. Neuropsychopharmacology : official publication of the American College of Neuropsychopharmacology. 2008;33:715.  **Abstract:** Although nicotine is the main addictive chemical in tobacco, there have been few studies of pure nicotine self-administration in humans. The goal of this study was to test the parameters of an intravenous (IV) nicotine self-administration model using nicotine doses presumed to be within the range of those of average intake from cigarette smoking. Six male and four female smokers participated in a double-blind, placebo-controlled, crossover study, which consisted of one adaptation and three experimental sessions. In each experimental session, subjects were randomly assigned to one of the three doses of nicotine (0.1, 0.4, or 0.7 mg). The lowest nicotine dose, 0.1 mg, was chosen to be approximately half the amount of nicotine inhaled from one puff of a cigarette. During each experimental session, subjects first sampled the assigned nicotine dose and placebo and then had the opportunity to choose between nicotine and placebo for a total of six choices over a 90-min period. Out of six options, the average (SEM) number of nicotine choices were 3.0 (0.48) for 0.1 mg, 4.7 (0.48) for 0.4 mg and 4.5 (0.46) for 0.7 mg, indicating a significant effect of nicotine dose on nicotine choice. Both the 0.4 and 0.7, but not the 0.1 mg, nicotine doses were preferred to placebo. These higher doses also produced increases in heart rate, blood pressure, and ratings of drug liking and high. Overall, these findings indicate that smokers chose both the 0.4 and the 0.7 mg nicotine doses over placebo. Our model may be useful in the evaluation of the effects of both behavioral and pharmacological manipulations on nicotine self-administration in humans. |
| 1325 | Joseph, Anne M, Hecht, Stephen S, Murphy, Sharon E, Lando, Harry, Carmella, Steven G, Gross, Myron, Bliss, Robin, Le, Chap T, Hatsukami, Dorothy K. Smoking reduction fails to improve clinical and biological markers of cardiac disease: a randomized controlled trial. Nicotine & tobacco research : official journal of the Society for Research on Nicotine and Tobacco. 2008;10:471.  **Abstract:** Cigarette reduction has been proposed as a treatment goal for smokers who are not interested in stopping completely. This randomized controlled trial was designed to determine the effect of a smoking reduction intervention on smoking behavior, symptoms of heart disease, and biomarkers of tobacco exposure. It included 152 patients with heart disease who did not intend to stop smoking in the next 30 days. Participants were randomly assigned to smoking reduction (SR) or usual care (UC). SR subjects received counseling and nicotine replacement therapy to encourage > or =50% reduction in cigarettes per day (CPD). They were followed at 1, 3, 6, 12 and 18 months to assess smoking, heart disease symptoms, quality of life and nicotine, cotinine, carbon monoxide (CO), white blood cell (WBC) count, fibrinogen, hs-C-reactive protein (hs-CRP), F2-isoprostane, 4-(methylnitrosamino)-1-(3-pyridyl)-1-butanol and its glucuronides (total NNAL), and 1-hydroxypyrene (1-HOP). At 6 months SR participants reduced by 10.9 CPD, compared with 7.4 CPD in UC (difference NS). At 18 months, 9/78 SR vs. 9/74 UC participants quit smoking. There were no significant differences between treatment groups in angina, quality of life or adverse events, nicotine, cotinine, CO, WBC count, fibrinogen, hs-CRP, F2-isoprostane, total NNAL or 1-HOP levels at any time point. To determine if smoking reduction, regardless of treatment condition, was associated with improved outcomes, we compared all subjects at 6 months to baseline (mean reduction in CPD from 27.4 to 18.1, p<.01). There were no significant changes in outcome variables except CO, which decreased by 5.5 ppm (p<.01). There were also no significant improvements considering only subjects who reduced by > or =50%, or those who had no history of reduction prior to enrollment in the study. The SR intervention did not significantly reduce CPD or toxin exposure, or improve smoking cessation or clinical outcomes compared to UC. These results emphasize the importance of abstinence for smokers with heart disease to minimize health risks from tobacco. |
| 1329 | Myers, Carol S, Taylor, Richard C, Moolchan, Eric T, Heishman, Stephen J. Dose-related enhancement of mood and cognition in smokers administered nicotine nasal spray. Neuropsychopharmacology : official publication of the American College of Neuropsychopharmacology. 2008;33:588.  **Abstract:** The discovery of the role of nicotinic receptors in attention and memory has led to the testing of nicotinic analogs as cognitive enhancing agents in patient populations. Empirical information about nicotine's ability to enhance elements of attention and memory in normal individuals might guide development of therapeutic uses of nicotine in cognitively impaired populations. The purpose of this study was to determine the effect of nicotine on continuous attention, working memory, and computational processing in tobacco-deprived and nondeprived smokers. A total of 28 smokers (14 men, 14 women) participated in a double-blind, placebo-controlled, within-subject study, in which they were overnight (12 h) tobacco deprived at one session and smoked ad libitum before the other session. At each session, participants received 0, 1, and 2 mg nicotine via nasal spray in random order at 90 min intervals. Before and after each dose, a battery of cognitive, subjective, and physiological measures was administered, and blood samples were taken for plasma nicotine concentration. Overnight tobacco deprivation resulted in impaired functioning on all cognitive tests and increased self-reports of tobacco craving and negative mood; nicotine normalized these deficits. In the nondeprived condition, nicotine enhanced performance on the continuous performance test (CPT) and an arithmetic test in a dose-related manner, but had no effect on working memory. In general, women were more sensitive than men to the subjective effects of nicotine. These results provide an unequivocal determination that nicotine enhanced attentional and computational abilities in nondeprived smokers and suggest these cognitive domains as substrates for novel therapeutic indications. |
| 1332 | Cheng, Sean S, Yeh, Janet, Flood, Pamela. Anesthesia matters: patients anesthetized with propofol have less postoperative pain than those anesthetized with isoflurane. Anesthesia and analgesia. 2008;106:264.  **Abstract:** BACKGROUNDPreclinical studies have suggested that some volatile anesthetics induce a hyperalgesic state that may be secondary to nicotinic inhibition. A previous trial of treatment with nicotine nasal spray demonstrated postoperative analgesia in women anesthetized with isoflurane. To determine whether the effect of nicotine was reversing hyperalgesia induced by isoflurane, or simply acting as an analgesic, we studied the effect of nicotine on postoperative pain in women anesthetized with isoflurane or propofol, with fentanyl.METHODSIn a randomized, prospective, double-blind trial, we assigned 80 women having open uterine surgery to be anesthetized with isoflurane or propofol. Within each anesthetic group, the subjects were further randomly assigned to receive nicotine 3 mg or placebo. Pain reported with a numerical analog scale was the primary outcome variable.RESULTSThe patient demographics were similar. Women who were anesthetized with propofol reported less pain and used less morphine during the first day after surgery than women who were anesthetized with isoflurane (P < 0.01, P < 0.01). Nicotine treatment did not change pain report or morphine use in either anesthetic group (P > 0.05).CONCLUSIONSGeneral anesthesia with propofol and is associated with less postoperative pain and morphine use than general anesthesia with isoflurane. Nicotine was not analgesic in this trial. If these results are repeated in other populations, reduced postoperative pain can be added to the previously described improvement in nausea and vomiting as a potential benefit of anesthesia with propofol. |
| 1333 | Kinnunen, Taru, Korhonen, Tellervo, Garvey, Arthur J. Role of nicotine gum and pretreatment depressive symptoms in smoking cessation: twelve-month results of a randomized placebo controlled trial. International journal of psychiatry in medicine. 2008;38:373.  **Abstract:** OBJECTIVEThe aim was to investigate the role of nicotine gum and pretreatment depressive symptoms in long-term smoking cessation.METHODS12-month follow-up of 608 participants within a randomized, controlled trial was conducted. Participants received brief counseling plus nicotine or placebo treatment. Based on the Center for Epidemiological Studies Depression Scale pre-treatment score (> or =16), 32% of the participants were classified as depressed at baseline.RESULTSAt the 12-month follow-up the non-depressed participants with nicotine replacement therapy (NRT) were most successful (20.1%), while the depressed ones with placebo were least successful (5.7%) (p = .004). However, the depressed participants with NRT (15.1%) were not significantly less successful than the non-depressed ones with NRT (20.1%, p = .23). Survival analysis showed significant depression (p = .01) and treatment effects (p = < .001) on the abstinence. Further, when adjusted for marital status and education, the treatment effect remained significant (p < .001), whereas the depression effect became non-significant (p = .08).CONCLUSIONSNRT improved cessation both among the depressed and non-depressed. To understand discrepancies in literature reporting depression and smoking cessation outcome, the pharmacological treatment modality and social-demographic variables should be examined as additional variables. |
| 1335 | Okuyemi, Kolawole S, Faseru, Babalola, Sanderson Cox, Lisa, Bronars, Carrie A, Ahluwalia, Jasjit S. Relationship between menthol cigarettes and smoking cessation among African American light smokers. Addiction (Abingdon, England). 2007;102:1979.  **Abstract:** AIMSTo determine whether African American light smokers who smoked menthol cigarettes had lower cessation when treated with nicotine replacement therapy and counseling.DESIGNData were derived from a clinical trial that assessed the efficacy of 2 mg nicotine gum (versus placebo) and counseling (motivational interviewing counseling versus Health Education) for smoking cessation among African American light smokers (smoked < or = 10 cigarettes per day).PARTICIPANTSThe sample consisted of 755 African American light smokers.MEASUREMENTSThe primary outcome variable was verified 7-day point-prevalence smoking cessation at 26 weeks follow-up. Verification was by salivary cotinine.FINDINGSCompared to non-menthol smokers, menthol smokers were younger and less confident to quit smoking (P = 0.023). At 26 weeks post-randomization, 7-day verified abstinence rate was significantly lower for menthol smokers (11.2% versus 18.8% for non-menthol, P = 0.015).CONCLUSIONSAmong African American light smokers, use of menthol cigarettes is associated with lower smoking cessation rates. Because the majority of African American smokers use menthol cigarettes, a better understanding of the mechanism for this lower quit rate is needed. |
| 1339 | Myung, Seung Kwon, Seo, Hong Gwan, Park, Sohee, Kim, Yeol, Kim, Dong Jin, Lee, Do Hoon, Seong, Moon Woo, Nam, Myung Hyun, Oh, Seung Won, Kim, Ji Ae, Kim, Mi Young. Sociodemographic and smoking behavioral predictors associated with smoking cessation according to follow-up periods: a randomized, double-blind, placebo-controlled trial of transdermal nicotine patches. Journal of Korean medical science. 2007;22:1065.  **Abstract:** This study investigated sociodemographic and smoking behavioral factors associated with smoking cessation according to follow-up periods. In this randomized, double-blind, placebo-controlled trial of transdermal nicotine patches, subjects were a total of 118 adult male smokers, who were followed up for 12 months. Univariable logistic regression analysis and stepwise multiple logistic regression analyses were performed to identify the predictors of smoking cessation. The overall self-reported point prevalence rates of abstinence were 20% (24/118) at 12 months follow-up, and there was no significant difference in abstinence rates between placebo and nicotine patch groups. In the univariable logistic regression analysis, predictors of successful smoking cessation were the low consumption of cigarettes per day and the low Fagerstrom Test for Nicotine Dependence (FTND) scores (p<0.05) at 3, 6, and 12 months follow-up. In the stepwise multiple logistic regression analyses, predictors of successful smoking cessation, which were different according to the follow- up periods, were found to be the low consumption of cigarettes per day at the short-term and midterm follow-up (< or =6 months), older age, and the low consumption of cigarettes per day at the long-term follow-up (12 months). |
| 1340 | Gallagher, Sandra M, Penn, Patricia E, Schindler, Eric, Layne, Wendy. A comparison of smoking cessation treatments for persons with schizophrenia and other serious mental illnesses. Journal of psychoactive drugs. 2007;39:487.  **Abstract:** Adults with any DSM-IV diagnosed mental illness smoke nearly half of the cigarettes consumed in the U.S. (Lasser et al. 2000). This study compared two smoking cessation interventions for persons with schizophrenia or other serious mental illness because national data suggests that: (1) they smoke at two to three times the rate of the general population; (2) cessation interventions for this population are understudied; (3) most cessation studies exclude persons with serious mental illness; and (4) cessation results in public health care savings and disposable income savings for clients. This study included a large number of persons with serious mental illness (N=181) who were randomly assigned to one of three groups: contingent reinforcement (CR), CR plus nicotine patch (21 mg, CR+NRT) for 16 weeks, and a minimal intervention, self-quit control group. These participants were followed for 36 weeks. CR was accomplished with escalating financial compensation for achieving and maintaining abstinence as verified by expired carbon monoxide (CO). Quit rates, as measured by expired CO, were higher and discordant with saliva cotinine quit rates. Cotinine showed lower quit rates and small differences between intervention and control participants at weeks 20 and 36. There was, however, evidence of reduced smoking and importantly, no evidence of psychiatric exacerbation. |
| 1342 | Cooney, Ned L, Litt, Mark D, Cooney, Judith L, Pilkey, David T, Steinberg, Howard R, Oncken, Cheryl A. Concurrent brief versus intensive smoking intervention during alcohol dependence treatment. Psychology of addictive behaviors : journal of the Society of Psychologists in Addictive Behaviors. 2007;21:570.  **Abstract:** Alcohol dependent smokers (N=118) enrolled in an intensive outpatient substance abuse treatment program were randomized to a concurrent brief or intensive smoking cessation intervention. Brief treatment consisted of a 15-min counseling session with 5 min of follow-up. Intensive intervention consisted of three 1-hr counseling sessions plus 8 weeks of nicotine patch therapy. The cigarette abstinence rate, verified by breath carbon monoxide, was significantly higher for the intensive treatment group (27.5%) versus the rate for the brief treatment group (6.6%) at 1 month after the quit date but not at 6 months, when abstinence rates fell to 9.1% for the intensive treatment group and 2.1% for the brief treatment group. Smoking treatment assignment did not significantly impact alcohol outcomes. Although intensive smoking treatment was associated with higher rates of short-term tobacco abstinence, other, perhaps more intensive, smoking interventions are needed to produce lasting smoking cessation in alcohol dependent smokers. |
| 1344 | Hollis, Jack F, McAfee, Timothy A, Fellows, Jeffrey L, Zbikowski, Susan M, Stark, Michael, Riedlinger, Karen. The effectiveness and cost effectiveness of telephone counselling and the nicotine patch in a state tobacco quitline. Tobacco control. 2007;16 Suppl 1:i53.  **Abstract:** OBJECTIVESState and national tobacco quitlines have expanded rapidly and offer a range of services. We examined the effectiveness and cost effectiveness of offering callers single session versus multisession counselling, with or without free nicotine patches.METHODSThis 3x2 randomised trial included 4614 Oregon tobacco quitline callers and compared brief (one 15-minute call), moderate (one 30-minute call and a follow-up call) and intensive (five proactive calls) intervention protocols, with or without offers of free nicotine patches (nicotine replacement therapy, NRT). Blinded staff assessed tobacco use by phone at 12 months.RESULTSAbstinence odds ratios were significant for moderate (OR = 1.22, CI = 1.01 to 1.48) and intensive (OR = 1.29, CI = 1.07 to 1.56) intervention, and for NRT (OR = 1.58, CI = 1.35 to 1.85). Intent to treat quit rates were as follows: brief no NRT (12%); brief NRT (17%); moderate no NRT (14%); moderate NRT (20%); intensive no NRT (14%); and intensive NRT (21%). Relative to brief no NRT, the added costs for each additional quit was 67 for brief NRT, 12 for moderate no NRT, 09 for moderate NRT, 41 for intensive no NRT, and 12 for intensive NRT.CONCLUSIONOffering free NRT and multisession telephone support within a state tobacco quitline led to higher quit rates, and similar costs per incremental quit, than less intensive protocols. |
| 1345 | Takahashi, Hidehiko, Fujimura, Yota, Takano, Harumasa, Ito, Hiroshi, Suhara, Tetsuya. Association between nicotine dependence and striatal dopamine release following nicotine administration. Journal of Cerebral Blood Flow and Metabolism. 2007;27:BP20.  **Abstract:** Background and aims: It has been suggested that mesolimbic dopamine pathway mediate the rewarding effect of nicotine. [11C]raclopride PET method has been used for the indirect measurement of changes in synaptic dopamine concentration in response to addictive drugs like cocaine and amphetamine. Since synaptic dopamine competes with [11C]raclopride for D2 receptor binding, dopamine release is thought to decrease [11C]raclopride binding potential (BP). Decreases in [11C]raclopride BP in the ventral striatum have also been demonstrated following tobacco smoke in smokers. However there is no PET study comparing dopamine release following nicotine administration in nonsmokers with those in smokers. In this study, we used nicotine gum aiming to administer nicotine in nonsmokers as in smokers. Methods: Twelve healthy male subjects (6 smokers, mean age 25.8 years and 6 nonsmokers mean age 23.7 years) participated in the study. The Fagerstrom test for nicotine dependence (FTND) was used for assessing nicotine dependence. Smokers were instructed not to smoke for 24 hours before scanning. The study was a double-blind, randomized, placebo-controlled cross-over design. Each subject participated in two PET measurements separated by at least 4 weeks. One hour before each PET measurement, subjects received two pieces of either nicotine or taste-matched placebo gum. Plasma nicotine concentration was measured just before gum administration, 60, 75, 90, 105, 120min after gum administration. Dynamic PET scans were performed for 60 minutes immediately after the injection of a bolus of [11C]raclopride. BP values were quantified using the simplified reference tissue model for regions-of-interest (ROI), those were the dorsal caudate, dorsal putamen, ventral caudate and ventral putamen. The cerebellum was used as reference region. BP values of each ROI following nicotine and placebo administration were compared by paired t-tests. Pearson correlation coefficients between changes in BP values of each ROI and nicotine dependence assessed with FTND were calculated. Results: Plasma concentrations of nicotine during PET scan using this procedure were similar to those achieved by smoking a typical cigarette (6-16 ng/ml). Although smokers and nonsmokers did not differ significantly in BP of each ROI in both nicotine and placebo conditions, smokers showed significant decreases in [11C]raclopride BP in the ventral caudate [2.75±0.24 (placebo), 2.46±0.21 (nicotine)] and ventral putamen [3.59±0.31(placebo), 3.14±0.33(nicotine)] in response to nicotine, but nonsmokers did not. Reduction of BP in the ventral striatum was correlated with the degree of nicotine dependence. Conclusions: Decrease in [11C]raclopride BP is thought to reflect dopamine release following nicotine administration in smokers. Enhanced dopamine release in smokers may underlie reinforced rewarding effect of nicotine in dependence after repeated exposure. |
| 1350 | Dautzenberg, Bertrand, Nides, Mitchell, Kienzler, Jean-Luc, Callens, Anne. Pharmacokinetics, safety and efficacy from randomized controlled trials of 1 and 2 mg nicotine bitartrate lozenges (Nicotinell). BMC clinical pharmacology. 2007;7:11.  **Abstract:** BACKGROUNDThe use of nicotine replacement therapy (NRT) can almost double the chances of success for smokers to quit. Nevertheless, there is still a considerable number of cessation attempts that are made without any treatment. This novel oral formulation, (lozenge containing nicotine bitartrate dihydrate) has been developed to enlarge the offer for efficient smoking cessation drug therapies, assuming that increasing treatment options will bring more smokers to find the support they personally need to stop smoking.METHODSThree pharmacokinetic (PK), one safety and two efficacy studies were carried out with Nicotinell lozenges. PK trials were: (1) a single-dose, three-way crossover study comparing 1 and 2 mg lozenges with 2 mg nicotine gum; (2) a multiple-dose, two-way crossover study comparing 1 mg lozenge with 2 mg gum; (3) a multiple-dose, three-way crossover study comparing 1 and 2 mg lozenges with 4 mg gum. Safety trial: (4) a single dose study to assess the safety of swallowing up to 12 lozenges containing 1 mg nicotine. Efficacy trials: two efficacy studies in (5) France and (6) the USA, including more than 900 smokers followed-up for up to one year, conducted with the 1 mg lozenge.RESULTSThe results of the individual PK trials showed that the 1 mg Nicotinell lozenge is bioequivalent to 2 mg polacrilex gum, as demonstrated by similar blood PK parameters (tmax, Cmax, AUC). The 2 mg lozenge was found to deliver quantities of nicotine that were intermediate between those delivered by 2 and 4 mg polacrilex gum. The short-term efficacy of the 1 mg lozenge in comparison with placebo was also demonstrated with significantly more subjects continuously abstinent from smoking with active lozenges on week 6 in two different populations: moderate to heavy smokers (FTND between 4 and 7) OR = 1.72 [95% CI: 1.05-2.80]; heavy to very heavy smokers (FTND 6 and over) OR = 2.87 [95% CI: 1.18-6.97]. Nicotinell lozenges were found to be safe with mainly mild and reversible adverse events. The safety of the 1 mg lozenge formulation, even when misused was also demonstrated.CONCLUSIONThe data presented in this review demonstrate high nicotine bioavailability, excellent safety profile and proven short-term efficacy of Nicotinell lozenges. At nominal equivalent doses 1 and 2 mg Nicotinell lozenges were shown to deliver larger amounts of bioavailable nicotine compared to the nicotine polacrilex gum. According to the data developed here, the systemic exposure to nicotine could be ranked: 4 mg polacrilex gum > 2 mg Nicotinell lozenge > 1 mg Nicotinell lozenge = 2 mg polacrilex gum.Adverse events observed during the clinical trials were mild or moderate in severity, transient and completely reversible. With respect to efficacy in smoking cessation, significantly higher continuous abstinence rates were achieved with lozenge compared to placebo. In conclusion, Nicotinell lozenges offer a valuable addition to the therapeutic armamentarium available for smoking cessation. |
| 1351 | Pollak, Kathryn I, Oncken, Cheryl A, Lipkus, Isaac M, Lyna, Pauline, Swamy, Geeta K, Pletsch, Pamela K, Peterson, Bercedis L, Heine, R Phillips, Brouwer, Rebecca J Namenek, Fish, Laura, Myers, Evan R. Nicotine replacement and behavioral therapy for smoking cessation in pregnancy. American journal of preventive medicine. 2007;33:297.  **Abstract:** BACKGROUNDThis study examines whether adding nicotine replacement therapy (NRT) to cognitive-behavioral therapy (CBT) for pregnant smokers increases rates of smoking cessation.METHODSAn open-label randomized trial (Baby Steps, n=181) of CBT-only versus CBT+NRT (choice of patch, gum, or lozenge; 1:2 randomization) was used. Data were collected from 2003 through 2005; analyses were conducted in 2006 and 2007. Outcomes were biochemically validated self-reported smoking status at 7 weeks post-randomization, 38 weeks gestation, and 3 months postpartum.RESULTSWomen in the CBT+NRT arm were almost three times more likely than women in the CBT-only arm to have biochemically validated cessation at both pregnancy time points (after 7 weeks: 24% vs 8%, p=0.02; at 38 weeks gestation: 18% vs 7%, p=0.04), but not at 3 months postpartum (20% vs 14%, p=0.55). Recruitment was suspended early by an Independent Data and Safety Monitoring Board when an interim analysis found a higher rate of negative birth outcomes in the CBT+NRT arm than in the CBT-only arm. In the final analysis, the difference between the arms in rate of negative birth outcomes was 0.09 (p=0.26), when adjusted for previous history of preterm birth.CONCLUSIONSThe addition of NRT to CBT promoted smoking cessation in pregnant women. This effect did not persist postpartum. More data are needed to determine safety parameters and to confirm the efficacy of NRT use during pregnancy. |
| 1352 | Munafò, M R, Elliot, K M, Murphy, M F G, Walton, R T, Johnstone, E C. Association of the mu-opioid receptor gene with smoking cessation. The pharmacogenomics journal. 2007;7:353.  **Abstract:** We investigated the association of the OPRM1 genotype with long-term smoking cessation and change in body mass index (BMI) following a smoking cessation attempt among smokers who attempted to quit using the nicotine replacement therapy (NRT) patch or placebo in a randomized controlled trial, and were followed-up over an 8-year period following their initial cessation attempt. We also investigated possible sex differences in these relationships, given evidence for sex differences in smoking cessation and central opioid mechanisms, as well as some evidence for sex differences in response to NRT. Our results indicate that OPRM1 genotype may moderate the effect of transdermal nicotine patch compared to placebo during active treatment, with a benefit of active NRT treatment evident in the OPRM1 AA genotype group only and those carrying one or more copies of the G allele demonstrating no benefit of active NRT versus placebo patch. Our results also indicate a sex difference in change in BMI at 8-year follow-up following a smoking cessation attempt, with ex-smokers demonstrating an increase in BMI, and this increase being greater in female subjects than in male subjects. We did not observe any association of OPRM1 genotype with change in BMI, although there was a trend for genotype to influence the observed sex difference in change in BMI over time. Future studies should attempt to replicate these findings, and investigate the relationship between both short- and long-term weight gain and smoking cessation and investigate possible mechanisms that may underlie these processes. Future studies should also investigate the role of OPRM1 genotype and smoking cessation on other appetitive and reward behaviours such as alcohol consumption. |
| 1354 | Blank, Melissa D, Kleykamp, Bethea A, Jennings, Janine M, Eissenberg, Thomas. Caffeine's influence on nicotine's effects in nonsmokers. American journal of health behavior. 2007;31:473.  **Abstract:** OBJECTIVETo determine if nicotine's effects are influenced by caffeine in nonsmoking, moderate-caffeine consuming individuals (N=20).METHODSThe first 3 sessions included one of 3 randomly ordered, double-blind caffeine doses (0, 75, or 150 mg, oral [po]) and 2 single-blind nicotine gum doses (2 and 4 mg) in ascending order. The fourth session (single blind) repeated the 0 mg caffeine condition.RESULTSNicotine increased heart rate and subjective ratings indicative of aversive effects, and decreased reaction times. These effects were independent of caffeine dose and reliable across sessions.CONCLUSIONSIn nonsmokers, nicotine effects are not influenced by moderate caffeine doses. |
| 1355 | Piper, Megan E, Federman, E Belle, McCarthy, Danielle E, Bolt, Daniel M, Smith, Stevens S, Fiore, Michael C, Baker, Timothy B. Efficacy of bupropion alone and in combination with nicotine gum. Nicotine & tobacco research : official journal of the Society for Research on Nicotine and Tobacco. 2007;9:947.  **Abstract:** In this double-blind, placebo-controlled smoking cessation treatment study, 608 participants were randomly assigned to receive active bupropion and active 4-mg gum (AA, n = 228), active bupropion and placebo gum (AP, n = 224), or placebo bupropion and placebo gum (PP, n = 156). Relative to the PP group, the AA and AP groups were each significantly more likely to be abstinent at 1 week, end of treatment, and 6 months but not at 12 months postquit. After the first week postquit there were no differences in abstinence rates between the AA and AP groups. We found no significant individual difference variables that moderated outcome beyond 1 week postquit. |
| 1356 | Covey, Lirio S, Glassman, Alexander H, Jiang, Huiping, Fried, Jane, Masmela, Jenny, LoDuca, Catherine, Petkova, Eva, Rodriguez, Kristina. A randomized trial of bupropion and/or nicotine gum as maintenance treatment for preventing smoking relapse. Addiction (Abingdon, England). 2007;102:1292.  **Abstract:** AIMTo investigate the efficacy of maintenance treatment with bupropion and/or nicotine gum for reducing smoking relapse.DESIGN, SETTING AND PARTICIPANTSA 48-week study was conducted at a university-based smoking cessation clinic between February 2001 and October 2005. A total of 588 smokers received bupropion and nicotine patch in 8 weeks of open-label treatment (OLT); 289 abstainers during the last 4 weeks of OLT were randomized in double-blind placebo-controlled fashion to one of four arms for 16 weeks of maintenance treatment (MT) followed by 24 weeks of non-treatment follow-up (NTFU).INTERVENTIONBupropion (300 mg/day) and 2 mg nicotine gum, used alone or combined, and comparable placebo pill and placebo gum. Behavioral counseling at all visits.OUTCOMETime to relapse (TTR) from randomization. Relapse is defined as the first 7 consecutive days of smoking. Abstinence verified by carbon monoxide <or= 8 parts per million.FINDINGSTTR was longer with extended active treatments compared to placebo (median days to relapse: bupropion + placebo = 136, nicotine + placebo = 98, bupropion + nicotine = 90, double placebo = 71). Hazard ratios (HR) for relapse were statistically significant for bupropion + placebo versus double placebo during MT (HR = 0.59, 95% CI = 0.37-0.92) and to the end of NTFU (HR = 0.66, 95% CI = 0.42-0.96). However, bupropion's advantage dissipated upon stopping the drug. Gum use was low, preventing a valid assessment; but analysis restricted to gum users suggested a weak effect of extended nicotine gum.CONCLUSIONMaintenance treatment with bupropion exerted a modest benefit for preventing smoking relapse; the optimum duration of bupropion treatment was unclear. Further research is needed to ascertain the merits of extended use of nicotine gum, other nicotine replacement agents and other treatments known to aid smokers for preventing relapse once abstinence is achieved. |
| 1362 | Ebbert, Jon O, Post, Jason A, Moyer, Thomas P, Dale, Lowell C, Schroeder, Darrell R, Hurt, Richard D. Nicotine percentage replacement among smokeless tobacco users with nicotine patch. Drug and alcohol dependence. 2007;89:223.  **Abstract:** To obtain preliminary evidence on the safety and efficacy of high dose nicotine patch therapy among smokeless tobacco (ST) users who consume > or =3 cans of ST per week, we conducted a randomized, placebo-controlled clinical trial with 42 ST users randomized to nicotine patch doses of 21, 42, and 63 mg/day or placebo. Serum nicotine concentrations were measured during ad libitum ST use and nicotine replacement therapy, and percentages of nicotine replacement were calculated. We observed substantial inter-subject variability in nicotine concentrations with ad lib ST use. The mean percentage replacement of ad lib ST use serum nicotine concentrations approximated 100% with the 42 mg/day patch dose (mean+/-S.D., 98.4%+/-45%). Dosing with the 21 mg/day nicotine patch was associated with mean "under-replacement" (53.2%+/-17.1%), and the 63 mg/day nicotine was associated with mean "over-replacement" (159.2%+/-121.9%). We observed symptoms of nausea consistent with nicotine toxicity in two subjects in the 63 mg/day group while no subjects in the 42 mg/day reported these symptoms. We conclude that the use of 42 mg/day nicotine patch therapy is safe and should be considered as initial therapy in the clinical setting among ST users who use > or =3 cans/week. |
| 1363 | Prapavessis, Harry, Cameron, Linda, Baldi, J Chris, Robinson, Stewart, Borrie, Kendra, Harper, Therese, Grove, J Robert. The effects of exercise and nicotine replacement therapy on smoking rates in women. Addictive behaviors. 2007;32:1416.  **Abstract:** PURPOSETo examine the individual effects of supervised and intensive exercise as well as the combined effects of exercise and nicotine replacement therapy (NRT) on (a) smoking cessation and reduction rates and (b) psychological and physiological processes during withdrawal.METHODSOne-hundred and forty-two inactive female smokers were randomised into the following four groups: exercise+nicotine patch; exercise+no nicotine patch; cognitive behavior therapy (CBT)+nicotine patch and CBT+no nicotine patch. Smoking abstinence (verified by saliva cotinine and expired carbon monoxide), cessation self-efficacy, and physical fitness and body weight were assessed at baseline (week 1), quit date (week 6), program termination (week 12), and 3- and 12-month follow-up.RESULTSThere were significant differences in a 7-day point prevalence but not continuous abstinence rates between treatment groups across targeted end points. Consistently higher cessation rates were seen when NRT was added to both treatment programs. Compared with CBT participants, exercise participants had significantly increased functional exercise capacity and had gained significantly less weight during program end points but these differences did not hold at a 12-month follow-up. Compared with exercise participants, CBT participants felt greater cessation efficacy and reported greater knowledge, coping and support resources across all end points.CONCLUSIONSExercise combined with NRT facilitates smoking cessation, improves functional exercise capacity, and delays weight gain in women smokers. We recommend that physicians and health care professionals recommend exercise and NRT together for highly motivated women interested in quitting smoking. |
| 1366 | Johnstone, Elaine C, Elliot, Katherine M, David, Sean P, Murphy, Michael F G, Walton, Robert T, Munafò, Marcus R. Association of COMT Val108/158Met genotype with smoking cessation in a nicotine replacement therapy randomized trial. Cancer epidemiology, biomarkers & prevention : a publication of the American Association for Cancer Research, cosponsored by the American Society of Preventive Oncology. 2007;16:1065.  **Abstract:** We investigated the association of catechol O-methyltransferase (COMT) genotype with abstinence following a smoking cessation attempt among a large cohort of smokers who attempted to quit using either the nicotine transdermal patch or placebo and were followed up over an 8-year period following their initial cessation attempt. In addition, we examined the possible moderating influence of sex on any association. The genotype x treatment interaction effect at 12-week follow-up indicated a greater benefit of active nicotine replacement treatment compared with placebo on likelihood of abstinence in the COMT Met/Met genotype group (33% versus 12%), in comparison to the Met/Val + Val/Val group (22% versus 16%). Our results indicate that COMT genotype may moderate the effect of active transdermal nicotine patch compared with placebo, with reduced relative benefit of nicotine replacement therapy in individuals with Met/Val or Val/Val genotype. Our data follow an emerging pattern of results suggesting that genetic variation in the dopamine pathway may provide a future basis for tailored smoking cessation therapies, but indicate that different genes influencing various components of this pathway may have different effects on response to smoking cessation pharmacotherapy. |
| 1368 | Uyar, Meral, Filiz, Ayten, Bayram, Nazan, Elbek, Osman, Herken, Hasan, Topcu, Ayfer, Dikensoy, Oner, Ekinci, Erhan. A randomized trial of smoking cessation. Medication versus motivation. Saudi medical journal. 2007;28:922.  **Abstract:** OBJECTIVEA prospective randomized study was undertaken to assess the effectiveness and side effect profiles of nicotine patch and bupropion therapies for smoking cessation.METHODSThree hundred and fifty patients were referred to our smoking cessation program in the Department of Pulmonary Diseases, Gaziantep University between September 2002 and July 2003. Of these, only 131 patients fulfilled the trial criteria. We randomized the patients into nicotine patch (n=50), bupropion (n=50) and control groups (n=31). Cases were followed up for 24 weeks. Questionnaires including the Fagerstrom test for nicotine dependence and Beck Depression Inventory were carried out at initial evaluation. Declaration of quitting and exhaled carbon monoxide level less than 10 ppm was accepted as success criteria.RESULTSSuccess rates were 26% for nicotine patch group, 26% for bupropion and 16% for control group at the end of the 24th week (p=0.56). Beck depression inventory scores did not differ significantly between the groups, however none of the cases with scores greater than 13 succeeded regardless of the group. Mean body weight at baseline and change at 6 months did not differ significantly between the groups. Sleep disturbance was significantly more common in nicotine patch and bupropion groups than the control group (p=0.008).CONCLUSIONThe present study reinforces the role of medical doctors and importance of close follow up in smoking cessation, and directed counseling is quite as effective as pharmacologic therapy and is the sole approach without any adverse effects. |
| 1369 | Binnie, Vivian I, McHugh, Siobhan, Jenkins, William, Borland, William, Macpherson, Lorna M. A randomised controlled trial of a smoking cessation intervention delivered by dental hygienists: a feasibility study. BMC oral health. 2007;7:5.  **Abstract:** BACKGROUNDTobacco use continues to be a global public health problem. Helping patients to quit is part of the preventive role of all health professionals. There is now increasing interest in the role that the dental team can play in helping their patients to quit smoking. The aim of this study was to determine the feasibility of undertaking a randomised controlled smoking cessation intervention, utilising dental hygienists to deliver tobacco cessation advice to a cohort of periodontal patients.METHODSOne hundred and eighteen patients who attended consultant clinics in an outpatient dental hospital department (Periodontology) were recruited into a trial. Data were available for 116 participants, 59 intervention and 57 control, and were analysed on an intention-to-treat basis. The intervention group received smoking cessation advice based on the 5As (ask, advise, assess, assist, arrange follow-up) and were offered nicotine replacement therapy (NRT), whereas the control group received 'usual care'. Outcome measures included self-reported smoking cessation, verified by salivary cotinine measurement and CO measurements. Self-reported measures in those trial participants who did not quit included number and length of quit attempts and reduction in smoking.RESULTSAt 3 months, 9/59 (15%) of the intervention group had quit compared to 5/57 (9%) of the controls. At 6 months, 6/59 (10%) of the intervention group quit compared to 3/57 (5%) of the controls. At one year, there were 4/59 (7%) intervention quitters, compared to 2/59 (4%) control quitters. In participants who described themselves as smokers, at 3 and 6 months, a statistically higher percentage of intervention participants reported that they had had a quit attempt of at least one week in the preceding 3 months (37% and 47%, for the intervention group respectively, compared with 18% and 16% for the control group).CONCLUSIONThis study has shown the potential that trained dental hygienists could have in delivering smoking cessation advice. While success may be modest, public health gain would indicate that the dental team should participate in this activity. However, to add to the knowledge-base, a multi-centred randomised controlled trial, utilising biochemical verification would be required to be undertaken. |
| 1372 | Etter, Jean-François, Laszlo, Evelyne. Postintervention effect of nicotine replacement therapy for smoking reduction: a randomized trial with a 5-year follow-up. Journal of clinical psychopharmacology. 2007;27:151.  **Abstract:** We tested whether a reduction of cigarette consumption obtained after 6 months of nicotine replacement therapy was maintained 5 years after the end of this treatment. Heavy smokers (mean = 30 cigarettes per day) who had no intention of quitting smoking were randomly assigned to a 6-month treatment of nicotine (15-mg patch, 4-mg gum, and/or 10-mg inhaler, n = 265), placebo (n = 269), or no intervention (n = 389). Products were sent by mail, and education was limited to a booklet. Of 923 participants, 879 (95%) were followed after 6 months and 671 (73%) after 5 years. After 6 months, smoking reduction was larger for nicotine (-10.9 cigarettes per day) than for placebo (-8.7) and no treatment (-4.9, all P <or= 0.022). After 5 years, cigarette consumption (20 cigarettes per day, all P >or= 0.2) and smoking cessation rates (17% to 21%, all P > 0.2) were similar in all groups. In smokers, 5-year continuous abstinence was higher in those who had reduced their cigarette consumption by at least 50% between baseline and 6 months than in those who did not reduce (11.9% vs 5.6%; P = 0.011; odds ratio, 2.3; 95% confidence interval, 1.2-4.2). Thus, the initial effect of the treatment on smoking reduction was not maintained after 5 years. However, reducing cigarette consumption was associated with a higher chance of subsequently quitting smoking. Nicotine replacement therapy in unmotivated smokers had no deleterious effect on dependence levels and smoking behavior. |
| 1379 | Dallery, Jesse, Raiff, Bethany R. Delay discounting predicts cigarette smoking in a laboratory model of abstinence reinforcement. Psychopharmacology. 2007;190:485.  **Abstract:** RATIONALEHigher rates of delay discounting, or impulsive choice, may be related to relapse during abstinence reinforcement interventions for cigarette smoking, and a transdermal nicotine patch may attenuate delay discounting.OBJECTIVEThe objectives of this study are to assess the relation between delay discounting and smoking after nicotine deprivation in a laboratory model of abstinence reinforcement and the effects of a transdermal nicotine patch on discounting and smoking.MATERIALS AND METHODSSmokers with no self-reported intention to quit were randomly assigned to an active (14 mg) or placebo patch group (n = 15 per group). In each of three sessions, after a 3-h deprivation period, participants completed a delay discounting task, mood, and craving measures and finally engaged in a laboratory model of abstinence reinforcement. Three abstinence reinforcement conditions were presented in counterbalanced order across the three sessions. During the control session, monetary consequences were delivered every 30 s regardless of smoking. During the low (5.00 dollars available) and high (20.00 dollars available) sessions, participants could earn a progressively increasing amount of money for each 30 s period of abstinence.RESULTSThe low and high conditions significantly increased the latency to smoke relative to control and significantly decreased the amount of smoking. The nicotine patch decreased negative affect, but it did not significantly affect delay discounting or smoking. Individuals who smoked during the low and high conditions showed higher rates of discounting.CONCLUSIONThe patch did not attenuate delay discounting or smoking after a period of deprivation, but contingencies for abstinence significantly decreased smoking. Higher rates of delay discounting were related to smoking in a model of abstinence reinforcement treatment. |
| 1381 | Oncken, Cheryl, Cooney, Judith, Feinn, Richard, Lando, Harry, Kranzler, Henry R. Transdermal nicotine for smoking cessation in postmenopausal women. Addictive behaviors. 2007;32:296.  **Abstract:** This study examined the efficacy of transdermal nicotine in postmenopausal smokers, and whether a history of depression or hormone replacement therapy (HRT) moderated smoking cessation outcomes. Postmenopausal smokers (N=152) received intensive smoking cessation counseling and were randomly assigned to use either a 21-mg nicotine patch for 3 months, with a 1-month taper, or a placebo patch. The primary outcome was biochemically validated 7-day point prevalence smoking abstinence during treatment (i.e., 1, 2, 6, and 12 weeks after the quit date) and 1 year after study medication was discontinued. Subjects who received transdermal nicotine were significantly more likely than placebo-treated subjects to remain abstinent from smoking during treatment, but not at the 1-year follow-up. The majority of subjects (>50%) in both groups accurately identified their treatment assignment. History of depression was associated with a decreased likelihood to abstain from smoking throughout the study. HRT did not moderate smoking outcomes. These data indicate that transdermal nicotine may provide short-term benefits for smoking cessation in postmenopausal women. However, efforts are needed to improve long-term abstinence rates and smoking outcomes among women with a history of depression. |
| 1382 | Okuyemi, Kolawole S, James, Aimee S, Mayo, Matthew S, Nollen, Nicole, Catley, Delwyn, Choi, Won S, Ahluwalia, Jasjit S. Pathways to health: a cluster randomized trial of nicotine gum and motivational interviewing for smoking cessation in low-income housing. Health education & behavior : the official publication of the Society for Public Health Education. 2007;34:43.  **Abstract:** Despite high smoking rates among those living in poverty, few cessation studies are conducted in these populations. This cluster-randomized trial tested nicotine gum plus motivational interviewing (MI) for smoking cessation in 20 low-income housing developments (HDs). Intervention participants (10 HDs, n = 66) received educational materials, 8 weeks of 4 mg nicotine gum, and 5 MI sessions on quitting smoking. Comparison participants (10 HDs, n = 107) received 5 MI sessions and educational materials addressing fruit and vegetable consumption. Participants had a mean age of 46.3 years and were predominantly female (70%) and African American (83%). Biochemically-verified 7-day abstinence rates at 8 weeks were 6.1% and 5.6% in the intervention and comparison arms, respectively (p = ns); and at 26 weeks were 7.6% and 9.3%, respectively (p = ns). Results suggest that nicotine gum plus MI were not effective for smoking cessation in low-income housing. Programs are needed to enhance the effectiveness of pharmacotherapy and counseling in underserved populations. |
| 1383 | Croghan, Ivana T, Hurt, Richard D, Dakhil, Shaker R, Croghan, Gary A, Sloan, Jeff A, Novotny, Paul J, Rowland, Kendrith M, Bernath, Albert, Loots, Mary L, Le-Lindqwister, Nguyet A, Tschetter, Loren K, Garneau, Stewart C, Flynn, Kathleen A, Ebbert, Larry P, Wender, Donald B, Loprinzi, Charles L. Randomized comparison of a nicotine inhaler and bupropion for smoking cessation and relapse prevention. Mayo Clinic proceedings. 2007;82:186.  **Abstract:** OBJECTIVETo compare the combination of a nicotine inhaler and bupropion to either treatment alone for initiating smoking abstinence and relapse prevention.METHODSSmokers were randomized to receive a nicotine inhaler, bupropion, or both for 3 months. At 3 months, smoking-abstinent study participants were randomized to their initial medications or placebo. Participants who were smoking at 3 months were randomized to an alternative treatment regimen or placebo. This study was conducted from July 2001 to January 2003.RESULTSA total of 1700 smokers were randomized to treatment (phase 1) for 3 months. Among the 941 study participants eligible for randomization to the phase 2 trial, 837 continued in the study. For the phase 2 trial, 405 smoking-abstinent participants were randomized to relapse prevention for 9 additional months, and 432 smokers were randomized to re-treatment for an additional 3 months. At the end of the initial 3 months of treatment (phase 1), 82 (14%) of 566, 145 (26%) of 567, and 194 (34%) of 567 study participants receiving a nicotine inhaler, bupropion, or both, respectively, were abstinent from smoking. Of the 405 smoking-abstinent participants at the end of 3 months, the bupropion group had more smokers than the placebo group (mean No. of smokers, 1.5 vs 1.1; P < .001), and the nicotine inhaler group had higher smoking abstinence rates at 12 months than the placebo group. Those receiving combination therapy had reduced rates of relapse to smoking for the first 3 months of relapse prevention, but this difference disappeared after the initial 3 months. Of the 432 study participants who were smoking at the end of 3 months and who received an alternative treatment regimen, the 223 smokers initially assigned to a nicotine inhaler were more likely to stop smoking at 6 months if they were re-treated with bupropion instead of placebo (8 [7%] of 111 vs 0 [0%] of 112; P = .003), and the 209 smokers initially treated with bupropion and re-treated with a nicotine inhaler did not have significantly higher smoking abstinence rates (6 [6%] of 104 vs 3 [3%] of 105; P = -.50).CONCLUSIONCombined therapy with a nicotine inhaler and bupropion increased smoking abstinence rates. Continuation of the initial combination therapy does not appear to prevent relapse to smoking. Timing of re-treatment and alternative approaches to relapse prevention should be further examined. |
| 1385 | David, Sean P, Munafò, Marcus R, Murphy, Michael F G, Walton, Robert T, Johnstone, Elaine C. The serotonin transporter 5-HTTLPR polymorphism and treatment response to nicotine patch: follow-up of a randomized controlled trial. Nicotine & tobacco research : official journal of the Society for Research on Nicotine and Tobacco. 2007;9:225.  **Abstract:** In this follow-up of a randomized placebo-controlled clinical trial of nicotine replacement transdermal patch for smoking cessation, 741 smokers of European ancestry who were randomized to receive active patch or placebo patch were genotyped for the serotonin transporter gene-linked polymorphic region. The study setting was a primary care research network in Oxfordshire, United Kingdom. The primary outcome measures were biochemically verified sustained abstinence from cigarette smoking at end of treatment and 24-week follow-up. The main effect of genotype was not associated with sustained abstinence from smoking at either end of treatment (SL: p=.33; SS: p=.81) or 24-week follow-up (SL: p=.05; SS: p=.21), and we found no evidence for a genotypextreatment interaction effect. In summary, despite the theoretically important contribution of serotonin neurotransmission to smoking cessation, the serotonin transporter gene was not associated with treatment response to nicotine patch for smoking cessation in this primary care-based trial. |
| 1392 | Ionescu, Daniela, Badescu, Cristina, Acalovschi, Iurie. Nicotine patch for the prevention of postoperative nausea and vomiting: a prospective randomised trial. Clinical drug investigation. 2007;27:559.  **Abstract:** BACKGROUND AND OBJECTIVEIt has been demonstrated that smoking significantly reduces postoperative nausea and vomiting (PONV). However, there are approximately 4000 substances in cigarette smoke that can be responsible for this effect. To demonstrate whether nicotine is the substance with antiemetic effects we applied a nicotine patch in patients undergoing laparoscopic cholecystectomy under general anaesthesia.METHODSSeventy-five patients classified as ASA (American Society of Anesthesiologists' classification) I/II were divided in three groups: group 1 (n = 25), which comprised non-smokers; group 2 (n = 25), which comprised patients who had given up smoking for the last 5 years and received perioperatively a nicotine patch that contained 16.6mg nicotine/patch; and group 3 (n = 25), which comprised actual smokers. Postoperatively, the incidence of PONV and the need for antiemetic rescue medication were monitored every 6 hours.RESULTSWe found a significant reduction in the incidence of PONV in group 2 (5/25 [20%], p = 0.0001 vs group 1) and group 3 (8/25 [32%], p = 0.002 vs group 1) compared with group 1 (18/25 [76%]). The difference in incidence of PONV between group 2 and group 3 was not significant (p > 0.05).CONCLUSIONSNicotine significantly reduced the incidence of PONV after laparoscopic cholecystectomy. |
| 1393 | Ebbert, Jon O, Dale, Lowell C, Patten, Christi A, Croghan, Ivana T, Schroeder, Darrell R, Moyer, Thomas P, Hurt, Richard D. Effect of high-dose nicotine patch therapy on tobacco withdrawal symptoms among smokeless tobacco users. Nicotine & tobacco research : official journal of the Society for Research on Nicotine and Tobacco. 2007;9:43.  **Abstract:** No pharmacotherapies have been shown to increase long-term (> or = 6-month) abstinence rates among smokeless tobacco (ST) users. Available evidence suggests that underdosing may occur with standard-dose nicotine replacement therapy (NRT) in ST users. We investigated the effect of high-dose nicotine therapy on tobacco withdrawal symptoms among ST users in a randomized, controlled clinical pilot study. A total of 42 ST users using at least 3 cans or pouches per week were randomized to nicotine patch doses of 63, 42, or 21 mg/day or placebo for 8 weeks. Multiple daily assessments of tobacco withdrawal and nicotine toxicity were obtained with an electronic diary. During the first week of nicotine patch therapy, we observed a dose-response relationship such that higher nicotine patch doses were associated with less decreased arousal (chi2 = 6.87, p = .009), less negative affect (chi2 = 3.85, p = .05), and less restlessness (chi2 = 3.90, p = .048). During the second week, higher nicotine patch doses were associated with less decreased arousal (chi2 = 6.77, p = .009). Overall, the frequency of nicotine toxicity symptoms did not differ by dose group. Of specific symptoms, nausea was observed to be more frequent in the 63 mg/day dose group compared with placebo (p = .035). In conclusion, high-dose nicotine patch therapy resulted in a greater reduction of tobacco withdrawal symptoms among ST users using at least 3 cans per week. High-dose nicotine patch therapy is safe and well tolerated in this population of tobacco users. |
| 1399 | Ferguson, Stuart G, Shiffman, Saul, Gwaltney, Chad J. Does reducing withdrawal severity mediate nicotine patch efficacy? A randomized clinical trial. Journal of consulting and clinical psychology. 2006;74:1153.  **Abstract:** Nicotine replacement therapy (NRT) repeatedly has been shown to improve smoking treatment outcome. The major mechanism posited for this improvement in outcome is that NRT reduces nicotine craving and withdrawal. The authors tested this hypothesized mechanism of action using real-time data on craving and withdrawal, collected by ecological momentary assessments administered on a palm-top computer. Smokers (N = 324) were randomized to receive either active high-dose (35 mg) 24-hr patches or placebo. Increases in positive affect and decreases in craving, negative affect, and attention disturbance severity were related to lower risk of lapsing. Although NRT treatment did significantly decrease withdrawal and craving severity, these reductions only partially accounted for NRT's impact on time to first lapse: The results from a mediation analysis showed that the hazard ratio for NRT, when controlling for withdrawal and craving severity, was only a third to a half lower than the uncontrolled hazard ratio for NRT alone. This suggests that other mechanisms for the effectiveness of NRT need to be examined. |
| 1401 | Oncken, Cheryl, Prestwood, Karen, Kleppinger, Alison, Wang, Yazhen, Cooney, Judith, Raisz, Lawrence. Impact of smoking cessation on bone mineral density in postmenopausal women. Journal of women's health (2002). 2006;15:1141.  **Abstract:** BACKGROUNDAlthough clinical guidelines recommend smoking cessation to improve bone health, the impact of short-term smoking cessation (i.e., 1 year) on bone mineral density (BMD) is not known. We examined the effects of smoking cessation on BMD measurements, markers of bone turnover, and hormone profiles in postmenopausal women.METHODSPostmenopausal women (n = 152) who smoked at least 10 cigarettes per day were randomly assigned to behavioral counseling and either nicotine or placebo patch for smoking cessation (3-month treatment with a 1-month taper) and followed for an additional year. The BMD at various sites (hip, spine, wrist, and total body), serum and urine biochemical markers of bone turnover, and sex hormones were measured at baseline and again 1 year after smoking treatment. Women who continuously abstained from smoking between the end of treatment and 1 year later (quitters) (n = 42) were compared with women who completed the study and continued to smoke (n = 77).RESULTSFemoral trochanter BMD increased by 2.9% among quitters vs. 0.6% among continued smokers (p = 0.02). Total hip BMD increased by 1.52% among quitters vs. 0.43% among continued smokers (p = 0.03). Changes in BMD at the femoral neck, radius, spine, and total body did not significantly differ between groups. The effects of smoking cessation on bone were mediated in part by weight gain. Smoking cessation was also associated with an increase in bone alkaline phosphatase.CONCLUSIONSSmoking cessation, relative to continued smoking, increases BMD at the femoral trochanter and total hip in postmenopausal women. |
| 1406 | Baker, Amanda, Richmond, Robyn, Haile, Melanie, Lewin, Terry J, Carr, Vaughan J, Taylor, Rachel L, Jansons, Sylvia, Wilhelm, Kay. A randomized controlled trial of a smoking cessation intervention among people with a psychotic disorder. The American journal of psychiatry. 2006;163:1934.  **Abstract:** OBJECTIVEDespite extremely high rates of smoking among individuals with psychotic disorders and the associated financial and health costs, few studies have investigated the efficacy of smoking cessation interventions among this group. The purpose of this study was to compare an integrated psychological and nicotine replacement therapy intervention for people with a psychotic disorder with routine care alone.METHODThe authors recruited 298 regular smokers with a psychotic disorder residing in the community and randomly assigned them to a routine care comparison condition (N=151) or an eight-session, individually administered smoking cessation intervention (N=147), which consisted of nicotine replacement therapy, motivational interviewing, and cognitive behavior therapy. Outcome variables included continuous and point-prevalence abstinence rates, smoking reduction status, and changes in symptoms and functioning.RESULTSWhile there were no overall differences between the treatment group and comparison group in abstinence rates, a significantly higher proportion of smokers who completed all treatment sessions stopped smoking at each of the follow-up occasions (point-prevalence rates: 3 months, 30.0% versus 6.0%; 6 months, 18.6% versus 4.0%; and 12 months, 18.6% versus 6.6%). Smokers who completed all treatment sessions were also more likely to have achieved continuous abstinence at 3 months (21.4% versus 4.0%). There was a strong dose-response relationship between treatment session attendance and smoking reduction status, with one-half of those who completed the intervention program achieving a 50% or greater reduction in daily cigarette consumption across the follow-ups, relative to less than one-fifth of the comparison subjects. There was no evidence of any associated deterioration in symptoms or functioning.CONCLUSIONSThese findings demonstrate the utility of a nicotine replacement therapy plus motivational interviewing/cognitive behavior therapy smoking cessation intervention among individuals with a psychotic disorder. Further development of more efficacious interventions is required for those who do not respond to existing interventions. |
| 1407 | McClernon, F Joseph, Hiott, F Berry, Westman, Eric C, Rose, Jed E, Levin, Edward D. Transdermal nicotine attenuates depression symptoms in nonsmokers: a double-blind, placebo-controlled trial. Psychopharmacology. 2006;189:125.  **Abstract:** RATIONALEDespite established links between nicotine dependence and depression, little research has examined the effects of nicotine on depression symptoms.OBJECTIVEThis study evaluated the acute and chronic effects of transdermal nicotine in nonsmokers with baseline depression symptoms during a 4-week, double-blind, placebo-controlled trial.METHODSNonsmokers with scores >or=10 on the Center for Epidemiological Studies Depression scale (CES-D) were recruited from the community. Mood and cognitive performance were measured at baseline (day 0) and at 1, 8, 21, and 28 days. Participants were randomly assigned to wear a placebo or nicotine patch for 4 weeks (3.5 mg/day during weeks 1 and 4; 7 mg/day during weeks 2 and 3). The final sample consisted of 11 nonsmokers with a mean baseline CES-D score of 27.36 (SD=10.53).RESULTSSalivary nicotine levels indicated the majority of participants were compliant with treatment. Acute nicotine did not alter mood. After adjusting for baseline values, chronic nicotine resulted in a significant decline in CES-D scores at day 8 (3.5 mg/day), but returned to placebo levels by the last visit. This return to baseline levels was coincident with a decrease in nicotine administration from 7 to 3.5 mg/day. A similar trend for improved response inhibition as measured by the Conners Continuous Performance Task was also observed. Reported side effects were infrequent and minimal.CONCLUSIONThese findings suggest a role for nicotinic receptor systems in the pathophysiology of depression and that nicotinic compounds should be evaluated for treating depression symptoms. |
| 1418 | Knott, V, McIntosh, J, Millar, A, Fisher, D, Villeneuve, C, Ilivitsky, V, Horn, E. Nicotine and smoker status moderate brain electric and mood activation induced by ketamine, an N-methyl-D-aspartate (NMDA) receptor antagonist. Pharmacology, biochemistry, and behavior. 2006;85:228.  **Abstract:** As the increased smoking prevalence in schizophrenics may be interpreted as an adaptive response to an underlying biological defect, investigations into nicotine's actions within N-methyl-d-aspartate (NMDA) antagonist drug models of schizophrenia may improve our understanding of the role of glutamatergic neurotransmission in initiating and maintaining nicotine dependence in this disorder. In this double-blind, placebo-controlled, randomized study, the electroencephalographic (EEG) and subjective response to a sub-psychotomimetic intravenous dose of the NMDA antagonist ketamine was examined in 20 regular smokers and 20 non-smokers pretreated with placebo or nicotine gum. Although nicotine increased EEG arousal, ketamine produced electrocerebral signs of brain activation (decreased slow wave power) and sedation (decreased fast wave power and frequency), which were not affected by nicotine pretreatment and were evident only in non-smokers. Ketamine increased a number of self-report indices of subjective arousal, some of which were attenuated and potentiated by nicotine in smokers and non-smokers, respectively. These findings suggest that long-term (evidenced by smoker vs. non-smoker comparisons) and short-term (acute) nicotine exposure may alter NMDA receptor-mediated arousal and mood systems in a way that promotes nicotine dependence in smokers, and addresses neurobiological deficiencies in smokers with schizophrenia. |
| 1421 | Cosci, Fiammetta, Abrams, Kenneth, Schruers, Koen R J, Rickelt, Judith, Griez, Eric J L. Effect of nicotine on 35% CO2-induced anxiety: A study in healthy volunteers. Nicotine & tobacco research : official journal of the Society for Research on Nicotine and Tobacco. 2006;8:511.  **Abstract:** Panic disorder and cigarette smoking co-occur at a rate that exceeds what would be expected by chance. Theoretically, cigarette smoking may (a) attenuate panicky symptoms via cognitive factors or pharmacological action, (b) contribute to the development of panic disorder, or (c) share an etiological vulnerability with panic. The present study was aimed at testing whether nicotine has a direct influence on laboratory-elicited panic. In a placebo-controlled, double-blind, randomized, cross-over study, 33 healthy nonsmokers underwent a 35% CO2 challenge after transdermal administration of a nicotine patch on one test day and a placebo patch on another test day. Physiological measures (blood pressure, heart rate) and rating scale scores (Panic Symptom List [PSL], Visual Analog Scale of Anxiety, State-Trait Anxiety Inventory) were assessed. Compared with the placebo condition, nicotine increased diastolic blood pressure (p < .1), heart rate (p < .001), and PSL scores (p < .005) prior to the CO2 challenge but did not affect responding to the CO2 challenge itself. Results are consistent with the notion that nicotine promotes autonomic activation. However, the present study did not provide direct evidence that nicotine elicits panic in healthy volunteers. Replication in a clinical sample is warranted. |
| 1423 | Rennard, Stephen I, Glover, Elbert D, Leischow, Scott, Daughton, David M, Glover, Penny N, Muramoto, Myra, Franzon, Mikael, Danielsson, Tobias, Landfeldt, Björn, Westin, Ake. Efficacy of the nicotine inhaler in smoking reduction: A double-blind, randomized trial. Nicotine & tobacco research : official journal of the Society for Research on Nicotine and Tobacco. 2006;8:555.  **Abstract:** Many smokers are not ready to quit but are interested in changing their smoking behavior, particularly if such a change is associated with a reduction in health risk. The present study evaluated the efficacy of the nicotine inhaler in reducing smoking. Exploratory studies assessed whether reduction in smoking was associated with reduction in markers of disease risk. A total of 429 healthy smokers (smoking at least 20 cigarettes/day) were randomly assigned to either nicotine-containing or placebo inhalers, which subjects were allowed to use ad libitum for up to 1 year. The nicotine inhaler was significantly superior to placebo in achieving reduction in daily cigarette consumption by at least 50% after 4 months, compared with baseline (18% vs. 8%, p = .004). Active treatment promoted smoking cessation: 8% of subjects in the nicotine group and 1% in the placebo group were abstinent at month 15. Throughout the study, smoking reduction, per se, independent of treatment group, was associated with a statistically significant decrease in exhaled carbon monoxide and serum cotinine and thiocyanate. Smoking reduction also improved established risk markers for cardiovascular disease over 4 months. The incidence of adverse events did not differ significantly between the active and placebo groups. The most common treatment-related adverse events were throat irritation and cough. In conclusion, the nicotine inhaler can help smokers who are unable or unwilling to quit to reduce daily cigarette consumption, which may be a health benefit on its own and may further promote quitting. |
| 1427 | Clark, Matthew M, Hurt, Richard D, Croghan, Ivana T, Patten, Christi A, Novotny, Paul, Sloan, Jeff A, Dakhil, Shaker R, Croghan, Gary A, Wos, Edward J, Rowland, Kendrith M, Bernath, Albert, Morton, Roscoe F, Thomas, Sachdex P, Tschetter, Loren K, Garneau, Stewart, Stella, Philip J, Ebbert, Larry P, Wender, Donald B, Loprinzi, Charles L. The prevalence of weight concerns in a smoking abstinence clinical trial. Addictive behaviors. 2006;31:1144.  **Abstract:** Recent research has demonstrated there is a high prevalence of weight concerns in smokers and that smokers with weight concerns may respond poorly to treatment for tobacco dependence. Most studies have focused only on females or have consisted of small samples. In this study of a 12-week randomized trial of nicotine inhaler, bupropion or both for smoking cessation, 50% of the 1012 female smokers and 26% of the 680 male smokers, at study entry, were weight concerned. In examining the impact of weight concerns on the 12-week point-prevalence smoking abstinence, 26% of non-weight-concerned smokers quit smoking compared to 22% of weight-concerned smokers (p=0.06). This study, which includes a large sample of both genders, provides further evidence that approximately half of females who are seeking smoking cessation treatment are weight concerned and that one quarter of male smokers are weight concerned. Additionally, being weight concerned may impact the short-term success rates of stopping smoking using pharmacotherapy. |
| 1429 | Mündel, Toby, Jones, David A. Effect of transdermal nicotine administration on exercise endurance in men. Experimental physiology. 2006;91:705.  **Abstract:** Nicotine is widely reported to increase alertness, improve co-ordination and enhance cognitive performance; however, to our knowledge there have been no attempts to replicate these findings in relation to exercise endurance. The purpose of this study was to determine the effects nicotine might have on cycling endurance, perception of exertion and a range of physiological variables. With local ethics committee approval and having obtained informed consent, 12 healthy, non-smoking men (22 +/- 3 years; maximal O2 uptake, 56 +/- 6 ml kg(-1) min(-1), mean +/- s.d.) cycled to exhaustion at 18 degrees C and 65% of their peak aerobic power, wearing either a 7 mg transdermal nicotine patch (NIC) or a colour-matched placebo (PLA) in a randomized cross-over design; water was available ad libitum. Subjects were exercising at approximately 75% of their maximal O2 uptake with no differences in cadence between trials. Ten out of 12 subjects cycled for longer with NIC administration, and this resulted in a significant 17 +/- 7% improvement in performance (P < 0.05). No differences were observed for perceived exertion, heart rate or ventilation. There were no differences in concentrations of plasma glucose, lactate or circulating fatty acids. In the absence of any effect on peripheral markers, we conclude that nicotine prolongs endurance by a central mechanism. Possible modes of action are suggested. |
| 1430 | Ahluwalia, Jasjit S, Okuyemi, Kolawole, Nollen, Nicole, Choi, Won S, Kaur, Harsohena, Pulvers, Kim, Mayo, Matthew S. The effects of nicotine gum and counseling among African American light smokers: a 2 x 2 factorial design. Addiction (Abingdon, England). 2006;101:883.  **Abstract:** AIMApproximately 50% of African American smokers are light smokers (smoke < or = 10 cigarettes a day). The prevalence of light smoking in the United States is increasing, yet there has not been a single smoking cessation clinical trial targeting light smokers. The purpose of this 2 x 2 factorial, randomized clinical trial was to evaluate the efficacy of nicotine gum (2 mg versus placebo) and counseling (motivational interviewing versus health education) for African American light smokers.DESIGNParticipants were assigned randomly to one of four study arms: 2 mg nicotine gum plus health education (HE); 2 mg nicotine gum plus motivational interviewing (MI); placebo gum plus HE; and placebo gum plus MI.PARTICIPANTS AND SETTINGA total of 755 African American light smokers (66% female, mean age = 45) were enrolled at a community health center over a 16-month period.INTERVENTION AND MEASUREMENTSParticipants received an 8-week supply of nicotine gum and six counseling sessions during the course of the 26-week study. Biochemical measures included expired carbon monoxide (CO) and serum and salivary cotinine.FINDINGSSeven-day quit rates for nicotine gum were no better than for the placebo group (14.2% versus 11.1%, P = 0.232) at 6 months. However, a counseling effect emerged, with HE performing significantly better than MI (16.7% versus 8.5%, P < 0.001). These results were consistent across outcome time-points (weeks 1, 8, and 26).CONCLUSIONSResults highlight the potential positive impact of directive information and advice-oriented counseling on smoking cessation. Studies are needed to assess other interventions that may further improve quit rates among African American light smokers who are motivated to quit. |
| 1432 | Najem, Boutaïna, Houssière, Anne, Pathak, Atul, Janssen, Christophe, Lemogoum, Daniel, Xhaët, Olivier, Cuylits, Nicolas, van de Borne, Philippe. Acute cardiovascular and sympathetic effects of nicotine replacement therapy. Hypertension (Dallas, Tex. : 1979). 2006;47:1162.  **Abstract:** Sympathetic overactivity is implicated in the increased cardiovascular risk of cigarette smokers. Excitatory nicotinic receptors are present on peripheral chemoreceptor cells. Chemoreceptors located in the carotid and aortic bodies increase ventilation (Ve), blood pressure (BP), heart rate (HR), and sympathetic nerve activity to muscle circulation (MSNA) in response to hypoxia. We tested the hypothesis that nicotine replacement therapy (NRT) increases MSNA and chemoreceptor sensitivity to hypoxia. Sixteen young healthy smokers were included in the study (8 women). After a randomized and blinded sublingual administration of a 4-mg tablet of nicotine or placebo, we measured minute Ve, HR, mean BP, and MSNA during normoxia and 5 minutes of isocapnic hypoxia. Maximal voluntary end-expiratory apneas were performed at baseline and at the end of the fifth minute of hypoxia. Nicotine increased HR by 7+/-3 bpm, mean BP by 5+/-2 mm Hg, and MSNA by 4+/-1 bursts/min, whereas subjects breathed room air (all P<0.05). During hypoxia, nicotine also raised HR by 8+/-2 bpm, mean BP by 2+/-1 mm Hg, and MSNA by 7+/-2 bursts/min (all P<0.05). Nicotine increased MSNA during the apneas performed in normoxia and hypoxia (P<0.05). Nicotine also raised the product of systolic BP and HR, a marker of cardiac oxygen consumption, during normoxia, hypoxia, and the apneas (P<0.05). Ve, apnea duration, and O2 saturation during hypoxia and the apneas remained unaffected. In conclusion, sympathoexcitatory effects of NRT are not because of an increased chemoreflex sensitivity to hypoxia. NRT increases myocardial oxygen consumption in periods of reduced oxygen availability. |
| 1435 | Nollen, Nicole L, Mayo, Matthew S, Sanderson Cox, Lisa, Okuyemi, Kolawole S, Choi, Won S, Kaur, Harsohena, Ahluwalia, Jasjit S. Predictors of quitting among African American light smokers enrolled in a randomized, placebo-controlled trial. Journal of general internal medicine. 2006;21:590.  **Abstract:** OBJECTIVETo examine the predictors of quitting among African American (AA) light smokers (<10 cigarettes per day) enrolled in a smoking cessation trial.METHODSBaseline variables were analyzed as potential predictors from a 2 x 2 cessation trial in which participants were randomly assigned to 1 of 4 treatment groups: nicotine gum plus health education (HE) counseling, nicotine gum plus motivational interviewing (MI) counseling, placebo gum plus HE counseling, or placebo gum plus MI counseling. Chi-square tests, 2 sample t-tests, and multiple logistic regression analyses were used to identify predictors of cotinine (COT) verified abstinence at month 6.RESULTSIn the final regression model, HE rather than MI counseling (odds ratio [OR]=2.26%, 95% confidence interval [CI]=1.36 to 3.74), older age (OR=1.03%, 95% CI=1.01 to 1.06), and higher body mass index (OR=1.04%, 95% CI=1.01 to 1.07) significantly increased the likelihood of quitting, while female gender (OR=0.46%, 95% CI=0.28 to 0.76),</=,800/month income (OR=0.60%, 95% CI=0.37 to 0.97), higher baseline COT (OR=0.948%, 95% CI=0.946 to 0.950), and not completing all counseling sessions (OR=0.48%, 95% CI=0.27 to 0.84) reduced the odds of quitting.CONCLUSIONSIndividual characteristics may decrease the likelihood of quitting; however, the provision of directive, advice-oriented counseling focused on the addictive nature of nicotine, health consequences of smoking, benefits of quitting, and development of a concrete quit plan may be an important and effective facilitator of quitting among AA light smokers. |
| 1436 | Knott, Verner, Blais, Crystal, Scherling, Carole, Camarda, Jordan, Millar, Anne, Fisher, Derek, McIntosh, Judy. Neural effects of nicotine during auditory selective attention in smokers: an event-related potential study. Neuropsychobiology. 2006;53:115.  **Abstract:** Acute nicotine has been found to improve task performance in smokers after smoking abstinence, but the attentional processes mediating these improvements are unclear. Since scalp-recorded event-related potentials (ERPs) have been shown to be sensitive indicators of selective attention, the effects of acutely administered nicotine were examined on ERPs and concomitant behavioural performance measures in an auditory selective attention task. Ten (6 males) overnight smoking-abstinent cigarette smokers received nicotine gum (4 mg) in a randomized, double-blind, placebo-controlled, crossover design. In a dichotic listening task [which required participants to attend and detect (target) deviant stimuli in one ear and to ignore similar stimuli in the other ear] which included ERP recordings and assessment of response speed and accuracy measures, nicotine gum failed to alter behavioural performance or amplitudes of ERP components sensitive to selective attention [reflected in the N100 and negative difference (Nd) component] or to pre-attentive detection of acoustic change [reflected in the mismatch negativity (MMN) component]. However, nicotine did influence the speed of these voluntary selective processes, as reflected by shortened latencies of the early Nd component. The findings are discussed in relation to the stimulus filter theory of smoking, and with respect to nicotine's actions on involuntary and controlled aspects of selective attention processes. |
| 1441 | Evans, Sarah E, Blank, Melissa, Sams, Cynthia, Weaver, Michael F, Eissenberg, Thomas. Transdermal nicotine-induced tobacco abstinence symptom suppression: nicotine dose and smokers' gender. Experimental and clinical psychopharmacology. 2006;14:121.  **Abstract:** An aversive tobacco abstinence syndrome, thought to reflect an underlying level of nicotine dependence, contributes to cigarette smokers' failed quit attempts. Nicotine replacement therapy (NRT) suppresses tobacco abstinence, but high relapse rates suggest room for improvement. Improving NRT's efficacy might begin with identifying factors that influence tobacco abstinence symptom suppression. Two such factors are smokers' gender and NRT dose. The purpose of this study was to determine the dose-related effects of transdermal nicotine (TN) on tobacco abstinence symptoms in 75 men and 53 women who regularly smoked cigarettes but who had abstained from smoking for at least 8-12 hr. Participants completed 4 double-blind, randomized 6.5-hr laboratory sessions that differed by TN dose (0, 7, 21, or 42 mg). Each session included blood sampling for plasma nicotine level, measurement of heart rate, participants' ratings of tobacco abstinence symptoms and effects of nicotine, and psychomotor performance. Increases in plasma nicotine level were related to TN dose and were independent of gender. TN-induced abstinence symptom suppression was dose-related for items assessing craving and urge to smoke and largely was independent of gender. TN increased heart rate and ratings of aversive side effects (e.g., nausea, lightheadedness) in a dose-related manner, and women were more sensitive at higher doses. Results from this laboratory study support the continued use of TN as a pharmacotherapy. Higher doses may ameliorate some abstinence symptoms, although the side effect profile, at least in the short term, may limit effectiveness, especially for women. |
| 1443 | Sørensen, Lars T, Jorgensen, Lars N, Zillmer, Rikke, Vange, Jakob, Hemmingsen, Ulla, Gottrup, Finn. Transdermal nicotine patch enhances type I collagen synthesis in abstinent smokers. Wound repair and regeneration : official publication of the Wound Healing Society [and] the European Tissue Repair Society. 2006;14:247.  **Abstract:** Cigarette smokers deposit less collagen, expressed as hydroxyproline, in granulation tissue than nonsmokers. We studied the effect of abstinence from smoking and transdermal nicotine patches on deposition of hydroxyproline, proline, type I procollagen, and total proteins. Fifty-four healthy smokers were studied during 10 days of smoking and again from days 10 to 20 following smoking cessation. After the first 10 days of abstinence they were randomized to double-blind treatment with transdermal nicotine patches of 25 mg/day or placebo for a period of 10 days. During this period and during smoking, an expanded polytetrafluoroethylene tube was implanted into the subcutis. Following removal of the implant, total amino acids and peptides were extracted. Hydroxyproline and proline were analyzed by high-pressure liquid chromatography, type I procollagen was analyzed by enzyme-linked immunoassay, and total proteins were determined colorimetrically. In the 39 subjects who complied with the study protocol, abstinence from smoking did not affect the deposition of hydroxyproline, proline, type I procollagen, or total protein in the implants. During abstinence, the type I procollagen level increased by 18% in the transdermal nicotine patches group and decreased by 10% in the placebo group (p<0.05). We conclude that 20 days of abstinence from smoking does not affect collagen deposition in granulation tissue. However, in abstinent smokers, transdermal nicotine patches appears to increase type I collagen synthesis. |
| 1445 | Hotham, Elizabeth D, Gilbert, Andrew L, Atkinson, Elinor R. A randomised-controlled pilot study using nicotine patches with pregnant women. Addictive behaviors. 2006;31:641.  **Abstract:** Stopping smoking in pregnancy is a public health priority and a clinical imperative. However, many women who have not been able to 'quit' in early pregnancy find it very difficult to do so. This randomised-controlled pilot study examined feasibility issues in offering free nicotine patches with counselling to a group of 20 mid-trimester pregnant women at the Women's and Children's Hospital, Adelaide. A further 20 were offered counselling only. Smoking status at each visit was measured by self-report, carbon monoxide monitoring, and salivary cotinine. The most common pattern (eleven of the twenty women) was intermittent patch use. Only five women used patches continuously up to the 12 week maximum available. Three women in the patch group were abstinent at delivery compared with none in the control group. Notable features of the study were the low interest in participation and the high withdrawal rate. Nicotine patches may not be highly useful for pregnant women. Continuing tobacco control measures and customized support for women and their partners, often smokers, may prove more fruitful. |
| 1446 | Halpin, Helen Ann, McMenamin, Sara B, Rideout, Jeffrey, Boyce-Smith, Gifford. The costs and effectiveness of different benefit designs for treating tobacco dependence: results from a randomized trial. Inquiry : a journal of medical care organization, provision and financing. 2006;43:54.  **Abstract:** This research estimated the costs and effectiveness of three different benefit designs for treating tobacco dependence: drugs only (nicotine replacement therapy patch, nasal spray, inhaler, and Zyban); drugs and counseling (drugs and proactive telephone counseling); and drugs if counseling (drugs conditional on enrollment in counseling). A sample of 393 adult smokers enrolled in a California preferred provider organization was randomly assigned to one of three study groups. After eight months, there were no significant increases in quit attempts or quit rates in the groups with covered drugs and counseling compared to the group with drug coverage only. Therefore, costs rose with no increase in quit rates when proactive telephone counseling was added to coverage of pharmacotherapy, regardless of benefit design. |
| 1447 | Shiffman, Saul, Scharf, Deborah M, Shadel, William G, Gwaltney, Chad J, Dang, Qianyu, Paton, Stephanie M, Clark, Duncan B. Analyzing milestones in smoking cessation: illustration in a nicotine patch trial in adult smokers. Journal of consulting and clinical psychology. 2006;74:276.  **Abstract:** Tests of addiction treatments seldom reveal where treatment exercises its effect (i.e., promoting initial abstinence, preventing lapses, and/or impeding progression from lapse to relapse). The authors illustrate analyses distinguishing effects on these milestones in a randomized trial of high-dose nicotine patch (35 mg; n = 188) versus placebo (n = 136) in adult smokers, who used electronic diaries to monitor smoking in real time during 5 weeks of treatment. High-dose patch promoted initial abstinence (hazard ratio [HR] = 1.3) and decreased the risk of lapsing among those who achieved abstinence (HR = 1.6). The biggest effect of treatment was to prevent progression to relapse among those who had lapsed (HR = 7.1). Analysis of effects by milestones may enhance understanding of cessation treatments and their mechanisms of action. |
| 1451 | Knott, Verner J, Scherling, Carole S, Blais, Crystal M, Camarda, Jordan, Fisher, Derek J, Millar, Anne, McIntosh, Judy F. Acute nicotine fails to alter event-related potential or behavioral performance indices of auditory distraction in cigarette smokers. Nicotine & tobacco research : official journal of the Society for Research on Nicotine and Tobacco. 2006;8:263.  **Abstract:** Behavioral studies have shown that nicotine enhances performance in sustained attention tasks, but they have not shown convincing support for the effects of nicotine on tasks requiring selective attention or attentional control under conditions of distraction. We investigated distractibility in 14 smokers (7 females) with event-related brain potentials (ERPs) and behavioral performance measures extracted from an auditory discrimination task requiring a choice reaction time response to short- and long-duration tones, both with and without embedded deviants. Nicotine gum (4 mg), administered in a randomized, double-blind, placebo-controlled crossover design, failed to counter deviant-elicited behavioral distraction (i.e., slower reaction times and increased response errors), and it did not influence the distracter-elicited mismatch negativity, the P300a, or the reorienting negativity ERP components reflecting acoustic change detection, involuntary attentional switching, and attentional reorienting, respectively. Results are discussed in relation to a stimulus-filter model of smoking and in relation to future research directions. |
| 1453 | Demazières, A, Luthringer, R, Coppel, E, Gilles, C, Fleury, C, Roegel, J C, Delarue, A, Laur, C, Lagrue, G. Ability of a new oral nicotine substitute to reduce smoking urge in moderate smokers. Addictive behaviors. 2006;31:537.  **Abstract:** The aim of this study was to assess the effectiveness of a new nicotine lozenge ( Nicopass 1.5 mg) in reducing smoking urge after an overnight abstinence. Twenty-four moderate smokers participated in a randomized, double-blind, placebo-controlled, 2-period crossover trial. The results showed that 1.5 mg-nicotine lozenge is superior to placebo in reducing smoking urge (p = 0.0001). In addition, nicotine lozenge, but not placebo, significantly improved vigilance and psychomotor performances (p < 0.05) and displayed a cardiac chronotropic effect. Thus, the 1.5-mg nicotine lozenge appears as an effective aid to alleviate acute tobacco withdrawal symptoms in moderate smokers. |
| 1454 | Shiffman, Saul, Ferguson, Stuart G, Gwaltney, Chad J, Balabanis, Mark H, Shadel, William G. Reduction of abstinence-induced withdrawal and craving using high-dose nicotine replacement therapy. Psychopharmacology. 2006;184:637.  **Abstract:** RATIONALEDecreasing withdrawal and craving during smoking cessation is a major aim of cessation medications. Prior studies have shown that Nicotine Replacement Therapy (NRT) decreases withdrawal symptom severity but have relied on retrospective reports and lacked robust measures of baseline symptoms or symptoms during unmedicated abstinence.OBJECTIVES AND METHODSWe tested the effect of high-dose (35 mg) nicotine patch on withdrawal and craving during abstinence using real-time assessment with electronic diaries during ad libitum smoking, a brief period of experimentally directed trial abstinence, and the first 3 days of cessation. Subjects were 324 smokers randomized to high-dose nicotine patches or placebo.RESULTSTreatment with active patches reduced withdrawal and craving during cessation and completely eliminated deprivation-related changes in affect or concentration.CONCLUSIONHigh-dose NRT reduces withdrawal symptoms and craving and can eliminate some symptoms entirely. |
| 1456 | Munafò, Marcus R, Johnstone, Elaine C, Wileyto, E Paul, Shields, Peter G, Elliot, Katherine M, Lerman, Caryn. Lack of association of 5-HTTLPR genotype with smoking cessation in a nicotine replacement therapy randomized trial. Cancer epidemiology, biomarkers & prevention : a publication of the American Association for Cancer Research, cosponsored by the American Society of Preventive Oncology. 2006;15:398.  **Abstract:** |
| 1458 | Gehricke, Jean-G, Whalen, Carol K, Jamner, Larry D, Wigal, Tim L, Steinhoff, Kenneth. The reinforcing effects of nicotine and stimulant medication in the everyday lives of adult smokers with ADHD: A preliminary examination. Nicotine & tobacco research : official journal of the Society for Research on Nicotine and Tobacco. 2006;8:37.  **Abstract:** Whereas the smoking prevalence rates in the general population are declining, rates among people diagnosed with attention-deficit/hyperactivity disorder (ADHD) continue to be elevated. Previous research has shown that nicotine may improve attention and mood, suggesting that nicotine may help ameliorate the attentional and emotional problems associated with ADHD. The present study examined the effects of nicotine with and without stimulant medication on ADHD symptoms, moods, and arousal in the everyday lives of smokers with ADHD. A total of 10 smokers with ADHD who were being treated with stimulant medication were asked to abstain from smoking while participating in the study. Participants underwent four conditions in randomized order: (a) Nicotine patch+stimulant medication, (b) nicotine patch only, (c) placebo patch+stimulant medication, and (d) placebo patch only. Each condition continued for 2 days, during which self-reports of ADHD symptoms and moods were obtained using electronic diaries. Lightweight ambulatory monitors recorded cardiovascular activity at each diary entry. Smoking abstinence was verified by expired carbon monoxide and salivary cotinine analysis. Results showed that nicotine patches and stimulant medication alone and in combination reduced difficulty concentrating and core ADHD symptoms compared with placebo patch only. Borderline improvement in impatience and self-control was seen with nicotine patch administration primarily on day 1. Nicotine patches also tended to elevate systolic and diastolic blood pressure compared with placebo patch during day 2. The findings suggest that smokers with ADHD experience nicotine-related reductions in ADHD symptoms during their everyday lives. |
| 1464 | Baldeweg, Torsten, Wong, Davina, Stephan, Klaas E. Nicotinic modulation of human auditory sensory memory: Evidence from mismatch negativity potentials. International journal of psychophysiology : official journal of the International Organization of Psychophysiology. 2006;59:49.  **Abstract:** Impairment in mismatch negativity (MMN) generation is a robust biological marker of schizophrenia. Understanding the physiological and pharmacological processes involved in its generation may therefore advance our understanding of this complex disorder. The present study tested if acute administration of nicotine modulates human auditory sensory memory as measured with MMN. ERP responses to tone duration deviants were recorded using a stimulation protocol with continuously changing (roving) standard stimuli in order to measure the effect of stimulus repetitions on encoding of new stimuli (MMN memory trace effect). Twenty healthy adult volunteers were randomly assigned to receive either a nicotine gum or placebo after a baseline ERP recording. Nicotine administration augmented MMN amplitude in the treatment group compared to the baseline recording, while no MMN change was found in the placebo group. The drug effect was due to a selective enhancement of a frontal positive potential to standard stimuli (from 80-200 ms post-stimulus), while the negativity to deviants remained unaffected. Furthermore, under nicotine stimulation this repetition positivity showed a more marked increase with stimulus repetition compared to baseline and placebo. These results have potential implications for schizophrenia by suggesting that nicotinic agonists could ameliorate patients' MMN deficits by improving stimulus encoding and sensory memory trace formation. |
| 1470 | Roozen, Hendrik G, Van Beers, Suzanne E C, Weevers, Harm Jan A, Breteler, Marinus H M, Willemsen, Marc C, Postmus, Pieter E, Kerkhof, Ad J F M. Effects on smoking cessation: naltrexone combined with a cognitive behavioral treatment based on the community reinforcement approach. Substance use & misuse. 2006;41:45.  **Abstract:** A promising option in substance abuse treatment is the Community Reinforcement Approach (CRA). The opioid antagonist naltrexone (NTX) may work in combination with nicotine replacement therapy (NRT) to block the effects of smoking stimuli in abstinent smokers. Effects of lower doses than 50 mg/dd. have not been reported. A study was conducted in Amsterdam in 2000/2001 with the objective to explore the effects of the combination NTX (25/50-mg dd.), NRT, and CRA in terms of craving and abstinence. In a randomized open label, 2 x 2 between subjects design, 25 recovered spontaneous pneumothorax (SP) participants received 8 weeks of treatment. Due to side effects, only 3 participants were compliant in the 50-mg NTX condition. Craving significantly declined between each measurement and there was a significant interaction between decline in craving and craving measured at baseline. The abstinence rate in the CRA group was nearly double that in the non-psychosocial therapy group (46% vs. 25%; NS) at 3 months follow-up after treatment. |
| 1471 | Korhonen, Tellervo, Kinnunen, Taru, Quiles, Zandra, Leeman, Robert F, Terwal, Donna Medaglia, Garvey, Arthur J. Cardiovascular risk behavior among sedentary female smokers and smoking cessation outcomes. Tobacco induced diseases. 2005;3:7.  **Abstract:** BACKGROUNDWe examined female sedentary smokers' additional cardiovascular disease (CVD) risk behaviors and their associations to smoking cessation.METHODSThis study was part of a randomized controlled trial testing the effectiveness of exercise and nicotine gum in smoking cessation. Included in the analyses were 148 participants. Dietary habits and alcohol consumption were measured as additional CVD risk behaviors. High-fat diet and heavy alcohol use were considered those risk behaviors. Nicotine dependence, length of the longest quit attempt, depressive symptoms, self-efficacy, and education were examined as other baseline variables. Abstinence from tobacco was recorded through 12 months.RESULTSDiet was related to depressive symptoms at baseline. Alcohol use was related to nicotine dependence and education level. Heavy alcohol use alone and accumulation of two added risk behaviors predicted poorer smoking cessation outcome. Although diet alone was not associated with cessation outcome the high-fat diet interacted with depressive symptoms, such that the depressed women with high-fat diet were significantly more likely to relapse in their quit attempt compared to other subgroups.CONCLUSIONNon-moderate alcohol use alone and accumulation of multiple CVD risk behaviors seem to be associated with lower success in smoking cessation. |
| 1472 | Batra, Anil, Klingler, Karl, Landfeldt, Björn, Friederich, Hubertus M, Westin, Ake, Danielsson, Tobias. Smoking reduction treatment with 4-mg nicotine gum: a double-blind, randomized, placebo-controlled study. Clinical pharmacology and therapeutics. 2005;78:689.  **Abstract:** BACKGROUNDSmoking reduction may provide a harm-reduction alternative treatment for smokers who are not ready to quit smoking. This study evaluated the efficacy of nicotine gum in helping smokers reduce or quit smoking.METHODSThis randomized, double-blind, placebo-controlled trial involved 364 smokers who were not ready to quit but were willing to reduce their smoking intensity. Participants received either 4-mg nicotine gum (n = 184) or placebo gum (n = 180) as desired for up to 12 months. The primary outcome was sustained smoking reduction, which was defined as a decrease in daily cigarette consumption of at least 50% compared with baseline. Secondary measures included point-prevalence abstinence, intention to quit, and cardiovascular risk markers.RESULTSAt 4 months, the sustained smoking reduction rate in the nicotine gum group was twice that of the placebo group (15.8% versus 6.7%, P = .008). Point-prevalence abstinence was 6.6% for the nicotine gum group and 2.2% for the placebo group (P = .07). At 13 months, there was a significant difference in the smoking reduction rate for the nicotine (8.2%) and placebo (2.8%) groups (P = .036). At month 13, the abstinence rates were 12% and 4.5% for the nicotine and placebo groups, respectively (P = .012). Concomitant use of nicotine gum and cigarette smoking was well tolerated. Carbon monoxide levels decreased significantly (P = .01).CONCLUSIONNicotine gum may be an efficacious harm-reduction alternative for smokers who are not ready to quit and may promote smoking cessation, the ultimate goal in the treatment of tobacco dependence. |
| 1473 | Marcus, Bess H, Lewis, Beth A, Hogan, Joseph, King, Teresa K, Albrecht, Anna E, Bock, Beth, Parisi, Alfred F, Niaura, Raymond, Abrams, David B. The efficacy of moderate-intensity exercise as an aid for smoking cessation in women: a randomized controlled trial. Nicotine & tobacco research : official journal of the Society for Research on Nicotine and Tobacco. 2005;7:871.  **Abstract:** Evidence suggests that vigorous-intensity exercise interventions may be effective for smoking cessation among women; however, few studies have examined the efficacy of a moderate-intensity exercise program. The present study examined the efficacy of moderate-intensity exercise for smoking cessation among female smokers. Healthy, sedentary female smokers (N = 217) were randomly assigned to an 8-week cognitive-behavioral smoking cessation program plus moderate-intensity exercise (CBT+EX) or to the same cessation program plus equal contact (CBT). A subsample received nicotine replacement therapy. Results indicated that the CBT+EX and CBT groups were equally likely to attain smoking cessation at the end of treatment, as measured by cotinine-verified 7-day point-prevalence abstinence (20.2% for CBT+EX vs. 18.5% for CBT). The CBT+EX group was more likely to report smoking cessation, as measured by 7-day point prevalence at the 3-month follow-up (11.9% vs. 4.6%, p<.05), compared with the CBT group. No group differences were found at 12 months by either 7-day point prevalence (7.3% for CBT+EX vs. 8.3% for CBT) or continuous abstinence (0.9% for CBT+EX vs. 0.9% for CBT). Additionally, among participants in the CBT+EX group, those with higher adherence to the exercise prescription were significantly more likely to achieve smoking cessation at the end of treatment than were participants reporting lower adherence to exercise. Our findings indicate that the empirical support for moderate-intensity exercise as an adjunctive treatment to CBT for smoking cessation may be limited. Perhaps future studies could compare moderate- vs. vigorous-intensity physical activity to test their relative efficacy. |
| 1477 | Ingram, J R, Rhodes, J, Collins, P W, Williams, G T, Newcombe, R G, Thomas, G A O. Plasma fibrinogen in ulcerative colitis: the effect of disease activity and nicotine therapy in a randomised controlled trial. Digestive and liver disease : official journal of the Italian Society of Gastroenterology and the Italian Association for the Study of the Liver. 2005;37:832.  **Abstract:** BACKGROUNDSmoking increases plasma fibrinogen and cardiovascular risk whereas transdermal nicotine may not. Fibrinogen is an acute phase protein and may reflect disease activity in ulcerative colitis.AIMSTo examine the effect of topical nicotine on plasma fibrinogen and any relationship between fibrinogen and ulcerative colitis disease activity.PATIENTSForty-eight non-smokers with moderately active ulcerative colitis.METHODSPatients were randomised to 6 mg nicotine enema or placebo for 6 weeks, followed by open nicotine therapy for 4 weeks. Plasma fibrinogen was measured at baseline and after 6 and 10 weeks; at each assessment sigmoidoscopy with a rectal biopsy was performed. RESULTS.: At 6 weeks median plasma fibrinogen was 3.30 g/l on nicotine compared to 3.05 g/l on placebo, P = 0.90 when adjusted for baseline values. There was a correlation between fibrinogen and the UC disease activity index (UCDAI) at weeks 0 and 10, P = 0.036 and 0.033, respectively, and between fibrinogen and sigmoidoscopic grade at each assessment, P = 0.014, 0.021 and 0.034. Changes in fibrinogen did not correlate with changes in disease severity.CONCLUSIONSThere was no significant effect of nicotine enemas, in either direction, on plasma fibrinogen-this was raised in moderately active UC and correlated with the sigmoidoscopic grade of colitis and the UCDAI; however, fibrinogen was not sufficiently sensitive to be of practical clinical value. |
| 1479 | Hatsukami, Dorothy K, Kotlyar, Michael, Allen, Sharon, Jensen, Joni, Li, Shelby, Le, Chap, Murphy, Sharon. Effects of cigarette reduction on cardiovascular risk factors and subjective measures. Chest. 2005;128:2528.  **Abstract:** STUDY OBJECTIVESTo assess the effect of continued smoking and smoking reduction on cardiovascular biomarkers (eg, WBC count, cholesterol concentrations, BP, heart rate).DESIGN, SETTING, AND PARTICIPANTSThis study, conducted at the University of Minnesota, randomized smokers interested in significantly reducing cigarette use but not quitting to either start 12 weeks of smoking reduction immediately (n = 102), assisted by nicotine replacement therapy, or to a 6-week wait list (n = 49). Those starting smoking reduction were required to reduce smoking by 25% for 2 weeks, 50% for 2 weeks, and 75% during the final 2 weeks. After 6 weeks, the subjects were asked to maintain a 50% reduction or quit. Nicotine gum and, if necessary, nicotine patch were used to achieve reduction goals. The wait list group (n = 49) smoked ad libitum for 6 weeks and then reduced smoking as previously described.MEASUREMENTS AND RESULTSCardiovascular biomarkers (eg, WBC count, cholesterol concentrations, BP, heart rate) were assessed at several time points after enrollment. During ad libitum smoking, cardiovascular biomarkers remained relatively stable with correlation coefficients across the various time measurements, ranging from 0.44 to 1.00 (p < 0.01 for all measures). Among successful nonabstinent reducers (64 of 151 subjects), significant improvements were found in many biomarkers (eg, hemoglobin, hematocrit, RBC and WBC counts, lipids, BP, heart rate, respiratory symptoms, all p < 0.0167).CONCLUSIONSThese results show the availability of reliable and dose-sensitive biomarkers and that reduction in smoking can lead to significant but only modest changes in cardiovascular risk factors in healthy smokers. It is not known whether the reductions in cardiovascular risk factors observed after smoking reduction are also associated with reduced disease risk. Additional research is necessary to address this issue. |
| 1483 | Emmons, Karen M, Puleo, Elaine, Park, Elyse, Gritz, Ellen R, Butterfield, Rita M, Weeks, Jane C, Mertens, Ann, Li, Frederick P. Peer-delivered smoking counseling for childhood cancer survivors increases rate of cessation: the partnership for health study. Journal of clinical oncology : official journal of the American Society of Clinical Oncology. 2005;23:6516.  **Abstract:** PURPOSECancer survivors smoke at rates that are only slightly lower than the general population. This article reports on the final outcomes of Partnership for Health, a smoking cessation intervention for smokers in the Childhood Cancer Survivors Study (CCSS).METHODSThis study is a randomized control trial with follow-up at 8 and 12 months that involved smokers (n = 796) enrolled onto the CCSS cohort. Participants were randomly assigned to either a self-help or a peer-counseling program that included up to six telephone calls from a trained childhood cancer survivor, tailored and targeted materials, and free nicotine replacement therapy. The intervention was delivered by telephone and postal service mail.RESULTSThe quit rate was significantly higher in the counseling group compared with the self-help group at both the 8-month (16.8% v 8.5%; P < .01) and 12-month follow-ups (15% v 9%; P < or = .01). Controlling for baseline self-efficacy and readiness to change, the intervention group was twice as likely to quit smoking, compared with the self-help group. Smoking cessation rate increased with an increase in the number of counseling calls. The cost of delivering the intervention was approximately 300 dollars per participant. The incremental cost-effectiveness of the intervention compared with controls was 5,371 dollars per additional quit.CONCLUSIONInterventions to prevent future illnesses are of critical importance to childhood cancer survivors. The Partnership for Health intervention resulted in a doubling of smoking cessation quit rates. Because of the seriousness of smoking among childhood cancer survivors, this intervention model may be appropriate as a multicomponent treatment program for survivors who smoke. |
| 1484 | Hotham, Elizabeth D, Gilbert, Andrew L, Atkinson, Elinor R. Case studies of three pregnant smokers and their use of nicotine replacement therapy. Midwifery. 2005;21:224.  **Abstract:** OBJECTIVETo examine the barriers encountered by pregnant women who attempt to stop smoking by highlighting three women who used nicotine patches.DESIGNA randomised-controlled trial of nicotine-replacement therapy (NRT) in the form of patches to test its acceptability for pregnant women. Ethics approval was granted despite NRT being contraindicated in Australia for pregnant women and having a low safety rating (category D) (Australian Drug Evaluation Committee, 1999). Salivary cotinine levels were used to assess nicotine exposure and provide some indicator of NRT safety. All participants were given pregnancy-specific cessation counselling, and the 20 women in the treatment arm were offered nicotine patches (15 mg/16 hr), with the option of weaning to lower strength patches if desired.SETTINGThe Women's and Children's Hospital, Adelaide, a public tertiary teaching hospital in South Australia, with almost 4000 births annually.SAMPLE40 'high-risk' pregnant smokers who expressed interest in stopping smoking.FINDINGSAs has been found in the general population, 'quit' rates with NRT use were low. Only three of the participants in this study, who became abstinent with patch use during pregnancy, were still abstinent at birth. The circumstances of two of these women, and a third woman who used patches to 'control' her smoking despite researcher advice, are detailed here. Only one of the two women 'abstinent at delivery' was still abstinent 3 months after birth, the last contact point of the study.KEY CONCLUSIONS AND IMPLICATIONS FOR PRACTICEAlthough health providers intuitively regard pregnancy as an appropriate time for women to stop smoking, the stressors during pregnancy seem to militate against cessation. This study does not indicate that use of NRT will provide an easier solution. It may be more fruitful to institute a concerted lifestyle approach with both the woman and her partner (or significant household members), and continue this support and education postnatally if cessation has not been achieved. Health professionals should also support better-targeted public health campaigns and tobacco-control initiatives generally, because, undoubtedly, the social environment is a major determinant of initiation and continuation of smoking. |
| 1485 | Allen, Sharon S, Hatsukami, Dorothy, Brintnell, Dawn M, Bade, Tracy. Effect of nicotine replacement therapy on post-cessation weight gain and nutrient intake: a randomized controlled trial of postmenopausal female smokers. Addictive behaviors. 2005;30:1273.  **Abstract:** This study of 94 postmenopausal female smokers evaluated the effect of nicotine replacement therapy (NRT) and hormone therapy (HT) on change in weight, energy intake, and physical activity during 2 weeks of smoking abstinence. Women, stratified by current use of HT, were randomized to nicotine or placebo patch. After 2 weeks of abstinence, women on nicotine patch had significantly larger increases in total caloric and fat intake than women on placebo patch and a trend toward larger increases in carbohydrates (total and sweet). Conversely, the nicotine group had less weight gain, 0.47 kg, than the placebo group, 1.02 kg (F=10.31, p=0.002). No effects were observed for hormone therapy. It appears that in short-term smoking abstinence, postmenopausal women on NRT gain less weight than do women on placebo, in spite of consuming more calories. This may be beneficial in the critical first 1-2 weeks of tobacco cessation, especially in light of postmenopausal weight gain. |
| 1491 | Jessen, A, Buemann, B, Toubro, S, Skovgaard, I M, Astrup, A. The appetite-suppressant effect of nicotine is enhanced by caffeine. Diabetes, obesity & metabolism. 2005;7:327.  **Abstract:** AIMTo test whether the anorectic effect of nicotine may be amplified by caffeine.METHODSChewing gums with nicotine and caffeine were administered to 12 healthy young men of normal weight. Different combinations of 0, 1 or 2 mg of nicotine and 0, 50 or 100 mg of caffeine were applied during a 2-h period in a randomized, double blind, cross over design. Appetite sensations were measured using visual analogue scales.RESULTSHunger and prospective food consumption were negatively associated with the increasing doses of nicotine, whereas satiety and fullness were positively associated with the increasing doses of nicotine (p < 0.05). Caffeine appeared to amplify the effects of nicotine on hunger and fullness as a caffeine x nicotine x time interaction was observed in these scores (p < 0.05). The 2-mg dose of nicotine in combination with the 100-mg dose of caffeine caused nausea in four of the non-smokers. However, the effects of nicotine and the caffeine x nicotine x time interaction persisted after the exclusion of these subjects.CONCLUSIONCaffeine added to nicotine chewing gum appears to amplify its attenuating effects on appetite and the combinations of 1-mg of nicotine with caffeine seem to be well tolerated. |
| 1493 | Warner, David O, Patten, Christi A, Ames, Steven C, Offord, Kenneth P, Schroeder, Darrell R. Effect of nicotine replacement therapy on stress and smoking behavior in surgical patients. Anesthesiology. 2005;102:1138.  **Abstract:** BACKGROUNDMany surgical patients are dependent on nicotine. Smoke-free policies in healthcare facilities mandate abstinence from smoking, which could contribute to psychological stress in the perioperative period. The authors tested the hypothesis that nicotine replacement therapy decreases psychological stress in cigarette smokers scheduled to undergo elective surgery and determined whether nicotine replacement therapy affects postoperative smoking behavior, even when not specifically prescribed to promote abstinence.METHODSIn this double-blind, placebo-controlled trial, 121 smokers, of whom 116 received a study intervention, were randomly assigned to receive either active (nicotine-containing) or placebo patches, beginning on the morning of surgery and continuing for up to 30 days after discharge from the hospital. Outcomes included the Perceived Stress Score, the Nicotine Withdrawal Score, and subject self-report of smoking behavior.RESULTSThe Perceived Stress Score and the Nicotine Withdrawal Score did not change significantly from baseline over the immediate perioperative period and did not differ between active or placebo patch groups (all P > 0.19). The percentage of placebo versus active patch subjects reporting 7-day abstinence at 30 days postoperatively (30% vs. 39%; P = 0.29) did not differ significantly between groups. At 30 days postoperatively, subjects in both groups significantly reduced their cigarettes smoked per day from baseline, but those receiving active patches reported a greater decrease (a mean decrease of 11 +/- 11 vs. 15 +/- 7 cigarettes/day in placebo and active groups; P = 0.045).CONCLUSIONRoutine nicotine replacement therapy is not indicated in smokers undergoing surgery for the purposes of managing nicotine withdrawal and stress but can modify some aspects of postoperative smoking behavior. |
| 1494 | Nagle, Amanda L, Hensley, Michael J, Schofield, Margot J, Koschel, Alison J. A randomised controlled trial to evaluate the efficacy of a nurse-provided intervention for hospitalised smokers. Australian and New Zealand journal of public health. 2005;29:285.  **Abstract:** OBJECTIVEDoes the provision of a nurse-based intervention lead to smoking cessation in hospital patients?METHODSAt tertiary teaching hospital in Newcastle, Australia, 4,779 eligible (aged 18-80, admitted for at least 24 hours, and able to provide informed consent) and consenting (73.4%) in-patients were recruited into a larger cross-sectional survey. 1,422 (29.7%) smokers (in the last 12 months) were randomly assigned to control (n = 711) or intervention group (n = 711). The brief nurse-delivered intervention incorporated: tailored information, assessment of withdrawal, offer of nicotine replacement therapy, booklets, and a discharge letter. Self-reported cessation at 12 months was validated with CO and salivary cotinine.RESULTSThere were no significant differences between groups in self-reported abstinence at three or 12 months post intervention, based on an intention to treat analysis. At three months, self-reported abstinence was 27.3% (I) and 27.5% (C); at 12 months was 18.5% (I) and 20.6% (C). There were no differences in validation of self-report between intervention and control groups at 12 months.CONCLUSIONThis brief nurse-provided in-patient intervention did not significantly increase the smoking cessation rates compared with the control group at either three or 12-month follow-up.IMPLICATIONSA systematic total quality improvement model of accountable outcome-focused treatment, incorporating assertive physician-led pharmacotherapy, routine assessment and recording of nicotine dependence (ICD 10 coding), in- and outpatient services and engagement from multidisciplinary teams of health professionals may be required to improve treatment modalities for this chronic addictive disorder. |
| 1499 | Wiseman, Eve J, Williams, D K, McMillan, D E. Effectiveness of payment for reduced carbon monoxide levels and noncontingent payments on smoking behaviors in cocaine-abusing outpatients wearing nicotine or placebo patches. Experimental and clinical psychopharmacology. 2005;13:102.  **Abstract:** In a 2-week intervention to reduce cigarette smoking among outpatients in treatment for cocaine addiction, 20 subjects were randomly assigned to a contingent group, receiving monetary vouchers for breath samples with carbon monoxide (CO) levels of 8 ppm or less, or to a noncontingent group, receiving vouchers regardless of CO level. Subjects wore either nicotine or placebo patches in a randomized crossover design. Contingent subjects had significantly lower CO levels and met the 8 ppm target significantly more often than did noncontingent subjects; however, number of cigarettes reported smoked did not differ between groups. Use of nicotine patches resulted in CO levels significantly lower than did use of placebo patches, but levels still exceeded 8 ppm regardless of type of patch. Because contingent reward helped cocaine-dependent smokers achieve nonsmoking CO targets, behavioral antismoking interventions merit continued study in similar populations. |
| 1506 | Dar, Reuven, Stronguin, Florencia, Etter, Jean-Francois. Assigned versus perceived placebo effects in nicotine replacement therapy for smoking reduction in Swiss smokers. Journal of consulting and clinical psychology. 2005;73:350.  **Abstract:** In this report, the authors explore the relationships of perceived treatment to outcome in a large, placebo-controlled trial of nicotine replacement treatment for smoking reduction. In the original study (J. F. Etter, E. Laszlo, J. P. Zellweger, C. Perrot, & T. V. Perneger, 2002), which was conducted in French-speaking Switzerland, smokers were randomly assigned to receive nicotine, matching placebo products, or no intervention. At the end of the 6-month study, participants were asked to guess whether they had received nicotine or placebo. In the present analysis, the authors examined the difference in smoking reduction between those who believed they had received nicotine and those who believed they had received placebo. Regardless of actual treatment, smokers who believed they had received nicotine had significantly better outcome than those who believed they had received placebo. |
| 1507 | Thiel, Christiane M, Zilles, Karl, Fink, Gereon R. Nicotine modulates reorienting of visuospatial attention and neural activity in human parietal cortex. Neuropsychopharmacology : official publication of the American College of Neuropsychopharmacology. 2005;30:810.  **Abstract:** Prior studies in animals and humans indicate that reorienting of visuospatial attention is modulated by the cholinergic agonist nicotine. We have previously identified neural correlates of alerting and reorienting attention in humans and found that the parietal cortex is specifically involved in reorienting. This study investigates whether the alerting and reorienting systems, especially in the parietal cortex, are modulated by nicotine. We used event-related functional magnetic resonance imaging (fMRI) and studied 15 nonsmoking volunteers under placebo and nicotine (NICORETTE) polacrilex gum 1 and 2 mg). Subjects performed a cued target detection task with four different types of randomly intermixed trials (no, neutral, valid, and invalid cue trials). Alerting was captured by comparing BOLD activity and reaction times (RTs) in neutrally cued trials with no cue trials. Reorienting was isolated by comparing invalidly with validly cued trials. On the behavioral level, nicotine affected reorienting of attention by speeding RTs in invalidly cued trials; alerting was not affected by nicotine. Neurally, however, nicotine modulated both attentional systems. Pharmacologic effects on alerting-related brain activity were mainly evident as modulation of BOLD responses in the right angular gyrus and right middle frontal gyrus due to a reduction of neural activity in no cue trials. In the reorienting system, effects of nicotine were mainly evident in the left intraparietal sulcus and precuneus and due to a reduction of neural activity in invalidly cued trials. We conclude that nicotine enhances reorienting of attention in visuospatial tasks and that one behavioral correlate of speeded RTs is reduced parietal activity. |
| 1513 | Shiffman, Saul. Nicotine lozenge efficacy in light smokers. Drug and alcohol dependence. 2005;77:311.  **Abstract:** BACKGROUNDNicotine replacement therapy (NRT) has been proven to be effective in heterogeneous groups of smokers. However, analyses have not specifically examined efficacy among light smokers (< or =15 cigarettes per day). The objective of this study is to assess the efficacy of a nicotine lozenge in light smokers.METHODSWe conducted a secondary analysis of a randomized, placebo-controlled clinical trial contrasting active 2mg nicotine lozenge with placebo, and contrasting light smokers (< or =15 cigarettes per day) with moderate-heavy smokers (>15 cigarettes per day). Participants were 917 smokers who smoked their first cigarette >30 min after waking, and were randomized to active (n = 459) or placebo (n = 458) lozenge. Biochemically verified continuous abstinence was measured at 6 weeks and 1 year.RESULTSNicotine lozenge significantly increased quit rates relative to placebo at 6 weeks (45.7% versus 31.1%; OR = 1.9 [1.3-2.8]) and at 1 year (19.2% versus 10.0%; OR = 2.3 [1.3-4.0]) among light smokers. Efficacy among light smokers did not differ from that among heavier smokers (ps>0.50).CONCLUSIONThe nicotine lozenge is effective for light smokers. |
| 1518 | Anthonisen, Nicholas R, Skeans, Melissa A, Wise, Robert A, Manfreda, Jure, Kanner, Richard E, Connett, John E. The effects of a smoking cessation intervention on 14.5-year mortality: a randomized clinical trial. Annals of internal medicine. 2005;142:233.  **Abstract:** BACKGROUNDRandomized clinical trials have not yet demonstrated the mortality benefit of smoking cessation.OBJECTIVETo assess the long-term effect on mortality of a randomly applied smoking cessation program.DESIGNThe Lung Health Study was a randomized clinical trial of smoking cessation. Special intervention participants received the smoking intervention program and were compared with usual care participants. Vital status was followed up to 14.5 years.SETTING10 clinical centers in the United States and Canada.PATIENTS5887 middle-aged volunteers with asymptomatic airway obstruction.MEASUREMENTSAll-cause mortality and mortality due to cardiovascular disease, lung cancer, and other respiratory disease.INTERVENTIONThe intervention was a 10-week smoking cessation program that included a strong physician message and 12 group sessions using behavior modification and nicotine gum, plus either ipratropium or a placebo inhaler.RESULTSAt 5 years, 21.7% of special intervention participants had stopped smoking since study entry compared with 5.4% of usual care participants. After up to 14.5 years of follow-up, 731 patients died: 33% of lung cancer, 22% of cardiovascular disease, 7.8% of respiratory disease other than cancer, and 2.3% of unknown causes. All-cause mortality was significantly lower in the special intervention group than in the usual care group (8.83 per 1000 person-years vs. 10.38 per 1000 person-years; P = 0.03). The hazard ratio for mortality in the usual care group compared with the special intervention group was 1.18 (95% CI, 1.02 to 1.37). Differences in death rates for both lung cancer and cardiovascular disease were greater when death rates were analyzed by smoking habit.LIMITATIONSResults apply only to individuals with airway obstruction.CONCLUSIONSmoking cessation intervention programs can have a substantial effect on subsequent mortality, even when successful in a minority of participants. |
| 1520 | Wolfenden, L, Wiggers, J, Knight, J, Campbell, E, Rissel, C, Kerridge, R, Spigelman, A D, Moore, K. A programme for reducing smoking in pre-operative surgical patients: randomised controlled trial. Anaesthesia. 2005;60:172.  **Abstract:** We assessed the efficacy of a comprehensive programme for stopping smoking in 210 smokers scheduled for surgery, before admission and 3 months after attending a pre-operative clinic. Participants were randomly allocated to receive an intervention incorporating nicotine replacement therapy for patients smoking more than 10 cigarettes per day ("dependent smokers"), or to a control group to receive usual care. Dependent smokers allocated to the intervention group were more likely to report abstinence before surgery than those allocated to receive usual-care (63 (73%) vs. 29 (56%), respectively; OR 2.2 (95% CI 1.0-4.8)), and 3 months after attendance (16 (18%) vs. 3 (5%), respectively; OR = 3.9 (95% CI 1.0-21.7). |
| 1522 | Shiffman, Saul, Sweeney, Christine T, Dresler, Carolyn M. Nicotine patch and lozenge are effective for women. Nicotine & tobacco research : official journal of the Society for Research on Nicotine and Tobacco. 2005;7:119.  **Abstract:** It has been hypothesized that women may be less likely to obtain therapeutic benefit from nicotine replacement therapy (NRT). The present study tested this hypothesis, using two different types of NRT medications. A secondary analysis of two randomized clinical trials was performed: One compared active 21-mg nicotine patch with placebo among 193 men and 309 women, and the other compared active 2-mg or 4-mg nicotine lozenge with placebo among 788 men and 1,030 women. Using logistic regression analysis of 6-month continuous abstinence and survival analysis, we assessed the efficacy of patch and lozenge among women and tested for a gender x treatment interaction. Active NRT was more effective than placebo among women, for both patch and lozenge. In the lozenge trial, women were less successful than men. The gender x treatment interaction was not significant in either study, whether assessed by logistic regression or survival analysis. In the lozenge trial, gender moderated the effects of smoking rate and dependence (but not treatment) on outcome: These variables affected success rates only among women. Treatment with nicotine patch or lozenge is effective for women, and the analysis did not reveal significant gender differences in efficacy. Gender differences in outcome may be moderated by nicotine dependence. |
| 1523 | Poltavski, D V, Petros, T. Effects of transdermal nicotine on prose memory and attention in smokers and nonsmokers. Physiology & behavior. 2005;83:833.  **Abstract:** Previous research investigating cognitive effects of nicotine has produced mixed findings partly due to the use of abstaining smokers and cigarettes as a delivery system. The present study examined effects of nicotine delivered via a transdermal patch on prose memory and sustained attention in male smokers (n=25) and nonsmokers (n=22), who were randomly assigned to either a placebo or a nicotine condition. All groups were matched on their verbal ability and gross personality characteristics (state/trait anxiety levels, extroversion-introversion, and impulsivity level). In the nicotine condition, smokers were treated with a 21-mg transdermal patch, while nonsmokers received a 7-mg nicotine patch. Six hours following patch application, their performance was assessed on a computerized prose memory task and the Rapid Visual Information Processing task (RVIP) in a counterbalanced order and double-blind fashion. The results demonstrated that smokers in the placebo group recalled a significantly greater number of propositions than their counterparts in the nicotine group. Nonsmokers in the nicotine condition also remembered significantly more of the prose material than smokers in the same condition and showed a trend towards better recall of propositions of medium importance in the nicotine condition in comparison to the nonsmokers in the placebo group. No between-group differences were found on the RVIP task. A significant effect of time was found for systolic blood pressure and heart rate. The results cannot be interpreted using the arousal theory of nicotine effects on attention and are explained on the basis of a dose-dependent nicotinic action possibly recruiting cholinergic cortical projections. |
| 1525 | Cooper, Theodore V, Klesges, Robert C, Debon, Margaret W, Zbikowski, Susan M, Johnson, Karen C, Clemens, Linda H. A placebo controlled randomized trial of the effects of phenylpropanolamine and nicotine gum on cessation rates and postcessation weight gain in women. Addictive behaviors. 2005;30:61.  **Abstract:** With smoking prevalence rates beginning to decline, studies designed to promote cessation in more challenging populations, like weight-concerned smokers, warrant attention. This study assessed the efficacy of two forms of pharmacotherapy [nicotine and phenylpropanolamine (PPA) gums] in addition to a 13-week cognitive behavioral smoking cessation program targeted for women. Participants were 439 females who met rigorous screening criteria and were randomized to one of the three treatment intervention groups (PPA gum, nicotine gum, or placebo gum). All participants attended a 13-week cognitive behavioral smoking cessation program and were given specific instructions on gum chewing. At posttest (13 weeks), and 6- and 12-month follow-ups, body weight and point prevalence abstinence were assessed. Analyses to determine potential differences between treatment groups on weight change and cessation rates were performed. Results indicated that neither change in body weight nor cessation rates significantly differed between groups. Attendance to sessions did appear to consistently increase the likelihood of quitting smoking at posttest and at each of the follow-ups. These results suggest that although the pharmacological interventions had no effect on cessation rates and postcessation weight gain, the behavioral component of the intervention was effective in increasing the odds of quitting smoking in weight-concerned women. Future efforts should focus on increasing adherence to behavioral program components, particularly session attendance. |
| 1530 | Shiffman, Saul, Di Marino, Michael E, Pillitteri, Janine L. The effectiveness of nicotine patch and nicotine lozenge in very heavy smokers. Journal of substance abuse treatment. 2005;28:49.  **Abstract:** The efficacy of nicotine replacement therapy (NRT) among very heavy and highly dependent smokers was examined in a secondary analysis of two randomized clinical trials of NRT. In the first trial, smokers were assigned to active patch (n=249) or placebo (n=253) plus intensive behavioral treatment. In the second trial, smokers were assigned to active 4-mg nicotine lozenge (n=450) or placebo (n=451) plus brief behavioral treatment. Nicotine patch and lozenge significantly increased 6-month continuous abstinence quit rates in both very heavy (>or=40 cigarettes per day) and highly dependent (Fagerström Tolerance Questionnaire or Fagerström Test for Nicotine Dependence score >7) smokers. The effect of active NRT treatment did not differ significantly by smoking rate or nicotine dependence, with the exception that the nicotine patch was significantly more effective than placebo in highly dependent smokers. The nicotine patch and lozenge are effective (vs. placebo) even in heavy and highly dependent smokers. |
| 1531 | Flood, Pamela, Daniel, Danette. Intranasal nicotine for postoperative pain treatment. Anesthesiology. 2004;101:1417.  **Abstract:** BACKGROUNDDespite pharmacological treatment, 70-80% of patients report moderate to severe pain after surgery. Because nicotine has been reported to have analgesic properties in animal and human volunteer studies, the authors assessed the analgesic efficacy of a single 3 mg dose of nicotine nasal spray administered before emergence from general anesthesia.METHODSThe authors conducted a randomized, double blind, placebo controlled trial of 20 healthy women (mean age 45 (SD 8) yr) who were to undergo uterine surgery through a low transverse incision. After the conclusion of surgery but before emergence from general anesthesia, the anesthesiologist administered either nicotine nasal spray or a placebo. Numerical analog pain score and morphine utilization and hemodynamic values were measured for 24 h.RESULTSThe patients treated with nicotine reported lower pain scores during the first hour after surgery (peak numerical analog score, 7.6 (SD 1.4) versus 5.3 (SD 1.6); P < 0.001) and used half the amount of morphine as the control group (12 (SD 6) versus 6 (SD 5) mg; P < 0.05). Patients who received nicotine still reported less pain than those in the control group 24 h after surgery (1.5 (SD 0.5) versus 4.9 (SD 1.4); P < 0.01). Systolic blood pressure was lower in the group that received nicotine (105 (SD 3) versus 122 (SD 3); P < 0.001), but there was no difference in diastolic blood pressure or heart rate.CONCLUSIONSTreatment with a single dose of nicotine immediately before emergence from anesthesia was associated with significantly lower reported pain scores during the first day after surgery. The decreased pain was associated with a reduction in morphine utilization and the analgesic effect of nicotine was not associated with hypertension or tachycardia. |
| 1532 | Waters, Andrew J, Shiffman, Saul, Sayette, Michael A, Paty, Jean A, Gwaltney, Chad J, Balabanis, Mark H. Cue-provoked craving and nicotine replacement therapy in smoking cessation. Journal of consulting and clinical psychology. 2004;72:1136.  **Abstract:** Cue exposure paradigms have been used to examine reactivity to smoking cues. However, it is not known whether cue-provoked craving is associated with smoking cessation outcomes or whether cue reactivity can be attenuated by nicotine replacement therapy (NRT) in clinical samples. Cue-provoked craving ratings and reaction time responses were measured on the 1st day of abstinence among 158 smokers who had been randomized to high-dose nicotine (35 mg) or placebo patch. The nicotine patch reduced overall levels of craving but did not attenuate cue-provoked craving increases or reaction time responses. Cue-provoked craving predicted relapse among participants on the nicotine patch but not among those on placebo. In summary, NRT users could benefit from treatment that attenuates cue-provoked craving. |
| 1541 | Wakefield, Melanie, Olver, Ian, Whitford, Hayley, Rosenfeld, Ellie. Motivational interviewing as a smoking cessation intervention for patients with cancer: randomized controlled trial. Nursing research. 2004;53:396.  **Abstract:** BACKGROUNDSmoking cessation of patients with cancer can improve treatment efficacy and survival.OBJECTIVETo determine whether a motivational interviewing intervention increased successful smoking cessation attempts of patients with cancer attending a South Australian public hospital, as compared with usual care.METHODSA randomized controlled trial was used to study 137 patients with mixed cancer sites, including 74 intervention patients and 63 control patients. The motivational interviewing intervention was delivered over a 3-month period. The intervention included a visit with a smoking cessation counselor, provision of smoking cessation booklets, nicotine replacement therapy, family advice to quit, and an in-person or telephone follow-up conversation.RESULTSAt the 6-month follow-up visit, an intention-to-treat analysis found no difference in biochemically confirmed 3-month prevalence quit rates between the intervention (5%) and control (6%) groups. A sensitivity analysis using more lenient criteria indicated quit rates of 29% for the intervention group and 18% for the control group (p = .32). The predictors of smoking cessation at 6 months for all the patients included a smoking-related cancer site, more cessation attempts in the year before enrollment in the study, and no radiation therapy.CONCLUSIONSFuture efforts to improve smoking cessation in this patient group might focus on the delivery of more direct methods for encouraging spouse cessation and support to the patient in quitting, and the use of bupropion (Zyban) as an adjunct to cessation for this heavy smoking patient group. |
| 1544 | Sørensen, Lars Tue, Nielsen, Henning Bay, Kharazmi, Arsalan, Gottrup, Finn. Effect of smoking and abstention on oxidative burst and reactivity of neutrophils and monocytes. Surgery. 2004;136:1047.  **Abstract:** BACKGROUNDSmoking is associated with surgical wound infections, impaired wound healing, and tissue-destructive disorders. The mechanisms are largely unknown, but changes in the function and activity of inflammatory cells may be involved.METHODSSeventy healthy volunteers (54 smokers and 16 never smokers) were included. The smokers were studied while they smoked and after 20 days of abstinence. After the first 10 days of abstinence, they were randomized to double-blind treatment with transdermal nicotine patch 25 mg per day or placebo. Venous blood neutrophils and monocytes were sampled and isolated. In 22 randomly selected smokers and in all never smokers, the oxidative burst and chemotaxis were determined by a chemiluminescence response assay and a modified Boyden chamber technique, respectively. Stimulants were opsonized zymosan, formyl-Met-Leu-Phe, and zymosan-activated serum.RESULTSThe neutrophil and monocyte oxidative burst was 50% and 68% lower, respectively, in smokers compared to never smokers (P < .05). Neutrophil chemotaxis was 93% higher in smokers (P < .05). Monocyte chemotaxis was lower in smokers compared to never smokers (P < .05). After 20 days of abstinence, neutrophil oxidative burst increased to the level of never smokers (P < .05); monocyte oxidative burst increased by 50% (P < .05). Chemotaxis was only marginally affected. The changes induced by abstinence were less pronounced in the transdermal nicotine patch group compared to the placebo group.CONCLUSIONSSmoking attenuates the oxidative burst of inflammatory cells and increases chemotaxis. Three weeks of abstinence normalize the oxidative burst, but affect chemotaxis only marginally. |
| 1548 | Mora-Martínez, José Miguel, González-Ortiz, Manuel, Balcázar-Muñoz, Blanca R, Martínez-Abundis, Esperanza. Acute effect of the transdermal administration of nicotine on insulin sensitivity in healthy individuals with and without a family history of type 2 diabetes mellitus in the first branch. Metabolic syndrome and related disorders. 2004;2:227.  **Abstract:** The aim of this study was to evaluate the acute effect of the transdermal administration of nicotine on insulin sensitivity in healthy individuals with and without family histories of type 2 diabetes mellitus (DM2) in the first branch. A randomized, double-blind, cross-over, placebo-controlled clinical trial with two parallel groups was carried out in 12 healthy individuals, six with a family history of diabetes and six without such family history. The volunteers were randomly assigned to administration of a nicotine or placebo patch and were crossed-over with a difference of at least 3 days between each patch. The insulin sensitivity was estimated by means of the total glucose metabolism, which was obtained with the euglycemic- hyperinsulinemic clamp technique before the randomized assignment and on the following day for the corresponding patch. At the beginning of each clamp technique procedure, the metabolic and hepatic profiles were measured. We found that the total glucose metabolism value was not modified with the administration of nicotine, either in the group with a family history of DM2 (3.9 +/- 1.0 vs. 3.5 +/- 0.7 mg/kg/min; p = 0.60) or in the group without such a family history (5.4 +/- 2.0 vs. 5.1 +/- 1.6 mg/kg/min; p = 0.34). The administration of placebo did not modify the insulin sensitivity in either group. We conclude that acute transdermal administration of nicotine did not modify insulin sensitivity in healthy individuals with or without a family history of DM2 in the first branch. |
| 1550 | Allen, Sharon S, Hatsukami, Dorothy K, Bade, Tracy, Center, Bruce. Transdermal nicotine use in postmenopausal women: does the treatment efficacy differ in women using and not using hormone replacement therapy?. Nicotine & tobacco research : official journal of the Society for Research on Nicotine and Tobacco. 2004;6:777.  **Abstract:** This study of postmenopausal female smokers (N = 94) asked: During short-term smoking abstinence, do the beneficial effects of transdermal nicotine replacement therapy (NRT) on acute symptomatology (i.e., withdrawal, cigarette craving, smoking urges, mood, depressive symptoms, motor speed, and reaction time) differ in women who use and do not use hormone replacement therapy (HRT)? Participants were recruited according to HRT and non-HRT use (self-selecting), then randomized within strata to active nicotine or placebo nicotine patch. After 1 baseline week of smoking, participants quit smoking for 2 weeks. Women received cessation counseling and were monitored for abstinence. Dependent measures were collected during five clinic visits. Two-way analysis of covariance (ANCOVA) were run on change scores for dependent variables, with nicotine patch group (active/placebo) and HRT group (HRT/non-HRT) as independent variables and age as a covariate. No interactions were found between HRT and patch condition, but both showed specific effects. During the first abstinent week, women on active nicotine patch (compared with placebo) experienced less severe withdrawal, greater reductions in cigarette cravings, and lower (more favorable) Factor 1 scores on the Questionnaire of Smoking Urges. During the second abstinent week, women using HRT (compared with the non-HRT group) exhibited better mood (Profile of Mood States scores) and less depression (Beck Depression Inventory scores). These results suggest the following: First, the efficacy of transdermal nicotine replacement is not adversely modified by women's HRT use; second, ovarian hormones might influence women's responses to smoking cessation, and thus should be considered in developing effective strategies for women to quit smoking. |
| 1557 | Leischow, Scott J, Ranger-Moore, James, Muramoto, Myra L, Matthews, Eva. Effectiveness of the nicotine inhaler for smoking cessation in an OTC setting. American journal of health behavior. 2004;28:291.  **Abstract:** OBJECTIVETo evaluate the effectiveness of the Nicotrol nicotine inhaler as an aid to smoking cessation in over-the-counter (OTC) versus health-care-provider (HCP) conditions.METHODSFive hundred twenty healthy smokers were randomized to the treatment conditions and followed for a year.RESULTSAt most follow-up visits, abstinence rates for the HCP group were 2 to 3 times those observed in the OTC group. Abstinence at 1 year was.77% in the OTC condition versus 3.08% in the HCP condition [P<.01]. Inhaler use was low.CONCLUSIONSOTC nicotine inhaler appears to be ineffective, though quit rates are improved with HCP assistance. |
| 1562 | Carpenter, Matthew J, Hughes, John R, Solomon, Laura J, Callas, Peter W. Both smoking reduction with nicotine replacement therapy and motivational advice increase future cessation among smokers unmotivated to quit. Journal of consulting and clinical psychology. 2004;72:371.  **Abstract:** Smokers not currently interested in quitting (N = 616) were randomized to receive telephone-based (a) reduction counseling plus nicotine replacement therapy (NRT) plus brief advice to quit, (b) motivational advice plus brief advice, or (c) no treatment. More smokers in the reduction (43%) and motivational (51%) conditions made a 24-hr quit attempt over 6 months than smokers in the no-treatment condition (16%; p < or = .01), but the 2 active conditions did not differ (p > or = .05). Similarly, 18%, 23%, and 4% of each condition were abstinent (7-day point prevalence) at 6 months (p < or = .01). Results indicate smoking reduction using NRT does not undermine cessation but rather increases the likelihood of quitting to a degree similar to motivational advice. |
| 1563 | Ratner, Pamela A, Johnson, Joy L, Richardson, Chris G, Bottorff, Joan L, Moffat, Barbara, Mackay, Martha, Fofonoff, Doreen, Kingsbury, Kori, Miller, Clara, Budz, Bernice. Efficacy of a smoking-cessation intervention for elective-surgical patients. Research in nursing & health. 2004;27:148.  **Abstract:** We tested an intervention to help smokers abstain (fast) from smoking before surgery, maintain abstinence postoperatively, and achieve long-term cessation. A randomized experiment included 237 patients admitted for presurgical assessment who smoked. The intervention included counseling and nicotine replacement therapy. Treatment group participants (73.0%) were more likely to fast than were controls (53.0%): chi(2)(1, N = 228) = 8.89, p =.003, and more likely to be abstinent 6 months after surgery (31.2% vs. 20.2%). There was no significant difference in the abstinence rates at 12 months after surgery, chi(2)(1, N = 169) <.001, p = 1.00. Encouraging patients to fast from smoking before surgery and postoperative support are efficacious ways to reduce preoperative and immediate post-operative tobacco use. |
| 1564 | Schuurmans, Macé M, Diacon, Andreas H, van Biljon, Xandra, Bolliger, Chris T. Effect of pre-treatment with nicotine patch on withdrawal symptoms and abstinence rates in smokers subsequently quitting with the nicotine patch: a randomized controlled trial. Addiction (Abingdon, England). 2004;99:634.  **Abstract:** AIMSTo determine whether 2-week pre-treatment with transdermal nicotine influences withdrawal symptoms or success rate of subsequent smoking cessation using nicotine patches.DESIGNRandomized controlled trial.SETTINGSmoking cessation clinic.PARTICIPANTSHealthy smokers (n = 200, 45% female) were allocated randomly to either active nicotine-patch (AP, 15 mg daily, n = 100) or placebo-patch (PP, n = 100) pre-treatment. Baseline characteristics were well balanced except for daily cigarette consumption: mean (+/- SD) 23.1 (8) and 26.4 (11) for AP and PP groups, respectively (P = 0.021).INTERVENTIONAt the screening visit (-2 weeks) subjects were counselled and started pre-treatment with daily patches (AP or PP). From the quit date (week 0) onwards all subjects received active nicotine patches for 12 weeks (15 mg daily for 8 weeks, 10 and 5 mg daily for 2 weeks each) and counselling.MEASUREMENTSFollow-up visits included measurement of exhaled carbon monoxide at the quit date, 2, 6, 10 and 26 weeks. Subjects documented daily cigarette consumption and severity of withdrawal symptoms (Wisconsin scale) from -2 weeks to week 2. Outcome measures were withdrawal symptoms composite score and abstinence rates.FINDINGSThere was no significant difference in withdrawal symptoms, but more subjects in the AP group were smoke-free during the 6-month study period. Overall sustained abstinence was documented in 17% of subjects at 6 months; 22% and 12% for AP and PP, respectively (P = 0.03). Retrospective subgroup analysis showed for subjects smoking >16 cigarettes/day sustained cessation rates were 22% and 9% for AP and PP, respectively (P = 0.01). No difference in adverse event rates was observed.CONCLUSIONSNicotine patch pre-treatment before cessation did not reduce early withdrawal symptoms but increased sustained abstinence rates at 6 months. The nicotine pre-treatment was equally effective in light and heavy smokers. |
| 1566 | Morgan, Thomas M, Crawford, Linda, Stoller, Adam, Toth, David, Yeo, Kiang-Teck J, Baron, John A. Acute effects of nicotine on serum glucose insulin growth hormone and cortisol in healthy smokers. Metabolism: clinical and experimental. 2004;53:578.  **Abstract:** Cigarette smoking impairs glucose tolerance and alters serum levels of hormones involved in glucose metabolism, but the role of nicotine in such hormonal alterations is not well understood. In order to isolate the effects of transdermal nicotine on serum glucose, insulin, growth hormone, and cortisol in smokers, we conducted a randomized double-blind placebo-controlled cross-over study involving 34 healthy volunteer smokers between 18 and 55 years of age. Administration of a 14-mg transdermal nicotine patch resulted in nonsignificantly lowered fasting quantitative insulin-sensitivity index (P =.11) and a nonsignificant 9.3-mg/dL mean increase in serum glucose levels during a 75-g oral glucose tolerance test (OGTT) at time 60 minutes (P =.12). There were no substantial differences between groups in the areas under the curve (AUCs) for glucose (P =.33) or insulin (P =.79) during the OGTT. Levels of insulin and cortisol also were not significantly altered by nicotine. A secondary finding observed in the overall study group (primarily in females) was that nicotine caused a 29% median decrease in serum growth hormone (P =.02). We conclude that nicotine patches may lead to mild hyperglycemia and lowered insulin sensitivity. Further research is needed to determine the clinical implications of the unexpected finding that nicotine decreased growth hormone levels in female smokers. |
| 1568 | Yudkin, Patricia, Munafò, Marcus, Hey, Kate, Roberts, Sarah, Welch, Sarah, Johnstone, Elaine, Murphy, Michael, Griffiths, Siân, Walton, Robert. Effectiveness of nicotine patches in relation to genotype in women versus men: randomised controlled trial. BMJ (Clinical research ed.). 2004;328:989.  **Abstract:** |
| 1569 | Katz, David A, Muehlenbruch, Donna R, Brown, Roger L, Fiore, Michael C, Baker, Timothy B. Effectiveness of implementing the agency for healthcare research and quality smoking cessation clinical practice guideline: a randomized, controlled trial. Journal of the National Cancer Institute. 2004;96:594.  **Abstract:** BACKGROUNDThe Agency for Healthcare Research and Quality (AHRQ) Smoking Cessation Clinical Practice Guideline recommends that all clinicians strongly advise their patients who use tobacco to quit.METHODSWe conducted a randomized, controlled trial of the effectiveness of Guideline implementation at eight community-based primary care clinics in southern Wisconsin (four test sites, four control sites) among 2163 consecutively enrolled adult patients who smoked at least one cigarette per day and presented for nonemergency care during the baseline period (June 16, 1999, to June 20, 2000) or the intervention period (from June 21, 2000, to May 3, 2001). After collecting baseline data, staff at test sites implemented the intervention over a 2-month period. The intervention included a tutorial for intake clinicians, group and individual performance feedback for intake clinicians, use of a modified vital signs stamp, an offer of free nicotine replacement therapy, and proactive telephone counseling. Staff at control sites received only general information about the AHRQ Guideline. Self-reported abstinence from smoking was determined by telephone interviews at 2- and 6-month follow-up assessments. Hierarchical logistic regression models were used to estimate the odds ratios (ORs) for treatment assignment after adjustment for patient characteristics. All statistical tests were two-sided.RESULTSThere were no statistically significant differences in smoking cessation rates between participants at test and control sites during the baseline period. Among participants treated during the intervention period, those at test sites were more likely than those at control sites to report being abstinent at the 2-month (16.4% versus 5.8%; adjusted OR = 3.3, 95% confidence interval [CI] = 1.9 to 5.6; P<.001) and 6-month (15.4% versus 9.8%; adjusted OR = 1.7, 95% CI = 1.2 to 2.6; P =.009) follow-up assessments and to report continuous abstinence, that is, abstinence at both 2 and 6 months (10.9% versus 3.8%; adjusted OR = 3.4, 95% CI = 1.8 to 6.3; P<.001).CONCLUSIONImplementation of a guideline-based smoking cessation intervention by intake clinicians in primary care is associated with higher abstinence among smokers. |
| 1570 | Etter, Jean-François, Laszlo, Evelyne, Perneger, Thomas V. Postintervention effect of nicotine replacement therapy on smoking reduction in smokers who are unwilling to quit: randomized trial. Journal of clinical psychopharmacology. 2004;24:174.  **Abstract:** The objective of this study was to assess the post-intervention effect of nicotine replacement therapy on reduction of cigarette consumption 1.5 years after the end of a 6-month treatment. Heavy smokers who had no intention of quitting smoking were recruited from the general population and were randomly assigned to a treatment of nicotine (choice of a 15-mg nicotine patch, a 4-mg nicotine gum, and/or a 10-mg nicotine inhaler, n = 265), matching placebo products (n = 269), or no intervention (n = 389). Products were sent to participants by mail. Education was limited to a booklet. Of 923 participants, 879 (95%) were followed 6 months after randomization and 846 (92%) were followed after 26 months. Mean baseline consumption was 30 cigarettes/day in all groups. After 6 months, cigarette consumption had decreased by a mean of 10.9 cigarettes/day in the nicotine group, 8.7 in the placebo group, and 4.9 among controls (P < or = 0.02 for all pairwise comparisons). After 26 months, compared with baseline, cigarette consumption had decreased by a mean of 9.8 cigarettes/day in the nicotine group, 7.7 in the placebo group, and 7.7 among controls (nicotine vs. placebo or control: P < or = 0.03). After 2 years, smoking cessation rates did not differ significantly among groups (nicotine 11.7%, placebo 9.3%, control, 10.0%; P = 0.6). Thus, a slight effect of nicotine replacement therapy on reduction of cigarette consumption was maintained 1.5 years after the end of the 6-month treatment, but the initially observed placebo effect was not maintained. Nicotine replacement therapy for smoking reduction had no deleterious impact on smoking cessation. |
| 1571 | Hughes, John, Lindgren, Paula, Connett, John, Nides, Mitchell. Smoking reduction in the Lung Health Study. Nicotine & tobacco research : official journal of the Society for Research on Nicotine and Tobacco. 2004;6:275.  **Abstract:** We examined the ability of smokers who failed to quit smoking in the Lung Health Study to reduce the number of cigarettes per day and maintain this reduction and whether reduction predicted increased or decreased future cessation. In the Lung Health Study, among smokers with early lung disease who wished to stop smoking, 3923 were randomized to a special intervention of counseling and nicotine gum for smoking cessation and to bronchodilator therapy or placebo. Among the 1722 who were still smoking at the first year follow-up, 27% smoked the same, 43% smoked 1%-49% fewer, and 30% smoked at least 50% fewer cigarettes per day. Reduction in cigarettes per day was accompanied by reduction in expired-air carbon monoxide. About half of the less-than-50% reducers and one-fifth of the at-least-50% reducers maintained or exceeded this reduction over the next 4 years. Reduction was associated with nicotine gum use. Greater reduction at year 1 predicted more quit attempts in year 2 but not more point prevalence abstinence at year 2 nor more quits or abstinence between years 2 and 5. We conclude that reduction can be maintained but such reduction neither predicts an increased nor decreased probability of future cessation. |
| 1573 | Patterson, Freda, Jepson, Christopher, Kaufmann, Vyga, Rukstalis, Margaret, Audrain-McGovern, Janet, Kucharski, Susan, Lerman, Caryn. Erratum: Predictors of attendance in a randomized clinical trial of nicotine replacement therapy with behavioral counseling (Drug and Alcohol Dependence (2003) 72 (123-131) PII: S0376871603001947). Drug and Alcohol Dependence. 2004;73:315.  **Abstract:** |
| 1574 | Botella-Carretero, J I, Escobar-Morreale, H F, Martín, I, Valero, A M, Alvarez, F, García, G, Varela, C, Cantarero, M. Weight gain and cardiovascular risk factors during smoking cessation with bupropion or nicotine. Hormone and metabolic research = Hormon- und Stoffwechselforschung = Hormones et metabolisme. 2004;36:178.  **Abstract:** Weight gain is frequent after smoking cessation, and may limit patient's will to quit and long-term success. Nicotine and bupropion are effective drugs for smoking withdrawal. However, their influence on weight gain, insulin resistance and other cardiovascular risk factors, as well as possible differences in obese and lean subjects, have not been fully evaluated. We randomised 25 lean and 25 obese smokers to receive either bupropion or nicotine patches. Clinical evaluation and lipid profile were performed at baseline and after treatment. Insulin resistance was also assessed at the end. Weight, BMI, waist-to-hip ratio, and diastolic blood pressure increased (p < 0.005), whereas lipid profile improved (p < 0.001) after smoking cessation independently of obesity at baseline or drug used. Obese patients had higher insulin resistance at the end (p < 0.05) regardless of drug used. Weight gain was inversely related to age (beta= - 0.125, R = 0.38, p = 0.046), and insulin resistance was related to obesity at baseline (beta = 0.85, R = 0.46, p = 0.02). In conclusion, weight gain after smoking cessation is not dependent on obesity or drug taken. A beneficial lipid profile is achieved after quitting smoking with either bupropion or nicotine patch in both obese and lean subjects. |
| 1577 | D'Orlando, Kay J, Fox, Barbara S. Tolerability and pharmacokinetics of single and repeated doses of nicotine with The Straw, a novel nicotine replacement product. Nicotine & tobacco research : official journal of the Society for Research on Nicotine and Tobacco. 2004;6:63.  **Abstract:** The Straw Nicotine Oral Delivery System is a novel form of nicotine replacement therapy designed to provide not only nicotine but also oral and manual stimulation and ease of dosing. The Straw is a single-use drinking straw containing loose nicotine bitartrate particles that are ingested with the first sip of a beverage. This article describes single- and repeated-dose pharmacokinetics and tolerability of The Straw in a group of 24 smokers. The subjects were randomized to receive 12, 8, or 4 mg nicotine or placebo via The Straw following overnight abstinence from smoking. On one occasion, subjects took only one dose of nicotine; a week later they took eight doses, one every 1.5 hr. The orally ingested nicotine was well tolerated with single and repeated dosing. Plasma levels of nicotine peaked between 1 and 2 hr after a single dose at levels ranging from 6 ng/ml (4-mg dose) to 20 ng/ml (8- and 12-mg doses). Nicotine levels were not different between the 8-mg and 12-mg doses, possibly related to individual variability. Dosing repeatedly with The Straw led to greater nicotine levels that continued to increase with additional dosing. Levels achieved were comparable with or greater than those of other replacement products. |
| 1578 | Johnstone, Elaine C, Yudkin, Patricia L, Hey, Kate, Roberts, Sarah J, Welch, Sarah J, Murphy, Michael F, Griffiths, Siân E, Walton, Robert T. Genetic variation in dopaminergic pathways and short-term effectiveness of the nicotine patch. Pharmacogenetics. 2004;14:83.  **Abstract:** Polymorphisms in the dopamine D2 receptor (DRD2 C/T and DRD2 A/G) and in dopamine beta hydroxylase (DBH A/G) have been implicated in modulation of smoking and other reward-seeking behaviours. We hypothesized that these alleles would predict the outcome of nicotine patch therapy for smoking cessation. In 1991-93, we performed a randomized controlled trial of the nicotine patch on 1686 heavy smokers (> or = 15 cigarettes/day). In 1999-2000, we contacted 1532 of the 1612 subjects still available; 767 (50%) completed a questionnaire and gave a blood sample. In the 755 cases in which DNA was successfully genotyped, we examined associations between the polymorphisms in DRD2 and DBH, and smoking cessation. At 1 week, the patch was more effective for smokers with DRD2 CT/TT genotype [patch/placebo odds ratio (OR) 2.8, 95% confidence interval (CI) 1.7-4.6] than with CC (OR 1.4, 0.9-2.1; P for difference in ORs 0.04). Smokers with both DRD2 CT/TT and DBH GA/AA genotypes had an OR of 3.6 (2.0-6.5) compared to 1.4 (1.0-2.1) for others (P = 0.01). At 12 weeks, the ORs for these genotypic groups were 3.6 (1.7-7.8) and 1.4 (0.9-2.3), respectively (P = 0.04). There was no association between patch effectiveness and DRD2 exon 8. Short-term effectiveness of the nicotine patch may be related to dopamine beta-hydroxylase and dopamine D2 receptor genotype. Our results support the need for further investigation into personalized therapies for smoking cessation based on individual genotype. |
| 1580 | Shiffman, Saul, Dresler, Carolyn M, Rohay, Jeffrey M. Successful treatment with a nicotine lozenge of smokers with prior failure in pharmacological therapy. Addiction (Abingdon, England). 2004;99:83.  **Abstract:** AIMSTo assess the influence of unsuccessful past quit attempts using pharmacological treatment on smoking cessation when using a new nicotine lozenge.DESIGNA double-blind, randomized, placebo-controlled trial.SETTINGFifteen sites in the United Kingdom and the United States.PARTICIPANTSA total of 1818 smokers seeking smoking cessation treatment; 1145 had had previous pharmacological treatment for smoking cessation.INTERVENTIONLozenge, 2 mg or 4 mg (or matched placebo); a higher dose was assigned to smokers who smoked their first cigarette of the day within 30 minutes, a sign of dependence. Smokers received minimal instruction and counseling.MEASUREMENTOutcome was 28-day, CO-verified continuous abstinence at 6 weeks. Past use of medications was ascertained by self-report.FINDINGSLozenge was efficacious among smokers with prior pharmacotherapy as well as among those without such history. The effect of lozenge (versus placebo) was significantly greater among those with previous treatment experience, because previous treatment was associated with significantly poorer outcome on placebo, and active lozenge treatment corrected this imbalance. Lozenge efficacy was similar whether smokers had previously tried patch or acute forms of nicotine replacement therapy (gum, inhaler and spray), and also similar for past use of Zyban (bupriopion).CONCLUSIONSSmokers with a history of past failure of pharmacological treatment have lower success rates without pharmacological treatment, but equally good outcomes with active lozenge treatment. Smokers who previously tried pharmacological treatments but resumed smoking should be encouraged to try quitting again with the new nicotine lozenge. |
| 1585 | Sanderson Cox, Lisa, Patten, Christi A., Krahn, Lois E., Hurt, Richard D., Croghan, Ivana T., Wolter, Troy D., Schroeder, Darrell R., Tri, Diane, Offord, Kenneth P.. The Effect of Nicotine Patch Therapy on Depression in Nonsmokers: A Preliminary Study. Journal of Addictive Diseases. 2004;22:75.  **Abstract:** Prior uncontrolled studies of nonsmokers with major depressive disorder (MDD) indicate rapid reduction in depressive symptoms with nicotine patch therapy. This randomized, double-blind, placebo-controlled pilot study examined the effect of nicotine patch therapy on depressive symptoms in non-medicated nonsmokers with current MDD. Due to recruitment difficulties, only 7 were enrolled and of these 6 (5 females, 1 male) completed the study. Participants received either placebo (n = 4) or active (n = 2) patch therapy for 8 days. They completed daily clinic visits during patch therapy and a final visit on Day 12. Depressive symptoms were assessed using the Hamilton Rating Scale for Depression (HRSD). The mean change in HRSD scores of all participants decreased (p = 0.021) from baseline by Day 1 of patch use. Similar decreases in HRSD scores were observed for placebo and active patch groups. Among the placebo participants, the mean HRSD score decreased (p = 0.038) by Day 2. The study needs replication with a larger sample and utilizing novel recruitment strategies. © 2003 by The Haworth Press. Inc. All rights reserved. |
| 1589 | Havermans, Remco C, Debaere, Saskia, Smulders, Fren T Y, Wiers, Reinout W, Jansen, Anita T M. Effect of cue exposure, urge to smoke, and nicotine deprivation on cognitive performance in smokers. Psychology of addictive behaviors : journal of the Society of Psychologists in Addictive Behaviors. 2003;17:336.  **Abstract:** The primary aim of this investigation was to test the hypothesis that the urge to smoke interferes directly with cognitive performance. Fifty-four smokers were randomly assigned to 1 of 3 groups: (a). ad lib, (b). deprived, or (c). nicotine patch. Participants rated their urge to smoke on continuous visual analogue scales. Cognitive performance was determined by measuring reaction times (RTs) on a Sternberg task. The deprived group reported a higher urge and had longer RTs than the ad lib group when exposed to smoking-related cues. However, the nicotine patch group reported a higher urge in the absence of longer RTs. The results indicated that nicotine deprivation affects cognitive performance and that the urge to smoke only partially mediated RTs. |
| 1593 | Salín-Pascual, Rafael J, Basañez-Villa, Elizabeth. Changes in compulsion and anxiety symptoms with nicotine transdermal patches in non-smoking obsessive-compulsive disorder patients. Revista de investigacion clinica; organo del Hospital de Enfermedades de la Nutricion. 2003;55:650.  **Abstract:** OBJECTIVESome of the obsessive-compulsive disorder (OCD) symptoms can be elicited in rats by the administration of quinpirole (D2/D3 dopaminergic agonist). Nicotine administration blocked some aspect of checking behavior in that model. The main goal of this study was to determine if the clinical manifestations of OCD non smoking patients change with the administration of transdermal nicotine patches.MATERIAL AND METHODSEleven patients were studied (6 female and 5 males), average age 29.7 +/- 5.5 years. All of them were OCD according to DSM-IV criteria. Clinical scorings were done with Yale-Brown Obsessive Compulsive Scale (Y-BOCS), Beck Depression Inventory (BDI) and Beck Anxiety Inventory (BAI). Nicotine (17.5 mg/day) or placebo transdermal patches were randomly administered for five continuous days.RESULTSNicotine administration reduced the total Y-BOCS and the compulsive score of that scale, but did not reduce obsessions. Also anxiety was reduced as was shown by the BAI scores, when patients were on nicotine patches, no changes were observed in BDI.CONCLUSIONSThe present results replicated the animal findings about reduction in compulsive behavior after nicotine administration. Also suggest that nicotine depending on the dysfunction of the different neurotransmitter systems may produce different behavior or cognitive effects, also that even nicotine showed some beneficial effect in OCD patients, the low rate of nicotine smoking in this type of patients, may show other mechanisms than may protect to smoke in some psychiatric patients. |
| 1595 | Wennike, Poul, Danielsson, Tobias, Landfeldt, Björn, Westin, Ake, Tønnesen, Philip. Smoking reduction promotes smoking cessation: results from a double blind, randomized, placebo-controlled trial of nicotine gum with 2-year follow-up. Addiction (Abingdon, England). 2003;98:1395.  **Abstract:** AIMTo test the effect of nicotine gum and placebo in smokers not motivated or not able to quit smoking with regard to smoking reduction and smoking cessation.DESIGNThis randomized study evaluated nicotine gum versus placebo for up to 1 year in 411 healthy smokers highly motivated to reduce cigarette use. Smoking reduction was defined as self-reported daily smoking less than 50% of baseline and any decrease (1 p.p.m. or more) in carbon monoxide.SETTINGPulmonary department, Copenhagen, Denmark.FINDINGSThe overall success rate for sustained smoking reduction was significantly higher at all time-points for active versus placebo gum (6.3% versus 0.5% after 24 months). Nicotine gum achieved significantly higher point prevalence cessation rates than placebo at 12 and 24 months [11.2% versus 3.9% (odds ratio = 3.1; 95% CI, 1.4-7.2 and 9.3% versus 3.4% (odds ratio = 2.9; 95% CI, 1.2-7.1), respectively]. There was a linear relationship between decrease in number of daily cigarettes and decrease in plasma cotinine, exhaled carbon monoxide and plasma thiocyanate, with significantly greater reduction in the nicotine gum group after 4 and 12 months (maximum treatment duration) but not after 24 months. The decrease in toxin intake was smaller than the decline in daily cigarette consumption, suggesting that compensatory smoking occurred.CONCLUSIONSNicotine gum promoted cessation in this population of smokers unwilling to quit. Among reducers, the toxin intake correlated with reduced cigarette consumption although some compensatory smoking occurred. |
| 1598 | Swanson, Nancy A, Burroughs, Charles C, Long, Mark A, Lee, Robert W. Controlled trial for smoking cessation in a Navy shipboard population using nicotine patch, sustained-release buproprion, or both. Military medicine. 2003;168:830.  **Abstract:** This study reports an experimental, randomized controlled clinical trial comparing three treatments for smoking cessation: sustained-release bupropion, nicotine patch, and combination nicotine and bupropion, to a counseling-only control group (N = 140), for smoking sailors aboard seven Navy ships. The purpose was to determine the effectiveness of different pharmcotherapies used in smoking cessation programs. Continuous abstinence was defined as the percentage of subjects who did not smoke since the quit date assessed at 6 and 12 months and having an expired carbon monoxide concentration of <10 ppm at educational sessions 2, 3, and 4. Nine subjects dropped out of the study, and 40 subjects were lost to follow-up. Eleven percent (15/140 subjects) had continuous abstinence at 12 months. The abstinence rates at 12 months were 47% in the control group, as compared with 27% in the nicotine patch/bupropion group, 20% in the nicotine patch group, and 7% in the bupropion group. |
| 1600 | Hughes, John R, Callas, Peter W. Past alcohol problems do not predict worse smoking cessation outcomes. Drug and alcohol dependence. 2003;71:269.  **Abstract:** Whether smokers with a past history of alcohol problems are less able to stop smoking and have a greater need for nicotine replacement therapy than smokers without this history is unclear. We conducted a secondary analysis of a prior study of 1039 smokers randomized to 0, 21, 35 or 42 mg/day nicotine patch for smoking cessation. Because higher dose patches were being tested, only smokers of > or =30 cigs/day were included. Although smokers with current alcohol abuse or dependence were excluded, 15% of the smokers had a past (>1 year ago) Short Alcohol Dependence Data (SADD) score of > or =9 suggesting past alcohol problems. Heavy smokers with a past history of alcohol problems did not relapse sooner, were not less likely to be abstinent and did not benefit more from nicotine treatment or from higher doses than heavy smokers without this history. We conclude that a past history of alcohol problems per se does not predict inability to stop smoking. |
| 1602 | Willoughby, John O, Pope, Kenneth J, Eaton, Vaughn. Nicotine as an antiepileptic agent in ADNFLE: an N-of-one study. Epilepsia. 2003;44:1238.  **Abstract:** PURPOSETo test nicotine patch treatment for a patient with a defined mutation for autosomal dominant nocturnal frontal lobe epilepsy (ADNFLE) whose seizures were refractory to standard antiepileptic therapy.METHODSOpen and double-blind trials of nicotine patches in an "n-of-one" study. The double-blind trial comprised periods during which either placebo or nicotine patches were each used for three periods of 2 weeks, randomized in a double-blind manner.RESULTSIn an open study, nicotine patches reduced seizures from 1.65 +/- 2.36 to 0.01 +/- 0.0 seizures per day (p < 0.0001). In a double-blinded placebo-controlled phase, the average frequency of seizures on nicotine versus placebo was 0 +/- 0 versus 0.56 +/- 1.14 seizures per day (p < 0.0001).CONCLUSIONSNicotine patches may be of benefit to some individuals with ADNFLE. |
| 1605 | Yudkin, Patricia, Hey, Kate, Roberts, Sarah, Welch, Sarah, Murphy, Michael, Walton, Robert. Abstinence from smoking eight years after participation in randomised controlled trial of nicotine patch. BMJ (Clinical research ed.). 2003;327:28.  **Abstract:** |
| 1607 | Sorensen, Lars Tue, Karlsmark, Tonny, Gottrup, Finn. Abstinence from smoking reduces incisional wound infection: a randomized controlled trial. Annals of surgery. 2003;238:1.  **Abstract:** OBJECTIVEClinical studies show that the incidence of postoperative wound complications is higher in smokers than nonsmokers. In this study, we evaluated the effect of abstinence from smoking on incisional wound infection.METHODSSeventy-eight healthy subjects (48 smokers and 30 never-smokers) were included in the study and followed for 15 weeks. In the first week of the study, the smokers smoked 20 cigarettes per day. Subsequently, they were randomized to continuous smoking, abstinence with transdermal nicotine patch (25 mg per day), or abstinence with placebo patch. At the end of the first week and 4, 8, and 12 weeks after randomization, incisional wounds were made lateral to the sacrum to excise punch biopsy wounds. At the same time identical wounds were made in 6 never-smokers. In 24 never-smokers a wound was made once. All wounds were followed for 2 weeks for development of wound complications.RESULTSA total of 228 wounds were evaluated. In smokers the wound infection rate was 12% (11 of 93 wounds) compared with 2% (1 of 48 wounds) in never-smokers (P <0.05). Wound infections were significantly fewer in abstinent smokers compared with continuous smokers after 4, 8, and 12 weeks after randomization. No difference between transdermal nicotine patch and placebo was found.CONCLUSIONSSmokers have a higher wound infection rate than never-smokers and 4 weeks of abstinence from smoking reduces the incidence of wound infections. |
| 1608 | Sørensen, Lars Tue, Jørgensen, Torben. Short-term pre-operative smoking cessation intervention does not affect postoperative complications in colorectal surgery: a randomized clinical trial. Colorectal disease : the official journal of the Association of Coloproctology of Great Britain and Ireland. 2003;5:347.  **Abstract:** BACKGROUNDSmokers have a higher risk of complicated tissue and wound healing after surgery than nonsmokers. We tested the hypothesis that short-term pre-operative cessation of smoking in colorectal surgery decreases the incidence of postoperative tissue and wound complications.METHODSFrom February 1998 to March 2001, 60 patients, who smoked daily, undergoing colorectal resection were randomly assigned 2-3 weeks before scheduled surgery to either abstinence from smoking, counselling and nicotine replacement therapy or maintenance of daily smoking habits. Postoperative tissue and wound complications necessitating surgical or medical treatment were evaluated at discharge and 30 days after surgery by blinded outcome assessment.RESULTSIn the pre-operative period of 15 days (8-24) (median, interquartile range (IQR)), 89% of the patients in the intervention group vs. 13% in the control group abstained from smoking or reduced by more than half (P < 0.05). In the postoperative period of 11 days (10-13), the corresponding figures were 92% and 50%, respectively (P < 0.05). Postoperative tissue and wound complications occurred in 33% (9 of 27) of the patients in the intervention group compared to 27% (8 of 30) in the control group (NS). Likewise, no difference in overall postoperative complication rate was found between the groups.CONCLUSIONShort-term cessation of smoking does not reduce the risk of complicated tissue and wound healing or other complications in colorectal surgery. |
| 1609 | Waters, Andrew J, Shiffman, Saul, Sayette, Michael A, Paty, Jean A, Gwaltney, Chad J, Balabanis, Mark H. Attentional bias predicts outcome in smoking cessation. Health psychology : official journal of the Division of Health Psychology, American Psychological Association. 2003;22:378.  **Abstract:** Most attempts to quit smoking end in failure, with many quitters relapsing in the first few days. Responses to smoking-related cues may precipitate relapse. A modified emotional Stroop task-which measures the extent to which smoking-related words disrupt performance on a reaction time (RT) task-was used to index the distracting effects of smoking-related cues. Smokers (N = 158) randomized to a high-dose nicotine patch (35 mg) or placebo patch completed the Stroop task on the 1st day of a quit attempt. Smokers using an active patch exhibited less attentional bias, making fewer errors on smoking-related words. Smokers who showed greater attentional bias (slowed RT on the first block of smoking words) were significantly more likely to lapse in the short-term, even when controlling for self-reported urges at the test session. Attentional bias measures may tap an important component of dependence. |
| 1610 | Mumenthaler, Martin S, Yesavage, Jerome A, Taylor, Joy L, O'Hara, Ruth, Friedman, Leah, Lee, Hana, Kraemer, Helena C. Psychoactive drugs and pilot performance: a comparison of nicotine, donepezil, and alcohol effects. Neuropsychopharmacology : official publication of the American College of Neuropsychopharmacology. 2003;28:1366.  **Abstract:** The cholinergic system plays a major role in cognitive abilities that are essential to piloting an aircraft: attention, learning, and memory. In previous studies, drugs that enhance the cholinergic system through different pharmacologic mechanisms have shown beneficial effects on cognition; but dissimilar cognitive measures were used and samples were not comparable. A comparison within the same cognitive tasks, within comparable samples appears desirable. Toward this aim, we compared effect sizes (ES) of performance-enhancing doses of nicotine (a nicotinic receptor agonist) and donepezil (an acetylcholinesterase inhibitor) as found in our prior work on pilot performance. We also compared cholinergic ES to those of performance-impairing doses of alcohol. In three randomized, placebo-controlled trials, we assessed the flight performance of aircraft pilots in a Frasca 141 simulator, testing I: the acute effects of nicotine gum 2 mg; II: the effects of administration of 5 mg donepezil/day for 30 days; and III: the acute and 8 h-carryover effects of alcohol after a target peak BAC of 0.10%. We calculated the ES of nicotine, donepezil, and alcohol on a flight summary score and on four flight component scores. Compared to placebo, nicotine and donepezil significantly improved, while alcohol significantly impaired overall flight performance: ES (nicotine)=0.80; ES (donepezil)=1.02; ES (alcohol acute)=-3.66; ES (alcohol 8 h)=-0.82. Both cholinergic drugs showed the largest effects on flight tasks requiring sustained visual attention. Although the two tested cholinergic drugs have different pharmacologic mechanisms, their effects on flight performance were similar in kind and size. The beneficial effects of the cholinergic drugs on overall flight performance were large and the absolute (ie nondirectional) sizes were about one-fourth of the absolute ES of acute alcohol intoxication and roughly the same as the absolute 8 h-carryover ES of alcohol. |
| 1612 | Hughes, John R, Novy, Pamela, Hatsukami, Dorothy K, Jensen, Joni, Callas, Peter W. Efficacy of nicotine patch in smokers with a history of alcoholism. Alcoholism, clinical and experimental research. 2003;27:946.  **Abstract:** BACKGROUNDSmokers with a history of alcohol dependence may have more difficulty quitting, might relapse to alcohol use, and might especially benefit from nicotine replacement therapy for smoking cessation.METHODSOne hundred fifteen smokers with a history of alcohol dependence (median of 5 years previously) were randomly assigned to either a 21-mg nicotine patch or placebo in a trial designed to be as similar as possible to a prior study that examined smokers with no history of alcoholism. Both studies were of heavy smokers with similar levels of nicotine dependence; thus, any differences in trials would be due to a history of alcohol problems per se.RESULTSIn the current trial, adjusted prolonged smoking abstinence in those with a history of alcohol dependence was higher in the active than the placebo group at end-of-treatment (28% vs. 11%; odds ratio, 3.2; p = 0.04) and at 6-month follow-up (24% vs. 6%; odds ratio, 4.9; p = 0.02). Among subjects not lost to follow-up, none reported drinking problems or increases in craving for alcohol. Smoking abstinence was not lower and the odds ratio for nicotine patch therapy was not greater in smokers with a history of alcohol dependence than in smokers with no such history.CONCLUSIONSHeavy smokers with a history of alcoholism benefit from nicotine patch treatment. A history of alcohol problems after a period of stable sobriety does not appear to influence smoking outcomes or response to nicotine replacement. Although no smokers relapsed to alcohol use, a trial that follows up all subjects is needed to verify this. |
| 1613 | Jessen, Anna B., Toubro, Søren, Astrup, Arne. Effect of chewing gum containing nicotine and caffeine on energy expenditure and substrate utilization in men 1-3. American Journal of Clinical Nutrition. 2003;77:1442.  **Abstract:** Background: Nicotine replacement therapy limits weight gain after smoking cessation. This finding is partly attributable to the thermogenic effect of nicotine, which may be enhanced by caffeine. Objective: We assessed the acute thermogenic effects of chewing gum containing different doses of nicotine and caffeine. Design: This randomized, double-blind, placebo-controlled, crossover study included 12 healthy, normal-weight men (aged 18-45 y). Energy expenditure was measured with indirect calorimetry before and 2.5 h after subjects chewed each of 7 different types of gum containing the following doses of nicotine/caffeine: 0/0, 1/0, 2/0, 1/50, 2/50, 1/100, and 2/100 mg/mg. Results: The thermogenic responses (increases over the response to placebo) were 3.7%, 4.9%, 7.9%, 6.3%, 8.5%, and 9.8%, respectively, for the gums containing 1/0, 2/0, 1/50, 2/50, 1/100, and 2/100 mg nicotine/mg caffeine (P < 0.05 for all). Adding caffeine to 1 and 2 mg nicotine significantly enhanced the thermogenic response, but changing the caffeine dose (from 50 to 100 mg) did not change the thermogenic effect. None of the combinations changed the respiratory quotient compared with placebo, which indicates that glucose and fat oxidation rates were increased to a similar extent. Side effects occurred only with 2 mg nicotine. Conclusions: One milligram of nicotine has a pronounced thermogenic effect, which can be increased by ≈100% by adding 100 mg caffeine. Increasing the nicotine dose to 2 mg does not increase the thermogenic effect but produces side effects in most subjects. Caffeine may be useful in preventing weight gain after smoking cessation if its thermogenic effect can be used to enhance nicotine's effect on long-term energy balance. |
| 1615 | Molyneux, A, Lewis, S, Leivers, U, Anderton, A, Antoniak, M, Brackenridge, A, Nilsson, F, McNeill, A, West, R, Moxham, J, Britton, J. Clinical trial comparing nicotine replacement therapy (NRT) plus brief counselling, brief counselling alone, and minimal intervention on smoking cessation in hospital inpatients. Thorax. 2003;58:484.  **Abstract:** BACKGROUNDGuidelines recommend that smoking cessation interventions are offered in all clinical settings to all smokers willing to make a quit attempt. Since the effectiveness of routine provision of behavioural counselling and nicotine replacement therapy (NRT) to smokers admitted to hospital has not been established, a randomised controlled trial of these interventions given together compared with counselling alone or minimal intervention was performed in hospital inpatients.METHODSMedical and surgical inpatients who were current smokers at the time of admission were randomised to receive either usual care (no additional advice at admission), counselling alone (20 minute intervention with written materials), or NRT plus counselling (counselling intervention with a 6 week course of NRT). Continuous and point prevalence abstinence from smoking (validated by exhaled carbon monoxide <10 ppm) was measured at discharge from hospital and at 3 and 12 months, and self-reported reduction in cigarette consumption in smokers was assessed at 3 and 12 months.RESULTS274 inpatient smokers were enrolled. Abstinence was higher in the NRT plus counselling group (n=91) than in the counselling alone (n=91) or usual care (n=92) groups. The difference between the groups was significant for validated point prevalence abstinence at discharge (55%, 43%, 37% respectively, p=0.045) and at 12 months (17%, 6%, 8%, p=0.03). The respective differences in continuous validated abstinence at 12 months were 11%, 4%, 8% (p=0.25). There was no significant difference between counselling alone and usual care, or in reduction in cigarette consumption between the treatment groups.CONCLUSIONSNRT given with brief counselling to hospital inpatients is an effective routine smoking cessation intervention. |
| 1620 | Reid, Robert, Pipe, Andrew, Higginson, Lyall, Johnson, Karin, D'Angelo, Monika Slovinec, Cooke, Debbie, Dafoe, William. Stepped care approach to smoking cessation in patients hospitalized for coronary artery disease. Journal of cardiopulmonary rehabilitation. 2003;23:176.  **Abstract:** PURPOSESmoking cessation is an important goal for smokers with coronary artery disease (CAD) because it reduces cardiac morbidity and mortality. Effective interventions for cigarette smokers with CAD exist, but they often are considered to be intensive and expensive. Stepped-care interventions have been proposed as a promising way to allocate smoking cessation treatments in a cost-effective manner. Stepped care refers to the practice of initiating treatment with low-intensity intervention and then exposing treatment failures to successively more intense interventions.METHODSTo address the efficacy of this approach, 254 cigarette smokers hospitalized with CAD were provided a brief cessation intervention. The participants then were assigned randomly to either a more intensive stepped-care treatment (counseling and nicotine patch therapy) or no additional treatment. Outcomes were point-prevalent abstinence measured 3 months and 1 year after hospital discharge.RESULTSStepped-care treatment increased smoking cessation rates from 42% to 53% during a 3-month follow-up period (P =.05), but showed little effect at the 1-year follow-up assessment, as evidenced by a cessation rate for the minimal intervention group of 36% versus 39% for the stepped-care group (P =.36).CONCLUSIONSA stepped-care approach to smoking cessation increased short-but not long-term point-prevalent abstinence in patients with CAD. For improvement of long-term effectiveness, refinement of the timing and content of stepped-care interventions needs to occur. |
| 1621 | Rodríguez-Artalejo, F, Lafuente Urdinguio, P, Guallar-Castillón, P, Garteizaurrekoa Dublang, P, Sáinz Martínez, O, Díez Azcárate, J I, Foj Alemán, M, Banegas, J R. One year effectiveness of an individualised smoking cessation intervention at the workplace: a randomised controlled trial. Occupational and environmental medicine. 2003;60:358.  **Abstract:** AIMSTo assess the effectiveness of a smoking cessation intervention at the workplace. The intervention was adapted to smokers' tobacco dependence, and included minimal structured counselling at the first visit (5-8 minutes), nicotine patches for three months, and three sessions of counselling for reinforcement of abstinence (2-3 minutes) over a three month period.METHODSOpen randomised trial with two groups: the intervention group, and the control group which was subjected to standard clinical practice, consisting of short (30 seconds to one minute) sporadic sessions of unstructured medical antismoking advice. The trial was carried out among 217 smokers of both sexes, aged 20-63 years, motivated to quit smoking and without contraindications for nicotine patches, who were employees at a public transport company and at two worksites of an electric company. The main outcome measure was self reported tobacco abstinence confirmed by carbon monoxide in expired air </=10 ppm. Analysis was performed according to intention-to-treat.RESULTSThe rate of continuous abstinence at 12 months was 20.2% for the intervention versus 8.7% for the control group (OR: 2.58; 95% CI: 1.13 to 5.90; p = 0.025). In subgroup analyses, effectiveness of the intervention did not vary substantially with age, tobacco dependence, number of cigarettes smoked per day, number of years of tobacco consumption, degree of desire to quit smoking, time spent with smokers, subjective health, and presence of tobacco related symptoms. Weight gain at 12 months was similar for both groups (1.69 kg in the intervention v 2.01 kg in the control group; p = 0.21).CONCLUSIONSA simple and easily generalisable intervention at the workplace is effective to achieve long term smoking cessation. In a setting similar to ours, nine subjects would have to be treated for three months for one to achieve continuous abstinence for 12 months. |
| 1624 | Glavas, Duska, Rumboldt, Mirjana, Rumboldt, Zvonko. Smoking cessation with nicotine replacement therapy among health care workers: randomized double-blind study. Croatian medical journal. 2003;44:219.  **Abstract:** AIMTo assess the smoking prevalence and efficacy of nicotine replacement therapy on the quitting rate after 3 weeks of therapy and after 1- and 5-year follow-up among health care workers with a smoking habit in Split, Croatia.METHODSAmong 311 hospital health professionals included in the study, there were 112 (36%) smokers; 44 (39%) of them were physicians and 68 (61%) nurses. In a randomized double-blind study, 112 smokers were divided in 2 groups, applying daily either a transdermal nicotine system (TNS) or placebo patch over 3 weeks. Abstinence was evaluated via questionnaire and on the basis of carbon monoxide concentration measured in the exhaled breath.RESULTSThe abstinence rates after the 3-week intervention period were 39% in the TNS group and almost 20% in the control group (chi-square test, p=0.038). After one year, these rates declined to 23% and 16%, respectively (p=0.476), and converged to 18% and 14% (p=0.797), respectively, at 5-year follow-up.CONCLUSIONSShort-term TNS treatment is effective in smoking cessation, although the effects steadily wane over time and the relapse rate is high. Continuous, structured, and composite efforts are needed for the maintenance of the non-smoking behavior. |
| 1628 | Ahmadi, Jamshid, Ashkani, Hamid, Ahmadi, Mojtaba, Ahmadi, Nahid. Twenty-four week maintenance treatment of cigarette smoking with nicotine gum, clonidine and naltrexone. Journal of substance abuse treatment. 2003;24:251.  **Abstract:** This research study investigated the effect of nicotine gum, clonidine, and naltrexone, in the maintenance treatment of cigarette smoking. In a double blind study, 171 nicotine-dependent male subjects who met DSM-IV criteria for nicotine dependence and smoking 10 cigarettes or more per day, were allocated randomly to three equal groups of 57. Subjects received nicotine gum, clonidine, or naltrexone over a 24-week treatment period. The nicotine gum dose was 2 mg every 1 to 2 h for the first 6 weeks, 2 mg every 2 to 4 h for the next 3 weeks, and 2 mg every 4 to 8 h for the remaining 15 weeks. The clonidine dose was 0.4 mg and the naltrexone dose was 50 mg per day. Continuous abstinence rates were recorded weekly for 24 weeks from the quit date. The abstinence rates by treatment groups were 36.8% for the nicotine gum group, 19.3% for the clonidine group, and 5.3% for the naltrexone group,and all between groups differences were significant. These results support the efficacy and safety of nicotine gum and clonidine for smoking relapse prevention among Iranian nicotine-dependent patients, but call in question the utility of naltrexone treatment for smoking relapse prevention. |
| 1630 | Cook, Mary R, Gerkovich, Mary M, Graham, Charles, Hoffman, Steven J, Peterson, Rebecca C. Effects of the nicotine patch on performance during the first week of smoking cessation. Nicotine & tobacco research : official journal of the Society for Research on Nicotine and Tobacco. 2003;5:169.  **Abstract:** Complaints of feeling unfocused and being unable to concentrate are common during smoking cessation, and such feelings may contribute to a subtle erosion of the motivation to quit. A heterogeneous sample of 21 established smokers (10 women, 11 men) completed this double-blind, placebo-controlled study to test the hypothesis that nicotine replacement during cessation therapy (using a 21-mg nicotine patch) would improve performance on tasks sensitive to nicotine deprivation. Participants were trained to stable performance on simple reaction time, mathematical processing, Sternberg memory, rapid visual-information processing, grammatical reasoning, and the Stroop Color-Word (Stroop) tasks. They received smoking cessation counseling and were randomly assigned to nicotine patch and placebo patch groups. Performance was assessed prior to cessation, and early (days 2 and 3) and late (days 5 through 7) in the first cessation week. The hypothesis was not supported. Increased accuracy was associated with the patch only for grammatical reasoning. No reaction time differences were found in the simple reaction time, grammatical reasoning, and mathematical processing tasks. Reaction time was faster in the placebo group on the more difficult portions of those tasks requiring sustained attention (rapid visual-information processing, Stroop Color-Word, and Sternberg memory tasks). These results differ substantially from those obtained when young adults are allowed to smoke or chew nicotine gum after relatively brief periods of deprivation. Evidence that smoking may interfere with cognition is accumulating; these results support this view. The subjective performance decrements noted by many smokers during cessation may be related to overall negative affect, rather than to direct effects on cognition and attention. |
| 1634 | Shiffman, Saul, Shadel, William G, Niaura, Raymond, Khayrallah, Moise A, Jorenby, Douglas E, Ryan, Charles F, Ferguson, Clifford L. Efficacy of acute administration of nicotine gum in relief of cue-provoked cigarette craving. Psychopharmacology. 2003;166:343.  **Abstract:** RATIONALEAcute cravings, often provoked by exposure to smoking cues, appear to be important triggers for smoking relapse. Relief of acute craving may therefore be an important step in preventing relapse.OBJECTIVESThis study was undertaken to assess the effectiveness of nicotine gum in relieving acute craving.METHODSA multi-center, randomized, placebo-controlled study was conducted with smokers ( n=296) who quit by using either active or inactive gum for 3 days. On their third day of abstinence, smokers participated in a laboratory session in which they were exposed to a provocative smoking cue, chewed active or inactive gum, and then rated their craving at 5-min intervals for 35 min.RESULTSCraving initially decreased in both groups. After 15 min, however, the smokers using active nicotine gum experienced significantly greater craving reductions.CONCLUSIONSThese results suggest that nicotine gum can effectively reduce acute craving following exposure to smoking cues. |
| 1640 | Smith, Stevens S, Jorenby, Douglas E, Leischow, Scott J, Nides, Mitchell A, Rennard, Stephen I, Johnston, J Andrew, Jamerson, Brenda, Fiore, Michael C, Baker, Timothy B. Targeting smokers at increased risk for relapse: treating women and those with a history of depression. Nicotine & tobacco research : official journal of the Society for Research on Nicotine and Tobacco. 2003;5:99.  **Abstract:** Some studies have shown that female smokers and smokers with a history of depression have an increased risk of relapse following smoking cessation treatment. This study examined the efficacy of bupropion sustained-release (SR) and the nicotine patch for smoking cessation in subgroups of smokers at possible risk for relapse. Data for this study were from a previously published randomized, double-blind, placebo-controlled clinical trial in which 893 smokers were randomized to four treatment conditions: placebo tablet + placebo patch, placebo tablet + 21 mg/24-hr nicotine patch, 300mg bupropion SR + placebo patch, and 300mg bupropion SR + 21 mg/24-hr nicotine patch. Study medication continued for 8 weeks after the quit day; brief individual cessation counseling was provided during weekly clinic visits. In comparison to the placebo tablet, bupropion SR approximately tripled 1-year non-smoking rates among women and previously depressed individuals. In contrast, the nicotine patch did not significantly improve cessation rates for any group. We conclude that bupropion SR is a first-line treatment for smoking that has the potential to benefit all smokers, especially women and the previously depressed. |
| 1652 | Bolliger, Chris T, Zellweger, Jean-Pierre, Danielsson, Tobias, van Biljon, Xandra, Robidou, Annik, Westin, Ake, Perruchoud, André P, Säwe, Urbain. Influence of long-term smoking reduction on health risk markers and quality of life. Nicotine & tobacco research : official journal of the Society for Research on Nicotine and Tobacco. 2002;4:433.  **Abstract:** We have recently published efficacy and safety data of a study using an oral nicotine inhaler in smoking reduction. The current analysis was undertaken to assess the secondary objectives of the trial: the influence of long-term smoking reduction on health risk markers. Four hundred healthy volunteers, unable or unwilling to stop smoking immediately, were enrolled in a double-blind, randomized, placebo-controlled trial in smoking reduction; 310 were evaluable up to 2 years. Participants were randomized to active or placebo inhalers as needed for up to 18 months, with subjects encouraged to reduce their smoking as much as possible; counseling took place over a 2-year period. For the current prospective cohort study, the number of daily cigarettes, carbon monoxide (CO), cotinine, cardiovascular risk factors, pulmonary function tests, and quality of life were compared between successful reducers (n = 25) and unsuccessful reducers (control group, n = 285). Success was defined as a reduction of daily cigarettes of at least 50% from week 6 to 2 years, verified by a decrease in exhaled CO at all visits compared with baseline. At 2 years, successful reducers showed a significantly greater decrease in cotinine levels (60% vs. 1%, p < 0.001), cholesterol/high-density lipoprotein (HDL) ratios (-2.42 vs. -1.67, p = 0.025), hemoglobin concentrations (-5.67 vs. -1.34 g/l, p = 0.023), pulse rate (-3.7 vs. +1.0 bpm, p = 0.043), and significantly improved general health score (9.40 vs. 2.34, p = 0.049); whereas no difference was found in forced expiratory volume during 1 s (FEV1) and forced vital capacity (FVC) compared with controls. The benefits of long-term smoking reduction of at least 50% of daily cigarettes were not offset by compensatory smoking, and the marked decrease in markers of smoke exposure had a positive influence on several cardiovascular risk markers and quality of life assessments. |
| 1653 | Glover, Elbert D, Glover, Penny N, Franzon, Mikael, Sullivan, C Rollynn, Cerullo, Connie C, Howell, Robert M, Keyes, Gordon G, Nilsson, Fredrik, Hobbs, Gerald R. A comparison of a nicotine sublingual tablet and placebo for smoking cessation. Nicotine & tobacco research : official journal of the Society for Research on Nicotine and Tobacco. 2002;4:441.  **Abstract:** This study assessed the efficacy and safety of a nicotine sublingual tablet in smoking cessation in a randomized, double-blind, placebo-controlled outpatient trial conducted between January 1996 and May 1997. Two hundred and forty-one adult smokers (> or = 10 cigarettes/day for at least 3 years) used nicotine 2-mg sublingual tablet (n = 120) or placebo (n = 121) for up to 6 months (a 3-month treatment period followed by a 3-month tapering period). Subjects who scored <7 on the Fagerström Tolerance Questionnaire used 1 tablet/h (up to maximum of 20/day), whereas subjects who scored (7 used 2 tablets/h (up to maximum of 40/day). Brief counseling was provided at baseline and at all visits. Self-reported abstinence was measured from week 2 onwards, confirmed by expired carbon monoxide (CO) levels <10 ppm at each visit (1, 2, 3 and 6 weeks and 3, 6 and 12 months). After 6 weeks of treatment, CO-validated abstinence rates were 48% in the active group and 23% in the placebo group (p < 0.0001). At the 3-, 6- and 12-month visits, abstinence rates (active vs. placebo) were 33% vs. 17% (p = 0.0046), 21% vs. 11% (p = 0.0304) and 18% vs. 10% (p = 0.0606). Adverse events were mild and transient and reflected those reported with existing nicotine replacement formulations. We concluded that the nicotine 2-mg sublingual tablet was effective as a smoking cessation aid. |
| 1654 | Shiffman, Saul, Gorsline, Jane, Gorodetzky, Charles W. Efficacy of over-the-counter nicotine patch. Nicotine & tobacco research : official journal of the Society for Research on Nicotine and Tobacco. 2002;4:477.  **Abstract:** This study aimed to assess the efficacy of the nicotine patch for smoking cessation, under simulated conditions of over-the-counter sale, absent any direct instruction or behavioral treatment. In a randomized, double-blind, placebo-controlled, multicenter study, 567 smokers were randomized to an active nicotine patch (n = 283) or placebo (n = 284). Treatment followed a three-step program of 21 mg/day for 6 weeks, 14 mg/day for 2 weeks and 7 mg/day for 2 weeks. Participants received brief written instructions, an audiotape and a written User's Guide. There was no other intervention and no contact with participants between enrollment and the primary outcome assessment at 6 weeks. Analyses were based on intent to treat, with lost subjects counted as failures, and claimed abstinence was verified by carbon monoxide measures. Use of active patch produced significantly higher abstinence rates. Continuous abstinence rates (subject to a 2-week grace period) for nicotine and placebo were 19.4% and 7.0% at 6 weeks (OR = 3.2; 95% CI 1.8-5.4) and 15.2% and 5.3% at 10 weeks (OR = 3.2; 95% CI 1.7-5.9), respectively. Seven-day point-prevalence rates for nicotine and placebo patches were 26.1% and 7.7% at 6 weeks (OR = 4.2; 95% CI 2.5-7.0) and 23.3% and 7.7% at 10 weeks (OR = 3.6; 95% CI 2.2-6.1), respectively. Reported adverse events were mild and consistent with prior observations of nicotine patch use. The nicotine patch was safe and effective for smoking cessation at least during 10 weeks of treatment under open-sale conditions, without face-to-face instruction or counseling. |
| 1655 | Teneggi, Vincenzo, Tiffany, Stephen T, Squassante, Lisa, Milleri, Stefano, Ziviani, Luigi, Bye, Alan. Smokers deprived of cigarettes for 72 h: effect of nicotine patches on craving and withdrawal. Psychopharmacology. 2002;164:177.  **Abstract:** RATIONALEResearch on the effects of nicotine abstinence and nicotine replacement has not provided consistent information about the impact of replacement therapies on tobacco withdrawal and craving.OBJECTIVE. This study investigated craving and withdrawal symptoms over a 72-h period of abstinence from cigarettes.METHODS. Twenty-four healthy volunteers, not intending to quit smoking, were housed in an experimental unit during three 72-h conditions, consisting of either free smoking, enforced smoking cessation with nicotine replacement therapy (NRT) patches, or enforced smoking cessation with placebo patches. The conditions were adhered to using a randomized crossover design, each separated by at least 10 days of washout. Patches, administered in a double-blind fashion, were given as nicotine (21 mg/24 h) and placebo every 24 h. Self-reported cigarette craving and withdrawal were assessed using multi-item scales at fixed intervals over each condition period. Urinary and plasma cortisol levels were also assayed at fixed intervals over each period.RESULTSCraving intensity was significantly lower with free smoke than with placebo and with NRT patches than with placebo. No difference in craving levels was found between those who smoked or those who had NRT patches. Withdrawal symptoms were significantly lower with free smoke than with either placebo or NRT patches, but there was no difference in levels of withdrawal between those on NRT patches and those on placebo. During the placebo and NRT patch periods, craving intensity displayed a circadian rhythm, with craving levels lowest in the morning and peaking in the evening. Nicotine delivered via the patch had no impact on these circadian variations in craving. There was no evidence of systematic temporal variations in craving levels during the free smoking period.CONCLUSIONSThe data suggested that craving and withdrawal symptoms may be sustained by different physiological pathways, and that only selected components of cigarette craving are influenced by NRT. |
| 1660 | Etter, Jean-François, Laszlo, Evelyne, Zellweger, Jean-Pierre, Perrot, Charles, Perneger, Thomas V. Nicotine replacement to reduce cigarette consumption in smokers who are unwilling to quit: a randomized trial. Journal of clinical psychopharmacology. 2002;22:487.  **Abstract:** The objective of this study was to assess whether nicotine replacement therapy, administered in a real-life situation, could reduce cigarette consumption in smokers who were not prepared to quit smoking. Daily smokers of more than 20 cigarettes per day who had no intention to quit smoking in the next 6 months were recruited from the general population and randomly assigned to either a 6-month treatment of nicotine (choice among a 15-mg nicotine patch, a 4-mg nicotine gum, a 10-mg nicotine inhaler, or a combination of these, N = 265), matching placebo products (N = 269), or no intervention (N = 389). Products were sent to participants by mail. Education was limited to a booklet. Of 923 participants, 879 (95%) were followed up after 6 months. Mean baseline consumption was 30 cigarettes per day in all groups. At 6 months, cigarette consumption decreased by a median of 10 cigarettes per day in the nicotine group, 7.5 in the placebo group, and 2.5 among controls ( < 0.04 for all pair-wise comparisons). Smoking cessation rates were low (2%-4%) and did not differ significantly between groups. Quit attempts were less frequent among controls (21%) than among the nicotine (28%, = 0.04) and placebo (27%, = 0.08) subjects. In conclusion, nicotine replacement therapy helped smokers reduce their cigarette consumption and maintain this reduction over 6 months, but a large part of this reduction was attributable to a placebo effect. Nicotine treatment for smoking reduction had no detectable impact on smoking cessation. |
| 1662 | Jolma, Catherine D, Samson, Ricardo A, Klewer, Scott E, Donnerstein, Richard L, Goldberg, Stanley J. Acute cardiac effects of nicotine in healthy young adults. Echocardiography (Mount Kisco, N.Y.). 2002;19:443.  **Abstract:** BACKGROUNDNicotine is known to have many physiologic effects. The influence of nicotine delivered in chewing gum upon cardiac hemodynamics and conduction has not been well-characterized.METHODSWe studied the effects of nicotine in nonsmoking adults (6 male, 5 female; ages 23-36 years) using a double-blind, randomized, cross-over study. Subjects chewed nicotine gum (4 mg) or placebo. After 20 minutes (approximate time to peak nicotine levels), echocardiograms and signal-averaged electrocardiograms (SAECG) were obtained. After 40 minutes, subjects were again given nicotine gum or placebo in cross-over fashion. Standard echocardiographic measurements were made from two-dimensional images. We then calculated end-systolic wall stress (ESWS), shortening fraction (SF), systemic vascular resistance (SVR), velocity for circumferential fiber shortening corrected for heart rate (Vcfc), stroke volume, and cardiac output. P wave and QRS duration were measured from SAECG.RESULTSSignificant differences (P < 0.05) from control or placebo were found for ESWS, mean blood pressure, cardiac output, SVR, heart rate, and P wave duration. No significant changes were seen in left ventricular ejection time (LVET), LV dimensions, SF, contractility (Vcfc), or QRS duration.CONCLUSIONSThese results suggest that nicotine chewing gum increases afterload and cardiac output. Cardiac contractility does not change acutely in response to nicotine gum. Heart rate and P wave duration are increased by chewing nicotine gum. |
| 1665 | Shiffman, Saul, Dresler, Carolyn M, Hajek, Peter, Gilburt, Simon J A, Targett, Darren A, Strahs, Kenneth R. Efficacy of a nicotine lozenge for smoking cessation. Archives of internal medicine. 2002;162:1267.  **Abstract:** BACKGROUNDSince nicotine gum was introduced in the 1980s, nicotine replacement therapy has become the most widely used pharmacological smoking cessation treatment. Some smokers prefer acute oral forms, but many smokers reject chewing gum. We tested the safety and efficacy of a new nicotine polacrilex lozenge for smoking cessation.METHODSDouble-blind, placebo-controlled, randomized clinical trial with parallel arms testing 2- and 4-mg nicotine lozenges. Smokers (n = 1818) were assigned to a lozenge dose on the basis of nicotine dependence, assessed by time to the first cigarette of the day. Low-dependence smokers were randomized to receive the 2-mg nicotine (n = 459) or placebo (n = 458) lozenge; high-dependence smokers, the 4-mg nicotine (n = 450) or placebo (n = 451) lozenge. We assessed abstinence at 6, 12, 24, and 52 weeks and analyzed craving and withdrawal symptoms.RESULTSTreatment with the nicotine lozenge resulted in significantly greater 28-day abstinence at 6 weeks, for the 2-mg (46.0% vs. 29.7%; odds ratio [OR], 2.10; 95% confidence interval [CI], 1.59-2.79; P<.001) and the 4-mg (48.7% vs. 20.8%; OR, 3.69; 95% CI, 2.74-4.96; P<.001) lozenges, compared with placebo. Significant treatment effects were maintained for a full year. Smokers who used more lozenges achieved significantly better treatment effects. Use of the active lozenge also resulted in reduced craving and withdrawal. Most adverse events were moderate and resembled those seen with nicotine gum.CONCLUSIONThe nicotine lozenge is a safe and effective new treatment for smoking cessation in low- and high-dependence smokers. |
| 1670 | Durcan, Michael J, White, Jonathan, Jorenby, Douglas E, Fiore, Michael C, Rennard, Stephen I, Leischow, Scott J, Nides, Mitchell A, Ascher, John A, Johnston, J Andrew. Impact of prior nicotine replacement therapy on smoking cessation efficacy. American journal of health behavior. 2002;26:213.  **Abstract:** OBJECTIVETo examine previous use of nicotine replacement therapy (NRT) on the smoking-cessation efficacy of bupropion sustained release (SR).METHODSSecondary analysis of a parallel-group, randomized, double-blind, placebo-controlled study. Smokers who had, based on self-report, no previous history of NRT (N = 453) or who had used NRT at least once (N = 440) were randomized to receive placebo, bupropion SR, nicotine transdermal system (NTS), or a combination of bupropion SR and NTS.RESULTSBupropion SR showed similar efficacy in participants with or without previous use of NRT.CONCLUSIONBupropion SR is effective in promoting smoking abstinence regardless of prior NRT use. |
| 1672 | Guslandi, Mario, Frego, Roberto, Viale, Edi, Testoni, Pier Alberto. Distal ulcerative colitis refractory to rectal mesalamine: role of transdermal nicotine versus oral mesalamine. Canadian journal of gastroenterology = Journal canadien de gastroenterologie. 2002;16:293.  **Abstract:** BACKGROUNDDistal ulcerative colitis usually responds to treatment with rectal mesalamine, but the management of refractory cases is poorly defined.AIMTo evaluate the possible therapeutic benefit of transdermal nicotine versus oral mesalamine.PATIENTS AND METHODSThirty patients with left-sided ulcerative colitis unresponsive to treatment with a mesalamine 4 g enema at bedtime were randomly allocated to additional therapy with either transdermal nicotine 15 mg daily or oral mesalamine 800 mg tid for four weeks. Clinical remission was evaluated by Rachmilewitz's activity index and confirmed by sigmoidoscopy.RESULTSRemission was observed in 12 of 15 patients receiving additional treatment with nicotine and in five of 15 patients receiving additional treatment with oral mesalamine (P=0.027).CONCLUSIONSThe addition of transdermal nicotine to treatment with mesalamine enemas is significantly superior to combined therapy with oral and rectal mesalamine in patients with distal ulcerative colitis refractory to rectal mesalamine alone. |
| 1675 | Thompson, J C, Wilby, G, Stough, C. The effects of transdermal nicotine on inspection time. Human psychopharmacology. 2002;17:157.  **Abstract:** RATIONALEThe acute administration of nicotine via a cigarette has been demonstrated to enhance inspection time (IT), a measure of rapid visual information processing, while the nicotinic antagonist mecamylamine impairs IT. However, as transdermal nicotine patches provide a slow delivery of nicotine, it is unclear if they produce similar effects to nicotine administered by more rapid methods.OBJECTIVESTo examine the effects of transdermal nicotine on inspection time.METHODSTen moderate smokers were administered nicotine (21 mg/day) or placebo via a transdermal patch in a randomized, double-blind, repeated measures design. Subjects were administered the IT task at baseline, then 1, 3, and 6 h subsequent to patch application.RESULTSThe change in IT from baseline was significantly different in the nicotine condition, relative to placebo, at 3 h but not 1 or 6 h.CONCLUSIONSThe findings suggest that nicotine administered via transdermal patches improves IT, but this improvement may be subject to tolerance. Data from previous studies on tolerance to the cognitive effects of nicotine have been inconclusive. However, the slow delivery of nicotine from patches may be more likely to produce such effects. |
| 1676 | Vial, Robyn J., Jones, Terry E., Ruffin, Richard E., Gilbert, Andrew L.. Smoking cessation program using nicotine patches linking hospital to the community. Journal of Pharmacy Practice and Research. 2002;32:57.  **Abstract:** Objective: To compare the quit rates achieved by participants in a smoking cessation program, initiated in hospital using nicotine patches and continued in either the community pharmacy or hospital setting, with quit rates in a minimal intervention group. Setting: The medical and surgical units at The Queen Elizabeth Hospital (TQEH), a major teaching hospital located in the western suburbs of Adelaide, South Australia. Participants: Inpatients aged over 18 years who smoked 10 or more cigarettes per day. Of 306 smokers screened, 102 were enrolled. Study Procedures: Participants were randomised to either the minimal intervention arm (n = 33), the hospital-based program (n = 35), or the community pharmacy-based program (n = 34). The minimal intervention group received written materials and advice about quitting. The two intervention arms started with a consultation with the research pharmacist and participants were commenced on nicotine patches and were followed after discharge with weekly counselling visits with either the researcher in an outpatient clinic or a community-based pharmacist. Patches were supplied for .00 per week for up to 16 weeks. Exhaled carbon monoxide measurements to confirm abstinence were performed whenever possible. Continuous and point prevalence of abstinence from smoking were assessed 3, 6 and 12 months after enrolment. Results: There was a statistically significant difference in the point prevalence of abstinence between groups at 12 months with 38%, 24% and 4.6% abstinence in the hospital, community pharmacy and minimal intervention arms respectively (X2 = 7.145, df = 2, p = 0.031). The differences seen in continuous abstinence at 12 months (24%, 19% and 4.6% respectively) did not reach statistical significance. Conclusion: A hospital-based smoking cessation program initiated with nicotine patches was equally effective whether continued by hospital- or community-based pharmacists and was superior to minimal intervention without nicotine patches. |
| 1678 | Møller, Ann M, Villebro, Nete, Pedersen, Tom, Tønnesen, Hanne. Effect of preoperative smoking intervention on postoperative complications: a randomised clinical trial. Lancet (London, England). 2002;359:114.  **Abstract:** BACKGROUNDSmokers are at higher risk of cardiopulmonary and wound-related postoperative complications than non-smokers. Our aim was to investigate the effect of preoperative smoking intervention on the frequency of postoperative complications in patients undergoing hip and knee replacement.METHODSWe did a randomised trial in three hospitals in Denmark. 120 patients were randomly assigned 6-8 weeks before scheduled surgery to either the control (n=60) or smoking intervention (60) group. Smoking intervention was counselling and nicotine replacement therapy, and either smoking cessation or at least 50% smoking reduction. An assessor, who was masked to the intervention, registered the occurrence of cardiopulmonary, renal, neurological, or surgical complications and duration of hospital admittance. The main analysis was by intention to treat.FINDINGSEight controls and four patients from the intervention group were excluded from the final analysis because their operations were either postponed or cancelled. Thus, 52 and 56 patients, respectively, were analysed for outcome. The overall complication rate was 18% in the smoking intervention group and 52% in controls (p=0.0003). The most significant effects of intervention were seen for wound-related complications (5% vs 31%, p=0.001), cardiovascular complications (0% vs 10%, p=0.08), and secondary surgery (4% vs 15%, p=0.07). The median length of stay was 11 days (range 7-55) in the intervention group and 13 days (8-65) in the control group.INTERPRETATIONAn effective smoking intervention programme 6-8 weeks before surgery reduces postoperative morbidity, and we recommend, on the basis of our results, this programme be adopted. |
| 1688 | Patten, C A, Martin, J E, Calfas, K J, Lento, J, Wolter, T D. Behavioral treatment for smokers with a history of alcoholism: predictors of successful outcome. Journal of consulting and clinical psychology. 2001;69:796.  **Abstract:** This study examined baseline predictors associated with smoking abstinence among 205 smokers (113 men, 92 women) with a past history of alcoholism. Their mean age was 41.8 years, and 93% were Caucasian. Participants were randomly assigned to standard treatment (ST), behavioral counseling plus exercise (BEX), or behavioral counseling plus nicotine gum (BNIC). Factors multivariately associated with point-prevalence smoking abstinence at posttreatment (1 week after target quit date) were a longer duration of prior smoking abstinence and an interaction between treatment group and having an active 12-step sponsor. ST was more effective for those with an active sponsor, whereas both BEX and BNIC were more effective for those without an active sponsor. At 1-year follow-up, independent predictors of point-prevalence smoking abstinence were a lower Fagerström Tolerance Questionnaire score (K. O. Fagerström, 1978) and fewer years of smoking. |
| 1689 | Vieregge, A, Sieberer, M, Jacobs, H, Hagenah, J M, Vieregge, P. Transdermal nicotine in PD: a randomized, double-blind, placebo-controlled study. Neurology. 2001;57:1032.  **Abstract:** BACKGROUNDAn inverse association between cigarette smoking and the risk of idiopathic PD has been found in many epidemiologic studies. The therapeutic and possible neuroprotective effects of nicotine formulations on parkinsonian symptoms are controversial.METHODSIn a 12-week, randomized, double-blind, placebo-controlled trial, the efficacy and tolerability of transdermal nicotine patches as an add-on treatment for cardinal symptoms were evaluated in 32 nonsmoking patients with PD. After a 1-week run-in phase, patients were randomized to receive nicotine patches (containing 17.5 mg nicotine in the first and 35.0 mg nicotine in the second and third weeks) or identically appearing placebo patches. After this treatment, 3 weeks without patch application followed. The same blinded examiner assessed the patients with the Columbia University Rating Scale, the Webster scale, the Schwab-England scale, a timed walking test, with an instrumental test for fine motor skills and hand tremor, and with the Hamilton Depression Scale.RESULTSNo significant drug effects between both groups were observed in any of the scores and quantitative tests. Side effects were mild and comparable in frequency between both groups.CONCLUSIONSWith the dosage and the period of treatment chosen, transdermal nicotine patches are not effective as an add-on treatment for symptoms of PD. |
| 1691 | Silver, A A, Shytle, R D, Philipp, M K, Wilkinson, B J, McConville, B, Sanberg, P R. Transdermal nicotine and haloperidol in Tourette's disorder: a double-blind placebo-controlled study. The Journal of clinical psychiatry. 2001;62:707.  **Abstract:** BACKGROUNDPreclinical animal and open-trial clinical trials using nicotine gum and the transdermal nicotine patch found that treatment with nicotine potentiates the effects of neuroleptics in reducing the dyskinetic symptoms of Tourette's disorder. We sought to verify and expand these findings in a prospective double-blind placebo-controlled trial.METHODSeventy patients with DSM-IV Tourette's disorder were treated with either transdermal nicotine (7 mg/24 hours) or placebo patches in a 33-day, randomized, double-blind study. Each patient received an individually based optimal dose of haloperidol for at least 2 weeks prior to random assignment to nicotine or placebo treatment. A new patch was worn each day for the first 5 days. On the sixth day, the dose of haloperidol was reduced by 50%. Daily patch applications were then continued for an additional 2 weeks (through day 19), at which time the patch was discontinued, but the 50% dose of haloperidol was continued for an additional 2 weeks (through day 33). Clinical and safety assessments were made at each visit.RESULTSPatients who completed all 19 days of nicotine (N = 27) or placebo (N = 29) patch treatment were used in efficacy analyses. As documented by the Clinician- and Parent-rated Global Improvement scales, transdermal nicotine was superior to placebo in reducing the symptoms of Tourette's disorder. The Yale Global Tic Severity Scale was less sensitive in detecting a placebo/drug difference than were the global improvement scores, suggesting that some of the improvement may not have been related to treatment-related changes in tic severity, but to the emotional and behavioral symptoms. The side effects of nausea and vomiting were significantly more common in the nicotine group (71% [N = 25] and 40% [N = 14]) than in the placebo group (17% [N = 6] and 9% [N = 3]) (nausea, p = .0001; vomiting, p = .004).CONCLUSIONTransdermal nicotine was superior to placebo in reducing behavioral symptoms when patients were receiving an optimal dose of haloperidol, when the dose of haloperidol was reduced by 50%, and when the patch had been discontinued for 2 weeks. These findings confirm earlier open-label findings and suggest that combining nicotinic receptor modulation and neuroleptics could be a therapeutic option for the treatment of Tourette's disorder. While side effects limit chronic use of nicotine, it may be useful on a p.r.n. basis. Further clinical research is warranted to investigate the use of novel nicotinic receptor modulating agents with improved safety profiles over nicotine. |
| 1694 | West, S G, Stoney, C M, Hughes, J W, Matacin, M, Emmons, K M. Oral contraceptive use is associated with increased cardiovascular reactivity in nonsmokers. Annals of behavioral medicine : a publication of the Society of Behavioral Medicine. 2001;23:149.  **Abstract:** Women who smoke and take oral contraceptives (OCs) have significantly increased risk of cardiovascular disease, but the exact mechanismsfor the increased risk are not known. Cardiovascular reactivity to psychological stress may be one mechanism for the enhanced risk, but the small number of studies examining whether OC users who smoke have greater reactivity have produced mixed results. The purpose of this study was to examine the effect of chronic cigarette smoking, acute nicotine administration, and OC use on cardiovascular and lipid reactivity. Sixty healthy women, half of whom had been using OCs for at least the previous 6 months, participated in the study. Approximately two thirds were smokers and were randomized to be tested after either a 12-hr nicotine deprivation or administration of nicotine gum. One third were nonsmokers. Heart rate, blood pressure, and lipid measures were taken at rest, during a videotaped speech task, and during recovery from the task. Results indicated that, among OC nonusers, there was no effect of smoking status or nicotine administration on cardiovascular reactivity. However, among OC users, nonsmokers had significantly greater heart rate and diastolic blood pressure reactivity to stress. These data show that acute nicotine administration, in the form of nicotine gum, has no effect on cardiovascular or lipid stress reactivity in women. However OC use among nonsmoking women is associated with greater cardiovascular reactivity to stress. |
| 1699 | Jamerson, B D, Nides, M, Jorenby, D E, Donahue, R, Garrett, P, Johnston, J A, Fiore, M C, Rennard, S I, Leischow, S J. Late-term smoking cessation despite initial failure: an evaluation of bupropion sustained release, nicotine patch, combination therapy, and placebo. Clinical therapeutics. 2001;23:744.  **Abstract:** OBJECTIVEThe purpose of this study was to evaluate the efficacy of long-term use of bupropion sustained release (SR), the nicotine patch, and the combination of these 2 treatments in patients who initially failed treatment.METHODSThis was a post hoc analysis of a multicenter, double-blind, randomized, placebo-controlled clinical trial in 893 smokers. Patients were randomly assigned to 9 weeks of treatment with placebo (n = 160), bupropion SR (n = 244), nicotine patch (n = 244), or a combination of nicotine patch and bupropion SR (n = 245). The study was originally designed with a follow-up period of 52 weeks. In this analysis, short-term success was defined as smoking cessation after 14 or 21 days of therapy and long-term success was defined as smoking cessation after >21 days of therapy. Patients who did not achieve short-term success were evaluated for long-term success at week 9 (end of treatment), 6 months, and 1 year after the start of the study.RESULTSThe mean age of the smokers was 44 years. The majority (93%) of patients were white, and 52% were female. The study subjects smoked an average of 27 cigarettes per day. Among the 467 patients who initially failed treatment in the first 3 weeks, treatment with bupropion SR alone and in combination with the nicotine patch produced significant increases in successful smoking cessation rates from weeks 4 to 9 (19% bupropion SR or combination, 7% nicotine patch, 7% placebo), at month 6 (11% bupropion SR, 13% combination, 2% nicotine patch, 3% placebo), and at month 12 (10% bupropion SR, 7% combination, 2% nicotine patch, 1% placebo) (P < 0.05 for bupropion SR and combination vs nicotine patch or placebo).CONCLUSIONAmong patients who initially failed treatment, continued therapy with bupropion SR, either alone or in combination with the nicotine patch, resulted in significantly higher short- and long-term smoking cessation rates than treatment with the nicotine patch alone or placebo. |
| 1705 | Thorsteinsson, H S, Gillin, J C, Patten, C A, Golshan, S, Sutton, L D, Drummond, S, Clark, C P, Kelsoe, J, Rapaport, M. The effects of transdermal nicotine therapy for smoking cessation on depressive symptoms in patients with major depression. Neuropsychopharmacology : official publication of the American College of Neuropsychopharmacology. 2001;24:350.  **Abstract:** This study examines the effects of transdermal nicotine patches for smoking cessation on depressive and withdrawal symptoms among 38 non-medicated subjects with Major Depressive Disorder. The study was conducted over a 29-day period, which included a 7 day baseline phase, a 14 day treatment phase, and an 8 day placebo phase. During the treatment phase subjects received either active nicotine patches (N = 18) or placebo patches (N = 20) that were administered in a randomized, double-blind fashion. The target quit date (TQD) was day 8. Significantly, more subjects in the placebo group than in the nicotine group resumed smoking following the TQD (50% vs. 22%). There was little evidence for effects of active nicotine patches on measures of mood (HRSD, BDI, POMS) or withdrawal symptoms among subjects that remained abstinent throughout the study (N = 24). Those who resumed smoking had more severe withdrawal symptoms than those who remained abstinent. One patient in the placebo group (n = 20) became more depressed after 2 weeks of abstinence. None of the patients in the nicotine group (n = 18) became more depressed. |
| 1706 | Gomeni, R, Teneggi, V, Iavarone, L, Squassante, L, Bye, A. Population pharmacokinetic-pharmacodynamic model of craving in an enforced smoking cessation population: indirect response and probabilistic modeling. Pharmaceutical research. 2001;18:537.  **Abstract:** PURPOSEA population pharmacokinetic-pharmacodynamic model accounting for placebo effect was used to relate nicotine concentration and enforced smoking cessation craving score measured by the Tiffany rating scale short form.METHODSTwenty-four smokers were enrolled in a placebo-controlled, randomized, double-blind, three periods, crossover trial. The study objective was to describe the nicotine-induced changes on craving scores. Two modeling strategies based on a mechanistic (indirect response models with drug-related inhibition on the k(in) synthesis rate and with a drug-related stimulation of the k(out) removal rate were evaluated) and a probabilistic (logistic regression) approach were used.RESULTSPlacebo response model properly fitted the circadian changes on craving scores. The analysis revealed that the indirect response model with inhibition on k(in) was the preferred model for the smoking data whereas the preferred model for the Nicotine Replacement Therapy data was the one with stimulation on k(out). The logistic analysis showed that the nicotine concentration was a significant predictor of reduction in craving during the free-smoking period.CONCLUSIONSNicotine dosage regimen can influence the nicotine mechanism of action: an instantaneous delivery at an individually selected time seems to inhibit the onset of craving while constant delivery at a pre-defined time seems to attenuate the craving. |
| 1715 | Kapur, Bhushan, Hackman, Richard, Selby, Peter, Klein, Julia, Koren, Gideon. Randomized, double-blind, placebo-controlled trial of nicotine replacement therapy in pregnancy. Current Therapeutic Research - Clinical and Experimental. 2001;62:274.  **Abstract:** Background: Smoking in pregnancy is associated with increased perinatal risks, including intrauterine growth retardation, stillbirth, and the sudden infant death syndrome. Reducing maternal smoking using nicotine replacement therapy (NRT) is a possible way to decrease the risks to the fetus. Objective: The purpose of this study was to examine the efficacy of NRT in reducing smoking among pregnant women who were heavy smokers and who could not quit smoking during their first trimester. Methods: In this double-blind, placebo-controlled trial, pregnant women (12 to 24 weeks' gestation) who smoked ≥ 15 cigarettes per day were randomized to receive a daily, 18-hour patch of nicotine 15 mg for 8 weeks, 10 mg for an additional 2 weeks, and 5 mg for the last 2 weeks, or an identical placebo patch. At baseline and at 1, 4, and 8 weeks, women received counseling, and serum and salivary cotinine levels were measured. Results: Seventeen women received NRT and 13 received placebo. In the NRT group, 4 women (23.5%) successfully completed the program and quit smoking during their second trimester. None of the 13 women who received placebo and counseling completed the program or quit smoking. The difference in success rates was not statistically significant (P = 0.11). Conclusions: The effectiveness of NRT beyond the first trimester in pregnant women who smoke heavily is questionable, but NRT may be helpful in a minority of these women. |
| 1718 | Wisborg, K, Henriksen, T B, Jespersen, L B, Secher, N J. Nicotine patches for pregnant smokers: a randomized controlled study. Obstetrics and gynecology. 2000;96:967.  **Abstract:** OBJECTIVETo assess the effect of nicotine patches on continine-validated smoking cessation in pregnant women and the effect of nicotine on birth weight and preterm delivery.METHODSPregnant women who smoked ten or more cigarettes after the first trimester (N = 250) were randomly assigned to receive nicotine patches (n = 124) or placebo patches (n = 126). Women randomized to nicotine were treated with 15-mg patches (16 hours/day) for 8 weeks, and 10-mg patches (16 hours/day) for 3 weeks.RESULTSOverall, 26% stopped smoking and 14% were nonsmokers 1 year after delivery. There was no difference between nicotine and placebo groups. At the end of the intervention, the mean value of cotinine in saliva in women assigned to nicotine was 120 ng/mL and placebo 153 ng/mL (mean difference -33; 95% CI -72, 6 ng/mL). Mean birth weight difference was 186 g (95% CI 35, 336 g) higher in the nicotine than placebo group, and there was an insignificantly lower rate of low birth weight (under 2500 g) in the former group. There was no difference in the rate of preterm delivery between the two groups.CONCLUSIONNicotine patches had no influence on smoking cessation during pregnancy, although they might increase birth weight in comparison with placebo. |
| 1720 | Hughes, J R, Rose, G L, Callas, P W. Nicotine is more reinforcing in smokers with a past history of alcoholism than in smokers without this history. Alcoholism, clinical and experimental research. 2000;24:1633.  **Abstract:** BACKGROUNDWhether smokers with a past history (PH) but not current history of alcohol dependence are more nicotine dependent than smokers with no such history (NH) is unclear. The present study was an experimental test of this hypothesis.METHODTwenty PH and 10 NH smokers abstained from smoking for 16 hr on each of 4 days. On each of 3 days, participants received three doses per day of 0, 2, or 4 mg nicotine gum in a within-subjects, randomized, double-blind, crossover design. To examine subjective effects, participants completed the Profile of Mood States, Addiction Research Inventory, and other ratings before and after each dose. To examine nicotine reinforcement, participants reported preference among the gums, reported on money versus gum choices, and, on the 4th day, underwent a double-blind self-administration test.RESULTSAcross the 21 subjective measures, with one exception, PH and NH smokers did not differ in subjective response to nicotine. However, across all three reinforcement measures, nicotine was a more potent reinforcer in PH than NH smokers.CONCLUSIONSThese results provide a behavioral mechanism to explain prior findings that PH smokers are more nicotine dependent than NH smokers. |
| 1721 | Fishbein, L, O'Brien, P, Hutson, A, Theriaque, D, Stacpoole, P W, Flotte, T. Pharmacokinetics and pharmacodynamic effects of nicotine nasal spray devices on cardiovascular and pulmonary function. Journal of investigative medicine : the official publication of the American Federation for Clinical Research. 2000;48:435.  **Abstract:** BACKGROUNDA nasal spray form of nicotine replacement therapy (Nicotrol NS, McNeil Consumer Products Co, Fort Washington, Pa) has been approved and, because of its rapid absorption across the nasal mucosa, may be more effective than nicotine gum or transdermal patches. We tested the hypothesis that the nicotine absorbed into the nasal mucosa would produce significant changes in hemodynamics and pulmonary function in 20 healthy, nonsmoking men and women.METHODSIn this double-blind, randomized study of Nicotrol NS versus placebo, we measured serum nicotine concentrations, blood pressure, heart rate, and indices of pulmonary function at timed intervals before and after nasal spray administration of 3 mg of nicotine.RESULTSA peak serum nicotine concentration of 4.71 +/- 3.16 ng/mL occurred 10 minutes after drug administration. The maximum change in systolic blood pressure occurred 5 minutes after dosing and was significantly related to nicotine administration (7.1 +/- 9.4% for the nicotine group vs -1.6 +/- 7.3% for the placebo; P = 0.03). In contrast, neither diastolic blood pressure (P = 0.8) nor heart rate (P = 0.07) changed significantly after nicotine administration, when compared with placebo. Pulmonary function was not altered acutely by a single inhalation of nicotine. Pharmacokinetic modeling revealed a classic one-compartment model in which nicotine is absorbed into the systemic circulation by a zero-order process and eliminated by a first-order process.CONCLUSIONSIn this population of nonsmokers, hemodynamic effects of the nicotine nasal spray were observed shortly after administration and before the peak serum nicotine concentration. |
| 1723 | Shiffman, S, Khayrallah, M, Nowak, R. Efficacy of the nicotine patch for relief of craving and withdrawal 7-10 weeks after cessation. Nicotine & tobacco research : official journal of the Society for Research on Nicotine and Tobacco. 2000;2:371.  **Abstract:** The objective of this study was to test the efficacy of the nicotine patch for relief of craving and withdrawal beyond 6 weeks (during weeks 7-10), among heavy, dependent smokers, using a multicenter, randomized, double-blind, placebo-controlled trial. The subjects were 421 nicotine-dependent smokers who had quit smoking with the NicoDerm CQ nicotine patch, recruited from among enrollees in the NicoDerm CQ Committed Quitters behavioral program. Following 6 weeks of 21-mg active patch use, participants who had not relapsed were randomized to receive either active or placebo patches for an additional 4 weeks with tapering (14-mg patch in weeks 7-8 and 7-mg patch in weeks 9-10). Craving and withdrawal symptoms were assessed daily by phone interview during weeks 7-10 following cessation. The active patch group reported significantly lower craving and withdrawal, and higher positive mood scores in weeks 7-8 (p < 0.001, 0.001, and 0.03, respectively) and weeks 9-10 (p < 0.02, 0.01, and 0.02, respectively) compared to the placebo group. It was concluded that some smokers may be best served by 10 weeks of patch treatment. This study demonstrated the utility of continuing patch treatment for symptom relief in weeks 7-10 of abstinence. Future research should examine the optimal duration of treatment, which may vary across different classes of smokers. |
| 1726 | Killen, J D, Fortmann, S P, Schatzberg, A F, Hayward, C, Sussman, L, Rothman, M, Strausberg, L, Varady, A. Nicotine patch and paroxetine for smoking cessation. Journal of consulting and clinical psychology. 2000;68:883.  **Abstract:** Smokers (N = 224) were randomized to 1 of 3 groups: (a) transdermal system (TNS) + placebo; (b) TNS + paroxetine (20 mg); (c) TNS + paroxetine (40 mg). Assignment to treatment was double-blind. Nicotine patch (TNS) treatment was provided for 8 weeks; paroxetine or placebo was provided for 9 weeks. Abstinence rates at Weeks 4, 10, and 26 were as follows: (a) TNS + placebo: 45%, 36%, and 25%; (b) TNS + paroxetine (20 mg): 48%, 33%, and 21%; (c) TNS + paroxetine (40 mg): 57%, 39%, and 27%. The differences were not statistically significant. The combined treatment was more effective in reducing both craving and depression symptoms associated with smoking cessation. A subgroup analysis comparing compliant participants was also conducted. Abstinence rates at Weeks 4, 10, and 26 were as follows: (a) TNS + placebo: 46%, 35%, and 24%; (b) TNS + paroxetine (20 mg): 64%, 43%, and 33%; (c) TNS + paroxetine (40 mg): 74%, 51%, and 38%. The differences between paroxetine groups and placebo at Week 4 were statistically significant. Although paroxetine may add value to the current standard of care in excess of potential risk, more conclusive evidence is needed. |
| 1730 | Bolliger, C T, Zellweger, J P, Danielsson, T, van Biljon, X, Robidou, A, Westin, A, Perruchoud, A P, Säwe, U. Smoking reduction with oral nicotine inhalers: double blind, randomised clinical trial of efficacy and safety. BMJ (Clinical research ed.). 2000;321:329.  **Abstract:** OBJECTIVESTo determine whether use of an oral nicotine inhaler can result in long term reduction in smoking and whether concomitant use of nicotine replacement and smoking is safe.DESIGNDouble blind, randomised, placebo controlled trial. Four month trial with a two year follow up.SETTINGTwo university hospital pulmonary clinics in Switzerland.PARTICIPANTS400 healthy volunteers, recruited through newspaper advertisements, willing to reduce their smoking but unable or unwilling to stop smoking immediately.INTERVENTIONActive or placebo inhaler as needed for up to 18 months, with participants encouraged to limit their smoking as much as possible.MAIN OUTCOME MEASURESNumber of cigarettes smoked per day from week six to end point. Decrease verified by a measurement of exhaled carbon monoxide at each time point compared with measurement at baseline.RESULTSAt four months sustained reduction of smoking was achieved in 52 (26%) participants in the active group and 18 (9%) in the placebo group (P<0.001; Fisher's test). Corresponding figures after two years were 19 (9.5%) and 6 (3.0%) (P=0.012).CONCLUSIONNicotine inhalers effectively and safely achieved sustained reduction in smoking over 24 months. Reduction with or without nicotine substitution may be a feasible first step towards smoking cessation in people not able or not willing to stop abruptly. |
| 1731 | Wallström, M, Nilsson, F, Hirsch, J M. A randomized, double-blind, placebo-controlled clinical evaluation of a nicotine sublingual tablet in smoking cessation. Addiction (Abingdon, England). 2000;95:1161.  **Abstract:** AIMSEvaluation of the clinical efficacy and safety of a nicotine 2-mg sublingual tablet in smoking cessation.DESIGNA randomized, double-blind, placebo-controlled study of smokers using the 2-mg tablet for 3-6 months with follow-up to 12 months. Dosing was established according to baseline nicotine dependence, scored on the Fagerström Tolerance Questionnaire (FTQ): FTQ > or = 7, two tablets/hour (maximum 40/day); FTQ < 7, one tablet/hour (maximum 20/day).SETTINGSmoking cessation programme in a department of oral and maxillofacial surgery.PARTICIPANTSA total of 247 adult smokers, smoking > or = 10 cigarettes/day for > or = 3 years, of whom 123 received active and 124 placebo treatment. The study was powered to detect difference at 6 months.MEASUREMENTSEfficacy and safety were evaluated at 6 weeks and 3, 6 and 12 months. Self-reported abstinence was verified by exhaled CO < 10 p.p.m.FINDINGSSuccess rates for complete abstinence (no slips after 2 weeks) for active vs. placebo were 50% vs. 29% at 6 weeks, 42% vs. 23% at 3 months, 33% vs. 18% at 6 months and 23% vs. 15% at 12 months (p < 0.001, 0.001, 0.005 and p = 0.14), respectively. Craving during the first 8 days was significantly reduced among highly dependent smokers on active treatment compared to placebo. Baseline mucosal lesions among abstinent subjects were reduced during the treatment period and at the non-treatment follow-up. Adverse events were mild and tolerable, the most common being irritation and soreness in the mouth and throat.CONCLUSIONThe nicotine sublingual tablet increased the smoking cessation rate compared to placebo, reduced craving in highly dependent smokers and was well tolerated. |
| 1733 | Allen, S S, Hatsukami, D, Christianson, D, Brown, S. Effects of transdermal nicotine on craving, withdrawal and premenstrual symptomatology in short-term smoking abstinence during different phases of the menstrual cycle. Nicotine & tobacco research : official journal of the Society for Research on Nicotine and Tobacco. 2000;2:231.  **Abstract:** Gender differences in nicotine response with regard to nicotine sensitivity and withdrawal symptomatology have been reported, with the suggestion that ovarian hormones play a role. Few studies, however, have directly assessed hormonal influences on nicotine response. This study focused on the effects of a transdermal nicotine patch in women during acute smoking abstinence when tested in different phases of their menstrual cycle. Thirty women were randomized to order of menstrual cycle phase (late luteal or follicular) and patch condition (active or placebo). Two 7-day outpatient-testing periods were conducted with 2 days of ad lib baseline smoking and 5 days of smoking abstinence. Dependent measures included scores from the Minnesota Nicotine Withdrawal Scale, Questionnaire on Smoking Urges, and Premenstrual Assessment Form, as well as weight. The severity of both premenstrual symptomatology and nicotine withdrawal symptoms was greater in the late luteal phase. Correlation coefficients confirmed overlap between premenstrual and withdrawal symptomatology, especially for the affect subscale. A significant patch effect was observed, showing diminished craving and premenstrual affect on pain subscale scores for women on active patch. Results showed that nicotine craving and premenstrual pain and water retention symptoms were diminished in women on transdermal patch, and that this effect was greatest in the late luteal phase. In addition, the greatest weight gain was demonstrated for participants in the late luteal phase, placebo condition. In summary, during short-term smoking abstinence in women, transdermal nicotine appears to have a more pronounced effect in the late luteal phase than in the follicular phase in reducing craving and certain premenstrual symptoms. |
| 1734 | Hill, A L, Roe, D J, Taren, D L, Muramoto, M M, Leischow, S J. Efficacy of transdermal nicotine in reducing post-cessation weight gain in a Hispanic sample. Nicotine & tobacco research : official journal of the Society for Research on Nicotine and Tobacco. 2000;2:247.  **Abstract:** This study examined nicotine replacement effects and pre-quit smoking characteristics with respect to post-cessation weight gain in a primarily Mexican-American sample of Hispanic smokers. Hispanic smokers (108) were randomly assigned to receive either nicotine transdermal patch or placebo patch for 10 weeks, during which time smoking status and weight change were measured. The overall weight gain experienced by the successful quitters was greater than that experienced by non-quitters. Differences between quitters and non-quitters were significant for both females (2.0 vs. 0.86 kg; p < 0.05) and for males (2.3 vs. 1.2 kg; p < 0.05) at 6 weeks post-randomization. At the end of the 10-week treatment, only the females showed a significant difference in weight gain between the quitters and non-quitters (2.8 vs. 1.1; p < 0.01). Results of the random effects regression models for quitters indicated that the active group gained weight at a significantly lower rate than the placebo group (p = 0.047) for females, but not for males (p = 0.87). Years of smoking (p = 0.001) but not baseline cigarettes (p = 0.13) were significant predictors of weight for females, but not for males (p = 0.24 and 0.72, respectively). The use of nicotine patch treatment for smoking cessation effectively attenuated weight gain for successful female quitters compared with placebo-treated quitters. Identification of pre-quit smoking characteristics may prove useful in predicting who is most likely to gain weight when quitting smoking. |
| 1735 | Hughes, J R, Rose, G L, Callas, P W. Do former smokers respond to nicotine differently from never smokers? A pilot study. Nicotine & tobacco research : official journal of the Society for Research on Nicotine and Tobacco. 2000;2:255.  **Abstract:** Nine former, 11 never, and 10 current smokers abstained from smoking for 16 h on each of 4 days. On each of 3 days, participants received three doses per day of 0-, 2-, or 4-mg nicotine gum in a randomized, double-blind, crossover design. To examine subjective effects, participants completed the POMS, ARCI, and other ratings before and after each dose. To examine nicotine reinforcement, participants reported preferences among the gums, reported gum vs. money choices plus, on a fourth day, underwent a double-blind self-administration test. Former and never smokers did not differ on any of the subjective or reinforcement outcomes. Current smokers did prefer and self-administer more nicotine than former and never smokers. These results contradict our prior finding that former and never smokers differ in their response to nicotine. We consider the present study a more valid test than our prior study for reasons outlined in the discussion. Our results fail to confirm theories that those destined to be smokers are more or less sensitive to nicotine and that nicotine use causes irreversible brain changes that increase nicotine reinforcement. |
| 1739 | Molander, L, Lunell, E, Fagerström, K O. Reduction of tobacco withdrawal symptoms with a sublingual nicotine tablet: a placebo controlled study. Nicotine & tobacco research : official journal of the Society for Research on Nicotine and Tobacco. 2000;2:187.  **Abstract:** Many smokers are unable to use gum as a cessation aid due to fillings, bridgework and dyspepsia or they reject it for esthetic reasons. The nicotine sublingual tablet is a new alternative to the nicotine polacrilex chewing gum that does not necessitate chewing. Twenty subjects used 2-mg sublingual nicotine tablets and placebo in a double-blind randomized crossover study of 2-day smoke-free periods. Craving and other withdrawal symptoms were rated on 100-mm visual analog scales (VAS) nine times over each 2-day period. Plasma nicotine concentrations in the afternoon of each study day were determined. A blood sample was also taken in the afternoon of a 2-day period with normal smoking. The mean number of nicotine tablets was 10 and seven on days 1 and 2, respectively. The corresponding number for placebo tablets was six and five. No subject used less than five tablets on any study day. The degree of nicotine substitution, defined as the quotient between the plasma levels achieved with the sublingual tablet and smoking, was 43, 30 and 23% for smokers with FTQ < or = 6, FTQ = 7 and FTQ > or = 8 (Fagerström Tolerance Questionnaire), respectively. Active treatment was significantly superior in decreasing craving and other withdrawal symptom scores compared to placebo treatment. Mean total scores were reduced by approximately 50%. Adverse events were mainly symptoms of local irritation such as a burning sensation in the throat or under the tongue, a lump in the throat and heartburn. These results demonstrate partial relief of the tobacco withdrawal syndrome with use of the sublingual tablet similar to that achieved from other forms of nicotine replacement. |
| 1740 | Hatsukami, D K, Grillo, M, Boyle, R, Allen, S, Jensen, J, Bliss, R, Brown, S. Treatment of spit tobacco users with transdermal nicotine system and mint snuff. Journal of consulting and clinical psychology. 2000;68:241.  **Abstract:** The purpose of this study was to examine the effects of nicotine patch and mint snuff (a nonnicotine product) on craving, withdrawal symptoms, and treatment outcome. This study involved a 2 x 2 factorial design, with Active Nicotine Versus Placebo Patch as one of the factors and Mint Snuff Versus No Mint Snuff as the other factor. Spit tobacco users (N = 402, n = 100-101 in each condition) were randomly assigned to 1 of the 4 treatment conditions for a period of 10 weeks. Treatment outcome was measured up to 62 weeks. The results showed that the nicotine patch was effective in increasing short-term abstinence over the placebo patch and in reducing craving and withdrawal signs and symptoms from spit tobacco. Although mint snuff was not effective in enhancing treatment outcome, it reduced craving and withdrawal symptoms. No interaction effects were observed. At this time, the use of the nicotine patch and mint snuff should be primarily considered for the reduction of craving and withdrawal symptoms. |
| 1742 | Patten, Christi A., Martin, John E., Calfas, Karen J., Brown, Sandra A., Schroeder, Darrell R.. Brief report effect of three smoking cessation treatments on nicotine withdrawal in 141 abstinent alcoholic smokers. Addictive Behaviors. 2000;25:301.  **Abstract:** This prospective study examined the effect of three behavioral smoking interventions and reductions in cigarettes smoked per day on nicotine withdrawal symptoms in 141 abstinent alcoholic smokers (73 men, 68 women). The participants' mean ± SD age was 41.4 ± 9.2 years. They smoked an average of 27.7 ± 12.1 cigarettes per day and reported 4.1 ± 4.3 years of current abstinent from alcohol and other drugs of dependence. Participants were randomly assigned to a 12-week program of standard treatment (ST, n = 61), behavioral counseling plus exercise (BEX, n = 39), or behavioral counseling plus nicotine gum (BNIC, n = 41). All three conditions included instructions to reduce the number of cigarettes smoked per day prior to the target quit date (TQD). The TQD was week 4 for ST subjects and week 8 for those in the BEX and BNIC groups. The post-treatment assessment occurred one week after TQD. The Profile of Mood States (POMS) and the Beck Depression Inventory were administered at baseline and posttreatment to assess nicotine withdrawal. Significant increases were detected for the POMS total mood disturbance score, and the depression, tension, anger and confusion subscales, while vigor scores decreased (all p < 0.03). Withdrawal change scores were not found to be associated with treatment condition or percentage reduction in cigarettes, and there was no evidence of a significant interaction of treatment and cigarette reduction. Results are discussed in relation to implications for treatment and for future research. Copyright (C) 2000 Elsevier Science Ltd. |
| 1744 | Garvey, A J, Kinnunen, T, Nordstrom, B L, Utman, C H, Doherty, K, Rosner, B, Vokonas, P S. Effects of nicotine gum dose by level of nicotine dependence. Nicotine & tobacco research : official journal of the Society for Research on Nicotine and Tobacco. 2000;2:53.  **Abstract:** We used the Heaviness of Smoking Index, a subset of the Fagerstrom Test for Nicotine Dependence, to classify 608 cigarette smokers planning a cessation attempt as low or high in nicotine dependence. Subjects within each level of dependence were then randomly assigned to placebo, 2-mg, or 4-mg nicotine gum treatment. Subjects were also provided brief (5-10 min per visit) behavioral counseling during a 1-year period of follow-up. At 1 year post-cessation, quit rates were 11.2, 19.5, and 18.4% for low-dependence smokers receiving placebo, 2-mg, and 4-mg gum, respectively (plinear trend = 0.20). For high-dependence smokers, quit rates at 1 year were 6.1, 15.7, and 20.7% for the placebo, 2-mg, and 4-mg gum conditions, respectively (plinear trend = 0.002). The interaction of nicotine-gum dose and dependence group was not significant (p = 0.42), nor did the 2-mg and 4-mg doses differ significantly in effectiveness, though both 2-mg and 4-mg gum were significantly more effective than placebo gum. We also found a significant dose-related effect for nicotine gum to moderate post-cessation heart-rate decline. Other variables related to abstinence at 1 year post-cessation were a longer period of abstinence on a prior quit attempt, being married, higher education level, and having a non-smoking spouse or significant other. Results indicate that nicotine gum is a significant aid to smoking cessation, more than doubling the odds of successful cessation compared to the odds for placebo-gum users. The 4-mg dose provided a non-significant increase in cessation rates for high-dependence smokers. Smoking history and demographic variables also have a significant impact on the outcome of a quit-smoking attempt. |
| 1755 | Hays, J T, Croghan, I T, Schroeder, D R, Offord, K P, Hurt, R D, Wolter, T D, Nides, M A, Davidson, M. Over-the-counter nicotine patch therapy for smoking cessation: results from randomized, double-blind, placebo-controlled, and open label trials. American journal of public health. 1999;89:1701.  **Abstract:** OBJECTIVESThe purpose of this study was to determine the efficacy and safety of the nicotine patch for smoking cessation in an over-the-counter environment. The years of study were 1994 to 1995.METHODSParallel 6-week trials were conducted: a placebo-controlled trial of no-cost 22-mg, 24-hour nicotine patch therapy and an open label trial of the same therapy with patches purchased by subjects. Participants (n = 958) were 18 years or older, had smoked at least 15 cigarettes daily for at least 6 months, and were enrolled at 3 study sites. The main outcome measure was self-reported smoking abstinence confirmed by expired carbon monoxide measurements.RESULTSSmoking cessation rates in the placebo-controlled trial were 16.8% and 9.6% at week 6 and 8.7% and 4.3% at week 24 for the active patch and placebo groups, respectively. Smoking cessation rates in the open label-pay trial were 19.0% and 10.8% at weeks 6 and 24, respectively. A slight increase in adverse cardiovascular events was noted only in the open label-pay group in comparison with the placebo group.CONCLUSIONSIn an over-the-counter environment, the 22-mg, 24-hour nicotine patch is effective and safe for smoking cessation treatment. |
| 1756 | Howard-Pitney, B, Killen, J D, Fortmann, S P. Quitting chew: results from a randomized trial using nicotine patches. Experimental and clinical psychopharmacology. 1999;7:362.  **Abstract:** The authors examined the efficacy of transdermal nicotine replacement for cessation in 410 adult nonsmoking chewing tobacco users. Participants were randomly assigned to 6 weeks of 15-mg nicotine patch plus behavioral treatment or placebo patch plus behavioral treatment. All participants received the same behavioral treatment of 2 pharmacy visits, 2 support calls, and self-help materials. At 6 months after treatment, biochemically confirmed point-prevalence rates (no chewing in the last 7 days) in the active (38%) and placebo (34%) groups were high and not significantly different. The difference in relapse (no chewing for 7 consecutive days) between the active patch group (33%) and placebo group (48%) was significant at 6 months (p = .003). Nicotine dependence and age predicted nonrelapse at 6 months. The results suggest that nicotine replacement may improve chewers' chances of abstinence. |
| 1759 | Nordstrom, B L, Kinnunen, T, Utman, C H, Garvey, A J. Long-term effects of nicotine gum on weight gain after smoking cessation. Nicotine & tobacco research : official journal of the Society for Research on Nicotine and Tobacco. 1999;1:259.  **Abstract:** Smoking cessation usually results in weight gain. Nicotine gum therapy has been found to reduce weight gain in the first months after cessation, but its long-term effects are not fully known. The present study randomly assigned 608 smokers to receive placebo, 2 or 4 mg nicotine gum. In a follow-up analysis to the short-term weight change results reported in a previous paper [Doherty, Militello, Kinnunen, & Garvey (1996), Journal of Consulting and Clinical Psychology 64:799-807], we examined the effects of the nicotine gum on weight change for 1 year after cessation among the 92 1-year abstainers. We found that weight change showed some variation with gum dose (active vs. placebo), but that weight change appeared to depend more strongly on the percentage of pre-cessation cotinine levels replaced by the nicotine gum. Participants who replaced higher proportions of their pre-cessation cotinine during the gum therapy period gained less weight during the first year post-cessation than those who replaced less cotinine, with those who replaced greater than 90% of their cotinine gaining only 1.7 kg by 1 year post-cessation. These findings suggest that future research is warranted to determine whether sufficiently high levels of nicotine replacement can help to permanently reduce cessation-related weight gain. |
| 1762 | Wong, G Y, Wolter, T D, Croghan, G A, Croghan, I T, Offord, K P, Hurt, R D. A randomized trial of naltrexone for smoking cessation. Addiction (Abingdon, England). 1999;94:1227.  **Abstract:** AIMSTo evaluate the efficacy and safety of orally administered naltrexone, alone or in combination with nicotine patches, as a treatment for cigarette smoking.DESIGNRandomized, partially-blinded, 2 x 2 factorial trial using naltrexone (active vs. placebo) and nicotine patches (active vs. none).PARTICIPANTSOne hundred cigarette smokers.INTERVENTIONTwelve weeks of either placebo-only, naltrexone-only, placebo with nicotine patches or naltrexone with nicotine patches. The naltrexone dose was 50 mg taken once daily, and the nicotine patch dose was 21 mg/24-hour for the first 8 weeks and 14 mg/24-hour for the remaining 4 weeks. Brief behavioral intervention was provided at each visit.MEASUREMENTSOne-week point-prevalence smoking abstinence rates confirmed by an expired air carbon monoxide level of 8 parts per million (ppm) or less, daily cigarette smoking and cigarette craving.FINDINGSAt the end of treatment, there was no effect of naltrexone on smoking abstinence. The smoking abstinence rates were 19% and 22% for the placebo only and naltrexone only treatment groups, respectively, and 48% and 46% for the placebo with nicotine patch and naltrexone with nicotine patch groups, respectively. However, the effect of the nicotine patch at this time was significant (p = 0.006), but not at the 6-month follow-up. No significant effect of naltrexone was observed on daily cigarette smoking on cigarette craving during the study.CONCLUSIONSThe opioid antagonist naltrexone was not found to be effective for smoking cessation and had no significant effect on daily cigarette consumption or craving. The results of the present study provide no support for the use of naltrexone, alone or in combination with nicotine patches, as a therapeutic treatment for smoking cessation. |
| 1766 | Dalack, G W, Becks, L, Hill, E, Pomerleau, O F, Meador-Woodruff, J H. Nicotine withdrawal and psychiatric symptoms in cigarette smokers with schizophrenia. Neuropsychopharmacology : official publication of the American College of Neuropsychopharmacology. 1999;21:195.  **Abstract:** The prevalence of smoking is markedly elevated in schizophrenia. Low smoking cessation rates and reports that some smokers with schizophrenia experience an acute increase in symptoms during attempts to quit smoking, suggest a self-medication model. Alternatively, smoking may modulate medication side effects. The effects of treated and untreated smoking abstinence on psychotic symptoms and medication side effects were examined in this study. Nineteen outpatients with schizophrenia or schizoaffective disorder participated in a randomized, double-blind, balanced crossover study: 1 day of ad libitum smoking followed by 3 days of acute smoking abstinence while wearing 22 mg/day active or placebo transdermal nicotine patches, with a return to 3 days of smoking between patch conditions. Daily symptom and side-effect ratings, nicotine and cotinine blood levels were collected. Twelve subjects completed the study. Neither positive symptoms nor mood symptoms changed. An increase in negative symptoms during the first abstinent day occurred in both placebo and active patch conditions, but was not sustained over subsequent abstinent days. Despite physiological signs of withdrawal, completers did not endorse increased nicotine withdrawal symptoms. Dropouts reported higher withdrawal symptoms, but also had no increase in psychiatric symptoms in either phase of the study. Of note, dyskinesias decreased during abstinence and placebo patch treatment, but increased during abstinence and the active patch conditions. Acute exacerbation of psychiatric symptoms is an unlikely explanation for any difficulty smokers with schizophrenia have in early abstinence. |
| 1771 | Hughes, J R, Lesmes, G R, Hatsukami, D K, Richmond, R L, Lichtenstein, E, Jorenby, D E, Broughton, J O, Fortmann, S P, Leischow, S J, McKenna, J P, Rennard, S I, Wadland, W C, Heatley, S A. Are higher doses of nicotine replacement more effective for smoking cessation?. Nicotine & tobacco research : official journal of the Society for Research on Nicotine and Tobacco. 1999;1:169.  **Abstract:** This study determined whether higher dose nicotine patches are more efficacious than lower dose patches among heavy smokers. A randomized double-blind study compared 0, 21, 35, and 42 mg/day of a 24-h patch in 1039 smokers (> or = 30 cigarettes/day) at 12 clinical sites in the USA and one in Australia. Daily patches were used for 6 weeks followed by tapering over the next 10 weeks. Weekly group therapy occurred. Biochemically validated self-reported quit rates at 6, 12, 26, and 52 weeks post-cessation were measured. Quit rates were dose-related at all follow-ups (p < 0.01). Continuous, biochemically verified abstinence rates for the 0, 21, 35, and 42 mg doses at the end of treatment (12 weeks) were 16, 24, 30, and 39%. At 6 months, the rates were 13, 20, 20, and 26%. Among the 11 sites with 12 month follow-up (n = 879), the quit rates were 7, 13, 9, and 19%. In post-hoc tests, none of the active doses were significantly different from each other at any follow-up. The rates of dropouts due to adverse events for 0, 21, 35, and 42 mg were 3, 1, 3, and 6% (p = n.s.). Our results are similar to most prior smaller studies; i.e., in heavy smokers higher doses increase quit rates slightly. Longer durations of treatment may be necessary to show greater advantages from higher doses. |
| 1773 | Wetter, D W, Fiore, M C, Young, T B, McClure, J B, de Moor, C A, Baker, T B. Gender differences in response to nicotine replacement therapy: objective and subjective indexes of tobacco withdrawal. Experimental and clinical psychopharmacology. 1999;7:135.  **Abstract:** K. A. Perkins (1996) recently proposed that nicotine reinforcement controls smoking to a greater degree among men than women and that consequently, nicotine replacement therapy (NRT) during smoking cessation should benefit men more than women. The authors tested this hypothesis. Polysomnographic measures of sleep and self-report indexes of tobacco withdrawal were collected pre- and postcessation from an active nicotine patch group and a placebo patch group in a randomized, double-blind clinical trial (N = 34). Objective sleep parameters supported Perkins's hypothesis and indicated that among women, NRT may be less effective at suppressing certain withdrawal responses compared with men and may produce some iatrogenic effects. Valid and reliable self-report measures of withdrawal did not reveal gender differences in response to NRT. |
| 1774 | Kanner, R E, Connett, J E, Williams, D E, Buist, A S. Effects of randomized assignment to a smoking cessation intervention and changes in smoking habits on respiratory symptoms in smokers with early chronic obstructive pulmonary disease: the Lung Health Study. The American journal of medicine. 1999;106:410.  **Abstract:** PURPOSETo evaluate the effects of randomly assigning smokers who have early chronic obstructive pulmonary disease (COPD) to a smoking-cessation intervention on the symptoms of chronic cough, chronic phlegm production, wheezing and shortness of breath, and to determine the effects of quitting smoking on these symptoms.SUBJECTS AND METHODSA total of 5,887 male and female smokers 35 to 60 years of age with early COPD [defined as a forced expiratory volume in the first second (FEV1) of 55% to 90% of predicted and FEV1/forced vital capacity (FVC) <0.70] were enrolled in a 5-year clinical trial. Two-thirds of participants were randomly assigned to smoking-intervention groups and one-third to a usual-care group. The intervention groups attended 12 intensive smoking-cessation sessions that included behavior modification techniques and the use of nicotine chewing gum. One intervention group was treated with ipratropium bromide by inhaler; the other intervention group received placebo inhalers. The usual-care group was advised to stop smoking. All participants were followed annually. Smoking status was biochemically validated by salivary cotinine measurements or exhaled carbon monoxide values.RESULTSValidated 5-year sustained smoking cessation occurred in 22% of participants in the intervention compared with only 5% of participants in the usual-care group. At the end of the study, the prevalence of each of the four symptoms in the two intervention groups was significantly less than in the usual-care group (P <0.0001). For example, among participants who did not report cough at baseline, 15% of those in the intervention groups had cough at least 3 months during the year, compared with 23% of those in usual care. Sustained quitters had the lowest prevalence of all four symptoms, whereas continuous smokers had the greatest prevalence of these symptoms. Changes in symptoms occurred primarily in the first year after smoking cessation. Respiratory symptoms were associated with greater declines in FEV1 during the study (P <0.001). Ipratropium bromide had no long-term effects on respiratory symptoms.CONCLUSIONSIn this prospective randomized trial using an intention-to-treat analysis, smokers with early COPD who were assigned to a smoking-cessation intervention had fewer respiratory symptoms after 5 years of follow-up. |
| 1775 | Blondal, T.. Correction: Nicotine nasal spray with nicotine patch for smoking cessation: Randomised trial with six year follow up (British Medical Journal (1999) 30 January (285-289)). British Medical Journal. 1999;318:764.  **Abstract:** |
| 1778 | Dey, P, Foy, R, Woodman, M, Fullard, B, Gibbs, A. Should smoking cessation cost a packet? A pilot randomized controlled trial of the cost-effectiveness of distributing nicotine therapy free of charge. The British journal of general practice : the journal of the Royal College of General Practitioners. 1999;49:127.  **Abstract:** This pilot study suggests that changes in prescribing policy for nicotine replacement patches should be made only when evidence of cost-effectiveness can be adduced from a randomized controlled trial. |
| 1779 | Tønnesen, P, Paoletti, P, Gustavsson, G, Russell, M A, Saracci, R, Gulsvik, A, Rijcken, B, Sawe, U. Higher dosage nicotine patches increase one-year smoking cessation rates: results from the European CEASE trial. Collaborative European Anti-Smoking Evaluation. European Respiratory Society. The European respiratory journal. 1999;13:238.  **Abstract:** The Collaborative European Anti-Smoking Evaluation (CEASE) was a European multicentre, randomized, double-blind placebo controlled smoking cessation study. The objectives were to determine whether higher dosage and longer duration of nicotine patch therapy would increase the success rate. Thirty-six chest clinics enrolled a total of 3,575 smokers. Subjects were allocated to one of five treatment arms: placebo and either standard or higher dose nicotine patches (15 mg and 25 mg daily) each given for 8 or 22 weeks with adjunctive moderately intensive support. The 12 month sustained success rates were: 25 mg patch for 22 weeks (L-25), 15.4%; 25 mg patch for 8 weeks (S-25), 15.9%; 15 mg patch for 22 weeks (L-15), 13.7%; 15 mg patch for 8 weeks (S-15), 11.7%; and placebo (P-0) 9.9% (placebo versus 15 mg, p<0.05; 25 mg versus 15 mg, p<0.03; 25 mg versus placebo, p<0.001, Chi-squared test). There was no significant difference in success rate between the two active treatment durations. Of the first week abstainers (n=1,698), 25.1% achieved success at 12 months as opposed to first week smokers, 2.7% of 1,877 subjects (p< 0.001). In summary, a higher than standard dose of nicotine patch was associated with an increase in the long-term success in smoking cessation but continuation of treatment beyond 8-12 weeks did not increase the success rates. |
| 1785 | Davidson, M, Epstein, M, Burt, R, Schaefer, C, Whitworth, G, McDonald, A. Efficacy and safety of an over-the-counter transdermal nicotine patch as an aid for smoking cessation. Archives of family medicine. 1998;7:569.  **Abstract:** OBJECTIVETo evaluate the efficacy and safety of a transdermal nicotine patch as an aid for smoking cessation in an over-the-counter setting.DESIGNMulticenter, double-blind, randomized, placebo-controlled trial of 6-week duration with 18 weeks of follow-up.SETTINGFour shopping mall precincts.PARTICIPANTSThe randomized sample consisted of 802 adults (mean age, 39 years) and was 89% white and 54% female. A smoking history of at least 20 cigarettes per day for 1 year and a score of 5 (on a 10-point scale) on a motivational assessment questionnaire were required for enrollment. Poststudy follow-up was limited to those who had quit smoking at the end of 6 weeks.INTERVENTIONNicotine patches were provided at the shopping mall. Guidance consisted only of package instructions and a smoking cessation self-help booklet.MAIN OUTCOME MEASURESQuit rates were defined as total abstinence from smoking for 4 consecutive weeks (treatment weeks 3-6), point prevalence smoking status at week 6, or nonsmoker at week 6 and week 24 (6-month postquit date). Smoking status was assessed by diaries, and verification for the first 2 quit rates was obtained by confirmation of carbon monoxide of 8 ppm or less in expired breath. Safety was evaluated by self-reported adverse events.RESULTSQuit rate was 12% for the active treatment group and 5.5% for the placebo group, based on total abstinence for 4 consecutive weeks (P = .001) compared with quit rates of 19.5% and 7.5% for active treatment and placebo groups, respectively, based on point prevalence data at week 6. At 24 weeks, 8.2% of nonsmokers in the active treatment group and 4.0% in the placebo group remained nonsmokers. At least 1 adverse event was reported by 57% receiving the nicotine patch and 39% receiving placebo (P<.001).CONCLUSIONSWhen the nicotine patch was used in an over-the-counter setting, quit rates were comparable to those reported for medical settings. A 2:1 quit rate advantage was achieved at week 6 and was maintained at 24 weeks. |
| 1786 | Hurt, R D, Offord, K P, Croghan, I T, Croghan, G A, Gomez-Dahl, L C, Wolter, T D, Dale, L C, Moyer, T P. Temporal effects of nicotine nasal spray and gum on nicotine withdrawal symptoms. Psychopharmacology. 1998;140:98.  **Abstract:** Nicotine nasal spray and nicotine gum have been found to be effective in relieving nicotine withdrawal symptoms. In this randomized single-blind study, 91 cigarette smokers were randomly assigned to a single 1 mg dose of active nicotine nasal spray (n=29), active 4 mg nicotine gum (n=31), saline placebo nasal spray (n=16) or placebo gum (n=15). Following overnight abstinence, subjects repeatedly completed visual analog scales for assessing nicotine withdrawal symptoms over 30 min preceding (time -30 min to time 0) and 120 min following a single dose of study medication. This sequence was performed 3 times during the day. Nicotine withdrawal symptoms were assessed on a 41-point visual analog scale (1=no withdrawal, 41=extreme withdrawal). At the initial session only, blood samples for serum nicotine levels were taken at baseline, then at 5, 10, 30 and 120 min following study drug administration. The mean (+/-SD) age of the subjects was 38.6 (+/-10.1) years, 48% were females, smoking rate was 24.5 (+/-7.8) cigarettes per day, and years of smoking was 19.9 (+/-10.0). A single 1 mg dose of nicotine nasal spray provided more immediate relief for craving for a cigarette compared to a single 4 mg dose of nicotine gum. Serum venous nicotine levels for the active nicotine nasal spray and nicotine gum were comparable at 5 and 10 min while the levels were higher for nicotine gum at 30 and 120 min. Changes in withdrawal symptoms were not found to be related to serum venous nicotine levels. Our findings provide a rationale for the as needed use of nicotine nasal spray to control withdrawal symptoms, possibly in combination with other medications with longer acting effects. |
| 1788 | Ahluwalia, J S, Resnicow, K, Clark, W S. Knowledge about smoking, reasons for smoking, and reasons for wishing to quit in inner-city African Americans. Ethnicity & disease. 1998;8:385.  **Abstract:** OBJECTIVETo determine knowledge about smoking, reasons for smoking, and reasons for wishing to quit and the association of these variables with abstinence at ten weeks and six months.DESIGNDescriptive study and longitudinal intervention.SETTINGInner-city public hospital clinics.PARTICIPANTS410 African-American cigarette smokers interested in quitting were surveyed at baseline and subsequently enrolled into a double-blind, placebo-controlled, randomized trial of the transdermal nicotine patch.MAIN OUTCOME MEASURESDescriptive information about smoking knowledge, reasons for smoking, and reasons for wishing to quit, and association of these variables with abstinence at 10 weeks and 6 months.RESULTSAmong the 410 patients randomized, mean age was 48 years, 61% were female, 41% had less than a high school education, 51% had an annual household income less than ,000, and the average number of cigarettes smoked a day was twenty. The average number of questions answered correctly was nine out of eleven (84%). The most cited reason for smoking was relaxation/tension reduction and the least cited were stimulation and handling of the cigarette. Ninety-nine percent of patients stated they wished to quit for health reasons. Knowledge, reasons for smoking, and reasons for wishing to quit were not significantly associated with 10-week or 6-month abstinence.CONCLUSIONSIn this group of inner-city African-American smokers, knowledge about cigarette smoking was high. Reasons for smoking were related to relaxation, craving, and pleasure, and reasons for wishing to quit were largely health-related. Knowledge, reasons for smoking, and reasons for wishing to quit were not associated with 10 week or 6 month abstinence. Since knowledge about smoking is already high, future efforts should be directed at promoting cessation through proven behavioral and pharmacological approaches, rather than didactic patient education. |
| 1790 | Daughton, D, Susman, J, Sitorius, M, Belenky, S, Millatmal, T, Nowak, R, Patil, K, Rennard, S I. Transdermal nicotine therapy and primary care. Importance of counseling, demographic, and participant selection factors on 1-year quit rates. The Nebraska Primary Practice Smoking Cessation Trial Group. Archives of family medicine. 1998;7:425.  **Abstract:** OBJECTIVETo evaluate the smoking cessation efficacy of nicotine patch therapy as an adjunct to low-intensity, primary care intervention.DESIGNRandomized, placebo-controlled, double-blind, multisite trial.SETTINGSTwenty-one primary care sites in Nebraska.PATIENTSA total of 369 smokers of 20 or more cigarettes per day.INTERVENTIONTwo brief primary care visits for smoking intervention with 10 weeks of active or placebo-patch therapy.MAIN OUTCOME MEASURESConfirmed self-reported abstinence 3, 6, and 12 months after the quit day.RESULTSCompared with placebo control subjects, participants assigned nicotine patches had higher 3-month (23.4% vs 11.4%; P < .01) and 6-month (18.5% vs 10.3%; P < .05) abstinence rates. The 1-year abstinence rates for the active and placebo patch groups were 14.7% and 8.7%, respectively (P = .07). Of smokers aged 45 years and older, 9 (18.8%) of 48 using active patches compared with 0 of 31 using placebo patches achieved 12-month abstinence (chi 2 = 6.56; P < .05). Among those with high nicotine dependency scores (Fagerstrom score > or = 7), 1-year abstinence rates were significantly higher in the nicotine patch group (19.1%) compared with the placebo group (5.0%) (chi 2 = 10.7; P = .001). However, there was no significant difference in 1-year quit rates for participants with low Fagerstrom scores (< 7).CONCLUSIONSNicotine patch therapy enhanced 6 month quit rates as an adjunct to brief primary care intervention. The highest quit rates were achieved by participants who specifically contacted the site to enroll in the study or to obtain a prescription for nicotine patches. Differences in participant selection factors may account, in part, for the lower smoking cessation rates associated with primary care intervention. Duration of counseling, patient age, and Fagerstrom scores may be important factors related to the long-term smoking cessation success of nicotine patch therapy. |
| 1791 | van Dijk, A P, Meijssen, M A, Brouwer, A J, Hop, W C, van Bergeijk, J D, Feyerabend, C, Wilson, J H, Zijlstra, F J. Transdermal nicotine inhibits interleukin 2 synthesis by mononuclear cells derived from healthy volunteers. European journal of clinical investigation. 1998;28:664.  **Abstract:** BACKGROUNDSmoking has either a beneficial or harmful effect on the course and recurrence of ulcerative colitis (UC) and Crohn's disease respectively. Transdermal application of nicotine had similar effects in UC and therefore was considered to be an effective basic drug that could be further developed in the search for new compounds in the treatment of acute exacerbations of corticosteroid-resistant UC. To clarify the hypothesis that nicotine exerts its anti-inflammatory effect in UC through selective inhibition of T-cell-derived cytokine synthesis, we studied in vivo effects of nicotine on cytokine production by human non-adherent mononuclear cells isolated from peripheral blood in a randomized, double-blind, placebo-controlled trial.METHODSHealthy non-smoking volunteers applied for 2 weeks of nicotine patches (n = 12) with incremental doses of nicotine during the first week to achieve a maintenance dose of 15 mg per day, or placebo (n = 12). Blood was obtained before treatment and 1, 2, 3 and 6 weeks after the start of treatment. Cells were cultured in the absence or presence of phytohaemagglutinin for 48 h, and total amounts of interleukin 2 (IL-2), IL-4, IL-10, IL-13, interferon gamma (IFN-gamma) and tumour necrosis factor alpha (TNF-alpha) were measured.RESULTSTransdermal nicotine caused a significant inhibition of IL-2 after 2 weeks' treatment compared with the placebo group. In addition, a diminished production of IL-10 and TNF-alpha in comparison with day 0 was observed.CONCLUSIONThe beneficial effect of transdermal nicotine in ulcerative colitis may be mediated by a selective inhibition of the IL-2 production by mucosal mononuclear cells, which could result in diminished cell proliferation and consequently a reduction in the inflammatory process. |
| 1792 | Rose, J E, Behm, F M, Westman, E C. Nicotine-mecamylamine treatment for smoking cessation: the role of pre-cessation therapy. Experimental and clinical psychopharmacology. 1998;6:331.  **Abstract:** The nicotinic antagonist mecamylamine was evaluated in a randomized smoking cessation trial. Four groups of participants (n = 20 per group) received nicotine plus mecamylamine, nicotine alone, mecamylamine alone, or no drug for 4 weeks before cessation. After the quit-smoking date, all subjects received nicotine plus mecamylamine treatment for 6 weeks. Nicotine skin patches (21 mg/24 hr) and mecamylamine capsules (2.5-5.0 mg twice per day) were used. Precessation mecamylamine significantly prolonged the duration of continuous smoking abstinence; abstinence rates at the end of treatment were 47.5% with mecamylamine and 27.5% without mecamylamine. Nicotine + mecamylamine reduced ad lib smoking, smoking satisfaction, and craving more than either drug alone. Moreover, the orthostatic decrease in blood pressure caused by mecamylamine was offset by nicotine. Mecamylamine before smoking cessation may be an effective adjunct to nicotine patch therapy. |
| 1793 | Perng, R P, Hsieh, W C, Chen, Y M, Lu, C C, Chiang, S J. Randomized, double-blind, placebo-controlled study of transdermal nicotine patch for smoking cessation. Journal of the Formosan Medical Association = Taiwan yi zhi. 1998;97:547.  **Abstract:** Smoking cessation is an arduous process because nicotine withdrawal syndrome, which occurs following sudden interruption of nicotine use in long-term smokers, frequently prevents them from giving up the habit. Nicotine supplement systems may relieve smokers' nicotine withdrawal symptoms and, thus, help in the process of abstinence from smoking. The purpose of the present study was to investigate the effectiveness and safety of a 30-mg transdermal nicotine patch in a smoking cessation program for Chinese smokers. In this randomized, double-blind, placebo-controlled study, 30 heavy smokers, who had smoked more than 20 cigarettes per day for more than a year, were treated with 30-mg transdermal nicotine patches, and 32 heavy smokers were given placebo patches during a 6-week smoking cessation program. The clinical characteristics of the two groups were similar. After 6 weeks, the use of the transdermal nicotine patch was associated with markedly reduced nicotine dependence and severity of withdrawal symptoms. Nineteen (63%, 95% confidence interval, CI, 46%-80%) of the smokers treated with the transdermal nicotine patch had successfully quit smoking at the end of the program (6 weeks) and nine (30%, 95% CI 14%-46%) remained abstinent 1 year later. In contrast, only 11 (34%, 95% CI 18%-50%) of the smokers in the placebo group had successfully stopped smoking after 6 weeks, and three remained abstinent 1 year later (9%, 95% CI 0%-19%). However, there was no statistically significant difference between the two groups of smokers after 1 year of follow-up (p = 0.08). Side-effects were minimal and did not affect the efficacy of the skin patch. The results indicate that the transdermal nicotine patch is an effective aid in smoking cessation programs. |
| 1794 | Tzivoni, D, Keren, A, Meyler, S, Khoury, Z, Lerer, T, Brunel, P. Cardiovascular safety of transdermal nicotine patches in patients with coronary artery disease who try to quit smoking. Cardiovascular drugs and therapy. 1998;12:239.  **Abstract:** Nicotine patches are commonly used by people who try to quit smoking. Because high doses of nicotine may increase heart rate and potentiate cardiac arrhythmia or ischemia, its use in patients with coronary artery disease was investigated. The objective was to assess the cardiovascular safety of nicotine patches in patients with coronary artery disease (CAD) who try to quit smoking. The study was conducted in a double-blind, placebo-controlled, randomized fashion over a 2-week period. One hundred and six patients with CAD who wished to stop smoking and were taking part in a smoking cessation program were included. Fifty-two patients received nicotine patches (Nicotinell) and 54 received placebo patches. The cardiovascular effects of nicotine patches were assessed by repeated ambulatory ECG monitoring (AEM) and exercise testing. There were no changes in the resting heart rate and in the systolic or diastolic blood pressure between the screening and the two phases of the study in both the Nicotinell and placebo groups. Repeated 48-hour AEM revealed that there were no significant changes in the number and duration of ischemic episodes in both groups. There was no change in the frequency of atrial or ventricular arrhythmias. Exercise duration and time to 1-mm ST-segment depression increased in both groups during the double-blind treatment phase. More patients in the Nicotinell group claimed tobacco abstinence compared with the placebo group (27% vs. 13%). The use of nicotine patches did not cause aggravation of myocardial ischemia or arrhythmia in coronary patients and therefore can be used as a method to promote smoking cessation in this high-risk group. |
| 1796 | Lawson, G M, Hurt, R D, Dale, L C, Offord, K P, Croghan, I T, Schroeder, D R, Jiang, N S. Application of serum nicotine and plasma cotinine concentrations to assessment of nicotine replacement in light, moderate, and heavy smokers undergoing transdermal therapy. Journal of clinical pharmacology. 1998;38:502.  **Abstract:** As part of a clinical trial investigating the level of nicotine replacement with different doses of transdermal therapy for smoking cessation, peak and trough serum nicotine and plasma cotinine concentrations were measured in 70 subjects while they were actively smoking (baseline) and daily for 6 consecutive inpatient days while they were receiving transdermal nicotine. Subjects were randomly assigned to a daily 24-hour patch delivering a transdermal nicotine dose of 0, 11, 22, or 44 mg and stratified by self-reported smoking rate as either light (10-15 cigarettes per day), moderate (16-30 cigarettes per day), or heavy (>30 cigarettes per day). Steady-state concentrations of nicotine and cotinine were attained in 1 and 3 days, respectively, at all doses and were independent of baseline smoking rate. Mean percentage replacement of nicotine was calculated by dividing steady-state peak nicotine or cotinine concentrations by their respective baseline concentrations. Significant underreplacement occurred in subjects receiving the 11 mg/day patch regardless of baseline smoking rate. Underreplacement also occurred in moderate and heavy smokers receiving 22 mg/day and in light smokers at this same dose. Complete replacement occurred only in subjects receiving the 44 mg/day patch. These results have several implications for transdermal nicotine therapy. First, with the higher nicotine and cotinine levels observed with heavier smoking, it is inherent that one size does not fit all, and there is a need to consider more individualization of dosage for nicotine patch therapy. Second, there is substantial underreplacement with the 22 mg/day dose in moderate to heavy smokers and in some light smokers. Third, even with twice the usual dose (i.e., 44 mg/day), there was no accumulation of either nicotine or cotinine. Plasma cotinine levels after achievement of steady state (i.e., after 3 days of patch therapy) can be collected at any time and used to calculate percent replacement using baseline levels. |
| 1797 | Lawson, G M, Hurt, R D, Dale, L C, Offord, K P, Croghan, I T, Schroeder, D R, Jiang, N S. Application of urine nicotine and cotinine excretion rates to assessment of nicotine replacement in light, moderate, and heavy smokers undergoing transdermal therapy. Journal of clinical pharmacology. 1998;38:510.  **Abstract:** As part of a clinical trial investigating the level of nicotine replacement with different doses of transdermal therapy for smoking cessation, urine excretion rates of nicotine and cotinine were measured in 70 subjects while they were actively smoking (baseline) and for 6 consecutive inpatient days while they were receiving transdermal nicotine therapy. Subjects were stratified according to baseline smoking rate as light (10-15 cigarettes per day), moderate (16-30 cigarettes per day), or heavy (>30 cigarettes per day) smokers and randomly assigned to a daily 24-hour patch delivering a transdermal nicotine dose of 0, 11, 22, or 44 mg. Steady-state excretion rates of nicotine and cotinine were attained in 2 and 3 days, respectively, at all doses and were independent of smoking rate. Percentage replacement of nicotine was calculated by dividing steady-state nicotine or cotinine excretion rates by their respective baseline excretion rates. Significant underreplacement occurred with the 11-mg/day dose, particularly in moderate and heavy smokers (<50%). At a dose of 22 mg/day, nicotine replacement was still <100% in the majority of subjects. Only at a dose of 44 mg/day did mean replacement exceed 100% regardless of baseline smoking rate. |
| 1799 | Senore, C, Battista, R N, Shapiro, S H, Segnan, N, Ponti, A, Rosso, S, Aimar, D. Predictors of smoking cessation following physicians' counseling. Preventive medicine. 1998;27:412.  **Abstract:** BACKGROUNDThe purpose of this study was, to identify predictors of quitting following general practitioners' (GP) anti-smoking counseling.METHODSWe studied determinants (characterized following the Precede framework) of successful quitting (1 year sustained abstinence, biochemically confirmed at 6- and 12-month follow-up) among 861 smokers randomized to the intervention groups based on repeated counseling (RC), RC + spirometric testing, and RC + nicotine gum, in a smoking cessation trial carried out in Turin, Italy.RESULTSGPs' intervention worked best for male (OR = 2.30; 95% CI, 1.13-4.52) and married (OR = 3.63; 95% CI, 1.37-9.59) smokers, for smokers who had maintained abstinence for at least 1 month in the past (OR = 6.78; 95% CI, 1.56-29.52) or at their first quit attempt (OR = 10.91; 95% CI, 2.37-50.13), and for those who spontaneously reduced their coffee consumption (OR = 3.30; 95% CI, 1.59-6.82); heavy smokers (> = 20 cig/day OR = 0.48; 95% CI, 0.24-0.93) and those living with other smokers (> = 1 smokers in the household: OR = 0.44; 95% CI, 0.22-0.90) were less likely to give up. Previous antismoking advice by the GP represented a strong barrier to success for healthy smokers (OR = 0.19; 95% CI, 0.07-0.52), but not for those reporting symptoms of shortness of breath (OR = 0.63; 95% CI, 0.39-9.20). There were no interactions between predictors and treatment conditions.CONCLUSIONSAssessment of factors influencing quitting would allow GPs to tailor their message to address existing barriers and to help patients utilize their resources for change. |
| 1801 | Stapleton, J A, Sutherland, G, Russell, M A. How much does relapse after one year erode effectiveness of smoking cessation treatments? Long-term follow up of randomised trial of nicotine nasal spray. BMJ (Clinical research ed.). 1998;316:830.  **Abstract:** |
| 1803 | Lewis, S F, Piasecki, T M, Fiore, M C, Anderson, J E, Baker, T B. Transdermal nicotine replacement for hospitalized patients: a randomized clinical trial. Preventive medicine. 1998;27:296.  **Abstract:** BACKGROUNDThis study was undertaken to assess the safety and efficacy of a treatment involving brief counseling and the nicotine patch among hospital inpatients and to identify variables associated with long-term smoking cessation following hospitalization.METHODSOne hundred eighty-five patients were randomly assigned to one of three smoking cessation interventions: (1) A Minimal Care (MC) condition, consisting of a brief physician-delivered motivational message to stop smoking, (2) a Counseling + Active Nicotine Patch (CAP) condition in which patients received the motivational message, a 6-week supply of nicotine patches, and extended bedside and telephone counseling, and (3) a Counseling + Placebo Patch (CPP) condition identical to the CAP condition except the supplied patches contained no nicotine.RESULTSAt 6-month follow-up, abstinence rates for the three treatments were 4.9, 6.5, and 9.7% for the MC, CPP, and CAP treatments, respectively. These differences were not statistically significant. Patients admitted for respiratory disease were more likely to quit than patients with any other diagnosis. The nicotine patch was well tolerated by hospital inpatients.CONCLUSIONSThe initiation of nicotine patch therapy during hospitalization appears to be safe when used among patients carrying a wide range of diagnoses. Our study provided no evidence of the superiority of nicotine patches versus placebo, but this does not preclude the possibility that future research using larger samples might detect differences between patch groups. Hospital interventions for smoking cessation may be most effective among patients hospitalized for a smoking-related illness such as respiratory disease. |
| 1804 | West, R, Willis, N. Double-blind placebo controlled trial of dextrose tablets and nicotine patch in smoking cessation. Psychopharmacology. 1998;136:201.  **Abstract:** In a placebo-controlled double-blind trial 308 smokers were individually randomly allocated to one of four groups: 1) 3 g dextrose tablets and 15 mg nicotine transdermal patch; 2) dextrose and placebo patch; 3) placebo tablets and nicotine patch; 4) placebo tablets and placebo patch. Patients were scheduled to attend weekly smokers clinic sessions starting 1 week before the quit date and continue for 4 weeks after that date. The primary outcome variable was biochemically verified abstinence at the final session, four weeks after the scheduled quit date. The proportion of smokers abstinent in the four groups was as follows: 49% - dextrose plus active patch; 44% - dextrose plus placebo patch; 36% - placebo tablet plus active patch; 30% - placebo tablet plus placebo patch. The difference between the dextrose and placebo tablets (13%) was statistically significant (P < 0.01, one-tailed); the difference between the active and placebo patches (6%) was not. There was no significant difference between the effect of the dextrose when accompanied by active versus placebo patches. There was no significant effect of dextrose on weight. The results suggest that dextrose supplementation to the diet may be a cheap and simple aid to giving up smoking. Further research is now needed to establish its long-term efficacy. |
| 1806 | Ahluwalia, J S, McNagny, S E, Clark, W S. Smoking cessation among inner-city African Americans using the nicotine transdermal patch. Journal of general internal medicine. 1998;13:1.  **Abstract:** OBJECTIVETo determine the efficacy of the transdermal nicotine patch for smoking cessation in inner-city African Americans.DESIGNDouble-blind, placebo-controlled, randomized trial.SETTINGOutpatient in an inner-city hospital.PATIENTS AND PARTICIPANTSA computer-generated random numbers table with a block size set at 20 was used to randomize 410 patients to one of two study arms.INTERVENTIONSThe transdermal nicotine patch for 10 weeks as an adjunct to brief counseling.MEASUREMENTS AND MAIN RESULTSOf the 410 patients randomized, mean age was 48 years, 65% were female, 41% had less than a high school education, 51% had an annual household income of less than ,000, and the average number of cigarettes smoked per day was 20. Quit rates at 10 weeks were 21.5% (44/205) with the nicotine patch, and 13.7% (28/205) with the placebo patch (p = .03). At 6 months, quit rates were 17.1% (35/205) with the nicotine patch, and 11.7% (24/205) with the placebo patch (p = .08). After adjusting for baseline differences in age and educational attainment, differences remained significant at 10 weeks (p = .04), but were not significant at 6 months (p = .14). Compliance rates for return visits were 83%, 78%, 55%, and 52%, at 1, 2, 6, and 10 weeks, respectively.CONCLUSIONSThe nicotine patch significantly improves short-term quit rates in inner-city African Americans who are interested in trying to quit smoking. Efforts should be made to reach underserved populations through smoking cessation programs, and to assist in maintaining abstinence. |
| 1807 | Dale, L C, Schroeder, D R, Wolter, T D, Croghan, I T, Hurt, R D, Offord, K P. Weight change after smoking cessation using variable doses of transdermal nicotine replacement. Journal of general internal medicine. 1998;13:9.  **Abstract:** OBJECTIVEExamine weight change in subjects receiving variable doses of transdermal nicotine replacement for smoking cessation.DESIGNRandomized, double-blind clinical trial.SETTINGOne-week inpatient treatment with outpatient follow-up through 1 year.INTERVENTIONThis report examines weight change after smoking cessation for 70 subjects randomized to placebo or to 11, 22, or 44 mg/d doses of transdermal nicotine. The study included 1 week of intensive inpatient treatment for nicotine dependence with active patch therapy continuing for another 7 weeks. Counseling sessions were provided weekly for the 8 weeks of patch therapy and with long-term follow-up visits at 3, 6, 9, and 12 months.MEASUREMENTS AND MAIN RESULTSForty-two subjects were confirmed biochemically (i.e., by expired carbon monoxide) to be nonsmokers at all weekly visits during patch therapy. Their 8-week weight change from baseline was 3.0 +/- 2.0 kg. For these subjects, 8-week weight change was found to be negatively correlated with percentage of cotinine replacement (r = -.38, p = .012) and positively correlated with baseline weight (r = .48, p = .001), and age (r = .35, p = .025). Men had higher (p = .003) 8-week weight gain (4.0 +/- 1.8 kg) than women (2.1 +/- 1.7 kg). Of the 21 subjects who abstained continuously for the entire year, 20 had their weight measured at 1-year follow-up. Among these 20 subjects, 1-year weight change was not found to be associated with gender, baseline weight, baseline smoking rate, total dose of transdermal nicotine, or average percentage of cotinine replacement during the 8 weeks of patch therapy.CONCLUSIONSThis study suggests that higher replacement levels of nicotine may delay postcessation weight gain. This effect is consistent for both men and women. We could not identify any factors that predict weight change with long-term abstinence from smoking. |
| 1808 | Hatsukami, D, Pentel, P R, Jensen, J, Nelson, D, Allen, S S, Goldman, A, Rafael, D. Cotinine: effects with and without nicotine. Psychopharmacology. 1998;135:141.  **Abstract:** The purpose of this study was to examine the effects of the metabolite of nicotine, cotinine, in comparison to the effects of the nicotine patch, and a combination thereof during cigarette abstinence. More specifically, this study examined the effects of cotinine on physiological measures, subjective measures assessing craving, withdrawal symptoms and mood, and performance measures. A between-subject, 2 x 2 factorial design was used, with the daily administration of a 15-mg nicotine patch (Nicotrol) versus placebo patch as one factor and 80 mg of oral cotinine fumarate versus placebo drug as the other factor. Baseline measures were obtained while the subjects smoked cigarettes on an ad lib basis for 1 week. Subjects (n = 106) were then randomly assigned to one of four treatment conditions and for the next 14 days were required to be abstinent from cigarettes and take the study drugs. Cotinine administration, with or without nicotine patch, produced serum cotinine concentrations 3 4 times higher than during ad lib smoking. Results showed a reduction of self-reported tobacco withdrawal symptoms using the nicotine patch alone. Cotinine alone had no effect on withdrawal symptoms. However, when nicotine patch was combined with cotinine, the beneficial effect of the nicotine patch on withdrawal symptoms was absent. Therefore, cotinine appears to antagonize the effects of nicotine in the alleviation of withdrawal symptoms at concentrations higher than that attained from normal smoking. This effect does not appear to be mediated by changes in nicotine disposition. |
| 1809 | Richmond, R L, Kehoe, L, de Almeida Neto, A C. Three year continuous abstinence in a smoking cessation study using the nicotine transdermal patch. Heart (British Cardiac Society). 1997;78:617.  **Abstract:** A total of 305 subjects from Sydney were randomly allocated to receive either an active (24 hour transdermal nicotine patch over a 10 week course) or placebo nicotine patch. All subjects participated in a multicomponent cognitive-behavioural smoking cessation programme over five weeks in two-hour group sessions. The continuous abstinence rates at three years (validated by expired carbon monoxide) were 13.8% for the active group and 5.2% for placebo group (p = 0.011). The active nicotine patch with behavioural therapy achieved more than double the abstinence rates early in treatment compared with placebo and this difference was maintained throughout the three year follow up. |
| 1811 | Ginsberg, J P, Klesges, R C, Johnson, K C, Eck, L H, Meyers, A W, Winders, S A. The relationship between a history of depression and adherence to a multicomponent smoking-cessation program. Addictive behaviors. 1997;22:783.  **Abstract:** The purpose of this study was to determine whether a history of depression in female smokers (age 18-65) who did not self-report any current depression was associated with adherence to a multisession, multicomponent smoking-cessation program. Participants in a 13-week cognitive-behavioral group program plus random assignment to nicotine gum, appetite suppressant gum, or placebo chewing gum were grouped by depressive-history and compared on attendance, average expired carbon monoxide after planned cessation, and number of pieces of gum chewed. No significant differences between the depressive history (yes/no) subgroups were found on any of the three measures of adherence. The power to detect a significant difference (alpha = 0.05) was calculated to be 0.89. Group cognitive-behavioral treatment appears to be the basis of an effective smoking-cessation program for women with a history of depression who are not currently depressed. |
| 1814 | Hjalmarson, A, Nilsson, F, Sjöström, L, Wiklund, O. The nicotine inhaler in smoking cessation. Archives of internal medicine. 1997;157:1721.  **Abstract:** BACKGROUNDNicotine replacement therapy has been shown to improve success rates in smoking cessation treatment. However, the available products cause adverse effects, which prevent some smokers from using them. A new method of delivering nicotine via inhaler supplies nicotine orally through inhalation from a plastic tube. This mode of delivering nicotine resembles smoking, as it includes handling and active inhalation.OBJECTIVESTo assess the efficacy and safety of the nicotine inhaler as an aid in smoking cessation.METHODSA 1-year, randomized, double-blind, placebo-controlled study was conducted in a smoking cessation clinic. Two hundred forty-seven smokers who smoked at least 10 cigarettes per day and who had previously made a serious attempt to stop smoking using nicotine chewing gum were recruited through advertisements. Randomization to treatment or control conditions were made at the first group session, with 123 participants receiving nicotine inhalers and 124 receiving placebo inhalers. The inhalers were distributed at the second session and participants were allowed to use the inhalers for 6 months.MAIN OUTCOME MEASUREBiochemically verified continuous abstinence from smoking after 2 and 6 weeks and at 3, 6, and 12 months.RESULTSSignificantly more participants who had used the nicotine inhalers were continuously abstinent compared with those who had used the placebo inhalers. The respective success rates after 12 months were 28% and 18% (P = .046). At 6 months, 20 participants (16%) in the nicotine group were still using the inhaler, compared with 4 (3%) in the control group (P < .001).CONCLUSIONThe nicotine inhaler was an effective smoking cessation aid that produced a few mild and transient adverse effects. |
| 1815 | Killen, J D, Fortmann, S P, Davis, L, Varady, A. Nicotine patch and self-help video for cigarette smoking cessation. Journal of consulting and clinical psychology. 1997;65:663.  **Abstract:** A total of 424 smokers were randomized in a 2 x 2 factorial experiment. A pharmacologic factor contained 2 levels: transdermal nicotine path (TNP; 21 mg) and placebo. A self-help behavioral treatment factor contained 2 levels: video-enhanced self-help treatment manual and self-help treatment manual only. At 2 months, TNP produced a higher level of abstinence (36%) than placebo (20%), p < .001. No other comparison was significant. In secondary analyses, (at 2 months) and compliance with patch treatment regimen (at 2, 6, and 12 months) were associated with less relapse. Although nicotine replacement therapy has improved our ability to produce smoking cessation, the production of sustained, longer term abstinence remains an elusive goal. |
| 1816 | Blöndal, T, Franzon, M, Westin, A. A double-blind randomized trial of nicotine nasal spray as an aid in smoking cessation. The European respiratory journal. 1997;10:1585.  **Abstract:** The objective of the study was to evaluate the therapeutic efficacy of nicotine nasal solution (NNS) for smoking cessation from the stopping day up to 3 months. We also followed the participants for 2 yrs after ceasing smoking to assess what happens after stopping using NNS. In a placebo-controlled, double-blind, 2 yr prospective study, 157 smokers were given either NNS, one dose containing 1 mg of nicotine per 100 microL (n=79), or placebo (n=78). Treatment was continued for up to 1 yr. One day after quitting smoking, the average number of daily doses was 11 in the group assigned NNS and 14 in the group assigned the placebo, and after 6 weeks, 14 and 6 doses, respectively, among abstinent participants still using spray. After 3 months, 65% of the abstainers in the nicotine group were still using the NNS. The abstinence rates were 51, 39 and 29% after 6 weeks, 3 and 6 months, respectively, as compared to 24, 19 and 18% in the placebo group (p=0.0003; p=0.003; p=0.050). The proportion abstinent at the 1 yr (25 vs 17%) and 2 yr follow-ups (19 vs 14%) was higher among those assigned to the nicotine than to the placebo group, but not significantly so for the numbers used in the study. In conclusion, the use of nicotine nasal spray significantly increased the abstinence rate during the first 6 months following the quitting day. |
| 1817 | Simon, J A, Solkowitz, S N, Carmody, T P, Browner, W S. Smoking cessation after surgery. A randomized trial. Archives of internal medicine. 1997;157:1371.  **Abstract:** BACKGROUNDCigarette smoking is the greatest cause of preventable mortality in the United States. Because most smokers would like to quit and most hospitals are smoke free, surgical admissions represent a window of opportunity for tobacco cessation interventions.METHODSA total of 324 patients (98% men), aged 25 to 82 years, who were current smokers and who underwent noncardiac surgery were enrolled in a randomized controlled trial at the Veterans Affairs Medical Center, San Francisco, Calif. One hundred sixty-eight participants (52%) received a multicomponent intervention designed to increase self-efficacy and coping skills that included face-to-face in-hospital counseling, viewing a smoking cessation videotape, self-help literature, nicotine replacement therapy, and 3 months of telephone follow-up. One hundred fifty-six participants (48%) received self-help literature and brief counseling lasting 10 minutes. Serum or saliva cotinine levels were measured to confirm self-reported smoking cessation.RESULTSAt 12 months of follow-up, the self-reported quit rate was 27% among the intervention group and 13% among the comparison group (relative risk, 2.1; 95% confidence interval, 1.2-3.5; P < .01). Based on biochemical confirmation, 15% of the intervention group, compared with 8% of the comparison group, quit smoking at 12 months (relative risk, 2.0; 95% confidence interval, 1.0-3.9; P = .04).CONCLUSIONSA smoking cessation intervention targeted at smokers hospitalized for noncardiac surgery can increase long-term quit rates. Surgical hospitalizations provide an opportunity to reach smokers who want to quit smoking. |
| 1820 | Sandborn, W J, Tremaine, W J, Offord, K P, Lawson, G M, Petersen, B T, Batts, K P, Croghan, I T, Dale, L C, Schroeder, D R, Hurt, R D. Transdermal nicotine for mildly to moderately active ulcerative colitis. A randomized, double-blind, placebo-controlled trial. Annals of internal medicine. 1997;126:364.  **Abstract:** BACKGROUNDUlcerative colitis is predominantly a disease of nonsmokers. Transdermal nicotine may help control clinical manifestations of this condition.OBJECTIVETo determine the efficacy of transdermal nicotine for controlling clinical disease activity in active ulcerative colitis.DESIGNRandomized, double-blind, placebo-controlled, single-center clinical trial.SETTINGMultispecialty group serving as an academic tertiary referral center.PATIENTS64 nonsmoking patients with mildly to moderately active ulcerative colitis despite the use of medication.INTERVENTIONPatients were stratified on the basis of smoking history, extent of disease, and concomitant medical therapy. After stratification, patients were randomly assigned to daily treatment with transdermal nicotine (n = 31) at the highest tolerated dose (11 mg for 1 week and then < or = 22 mg for 3 weeks) or placebo (n = 33).MEASUREMENTSClinical features were assessed at baseline and 4 weeks by endoscopy, physician assessment, and a patient diary of daily symptoms. Serum concentrations of nicotine were determined by using gas chromatography and mass spectrometry, and plasma concentrations of cotinine were measured by using high-performance liquid chromatography.RESULTSAt 4 weeks, 12 of 31 patients (39%) who received nicotine showed clinical improvement compared with 3 of 33 patients (9%) who received placebo (P = 0.007). Four patients receiving nicotine discontinued therapy because of side effects (contact dermatitis [n = 2], nausea [n = 1], and acute pancreatitis [n = 1]). At week 4, the nicotine group had a mean (+/-SD) trough serum nicotine concentration of 11.3 +/- 8.4 ng/mL and a mean trough plasma cotinine concentration of 192 +/- 95 ng/mL.CONCLUSIONSTransdermal nicotine administered at the highest tolerated dosage (< or = 22 mg/d) for 4 weeks is efficacious for controlling clinical manifestations of mildly to moderately active ulcerative colitis. |
| 1823 | Sønderskov, J, Olsen, J, Sabroe, S, Meillier, L, Overvad, K. Nicotine patches in smoking cessation: a randomized trial among over-the-counter customers in Denmark. American journal of epidemiology. 1997;145:309.  **Abstract:** The authors examined the effect of 24-hour nicotine patches in smoking cessation among over-the-counter customers in Denmark, based on a randomized double-blind placebo-controlled trial. Participants were consecutive customers to whom nicotine patches were offered as the only treatment. Forty-two pharmacies in the areas of Aarhus and Copenhagen in Denmark participated in the trial, and 522 customers who smoked 10 or more cigarettes per day were randomized to either nicotine patches or placebo from January to March 1994. Customers with chronic diseases and pregnant or breastfeeding women were excluded from the trial. Twenty-four-hour patches were offered free of charge during a 3-month period. Those smoking 20 or more cigarettes per day started on a dose of 21-mg/day patches. Customers who smoked less started on patches of 14 mg/day; and for all of the participants, the dose was gradually reduced to 7-mg/day patches during the study period. Smoking behavior and compliance were recorded by means of self-administered questionnaires and telephone interviews. Smoking status was recorded in intervals of 4 weeks, which was fixed to be a treatment period, and 26 weeks after inclusion. There was a significant increase in smoking cessation rates after 8 weeks of follow-up but only among smokers who started on 21-mg/day patches. There was a marked placebo effect at each time of contact during the trial, especially in those smoking fewer than 20 cigarettes per day. Although the noncompliance rate was high overall due to discontinuation in the use of patches by relapsed smokers, noncompliance among successful quitters was low. More side effects were seen in the nicotine group than in the placebo group, but none of the reported side effects were serious. It appears that regular healthy smokers who were customers of nonprescribed nicotine patches and who received 21-mg/day nicotine patches benefited from the active treatment (44.1% stopped smoking after 4 weeks), but almost as many stopped smoking in the placebo group (37.3% after 4 weeks). No significant differences in smoking cessation rates were seen among smokers who started with the low-dose nicotine or placebo patches. |
| 1825 | Martin, J E, Calfas, K J, Patten, C A, Polarek, M, Hofstetter, C R, Noto, J, Beach, D. Prospective evaluation of three smoking interventions in 205 recovering alcoholics: one-year results of Project SCRAP-Tobacco. Journal of consulting and clinical psychology. 1997;65:190.  **Abstract:** A total of 205 (113 male, 92 female) nonhospitalized recovering alcoholics with > 3 months of continuous abstinence from alcohol and drugs and relatively heavy tobacco dependence (Fagerstrom Tolerance Questionnaire score = 7.7; mean number of cigarettes per day, 26.8; mean number of years smoked, 24.4) were randomized to standard treatment (ST) American Lung Association quit program plus nicotine anonymous meetings (n = 70), behavioral counseling plus physical exercise (BEX; n = 72), or behavioral counseling plus nicotine gum (BNIC; n = 63). A 3 x 4 repeated measures design was used to evaluate the effectiveness of the interventions on smoking outcome at baseline, posttreatment, and 6- and 12-month follow-ups. Self-reported smoking status was verified with biochemical and informant report. Verified self-report indicated that significantly more smokers in BEX quit by posttreatment (60%) than in either BNIC (52%) or ST (31%), chi 2 (2, N = 205) = 17.85, p < .01, but not at the 6-month (29%, 27%, and 21%, respectively) or 12-month (27%, 27%, and 26%, respectively) follow-up. Only 4% (7 of 188) relapsed to alcohol or drugs. Alcohol relapse did not differ by treatment group or smoking status. Length of alcohol abstinence was not associated with smoking cessation outcome. |
| 1826 | Mattsson, U, Krogstad, A L, Pegenius, G, Elam, M, Jontell, M. Assessment of erythema and skin perfusion by digital image analysis and scanner laser Doppler. Skin research and technology : official journal of International Society for Bioengineering and the Skin (ISBS) [and] International Society for Digital Imaging of Skin (ISDIS) [and] International Society for Skin Imaging (ISSI). 1997;3:53.  **Abstract:** BACKGROUND/AIMSIn the present study, a model for measuring the intensity of skin erythema by computer-assisted digital image analysis (DIA) was elaborated. The DIA technique was compared with laser Doppler perfusion imaging (PIM) to evaluate the relationship between erythema and skin perfusion.METHODSTo produce erythema, three different types of nicotine patches (Nicotinell®, Nicorette® and Nicorette® placebo) were randomly applied ventrally on the upper arms for the time period recommended by the manufactures. After removal of the patches, colour photographs for subsequent DIA were taken and laser Doppler scanning for PIM was performed. The relationship between PIM and DIA was estimated intra- and inter-individually.RESULTS/CONCLUSIONSA statistically significant linear correlation between PIM and DIA data was found, but the relationship was relatively weak. This may be explained by the fact that DIA and PIM are influenced by different physiological factors such as skin chromophores and velocity of moving cells. The results strengthen the theory that erythema and perfusion measurements reflect separate physiological phenomena and that the methods should be considered complements to each other in the experimental situation. |
| 1827 | Pulerà, N, Petruzzelli, S, Celi, A, Puntoni, R, Fornai, E, Säwe, U, Paoletti, P, Giuntini, C. Presence and persistence of serum anti-benzoapyrene diolepoxide-DNA adduct antibodies in smokers: effects of smoking reduction and cessation. International journal of cancer. 1997;70:145.  **Abstract:** Among biomarkers of tobacco smoke (TS)-induced genotoxic damage, benzo[a]pyrene diolepoxide-DNA adducts (BPDE-DNA) are extensively studied. Adducted DNA becomes antigenic and antibodies anti-BPDE-DNA (BPDE-DNA-Abs) may be found in serum of exposed subjects. Little is known about the persistence of BPDE-DNA, and no study has been performed to evaluate the persistence of BPDE-DNA-Abs after cessation of exposure. Fifty heavy smokers, enrolled in a smoking cessation program with nicotine patch substitution therapy, were evaluated for the presence of BPDE-DNA-Abs before (w0) and 1, 3, 6 and 12 weeks (w1-12) after the start of the program. Nicotine or placebo patches were randomly assigned to the subjects. BPDE-DNA-Abs were determined in serum by non-competitive ELISA. After the start of the cessation program, 28 subjects quit smoking (group Q) and the other 22 reduced by about 75% the number of cigarettes smoked per day (group R). At the start of the program (w0) 8% of subjects were positive. At w1 the prevalence of positivity had increased both in subjects who quit smoking (Q: 21%) and in subjects who had reduced the number of cigarettes per day (R: 27%). Positivity remained stable up to w12 (21%) for group Q, whereas it increased to 41% in group R. Serum BPDE-DNA-Abs can be detected in smokers, and their persistence for months after smoking cessation suggests their usefulness for relatively long-term surveys. The low percentage of positivity in actual heavy smokers and the increase in antibody positivity with smoking cessation or reduction must be taken into account when interpreting serum BPDE-DNA-Ab measurement in exposed individuals. |
| 1828 | Richmond, R L, Kehoe, L, de Almeida Neto, A C. Effectiveness of a 24-hour transdermal nicotine patch in conjunction with a cognitive behavioural programme: one year outcome. Addiction (Abingdon, England). 1997;92:27.  **Abstract:** Hospital-based outpatients participating in a cognitive-behavioural group programme (2 hours/week over 5 weeks) were studied to evaluate the effectiveness of a 24-hour transdermal nicotine patch in assisting smokers to stop smoking when used as an adjunct to a cognitive-behavioural group programme, and to assess weight gain following cessation, using a randomized, double-blind, placebo-controlled trial with 12 months' follow-up. The main outcome measure was a comparison between point prevalence abstinence, continuous abstinence and 12-months sustained, biochemically validated abstinence. The validated abstinence rates at 12 months for point prevalence, sustained and continuous abstinence for subjects in the active and placebo groups were all statistically significant: 28 vs. 12%; 24 vs. 11%; and 19 vs. 9%, respectively. Moderately dependent smokers using the nicotine patch were more successful than highly dependent smokers at 3 and 6 months (p < 0.05). Weight gain was greater among sustained abstainers at 12 months compared to continuing smokers (p < 0.001). When the active nicotine patch was used in combination with cognitive-behavioural therapy in an outpatient hospital clinic, 12-months sustained abstinence rates were more than double those of a placebo patch. |
| 1831 | Hughes, J R, Hatsukami, D K. Effects of three doses of transdermal nicotine on post-cessation eating, hunger and weight. Journal of substance abuse. 1997;9:151.  **Abstract:** Smokers were randomly assigned to 0, 7, 14 or 21 mg doses of transdermal nicotine and had their eating and weight followed for 6 weeks. Among the 66 subjects who were abstinent for all 6 weeks, caloric, carbohydrate and fat intake, hunger, and weight increased in those on placebo, but alcohol, caffeine and protein intake did not. Nicotine patch decreased caloric, carbohydrate and fat intake in a dose-related manner, showed a trend for weight and showed no effect for hunger. Neither abstinence from smoking nor nicotine therapy shifted the percent of calories from sweets or carbohydrates. We conclude nicotine replacement reduces post-cessation increases in eating but does not have a specific effect on sweet intake. |
| 1832 | Ohnishi, H. Cisapride improves nicotine-evoked antral hypomotility in smokers. The Kurume medical journal. 1997;44:253.  **Abstract:** It has recently been shown that nicotine administration reduces antral motor function. The aim of this study was to test whether cisapride administration can improve nicotine-evoked antral hypomotility in humans. Eight healthy smoking volunteers were selected for the study. After an overnight fast, manometric recordings and electrogastrographic recordings were performed before and after the ingestion of a mixed solid meal. The measurements (manometric and electrogastrographic protocols) were carried out on two separate days. In each protocol, the measurements were made under three different conditions on three separate days; a placebo day, a nicotine day [nicotine patch (14 mg/day)], and a nicotine plus cisapride day (the nicotine patch and 5 mg cisapride) in a crossover randomized design. The number of antral phase III complexes, antral phase III motility indexes (the sum of the highest of all antral phasic contractions > 10 mmHg in 10 min), and postprandial antral motility indexes (the sum of the highest of all antral phasic contractions > 10 mmHg for the duration of 30 min) were significantly lower on the nicotine day than on the placebo day (P < 0.01, P < 0.05, and P < 0.01). However, the values obtained on the nicotine plus cisapride day were significantly higher in comparison with those obtained on the nicotine day (P < 0.05, P < 0.05, and P < 0.01) and were similar to those obtained on the placebo day (P = 0.92, P = 0.79, and P = 0.36). The frequency stability index (maximal signal amplitude for a given 2-minute recording segment occurring at a frequency of > or = 2 cycle/min and < or = 4 cycle/min) was similar on the placebo day, the nicotine day, and the nicotine plus cisapride day in both the fasting (P = 0.69) and postprandial periods (P = 0.22). These results indicate that (a) transdermal nicotine administration reduces antral gastric motor function without modification of slow wave rhythmicity and (b) cisapride administration improves nicotine-evoked antral hypomotility. |
| 1833 | Clavel-Chapelon, F, Paoletti, C, Benhamou, S. Smoking cessation rates 4 years after treatment by nicotine gum and acupuncture. Preventive medicine. 1997;26:25.  **Abstract:** BACKGROUNDThis study was done to estimate the smoking cessation rates 4 years after treatment with acupuncture and nicotine gum.METHODSParticipants were randomized in a 2 x 2 factorial design to four groups: double active treatments (nicotine gum and acupuncture), double placebo, and the combination of one active treatment and placebo.RESULTSThe success rates were quite similar in the four groups at the different points of follow-up. They sharply decreased between 1 month (around 23%) and 1 year (around 10%). The decrease slowed down thereafter to around 6% at 4 years.CONCLUSIONSResults from our study suggest that the two treatments did not offer any long-term improvement over placebo. Additional investigations are necessary to estimate the magnitude of their long-term success rate. |
| 1835 | Joseph, A M, Norman, S M, Ferry, L H, Prochazka, A V, Westman, E C, Steele, B G, Sherman, S E, Cleveland, M, Antonuccio, D O, Antonnucio, D O, Hartman, N, McGovern, P G. The safety of transdermal nicotine as an aid to smoking cessation in patients with cardiac disease. The New England journal of medicine. 1996;335:1792.  **Abstract:** BACKGROUNDTransdermal nicotine therapy is widely used to aid smoking cessation, but there is uncertainty about its safety in patients with cardiac disease.METHODSIn a randomized, double-blind, placebo-controlled trial at 10 Veterans Affairs medical centers, we randomly assigned 584 outpatients (of whom 576 were men) with at least one diagnosis of cardiovascular disease to a 10-week course of transdermal nicotine or placebo as an aid to smoking cessation. The subjects were monitored for a total of 14 weeks for the primary end points of the study (death, myocardial infarction, cardiac arrest, and admission to the hospital due to increased severity of angina, arrhythmia, or congestive heart failure); the secondary end points (admission to the hospital for other reasons and outpatient visits necessitated by increased severity of heart disease); any side effects of therapy; and abstinence from smoking.RESULTSThere were 48 primary and 78 secondary end points noted in a total of 95 subjects. At least one of the primary end points was reached by 5.4 percent of the subjects in the nicotine group and 7.9 percent of the subjects in the placebo group (difference, 2.5 percent; 95 percent confidence interval, -1.6 to 6.5 percent; P=0.23). In the nicotine group, 11.9 percent of the subjects had at least one of the secondary end points, as compared with 9.7 percent in the placebo group (difference, 2.2 percent; 95 percent confidence interval, -2.2 to 7.4 percent; P= 0.37). After 14 weeks the rate of abstinence from smoking was 21 percent in the nicotine group, as compared with 9 percent in the placebo group (P=0.001), but after 24 weeks the abstinence rates were not significantly different (14 percent vs. 11 percent, P= 0.67).CONCLUSIONSTransdermal nicotine does not cause a significant increase in cardiovascular events in high-risk outpatients with cardiac disease. However, the efficacy of transdermal nicotine as an aid to smoking cessation in such patients is limited and may not be sustained over time. |
| 1836 | Taylor, C B, Miller, N H, Herman, S, Smith, P M, Sobel, D, Fisher, L, DeBusk, R F. A nurse-managed smoking cessation program for hospitalized smokers. American journal of public health. 1996;86:1557.  **Abstract:** OBJECTIVESThis study evaluated a nurse-managed smoking cessation program for smokers hospitalized for a variety of conditions.METHODSHospitalized patients who smoked prior to hospitalization and who were motivated to quit (n = 660) were randomized to intervention or usual-care groups and followed for the next year. The intervention included a meeting with the nurse-case manager; the use of a videotape, workbook, relaxation audiotape, and nicotine replacement therapy; and nurse-initiated phone contacts after discharge.RESULTSThe 12-month confirmed cessation rates were 21% and 31% for, respectively, the usual-care and intervention groups (odds ratio = 1.7; 95% confidence interval = 1.1, 2.3).CONCLUSIONSA nurse-managed smoking cessation intervention can significantly increase cessation rates for hospitalized patients. |
| 1837 | Levin, E D, Wilson, W, Rose, J E, McEvoy, J. Nicotine-haloperidol interactions and cognitive performance in schizophrenics. Neuropsychopharmacology : official publication of the American College of Neuropsychopharmacology. 1996;15:429.  **Abstract:** Nearly 90% of schizophrenics smoke cigarettes, considerably higher than the general population's rate of 25%. There is some indication that schizophrenics may smoke as a form of self-medication. Nicotine has a variety of pharmacologic effects that may both counteract some of the cognitive deficits of schizophrenia and counteract some of the adverse side effects of antipsychotic drugs. In the current study, we assessed the interactions of haloperidol and nicotine on cognitive performance of a group of schizophrenics. These patients were in a double-blind study, randomly assigning them to low, moderate, and high dose levels of haloperidol. The subjects, all smokers, came to the laboratory on four different mornings after overnight deprivation from cigarettes. In a double-blind fashion, they were administered placebo, low (7 mg/day), medium (14 mg/day), or high (21 mg/day) dose nicotine skin patches. Three hours after administration of the skin patch, the subjects were given a computerized cognitive test battery including: simple reaction time, complex reaction time (spatial rotation), delayed matching to sample, the Sternberg memory test, and the Conners continuous performance test (CPT). With the placebo nicotine patch, there was a haloperidol dose-related impairment in delayed matching to sample choice accuracy and an increase in response time on the complex reaction time task. Nicotine caused a dose-related reversal of the haloperidol-induced impairments in memory performance and complex reaction time. In the CPT, nicotine reduced the variability in response that is associated with attentional deficit. These results demonstrate the effects of nicotine in reversing some of the adverse side effects of haloperidol and improving cognitive performance in schizophrenia. |
| 1838 | Krogstad, A L, Pegenius, G, Elam, M. Visual scoring and laser Doppler perfusion imaging of skin irritancy induced by different nicotine patches. Skin research and technology : official journal of International Society for Bioengineering and the Skin (ISBS) [and] International Society for Digital Imaging of Skin (ISDIS) [and] International Society for Skin Imaging (ISSI). 1996;2:158.  **Abstract:** BACKGROUND/AIMSLocal skin reactions are the most common reason for discontinuation of transdermal nicotine replacement therapy in smoking cessation programs. The aims of the present study were (1) to quantify the intensity of skin reactions induced by different types of nicotine patches and (2) to compare the clinical evaluation of skin erythema using visual scores with independently performed quantitative estimates of skin perfusion.METHODSThirty-three subjects were included in the study, each receiving 2 different types of nicotine patches (Nicotinell and Nicorette) and 1 type of placebo patch (Nicorette), placed ventrally on the upper arms according to a randomized protocol. Patches were removed after 24 h (Nicotinell) and 16 h (Nicorette), respectively, according to recommended application times. Visual scoring and laser Doppler perfusion imaging were performed 45 min after removal of patches, in a randomized order.RESULTSNicotinell patches induced the highest cumulative clinical score for skin irritancy. All 3 investigated patches gave rise to a slight but significant skin perfusion increase and individual visual scores and perfusion data correlated. Conclusion The degree of skin irritancy and underlying perfusion increase induced by 1 daily maintenance dose of transdermal nicotine via a patch is low, but differs between patch types. |
| 1840 | Nilsson, P, Lundgren, H, Söderström, M, Fagerström, K O, Nilsson-Ehle, P. Effects of smoking cessation on insulin and cardiovascular risk factors--a controlled study of 4 months' duration. Journal of internal medicine. 1996;240:189.  **Abstract:** OBJECTIVESTo investigate the effects on serum lipids, plasma fibrinogen, plasma insulin, plasma C-peptide and blood glucose, of smoking cessation after 4 months. To develop a group-based smoking intervention programme in primary health care.SETTINGTwenty health centres in primary health care in southern Sweden.SUBJECTSFour hundred habitual smokers (> 10 cigarettes per day-1, > 10 years), recruited by advertisement in local papers.INTERVENTIONThe smokers were randomized, after stratification for age and sex, to one intervention group (n = 200) and one control group (n = 200). The intervention group was offered supportive group sessions and free nicotine supplementation (patches, chewing gum).MAIN OUTCOME MEASURESAll participants were investigated at the start and after 4 months (medical history, physical examination, laboratory evaluation). Blood samples were drawn for determination of glucose, insulin and C-peptide, both in the fasting state and during an oral glucose tolerance test (OGTT), and for measurement of lipoproteins, fibrinogen, nicotine and cotinine.RESULTSIn the intervention group 98 of the subjects (48%) had quit smoking after 4 months. They were compared with the 156 subjects in the control group (91%) who were still daily smokers during the whole period. There were no significant differences in any variable between the two (total) experimental groups at baseline. Plasma nicotine and cotinine decreased (P < 0.001) in the intervention group following smoking cessation, and weight increased by 2.7 kg. In the intervention group HDL-cholesterol increased by 11% (P < 0.001), whereas HbA1c increased by 2% (P < 0.05) only in the control group. No changes occurred in levels of glucose, insulin, C-peptide and fibrinogen.CONCLUSIONThe smoking cessation programme had a success rate of almost 50% over 4 months. Smoking cessation was associated with a marked increase in HDL-cholesterol levels but did not affect glucose tolerance. A concomitant weight increase may have blunted any independent beneficial effect of smoking cessation on glucose metabolism. |
| 1842 | Schneider, N G, Olmstead, R, Nilsson, F, Mody, F V, Franzon, M, Doan, K. Efficacy of a nicotine inhaler in smoking cessation: a double-blind, placebo-controlled trial. Addiction (Abingdon, England). 1996;91:1293.  **Abstract:** A non-combustible nicotine inhaler, administered orally, has been developed for treatment of smokers. The inhaler allows weaning from nicotine while maintaining partial reinforcement of the ritual/sensory phenomena of smoking. Subjects were randomly assigned to active (n = 112) and placebo (n = 111) groups. Some behavioral intervention occurred as a function of participation. Strict abstinence (primary outcome criterion) was defined by CO < or = 8 ppm with no slips allowed at any time and cotinine values < or = 14 at 1 year. Survival analysis showed active inhaler was superior to placebo (p < 0.01). Active vs. placebo success rates were: 63% vs. 47% (day 3), 46% vs. 28% (week 1), 36% vs. 19% (week 2), 33% vs. 16% (week 3), 29% vs. 14% (week 6), 24% vs. 10% (3 months), 17% vs. 9% (6 months) and 13% vs. 8% (1 year). chi 2 analyses were significant through 3 months but not at 6 months (p < 0.08) or 1 year. Craving was relieved with active inhalers at day 3 and week 1. Subjects averaged six inhalers/day. Cotinine levels were 57-61% of smoking levels. Common side effects included throat/mouth irritation and coughing. Failure was predicted by early slips. The inhaler is clearly useful for short-term smoking cessation with potential for long-term efficacy. Extended access to the inhaler and relapse prevention training could improve success rates. Another promising approach would be to combine the inhaler with a nicotine patch. |
| 1845 | Thomas, G A, Rhodes, J, Ragunath, K, Mani, V, Williams, G T, Newcombe, R G, Russell, M A, Feyerabend, C. Transdermal nicotine compared with oral prednisolone therapy for active ulcerative colitis. European journal of gastroenterology & hepatology. 1996;8:769.  **Abstract:** BACKGROUNDUlcerative colitis is largely a disease of non-smokers. Previous controlled trials have shown benefit with transdermal nicotine when given with 5-aminosalicylic acid in active disease but not when given alone as maintenance therapy.OBJECTIVETo examine nicotine alone compared with prednisolone in active disease.PATIENTS AND METHODSSixty-one patients with active ulcerative colitis were treated with either transdermal nicotine patches or 15 mg prednisolone for 6 weeks in a randomized, double-blind study. Incremental doses of nicotine were given for the first 9 days; patients tolerated between 15 and 25 mg daily. Most patients were taking mesalazine at entry which was discontinued at day 10; a few were taking topical steroids which were discontinued at the onset. Clinical, sigmoidoscopic and histological assessments were made at baseline and 6 weeks, or at premature withdrawal. Symptoms were recorded on a diary card, and the clinician made a global clinical assessment. Side effects and serum nicotine and cotinine concentrations were monitored throughout the study.RESULTSForty-three patients completed the 6-week trial; of these, 6 of 19 in the nicotine group achieved full sigmoidoscopic remission compared with 14 of 24 with prednisolone (P = 0.08). In those who completed the 6-week study, there was significant improvement within both the nicotine and prednisolone group for the St Mark's score (P < 0.05 and P < 0.01, respectively), Global Clinical Grade (P < 0.01 for both), blood in the stool (P < 0.05 and P < 0.01), abdominal pain (P < 0.05 and P < 0.01) and sigmoidoscopic score (P < 0.01 and P < 0.001); differences between groups tend to favour prednisolone, but none reach statistical significance. However, on intention-to-treat analyses there is little clear evidence of improvement in either group apart from sigmoidoscopic score in which prednisolone was associated with a significantly greater improvement than nicotine (P < or = 0.05). The nicotine group had more withdrawals than the prednisolone group, 11 versus 7, respectively (P = 0.23), both for deterioration (6 vs. 5) and side effects (5 vs. 2, P = 0.15). Side effects were more frequently reported in the nicotine group (average 1.47 episodes per person) than the prednisolone group (average 0.61; P = 0.03), the most common of which were nausea, light-headedness and tremor.CONCLUSIONIn those who managed to complete the 6-week study, nicotine alone appeared to be of only very modest benefit in acute colitis and was not as effective as 15 mg of prednisolone daily. |
| 1848 | Khoury, Z, Comans, P, Keren, A, Lerer, T, Gavish, A, Tzivoni, D. Effects of transdermal nicotine patches on ambulatory ECG monitoring findings: a double-blind study in healthy smokers. Cardiovascular drugs and therapy. 1996;10:179.  **Abstract:** The cardiovascular effects and safety of transdermal nicotine patches were assessed in 50 healthy smokers using repeated 48 hour ambulatory electrocardiographic monitoring as a part of a smoking cessation program. Following baseline measurements, subjects were randomized to active (n = 25) or placebo (n = 25) treatment groups for a period of 2 weeks. Twenty-two patients in each group completed the trial. During the treatment period, subjects also received behavioral supportive therapy. Heart rate and blood pressure were significantly reduced relative to baseline both groups. In the active treatment group, mean values at baseline and after 2 weeks of double-blind treatments were as follows: heart rates, 74.0 and 71.3 beats/min, respectively; systolic blood pressure, 108.9 and 106.9 mmHg; and diastolic blood pressure, 69.7 and 68.2 mmHg, respectively. Values for the placebo group were as follows: heart rate, 73.2 and 69.6 beats/min; systolic blood pressure, 110.6 and 105.3 mmHg; diastolic blood pressure, 71.4 and 70.5 mmHg. The confidence intervals of the mean for the differences between the groups are as follows: heart rate, -4.0 +8.2; systolic blood pressure, -6.7, +10.1; diastolic blood pressure, -7.9, +3.9. There were no changes in the frequency of atrial or ventricular arrhythmia as documented by repeated 48 hour ambulatory ECG recordings from baseline period before stopping smoking compared with the treatment period in the active as well as in the placebo groups. No ischemic events were detected in any of the volunteers during either the baseline or treatment periods. As a measure of smoking abstinence, we assessed the level of carbon monoxide in expired air; in the active group, the mean levels fell from 14.2 to 4.4 ppm after smoking cessation and in the placebo group from 13.2 to 4.2 ppm. The mean urine cotinine level fell from 8.18 mmol/l at baseline to 5.74 mmol/l after 2 weeks of treatment in the active group and from 8.78 to 3.93 mmol/l in the placebo group. The number of cigarettes smoked per week in both treatment groups was significantly reduced: from 175.2 to 8.4 for the active group and from 136 to 8.6 for the placebo group. Eleven out of 22 subjects in each group quit smoking completely. Smoking withdrawal symptoms during the first week of treatment were more severe in the placebo group than in those on active treatment. There were no significant side effects in either the placebo or the active treatment groups. These results indicate that nicotine delivered by transdermal system is free of cardiac adverse effects in healthy volunteers. |
| 1850 | Paoletti, P, Fornai, E, Maggiorelli, F, Puntoni, R, Viegi, G, Carrozzi, L, Corlando, A, Gustavsson, G, Säwe, U, Giuntini, C. Importance of baseline cotinine plasma values in smoking cessation: results from a double-blind study with nicotine patch. The European respiratory journal. 1996;9:643.  **Abstract:** Nicotine replacement by transdermal patches is more effective than placebo in smoking cessation, but has a low success rate after one year (9-18%). We tested whether this was attributed to insufficient nicotine replacement. We conducted a randomized trial to investigate the effect on outcome of different doses of transdermal nicotine replacement after stratification according to baseline plasma cotinine values. Two hundred and ninety seven adult smokers were enrolled. Those with baseline cotinine < or = 250 ng.ml-1 (low cotinine) were randomly assigned to placebo (LC-P) or to 15 mg 16 h nicotine patches (LC-15), and those with baseline cotinine > 250 ng.mL-1 (high cotinine) were randomly assigned to 15 mg (HC-15) or 25 mg (HC-25) 16 h nicotine patches. Plasma nicotine and cotinine values, expired carbon monoxide and withdrawal symptoms were measured at scheduled intervals during treatment. Smokers in the LC-15 group had a significantly higher success rate than placebo (28 vs 9%). Smokers with high baseline cotinine had lower success rates, and a high dose of nicotine did not increase success rate (HC-25 9% vs HC-15 11%). Subjects in the HC-15 group had the lowest percentage of nicotine replacement and a higher prevalence of withdrawal symptoms than the HC-25 group. Replacement was similar in groups LC-15 and HC-25, but the success rate was significantly lower in HC-25 group, despite similar levels of withdrawal symptoms. We conclude that a higher success rate was obtained after one year in smokers with low baseline plasma cotinine values. Determination of plasma cotinine values may be, thus, helpful in identifying smokers who could benefit from transdermal nicotine replacement. |
| 1851 | Yudkin, P L, Jones, L, Lancaster, T, Fowler, G H. Which smokers are helped to give up smoking using transdermal nicotine patches? Results from a randomized, double-blind, placebo-controlled trial. The British journal of general practice : the journal of the Royal College of General Practitioners. 1996;46:145.  **Abstract:** BACKGROUNDNicotine replacement therapy is effective in helping people to give up smoking. The three forms now available--transdermal patches, chewing gum and nasal spray--deliver nicotine at different rates and to different levels. Therefore, it might be expected that smokers with different characteristics, and at different levels of nicotine dependence, will be helped more by one or other method.AIMThe aim of the study was to examine whether the effectiveness of transdermal patches is related to nicotine dependence or to other smoker characteristics and to investigate factors predicting smoking cessation using patches.METHODData from a randomized, double-blind, placebo-controlled trial of nicotine transdermal patches were analysed retrospectively. The trial, conducted in 1990-1992, involved 1686 patients recruited from 19 general practices in Oxfordshire. The main outcome measure was continuous smoking cessation from 8 to 52 weeks after the start of patch use, biochemically validated at 12, 24 and 52 weeks. The effectiveness of the patches was measured by the relative odds of sustained cessation using nicotine patches compared with placebo patches.RESULTSNicotine transdermal patches were more effective in smokers with moderate nicotine dependence [odds ratio (OR) 1.94; 95% confidence interval (CI) 1.24-3.04] than in mildly or highly dependent smokers (OR 0.98; 95% CI 0.58-1.65) (difference in ORs P < 0.05) and more effective in those aged 24-49 years (OR 1.89; 95% CI 1.24-2.87) than in older smokers aged 50-65 years (OR 0.88; 95% CI 0.49-1.59) (difference in ORs P < 0.05). Abstinence from smoking in the first week of the trial was the strongest predictor of sustained cessation and was more common among smokers using nicotine patches than those using placebo patches (33% of 842 compared with 22% of 844; P < 0.001). Of first-week abstainers, 25 and 28% of 277 and 182 in the nicotine and placebo groups, respectively, achieved sustained cessation compared with 4% of 565 and 2% of 662 first-week smokers.CONCLUSIONNicotine transdermal patches were most effective for smokers with moderate nicotine dependence and for younger smokers. Early abstinence from smoking was the strongest predictor of sustained cessation. A week's trial of the patch proceeding to longer term use if abstinence is achieved may be an effective policy. |
| 1852 | Murray, R P, Bailey, W C, Daniels, K, Bjornson, W M, Kurnow, K, Connett, J E, Nides, M A, Kiley, J P. Safety of nicotine polacrilex gum used by 3,094 participants in the Lung Health Study. Lung Health Study Research Group. Chest. 1996;109:438.  **Abstract:** STUDY OBJECTIVETo assess cardiovascular conditions and other side effects associated with the use of nicotine polacrilex (NP), 2 mg.DESIGNA multicentered randomized control trial of early intervention for the prevention of COPD.SETTINGTen university medical centers in the United States and Canada.PARTICIPANTSAdult smoking volunteers with evidence of early COPD; 3,923 in intervention and 1,964 controls.INTERVENTIONSmoking cessation program, including NP.MEASUREMENTSData on hospitalizations were collected annually. Data on reported NP side effects were collected at 4-month intervals for intervention participants.RESULTSThe rates of hospitalization for cardiovascular conditions and cardiovascular deaths during the 5 years of the study were not related to use of NP, to dose of NP, or to concomitant use of NP and cigarettes. About 25% of NP users reported at least one side effect, but most were very minor and transient. Side effects associated with discontinuance of NP in 5% or more of users included headache, indigestion, mouth irritation, mouth ulcers, and nausea. There was no evidence that concomitant use of NP and cigarettes was associated with elevated rates of reported side effects. Participants in the smoking cessation intervention who received intensive levels of instruction and monitoring of NP use (initially at 12 meetings during 3 months) appeared to report significantly lower rates of side effects (dizziness, headache, and throat irritation) than control participants, presumed to have less instruction and monitoring.CONCLUSIONSNP, as used in the Lung Health Study, appears to be safe and unrelated to any cardiovascular illnesses or other serous side effects. |
| 1853 | Hatsukami, D, Jensen, J, Allen, S, Grillo, M, Bliss, R. Effects of behavioral and pharmacological treatment on smokeless tobacco users. Journal of consulting and clinical psychology. 1996;64:153.  **Abstract:** The purpose of this study was to examine the effects of 2 mg of nicotine polacrilex versus placebo gum and of group behavioral treatment versus minimal contact on cessation of smokeless tobacco use. Participants (N = 210) were randomly assigned 1 of the 4 treatment conditions. Withdrawal symptoms were assessed throughout the treatment. Follow-up assessments were made at 1, 6, and 12 months posttreatment. Survival curve analysis showed that any of the 3 treatment groups involving group behavioral therapy or placebo gum were equally effective and superior to the minimal contact plus 2 mg of nicotine gum treatment in terms of abstinence. On the other hand, withdrawal symptoms were significantly reduced by nicotine gum, compared with placebo during the initial phases of cessation. The ineffectiveness of nicotine gum on treatment outcome may be attributed to its similarity with smokeless tobacco. |
| 1854 | Murray, Robert P., Bailey, William C., Daniels, Kathleen, Bjornson, Wendy M., Kurnow, Karole, Connett, John E., Nides, Mitchell A., Kiley, James P.. Safety of nicotine polacrilex gum used by 3,094 participants in the Lung Health Study. Chest. 1996;109:438.  **Abstract:** Study objective: To assess cardiovascular conditions and other side effects associated with the use of nicotine polacrilex (NP), 2 mg. Design: A multicentered randomized control trial of early intervention for the prevention of COPD. Setting: Ten university medical centers in the United States and Canada. Participants: Adult smoking volunteers with evidence of early COPD; 3,923 in intervention and 1,964 controls. Intervention: Smoking cessation program, including NP. Measurements: Data on hospitalizations were collected annually. Data on reported NP side effects were collected at 4- month intervals for intervention participants. Results: The rates of hospitalization for cardiovascular conditions and cardiovascular deaths during the 5 years of the study were not related to use of NP, to dose of NP, or to concomitant use of NP and cigarettes. About 25% of NP users reported at least one side effect, but most were very minor and transient. Side effects associated with discontinuance of NP in 5% or more of users included headache, indigestion, mouth irritation, mouth ulcers, and nausea. There was no evidence that concomitant use of NP and cigarettes was associated with elevated rates of reported side effects. Participants in the smoking cessation intervention who received intensive levels of instruction and monitoring of NP use (initially at 12 meetings during 3 months) appeared to report significantly lower rates of side effects (dizziness, headache, and throat irritation) than control participants, presumed to have less instruction and monitoring. Conclusions: NP, as used in the Lung Health Study, appears to be safe and unrelated to any cardiovascular illnesses or other serious side effects. |
| 1855 | Schneider, N G, Olmstead, R, Mody, F V, Doan, K, Franzon, M, Jarvik, M E, Steinberg, C. Efficacy of a nicotine nasal spray in smoking cessation: a placebo-controlled, double-blind trial. Addiction (Abingdon, England). 1995;90:1671.  **Abstract:** Laboratory trials have demonstrated the efficacy of nicotine replacement in smoking cessation but absolute success rates are low. For many, nicotine gum is hard to use and transdermal nicotine is slow-acting and passive. A new, faster-acting nicotine nasal spray (NNS) can provide easily self-administered relief from cigarette withdrawal. The NNS was tested for safety and efficacy in smoking cessation. Two hundred and fifty-five smokers were randomized to NNS or a piperine placebo. Drug use was limited to 8-32 doses/day for 6 months. Subjects were tested while smoking and at post-cessation daily (week 1) with follow-up at weeks 2, 3, 6 and at 3 months, 6 months and 1 year. Continuous abstinence analyses (CO < or = 8 ppm; no slips) showed that NNS significantly enhanced success rates over placebo overall (p < 0.001) and at all test intervals. Differences at key intervals between active and placebo were: 63% vs. 40% (day 5), 51% vs. 30% (week 3), 43% vs. 20% (6 weeks), 34% vs. 13% (3 months), 25% vs. 10% (6 months) and 18% vs. 8% (1 year). Side effects were common but tolerable. Cotinine measures showed that replacement of nicotine approximated 30% of smoking levels. Hazard functions revealed relapse risks peaked at day 1, day 5 and 3 weeks for strict abstinence. It is concluded NNS is safe, efficacious and a viable alternative treatment for smoking cessation. |
| 1858 | Dale, L C, Hurt, R D, Offord, K P, Lawson, G M, Croghan, I T, Schroeder, D R. High-dose nicotine patch therapy. Percentage of replacement and smoking cessation. JAMA. 1995;274:1353.  **Abstract:** OBJECTIVETo assess the level of nicotine replacement, evidence of nicotine toxicity, and withdrawal symptom relief with placebo and 11-, 22-, and 44-mg/d doses of transdermal nicotine. A secondary objective was to assess short- and long-term smoking cessation rates.DESIGNRandomized, double-blind, placebo-controlled inpatient/outpatient trial.SUBJECTSSeventy-one cigarette smokers stratified according to light (n = 23), moderate (n = 24), and heavy (n = 24) smoking rates.INTERVENTIONSAfter baseline measures were obtained, subjects were randomly assigned to placebo or an 11-, 22-, or 44-mg/d dose of transdermal nicotine and admitted to a special hospital unit for intensive inpatient treatment of nicotine dependence. During the 6-day inpatient stay, daily nicotine and cotinine levels were determined from trough and peak blood samples. Outpatient patch therapy continued for 7 weeks following the hospital stay, and those initially assigned to placebo were randomly assigned to 11 or 22 mg/d. At week 4, the dosage of those initially assigned to 44 mg/d was reduced to 22 mg/d.MAIN OUTCOME MEASURESPercentage of replacement of cotinine was calculated by dividing the steady-state levels attained during patch therapy by the corresponding baseline levels. Abstinence from smoking was verified by expired air carbon monoxide. Withdrawal symptoms and nicotine toxicity were assessed daily through questionnaires during the inpatient stay. Follow-up visits were at 3, 6, 9, and 12 months.RESULTSThere was a statistically significant relationship between baseline smoking rate and baseline trough and peak blood cotinine levels (rs = 0.39, rs = 0.45; P < .001 in both instances). A dose-response relationship was observed with higher patch doses, which produced a higher percentage of cotinine replacement and better withdrawal symptom relief. Only one subject (a light smoker assigned to the 44-mg dose) developed signs of nicotine toxicity. There was a positive association between the week 2 patch dose and the biochemically confirmed abstinence at the end of patch therapy (P = .007) but not for subsequent follow-up times. A higher percentage of cotinine replacement was associated with the higher 8-week smoking abstinence rate (P = .03), an association not found at long-term follow-up.CONCLUSIONA 44-mg/d dose of nicotine patch therapy appears to be safe for use in heavy smokers. Cigarette smoking rates can be used to estimate the initial nicotine patch dose. Monitoring blood cotinine levels at baseline and steady state can be used for assessing the adequacy of nicotine replacement. Withdrawal symptom relief can be improved with more complete nicotine replacement. Achieving a greater percentage of nicotine replacement may increase the efficacy of nicotine patch therapy. |
| 1860 | Kavin, H, Shivley, S. Absence of effect of nicotine on rectal sensation, rectal compliance, and anal sphincter pressures in healthy subjects. Digestive diseases and sciences. 1995;40:2239.  **Abstract:** We examined the effect of nicotine on rectal sensation, rectal compliance, and anorectal sphincter function in healthy volunteers. Eleven healthy (ex-smoker) subjects were randomized in a double-blind, crossover, placebo-controlled study of 12 mg nicotine-containing chewing gum. All treatment periods (nicotine or placebo chewing gum) were preceded by a control period without chewing gum. Crossover study was done after a washout period of more than seven days. The following measurements were made: highest anorectal sphincter tone, highest anorectal sphincter squeeze tone, percentage relaxation of the anorectal sphincter with rectal balloon distension, threshold of rectal sensation, maximal tolerable volume of air inflation of a rectal balloon, and rectal compliance. There was no significant difference in the two control periods. Chewing placebo gum had no significant effect on any of the measurements when compared with control. Compared with placebo, nicotine did not significantly affect on any of the measurements. We conclude that neither nicotine nor the sham-feeding effect of chewing placebo gum appear to have any effect on anorectal sensorimotor function or on rectal compliance in healthy ex-smokers. |
| 1861 | Gourlay, S G, Forbes, A, Marriner, T, Pethica, D, McNeil, J J. Double blind trial of repeated treatment with transdermal nicotine for relapsed smokers. BMJ (Clinical research ed.). 1995;311:363.  **Abstract:** OBJECTIVETo assess the efficacy and safety of a repeat course of treatment with transdermal nicotine for cessation of smoking in a brief intervention setting.STUDY DESIGNRandomised, double blind, placebo controlled trial with follow up for 26 weeks.SUBJECTS629 smokers who had unsuccessfully attempted to stop smoking by using active transdermal nicotine and brief behavioural counselling. Smokers were motivated to quit smoking for a second time and smoked > or = 15 cigarettes a day.INTERVENTIONSTwelve weeks' treatment with active transdermal nicotine patches or placebo and brief counselling at monthly visits.MAIN OUTCOME MEASURESustained smoking cessation for the 28 days before the visit at week 12 verified by expired carbon monoxide concentrations.RESULTSAt 12 weeks 21/315 (6.7%) subjects allocated to active treatment had stopped smoking compared with 6/314 (1.9%) allocated to placebo (absolute difference 4.7%; 95% confidence interval 1.6% to 7.9%; P = 0.003). At 26 weeks the rates were 20/315 (6.4%) and 8/314 (2.6%) (3.8%; 0.6% to 7.0%; P = 0.021). Difficulty in sleeping was reported by 43/179 (24.0%) on active treatment and 19/143 (13.3%) on placebo (P = 0.015). Severe reactions at the site of application were rare (6/322; 1.9%).CONCLUSIONSRepeated treatment with transdermal nicotine together with brief counselling can improve the low success rates of smoking cessation in recently relapsed, moderate to heavy smokers. Questions remain about whether more intensive interventions or higher doses of nicotine could be more effective. The likelihood of severe reactions at the site of application with repeated treatment is low. |
| 1862 | Herrera, N, Franco, R, Herrera, L, Partidas, A, Rolando, R, Fagerström, K O. Nicotine gum, 2 and 4 mg, for nicotine dependence. A double-blind placebo-controlled trial within a behavior modification support program. Chest. 1995;108:447.  **Abstract:** The effectiveness of nicotine gum in combination with a behavior modification program was studied. The nicotine dependence of participating smokers (N = 322) was assessed. One hundred sixty-eight smokers were labeled as high nicotine dependent and 154 as moderate to low dependent. In a randomized double-blind procedure, the high-dependent smokers were given gum containing 4 mg of nicotine (87) or 2 mg of nicotine (81) and the smokers with medium or low dependence were given gum containing 2 mg (76) or a placebo gum (78). The smokers were also randomized to familiarizing themselves with the medication a week before quit day (112) or to regular use, that is starting gum use on the quit day (122). In the high-dependent group, sustained and chemically verified nonsmoking rates at 6 weeks, 1 year, and 2 years were, respectively, 60%, 39%, and 34% in the subjects given the 4-mg dose compared with 41%, 16%, and 16% for those using the 2-mg dose. In the group with medium or low dependence, the success rates at the same time periods were 70%, 49%, and 39% for the subjects given the 2-mg dose and 38%, 22%, and 17% for those given placebo gum. The differences in success rates were significant at least at the p < 0.02% level for all comparisons. Familiarizing with the gum as compared with regular use gave fewer reports of side effects, 15% vs 34%, p < 0.001. A trend toward better success rates at 6 weeks, although not statistically significant, was observed for the familiarization group, 61% vs 52%. The study shows that high nicotine-dependent smokers need higher doses of nicotine replacement, in this case the 4-mg dose rather than the 2-mg dose, whereas 2 mg is superior to placebo among less dependent smokers. These results compare favorably with those reported from the more recent nicotine patch therapy. |
| 1863 | Wetter, D W, Fiore, M C, Baker, T B, Young, T B. Tobacco withdrawal and nicotine replacement influence objective measures of sleep. Journal of consulting and clinical psychology. 1995;63:658.  **Abstract:** Research has not adequately characterized the impact of tobacco withdrawal on objectively assessed sleep parameters despite the recent inclusion of insomnia as a nicotine withdrawal sign in the Diagnostic and Statistical Manual of Mental Disorders (4th ed.; American Psychiatric Association, 1994). Moreover, whether 24-hr nicotine replacement aids or interferes with sleep during withdrawal is unknown. In a double-masked, randomized clinical trial, 34 cigarette smokers who were motivated to quit received either active nicotine patches or placebo patches while quitting. Sleep was polysomnographically monitored for 2 precessation nights and 3 postcessation nights. The study demonstrates that among dependent smokers (a) tobacco withdrawal increases objectively assessed sleep disturbance (sleep fragmentation) and (b) nicotine replacement results in postcessation improvements in important polysomnographic measures of sleep quality (sleep fragmentation, Stage 3 and Stage 4 sleep). |
| 1864 | Allen, S S, Hatsukami, D, Jensen, J, Grillo, M, Bliss, R. Effects of treatment on cardiovascular risk among smokeless tobacco users. Preventive medicine. 1995;24:357.  **Abstract:** BACKGROUNDStudies show sustained levels of nicotine among young males using smokeless tobacco, causing concern for subsequent cardiovascular risk. Also, there is little information on effects of nicotine replacement on cardiovascular risk in cessation programs. This study investigates the effects of nicotine gum replacement in smokeless tobacco cessation on cardiovascular risk factors.METHODSSmokeless tobacco users, ages 18-65, were randomly assigned in a double-blind fashion to 2-mg nicotine or placebo gum. At baseline, Week 4, and Week 8, dependent measurements, heart rate, blood pressure, and weight were recorded, and fasting lipoprotein profiles were drawn.RESULTSThis paper focuses on the smokeless tobacco users who refrained from use during the study period (N = 56). The nicotine gum group weighed less (P = 0.033) than the placebo group throughout the study and weight increased at a significant rate between Weeks 4 and 8 for both groups as gum decreased. Triglycerides were higher for the nicotine gum group than the placebo group (P = 0.031), with triglycerides decreasing between Weeks 4 and 8, with a similar effect seen among nonabstinent smokeless tobacco users. There was no dose, time, or dose by time effect for the other dependent measures.CONCLUSIONSAmong smokeless tobacco users who were abstinent, weight increased, with subjects on nicotine gum weighing less throughout the study. The lipoprotein profile, heart rate, and blood pressure did not improve over time, contrary to smokers in whom HDL increases and heart rate decreases with cessation. This could relate to different routes of administration, pharmacokinetics, or by-products of tobacco smoking being absent in smokeless tobacco. In addition, nicotine gum appeared to have neither an adverse nor a positive effect on heart rate, blood pressure, LDL, HDL, or total cholesterol. |
| 1865 | Fortmann, S P, Killen, J D. Nicotine gum and self-help behavioral treatment for smoking relapse prevention: results from a trial using population-based recruitment. Journal of consulting and clinical psychology. 1995;63:460.  **Abstract:** Smokers aged 18 to 65 years (N = 1,044) who were able to quit for 24 hr were randomized using a 2 x 2 factorial design to compare nicotine gum to no gum use and self-help materials to no use of materials. All participants were offered a 0 incentive to quit and stay quit for 6 months. Six-month abstinence was 27% in the gum groups, compared with 19% in the no-gum group (p = .002). Compared with the no-gum group, relapse occurred at a significantly lower rate in the gum group for the entire 12 months of follow-up (odds of relapse in the gum group was 0.72, 95% confidence interval, 0.62 to 0.83). There was no significant main effect for the self-help materials, no interaction between gum and materials, and no evidence that the effectiveness of gum differed between the sexes or between heavy and light smokers. Nicotine gum is an effective adjunct to minimal-contact smoking cessation materials plus monetary incentive in a population-based sample of smokers. |
| 1866 | Søreide, E, Holst-Larsen, H, Veel, T, Steen, P A. The effects of chewing gum on gastric content prior to induction of general anesthesia. Anesthesia and analgesia. 1995;80:985.  **Abstract:** To study the effects on gastric content and subjective well being of chewing gum in the immediate preoperative period, 60 female nonsmokers were randomized to use regular, sugar-free chewing gum preoperatively or to continue the overnight fast. In a similar fashion 44 habitual smokers were randomized to use nicotine gum 2 mg or not. Nonsmokers using chewing gum had significantly larger gastric fluid volumes than controls (mean 30 +/- 19 mL vs 20 +/- 15 mL; 95% confidence interval (CI) for difference 1-19 mL; P = 0.03), with no difference in gastric fluid acidity. In smokers, neither gastric fluid volume nor acidity differed significantly between those who were or were not chewing gum. Although the use of nicotine gum in smokers was associated with a reduction in dryness of the mouth, thirst, and irritability, nonsmokers chewing regular gum did not report significant improvements in patient well being. In habitual smokers unable to abstain from nicotine, the use of nicotine gum on the morning of surgery may be beneficial. Although it is difficult to prove a direct influence on the incidence of pulmonary aspiration of increased gastric contents, the fact that regular, sugar-free chewing gum increased gastric fluid volumes probably means that it should not be used on the morning of surgery. |
| 1869 | Thomas, G A, Rhodes, J, Mani, V, Williams, G T, Newcombe, R G, Russell, M A, Feyerabend, C. Transdermal nicotine as maintenance therapy for ulcerative colitis. The New England journal of medicine. 1995;332:988.  **Abstract:** BACKGROUNDUlcerative colitis is largely a disease of nonsmokers. Having found previously that treatment with transdermal nicotine patches and mesalamine (5-aminosalicylic acid) has a beneficial effect on active colitis, we examined the value of transdermal nicotine for the maintenance of remission.METHODSWe treated 80 patients with ulcerative colitis in remission with either transdermal nicotine or placebo patches for six months in a randomized, double-blind study. Incremental doses of nicotine were given for the first three weeks to achieve a maintenance dose; most patients tolerated 15 mg for 16 hours daily. All patients were taking mesalamine preparations as maintenance treatment at entry into the study; this treatment was stopped once the maintenance dose of nicotine was achieved. Clinical, sigmoidoscopic, and histologic assessments were made at the beginning and the end of the study, or at relapse. Side effects and serum nicotine and cotinine concentrations were monitored throughout the study.RESULTSThere was no significant difference in the number of relapses between the groups. Twenty-two patients in the nicotine group were prematurely withdrawn from the study, 14 because of relapse and 8 for other reasons, including side effects and protocol violations. In the placebo group, 20 patients were withdrawn prematurely, 17 because of relapse and 3 for other reasons. Among patients using 15-mg nicotine patches, serum nicotine and cotinine concentrations were lower than expected and may reflect poor compliance. Side effects were reported by 35 patients--21 in the nicotine group and 14 in the placebo group--the most common of which were nausea, lightheadedness, and itching.CONCLUSIONSTransdermal nicotine alone was no better than placebo in the maintenance of remission of ulcerative colitis, and premature withdrawal due to side effects was more common in the nicotine group. |
| 1870 | Hurt, R D, Offord, K P, Lauger, G G, Marusić, Z, Fagerström, K O, Enright, P L, Scanlon, P D. Cessation of long-term nicotine gum use--a prospective, randomized trial. Addiction (Abingdon, England). 1995;90:407.  **Abstract:** Nicotine gum is an important adjunct for smoking cessation for many smokers, and long-term use of nicotine gum will occur in a small percentage of patients. To date, no method of cessation in long-term users has been studied in a randomized trial. We enrolled 26 subjects at the Mayo Clinic site of the Lung Health Study who had used nicotine gum for more than 6 months to participate in a trial where subjects were randomly assigned to: (1) abrupt cessation, (2) taper with placebo gum, or (3) taper with active gum. At the end of the 6-week trial, the percentage of subjects abstinent from gum use and not smoking was not different among the three groups: 66.7% for the abrupt cessation group, 71.4% for the taper with placebo gum group and 60% for the taper with active gum group. One subject in the taper with placebo gum group relapsed to smoking during the trial but was abstinent from smoking again at long-term follow-up. Long-term follow-up (median 284 days) showed 65% of subjects were abstinent from all nicotine products. Motivated subjects can stop long-term nicotine gum use without relapse to gum use or smoking by either abrupt cessation or brief tapering. |
| 1871 | Stapleton, J A, Russell, M A, Feyerabend, C, Wiseman, S M, Gustavsson, G, Sawe, U, Wiseman, D. Dose effects and predictors of outcome in a randomized trial of transdermal nicotine patches in general practice. Addiction (Abingdon, England). 1995;90:31.  **Abstract:** The transdermal nicotine patch has proved an effective aid to smoking cessation. The ease of securing good compliance gives it a potential advantage over nicotine gum as an adjunct to brief advice and support in primary care settings where the major public health impact is obtained. In a preliminary report of half the sample of a randomized placebo controlled trial, we showed the patch to be effective in a general practice setting. We report here the definitive results of the full sample, including dose effects, predictors of outcome and other issues of theoretical and practical interest. A total of 1200 heavy smokers (> or = 15 per day), attending 30 general practices in 15 English counties received brief GP advice, a booklet and 16 hours per day patch treatment for 18 weeks. Dose increase and abrupt vs. gradual reduction of patch dosage were also randomized and follow-ups conducted at 1, 3, 6, 12, 26 and 52 weeks. Outcome was measured by self-reported complete abstinence from week 3 to 52 with biochemical validation at all follow-up points. Nicotine patch treatment doubled the rate of continuous abstinence up to 1 year (nicotine 9.6%, placebo 4.8%, p < 0.01); it most likely worked by reducing withdrawal symptoms. It enhanced cessation during the first week and reduced relapse during the second week. The dose increase after week 1 produced no sustained increase in cessation. Gradual reduction was no better at preventing relapse than abrupt withdrawal of patches after week 12. Whether relapse would have increased by ending treatment at some point between weeks 3 and 12 was not tested. Although pre-treatment dependence on cigarettes was prognostic of failure, the patches were equally helpful to both highly and less dependent smokers. Patches were particularly helpful to smokers with pre-treatment subclinical dysthymic symptoms. All but one of the 96 subjects eventually achieving long-term abstinence in the study quit during the first week of cessation. |
| 1874 | Lunell, E, Molander, L, Leischow, S J, Fagerström, K O. Effect of nicotine vapour inhalation on the relief of tobacco withdrawal symptoms. European journal of clinical pharmacology. 1995;48:235.  **Abstract:** Fifteen subjects participated in a randomised, placebo-controlled cross-over study to assess the effect of a nicotine vapour inhaler on craving and other withdrawal symptoms during a two-day smoking-free period. Craving and withdrawal symptoms were rated nine times over the two-day period on 10 cm visual analogue scales. Plasma nicotine concentrations in the afternoon of each study day were determined. The results show that active treatment was significantly superior to placebo in decreasing craving and other withdrawal symptom scores. No difference was found between two inhalation techniques, one with shallow, frequent inhalations (buccal technique), and the other with deep inhalations (pulmonary technique). The average number of active nicotine vapour inhalers and placebo inhalers used during the two-day sessions was 12 and 11, respectively. Afternoon plasma nicotine levels of approximately 7 ng/ml were obtained with both inhalation techniques. A strong correlation was found between the afternoon plasma nicotine levels and craving, a high nicotine level being associated with a low craving score. The study has provided information about how to use the nicotine vapour inhaler that could have important implications if it were to be approved for the treatment of tobacco dependence. The use of withdrawal symptom reduction as a surrogate end-point is discussed. |
| 1877 | Kornitzer, M, Boutsen, M, Dramaix, M, Thijs, J, Gustavsson, G. Combined use of nicotine patch and gum in smoking cessation: a placebo-controlled clinical trial. Preventive medicine. 1995;24:41.  **Abstract:** BACKGROUNDSmoking is considered as an addiction to nicotine for most subjects consuming 10 cigarettes or more per day. Hence, nicotine replacement therapy by way of gum, patch, or spray has been advocated. The rationale of this study is to evaluate the possible beneficial effects of adding nicotine gum to the routine of subjects using the nicotine patch. The effect of the nicotine patch against the placebo, both groups receiving placebo nicotine gum, has also been assessed.METHODSHealthy subjects (374) were randomized at their work-setting in a 1-year double-blind placebo-controlled trial: 149 subjects to active nicotine patch + active gum (group 1), 150 to active nicotine patch + placebo gum (group 2), and 75 to placebo patch + gum (group 3). Treatment duration was 12 weeks with a 16-hr transdermal patch of 15 mg, followed by a 6 + 6-weeks weaning period on respectively 10 and 5 mg patches. Gum use was not restricted during the first 6 months, with recommendations to use at least four pieces a day. A strict definition of smoking abstinence was used in this study, which did not allow smoking any cigarette after Week 1. Nonsmoking status at each visit, as reported by the subjects, was verified by CO below 10 ppm in expired air.RESULTSAbstinence rates in group 1 against group 2 were 34.2 and 22.7% (P = 0.027) at 12 weeks, 27.5 and 15.3% (P = 0.010) at 24 weeks, and 18.1 and 12.7% (P = 0.191) at 52 weeks. In group 3, abstinence rates were 17.3, 14.7, and 13.3% respectively at 12, 24, and 52 weeks. Using logistic regression with adjustment for six baseline covariates, odds ratios for abstinence (with 95% CI) were computed. For group 1/group 2, OR at 12, 24, and 52 weeks were 1.72 (1.03-2.94) (P = 0.039), 2.04 (1.14-3.57) (P = 0.018), and 1.47 (0.76-2.76) (P = 0.125). No significant differences in OR were observed when comparing groups 2 and 3. Time to relapse is significantly longer in group 1 as compared to that of group 2 (P = 0.041), whereas no significant differences between groups 2 and 3 were observed. No significant differences between the three groups in systemic and local adverse drug events were observed.CONCLUSIONAdding active gum use to active patch use in subjects smoking 10 cigarettes or more a day increased abstinence rates, which are statistically significant up to 24 weeks. |
| 1878 | Clemens, P, Baron, J A, Coffey, D, Reeves, A. The short-term effect of nicotine chewing gum in patients with Parkinson's disease. Psychopharmacology. 1995;117:253.  **Abstract:** Because of the inverse association of cigarette smoking with the risk of Parkinson's disease, we performed a short-term, double-blind, randomized controlled trial of nicotine polacrilex resin gum in patients with this disease. Forty-eight subjects were randomly assigned to chew either nicotine gum or placebo gum three times at 2-h intervals, with evaluation of symptoms before and after the trial. The nicotine gum was reasonably well tolerated, but there were no substantial differences in Parkinson's disease symptoms between the two treatment groups. |
| 1880 | Hjalmarson, A, Franzon, M, Westin, A, Wiklund, O. Effect of nicotine nasal spray on smoking cessation. A randomized, placebo-controlled, double-blind study. Archives of internal medicine. 1994;154:2567.  **Abstract:** BACKGROUNDNicotine replacement therapies have proved to be of value in smoking cessation. However, not all smokers can use the nicotine gum or nicotine patch owing to side effects. In addition, the absorption of nicotine from these formulas is slow compared with smoking. A nicotine nasal spray delivers nicotine more rapidly. The objective of this study was to evaluate the efficacy and safety of the nicotine nasal spray for smoking cessation.METHODSSubjects were recruited through advertisements in newspapers and among patients referred to the smoking cessation clinic at Sahlgren's Hospital, Göteborg, Sweden. Two hundred forty-eight smokers were treated in small groups with eight counseling sessions over 6 weeks. At their first group session, subjects were randomized to a group receiving nicotine spray (n = 125), 0.5 mg of nicotine per single spray, or to a placebo group (n = 123). The procedure was double blind. Success rates were measured up to 12 months. The nonsmoking status was verified by expired carbon monoxide less than 10 ppm.RESULTSSignificantly more subjects in the nicotine group were continuously abstinent for 12 months than in the placebo group (27% vs 15%; odds ratio, 2.16; 95% confidence interval, 1.15 to 4.12). Ten of the 34 abstinent subjects in the nicotine group used the spray for 1 year. Mild or moderate side effects were rather frequent for both sprays, but they were significantly more for the nicotine spray. Subjects with high scores (> 7) on Fagerström's tolerance questionnaire had a significantly lower success rate with placebo than with the nicotine spray. For subjects with low scores, there was no difference.CONCLUSIONNicotine nasal spray in combination with group treatment is an effective aid to smoking cessation. |
| 1882 | Wewers, M E, Tejwani, G A, Anderson, J. Plasma nicotine, plasma beta-endorphin and mood states during periods of chronic smoking, abstinence and nicotine replacement. Psychopharmacology. 1994;116:98.  **Abstract:** Nicotine is known to release neuroendocrine substances which may subsequently reinforce smoking behavior by improving mood states. The purpose of this study was to examine changes in plasma beta-endorphin and mood states during periods of chronic smoking, abstinence from smoking, and abstinence while chewing nicotine gum. A modified A-B-A-C design was used. Normal male volunteers were randomly assigned to an experimental or control group. Over a 12-day protocol, experimental subjects smoked ad libitum for 2 days, were abstinent for 4 days, resumed smoking for 2 days, and then chewed nicotine gum for the final 4 days. Control subjects smoked ad libitum throughout the entire protocol. Results indicated that changes in plasma beta-endorphin levels were not related to changes in the four smoking conditions. Plasma nicotine and mood states were related, such that dysphoric moods increased during abstinence from smoking in comparison to the control group. To investigate further the relationships between nicotine, beta-endorphin and reinforcement for smoking, it may be necessary to characterize endogenous opioid peptide release in the central nervous system during smoking. |
| 1884 | Davila, D G, Hurt, R D, Offord, K P, Harris, C D, Shepard, J W, Jr. Acute effects of transdermal nicotine on sleep architecture, snoring, and sleep-disordered breathing in nonsmokers. American journal of respiratory and critical care medicine. 1994;150:469.  **Abstract:** Previous research has suggested that nicotine may be therapeutically useful in the treatment of sleep-disordered breathing. The development of transdermal nicotine delivery systems has allowed us to test the overnight effectiveness of nicotine. Twenty nonsmoking subjects (10 men, 10 women) were recruited on the basis of a history of habitual snoring that was confirmed by overnight laboratory monitoring. Subjects were then randomized (double-blind crossover design) to receive either placebo or an active patch that delivers 11 mg of nicotine over a 24-h period. Patches were applied at 6 P.M. and removed at 6 A.M. the following morning, at which time venous blood was obtained for determination of serum nicotine concentrations. Polysomnography was performed using standard techniques to assess sleep architecture and sleep-disordered breathing. Snoring was monitored with a sound-level meter and quantitatively analyzed to determine the snoring index (SI) (number of snores per hour of sleep) and mean and maximum snoring intensities. The age of the subjects was 46.9 +/- 11.4 yr (mean +/- SD) and their mean body mass index (BMI) 33.3 +/- 4.6 kg/m2. A mean nicotine level was nondetectable with placebo and 7.8 +/- 2.3 ng/ml with wearing of an active patch. Nicotine decreased total sleep time (TST) by 33 min (p < or 0.01), sleep efficiency from 89.7 to 83.5% (p < or = 0.01), and percent rapid eye movement (REM) sleep from 18.8 to 15.1% (p < or = 0.01), and prolonged initial sleep latency (ISL) from 6.7 to 18.2 min (p < or = 0.01).(ABSTRACT TRUNCATED AT 250 WORDS) |
| 1885 | Richmond, R L, Harris, K, de Almeida Neto, A. The transdermal nicotine patch: results of a randomised placebo-controlled trial. The Medical journal of Australia. 1994;161:130.  **Abstract:** OBJECTIVESTo evaluate the efficacy of the transdermal nicotine patch as an aid to smoking cessation when used as an adjunct to a cognitive-behavioural group intervention and to assess the effectiveness of the patch in ameliorating withdrawal symptoms.DESIGN AND SETTINGDouble-blind placebo-controlled randomised trial with six months' follow-up of those who returned to the hospital-based outpatients smoking cessation clinic.SUBJECTSThree hundred and thirteen smokers recruited from the local community. Mean age was 42 years, 48% were male, mean cigarette consumption was 29 per day and mean duration of smoking was 24 years.INTERVENTIONCognitive-behavioural intervention delivered in a group context, two hours once a week over five weeks, self-help manual, and 24-hour daily nicotine patch treatment for 10 weeks.MAIN OUTCOME MEASURESPoint prevalence abstinence at three and six months, and sustained cessation from end of treatment to six months, with biochemical validation (expired carbon monoxide). We examined the effectiveness of the nicotine patch in ameliorating specific withdrawal symptoms by self-report of degree of severity.RESULTSThe active nicotine patch resulted in significantly higher biochemically confirmed abstinence rates when compared with placebo at three months (48% v. 21%) and at six months (33% v. 14%). Six-months' continuous abstinence rates were also significantly higher among the active nicotine group (25%) compared with placebo (12%). The most common adverse events among active patch users were sleep disturbance and local skin irritation. The nicotine patch reduced the severity of some withdrawal symptoms. A low level of dependence was the strongest predictor of three and six months' abstinence.CONCLUSIONSThe transdermal nicotine patch is effective when used as an adjunct to a group cognitive-behavioural intervention and it provides relief from withdrawal symptoms associated with nicotine dependence. |
| 1888 | . Randomised trial of nicotine patches in general practice: results at one year. Imperial Cancer Research Fund General Practice Research Group. BMJ (Clinical research ed.). 1994;308:1476.  **Abstract:** |
| 1889 | Fowler, Godfrey. Randomised trial of nicotine patches in general practice: Results at one year. British Medical Journal. 1994;308:1476.  **Abstract:** |
| 1890 | Spiga, R., Bennett, R.H., Schmitz, J., Cherek, D.R.. Effects of nicotine on cooperative responding among abstinent male smokers. Behavioural pharmacology. 1994;5:337.  **Abstract:** The effects of nicotine on human cooperative responding in abstinent male smokers were examined. During episodes occurring at random times through a session, concurrently available cooperative and independent responses were maintained by points exchangeable for money. Cooperative responses simultaneously added points to counters marked "Your Earnings" and "Other's Earnings" only if the subject's and another person's responses ostensibly coincided. Independent responses added points only to the counter marked "Your Earnings". After the first daily session abstinent subjects smoked ad libitum, received either 0, 2 or 4mg nicotine gum or abstained from smoking. Increases from this first session in time allocated to the cooperative response option, proportion of cooperative responses and cooperative response rate were significantly greater following ad libitum smoking or acute administration of 4mg nicotine. No effects of nicotine abstinence were observed on independent response rate. These results suggest effects on sociability may maintain nicotine use and increase relapse risk in abstinent smokers. |
| 1891 | Pickworth, W B, Bunker, E B, Henningfield, J E. Transdermal nicotine: reduction of smoking with minimal abuse liability. Psychopharmacology. 1994;115:9.  **Abstract:** Cigarette consumption as well as the physiologic, performance and subjective effects of the nicotine patch were evaluated in ten subjects who smoked ad libitum while residing on a residential research ward for 30 days. Nicotine transdermal systems ("patches") delivering a total of 0, 22 or 44 mg per 24 h were applied daily at a constant dose during each 7-day condition; the order of dosing conditions was varied according to a randomized, double-blind, crossover design. Nicotine patches significantly but modestly reduced spontaneous smoking and significantly increased venous plasma nicotine levels. Self ratings of patch liking, satisfaction with cigarettes and the ability to identify the patch condition did not change as a function of the nicotine dose, indicating minimal abuse liability. There were no consistent changes in the puffing pattern measures; however, in all patch conditions, subjects with extensive histories of illicit drug use smoked cigarettes faster than subjects with histories of occasional drug use. Small changes in resting heart rate, pulse and blood pressure occurred when the nicotine patch was worn. Thus large changes in venous plasma nicotine levels engender only modest changes in ad libitum cigarette consumption, measures of abuse liability and cardiovascular effects. These findings are consistent with the notion that the addictive and toxic effects of nicotine are partially determined by the rate of drug administration. |
| 1893 | Fortmann, Stephen P.. Nicotine replacement therapy for patients with coronary artery disease. Archives of Internal Medicine. 1994;154:989.  **Abstract:** Background: Because nicotine can cause adverse cardiovascular effects, we assessed the safety of transdermal nicotine for smoking cessation in patients with coronary artery disease. Methods: This 5-week, double-blind, randomized, placebo-controlled, multicenter study enrolled 156 patients with coronary artery disease who smoked at least one pack of cigarettes daily. Patients were randomized to receive either transdermal nicotine (14 mg/d) or transdermal placebo. After 1 week, patients who had smoked more than seven cigarettes were able to have their dose of blinded study medication increased to 21 mg/d of transdermal nicotine or corresponding placebo. All participants then continued to receive their chosen medication dose for an additional 4 weeks and attended weekly group counseling sessions. Adverse effects, cardiac events, electrocardiographic changes, frequency of angina, other cardiac symptoms, vital signs, and weight were monitored. Serial 24-hour ambulatory electrocardiographic monitoring was performed at one center. Smoking cessation rates were determined from diary reports of no smoking during the last 4 weeks of the study, corroborated weekly by expired breath carbon monoxide levels no higher than 8 ppm. Results: Eight of 79 patients randomized to receive placebo and three of 77 patients randomized to receive transdermal nicotine withdrew during the trial owing to adverse effects (P=.13), most of which were cardiovascular. Transdermal nicotine did not affect angina frequency, overall cardiac symptom status, nocturnal events, arrhythmias, or episodes of ischemic ST segment depression. Smoking cessation was achieved by 36% and 22% of patients receiving transdermal nicotine and placebo, respectively (P<.05). Conclusion: Transdermal nicotine was well tolerated by patients with stable coronary artery disease in this trial. |
| 1895 | Ockene, J K, Kristeller, J, Pbert, L, Hebert, J R, Luippold, R, Goldberg, R J, Landon, J, Kalan, K. The physician-delivered smoking intervention project: can short-term interventions produce long-term effects for a general outpatient population?. Health psychology : official journal of the Division of Health Psychology, American Psychological Association. 1994;13:278.  **Abstract:** Patterns of smoking cessation using 6- and 12-month follow-up data are reported for 1,261 primary care patients randomized to 3 physician-delivered smoking interventions: advice only (AO), counseling (CI), and counseling plus availability of nicotine-containing gum (CI + NCG). One-week-point-prevalence cessation rates at 12 months did not differ among the interventions: AO (15.2%), CI (12.9%) and CI + NCG (16.7%). However, maintained cessation rates (abstinent at both 6 and 12 months) increased with intervention intensity: AO (6.0%), CI (7.8%) and CI + NCG (10.0%): Test of trend chi 2 = 5.06, p = .02. CI + NCG was significantly higher than AO (p = .02). The findings support the following conclusions: Brief physician-delivered intervention with availability of nicotine-containing gum can have a beneficial long-term effect on smoking cessation, and cohort data as well as point-prevalence rates are important when assessing the long-term impact of lifestyle interventions. |
| 1896 | Pullan, R D, Rhodes, J, Ganesh, S, Mani, V, Morris, J S, Williams, G T, Newcombe, R G, Russell, M A, Feyerabend, C, Thomas, G A. Transdermal nicotine for active ulcerative colitis. The New England journal of medicine. 1994;330:811.  **Abstract:** BACKGROUNDUlcerative colitis is largely a disease of nonsmokers. Because anecdotal reports suggest that smoking and nicotine may improve the symptoms of the disease, we examined the effect of nicotine as a supplemental treatment for ulcerative colitis.METHODSWe treated 72 patients with active ulcerative colitis with either transdermal nicotine patches or placebo patches for six weeks in a randomized, double-blind study. Incremental doses of nicotine were given; most patients tolerated doses of 15 to 25 mg per 24 hours. All the patients had been taking mesalamine, and 12 were receiving low doses of glucocorticoids; these medications were continued without change during the study. Clinical, sigmoidoscopic, and histologic assessments were made at base line and at the end of the study; symptoms were recorded daily on a diary card, and the clinician made a global assessment. Side effects and plasma nicotine and cotinine concentrations were monitored throughout the study.RESULTSSeventeen of the 35 patients in the nicotine group had complete remissions, as compared with 9 of the 37 patients in the placebo group (P = 0.03). The patients in the nicotine group had greater improvement in the global clinical grade of colitis (P < 0.001) and the histologic grade (P = 0.03), lower stool frequency (a difference of 1.6 stools daily; P = 0.008), less abdominal pain (P = 0.05), and less fecal urgency (P = 0.009). More patients in the nicotine group had side effects (23, vs. 11 in the placebo group; P = 0.002), the most common of which were nausea, lightheadedness, headache, and sleep disturbance. Withdrawals due to ineffective therapy were more common in the placebo group (3 vs. 8, P = 0.12).CONCLUSIONSThe addition of transdermal nicotine to conventional maintenance therapy improves symptoms in patients with ulcerative colitis. |
| 1897 | Kenford, S L, Fiore, M C, Jorenby, D E, Smith, S S, Wetter, D, Baker, T B. Predicting smoking cessation. Who will quit with and without the nicotine patch. JAMA. 1994;271:589.  **Abstract:** OBJECTIVETo identify predictors of smoking cessation success or failure with and without transdermal nicotine patch treatment.DESIGNTwo independent randomized, double-blind, placebo-controlled studies using the nicotine patch assessing outcome at the end of treatment and at 6-month follow-up; each study used a different mode of adjuvant counseling.PATIENTSSubjects were daily smokers (> or = 15 cigarettes per day), aged 21 to 65 years with expired air carbon monoxide levels of at least 10 ppm, and motivated to quit. Eighty-eight subjects participated in study 1, and 112 subjects participated in study 2.INTERVENTIONStudy 1 consisted of 8 weeks of 22-mg nicotine patch therapy with intensive group counseling. Study 2 consisted of 4 weeks of 22-mg nicotine patch therapy and 2 weeks of 11-mg nicotine patch therapy with brief individual counseling.MAIN OUTCOME MEASURESThe prediction of smoking cessation (at end of treatment and after 6 months) based on pretreatment and intratreatment measures in smokers using active or placebo nicotine patches.RESULTSPretreatment markers, such as the Fagerstrom Tolerance Questionnaire score, number of cigarettes smoked per day, years smoked, expired air carbon monoxide level, or baseline blood nicotine and cotinine levels, showed no consistent relationship with successful smoking cessation across both studies. Of the intratreatment markers examined, withdrawal severity and nicotine replacement levels also were not consistently predictive of cessation success. However, any smoking during the second week of treatment was a consistent and powerful predictor of failure at the end of treatment and after 6 months. Among active nicotine patch patients who smoked at all during week 2 after quitting, 83% and 97% (studies 1 and 2, respectively) were smoking at 6-month follow-up. Conversely, abstinence during the second week of treatment predicted successful smoking cessation. Among active nicotine patch patients who were totally abstinent during week 2 after quitting, 46% and 41% (studies 1 and 2, respectively) were abstinent at 6-month follow-up. Of all nicotine patch patients in both studies who were smoking at 6-month follow-up, 74% began smoking during week 1 or 2. Among all placebo patch patients who were smoking at 6-month follow-up, 86% began smoking during week 1 or 2.CONCLUSIONSSmoking status (abstinent or smoking) during the first 2 weeks of nicotine patch therapy, particularly week 2, was highly correlated with clinical outcome and can serve as a powerful predictor of smoking cessation. Early smoking behavior also predicted outcome among placebo patch users. Traditional measures of dependence are not consistently predictive of cessation success. Clinicians are advised to emphasize the importance of total abstinence after a quit attempt and to follow-up with patients within the first 2 weeks of quitting; smoking during this critical time should be assessed and treatment may be altered as appropriate. |
| 1898 | Hurt, R D, Dale, L C, Fredrickson, P A, Caldwell, C C, Lee, G A, Offord, K P, Lauger, G G, Marŭsić, Z, Neese, L W, Lundberg, T G. Nicotine patch therapy for smoking cessation combined with physician advice and nurse follow-up. One-year outcome and percentage of nicotine replacement. JAMA. 1994;271:595.  **Abstract:** OBJECTIVETo determine the efficacy of a 22-mg nicotine patch combined with the National Cancer Institute program for physician advice and nurse follow-up in providing withdrawal symptom relief, 1-year smoking cessation outcome, and percentage of nicotine replacement.DESIGNRandomized, double-blind, placebo-controlled trial.SUBJECTSTwo-hundred forty healthy volunteers who were smoking at least 20 cigarettes per day.INTERVENTIONSBased on the National Cancer Institute program, subjects received smoking cessation advice from a physician. Follow-up and relapse prevention were provided by a study nurse during individual counseling sessions. Subjects were randomly assigned to 8 weeks of a 22-mg nicotine or placebo patch.MAIN OUTCOME MEASURESAbstinence from smoking was verified by expired air carbon monoxide levels. Withdrawal symptoms were recorded during patch therapy, and the percentage of nicotine replacement was calculated by dividing serum nicotine and cotinine levels at week 8 of patch therapy by levels obtained while smoking.RESULTSHigher smoking cessation rates were observed in the active nicotine patch group at 8 weeks (46.7% vs 20%) (P < .001) and at 1 year (27.5% vs 14.2%) (P = .011). Higher smoking cessation rates were also observed in subjects assigned to the active patch who had lower serum levels of nicotine and cotinine at baseline, and withdrawal symptom relief was better in the active patch group compared with placebo.CONCLUSIONSClinically significant smoking cessation can be achieved using nicotine patch therapy combined with physician intervention, nurse counseling, follow-up, and relapse prevention. Smokers with lower baseline nicotine and cotinine levels had better cessation rates, which provides indirect evidence that they had more adequate nicotine replacement with this fixed dose of transdermal nicotine than those smokers with higher baseline levels. |
| 1900 | Fiore, M C, Kenford, S L, Jorenby, D E, Wetter, D W, Smith, S S, Baker, T B. Two studies of the clinical effectiveness of the nicotine patch with different counseling treatments. Chest. 1994;105:524.  **Abstract:** OBJECTIVETo assess the effectiveness of transdermal nicotine therapy for smoking cessation and suppression of withdrawal severity in conjunction with two different adjuvant counseling treatments.DESIGNTwo independent randomized placebo-controlled double-blind trials.SETTINGSmoking cessation clinic.SUBJECTSEighty-eight (study 1) and 112 (study 2) adult volunteers motivated to quit smoking.INTERVENTIONSEight weeks of 22-mg transdermal nicotine therapy with group counseling (study 1); 4 weeks of 22 mg followed by 2 weeks of 11-mg transdermal nicotine therapy with brief individual counseling (study 2).MAIN OUTCOME MEASURESModified point prevalence (7 consecutive days of nonsmoking) at the end of patch treatment and 6 months after treatment initiation was assessed by self-report and biochemically confirmed; survival analyses were also conducted for both studies to compare treatment efficacy. Also, we examined the impact of the nicotine patch on specific withdrawal symptoms (anger, anxiety, awakening, difficulty concentrating, depression, hunger, impatience, and craving).RESULTSTransdermal nicotine treatment produced higher cessation rates at the end of treatment than did placebo with both adjuvant counseling interventions: 59 percent vs 40 percent (p < 0.05 in study 1) and 37 percent vs 20 percent (p < 0.05 in study 2), respectively. Smoking cessation efficacy was maintained 6 months after initiation of treatment: 34 percent vs 21 percent (p = 0.08 in study 1) and 18 percent vs 7 percent (p = 0.05 in study 2). Survival analyses also revealed significant group differences in efficacy in both studies. Nicotine patches also suppressed a variety of withdrawal symptoms, including craving in the first weeks after patients quit smoking.CONCLUSIONTransdermal nicotine effectively augments smoking cessation rates with two different types of counseling treatment. Overall, the nicotine patch approximately doubles the sustained rate of smoking cessation. Additionally, the nicotine patch provides relief from some tobacco withdrawal symptoms. |
| 1908 | Niaura, R, Goldstein, M G, Abrams, D B. Matching high- and low-dependence smokers to self-help treatment with or without nicotine replacement. Preventive medicine. 1994;23:70.  **Abstract:** BACKGROUNDThis study investigated the effect of matching smokers to nicotine replacement therapy (nicotine gum) on the basis of the pretreatment level of nicotine dependence.METHODSOne hundred seventy-three smokers, classified as high or low on nicotine dependence using the Fagerstrom Tolerance Questionnaire, were assigned at random to receive nicotine gum (2 mg), chewed ad libitum, or to not receive nicotine gum. All smokers participated concurrently in a 5-week treatment program consisting of four brief counseling sessions combined with a self-help manual provided by the American Lung Association.RESULTSResults showed that smokers with high levels of nicotine dependence were significantly more likely to quit smoking during treatment if they received nicotine gum (31.9%) than high-dependence smokers who did not receive the gum (12.2%). There was a tendency for smokers with low levels of nicotine dependence to do worse during treatment if they received gum (13.5% quit) than they did when they received no gum (20% quit), although this difference was not statistically significant. The relative differences in outcomes persisted after 1 year of follow-up, but the high overall rate of relapse (92.5%) rendered the absolute size of the differences statistically insignificant.CONCLUSIONThe results of the study are discussed in terms of strategies that might improve the success of treatment-matching approaches with newer forms of nicotine replacement therapies. |
| 1910 | Sachs, D P, Säwe, U, Leischow, S J. Effectiveness of a 16-hour transdermal nicotine patch in a medical practice setting, without intensive group counseling. Archives of internal medicine. 1993;153:1881.  **Abstract:** BACKGROUNDTo determine the effectiveness of a 16-hour transdermal nicotine patch in assisting smokers to stop smoking, when used in a primary medical practice model.METHODSA single-site, randomized, double-blind, out-patient, parallel-group, placebo-controlled trial consisting of 220 regular, otherwise healthy cigarette smokers. Patients participated in a 12-week patch treatment phase plus a 6-week tapering phase. A standard medical office model of physician intervention, such as could easily be employed by any primary care physician, without need for any special psychological services, training, or skills, was the behavioral intervention.RESULTSSustained abstinence, determined at each visit by absolutely no cigarette use, carbon monoxide level of 9 ppm or less, and serum cotinine level of 15 ng/mL or less (after week 18), was significantly greater for those patients receiving the active nicotine patch than for those receiving the placebo patch: the percent of patients not smoking at 6, 12, 18, 26, and 52 weeks was 61% vs 35%, 45% vs 26%, 41% vs 16%, 34% vs 12%, and 25% vs 9%, respectively (P < .001). This 16-hour nicotine patch produced no systemic side effects and minimal skin irritation.CONCLUSIONSNicotine replacement therapy via a 16-hour transdermal nicotine patch provided safe and effective treatment for tobacco-dependent patients. One-year sustained nonsmoking rates were nearly three times higher in the active than in the placebo condition, when the patch was used in an easily applicable standard medical practice setting, without the need for psychological interventions. This outcome was as good as or better than results achieved by nicotine patches using behavior modification or group counseling. |
| 1911 | Westman, E C, Levin, E D, Rose, J E. The nicotine patch in smoking cessation. A randomized trial with telephone counseling. Archives of internal medicine. 1993;153:1917.  **Abstract:** BACKGROUNDThis study was conducted to determine the efficacy of the nicotine patch in smoking cessation when combined with self-help materials, three brief visits, and telephone counseling.METHODSOne hundred fifty-nine healthy volunteers who smoked at least one pack of cigarettes per day and desired to quit smoking were enrolled in a double-blind trial with 6-week treatment and 6-month follow-up periods. After review of self-help materials, subjects were randomly assigned to regimens of nicotine or placebo patches. Subjects wore two patches per day for 4 weeks (25 mg of nicotine per 24 hours), then one patch per day for 2 weeks. Return visits were at the ends of weeks 4 and 6. Telephone counseling was given during weeks 1, 2, 3, and 5. Abstinence at 6 weeks was defined as zero cigarettes smoked for the previous 28 days, verified by exhaled carbon monoxide less than 8 ppm at 4 weeks and 6 weeks. Abstinence at 3 and 6 months was defined as self-report of zero cigarettes since the previous contact, verified by carbon monoxide value at 6 months.RESULTSAbstinence rates at 6 weeks, 3 months, and 6 months were 29.5%, 21.8%, and 20.5% in the active group, and 8.8%, 3.8%, and 2.5% in the placebo group (P < or = .001 for each comparison), respectively. Skin irritation was the main side effect, causing 1.3% to drop out.CONCLUSIONThe nicotine patch is efficacious in smoking cessation over a 6-month period, when combined with only self-help materials, three brief visits, and telephone counseling. |
| 1912 | Smits, P, Temme, L, Thien, T. The cardiovascular interaction between caffeine and nicotine in humans. Clinical pharmacology and therapeutics. 1993;54:194.  **Abstract:** In a placebo-controlled, double-blind randomized design, we investigated the cardiovascular interaction between caffeine (250 mg intravenously) and nicotine (4 mg chewing gum) in 10 healthy volunteers, both under baseline conditions and during physical and mental stress (standing up and mental arithmetic). Caffeine alone induced a significant increase in blood pressure associated with a decrease in heart rate, whereas nicotine alone increased both blood pressure and heart rate. The combination of caffeine and nicotine increased systolic and diastolic blood pressure by 10.8 +/- 2.0 and 12.4 +/- 1.9 mm Hg, respectively. This pressor response did not differ significantly from the calculated additive effects of caffeine and nicotine on blood pressure, measuring 12.9 +/- 2.0 and 14.2 +/- 2.1 mm Hg, respectively. Heart rate and forearm blood flow also showed a similar response when the combination of caffeine and nicotine was compared with the calculated sum. During physical stress (standing up), blood pressure, heart rate, and plasma catecholamines increased in the placebo test. The pressor response to standing up was less pronounced after the combination of caffeine and nicotine compared with the sum of the separate effects (combination versus sum: delta diastolic blood pressure, 24.7 +/- 1.9 versus 35.2 +/- 2.6 mm Hg [p < 0.01]; delta mean arterial pressure, 22.1 +/- 2.0 mm Hg versus 28.6 +/- 1.6 mm Hg [p < 0.05]). The plasma catecholamine response did not differ between the combination and the sum of both drugs. During mental arithmetic, blood pressure, heart rate, and forearm blood flow increased in the placebo test. The forearm vasodilator response to mental stress was attenuated by the combination of caffeine and nicotine compared with the sum of both drugs (combination versus sum: delta forearm blood flow, -0.1 +/- 0.3 versus 1.4 +/- 0.5 ml/100 ml/min [p < 0.05]). We conclude that the combined administration of caffeine and nicotine shows additive effects on cardiovascular parameters during baseline conditions but less than additive effects during sympathoadrenal stimulation. |
| 1913 | Turner, J A, McNicol, M W. The effect of nicotine and carbon monoxide on exercise performance in normal subjects. Respiratory medicine. 1993;87:427.  **Abstract:** In a partially blind randomized control study, nine healthy adult men performed progressive sub-maximal exercise tests, either after chewing nicotine chewing gum (4 mg) or breathing carbon monoxide (320 p.p.m.). Neither agent significantly affected exercise performance, despite a mean rise in blood nicotine to 17.0 ng ml-1 during nicotine administration and a COHb rise to 6.9% whilst breathing carbon monoxide. There was a significant rise in heart rate and blood pressure (P < 0.01) after nicotine administration. The reasons for absence of significant effect on cardiovascular or respiratory performance are discussed. |
| 1914 | . Effectiveness of a nicotine patch in helping people stop smoking: results of a randomised trial in general practice. Imperial Cancer Research Fund General Practice Research Group. BMJ (Clinical research ed.). 1993;306:1304.  **Abstract:** OBJECTIVETo assess the effectiveness of 12 weeks' treatment with a 24 hour transdermal nicotine patch in helping heavy smokers to stop smoking; also to assess the value of a specially written support booklet about smoking cessation and patch use compared with a simple advice pamphlet.DESIGNDouble blind placebo controlled randomised trial with a 2 x 2 factorial design.SETTING19 general practices in Oxfordshire.SUBJECTS1686 heavy smokers aged 25-64 (mean cigarette consumption 24/day; mean duration of smoking 25 years).MAIN OUTCOME MEASURESustained cessation for the last four weeks of the 12 week treatment period, confirmed by saliva cotinine estimation (226/262 cases; 86.3%) or expired carbon monoxide concentration (36/262; 13.7%). Patients lost to follow up (155/1686; 9%) were assumed to have continued to smoke.RESULTSCessation was confirmed in 163 patients (19.4%) using the nicotine patch and 99 patients (11.7%) using the placebo patch (difference 7.6% (95% confidence interval 4.2% to 11.1%); p < 0.0001). There was no significant advantage in using the more detailed written support material. The most important adverse effect of the patch was local skin irritation, which occurred in 15.8% (133/842) and 5.1% (43/844) of patients using the nicotine and placebo patches respectively, was graded as severe in 4.8% (40) and 1.1% (nine), and was stated as a reason for withdrawal from the trial in 9.5% (80) and 2.8% (24).CONCLUSIONNicotine patches are effective in a general practice setting with nursing support, but the extent to which this effect is sustained cannot be assessed until the results of longer term follow up are known. |
| 1915 | Russell, M A, Stapleton, J A, Feyerabend, C, Wiseman, S M, Gustavsson, G, Sawe, U, Connor, P. Targeting heavy smokers in general practice: randomised controlled trial of transdermal nicotine patches. BMJ (Clinical research ed.). 1993;306:1308.  **Abstract:** OBJECTIVES(a) To evaluate the efficacy of transdermal nicotine patches as an aid to stopping smoking when used as an adjunct to brief advice and support in a general practice setting; (b) to see whether an increase in nicotine patch dosage enhances the rate of initial cessation.DESIGNRandomised double blind placebo controlled parallel group study with one year of follow up.SETTING30 general practices in 15 English counties.SUBJECTS600 dependent heavy smokers (> or = 15 cigarettes daily) who were well motivated to give up.INTERVENTIONSBrief general practitioner advice, booklet, and 16 hours per day patch treatment for 18 weeks with brief support and follow up at one, three, six, 12, 26, and 52 weeks.MAIN OUTCOME MEASURESSelf reported complete abstinence for up to one year with biochemical validation at all follow up points.RESULTSNicotine patches reduced the severity of craving and adverse mood changes in the first weeks of withdrawal and doubled the rate of initial cessation at week 3 (nicotine group 36% of patients (144/400), placebo group 16.5% of patients (33/200)) and of continuous abstinence throughout one year (nicotine group 9.3% (37), placebo group 5.0% (10)). A dose increase at week 1 among patients experiencing difficulty in quitting increased the proportion who achieved abstinence at week 3. There were no adverse systemic effects attributable to nicotine, but the incidence of moderate or severe local irritation or itching at the patch site was 16.4% (63 patients), compared with 3.8% (seven) with placebo.CONCLUSIONTransdermal nicotine patches used as an adjunct to brief advice and support in a general practice setting are an effective aid to long term cessation of smoking in highly dependent smokers. |
| 1916 | Foulds, J, Stapleton, J, Hayward, M, Russell, M A, Feyerabend, C, Fleming, T, Costello, J. Transdermal nicotine patches with low-intensity support to aid smoking cessation in outpatients in a general hospital. A placebo-controlled trial. Archives of family medicine. 1993;2:417.  **Abstract:** OBJECTIVETo assess whether transdermal nicotine patches combined with low-intensity support can help outpatients in a general hospital stop smoking.DESIGNRandomized, double-blind, placebo-controlled trial with 12 weeks of follow-up.SETTINGDepartment of Thoracic Medicine in an inner-city public general hospital, London, England.SUBJECTSTwo hundred forty-eight outpatients in a general hospital, who smoked at least 10 cigarettes per day (the majority were being treated for smoking-related diseases), referred by clinicians at the hospital.INTERVENTIONBrief advice to stop smoking and daily application of transdermal nicotine patches (delivering 15 mg over 16 hours) or placebo, with follow-up appointments at 1, 3, 6, and 12 weeks, with a doubling of the dosage for continuing smokers at week 1.MAIN OUTCOME MEASURESustained abstinence from tobacco from week 3 to week 12 validated with measurement of expired-air carbon monoxide concentration at weeks 3, 6, and 12.RESULTSTwenty-nine (23.4%) of 124 subjects assigned to the nicotine group were validated as having abstained from smoking at both weeks 3 and 6, compared with 16 (12.9%) of 124 subjects receiving placebo (P = .008). At week 12, 22 (17.7%) of the subjects in the nicotine group were validated as having abstained at all three points as were 15 (12.1%) of the subjects in the placebo group (P = .058).CONCLUSIONTransdermal nicotine patches combined with low-intensity support are effective in helping outpatients in a general hospital stop smoking but do not prevent relapse after 6 weeks. |
| 1917 | Tønnesen, P, Nørregaard, J, Mikkelsen, K, Jørgensen, S, Nilsson, F. A double-blind trial of a nicotine inhaler for smoking cessation. JAMA. 1993;269:1268.  **Abstract:** OBJECTIVETo evaluate the efficacy of a new nicotine inhaler system for smoking cessation.DESIGNA 1-year, randomized, double-blind, placebo-controlled study.SETTINGMedical outpatient clinic with physicians experienced in smoking cessation assistance.SUBJECTSA total of 286 volunteers who smoked at least 10 cigarettes daily recruited through a local newspaper.INTERVENTIONSubjects were randomly allocated to nicotine inhalers (n = 145) or placebo (n = 141) to be used for 3 months followed by tapering for 3 months in the context of minimal levels of advice and support.MAIN OUTCOME MEASUREContinuous smoking abstinence at weeks 6, 12, 24, and 52, verified by measurements of carbon monoxide in expired air.RESULTSContinuous smoking abstinence was significantly higher for the active nicotine inhaler group compared with the placebo inhaler group. The respective success rates were 28% and 12% after 6 weeks, 21% and 9% after 12 weeks, 17% and 8% after 6 months, and 15% and 5% after 1 year (P = .02 to .001). The mean nicotine substitution based on cotinine determinations after 2 weeks was 43% (SD, 45%) of smoking levels. The treatment was well tolerated, and no serious adverse events were reported.CONCLUSIONSIn this setting the nicotine inhaler appeared safe to use and increased success rates of smoking cessation attempts. |
| 1918 | Hill, R.D., Rigdon, M., Johnson, S.. Behavioral smoking cessation treatment for older chronic smokers. Behavior Therapy. 1993;24:321.  **Abstract:** The benefits of behavioral smoking cessation treatment were examined in 82 chronic smokers 50 years of age and older. Participants were randomized into one of four intervention conditions: (1) behavioral training only, (2) behavioral training and nicotine gum, (3) behavioral training and physical exercise, and (4) physical exercise only. Behavioral training involved 3 months of group treatment, and physical exercise consisted of a 3-month walking program. Quit rates were assessed at program end and at 4, 7, and 12 months postrandomization. At 12 months the proportion quit across groups was respectively 31.8, 36.4, 27.8, and 10.0%. One-year quit data indicated that behavioral training facilitated cessation over the physical exercise only condition. |
| 1919 | Burke, P., Chivers, A., Clements, J., Dawes, M., Eastwood, I., Ebbs, D., Godlee, R., Harrington, R., Kearley, K., MacLennan, N., Mant, D., Murray, J., Nichols, M., Van Oss, H., Stern, D., Stevens, R., Thurston, D., Wilson, R., Wood, S.. Effectiveness of a nicotine patch in helping people stop smoking: Results of a randomised trial in general practice. British Medical Journal. 1993;306:1304.  **Abstract:** Objective - To assess the effectiveness of 12 weeks' treatment with a 24 hour transdermal nicotine patch in helping heavy smokers to stop smoking; also to assess the value of a specially written support booklet about smoking cessation and patch use compared with a simple advice pamphlet. Design - Double blind placebo controlled randomised trial with a 2 x 2 factorial design. Setting - 19 general practices in Oxfordshire. Subjects - 1686 heavy smokers aged 25-64 (mean cigarette consumption 24/day; mean duration of smoking 25 years). Main outcome measure - Sustained cessation for the last four weeks of the 12 week treatment period, confirmed by saliva cotinine estimation (226/262 cases; 86.3%) or expired carbon monoxide concentration (36/262; 13.7%). Patients lost to follow up (155/1686; 9%) were assumed to have continued to smoke. Results - Cessation was confirmed in 163 patients (19.4%) using the nicotine patch and 99 patients (11.7%) using the placebo patch (difference 7.6% (95% confidence interval 4.2% to 11.1%); p < 0.0001). There was no significant advantage in using the more detailed written support material. The most important adverse effect of the patch was local skin irritation, which occurred in 15.8% (133/842) and 5.1% (43/844) of patients using the nicotine and placebo patches respectively, was graded as severe in 4.8% (40) and 1.1% (nine), and was stated as a reason for withdrawal from the trial in 9.5% (80) and 2.8% (24). Conclusion - Nicotine patches are effective in a general practice setting with nursing support, but the extent to which this effect is sustained cannot be assessed until the results of long term follow up are known. |
| 1923 | Pirie, P L, McBride, C M, Hellerstedt, W, Jeffery, R W, Hatsukami, D, Allen, S, Lando, H. Smoking cessation in women concerned about weight. American journal of public health. 1992;82:1238.  **Abstract:** BACKGROUNDWeight gain after smoking cessation is often cited by women smokers as a primary reason for not attempting to quit smoking or for relapsing after a cessation attempt.METHODSA randomized trial of 417 women smokers was conducted to test the addition of two weight control strategies to a smoking cessation program. Participants received the standard smoking cessation program, the program plus nicotine gum, the program plus behavioral weight control, or the program plus both nicotine gum and behavioral weight control. Weight and smoking status were measured at the end of treatment and at 6 and 12 months posttreatment.RESULTSSmoking cessation rates were highest in the group receiving the smoking cessation program plus nicotine gum. Weight gain did not vary by treatment condition, so its effect on relapse could not be examined by group. There was no significant relationship between weight gained and relapse in individuals.CONCLUSIONSThe added behavioral weight control program was attractive to the participants and did not reduce smoking cessation rates. However, it did not produce the expected effect on weight, thereby restricting our ability to examine the effect of weight control on smoking cessation and relapse. |
| 1925 | Sutherland, G, Stapleton, J A, Russell, M A, Jarvis, M J, Hajek, P, Belcher, M, Feyerabend, C. Randomised controlled trial of nasal nicotine spray in smoking cessation. Lancet (London, England). 1992;340:324.  **Abstract:** Studies with nicotine chewing gum and nicotine skin patches indicate that nicotine replacement can help people to give up smoking. The rapidity with which nicotine is absorbed when given as a nasal spray suggests that it might be effective for those for whom the other means of replacement are too slow. The efficacy and safety of a nasal nicotine spray as an adjunct to group treatment for stopping smoking were assessed in a randomised, double-blind, placebo-controlled trial in which 227 cigarette smokers attending the Maudsley Hospital Smokers Clinic received 4 weeks of supportive group treatment plus active nicotine (0.5 mg per shot) or placebo nasal spray. The main end-point was biochemically validated complete abstinence from smoking from the third week of group treatment until the 12-month follow-up. Side-effects were assessed by self-reports and, where necessary, by physical examination. Of subjects assigned to active treatment 26% (n = 30) were validated abstinent throughout the year, compared with 10% (n = 11) of those assigned to placebo (relative abstinence rate 2.6, 95% CI 1.5-4.5, p less than 0.001). The advantage of the active spray was greatest in the heaviest smokers. Plasma nicotine concentrations from the spray were typically between one-half and three-quarters of baseline smoking levels. Tobacco-withdrawal symptoms, craving for cigarettes, and weight gain in abstinent subjects were reduced by the active spray. Minor irritant side-effects were frequent in both active and placebo sprays, but only 2 subjects had the spray discontinued as a result. No serious adverse effects were encountered. Nasal nicotine spray combined with supportive group treatment is an effective aid to smoking cessation. |
| 1930 | Hatsukami, D, Anton, D, Keenan, R, Callies, A. Smokeless tobacco abstinence effects and nicotine gum dose. Psychopharmacology. 1992;106:60.  **Abstract:** There were two experiments on abstinence from smokeless tobacco. The purpose of the first experiment was to determine abstinence effects from smokeless tobacco. The purpose of the second experiment was to examine the effects of different doses of nicotine gum on smokeless tobacco abstinence effects. The subjects were male Copenhagen smokeless tobacco users who underwent 3 days of baseline measurement while continuing to use smokeless tobacco ad libitum, and 5 days of the experimental condition. In the first experiment, the subjects were assigned randomly to one of two groups and compared: continuous smokeless tobacco users (n = 10), and deprivation plus no nicotine gum (n = 10). In the second experiment, subjects were assigned randomly and in a double-blind fashion to one of three groups and compared: (1) deprivation plus 0 mg nicotine gum (n = 20); (2) deprivation plus 2 mg nicotine gum (n = 20); and (3) deprivation plus 4 mg nicotine gum (n = 20). The first experiment showed significant increases upon abstinence for the following variables: (1) craving; (2) difficulty concentrating; (3) restlessness; (4) excessive hunger; (5) eating; (6) reaction time; (7) variability of reaction time and (8) total withdrawal scores for both the self-rated and the observer-rated forms. The second experiment showed that nicotine gum failed to significantly reduce smokeless tobacco abstinence effects, although those with high cotinine levels may receive some benefit from nicotine gum. |
| 1931 | Foulds, J, Stapleton, J, Feyerabend, C, Vesey, C, Jarvis, M, Russell, M A. Effect of transdermal nicotine patches on cigarette smoking: a double blind crossover study. Psychopharmacology. 1992;106:421.  **Abstract:** The effect of transdermal nicotine patches on ad libitum cigarette smoking was examined in 30 subjects by measuring behavioural, biochemical and subjective aspects of smoking during a week of smoking without patches, and then a week each of nicotine and placebo patches in a randomised double blind crossover design. While wearing nicotine patches the subjects did not reduce the number of cigarettes smoked, but their expired carbon monoxide was reduced by 14%, they obtained less satisfaction from their cigarettes, and reported fewer and weaker urges to smoke. Down-regulation of nicotine intake from cigarettes was imprecise, such that when subjects wore nicotine patches their post-cigarette plasma nicotine concentration increased to an average of 45 ng/ml compared with 37 ng/ml in both no patch and placebo patch conditions. As the nicotine patches produced a plasma nicotine concentration of 15.9 ng/ml in abstinent subjects, this suggests a 22% reduction in nicotine intake from cigarettes while wearing nicotine patches. No serious symptoms of nicotine overdose were reported. It is suggested that the continuous absorption of nicotine from the patch may cause a build-up of acute tolerance to both toxic and pleasant subjective effects from smoking. |
| 1932 | Clavel-Chapelon, F, Paoletti, C, Benhamou, S. A randomised 2 x 2 factorial design to evaluate different smoking cessation methods. Revue d'epidemiologie et de sante publique. 1992;40:187.  **Abstract:** A study with a 2 x 2 factorial design was performed to evaluate the effectiveness of acupuncture, of nicotine gum and the effect of the association on smoking cessation. After a one-year follow-up period, the success rates were in the same order of magnitude for nicotine gum (active treatment: 10%, placebo: 8%) group and for acupuncture (active treatment: 8%, placebo: 10%) group. |
| 1934 | Hughes, J R, Gust, S W, Keenan, R, Fenwick, J W, Skoog, K, Higgins, S T. Long-term use of nicotine vs placebo gum. Archives of internal medicine. 1991;151:1993.  **Abstract:** Medical patients (n = 315) who wished to quit smoking were randomly assigned in a double-blind manner to receive either nicotine or placebo gum. Subjects were advised to stop gum use by 4 months. Among abstinent smokers, 46% of those receiving nicotine gum and 17% of those receiving placebo gum used the gum beyond the recommended 4-month period. By 10 months after cessation 17% of quitters receiving nicotine gum and 6% receiving placebo gum were still using gum. Gradual reduction of nicotine gum did not result in withdrawal and cessation of nicotine gum did not increase the probability of relapse to smoking or weight gain. We conclude that use of nicotine gum is due, in part, to the effects of nicotine; however, long-term use is uncommon. |
| 1935 | Tønnesen, P, Nørregaard, J, Simonsen, K, Säwe, U. A double-blind trial of a 16-hour transdermal nicotine patch in smoking cessation. The New England journal of medicine. 1991;325:311.  **Abstract:** BACKGROUNDThe use of nicotine chewing gum combined with psychological support improves the success rate in quitting smoking. We studied the safety and efficacy of a transdermal nicotine patch in smoking cessation.METHODSWe conducted a double-blind randomized study comparing the effects of a 16-hour nicotine patch (15 +/- 3.5 mg of nicotine in 16 hours) with those of a placebo patch. Of the 289 smokers (207 women and 82 men) enrolled in the study, 145 were treated with nicotine patches and 144 with placebo patches for 16 weeks.RESULTSRates of sustained abstinence were significantly better with active treatment than with placebo: 53, 41, 24, and 17 percent of those in the nicotine-patch group were abstinent after 6, 12, 26, and 52 weeks, respectively, as compared with 17, 10, 5, and 4 percent of those in the placebo-patch group (P less than 0.0001). Only two subjects with the nicotine patch and one with the placebo patch had to withdraw from the study because of side effects.CONCLUSIONSThe nicotine skin patch proved to be safe and effective, as demonstrated by a higher rate of abstinence than with placebo. However, the absolute rate of abstinence after one year was only 17 percent, which is lower than the rate in studies that have combined the use of nicotine chewing gum with behavioral therapy. |
| 1936 | Segnan, N, Ponti, A, Battista, R N, Senore, C, Rosso, S, Shapiro, S H, Aimar, D. A randomized trial of smoking cessation interventions in general practice in Italy. Cancer causes & control : CCC. 1991;2:239.  **Abstract:** The purpose of this study was to examine the effectiveness of different practice-based approaches to assist patients of primary care physicians to quit smoking and sustain cessation. Forty-four nonsmoking general practitioners volunteered for the study. After a period of training, they randomized 923 smoking clients, unselected for motivation toward quitting, to four different intervention groups: (i) minimal intervention, consisting of one single counselling session and a brief handout on quitting techniques; (ii) repeated counselling including reinforcing sessions at Months 1, 3, 6, and 9; (iii) repeated counselling and use of nicotine gum; and (iv) repeated counselling and spirometry. Biochemically validated smoking status was assessed at six and 12 months after recruitment. The proportion of verified quitters at 12 months was 4.8 percent among subjects randomized to the minimal intervention group, compared to 5.5 percent, 7.5 percent, and 6.5 percent among those randomized to the three repeated-counselling groups. In no treatment group was the outcome significantly different from that for one-time counselling at the (P less than 0.05) level. Lack of power, contamination, and low attendance at reinforcing sessions should be taken into account in interpreting the results. |
| 1938 | Daughton, D M, Heatley, S A, Prendergast, J J, Causey, D, Knowles, M, Rolf, C N, Cheney, R A, Hatlelid, K, Thompson, A B, Rennard, S I. Effect of transdermal nicotine delivery as an adjunct to low-intervention smoking cessation therapy. A randomized, placebo-controlled, double-blind study. Archives of internal medicine. 1991;151:749.  **Abstract:** To assess the smoking cessation efficacy of transdermal nicotine patches as an adjunct to low-intervention therapy, we conducted a double-blind, placebo-controlled trial in 158 smokers. Participants were randomly assigned to one of the following three study regimens that required daily application of two 15-cm2 patches: (1) 24-hour nicotine delivery, (2) nicotine delivery during wakeful hours only, and (3) placebo. The impact of the three regimens on smoking cessation rates and tobacco withdrawal symptoms was examined. During the last 2 weeks of the trial, 39% of the 24-hour nicotine regimen delivery group, 35% of those on wakeful hour nicotine regimens, and 13.5% of the placebo treatment group achieved abstinence. Self-reported quit rates for the two nicotine patch-wearing regimens, as compared with that for the placebo group, continued to be significantly higher at 6 months. Moreover, compared with placebo, the transdermal nicotine patches significantly reduced tobacco withdrawal symptoms during the first few weeks of quitting. The differences in quit rates and tobacco withdrawal symptoms between the two active groups were not statistically significant. The patches were well tolerated both topically and systemically. We concluded that transdermal nicotine, when used as an adjunct to low-intervention therapy, significantly reduced tobacco withdrawal symptoms and enhanced smoking cessation rates. |
| 1940 | Hughes, J R, Gust, S W, Skoog, K, Keenan, R M, Fenwick, J W. Symptoms of tobacco withdrawal. A replication and extension. Archives of general psychiatry. 1991;48:52.  **Abstract:** Smokers (n = 315) who wished to quit were randomly assigned in a double-blind manner to groups using either nicotine or placebo gum. Self-reported and observed symptoms of tobacco withdrawal were collected before cessation and at follow-ups of 1 to 2 weeks, 1 month, and 6 months. Self-reported and/or observed anger, anxiety, craving, difficulty concentrating, hunger, impatience, and restlessness were the most prominent symptoms of tobacco withdrawal. These symptoms had returned to precessation levels by 1 month except increased weight, hunger, and craving continued for 6 months in many smokers. Nicotine gum decreased most symptoms, including craving and hunger but not weight. Abstinent smokers with more intense withdrawal were not more likely to relapse. Abstinent smokers who gained more weight were less likely to relapse. |
| 1941 | Ockene, J K, Kristeller, J, Goldberg, R, Amick, T L, Pekow, P S, Hosmer, D, Quirk, M, Kalan, K. Increasing the efficacy of physician-delivered smoking interventions: a randomized clinical trial. Journal of general internal medicine. 1991;6:1.  **Abstract:** OBJECTIVETo assess the relative impacts of three physician-delivered smoking interventions in combination with follow-up contact from behavioral counselors.DESIGNRandomized controlled trial with pre- post measures of smoking rates. This paper reports six-month outcome data.SETTINGParticipants were recruited from among patients seen by 196 medical and family practice residents in five primary care clinics.PARTICIPANTSParticipants were 1,286 patients out of 1,946 eligible smokers approached. The patient group was 57% female and 91% white, had an average age of 35 years, and smoked, on average, slightly over one pack per day.INTERVENTIONPhysicians were trained to provide each of three interventions: advice only, brief patient-centered counseling, and counseling plus prescription of nicotine-containing gum (Nicorette). Half the patients received follow-up in the form of telephone counseling at three-monthly intervals from behavioral counselors.MEASUREMENTS AND MAIN RESULTSChanges in smoking behaviors were assessed by telephone interview six months after physician intervention. The differences in one-week point prevalence cessation rates among the physician interventions were significant (p less than 0.01): advice only, 9.1%; counseling, 11.9%; counseling plus gum, 17.4%; with no effect for telephone counseling. The time elapsed from physician encounter to initial quitting and the length of that period of abstinence also showed significant benefit of the counseling interventions. Patients receiving physician counseling were much more likely than those not receiving counseling to rate their physician as very helpful (p less than 0.001). Multiple regression analyses are also reported.CONCLUSIONSmoking intervention counseling provided by physicians is well received by patients and significantly increases the likelihood of cessation at six months, an effect that is augmented by the prescription of nicotine-containing gum, when compared with physician-delivered advice. Follow-up telephone counseling does not contribute significantly to smoking behavior changes. |
| 1942 | Hatsukami, D, McBride, C, Pirie, P, Hellerstedt, W, Lando, H. Effects of nicotine gum on prevalence and severity of withdrawal in female cigarette smokers. Journal of substance abuse. 1991;3:427.  **Abstract:** This study examines the extent to which 2 mg nicotine gum reduces the prevalence and severity of cigarette withdrawal signs and symptoms. The sample was comprised of women who were randomly assigned to chew 2 mg nicotine gum (N = 206) or no nicotine gum (N = 211). Signs and symptoms of withdrawal were assessed at days 2, 7, 14, and 28 post-cessation. The results showed a significant effect of 2 mg nicotine compared to no gum at 2 days post-cessation on the prevalence of the following symptoms: anxious/tense, difficulty concentrating, restless, impatient, somatic symptoms, insomnia, increased eating, and drowsiness. There were additional differences between the two groups for the severity of craving for cigarettes, irritable/angry, excessive hunger, and total withdrawal score. Over the course of 28 days post-cessation, significant Group and/or Group x Time interaction effects were found with regard to the severity of signs and symptoms for the following variables: impatient, insomnia, increased eating, irritable, difficulty concentrating, restless, somatic complaints, and total withdrawal score. These results are similar to those obtained from placebo-controlled trials for nicotine gum. |
| 1943 | Jensen, E J, Schmidt, E, Pedersen, B, Dahl, R. Effect on smoking cessation of silver acetate, nicotine and ordinary chewing gum. Influence of smoking history. Psychopharmacology. 1991;104:470.  **Abstract:** In a randomized smoking cessation study 211, 203 and 82 persons were supported with nicotine, silver acetate and ordinary chewing gum, respectively. After 26 weeks there was no overall difference in number of abstainers between treatments. Participants were divided into subsets with low and high weighted packyears consumption (WPY) which modifies tobacco consumption by nicotine content. Abstainer rates in the total population controlled for treatment decreased with increasing WPY (P less than 0.005). In participants with low WPY abstainer rate was higher in the silver acetate group compared to the nicotine (P less than 0.0005) and ordinary (P less than 0.05) chewing gum groups. Nicotine chewing gum was more effective than silver acetate (P less than 0.05) and ordinary (P less than 0.05) chewing gum in smokers with high WPY. Ratings on some inconveniences experienced during earlier attempts to quit smoking influenced the ability to break the habit but had no influence on chewing gum effects. This study indicated that through consideration of smoking history it should be possible to individualize pharmacological support to smokers wanting to quit, with silver acetate chewing gum most effective for smokers with a low WPY and nicotine chewing gum most effective for smokers with a high WPY. |
| 1944 | Hatsukami, D K, Skoog, K, Huber, M, Hughes, J. Signs and symptoms from nicotine gum abstinence. Psychopharmacology. 1991;104:496.  **Abstract:** This study examined and compared withdrawal signs and symptoms from cessation of 0, 2 and 4 mg nicotine gum. In addition, a comparison was made between nicotine gum versus cigarette withdrawal symptoms. Smokers first underwent cigarette deprivation for 4 days and then were randomly assigned to 0 (N = 16), 2 (N = 25), and 4 (N = 21) mg gum. They were asked to chew the gum for 1 month and then to undergo a 4-day nicotine gum deprivation period. The results showed a number of significant changes occurring after deprivation from 4 mg gum, one change from 2 mg gum, and no changes from 0 mg gum. There were no significant differences in severity of withdrawal among the various doses of nicotine gum. There were more severe symptoms of withdrawal from cigarette as opposed to 4 mg nicotine gum deprivation. |
| 1945 | Hurt, R D, Lauger, G G, Offord, K P, Kottke, T E, Dale, L C. Nicotine-replacement therapy with use of a transdermal nicotine patch--a randomized double-blind placebo-controlled trial. Mayo Clinic proceedings. 1990;65:1529.  **Abstract:** The rate of smoking was significantly reduced in volunteer subjects by providing effective nicotine replacement, self-help material, and weekly visits with a nurse for 6 weeks. Nicotine-replacement therapy with a transdermal nicotine patch (Nicolan) almost doubled the 6-week smoking-cessation rate in comparison with that in a placebo group (77% versus 39%; P = 0.002) among subjects who were smoking at least 20 cigarettes per day at baseline. Although most subjects who used the active nicotine patches had skin reactions, the reactions were primarily mild. For use of both active and placebo patches, the level of patient compliance was high. Among subjects who continued to smoke, the use of cigarettes was decreased to less than 50% of the baseline smoking level in 7 of 7 with active nicotine patches and in 15 of 19 with placebo patches. Outcomes beyond 6 weeks showed a substantial relapse rate in both groups. Thus, when nicotine-replacement therapy is provided, a need exists for concurrent behavioral intervention and training for prevention of a relapse, neither of which was part of this protocol. |
| 1947 | Lashner, B A, Hanauer, S B, Silverstein, M D. Testing nicotine gum for ulcerative colitis patients. Experience with single-patient trials. Digestive diseases and sciences. 1990;35:827.  **Abstract:** Epidemiologic studies have documented an association between nonsmoking and ulcerative colitis and case reports have demonstrated that symptoms improve with smoking and worsen with removal of a nicotine source. A double-blind randomized crossover trial for individual ulcerative colitis patients (single-patient trial, or N of 1 clinical trial) was designed to study the safety, patient acceptance, and the effectiveness of nicotine gum in improving patient symptoms and proctoscopic appearance of involved colon. Seven nonsmoking patients chewed up to 10 squares/day (20 mg) of nicotine gum or placebo gum for two weeks. Therapy was crossed-over every two weeks over the eight-week trial. Effectiveness was judged from comparisons between nicotine-gum and placebo-gum periods of patient self-reported symptoms at the conclusion of each two-week period using visual analog scales and proctoscopic appearance using ordered categorical scales. Three of seven patients, all three of whom were former smokers, demonstrated sufficient improvement without adverse effects to warrant institution of nicotine gum into their drug treatment regimens. Three patients demonstrated an uncertain response, despite tolerating the drug, and have not had nicotine gum added to their regimens. One patient could not tolerate the medication and was withdrawn from the study. No serious side effects were noted. We conclude that a randomized trial for an individual patient is a useful method for evaluating treatment regimens for ulcerative colitis and that nicotine gum may be effective therapy for individual patients with ulcerative colitis who demonstrate an objective response with few adverse effects. |
| 1948 | Rose, J E, Levin, E D, Behm, F M, Adivi, C, Schur, C. Transdermal nicotine facilitates smoking cessation. Clinical pharmacology and therapeutics. 1990;47:323.  **Abstract:** The efficacy of a transdermal nicotine patch in facilitation of smoking cessation was evaluated in a randomized double-blind trial. Sixty-five smokers who were highly dependent on cigarettes participated in the study, which included a behavioral smoking-cessation program. The rates of continuous abstinence were significantly higher in the nicotine group both initially (55% versus 34%) and at 3 weeks (18% versus 6%). Certain smoking withdrawal symptoms, including negative affect and hypoarousal, were effectively relieved by the nicotine patch. There was a trend toward a reduction in cigarette craving, whereas hunger and habit withdrawal symptoms were not affected. The main side effect associated with the nicotine patch was skin irritation. These findings suggest that a nicotine skin patch may be a useful aid to smoking cessation; however, the combination of other techniques with nicotine replacement may provide a more effective treatment for symptoms such as craving for cigarettes. |
| 1949 | Hughes, J R, Gust, S W, Keenan, R M, Fenwick, J W. Effect of dose on nicotine's reinforcing, withdrawal-suppression and self-reported effects. The Journal of pharmacology and experimental therapeutics. 1990;252:1175.  **Abstract:** Seventy-eight smokers trying to quit were randomly assigned to 0-, 0.5-, 2- and 4-mg doses of nicotine gum and tracked for 9 months. In the 1st week of abstinence, self-administration of gum did not differ by dose (4 mg = 2 mg = 0.5 mg = 0 mg). Higher doses did not more effectively suppress withdrawal than lower doses nor induce more accurate nicotine identification (4 mg = 2 mg = 0.5 mg greater than 0 mg). Higher doses slightly increased ratings of adverse effects in the 1st week (4 mg greater than 2 mg greater than 0.5 mg greater than 0 mg). Over the next 6 months, the two higher doses appeared to support more gum self-administration than the two lower doses (4 mg = 2 mg greater than 0.5 mg = 0 mg) in the subsample of long-term quitters (n = 20). Our results suggest the dose-response curves differ for nicotine gum's reinforcing, withdrawal-suppression and self-reported effects. |
| 1950 | Killen, J D, Fortmann, S P, Newman, B, Varady, A. Evaluation of a treatment approach combining nicotine gum with self-guided behavioral treatments for smoking relapse prevention. Journal of consulting and clinical psychology. 1990;58:85.  **Abstract:** 1,218 smokers able to quit smoking for 48 hr were randomly assigned to one of 12 cells in a 4 x 3 fully crossed factorial experiment. A pharmacologic factor contained four levels: nicotine polacrilex (gum) delivered ad lib or on a fixed regimen, placebo gum, and no gum. A self-guided behavioral treatment factor contained three levels: self-selected relapse prevention modules, randomly administered modules, and no modules. Those receiving nicotine gum were more likely to be abstinent at the 2- and 6-month follow-ups. The fixed regimen accounted for most of the effect for gum. There was no effect for the relapse prevention module factor. Men and women showed a differential treatment response. Men who received nicotine gum were more likely to be abstinent at each follow-up (2, 6, and 12 months). No treatment was significantly better among women. We conclude that research on different gum chewing regimens is warranted and that further examination of possible gender differences in response to replacement therapy is needed. |
| 1951 | Smits, P, Eijsbouts, A, Thien, T. Nicotine enhances the circulatory effects of adenosine in human beings. Clinical pharmacology and therapeutics. 1989;46:272.  **Abstract:** A randomized, double-blind, and placebo-controlled study was performed in 10 healthy volunteers to evaluate a possible interaction between adenosine and nicotine in human beings. The infusion of adenosine alone (0.07 mg/kg/min) induced an increase in heart rate of 4.7 beats/min versus 0.2 beats/min after placebo administration (p less than 0.02). The infusion of adenosine alone induced a decrease in finger skin temperature compared with placebo administration (-1.0 degrees versus 0.0 degrees C, p less than 0.01). When compared with baseline values, nicotine gum chewing increased systolic and diastolic blood pressures by 6.2 and 7.0 mm Hg, respectively (p less than 0.001), heart rate by 5.5 beats/min (p less than 0.01), and plasma adrenaline levels by 0.03 nmol/L (p less than 0.025), whereas skin temperatures fell by 1.3 degrees C (p less than 0.001). The nicotine-induced increase in heart rate was larger during adenosine infusion than during placebo administration (14.9 versus 5.5 beats/min, p less than 0.001), whereas the increment of diastolic blood pressure was lower (1.1 versus 4.0 mm Hg, p less than 0.05). The increment in systolic blood pressure was not altered by concomitant adenosine infusion. The rise in plasma noradrenaline levels during the combined administration of nicotine and adenosine differed significantly from the response to nicotine alone (+ 0.23 versus -0.05 nmol/L, p less than 0.02). We concluded that, in human beings, the characteristic hemodynamic response to adenosine infusion is enhanced by nicotine. |
| 1953 | Hughes, J R, Gust, S W, Keenan, R M, Fenwick, J W, Healey, M L. Nicotine vs placebo gum in general medical practice. JAMA. 1989;261:1300.  **Abstract:** Three hundred fifteen smokers who attended a family practice clinic and wished to quit smoking were assigned in a random, double-blind manner to receive either nicotine (2 mg) or placebo gum. Smokers initially received brief advice from a physician and nurse, a slide presentation and written materials (29 to 35 minutes), and a single follow-up visit (12 to 20 minutes) one week after cessation. After corrections for marital status and income, 10% of those who received nicotine gum and 7% of those who received placebo gum reported continuous abstinence for 11 months and passed observer and biochemical verification (this difference was not statistically significant). We conclude that, when used in a nonselected group of smokers along with a brief intervention in a general medical practice, the pharmacologic effects of nicotine gum to increase cessation are either small or nonexistent. |
| 1954 | Gross, J, Stitzer, M L, Maldonado, J. Nicotine replacement: effects of postcessation weight gain. Journal of consulting and clinical psychology. 1989;57:87.  **Abstract:** The present study examined nicotine replacement effects on postcessation weight gain in smoking cessation clinic volunteers using objective indices of cigarette smoking, gum use, and body weight. After they achieved abstinence, subjects were randomly assigned to either active nicotine or placebo gum conditions for 10 weeks, during which smoking status was carefully monitored. Analyses revealed strong evidence for a gum effect on weight gain, with active gum users gaining a mean total of 3.8 lb compared with 7.8 lb for placebo gum users at the end of the 10-week trial. Evidence for a dose-response relation was found, suggesting that more gum use (greater than or equal to 6.5 pieces/day) resulted in greater weight suppression. Placebo gum subjects reported greater postcessation increases in eating and hunger compared with active gum subjects. The implications of the weight suppression effect of nicotine gum for smoking cessation treatments are discussed. |
| 1955 | Abelin, T, Buehler, A, Müller, P, Vesanen, K, Imhof, P R. Controlled trial of transdermal nicotine patch in tobacco withdrawal. Lancet (London, England). 1989;1:7.  **Abstract:** A transdermal nicotine patch, which delivers 0.7 mg/cm2 per 24 h and is available in sizes of 10, 20, and 30 cm2 was tested in subjects from 21 general medical practices in a 3-month, placebo-controlled randomised double-blind study. The nicotine group (n = 100) and the placebo group (n = 99) were similar at entry. Participants who smoked more than 20 cigarettes a day were treated with the 30 cm2 patch and the others with the 20 cm2 patch. When abstinence, defined as smoking 0-3 cigarettes per week and verified by CO measurement, was achieved, the next smallest patch was applied. After 1, 2, and 3 months of treatment 41, 36, and 36%, respectively, in the nicotine group were abstinent. The corresponding figures in the placebo group were 19, 20, and 23%. The differences were significant for all 3 months. Body weight did not increase in the nicotine group, but in the placebo group the mean increase was 4.4 kg. Craving and withdrawal symptoms decreased more with nicotine substitution for cigarettes. The patches were generally well tolerated, although 25% of subjects in the nicotine group and 13% in the placebo group had transient local erythema after application of the patch; 5 members of the nicotine group withdrew because of poor cutaneous tolerance. |
| 1956 | Buchkremer, G, Bents, H, Horstmann, M, Opitz, K, Tölle, R. Combination of behavioral smoking cessation with transdermal nicotine substitution. Addictive behaviors. 1989;14:229.  **Abstract:** Effects of a transdermal nicotine substitution on psychological smoking cessation were investigated in a double-blind prospective study. 131 smokers were randomly assigned to three treatment conditions: All smokers underwent nine weeks of self-controlled smoking cessation. During six weeks one group was additionally treated with nicotine patches continuously releasing nicotine through the skin into the blood circuit. The second group received placebo patches; while the third group was treated with behavioral training alone. Treatment effects were measured by daily cigarette consumption. Nicotine-treated subjects reached significantly higher abstinence rates during and at the end of treatment than both placebo- and control-subjects: 69% in the nicotine condition, 51.2% and 44.4% under placebo and control conditions respectively. Effects are influenced by initial cigarette consumption, with the light smokers benefitting most from the nicotine patch. Self-assessments suggest an aversive effect of transdermal nicotine on cigarette taste. Our results, although not yet verified by long-term observations, demonstrate that transdermal nicotine substitution significantly enhances the effectiveness of behavioral smoking cessation methods. |
| 1959 | Gilbert, J R, Wilson, D M, Best, J A, Taylor, D W, Lindsay, E A, Singer, J, Willms, D G. Smoking cessation in primary care. A randomized controlled trial of nicotine-bearing chewing gum. The Journal of family practice. 1989;28:49.  **Abstract:** The results are reported from a multicentered, randomized clinical trial of a physician-delivered smoking cessation intervention package. All physicians attended a four-hour training session during which the rationales for the different aspects of the intervention were discussed, including a detailed description of the proper use of nicotine-bearing chewing gum. Patients were randomized to receive an offer of a prescription of 2 mg of nicotine chewing gum in addition to the basic intervention (n = 111) or the basic intervention alone (n = 112). The basic intervention included advice, setting a date for quitting, self-help materials, and the offer of supportive follow-up visits. Receptionists were instructed to recruit the first two smokers attending the practice each day. One-year smoking cessation was validated by cotinine saliva analysis. The validated three-month sustained abstinence rates at one year were 8.1 percent and 9.8 percent in the gum and no-gum groups, respectively. The 95 percent confidence interval about this difference was -9.3 percent to 6.4 percent. There is no evidence from this study that the offer of 2 mg of nicotine-bearing gum enhances smoking cessation rates when added to a comprehensive intervention offered to all smokers in primary care. Until larger trials are completed, however, the possibility that this dose of nicotine gum may produce small beneficial effects cannot be excluded. |
| 1961 | Hughes, J R, Gulliver, S B, Amori, G, Mireault, G C, Fenwick, J F. Effect of instructions and nicotine on smoking cessation, withdrawal symptoms and self-administration of nicotine gum. Psychopharmacology. 1989;99:486.  **Abstract:** Seventy-seven smokers quit smoking and were randomly assigned to a 3 x 2 design contrasting instructions (told received nicotine gum versus told received placebo gum versus not told which gum received) and receipt of nicotine (received nicotine gum versus received placebo gum). Both being told one received nicotine and actual receipt of nicotine increased the number of days abstinent and decreased the number of cigarettes smoked (P less than 0.05). Receipt of nicotine but not instructions appeared to influence withdrawal (P = 0.06). Instructions but not receipt of nicotine appeared to influence craving (P = 0.08), gum self-administration (P = 0.06) and reported helpfulness of the gum (P = 0.02). Neither nicotine nor instructions influenced side-effects. Instructions and nicotine interacted in several ways. For example, nicotine appeared to increase abstinence in the blind and told placebo conditions more than in the told nicotine condition (P less than 0.05). Our results suggest the effects of instructions and nicotine 1) are not mutually exclusive, 2) vary across dependent variables and 3) can interact such that instructions modify the therapeutic and subjective effects of nicotine. |
| 1963 | Wilson, D M, Taylor, D W, Gilbert, J R, Best, J A, Lindsay, E A, Willms, D G, Singer, J. A randomized trial of a family physician intervention for smoking cessation. JAMA. 1988;260:1570.  **Abstract:** We assessed the impact of three conditions on one-year smoking cessation rates. Physicians in 70 community general practices were randomly allocated by practice to one of three groups: In the usual care group, smoking patients were to receive the care they normally would receive. In the gum only group, physicians were asked to speak to patients about smoking cessation and offer nicotine gum. In the gum plus group, physicians were trained in the experimental intervention. This intervention involved advice to stop smoking, the setting of a quit date, the offer of nicotine gum, and four follow-up visits. Smoking cessation was measured by self-report after one year and validated using saliva cotinine measures. Using a criterion of at least three months of abstinence, 8.8% of the patients of the trained physicians had stopped smoking at the one-year follow-up compared with 4.4% and 6.1% of the patients in the usual care and gum only groups, respectively. |
| 1964 | Fortmann, S P, Killen, J D, Telch, M J, Newman, B. Minimal contact treatment for smoking cessation. A placebo controlled trial of nicotine polacrilex and self-directed relapse prevention: initial results of the Stanford Stop Smoking Project. JAMA. 1988;260:1575.  **Abstract:** To determine the effectiveness of nicotine polacrilex combined with self-administered relapse prevention materials in maintaining smoking cessation, we conducted a randomized, double-blind, placebo controlled trial. Volunteers aged 18 to 65 years responding to media announcements were required to quit smoking for 48 hours without assistance. Of 1844 potential participants, 136 were medically excluded, 535 declined to make a quit attempt, and 573 were unable to quit, leaving 600 participants (35%) who were randomized. Eight self-help relapse prevention modules were mailed weekly. Gum was used either ad lib for smoking urges or on a fixed, hourly schedule (12 pieces per day). Only 15% of the subjects in each gum group stopped using the gum altogether because of side effects, but only 20% of the ad lib groups and 40% of the fixed-dosage group used at least eight pieces of gum per day during the first week. The abstinence rates (for at least seven days) at the six-month follow-up were 31% in both active gum groups and 22% in the placebo and no gum groups. Relapse rates in the two active gum groups were about half those in the placebo and no gum groups. Nicotine polacrilex may be a useful adjunct to minimal contact smoking cessation formats, which have broad appeal. Also, minimal contact relapse prevention programs may assist physicians in helping patients to maintain smoking cessation using nicotine polacrilex. |
| 1966 | Pickworth, W B, Herning, R I, Henningfield, J E. Mecamylamine reduces some EEG effects of nicotine chewing gum in humans. Pharmacology, biochemistry, and behavior. 1988;30:149.  **Abstract:** Spontaneous EEG was recorded in nine cigarette smokers who had been abstinent from tobacco for 12 hr. Subjects were treated with a capsule containing either centrally acting nicotine blocker, mecamylamine (10 mg), or placebo. At each of three 60-min intervals after the capsule was ingested, the subjects chewed two pieces of gum containing a total of 0, 4 or 8 mg of nicotine. Nicotine and mecamylamine dose combinations were randomized across subjects. Two three-minute periods of spontaneous EEG were recorded before the capsule and before and after gum chewing from bipolar electrode montages at the following positions: Cz-T5, Cz-T6, Cz-F7 and Cz-F8. During one period the subjects relaxed with eyes closed, in the other period they performed a math task with eyes open. When the drugs were given individually, mecamylamine decreased beta power and nicotine gum (4 and 8 mg) increased alpha frequency. Mecamylamine pretreatment prevented the increase in alpha frequency caused by the 4 mg gum dose but not the 8 mg dose. Alpha power was increased by the 8 mg gum dose and that increase was prevented by mecamylamine. Self-reported ratings of the "strength" of the gum were significantly diminished by mecamylamine pretreatment. The data are consistent with the results of earlier studies which indicate that the effects of tobacco administration and withdrawal are mediated by central actions of nicotine. |
| 1967 | Tønnesen, P, Fryd, V, Hansen, M, Helsted, J, Gunnersen, A B, Forchammer, H, Stockner, M. Effect of nicotine chewing gum in combination with group counseling on the cessation of smoking. The New England journal of medicine. 1988;318:15.  **Abstract:** We studied the effectiveness of chewing gum containing nicotine, in combination with group counseling, for subjects who were attempting to stop smoking. We used the Horn-Russell scale, based on a smoking questionnaire, to measure dependence on cigarettes; 173 smokers were grouped as highly dependent on nicotine or as having medium to low degrees of dependence. In a randomized double-blind study, the 60 highly dependent smokers were given gum containing 4 mg of nicotine (n = 27) or 2 mg of nicotine (n = 33), and the 113 smokers with medium or low dependence were given gum containing 2 mg of nicotine (n = 60) or a placebo gum (n = 53). All smokers took part in group counseling. In the highly dependent group, abstinence from cigarettes was chemically verified after six weeks, one year, and two years; 81.5 percent, 44.4 percent, and 33.3 percent of the subjects given gum containing 4 mg of nicotine were abstinent after those follow-up periods; the rates of abstinence were 54.5 percent, 12.1 percent, and 6.1 percent, respectively, for the subjects given gum containing 2 mg of nicotine. In the group with medium or low dependence, the rates of abstinence after the same periods were 73.3 percent, 38.3 percent, and 28.3 percent for the subjects given gum containing 2 mg of nicotine and 41.5 percent, 22.6 percent, and 9.4 percent for those given placebo gum. The differences in outcomes were significant at the 5 percent level for all comparisons, with the exception of the 2-mg nicotine gum versus the placebo gum at one year. This study indicates that the effectiveness of nicotine gum is not due to a placebo effect and that it is related to dose. The use of nicotine gum in appropriate doses should be helpful to persons who are attempting to stop smoking. |
| 1968 | Tønnesen, P, Fryd, V, Hansen, M, Helsted, J, Gunnersen, A B, Forchammer, H, Stockner, M. Two and four mg nicotine chewing gum and group counselling in smoking cessation: an open, randomized, controlled trial with a 22 month follow-up. Addictive behaviors. 1988;13:17.  **Abstract:** The aim of this study was to examine any dose response effect of 2 and 4 mg nicotine gum (Nicorette) as well as the effect of the treatment in relation to a control group. One hundred seventy-two cigarette smokers were randomly allocated to group counselling and 2 mg- (n = 62) or 4 mg- (n = 54) nicotine chewing gum or to an "advice only" control group (n = 56). Six group meetings in a three month period were guided by physicians in groups of 10-12 members. The 4 mg gum was replaced by 2 mg after 4 weeks. The overall chemical verified success rate was significantly higher for the treatment group compared with the control group. The success rate for the gum group was 45.6% after three months versus 7.1% for the control group, 33.3% versus 3.7% after 12 months, and 27.2% versus 5.7% after 22 months, respectively (p less than 0.001). We could not demonstrate any significant dose response effect of 2 and 4 mg nicotine gum. The low dependent smokers had the best outcome. The subgroup of self-reported chronic bronchitis subjects had a significantly lower success rate than their healthy counterparts (p less than 0.002). Neither the p-nicotine level after smoking or chewing gum nor the difference between these two could predict the outcome. The gum p-nicotine after a week was only 37.1% for 2 mg and 64.3% for 4 mg of the corresponding smoking p-nicotine levels. Nicotine compensation was best for the high dependent smokers. |
| 1970 | Harackiewicz, J M, Blair, L W, Sansone, C, Epstein, J A, Stuchell, R N. Nicotine gum and self-help manuals in smoking cessation: an evaluation in a medical context. Addictive behaviors. 1988;13:319.  **Abstract:** This study evaluated the effectiveness of nicotine chewing gum in smoking cessation, when incorporated into a behaviorally oriented self-help program. One hundred ninety-seven patients were randomly assigned to nicotine gum with a self-help manual, a self-help manual without gum, or a control condition, but received no further treatment from the prescribing physician. At six weeks, the nicotine gum group was superior to both the self-help and control conditions. By one year, many gum patients had relapsed, and the treatment effect was no longer significant. Patients who were able to quit initially were most likely to remain ex-smokers in the self-help condition. The clinical importance of these findings is discussed. |
| 1971 | Areechon, W, Punnotok, J. Smoking cessation through the use of nicotine chewing gum: a double-blind trial in Thailand. Clinical therapeutics. 1988;10:183.  **Abstract:** Two hundred cigarette smokers in Thailand were randomly assigned to receive a chewing gum containing nicotine or placebo. After six months, significantly more of the group receiving the active gum than those receiving the placebo had given up smoking. The results suggested that this treatment was more effective in highly dependent smokers than in those who were less dependent. Few side effects were reported. |
| 1972 | Michel Ch., Hasenfratz, M., Nil, R., Battig, K.. Cardiovascular, electrocortical, and behavioral effects of nicotine chewing gum. Klinische Wochenschrift. 1988;66:72.  **Abstract:** The cardiovascular, electrocortical, and behavioral effects of orally administered nicotine during rapid information processing were assessed in deprived female smokers. In a pre-post treatment design, 10 subjets received a 4-mg nicotine chewing gum and 10 subjects a placebo. The mental task required the subjects to watch single digits presented in a pseudo-random order on a screen and to press a button whenever the last three digits were either odd or even. The presentation rate decreased after each error and increased after each correct response and was used as the index of performance. Event-related brain potentials (ERP) to each of the three digits of the correctly answered triads were analyzed. The ERPs showed a distinct CNV potential for the second digit only (expectancy) and a P300 response for the third digit only (response decision). The mean EEG power spectrum was computed for each 5-min resting period, set before each trial and at the end of the session. A single administration of 4-mg nicotine chewing gum was followed by heart rate increase, acrodermal vasoconstriction, increase in theta and alpha frequency, decrease in delta power, and increase in the CNV magnitude. However, the chewing gum neither increased performance or reaction time nor decreased any ERP latencies or amplitudes, as has been reported after cigarette smoking. |
| 1974 | Clavel, F, Benhamou, S, Flamant, R. Nicotine dependence and secondary effects of smoking cessation. Journal of behavioral medicine. 1987;10:555.  **Abstract:** The secondary effects of smoking cessation among subjects included in a randomized trial, performed to test the efficacy of acupuncture and nicotine chewing gum vs. the control group, are reported according to nicotine dependence. After 1 month of cessation, ex-smokers had modified their nutritional intake, had gained weight, and felt more irritable compared to subjects still smoking. After 1 year, secondary effects concerned greater weight gain and irritability. The percentage of subjects who presented these secondary effects was more frequent among high-dependent than among low-dependent ex-smokers. |
| 1975 | Sutton, S, Hallett, R. Randomized trial of brief individual treatment for smoking using nicotine chewing gum in a workplace setting. American journal of public health. 1987;77:1210.  **Abstract:** In a controlled trial of brief treatment for smoking using nicotine chewing gum in a workplace setting, 270 of 334 cigarette smokers who expressed interest were invited to take part in the program, which consisted of two individual consultations; 172 attended. The remaining 64 smokers constituted a no-intervention control group. Using a criterion of sustained one-year abstinence with biochemical validation, success rates were 12 per cent among participants, 1 per cent among those who were invited but did not attend, and 2 per cent in the control group. |
| 1979 | Lindblad, A, Marsál, K. Influence of nicotine chewing gum on fetal blood flow. Journal of perinatal medicine. 1987;15:13.  **Abstract:** Several studies have shown that smoking is not beneficial to the fetus. Among the acute effects, influence on fetal circulation has been reported. Nicotine is considered to be one of the active agents in cigarette smoke. To test the nicotine effects on the human fetus, maternal and fetal cardiovascular dynamics were studied in 20 pregnant women when chewing a chewing gum containing 4 mg of nicotine and a chewing gum without nicotine given in a randomized double blind order. The fetal blood flow was measured with a method combining real-time ultrasonography and pulsed Doppler technique. Registrations were made in ten fetuses from the thoracic part of the descending aorta and in ten fetuses from the intra-abdominal part of the umbilical vein. In 15 of the fetuses registrations were also made from the umbilical artery. Concentrations of nicotine in plasma were analyzed in six women. The maternal plasma nicotine concentrations increased after the nicotine gum to a maximum value of 12.4 ng X ml-1 (median) at 25 min (figure 1). The 4 mg nicotine gum increased significantly maternal heart rate, systolic and diastolic blood pressure (figure 2). There was no influence on fetal heart rate or fetal blood flow, neither in the thoracic part of the descending aorta, nor in the intra-abdominal part of the umbilical vein (figure 3). There were no changes in the waveforms of the maximum blood velocity curves, neither of the aorta nor the umbilical artery. This indicates that there was no increased resistance in the placenta, neither after the nicotine gum nor the placebo gum.(ABSTRACT TRUNCATED AT 250 WORDS) |
| 1982 | Hughes, J R, Hatsukami, D K, Skoog, K P. Physical dependence on nicotine in gum. A placebo substitution trial. JAMA. 1986;255:3277.  **Abstract:** To test whether ex-smokers become physically dependent on nicotine in gum, we entered eight ex-smokers who were using nicotine gum into a randomized, double-blind, placebo-substitution trial. When placebo was substituted, seven of the eight subjects were observed to have withdrawal symptoms and two relapsed to smoking or nicotine gum. This result suggests that physical dependence (ie, withdrawal) may be a cause of behavioral dependence on nicotine gum (ie, use of gum beyond the recommended period) and physicians should emphasize the need for gradual reduction of nicotine gum. |
| 1983 | Nemeth-Coslett, R, Henningfield, J E. Effects of nicotine chewing gum on cigarette smoking and subjective and physiologic effects. Clinical pharmacology and therapeutics. 1986;39:625.  **Abstract:** Our study was conducted to assess the effects of multiple doses of nicotine chewing gum on a variety of measures of cigarette smoking, affect, and physiologic response. Cigarette smokers resided on a research unit for the duration of the study, during which time their smoking behavior was measured during nine 12-hour test sessions. At the start of each session and every 2 hours thereafter, subjects received oral doses of nicotine (2 or 4 mg) or placebo in the form of a chewing gum (nicotine polacrilex). Each dose of active drug and placebo was given for three sessions in a randomized block sequence. Total number of puffs per day was significantly decreased at both the 2 and 4 mg doses when compared with placebo, and the total number of cigarettes smoked per day was significantly decreased at the 4 mg dose. There were dose-related changes in certain subjective effects: Self-reported ratings of dose strength were directly related to dose, desire to smoke tended to be inversely related to dose, and prominent measures of abuse liability did not change. The only cardiovascular measure that was significantly changed by nicotine dose was systolic blood pressure, which showed an attenuation of the diurnal pattern as the dose increased. |
| 1984 | Russell, P O, Epstein, L H, Sittenfield, S L, Block, D R. The effects of nicotine chewing gum on the sensitivity to muscle tension. Psychopharmacology. 1986;89:230.  **Abstract:** It has been shown that smoking can alter the sensitivity to muscle activity in female smokers. The present study was designed to assess the effects of smoking cessation and nicotine replacement on sensitivity to muscle tension. Twenty-five women were randomly assigned to one of two groups. One group was given nicotine chewing gum during the withdrawal period and a second group was given no nicotine replacement. Results showed a significant difference in sensitivity at post-test for subjects given nicotine gum compared to subjects receiving no nicotine replacement. These results are consistent with the hypothesis that nicotine alters sensitivity to muscle tension. |
| 1985 | Clavel, F, Benhamou, S, Company-Huertas, A, Flamant, R. Helping people to stop smoking: randomised comparison of groups being treated with acupuncture and nicotine gum with control group. British medical journal (Clinical research ed.). 1985;291:1538.  **Abstract:** |
| 1986 | Christen, A G, Beiswanger, B B, Mallatt, M E, Tomich, C E, Drook, C A, McDonald, J L, Jr, Olson, B L, Stookey, G K. Effects of nicotine-containing chewing gum on oral soft and hard tissues: A clinical study. Oral surgery, oral medicine, and oral pathology. 1985;59:37.  **Abstract:** A double-blind clinical trial was conducted to determine whether the use of a chewing gum containing 2.0 mg nicotine (as an adjunct to a stop-smoking program) had any effects upon oral health. A total of 193 adults who smoked cigarettes volunteered with informed consent, were given routine dental prophylaxes, and were examined for the presence of plaque, stained pellicle, gingivitis, calculus, and general oral pathosis. The subjects were then randomly assigned to use either a nicotine-containing or a placebo chewing gum. After 15 weeks the subjects were recalled and re-examined. Smoking cessation was determined through questionnaire and analysis of the carbon monoxide content of alveolar air. At the completion of the study, 79 subjects had used the placebo gum and 78 had used the nicotine gum. Data analysis indicated that the nicotine chewing gum had no significant influence on any of the oral health parameters graded, as compared to the placebo gum. The continuation of smoking, however, was associated with significant increases in gingivitis and calculus rates. |
| 1987 | Hjalmarson, A I. Effect of nicotine chewing gum in smoking cessation. A randomized, placebo-controlled, double-blind study. JAMA. 1984;252:2835.  **Abstract:** The effect of 2-mg nicotine chewing gum as an adjunct to group therapy for smoking cessation was studied in a placebo-controlled, double-blind, randomized trial. After one year, 29% of the 106 subjects treated with nicotine chewing gum had remained abstinent throughout the year compared with 16% of the 99 subjects treated with placebo. The results were confirmed by measurement of levels of expired CO. More subjects in the nicotine group (70/94 v 45/93) reported that the gum reduced the craving for smoking. The adverse effects were few and not serious. In the nicotine group, 3% were still using the nicotine gum after two years. No subjects in the placebo group used the chewing gum beyond six months. Nicotine chewing gum is effective in improving the success rates in smoking cessation based on group therapy. |
| 1989 | Jamrozik, K, Fowler, G, Vessey, M, Wald, N. Placebo controlled trial of nicotine chewing gum in general practice. British medical journal (Clinical research ed.). 1984;289:794.  **Abstract:** Of 2110 adult cigarette smokers originally recruited to a study of the effect of antismoking advice in general practice, 429 who reported at follow up after one year that they had tried unsuccessfully to stop smoking were offered "a special antismoking chewing gum," either nicotine gum or a placebo gum, in a double blind study. Of 200 who were willing to try the gum, 101 were randomly allocated to the nicotine gum and 99 to the placebo gum. They were followed up at six months by an unannounced home visit, at which they were interviewed and asked to provide a breath sample for analysis of carbon monoxide. Twenty five claimed that they had stopped smoking, but, of them, seven exhaled levels of carbon monoxide indicative of continued smoking. Of the 18 in whom giving up smoking was validated, 10 had received active gum and eight placebo gum, a difference which was not significant (odds in favour of nicotine gum = 1.25, 95% confidence limits 0.47-3.31). The value of nicotine chewing gum, if any, can be quite small when it is used in general practice. |
| 1990 | Fagerström, K O. Effects of nicotine chewing gum and follow-up appointments in physician-based smoking cessation. Preventive medicine. 1984;13:517.  **Abstract:** The effect of different follow-up programs reinforced or not reinforced with the nicotine chewing gum Nicorette was tested by 13 physicians working in the open health care system. A total of 151 patients were advised to stop smoking, and were asked to participate in the program if judged sufficiently motivated by the physicians. After inclusion they were randomized into short or long follow-up, and nicotine gum vs no gum. The physicians conducted the follow-up therapy in their own personal way. Short follow-up was comprised of one appointment 14 days after cessation, while long follow-up consisted of a telephone call (1 week), an appointment (2 weeks), a second appointment (1 month), and a letter (3 months). The results at 12 months were that long follow-up showed a trend (P less than 0.12 toward being better than short follow-up, while nicotine gum was significantly better than no gum (P less than 0.05) in maintaining abstinence. The group with the best outcome was the one receiving long follow-up and nicotine gum, which yielded an expired air carbon monoxide-controlled, 12-month abstinence rate of 27%. The abstinence outcomes at 12 months for the other groups were short follow-up and nicotine gum, 22%; long follow-up and no gum, 15%; and short follow-up and no gum, 3%. The physicians' reactions to the smoking cessation treatment were largely positive. The nicotine gum seems to be a relatively simple, cost-effective, and practical tool for physicians to enhance and reinforce their antismoking advice. With a longer active follow-up period than was used here (1 month), even better results may be possible. |
| 1991 | Killen, J.D., Maccoby, N., Taylor, C.B.. Nicotine gum and self-regulation training in smoking relapse prevention. Behavior Therapy. 1984;15:234.  **Abstract:** Participants were assigned randomly to one of three maintenance treatment conditions: 1-nicotine gum (n = 22); 2-skills training (n = 20); 3-combined (skills training + nicotine gum) (n = 22). Prior to maintenance training, groups met on four consecutive days to receive aversion training and skills training. Those in Conditions 1 and 3 began using gum to manage withdrawal symptoms. During the maintenance phase, participants in Condition 1 attended a weekly 20 min drop-in clinic for 7 weeks to receive nicotine gum and complete assessments. Those in Conditions 2 and 3 received self-regulatory skills training. Abstinence rates at a 10.5 month follow-up were: Condition 1-23%; Condition 2-30%; Condition 3-50%. Expired air carbon monoxide (CO) and serum thiocyanate (SCN) levels verified participants' self-reports. Significant increases in perceived self-regulatory efficacy were observed from baseline to follow-up. Nicotine gum users reported less severe withdrawal symptoms than nonusers. |
| 1993 | Hughes, J R, Hatsukami, D K, Pickens, R W, Krahn, D, Malin, S, Luknic, A. Effect of nicotine on the tobacco withdrawal syndrome. Psychopharmacology. 1984;83:82.  **Abstract:** This study tested the ability of nicotine to alleviate the tobacco withdrawal syndrome. Signs and symptoms of tobacco withdrawal were measured in 100 smokers who fulfilled DSM-III criteria for tobacco dependence and a past history of tobacco withdrawal. After 2 evenings of baseline measurement, subjects were randomly assigned to receive either nicotine or placebo gum in a double blind manner. Subjects then stopped smoking, chewed gum freely, and returned on the 1st, 2nd, and 4th evenings of abstinence for further measurement. Nicotine reduced the increase in irritability, anxiety, difficulty concentrating, restlessness, impatience, and somatic complaints that subjects reported after cessation. Reductions in these withdrawal symptoms by nicotine were confirmed by ratings of significant others and by subjects' scores on the Profile of Mood States. Nicotine did not reduce the increases in cigarette craving, hunger, eating, insomnia, tremulousness , or supine heart rate after cessation. The effects of nicotine occurred immediately and persisted throughout the study. Although many subjects correctly identified their drug group, the efficacy of the gum was independent of subjects' identifications of drug. The relief of tobacco withdrawal by nicotine gum suggests that the tobacco withdrawal syndrome is caused, in part, by nicotine deprivation. |
| 1994 | Fagerström, K O. A comparison of psychological and pharmacological treatment in smoking cessation. Journal of behavioral medicine. 1982;5:343.  **Abstract:** A double-blind trial of a smoking-withdrawal chewing gum containing 2 mg nicotine was conducted with 100 consecutive patients in a smoking cessation clinic. All patients received the usual psychological treatment given at the clinic. In addition, the patients were randomly assigned to a nicotine gum (the experimental group) or a placebo chewing gum (control group). The abstinence rates for the experimental group at 1, 3, and 6 months after quitting were 90, 76, and 63%, respectively. The comparable abstinence rates for the control group were 60, 52, and 45%. The differences were significant at the 5% level at all three follow-up periods. When nicotine dependence, as measured by a standard questionnaire, was taken into consideration, it was found that 71% of the high-nicotine-dependent smokers in the experimental group were abstinent after 6 months, as compared to 39% in the placebo group. In contrast, low-nicotine-dependent patients achieved 75 and 65%, respectively, for the same time interval. The gum was well accepted by patients and gave no serious side effects. |
| 1996 | Schnoll, Robert A., Wileyto, E. Paul, Lerman, Caryn. Extended duration therapy with transdermal nicotine may attenuate weight gain following smoking cessation. Addictive Behaviors. 2012; 37(4):565.  **Abstract:** Aim: People who quit smoking often gain 11-12 lb, on average, which can frequently lead to a relapse to smoking. This study evaluated whether extended vs. standard duration treatment with nicotine patch helps those able to quit smoking to reduce cessation-induced weight gain and explored nicotine patch adherence as a mediator of treatment effects. Design and setting: We examined data from a completed randomized placebo-controlled clinical trial of extended (24 weeks) vs. standard (8 weeks plus 16 weeks of placebo) transdermal nicotine patch therapy. Changes in measured weight over 24 weeks were compared across the two treatment arms, controlling for gender, baseline smoking rate, and previous weight. Adherence to patch use was assessed using self-report of daily use over 24 weeks. Participants: 139 clinical trial participants who were confirmed to be abstinent at weeks 8 and 24. Findings: Compared to participants who received 8 weeks of nicotine patch therapy, participants who received 24 weeks of treatment showed significantly less weight gain from pre-treatment to week 24 (β = -4.76, 95% CI: -7.68 to -1.84, p = .002) and significantly less weight gain from week 8 to week 24 (β = -2.31, 95% CI: -4.39 to -0.23, p = .03). Extended treatment increased patch adherence which, in turn, reduced weight gain; patch adherence accounted for 20% of the effect of treatment arm on weight gain. Conclusion: Compared to 8 weeks of transdermal nicotine therapy, 24 weeks of patch treatment may help to reduce the weight gain that is typical among smokers who are able to achieve abstinence from tobacco use. Extended treatment increased nicotine patch adherence which, in turn, reduced weight gain. © 2011 Elsevier Ltd. All rights reserved. |

## C. Excluded Full-Text Articles (n=658)

| **Refid** | **Bibliography** | **Reason for Exclusion** |
| --- | --- | --- |
| 5 | Hsueh, Kuang-Chieh, Tang, Pei-Ling, McRobbie, Hayden. Effectiveness of Varenicline Versus Combination Nicotine Replacement Therapy for Smoking Cessation: One-Year Outcomes in a Smoking Cessation Clinic in Taiwan. Nicotine & tobacco research : official journal of the Society for Research on Nicotine and Tobacco. 2021;23:1094. | Non-randomized controlled or uncontrolled interventional studies. |
| 11 | Crouser, Elliott D, Smith, Rachel M, Culver, Daniel A, Julian, Mark W, Martin, Karen, Baran, Joanne, Diaz, Christopher, Erdal, Barbaros Selnur, Hade, Erinn M. A Pilot Randomized Trial of Transdermal Nicotine for Pulmonary Sarcoidosis. Chest. 2021; 203:A1329. | RCTs that did not provide data on the outcome of interest (i.e., arrhythmia, nonfatal myocardial infarction, nonfatal stroke, cardiovascular death) during the treatment period. |
| 14 | Vega, Jennifer N, Albert, Kimberly M, Mayer, Ingrid A, Taylor, Warren D, Newhouse, Paul A. Subjective cognition and mood in persistent chemotherapy-related cognitive impairment. Journal of cancer survivorship : research and practice. 2021. Epub ahead of print.. | RCTs that did not provide data on the outcome of interest (i.e., arrhythmia, nonfatal myocardial infarction, nonfatal stroke, cardiovascular death) during the treatment period. |
| 18 | Jackson, Christopher D, Carter, Jocelyn, Kansagara, Devan. E-Cigarettes Versus Nicotine Replacement Therapy for Smoking Cessation : Hajek P, Phillips-waller A, Przulj D, et al. A Randomized Trial of E-Cigarettes versus Nicotine-Replacement Therapy. N Engl J Med. 2019;380(7):629. Journal of general internal medicine. 2021;36:1481. | Letters to the editors, opinions, editorials, press releases, manufacturers’ advertisements, and other non-peer reviewed publications, unless the publication contained original data from RCTs. |
| 21 | Cook, Jessica W, Baker, Timothy B, Fiore, Michael C, Collins, Linda M, Piper, Megan E, Schlam, Tanya R, Bolt, Daniel M, Smith, Stevens S, Zwaga, Deejay, Jorenby, Douglas E, Mermelstein, Robin. Evaluating four motivation-phase intervention components for use with primary care patients unwilling to quit smoking: a randomized factorial experiment. Addiction (Abingdon, England). 2021. Epub ahead of print.. | RCTs that did not provide data on the outcome of interest (i.e., arrhythmia, nonfatal myocardial infarction, nonfatal stroke, cardiovascular death) during the treatment period. |
| 24 | Beard, Emma, Jackson, Sarah E, Anthenelli, Robert M, Benowitz, Neal L, Aubin, Lisa St, McRae, Thomas, Lawrence, David, Russ, Cristina, Krishen, Alok, Evins, A Eden, West, Robert. Estimation of risk of neuropsychiatric adverse events from varenicline,bupropion and nicotine patch versus placebo: secondary analysis of results from the EAGLES trial using Bayes factors. Addiction (Abingdon,England). 2021; 116(10):2816. | RCTs that did not provide data on the outcome of interest (i.e., arrhythmia, nonfatal myocardial infarction, nonfatal stroke, cardiovascular death) during the treatment period. |
| 27 | Kim, Nayoung, McCarthy, Danielle E, Piper, Megan E, Baker, Timothy B. Comparative effects of varenicline or combination nicotine replacement therapy versus patch monotherapy on candidate mediators of early abstinence in a smoking cessation attempt. Addiction (Abingdon, England). 2021;116:926. | RCTs that did not provide data on the outcome of interest (i.e., arrhythmia, nonfatal myocardial infarction, nonfatal stroke, cardiovascular death) during the treatment period. |
| 30 | Brett, Emma I, Chavarria, Jesus, Liu, Melissa, Hedeker, Donald, King, Andrea C. Effects of a brief motivational smoking intervention in non-treatment seeking disadvantaged Black smokers. Journal of consulting and clinical psychology. 2021;89:241. | RCTs that did not provide data on the outcome of interest (i.e., arrhythmia, nonfatal myocardial infarction, nonfatal stroke, cardiovascular death) during the treatment period. |
| 32 | Correa, John B, Lawrence, David, McKenna, Benjamin S, Gaznick, Natassia, Saccone, Phillip A, Dubrava, Sarah, Doran, Neal, Anthenelli, Robert M. Psychiatric co-morbidity and multi-morbidity in the EAGLES trial: Descriptive correlates and associations with neuropsychiatric adverse events, treatment adherence, and smoking cessation. Nicotine & tobacco research: official journal of the Society for Research on Nicotine and Tobacco. 2021; 23(10):1646. | RCTs that did not provide data on the outcome of interest (i.e., arrhythmia, nonfatal myocardial infarction, nonfatal stroke, cardiovascular death) during the treatment period. |
| 35 | Fernandes, Thiago P, Almeida, Natalia L, Silva, Gabriella M, Santos, Natanael A. Nicotine gum enhances visual processing in healthy nonsmokers. Brain imaging and behavior. 2021. Epub ahead of print. | RCTs that did not provide data on the outcome of interest (i.e., arrhythmia, nonfatal myocardial infarction, nonfatal stroke, cardiovascular death) during the treatment period. |
| 42 | Sahr, Michelle, Kelsh, Shelby, Blower, Noah, Sohn, Minji. Pilot Study of Electronic Nicotine Delivery Systems (ENDS) Cessation Methods. Pharmacy (Basel,Switzerland). 2021; 9(1):21. | RCTs that did not provide data on the outcome of interest (i.e., arrhythmia, nonfatal myocardial infarction, nonfatal stroke, cardiovascular death) during the treatment period. |
| 53 | Nollen, Nicole L, Ahluwalia, Jasjit S, Sanderson Cox, Lisa, Okuyemi, Kolawole, Lawrence, David, Samuels, Larry, Benowitz, Neal L. Assessment of Racial Differences in Pharmacotherapy Efficacy for Smoking Cessation: Secondary Analysis of the EAGLES Randomized Clinical Trial. JAMA network open. 2021;4:e2032053. | RCTs that did not provide data on the outcome of interest (i.e., arrhythmia, nonfatal myocardial infarction, nonfatal stroke, cardiovascular death) during the treatment period. |
| 54 | Smaily, Hussein, Khalaf, Michel, Melkane, Antoine E, Helou, Diane, Richa, Tony, Khoury, Clement, Azoury, Fares, Farha, Georges, Haddad, Amine, Matar, Nayla. Smoking cessation intervention for patients with head and neck cancer: A prospective randomized controlled trial. American journal of otolaryngology. 2021;42:102832. | RCTs that did not provide data on the outcome of interest (i.e., arrhythmia, nonfatal myocardial infarction, nonfatal stroke, cardiovascular death) during the treatment period. |
| 56 | Fernandes, Thiago P, Butler, Pamela D, Rodrigues, Stephanye J, Silva, Gabriella M, Anchieta, Marcos V, Souto, Jandirlly J S, Gomes, Giulliana H V, Almeida, Natalia L, Santos, Natanael A. Short-term effects of nicotine gum on facial detection in healthy nonsmokers: a pilot randomized controlled trial. Journal of addictive diseases. 2021;39:15. | RCTs that did not provide data on the outcome of interest (i.e., arrhythmia, nonfatal myocardial infarction, nonfatal stroke, cardiovascular death) during the treatment period. |
| 58 | Silvestri, Nathaniel J, Dahne, Jennifer, Wahlquist, Amy E, Toll, Benjamin, Carpenter, Matthew J. Does Medication Sampling Improve Compliance with Brief Advice? Results from a Pragmatic Randomized Clinical Trial. Journal of smoking cessation. 2021; 2021:6638872. | RCTs that did not provide data on the outcome of interest (i.e., arrhythmia, nonfatal myocardial infarction, nonfatal stroke, cardiovascular death) during the treatment period. |
| 59 | Evins, A Eden, West, Robert, Benowitz, Neal L, Russ, Cristina, Lawrence, David, McRae, Thomas, Maravic, Melissa Culhane, Heffner, Jaimee L, Anthenelli, Robert M. Efficacy and Safety of Pharmacotherapeutic Smoking Cessation Aids in Schizophrenia Spectrum Disorders: Subgroup Analysis of EAGLES. Psychiatric services (Washington, D.C.). 2021;72:7. | RCTs that did not provide data on the outcome of interest (i.e., arrhythmia, nonfatal myocardial infarction, nonfatal stroke, cardiovascular death) during the treatment period. |
| 67 | Goldenson, Nicholas I, Buchhalter, August R, Augustson, Erik M, Rubinstein, Mark L, Van Hoof, Dennis, Henningfield, Jack E. Abuse liability assessment of the JUUL system in two nicotine concentrations compared to combustible cigarette, nicotine gum and comparator electronic nicotine delivery system. Drug and alcohol dependence. 2020;217:108441. | RCTs that did not provide data on the outcome of interest (i.e., arrhythmia, nonfatal myocardial infarction, nonfatal stroke, cardiovascular death) during the treatment period. |
| 68 | McRobbie, Hayden J, Phillips-Waller, Anna, El Zerbi, Catherine, McNeill, Ann, Hajek, Peter, Pesola, Francesca, Balmford, James, Ferguson, Stuart G, Li, Lin, Lewis, Sarah, Courtney, Ryan J, Gartner, Coral, Bauld, Linda, Borland, Ron. Nicotine replacement treatment, e-cigarettes and an online behavioural intervention to reduce relapse in recent ex-smokers: a multinational four-arm RCT. Health technology assessment (Winchester, England). 2020;24:1. | RCTs that did not provide data on the outcome of interest (i.e., arrhythmia, nonfatal myocardial infarction, nonfatal stroke, cardiovascular death) during the treatment period. |
| 74 | Mahabee-Gittens, E Melinda, Ammerman, Robert T, Khoury, Jane C, Tabangin, Meredith E, Ding, Lili, Merianos, Ashley L, Stone, Lara, Gordon, Judith S. A Parental Smoking Cessation Intervention in the Pediatric Emergency Setting: A Randomized Trial. International journal of environmental research and public health. 2020; 17(21):8151. | RCTs that did not provide data on the outcome of interest (i.e., arrhythmia, nonfatal myocardial infarction, nonfatal stroke, cardiovascular death) during the treatment period. |
| 75 | Shiffman, Saul, Ferguson, Stuart G, Mao, Jason, Scholl, Sarah M, Hedeker, Donald, Tindle, Hilary A. Effectiveness of nicotine gum in preventing lapses in the face of temptation to smoke among non-daily smokers: a secondary analysis. Addiction (Abingdon, England). 2020;115:2123. | RCTs that did not provide data on the outcome of interest (i.e., arrhythmia, nonfatal myocardial infarction, nonfatal stroke, cardiovascular death) during the treatment period. |
| 78 | Landim, F S, Laureano Filho, J R, Nascimento, J, do Egito Vasconcelos, B C. Effectiveness of nicotine patch for the control of pain, oedema, and trismus following third molar surgery: a randomized clinical trial. International journal of oral and maxillofacial surgery. 2020;49:1508. | RCTs that did not provide data on the outcome of interest (i.e., arrhythmia, nonfatal myocardial infarction, nonfatal stroke, cardiovascular death) during the treatment period. |
| 94 | Cheung, Yee Tak Derek, Cheung Li, William Ho, Wang, Man Ping, Lam, Tai Hing. Delivery of a Nicotine Replacement Therapy Sample at Outdoor Smoking Hotspots for Promoting Quit Attempts: A Pilot Randomized Controlled Trial. Nicotine & tobacco research : official journal of the Society for Research on Nicotine and Tobacco. 2020;22:1468. | RCTs that did not provide data on the outcome of interest (i.e., arrhythmia, nonfatal myocardial infarction, nonfatal stroke, cardiovascular death) during the treatment period. |
| 96 | Kruse, Gina R, Park, Elyse R, Chang, Yuchiao, Haberer, Jessica E, Abroms, Lorien C, Shahid, Naysha N, Howard, Sydney, Haas, Jennifer S, Rigotti, Nancy A. Proactively Offered Text Messages and Mailed Nicotine Replacement Therapy for Smokers in Primary Care Practices: A Pilot Randomized Trial. Nicotine & tobacco research : official journal of the Society for Research on Nicotine and Tobacco. 2020;22:1509. | RCTs that did not provide data on the outcome of interest (i.e., arrhythmia, nonfatal myocardial infarction, nonfatal stroke, cardiovascular death) during the treatment period. |
| 103 | Nollen, Nicole L, Cox, Lisa Sanderson, Mayo, Matthew S, Ellerbeck, Edward F, Ahluwalia, Jasjit S. Counseling alone or in combination with nicotine replacement therapy for treatment of black non-daily smokers: a randomized trial. Addiction (Abingdon, England). 2020;115:1547. | RCTs that did not provide data on the outcome of interest (i.e., arrhythmia, nonfatal myocardial infarction, nonfatal stroke, cardiovascular death) during the treatment period. |
| 108 | Carpenter, Matthew J, Wahlquist, Amy E, Dahne, Jennifer, Gray, Kevin M, Garrett-Mayer, Elizabeth, Cummings, K Michael, Davis, Robert, Egan, Brent M. Nicotine replacement therapy sampling for smoking cessation within primary care: results from a pragmatic cluster randomized clinical trial. Addiction (Abingdon, England). 2020;115:1358. | RCTs that did not provide data on the outcome of interest (i.e., arrhythmia, nonfatal myocardial infarction, nonfatal stroke, cardiovascular death) during the treatment period. |
| 110 | Kaye, Jesse T, Johnson, Adrienne L, Baker, Timothy B, Piper, Megan E, Cook, Jessica W. Searching for Personalized Medicine for Binge Drinking Smokers: Smoking Cessation Using Varenicline, Nicotine Patch, or Combination Nicotine Replacement Therapy. Journal of studies on alcohol and drugs. 2020;81:426. | RCTs that did not provide data on the outcome of interest (i.e., arrhythmia, nonfatal myocardial infarction, nonfatal stroke, cardiovascular death) during the treatment period. |
| 122 | Wang, Kainan S, Zegel, Maya, Molokotos, Elena, Moran, Lauren V, Olson, David P, Pizzagalli, Diego A, Janes, Amy C. The acute effects of nicotine on corticostriatal responses to distinct phases of reward processing. Neuropsychopharmacology : official publication of the American College of Neuropsychopharmacology. 2020;45:1207. | RCTs that did not provide data on the outcome of interest (i.e., arrhythmia, nonfatal myocardial infarction, nonfatal stroke, cardiovascular death) during the treatment period. |
| 123 | Chai, Stephanie H, Leventhal, Adam M, Kirkpatrick, Matthew G, Eisenlohr-Moul, Tory A, Rapkin, Andrea J, D'Orazio, Lina, Pang, Raina D. Effectiveness of transdermal nicotine patch in premenopausal female smokers is moderated by within-subject severity of negative affect and physical symptoms. Psychopharmacology. 2020;237:1737. | RCTs that did not provide data on the outcome of interest (i.e., arrhythmia, nonfatal myocardial infarction, nonfatal stroke, cardiovascular death) during the treatment period. |
| 129 | Bernstein, Steven L., Dziura, James, Weiss, June, Harper-Brooks, Avis, Miller, Ted, Vickerman, Katrina, Grau, Lauretta E., Pantalon, Michael V., Abroms, Lorien, Collins, Linda, Toll, Benjamin. Successful tobacco dependence treatment in the emergency department: A randomized trial using the multiphase optimization strategy. Academic Emergency Medicine. 2020;27:S52. | RCTs that did not provide data on the outcome of interest (i.e., arrhythmia, nonfatal myocardial infarction, nonfatal stroke, cardiovascular death) during the treatment period. |
| 130 | Cunningham, John A, Kushnir, Vladyslav, Selby, Peter, Zawertailo, Laurie, Tyndale, Rachel F, Leatherdale, Scott T, Schell, Christina. Five-Year Follow-up of a Randomized Clinical Trial Testing Mailed Nicotine Patches to Promote Tobacco Cessation. JAMA internal medicine. 2020;180:792. | RCTs that did not provide data on the outcome of interest (i.e., arrhythmia, nonfatal myocardial infarction, nonfatal stroke, cardiovascular death) during the treatment period. |
| 131 | Zakiniaeiz, Yasmin, Liu, Heather, Gao, Hong, Najafzadeh, Soheila, Ropchan, Jim, Nabulsi, Nabeel, Huang, Yiyun, Cosgrove, Kelly, Morris, Evan. Nicotine patch reduces striatal smoking-induced dopamine release compared to placebo patch. Journal of Nuclear Medicine. 2020; 61(S1):1566. | RCTs that did not provide data on the outcome of interest (i.e., arrhythmia, nonfatal myocardial infarction, nonfatal stroke, cardiovascular death) during the treatment period. |
| 132 | Reid, Carol, Fenech, Mary, Jones, Lee, Salehi, Nasim. Nurse practitioner interventions for smokers with chronic hepatitis C. Journal of the American Association of Nurse Practitioners. 2020;32:380. | RCTs that did not provide data on the outcome of interest (i.e., arrhythmia, nonfatal myocardial infarction, nonfatal stroke, cardiovascular death) during the treatment period. |
| 143 | Nides, Mitchell, Danielsson, Tobias, Saunders, Frederick, Perfekt, Roland, Kapikian, Roxanne, Solla, Janice, Leischow, Scott J, Myers, Andrew. Efficacy and Safety of a Nicotine Mouth Spray for Smoking Cessation: A Randomized, Multicenter, Controlled Study in a Naturalistic Setting. Nicotine & tobacco research : official journal of the Society for Research on Nicotine and Tobacco. 2020;22:339. | RCTs that did not provide data on the outcome of interest (i.e., arrhythmia, nonfatal myocardial infarction, nonfatal stroke, cardiovascular death) during the treatment period. |
| 146 | Shiffman, Saul, Scholl, Sarah M, Mao, Jason, Ferguson, Stuart G, Hedeker, Donald, Primack, Brian, Tindle, Hilary A. Using Nicotine Gum to Assist Nondaily Smokers in Quitting: A Randomized Clinical Trial. Nicotine & tobacco research : official journal of the Society for Research on Nicotine and Tobacco. 2020;22:390. | RCTs that did not provide data on the outcome of interest (i.e., arrhythmia, nonfatal myocardial infarction, nonfatal stroke, cardiovascular death) during the treatment period. |
| 152 | Ayers, Catherine R, Heffner, Jaimee L, Russ, Cristina, Lawrence, David, McRae, Thomas, Evins, A Eden, Anthenelli, Robert M. Efficacy and safety of pharmacotherapies for smoking cessation in anxiety disorders: Subgroup analysis of the randomized, active- and placebo-controlled EAGLES trial. Depression and anxiety. 2020;37:247. | RCTs that did not provide data on the outcome of interest (i.e., arrhythmia, nonfatal myocardial infarction, nonfatal stroke, cardiovascular death) during the treatment period. |
| 156 | Pham, Carol Q, Kapolowicz, Michelle R, Metherate, Raju, Zeng, Fan-Gang. Nicotine enhances auditory processing in healthy and normal-hearing young adult nonsmokers. Psychopharmacology. 2020;237:833. | RCTs that did not provide data on the outcome of interest (i.e., arrhythmia, nonfatal myocardial infarction, nonfatal stroke, cardiovascular death) during the treatment period. |
| 158 | Webb, A R, Coward, L, Soh, L, Waugh, L, Parsons, L, Lynch, M, Stokan, L-A, Borland, R. Smoking cessation in elective surgical patients offered free nicotine patches at listing: a pilot study. Anaesthesia. 2020;75:171. | RCTs that did not include a nicotine versus no nicotine intervention. |
| 161 | Schlam, Tanya R, Baker, Timothy B, Smith, Stevens S, Cook, Jessica W, Piper, Megan E. Anxiety Sensitivity and Distress Tolerance in Smokers: Relations With Tobacco Dependence, Withdrawal, and Quitting Success†. Nicotine & tobacco research : official journal of the Society for Research on Nicotine and Tobacco. 2020;22:58. | RCTs that did not provide data on the outcome of interest (i.e., arrhythmia, nonfatal myocardial infarction, nonfatal stroke, cardiovascular death) during the treatment period. |
| 163 | Riley, Shannon, Anand, Anju, Stanbrook, Matthew. Twitter discussions from a respirology journal club: A randomized trial of e-cigarettes versus nicotine-replacement therapy. Canadian Journal of Respiratory, Critical Care, and Sleep Medicine. 2020;4:64. | Non-clinical, non-human studies, such as laboratory research, animal studies, or *in vitro* or *ex vivo* studies. |
| 166 | Nollen, Nicole L., Ahluwalia, Jasjit S., Sanderson Cox, Lisa, Okuyemi, Kolawole, Lawrence, David, Samuels, Larry, Benowitz, Neal L.. Assessment of Racial Differences in Pharmacotherapy Efficacy for Smoking Cessation: Secondary Analysis of the EAGLES Randomized Clinical Trial. JAMA Network Open. 2020; 4(1): e2032053. | RCTs that did not provide data on the outcome of interest (i.e., arrhythmia, nonfatal myocardial infarction, nonfatal stroke, cardiovascular death) during the treatment period. |
| 170 | Palmisano, Alexandra N, Astur, Robert S. Nicotine Facilitation of Conditioned Place Preference to Food Reward in Humans. Substance use & misuse. 2020;55:2156. | RCTs that did not provide data on the outcome of interest (i.e., arrhythmia, nonfatal myocardial infarction, nonfatal stroke, cardiovascular death) during the treatment period. |
| 174 | Zarghami, Mehran, Taghizadeh, Fatemeh, Sharifpour, Ali, Alipour, Abbas. Efficacy of guided self-change for smoking cessation in chronic obstructive pulmonary disease patients: A randomized controlled clinical trial. Tobacco induced diseases. 2019;17:90. | RCTs that did not provide data on the outcome of interest (i.e., arrhythmia, nonfatal myocardial infarction, nonfatal stroke, cardiovascular death) during the treatment period. |
| 175 | Thao, Viengneesee, Nyman, John A, Nelson, David B, Joseph, Anne M, Clothier, Barbara, Hammett, Patrick J, Fu, Steven S. Cost-effectiveness of population-level proactive tobacco cessation outreach among socio-economically disadvantaged smokers: evaluation of a randomized control trial. Addiction (Abingdon, England). 2019;114:2206. | RCTs that did not provide data on the outcome of interest (i.e., arrhythmia, nonfatal myocardial infarction, nonfatal stroke, cardiovascular death) during the treatment period. |
| 177 | Gilbert, David G, Rabinovich, Norka E, Gilbert-Matuskowitz, Elizabeth A, Klein, Keith P, Pergadia, Michele L. Smoking abstinence symptoms across 67 days compared with randomized controls-Moderation by nicotine replacement therapy, bupropion, and negative-affect traits. Experimental and clinical psychopharmacology. 2019;27:536. | RCTs that did not provide data on the outcome of interest (i.e., arrhythmia, nonfatal myocardial infarction, nonfatal stroke, cardiovascular death) during the treatment period. |
| 178 | Perry, Robin N, Schlagintweit, Hera E, Darredeau, Christine, Helmick, Carl, Newman, Aaron J, Good, Kimberley P, Barrett, Sean P. The impacts of actual and perceived nicotine administration on insula functional connectivity with the anterior cingulate cortex and nucleus accumbens. Journal of psychopharmacology (Oxford, England). 2019;33:1600. | RCTs that did not provide data on the outcome of interest (i.e., arrhythmia, nonfatal myocardial infarction, nonfatal stroke, cardiovascular death) during the treatment period. |
| 180 | Fischell, Sarah Aronson, Ross, Thomas, Salmeron, Betty Jo, Stein, Elliot. Transcranial direct current stimulation applied to the left dorsolateral prefrontal cortex in smokers modifies cognitive circuits implicated in the nicotine withdrawal syndrome. Neuropsychopharmacology. 2019;44:523. | Letters to the editors, opinions, editorials, press releases, manufacturers’ advertisements, and other non-peer reviewed publications, unless the publication contained original data from RCTs. |
| 190 | McCarthy, Danielle E, Versella, Mark V. Quitting Failure and Success With and Without Using Medication: Latent Classes of Abstinence and Adherence to Nicotine Monotherapy, Combination Therapy, and Varenicline. Nicotine & tobacco research : official journal of the Society for Research on Nicotine and Tobacco. 2019;21:1488. | RCTs that did not provide data on the outcome of interest (i.e., arrhythmia, nonfatal myocardial infarction, nonfatal stroke, cardiovascular death) during the treatment period. |
| 201 | Vega, Jennifer N, Albert, Kimberly M, Mayer, Ingrid A, Taylor, Warren D, Newhouse, Paul A. Nicotinic treatment of post-chemotherapy subjective cognitive impairment: a pilot study. Journal of cancer survivorship : research and practice. 2019;13:673. | RCTs that did not provide data on the outcome of interest (i.e., arrhythmia, nonfatal myocardial infarction, nonfatal stroke, cardiovascular death) during the treatment period. |
| 204 | Hahn, Britta, Shrieves, Megan E, Yuille, Marie B, Buchanan, Robert W, Wells, Ashleigh K. Nicotine effects on cognitive remediation training outcome in people with schizophrenia: A pilot study. Psychiatry research. 2019;280:112498. | RCTs that did not provide data on the outcome of interest (i.e., arrhythmia, nonfatal myocardial infarction, nonfatal stroke, cardiovascular death) during the treatment period. |
| 209 | Claire, Ravinder, Coleman, Tim, Leonardi-Bee, Jo, Berlin, Ivan. Saliva cotinine concentrations in pregnant women who smoke and use nicotine patches. Addiction (Abingdon, England). 2019;114:1651. | RCTs that did not provide data on the outcome of interest (i.e., arrhythmia, nonfatal myocardial infarction, nonfatal stroke, cardiovascular death) during the treatment period. |
| 214 | Dai, Siyu, Chan, Kate Ching Ching. Effectiveness of a smoking reduction intervention programme for smoking parents of paediatric patients in Hong Kong. European Respiratory Journal. 2019; 54(S63):2864. | RCTs that did not provide data on the outcome of interest (i.e., arrhythmia, nonfatal myocardial infarction, nonfatal stroke, cardiovascular death) during the treatment period. |
| 216 | Heffner, Jaimee L, Evins, A Eden, Russ, Cristina, Lawrence, David, Ayers, Catherine R, McRae, Thomas, Aubin, Lisa St, Krishen, Alok, West, Robert, Anthenelli, Robert M. Safety and efficacy of first-line smoking cessation pharmacotherapies in bipolar disorders: Subgroup analysis of a randomized clinical trial. Journal of affective disorders. 2019;256:267. | Duplicate articles or articles with the exact same study outcome data as another published article. |
| 223 | O'Reilly, Christian, Chapotot, Florian, Pittau, Francesca, Mella, Nathalie, Picard, Fabienne. Nicotine increases sleep spindle activity. Journal of sleep research. 2019;28:e12800. | RCTs that did not provide data on the outcome of interest (i.e., arrhythmia, nonfatal myocardial infarction, nonfatal stroke, cardiovascular death) during the treatment period. |
| 225 | Iyen, Barbara, Vaz, Luis R, Taggar, Jaspal, Cooper, Sue, Lewis, Sarah, Coleman, Tim. Is the apparently protective effect of maternal nicotine replacement therapy (NRT) used in pregnancy on infant development explained by smoking cessation?: secondary analyses of a randomised controlled trial. BMJ open. 2019;9:e024923. | RCTs that did not provide data on the outcome of interest (i.e., arrhythmia, nonfatal myocardial infarction, nonfatal stroke, cardiovascular death) during the treatment period. |
| 226 | Nakagata, Takashi, Fukao, Kosuke, Kobayashi, Hiroyuki, Katamoto, Shizuo, Naito, Hisashi. The Effects of Transdermal Nicotine Patches on the Cardiorespiratory and Lactate Responses During Exercise from Light to Moderate Intensity: Implications for Exercise Prescription during Smoking Cessation. Medicina (Kaunas,Lithuania). 2019; 55(7):348. | RCTs that did not provide data on the outcome of interest (i.e., arrhythmia, nonfatal myocardial infarction, nonfatal stroke, cardiovascular death) during the treatment period. |
| 231 | Anthenelli, Robert M, Gaffney, Michael, Benowitz, Neal L, West, Robert, McRae, Thomas, Russ, Cristina, Lawrence, David, St Aubin, Lisa, Krishen, Alok, Evins, A Eden. Predictors of Neuropsychiatric Adverse Events with Smoking Cessation Medications in the Randomized Controlled EAGLES Trial. Journal of general internal medicine. 2019;34:862. | RCTs that did not provide data on the outcome of interest (i.e., arrhythmia, nonfatal myocardial infarction, nonfatal stroke, cardiovascular death) during the treatment period. |
| 235 | Kaye, J.T., Piper, M.E., Baker, T.B., Cook, J.W.. Searching for personalized medicine for heavy drinking smokers: smoking cessation using varenicline, nicotine patch, or combination nicotine replacement therapy. Alcoholism: Clinical and Experimental Research. 2019;43:247A. | RCTs that did not provide data on the outcome of interest (i.e., arrhythmia, nonfatal myocardial infarction, nonfatal stroke, cardiovascular death) during the treatment period. |
| 243 | Hahn, Britta, McComas, Megan, Marie, Yuille, Robert, Buchanan, Ashleigh, Wells. Effects of Nicotine on Cognitive Remediation Training in Schizophrenia. Biological Psychiatry. 2019;85:S192. | RCTs that did not provide data on the outcome of interest (i.e., arrhythmia, nonfatal myocardial infarction, nonfatal stroke, cardiovascular death) during the treatment period. |
| 253 | Kruse, Gina R., Park, Elyse, Chang, Yuchiao, Haberer, Jessica, Abroms, Lorien, Shahid, Naysha N., Howard, Sydney, Haas, Jennifer, Rigotti, Nancy A.. A proactive text messaging intervention with medication adherence support tailored to primary care populations: A pilot randomized trial of getready2quit. Journal of General Internal Medicine. 2019;34:S114. | RCTs that did not provide data on the outcome of interest (i.e., arrhythmia, nonfatal myocardial infarction, nonfatal stroke, cardiovascular death) during the treatment period. |
| 258 | Engle, Jessica L, Mermelstein, Robin, Baker, Timothy B, Smith, Stevens S, Schlam, Tanya R, Piper, Megan E, Jorenby, Douglas E, Collins, Linda M, Cook, Jessica W. Effects of motivation phase intervention components on quit attempts in smokers unwilling to quit: A factorial experiment. Drug and alcohol dependence. 2019;197:149. | RCTs that did not provide data on the outcome of interest (i.e., arrhythmia, nonfatal myocardial infarction, nonfatal stroke, cardiovascular death) during the treatment period. |
| 259 | Mündel, Toby, Houltham, Stuart D, Barnes, Matthew J, Stannard, Stephen R. Nicotine Supplementation Does Not Influence Performance of a 1h Cycling Time-Trial in Trained Males. Frontiers in physiology. 2019;10:292. | RCTs that did not provide data on the outcome of interest (i.e., arrhythmia, nonfatal myocardial infarction, nonfatal stroke, cardiovascular death) during the treatment period. |
| 263 | Oncken, Cheryl, Dornelas, Ellen A, Kuo, Chia-Ling, Sankey, Heather Z, Kranzler, Henry R, Mead, Erin L, Thurlow, Ms Sheila D. Randomized Trial of Nicotine Inhaler for Pregnant Smokers. American journal of obstetrics & gynecology MFM. 2019;1:10. | RCTs that did not provide data on the outcome of interest (i.e., arrhythmia, nonfatal myocardial infarction, nonfatal stroke, cardiovascular death) during the treatment period. |
| 264 | Evins, A Eden, Benowitz, Neal L, West, Robert, Russ, Cristina, McRae, Thomas, Lawrence, David, Krishen, Alok, St Aubin, Lisa, Maravic, Melissa Culhane, Anthenelli, Robert M. Neuropsychiatric Safety and Efficacy of Varenicline, Bupropion, and Nicotine Patch in Smokers With Psychotic, Anxiety, and Mood Disorders in the EAGLES Trial. Journal of clinical psychopharmacology. 2019;39:108. | RCTs that did not provide data on the outcome of interest (i.e., arrhythmia, nonfatal myocardial infarction, nonfatal stroke, cardiovascular death) during the treatment period. |
| 265 | Robinson, Jason D, Li, Liang, Chen, Minxing, Lerman, Caryn, Tyndale, Rachel F, Schnoll, Robert A, Hawk, Larry W, George, Tony P, Benowitz, Neal L, Cinciripini, Paul M. Evaluating the temporal relationships between withdrawal symptoms and smoking relapse. Psychology of addictive behaviors : journal of the Society of Psychologists in Addictive Behaviors. 2019;33:105. | RCTs that did not provide data on the outcome of interest (i.e., arrhythmia, nonfatal myocardial infarction, nonfatal stroke, cardiovascular death) during the treatment period. |
| 267 | Aung, Myo Nyein, Yuasa, Motoyuki, Moolphate, Saiyud, Lorga, Thaworn, Yokokawa, Hirohide, Fukuda, Hiroshi, Kitajima, Tsutomu, Tanimura, Susumu, Hiratsuka, Yoshimune, Ono, Koichi, Thinuan, Payom, Minematsu, Kazuo, Deerojanawong, Jitladda, Suya, Yaoyanee, Marui, Eiji. Effectiveness of a new multi-component smoking cessation service package for patients with hypertension and diabetes in northern Thailand: a randomized controlled trial (ESCAPE study). Substance abuse treatment, prevention, and policy. 2019;14:10. | RCTs that did not provide data on the outcome of interest (i.e., arrhythmia, nonfatal myocardial infarction, nonfatal stroke, cardiovascular death) during the treatment period. |
| 272 | DiFrancisco-Donoghue, Joanne, Jung, Min-Kyung, Leder, Adena. Nicotine Gum as a Therapeutic Approach for Low Blood Pressure in Parkinson's Disease: A Randomized Pilot Study. Nicotine & tobacco research : official journal of the Society for Research on Nicotine and Tobacco. 2019;21:253. | RCTs that did not provide data on the outcome of interest (i.e., arrhythmia, nonfatal myocardial infarction, nonfatal stroke, cardiovascular death) during the treatment period. |
| 283 | Hajek, Peter, Lewis, Sarah, Munafo, Marcus, Lindson, Nicola, Coleman, Tim, Aveyard, Paul. Mediators of the effect of nicotine pre-treatment on quitting smoking. Addiction (Abingdon, England). 2018;113:2280. | RCTs that did not provide data on the outcome of interest (i.e., arrhythmia, nonfatal myocardial infarction, nonfatal stroke, cardiovascular death) during the treatment period. |
| 289 | Wells, Quinn S, Freiberg, Matthew S, Greevy, Robert A, Jr, Tyndale, Rachel F, Kundu, Suman, Duncan, Meredith S, King, Stephen, Abney, Lesa, Scoville, Elizabeth, Beaulieu, Dawn B, Gatskie, Vanessa, Tindle, Hilary A. Nicotine Metabolism-informed Care for Smoking Cessation: A Pilot Precision RCT. Nicotine & tobacco research : official journal of the Society for Research on Nicotine and Tobacco. 2018;20:1489. | RCTs that did not provide data on the outcome of interest (i.e., arrhythmia, nonfatal myocardial infarction, nonfatal stroke, cardiovascular death) during the treatment period. |
| 290 | Renwick, Charlotte, Wu, Qi, Breton, Magdalena Opazo, Thorley, Rebecca, Britton, John, Lewis, Sarah, Ratschen, Elena, Parrott, Steve. Cost-effectiveness of a complex intervention to reduce children's exposure to second-hand smoke in the home. BMC public health. 2018;18:1252. | RCTs that did not provide data on the outcome of interest (i.e., arrhythmia, nonfatal myocardial infarction, nonfatal stroke, cardiovascular death) during the treatment period. |
| 296 | Zhang, K.M., Clyde, M., Pipe, A., Reid, R., Els, C., Tulloch, H.E.. Do women and men differ in baseline smoking characteristics and quit rates folllowing treatment with smoking cessation medications? A secondary analysis of the flex study. Journal of Cardiopulmonary Rehabilitation and Prevention. 2018;38:E21. | RCTs that did not provide data on the outcome of interest (i.e., arrhythmia, nonfatal myocardial infarction, nonfatal stroke, cardiovascular death) during the treatment period. |
| 300 | Oertel, W., Müller, H., Schade-Brittinger, C., Kamp, C., Balthasar, K., Articus, K., Brinkman, M., Venuto, C., Unger, M., Eggert, K., Vadasz, D., Kieburtz, K., Boyd, J.. The NIC-PD-study-A randomized, placebo-controlled, double-blind, multi-centre trial to assess the disease-modifying potential of transdermal nicotine in early Parkinson's disease in Germany and N. America. Movement Disorders. 2018;33:S159. | RCTs that did not provide data on the outcome of interest (i.e., arrhythmia, nonfatal myocardial infarction, nonfatal stroke, cardiovascular death) during the treatment period. |
| 305 | Dahne, Jennifer, Wahlquist, Amy E, Boatright, Amy S, Garrett-Mayer, Elizabeth, Fleming, Douglas O, Davis, Robert, Egan, Brent, Carpenter, Matthew J. Nicotine replacement therapy sampling via primary care: Methods from a pragmatic cluster randomized clinical trial. Contemporary clinical trials. 2018;72:1. | RCTs that did not provide data on the outcome of interest (i.e., arrhythmia, nonfatal myocardial infarction, nonfatal stroke, cardiovascular death) during the treatment period. |
| 307 | Clyde, Matthew, Pipe, Andrew, Els, Charl, Reid, Robert, Fu, Angel, Clark, Alexa, Tulloch, Heather. Nicotine metabolite ratio and smoking outcomes using nicotine replacement therapy and varenicline among smokers with and without psychiatric illness. Journal of psychopharmacology (Oxford, England). 2018;32:979. | RCTs that did not provide data on the outcome of interest (i.e., arrhythmia, nonfatal myocardial infarction, nonfatal stroke, cardiovascular death) during the treatment period. |
| 308 | Liberman, Keliane, Van Schuerbeek, Peter, Herremans, Sarah, Meysman, Marc, De Mey, Johan, Buls, Nico. The effect of nicotine patches on craving in the brain: A functional MRI study on heavy smokers. Medicine. 2018;97:e12415. | RCTs that did not provide data on the outcome of interest (i.e., arrhythmia, nonfatal myocardial infarction, nonfatal stroke, cardiovascular death) during the treatment period. |
| 309 | Krishnan, Nandita, Gittelsohn, Joel, Ross, Alexandra, Elf, Jessica, Chon, Sandy, Niaura, Raymond, Martinson, Neil, Golub, Jonathan E. Qualitative Exploration of a Smoking Cessation Trial for People Living With HIV in South Africa. Nicotine & tobacco research : official journal of the Society for Research on Nicotine and Tobacco. 2018;20:1117. | RCTs that did not provide data on the outcome of interest (i.e., arrhythmia, nonfatal myocardial infarction, nonfatal stroke, cardiovascular death) during the treatment period. |
| 310 | Cooperman, Nina A, Lu, Shou-En, Richter, Kimber P, Bernstein, Steven L, Williams, Jill M. Pilot Study of a Tailored Smoking Cessation Intervention for Individuals in Treatment for Opioid Dependence. Nicotine & tobacco research : official journal of the Society for Research on Nicotine and Tobacco. 2018;20:1152. | RCTs that did not provide data on the outcome of interest (i.e., arrhythmia, nonfatal myocardial infarction, nonfatal stroke, cardiovascular death) during the treatment period. |
| 312 | West, Robert, Evins, A Eden, Benowitz, Neal L, Russ, Cristina, McRae, Thomas, Lawrence, David, St Aubin, Lisa, Krishen, Alok, Maravic, Melissa C, Anthenelli, Robert M. Factors associated with the efficacy of smoking cessation treatments and predictors of smoking abstinence in EAGLES. Addiction (Abingdon, England). 2018;113:1507. | RCTs that did not provide data on the outcome of interest (i.e., arrhythmia, nonfatal myocardial infarction, nonfatal stroke, cardiovascular death) during the treatment period. |
| 316 | Aveyard, Paul, Lindson, Nicola, Tearne, Sarah, Adams, Rachel, Ahmed, Khaled, Alekna, Rhona, Banting, Miriam, Healy, Mike, Khan, Shahnaz, Rai, Gurmail, Wood, Carmen, Anderson, Emma C, Ataya-Williams, Alia, Attwood, Angela, Easey, Kayleigh, Fluharty, Megan, Freuler, Therese, Hurse, Megan, Khouja, Jasmine, Lacey, Lindsey, Munafò, Marcus, Lycett, Deborah, McEwen, Andy, Coleman, Tim, Dickinson, Anne, Lewis, Sarah, Orton, Sophie, Perdue, Johanna, Randall, Clare, Anderson, Rebecca, Bisal, Natalie, Hajek, Peter, Homsey, Celine, McRobbie, Hayden J, Myers-Smith, Katherine, Phillips, Anna, Przulj, Dunja, Li, Jinshuo, Coyle, Doug, Coyle, Katherine, Pokhrel, Subhash. Nicotine preloading for smoking cessation: the Preloading RCT. Health technology assessment (Winchester, England). 2018;22:1. | Duplicate articles or articles with the exact same study outcome data as another published article. |
| 317 | Schuster, Randi Melissa, Pachas, Gladys N, Stoeckel, Luke, Cather, Corinne, Nadal, Mireya, Mischoulon, David, Schoenfeld, David A, Zhang, Haiyue, Ulysse, Christine, Dodds, Elisabeth B, Sobolewski, Sara, Hudziak, Vicenta, Hanly, Ailish, Fava, Maurizio, Evins, A Eden. Phase IIb Trial of an α7 Nicotinic Receptor Partial Agonist With and Without Nicotine Patch for Withdrawal-Associated Cognitive Deficits and Tobacco Abstinence. Journal of clinical psychopharmacology. 2018;38:307. | RCTs that did not provide data on the outcome of interest (i.e., arrhythmia, nonfatal myocardial infarction, nonfatal stroke, cardiovascular death) during the treatment period. |
| 318 | Dedert, Eric A, Dennis, Paul A, Calhoun, Patrick S, Dennis, Michelle F, Beckham, Jean C. A Randomized Clinical Trial of Nicotine Preloading for Smoking Cessation in People with Posttraumatic Stress Disorder. Journal of dual diagnosis. 2018;14:148. | RCTs that did not provide data on the outcome of interest (i.e., arrhythmia, nonfatal myocardial infarction, nonfatal stroke, cardiovascular death) during the treatment period. |
| 319 | Zarghami, Mehran, Taghizadeh, Fatemeh, Sharifpour, Ali, Alipour, Abbas. Efficacy of Smoking Cessation on Stress, Anxiety, and Depression in Smokers with Chronic Obstructive Pulmonary Disease: A Randomized Controlled Clinical Trial. Addiction & health. 2018;10:137. | RCTs that did not provide data on the outcome of interest (i.e., arrhythmia, nonfatal myocardial infarction, nonfatal stroke, cardiovascular death) during the treatment period. |
| 323 | Martins Filho, Euclides Dias, Vasconcelos, César Freire DE Melo, Oliveira, Fernando DE Santa Cruz, Pereira, Adriano DA Fonseca, Ferraz, Álvaro Antônio Bandeira. Evaluation of nicotine patch in pain control of patients undergoing laparoscopic cholecystectomy. Revista do Colegio Brasileiro de Cirurgioes. 2018;45:e1756. | RCTs that did not provide data on the outcome of interest (i.e., arrhythmia, nonfatal myocardial infarction, nonfatal stroke, cardiovascular death) during the treatment period. |
| 327 | Sharma, Surendra Kumar, Mohan, Alladi, Singh, Achintya Dinesh, Mishra, Hridesh, Jhanjee, Sonali, Pandey, Ravindra Mohan, Singh, Binit Kumar, Sharma, Rohini, Pallipamu, Prakash Babu, Pai, Madhukar, Dheda, Keertan. Impact of nicotine replacement therapy as an adjunct to anti-tuberculosis treatment and behaviour change counselling in newly diagnosed pulmonary tuberculosis patients: an open-label, randomised controlled trial. Scientific reports. 2018;8:8828. | RCTs that did not provide data on the outcome of interest (i.e., arrhythmia, nonfatal myocardial infarction, nonfatal stroke, cardiovascular death) during the treatment period. |
| 328 | Rasmussen, Scott, Horkan, Kathleen Halabuk, Kotler, Mitchell. Pharmacokinetic Evaluation of Two Nicotine Patches in Smokers. Clinical pharmacology in drug development. 2018;7:506. | RCTs that did not include a nicotine versus no nicotine intervention. |
| 332 | Christiansen, Bruce A, Carbin, Julianne, TerBeek, Erin, Fiore, Michael C. Helping Smokers with Severe Mental Illness Who Do Not Want to Quit. Substance use & misuse. 2018;53:949. | RCTs that did not provide data on the outcome of interest (i.e., arrhythmia, nonfatal myocardial infarction, nonfatal stroke, cardiovascular death) during the treatment period. |
| 339 | Park, Elyse R., Perez, Giselle Katiria, Regan, Susan, Muzikanksy, Alona, Rigotti, Nancy, Levy, Douglas E., Temel, Jennifer S., Cooley, Mary E., Partridge, Ann H., Pirl, William F., Irwin, Kelly, Friedman, Emily R., Borderud, Sarah, Hyland, Kelly, Rabin, Julia, Sprunck, Kim, Kwon, Diana, Ostroff, Jamie S.. Integrating tobacco treatment into cancer care: A first snapshot of RCT findings. Journal of Clinical Oncology. 2018; 36(S15):6505. | RCTs that did not provide data on the outcome of interest (i.e., arrhythmia, nonfatal myocardial infarction, nonfatal stroke, cardiovascular death) during the treatment period. |
| 340 | Vickerman, Katrina A, Keller, Paula A, Deprey, Mona, Lachter, Randi B, Jenssen, Jacalyn, Dreher, Marietta. Never Quit Trying: Reengaging Tobacco Users in Statewide Cessation Services. Journal of public health management and practice : JPHMP. 2018;24:e25. | RCTs that did not include a nicotine versus no nicotine intervention. |
| 341 | Hall, Sharon M, Humfleet, Gary L, Gasper, James J, Delucchi, Kevin L, Hersh, David F, Guydish, Joseph R. Cigarette Smoking Cessation Intervention for Buprenorphine Treatment Patients. Nicotine & tobacco research : official journal of the Society for Research on Nicotine and Tobacco. 2018;20:628. | RCTs that did not provide data on the outcome of interest (i.e., arrhythmia, nonfatal myocardial infarction, nonfatal stroke, cardiovascular death) during the treatment period. |
| 342 | Graham, Amanda L, Papandonatos, George D, Cha, Sarah, Erar, Bahar, Amato, Michael S. Improving Adherence to Smoking Cessation Treatment: Smoking Outcomes in a Web-based Randomized Trial. Annals of behavioral medicine : a publication of the Society of Behavioral Medicine. 2018;52:331. | RCTs that did not provide data on the outcome of interest (i.e., arrhythmia, nonfatal myocardial infarction, nonfatal stroke, cardiovascular death) during the treatment period. |
| 344 | Wang, Ying-Ying, Liu, Zhao, Wu, Yuan, Yang, Li, Guo, Lang-Tao, Zhang, Hao-Bin, Yang, Jin-Sheng. Efficacy of Acupuncture Is Noninferior to Nicotine Replacement Therapy for Tobacco Cessation: Results of a Prospective, Randomized, Active-Controlled Open-Label Trial. Chest. 2018;153:680. | RCTs that did not provide data on the outcome of interest (i.e., arrhythmia, nonfatal myocardial infarction, nonfatal stroke, cardiovascular death) during the treatment period. |
| 348 | Sukhija, Manpreet, Srivastava, Reena, Kaushik, Aditya. Pharmacokinetic characterization of three novel 4-mg nicotine lozenges . International journal of clinical pharmacology and therapeutics. 2018;56:113. | RCTs that did not include a nicotine versus no nicotine intervention. |
| 354 | Ratschen, Elena, Thorley, Rebecca, Jones, Laura, Opazo Breton, Magdalena, Cook, Juliette, McNeill, Ann, Britton, John, Coleman, Tim, Lewis, Sarah. A randomised controlled trial of a complex intervention to reduce children's exposure to secondhand smoke in the home. Tobacco control. 2018;27:155. | RCTs that did not provide data on the outcome of interest (i.e., arrhythmia, nonfatal myocardial infarction, nonfatal stroke, cardiovascular death) during the treatment period. |
| 368 | Kalkhoran, Sara, Inman, Elizabeth, Kulesa Kelley, Jennifer H., Ashburner, Jeffrey M., Rigotti, Nancy A.. Proactive population health strategy to offer tobacco dependence treatment to smokersin aprima-ry care practice network. Journal of General Internal Medicine. 2018;33:312. | RCTs that did not include a nicotine versus no nicotine intervention. |
| 372 | Cooperman, Nina A., Lu, Shou-En, Richter, Kimber P., Bernstein, Steven L., Williams, Jill M.. Pilot study of atailored smoking cessation intervention for individuals intreatment for opioid dependence. Nicotine and Tobacco Research. 2018;20:1152. | RCTs that did not provide data on the outcome of interest (i.e., arrhythmia, nonfatal myocardial infarction, nonfatal stroke, cardiovascular death) during the treatment period. |
| 373 | Noor, F., Koegelenberg, C.F.N., Esterhuizen, T.M., Irusen, E.M.. Predictors of treatment success in smoking cessation with varenicline combined with nicotine replacement therapy v. Varenicline alone. South African Medical Journal. 2018;108:45. | RCTs that did not provide data on the outcome of interest (i.e., arrhythmia, nonfatal myocardial infarction, nonfatal stroke, cardiovascular death) during the treatment period. |
| 374 | Aveyard, Paul. Effects on abstinence of nicotine patch treatment before quitting smoking: Parallel, two arm, pragmatic randomised trial: The preloading investigators. The BMJ. 2018; 361:k2164. | Duplicate articles or articles with the exact same study outcome data as another published article. |
| 377 | Murray, R.L., Thorley, R., Lewis, S., Breton, M. Opazo, Nyakutsikwa, B., Cheema, K., Ablewhite, J., Britton, J.. Systematic delivery of a smoking cessation intervention after discharge from a secondary care setting. Tobacco Induced Diseases. 2018;16:198. | RCTs that did not include a nicotine versus no nicotine intervention. |
| 381 | Mündel, Toby, Machal, Marine, Cochrane, Darryl J, Barnes, Matthew J. A Randomised, Placebo-Controlled, Crossover Study Investigating the Effects of Nicotine Gum on Strength, Power and Anaerobic Performance in Nicotine-Naïve, Active Males. Sports medicine - open. 2017;3:5. | RCTs that did not provide data on the outcome of interest (i.e., arrhythmia, nonfatal myocardial infarction, nonfatal stroke, cardiovascular death) during the treatment period. |
| 387 | Bekiroglu, Korkut, Russell, Michael A, Lagoa, Constantino M, Lanza, Stephanie T, Piper, Megan E. Evaluating the effect of smoking cessation treatment on a complex dynamical system. Drug and alcohol dependence. 2017;180:215. | RCTs that did not provide data on the outcome of interest (i.e., arrhythmia, nonfatal myocardial infarction, nonfatal stroke, cardiovascular death) during the treatment period. |
| 399 | Hansen, Eva Ø, Arendt-Nielsen, Lars, Boudreau, Shellie A. A Comparison of Oral Sensory Effects of Three TRPA1 Agonists in Young Adult Smokers and Non-smokers. Frontiers in physiology. 2017;8:663. | RCTs that did not provide data on the outcome of interest (i.e., arrhythmia, nonfatal myocardial infarction, nonfatal stroke, cardiovascular death) during the treatment period. |
| 403 | Schuppers, A., De Jong, B., Van Den Oever, H., Arbouw, M., Kruisdijk-Gerritsen, A., Van Zanten, A.. Nicotine replacement therapy in the intensive care unit: A randomized controlled pilot study. Intensive Care Medicine Experimental. 2017; 5(S2):0437. | RCTs that did not provide data on the outcome of interest (i.e., arrhythmia, nonfatal myocardial infarction, nonfatal stroke, cardiovascular death) during the treatment period. |
| 410 | Malaithong, Wanwipha, Munjupong, Sithapan. Efficacy of a transdermal nicotine patch in pain relief after arthroscopic shoulder surgery: A randomized controlled trial. Journal of the Medical Association of Thailand. 2017;100:901. | RCTs that did not provide data on the outcome of interest (i.e., arrhythmia, nonfatal myocardial infarction, nonfatal stroke, cardiovascular death) during the treatment period. |
| 411 | Schlagintweit, Hera E, Campbell, Niamh K, Barrett, Sean P. Quit Intentions Moderate Subjective and Physiological Responses to Acute Nicotine Replacement Therapy Administration in Dependent Smokers. Nicotine & tobacco research : official journal of the Society for Research on Nicotine and Tobacco. 2017;19:922. | RCTs that did not provide data on the outcome of interest (i.e., arrhythmia, nonfatal myocardial infarction, nonfatal stroke, cardiovascular death) during the treatment period. |
| 413 | Murphy, Cara M, MacKillop, James, Martin, Rosemarie A, Tidey, Jennifer W, Colby, Suzanne M, Rohsenow, Damaris J. Effects of varenicline versus transdermal nicotine replacement therapy on cigarette demand on quit day in individuals with substance use disorders. Psychopharmacology. 2017;234:2443. | RCTs that did not provide data on the outcome of interest (i.e., arrhythmia, nonfatal myocardial infarction, nonfatal stroke, cardiovascular death) during the treatment period. |
| 415 | Kushnir, Vladyslav, Sproule, Beth A, Cunningham, John A. Impact of large-scale distribution and subsequent use of free nicotine patches on primary care physician interaction. BMC public health. 2017;18:4. | RCTs that did not provide data on the outcome of interest (i.e., arrhythmia, nonfatal myocardial infarction, nonfatal stroke, cardiovascular death) during the treatment period. |
| 417 | Ikonomidis, Ignatios, Marinou, Margarita, Vlastos, Dimitrios, Kourea, Kallirhoe, Andreadou, Ioanna, Liarakos, Nikolaos, Triantafyllidi, Helen, Pavlidis, George, Tsougos, Elias, Parissis, John, Lekakis, John. Effects of varenicline and nicotine replacement therapy on arterial elasticity, endothelial glycocalyx and oxidative stress during a 3-month smoking cessation program. Atherosclerosis. 2017;262:123. | RCTs that did not provide data on the outcome of interest (i.e., arrhythmia, nonfatal myocardial infarction, nonfatal stroke, cardiovascular death) during the treatment period. |
| 419 | Lu, Wenying, Chappell, Kate, Walters, Julia A E, Jacobson, Glenn A, Patel, Rahul, Schüz, Natalie, Ferguson, Stuart G. The effect of varenicline and nicotine patch on smoking rate and satisfaction with smoking: an examination of the mechanism of action of two pre-quit pharmacotherapies. Psychopharmacology. 2017;234:1969. | RCTs that did not provide data on the outcome of interest (i.e., arrhythmia, nonfatal myocardial infarction, nonfatal stroke, cardiovascular death) during the treatment period. |
| 427 | Lesage, Elise, Aronson, Sarah E, Sutherland, Matthew T, Ross, Thomas J, Salmeron, Betty Jo, Stein, Elliot A. Neural Signatures of Cognitive Flexibility and Reward Sensitivity Following Nicotinic Receptor Stimulation in Dependent Smokers: A Randomized Trial. JAMA psychiatry. 2017;74:632. | RCTs that did not provide data on the outcome of interest (i.e., arrhythmia, nonfatal myocardial infarction, nonfatal stroke, cardiovascular death) during the treatment period. |
| 428 | Cook, Jessica W, Lanza, Stephanie T, Chu, Wanghuan, Baker, Timothy B, Piper, Megan E. Anhedonia: Its Dynamic Relations With Craving, Negative Affect, and Treatment During a Quit Smoking Attempt. Nicotine & tobacco research : official journal of the Society for Research on Nicotine and Tobacco. 2017;19:703. | RCTs that did not provide data on the outcome of interest (i.e., arrhythmia, nonfatal myocardial infarction, nonfatal stroke, cardiovascular death) during the treatment period. |
| 444 | Goodney, Philip P, Spangler, Emily L, Newhall, Karina, Brooke, Benjamin S, Schanzer, Andres, Tan, Tze-Woei, Beck, Adam W, Hallett, John H, MacKenzie, Todd A, Edelen, Maria O, Hoel, Andrew W, Rigotti, Nancy A, Farber, Alik. Feasibility and pilot efficacy of a brief smoking cessation intervention delivered by vascular surgeons in the Vascular Physician Offer and Report (VAPOR) Trial. Journal of vascular surgery. 2017;65:1152. | RCTs that did not provide data on the outcome of interest (i.e., arrhythmia, nonfatal myocardial infarction, nonfatal stroke, cardiovascular death) during the treatment period. |
| 449 | Graham, Amanda L, Papandonatos, George D, Cha, Sarah, Erar, Bahar, Amato, Michael S, Cobb, Nathan K, Niaura, Raymond S, Abrams, David B. Improving Adherence to Smoking Cessation Treatment: Intervention Effects in a Web-Based Randomized Trial. Nicotine & tobacco research : official journal of the Society for Research on Nicotine and Tobacco. 2017;19:324. | RCTs that did not provide data on the outcome of interest (i.e., arrhythmia, nonfatal myocardial infarction, nonfatal stroke, cardiovascular death) during the treatment period. |
| 451 | Ben Taleb, Ziyad, Ward, Kenneth D, Asfar, Taghrid, Jaber, Rana, Bahelah, Raed, Maziak, Wasim. Smoking Cessation and Changes in Body Mass Index: Findings From the First Randomized Cessation Trial in a Low-Income Country Setting. Nicotine & tobacco research : official journal of the Society for Research on Nicotine and Tobacco. 2017;19:351. | RCTs that did not provide data on the outcome of interest (i.e., arrhythmia, nonfatal myocardial infarction, nonfatal stroke, cardiovascular death) during the treatment period. |
| 458 | Piper, Megan E, Cook, Jessica W, Schlam, Tanya R, Smith, Stevens S, Bolt, Daniel M, Collins, Linda M, Mermelstein, Robin, Fiore, Michael C, Baker, Timothy B. Toward precision smoking cessation treatment II: Proximal effects of smoking cessation intervention components on putative mechanisms of action. Drug and alcohol dependence. 2017;171:50. | RCTs that did not provide data on the outcome of interest (i.e., arrhythmia, nonfatal myocardial infarction, nonfatal stroke, cardiovascular death) during the treatment period. |
| 460 | Mamey, Mary Rose, Burns, Leonard, Barbosa-Leiker, Celestina, Parks, Craig, McPherson, Sterling. Parallel growth modeling to better understand co-addiction: A randomized clinical trial of tobacco smoking and stimulant use. Drug and Alcohol Dependence. 2017;171:e127. | RCTs that did not provide data on the outcome of interest (i.e., arrhythmia, nonfatal myocardial infarction, nonfatal stroke, cardiovascular death) during the treatment period. |
| 471 | Brody, Arthur L, Zorick, Todd, Hubert, Robert, Hellemann, Gerhard S, Balali, Shabnam, Kawasaki, Sarah S, Garcia, Lizette Y, Enoki, Ryutaro, Abraham, Paul, Young, Paulina, McCreary, Charles. Combination Extended Smoking Cessation Treatment Plus Home Visits for Smokers With Schizophrenia: A Randomized Controlled Trial. Nicotine & tobacco research : official journal of the Society for Research on Nicotine and Tobacco. 2017;19:68. | RCTs that did not include a nicotine versus no nicotine intervention. |
| 474 | Johns, D.. Randomised controlled trial comparing nicotine replacement therapy (nrt) and counselling on smoking cessation in patients prone to lung cancer using bed font micro-smokerlyzer. Supportive Care in Cancer. 2017;25:S102. | RCTs that did not provide data on the outcome of interest (i.e., arrhythmia, nonfatal myocardial infarction, nonfatal stroke, cardiovascular death) during the treatment period. |
| 476 | Rozario, H.P.. Nicotine dependence assessment using Fagerstrom test and Nicotine Replacement therapy (NRT) recommendation techniques for smoking cessation among Paniya tribes. Annals of Oncology. 2016;27:ix187. | RCTs that did not provide data on the outcome of interest (i.e., arrhythmia, nonfatal myocardial infarction, nonfatal stroke, cardiovascular death) during the treatment period. |
| 478 | Bernstein, Steven L, Weiss, June-Marie, Toll, Benjamin, Zbikowski, Susan M. Association Between Utilization of Quitline Services and Probability of Tobacco Abstinence in Low-Income Smokers. Journal of substance abuse treatment. 2016;71:58. | RCTs that did not provide data on the outcome of interest (i.e., arrhythmia, nonfatal myocardial infarction, nonfatal stroke, cardiovascular death) during the treatment period. |
| 485 | Moreno-Coutiño, Ana, Pérez-López, Alejandro, Gallegos, Luis Villalobos. Predictors of retention in a multicomponent treatment for smokers. Revista de Psiquiatria Clinica. 2016;43:134. | RCTs that did not provide data on the outcome of interest (i.e., arrhythmia, nonfatal myocardial infarction, nonfatal stroke, cardiovascular death) during the treatment period. |
| 486 | Taylor, Gemma M.J., Taylor, Amy E., Thomas, Kyla H., Jones, Tim, Martin, Richard M., Munafò, Marcus R., Windmeijer, Frank, Davies, Neil M.. Effectiveness of varenicline versus nicotine replacement therapy on long-term smoking cessation in primary care: A prospective, cohort study of electronic medical records. The Lancet. 2016;388:107. | Observational studies (e.g., cohort, case-control, and cross-sectional studies). |
| 489 | Cummins, Sharon E, Gamst, Anthony C, Brandstein, Kendra, Seymann, Gregory B, Klonoff-Cohen, Hillary, Kirby, Carrie A, Tong, Elisa K, Chaplin, Edward, Tedeschi, Gary J, Zhu, Shu-Hong. Helping Hospitalized Smokers: A Factorial RCT of Nicotine Patches and Counseling. American journal of preventive medicine. 2016;51:578. | RCTs that did not provide data on the outcome of interest (i.e., arrhythmia, nonfatal myocardial infarction, nonfatal stroke, cardiovascular death) during the treatment period. |
| 494 | Janum, Susanne, Nielsen, Signe T, Werner, Mads U, Mehlsen, Jesper, Kehlet, Henrik, Møller, Kirsten. Pain perception in healthy volunteers: effect of repeated exposure to experimental systemic inflammation. Innate immunity. 2016;22:546. | RCTs that did not provide data on the outcome of interest (i.e., arrhythmia, nonfatal myocardial infarction, nonfatal stroke, cardiovascular death) during the treatment period. |
| 498 | Vaz, Luis R, Aveyard, Paul, Cooper, Sue, Leonardi-Bee, Jo, Coleman, Tim. The Association Between Treatment Adherence to Nicotine Patches and Smoking Cessation in Pregnancy: A Secondary Analysis of a Randomized Controlled Trial. Nicotine & tobacco research : official journal of the Society for Research on Nicotine and Tobacco. 2016;18:1952. | RCTs that did not provide data on the outcome of interest (i.e., arrhythmia, nonfatal myocardial infarction, nonfatal stroke, cardiovascular death) during the treatment period. |
| 507 | Kushnir, Vladyslav, Sproule, Beth A, Zawertailo, Laurie, Selby, Peter, Tyndale, Rachel F, Leatherdale, Scott T, Cunningham, John A. Impact of self-reported lifetime depression or anxiety on effectiveness of mass distribution of nicotine patches. Tobacco control. 2016;26:526. | RCTs that did not provide data on the outcome of interest (i.e., arrhythmia, nonfatal myocardial infarction, nonfatal stroke, cardiovascular death) during the treatment period. |
| 511 | Dennis, Paul A, Kimbrel, Nathan A, Dedert, Eric A, Beckham, Jean C, Dennis, Michelle F, Calhoun, Patrick S. Supplemental nicotine preloading for smoking cessation in posttraumatic stress disorder: Results from a randomized controlled trial. Addictive behaviors. 2016;59:24. | RCTs that did not provide data on the outcome of interest (i.e., arrhythmia, nonfatal myocardial infarction, nonfatal stroke, cardiovascular death) during the treatment period. |
| 514 | Ikonomidis, I., Kourea, K., Vlastos, D., Marinou, M., Vlachos, S., Varoudi, M., Tympas, K., Liarakos, N., Andreadou, I., Triantafyllidi, H., Pavlidis, G., Tsougos, E., Lekakis, J.. Varenicline vs. Nicotine replacement therapy: A prospective study of changes in arterial stiffness, endothelial glycocalyx and oxidative stress in smokers during 1 year follow-up. European Heart Journal. 2016;37:553. | RCTs that did not provide data on the outcome of interest (i.e., arrhythmia, nonfatal myocardial infarction, nonfatal stroke, cardiovascular death) during the treatment period. |
| 518 | Geiser, Christian, Griffin, Daniel, Shiffman, Saul. Using Multigroup-Multiphase Latent State-Trait Models to Study Treatment-Induced Changes in Intra-Individual State Variability: An Application to Smokers' Affect. Frontiers in psychology. 2016;7:1043. | RCTs that did not provide data on the outcome of interest (i.e., arrhythmia, nonfatal myocardial infarction, nonfatal stroke, cardiovascular death) during the treatment period. |
| 519 | Bernstein, Steven L, Rosner, June, Toll, Benjamin. A Multicomponent Intervention Including Texting to Promote Tobacco Abstinence in Emergency Department Smokers: A Pilot Study. Academic emergency medicine : official journal of the Society for Academic Emergency Medicine. 2016;23:803. | RCTs that did not provide data on the outcome of interest (i.e., arrhythmia, nonfatal myocardial infarction, nonfatal stroke, cardiovascular death) during the treatment period. |
| 523 | Esmat, Ibrahim M., Kassim, Dina Y.. Comparative study between transdermal nicotine and melatonin patches on postoperative pain relief after laparoscopic cholecystectomy, a double-blind, placebo-controlled trial. Egyptian Journal of Anaesthesia. 2016;32:299. | RCTs that did not provide data on the outcome of interest (i.e., arrhythmia, nonfatal myocardial infarction, nonfatal stroke, cardiovascular death) during the treatment period. |
| 524 | Ben Taleb, Ziyad, Ward, Kenneth D, Asfar, Taghrid, Jaber, Rana, Auf, Rehab, Maziak, Wasim. Predictors of nicotine withdrawal symptoms: findings from the first randomized smoking cessation trial in a low-income country setting. International journal of public health. 2016;61:701. | RCTs that did not provide data on the outcome of interest (i.e., arrhythmia, nonfatal myocardial infarction, nonfatal stroke, cardiovascular death) during the treatment period. |
| 525 | Anthenelli, Robert M, Benowitz, Neal L, West, Robert, St Aubin, Lisa, McRae, Thomas, Lawrence, David, Ascher, John, Russ, Cristina, Krishen, Alok, Evins, A Eden. Neuropsychiatric safety and efficacy of varenicline, bupropion, and nicotine patch in smokers with and without psychiatric disorders (EAGLES): a double-blind, randomised, placebo-controlled clinical trial. Lancet (London, England). 2016;387:2507. | RCTs that did not provide data on the outcome of interest (i.e., arrhythmia, nonfatal myocardial infarction, nonfatal stroke, cardiovascular death) during the treatment period. |
| 527 | Tulloch, Heather E, Pipe, Andrew L, Els, Charl, Clyde, Matthew J, Reid, Robert D. Flexible, dual-form nicotine replacement therapy or varenicline in comparison with nicotine patch for smoking cessation: a randomized controlled trial. BMC medicine. 2016;14:80. | RCTs that did not provide data on the outcome of interest (i.e., arrhythmia, nonfatal myocardial infarction, nonfatal stroke, cardiovascular death) during the treatment period. |
| 528 | Rabin, Rachel A, Ashare, Rebecca L, Schnoll, Robert A, Cinciripini, Paul M, Hawk, Larry W, Jr, Lerman, Caryn, Tyndale, Rachel F, George, Tony P. Does cannabis use moderate smoking cessation outcomes in treatment-seeking tobacco smokers? Analysis from a large multi-center trial. The American journal on addictions. 2016;25:291. | RCTs that did not provide data on the outcome of interest (i.e., arrhythmia, nonfatal myocardial infarction, nonfatal stroke, cardiovascular death) during the treatment period. |
| 529 | Prapavessis, Harry, De Jesus, Stefanie, Fitzgeorge, Lindsay, Faulkner, Guy, Maddison, Ralph, Batten, Sandra. Exercise to Enhance Smoking Cessation: the Getting Physical on Cigarette Randomized Control Trial. Annals of behavioral medicine : a publication of the Society of Behavioral Medicine. 2016;50:358. | RCTs that did not include a nicotine versus no nicotine intervention. |
| 532 | Schoenberg, Nancy E, Studts, Christina R, Shelton, Brent J, Liu, Meng, Clayton, Richard, Bispo, Jordan Baeker, Fields, Nell, Dignan, Mark, Cooper, Thomas. A randomized controlled trial of a faith-placed, lay health advisor delivered smoking cessation intervention for rural residents. Preventive medicine reports. 2016;3:317. | RCTs that did not provide data on the outcome of interest (i.e., arrhythmia, nonfatal myocardial infarction, nonfatal stroke, cardiovascular death) during the treatment period. |
| 537 | Arendt Nielsen, Thomas, Nielsen, Bruno Provstgaard, Wang, Kelun, Arendt-Nielsen, Lars, Boudreau, Shellie A. Psychophysical and Vasomotor Responses of the Oral Tissues: A Nicotine Dose-Response and Menthol Interaction Study. Nicotine & tobacco research : official journal of the Society for Research on Nicotine and Tobacco. 2016;18:596. | RCTs that did not provide data on the outcome of interest (i.e., arrhythmia, nonfatal myocardial infarction, nonfatal stroke, cardiovascular death) during the treatment period. |
| 539 | McRobbie, Hayden, Przulj, Dunja, Smith, Katherine Myers, Cornwall, Danielle. Complementing the Standard Multicomponent Treatment for Smokers With Denicotinized Cigarettes: A Randomized Trial. Nicotine & tobacco research : official journal of the Society for Research on Nicotine and Tobacco. 2016;18:1134. | RCTs that did not provide data on the outcome of interest (i.e., arrhythmia, nonfatal myocardial infarction, nonfatal stroke, cardiovascular death) during the treatment period. |
| 541 | Fu, Steven S, van Ryn, Michelle, Nelson, David, Burgess, Diana J, Thomas, Janet L, Saul, Jessie, Clothier, Barbara, Nyman, John A, Hammett, Patrick, Joseph, Anne M. Proactive tobacco treatment offering free nicotine replacement therapy and telephone counselling for socioeconomically disadvantaged smokers: a randomised clinical trial. Thorax. 2016;71:446. | RCTs that did not provide data on the outcome of interest (i.e., arrhythmia, nonfatal myocardial infarction, nonfatal stroke, cardiovascular death) during the treatment period. |
| 544 | Eugen-Olsen, Jesper, Ladelund, Steen, Sørensen, Lars Tue. Plasma suPAR is lowered by smoking cessation: a randomized controlled study. European journal of clinical investigation. 2016;46:305. | RCTs that did not provide data on the outcome of interest (i.e., arrhythmia, nonfatal myocardial infarction, nonfatal stroke, cardiovascular death) during the treatment period. |
| 545 | Berndt, Nadine, Bolman, Catherine, Lechner, Lilian, Max, Wendy, Mudde, Aart, de Vries, Hein, Evers, Silvia. Economic evaluation of a telephone- and face-to-face-delivered counseling intervention for smoking cessation in patients with coronary heart disease. The European journal of health economics : HEPAC : health economics in prevention and care. 2016;17:269. | RCTs that did not include a nicotine versus no nicotine intervention. |
| 554 | Tingen, Martha S., Andrews, Jeannette O., Heath, Janie, Williams, Lovoria B., Schroeder, Carsten, Dainer, Paul, Khleif, Samir N., Waller, Jennifer L.. Tailored parental cessation delivered concurrently with tobacco prevention in children enrolled in urban and rural southern elementary schools. Cancer Epidemiology Biomarkers and Prevention. 2016;25(3):B62. | RCTs that did not provide data on the outcome of interest (i.e., arrhythmia, nonfatal myocardial infarction, nonfatal stroke, cardiovascular death) during the treatment period. |
| 559 | Unrod, Marina, Simmons, Vani N, Sutton, Steven K, Cummings, K Michael, Celestino, Paula, Craig, Benjamin M, Lee, Ji-Hyun, Meltzer, Lauren R, Brandon, Thomas H. Relapse-Prevention Booklets as an Adjunct to a Tobacco Quitline: A Randomized Controlled Effectiveness Trial. Nicotine & tobacco research : official journal of the Society for Research on Nicotine and Tobacco. 2016;18:298. | RCTs that did not provide data on the outcome of interest (i.e., arrhythmia, nonfatal myocardial infarction, nonfatal stroke, cardiovascular death) during the treatment period. |
| 564 | Cunningham, John A, Kushnir, Vladyslav, Selby, Peter, Tyndale, Rachel F, Zawertailo, Laurie, Leatherdale, Scott T. Effect of Mailing Nicotine Patches on Tobacco Cessation Among Adult Smokers: A Randomized Clinical Trial. JAMA internal medicine. 2016;176:184. | RCTs that did not provide data on the outcome of interest (i.e., arrhythmia, nonfatal myocardial infarction, nonfatal stroke, cardiovascular death) during the treatment period. |
| 565 | Raja, Mitali, Saha, Sabyasachi, Krishna-Reddy, Vamsi, Mohd, Shafaat, Narang, Ridhi, Sood, Poonam. Effectiveness of oral health education versus nicotine replacement therapy for tobacco cessation- a parallel randomized clinical trial. Journal of clinical and experimental dentistry. 2016;8:e64. | RCTs that did not provide data on the outcome of interest (i.e., arrhythmia, nonfatal myocardial infarction, nonfatal stroke, cardiovascular death) during the treatment period. |
| 567 | Baker, Timothy B, Piper, Megan E, Stein, James H, Smith, Stevens S, Bolt, Daniel M, Fraser, David L, Fiore, Michael C. Effects of Nicotine Patch vs Varenicline vs Combination Nicotine Replacement Therapy on Smoking Cessation at 26 Weeks: A Randomized Clinical Trial. JAMA. 2016;315:371. | RCTs that did not provide data on the outcome of interest (i.e., arrhythmia, nonfatal myocardial infarction, nonfatal stroke, cardiovascular death) during the treatment period. |
| 568 | Cook, Jessica W, Collins, Linda M, Fiore, Michael C, Smith, Stevens S, Fraser, David, Bolt, Daniel M, Baker, Timothy B, Piper, Megan E, Schlam, Tanya R, Jorenby, Douglas, Loh, Wei-Yin, Mermelstein, Robin. Comparative effectiveness of motivation phase intervention components for use with smokers unwilling to quit: a factorial screening experiment. Addiction (Abingdon, England). 2016;111:117. | RCTs that did not provide data on the outcome of interest (i.e., arrhythmia, nonfatal myocardial infarction, nonfatal stroke, cardiovascular death) during the treatment period. |
| 569 | Piper, Megan E, Fiore, Michael C, Smith, Stevens S, Fraser, David, Bolt, Daniel M, Collins, Linda M, Mermelstein, Robin, Schlam, Tanya R, Cook, Jessica W, Jorenby, Douglas E, Loh, Wei-Yin, Baker, Timothy B. Identifying effective intervention components for smoking cessation: a factorial screening experiment. Addiction (Abingdon, England). 2016;111:129. | RCTs that did not include a nicotine versus no nicotine intervention. |
| 570 | Schlam, Tanya R, Fiore, Michael C, Smith, Stevens S, Fraser, David, Bolt, Daniel M, Collins, Linda M, Mermelstein, Robin, Piper, Megan E, Cook, Jessica W, Jorenby, Douglas E, Loh, Wei-Yin, Baker, Timothy B. Comparative effectiveness of intervention components for producing long-term abstinence from smoking: a factorial screening experiment. Addiction (Abingdon, England). 2016;111:142. | RCTs that did not include a nicotine versus no nicotine intervention. |
| 571 | Xiao, Dan, Kang, Jian, Kotler, Mitchell, Wang, Chen. A multicenter randomized, double-blind, parallel, placebo-controlled clinical study to evaluate the efficacy and safety of nicotine mint lozenge (2 mg and 4 mg) in smoking cessation. Chest. 2016;149:A589. | RCTs that did not provide data on the outcome of interest (i.e., arrhythmia, nonfatal myocardial infarction, nonfatal stroke, cardiovascular death) during the treatment period. |
| 580 | Wilcox, Charles, Oskooilar, Nader, Guevarra, Kimberly, -Linh Tong, My, Grosz, Daniel, Morrissey, Judy, Henry, Mellissa, De Francisco, Don. A double-blind, active-and placebo-controlled evaluation of the neuropsychiatric safety and efficacy of varenicline and bupropion for smoking cessation in subjects with (Pre-Existing) psychiatric disorders: An objective blinded analysis. Neuropsychopharmacology. 2015;40:S260. | RCTs that did not provide data on the outcome of interest (i.e., arrhythmia, nonfatal myocardial infarction, nonfatal stroke, cardiovascular death) during the treatment period. |
| 588 | Rohsenow, Damaris, Tidey, Jennifer W., Martin, Rosemarie A., Colby, Suzanne, Monti, Peter M.. Varenicline versus nicotine patch plus brief advice for sober smokers in substance treatment. Drug and Alcohol Dependence. 2015;156:e191. | RCTs that did not provide data on the outcome of interest (i.e., arrhythmia, nonfatal myocardial infarction, nonfatal stroke, cardiovascular death) during the treatment period. |
| 589 | Vaz, Luis R, Coleman, Tim, Cooper, Sue, Aveyard, Paul, Leonardi-Bee, Jo. The Nicotine Metabolite Ratio in Pregnancy Measured by trans-3'-Hydroxycotinine to Cotinine Ratio: Characteristics and Relationship With Smoking Cessation. Nicotine & tobacco research : official journal of the Society for Research on Nicotine and Tobacco. 2015;17:1318. | RCTs that did not provide data on the outcome of interest (i.e., arrhythmia, nonfatal myocardial infarction, nonfatal stroke, cardiovascular death) during the treatment period. |
| 593 | Jalali, Farzad, Afshari, Reza, Babaei, Ali, Abasspour, Hassan, Vahedian-Shahroodi, Mohammad. Comparing Motivational Interviewing-Based Treatment and its combination with Nicotine Replacement Therapy on smoking cessation in prisoners: a randomized controlled clinical trial. Electronic physician. 2015;7:1318. | RCTs that did not provide data on the outcome of interest (i.e., arrhythmia, nonfatal myocardial infarction, nonfatal stroke, cardiovascular death) during the treatment period. |
| 595 | Chandrashekar, M, Sattar, F A, Bondade, S, Kumar, K Kiran. A comparative study of different modalities of treatment in nicotine dependence syndrome. Asian journal of psychiatry. 2015;17:29. | RCTs that did not provide data on the outcome of interest (i.e., arrhythmia, nonfatal myocardial infarction, nonfatal stroke, cardiovascular death) during the treatment period. |
| 599 | Cosci, Fiammetta, Anna Aldi, Giulia, Nardi, Antonio Egidio. Does smoking abstinence influence distress tolerance? An experimental study comparing the response to a breath-holding test of smokers under tobacco withdrawal and under nicotine replacement therapy. Psychiatry research. 2015;229:89. | RCTs that did not provide data on the outcome of interest (i.e., arrhythmia, nonfatal myocardial infarction, nonfatal stroke, cardiovascular death) during the treatment period. |
| 601 | Karip, Bora, Metin, Keskin, Yalin, Iscan, Emre, Balik. Nicotine gum chewing for postoperative ileus after colorectal surgery. Colorectal Disease. 2015;17:29. | RCTs that did not provide data on the outcome of interest (i.e., arrhythmia, nonfatal myocardial infarction, nonfatal stroke, cardiovascular death) during the treatment period. |
| 602 | Chen, Li-Shiun, Baker, Timothy B, Jorenby, Douglas, Piper, Megan, Saccone, Nancy, Johnson, Eric, Breslau, Naomi, Hatsukami, Dorothy, Carney, Robert M, Bierut, Laura J. Genetic variation (CHRNA5), medication (combination nicotine replacement therapy vs. varenicline), and smoking cessation. Drug and alcohol dependence. 2015;154:278. | RCTs that did not provide data on the outcome of interest (i.e., arrhythmia, nonfatal myocardial infarction, nonfatal stroke, cardiovascular death) during the treatment period. |
| 603 | Guillot, Casey R, Stone, Matthew D, Geary, Bree A, Kirkpatrick, Matthew G, Tidey, Jennifer W, Cook, Jessica W, Leventhal, Adam M. Pharmacological, sensorimotor, and expectancy effects on tobacco withdrawal: a preliminary study. Human psychopharmacology. 2015;30:364. | RCTs that did not provide data on the outcome of interest (i.e., arrhythmia, nonfatal myocardial infarction, nonfatal stroke, cardiovascular death) during the treatment period. |
| 607 | Impey, Danielle, El-Marj, Nicole, Parks, Andrea, Choueiry, Joelle, Fisher, Derek, Knott, Verner J. Mismatch negativity in tobacco-naïve cannabis users and its alteration with acute nicotine administration. Pharmacology, biochemistry, and behavior. 2015;136:73. | RCTs that did not provide data on the outcome of interest (i.e., arrhythmia, nonfatal myocardial infarction, nonfatal stroke, cardiovascular death) during the treatment period. |
| 616 | Bernstein, Steven L, D'Onofrio, Gail, Rosner, June, O'Malley, Stephanie, Makuch, Robert, Busch, Susan, Pantalon, Michael V, Toll, Benjamin. Successful Tobacco Dependence Treatment in Low-Income Emergency Department Patients: A Randomized Trial. Annals of emergency medicine. 2015;66:140. | RCTs that did not provide data on the outcome of interest (i.e., arrhythmia, nonfatal myocardial infarction, nonfatal stroke, cardiovascular death) during the treatment period. |
| 618 | Ben Taleb, Ziyad, Ward, Kenneth D, Asfar, Taghrid, Bahelah, Raed, Maziak, Wasim. Predictors of adherence to pharmacological and behavioral treatment in a cessation trial among smokers in Aleppo, Syria. Drug and alcohol dependence. 2015;153:167. | RCTs that did not provide data on the outcome of interest (i.e., arrhythmia, nonfatal myocardial infarction, nonfatal stroke, cardiovascular death) during the treatment period. |
| 622 | McCarthy, Danielle E, Ebssa, Lemma, Witkiewitz, Katie, Shiffman, Saul. Paths to tobacco abstinence: A repeated-measures latent class analysis. Journal of consulting and clinical psychology. 2015;83:696. | RCTs that did not provide data on the outcome of interest (i.e., arrhythmia, nonfatal myocardial infarction, nonfatal stroke, cardiovascular death) during the treatment period. |
| 633 | Kroemer, Nils B., Wuttig, Franziska, Bidlingmaier, Martin, Zimmermann, Ulrich S., Smolka, Michael N.. Nicotine enhances modulation of food-cue reactivity by leptin and ghrelin in the ventromedial prefrontal cortex. Addiction Biology. 2015;20:832. | RCTs that did not provide data on the outcome of interest (i.e., arrhythmia, nonfatal myocardial infarction, nonfatal stroke, cardiovascular death) during the treatment period. |
| 634 | Bowers, H, Smith, D, de la Salle, S, Choueiry, J, Impey, D, Philippe, T, Dort, H, Millar, A, Daigle, M, Albert, P R, Beaudoin, A, Knott, V. COMT polymorphism modulates the resting-state EEG alpha oscillatory response to acute nicotine in male non-smokers. Genes, brain, and behavior. 2015;14:466. | RCTs that did not provide data on the outcome of interest (i.e., arrhythmia, nonfatal myocardial infarction, nonfatal stroke, cardiovascular death) during the treatment period. |
| 638 | Gray, Kevin M, McClure, Erin A, Baker, Nathaniel L, Hartwell, Karen J, Carpenter, Matthew J, Saladin, Michael E. An exploratory short-term double-blind randomized trial of varenicline versus nicotine patch for smoking cessation in women. Addiction (Abingdon, England). 2015;110:1027. | RCTs that did not provide data on the outcome of interest (i.e., arrhythmia, nonfatal myocardial infarction, nonfatal stroke, cardiovascular death) during the treatment period. |
| 641 | Essex, Holly N, Parrott, Steve, Wu, Qi, Li, Jinshuo, Cooper, Sue, Coleman, Tim. Cost-Effectiveness of Nicotine Patches for Smoking Cessation in Pregnancy: A Placebo Randomized Controlled Trial (SNAP). Nicotine & tobacco research : official journal of the Society for Research on Nicotine and Tobacco. 2015;17:636. | RCTs that did not provide data on the outcome of interest (i.e., arrhythmia, nonfatal myocardial infarction, nonfatal stroke, cardiovascular death) during the treatment period. |
| 644 | Smucny, Jason, Olincy, Ann, Eichman, Lindsay S, Tregellas, Jason R. Neuronal effects of nicotine during auditory selective attention. Psychopharmacology. 2015;232:2017. | RCTs that did not provide data on the outcome of interest (i.e., arrhythmia, nonfatal myocardial infarction, nonfatal stroke, cardiovascular death) during the treatment period. |
| 648 | Bernstein, Steven L., Rosner, June, Toll, Benjamin. Texting to promote tobacco abstinence in emergency department smokers: A pilot study. Academic Emergency Medicine. 2015;22:S74. | RCTs that did not provide data on the outcome of interest (i.e., arrhythmia, nonfatal myocardial infarction, nonfatal stroke, cardiovascular death) during the treatment period. |
| 657 | Saladin, Michael E, McClure, Erin A, Baker, Nathaniel L, Carpenter, Matthew J, Ramakrishnan, Viswanathan, Hartwell, Karen J, Gray, Kevin M. Increasing progesterone levels are associated with smoking abstinence among free-cycling women smokers who receive brief pharmacotherapy. Nicotine & tobacco research : official journal of the Society for Research on Nicotine and Tobacco. 2015;17:398. | RCTs that did not provide data on the outcome of interest (i.e., arrhythmia, nonfatal myocardial infarction, nonfatal stroke, cardiovascular death) during the treatment period. |
| 661 | O'Brien, Brigid, Knight-West, Oliver, Walker, Natalie, Parag, Varsha, Bullen, Christopher. E-cigarettes versus NRT for smoking reduction or cessation in people with mental illness: secondary analysis of data from the ASCEND trial. Tobacco induced diseases. 2015;13:5. | RCTs that did not provide data on the outcome of interest (i.e., arrhythmia, nonfatal myocardial infarction, nonfatal stroke, cardiovascular death) during the treatment period. |
| 662 | Minami, Haruka, Kahler, Christopher W., Bloom, Erika L., Strong, David R., Abrantes, Ana M., Zywiak, William H., Price, Lawrence H., Brown, Richard A.. Effects of depression history and sex on the efficacy of sequential versus standard Fluoxetine for smoking cessation in elevated depressive symptom smokers. Addictive Disorders and their Treatment. 2015;14:29. | RCTs that did not provide data on the outcome of interest (i.e., arrhythmia, nonfatal myocardial infarction, nonfatal stroke, cardiovascular death) during the treatment period. |
| 665 | Danaher, Brian G., Severson, Herbert H., Crowley, Ryann, van Meter, Nora, Tyler, Milagra S., Widdop, Chris, Lichtenstein, Edward, Ebbert, Jon O.. Randomized controlled trial examining the adjunctive use of nicotine lozenges with MyLastDip: An eHealth smokeless tobacco cessation intervention. Internet Interventions. 2015;2:69. | RCTs that did not provide data on the outcome of interest (i.e., arrhythmia, nonfatal myocardial infarction, nonfatal stroke, cardiovascular death) during the treatment period. |
| 666 | Severson, Herbert H, Danaher, Brian G, Ebbert, Jon O, van Meter, Nora, Lichtenstein, Edward, Widdop, Chris, Crowley, Ryann, Akers, Laura, Seeley, John R. Randomized trial of nicotine lozenges and phone counseling for smokeless tobacco cessation. Nicotine & tobacco research : official journal of the Society for Research on Nicotine and Tobacco. 2015;17:309. | RCTs that did not provide data on the outcome of interest (i.e., arrhythmia, nonfatal myocardial infarction, nonfatal stroke, cardiovascular death) during the treatment period. |
| 673 | Haas, Jennifer S, Linder, Jeffrey A, Park, Elyse R, Gonzalez, Irina, Rigotti, Nancy A, Klinger, Elissa V, Kontos, Emily Z, Zaslavsky, Alan M, Brawarsky, Phyllis, Marinacci, Lucas X, St Hubert, Stella, Fleegler, Eric W, Williams, David R. Proactive tobacco cessation outreach to smokers of low socioeconomic status: a randomized clinical trial. JAMA internal medicine. 2015;175:218. | RCTs that did not provide data on the outcome of interest (i.e., arrhythmia, nonfatal myocardial infarction, nonfatal stroke, cardiovascular death) during the treatment period. |
| 674 | Cook, Jessica W, Piper, Megan E, Leventhal, Adam M, Schlam, Tanya R, Fiore, Michael C, Baker, Timothy B. Anhedonia as a component of the tobacco withdrawal syndrome. Journal of abnormal psychology. 2015;124:215. | RCTs that did not provide data on the outcome of interest (i.e., arrhythmia, nonfatal myocardial infarction, nonfatal stroke, cardiovascular death) during the treatment period. |
| 675 | Bhatia, S., Waller, J.L., Tingen, M.. Examining the effectiveness of a multi-level intervention on parental self-efficacy for delivering an anti-tobacco socialization message in a predominantly African American population. Journal of Investigative Medicine. 2015;63:461. | RCTs that did not provide data on the outcome of interest (i.e., arrhythmia, nonfatal myocardial infarction, nonfatal stroke, cardiovascular death) during the treatment period. |
| 681 | Vorel, Stanislav R., Mahoney, Melissa, Bisaga, A.. Influence of nicotine replacement therapy on heart rate variability and relapse. Drug and Alcohol Dependence. 2015;146:e12. | RCTs that did not provide data on the outcome of interest (i.e., arrhythmia, nonfatal myocardial infarction, nonfatal stroke, cardiovascular death) during the treatment period. |
| 686 | O'Brien, Brigid, Knight-West, Oliver, Walker, Natalie, Parag, Varsha, Bullen, Christopher. E-cigarettes versus NRT for smoking reduction or cessation in people with mental illness: Secondary analysis of data from the ASCEND trial Dr Maciej L. Goniewicz. Tobacco Induced Diseases. 2015; 13(1):5. | Duplicate articles or articles with the exact same study outcome data as another published article. |
| 687 | Walker, Natalie, Howe, Colin, Glover, Marewa, McRobbie, Hayden, Barnes, Joanne, Nosa, Vili, Parag, Varsha, Bassett, Bruce, Bullen, Christopher. Cytisine versus nicotine for smoking cessation. The New England journal of medicine. 2014;371:2353. | RCTs that did not include a nicotine versus no nicotine intervention. |
| 691 | Smith, Stevens S, Fiore, Michael C, Baker, Timothy B. Smoking cessation in smokers who smoke menthol and non-menthol cigarettes. Addiction (Abingdon, England). 2014;109:2107. | RCTs that did not provide data on the outcome of interest (i.e., arrhythmia, nonfatal myocardial infarction, nonfatal stroke, cardiovascular death) during the treatment period. |
| 697 | Fraser, D, Kobinsky, K, Smith, S S, Kramer, J, Theobald, W E, Baker, T B. Five population-based interventions for smoking cessation: a MOST trial. Translational behavioral medicine. 2014;4:382. | RCTs that did not provide data on the outcome of interest (i.e., arrhythmia, nonfatal myocardial infarction, nonfatal stroke, cardiovascular death) during the treatment period. |
| 705 | de Dios, Marcel A, Anderson, Bradley J, Caviness, Celeste M, Stein, Michael D. Early quit days among methadone-maintained smokers in a smoking cessation trial. Nicotine & tobacco research : official journal of the Society for Research on Nicotine and Tobacco. 2014;16:1463. | RCTs that did not provide data on the outcome of interest (i.e., arrhythmia, nonfatal myocardial infarction, nonfatal stroke, cardiovascular death) during the treatment period. |
| 714 | Kawachi, E., Imaizumi, S., Takata, K., Abe, S., Matsuo, Y., Yahiro, E., Uehara, Y., Zhang, B., Miura, S., Saku, K.. Effect of smoking cessation on HDL function. European Heart Journal. 2014;35:902. | RCTs that did not provide data on the outcome of interest (i.e., arrhythmia, nonfatal myocardial infarction, nonfatal stroke, cardiovascular death) during the treatment period. |
| 715 | Knott, Verner, Impey, Danielle, Philippe, Tristan, Smith, Dylan, Choueiry, Joelle, de la Salle, Sara, Dort, Heather. Modulation of auditory deviance detection by acute nicotine is baseline and deviant dependent in healthy nonsmokers: a mismatch negativity study. Human psychopharmacology. 2014;29:446. | RCTs that did not provide data on the outcome of interest (i.e., arrhythmia, nonfatal myocardial infarction, nonfatal stroke, cardiovascular death) during the treatment period. |
| 720 | Cooper, Sue, Taggar, Jaspal, Lewis, Sarah, Marlow, Neil, Dickinson, Anne, Whitemore, Rachel, Coleman, Tim. Effect of nicotine patches in pregnancy on infant and maternal outcomes at 2 years: follow-up from the randomised, double-blind, placebo-controlled SNAP trial. The Lancet. Respiratory medicine. 2014;2:728. | RCTs that did not provide data on the outcome of interest (i.e., arrhythmia, nonfatal myocardial infarction, nonfatal stroke, cardiovascular death) during the treatment period. |
| 723 | Cooper, Sue, Lewis, Sarah, Thornton, James G, Marlow, Neil, Watts, Kim, Britton, John, Grainge, Matthew J, Taggar, Jaspal, Essex, Holly, Parrott, Steve, Dickinson, Anne, Whitemore, Rachel, Coleman, Tim. The SNAP trial: a randomised placebo-controlled trial of nicotine replacement therapy in pregnancy--clinical effectiveness and safety until 2 years after delivery, with economic evaluation. Health technology assessment (Winchester, England). 2014;18:1. | Duplicate articles or articles with the exact same study outcome data as another published article. |
| 724 | Schlagintweit, Hera E, Good, Kimberley P, Barrett, Sean P. The impact of nicotine lozenges and stimulus expectancies on cigarette craving. Journal of psychopharmacology (Oxford, England). 2014;28:773. | RCTs that did not provide data on the outcome of interest (i.e., arrhythmia, nonfatal myocardial infarction, nonfatal stroke, cardiovascular death) during the treatment period. |
| 728 | Koegelenberg, Coenraad F N, Noor, Firdows, Bateman, Eric D, van Zyl-Smit, Richard N, Bruning, Axel, O'Brien, John A, Smith, Clifford, Abdool-Gaffar, Mohamed S, Emanuel, Shaunagh, Esterhuizen, Tonya M, Irusen, Elvis M. Efficacy of varenicline combined with nicotine replacement therapy vs varenicline alone for smoking cessation: a randomized clinical trial. JAMA. 2014;312:155. | RCTs that did not provide data on the outcome of interest (i.e., arrhythmia, nonfatal myocardial infarction, nonfatal stroke, cardiovascular death) during the treatment period. |
| 733 | Ostroff, Jamie S, Burkhalter, Jack E, Cinciripini, Paul M, Li, Yuelin, Shiyko, Mariya P, Lam, Cho Y, Hay, Jennifer L, Dhingra, Lara K, Lord-Bessen, Jennifer, Holland, Susan M, Manna, Ruth. Randomized trial of a presurgical scheduled reduced smoking intervention for patients newly diagnosed with cancer. Health psychology : official journal of the Division of Health Psychology, American Psychological Association. 2014;33:737. | RCTs that did not include a nicotine versus no nicotine intervention. |
| 734 | Pilhatsch, Maximilian, Scheuing, Hannah, Kroemer, Nils, Kobiella, Andrea, Bidlingmaier, Martin, Farger, Gisbert, Smolka, Michael N, Zimmermann, Ulrich S. Nicotine administration in healthy non-smokers reduces appetite but does not alter plasma ghrelin. Human psychopharmacology. 2014;29:384. | RCTs that did not provide data on the outcome of interest (i.e., arrhythmia, nonfatal myocardial infarction, nonfatal stroke, cardiovascular death) during the treatment period. |
| 735 | Brody, Arthur L, Mukhin, Alexey G, Mamoun, Michael S, Luu, Trinh, Neary, Meaghan, Liang, Lidia, Shieh, Jennifer, Sugar, Catherine A, Rose, Jed E, Mandelkern, Mark A. Brain nicotinic acetylcholine receptor availability and response to smoking cessation treatment: a randomized trial. JAMA psychiatry. 2014;71:797. | RCTs that did not provide data on the outcome of interest (i.e., arrhythmia, nonfatal myocardial infarction, nonfatal stroke, cardiovascular death) during the treatment period. |
| 736 | Xu, Xiaomeng, Clark, Uraina S, David, Sean P, Mulligan, Richard C, Knopik, Valerie S, McGeary, John, MacKillop, James, McCaffery, Jeanne, Niaura, Raymond S, Sweet, Lawrence H. Effects of nicotine deprivation and replacement on BOLD-fMRI response to smoking cues as a function of DRD4 VNTR genotype. Nicotine & tobacco research : official journal of the Society for Research on Nicotine and Tobacco. 2014;16:939. | RCTs that did not provide data on the outcome of interest (i.e., arrhythmia, nonfatal myocardial infarction, nonfatal stroke, cardiovascular death) during the treatment period. |
| 737 | Jardin, Bianca F, Cropsey, Karen L, Wahlquist, Amy E, Gray, Kevin M, Silvestri, Gerard A, Cummings, K Michael, Carpenter, Matthew J. Evaluating the effect of access to free medication to quit smoking: a clinical trial testing the role of motivation. Nicotine & tobacco research : official journal of the Society for Research on Nicotine and Tobacco. 2014;16:992. | RCTs that did not provide data on the outcome of interest (i.e., arrhythmia, nonfatal myocardial infarction, nonfatal stroke, cardiovascular death) during the treatment period. |
| 738 | Knott, Verner, Choueiry, Joelle, Dort, Heather, Smith, Dylan, Impey, Danielle, de la Salle, Sara, Philippe, Tristan. Baseline-dependent modulating effects of nicotine on voluntary and involuntary attention measured with brain event-related P3 potentials. Pharmacology, biochemistry, and behavior. 2014;122:107. | RCTs that did not provide data on the outcome of interest (i.e., arrhythmia, nonfatal myocardial infarction, nonfatal stroke, cardiovascular death) during the treatment period. |
| 739 | Ferguson, Stuart G, Shiffman, Saul. Effect of high-dose nicotine patch on craving and negative affect leading up to lapse episodes. Psychopharmacology. 2014;231:2595. | RCTs that did not provide data on the outcome of interest (i.e., arrhythmia, nonfatal myocardial infarction, nonfatal stroke, cardiovascular death) during the treatment period. |
| 740 | Hsueh, Kuang-Chieh, Hsueh, Shu-Chun, Chou, Ming-Yueh, Pan, Lee-Fei, Tu, Ming-Shium, McEwen, Andy, West, Robert. Varenicline versus transdermal nicotine patch: a 3-year follow-up in a smoking cessation clinic in Taiwan. Psychopharmacology. 2014;231:2819. | RCTs that did not provide data on the outcome of interest (i.e., arrhythmia, nonfatal myocardial infarction, nonfatal stroke, cardiovascular death) during the treatment period. |
| 755 | Bernstein, Steven L., Rosner, June, O'Malley, Stephanie, D'Onofrio, Gail, Makuch, Robert, Busch, Susan, Toll, Benjamin. Successful tobacco dependence treatment achieved via pharmacotherapy and motivational interviewing in low-income emergency department patients. Academic Emergency Medicine. 2014;21:S59. | RCTs that did not provide data on the outcome of interest (i.e., arrhythmia, nonfatal myocardial infarction, nonfatal stroke, cardiovascular death) during the treatment period. |
| 756 | Bernstein, Steven L., Zbikowski, Susan, Rosner, June, O'Malley, Stephanie, D'Onofrio, Gail, Makuch, Robert, Busch, Susan, Toll, Benjamin. Association between intensity of quitline services and probability of smoking abstinence. Academic Emergency Medicine. 2014;21:S239. | RCTs that did not provide data on the outcome of interest (i.e., arrhythmia, nonfatal myocardial infarction, nonfatal stroke, cardiovascular death) during the treatment period. |
| 763 | Winhusen, Theresa M, Brigham, Gregory S, Kropp, Frankie, Lindblad, Robert, Gardin, John G, 2nd, Penn, Pat, Hodgkins, Candace, Kelly, Thomas M, Douaihy, Antoine, McCann, Michael, Love, Lee D, DeGravelles, Eliot, Bachrach, Ken, Sonne, Susan C, Hiott, Bob, Haynes, Louise, Sharma, Gaurav, Lewis, Daniel F, VanVeldhuisen, Paul, Theobald, Jeff, Ghitza, Udi. A randomized trial of concurrent smoking-cessation and substance use disorder treatment in stimulant-dependent smokers. The Journal of clinical psychiatry. 2014;75:336. | RCTs that did not provide data on the outcome of interest (i.e., arrhythmia, nonfatal myocardial infarction, nonfatal stroke, cardiovascular death) during the treatment period. |
| 769 | Davis, James M, Goldberg, Simon B, Anderson, Maggie C, Manley, Alison R, Smith, Stevens S, Baker, Timothy B. Randomized trial on mindfulness training for smokers targeted to a disadvantaged population. Substance use & misuse. 2014;49:571. | RCTs that did not provide data on the outcome of interest (i.e., arrhythmia, nonfatal myocardial infarction, nonfatal stroke, cardiovascular death) during the treatment period. |
| 772 | Berlin, Ivan, Grangé, Gilles, Jacob, Nelly, Tanguy, Marie-Laure. Nicotine patches in pregnant smokers: randomised, placebo controlled, multicentre trial of efficacy. BMJ (Clinical research ed.). 2014;348:g1622. | RCTs that did not provide data on the outcome of interest (i.e., arrhythmia, nonfatal myocardial infarction, nonfatal stroke, cardiovascular death) during the treatment period. |
| 773 | Ghanem, Maha. Evaluation of intensive outpatient antismoking counselling program in a low-income country. Chest. 2014;145(S3):617A. | RCTs that did not include a nicotine versus no nicotine intervention. |
| 775 | Buller, David B, Halperin, Abigail, Severson, Herbert H, Borland, Ron, Slater, Michael D, Bettinghaus, Erwin P, Tinkelman, David, Cutter, Gary R, Woodall, William Gill. Effect of nicotine replacement therapy on quitting by young adults in a trial comparing cessation services. Journal of public health management and practice : JPHMP. 2014;20:E7. | RCTs that did not include a nicotine versus no nicotine intervention. |
| 778 | Jaehne, A, Unbehaun, T, Feige, B, Herr, S, Appel, A, Riemann, D. The influence of 8 and 16 mg nicotine patches on sleep in healthy non-smokers. Pharmacopsychiatry. 2014;47:73. | RCTs that did not provide data on the outcome of interest (i.e., arrhythmia, nonfatal myocardial infarction, nonfatal stroke, cardiovascular death) during the treatment period. |
| 781 | Barnett, Paul G, Wong, Wynnie, Jeffers, Abra, Munoz, Ricardo, Humfleet, Gary, Hall, Sharon. Cost-effectiveness of extended cessation treatment for older smokers. Addiction (Abingdon, England). 2014;109:314. | RCTs that did not provide data on the outcome of interest (i.e., arrhythmia, nonfatal myocardial infarction, nonfatal stroke, cardiovascular death) during the treatment period. |
| 782 | Hasan, Faysal M, Zagarins, Sofija E, Pischke, Karen M, Saiyed, Shamila, Bettencourt, Ann Marie, Beal, Laura, Macys, Diane, Aurora, Sanjay, McCleary, Nancy. Hypnotherapy is more effective than nicotine replacement therapy for smoking cessation: results of a randomized controlled trial. Complementary therapies in medicine. 2014;22:1. | RCTs that did not provide data on the outcome of interest (i.e., arrhythmia, nonfatal myocardial infarction, nonfatal stroke, cardiovascular death) during the treatment period. |
| 787 | Heydari, G, Talischi, F, Batmanghelidj, E, Pajooh, M R, Boroomand, A, Zamani, M, Salehi, A, Maddah, S. Dual addictions, parallel treatments: nicotine replacement therapy for patients receiving methadone treatment in the Islamic Republic of Iran. Eastern Mediterranean health journal = La revue de sante de la Mediterranee orientale = al-Majallah al-sihhiyah li-sharq al-mutawassit. 2014;19 Suppl 3:S25. | RCTs that did not provide data on the outcome of interest (i.e., arrhythmia, nonfatal myocardial infarction, nonfatal stroke, cardiovascular death) during the treatment period. |
| 791 | Chen, Li-Shiun, Bloom, A Joseph, Baker, Timothy B, Smith, Stevens S, Piper, Megan E, Martinez, Maribel, Saccone, Nancy, Hatsukami, Dorothy, Goate, Alison, Bierut, Laura. Pharmacotherapy effects on smoking cessation vary with nicotine metabolism gene (CYP2A6). Addiction (Abingdon, England). 2014;109:128. | RCTs that did not provide data on the outcome of interest (i.e., arrhythmia, nonfatal myocardial infarction, nonfatal stroke, cardiovascular death) during the treatment period. |
| 792 | Koegelenberg, C.F., Noor, F., Bolliger, C.T., Bateman, E.D., Van Zyl-Smit, R.N., O'Brien, J.A., Bruning, A., Smith, C., Abdool-Gaffar, M.S., Emanuel, S., Irusen, E.M.. A 24-week multicentre, randomised, double-blind study to evaluate the efficacy and safety of varenicline in combination with nicotine replacement therapy vs. varenicline alone for smoking cessation. American Journal of Respiratory and Critical Care Medicine. 2014; 189:A1083. | RCTs that did not provide data on the outcome of interest (i.e., arrhythmia, nonfatal myocardial infarction, nonfatal stroke, cardiovascular death) during the treatment period. |
| 793 | VanNostrand, K., Fisher, K., Khan, B.A.. Smoking cessation after surviving critical illness-is it feasible?-Authors experience with implementation and recruitment. American Journal of Respiratory and Critical Care Medicine. 2014; 189:A4482. | RCTs that did not provide data on the outcome of interest (i.e., arrhythmia, nonfatal myocardial infarction, nonfatal stroke, cardiovascular death) during the treatment period. |
| 794 | Pei, Hao, Zhang, Ling-Jie, Zeng, Li-Ming, Yu, Feng. Effect of preoperative smoking intervention on postoperative complications of total hip replacement. Chinese Journal of Evidence-Based Medicine. 2014;14:399. | Articles in which the abstract and full text was non-English. |
| 795 | Takata, Kohei, Imaizumi, Satoshi, Kawachi, Emi, Suematsu, Yasunori, Shimizu, Tomohiko, Abe, Satomi, Matsuo, Yoshino, Tsukahara, Hitomi, Noda, Keita, Yahiro, Eiji, Zhang, Bo, Uehara, Yoshinari, Miura, Shin-Ichiro, Saku, Keijiro. Impact of cigarette smoking cessation on high-density lipoprotein functionality: – VN-SEESAW-HDL –. Circulation Journal. 2014;78:2955. | RCTs that did not provide data on the outcome of interest (i.e., arrhythmia, nonfatal myocardial infarction, nonfatal stroke, cardiovascular death) during the treatment period. |
| 803 | Kotz, Daniel, Brown, Jamie, West, Robert. Effectiveness of varenicline versus nicotine replacement therapy for smoking cessation with minimal professional support: evidence from an English population study. Psychopharmacology. 2014;231:37. | Observational studies (e.g., cohort, case-control, and cross-sectional studies). |
| 807 | Stapleton, John, West, Robert, Hajek, Peter, Wheeler, Jenny, Vangeli, Eleni, Abdi, Zeinab, O'Gara, Colin, McRobbie, Hayden, Humphrey, Kirsty, Ali, Rachel, Strang, John, Sutherland, Gay. Randomized trial of nicotine replacement therapy (NRT), bupropion and NRT plus bupropion for smoking cessation: effectiveness in clinical practice. Addiction (Abingdon, England). 2013;108:2193. | RCTs that did not provide data on the outcome of interest (i.e., arrhythmia, nonfatal myocardial infarction, nonfatal stroke, cardiovascular death) during the treatment period. |
| 808 | McClure, Erin A, Saladin, Michael E, Baker, Nathaniel L, Carpenter, Matthew J, Gray, Kevin M. Smoking topography and abstinence in adult female smokers. Addictive behaviors. 2013;38:2833. | RCTs that did not provide data on the outcome of interest (i.e., arrhythmia, nonfatal myocardial infarction, nonfatal stroke, cardiovascular death) during the treatment period. |
| 809 | Cosci, Fiammetta, Bertoli, Giuly, Abrams, Kenneth. Effects of nicotine withdrawal on panic-like response to breath holding: a placebo-controlled, double-blind, crossover patch study. Depression and anxiety. 2013;30:1217. | RCTs that did not include a nicotine versus no nicotine intervention. |
| 811 | Liu, Xiaoyu, Li, Runze, Lanza, Stephanie T, Vasilenko, Sara A, Piper, Megan. Understanding the role of cessation fatigue in the smoking cessation process. Drug and alcohol dependence. 2013;133:548. | RCTs that did not provide data on the outcome of interest (i.e., arrhythmia, nonfatal myocardial infarction, nonfatal stroke, cardiovascular death) during the treatment period. |
| 812 | Allen, Alicia M, Kleppinger, Alison, Lando, Harry, Oncken, Cheryl. Effect of nicotine patch on energy intake and weight gain in postmenopausal women during smoking cessation. Eating behaviors. 2013;14:420. | RCTs that did not provide data on the outcome of interest (i.e., arrhythmia, nonfatal myocardial infarction, nonfatal stroke, cardiovascular death) during the treatment period. |
| 813 | Hawkins, Kirsten A, Cougle, Jesse R. The effects of nicotine on intrusive memories in nonsmokers. Experimental and clinical psychopharmacology. 2013;21:434. | RCTs that did not provide data on the outcome of interest (i.e., arrhythmia, nonfatal myocardial infarction, nonfatal stroke, cardiovascular death) during the treatment period. |
| 814 | Brody, Arthur, Mukhin, Alexey, Mamoun, Michael, Luu, Trinh, Neary, Meaghan, Liang, Lidia, Shieh, Jennifer, Sugar, Catherine A., Rose, Jed, Mandelkern, Mark. Nicotinic acetylcholine receptor density as a predictor of quitting smoking with treatment. Neuropsychopharmacology. 2013;38:S140. | RCTs that did not provide data on the outcome of interest (i.e., arrhythmia, nonfatal myocardial infarction, nonfatal stroke, cardiovascular death) during the treatment period. |
| 819 | Bullen, Christopher, Howe, Colin, Laugesen, Murray, McRobbie, Hayden, Parag, Varsha, Williman, Jonathan, Walker, Natalie. Electronic cigarettes for smoking cessation: a randomised controlled trial. Lancet (London, England). 2013;382:1629. | RCTs that did not provide data on the outcome of interest (i.e., arrhythmia, nonfatal myocardial infarction, nonfatal stroke, cardiovascular death) during the treatment period. |
| 825 | Cropsey, Karen L, Hendricks, Peter S, Jardin, Bianca, Clark, C Brendan, Katiyar, Nandan, Willig, James, Mugavero, Michael, Raper, James L, Saag, Michael, Carpenter, Matthew J. A pilot study of screening, brief intervention, and referral for treatment (SBIRT) in non-treatment seeking smokers with HIV. Addictive behaviors. 2013;38:2541. | RCTs that did not provide data on the outcome of interest (i.e., arrhythmia, nonfatal myocardial infarction, nonfatal stroke, cardiovascular death) during the treatment period. |
| 832 | Pathak, Vikas, Rendon, Iliana Samara Hurtado, Lupu, Roxana, Tactuk, Nancy, Olutade, Toyosi, Durham, Carolyn, Stumacher, Richard. Outcome of nicotine replacement therapy in patients admitted to ICU: a randomized controlled double-blind prospective pilot study. Respiratory care. 2013;58:1625. | RCTs that did not provide data on the outcome of interest (i.e., arrhythmia, nonfatal myocardial infarction, nonfatal stroke, cardiovascular death) during the treatment period. |
| 833 | Kochunov, Peter, Du, Xiaoming, Moran, Lauren V, Sampath, Hemalatha, Wijtenburg, S Andrea, Yang, Yihong, Rowland, Laura M, Stein, Elliot A, Hong, L Elliot. Acute nicotine administration effects on fractional anisotropy of cerebral white matter and associated attention performance. Frontiers in pharmacology. 2013;4:117. | RCTs that did not provide data on the outcome of interest (i.e., arrhythmia, nonfatal myocardial infarction, nonfatal stroke, cardiovascular death) during the treatment period. |
| 834 | Lee, Susan M, Landry, Jennifer, Jones, Philip M, Buhrmann, Ozzie, Morley-Forster, Patricia. The effectiveness of a perioperative smoking cessation program: a randomized clinical trial. Anesthesia and analgesia. 2013;117:605. | RCTs that did not provide data on the outcome of interest (i.e., arrhythmia, nonfatal myocardial infarction, nonfatal stroke, cardiovascular death) during the treatment period. |
| 835 | Bullen, Christopher, Howe, Colin, Laugesen, Murray, McRobbie, Hayden, Parag, Varsha, Williman, Jonathan, Walker, Natalie. Do electronic cigarettes help smokers quit? Results from a randomized controlled trial. European Respiratory Journal. 2013; 42(S57):1047. | RCTs that did not provide data on the outcome of interest (i.e., arrhythmia, nonfatal myocardial infarction, nonfatal stroke, cardiovascular death) during the treatment period. |
| 837 | Brown, Jamie, Hajek, Peter, McRobbie, Hayden, Locker, Jo, Gillison, Fiona, McEwen, Andy, Beard, Emma, West, Robert. Cigarette craving and withdrawal symptoms during temporary abstinence and the effect of nicotine gum. Psychopharmacology. 2013;229:209. | RCTs that did not provide data on the outcome of interest (i.e., arrhythmia, nonfatal myocardial infarction, nonfatal stroke, cardiovascular death) during the treatment period. |
| 843 | Rose, Jed E, Behm, Frédérique M. Adapting smoking cessation treatment according to initial response to precessation nicotine patch. The American journal of psychiatry. 2013;170:860. | RCTs that did not provide data on the outcome of interest (i.e., arrhythmia, nonfatal myocardial infarction, nonfatal stroke, cardiovascular death) during the treatment period. |
| 846 | Petrovsky, Nadine, Ettinger, Ulrich, Quednow, Boris B, Landsberg, Martin W, Drees, Judith, Lennertz, Leonhard, Frommann, Ingo, Heilmann, Katharina, Sträter, Birgitta, Kessler, Henrik, Dahmen, Norbert, Mössner, Rainald, Maier, Wolfgang, Wagner, Michael. Nicotine enhances antisaccade performance in schizophrenia patients and healthy controls. The international journal of neuropsychopharmacology. 2013;16:1473. | RCTs that did not provide data on the outcome of interest (i.e., arrhythmia, nonfatal myocardial infarction, nonfatal stroke, cardiovascular death) during the treatment period. |
| 849 | El-Mohandes, Ayman A E, Windsor, Richard, Tan, Sylvia, Perry, David C, Gantz, Marie G, Kiely, Michele. A randomized clinical trial of trans-dermal nicotine replacement in pregnant African-American smokers. Maternal and child health journal. 2013;17:897. | RCTs that did not provide data on the outcome of interest (i.e., arrhythmia, nonfatal myocardial infarction, nonfatal stroke, cardiovascular death) during the treatment period. |
| 850 | Duffy, Sonia A, Fowler, Karen E, Flanagan, Petra S, Ronis, David L, Ewing, Lee A, Waltje, Andrea H. The development of the tobacco tactics website. JMIR research protocols. 2013;2:e22. | RCTs that did not provide data on the outcome of interest (i.e., arrhythmia, nonfatal myocardial infarction, nonfatal stroke, cardiovascular death) during the treatment period. |
| 851 | Rasmussen, Louise, Ladelund, Steen, Brünner, Nils, Jørgensen, Lars N, Nielsen, Hans J, Sørensen, Lars T. Tissue inhibitor of metalloproteinases-1 as a biological marker in colorectal cancer: influence of smoking on plasma levels?. The International journal of biological markers. 2013;28:226. | RCTs that did not provide data on the outcome of interest (i.e., arrhythmia, nonfatal myocardial infarction, nonfatal stroke, cardiovascular death) during the treatment period. |
| 857 | Weimer, Katja, Horing, Björn, Stürmer, Julian, Klosterhalfen, Sibylle, Zipfel, Stephan, Enck, Paul. Nicotine stimulus expectancy differentially affects reaction time in healthy nonsmokers and smokers depending on sex: a pilot study. Experimental and clinical psychopharmacology. 2013;21:181. | RCTs that did not provide data on the outcome of interest (i.e., arrhythmia, nonfatal myocardial infarction, nonfatal stroke, cardiovascular death) during the treatment period. |
| 860 | Hajek, Peter, Smith, Katie Myers, Dhanji, Al-Rehan, McRobbie, Hayden. Is a combination of varenicline and nicotine patch more effective in helping smokers quit than varenicline alone? A randomised controlled trial. BMC medicine. 2013;11:140. | RCTs that did not provide data on the outcome of interest (i.e., arrhythmia, nonfatal myocardial infarction, nonfatal stroke, cardiovascular death) during the treatment period. |
| 862 | Ebbert, Jon O, Severson, Herbert H, Croghan, Ivana T, Danaher, Brian G, Schroeder, Darrell R. Comparative effectiveness of the nicotine lozenge and tobacco-free snuff for smokeless tobacco reduction. Addictive behaviors. 2013;38:2140. | RCTs that did not provide data on the outcome of interest (i.e., arrhythmia, nonfatal myocardial infarction, nonfatal stroke, cardiovascular death) during the treatment period. |
| 866 | Perkins, Kenneth A, Karelitz, Joshua L, Jao, Nancy C. Optimal carbon monoxide criteria to confirm 24-hr smoking abstinence. Nicotine & tobacco research : official journal of the Society for Research on Nicotine and Tobacco. 2013;15:978. | RCTs that did not include a nicotine versus no nicotine intervention. |
| 867 | Impey, D, Chique-Alfonzo, M, Shah, D, Fisher, D J, Knott, V J. Effects of nicotine on visuospatial attentional orienting in non-smokers. Pharmacology, biochemistry, and behavior. 2013;106:1. | RCTs that did not provide data on the outcome of interest (i.e., arrhythmia, nonfatal myocardial infarction, nonfatal stroke, cardiovascular death) during the treatment period. |
| 869 | Negoescu, R.M., Bunea, M., Ungurean, C.. Smoking cessation: NRT or educated volition? Best, both of them. European Journal of Preventive Cardiology. 2013;20:S90. | RCTs that did not provide data on the outcome of interest (i.e., arrhythmia, nonfatal myocardial infarction, nonfatal stroke, cardiovascular death) during the treatment period. |
| 871 | Ghiasi, Faramarz, Farhang, Sara, Farnam, Alireza, Safikhanlou, Salman. The short term effect of nicotine abstinence on visuospatial working memory in smoking patients with schizophrenia. Nordic journal of psychiatry. 2013;67:104. | RCTs that did not provide data on the outcome of interest (i.e., arrhythmia, nonfatal myocardial infarction, nonfatal stroke, cardiovascular death) during the treatment period. |
| 872 | Weimer, Katja, Horing, Björn, Walentin, Susanne, Winkler, Simone, Klosterhalfen, Sibylle, Zipfel, Stephan, Enck, Paul. Nicotine stimulus expectancy but not nicotine affects cognitive performance in healthy smokers and non-smokers. Psychosomatic Medicine. 2013;75:A. | RCTs that did not provide data on the outcome of interest (i.e., arrhythmia, nonfatal myocardial infarction, nonfatal stroke, cardiovascular death) during the treatment period. |
| 875 | Smith, Stevens S, Keller, Paula A, Kobinsky, Kate H, Baker, Timothy B, Fraser, David L, Bush, Terry, Magnusson, Brooke, Zbikowski, Susan M, McAfee, Timothy A, Fiore, Michael C. Enhancing tobacco quitline effectiveness: identifying a superior pharmacotherapy adjuvant. Nicotine & tobacco research : official journal of the Society for Research on Nicotine and Tobacco. 2013;15:718. | RCTs that did not include a nicotine versus no nicotine intervention. |
| 878 | Koychev, Ivan, McMullen, Katrina, Lees, Jane, Dadhiwala, Rukiya, Grayson, Lois, Perry, Charlotte, Schmechtig, Anne, Walters, James, Craig, Kevin J., Dawson, Gerard R., Dourish, Colin T., Ettinger, Ulrich, Wilkinson, Lawrence, Williams, Steve, Deakin, John Francis William, Barkus, Emma. Differential effect of amisulpride on cognition in schizotypy: Validation of models for the early identification of cognitive enhancing agents. The Lancet. 2013;381:S59. | RCTs that did not provide data on the outcome of interest (i.e., arrhythmia, nonfatal myocardial infarction, nonfatal stroke, cardiovascular death) during the treatment period. |
| 880 | Ward, Kenneth D, Asfar, Taghrid, Al Ali, Radwan, Rastam, Samer, Weg, Mark W Vander, Eissenberg, Thomas, Maziak, Wasim. Randomized trial of the effectiveness of combined behavioral/pharmacological smoking cessation treatment in Syrian primary care clinics. Addiction (Abingdon, England). 2013;108:394. | RCTs that did not provide data on the outcome of interest (i.e., arrhythmia, nonfatal myocardial infarction, nonfatal stroke, cardiovascular death) during the treatment period. |
| 881 | Rose, Emma Jane, Ross, Thomas J, Salmeron, Betty Jo, Lee, Mary, Shakleya, Diaa M, Huestis, Marilyn A, Stein, Elliot A. Acute nicotine differentially impacts anticipatory valence- and magnitude-related striatal activity. Biological psychiatry. 2013;73:280. | RCTs that did not provide data on the outcome of interest (i.e., arrhythmia, nonfatal myocardial infarction, nonfatal stroke, cardiovascular death) during the treatment period. |
| 882 | Julian, Mark W, Shao, Guohong, Schlesinger, Larry S, Huang, Qin, Cosmar, David G, Bhatt, Nitin Y, Culver, Daniel A, Baughman, Robert P, Wood, Karen L, Crouser, Elliott D. Nicotine treatment improves Toll-like receptor 2 and Toll-like receptor 9 responsiveness in active pulmonary sarcoidosis. Chest. 2013;143:461. | RCTs that did not provide data on the outcome of interest (i.e., arrhythmia, nonfatal myocardial infarction, nonfatal stroke, cardiovascular death) during the treatment period. |
| 886 | Shiffman, Saul, Sembower, Mark A, Rohay, Jeffrey M, Gitchell, Joe G, Garvey, Arthur J. Assigning dose of nicotine gum by time to first cigarette. Nicotine & tobacco research : official journal of the Society for Research on Nicotine and Tobacco. 2013;15:407. | RCTs that did not provide data on the outcome of interest (i.e., arrhythmia, nonfatal myocardial infarction, nonfatal stroke, cardiovascular death) during the treatment period. |
| 889 | Bergen, Andrew W, Javitz, Harold S, Krasnow, Ruth, Nishita, Denise, Michel, Martha, Conti, David V, Liu, Jinghua, Lee, Won, Edlund, Christopher K, Hall, Sharon, Kwok, Pui-Yan, Benowitz, Neal L, Baker, Timothy B, Tyndale, Rachel F, Lerman, Caryn, Swan, Gary E. Nicotinic acetylcholine receptor variation and response to smoking cessation therapies. Pharmacogenetics and genomics. 2013;23:94. | Review articles, systematic reviews, and meta-analyses. |
| 899 | Heydari, G., Talischi, F., Batmanghelidj, E., Pajooh, M.R., Boroomand, A., Zamani, M., Salehi, A., Maddah, S.. Dual addictions, parallel treatments: Nicotine replacement therapy for patients receiving methadone treatment in the Islamic Republic of Iran. Eastern Mediterranean Health Journal. 2013;19:25. | RCTs that did not provide data on the outcome of interest (i.e., arrhythmia, nonfatal myocardial infarction, nonfatal stroke, cardiovascular death) during the treatment period. |
| 902 | Zincir, Selma Bozkurt, Zincir, Serkan, Kaymak, Esra, Sunbul, Esra Aydin. Comparison of the effectiveness of varenicline, extended-release bupropion and nicotine replacement therapy on the success and the maintenance of a smoking cessation program. Klinik Psikofarmakoloji Bulteni. 2013;23:224. | RCTs that did not provide data on the outcome of interest (i.e., arrhythmia, nonfatal myocardial infarction, nonfatal stroke, cardiovascular death) during the treatment period. |
| 906 | Fisher, Derek J, Knobelsdorf, Amy, Jaworska, Natalia, Daniels, Richelle, Knott, Verner J. Effects of nicotine on electroencephalographic (EEG) and behavioural measures of visual working memory in non-smokers during a dual-task paradigm. Pharmacology, biochemistry, and behavior. 2013;103:494. | RCTs that did not provide data on the outcome of interest (i.e., arrhythmia, nonfatal myocardial infarction, nonfatal stroke, cardiovascular death) during the treatment period. |
| 909 | Sharifirad, Gholam Reza, Eslami, Ahmad Ali, Charkazi, Abdurrahman, Mostafavi, Firozeh, Shahnazi, Hossein. The effect of individual counseling, line follow-up, and free nicotine replacement therapy on smoking cessation in the samples of Iranian smokers: Examination of transtheoretical model. Journal of research in medical sciences : the official journal of Isfahan University of Medical Sciences. 2012;17:1128. | RCTs that did not provide data on the outcome of interest (i.e., arrhythmia, nonfatal myocardial infarction, nonfatal stroke, cardiovascular death) during the treatment period. |
| 912 | Kochunov, Peter, Stein, Elliot A., Hong, Elliot. Acute nicotine administration improves white matter integrity and associated attention performance. Neuropsychopharmacology. 2012;38:S134. | Duplicate articles or articles with the exact same study outcome data as another published article. |
| 914 | Lee, W, Bergen, A W, Swan, G E, Li, D, Liu, J, Thomas, P, Tyndale, R F, Benowitz, N L, Lerman, C, Conti, D V. Gender-stratified gene and gene-treatment interactions in smoking cessation. The pharmacogenomics journal. 2012;12:521. | Observational studies (e.g., cohort, case-control, and cross-sectional studies). |
| 916 | Moran, Lauren V, Sampath, Hemalatha, Stein, Elliot A, Hong, L Elliot. Insular and anterior cingulate circuits in smokers with schizophrenia. Schizophrenia research. 2012;142:223. | RCTs that did not provide data on the outcome of interest (i.e., arrhythmia, nonfatal myocardial infarction, nonfatal stroke, cardiovascular death) during the treatment period. |
| 922 | Fenech, Mary A., Reid, Carol. A smoking cessation intervention for people with chronic hepatitis C: A randomised controlled trial. Hepatology. 2012;56:229A. | RCTs that did not provide data on the outcome of interest (i.e., arrhythmia, nonfatal myocardial infarction, nonfatal stroke, cardiovascular death) during the treatment period. |
| 927 | Knott, Verner, Shah, Dhrasti, Millar, Anne, McIntosh, Judy, Fisher, Derek, Blais, Crystal, Ilivitsky, Vadim. Nicotine, Auditory Sensory Memory, and sustained Attention in a Human Ketamine Model of Schizophrenia: Moderating Influence of a Hallucinatory Trait. Frontiers in pharmacology. 2012;3:172. | RCTs that did not provide data on the outcome of interest (i.e., arrhythmia, nonfatal myocardial infarction, nonfatal stroke, cardiovascular death) during the treatment period. |
| 936 | Heydari, Gholamreza, Tafti, Saeid Fallah. Quit smoking with Champix: Parallel, randomised clinical trial of efficacy for the first time in Iran. European Respiratory Journal. 2012; 40(S56):1378. | RCTs that did not provide data on the outcome of interest (i.e., arrhythmia, nonfatal myocardial infarction, nonfatal stroke, cardiovascular death) during the treatment period. |
| 937 | Tønnesen, Philip, Lauri, Hans, Perfekt, Roland, Mann, Karl, Batra, Anil. Efficacy of a nicotine mouth spray in smoking cessation: a randomised, double-blind trial. The European respiratory journal. 2012;40:548. | RCTs that did not provide data on the outcome of interest (i.e., arrhythmia, nonfatal myocardial infarction, nonfatal stroke, cardiovascular death) during the treatment period. |
| 938 | Berlin, Ivan, Hunneyball, Ian M, Greiling, Doris, Jones, Stephen P, Fuder, Hermann, Stahl, Hans-Detlev. A selective reversible monoamine oxidase B inhibitor in smoking cessation: effects on its own and in association with transdermal nicotine patch. Psychopharmacology. 2012;223:89. | RCTs that did not provide data on the outcome of interest (i.e., arrhythmia, nonfatal myocardial infarction, nonfatal stroke, cardiovascular death) during the treatment period. |
| 943 | Dawkins, Lynne, Turner, John, Hasna, Surrayyah, Soar, Kirstie. The electronic-cigarette: effects on desire to smoke, withdrawal symptoms and cognition. Addictive behaviors. 2012;37:970. | RCTs that did not provide data on the outcome of interest (i.e., arrhythmia, nonfatal myocardial infarction, nonfatal stroke, cardiovascular death) during the treatment period. |
| 950 | Eades, Sandra J, Sanson-Fisher, Rob W, Wenitong, Mark, Panaretto, Katie, D'Este, Catherine, Gilligan, Conor, Stewart, Jessica. An intensive smoking intervention for pregnant Aboriginal and Torres Strait Islander women: a randomised controlled trial. The Medical journal of Australia. 2012;197:42. | RCTs that did not provide data on the outcome of interest (i.e., arrhythmia, nonfatal myocardial infarction, nonfatal stroke, cardiovascular death) during the treatment period. |
| 951 | Ferguson, Stuart G, Gitchell, Joseph G, Shiffman, Saul. Continuing to wear nicotine patches after smoking lapses promotes recovery of abstinence. Addiction (Abingdon, England). 2012;107:1349. | RCTs that did not provide data on the outcome of interest (i.e., arrhythmia, nonfatal myocardial infarction, nonfatal stroke, cardiovascular death) during the treatment period. |
| 954 | Locatelli, Isabella, Clair, Carole, Collet, Tinh-Hai, Rodondi, Nicolas, Cornuz, Jacques. The interaction between gender and amount of smoking in the prediction of one-year weight gain after smoking cessation. Journal of General Internal Medicine. 2012;27:S333. | RCTs that did not provide data on the outcome of interest (i.e., arrhythmia, nonfatal myocardial infarction, nonfatal stroke, cardiovascular death) during the treatment period. |
| 961 | Barrett, Sean P, Darredeau, Christine. The acute effects of nicotine on the subjective and behavioural responses to denicotinized tobacco in dependent smokers. Behavioural pharmacology. 2012;23:221. | RCTs that did not provide data on the outcome of interest (i.e., arrhythmia, nonfatal myocardial infarction, nonfatal stroke, cardiovascular death) during the treatment period. |
| 962 | Lee, Susan M., Jones, Philip, Morley-Forster, Patricia K., Landry, Jennifer, Buhrmann, Ozzie. Effectiveness of a perioperative smoking cessation program: An RCT. Canadian Journal of Anesthesia. 2012; 59:92. | Duplicate articles or articles with the exact same study outcome data as another published article. |
| 968 | Heydari, Gholamreza. Quit smoking with champix: Parallel, randomised clinical trial of efficacy for the first time in Iran. Circulation. 2012;125:e895. | RCTs that did not provide data on the outcome of interest (i.e., arrhythmia, nonfatal myocardial infarction, nonfatal stroke, cardiovascular death) during the treatment period. |
| 969 | Carmody, Timothy P, Delucchi, Kevin, Duncan, Carol L, Banys, Peter, Simon, Joel A, Solkowitz, Sharon N, Huggins, Joy, Lee, Sharon K, Hall, Sharon M. Intensive intervention for alcohol-dependent smokers in early recovery: a randomized trial. Drug and alcohol dependence. 2012;122:186. | RCTs that did not provide data on the outcome of interest (i.e., arrhythmia, nonfatal myocardial infarction, nonfatal stroke, cardiovascular death) during the treatment period. |
| 970 | Marine Gambaryan, M.H., Kalinina, A.M., Grigoryan, T.A., Vigodin, V.A.. Intervention strategies for smoking cessation in primary health care in Russia: Opportunities and challenges. European Journal of Preventive Cardiology. 2012;19:S38. | RCTs that did not provide data on the outcome of interest (i.e., arrhythmia, nonfatal myocardial infarction, nonfatal stroke, cardiovascular death) during the treatment period. |
| 972 | Ferketich, Amy K, Otterson, Gregory A, King, Mark, Hall, Nathan, Browning, Kristine K, Wewers, Mary Ellen. A pilot test of a combined tobacco dependence treatment and lung cancer screening program. Lung cancer (Amsterdam, Netherlands). 2012;76:211. | RCTs that did not provide data on the outcome of interest (i.e., arrhythmia, nonfatal myocardial infarction, nonfatal stroke, cardiovascular death) during the treatment period. |
| 975 | Ferguson, Janet, Docherty, Graeme, Bauld, Linda, Lewis, Sarah, Lorgelly, Paula, Boyd, Kathleen Anne, McEwen, Andy, Coleman, Tim. Effect of offering different levels of support and free nicotine replacement therapy via an English national telephone quitline: randomised controlled trial. BMJ (Clinical research ed.). 2012;344:e1696. | RCTs that did not provide data on the outcome of interest (i.e., arrhythmia, nonfatal myocardial infarction, nonfatal stroke, cardiovascular death) during the treatment period. |
| 978 | Schnoll, Robert A, Wileyto, E Paul, Lerman, Caryn. Extended duration therapy with transdermal nicotine may attenuate weight gain following smoking cessation. Addictive behaviors. 2012;37:565. | RCTs that did not provide data on the outcome of interest (i.e., arrhythmia, nonfatal myocardial infarction, nonfatal stroke, cardiovascular death) during the treatment period. |
| 979 | Brunner Frandsen, Nicole, Sørensen, Margit, Hyldahl, Tanja Kirstine, Henriksen, Rikke Mitzi, Bak, Søren. Smoking cessation intervention after ischemic stroke or transient ischemic attack. A randomized controlled pilot trial. Nicotine & tobacco research : official journal of the Society for Research on Nicotine and Tobacco. 2012;14:443. | RCTs that did not provide data on the outcome of interest (i.e., arrhythmia, nonfatal myocardial infarction, nonfatal stroke, cardiovascular death) during the treatment period. |
| 983 | Singh, Prabhat K., Gautam, Sujeet K., Gupta, Devendra, Agarwal, Anil, Singh, Uttam. Evaluation of trans-dermal nicotine patch for attenuation of venous cannulation pain. British Journal of Anaesthesia. 2012;108:ii350. | RCTs that did not provide data on the outcome of interest (i.e., arrhythmia, nonfatal myocardial infarction, nonfatal stroke, cardiovascular death) during the treatment period. |
| 987 | Coleman, Tim, Cooper, Sue, Thornton, James G, Grainge, Matthew J, Watts, Kim, Britton, John, Lewis, Sarah. A randomized trial of nicotine-replacement therapy patches in pregnancy. The New England journal of medicine. 2012;366:808. | RCTs that did not provide data on the outcome of interest (i.e., arrhythmia, nonfatal myocardial infarction, nonfatal stroke, cardiovascular death) during the treatment period. |
| 989 | Heydari, G, Talischi, F, Tafti, S F, Masjedi, M R. Quitting smoking with varenicline: parallel, randomised efficacy trial in Iran. The international journal of tuberculosis and lung disease : the official journal of the International Union against Tuberculosis and Lung Disease. 2012;16:268. | RCTs that did not provide data on the outcome of interest (i.e., arrhythmia, nonfatal myocardial infarction, nonfatal stroke, cardiovascular death) during the treatment period. |
| 991 | Bolt, Daniel M, Piper, Megan E, Theobald, Wendy E, Baker, Timothy B. Why two smoking cessation agents work better than one: role of craving suppression. Journal of consulting and clinical psychology. 2012;80:54. | RCTs that did not provide data on the outcome of interest (i.e., arrhythmia, nonfatal myocardial infarction, nonfatal stroke, cardiovascular death) during the treatment period. |
| 992 | Newhouse, P, Kellar, K, Aisen, P, White, H, Wesnes, K, Coderre, E, Pfaff, A, Wilkins, H, Howard, D, Levin, E D. Nicotine treatment of mild cognitive impairment: a 6-month double-blind pilot clinical trial. Neurology. 2012;78:91. | RCTs that did not provide data on the outcome of interest (i.e., arrhythmia, nonfatal myocardial infarction, nonfatal stroke, cardiovascular death) during the treatment period. |
| 995 | Pai, Anuradha, Prasad, Shesha. Attempting tobacco cessation--an oral physician's perspective. Asian Pacific journal of cancer prevention : APJCP. 2012;13:4973. | RCTs that did not provide data on the outcome of interest (i.e., arrhythmia, nonfatal myocardial infarction, nonfatal stroke, cardiovascular death) during the treatment period. |
| 996 | Knott, V.J., Millar, A.M., McIntosh, J.F., Shah, D.K., Fisher, D.J., Blais, C.M., Ilivitsky, V., Horn, E.. Neurochemical modulation of auditory processing in healthy controls stratified for low and high auditory hallucinatory experiences. European Psychiatry. 2012; 27:1. | RCTs that did not provide data on the outcome of interest (i.e., arrhythmia, nonfatal myocardial infarction, nonfatal stroke, cardiovascular death) during the treatment period. |
| 997 | Zincir, Selma Bozkurt, Kaymak, Esra, Sünbül, Esra Aydin, Zincir, Nihat. Comparison of the effects of varenicline, bupropion and nicotine replacement therapy on smoking cessation program. Klinik Psikofarmakoloji Bulteni. 2012;22:S35. | RCTs that did not provide data on the outcome of interest (i.e., arrhythmia, nonfatal myocardial infarction, nonfatal stroke, cardiovascular death) during the treatment period. |
| 1002 | Cabioǧlu, Mehmet Tuǧrul, Kutlu, Ruhuşen, Albayrak, Canan, Marakoǧlu, Kamile, Ergene, Neyhan. Changes in the levels of serum beta endorphin, serotonin, adrenaline, noradrenaline and dopamine during smoking cessation by electroacupuncture and nicotine patch. Turkiye Klinikleri Cardiovascular Sciences. 2012;24:95. | RCTs that did not provide data on the outcome of interest (i.e., arrhythmia, nonfatal myocardial infarction, nonfatal stroke, cardiovascular death) during the treatment period. |
| 1006 | Sutherland, Matthew T., Carroll, Allison J., Jo Salmeron, Betty, Ross, Thomas J., Stein, Elliot A.. Insula functional connectivity with default-mode network is modulated by varenicline and nicotine in abstinent smokers. Neuropsychopharmacology. 2011;36:S367. | RCTs that did not provide data on the outcome of interest (i.e., arrhythmia, nonfatal myocardial infarction, nonfatal stroke, cardiovascular death) during the treatment period. |
| 1010 | Joseph, Anne M, Fu, Steven S, Lindgren, Bruce, Rothman, Alexander J, Kodl, Molly, Lando, Harry, Doyle, Brandon, Hatsukami, Dorothy. Chronic disease management for tobacco dependence: a randomized, controlled trial. Archives of internal medicine. 2011;171:1894. | RCTs that did not provide data on the outcome of interest (i.e., arrhythmia, nonfatal myocardial infarction, nonfatal stroke, cardiovascular death) during the treatment period. |
| 1011 | Carpenter, Matthew J, Hughes, John R, Gray, Kevin M, Wahlquist, Amy E, Saladin, Michael E, Alberg, Anthony J. Nicotine therapy sampling to induce quit attempts among smokers unmotivated to quit: a randomized clinical trial. Archives of internal medicine. 2011;171:1901. | RCTs that did not provide data on the outcome of interest (i.e., arrhythmia, nonfatal myocardial infarction, nonfatal stroke, cardiovascular death) during the treatment period. |
| 1017 | Grainge, Matthew, Coleman, Tim, Cooper, Sue, Thornton, Jim, Watts, Kim, Britton, John, Lewis, Sarah. Double-blind, placebo-controlled, randomised trial of nicotine replacement therapy patches in pregnancy. Journal of Perinatal Medicine. 2011; 39(S1):A172. | RCTs that did not provide data on the outcome of interest (i.e., arrhythmia, nonfatal myocardial infarction, nonfatal stroke, cardiovascular death) during the treatment period. |
| 1018 | Wardle, Margaret C, Munafò, Marcus R, de Wit, Harriet. Effect of social stress during acute nicotine abstinence. Psychopharmacology. 2011;218:39. | RCTs that did not provide data on the outcome of interest (i.e., arrhythmia, nonfatal myocardial infarction, nonfatal stroke, cardiovascular death) during the treatment period. |
| 1030 | Reid, Malcolm S, Jiang, Huiping, Fallon, Bryan, Sonne, Susan, Rinaldi, Paul, Turrigiano, Eva, Arfken, Cynthia, Robinson, James, Rotrosen, John, Nunes, Edward V. Smoking cessation treatment among patients in community-based substance abuse rehabilitation programs: exploring predictors of outcome as clues toward treatment improvement. The American journal of drug and alcohol abuse. 2011;37:472. | RCTs that did not provide data on the outcome of interest (i.e., arrhythmia, nonfatal myocardial infarction, nonfatal stroke, cardiovascular death) during the treatment period. |
| 1031 | Knott, Verner J, Millar, Anne M, McIntosh, Judy F, Shah, Dhrasti K, Fisher, Derek J, Blais, Crystal M, Ilivitsky, Vadim, Horn, Ed. Separate and combined effects of low dose ketamine and nicotine on behavioural and neural correlates of sustained attention. Biological psychology. 2011;88:83. | RCTs that did not provide data on the outcome of interest (i.e., arrhythmia, nonfatal myocardial infarction, nonfatal stroke, cardiovascular death) during the treatment period. |
| 1035 | Mobascher, A., Warbrick, T., Brinkmeyer, J., Musso, F., Stoecker, T., Shah, N.J., Vossel, S., Winterer, G.. Nicotine effects on attention in schizophrenia: A simultaneous EEG-fMRI study. European Neuropsychopharmacology. 2011;21:S515. | RCTs that did not provide data on the outcome of interest (i.e., arrhythmia, nonfatal myocardial infarction, nonfatal stroke, cardiovascular death) during the treatment period. |
| 1036 | Valencia, Borja, Ortega, Francisco, Vellisco, Arturo, Márquez-Martín, Eduardo, Campos, Jose Luis López, Rodríguez, Ana María, Ferrer, Marta, Cejudo, Pilar, Barrot, Emilia. Effectiveness of a cognitive orientation program with and without nicotine replacement therapy in stopping smoking in hospitalised patients. European Respiratory Journal. 2011; 38(S55):4224. | Articles in which the abstract and full text was non-English. |
| 1038 | Williams, David M, Dunsiger, Shira, Whiteley, Jessica A, Ussher, Michael H, Ciccolo, Joseph T, Jennings, Ernestine G. Acute effects of moderate intensity aerobic exercise on affective withdrawal symptoms and cravings among women smokers. Addictive behaviors. 2011;36:894. | RCTs that did not include a nicotine versus no nicotine intervention. |
| 1039 | Fukada, Tomoko, Iwakiri, Hiroko, Ozaki, Makoto. A randomised double-blind crossover trial of the potential analgesic effect of a transdermal nicotine patch in non-smokers based on objective and subjective assessment. European journal of anaesthesiology. 2011;28:592. | RCTs that did not provide data on the outcome of interest (i.e., arrhythmia, nonfatal myocardial infarction, nonfatal stroke, cardiovascular death) during the treatment period. |
| 1050 | Bernstein, Steven L, Bijur, Polly, Cooperman, Nina, Jearld, Saba, Arnsten, Julia H, Moadel, Alyson, Gallagher, E John. A randomized trial of a multicomponent cessation strategy for emergency department smokers. Academic emergency medicine : official journal of the Society for Academic Emergency Medicine. 2011;18:575. | RCTs that did not provide data on the outcome of interest (i.e., arrhythmia, nonfatal myocardial infarction, nonfatal stroke, cardiovascular death) during the treatment period. |
| 1051 | Chan, Sophia S C, Leung, Doris Y P, Abdullah, Abu S M, Wong, Vivian T, Hedley, Anthony J, Lam, Tai-Hing. A randomized controlled trial of a smoking reduction plus nicotine replacement therapy intervention for smokers not willing to quit smoking. Addiction (Abingdon, England). 2011;106:1155. | RCTs that did not provide data on the outcome of interest (i.e., arrhythmia, nonfatal myocardial infarction, nonfatal stroke, cardiovascular death) during the treatment period. |
| 1052 | Walker, Natalie, Howe, Colin, Bullen, Chris, Grigg, Michele, Glover, Marewa, McRobbie, Hayden, Laugesen, Murray, Jiang, Joy, Chen, Mei-Hua, Whittaker, Robyn, Rodgers, Anthony. Does improved access and greater choice of nicotine replacement therapy affect smoking cessation success? Findings from a randomized controlled trial. Addiction (Abingdon, England). 2011;106:1176. | RCTs that did not include a nicotine versus no nicotine intervention. |
| 1056 | Richardson, Elizabeth J., Richards, J. Scott, Stewart, Christopher C., Ness, Timothy J.. Effects of nicotine on spinal cord injury related pain: A randomized, double-blind, placebo-controlled crossover trial. Topics in Spinal Cord Injury Rehabilitation. 2011;16:31. | RCTs that did not provide data on the outcome of interest (i.e., arrhythmia, nonfatal myocardial infarction, nonfatal stroke, cardiovascular death) during the treatment period. |
| 1058 | Rigotti, Nancy A., Bitton, Asaf, Kelley, Jennifer K., Hoeppner, Bettina B., Levy, Douglas E., Mort, Elizabeth. Supplementing office-based care with a populationbased direct-to-smoker outreach intervention offering free treatment to smokers in a community health center: A randomized controlled trial. Journal of General Internal Medicine. 2011;26:S33. | RCTs that did not include a nicotine versus no nicotine intervention. |
| 1061 | Yagoubian, Benjamin, Akkara, Joseph, Afzali, Payam, Alfi, David M, Olson, Luke, Conell-Price, Jessamyn, Yeh, Janet, Eisig, Sidney B, Flood, Pamela. Nicotine nasal spray as an adjuvant analgesic for third molar surgery. Journal of oral and maxillofacial surgery : official journal of the American Association of Oral and Maxillofacial Surgeons. 2011;69:1316. | RCTs that did not provide data on the outcome of interest (i.e., arrhythmia, nonfatal myocardial infarction, nonfatal stroke, cardiovascular death) during the treatment period. |
| 1067 | Allen, Michael H, Debanné, Martin, Lazignac, Coralie, Adam, Eric, Dickinson, L Miriam, Damsa, Cristian. Effect of nicotine replacement therapy on agitation in smokers with schizophrenia: a double-blind, randomized, placebo-controlled study. The American journal of psychiatry. 2011;168:395. | RCTs that did not provide data on the outcome of interest (i.e., arrhythmia, nonfatal myocardial infarction, nonfatal stroke, cardiovascular death) during the treatment period. |
| 1070 | Hilberink, Sander R, Jacobs, Johanna E, Breteler, Marinus H M, de Vries, Hein, Grol, Richard P T M. General practice counseling for patients with chronic obstructive pulmonary disease to quit smoking: impact after 1 year of two complex interventions. Patient education and counseling. 2011;83:120. | RCTs that did not include a nicotine versus no nicotine intervention. |
| 1072 | Ferguson, Stuart G, Shiffman, Saul, Rohay, Jeffrey M, Gitchell, Joseph G, Garvey, Arthur J. Effect of compliance with nicotine gum dosing on weight gained during a quit attempt. Addiction (Abingdon, England). 2011;106:651. | RCTs that did not provide data on the outcome of interest (i.e., arrhythmia, nonfatal myocardial infarction, nonfatal stroke, cardiovascular death) during the treatment period. |
| 1073 | Czarnetzki, Christoph, Schiffer, Eduardo, Lysakowski, Christopher, Haller, Guy, Bertrand, Daniel, Tramèr, Martin R. Transcutaneous nicotine does not prevent postoperative nausea and vomiting: a randomized controlled trial. British journal of clinical pharmacology. 2011;71:383. | RCTs that did not provide data on the outcome of interest (i.e., arrhythmia, nonfatal myocardial infarction, nonfatal stroke, cardiovascular death) during the treatment period. |
| 1075 | Moran, Lauren V., Sampath, Hemalatha, Yang, Yihong, Ross, Thomas J., Jo Salmeron, Betty, Buchholz, Brittany, Thaker, Gunvant K., Stein, Elliot A., Hong, Elliot. Insula, dorsal anterior cingulate and ventral striatum resting state functional connectivity network in nicotine administration, nicotine addiction and schizophrenia. Schizophrenia Bulletin. 2011;37:147. | RCTs that did not provide data on the outcome of interest (i.e., arrhythmia, nonfatal myocardial infarction, nonfatal stroke, cardiovascular death) during the treatment period. |
| 1076 | Hong, L Elliot, Schroeder, Matthew, Ross, Thomas J, Buchholz, Brittany, Salmeron, Betty Jo, Wonodi, Ikwunga, Thaker, Gunvant K, Stein, Elliot A. Nicotine enhances but does not normalize visual sustained attention and the associated brain network in schizophrenia. Schizophrenia bulletin. 2011;37:416. | RCTs that did not provide data on the outcome of interest (i.e., arrhythmia, nonfatal myocardial infarction, nonfatal stroke, cardiovascular death) during the treatment period. |
| 1081 | Piper, Megan E, Cook, Jessica W, Schlam, Tanya R, Jorenby, Douglas E, Baker, Timothy B. Anxiety diagnoses in smokers seeking cessation treatment: relations with tobacco dependence, withdrawal, outcome and response to treatment. Addiction (Abingdon, England). 2011;106:418. | RCTs that did not provide data on the outcome of interest (i.e., arrhythmia, nonfatal myocardial infarction, nonfatal stroke, cardiovascular death) during the treatment period. |
| 1083 | Knott, Verner, Heenan, Adam, Shah, Dhrasti, Bolton, Kiley, Fisher, Derek, Villeneuve, Crystal. Electrophysiological evidence of nicotine's distracter-filtering properties in non-smokers. Journal of psychopharmacology (Oxford, England). 2011;25:239. | RCTs that did not provide data on the outcome of interest (i.e., arrhythmia, nonfatal myocardial infarction, nonfatal stroke, cardiovascular death) during the treatment period. |
| 1088 | Ortega, Francisco, Vellisco, Arturo, Márquez, Eduardo, López-Campos, José Luis, Rodríguez, Ana, de los Ángeles Sánchez, María, Barrot, Emilia, Cejudo, Pilar. Effectiveness of a cognitive orientation program with and without nicotine replacement therapy in stopping smoking in hospitalised patients. Archivos de bronconeumologia. 2011;47:3. | Articles in which the abstract and full text was non-English. |
| 1089 | Barrett, Sean P, Campbell, Mallory L, Temporale, Kirsten, Good, Kim Berley P. The acute effect of Swedish-style snus on cigarette craving and self-administration in male and female smokers. Human psychopharmacology. 2011;26:58. | RCTs that did not provide data on the outcome of interest (i.e., arrhythmia, nonfatal myocardial infarction, nonfatal stroke, cardiovascular death) during the treatment period. |
| 1095 | Arias, Albert J., Feinn, Richard, Kranzler, Henry R., Oncken, Cheryl. Allelic association of the GluR5 kainate receptor subunit gene GRIK1 with adverse effects of topiramate. Neuropsychopharmacology. 2010;35:S114. | RCTs that did not provide data on the outcome of interest (i.e., arrhythmia, nonfatal myocardial infarction, nonfatal stroke, cardiovascular death) during the treatment period. |
| 1102 | Darredeau, Christine, Barrett, Sean P. The role of nicotine content information in smokers' subjective responses to nicotine and placebo inhalers. Human psychopharmacology. 2010;25:577. | RCTs that did not provide data on the outcome of interest (i.e., arrhythmia, nonfatal myocardial infarction, nonfatal stroke, cardiovascular death) during the treatment period. |
| 1105 | Sørensen, Lars Tue, Toft, Birgitte G, Rygaard, Jørgen, Ladelund, Steen, Paddon, Maria, James, Tim, Taylor, Richard, Gottrup, Finn. Effect of smoking, smoking cessation, and nicotine patch on wound dimension, vitamin C, and systemic markers of collagen metabolism. Surgery. 2010;148:982. | RCTs that did not provide data on the outcome of interest (i.e., arrhythmia, nonfatal myocardial infarction, nonfatal stroke, cardiovascular death) during the treatment period. |
| 1106 | Reid, R.D., Armstrong, A., McDonnell, L., Aitken, D.A., Robert, L., LaRue, A., Laroche, M., Pipe, A.L.. Varenicline versus transdermal nicotine patch for smoking cessation in patients with coronary heart disease: A pilot randomized trial. Canadian Journal of Cardiology. 2010;26:53D. | RCTs that did not provide data on the outcome of interest (i.e., arrhythmia, nonfatal myocardial infarction, nonfatal stroke, cardiovascular death) during the treatment period. |
| 1109 | Dulude, Louise, Labelle, Alain, Knott, Verner J. Acute nicotine alteration of sensory memory impairment in smokers with schizophrenia. Journal of clinical psychopharmacology. 2010;30:541. | RCTs that did not provide data on the outcome of interest (i.e., arrhythmia, nonfatal myocardial infarction, nonfatal stroke, cardiovascular death) during the treatment period. |
| 1110 | Okuyemi, Kolawole S, Thomas, Janet L, Warren, Jennifer, Guo, Hongfei, Ahluwalia, Jasjit S. Relationship between smoking reduction and cessation among light smokers. Nicotine & tobacco research : official journal of the Society for Research on Nicotine and Tobacco. 2010;12:1005. | RCTs that did not provide data on the outcome of interest (i.e., arrhythmia, nonfatal myocardial infarction, nonfatal stroke, cardiovascular death) during the treatment period. |
| 1116 | Okuyemi, Kolawole S, Zheng, Hui, Guo, Hongfei, Ahluwalia, Jasjit S. Predictors of adherence to nicotine gum and counseling among African-American light smokers. Journal of general internal medicine. 2010;25:969. | RCTs that did not provide data on the outcome of interest (i.e., arrhythmia, nonfatal myocardial infarction, nonfatal stroke, cardiovascular death) during the treatment period. |
| 1117 | Unbehaun, T., Jaehne, A., Feige, B., Riemann, D.. Nicotine and sleep - studying smokers and healthy non-smokers during nicotine consumption and withdrawal. Journal of Sleep Research. 2010;19:107. | RCTs that did not provide data on the outcome of interest (i.e., arrhythmia, nonfatal myocardial infarction, nonfatal stroke, cardiovascular death) during the treatment period. |
| 1118 | Bailey, Steffani R, Fong, Dalea M, Bryson, Susan W, Fortmann, Stephen P, Killen, Joel D. Perceived drug assignment and treatment outcome in smokers given nicotine patch therapy. Journal of substance abuse treatment. 2010;39:150. | RCTs that did not provide data on the outcome of interest (i.e., arrhythmia, nonfatal myocardial infarction, nonfatal stroke, cardiovascular death) during the treatment period. |
| 1123 | Kras, M., Stough, C., Scholey, A., Kure, C., Camfield, D.. Hypericum perforatum, nicotine patches and combination hypericum perforatum/nicotine patches for smoking cessation. European Neuropsychopharmacology. 2010;20:S608. | RCTs that did not provide data on the outcome of interest (i.e., arrhythmia, nonfatal myocardial infarction, nonfatal stroke, cardiovascular death) during the treatment period. |
| 1126 | Ebbert, Jon O, Edmonds, Amanda, Luo, Xianghua, Jensen, Joni, Hatsukami, Dorothy K. Smokeless tobacco reduction with the nicotine lozenge and behavioral intervention. Nicotine & tobacco research : official journal of the Society for Research on Nicotine and Tobacco. 2010;12:823. | RCTs that did not provide data on the outcome of interest (i.e., arrhythmia, nonfatal myocardial infarction, nonfatal stroke, cardiovascular death) during the treatment period. |
| 1134 | McRobbie, Hayden, Thornley, Simon, Bullen, Chris, Lin, Ruey-Bin, Senior, Hugh, Laugesen, Murray, Whittaker, Robyn, Hajek, Peter. A randomized trial of the effects of two novel nicotine replacement therapies on tobacco withdrawal symptoms and user satisfaction. Addiction (Abingdon, England). 2010;105:1290. | RCTs that did not provide data on the outcome of interest (i.e., arrhythmia, nonfatal myocardial infarction, nonfatal stroke, cardiovascular death) during the treatment period. |
| 1135 | Gordon, Judith S, Andrews, Judy A, Albert, David A, Crews, Karen M, Payne, Thomas J, Severson, Herbert H. Tobacco cessation via public dental clinics: results of a randomized trial. American journal of public health. 2010;100:1307. | RCTs that did not provide data on the outcome of interest (i.e., arrhythmia, nonfatal myocardial infarction, nonfatal stroke, cardiovascular death) during the treatment period. |
| 1136 | Mumenthaler, Martin S, Benowitz, Neal L, Taylor, Joy L, Friedman, Leah, Noda, Art, Yesavage, Jerome A. Nicotine deprivation and pilot performance during simulated flight. Aviation, space, and environmental medicine. 2010;81:660. | RCTs that did not provide data on the outcome of interest (i.e., arrhythmia, nonfatal myocardial infarction, nonfatal stroke, cardiovascular death) during the treatment period. |
| 1138 | Ferguson, Stuart G, Shiffman, Saul. Effect of high-dose nicotine patch on the characteristics of lapse episodes. Health psychology : official journal of the Division of Health Psychology, American Psychological Association. 2010;29:358. | RCTs that did not provide data on the outcome of interest (i.e., arrhythmia, nonfatal myocardial infarction, nonfatal stroke, cardiovascular death) during the treatment period. |
| 1142 | Tsukahara, H., Noda, K., Uehara, Y., Saku, K.. A randomized open trial of varenicline versus nicotine patch in adult smokers: Efficacy, safety and withdrawal symptoms (the VN-seesaw study). Atherosclerosis Supplements. 2010;11:151. | Duplicate articles or articles with the exact same study outcome data as another published article. |
| 1151 | Ebbert, Jon O, Severson, Herbert H, Croghan, Ivana T, Danaher, Brian G, Schroeder, Darrell R. A pilot study of mailed nicotine lozenges with assisted self-help for the treatment of smokeless tobacco users. Addictive behaviors. 2010;35:522. | RCTs that did not provide data on the outcome of interest (i.e., arrhythmia, nonfatal myocardial infarction, nonfatal stroke, cardiovascular death) during the treatment period. |
| 1153 | Brunner-Frandsen, N.S., Bak, S.. Smoking cessation intervention after stroke or transient ischemic attack. A randomised controlled trial. Cerebrovascular Diseases. 2010;29:323. | RCTs that did not provide data on the outcome of interest (i.e., arrhythmia, nonfatal myocardial infarction, nonfatal stroke, cardiovascular death) during the treatment period. |
| 1156 | Marton, J.P., Zou, K.H., Russ, C., Willke, R.J.. Comparison of patient reported outcomes based on the minnesota nicotine withdrawal scale (MNWS) using abstinence profiles in treatments with varenicline and transdermal nicotine patch (NRT). Value in Health. 2010;13:A202. | RCTs that did not provide data on the outcome of interest (i.e., arrhythmia, nonfatal myocardial infarction, nonfatal stroke, cardiovascular death) during the treatment period. |
| 1158 | Tsukahara, Hitomi, Noda, Keita, Saku, Keijiro. A randomized controlled open comparative trial of varenicline vs nicotine patch in adult smokers: efficacy, safety and withdrawal symptoms (the VN-SEESAW study). Circulation journal : official journal of the Japanese Circulation Society. 2010;74:771. | RCTs that did not provide data on the outcome of interest (i.e., arrhythmia, nonfatal myocardial infarction, nonfatal stroke, cardiovascular death) during the treatment period. |
| 1160 | Knott, V, Millar, A, Fisher, D, Albert, P. Effects of nicotine on the amplitude and gating of the auditory P50 and its influence by dopamine D2 receptor gene polymorphism. Neuroscience. 2010;166:145. | RCTs that did not provide data on the outcome of interest (i.e., arrhythmia, nonfatal myocardial infarction, nonfatal stroke, cardiovascular death) during the treatment period. |
| 1162 | Sonne, Susan C, Nunes, Edward V, Jiang, Huiping, Tyson, Clare, Rotrosen, John, Reid, Malcolm S. The relationship between depression and smoking cessation outcomes in treatment-seeking substance abusers. The American journal on addictions. 2010;19:111. | RCTs that did not provide data on the outcome of interest (i.e., arrhythmia, nonfatal myocardial infarction, nonfatal stroke, cardiovascular death) during the treatment period. |
| 1163 | Barrett, Sean P. The effects of nicotine, denicotinized tobacco, and nicotine-containing tobacco on cigarette craving, withdrawal, and self-administration in male and female smokers. Behavioural pharmacology. 2010;21:144. | RCTs that did not provide data on the outcome of interest (i.e., arrhythmia, nonfatal myocardial infarction, nonfatal stroke, cardiovascular death) during the treatment period. |
| 1166 | Bock, Beth C, Hudmon, Karen S, Christian, James, Graham, Amanda L, Bock, Frederick R. A tailored intervention to support pharmacy-based counseling for smoking cessation. Nicotine & tobacco research : official journal of the Society for Research on Nicotine and Tobacco. 2010;12:217. | RCTs that did not provide data on the outcome of interest (i.e., arrhythmia, nonfatal myocardial infarction, nonfatal stroke, cardiovascular death) during the treatment period. |
| 1168 | Sørensen, Lars Tue, Toft, Birgitte, Rygaard, Jørgen, Ladelund, Steen, Teisner, Børge, Gottrup, Finn. Smoking attenuates wound inflammation and proliferation while smoking cessation restores inflammation but not proliferation. Wound repair and regeneration : official publication of the Wound Healing Society [and] the European Tissue Repair Society. 2010;18:186. | RCTs that did not provide data on the outcome of interest (i.e., arrhythmia, nonfatal myocardial infarction, nonfatal stroke, cardiovascular death) during the treatment period. |
| 1170 | Fisher, Derek J, Scott, Terri Lynne, Shah, Dhrasti K, Prise, Stephanie, Thompson, Mackenzie, Knott, Verner J. Light up and see: enhancement of the visual mismatch negativity (vMMN) by nicotine. Brain research. 2010;1313:162. | RCTs that did not provide data on the outcome of interest (i.e., arrhythmia, nonfatal myocardial infarction, nonfatal stroke, cardiovascular death) during the treatment period. |
| 1173 | Hukkanen, Janne, Jacob Iii, Peyton, Peng, Margaret, Dempsey, Delia, Benowitz, Neal L. Effects of nicotine on cytochrome P450 2A6 and 2E1 activities. British journal of clinical pharmacology. 2010;69:152. | RCTs that did not provide data on the outcome of interest (i.e., arrhythmia, nonfatal myocardial infarction, nonfatal stroke, cardiovascular death) during the treatment period. |
| 1177 | Croghan, Ivana T., Hurt, Richard D., Ebbert, Jon O., Croghan, Gary A., Polk Jr., Octavius D., Stella, Philip J., Novotny, Paul J., Sloan, Jeff, Loprinzi, Charles L.. Racial differences in smoking abstinence rates in a multicenter, randomized, open-label trial in the United States. Journal of Public Health. 2010;18:59. | RCTs that did not provide data on the outcome of interest (i.e., arrhythmia, nonfatal myocardial infarction, nonfatal stroke, cardiovascular death) during the treatment period. |
| 1184 | West, Robert, May, Sylvia, McEwen, Andy, McRobbie, Hayden, Hajek, Peter, Vangeli, Eleni. A randomised trial of glucose tablets to aid smoking cessation. Psychopharmacology. 2010;207:631. | RCTs that did not include a nicotine versus no nicotine intervention. |
| 1186 | Erden, Veysel, Başaranoǧlu, Gökçen, Erkalp, Kerem, Yangin, Zehra, Delatioǧlu, Hamdi, Teker, M. Gökhan, Saitoǧlu, Leyla. The effect of nicotine treatment on propofol injection pain. Trakya Universitesi Tip Fakultesi Dergisi. 2010;27:18. | RCTs that did not provide data on the outcome of interest (i.e., arrhythmia, nonfatal myocardial infarction, nonfatal stroke, cardiovascular death) during the treatment period. |
| 1187 | Smith, Stevens S, McCarthy, Danielle E, Japuntich, Sandra J, Christiansen, Bruce, Piper, Megan E, Jorenby, Douglas E, Fraser, David L, Fiore, Michael C, Baker, Timothy B, Jackson, Thomas C. Comparative effectiveness of 5 smoking cessation pharmacotherapies in primary care clinics. Archives of internal medicine. 2009;169:2148. | RCTs that did not provide data on the outcome of interest (i.e., arrhythmia, nonfatal myocardial infarction, nonfatal stroke, cardiovascular death) during the treatment period. |
| 1188 | Olson, Luke C, Hong, Daewha, Conell-Price, Jessamyn S, Cheng, Sean, Flood, Pamela. A transdermal nicotine patch is not effective for postoperative pain management in smokers: a pilot dose-ranging study. Anesthesia and analgesia. 2009;109:1987. | RCTs that did not provide data on the outcome of interest (i.e., arrhythmia, nonfatal myocardial infarction, nonfatal stroke, cardiovascular death) during the treatment period. |
| 1189 | Bolin, Kristian, Wilson, Koo, Benhaddi, Hicham, de Nigris, Enrico, Marbaix, Sophie, Mork, Ann-Christin, Aubin, Henri-Jean. Cost-effectiveness of varenicline compared with nicotine patches for smoking cessation--results from four European countries. European journal of public health. 2009;19:650. | RCTs that did not provide data on the outcome of interest (i.e., arrhythmia, nonfatal myocardial infarction, nonfatal stroke, cardiovascular death) during the treatment period. |
| 1191 | Ebbert, Jon O, Severson, Herbert H, Croghan, Ivana T, Danaher, Brian G, Schroeder, Darrell R. A randomized clinical trial of nicotine lozenge for smokeless tobacco use. Nicotine & tobacco research : official journal of the Society for Research on Nicotine and Tobacco. 2009;11:1415. | RCTs that did not provide data on the outcome of interest (i.e., arrhythmia, nonfatal myocardial infarction, nonfatal stroke, cardiovascular death) during the treatment period. |
| 1196 | Piper, Megan E, Smith, Stevens S, Schlam, Tanya R, Fiore, Michael C, Jorenby, Douglas E, Fraser, David, Baker, Timothy B. A randomized placebo-controlled clinical trial of 5 smoking cessation pharmacotherapies. Archives of general psychiatry. 2009;66:1253. | RCTs that did not provide data on the outcome of interest (i.e., arrhythmia, nonfatal myocardial infarction, nonfatal stroke, cardiovascular death) during the treatment period. |
| 1206 | Ferguson, Stuart G, Gitchell, Joseph G, Shiffman, Saul, Sembower, Mark A. Prediction of abstinence at 10 weeks based on smoking status at 2 weeks during a quit attempt: secondary analysis of two parallel, 10-week, randomized, double-blind, placebo-controlled clinical trials of 21-mg nicotine patch in adult smokers. Clinical therapeutics. 2009;31:1957. | RCTs that did not provide data on the outcome of interest (i.e., arrhythmia, nonfatal myocardial infarction, nonfatal stroke, cardiovascular death) during the treatment period. |
| 1211 | Adamopoulos, Dionysios, Argacha, Jean-François, Gujic, Marko, Preumont, Nicolas, Degaute, Jean-Paul, van de Borne, Philippe. Acute effects of nicotine on arterial stiffness and wave reflection in healthy young non-smokers. Clinical and experimental pharmacology & physiology. 2009;36:784. | RCTs that did not provide data on the outcome of interest (i.e., arrhythmia, nonfatal myocardial infarction, nonfatal stroke, cardiovascular death) during the treatment period. |
| 1220 | Hall, Sharon M, Humfleet, Gary L, Muñoz, Ricardo F, Reus, Victor I, Robbins, Julie A, Prochaska, Judith J. Extended treatment of older cigarette smokers. Addiction (Abingdon, England). 2009;104:1043. | RCTs that did not provide data on the outcome of interest (i.e., arrhythmia, nonfatal myocardial infarction, nonfatal stroke, cardiovascular death) during the treatment period. |
| 1226 | Elkader, Alexander K, Brands, Bruna, Selby, Peter, Sproule, Beth A. Methadone-nicotine interactions in methadone maintenance treatment patients. Journal of clinical psychopharmacology. 2009;29:231. | RCTs that did not provide data on the outcome of interest (i.e., arrhythmia, nonfatal myocardial infarction, nonfatal stroke, cardiovascular death) during the treatment period. |
| 1227 | Thornley, Simon, McRobbie, Hayden, Lin, Ruey-Bin, Bullen, Chris, Hajek, Peter, Laugesen, Murray, Senior, Hugh, Whittaker, Robyn. A single-blind, randomized, crossover trial of the effects of a nicotine pouch on the relief of tobacco withdrawal symptoms and user satisfaction. Nicotine & tobacco research : official journal of the Society for Research on Nicotine and Tobacco. 2009;11:715. | RCTs that did not provide data on the outcome of interest (i.e., arrhythmia, nonfatal myocardial infarction, nonfatal stroke, cardiovascular death) during the treatment period. |
| 1229 | Gilbert, David G, Zuo, Yantao, Rabinovich, Norka E, Riise, Hege, Needham, Rachel, Huggenvik, Jodi I. Neurotransmission-related genetic polymorphisms, negative affectivity traits, and gender predict tobacco abstinence symptoms across 44 days with and without nicotine patch. Journal of abnormal psychology. 2009;118:322. | RCTs that did not provide data on the outcome of interest (i.e., arrhythmia, nonfatal myocardial infarction, nonfatal stroke, cardiovascular death) during the treatment period. |
| 1231 | Knott, Verner J, Bolton, Kiley, Heenan, Adam, Shah, Dhrasti, Fisher, Derek J, Villeneuve, Crystal. Effects of acute nicotine on event-related potential and performance indices of auditory distraction in nonsmokers. Nicotine & tobacco research : official journal of the Society for Research on Nicotine and Tobacco. 2009;11:519. | RCTs that did not provide data on the outcome of interest (i.e., arrhythmia, nonfatal myocardial infarction, nonfatal stroke, cardiovascular death) during the treatment period. |
| 1232 | Perkins, Kenneth A, Grottenthaler, Amy, Ciccocioppo, Melinda M, Conklin, Cynthia A, Sayette, Michael A, Wilson, Annette S. Mood, nicotine, and dose expectancy effects on acute responses to nicotine spray. Nicotine & tobacco research : official journal of the Society for Research on Nicotine and Tobacco. 2009;11:540. | RCTs that did not provide data on the outcome of interest (i.e., arrhythmia, nonfatal myocardial infarction, nonfatal stroke, cardiovascular death) during the treatment period. |
| 1233 | Sørensen, Lars Tue, Zillmer, Rikke, Agren, Magnus, Ladelund, Steen, Karlsmark, Tonny, Gottrup, Finn. Effect of smoking, abstention, and nicotine patch on epidermal healing and collagenase in skin transudate. Wound repair and regeneration : official publication of the Wound Healing Society [and] the European Tissue Repair Society. 2009;17:347. | RCTs that did not provide data on the outcome of interest (i.e., arrhythmia, nonfatal myocardial infarction, nonfatal stroke, cardiovascular death) during the treatment period. |
| 1240 | Davenport, Paul W, Vovk, Andrea, Duke, Rita K, Bolser, Donald C, Robertson, Erin. The urge-to-cough and cough motor response modulation by the central effects of nicotine. Pulmonary pharmacology & therapeutics. 2009;22:82. | RCTs that did not provide data on the outcome of interest (i.e., arrhythmia, nonfatal myocardial infarction, nonfatal stroke, cardiovascular death) during the treatment period. |
| 1242 | Newhouse, Paul, Levin, Edward, Kellar, Kenneth, Coderre, Emily, Wilkins, Heather, Aisen, Paul, White, Heidi, Wesnes, Keith. Transdermal nicotine treatment of Mild Cognitive Impairment (MCI): A multi-center pilot study. American Journal of Geriatric Psychiatry. 2009;17:A102. | RCTs that did not provide data on the outcome of interest (i.e., arrhythmia, nonfatal myocardial infarction, nonfatal stroke, cardiovascular death) during the treatment period. |
| 1243 | Sadr Azodi, O, Lindström, D, Adami, J, Tønnesen, H, Nåsell, H, Gilljam, H, Wladis, A. The efficacy of a smoking cessation programme in patients undergoing elective surgery: a randomised clinical trial. Anaesthesia. 2009;64:259. | RCTs that did not provide data on the outcome of interest (i.e., arrhythmia, nonfatal myocardial infarction, nonfatal stroke, cardiovascular death) during the treatment period. |
| 1244 | Salize, Hans Joachim, Merkel, Silke, Reinhard, Iris, Twardella, Dorothee, Mann, Karl, Brenner, Hermann. Cost-effective primary care-based strategies to improve smoking cessation: more value for money. Archives of internal medicine. 2009;169:230. | RCTs that did not provide data on the outcome of interest (i.e., arrhythmia, nonfatal myocardial infarction, nonfatal stroke, cardiovascular death) during the treatment period. |
| 1246 | Rigotti, Nancy A, Gonzales, David, Dale, Lowell C, Lawrence, Daniel, Chang, Yuchiao. A randomized controlled trial of adding the nicotine patch to rimonabant for smoking cessation: efficacy, safety and weight gain. Addiction (Abingdon, England). 2009;104:266. | RCTs that did not provide data on the outcome of interest (i.e., arrhythmia, nonfatal myocardial infarction, nonfatal stroke, cardiovascular death) during the treatment period. |
| 1252 | Cosci, Fiammetta, Corlando, Annalisa, Fornai, Edo, Pistelli, Francesco, Paoletti, Paolo, Carrozzi, Laura. Nicotine dependence, psychological distress and personality traits as possible predictors of smoking cessation. Results of a double-blind study with nicotine patch. Addictive behaviors. 2009;34:28. | RCTs that did not provide data on the outcome of interest (i.e., arrhythmia, nonfatal myocardial infarction, nonfatal stroke, cardiovascular death) during the treatment period. |
| 1255 | Martins, Luíz Cláudio, Ferreira-Melo, Sílvia Elaine, Sabha, Maricene, Coelho, Otávio Rizzi, Yugar-Toledo, Juan Carlos, Quinaglia, Thiago, Moreira, Marcos Mello, Coca, Antonio, Moreno Júnior, Heitor. Acute effects of pharmacotherapies in blood pressure in normotensive moderate smokers. Blood pressure. 2009;18:255. | RCTs that did not provide data on the outcome of interest (i.e., arrhythmia, nonfatal myocardial infarction, nonfatal stroke, cardiovascular death) during the treatment period. |
| 1260 | Glasgow, Russell E, Gaglio, Bridget, Estabrooks, Paul A, Marcus, Alfred C, Ritzwoller, Debra P, Smith, Tammy L, Levinson, Arnold H, Sukhanova, Anna, O'Donnell, Colin, Ferro, Erica F, France, Eric K. Long-term results of a smoking reduction program. Medical care. 2009;47:115. | RCTs that did not include a nicotine versus no nicotine intervention. |
| 1262 | Colrain, I.M., Javitz, H.S., Baker, F.C., Krasnow, R., Swan, G.E.. The impact of smoking cessation and pharmacotherapy on sleep assessed across the first 10 days after quitting. Sleep. 2009;32:A364. | RCTs that did not provide data on the outcome of interest (i.e., arrhythmia, nonfatal myocardial infarction, nonfatal stroke, cardiovascular death) during the treatment period. |
| 1265 | Joyce, Geoffrey F, Niaura, Raymond, Maglione, Margaret, Mongoven, Jennifer, Larson-Rotter, Carrie, Coan, James, Lapin, Pauline, Morton, Sally. The effectiveness of covering smoking cessation services for medicare beneficiaries. Health services research. 2008;43:2106. | RCTs that did not include a nicotine versus no nicotine intervention. |
| 1267 | Wong, Candice C, Tsoh, Janice Y, Tong, Elisa K, Hom, Fred B, Cooper, Bruce, Chow, Edward A. The Chinese community smoking cessation project: a community sensitive intervention trial. Journal of community health. 2008;33:363. | RCTs that did not provide data on the outcome of interest (i.e., arrhythmia, nonfatal myocardial infarction, nonfatal stroke, cardiovascular death) during the treatment period. |
| 1277 | Cropsey, Karen, Eldridge, Gloria, Weaver, Michael, Villalobos, Gabriela, Stitzer, Maxine, Best, Al. Smoking cessation intervention for female prisoners: addressing an urgent public health need. American journal of public health. 2008;98:1894. | RCTs that did not provide data on the outcome of interest (i.e., arrhythmia, nonfatal myocardial infarction, nonfatal stroke, cardiovascular death) during the treatment period. |
| 1278 | Shiffman, Saul, Sweeney, Christine T, Ferguson, Stuart G, Sembower, Mark A, Gitchell, Joseph G. Relationship between adherence to daily nicotine patch use and treatment efficacy: secondary analysis of a 10-week randomized, double-blind, placebo-controlled clinical trial simulating over-the-counter use in adult smokers. Clinical therapeutics. 2008;30:1852. | RCTs that did not provide data on the outcome of interest (i.e., arrhythmia, nonfatal myocardial infarction, nonfatal stroke, cardiovascular death) during the treatment period. |
| 1282 | Oncken, Cheryl, Dornelas, Ellen, Greene, John, Sankey, Heather, Glasmann, Allen, Feinn, Richard, Kranzler, Henry R. Nicotine gum for pregnant smokers: a randomized controlled trial. Obstetrics and gynecology. 2008;112:859. | RCTs that did not provide data on the outcome of interest (i.e., arrhythmia, nonfatal myocardial infarction, nonfatal stroke, cardiovascular death) during the treatment period. |
| 1283 | Habib, Ashraf S, White, William D, El Gasim, Magdi A, Saleh, Gamal, Polascik, Thomas J, Moul, Judd W, Gan, Tong J. Transdermal nicotine for analgesia after radical retropubic prostatectomy. Anesthesia and analgesia. 2008;107:999. | RCTs that did not provide data on the outcome of interest (i.e., arrhythmia, nonfatal myocardial infarction, nonfatal stroke, cardiovascular death) during the treatment period. |
| 1284 | Hong, Daewha, Conell-Price, Jessamyn, Cheng, Sean, Flood, Pamela. Transdermal nicotine patch for postoperative pain management: a pilot dose-ranging study. Anesthesia and analgesia. 2008;107:1005. | RCTs that did not provide data on the outcome of interest (i.e., arrhythmia, nonfatal myocardial infarction, nonfatal stroke, cardiovascular death) during the treatment period. |
| 1285 | Turan, Alparslan, White, Paul F, Koyuncu, Onur, Karamanliodlu, Beyhan, Kaya, Gaye, Apfel, Christian C. Transdermal nicotine patch failed to improve postoperative pain management. Anesthesia and analgesia. 2008;107:1011. | RCTs that did not provide data on the outcome of interest (i.e., arrhythmia, nonfatal myocardial infarction, nonfatal stroke, cardiovascular death) during the treatment period. |
| 1288 | Goedert, J J, Scoppio, B M, Pfeiffer, R, Neve, L, Federici, A B, Long, L R, Dolan, B M, Brambati, M, Bellinvia, M, Lauria, C, Preiss, L, Boneschi, V, Whitby, D, Brambilla, L. Treatment of classic Kaposi sarcoma with a nicotine dermal patch: a phase II clinical trial. Journal of the European Academy of Dermatology and Venereology : JEADV. 2008;22:1101. | RCTs that did not include a nicotine versus no nicotine intervention. |
| 1292 | Shiffman, Saul. Effect of nicotine lozenges on affective smoking withdrawal symptoms: secondary analysis of a randomized, double-blind, placebo-controlled clinical trial. Clinical therapeutics. 2008;30:1461. | RCTs that did not provide data on the outcome of interest (i.e., arrhythmia, nonfatal myocardial infarction, nonfatal stroke, cardiovascular death) during the treatment period. |
| 1293 | Hong, L Elliot, Wonodi, Ikwunga, Lewis, Jada, Thaker, Gunvant K. Nicotine effect on prepulse inhibition and prepulse facilitation in schizophrenia patients. Neuropsychopharmacology : official publication of the American College of Neuropsychopharmacology. 2008;33:2167. | RCTs that did not provide data on the outcome of interest (i.e., arrhythmia, nonfatal myocardial infarction, nonfatal stroke, cardiovascular death) during the treatment period. |
| 1303 | Reid, Malcolm S, Fallon, Bryan, Sonne, Susan, Flammino, Frank, Nunes, Edward V, Jiang, Huiping, Kourniotis, Eva, Lima, Jennifer, Brady, Ron, Burgess, Cynthia, Arfken, Cynthia, Pihlgren, Eric, Giordano, Louis, Starosta, Aron, Robinson, James, Rotrosen, John. Smoking cessation treatment in community-based substance abuse rehabilitation programs. Journal of substance abuse treatment. 2008;35:68. | RCTs that did not provide data on the outcome of interest (i.e., arrhythmia, nonfatal myocardial infarction, nonfatal stroke, cardiovascular death) during the treatment period. |
| 1306 | Sherman, Scott E, Aldana, Ileana, Estrada, Maribel, York, Laura. Comparing the tolerability and effectiveness of two treatment regimens in a smoking clinic. Military medicine. 2008;173:550. | RCTs that did not provide data on the outcome of interest (i.e., arrhythmia, nonfatal myocardial infarction, nonfatal stroke, cardiovascular death) during the treatment period. |
| 1309 | Barnett, Paul G, Wong, Wynnie, Hall, Sharon. The cost-effectiveness of a smoking cessation program for out-patients in treatment for depression. Addiction (Abingdon, England). 2008;103:834. | RCTs that did not provide data on the outcome of interest (i.e., arrhythmia, nonfatal myocardial infarction, nonfatal stroke, cardiovascular death) during the treatment period. |
| 1313 | Takahashi, Hidehiko, Fujimura, Yota, Hayashi, Mika, Takano, Harumasa, Kato, Motoichiro, Okubo, Yoshiro, Kanno, Iwao, Ito, Hiroshi, Suhara, Tetsuya. Enhanced dopamine release by nicotine in cigarette smokers: a double-blind, randomized, placebo-controlled pilot study. The international journal of neuropsychopharmacology. 2008;11:413. | RCTs that did not provide data on the outcome of interest (i.e., arrhythmia, nonfatal myocardial infarction, nonfatal stroke, cardiovascular death) during the treatment period. |
| 1315 | Harte, Christopher B, Meston, Cindy M. The inhibitory effects of nicotine on physiological sexual arousal in nonsmoking women: results from a randomized, double-blind, placebo-controlled, cross-over trial. The journal of sexual medicine. 2008;5:1184. | RCTs that did not provide data on the outcome of interest (i.e., arrhythmia, nonfatal myocardial infarction, nonfatal stroke, cardiovascular death) during the treatment period. |
| 1317 | Pan, Alan X, de la Peña, Amparo, Yeo, Kwee P, Chan, Clark, Loh, Mei T, Wise, Stephen D, Silverman, Bernard L, Muchmore, Douglas B. Effects of smoking cessation, acute re-exposure and nicotine replacement in smokers on AIR inhaled insulin pharmacokinetics and glucodynamics. British journal of clinical pharmacology. 2008;65:480. | RCTs that did not provide data on the outcome of interest (i.e., arrhythmia, nonfatal myocardial infarction, nonfatal stroke, cardiovascular death) during the treatment period. |
| 1319 | David, S P, Munafò, M R, Murphy, M F G, Proctor, M, Walton, R T, Johnstone, E C. Genetic variation in the dopamine D4 receptor (DRD4) gene and smoking cessation: follow-up of a randomised clinical trial of transdermal nicotine patch. The pharmacogenomics journal. 2008;8:122. | RCTs that did not provide data on the outcome of interest (i.e., arrhythmia, nonfatal myocardial infarction, nonfatal stroke, cardiovascular death) during the treatment period. |
| 1323 | Sofuoglu, Mehmet, Yoo, Sonah, Hill, Kevin P, Mooney, Marc. Self-administration of intravenous nicotine in male and female cigarette smokers. Neuropsychopharmacology : official publication of the American College of Neuropsychopharmacology. 2008;33:715. | RCTs that did not provide data on the outcome of interest (i.e., arrhythmia, nonfatal myocardial infarction, nonfatal stroke, cardiovascular death) during the treatment period. |
| 1325 | Joseph, Anne M, Hecht, Stephen S, Murphy, Sharon E, Lando, Harry, Carmella, Steven G, Gross, Myron, Bliss, Robin, Le, Chap T, Hatsukami, Dorothy K. Smoking reduction fails to improve clinical and biological markers of cardiac disease: a randomized controlled trial. Nicotine & tobacco research : official journal of the Society for Research on Nicotine and Tobacco. 2008;10:471. | RCTs that did not provide data on the outcome of interest (i.e., arrhythmia, nonfatal myocardial infarction, nonfatal stroke, cardiovascular death) during the treatment period. |
| 1329 | Myers, Carol S, Taylor, Richard C, Moolchan, Eric T, Heishman, Stephen J. Dose-related enhancement of mood and cognition in smokers administered nicotine nasal spray. Neuropsychopharmacology : official publication of the American College of Neuropsychopharmacology. 2008;33:588. | RCTs that did not provide data on the outcome of interest (i.e., arrhythmia, nonfatal myocardial infarction, nonfatal stroke, cardiovascular death) during the treatment period. |
| 1332 | Cheng, Sean S, Yeh, Janet, Flood, Pamela. Anesthesia matters: patients anesthetized with propofol have less postoperative pain than those anesthetized with isoflurane. Anesthesia and analgesia. 2008;106:264. | RCTs that did not provide data on the outcome of interest (i.e., arrhythmia, nonfatal myocardial infarction, nonfatal stroke, cardiovascular death) during the treatment period. |
| 1333 | Kinnunen, Taru, Korhonen, Tellervo, Garvey, Arthur J. Role of nicotine gum and pretreatment depressive symptoms in smoking cessation: twelve-month results of a randomized placebo controlled trial. International journal of psychiatry in medicine. 2008;38:373. | RCTs that did not provide data on the outcome of interest (i.e., arrhythmia, nonfatal myocardial infarction, nonfatal stroke, cardiovascular death) during the treatment period. |
| 1335 | Okuyemi, Kolawole S, Faseru, Babalola, Sanderson Cox, Lisa, Bronars, Carrie A, Ahluwalia, Jasjit S. Relationship between menthol cigarettes and smoking cessation among African American light smokers. Addiction (Abingdon, England). 2007;102:1979. | RCTs that did not provide data on the outcome of interest (i.e., arrhythmia, nonfatal myocardial infarction, nonfatal stroke, cardiovascular death) during the treatment period. |
| 1340 | Gallagher, Sandra M, Penn, Patricia E, Schindler, Eric, Layne, Wendy. A comparison of smoking cessation treatments for persons with schizophrenia and other serious mental illnesses. Journal of psychoactive drugs. 2007;39:487. | RCTs that did not provide data on the outcome of interest (i.e., arrhythmia, nonfatal myocardial infarction, nonfatal stroke, cardiovascular death) during the treatment period. |
| 1342 | Cooney, Ned L, Litt, Mark D, Cooney, Judith L, Pilkey, David T, Steinberg, Howard R, Oncken, Cheryl A. Concurrent brief versus intensive smoking intervention during alcohol dependence treatment. Psychology of addictive behaviors : journal of the Society of Psychologists in Addictive Behaviors. 2007;21:570. | RCTs that did not provide data on the outcome of interest (i.e., arrhythmia, nonfatal myocardial infarction, nonfatal stroke, cardiovascular death) during the treatment period. |
| 1344 | Hollis, Jack F, McAfee, Timothy A, Fellows, Jeffrey L, Zbikowski, Susan M, Stark, Michael, Riedlinger, Karen. The effectiveness and cost effectiveness of telephone counselling and the nicotine patch in a state tobacco quitline. Tobacco control. 2007;16 Suppl 1:i53. | RCTs that did not provide data on the outcome of interest (i.e., arrhythmia, nonfatal myocardial infarction, nonfatal stroke, cardiovascular death) during the treatment period. |
| 1345 | Takahashi, Hidehiko, Fujimura, Yota, Takano, Harumasa, Ito, Hiroshi, Suhara, Tetsuya. Association between nicotine dependence and striatal dopamine release following nicotine administration. Journal of Cerebral Blood Flow and Metabolism. 2007;27:BP20. | RCTs that did not provide data on the outcome of interest (i.e., arrhythmia, nonfatal myocardial infarction, nonfatal stroke, cardiovascular death) during the treatment period. |
| 1350 | Dautzenberg, Bertrand, Nides, Mitchell, Kienzler, Jean-Luc, Callens, Anne. Pharmacokinetics, safety and efficacy from randomized controlled trials of 1 and 2 mg nicotine bitartrate lozenges (Nicotinell). BMC clinical pharmacology. 2007;7:11. | RCTs that did not provide data on the outcome of interest (i.e., arrhythmia, nonfatal myocardial infarction, nonfatal stroke, cardiovascular death) during the treatment period. |
| 1351 | Pollak, Kathryn I, Oncken, Cheryl A, Lipkus, Isaac M, Lyna, Pauline, Swamy, Geeta K, Pletsch, Pamela K, Peterson, Bercedis L, Heine, R Phillips, Brouwer, Rebecca J Namenek, Fish, Laura, Myers, Evan R. Nicotine replacement and behavioral therapy for smoking cessation in pregnancy. American journal of preventive medicine. 2007;33:297. | RCTs that did not provide data on the outcome of interest (i.e., arrhythmia, nonfatal myocardial infarction, nonfatal stroke, cardiovascular death) during the treatment period. |
| 1352 | Munafò, M R, Elliot, K M, Murphy, M F G, Walton, R T, Johnstone, E C. Association of the mu-opioid receptor gene with smoking cessation. The pharmacogenomics journal. 2007;7:353. | RCTs that did not provide data on the outcome of interest (i.e., arrhythmia, nonfatal myocardial infarction, nonfatal stroke, cardiovascular death) during the treatment period. |
| 1354 | Blank, Melissa D, Kleykamp, Bethea A, Jennings, Janine M, Eissenberg, Thomas. Caffeine's influence on nicotine's effects in nonsmokers. American journal of health behavior. 2007;31:473. | RCTs that did not include a nicotine versus no nicotine intervention. |
| 1355 | Piper, Megan E, Federman, E Belle, McCarthy, Danielle E, Bolt, Daniel M, Smith, Stevens S, Fiore, Michael C, Baker, Timothy B. Efficacy of bupropion alone and in combination with nicotine gum. Nicotine & tobacco research : official journal of the Society for Research on Nicotine and Tobacco. 2007;9:947. | RCTs that did not provide data on the outcome of interest (i.e., arrhythmia, nonfatal myocardial infarction, nonfatal stroke, cardiovascular death) during the treatment period. |
| 1362 | Ebbert, Jon O, Post, Jason A, Moyer, Thomas P, Dale, Lowell C, Schroeder, Darrell R, Hurt, Richard D. Nicotine percentage replacement among smokeless tobacco users with nicotine patch. Drug and alcohol dependence. 2007;89:223. | RCTs that did not provide data on the outcome of interest (i.e., arrhythmia, nonfatal myocardial infarction, nonfatal stroke, cardiovascular death) during the treatment period. |
| 1363 | Prapavessis, Harry, Cameron, Linda, Baldi, J Chris, Robinson, Stewart, Borrie, Kendra, Harper, Therese, Grove, J Robert. The effects of exercise and nicotine replacement therapy on smoking rates in women. Addictive behaviors. 2007;32:1416. | RCTs that did not provide data on the outcome of interest (i.e., arrhythmia, nonfatal myocardial infarction, nonfatal stroke, cardiovascular death) during the treatment period. |
| 1366 | Johnstone, Elaine C, Elliot, Katherine M, David, Sean P, Murphy, Michael F G, Walton, Robert T, Munafò, Marcus R. Association of COMT Val108/158Met genotype with smoking cessation in a nicotine replacement therapy randomized trial. Cancer epidemiology, biomarkers & prevention : a publication of the American Association for Cancer Research, cosponsored by the American Society of Preventive Oncology. 2007;16:1065. | RCTs that did not provide data on the outcome of interest (i.e., arrhythmia, nonfatal myocardial infarction, nonfatal stroke, cardiovascular death) during the treatment period. |
| 1369 | Binnie, Vivian I, McHugh, Siobhan, Jenkins, William, Borland, William, Macpherson, Lorna M. A randomised controlled trial of a smoking cessation intervention delivered by dental hygienists: a feasibility study. BMC oral health. 2007;7:5. | RCTs that did not include a nicotine versus no nicotine intervention. |
| 1372 | Etter, Jean-François, Laszlo, Evelyne. Postintervention effect of nicotine replacement therapy for smoking reduction: a randomized trial with a 5-year follow-up. Journal of clinical psychopharmacology. 2007;27:151. | RCTs that did not provide data on the outcome of interest (i.e., arrhythmia, nonfatal myocardial infarction, nonfatal stroke, cardiovascular death) during the treatment period. |
| 1379 | Dallery, Jesse, Raiff, Bethany R. Delay discounting predicts cigarette smoking in a laboratory model of abstinence reinforcement. Psychopharmacology. 2007;190:485. | RCTs that did not provide data on the outcome of interest (i.e., arrhythmia, nonfatal myocardial infarction, nonfatal stroke, cardiovascular death) during the treatment period. |
| 1381 | Oncken, Cheryl, Cooney, Judith, Feinn, Richard, Lando, Harry, Kranzler, Henry R. Transdermal nicotine for smoking cessation in postmenopausal women. Addictive behaviors. 2007;32:296. | RCTs that did not provide data on the outcome of interest (i.e., arrhythmia, nonfatal myocardial infarction, nonfatal stroke, cardiovascular death) during the treatment period. |
| 1382 | Okuyemi, Kolawole S, James, Aimee S, Mayo, Matthew S, Nollen, Nicole, Catley, Delwyn, Choi, Won S, Ahluwalia, Jasjit S. Pathways to health: a cluster randomized trial of nicotine gum and motivational interviewing for smoking cessation in low-income housing. Health education & behavior : the official publication of the Society for Public Health Education. 2007;34:43. | RCTs that did not provide data on the outcome of interest (i.e., arrhythmia, nonfatal myocardial infarction, nonfatal stroke, cardiovascular death) during the treatment period. |
| 1383 | Croghan, Ivana T, Hurt, Richard D, Dakhil, Shaker R, Croghan, Gary A, Sloan, Jeff A, Novotny, Paul J, Rowland, Kendrith M, Bernath, Albert, Loots, Mary L, Le-Lindqwister, Nguyet A, Tschetter, Loren K, Garneau, Stewart C, Flynn, Kathleen A, Ebbert, Larry P, Wender, Donald B, Loprinzi, Charles L. Randomized comparison of a nicotine inhaler and bupropion for smoking cessation and relapse prevention. Mayo Clinic proceedings. 2007;82:186. | RCTs that did not provide data on the outcome of interest (i.e., arrhythmia, nonfatal myocardial infarction, nonfatal stroke, cardiovascular death) during the treatment period. |
| 1385 | David, Sean P, Munafò, Marcus R, Murphy, Michael F G, Walton, Robert T, Johnstone, Elaine C. The serotonin transporter 5-HTTLPR polymorphism and treatment response to nicotine patch: follow-up of a randomized controlled trial. Nicotine & tobacco research : official journal of the Society for Research on Nicotine and Tobacco. 2007;9:225. | RCTs that did not provide data on the outcome of interest (i.e., arrhythmia, nonfatal myocardial infarction, nonfatal stroke, cardiovascular death) during the treatment period. |
| 1392 | Ionescu, Daniela, Badescu, Cristina, Acalovschi, Iurie. Nicotine patch for the prevention of postoperative nausea and vomiting: a prospective randomised trial. Clinical drug investigation. 2007;27:559. | RCTs that did not provide data on the outcome of interest (i.e., arrhythmia, nonfatal myocardial infarction, nonfatal stroke, cardiovascular death) during the treatment period. |
| 1399 | Ferguson, Stuart G, Shiffman, Saul, Gwaltney, Chad J. Does reducing withdrawal severity mediate nicotine patch efficacy? A randomized clinical trial. Journal of consulting and clinical psychology. 2006;74:1153. | RCTs that did not provide data on the outcome of interest (i.e., arrhythmia, nonfatal myocardial infarction, nonfatal stroke, cardiovascular death) during the treatment period. |
| 1401 | Oncken, Cheryl, Prestwood, Karen, Kleppinger, Alison, Wang, Yazhen, Cooney, Judith, Raisz, Lawrence. Impact of smoking cessation on bone mineral density in postmenopausal women. Journal of women's health (2002). 2006;15:1141. | RCTs that did not provide data on the outcome of interest (i.e., arrhythmia, nonfatal myocardial infarction, nonfatal stroke, cardiovascular death) during the treatment period. |
| 1406 | Baker, Amanda, Richmond, Robyn, Haile, Melanie, Lewin, Terry J, Carr, Vaughan J, Taylor, Rachel L, Jansons, Sylvia, Wilhelm, Kay. A randomized controlled trial of a smoking cessation intervention among people with a psychotic disorder. The American journal of psychiatry. 2006;163:1934. | RCTs that did not provide data on the outcome of interest (i.e., arrhythmia, nonfatal myocardial infarction, nonfatal stroke, cardiovascular death) during the treatment period. |
| 1407 | McClernon, F Joseph, Hiott, F Berry, Westman, Eric C, Rose, Jed E, Levin, Edward D. Transdermal nicotine attenuates depression symptoms in nonsmokers: a double-blind, placebo-controlled trial. Psychopharmacology. 2006;189:125. | RCTs that did not provide data on the outcome of interest (i.e., arrhythmia, nonfatal myocardial infarction, nonfatal stroke, cardiovascular death) during the treatment period. |
| 1418 | Knott, V, McIntosh, J, Millar, A, Fisher, D, Villeneuve, C, Ilivitsky, V, Horn, E. Nicotine and smoker status moderate brain electric and mood activation induced by ketamine, an N-methyl-D-aspartate (NMDA) receptor antagonist. Pharmacology, biochemistry, and behavior. 2006;85:228. | RCTs that did not provide data on the outcome of interest (i.e., arrhythmia, nonfatal myocardial infarction, nonfatal stroke, cardiovascular death) during the treatment period. |
| 1421 | Cosci, Fiammetta, Abrams, Kenneth, Schruers, Koen R J, Rickelt, Judith, Griez, Eric J L. Effect of nicotine on 35% CO2-induced anxiety: A study in healthy volunteers. Nicotine & tobacco research : official journal of the Society for Research on Nicotine and Tobacco. 2006;8:511. | RCTs that did not provide data on the outcome of interest (i.e., arrhythmia, nonfatal myocardial infarction, nonfatal stroke, cardiovascular death) during the treatment period. |
| 1423 | Rennard, Stephen I, Glover, Elbert D, Leischow, Scott, Daughton, David M, Glover, Penny N, Muramoto, Myra, Franzon, Mikael, Danielsson, Tobias, Landfeldt, Björn, Westin, Ake. Efficacy of the nicotine inhaler in smoking reduction: A double-blind, randomized trial. Nicotine & tobacco research : official journal of the Society for Research on Nicotine and Tobacco. 2006;8:555. | RCTs that did not provide data on the outcome of interest (i.e., arrhythmia, nonfatal myocardial infarction, nonfatal stroke, cardiovascular death) during the treatment period. |
| 1427 | Clark, Matthew M, Hurt, Richard D, Croghan, Ivana T, Patten, Christi A, Novotny, Paul, Sloan, Jeff A, Dakhil, Shaker R, Croghan, Gary A, Wos, Edward J, Rowland, Kendrith M, Bernath, Albert, Morton, Roscoe F, Thomas, Sachdex P, Tschetter, Loren K, Garneau, Stewart, Stella, Philip J, Ebbert, Larry P, Wender, Donald B, Loprinzi, Charles L. The prevalence of weight concerns in a smoking abstinence clinical trial. Addictive behaviors. 2006;31:1144. | RCTs that did not provide data on the outcome of interest (i.e., arrhythmia, nonfatal myocardial infarction, nonfatal stroke, cardiovascular death) during the treatment period. |
| 1429 | Mündel, Toby, Jones, David A. Effect of transdermal nicotine administration on exercise endurance in men. Experimental physiology. 2006;91:705. | RCTs that did not provide data on the outcome of interest (i.e., arrhythmia, nonfatal myocardial infarction, nonfatal stroke, cardiovascular death) during the treatment period. |
| 1430 | Ahluwalia, Jasjit S, Okuyemi, Kolawole, Nollen, Nicole, Choi, Won S, Kaur, Harsohena, Pulvers, Kim, Mayo, Matthew S. The effects of nicotine gum and counseling among African American light smokers: a 2 x 2 factorial design. Addiction (Abingdon, England). 2006;101:883. | RCTs that did not provide data on the outcome of interest (i.e., arrhythmia, nonfatal myocardial infarction, nonfatal stroke, cardiovascular death) during the treatment period. |
| 1432 | Najem, Boutaïna, Houssière, Anne, Pathak, Atul, Janssen, Christophe, Lemogoum, Daniel, Xhaët, Olivier, Cuylits, Nicolas, van de Borne, Philippe. Acute cardiovascular and sympathetic effects of nicotine replacement therapy. Hypertension (Dallas, Tex. : 1979). 2006;47:1162. | RCTs that did not provide data on the outcome of interest (i.e., arrhythmia, nonfatal myocardial infarction, nonfatal stroke, cardiovascular death) during the treatment period. |
| 1435 | Nollen, Nicole L, Mayo, Matthew S, Sanderson Cox, Lisa, Okuyemi, Kolawole S, Choi, Won S, Kaur, Harsohena, Ahluwalia, Jasjit S. Predictors of quitting among African American light smokers enrolled in a randomized, placebo-controlled trial. Journal of general internal medicine. 2006;21:590. | RCTs that did not provide data on the outcome of interest (i.e., arrhythmia, nonfatal myocardial infarction, nonfatal stroke, cardiovascular death) during the treatment period. |
| 1436 | Knott, Verner, Blais, Crystal, Scherling, Carole, Camarda, Jordan, Millar, Anne, Fisher, Derek, McIntosh, Judy. Neural effects of nicotine during auditory selective attention in smokers: an event-related potential study. Neuropsychobiology. 2006;53:115. | RCTs that did not provide data on the outcome of interest (i.e., arrhythmia, nonfatal myocardial infarction, nonfatal stroke, cardiovascular death) during the treatment period. |
| 1441 | Evans, Sarah E, Blank, Melissa, Sams, Cynthia, Weaver, Michael F, Eissenberg, Thomas. Transdermal nicotine-induced tobacco abstinence symptom suppression: nicotine dose and smokers' gender. Experimental and clinical psychopharmacology. 2006;14:121. | RCTs that did not provide data on the outcome of interest (i.e., arrhythmia, nonfatal myocardial infarction, nonfatal stroke, cardiovascular death) during the treatment period. |
| 1443 | Sørensen, Lars T, Jorgensen, Lars N, Zillmer, Rikke, Vange, Jakob, Hemmingsen, Ulla, Gottrup, Finn. Transdermal nicotine patch enhances type I collagen synthesis in abstinent smokers. Wound repair and regeneration : official publication of the Wound Healing Society [and] the European Tissue Repair Society. 2006;14:247. | RCTs that did not provide data on the outcome of interest (i.e., arrhythmia, nonfatal myocardial infarction, nonfatal stroke, cardiovascular death) during the treatment period. |
| 1445 | Hotham, Elizabeth D, Gilbert, Andrew L, Atkinson, Elinor R. A randomised-controlled pilot study using nicotine patches with pregnant women. Addictive behaviors. 2006;31:641. | RCTs that did not provide data on the outcome of interest (i.e., arrhythmia, nonfatal myocardial infarction, nonfatal stroke, cardiovascular death) during the treatment period. |
| 1446 | Halpin, Helen Ann, McMenamin, Sara B, Rideout, Jeffrey, Boyce-Smith, Gifford. The costs and effectiveness of different benefit designs for treating tobacco dependence: results from a randomized trial. Inquiry : a journal of medical care organization, provision and financing. 2006;43:54. | RCTs that did not include a nicotine versus no nicotine intervention. |
| 1451 | Knott, Verner J, Scherling, Carole S, Blais, Crystal M, Camarda, Jordan, Fisher, Derek J, Millar, Anne, McIntosh, Judy F. Acute nicotine fails to alter event-related potential or behavioral performance indices of auditory distraction in cigarette smokers. Nicotine & tobacco research : official journal of the Society for Research on Nicotine and Tobacco. 2006;8:263. | RCTs that did not provide data on the outcome of interest (i.e., arrhythmia, nonfatal myocardial infarction, nonfatal stroke, cardiovascular death) during the treatment period. |
| 1453 | Demazières, A, Luthringer, R, Coppel, E, Gilles, C, Fleury, C, Roegel, J C, Delarue, A, Laur, C, Lagrue, G. Ability of a new oral nicotine substitute to reduce smoking urge in moderate smokers. Addictive behaviors. 2006;31:537. | RCTs that did not provide data on the outcome of interest (i.e., arrhythmia, nonfatal myocardial infarction, nonfatal stroke, cardiovascular death) during the treatment period. |
| 1454 | Shiffman, Saul, Ferguson, Stuart G, Gwaltney, Chad J, Balabanis, Mark H, Shadel, William G. Reduction of abstinence-induced withdrawal and craving using high-dose nicotine replacement therapy. Psychopharmacology. 2006;184:637. | RCTs that did not provide data on the outcome of interest (i.e., arrhythmia, nonfatal myocardial infarction, nonfatal stroke, cardiovascular death) during the treatment period. |
| 1456 | Munafò, Marcus R, Johnstone, Elaine C, Wileyto, E Paul, Shields, Peter G, Elliot, Katherine M, Lerman, Caryn. Lack of association of 5-HTTLPR genotype with smoking cessation in a nicotine replacement therapy randomized trial. Cancer epidemiology, biomarkers & prevention : a publication of the American Association for Cancer Research, cosponsored by the American Society of Preventive Oncology. 2006;15:398. | RCTs that did not provide data on the outcome of interest (i.e., arrhythmia, nonfatal myocardial infarction, nonfatal stroke, cardiovascular death) during the treatment period. |
| 1458 | Gehricke, Jean-G, Whalen, Carol K, Jamner, Larry D, Wigal, Tim L, Steinhoff, Kenneth. The reinforcing effects of nicotine and stimulant medication in the everyday lives of adult smokers with ADHD: A preliminary examination. Nicotine & tobacco research : official journal of the Society for Research on Nicotine and Tobacco. 2006;8:37. | RCTs that did not provide data on the outcome of interest (i.e., arrhythmia, nonfatal myocardial infarction, nonfatal stroke, cardiovascular death) during the treatment period. |
| 1464 | Baldeweg, Torsten, Wong, Davina, Stephan, Klaas E. Nicotinic modulation of human auditory sensory memory: Evidence from mismatch negativity potentials. International journal of psychophysiology : official journal of the International Organization of Psychophysiology. 2006;59:49. | RCTs that did not provide data on the outcome of interest (i.e., arrhythmia, nonfatal myocardial infarction, nonfatal stroke, cardiovascular death) during the treatment period. |
| 1470 | Roozen, Hendrik G, Van Beers, Suzanne E C, Weevers, Harm Jan A, Breteler, Marinus H M, Willemsen, Marc C, Postmus, Pieter E, Kerkhof, Ad J F M. Effects on smoking cessation: naltrexone combined with a cognitive behavioral treatment based on the community reinforcement approach. Substance use & misuse. 2006;41:45. | RCTs that did not include a nicotine versus no nicotine intervention. |
| 1471 | Korhonen, Tellervo, Kinnunen, Taru, Quiles, Zandra, Leeman, Robert F, Terwal, Donna Medaglia, Garvey, Arthur J. Cardiovascular risk behavior among sedentary female smokers and smoking cessation outcomes. Tobacco induced diseases. 2005;3:7. | RCTs that did not provide data on the outcome of interest (i.e., arrhythmia, nonfatal myocardial infarction, nonfatal stroke, cardiovascular death) during the treatment period. |
| 1472 | Batra, Anil, Klingler, Karl, Landfeldt, Björn, Friederich, Hubertus M, Westin, Ake, Danielsson, Tobias. Smoking reduction treatment with 4-mg nicotine gum: a double-blind, randomized, placebo-controlled study. Clinical pharmacology and therapeutics. 2005;78:689. | RCTs that did not provide data on the outcome of interest (i.e., arrhythmia, nonfatal myocardial infarction, nonfatal stroke, cardiovascular death) during the treatment period. |
| 1473 | Marcus, Bess H, Lewis, Beth A, Hogan, Joseph, King, Teresa K, Albrecht, Anna E, Bock, Beth, Parisi, Alfred F, Niaura, Raymond, Abrams, David B. The efficacy of moderate-intensity exercise as an aid for smoking cessation in women: a randomized controlled trial. Nicotine & tobacco research : official journal of the Society for Research on Nicotine and Tobacco. 2005;7:871. | RCTs that did not include a nicotine versus no nicotine intervention. |
| 1477 | Ingram, J R, Rhodes, J, Collins, P W, Williams, G T, Newcombe, R G, Thomas, G A O. Plasma fibrinogen in ulcerative colitis: the effect of disease activity and nicotine therapy in a randomised controlled trial. Digestive and liver disease : official journal of the Italian Society of Gastroenterology and the Italian Association for the Study of the Liver. 2005;37:832. | RCTs that did not provide data on the outcome of interest (i.e., arrhythmia, nonfatal myocardial infarction, nonfatal stroke, cardiovascular death) during the treatment period. |
| 1479 | Hatsukami, Dorothy K, Kotlyar, Michael, Allen, Sharon, Jensen, Joni, Li, Shelby, Le, Chap, Murphy, Sharon. Effects of cigarette reduction on cardiovascular risk factors and subjective measures. Chest. 2005;128:2528. | RCTs that did not provide data on the outcome of interest (i.e., arrhythmia, nonfatal myocardial infarction, nonfatal stroke, cardiovascular death) during the treatment period. |
| 1483 | Emmons, Karen M, Puleo, Elaine, Park, Elyse, Gritz, Ellen R, Butterfield, Rita M, Weeks, Jane C, Mertens, Ann, Li, Frederick P. Peer-delivered smoking counseling for childhood cancer survivors increases rate of cessation: the partnership for health study. Journal of clinical oncology : official journal of the American Society of Clinical Oncology. 2005;23:6516. | RCTs that did not provide data on the outcome of interest (i.e., arrhythmia, nonfatal myocardial infarction, nonfatal stroke, cardiovascular death) during the treatment period. |
| 1484 | Hotham, Elizabeth D, Gilbert, Andrew L, Atkinson, Elinor R. Case studies of three pregnant smokers and their use of nicotine replacement therapy. Midwifery. 2005;21:224. | RCTs that did not include a nicotine versus no nicotine intervention. |
| 1485 | Allen, Sharon S, Hatsukami, Dorothy, Brintnell, Dawn M, Bade, Tracy. Effect of nicotine replacement therapy on post-cessation weight gain and nutrient intake: a randomized controlled trial of postmenopausal female smokers. Addictive behaviors. 2005;30:1273. | RCTs that did not provide data on the outcome of interest (i.e., arrhythmia, nonfatal myocardial infarction, nonfatal stroke, cardiovascular death) during the treatment period. |
| 1491 | Jessen, A, Buemann, B, Toubro, S, Skovgaard, I M, Astrup, A. The appetite-suppressant effect of nicotine is enhanced by caffeine. Diabetes, obesity & metabolism. 2005;7:327. | RCTs that did not provide data on the outcome of interest (i.e., arrhythmia, nonfatal myocardial infarction, nonfatal stroke, cardiovascular death) during the treatment period. |
| 1493 | Warner, David O, Patten, Christi A, Ames, Steven C, Offord, Kenneth P, Schroeder, Darrell R. Effect of nicotine replacement therapy on stress and smoking behavior in surgical patients. Anesthesiology. 2005;102:1138. | RCTs that did not provide data on the outcome of interest (i.e., arrhythmia, nonfatal myocardial infarction, nonfatal stroke, cardiovascular death) during the treatment period. |
| 1494 | Nagle, Amanda L, Hensley, Michael J, Schofield, Margot J, Koschel, Alison J. A randomised controlled trial to evaluate the efficacy of a nurse-provided intervention for hospitalised smokers. Australian and New Zealand journal of public health. 2005;29:285. | RCTs that did not include a nicotine versus no nicotine intervention. |
| 1499 | Wiseman, Eve J, Williams, D K, McMillan, D E. Effectiveness of payment for reduced carbon monoxide levels and noncontingent payments on smoking behaviors in cocaine-abusing outpatients wearing nicotine or placebo patches. Experimental and clinical psychopharmacology. 2005;13:102. | RCTs that did not provide data on the outcome of interest (i.e., arrhythmia, nonfatal myocardial infarction, nonfatal stroke, cardiovascular death) during the treatment period. |
| 1506 | Dar, Reuven, Stronguin, Florencia, Etter, Jean-Francois. Assigned versus perceived placebo effects in nicotine replacement therapy for smoking reduction in Swiss smokers. Journal of consulting and clinical psychology. 2005;73:350. | RCTs that did not provide data on the outcome of interest (i.e., arrhythmia, nonfatal myocardial infarction, nonfatal stroke, cardiovascular death) during the treatment period. |
| 1507 | Thiel, Christiane M, Zilles, Karl, Fink, Gereon R. Nicotine modulates reorienting of visuospatial attention and neural activity in human parietal cortex. Neuropsychopharmacology : official publication of the American College of Neuropsychopharmacology. 2005;30:810. | RCTs that did not provide data on the outcome of interest (i.e., arrhythmia, nonfatal myocardial infarction, nonfatal stroke, cardiovascular death) during the treatment period. |
| 1513 | Shiffman, Saul. Nicotine lozenge efficacy in light smokers. Drug and alcohol dependence. 2005;77:311. | RCTs that did not provide data on the outcome of interest (i.e., arrhythmia, nonfatal myocardial infarction, nonfatal stroke, cardiovascular death) during the treatment period. |
| 1518 | Anthonisen, Nicholas R, Skeans, Melissa A, Wise, Robert A, Manfreda, Jure, Kanner, Richard E, Connett, John E. The effects of a smoking cessation intervention on 14.5-year mortality: a randomized clinical trial. Annals of internal medicine. 2005;142:233. | RCTs that did not provide data on the outcome of interest (i.e., arrhythmia, nonfatal myocardial infarction, nonfatal stroke, cardiovascular death) during the treatment period. |
| 1520 | Wolfenden, L, Wiggers, J, Knight, J, Campbell, E, Rissel, C, Kerridge, R, Spigelman, A D, Moore, K. A programme for reducing smoking in pre-operative surgical patients: randomised controlled trial. Anaesthesia. 2005;60:172. | RCTs that did not provide data on the outcome of interest (i.e., arrhythmia, nonfatal myocardial infarction, nonfatal stroke, cardiovascular death) during the treatment period. |
| 1522 | Shiffman, Saul, Sweeney, Christine T, Dresler, Carolyn M. Nicotine patch and lozenge are effective for women. Nicotine & tobacco research : official journal of the Society for Research on Nicotine and Tobacco. 2005;7:119. | RCTs that did not provide data on the outcome of interest (i.e., arrhythmia, nonfatal myocardial infarction, nonfatal stroke, cardiovascular death) during the treatment period. |
| 1523 | Poltavski, D V, Petros, T. Effects of transdermal nicotine on prose memory and attention in smokers and nonsmokers. Physiology & behavior. 2005;83:833. | RCTs that did not provide data on the outcome of interest (i.e., arrhythmia, nonfatal myocardial infarction, nonfatal stroke, cardiovascular death) during the treatment period. |
| 1525 | Cooper, Theodore V, Klesges, Robert C, Debon, Margaret W, Zbikowski, Susan M, Johnson, Karen C, Clemens, Linda H. A placebo controlled randomized trial of the effects of phenylpropanolamine and nicotine gum on cessation rates and postcessation weight gain in women. Addictive behaviors. 2005;30:61. | RCTs that did not provide data on the outcome of interest (i.e., arrhythmia, nonfatal myocardial infarction, nonfatal stroke, cardiovascular death) during the treatment period. |
| 1530 | Shiffman, Saul, Di Marino, Michael E, Pillitteri, Janine L. The effectiveness of nicotine patch and nicotine lozenge in very heavy smokers. Journal of substance abuse treatment. 2005;28:49. | RCTs that did not provide data on the outcome of interest (i.e., arrhythmia, nonfatal myocardial infarction, nonfatal stroke, cardiovascular death) during the treatment period. |
| 1531 | Flood, Pamela, Daniel, Danette. Intranasal nicotine for postoperative pain treatment. Anesthesiology. 2004;101:1417. | RCTs that did not provide data on the outcome of interest (i.e., arrhythmia, nonfatal myocardial infarction, nonfatal stroke, cardiovascular death) during the treatment period. |
| 1532 | Waters, Andrew J, Shiffman, Saul, Sayette, Michael A, Paty, Jean A, Gwaltney, Chad J, Balabanis, Mark H. Cue-provoked craving and nicotine replacement therapy in smoking cessation. Journal of consulting and clinical psychology. 2004;72:1136. | RCTs that did not provide data on the outcome of interest (i.e., arrhythmia, nonfatal myocardial infarction, nonfatal stroke, cardiovascular death) during the treatment period. |
| 1541 | Wakefield, Melanie, Olver, Ian, Whitford, Hayley, Rosenfeld, Ellie. Motivational interviewing as a smoking cessation intervention for patients with cancer: randomized controlled trial. Nursing research. 2004;53:396. | RCTs that did not provide data on the outcome of interest (i.e., arrhythmia, nonfatal myocardial infarction, nonfatal stroke, cardiovascular death) during the treatment period. |
| 1544 | Sørensen, Lars Tue, Nielsen, Henning Bay, Kharazmi, Arsalan, Gottrup, Finn. Effect of smoking and abstention on oxidative burst and reactivity of neutrophils and monocytes. Surgery. 2004;136:1047. | RCTs that did not provide data on the outcome of interest (i.e., arrhythmia, nonfatal myocardial infarction, nonfatal stroke, cardiovascular death) during the treatment period. |
| 1548 | Mora-Martínez, José Miguel, González-Ortiz, Manuel, Balcázar-Muñoz, Blanca R, Martínez-Abundis, Esperanza. Acute effect of the transdermal administration of nicotine on insulin sensitivity in healthy individuals with and without a family history of type 2 diabetes mellitus in the first branch. Metabolic syndrome and related disorders. 2004;2:227. | RCTs that did not provide data on the outcome of interest (i.e., arrhythmia, nonfatal myocardial infarction, nonfatal stroke, cardiovascular death) during the treatment period. |
| 1550 | Allen, Sharon S, Hatsukami, Dorothy K, Bade, Tracy, Center, Bruce. Transdermal nicotine use in postmenopausal women: does the treatment efficacy differ in women using and not using hormone replacement therapy?. Nicotine & tobacco research : official journal of the Society for Research on Nicotine and Tobacco. 2004;6:777. | RCTs that did not provide data on the outcome of interest (i.e., arrhythmia, nonfatal myocardial infarction, nonfatal stroke, cardiovascular death) during the treatment period. |
| 1557 | Leischow, Scott J, Ranger-Moore, James, Muramoto, Myra L, Matthews, Eva. Effectiveness of the nicotine inhaler for smoking cessation in an OTC setting. American journal of health behavior. 2004;28:291. | RCTs that did not include a nicotine versus no nicotine intervention. |
| 1562 | Carpenter, Matthew J, Hughes, John R, Solomon, Laura J, Callas, Peter W. Both smoking reduction with nicotine replacement therapy and motivational advice increase future cessation among smokers unmotivated to quit. Journal of consulting and clinical psychology. 2004;72:371. | RCTs that did not provide data on the outcome of interest (i.e., arrhythmia, nonfatal myocardial infarction, nonfatal stroke, cardiovascular death) during the treatment period. |
| 1563 | Ratner, Pamela A, Johnson, Joy L, Richardson, Chris G, Bottorff, Joan L, Moffat, Barbara, Mackay, Martha, Fofonoff, Doreen, Kingsbury, Kori, Miller, Clara, Budz, Bernice. Efficacy of a smoking-cessation intervention for elective-surgical patients. Research in nursing & health. 2004;27:148. | RCTs that did not provide data on the outcome of interest (i.e., arrhythmia, nonfatal myocardial infarction, nonfatal stroke, cardiovascular death) during the treatment period. |
| 1564 | Schuurmans, Macé M, Diacon, Andreas H, van Biljon, Xandra, Bolliger, Chris T. Effect of pre-treatment with nicotine patch on withdrawal symptoms and abstinence rates in smokers subsequently quitting with the nicotine patch: a randomized controlled trial. Addiction (Abingdon, England). 2004;99:634. | RCTs that did not provide data on the outcome of interest (i.e., arrhythmia, nonfatal myocardial infarction, nonfatal stroke, cardiovascular death) during the treatment period. |
| 1566 | Morgan, Thomas M, Crawford, Linda, Stoller, Adam, Toth, David, Yeo, Kiang-Teck J, Baron, John A. Acute effects of nicotine on serum glucose insulin growth hormone and cortisol in healthy smokers. Metabolism: clinical and experimental. 2004;53:578. | RCTs that did not provide data on the outcome of interest (i.e., arrhythmia, nonfatal myocardial infarction, nonfatal stroke, cardiovascular death) during the treatment period. |
| 1568 | Yudkin, Patricia, Munafò, Marcus, Hey, Kate, Roberts, Sarah, Welch, Sarah, Johnstone, Elaine, Murphy, Michael, Griffiths, Siân, Walton, Robert. Effectiveness of nicotine patches in relation to genotype in women versus men: randomised controlled trial. BMJ (Clinical research ed.). 2004;328:989. | RCTs that did not provide data on the outcome of interest (i.e., arrhythmia, nonfatal myocardial infarction, nonfatal stroke, cardiovascular death) during the treatment period. |
| 1569 | Katz, David A, Muehlenbruch, Donna R, Brown, Roger L, Fiore, Michael C, Baker, Timothy B. Effectiveness of implementing the agency for healthcare research and quality smoking cessation clinical practice guideline: a randomized, controlled trial. Journal of the National Cancer Institute. 2004;96:594. | RCTs that did not provide data on the outcome of interest (i.e., arrhythmia, nonfatal myocardial infarction, nonfatal stroke, cardiovascular death) during the treatment period. |
| 1570 | Etter, Jean-François, Laszlo, Evelyne, Perneger, Thomas V. Postintervention effect of nicotine replacement therapy on smoking reduction in smokers who are unwilling to quit: randomized trial. Journal of clinical psychopharmacology. 2004;24:174. | RCTs that did not provide data on the outcome of interest (i.e., arrhythmia, nonfatal myocardial infarction, nonfatal stroke, cardiovascular death) during the treatment period. |
| 1571 | Hughes, John, Lindgren, Paula, Connett, John, Nides, Mitchell. Smoking reduction in the Lung Health Study. Nicotine & tobacco research : official journal of the Society for Research on Nicotine and Tobacco. 2004;6:275. | RCTs that did not provide data on the outcome of interest (i.e., arrhythmia, nonfatal myocardial infarction, nonfatal stroke, cardiovascular death) during the treatment period. |
| 1573 | Patterson, Freda, Jepson, Christopher, Kaufmann, Vyga, Rukstalis, Margaret, Audrain-McGovern, Janet, Kucharski, Susan, Lerman, Caryn. Erratum: Predictors of attendance in a randomized clinical trial of nicotine replacement therapy with behavioral counseling (Drug and Alcohol Dependence (2003) 72 (123-131) PII: S0376871603001947). Drug and Alcohol Dependence. 2004;73:315. | RCTs that did not include a nicotine versus no nicotine intervention. |
| 1574 | Botella-Carretero, J I, Escobar-Morreale, H F, Martín, I, Valero, A M, Alvarez, F, García, G, Varela, C, Cantarero, M. Weight gain and cardiovascular risk factors during smoking cessation with bupropion or nicotine. Hormone and metabolic research = Hormon- und Stoffwechselforschung = Hormones et metabolisme. 2004;36:178. | RCTs that did not provide data on the outcome of interest (i.e., arrhythmia, nonfatal myocardial infarction, nonfatal stroke, cardiovascular death) during the treatment period. |
| 1577 | D'Orlando, Kay J, Fox, Barbara S. Tolerability and pharmacokinetics of single and repeated doses of nicotine with The Straw, a novel nicotine replacement product. Nicotine & tobacco research : official journal of the Society for Research on Nicotine and Tobacco. 2004;6:63. | RCTs that did not provide data on the outcome of interest (i.e., arrhythmia, nonfatal myocardial infarction, nonfatal stroke, cardiovascular death) during the treatment period. |
| 1578 | Johnstone, Elaine C, Yudkin, Patricia L, Hey, Kate, Roberts, Sarah J, Welch, Sarah J, Murphy, Michael F, Griffiths, Siân E, Walton, Robert T. Genetic variation in dopaminergic pathways and short-term effectiveness of the nicotine patch. Pharmacogenetics. 2004;14:83. | RCTs that did not provide data on the outcome of interest (i.e., arrhythmia, nonfatal myocardial infarction, nonfatal stroke, cardiovascular death) during the treatment period. |
| 1580 | Shiffman, Saul, Dresler, Carolyn M, Rohay, Jeffrey M. Successful treatment with a nicotine lozenge of smokers with prior failure in pharmacological therapy. Addiction (Abingdon, England). 2004;99:83. | RCTs that did not provide data on the outcome of interest (i.e., arrhythmia, nonfatal myocardial infarction, nonfatal stroke, cardiovascular death) during the treatment period. |
| 1585 | Sanderson Cox, Lisa, Patten, Christi A., Krahn, Lois E., Hurt, Richard D., Croghan, Ivana T., Wolter, Troy D., Schroeder, Darrell R., Tri, Diane, Offord, Kenneth P.. The Effect of Nicotine Patch Therapy on Depression in Nonsmokers: A Preliminary Study. Journal of Addictive Diseases. 2004;22:75. | RCTs that did not provide data on the outcome of interest (i.e., arrhythmia, nonfatal myocardial infarction, nonfatal stroke, cardiovascular death) during the treatment period. |
| 1589 | Havermans, Remco C, Debaere, Saskia, Smulders, Fren T Y, Wiers, Reinout W, Jansen, Anita T M. Effect of cue exposure, urge to smoke, and nicotine deprivation on cognitive performance in smokers. Psychology of addictive behaviors : journal of the Society of Psychologists in Addictive Behaviors. 2003;17:336. | RCTs that did not provide data on the outcome of interest (i.e., arrhythmia, nonfatal myocardial infarction, nonfatal stroke, cardiovascular death) during the treatment period. |
| 1593 | Salín-Pascual, Rafael J, Basañez-Villa, Elizabeth. Changes in compulsion and anxiety symptoms with nicotine transdermal patches in non-smoking obsessive-compulsive disorder patients. Revista de investigacion clinica; organo del Hospital de Enfermedades de la Nutricion. 2003;55:650. | RCTs that did not provide data on the outcome of interest (i.e., arrhythmia, nonfatal myocardial infarction, nonfatal stroke, cardiovascular death) during the treatment period. |
| 1595 | Wennike, Poul, Danielsson, Tobias, Landfeldt, Björn, Westin, Ake, Tønnesen, Philip. Smoking reduction promotes smoking cessation: results from a double blind, randomized, placebo-controlled trial of nicotine gum with 2-year follow-up. Addiction (Abingdon, England). 2003;98:1395. | RCTs that did not provide data on the outcome of interest (i.e., arrhythmia, nonfatal myocardial infarction, nonfatal stroke, cardiovascular death) during the treatment period. |
| 1598 | Swanson, Nancy A, Burroughs, Charles C, Long, Mark A, Lee, Robert W. Controlled trial for smoking cessation in a Navy shipboard population using nicotine patch, sustained-release buproprion, or both. Military medicine. 2003;168:830. | RCTs that did not provide data on the outcome of interest (i.e., arrhythmia, nonfatal myocardial infarction, nonfatal stroke, cardiovascular death) during the treatment period. |
| 1600 | Hughes, John R, Callas, Peter W. Past alcohol problems do not predict worse smoking cessation outcomes. Drug and alcohol dependence. 2003;71:269. | RCTs that did not provide data on the outcome of interest (i.e., arrhythmia, nonfatal myocardial infarction, nonfatal stroke, cardiovascular death) during the treatment period. |
| 1602 | Willoughby, John O, Pope, Kenneth J, Eaton, Vaughn. Nicotine as an antiepileptic agent in ADNFLE: an N-of-one study. Epilepsia. 2003;44:1238. | RCTs that did not provide data on the outcome of interest (i.e., arrhythmia, nonfatal myocardial infarction, nonfatal stroke, cardiovascular death) during the treatment period. |
| 1605 | Yudkin, Patricia, Hey, Kate, Roberts, Sarah, Welch, Sarah, Murphy, Michael, Walton, Robert. Abstinence from smoking eight years after participation in randomised controlled trial of nicotine patch. BMJ (Clinical research ed.). 2003;327:28. | Letters to the editors, opinions, editorials, press releases, manufacturers’ advertisements, and other non-peer reviewed publications, unless the publication contained original data from RCTs. |
| 1607 | Sorensen, Lars Tue, Karlsmark, Tonny, Gottrup, Finn. Abstinence from smoking reduces incisional wound infection: a randomized controlled trial. Annals of surgery. 2003;238:1. | RCTs that did not provide data on the outcome of interest (i.e., arrhythmia, nonfatal myocardial infarction, nonfatal stroke, cardiovascular death) during the treatment period. |
| 1608 | Sørensen, Lars Tue, Jørgensen, Torben. Short-term pre-operative smoking cessation intervention does not affect postoperative complications in colorectal surgery: a randomized clinical trial. Colorectal disease : the official journal of the Association of Coloproctology of Great Britain and Ireland. 2003;5:347. | RCTs that did not provide data on the outcome of interest (i.e., arrhythmia, nonfatal myocardial infarction, nonfatal stroke, cardiovascular death) during the treatment period. |
| 1609 | Waters, Andrew J, Shiffman, Saul, Sayette, Michael A, Paty, Jean A, Gwaltney, Chad J, Balabanis, Mark H. Attentional bias predicts outcome in smoking cessation. Health psychology : official journal of the Division of Health Psychology, American Psychological Association. 2003;22:378. | RCTs that did not provide data on the outcome of interest (i.e., arrhythmia, nonfatal myocardial infarction, nonfatal stroke, cardiovascular death) during the treatment period. |
| 1610 | Mumenthaler, Martin S, Yesavage, Jerome A, Taylor, Joy L, O'Hara, Ruth, Friedman, Leah, Lee, Hana, Kraemer, Helena C. Psychoactive drugs and pilot performance: a comparison of nicotine, donepezil, and alcohol effects. Neuropsychopharmacology : official publication of the American College of Neuropsychopharmacology. 2003;28:1366. | RCTs that did not provide data on the outcome of interest (i.e., arrhythmia, nonfatal myocardial infarction, nonfatal stroke, cardiovascular death) during the treatment period. |
| 1613 | Jessen, Anna B., Toubro, Søren, Astrup, Arne. Effect of chewing gum containing nicotine and caffeine on energy expenditure and substrate utilization in men 1-3. American Journal of Clinical Nutrition. 2003;77:1442. | RCTs that did not provide data on the outcome of interest (i.e., arrhythmia, nonfatal myocardial infarction, nonfatal stroke, cardiovascular death) during the treatment period. |
| 1615 | Molyneux, A, Lewis, S, Leivers, U, Anderton, A, Antoniak, M, Brackenridge, A, Nilsson, F, McNeill, A, West, R, Moxham, J, Britton, J. Clinical trial comparing nicotine replacement therapy (NRT) plus brief counselling, brief counselling alone, and minimal intervention on smoking cessation in hospital inpatients. Thorax. 2003;58:484. | RCTs that did not provide data on the outcome of interest (i.e., arrhythmia, nonfatal myocardial infarction, nonfatal stroke, cardiovascular death) during the treatment period. |
| 1620 | Reid, Robert, Pipe, Andrew, Higginson, Lyall, Johnson, Karin, D'Angelo, Monika Slovinec, Cooke, Debbie, Dafoe, William. Stepped care approach to smoking cessation in patients hospitalized for coronary artery disease. Journal of cardiopulmonary rehabilitation. 2003;23:176. | RCTs that did not provide data on the outcome of interest (i.e., arrhythmia, nonfatal myocardial infarction, nonfatal stroke, cardiovascular death) during the treatment period. |
| 1621 | Rodríguez-Artalejo, F, Lafuente Urdinguio, P, Guallar-Castillón, P, Garteizaurrekoa Dublang, P, Sáinz Martínez, O, Díez Azcárate, J I, Foj Alemán, M, Banegas, J R. One year effectiveness of an individualised smoking cessation intervention at the workplace: a randomised controlled trial. Occupational and environmental medicine. 2003;60:358. | RCTs that did not provide data on the outcome of interest (i.e., arrhythmia, nonfatal myocardial infarction, nonfatal stroke, cardiovascular death) during the treatment period. |
| 1624 | Glavas, Duska, Rumboldt, Mirjana, Rumboldt, Zvonko. Smoking cessation with nicotine replacement therapy among health care workers: randomized double-blind study. Croatian medical journal. 2003;44:219. | RCTs that did not provide data on the outcome of interest (i.e., arrhythmia, nonfatal myocardial infarction, nonfatal stroke, cardiovascular death) during the treatment period. |
| 1628 | Ahmadi, Jamshid, Ashkani, Hamid, Ahmadi, Mojtaba, Ahmadi, Nahid. Twenty-four week maintenance treatment of cigarette smoking with nicotine gum, clonidine and naltrexone. Journal of substance abuse treatment. 2003;24:251. | RCTs that did not provide data on the outcome of interest (i.e., arrhythmia, nonfatal myocardial infarction, nonfatal stroke, cardiovascular death) during the treatment period. |
| 1630 | Cook, Mary R, Gerkovich, Mary M, Graham, Charles, Hoffman, Steven J, Peterson, Rebecca C. Effects of the nicotine patch on performance during the first week of smoking cessation. Nicotine & tobacco research : official journal of the Society for Research on Nicotine and Tobacco. 2003;5:169. | RCTs that did not provide data on the outcome of interest (i.e., arrhythmia, nonfatal myocardial infarction, nonfatal stroke, cardiovascular death) during the treatment period. |
| 1634 | Shiffman, Saul, Shadel, William G, Niaura, Raymond, Khayrallah, Moise A, Jorenby, Douglas E, Ryan, Charles F, Ferguson, Clifford L. Efficacy of acute administration of nicotine gum in relief of cue-provoked cigarette craving. Psychopharmacology. 2003;166:343. | RCTs that did not provide data on the outcome of interest (i.e., arrhythmia, nonfatal myocardial infarction, nonfatal stroke, cardiovascular death) during the treatment period. |
| 1640 | Smith, Stevens S, Jorenby, Douglas E, Leischow, Scott J, Nides, Mitchell A, Rennard, Stephen I, Johnston, J Andrew, Jamerson, Brenda, Fiore, Michael C, Baker, Timothy B. Targeting smokers at increased risk for relapse: treating women and those with a history of depression. Nicotine & tobacco research : official journal of the Society for Research on Nicotine and Tobacco. 2003;5:99. | RCTs that did not provide data on the outcome of interest (i.e., arrhythmia, nonfatal myocardial infarction, nonfatal stroke, cardiovascular death) during the treatment period. |
| 1652 | Bolliger, Chris T, Zellweger, Jean-Pierre, Danielsson, Tobias, van Biljon, Xandra, Robidou, Annik, Westin, Ake, Perruchoud, André P, Säwe, Urbain. Influence of long-term smoking reduction on health risk markers and quality of life. Nicotine & tobacco research : official journal of the Society for Research on Nicotine and Tobacco. 2002;4:433. | RCTs that did not provide data on the outcome of interest (i.e., arrhythmia, nonfatal myocardial infarction, nonfatal stroke, cardiovascular death) during the treatment period. |
| 1653 | Glover, Elbert D, Glover, Penny N, Franzon, Mikael, Sullivan, C Rollynn, Cerullo, Connie C, Howell, Robert M, Keyes, Gordon G, Nilsson, Fredrik, Hobbs, Gerald R. A comparison of a nicotine sublingual tablet and placebo for smoking cessation. Nicotine & tobacco research : official journal of the Society for Research on Nicotine and Tobacco. 2002;4:441. | RCTs that did not provide data on the outcome of interest (i.e., arrhythmia, nonfatal myocardial infarction, nonfatal stroke, cardiovascular death) during the treatment period. |
| 1654 | Shiffman, Saul, Gorsline, Jane, Gorodetzky, Charles W. Efficacy of over-the-counter nicotine patch. Nicotine & tobacco research : official journal of the Society for Research on Nicotine and Tobacco. 2002;4:477. | RCTs that did not provide data on the outcome of interest (i.e., arrhythmia, nonfatal myocardial infarction, nonfatal stroke, cardiovascular death) during the treatment period. |
| 1655 | Teneggi, Vincenzo, Tiffany, Stephen T, Squassante, Lisa, Milleri, Stefano, Ziviani, Luigi, Bye, Alan. Smokers deprived of cigarettes for 72 h: effect of nicotine patches on craving and withdrawal. Psychopharmacology. 2002;164:177. | RCTs that did not provide data on the outcome of interest (i.e., arrhythmia, nonfatal myocardial infarction, nonfatal stroke, cardiovascular death) during the treatment period. |
| 1662 | Jolma, Catherine D, Samson, Ricardo A, Klewer, Scott E, Donnerstein, Richard L, Goldberg, Stanley J. Acute cardiac effects of nicotine in healthy young adults. Echocardiography (Mount Kisco, N.Y.). 2002;19:443. | RCTs that did not provide data on the outcome of interest (i.e., arrhythmia, nonfatal myocardial infarction, nonfatal stroke, cardiovascular death) during the treatment period. |
| 1665 | Shiffman, Saul, Dresler, Carolyn M, Hajek, Peter, Gilburt, Simon J A, Targett, Darren A, Strahs, Kenneth R. Efficacy of a nicotine lozenge for smoking cessation. Archives of internal medicine. 2002;162:1267. | RCTs that did not provide data on the outcome of interest (i.e., arrhythmia, nonfatal myocardial infarction, nonfatal stroke, cardiovascular death) during the treatment period. |
| 1670 | Durcan, Michael J, White, Jonathan, Jorenby, Douglas E, Fiore, Michael C, Rennard, Stephen I, Leischow, Scott J, Nides, Mitchell A, Ascher, John A, Johnston, J Andrew. Impact of prior nicotine replacement therapy on smoking cessation efficacy. American journal of health behavior. 2002;26:213. | RCTs that did not provide data on the outcome of interest (i.e., arrhythmia, nonfatal myocardial infarction, nonfatal stroke, cardiovascular death) during the treatment period. |
| 1672 | Guslandi, Mario, Frego, Roberto, Viale, Edi, Testoni, Pier Alberto. Distal ulcerative colitis refractory to rectal mesalamine: role of transdermal nicotine versus oral mesalamine. Canadian journal of gastroenterology = Journal canadien de gastroenterologie. 2002;16:293. | RCTs that did not provide data on the outcome of interest (i.e., arrhythmia, nonfatal myocardial infarction, nonfatal stroke, cardiovascular death) during the treatment period. |
| 1675 | Thompson, J C, Wilby, G, Stough, C. The effects of transdermal nicotine on inspection time. Human psychopharmacology. 2002;17:157. | RCTs that did not provide data on the outcome of interest (i.e., arrhythmia, nonfatal myocardial infarction, nonfatal stroke, cardiovascular death) during the treatment period. |
| 1676 | Vial, Robyn J., Jones, Terry E., Ruffin, Richard E., Gilbert, Andrew L.. Smoking cessation program using nicotine patches linking hospital to the community. Journal of Pharmacy Practice and Research. 2002;32:57. | RCTs that did not provide data on the outcome of interest (i.e., arrhythmia, nonfatal myocardial infarction, nonfatal stroke, cardiovascular death) during the treatment period. |
| 1688 | Patten, C A, Martin, J E, Calfas, K J, Lento, J, Wolter, T D. Behavioral treatment for smokers with a history of alcoholism: predictors of successful outcome. Journal of consulting and clinical psychology. 2001;69:796. | RCTs that did not provide data on the outcome of interest (i.e., arrhythmia, nonfatal myocardial infarction, nonfatal stroke, cardiovascular death) during the treatment period. |
| 1689 | Vieregge, A, Sieberer, M, Jacobs, H, Hagenah, J M, Vieregge, P. Transdermal nicotine in PD: a randomized, double-blind, placebo-controlled study. Neurology. 2001;57:1032. | RCTs that did not provide data on the outcome of interest (i.e., arrhythmia, nonfatal myocardial infarction, nonfatal stroke, cardiovascular death) during the treatment period. |
| 1691 | Silver, A A, Shytle, R D, Philipp, M K, Wilkinson, B J, McConville, B, Sanberg, P R. Transdermal nicotine and haloperidol in Tourette's disorder: a double-blind placebo-controlled study. The Journal of clinical psychiatry. 2001;62:707. | RCTs that did not provide data on the outcome of interest (i.e., arrhythmia, nonfatal myocardial infarction, nonfatal stroke, cardiovascular death) during the treatment period. |
| 1694 | West, S G, Stoney, C M, Hughes, J W, Matacin, M, Emmons, K M. Oral contraceptive use is associated with increased cardiovascular reactivity in nonsmokers. Annals of behavioral medicine : a publication of the Society of Behavioral Medicine. 2001;23:149. | RCTs that did not provide data on the outcome of interest (i.e., arrhythmia, nonfatal myocardial infarction, nonfatal stroke, cardiovascular death) during the treatment period. |
| 1699 | Jamerson, B D, Nides, M, Jorenby, D E, Donahue, R, Garrett, P, Johnston, J A, Fiore, M C, Rennard, S I, Leischow, S J. Late-term smoking cessation despite initial failure: an evaluation of bupropion sustained release, nicotine patch, combination therapy, and placebo. Clinical therapeutics. 2001;23:744. | RCTs that did not provide data on the outcome of interest (i.e., arrhythmia, nonfatal myocardial infarction, nonfatal stroke, cardiovascular death) during the treatment period. |
| 1705 | Thorsteinsson, H S, Gillin, J C, Patten, C A, Golshan, S, Sutton, L D, Drummond, S, Clark, C P, Kelsoe, J, Rapaport, M. The effects of transdermal nicotine therapy for smoking cessation on depressive symptoms in patients with major depression. Neuropsychopharmacology : official publication of the American College of Neuropsychopharmacology. 2001;24:350. | RCTs that did not provide data on the outcome of interest (i.e., arrhythmia, nonfatal myocardial infarction, nonfatal stroke, cardiovascular death) during the treatment period. |
| 1706 | Gomeni, R, Teneggi, V, Iavarone, L, Squassante, L, Bye, A. Population pharmacokinetic-pharmacodynamic model of craving in an enforced smoking cessation population: indirect response and probabilistic modeling. Pharmaceutical research. 2001;18:537. | RCTs that did not provide data on the outcome of interest (i.e., arrhythmia, nonfatal myocardial infarction, nonfatal stroke, cardiovascular death) during the treatment period. |
| 1715 | Kapur, Bhushan, Hackman, Richard, Selby, Peter, Klein, Julia, Koren, Gideon. Randomized, double-blind, placebo-controlled trial of nicotine replacement therapy in pregnancy. Current Therapeutic Research - Clinical and Experimental. 2001;62:274. | RCTs that did not provide data on the outcome of interest (i.e., arrhythmia, nonfatal myocardial infarction, nonfatal stroke, cardiovascular death) during the treatment period. |
| 1718 | Wisborg, K, Henriksen, T B, Jespersen, L B, Secher, N J. Nicotine patches for pregnant smokers: a randomized controlled study. Obstetrics and gynecology. 2000;96:967. | RCTs that did not provide data on the outcome of interest (i.e., arrhythmia, nonfatal myocardial infarction, nonfatal stroke, cardiovascular death) during the treatment period. |
| 1720 | Hughes, J R, Rose, G L, Callas, P W. Nicotine is more reinforcing in smokers with a past history of alcoholism than in smokers without this history. Alcoholism, clinical and experimental research. 2000;24:1633. | RCTs that did not provide data on the outcome of interest (i.e., arrhythmia, nonfatal myocardial infarction, nonfatal stroke, cardiovascular death) during the treatment period. |
| 1721 | Fishbein, L, O'Brien, P, Hutson, A, Theriaque, D, Stacpoole, P W, Flotte, T. Pharmacokinetics and pharmacodynamic effects of nicotine nasal spray devices on cardiovascular and pulmonary function. Journal of investigative medicine : the official publication of the American Federation for Clinical Research. 2000;48:435. | RCTs that did not provide data on the outcome of interest (i.e., arrhythmia, nonfatal myocardial infarction, nonfatal stroke, cardiovascular death) during the treatment period. |
| 1723 | Shiffman, S, Khayrallah, M, Nowak, R. Efficacy of the nicotine patch for relief of craving and withdrawal 7-10 weeks after cessation. Nicotine & tobacco research : official journal of the Society for Research on Nicotine and Tobacco. 2000;2:371. | RCTs that did not provide data on the outcome of interest (i.e., arrhythmia, nonfatal myocardial infarction, nonfatal stroke, cardiovascular death) during the treatment period. |
| 1726 | Killen, J D, Fortmann, S P, Schatzberg, A F, Hayward, C, Sussman, L, Rothman, M, Strausberg, L, Varady, A. Nicotine patch and paroxetine for smoking cessation. Journal of consulting and clinical psychology. 2000;68:883. | RCTs that did not include a nicotine versus no nicotine intervention. |
| 1730 | Bolliger, C T, Zellweger, J P, Danielsson, T, van Biljon, X, Robidou, A, Westin, A, Perruchoud, A P, Säwe, U. Smoking reduction with oral nicotine inhalers: double blind, randomised clinical trial of efficacy and safety. BMJ (Clinical research ed.). 2000;321:329. | RCTs that did not provide data on the outcome of interest (i.e., arrhythmia, nonfatal myocardial infarction, nonfatal stroke, cardiovascular death) during the treatment period. |
| 1733 | Allen, S S, Hatsukami, D, Christianson, D, Brown, S. Effects of transdermal nicotine on craving, withdrawal and premenstrual symptomatology in short-term smoking abstinence during different phases of the menstrual cycle. Nicotine & tobacco research : official journal of the Society for Research on Nicotine and Tobacco. 2000;2:231. | RCTs that did not provide data on the outcome of interest (i.e., arrhythmia, nonfatal myocardial infarction, nonfatal stroke, cardiovascular death) during the treatment period. |
| 1734 | Hill, A L, Roe, D J, Taren, D L, Muramoto, M M, Leischow, S J. Efficacy of transdermal nicotine in reducing post-cessation weight gain in a Hispanic sample. Nicotine & tobacco research : official journal of the Society for Research on Nicotine and Tobacco. 2000;2:247. | RCTs that did not provide data on the outcome of interest (i.e., arrhythmia, nonfatal myocardial infarction, nonfatal stroke, cardiovascular death) during the treatment period. |
| 1735 | Hughes, J R, Rose, G L, Callas, P W. Do former smokers respond to nicotine differently from never smokers? A pilot study. Nicotine & tobacco research : official journal of the Society for Research on Nicotine and Tobacco. 2000;2:255. | RCTs that did not provide data on the outcome of interest (i.e., arrhythmia, nonfatal myocardial infarction, nonfatal stroke, cardiovascular death) during the treatment period. |
| 1739 | Molander, L, Lunell, E, Fagerström, K O. Reduction of tobacco withdrawal symptoms with a sublingual nicotine tablet: a placebo controlled study. Nicotine & tobacco research : official journal of the Society for Research on Nicotine and Tobacco. 2000;2:187. | RCTs that did not provide data on the outcome of interest (i.e., arrhythmia, nonfatal myocardial infarction, nonfatal stroke, cardiovascular death) during the treatment period. |
| 1740 | Hatsukami, D K, Grillo, M, Boyle, R, Allen, S, Jensen, J, Bliss, R, Brown, S. Treatment of spit tobacco users with transdermal nicotine system and mint snuff. Journal of consulting and clinical psychology. 2000;68:241. | RCTs that did not provide data on the outcome of interest (i.e., arrhythmia, nonfatal myocardial infarction, nonfatal stroke, cardiovascular death) during the treatment period. |
| 1742 | Patten, Christi A., Martin, John E., Calfas, Karen J., Brown, Sandra A., Schroeder, Darrell R.. Brief report effect of three smoking cessation treatments on nicotine withdrawal in 141 abstinent alcoholic smokers. Addictive Behaviors. 2000;25:301. | RCTs that did not provide data on the outcome of interest (i.e., arrhythmia, nonfatal myocardial infarction, nonfatal stroke, cardiovascular death) during the treatment period. |
| 1744 | Garvey, A J, Kinnunen, T, Nordstrom, B L, Utman, C H, Doherty, K, Rosner, B, Vokonas, P S. Effects of nicotine gum dose by level of nicotine dependence. Nicotine & tobacco research : official journal of the Society for Research on Nicotine and Tobacco. 2000;2:53. | RCTs that did not provide data on the outcome of interest (i.e., arrhythmia, nonfatal myocardial infarction, nonfatal stroke, cardiovascular death) during the treatment period. |
| 1756 | Howard-Pitney, B, Killen, J D, Fortmann, S P. Quitting chew: results from a randomized trial using nicotine patches. Experimental and clinical psychopharmacology. 1999;7:362. | RCTs that did not provide data on the outcome of interest (i.e., arrhythmia, nonfatal myocardial infarction, nonfatal stroke, cardiovascular death) during the treatment period. |
| 1759 | Nordstrom, B L, Kinnunen, T, Utman, C H, Garvey, A J. Long-term effects of nicotine gum on weight gain after smoking cessation. Nicotine & tobacco research : official journal of the Society for Research on Nicotine and Tobacco. 1999;1:259. | RCTs that did not provide data on the outcome of interest (i.e., arrhythmia, nonfatal myocardial infarction, nonfatal stroke, cardiovascular death) during the treatment period. |
| 1762 | Wong, G Y, Wolter, T D, Croghan, G A, Croghan, I T, Offord, K P, Hurt, R D. A randomized trial of naltrexone for smoking cessation. Addiction (Abingdon, England). 1999;94:1227. | RCTs that did not provide data on the outcome of interest (i.e., arrhythmia, nonfatal myocardial infarction, nonfatal stroke, cardiovascular death) during the treatment period. |
| 1766 | Dalack, G W, Becks, L, Hill, E, Pomerleau, O F, Meador-Woodruff, J H. Nicotine withdrawal and psychiatric symptoms in cigarette smokers with schizophrenia. Neuropsychopharmacology : official publication of the American College of Neuropsychopharmacology. 1999;21:195. | RCTs that did not provide data on the outcome of interest (i.e., arrhythmia, nonfatal myocardial infarction, nonfatal stroke, cardiovascular death) during the treatment period. |
| 1771 | Hughes, J R, Lesmes, G R, Hatsukami, D K, Richmond, R L, Lichtenstein, E, Jorenby, D E, Broughton, J O, Fortmann, S P, Leischow, S J, McKenna, J P, Rennard, S I, Wadland, W C, Heatley, S A. Are higher doses of nicotine replacement more effective for smoking cessation?. Nicotine & tobacco research : official journal of the Society for Research on Nicotine and Tobacco. 1999;1:169. | RCTs that did not provide data on the outcome of interest (i.e., arrhythmia, nonfatal myocardial infarction, nonfatal stroke, cardiovascular death) during the treatment period. |
| 1773 | Wetter, D W, Fiore, M C, Young, T B, McClure, J B, de Moor, C A, Baker, T B. Gender differences in response to nicotine replacement therapy: objective and subjective indexes of tobacco withdrawal. Experimental and clinical psychopharmacology. 1999;7:135. | RCTs that did not provide data on the outcome of interest (i.e., arrhythmia, nonfatal myocardial infarction, nonfatal stroke, cardiovascular death) during the treatment period. |
| 1774 | Kanner, R E, Connett, J E, Williams, D E, Buist, A S. Effects of randomized assignment to a smoking cessation intervention and changes in smoking habits on respiratory symptoms in smokers with early chronic obstructive pulmonary disease: the Lung Health Study. The American journal of medicine. 1999;106:410. | RCTs that did not provide data on the outcome of interest (i.e., arrhythmia, nonfatal myocardial infarction, nonfatal stroke, cardiovascular death) during the treatment period. |
| 1775 | Blondal, T.. Correction: Nicotine nasal spray with nicotine patch for smoking cessation: Randomised trial with six year follow up (British Medical Journal (1999) 30 January (285-289)). British Medical Journal. 1999;318:764. | Letters to the editors, opinions, editorials, press releases, manufacturers’ advertisements, and other non-peer reviewed publications, unless the publication contained original data from RCTs. |
| 1778 | Dey, P, Foy, R, Woodman, M, Fullard, B, Gibbs, A. Should smoking cessation cost a packet? A pilot randomized controlled trial of the cost-effectiveness of distributing nicotine therapy free of charge. The British journal of general practice : the journal of the Royal College of General Practitioners. 1999;49:127. | RCTs that did not include a nicotine versus no nicotine intervention. |
| 1785 | Davidson, M, Epstein, M, Burt, R, Schaefer, C, Whitworth, G, McDonald, A. Efficacy and safety of an over-the-counter transdermal nicotine patch as an aid for smoking cessation. Archives of family medicine. 1998;7:569. | RCTs that did not provide data on the outcome of interest (i.e., arrhythmia, nonfatal myocardial infarction, nonfatal stroke, cardiovascular death) during the treatment period. |
| 1786 | Hurt, R D, Offord, K P, Croghan, I T, Croghan, G A, Gomez-Dahl, L C, Wolter, T D, Dale, L C, Moyer, T P. Temporal effects of nicotine nasal spray and gum on nicotine withdrawal symptoms. Psychopharmacology. 1998;140:98. | RCTs that did not provide data on the outcome of interest (i.e., arrhythmia, nonfatal myocardial infarction, nonfatal stroke, cardiovascular death) during the treatment period. |
| 1788 | Ahluwalia, J S, Resnicow, K, Clark, W S. Knowledge about smoking, reasons for smoking, and reasons for wishing to quit in inner-city African Americans. Ethnicity & disease. 1998;8:385. | RCTs that did not provide data on the outcome of interest (i.e., arrhythmia, nonfatal myocardial infarction, nonfatal stroke, cardiovascular death) during the treatment period. |
| 1790 | Daughton, D, Susman, J, Sitorius, M, Belenky, S, Millatmal, T, Nowak, R, Patil, K, Rennard, S I. Transdermal nicotine therapy and primary care. Importance of counseling, demographic, and participant selection factors on 1-year quit rates. The Nebraska Primary Practice Smoking Cessation Trial Group. Archives of family medicine. 1998;7:425. | RCTs that did not provide data on the outcome of interest (i.e., arrhythmia, nonfatal myocardial infarction, nonfatal stroke, cardiovascular death) during the treatment period. |
| 1791 | van Dijk, A P, Meijssen, M A, Brouwer, A J, Hop, W C, van Bergeijk, J D, Feyerabend, C, Wilson, J H, Zijlstra, F J. Transdermal nicotine inhibits interleukin 2 synthesis by mononuclear cells derived from healthy volunteers. European journal of clinical investigation. 1998;28:664. | RCTs that did not provide data on the outcome of interest (i.e., arrhythmia, nonfatal myocardial infarction, nonfatal stroke, cardiovascular death) during the treatment period. |
| 1792 | Rose, J E, Behm, F M, Westman, E C. Nicotine-mecamylamine treatment for smoking cessation: the role of pre-cessation therapy. Experimental and clinical psychopharmacology. 1998;6:331. | RCTs that did not provide data on the outcome of interest (i.e., arrhythmia, nonfatal myocardial infarction, nonfatal stroke, cardiovascular death) during the treatment period. |
| 1793 | Perng, R P, Hsieh, W C, Chen, Y M, Lu, C C, Chiang, S J. Randomized, double-blind, placebo-controlled study of transdermal nicotine patch for smoking cessation. Journal of the Formosan Medical Association = Taiwan yi zhi. 1998;97:547. | RCTs that did not provide data on the outcome of interest (i.e., arrhythmia, nonfatal myocardial infarction, nonfatal stroke, cardiovascular death) during the treatment period. |
| 1794 | Tzivoni, D, Keren, A, Meyler, S, Khoury, Z, Lerer, T, Brunel, P. Cardiovascular safety of transdermal nicotine patches in patients with coronary artery disease who try to quit smoking. Cardiovascular drugs and therapy. 1998;12:239. | RCTs that did not include a nicotine versus no nicotine intervention. |
| 1796 | Lawson, G M, Hurt, R D, Dale, L C, Offord, K P, Croghan, I T, Schroeder, D R, Jiang, N S. Application of serum nicotine and plasma cotinine concentrations to assessment of nicotine replacement in light, moderate, and heavy smokers undergoing transdermal therapy. Journal of clinical pharmacology. 1998;38:502. | RCTs that did not provide data on the outcome of interest (i.e., arrhythmia, nonfatal myocardial infarction, nonfatal stroke, cardiovascular death) during the treatment period. |
| 1797 | Lawson, G M, Hurt, R D, Dale, L C, Offord, K P, Croghan, I T, Schroeder, D R, Jiang, N S. Application of urine nicotine and cotinine excretion rates to assessment of nicotine replacement in light, moderate, and heavy smokers undergoing transdermal therapy. Journal of clinical pharmacology. 1998;38:510. | Duplicate articles or articles with the exact same study outcome data as another published article. |
| 1799 | Senore, C, Battista, R N, Shapiro, S H, Segnan, N, Ponti, A, Rosso, S, Aimar, D. Predictors of smoking cessation following physicians' counseling. Preventive medicine. 1998;27:412. | RCTs that did not provide data on the outcome of interest (i.e., arrhythmia, nonfatal myocardial infarction, nonfatal stroke, cardiovascular death) during the treatment period. |
| 1801 | Stapleton, J A, Sutherland, G, Russell, M A. How much does relapse after one year erode effectiveness of smoking cessation treatments? Long-term follow up of randomised trial of nicotine nasal spray. BMJ (Clinical research ed.). 1998;316:830. | RCTs that did not provide data on the outcome of interest (i.e., arrhythmia, nonfatal myocardial infarction, nonfatal stroke, cardiovascular death) during the treatment period. |
| 1804 | West, R, Willis, N. Double-blind placebo controlled trial of dextrose tablets and nicotine patch in smoking cessation. Psychopharmacology. 1998;136:201. | RCTs that did not provide data on the outcome of interest (i.e., arrhythmia, nonfatal myocardial infarction, nonfatal stroke, cardiovascular death) during the treatment period. |
| 1806 | Ahluwalia, J S, McNagny, S E, Clark, W S. Smoking cessation among inner-city African Americans using the nicotine transdermal patch. Journal of general internal medicine. 1998;13:1. | RCTs that did not provide data on the outcome of interest (i.e., arrhythmia, nonfatal myocardial infarction, nonfatal stroke, cardiovascular death) during the treatment period. |
| 1807 | Dale, L C, Schroeder, D R, Wolter, T D, Croghan, I T, Hurt, R D, Offord, K P. Weight change after smoking cessation using variable doses of transdermal nicotine replacement. Journal of general internal medicine. 1998;13:9. | RCTs that did not provide data on the outcome of interest (i.e., arrhythmia, nonfatal myocardial infarction, nonfatal stroke, cardiovascular death) during the treatment period. |
| 1808 | Hatsukami, D, Pentel, P R, Jensen, J, Nelson, D, Allen, S S, Goldman, A, Rafael, D. Cotinine: effects with and without nicotine. Psychopharmacology. 1998;135:141. | RCTs that did not provide data on the outcome of interest (i.e., arrhythmia, nonfatal myocardial infarction, nonfatal stroke, cardiovascular death) during the treatment period. |
| 1809 | Richmond, R L, Kehoe, L, de Almeida Neto, A C. Three year continuous abstinence in a smoking cessation study using the nicotine transdermal patch. Heart (British Cardiac Society). 1997;78:617. | RCTs that did not provide data on the outcome of interest (i.e., arrhythmia, nonfatal myocardial infarction, nonfatal stroke, cardiovascular death) during the treatment period. |
| 1811 | Ginsberg, J P, Klesges, R C, Johnson, K C, Eck, L H, Meyers, A W, Winders, S A. The relationship between a history of depression and adherence to a multicomponent smoking-cessation program. Addictive behaviors. 1997;22:783. | RCTs that did not provide data on the outcome of interest (i.e., arrhythmia, nonfatal myocardial infarction, nonfatal stroke, cardiovascular death) during the treatment period. |
| 1814 | Hjalmarson, A, Nilsson, F, Sjöström, L, Wiklund, O. The nicotine inhaler in smoking cessation. Archives of internal medicine. 1997;157:1721. | RCTs that did not provide data on the outcome of interest (i.e., arrhythmia, nonfatal myocardial infarction, nonfatal stroke, cardiovascular death) during the treatment period. |
| 1815 | Killen, J D, Fortmann, S P, Davis, L, Varady, A. Nicotine patch and self-help video for cigarette smoking cessation. Journal of consulting and clinical psychology. 1997;65:663. | RCTs that did not provide data on the outcome of interest (i.e., arrhythmia, nonfatal myocardial infarction, nonfatal stroke, cardiovascular death) during the treatment period. |
| 1816 | Blöndal, T, Franzon, M, Westin, A. A double-blind randomized trial of nicotine nasal spray as an aid in smoking cessation. The European respiratory journal. 1997;10:1585. | RCTs that did not provide data on the outcome of interest (i.e., arrhythmia, nonfatal myocardial infarction, nonfatal stroke, cardiovascular death) during the treatment period. |
| 1817 | Simon, J A, Solkowitz, S N, Carmody, T P, Browner, W S. Smoking cessation after surgery. A randomized trial. Archives of internal medicine. 1997;157:1371. | RCTs that did not provide data on the outcome of interest (i.e., arrhythmia, nonfatal myocardial infarction, nonfatal stroke, cardiovascular death) during the treatment period. |
| 1823 | Sønderskov, J, Olsen, J, Sabroe, S, Meillier, L, Overvad, K. Nicotine patches in smoking cessation: a randomized trial among over-the-counter customers in Denmark. American journal of epidemiology. 1997;145:309. | RCTs that did not provide data on the outcome of interest (i.e., arrhythmia, nonfatal myocardial infarction, nonfatal stroke, cardiovascular death) during the treatment period. |
| 1825 | Martin, J E, Calfas, K J, Patten, C A, Polarek, M, Hofstetter, C R, Noto, J, Beach, D. Prospective evaluation of three smoking interventions in 205 recovering alcoholics: one-year results of Project SCRAP-Tobacco. Journal of consulting and clinical psychology. 1997;65:190. | RCTs that did not provide data on the outcome of interest (i.e., arrhythmia, nonfatal myocardial infarction, nonfatal stroke, cardiovascular death) during the treatment period. |
| 1826 | Mattsson, U, Krogstad, A L, Pegenius, G, Elam, M, Jontell, M. Assessment of erythema and skin perfusion by digital image analysis and scanner laser Doppler. Skin research and technology : official journal of International Society for Bioengineering and the Skin (ISBS) [and] International Society for Digital Imaging of Skin (ISDIS) [and] International Society for Skin Imaging (ISSI). 1997;3:53. | RCTs that did not provide data on the outcome of interest (i.e., arrhythmia, nonfatal myocardial infarction, nonfatal stroke, cardiovascular death) during the treatment period. |
| 1827 | Pulerà, N, Petruzzelli, S, Celi, A, Puntoni, R, Fornai, E, Säwe, U, Paoletti, P, Giuntini, C. Presence and persistence of serum anti-benzoapyrene diolepoxide-DNA adduct antibodies in smokers: effects of smoking reduction and cessation. International journal of cancer. 1997;70:145. | RCTs that did not provide data on the outcome of interest (i.e., arrhythmia, nonfatal myocardial infarction, nonfatal stroke, cardiovascular death) during the treatment period. |
| 1828 | Richmond, R L, Kehoe, L, de Almeida Neto, A C. Effectiveness of a 24-hour transdermal nicotine patch in conjunction with a cognitive behavioural programme: one year outcome. Addiction (Abingdon, England). 1997;92:27. | RCTs that did not provide data on the outcome of interest (i.e., arrhythmia, nonfatal myocardial infarction, nonfatal stroke, cardiovascular death) during the treatment period. |
| 1831 | Hughes, J R, Hatsukami, D K. Effects of three doses of transdermal nicotine on post-cessation eating, hunger and weight. Journal of substance abuse. 1997;9:151. | RCTs that did not provide data on the outcome of interest (i.e., arrhythmia, nonfatal myocardial infarction, nonfatal stroke, cardiovascular death) during the treatment period. |
| 1832 | Ohnishi, H. Cisapride improves nicotine-evoked antral hypomotility in smokers. The Kurume medical journal. 1997;44:253. | RCTs that did not provide data on the outcome of interest (i.e., arrhythmia, nonfatal myocardial infarction, nonfatal stroke, cardiovascular death) during the treatment period. |
| 1833 | Clavel-Chapelon, F, Paoletti, C, Benhamou, S. Smoking cessation rates 4 years after treatment by nicotine gum and acupuncture. Preventive medicine. 1997;26:25. | RCTs that did not provide data on the outcome of interest (i.e., arrhythmia, nonfatal myocardial infarction, nonfatal stroke, cardiovascular death) during the treatment period. |
| 1836 | Taylor, C B, Miller, N H, Herman, S, Smith, P M, Sobel, D, Fisher, L, DeBusk, R F. A nurse-managed smoking cessation program for hospitalized smokers. American journal of public health. 1996;86:1557. | RCTs that did not include a nicotine versus no nicotine intervention. |
| 1837 | Levin, E D, Wilson, W, Rose, J E, McEvoy, J. Nicotine-haloperidol interactions and cognitive performance in schizophrenics. Neuropsychopharmacology : official publication of the American College of Neuropsychopharmacology. 1996;15:429. | RCTs that did not provide data on the outcome of interest (i.e., arrhythmia, nonfatal myocardial infarction, nonfatal stroke, cardiovascular death) during the treatment period. |
| 1838 | Krogstad, A L, Pegenius, G, Elam, M. Visual scoring and laser Doppler perfusion imaging of skin irritancy induced by different nicotine patches. Skin research and technology : official journal of International Society for Bioengineering and the Skin (ISBS) [and] International Society for Digital Imaging of Skin (ISDIS) [and] International Society for Skin Imaging (ISSI). 1996;2:158. | RCTs that did not provide data on the outcome of interest (i.e., arrhythmia, nonfatal myocardial infarction, nonfatal stroke, cardiovascular death) during the treatment period. |
| 1840 | Nilsson, P, Lundgren, H, Söderström, M, Fagerström, K O, Nilsson-Ehle, P. Effects of smoking cessation on insulin and cardiovascular risk factors--a controlled study of 4 months' duration. Journal of internal medicine. 1996;240:189. | RCTs that did not provide data on the outcome of interest (i.e., arrhythmia, nonfatal myocardial infarction, nonfatal stroke, cardiovascular death) during the treatment period. |
| 1842 | Schneider, N G, Olmstead, R, Nilsson, F, Mody, F V, Franzon, M, Doan, K. Efficacy of a nicotine inhaler in smoking cessation: a double-blind, placebo-controlled trial. Addiction (Abingdon, England). 1996;91:1293. | RCTs that did not provide data on the outcome of interest (i.e., arrhythmia, nonfatal myocardial infarction, nonfatal stroke, cardiovascular death) during the treatment period. |
| 1845 | Thomas, G A, Rhodes, J, Ragunath, K, Mani, V, Williams, G T, Newcombe, R G, Russell, M A, Feyerabend, C. Transdermal nicotine compared with oral prednisolone therapy for active ulcerative colitis. European journal of gastroenterology & hepatology. 1996;8:769. | RCTs that did not provide data on the outcome of interest (i.e., arrhythmia, nonfatal myocardial infarction, nonfatal stroke, cardiovascular death) during the treatment period. |
| 1848 | Khoury, Z, Comans, P, Keren, A, Lerer, T, Gavish, A, Tzivoni, D. Effects of transdermal nicotine patches on ambulatory ECG monitoring findings: a double-blind study in healthy smokers. Cardiovascular drugs and therapy. 1996;10:179. | RCTs that did not include a nicotine versus no nicotine intervention. |
| 1850 | Paoletti, P, Fornai, E, Maggiorelli, F, Puntoni, R, Viegi, G, Carrozzi, L, Corlando, A, Gustavsson, G, Säwe, U, Giuntini, C. Importance of baseline cotinine plasma values in smoking cessation: results from a double-blind study with nicotine patch. The European respiratory journal. 1996;9:643. | RCTs that did not provide data on the outcome of interest (i.e., arrhythmia, nonfatal myocardial infarction, nonfatal stroke, cardiovascular death) during the treatment period. |
| 1851 | Yudkin, P L, Jones, L, Lancaster, T, Fowler, G H. Which smokers are helped to give up smoking using transdermal nicotine patches? Results from a randomized, double-blind, placebo-controlled trial. The British journal of general practice : the journal of the Royal College of General Practitioners. 1996;46:145. | RCTs that did not provide data on the outcome of interest (i.e., arrhythmia, nonfatal myocardial infarction, nonfatal stroke, cardiovascular death) during the treatment period. |
| 1852 | Murray, R P, Bailey, W C, Daniels, K, Bjornson, W M, Kurnow, K, Connett, J E, Nides, M A, Kiley, J P. Safety of nicotine polacrilex gum used by 3,094 participants in the Lung Health Study. Lung Health Study Research Group. Chest. 1996;109:438. | RCTs that did not include a nicotine versus no nicotine intervention. |
| 1853 | Hatsukami, D, Jensen, J, Allen, S, Grillo, M, Bliss, R. Effects of behavioral and pharmacological treatment on smokeless tobacco users. Journal of consulting and clinical psychology. 1996;64:153. | RCTs that did not provide data on the outcome of interest (i.e., arrhythmia, nonfatal myocardial infarction, nonfatal stroke, cardiovascular death) during the treatment period. |
| 1854 | Murray, Robert P., Bailey, William C., Daniels, Kathleen, Bjornson, Wendy M., Kurnow, Karole, Connett, John E., Nides, Mitchell A., Kiley, James P.. Safety of nicotine polacrilex gum used by 3,094 participants in the Lung Health Study. Chest. 1996;109:438. | Duplicate articles or articles with the exact same study outcome data as another published article. |
| 1855 | Schneider, N G, Olmstead, R, Mody, F V, Doan, K, Franzon, M, Jarvik, M E, Steinberg, C. Efficacy of a nicotine nasal spray in smoking cessation: a placebo-controlled, double-blind trial. Addiction (Abingdon, England). 1995;90:1671. | RCTs that did not provide data on the outcome of interest (i.e., arrhythmia, nonfatal myocardial infarction, nonfatal stroke, cardiovascular death) during the treatment period. |
| 1858 | Dale, L C, Hurt, R D, Offord, K P, Lawson, G M, Croghan, I T, Schroeder, D R. High-dose nicotine patch therapy. Percentage of replacement and smoking cessation. JAMA. 1995;274:1353. | RCTs that did not provide data on the outcome of interest (i.e., arrhythmia, nonfatal myocardial infarction, nonfatal stroke, cardiovascular death) during the treatment period. |
| 1861 | Gourlay, S G, Forbes, A, Marriner, T, Pethica, D, McNeil, J J. Double blind trial of repeated treatment with transdermal nicotine for relapsed smokers. BMJ (Clinical research ed.). 1995;311:363. | RCTs that did not include a nicotine versus no nicotine intervention. |
| 1862 | Herrera, N, Franco, R, Herrera, L, Partidas, A, Rolando, R, Fagerström, K O. Nicotine gum, 2 and 4 mg, for nicotine dependence. A double-blind placebo-controlled trial within a behavior modification support program. Chest. 1995;108:447. | RCTs that did not provide data on the outcome of interest (i.e., arrhythmia, nonfatal myocardial infarction, nonfatal stroke, cardiovascular death) during the treatment period. |
| 1863 | Wetter, D W, Fiore, M C, Baker, T B, Young, T B. Tobacco withdrawal and nicotine replacement influence objective measures of sleep. Journal of consulting and clinical psychology. 1995;63:658. | RCTs that did not provide data on the outcome of interest (i.e., arrhythmia, nonfatal myocardial infarction, nonfatal stroke, cardiovascular death) during the treatment period. |
| 1864 | Allen, S S, Hatsukami, D, Jensen, J, Grillo, M, Bliss, R. Effects of treatment on cardiovascular risk among smokeless tobacco users. Preventive medicine. 1995;24:357. | RCTs that did not provide data on the outcome of interest (i.e., arrhythmia, nonfatal myocardial infarction, nonfatal stroke, cardiovascular death) during the treatment period. |
| 1865 | Fortmann, S P, Killen, J D. Nicotine gum and self-help behavioral treatment for smoking relapse prevention: results from a trial using population-based recruitment. Journal of consulting and clinical psychology. 1995;63:460. | RCTs that did not provide data on the outcome of interest (i.e., arrhythmia, nonfatal myocardial infarction, nonfatal stroke, cardiovascular death) during the treatment period. |
| 1866 | Søreide, E, Holst-Larsen, H, Veel, T, Steen, P A. The effects of chewing gum on gastric content prior to induction of general anesthesia. Anesthesia and analgesia. 1995;80:985. | RCTs that did not provide data on the outcome of interest (i.e., arrhythmia, nonfatal myocardial infarction, nonfatal stroke, cardiovascular death) during the treatment period. |
| 1870 | Hurt, R D, Offord, K P, Lauger, G G, Marusić, Z, Fagerström, K O, Enright, P L, Scanlon, P D. Cessation of long-term nicotine gum use--a prospective, randomized trial. Addiction (Abingdon, England). 1995;90:407. | RCTs that did not provide data on the outcome of interest (i.e., arrhythmia, nonfatal myocardial infarction, nonfatal stroke, cardiovascular death) during the treatment period. |
| 1871 | Stapleton, J A, Russell, M A, Feyerabend, C, Wiseman, S M, Gustavsson, G, Sawe, U, Wiseman, D. Dose effects and predictors of outcome in a randomized trial of transdermal nicotine patches in general practice. Addiction (Abingdon, England). 1995;90:31. | RCTs that did not provide data on the outcome of interest (i.e., arrhythmia, nonfatal myocardial infarction, nonfatal stroke, cardiovascular death) during the treatment period. |
| 1874 | Lunell, E, Molander, L, Leischow, S J, Fagerström, K O. Effect of nicotine vapour inhalation on the relief of tobacco withdrawal symptoms. European journal of clinical pharmacology. 1995;48:235. | RCTs that did not provide data on the outcome of interest (i.e., arrhythmia, nonfatal myocardial infarction, nonfatal stroke, cardiovascular death) during the treatment period. |
| 1877 | Kornitzer, M, Boutsen, M, Dramaix, M, Thijs, J, Gustavsson, G. Combined use of nicotine patch and gum in smoking cessation: a placebo-controlled clinical trial. Preventive medicine. 1995;24:41. | RCTs that did not provide data on the outcome of interest (i.e., arrhythmia, nonfatal myocardial infarction, nonfatal stroke, cardiovascular death) during the treatment period. |
| 1878 | Clemens, P, Baron, J A, Coffey, D, Reeves, A. The short-term effect of nicotine chewing gum in patients with Parkinson's disease. Psychopharmacology. 1995;117:253. | RCTs that did not provide data on the outcome of interest (i.e., arrhythmia, nonfatal myocardial infarction, nonfatal stroke, cardiovascular death) during the treatment period. |
| 1880 | Hjalmarson, A, Franzon, M, Westin, A, Wiklund, O. Effect of nicotine nasal spray on smoking cessation. A randomized, placebo-controlled, double-blind study. Archives of internal medicine. 1994;154:2567. | RCTs that did not provide data on the outcome of interest (i.e., arrhythmia, nonfatal myocardial infarction, nonfatal stroke, cardiovascular death) during the treatment period. |
| 1882 | Wewers, M E, Tejwani, G A, Anderson, J. Plasma nicotine, plasma beta-endorphin and mood states during periods of chronic smoking, abstinence and nicotine replacement. Psychopharmacology. 1994;116:98. | RCTs that did not provide data on the outcome of interest (i.e., arrhythmia, nonfatal myocardial infarction, nonfatal stroke, cardiovascular death) during the treatment period. |
| 1884 | Davila, D G, Hurt, R D, Offord, K P, Harris, C D, Shepard, J W, Jr. Acute effects of transdermal nicotine on sleep architecture, snoring, and sleep-disordered breathing in nonsmokers. American journal of respiratory and critical care medicine. 1994;150:469. | RCTs that did not provide data on the outcome of interest (i.e., arrhythmia, nonfatal myocardial infarction, nonfatal stroke, cardiovascular death) during the treatment period. |
| 1885 | Richmond, R L, Harris, K, de Almeida Neto, A. The transdermal nicotine patch: results of a randomised placebo-controlled trial. The Medical journal of Australia. 1994;161:130. | RCTs that did not provide data on the outcome of interest (i.e., arrhythmia, nonfatal myocardial infarction, nonfatal stroke, cardiovascular death) during the treatment period. |
| 1888 | . Randomised trial of nicotine patches in general practice: results at one year. Imperial Cancer Research Fund General Practice Research Group. BMJ (Clinical research ed.). 1994;308:1476. | Letters to the editors, opinions, editorials, press releases, manufacturers’ advertisements, and other non-peer reviewed publications, unless the publication contained original data from RCTs. |
| 1889 | Fowler, Godfrey. Randomised trial of nicotine patches in general practice: Results at one year. British Medical Journal. 1994;308:1476. | RCTs that did not provide data on the outcome of interest (i.e., arrhythmia, nonfatal myocardial infarction, nonfatal stroke, cardiovascular death) during the treatment period. |
| 1890 | Spiga, R., Bennett, R.H., Schmitz, J., Cherek, D.R.. Effects of nicotine on cooperative responding among abstinent male smokers. Behavioural pharmacology. 1994;5:337. | RCTs that did not provide data on the outcome of interest (i.e., arrhythmia, nonfatal myocardial infarction, nonfatal stroke, cardiovascular death) during the treatment period. |
| 1891 | Pickworth, W B, Bunker, E B, Henningfield, J E. Transdermal nicotine: reduction of smoking with minimal abuse liability. Psychopharmacology. 1994;115:9. | RCTs that did not provide data on the outcome of interest (i.e., arrhythmia, nonfatal myocardial infarction, nonfatal stroke, cardiovascular death) during the treatment period. |
| 1893 | Fortmann, Stephen P.. Nicotine replacement therapy for patients with coronary artery disease. Archives of Internal Medicine. 1994;154:989. | RCTs that did not include a nicotine versus no nicotine intervention. |
| 1895 | Ockene, J K, Kristeller, J, Pbert, L, Hebert, J R, Luippold, R, Goldberg, R J, Landon, J, Kalan, K. The physician-delivered smoking intervention project: can short-term interventions produce long-term effects for a general outpatient population?. Health psychology : official journal of the Division of Health Psychology, American Psychological Association. 1994;13:278. | RCTs that did not provide data on the outcome of interest (i.e., arrhythmia, nonfatal myocardial infarction, nonfatal stroke, cardiovascular death) during the treatment period. |
| 1896 | Pullan, R D, Rhodes, J, Ganesh, S, Mani, V, Morris, J S, Williams, G T, Newcombe, R G, Russell, M A, Feyerabend, C, Thomas, G A. Transdermal nicotine for active ulcerative colitis. The New England journal of medicine. 1994;330:811. | Duplicate articles or articles with the exact same study outcome data as another published article. |
| 1897 | Kenford, S L, Fiore, M C, Jorenby, D E, Smith, S S, Wetter, D, Baker, T B. Predicting smoking cessation. Who will quit with and without the nicotine patch. JAMA. 1994;271:589. | RCTs that did not provide data on the outcome of interest (i.e., arrhythmia, nonfatal myocardial infarction, nonfatal stroke, cardiovascular death) during the treatment period. |
| 1898 | Hurt, R D, Dale, L C, Fredrickson, P A, Caldwell, C C, Lee, G A, Offord, K P, Lauger, G G, Marŭsić, Z, Neese, L W, Lundberg, T G. Nicotine patch therapy for smoking cessation combined with physician advice and nurse follow-up. One-year outcome and percentage of nicotine replacement. JAMA. 1994;271:595. | RCTs that did not provide data on the outcome of interest (i.e., arrhythmia, nonfatal myocardial infarction, nonfatal stroke, cardiovascular death) during the treatment period. |
| 1900 | Fiore, M C, Kenford, S L, Jorenby, D E, Wetter, D W, Smith, S S, Baker, T B. Two studies of the clinical effectiveness of the nicotine patch with different counseling treatments. Chest. 1994;105:524. | RCTs that did not provide data on the outcome of interest (i.e., arrhythmia, nonfatal myocardial infarction, nonfatal stroke, cardiovascular death) during the treatment period. |
| 1908 | Niaura, R, Goldstein, M G, Abrams, D B. Matching high- and low-dependence smokers to self-help treatment with or without nicotine replacement. Preventive medicine. 1994;23:70. | RCTs that did not provide data on the outcome of interest (i.e., arrhythmia, nonfatal myocardial infarction, nonfatal stroke, cardiovascular death) during the treatment period. |
| 1911 | Westman, E C, Levin, E D, Rose, J E. The nicotine patch in smoking cessation. A randomized trial with telephone counseling. Archives of internal medicine. 1993;153:1917. | RCTs that did not provide data on the outcome of interest (i.e., arrhythmia, nonfatal myocardial infarction, nonfatal stroke, cardiovascular death) during the treatment period. |
| 1912 | Smits, P, Temme, L, Thien, T. The cardiovascular interaction between caffeine and nicotine in humans. Clinical pharmacology and therapeutics. 1993;54:194. | RCTs that did not provide data on the outcome of interest (i.e., arrhythmia, nonfatal myocardial infarction, nonfatal stroke, cardiovascular death) during the treatment period. |
| 1913 | Turner, J A, McNicol, M W. The effect of nicotine and carbon monoxide on exercise performance in normal subjects. Respiratory medicine. 1993;87:427. | RCTs that did not provide data on the outcome of interest (i.e., arrhythmia, nonfatal myocardial infarction, nonfatal stroke, cardiovascular death) during the treatment period. |
| 1914 | . Effectiveness of a nicotine patch in helping people stop smoking: results of a randomised trial in general practice. Imperial Cancer Research Fund General Practice Research Group. BMJ (Clinical research ed.). 1993;306:1304. | RCTs that did not provide data on the outcome of interest (i.e., arrhythmia, nonfatal myocardial infarction, nonfatal stroke, cardiovascular death) during the treatment period. |
| 1915 | Russell, M A, Stapleton, J A, Feyerabend, C, Wiseman, S M, Gustavsson, G, Sawe, U, Connor, P. Targeting heavy smokers in general practice: randomised controlled trial of transdermal nicotine patches. BMJ (Clinical research ed.). 1993;306:1308. | RCTs that did not provide data on the outcome of interest (i.e., arrhythmia, nonfatal myocardial infarction, nonfatal stroke, cardiovascular death) during the treatment period. |
| 1917 | Tønnesen, P, Nørregaard, J, Mikkelsen, K, Jørgensen, S, Nilsson, F. A double-blind trial of a nicotine inhaler for smoking cessation. JAMA. 1993;269:1268. | RCTs that did not provide data on the outcome of interest (i.e., arrhythmia, nonfatal myocardial infarction, nonfatal stroke, cardiovascular death) during the treatment period. |
| 1918 | Hill, R.D., Rigdon, M., Johnson, S.. Behavioral smoking cessation treatment for older chronic smokers. Behavior Therapy. 1993;24:321. | RCTs that did not provide data on the outcome of interest (i.e., arrhythmia, nonfatal myocardial infarction, nonfatal stroke, cardiovascular death) during the treatment period. |
| 1919 | Burke, P., Chivers, A., Clements, J., Dawes, M., Eastwood, I., Ebbs, D., Godlee, R., Harrington, R., Kearley, K., MacLennan, N., Mant, D., Murray, J., Nichols, M., Van Oss, H., Stern, D., Stevens, R., Thurston, D., Wilson, R., Wood, S.. Effectiveness of a nicotine patch in helping people stop smoking: Results of a randomised trial in general practice. British Medical Journal. 1993;306:1304. | RCTs that did not provide data on the outcome of interest (i.e., arrhythmia, nonfatal myocardial infarction, nonfatal stroke, cardiovascular death) during the treatment period. |
| 1923 | Pirie, P L, McBride, C M, Hellerstedt, W, Jeffery, R W, Hatsukami, D, Allen, S, Lando, H. Smoking cessation in women concerned about weight. American journal of public health. 1992;82:1238. | RCTs that did not provide data on the outcome of interest (i.e., arrhythmia, nonfatal myocardial infarction, nonfatal stroke, cardiovascular death) during the treatment period. |
| 1925 | Sutherland, G, Stapleton, J A, Russell, M A, Jarvis, M J, Hajek, P, Belcher, M, Feyerabend, C. Randomised controlled trial of nasal nicotine spray in smoking cessation. Lancet (London, England). 1992;340:324. | RCTs that did not provide data on the outcome of interest (i.e., arrhythmia, nonfatal myocardial infarction, nonfatal stroke, cardiovascular death) during the treatment period. |
| 1930 | Hatsukami, D, Anton, D, Keenan, R, Callies, A. Smokeless tobacco abstinence effects and nicotine gum dose. Psychopharmacology. 1992;106:60. | RCTs that did not provide data on the outcome of interest (i.e., arrhythmia, nonfatal myocardial infarction, nonfatal stroke, cardiovascular death) during the treatment period. |
| 1931 | Foulds, J, Stapleton, J, Feyerabend, C, Vesey, C, Jarvis, M, Russell, M A. Effect of transdermal nicotine patches on cigarette smoking: a double blind crossover study. Psychopharmacology. 1992;106:421. | RCTs that did not provide data on the outcome of interest (i.e., arrhythmia, nonfatal myocardial infarction, nonfatal stroke, cardiovascular death) during the treatment period. |
| 1932 | Clavel-Chapelon, F, Paoletti, C, Benhamou, S. A randomised 2 x 2 factorial design to evaluate different smoking cessation methods. Revue d'epidemiologie et de sante publique. 1992;40:187. | RCTs that did not provide data on the outcome of interest (i.e., arrhythmia, nonfatal myocardial infarction, nonfatal stroke, cardiovascular death) during the treatment period. |
| 1934 | Hughes, J R, Gust, S W, Keenan, R, Fenwick, J W, Skoog, K, Higgins, S T. Long-term use of nicotine vs placebo gum. Archives of internal medicine. 1991;151:1993. | RCTs that did not provide data on the outcome of interest (i.e., arrhythmia, nonfatal myocardial infarction, nonfatal stroke, cardiovascular death) during the treatment period. |
| 1935 | Tønnesen, P, Nørregaard, J, Simonsen, K, Säwe, U. A double-blind trial of a 16-hour transdermal nicotine patch in smoking cessation. The New England journal of medicine. 1991;325:311. | RCTs that did not provide data on the outcome of interest (i.e., arrhythmia, nonfatal myocardial infarction, nonfatal stroke, cardiovascular death) during the treatment period. |
| 1936 | Segnan, N, Ponti, A, Battista, R N, Senore, C, Rosso, S, Shapiro, S H, Aimar, D. A randomized trial of smoking cessation interventions in general practice in Italy. Cancer causes & control : CCC. 1991;2:239. | RCTs that did not provide data on the outcome of interest (i.e., arrhythmia, nonfatal myocardial infarction, nonfatal stroke, cardiovascular death) during the treatment period. |
| 1938 | Daughton, D M, Heatley, S A, Prendergast, J J, Causey, D, Knowles, M, Rolf, C N, Cheney, R A, Hatlelid, K, Thompson, A B, Rennard, S I. Effect of transdermal nicotine delivery as an adjunct to low-intervention smoking cessation therapy. A randomized, placebo-controlled, double-blind study. Archives of internal medicine. 1991;151:749. | RCTs that did not provide data on the outcome of interest (i.e., arrhythmia, nonfatal myocardial infarction, nonfatal stroke, cardiovascular death) during the treatment period. |
| 1940 | Hughes, J R, Gust, S W, Skoog, K, Keenan, R M, Fenwick, J W. Symptoms of tobacco withdrawal. A replication and extension. Archives of general psychiatry. 1991;48:52. | RCTs that did not provide data on the outcome of interest (i.e., arrhythmia, nonfatal myocardial infarction, nonfatal stroke, cardiovascular death) during the treatment period. |
| 1941 | Ockene, J K, Kristeller, J, Goldberg, R, Amick, T L, Pekow, P S, Hosmer, D, Quirk, M, Kalan, K. Increasing the efficacy of physician-delivered smoking interventions: a randomized clinical trial. Journal of general internal medicine. 1991;6:1. | RCTs that did not provide data on the outcome of interest (i.e., arrhythmia, nonfatal myocardial infarction, nonfatal stroke, cardiovascular death) during the treatment period. |
| 1942 | Hatsukami, D, McBride, C, Pirie, P, Hellerstedt, W, Lando, H. Effects of nicotine gum on prevalence and severity of withdrawal in female cigarette smokers. Journal of substance abuse. 1991;3:427. | RCTs that did not provide data on the outcome of interest (i.e., arrhythmia, nonfatal myocardial infarction, nonfatal stroke, cardiovascular death) during the treatment period. |
| 1943 | Jensen, E J, Schmidt, E, Pedersen, B, Dahl, R. Effect on smoking cessation of silver acetate, nicotine and ordinary chewing gum. Influence of smoking history. Psychopharmacology. 1991;104:470. | RCTs that did not provide data on the outcome of interest (i.e., arrhythmia, nonfatal myocardial infarction, nonfatal stroke, cardiovascular death) during the treatment period. |
| 1944 | Hatsukami, D K, Skoog, K, Huber, M, Hughes, J. Signs and symptoms from nicotine gum abstinence. Psychopharmacology. 1991;104:496. | RCTs that did not provide data on the outcome of interest (i.e., arrhythmia, nonfatal myocardial infarction, nonfatal stroke, cardiovascular death) during the treatment period. |
| 1945 | Hurt, R D, Lauger, G G, Offord, K P, Kottke, T E, Dale, L C. Nicotine-replacement therapy with use of a transdermal nicotine patch--a randomized double-blind placebo-controlled trial. Mayo Clinic proceedings. 1990;65:1529. | RCTs that did not provide data on the outcome of interest (i.e., arrhythmia, nonfatal myocardial infarction, nonfatal stroke, cardiovascular death) during the treatment period. |
| 1947 | Lashner, B A, Hanauer, S B, Silverstein, M D. Testing nicotine gum for ulcerative colitis patients. Experience with single-patient trials. Digestive diseases and sciences. 1990;35:827. | RCTs that did not provide data on the outcome of interest (i.e., arrhythmia, nonfatal myocardial infarction, nonfatal stroke, cardiovascular death) during the treatment period. |
| 1948 | Rose, J E, Levin, E D, Behm, F M, Adivi, C, Schur, C. Transdermal nicotine facilitates smoking cessation. Clinical pharmacology and therapeutics. 1990;47:323. | RCTs that did not provide data on the outcome of interest (i.e., arrhythmia, nonfatal myocardial infarction, nonfatal stroke, cardiovascular death) during the treatment period. |
| 1949 | Hughes, J R, Gust, S W, Keenan, R M, Fenwick, J W. Effect of dose on nicotine's reinforcing, withdrawal-suppression and self-reported effects. The Journal of pharmacology and experimental therapeutics. 1990;252:1175. | RCTs that did not provide data on the outcome of interest (i.e., arrhythmia, nonfatal myocardial infarction, nonfatal stroke, cardiovascular death) during the treatment period. |
| 1950 | Killen, J D, Fortmann, S P, Newman, B, Varady, A. Evaluation of a treatment approach combining nicotine gum with self-guided behavioral treatments for smoking relapse prevention. Journal of consulting and clinical psychology. 1990;58:85. | RCTs that did not provide data on the outcome of interest (i.e., arrhythmia, nonfatal myocardial infarction, nonfatal stroke, cardiovascular death) during the treatment period. |
| 1951 | Smits, P, Eijsbouts, A, Thien, T. Nicotine enhances the circulatory effects of adenosine in human beings. Clinical pharmacology and therapeutics. 1989;46:272. | RCTs that did not provide data on the outcome of interest (i.e., arrhythmia, nonfatal myocardial infarction, nonfatal stroke, cardiovascular death) during the treatment period. |
| 1953 | Hughes, J R, Gust, S W, Keenan, R M, Fenwick, J W, Healey, M L. Nicotine vs placebo gum in general medical practice. JAMA. 1989;261:1300. | RCTs that did not provide data on the outcome of interest (i.e., arrhythmia, nonfatal myocardial infarction, nonfatal stroke, cardiovascular death) during the treatment period. |
| 1954 | Gross, J, Stitzer, M L, Maldonado, J. Nicotine replacement: effects of postcessation weight gain. Journal of consulting and clinical psychology. 1989;57:87. | RCTs that did not provide data on the outcome of interest (i.e., arrhythmia, nonfatal myocardial infarction, nonfatal stroke, cardiovascular death) during the treatment period. |
| 1955 | Abelin, T, Buehler, A, Müller, P, Vesanen, K, Imhof, P R. Controlled trial of transdermal nicotine patch in tobacco withdrawal. Lancet (London, England). 1989;1:7. | RCTs that did not provide data on the outcome of interest (i.e., arrhythmia, nonfatal myocardial infarction, nonfatal stroke, cardiovascular death) during the treatment period. |
| 1956 | Buchkremer, G, Bents, H, Horstmann, M, Opitz, K, Tölle, R. Combination of behavioral smoking cessation with transdermal nicotine substitution. Addictive behaviors. 1989;14:229. | RCTs that did not provide data on the outcome of interest (i.e., arrhythmia, nonfatal myocardial infarction, nonfatal stroke, cardiovascular death) during the treatment period. |
| 1959 | Gilbert, J R, Wilson, D M, Best, J A, Taylor, D W, Lindsay, E A, Singer, J, Willms, D G. Smoking cessation in primary care. A randomized controlled trial of nicotine-bearing chewing gum. The Journal of family practice. 1989;28:49. | RCTs that did not provide data on the outcome of interest (i.e., arrhythmia, nonfatal myocardial infarction, nonfatal stroke, cardiovascular death) during the treatment period. |
| 1961 | Hughes, J R, Gulliver, S B, Amori, G, Mireault, G C, Fenwick, J F. Effect of instructions and nicotine on smoking cessation, withdrawal symptoms and self-administration of nicotine gum. Psychopharmacology. 1989;99:486. | RCTs that did not provide data on the outcome of interest (i.e., arrhythmia, nonfatal myocardial infarction, nonfatal stroke, cardiovascular death) during the treatment period. |
| 1963 | Wilson, D M, Taylor, D W, Gilbert, J R, Best, J A, Lindsay, E A, Willms, D G, Singer, J. A randomized trial of a family physician intervention for smoking cessation. JAMA. 1988;260:1570. | RCTs that did not provide data on the outcome of interest (i.e., arrhythmia, nonfatal myocardial infarction, nonfatal stroke, cardiovascular death) during the treatment period. |
| 1964 | Fortmann, S P, Killen, J D, Telch, M J, Newman, B. Minimal contact treatment for smoking cessation. A placebo controlled trial of nicotine polacrilex and self-directed relapse prevention: initial results of the Stanford Stop Smoking Project. JAMA. 1988;260:1575. | RCTs that did not provide data on the outcome of interest (i.e., arrhythmia, nonfatal myocardial infarction, nonfatal stroke, cardiovascular death) during the treatment period. |
| 1966 | Pickworth, W B, Herning, R I, Henningfield, J E. Mecamylamine reduces some EEG effects of nicotine chewing gum in humans. Pharmacology, biochemistry, and behavior. 1988;30:149. | RCTs that did not provide data on the outcome of interest (i.e., arrhythmia, nonfatal myocardial infarction, nonfatal stroke, cardiovascular death) during the treatment period. |
| 1967 | Tønnesen, P, Fryd, V, Hansen, M, Helsted, J, Gunnersen, A B, Forchammer, H, Stockner, M. Effect of nicotine chewing gum in combination with group counseling on the cessation of smoking. The New England journal of medicine. 1988;318:15. | RCTs that did not provide data on the outcome of interest (i.e., arrhythmia, nonfatal myocardial infarction, nonfatal stroke, cardiovascular death) during the treatment period. |
| 1968 | Tønnesen, P, Fryd, V, Hansen, M, Helsted, J, Gunnersen, A B, Forchammer, H, Stockner, M. Two and four mg nicotine chewing gum and group counselling in smoking cessation: an open, randomized, controlled trial with a 22 month follow-up. Addictive behaviors. 1988;13:17. | RCTs that did not provide data on the outcome of interest (i.e., arrhythmia, nonfatal myocardial infarction, nonfatal stroke, cardiovascular death) during the treatment period. |
| 1970 | Harackiewicz, J M, Blair, L W, Sansone, C, Epstein, J A, Stuchell, R N. Nicotine gum and self-help manuals in smoking cessation: an evaluation in a medical context. Addictive behaviors. 1988;13:319. | RCTs that did not provide data on the outcome of interest (i.e., arrhythmia, nonfatal myocardial infarction, nonfatal stroke, cardiovascular death) during the treatment period. |
| 1971 | Areechon, W, Punnotok, J. Smoking cessation through the use of nicotine chewing gum: a double-blind trial in Thailand. Clinical therapeutics. 1988;10:183. | RCTs that did not provide data on the outcome of interest (i.e., arrhythmia, nonfatal myocardial infarction, nonfatal stroke, cardiovascular death) during the treatment period. |
| 1972 | Michel Ch., Hasenfratz, M., Nil, R., Battig, K.. Cardiovascular, electrocortical, and behavioral effects of nicotine chewing gum. Klinische Wochenschrift. 1988;66:72. | RCTs that did not provide data on the outcome of interest (i.e., arrhythmia, nonfatal myocardial infarction, nonfatal stroke, cardiovascular death) during the treatment period. |
| 1974 | Clavel, F, Benhamou, S, Flamant, R. Nicotine dependence and secondary effects of smoking cessation. Journal of behavioral medicine. 1987;10:555. | RCTs that did not provide data on the outcome of interest (i.e., arrhythmia, nonfatal myocardial infarction, nonfatal stroke, cardiovascular death) during the treatment period. |
| 1975 | Sutton, S, Hallett, R. Randomized trial of brief individual treatment for smoking using nicotine chewing gum in a workplace setting. American journal of public health. 1987;77:1210. | RCTs that did not provide data on the outcome of interest (i.e., arrhythmia, nonfatal myocardial infarction, nonfatal stroke, cardiovascular death) during the treatment period. |
| 1979 | Lindblad, A, Marsál, K. Influence of nicotine chewing gum on fetal blood flow. Journal of perinatal medicine. 1987;15:13. | RCTs that did not provide data on the outcome of interest (i.e., arrhythmia, nonfatal myocardial infarction, nonfatal stroke, cardiovascular death) during the treatment period. |
| 1982 | Hughes, J R, Hatsukami, D K, Skoog, K P. Physical dependence on nicotine in gum. A placebo substitution trial. JAMA. 1986;255:3277. | RCTs that did not provide data on the outcome of interest (i.e., arrhythmia, nonfatal myocardial infarction, nonfatal stroke, cardiovascular death) during the treatment period. |
| 1983 | Nemeth-Coslett, R, Henningfield, J E. Effects of nicotine chewing gum on cigarette smoking and subjective and physiologic effects. Clinical pharmacology and therapeutics. 1986;39:625. | RCTs that did not provide data on the outcome of interest (i.e., arrhythmia, nonfatal myocardial infarction, nonfatal stroke, cardiovascular death) during the treatment period. |
| 1984 | Russell, P O, Epstein, L H, Sittenfield, S L, Block, D R. The effects of nicotine chewing gum on the sensitivity to muscle tension. Psychopharmacology. 1986;89:230. | RCTs that did not provide data on the outcome of interest (i.e., arrhythmia, nonfatal myocardial infarction, nonfatal stroke, cardiovascular death) during the treatment period. |
| 1985 | Clavel, F, Benhamou, S, Company-Huertas, A, Flamant, R. Helping people to stop smoking: randomised comparison of groups being treated with acupuncture and nicotine gum with control group. British medical journal (Clinical research ed.). 1985;291:1538. | Letters to the editors, opinions, editorials, press releases, manufacturers’ advertisements, and other non-peer reviewed publications, unless the publication contained original data from RCTs. |
| 1986 | Christen, A G, Beiswanger, B B, Mallatt, M E, Tomich, C E, Drook, C A, McDonald, J L, Jr, Olson, B L, Stookey, G K. Effects of nicotine-containing chewing gum on oral soft and hard tissues: A clinical study. Oral surgery, oral medicine, and oral pathology. 1985;59:37. | RCTs that did not provide data on the outcome of interest (i.e., arrhythmia, nonfatal myocardial infarction, nonfatal stroke, cardiovascular death) during the treatment period. |
| 1987 | Hjalmarson, A I. Effect of nicotine chewing gum in smoking cessation. A randomized, placebo-controlled, double-blind study. JAMA. 1984;252:2835. | RCTs that did not provide data on the outcome of interest (i.e., arrhythmia, nonfatal myocardial infarction, nonfatal stroke, cardiovascular death) during the treatment period. |
| 1989 | Jamrozik, K, Fowler, G, Vessey, M, Wald, N. Placebo controlled trial of nicotine chewing gum in general practice. British medical journal (Clinical research ed.). 1984;289:794. | RCTs that did not provide data on the outcome of interest (i.e., arrhythmia, nonfatal myocardial infarction, nonfatal stroke, cardiovascular death) during the treatment period. |
| 1990 | Fagerström, K O. Effects of nicotine chewing gum and follow-up appointments in physician-based smoking cessation. Preventive medicine. 1984;13:517. | RCTs that did not provide data on the outcome of interest (i.e., arrhythmia, nonfatal myocardial infarction, nonfatal stroke, cardiovascular death) during the treatment period. |
| 1991 | Killen, J.D., Maccoby, N., Taylor, C.B.. Nicotine gum and self-regulation training in smoking relapse prevention. Behavior Therapy. 1984;15:234. | RCTs that did not provide data on the outcome of interest (i.e., arrhythmia, nonfatal myocardial infarction, nonfatal stroke, cardiovascular death) during the treatment period. |
| 1993 | Hughes, J R, Hatsukami, D K, Pickens, R W, Krahn, D, Malin, S, Luknic, A. Effect of nicotine on the tobacco withdrawal syndrome. Psychopharmacology. 1984;83:82. | RCTs that did not provide data on the outcome of interest (i.e., arrhythmia, nonfatal myocardial infarction, nonfatal stroke, cardiovascular death) during the treatment period. |
| 1994 | Fagerström, K O. A comparison of psychological and pharmacological treatment in smoking cessation. Journal of behavioral medicine. 1982;5:343. | RCTs that did not provide data on the outcome of interest (i.e., arrhythmia, nonfatal myocardial infarction, nonfatal stroke, cardiovascular death) during the treatment period. |
| 1996 | Schnoll, Robert A., Wileyto, E. Paul, Lerman, Caryn. Extended duration therapy with transdermal nicotine may attenuate weight gain following smoking cessation. Addictive Behaviors. 2012; 37(4):565. | RCTs that did not provide data on the outcome of interest (i.e., arrhythmia, nonfatal myocardial infarction, nonfatal stroke, cardiovascular death) during the treatment period. |

## D. Included Full Text Articles (n=42)

| **Refid** | **Bibliography** |
| --- | --- |
| 64 | Chen LS, Baker TB, Miller JP, et al. Genetic Variant in CHRNA5 and Response to Varenicline and Combination Nicotine Replacement in a Randomized Placebo-Controlled Trial. Clin Pharmacol Ther. 2020;108(6):1315-1325. |
| 133 | Gilbert DG, Rabinovich NE, McDaniel JT. Nicotine patch for cannabis withdrawal symptom relief: a randomized controlled trial. Psychopharmacology (Berl). 2020;237(5):1507-1519. |
| 167 | Xiao D, Kotler M, Kang J, Wang C. A Multicenter, Randomized, Double-blind, Parallel, Placebo-controlled Clinical Study to Evaluate the Efficacy and Safety of a Nicotine Mint Lozenge (2 and 4_mg) in Smoking Cessation. J Addict Med. 2020;14(1):69-77. |
| 285 | de Jong B, Schuppers AS, Kruisdijk-Gerritsen A, Arbouw MEL, van den Oever HLA, van Zanten ARH. The safety and efficacy of nicotine replacement therapy in the intensive care unit: a randomised controlled pilot study. Ann Intensive Care. 2018;8(1):70. |
| 326 | Effects on abstinence of nicotine patch treatment before quitting smoking: parallel, two arm, pragmatic randomised trial. BMJ. 2018;361:k2164. |
| 338 | Benowitz NL, Pipe A, West R, et al. Cardiovascular Safety of Varenicline, Bupropion, and Nicotine Patch in Smokers: A Randomized Clinical Trial. JAMA Intern Med. 2018;178(5):622-631. |
| 391 | Rohsenow DJ, Tidey JW, Martin RA, et al. Varenicline versus nicotine patch with brief advice for smokers with substance use disorders with or without depression: effects on smoking, substance use and depressive symptoms. Addiction. 2017;112(10):1808-1820. |
| 402 | Lambrichts DP V, Boersema GSA, Tas B, et al. Nicotine chewing gum for the prevention of postoperative ileus after colorectal surgery-A multicentre, double-blind, randomised, controlled pilot study. Eur Surg Res. 2017;58:35. |
| 521 | Tuisku A, Salmela M, Nieminen P, Toljamo T. Varenicline and Nicotine Patch Therapies in Young Adults Motivated to Quit Smoking: A Randomized, Placebo-controlled, Prospective Study. Basic Clin Pharmacol Toxicol. 2016;119(1):78-84. |
| 677 | Lerman C, Schnoll RA, Hawk Jr LW, et al. Use of the nicotine metabolite ratio as a genetically informed biomarker of response to nicotine patch or varenicline for smoking cessation: a randomised, double-blind placebo-controlled trial. Lancet Respir Med. 2015;3(2):131-138. |
| 707 | Ramon JM, Morchon S, Baena A, Masuet-Aumatell C. Combining varenicline and nicotine patches: a randomized controlled trial study in smoking cessation. BMC Med. 2014;12:172. |
| 776 | Oncken C, Arias AJ, Feinn R, et al. Topiramate for smoking cessation: a randomized, placebo-controlled pilot study. Nicotine Tob Res. 2014;16(3):288-296. |
| 810 | Stein MD, Caviness CM, Kurth ME, Audet D, Olson J, Anderson BJ. Varenicline for smoking cessation among methadone-maintained smokers: a randomized clinical trial. Drug Alcohol Depend. 2013;133(2):486-493. |
| 816 | Ebbert JO, Croghan IT, Schroeder DR, Hurt RD. A randomized phase II clinical trial of high-dose nicotine patch therapy for smokeless tobacco users. Nicotine Tob Res. 2013;15(12):2037-2044. |
| 910 | Rungruanghiranya S, Ekpanyaskul C, Sakulisariyaporn C, Watcharanat P, Akkalakulawas K. Efficacy of fresh lime for smoking cessation. J Med Assoc Thai. 2012;95 Suppl 1:S76-82. |
| 1065 | Stapleton JA, Sutherland G. Treating heavy smokers in primary care with the nicotine nasal spray: randomized placebo-controlled trial. Addiction. 2011;106(4):824-832. |
| 1074 | Koychev I, McMullen K, Lees J, et al. A validation of cognitive biomarkers for the early identification of cognitive enhancing agents in schizotypy. Eur Neuropsychopharmacol. 2011;21:S86-S87. |
| 1104 | Thomsen T, Tønnesen H, Okholm M, et al. Brief smoking cessation intervention in relation to breast cancer surgery: a randomized controlled trial. Nicotine Tob Res. 2010;12(11):1118-1124. |
| 1194 | Kralikova E, Kozak JT, Rasmussen T, Gustavsson G, Le Houezec J. Smoking cessation or reduction with nicotine replacement therapy: a placebo-controlled double blind trial with nicotine gum and inhaler. BMC Public Health. 2009;9:433. |
| 1247 | Shiffman S, Ferguson SG, Strahs KR. Quitting by gradual smoking reduction using nicotine gum: a randomized controlled trial. Am J Prev Med. 2009;36(2):96-104.e1. |
| 1253 | Sun HQ, Guo S, Chen DF, et al. Family support and employment as predictors of smoking cessation success: a randomized, double-blind, placebo-controlled trial of nicotine sublingual tablets in chinese smokers. Am J Drug Alcohol Abuse. 2009;35(3):183-188. |
| 1272 | Rungruanghiranya S, Ekpanyaskul C, Hattapornsawan Y, Tundulawessa Y. Effect of nicotine polyestex gum on smoking cessation and quality of life. J Med Assoc Thai. 2008;91(11):1656-1662. |
| 1279 | Shiffman S, Ferguson SG. The effect of a nicotine patch on cigarette craving over the course of the day: results from two randomized clinical trials. Curr Med Res Opin. 2008;24(10):2795-2804. |
| 1295 | Aubin HJ, Bobak A, Britton JR, et al. Varenicline versus transdermal nicotine patch for smoking cessation: results from a randomised open-label trial. Thorax. 2008;63(8):717-724. |
| 1339 | Myung SK, Seo HG, Park S, et al. Sociodemographic and smoking behavioral predictors associated with smoking cessation according to follow-up periods: a randomized, double-blind, placebo-controlled trial of transdermal nicotine patches. J Korean Med Sci. 2007;22(6):1065-1070. |
| 1356 | Covey LS, Glassman AH, Jiang H, et al. A randomized trial of bupropion and/or nicotine gum as maintenance treatment for preventing smoking relapse. Addiction. 2007;102(8):1292-1302. |
| 1368 | Uyar M, Filiz A, Bayram N, et al. A randomized trial of smoking cessation. Medication versus motivation. Saudi Med J. 2007;28(6):922-926. |
| 1393 | Ebbert JO, Dale LC, Patten CA, et al. Effect of high-dose nicotine patch therapy on tobacco withdrawal symptoms among smokeless tobacco users. Nicotine Tob Res. 2007;9(1):43-52. |
| 1447 | Shiffman S, Scharf DM, Shadel WG, et al. Analyzing milestones in smoking cessation: illustration in a nicotine patch trial in adult smokers. J Consult Clin Psychol. 2006;74(2):276-285. |
| 1612 | Hughes JR, Novy P, Hatsukami DK, Jensen J, Callas PW. Efficacy of nicotine patch in smokers with a history of alcoholism. Alcohol Clin Exp Res. 2003;27(6):946-954. |
| 1660 | Etter JF, Laszlo E, Zellweger JP, Perrot C, Perneger TV. Nicotine replacement to reduce cigarette consumption in smokers who are unwilling to quit: a randomized trial. J Clin Psychopharmacol. 2002;22(5):487-495. |
| 1678 | Møller AM, Villebro N, Pedersen T, Tønnesen H. Effect of preoperative smoking intervention on postoperative complications: a randomised clinical trial. Lancet (London, England). 2002;359(9301):114-117. |
| 1731 | Wallström M, Nilsson F, Hirsch JM. A randomized, double-blind, placebo-controlled clinical evaluation of a nicotine sublingual tablet in smoking cessation. Addiction. 2000;95(8):1161-1171. |
| 1755 | Hays JT, Croghan IT, Schroeder DR, et al. Over-the-counter nicotine patch therapy for smoking cessation: results from randomized, double-blind, placebo-controlled, and open label trials. Am J Public Health. 1999;89(11):1701-1707. |
| 1779 | Tønnesen P, Paoletti P, Gustavsson G, et al. Higher dosage nicotine patches increase one-year smoking cessation rates: results from the European CEASE trial. Collaborative European Anti-Smoking Evaluation. European Respiratory Society. Eur Respir J. 1999;13(2):238-246. |
| 1803 | Lewis SF, Piasecki TM, Fiore MC, Anderson JE, Baker TB. Transdermal nicotine replacement for hospitalized patients: a randomized clinical trial. Prev Med (Baltim). 1998;27(2):296-303. |
| 1820 | Sandborn WJ, Tremaine WJ, Offord KP, et al. Transdermal nicotine for mildly to moderately active ulcerative colitis. A randomized, double-blind, placebo-controlled trial. Ann Intern Med. 1997;126(5):364-371. |
| 1835 | Joseph AM, Norman SM, Ferry LH, et al. The safety of transdermal nicotine as an aid to smoking cessation in patients with cardiac disease. N Engl J Med. 1996;335(24):1792-1798. |
| 1860 | Kavin H, Shivley S. Absence of effect of nicotine on rectal sensation, rectal compliance, and anal sphincter pressures in healthy subjects. Dig Dis Sci. 1995;40(10):2239-2243. |
| 1869 | Thomas GA, Rhodes J, Mani V, et al. Transdermal nicotine as maintenance therapy for ulcerative colitis. N Engl J Med. 1995;332(15):988-992. |
| 1910 | Sachs DP, Säwe U, Leischow SJ. Effectiveness of a 16-hour transdermal nicotine patch in a medical practice setting, without intensive group counseling. Arch Intern Med. 1993;153(16):1881-1890. |
| 1916 | Foulds J, Stapleton J, Hayward M, et al. Transdermal nicotine patches with low-intensity support to aid smoking cessation in outpatients in a general hospital. A placebo-controlled trial. Arch Fam Med. 1993;2(4):417-423. |

## E. Characteristics of Included Studies and Outcome Measures

| RefID | Trial Details | Subject Details | Outcomes |
| --- | --- | --- | --- |
| 1295 | Aubin et al., 2008  Location: Multinational (Belgium; France; Netherlands; UK; US)  Setting: Clinical  Trial Name: An Open Label Study That Compares Varenicline to Transdermal Nicotine Patch for Smoking Cessation  Registration/Protocol: NCT00143325  Funding: Pfizer Inc.  Design  Open-label RCT  Eligibility  Inclusion: Age 18-75 yrs; >45.5 kg and BMI 15-38 kg/m^2^; smoking ≥15 cpd with no period of abstinence >3 months in the past yr.  Exclusion: History of cancer; any other serious or unstable disease within the previous 6 months; diagnoses of or treatment for depression or other psychological disorder; drug or alcohol dependence within the previous 12 months; clinically significant allergic reactions to drugs or adhesive tapes; skin disorders; systolic blood pressure >150 mmHg or diastolic blood pressure >95 mmHg; clinically significant renal or hepatic impairment; evidence of liver dysfunction or other abnormal laboratory tests; taking medication that may interfere with the study outcome; previous participation in a varenicline study in the previous year; use of any form of NRT in the previous 6 months; breastfeeding, pregnancy, or at risk of becoming pregnant.  Recruitment  Recruited in smoking cessation clinics or via local advertising.  Intervention  Nicotine patch group: Subjects received nicotine patch (NicoDerm^®^ CQ^®^ Clear (GlaxoSmithKline) in the US and NiQuitin^®^ CQ^®^ Clear (GlaxoSmithKline) in Europe) each morning starting on the target quit date for 10 weeks. Doses of nicotine patch were 21 mg/day for the first 6 weeks, 14 mg/day for 2 weeks, then 7 mg/day for 2 weeks. Subjects also received counselling and an educational booklet on smoking cessation.  Control  Varenicline group: Subjects began receiving varenicline (0.5 mg per day for 3 days, 0.5 mg twice daily for 4 days, then 1 mg twice daily thereafter) 1 week before the target quit date. Full dosing was achieved by the target quit date and continued for a further 11 weeks. Subjects also received counselling and an educational booklet on smoking cessation.  Study Start/End Date  January 2005 to June 2006 | Study Population  Adult cigarette smokers (motivated to quit)  Sample Size  Total: n=746 subjects  Nicotine patch group: n=370 subjects  Varenicline group: n=376 subjects  Subject Characteristics  *Mean age±SD*  Nicotine patch group: 42.9±12.0 yrs  Varenicline group: 42.9±10.5 yrs  *Sex*  Nicotine patch group: male, 50.0%; female, 50%  Varenicline group: male, 48.4%; female, 51.6%  *Race/ethnicity*  Nicotine patch group: Caucasian, 93.5%; Black, 1.6%; Asian, 2.4%; Other, 2.4%  Varenicline group: Caucasian, 92.6%; Black, 2.9%; Asian, 2.4%; Other, 2.4%  *Mean yrs smoked (range)*  Nicotine patch group: 25.2 (1-62)  Varenicline group: 25.9 (2-58)  *Mean past-month cpd (range)*  Nicotine patch group: 22.4 (11-60)  Varenicline group: 23.0 (15-80)  Abstinence Characteristics  *eCO-validated (≤10 ppm) continuous abstinence for the last 4 weeks of treatment (%)*  Nicotine patch group: 43.2% (n=160)  Varenicline group: 55.9% (n=210) | Arrhythmia  NR  Nonfatal Myocardial Infarction  Nicotine patch group: n=2  Varenicline group: n=0  Nonfatal Stroke*  Nicotine patch group: n=0  Varenicline group: n=0  *Reported serious AEs were listed, none of which were nonfatal stroke.  Cardiovascular Death  Nicotine patch group: n=0  Varenicline group: n=0 |
| 338 | Benowitz et al., 2018  Location: Multinational (Argentina; Australia; Brazil; Bulgaria; Canada; Chile; Denmark; Finland; Germany; Mexico; New Zealand; Russian Federation; Slovakia; South Africa; Spain; US)  Setting: Clinical (study sites included clinical trial centers, academic centers, and outpatient clinics)  Trial Name: Evaluating Adverse Events in a Global Smoking Cessation Study (EAGLES)  Registration/Protocol: NCT01574703  Funding: “Supported by Elan Pharmaceutical Research Corp, Gainesville, GA”  Design  Randomized, double-blind, triple-dummy, placebo- and active-controlled trial.  Eligibility  Inclusion: Age 18 to 75 yrs; smoking ≥10 cpd; interested in quitting smoking; completed the Week 24 visit of EAGLES (subjects were eligible for inclusion if they stopped study medication prematurely during EAGLES, so long as they had completed all EAGLES study visits).  Exclusion: Unstable psychiatric illness; active substance abuse; clinically significant CVD in the 2 months prior to study entry (e.g., myocardial infarction or coronary artery bypass graft); clinically significant cerebrovascular disease in the 2 months prior to study entry (e.g., stroke or documented transient ischemic attack); inadequate control of hypertension, as judged by investigators at screening and baseline.  Recruitment  Potential subjects were recruited from the investigators’ own clinics through newspaper, radio, and television advertising, fliers, and posters.  Intervention  Nicotine patch group: Subjects received: a transdermal nicotine patch (21 mg per day for 7 weeks, followed by 14 mg per day for 2 weeks, and 7 mg per day for 2 weeks); placebo bupropion; and placebo varenicline. Subjects were instructed to use all three daily (according to instructions) during the 12-week active treatment phase. Note: Among subjects who received one or more partial doses of the nicotine patch, the mean number of days of medication exposure (assessed by patch/pill count) was 73.7±23.6 days; similar to the other treatment groups.  Control  Varenicline group: Subjects received: varenicline (0.5 mg once a day for 3 days; 0.5 mg twice a day for 4 days; 1 mg twice a day for 11 weeks); placebo nicotine patch; and placebo bupropion. Subjects were instructed to use all three daily (according to instructions) during the 12-week active treatment phase. Note: Among subjects who received one or more partial doses of varenicline, the mean number of days of medication exposure (assessed by patch/pill count) was 74.4±23.1 days; similar to the other treatment groups.  Bupropion group: Subjects received: bupropion (150 mg once a day for 3 days; 150 mg twice a day for the remainder of the treatment period); placebo nicotine patch; and placebo varenicline. Subjects were instructed to use all three daily (according to instructions) during the 12-week active treatment phase. Note: Among subjects who received one or more partial doses of bupropion, the mean number of days of medication exposure (assessed by patch/pill count) was 73.7±23.8 days; similar to the other treatment groups.  Placebo group: Subjects received placebo nicotine patch, placebo varenicline, and placebo bupropion. Subjects were instructed to use all three daily (according to instructions) during the 12-week active treatment phase. Note: Among subjects who received one or more partial exposure (assessed by patch/pill count) was 73.6±23.6 days; similar to the other treatment groups.  Study Start/End Date  November 2011 to January 2015 | Study Population  Adult cigarette smokers with or without psychiatric disease  Sample Size  Total: n=8,058 subjects  Nicotine patch group: n=2,022 subjects  Varenicline group: n=2,016 subjects  Bupropion group: n=2,006 subjects  Placebo group: n=2,014 subjects  Subject Characteristics  *Mean age*±*SD*  Nicotine patch group: 46.9±12.2 yrs  Varenicline group: 46.5±12.4 yrs  Bupropion group: 46.3±12.6 yrs  Placebo group: 46.4±12.1 yrs  *Sex (% male)*  Nicotine patch group: 43.7% (n=883)  Varenicline group: 44.7% (n=902)  Bupropion group: 44.5% (n=892)  Placebo group: 43.5% (n=876)  *Race/ethnicity (% White)*  Nicotine patch group: 81.2% (n=1,641)  Varenicline group: 82.7% (n=1,668)  Bupropion group: 81.6% (n=1,636)  Placebo group: 81.4% (n=1,639)  *Yrs smoked*  NR  *Average cpd*  NR  Abstinence Characteristics  *Continuous abstinence rates from Weeks 9 to 12 (%)*  Nicotine patch group: 23.4%  Varenicline group: 33.5%  Bupropion group: 22.6%  Placebo group: 12.5% | Arrhythmia*  Nicotine patch group: n=3 (0.1%)  Varenicline group: n=2 (0.1%)  Bupropion group: n=1 (<0.1%)  Placebo group: n=0  *Reported as serious cardiac arrhythmia  Nonfatal Myocardial Infarction  Nicotine patch group: n=1 (<0.1%)  Varenicline group: n=1 (<0.1%)  Bupropion group: n=1 (<0.1%)  Placebo group: n=3 (0.1%)  Nonfatal Stroke  Nicotine patch group: n=0  Varenicline group: n=0  Bupropion group: n=0  Placebo group: n=0  Cardiovascular Death  Nicotine patch group: n=0  Varenicline group: n=0  Bupropion group: n=1 (<0.1%)  Placebo group: n=1 (<0.1%)  A White male, aged 52 yrs, allocated to the bupropion group died on Day 77 of the treatment period. The subject’s wife reported that he experienced intense pain in his chest and both arms and died on a public road with no medical assistance. The death certificate listed the cause as “non-traumatic cardio-respiratory arrest.”  A Black female, aged 42 yrs, allocated to the placebo group died on Day 60 of the treatment period. The subject was found dead; an autopsy determined the cause to be bilateral pulmonary thromboemboli, and toxicology showed cocaine abuse. |
| 64 | Chen et al., 2020  Location: US  Setting: Community  Trial Name: Genetically Informed Smoking Cessation (GISC)  Registration/Protocol: NCT02351167  Funding: NIDA, Siteman Cancer Center, NCI Cancer Center, and partially by National Center for Advancing Translation Sciences and the Cancer Prevention Research Institute of Texas. Free supply of study medication (varenicline and placebo) obtained from Pfizer with an investigator-initiated research agreement (IIR).  Design  A three-arm, double-blind, placebo-controlled clinical trial, stratified by rs16969968 genotype in six blocks of subjects (two per treatment arm).  Eligibility  Inclusion: Adult (≥21 yrs) active cigarette smokers (≥5 cpd and eCO ≥8 ppm); seeking treatment for smoking cessation; able to speak English; agree to participate in the follow up assessments up to 12 months.  Exclusion: Pregnancy or breastfeeding; active use or recent use (within the past month) of medication or e-cigarettes for nicotine dependence/smoking cessation, or use of e-cigarettes for >9 days in the prior month; allergy to nicotine patch, lozenge, or varenicline; unwillingness to prevent pregnancy during the medication phase and 1 month afterwards (women only); significant cardiac conditions (myocardial infarction, unstable angina, coronary angioplasty, cardiac bypass) or serious arrhythmia in past 6 months; current heavy alcohol consumption (≥6 drinks/day, 6 days/week); active psychosis or poorly controlled depression within the past 6 months; any prior suicide attempt or suicidal ideation within the past 6 months; and end stage renal disease with hemodialysis.  Recruitment  Prior subjects of the Collaborative Genetic Study of Nicotine Dependence (COGEND) studies who provided consent for data sharing and to be contacted for future studies were recruited. Subjects were also recruited from the community via Facebook, flyers, clinical referrals, and a local news media.  Intervention  NRT group: Subjects received 12 weeks of nicotine patches (21 mg for 8 weeks, 14 mg for 2 weeks, and 7 mg for 2 weeks) to use from the morning of target-quit date, as well as 13 weeks of nicotine lozenges for use as needed (4 mg for those who smoke within 30 min of awaking and 2 mg otherwise), starting from the week prior to the target-quit date. Note: Medication adherence was 65% (95% CI 61‑69) for the NRT group.  Control  Varenicline group: Subjects received varenicline pills for 1 week of titration (days 1–3 at 0.5 mg once daily and days 4–7 at 0.5 mg twice daily) for use before the target quit date, followed by 12 weeks at 1.0 mg twice daily for use after the target quit date. Note: Medication adherence was 66% (95% CI 61–70) for the varenicline group.  Placebo group: Subjects received either a placebo nicotine patches/lozenges or varenicline regimen, as defined for the respective active arms, but administered with placebo pills and/or patches. Note: Medication adherence was 62% (95% CI 58–67) for the placebo group.  Study Start/End Date  May 20, 2015 to August 31, 2019 | Study Population  Adult cigarette smokers  Sample Size  Total: n=822 subjects  NRT group: n=275 subjects  Varenicline group: n=274 subjects  Placebo group: n=273 subjects  Subject Characteristics  *Mean age±SD*  NRT group: 46.3±11.2 yrs  Varenicline group: 46.5±11.2 yrs  Placebo group: 46.6±11.5 yrs  *Sex*  NRT group: female, 52.4% (n=144); male, 47.6% (n=131)  Varenicline group: female, 52.6% (n=144); male, 47.5% (n=130)  Placebo group: female, 59.0% (n=161); male, 41.0% (n=112)  *Race/ethnicity*  NRT group: White, 61.1% (n=168); Black, 35.3% (n=97); Other, 3.6% (n=10)  Varenicline group: White, 61.7% (n=169); Black, 33.2% (n=91); Other, 5.1% (n=14)  Placebo group: White, 65.6% (n=179); Black, 30.0% (n=82); Other, 4.4% (n=12)  *Yrs smoked*  NR  *Mean cpd±SD*  Total: 17.6±7.8  NRT group: 17.0±8.1  Varenicline group: 17.5±7.7  Placebo group: 18.2±7.5  Abstinence Characteristics  *7-day PPA at end of treatment period (%)*  NRT group: 20.0%  Varenicline group: 25.5%  Placebo group: 8.8% | Arrhythmia  NR  Nonfatal Myocardial Infarction  NR  Nonfatal Stroke  NRT group: n=0 (0%)  Varenicline group: n=1 (0.36%)  Placebo group: n=1 (0.37%)  Cardiovascular Death  NRT group: n=1 (0.36%)  Varenicline group: n=0 (0%)  Placebo group: n=1 (0.37%)  The one death in the NRT group was due to cardiorespiratory arrest; the one death in the placebo group was due to congestive heart failure. |
| 1356 | Covey et al., 2007  Location: US  Setting: Columbia University Medical Center  Trial Name: NR  Registration/Protocol: NR  Funding: NIDA  Design  Randomized, double-blind, placebo-controlled trial. During the open label treatment, all subjects received bupropion (150 mg per day for 3 days and increased on day 4 to 150 mg/day twice daily to reach therapeutic level by quit day on day 7) for 8 weeks and nicotine patch (21 mg for 4 weeks, 14 mg for 2 weeks, and 7 mg for 1 week) for 7 weeks before being randomized to a treatment group.  Eligibility  Inclusion: Age ≥21 yrs; smoking ≥10 cpd; a previous quit attempt; continuous abstinence during the last 4 weeks of open-label treatment.  Exclusion: Contraindications to the use of the study medications including history of eating disorder or seizure, severe dermatitis, high blood pressure, unstable medical condition, oral or dental problems, lactation, pregnancy or intention to become pregnant during the study period; current use of smoking cessation or other psychoactive medications; psychiatric conditions including current major depressive disorder, dependence on alcohol or drugs within the past yr, a life-time history of bipolar disorder, or any psychotic illness.  Recruitment  Advertisements on radio, television, newspapers, the internet, and various community outlets.  Intervention  Nicotine gum+bupropion group: Subjects received 2 mg nicotine gum and bupropion for 16 weeks. Subjects also received 4 counselling sessions.  Nicotine gum+placebo pill group: Subjects received 2 mg nicotine gum and placebo pills for 16 weeks. Subjects also received 4 counselling sessions.  Note: 45% of subjects randomized to maintenance treatment used the nicotine or placebo gum.  Control  Placebo gum+bupropion group: Subjects received placebo gum and bupropion pills for 16 weeks. Subjects also received 4 counselling sessions.  Placebo gum+placebo pill group: Subjects received placebo gum and placebo pills for 16 weeks. Subjects also received 4 counselling sessions.  Note: 45% of subjects randomized to maintenance treatment used the nicotine or placebo gum.  Study Start/End Date  February 2001 to October 2005 | Study Population  Adult cigarette smokers  Sample Size  Total: n=289 subjects  Nicotine gum+bupropion group: n=73 subjects  Nicotine gum+placebo pill group: n=72 subjects  Placebo gum+bupropion group: n=73 subjects  Placebo gum+placebo pill group: n=71 subjects  Subject Characteristics  *Mean age±SD*  Nicotine gum+bupropion group: 40.3±9.9 yrs  Nicotine gum+placebo pill group: 43.5±10.8 yrs  Placebo gum+bupropion group: 43.7±10.8 yrs  Placebo gum+placebo pill group: 42.5±10.6 yrs  *Sex (% male)*  Nicotine gum+bupropion group: 57.5%  Nicotine gum+placebo pill group: 54.2%  Placebo gum+bupropion group: 53.4%  Placebo gum+placebo pill group: 53.5%  *Race/ethnicity*  Nicotine gum+bupropion group: Caucasian, 68.5%; African American, 19.2%; Hispanic, 5.5%; Asian, 6.8%  Nicotine gum+placebo pill group: Caucasian, 66.7%; African American, 16.7%; Hispanic, 9.7%; Asian, 6.9%  Placebo gum+bupropion group: Caucasian, 76.7%; African American, 11.0%; Hispanic, 8.2%; Asian, 4.1%  Placebo gum+placebo pill group: Caucasian, 66.2%; African American, 15.5%; Hispanic, 14.1%; Asian, 4.2%  *Yrs smoked*  NR  *Mean past-week cpd±SD*  Nicotine gum+bupropion group: 20.3±7.9  Nicotine gum+placebo pill group: 21.3±8.3  Placebo gum+bupropion group: 20.6±7.9  Placebo gum+placebo pill group: 22.8±10.0  Abstinence Characteristics  NR | Arrhythmia  NR  Nonfatal Myocardial Infarction  Nicotine gum+bupropion group: n=0  Nicotine gum+placebo pill group: n=1  Placebo gum+bupropion group: n=0  Placebo gum+placebo pill group: n=0  The one subject who suffered a myocardial infarction had been randomized to the nicotine gum+placebo pill group but did not use the gum.  Nonfatal Stroke*  Nicotine gum+bupropion group: n=0  Nicotine gum+placebo pill group: n=0  Placebo gum+bupropion group: n=0  Placebo gum+placebo pill group: n=0  Cardiovascular Death*  Nicotine gum+bupropion group: n=0  Nicotine gum+placebo pill group: n=0  Placebo gum+bupropion group: n=0  Placebo gum+placebo pill group: n=0  *Reported serious AEs were listed, none of which were nonfatal stroke or cardiovascular death. |
| 285 | de Jong et al., 2018  Location: Netherlands  Setting: Medical-surgical ICU of two university-affiliated teaching hospitals.  Trial Name: Nicotine Replacement Therapy in the Intensive Care Unit (NicGoWell)  Registration/Protocol: NCT01362959  Funding: NR  Design  Randomized, controlled, double-blind, pilot study.  Eligibility  Inclusion: Mechanically ventilated and actively smoking subjects admitted to the medical-surgical ICU.  Exclusion: Age <18 yrs; last smoked >72 hrs before inclusion; smoking ≤10 cpd; >48 hrs after hospital admission admitted to the ICU; expected duration of mechanical ventilation ≤48 hrs; pregnant or breastfeeding; history of dementia or psychosis, neurologic disease on admission such as traumatic brain injury, intracranial hemorrhage, seizures, meningitis, encephalitis, intracranial tumor, cerebrovascular accident; used NRT <2 weeks before ICU admission; acute myocardial infarction; severe cardiac arrhythmia; unstable angina pectoris; generalized skin diseases; severe hearing deficiency; hypersensitivity to nicotine or patches; insufficient Dutch language skills; imminent death or participation in another intervention study.  Recruitment  NR  Intervention  Nicotine patch group: Subjects received nicotine patches (Nicotinell^®^ TTS 20 and 30, Novartis Consumer Health). Patches were applied and replaced every 24 hrs until ICU discharge, or Day 30. Subjects smoking <21 cpd received patches delivering 14 mg nicotine per 24 hrs, and those smoking ≥21 cpd received patches delivering 21 mg nicotine per 24 hrs.  Control  Placebo patch group: Subjects received placebo patches (DuoDerm^®^ Extra Thin, ConvaTec) similar in size and shape to nicotine patches.  Study Start/End Date  July 2012 to June 2016 | Study Population  Adult smokers admitted to the ICU  Sample Size  Total: n=47 subjects  Nicotine patch group: n=21 subjects  Placebo patch group: n=26 subjects  Subject Characteristics  *Mean age±SD*  Nicotine patch group: 60.1±10.55 yrs  Placebo patch group: 65.2±9.13 yrs  *Sex (% male)*  Nicotine patch group: 57% (n=12)  Placebo patch group: 62% (n=16)  *Race/ethnicity*  NR  *Yrs smoked*  NR  *Median cpd (IQR)*  Nicotine patch group: 20 (12.5-27.5)  Placebo patch group: 15 (14.5-25.0)  Abstinence Characteristics  NR | Arrhythmia  Nicotine patch group: n=5  Placebo patch group: n=19  Nonfatal Myocardial Infarction*  Nicotine patch group: n=1  Placebo patch group: n=5  *Reported as cardiac ischemia  Nonfatal Stroke**  Nicotine patch group: n=0  Placebo patch group: n=0  **Reported serious AEs were listed, none of which were nonfatal stroke.  Cardiovascular Death  Nicotine patch group: n=0  Placebo patch group: n=0 |
| 816 | Ebbert et al., 2013  Location: US  Setting: Community  Trial Name: NR  Registration/Protocol: NR  Funding: NIH  Design  RCT.  Eligibility  Inclusion: Age 18-55 yrs; daily smokeless tobacco use for the past 12 months; identified smokeless tobacco as their primary tobacco product; use ≥3 cans/pouches per week; in good health as determined by medical history and screening physical examination; had been provided with, understood, and signed the informed consent.  Exclusion: Currently (past 30 days) using any other behavioral or pharmacologic tobacco dependence treatment program; currently pregnant or lactating; had unstable angina, myocardial infarction, or coronary angioplasty within the past 3 months; history of arrhythmia or an abnormal tracing on the baseline electrocardiogram; blood pressure of >140/90 or a pulse of >110 beats/ per min; history of severe skin allergies or dermatoses; history of intolerance to nicotine patches; another member of household already participating in the study; taking a medication known to interact with nicotine; taking any prescription medication for depression or asthma.  Recruitment  Press releases and advertisements.  Intervention  Nicotine patch group: Subjects received nicotine patches for an 8‑week treatment period. Subjects applied two 21 mg nicotine patches per day for 6 weeks and one 21 mg patch per day for 2 weeks. Subjects also received behavioral counseling. Note: Medication adherence was 93%.  Control  Placebo patch group: Subjects received placebo patches for an 8-week treatment period. Subjects applied two identical-appearing placebo patches for 6 weeks and one placebo patch per day for 2 weeks. Subjects also received behavioral counseling. Note: Medication adherence was 61%.  Study Start/End Date  July 2010 to June 2012 (enrollment) | Study Population  Adult smokeless tobacco users in good health  Sample Size  Total: n=52 subjects  Nicotine patch group: n=25 subjects  Placebo patch group: n=27 subjects  Subject Characteristics  *Mean age±SD*  Nicotine patch group: 39.2±9.1 yrs  Placebo patch group: 35.3±9.5 yrs  *Sex (% male)*  Nicotine patch group: 100% (n=25)  Placebo patch group: 100% (n=27)  *Race/ethnicity*  Nicotine patch group: Caucasian, non-Hispanic, 92% (n=23); Caucasian, Hispanic, 8% (n=2)  Placebo patch group: Caucasian, non-Hispanic, 96% (n=26); Caucasian, Hispanic, 4% (n=1)  *Yrs smoked*  NR  *Average cpd*  NR  Abstinence Characteristics  *7-day PPA at the end of treatment (confirmed by urinary anabasine <2 ng/ml) (%)*  Nicotine patch group: 44% (n=11)  Placebo patch group: 22% (n=6)  *Prolonged abstinence* (%)*  Nicotine patch group: 44% (n=11)  Placebo patch group: 22% (n=6)  *Defined as meeting criteria for 7-day PPA and also reporting no tobacco use for 7 consecutive days, nor at least once each week, on 2 consecutive weeks, since 2 weeks following their target quit date. | Arrhythmia  NR  Nonfatal Myocardial Infarction*  Nicotine patch group: n=0  Placebo patch group: n=0  Nonfatal Stroke*  Nicotine patch group: n=0  Placebo patch group: n=0  Cardiovascular Death*  Nicotine patch group: n=0  Placebo patch group: n=0  *No serious AEs were observed in the study. |
| 1393 | Ebbert et al., 2007  Location: US  Setting: Clinical  Trial Name: NR  Registration/Protocol: NR  Funding: NR  Design  Randomized, controlled clinical pilot study  Eligibility  Inclusion: Age ≥18 yrs; good general health; daily smokeless tobacco use for the past yr; ≥3 pouches or cans of smokeless tobacco per week at time of enrollment; interested in stopping smokeless tobacco use.  Exclusion: Unstable angina, myocardial infarction or coronary angioplasty in the past 3 months; clinically significant depression; a history of active alcoholism or drug abuse in the past yr; hypersensitivity to nicotine patches; serious skin allergies or dermatoses; use of another form of tobacco (cigarettes, pipes, or cigars) in the past 10 days; use of an investigational drug, an antipsychotic or antidepressant medication, or another tobacco treatment intervention within 30 days of enrollment; pregnant or lactating or likely to become pregnant during the medication phase; unable or unwilling to use a personal digital assistant electronic diary; another member of their household participated in the study.  Recruitment  Press releases and local advertisements.  Intervention  21 mg nicotine patch group: Subjects received three nicotine patches, one of which was an active 21 mg/day patch and two of which were placebos, for a total daily dose of 21 mg. Daily patch application was staggered to avoid possible nicotine toxicity. Subjects received 8 weeks of nicotine patch therapy with a taper: three patches on weeks 1 to 4, two on weeks 5 and 6, and one on weeks 7 and 8.  42 mg nicotine patch group: Subjects received three nicotine patches, two of which were active 21 mg/day patches and one of which was a placebo, for a total daily dose of 42 mg. Daily patch application was staggered to avoid possible nicotine toxicity. Subjects received 8 weeks of nicotine patch therapy with a taper: three patches on weeks 1 to 4, two on weeks 5 and 6, and one on weeks 7 and 8.  63 mg nicotine patch group: Subjects received three active 21 mg/day nicotine patches, for a total daily dose of 63 mg. Daily patch application was staggered to avoid possible nicotine toxicity. Subjects received 8 weeks of nicotine patch therapy with a taper: three patches on weeks 1 to 4, two on weeks 5 and 6, and one on weeks 7 and 8.  Control  Placebo patch group: Subjects received three placebo nicotine patches, for a total daily dose of 0 mg. Daily patch application was staggered to avoid possible nicotine toxicity. Subjects received 8 weeks of nicotine patch therapy with a taper: three patches on weeks 1 to 4, two on weeks 5 and 6, and one on weeks 7 and 8.  Study Start/End Date  NR | Study Population  Smokeless tobacco users in good general health  Sample Size  Total: n=42 subjects  21 mg nicotine patch group: n=10 subjects  42 mg nicotine patch group: n=11 subjects  63 mg nicotine patch group: n=10 subjects  Placebo patch group: n=11 subjects  Subject Characteristics  *Mean age±SD*  21 mg nicotine patch group: 34.1±9.4 yrs  42 mg nicotine patch group: 34.0±6.2 yrs  63 mg nicotine patch group: 36.6±7.7 yrs  Placebo patch group: 38.0±6.9 yrs  *Sex (% male)*  21 mg nicotine patch group: 100% (n=10)  42 mg nicotine patch group: 100% (n=11)  63 mg nicotine patch group: 100% (n=10)  Placebo patch group: 100% (n=11)  *Race/ethnicity*  NR  *Mean yrs of regular smokeless tobacco use±SD*  21 mg nicotine patch group: 16.1±6.7  42 mg nicotine patch group: 18.7±7.6  63 mg nicotine patch group: 16.2±8.2  Placebo patch group: 16.8±7.3  *Mean smokeless tobacco uses per day±SD*  21 mg nicotine patch group: 12.4±9.0  42 mg nicotine patch group: 13.8±7.4  63 mg nicotine patch group: 12.6±8.1  Placebo patch group: 11.3±5.4  Abstinence Characteristics  *Continuous tobacco abstinence at Week 8 (duration of intervention) (%)*  21 mg nicotine patch group: 40% (n=4)  42 mg nicotine patch group: 55% (n=6)  63 mg nicotine patch group: 70% (n=7)  Placebo patch group: 45% (n=5)  *7-day PPA at Week 8 (%)*  21 mg nicotine patch group: 40% (n=4)  42 mg nicotine patch group: 73% (n=8)  63 mg nicotine patch group: 70% (n=7)  Placebo patch group: 73% (n=8) | Arrhythmia  NR  Nonfatal Myocardial Infarction*  21 mg nicotine patch group: n=0  42 mg nicotine patch group: n=0  63 mg nicotine patch group: n=0  Placebo patch group: n=0  Nonfatal Stroke*  21 mg nicotine patch group: n=0  42 mg nicotine patch group: n=0  63 mg nicotine patch group: n=0  Placebo patch group: n=0  Cardiovascular Death*  21 mg nicotine patch group: n=0  42 mg nicotine patch group: n=0  63 mg nicotine patch group: n=0  Placebo patch group: n=0  *Authors reported that no serious AEs occurred during study. |
| 1660 | Etter et al., 2002  Location: Switzerland  Setting: Community  Trial Name: NR  Registration/Protocol: NR  Funding: Supported by grants from the Swiss National Science Foundation and the Swiss Federal Office of Public Health; nicotine and placebo products were provided by Pharmacia; authors received reimbursement from Pharmacia for attending international conferences; primary author was paid by Novartis for lectures; the Institute of Social and Preventive Medicine of the University of Geneva received financial support from Novartis to develop an education program for users of Nicotinell^®^ products; third author received research funds from Pharmacia  Design  Three-armed, single-blind, RCT.  Eligibility  Inclusion: Adult (18-60 yrs) cigarette smokers (for ≥3 yrs; smoking <20 cpd) with no intention to quit smoking in the next 6 months. Only one subject per household was recruited to avoid cross-contamination of study groups. Subjects with smoking-related diseases were enrolled only if their physicians agreed. All subjects committed to try to reduce by half their daily cigarette consumption, and to not using commercial NRT products during the study.  Exclusion: Pregnancy or intention to become pregnant; currently breast-feeding; currently treated for severe psychiatric disorder; DSM diagnosis of addiction to alcohol or illicit drugs; active peptic ulcer; current use of NRT; current participation in a smoking cessation program; severe or unstable coronary heart disease or serious cardiac arrhythmia; myocardial infarction or stroke in the past 3 months; and cancer patients under treatment.  Recruitment  Physicians contacted (by mail) and invited to enroll their patients; 23 advertisements in newspapers were placed; and invitations were mailed to a random sample of 70,000 residents of French-speaking Switzerland.  Intervention  Nicotine group: Subjects could choose among a nicotine transdermal patch (containing 25 mg and delivering 15 mg nicotine over 16 hrs), nicotine gum (containing 4 mg and delivering 2 mg nicotine), or a nicotine inhaler (containing 10 mg and delivering 5 mg nicotine) (Nicorette^®^; Pharmacia, Helsingborg, Sweden). Subjects could switch between products or use several products at the same time; products were sent by mail for the full 6-month trial duration. Note: At Month 6, 36.1% of subjects used NRT daily, 25.2% used NRT occasionally (non-daily), and 32.9% of subjects never used NRT.  Control  Placebo group: Subjects could choose among placebo patches, gums, and inhalers matching those described in the intervention (Pharmacia). Subjects could switch between products or use several products at the same time; products were sent by mail for the full 6-month trial duration. Note: At Month 6, 25.3% of subjects used placebo daily, 30.4% occasionally (non-daily), and 44.3% of subjects never used placebo.  Control group: Subjects received no treatment. Note: At Month 6, 2.4% of subjects used NRT daily, 2.6% used NRT occasionally (non-daily), and 95.0% of subjects never used NRT.  Study Start/End Date  NR (6-month study duration; yrs of study 1999 to 2001) | Study Population  Adult smokers from the general population of French-speaking Switzerland  Sample Size  Total: n=923 subjects  Nicotine group: n=265 subjects  Placebo group: n=269 subjects  Control group: n=389 subjects  Subject Characteristics  *Mean age*  Nicotine group: 43.2 yrs  Placebo group: 42.7 yrs  Control group: 42.9 yrs  *Sex (% male)*  Nicotine group: 54%  Placebo group: 49%  Control group: 44%  *Race/ethnicity*  NR  *Yrs smoked*  NR  *Mean cpd±SD*  Nicotine group: 29.8±10.3  Placebo group: 29.4±9.4  Control group: 30.2±10.4  Abstinence Characteristics  *Prevalence of past 7-day abstinence (no puff of tobacco) (%)*  Nicotine group: 5.3%  Placebo group: 2.2%  Control group: 4.1%  *Prevalence of past 4-week abstinence (no puff of* tobacco*) (%)*  Nicotine group: 4.2%  Placebo group: 1.9%  Control group: 3.9% | Arrhythmia  NR  Nonfatal Myocardial Infarction  NR  Nonfatal Stroke  NR  Cardiovascular Death  Nicotine group: n=1  Placebo group: n=0  Control group: n=0  One subject in the nicotine group died during the study from a cerebral hemorrhage. |
| 1916 | Foulds et al., 1993  Location: UK  Setting: Public general hospital  Trial Name: NR  Registration/Protocol: NR  Funding: Medical Research Council; Imperial Cancer Research Fund; Kabi Pharmacia Therapeutics  Design  Randomized, double-blind, placebo-controlled study.  Eligibility  Inclusion: Age 18-68 yrs; smoking ≥10 cpd; desire to stop smoking.  Exclusion: Pregnant or lactating; using psychotropic medication; receiving smoking cessation treatment.  Recruitment  Physician referral.  Intervention  Nicotine patch group: Subjects received 30 cm^2^ nicotine patches containing 0.83 mg nicotine per cm^2^ releasing a mean of 15±3.5 mg during 16 hrs (Cygnus Research Corp, Redwood City, California, supplied by Kabi Pharmacia AB, Helsingborg, Sweden). Subjects used one patch per day, sticking it on in the morning and removing it at night. For continuing smokers at Week 1, a doubling of the dose (2 patches/day) was indicated. Note: 29% of subjects were using patches at Week 12.  Control  Placebo patch group: Subjects received placebo patches identical in size and appearance to nicotine patches, but not containing any nicotine. Subjects used one patch per day, sticking it on in the morning and removing it at night. For continuing smokers at Week 1, a doubling of the dose (2 patches/day) was indicated. Note: 16% of subjects were using patches at Week 12.  Study Start/End Date  NR | Study Population  Adult smokers  Sample Size  Total: n=248 subjects  Nicotine patch group: n=124 subjects  Placebo patch group: n=124 subjects  Subject Characteristics  *Mean age±SD*  Nicotine patch group: 45.8±12 yrs  Placebo patch group: 46.9±12 yrs  *Sex (% female)*  Nicotine patch group: 50% (n=62)  Placebo patch group: 49% (n=61)  *Race/ethnicity*  NR  *Mean yrs smoked±SD*  Nicotine patch group: 29.4±13  Placebo patch group: 30.8±12  *Mean cpd±SD*  Nicotine patch group: 25.4±12  Placebo patch group: 23.9±12  Abstinence Characteristics  *Continuous abstinence (eCO<10 ppm) from Week 6-Week 12 (%)*  Nicotine patch group: 17/124 (13.7%)  Placebo patch group: 13/124 (10.5%) | Arrhythmia  NR  Nonfatal Myocardial Infarction  Nicotine patch group: n=0  Placebo patch group: n=1  Nonfatal Stroke  NR  Cardiovascular Death  Nicotine patch group: n=0  Placebo patch group: n=0  One subject in the nicotine patch group died of atherosclerotic vascular disease during the first 12 weeks; however, this was after dropping out of the study due to imprisonment. |
| 133 | Gilbert et al., 2020  Location: US  Setting: Southern Illinois University Carbondale  Trial Name: Nicotine Patch for Marijuana Withdrawal (NMW)  Registration/Protocol: NCT01400243  Funding: NIDA; NIH  Design  Double-blind RCT  Eligibility  Inclusion: Age 18-50 yrs; use cannabis ≥10 times per week during the last yr; willingness to abstain from cannabis and tobacco/nicotine for 15 days; baseline urine cotinine concentration ≤100-200 ng/ml during nicotine-free baseline period; smoking ≤5 cpd or equivalent tobacco/nicotine use during the past 2 yrs.  Exclusion: Weekly use of psychoactive drugs or medications (other than cannabis, alcohol, or caffeine); smoking ≥5 cpd; drinking >28 alcoholic drinks per week; non-English speaking; education <12 yrs; atypical sleep cycles; pregnancy; myocardial infarction; BMI <18.5 or >35; use of creatinine-containing supplements; diagnoses of major depression or psychotic disorder.  Recruitment  University postings and newspaper ads.  Intervention  Nicotine patch group: Subjects were instructed to use the low-dose (7 mg) nicotine patch (Habitrol^®^) for 15 days.  Control  Placebo patch group: Subjects were instructed to use non-nicotine patches (Rejuvenation Labs, Salt Lake City, UT) for 15 days.  Study Start/End Date  April 2011 to June 2014 | Study Population  Individuals with DSM-V-assessed cannabis use disorder  Sample Size*  Total: n=120 subjects  Nicotine patch group: n=61 subjects  Placebo patch group: n=59 subjects  Subject Characteristics**  *Mean age±SD*  Nicotine patch group: 210.1±3.83 yrs  Placebo patch group: 20.48±2.34 yrs  *Sex (% male)*  Nicotine patch group: 78.4% (n=40)  Placebo patch group: 68.6% (n=35)  *Race/ethnicity*  Nicotine patch group: African American, 41.2% (n=21); Asian, 0.0% (n=0); Caucasian, 45.1% (n=23); Other, 13.7% (n=7)  Placebo patch group: African American, 40.0% (n=20); Asian, 2.0% (n=1); Caucasian, 52.0% (n=26); Other, 6.0% (n=3)  *Yrs smoked*  NR  *Average cpd*  NR  *Reporting of serious AEs was among the entire randomized sample (n=127).  **Subject characteristics are based on the analytical sample (total, n=101; nicotine patch group, n=51; placebo patch group, n=50).  Abstinence Characteristics  *eCO concentration (eCO values NR)*  Nicotine patch group: <5 ppm  Placebo patch group: <5 ppm  No subjects in either group reported an eCO of ≥5 ppm throughout treatment period. | Arrhythmia  NR  Nonfatal Myocardial Infarction*  Nicotine patch group: n=0  Placebo patch group: n=0  Nonfatal Stroke*  Nicotine patch group: n=0  Placebo patch group: n=0  Cardiovascular Death*  Nicotine patch group: n=0  Placebo patch group: n=0  *No serious AEs were observed in the study. |
| 1755 | Hays et al., 1999  Location: US  Setting: Community  Trial Name: NR  Registration/Protocol: NR  Funding: Elan Pharmaceutical Research Corp, Gainesville, GA  Design  Simulation of an over-the-counter environment, via two parallel smoking cessation trials: a double-blind, placebo-controlled trial of active versus placebo nicotine patch (provided free of charge); and an open-label trial in which subjects paid for active nicotine patches. Note: Only data from the double-blind trial are reported, as the open‑label trial was singe-armed.  Eligibility  Inclusion: Adult (≥18 yrs) cigarette smokers (had smoked for at ≥1 yr and smoked ≥15 cpd during the past 6 months) who were motivated to stop smoking.  Exclusion: Pregnant or lactating women; individuals with a recent myocardial infarction (within the past month), those using any tobacco products other than cigarettes (in the past 30 days); and those currently using other forms of nicotine (e.g., nicotine gum).  Recruitment  Media advertisements.  Intervention  Nicotine group: Subjects were given a 1-week supply of 22 mg/24 hr nicotine patches on their first visit, to be used once per day. Subjects were required to return weekly for 6 weeks to obtain their subsequent 1-week supplies. Note: There was no significant difference in the number of boxes of patches dispensed to both groups in the placebo-controlled trial (patches dispensed: nicotine group, 5.1±2.1; placebo patch group, 4.8±2.1; p=0.132).  Control  Placebo patch group: Subjects were given a 1-week supply of placebo nicotine patches on their first visit, to be used once per day. Subjects were required to return weekly for 6 weeks to obtain their subsequent 1-week supplies. Note: There was no significant difference in the number of boxes of patches dispensed to both groups in the placebo-controlled trial (patches dispensed: nicotine group, 5.1±2.1; placebo patch group, 4.8±2.1; P=0.132).  Study Start/End Date  NR (24-week follow-up period; yrs of study 1994 to 1995) | Study Population  Adult cigarette smokers  Sample Size  Total: n=643 subjects  Nicotine patch group: n=321 subjects  Placebo patch group: n=322 subjects  Subject Characteristics  *Mean age±SD*  Nicotine patch group: 43.5±11.2 yrs  Placebo patch group: 44.1±11.6 yrs  *Sex*  Nicotine patch group: female, 51.4%; male, 48.6%  Placebo patch group: female, 47.5%; male, 52.5%  *Race/ethnicity*  NR  *Yrs smoked±SD*  Nicotine patch group: 25.2±11.4  Placebo patch group: 25.9.1±11.9  *Distribution of mean cpd*  Nicotine patch group: 16 to 20 cpd (35.5%); 21 to 40 cpd (54.2%); >40 cpd (10.3%)  Placebo patch group: 16 to 20 cpd (32.0%); 21 to 40 cpd (58.4%); >40 cpd (9.6%)  Abstinence Characteristics  *Prevalence of biochemically-verified 7day PPA at Week 6 (%)*  Nicotine patch group: 16.8%  Placebo patch group: 9.6% | Arrhythmia  NR  Nonfatal Myocardial Infarction  NR  Nonfatal Stroke  NR  Cardiovascular Death  Nicotine patch group: n=1  Placebo patch group: n=0  One cardiovascular death due to myocardial infarction occurred in the nicotine patch group; subject had failed to return after 3 weeks, and had reported continued smoking at each follow-up that had been attended; family members also reported that nicotine patches were not being used, and that the subject had continued smoking. |
| 1612 | Hughes et al., 2003  Location: US  Setting: Community  Trial Name: NR  Registration/Protocol: NR  Funding: NIAAA and NIDA  Design  RCT.  Eligibility  Inclusion: Age ≥18 yrs; smoking ≥20 cpd; desire to quit smoking; not using non-cigarette tobacco products; past alcohol dependence but no alcohol or illicit drug intake in the last 30 days; not currently using disulfiram or naltrexone; no current major psychiatric disorder; not taking a psychoactive medication; no cardiac or dermatological disorder that is a risk factor when using nicotine patches; using adequate birth control (women).  Exclusion: NR  Recruitment  Newspaper and radio advertisements, fliers at outpatient alcohol treatment sites, and at Alcoholics Anonymous meetings.  Intervention  Nicotine patch group: Subjects received a Nicoderm^®^ 21 mg per day nicotine patch for 6 weeks; a 14 mg per day nicotine patch for 2 weeks; a 7 mg per day nicotine patch for the following 2 weeks; and placebo patch for the final 2 weeks. Subjects also attended behavioral therapy group sessions. Note: 77% of subjects across both groups wore the patch on all days that they did not smoke (rate per group NR).  Control  Placebo patch group: Subjects received a placebo patch for 12 weeks, in a parallel manner to the intervention. Subjects also attended behavioral therapy group sessions. Note: 77% of subjects across both groups wore the patch on all days that they did not smoke (rate per group NR).  Study Start/End Date  NR | Study Population  Adult cigarette smokers with past alcohol dependence  Sample Size  Total: n=115 subjects  Nicotine patch group: n=61 subjects  Placebo patch group: n=54 subjects  Subject Characteristics  *Mean age±SD*  Nicotine patch group: 43±8 yrs  Placebo patch group: 43±9 yrs  *Sex (% male)*  Nicotine patch group: 72%  Placebo patch group: 63%  *Race/ethnicity (% Caucasian)*  Nicotine patch group: 97%  Placebo patch group: 89%  *Mean age at smoking onset±SD*  Nicotine patch group: 15±3 yrs  Placebo patch group: 16±5 yrs  *Mean cpd±SD*  Nicotine patch group: 30±11  Placebo patch group: 29±11  Abstinence Characteristics  *Adjusted prolonged smoking abstinence rates (verified by eCO <10 ppm on all visits after the first 2 weeks) at Week 16 (%, 95% CI)*  Nicotine patch group: 28% (15%-46%)  Placebo patch group: 11% (4%-24%) | Arrhythmia  NR  Nonfatal Myocardial Infarction*  Nicotine patch group: n=0  Placebo patch group: n=0  Nonfatal Stroke*  Nicotine patch group: n=0  Placebo patch group: n=0  Cardiovascular Death*  Nicotine patch group: n=0  Placebo patch group: n=0  *There were no serious AEs and no dropouts due to AEs. |
| 1835 | Joseph et al., 1996  Location: US  Setting: Clinical  Trial Name: NR  Registration/Protocol: NR  Funding: Hoechst Marion Roussel  Design  Randomized, double-blind, placebo-controlled trial.  Eligibility  Inclusion: Smokers, age ≥45 yrs, smoking ≥15 cpd for ≥5 yrs, a minimum of 2 previous quit attempts, and eCO >8 ppm. All subjects had one or more of the following diagnosed conditions: history of myocardial infarction, coronary artery bypass surgery or angioplasty; stenosis of ≥ 50% in ≥1 major coronary artery; clinical history of angina, congestive heart failure, cor pulmonale, arrhythmia, peripheral vascular disease, or cerebrovascular disease.  Exclusion: Any of the following in the 2 weeks before randomization: unstable angina, myocardial infarction, coronary artery bypass surgery, angioplasty, hospitalization for cardiac arrhythmia; a history of continuous use of transdermal nicotine for >48 hrs, current use of other tobacco products or nicotine gum, unstable psychiatric illness or an unstable disorder involving the use of alcohol or controlled substances, history of severe dermatitis, or pregnancy.  Recruitment  Subjects recruited from 10 Veterans Affairs medical centers.  Intervention  Nicotine patch group: Transdermal nicotine (Nicoderm^®^, Hoechst Marion Roussel) administered as a 21 mg patch for 6 weeks, 14 mg patch for 2 weeks, and a 7 mg patch for 2 weeks, for a total course of treatment of 10 weeks. Subjects were instructed to apply a new patch each morning. Note: At Week 6, 73% of subjects were still wearing nicotine patches.  Control  Placebo patch group: Transdermal placebo patch identical in size, appearance, and odor to nicotine patches. Note: At Week 6, 56% of subjects were still wearing placebo patches.  Study Start/End Date  November 1994 to June 1995 | Study Population  Smokers with a history of ≥1 major CVD  Sample Size  Total: n=584 subjects  Nicotine patch group: n=294 subjects  Placebo patch group: n=290 subjects  Subject Characteristics  *Average age*  Nicotine patch group: 61 yrs  Placebo patch group: 60 yrs  *Sex*  Total: female, n=8; male, n=576  *Race/ethnicity*  NR  *Mean yrs smoked*  Nicotine patch group: 44  Placebo patch group: 44  *Mean cpd*  Nicotine patch group: 28  Placebo patch group: 28  Abstinence Characteristics  *eCO-verified continuous (8-week) abstinence at 14 weeks (%)*  Nicotine patch group: 21%  Placebo patch group: 9% | Arrhythmia  Nicotine patch group: n=5  Placebo patch group: n=3  Of the 5 subjects in the nicotine group, 4 were currently smoking cigarettes at time admission for arrhythmia, and one was not.  Of the three subjects in the placebo patch group, three were currently smoking cigarettes at time admission for arrhythmia.  Nonfatal Myocardial Infarction  Nicotine patch group: n=0  Placebo patch group: n=1  The subject was reported as currently smoking cigarettes at time of myocardial infarction.  Nonfatal Stroke  NR  Cardiovascular Death  NR |
| 1860 | Kavin and Shivey, 1995  Location: US  Setting: Lutheran General Hospital  Trial Name: NR  Registration/Protocol: NR  Funding: Park Ridge Health Foundation  Design  Randomized, double-blind, crossover, placebo-controlled study.  Note: The objective of nicotine treatment was to assess changes in anorectal sensorimotor function.  Eligibility  Inclusion: Quit smoking for ≥1 month  Exclusion: NR  Recruitment  NR  Intervention  Nicotine gum group: Subjects were instructed to chew 3 pieces of nicotine gum containing 2 mg nicotine (Nicorette, Marion Merrill Dow Inc. Kansas City, Missouri) 15 times and then "park" the gum bolus between the cheek and gum for 2 min repeating this cycle for 30 min. At 30 min, the gum was replaced with three identical fresh pieces of gum. Treatment was administered in a cross-over design, with a washout period 7 or more between treatments.  Control  Placebo gum group: Subjects were instructed to chew a placebo gum identical in appearance and taste to the nicotine gum. Placebo gum contained 1 mg of biologically inactivated nicotine. Treatment was administered in a cross-over design, with a washout period 7 or more days between treatments.  Study Start/End Date  NR | Study Population  Healthy former smokers  Sample Size  Total: n=11 subjects  Nicotine gum group: n=11 subjects  Placebo gum group: n=11 subjects  Subject Characteristics  *Average age*  NR  *Sex*  NR  *Race/ethnicity*  NR  *Yrs smoked*  NA  *Average cpd*  NA  Abstinence Characteristics  NA | Arrhythmia  Nicotine gum group: n=7  Placebo gum group: n=0  Chewing nicotine gum induced mild tachycardia in 7 subjects.  Nonfatal Myocardial Infarction  NR  Nonfatal Stroke  NR  Cardiovascular Death  NR |
| 1074 | Koychev et al., 2011  Location: UK  Setting: 3 sites in Manchester, London and Cardiff  Trial Name: NR  Registration/Protocol: NR  Funding: P1vital CNS Experimental Medicine Consortium (members AstraZeneca, GlaxoSmithKline, Lundbeck, Organon (a subsidiary of Merck and Pfizer)  Design  Double-blind, randomized, placebo-controlled, clinical trial. Note: Nicotine was used as a cognitive enhancing agent, not as a smoking cessation agent.  Eligibility  Inclusion: Age 18–45 yrs; fluent in English; physically healthy (BMI, blood pressure, ECG, and blood biochemistry all within normal range).  Exclusion: Not providing a clear screen for alcohol, illicit drug use and pregnancy on the screening and day of testing; known or suspected intolerance or allergy to the study drugs; smoking >5 cpd; consumption of >8 standard caffeinated drinks per day; history of migraines; significant visual or hearing impairment; participation in any other drug trial in the 84 days before the randomization visit; prescribed medication 14 days prior to or over the counter medicine 48 hrs before randomization.  Recruitment  Recruited from the sites via an online questionnaire measuring schizotypy (Schizotypal Personality Questionnaire in its short and full version).  Intervention  Nicotine patch group: Subjects received a 7 mg nicotine patch (brand NR) and a placebo capsule. The nicotine patch was applied first and the placebo capsule was administered 3 hrs later. Tests were performed 4.5 hrs after the application of the nicotine patch.  Control  Amisulpride group: Subjects received a placebo patch and 400 mg amisulpride capsule. The placebo patch was applied first and the amisulpride capsule was administered 3 hrs later.  Risperidone group: Subject received a placebo patch and 2 mg risperidone capsule. The placebo patch was applied first and the risperidone capsule was administered 3 hrs later.  Placebo group: Subjects received placebo patch and placebo capsule. The placebo patch was applied first and the placebo capsule was administered 3 hrs later.  Study Start/End Date  NR | Study Population  Healthy subjects scoring high and average on a schizotypy personality measure  Sample Size  Total: n=244 subjects  Nicotine patch group: n=61 subjects  Amisulpride group: n=62 subjects  Risperidone group: n=62 subjects  Placebo group: n=59 subjects  Subject Characteristics  *Average age*  NR  *Sex*  NR  *Race/ethnicity*  NR  *Average yrs smoked*  NR  *Average cpd*  NR  Abstinence Characteristics  NA | Arrhythmia  NR  Nonfatal Myocardial Infarction*  Nicotine patch group: n=0  Amisulpride group: n=0  Risperidone group: n=0  Placebo group: n=0  Nonfatal Stroke*  Nicotine patch group: n=0  Amisulpride group: n=0  Risperidone group: n=0  Placebo group: n=0  Cardiovascular Death*  Nicotine patch group: n=0  Amisulpride group: n=0  Risperidone group: n=0  Placebo group: n=0  *No discontinuation during randomization was due to serious AEs (i.e., it is assumed that study was reporting on serious AEs). |
| 1194 | Kralikova et al., 2009  Location: Czech Republic  Setting: Community  Trial Name: NR  Registration/Protocol: NR  Funding: McNeil AB, Helsingborg, Sweden  Design  Randomized, double-blind, placebo-controlled study.  Eligibility  Inclusion: Age ≥18 yrs; smoking ≥15 cpd; smoked regularly for ≥3 yrs; eCO ≥10 ppm; desire to reduce smoking; ≥1 previous failed quit attempt.  Exclusion: Current use of NRT or other nicotine-containing products (e.g., cigars, pipes, snuff); current involvement in other smoking cessation or reduction programs; unstable angina pectoris or myocardial infarction within the previous 3 months; pregnancy, lactation or intended pregnancy; psychiatric treatment or medication; co-existing alcohol or other drug problems.  Recruitment  Advertisement in the local newspaper and local leaflets.  Intervention  NRT group: Subjects were instructed to reduce smoking by replacing as many cigarettes as possible with either nicotine inhaler (10 mg nicotine) or gum (4 mg nicotine), according to their choice for 6 months, followed by up to 3 months of voluntary tapering. Subjects also received up to 10 min of behavioral counselling during the study. Note: At 9 months, 45% of subjects used NRT daily.  Control  Placebo group: Subjects were instructed to reduce smoking by replacing as many cigarettes as possible with either placebo inhaler or placebo gum. Subjects also received up to 10 min of behavioral counselling during the study. Note: At 9 months, 39% of subjects used the placebo treatment daily.  Study Start/End Date  January 1999 to May 1999 (recruitment) | Study Population  Adult cigarette smokers  Sample Size  Total: n=314 subjects  NRT group: n=209 subjects  Placebo group: n=105 subjects  Subject Characteristics  *Mean age±SD (range)*  NRT group: 46.1±10.5 (20-68) yrs  Placebo group: 46.6±10.0 (20-67) yrs  *Sex*  NRT group: male, n=89; female, n=120  Placebo group: male, n=42; female, n=63  *Race/ethnicity*  NR  *Yrs smoked*  NR  *Mean cpd±SD (range)*  NRT group: 25.7±9.8 (13-60)  Placebo group: 25.2±8.2 (15-70)  Abstinence Characteristics  *Sustained abstinence rate (not a single cigarette smoked and eCO <10 ppm at each visit) (%)*  From Week 6 to Month 4  NRT group: 20.1% (n=42)  Placebo group: 8.6% (n=9)  From Month 6 to Month 12:  NRT group: 18.7% (n=39)  Placebo group: 8.6% (n=9) | Arrhythmia  NR  Nonfatal Myocardial Infarction*  NRT group: n=0  Placebo group: n=0  *Reported serious AEs were listed, none of which were nonfatal myocardial infarction.  Nonfatal Stroke**  NRT group: n=0  Placebo group: n=1  **Cerebrovascular disorder in a female subject, 52 yrs old, having smoked for 27 yrs and smoking 33 cpd at baseline.  Information based on additional information sent by the author.  Cardiovascular Death  NRT group: n=0  Placebo group: n=0 |
| 402 | Lambrichts et al., 2017  Location: Netherlands  Setting: 3 clinical centers  Trial Name: NR  Registration/Protocol: NR  Funding: Stichting Coolsingel  Design  Randomized, double-blind, placebo-controlled study. Note: The objective of nicotine treatment was to improve gastrointestinal motility and bowel recovery after colorectal resection.  Eligibility  Inclusion: Subjects who underwent an elective oncologic colorectal resection.  Exclusion: Severe chronic or acute CVD; severe liver or kidney disease; oral or pharyngeal infection; esophagitis; hypersensitivity to any component of the nicotine gum; previous colorectal surgery; pregnancy; breast feeding; elevated risk of choking or being unable to chew gum.  Recruitment  NR  Intervention  Nicotine gum group: Subjects were instructed to chew Nicorette^®^ 2 mg chewing gum 2 hrs preoperatively and 3 times a day postoperatively for a half an hr at a time, until the first passage of feces and tolerance of solid food for >24 hrs. Note: Compliance in the nicotine group was 15% (n=3) as per protocol; 65% (n=13) less than protocol; 0% (n=0) more than protocol; 20% (n=4) NR at postoperative Day 3.  Control  Placebo gum group: Subjects received placebo chewing gum. Subjects were instructed to chew placebo chewing gum 2 hrs preoperatively and 3 times a day postoperatively for a half an hr at a time, until the first passage of feces and tolerance of solid food for >24 hrs. Note: Compliance in the placebo gum group was 15% (n=3) as per protocol; 55% (n=11) less than protocol; 0% (n=0) more than protocol; 30% (n=6) NR at postoperative Day 3.  Study Start/End Date  January 2015 to July 2016 | Study Population  Adult smokers and nonsmokers undergoing colorectal resection  Sample Size  Total: n=46 subjects  Nicotine gum group: n=23 subjects  Placebo gum group: n=23 subjects  Subject Characteristics*  *Median age (IQR)*  Nicotine gum group: 69.00 (62.50-70.00) yrs  Placebo gum group: 67.50 (60.75‑74.75) yrs  *Sex*  Nicotine gum group: male, 70% (n=14); female, 30% (n=6)  Placebo gum group: male, 65% (n=13); female, 35% (n=7)  *Race/ethnicity*  NR  *Smoking status (% smokers)*  Nicotine gum group: 20% (n=4)  Placebo gum group: 0% (n=2)  *Yrs smoked*  NR  *Average cpd*  NR  *Subject characteristics are based on the analytical sample (Total, n=40; nicotine gum group, n=20; placebo gum group, n= 20)  Abstinence Characteristics  NR | Arrhythmia  NR  Atrial fibrillation occurred in one subject in the nicotine gum group during the first 30 days after surgery. However, this was unlikely to have occurred during the treatment period, given that nicotine gum was only administered until first passage of feces and tolerance of solid food for >24 hrs, and average time to this primary endpoint was 3.5 days for this group. Further, the authors note that this subject was known to have had previous episodes of paroxysmal atrial fibrillation.  Nonfatal Myocardial Infarction  Nicotine gum group: n=0  Placebo gum group: n=0  Nonfatal Stroke  NR  Cardiovascular Death  Nicotine gum group: n=0  Placebo gum group: n=0 |
| 677 | Lerman et al., 2015  Location: Multinational (Canada; US)  Setting: 4 academic medical centers  Trial Name: Pharmacogenetics of Nicotine Addiction Treatment  Registration/Protocol: NCT01314001  Funding: NIDA, NCI, National Human Genome Research Institute, National Institute on General Medical Sciences, Abramson Cancer Center at the University of Pennsylvania, Commonwealth of Pennsylvania Department of Health, Canadian Institutes of Health Research, endowed Chair in Addiction for the Department of Psychiatry, CAMH Foundation, Canada Foundation for Innovation, Ontario Ministry of Research and Innovation  Design  Double-blind, placebo-controlled randomized trial.  Eligibility  Inclusion: Age 18-65 yrs; smoking ≥10 cpd for ≥6 months (verified by eCO concentration >10 ppm).  Exclusion: Use of non-cigarette tobacco products, e-cigarettes, or current smoking treatment; history of substance misuse treatment; current use of cocaine or methamphetamine, or >25 alcoholic drinks per week; medical contraindications (pregnancy, history of cancer, kidney or liver disease, or transplant, clinically significant cardiac dysrhythmias, stroke, angina, heart attack, or uncontrolled hypertension); history of DSM-IV Axis 1 psychiatric disorder or suicide risk score on the MINI International Neuropsychiatric Interview >1, or current major depression; current use of antipsychotics, stimulants, opiate medications, anti-coagulants, rescue inhalers, antiarrhythmics, or medications altering CYP2A6 activity; inability to provide informed consent or any condition that could compromise safety.  Recruitment  Advertisements for a free smoking cessation program.  Intervention  Nicotine patch+placebo pill group: Subjects received nicotine patches (Nicoderm^®^ CQ^®^, GlaxoSmith Kline) containing 21 mg nicotine for 6 weeks, 14 mg nicotine for 2 weeks, and 7 mg nicotine for 3 weeks, as well as placebo pills for 12 weeks, in a parallel manner to the placebo patch + varenicline group. Subjects also received placebo pills Subjects also received counselling sessions. Note: On average, 62% of subjects used ≥80% the pill dose recommended; 63% of subjects used ≥80% the patches recommended.  Control  Placebo patch+varenicline: Subjects received varenicline (0.5 mg once daily Days 1-3, 0.5 mg twice daily on Days 4-7, and 1.0 mg twice daily on Days 8-84; Pfizer) starting 1 week before quit date, and placebo patches for 11 weeks, in a parallel manner to the nicotine patch+placebo pill group. Subjects also received counselling sessions.  Placebo patch+placebo pill: Subjects received placebo patches and placebo pills in a parallel manner to the active treatment arms. Subjects also received counselling sessions.  Note: On average, 62% of subjects used ≥80% the pill dose recommended; 63% of subjects used ≥80% the patches recommended.  Study Start/End Date  November 2010 to September 2014 | Study Population  Adult cigarette smokers  Sample Size  Total: n=1,246 subjects  Nicotine patch+placebo pill group: n=418 subjects  Placebo patch+varenicline group: n=420 subjects  Placebo patch+placebo pill group: n=408 subjects  Subject Characteristics  *Mean age±SD*  Nicotine patch+placebo pill group: 46±11 yrs  Placebo patch+varenicline group: 45±12 yrs  Placebo patch+placebo pill group: 46±11 yrs  *Sex (% female)*  Nicotine patch+placebo pill group: 44% (n=182)  Placebo patch+varenicline group: 45% (n=187)  Placebo patch+placebo pill group: 43% (n=174)  *Race/ethnicity*  Nicotine patch+placebo pill group: White, 56% (n=236); Black, 36% (n=151); Other, 7% (n=31)  Placebo patch+varenicline group: White, 55% (n=232); Black, 38% (n=160); Other, 7% (n=28)  Placebo patch+placebo pill group: White, 55% (n=225); Black, 37% (n=151); Other, 8% (n=32)  *Yrs smoked*  NR  *Mean cpd±SD*  Nicotine patch+placebo pill group: 18.0±7.0  Placebo patch+varenicline group: 17.5±5.9  Placebo patch+placebo pill group: 18.5±7.9  Abstinence Characteristics  *Rate of eCO-verified (≤8 ppm) 7-day PPA at end of treatment*  “Varenicline was more efficacious than nicotine patch in normal metabolisers (OR 2·17, 95% CI 1·38–3·42; p=0·001), but not in slow metabolisers (OR 1.13, 0.74–1.71; p=0.56).” | Arrhythmia  Nicotine patch+placebo pill group: n=0  Placebo patch+varenicline group: n=0  Placebo patch+placebo pill group: n=1 (atrial fibrillation; smoked 15 cpd at time of event)  Nonfatal Myocardial Infarction  Nicotine patch+placebo pill group: n=0  Placebo patch+varenicline group: n=0  Placebo patch+placebo pill group: n=0  Nonfatal Stroke  Nicotine patch+placebo pill group: n=0  Placebo patch+varenicline group: n=0  Placebo patch+placebo pill group: n=2  One subject in the placebo patch+placebo pill group had a stroke at Week 11 and was smoking 0 cpd at time of event; one subject in the placebo patch+placebo pill group had a stroke at Week 8 and was smoking 15 cpd at time of event.  Cardiovascular Death  NR  Two deaths (one in the nicotine patch+placebo pill group and one in the placebo patch + placebo pill group) occurred during treatment period, but cause of death was not specified. |
| 1803 | Lewis et al., 1998  Location: US  Setting: Clinical  Trial Name: NR  Registration/Protocol: NR  Funding: NR  Design  Randomized, double-blind, placebo-controlled trial.  Eligibility  Inclusion: Age ≥18 yrs; regular cigarette use for ≥1 yr preceding admission; smoking ≥10 cpd during the week prior to admission; commitment to quit smoking; wanted to participate and could provide informed consent; attending physician approval; medical appropriateness for nicotine patch treatment.  Exclusion: Drug or alcohol abuse within 6 months of admission; history of major psychiatric illness; pregnant women or women of reproductive age not using birth control; use of nicotine-containing products other than cigarettes; skin sensitivities; terminal illness; discharge planned within 24 hrs of admission; admission to intensive care unit; unstable angina; acute myocardial infraction; unstable arrhythmia; major cardiac or vascular surgery or angioplasty.  Recruitment  Subjects admitted to a hospital that expressed an interest in participating were recruited.  Intervention  Nicotine patch group: Subjects received a set of twenty-one 22 mg nicotine patches for Weeks 1-3, and a set of twenty-one 11 mg nicotine patches for Weeks 4‑6. Subjects also received extended bedside and telephone counselling. Brief physician-delivered motivational message (2-3 min) and a smoking cessation pamphlet were also provided. Note: Treatment compliance rate at Week 1 was 70% (n=40).    Control  Placebo patch group: Subjects received placebo patches for Weeks 1‑3 and a set for Weeks 4-6. Subjects also received extended bedside and telephone counselling. Brief physician-delivered motivational message (2-3 min) and a smoking cessation pamphlet were also provided. Note: Treatment compliance rate at Week 1 was 60% (n=34).  Minimal care group: Subjects received a brief physician-delivered motivational message (2-3 min) and a smoking cessation pamphlet.  Study Start/End Date  NR | Study Population  Subjects admitted to the University of Wisconsin Hospital and Clinics  Sample Size  Total: n=185 subjects  Nicotine patch group: n=62 subjects  Placebo patch group: n=62 subjects  Minimal care group: n=61 subjects  Subject Characteristics  *Mean age±SD*  Nicotine patch group: 43.4±13.6 yrs  Placebo patch group: 44.7±13.6 yrs  Minimal care group: 43.0±11.6 yrs  *Sex (% female)*  Nicotine patch group: 44.3% (n=27)  Placebo patch group: 51.6% (n=32)  Minimal care group: 42.6% (n=26)  *Race/ethnicity*  NR  *Mean yrs smoked±SD*  Nicotine patch group: 26.3±13.4  Placebo patch group: 27.5±13.8  Minimal care group: 25.4±12.7  *Mean cpd±SD*  Nicotine patch group: 24.0±15.8  Placebo patch group: 24.9±10.9  Minimal care group: 22.5±10.6  Abstinence Characteristics  *Self-reported 7-day PPA (%) at End of Treatment (Week 6)*  Nicotine patch group: 30.6% (n=19)  Placebo patch group: 17.7% (n=11)  Minimal care group: NR | Arrhythmia*  Nicotine patch group: n=0  Placebo patch group: n=0  Minimal care group: n=NR  Nonfatal Myocardial Infarction*  Nicotine patch group: n=0  Placebo patch group: n=0  Minimal care group: n=NR  Nonfatal Stroke*  Nicotine patch group: n=0  Placebo patch group: n=0  Minimal care group: n=NR  Cardiovascular Death*  Nicotine patch group: n=0  Placebo patch group: n=0  Minimal care group: n=NR  *No serious or life-threatening AEs (i.e., arrhythmias or myocardial infarction) were reported in either patch group. |
| 1678 | Møller et al., 2002  Location: Denmark  Setting: Hospital  Trial Name: NR  Registration/Protocol: NR  Funding: Danish Research Councils, Danish Ministry of Health, and Director Danielsen’s Foundation; Pharmacia Upjohn sponsored the nicotine substitution products used in the study  Design  RCT.  Eligibility  Inclusion: Daily cigarette smokers  Exclusion: Weekly alcohol intake >35 units  Recruitment  Patient referral.  Intervention  NRT group: Subjects received personalized nicotine substitution products (Pharmacia Upjohn) based on their preference (type of NRT not specified) for 6 to 8 weeks prior to surgery and 10 days after surgery. Subjects were encouraged to stop smoking completely, but had the option to reduce tobacco consumption by ≥50%.  Control  Standard care group: Subjects received standard care, consisting of little or no information about the risk of tobacco smoking or smoking cessation counselling.  Study Start/End Date  NR | Study Population  Cigarette smokers scheduled for primary elective hip or knee alloplasty  Sample Size  Total: n=108 subjects  NRT group: n=56 subjects  Standard care group: n=52 subjects  Subject Characteristics  *Median age (range)*  NRT group: 66 (41-83) yrs  Standard care group: 64 (30-85) yrs  *Sex (% female)*  NRT group: 57% (n=32)  Standard care group: 58% (n=30)  *Race/ethnicity*  NR  *Yrs smoked*  NR  *Median cpd (range)*  NRT group: 15 (5-30)  Standard care group: 15 (3-30)  Abstinence Characteristics  NR | Arrhythmia  NR  Nonfatal Myocardial Infarction  NR  Study reported five cases of cardiovascular insufficiency in the standard care group, defined as myocardial infarction or congestive heart failure; however, it could not be determined which (if any) of these cases were myocardial infarctions  Nonfatal Stroke*  NRT group: n=0  Standard care group: n=0  Cardiovascular Death  NRT group: n=0  Standard care group: n=0  *Reported postoperative complications were listed, none of which were nonfatal stroke. |
| 1339 | Myung et al., 2007  Location: South Korea  Setting: Workers at Kyobo Life Insurance Co.  Trial Name: NR  Registration/Protocol: NR  Funding: “Supported in part by patches from a pharmaceutical company; the company does not want to disclose its name because there were no significant positive findings regarding the effectiveness of nicotine patch therapy in this trial”  Design  Randomized, double-blind, placebo-controlled trial.  Eligibility  Inclusion: Age 20-55 yrs; no major physical or psychiatric illness; motivated to quit smoking, regardless of daily cigarette consumption.  Exclusion: Used NRT within 6 months before enrollment; alcohol or narcotic drug abuse; allergic skin diseases; heart problems including ischemic heart disease, myocardial infarction, arrhythmia, or cerebrovascular disease.  Recruitment  Subjects were recruited from Kyobo Life Insurance Co. through bulletin board announcements and e-mail.  Intervention  Nicotine patch group: Subjects received transdermal nicotine patches for 6 weeks. Subjects received 30 cm^2^ patches containing 57 mg nicotine per patch (delivering 21 mg of nicotine per day) for 2 weeks, 20 cm^2^ patches containing 38 mg per patch (delivering 14 mg of nicotine per day) for the following 2 weeks, and 10 cm^2^ patches containing 19 mg per patch (delivering 7 mg of nicotine per day) for the final 2 weeks. Subjects also received behavioral counseling.  Control  Placebo patch group: Subjects received placebo patches identical in appearance to nicotine patches for 6 weeks. Subjects also received behavioral counseling.  Study Start/End Date  July 2004 to August 2005 | Study Population  Male adult cigarette smokers  Sample Size  Total: n=118 subjects  Nicotine patch group: n=59 subjects  Placebo patch group: n=59 subjects  Subject Characteristics  *Mean age±SD*  Nicotine patch group: 36.6±5.6 yrs  Placebo patch group: 36.7±6.2 yrs  *Sex*  NA  *Race/ethnicity*  NR  *Mean yrs smoked±SD*  Nicotine patch group: 17.1±5.7  Placebo patch group: 16.5±5.8  *Mean cpd±SD*  Nicotine patch group: 15.0±5.8  Placebo patch group: 15.3±5.1  Abstinence Characteristics  *Self-reported point prevalence at 3 months (%)*  Nicotine patch group: 23.7% (n=14)  Placebo patch group: 16.9% (n=10) | Arrhythmia  NR  Nonfatal Myocardial Infarction*  Nicotine patch group: n=0  Placebo patch group: n=0  Nonfatal Stroke*  Nicotine patch group: n=0  Placebo patch group: n=0  Cardiovascular Death*  Nicotine patch group: n=0  Placebo patch group: n=0  *No serious AEs were observed in either group. |
| 776 | Oncken et al., 2014  Location: US  Setting: University of Connecticut Health Center  Trial Name: Topiramate Alone and in Combination With the Nicotine Patch for Smoking Cessation: A Pilot Study  Registration/Protocol: NCT00755716  Funding: Department of Medicine at the University of Connecticut Health Center; General Clinical Research Center; NIAAA  Design  Randomized, placebo-controlled study.  Eligibility  Inclusion: Age 18-65 yrs; smoking ≥10 cpd during the past yr with no period of abstinence >3 months; medically stable (i.e., no serious or unstable medical or psychiatric condition for ≥6 months); women were required to be postmenopausal for ≥2 yrs or practice an effective method of birth control; only 1 member of a household was allowed to participate in the study.  Exclusion: Current major depressive episode; treatment of depression within the last 12 months; history of psychotic, panic, or bipolar disorders; BMI <18 kg/in^2^,uncontrolled hypertension; clinically significant hepatic or renal impairment; history of nephrolithiasis or glaucoma; a previous serious AE or hypersensitivity to topiramate; a significant or severe drug allergy; a past-yr history of drug or alcohol abuse or dependence; investigational drug treatment within 30 days of the baseline visit; tobacco dependence treatment within the past 6 months; use of non-cigarette tobacco products; lactose intolerance; use of medication known to have a clinically significant interaction with topiramate.  Recruitment  Subjects were recruited via advertising.  Intervention  Nicotine patch+topiramate group: Subjects received topiramate starting at the baseline visit and the dosage was titrated up over 5 weeks (25 mg per day for 1 week, 25 mg twice daily for 1 week, 50 mg twice daily for 1 week, 75 mg twice daily for 1 week, 100 mg twice daily for 5 weeks). During the last week of the study (Week 10) the dosage was decreased by 30% every 3 days. Starting on quit day (2 weeks after baseline visit), subjects received nicotine patches (21 mg per day) for 7 weeks, followed by 14 mg per day for 3 days, and 7 mg per day for 4 days. Note: Treatment adherence rate was 93%±10.1%.  Control  Placebo tablet group: Subjects received a placebo tablet starting at the baseline visit for 10 weeks. Note: Treatment adherence rate was 93.8%±8.0%.  Topiramate group: Subjects received topiramate starting at the baseline visit and the dosage was titrated up over 5 weeks (25 mg per day for 1 week, 25 mg twice daily for 1 week, 50 mg twice daily for 1 week, 75 mg twice daily for 1 week, 100 mg twice daily for 5 weeks). During the last week of the study (Week 10) the dosage was decreased by 30% every 3 days. A quit date was set at 2 weeks after the initiation of topiramate. Note: Treatment adherence rate was 89.4%±14.0%.  Study Start/End Date  April 2006 to December 2008 | Study Population  Adult cigarette smokers  Sample Size  Total: n=57 subjects  Nicotine patch+topiramate group: n=19 subjects  Placebo tablet group: n=19 subjects  Topiramate group: n=19 subjects  Subject Characteristics  *Mean age±SD*  Nicotine patch+topiramate group: 50.1±9.6 yrs  Placebo tablet group: 45.4±7.6 yrs  Topiramate group: 46.1±11.1 yrs  *Sex*  Nicotine patch+topiramate group: female, 68.4%; male, 31.6%  Placebo tablet group: female, 57.9%; male, 42.1%  Topiramate group: female, 52.6%; male, 47.4%  *Race/ethnicity*  Nicotine patch+topiramate group: Caucasian, 84.2%; Hispanic, 10.5%; Other, 5.3%  Placebo tablet group: Caucasian, 84.2%; Hispanic, 10.5%; Other, 5.3%  Topiramate group: Caucasian, 84.2%; Hispanic, 5.3%; Other, 10.5%  *Mean yrs smoked±SD*  Nicotine patch+topiramate group: 30.7±10.3  Placebo tablet group: 28.6±9.9  Topiramate group: 27.3±11.3  *Mean cpd±SD*  Nicotine patch+topiramate group: 20.9±7.2  Placebo tablet group: 22.1±6.9  Topiramate group: 21.1±6.1  Abstinence Characteristics  *eCO-confirmed abstinence rate during the last 4 weeks of treatment (%)*  Nicotine patch+topiramate group: 37% (n=7)  Placebo tablet group: 5% (n=1)  Topiramate group: 26% (n=5) | Arrhythmia  NR  Nonfatal Myocardial Infarction*  Nicotine patch+topiramate group: n=0  Placebo tablet group: n=0  Topiramate group: n=0  Nonfatal Stroke*  Nicotine patch+topiramate group: n=0  Placebo tablet group: n=0  Topiramate group: n=0  Cardiovascular Death*  Nicotine patch+topiramate group: n=0  Placebo tablet group: n=0  Topiramate group: n=0  *No serious AEs (i.e., death, disability, hospitalization, or an important medical event that may jeopardize the subject’s life or require surgical or medical intervention) occurred during the study. |
| 326 | The Preloading Investigators, 2018  Location: UK  Setting: Clinical  Trial Name: NR  Registration/Protocol: NR  Funding: National Institute for Health Research Health Technology Assessment  Design  Open-label, superiority RCT  Eligibility  Inclusion: Age ≥18 yrs; regular smokers of cigarettes, cigars, or roll-up tobacco cigarettes with our without marijuana; suitable for preloading according to researcher’s judgement; seeking support to stop smoking; willing to set a quit day in 4 weeks; able to understand and willing to adhere to study procedures.  Exclusion: Pregnant or breast feeding; skin disorders that precluded use of the patch; acute coronary syndrome or stroke in the past 3 weeks; active phaeocromocytoma or uncontrolled hyperthyroidism that would increase the risk of arrhythmias from the nicotine patch.  Recruitment  GPs spoke or wrote to, emailed, or texted subjects listed as smokers and invited them to join the trial; in one center, a smoking cessation clinic invited subjects seeking treatment to participate in the trial.  Intervention  Nicotine patch group: Subjects were instructed to use a 21 mg per 24 hrs nicotine patch daily for approximately 4 weeks before quit day. Subjects were instructed to smoke as normal and not reduce consumption. Starting 1-2 weeks before quit day, subjects received weekly behavioral support for at least 4 weeks after the quit day. Note: During the first week, three quarters of subjects used the patch daily and four fifths so in did the subsequent weeks. Overall, 5.5% (n=49) of subjects prematurely discontinued preloading.  Control  Control group: Subjects received a behavioral intervention. Subjects were asked to consider their smoking pattern, riggers for use of particular cigarettes, and to plan ways to reduce these cues. Starting 1-2 weeks before quit day, subjects received weekly behavioral support for at least 4 weeks after the quit day. Note: During the first week, three quarters of subjects used the patch daily and four fifths so in did the subsequent weeks. Overall, 5.5% (n=49) of subjects prematurely discontinued preloading.  Study Start/End Date  August 2012 to March 2015 | Study Population  Adult cigarette, cigar, or roll-up tobacco smokers  Sample Size  Total: n=1,792 subjects  Nicotine patch group: n=899 subjects  Control group: n=893 subjects  Subject Characteristics  *Mean age±SD*  Nicotine patch group: 49.1±13.3 yrs  Control group: 48.8±13.4 yrs  *Sex*  Nicotine patch group: men, 52.6% (n=473); women, 47.4% (n=426)  Control group: men, 52.6% (n=469); women, 47.3% (n=422)    *Race/ethnicity*  Nicotine patch group: White British, 75.6% (n=680); White Irish, 2.8% (n=25); White other, 6.1% (n=55); White and Black Caribbean, 1.7% (n=15); White and Black African, 0.6% (n=5);White and Asian, 0.7% (n=6); Mixed other, 0.9% (n=8); Indian, 1.1% (n=10); Pakistani, 0.7% (n=6); Bangladeshi, 1.5% (n=13); Asian other, 0.3% (n=3); Black-Caribbean, 3.8% (n=34); Black African, 1.5% (n=13); Black other, 0.3% (n=3); Chinese, 0.2% (n=2); Other, 1.6 (n=14); More than one option, 0.4% (n=4); Missing, 0.8% (n=7)  Control group: White British, 75.6% (n=675); White Irish, 4.0% (n=36); White other, 6.4% (n=57); White and Black Caribbean, 1.9% (n=17); White and Black African, 0.3% (n=3); White and Asian, 0.9% (n=8); Mixed other, 0.8% (n=7); Indian, 1.2% (n=11); Pakistani, 1.0% (n=9); Bangladeshi, 0.2% (n=2); Asian other, 0.3% (n=3); Black-Caribbean, 3.3% (n=29); Black African, 0.9% (n=8); Black other, 0.5% (n=4); Chinese, 0.3% (n=3); Other, 1.3% (n=12); More than one option, 0% (n=0); Missing, 1.0% (n=9)  *Yrs smoked*  NR  *Mean cpd*±*SD*  Nicotine patch group: 19.1±9.6  Control group: 18.7±9.0  Abstinence Characteristics  NA (subjects continued to smoke during treatment period, i.e., preloading period) | Arrhythmia  NR  Nonfatal Myocardial Infarction  Nicotine patch group: n=1  Control group: n=0  One subject in the nicotine patch group was allocated to preloading but had stopped the medication prematurely 2 days before the myocardial infarction (author correspondence).  Nonfatal Stroke*  Nicotine patch group: n=0  Control group: n=0  Cardiovascular Death*  Nicotine patch group: n=0  Control group: n=0  *Reported serious AEs were listed, none of which were nonfatal myocardial infarction or cardiovascular death. |
| 707 | Ramon et al., 2014  Location: Spain  Setting: Smoking cessation clinic  Trial Name: Clinical Trial to Evaluate the Efficacy of Smoking Cessation (COMBIVAR)  Registration/Protocol: NCT01538394  Funding: NR; grant from Pfizer used for the recruitment and acquisition of active patches.  Design  Randomized, placebo-controlled study.  Eligibility  Inclusion: Age ≥18 yrs; smoking ≥20 cpd for the last 6 months; provided consent to participate; no period of smoking abstinence >3 months in the last yr; not breastfeeding, pregnant, or at risk of becoming pregnant.  Exclusion: Current or past psychotic disorder; history of suicide attempts; not understanding the Spanish language; current or past alcoholism or other drug addictions; used nicotine transdermal patches or varenicline in the last 6 months.  Recruitment  NR  Intervention  Nicotine patch group: Subjects received varenicline (0.5 mg daily for 3 days, then 0.5 mg twice daily for 4 days, followed by 1 mg twice daily for 11 weeks) and nicotine patch (Nicotinell^®^, 21 mg/24 hrs nicotine) for 11 weeks. Note: 1 subject did not use the patch; 1 subject did not use varenicline between Weeks 4 and 12.  Control  Placebo patch group: Subjects received varenicline (0.5 mg daily for 3 days, then 0.5 mg twice daily for 4 days, followed by 1 mg twice daily for 11 weeks) and placebo patches (identical packaging to nicotine patches) for 11 weeks. Note: 2 subjects did not use the patch; all subjects used varenicline.  Study Start/End Date  February 2012 to December 2013 | Study Population  Adult cigarette smokers  Sample Size  Total: n=341 subjects  Nicotine patch group: n=170 subjects  Placebo patch group: n=171 subjects  Subject Characteristics  *Mean age±SD*  Nicotine patch group: 44.1±14.8 yrs  Placebo patch group: 46.2±13.1 yrs  *Sex*  Nicotine patch group: male, 55.9% (n=95); female, 44.1% (n=75)  Placebo patch group: male, 59.6% (n=102); female, 40.4% (n=69)  *Race/ethnicity*  NR  *Yrs smoked*  NR  *Distribution of mean cpd (%)*  Nicotine patch group:  ≤29 cpd: 45.9% (n=78)  >29 cpd: 54.1% (n=92)  Placebo patch group:  ≤29 cpd: 49.1% (n=84)  >29 cpd: 50.9% (n=87)  Abstinence Characteristics  *Continuous abstinence from Week 2-Week 12 with eCO concentrations of <10 ppm at 12 weeks (%)*  Nicotine patch group: 39.1% (n=66)  Placebo patch group: 31.8% (n=54) | Arrhythmia  NR  Nonfatal Myocardial Infarction*  Nicotine patch group: n=0  Placebo patch group: n=0  Nonfatal Stroke*  Nicotine patch group: n=0  Placebo patch group: n=0  Cardiovascular Death*  Nicotine patch group: n=0  Placebo patch group: n=0  *No serious AEs occurred during the study. |
| 391 | Rohsenow et al., 2017  Location: US  Setting: University offices in Providence, RI  Trial Name: Varenicline and Motivational Advice for Smokers With Substance Use Disorders (VARSUD)  Registration/Protocol: NCT00756275  Funding: NIDA and the Department of Veteran Affairs; Brown University (sponsor)  Design  Double blind, randomized, placebo controlled study.  Eligibility  Inclusion: Age 18-75 yrs; substance use disorder diagnosis; enrollment in any outpatient substance use disorder treatment; smoking ≥10 cpd for the past 6 months.  Exclusion: Evidence of hallucinations or delusions; current smoking cessation treatment; contraindications for varenicline or nicotine patch (e.g., pregnancy, uncontrolled hypertension, or severe renal impairment); using medications affected by smoking cessation (antipsychotics, warfarin, theophylline and insulin); suicidal ideation or attempts in past 5 yrs; not willing to try to quit smoking; substance use reported on the day of or before recruitment, or positive breath alcohol at recruitment.  Recruitment  Advertisements.  Intervention  Nicotine patch group: Starting on quit date, subjects received nicotine patches containing 21 mg nicotine per day for 4 weeks, followed by 14 mg per day for 4 weeks, and 7 mg per day for 4 weeks (a total of 12 weeks). Subjects received placebo capsules starting 7 days before quit day, in a parallel manner to the varenicline group. Subjects also received counselling. Note: Subjects used 43.3%±41.0% of nicotine patches 42.4%±39.7% of placebo capsules, and completed 4.2±2.6 counselling sessions.  Control  Varenicline group: Starting 7 days before quit day, subjects received 0.5 mg of varenicline per day for 3 mornings, 1 mg per day (0.5 mg 2 times daily) for the next four days (dose run-up), and 2 mg per day (1.0 mg 2 times daily) thereafter, through 12 weeks of treatment.  Subjects also received placebo patches (Rejuvenation Labs, Inc.) for 12 weeks from quit date, in a parallel manner to the varenicline group. Subjects also received counselling. Note: Subjects used 40.0%±37.5% of placebo patches, 37.4%±35.1% of varenicline capsules, and completed 4.2%±2.4% of counselling sessions.  Study Start/End Date  January 2009 to September 2014 | Study Population  Adult smokers in substance use disorder treatment  Sample Size  Total: n=137 subjects  Nicotine patch group: n=60 subjects  Varenicline group: n=77 subjects  Subject Characteristics  *Mean age±SD*  Nicotine patch group: 39.2±9.7 yrs  Varenicline group: 40.0±10.5 yrs  *Sex (% male)*  Nicotine patch group: 51%  Varenicline group: 55%  *Race/ethnicity*  Nicotine patch group: White/Caucasian, 83%; Black/African American, 15%; Asian/Pacific Islander, 0%; Multi-racial, 2%; Hispanic, 7%  Varenicline group: White/Caucasian, 82%; Black/African American, 16%; Asian/Pacific Islander, 1%; Multi-racial, 1%; Hispanic, 4%  *Yrs smoked*  NR  *Mean cpd±SD*  Nicotine patch group: 18.3±9.3  Varenicline group: 20.4±11.1  Abstinence Characteristics  *Confirmed 7-day smoking abstinence (eCO ≤4 ppm and salivary cotinine ≤15 ng/ml) at 3 months (%)*  Nicotine patch group: 3% (n=2)  Varenicline group: 13% (n=10) | Arrhythmia  NR  Nonfatal Myocardial Infarction*  Nicotine patch group: n=0  Varenicline group: n=0  Nonfatal Stroke*  Nicotine patch group: n=0  Varenicline group: n=0  Cardiovascular Death*  Nicotine patch group: n=0  Varenicline group: n=0  *No serious AEs attributable to medication were observed in the study. |
| 1272 | Rungruanghiranya et al., 2008  Location: Thailand  Setting: Clinical  Trial Name: NR  Registration/Protocol: NR  Funding: Nicotine gum supplied by Srinakharinwirot University  Design  Double-blind, placebo-controlled RCT.  Eligibility  Inclusion: Age ≥18 yrs; smoking an average of ≥10 cpd for the past year; interested in quitting smoking within the next 30 days; only one smoker per household was eligible.  Exclusion: Pregnancy; previous use of other smoking cessation aids in the past month; history or current diagnosis of coronary artery disease; stomach ulcers; uncontrolled hypertension; diabetes mellitus; use of illicit drugs; nonadherence to treatment.  Recruitment  NR  Intervention  Nicotine gum group: Subjects received 2 mg nicotine polyestex gums. Subjects with FTND scores ≥6 received a 4 mg nicotine dose (two pieces of 2 mg gum), and subjects with FTND scores <6 received 2 mg nicotine (one piece of 2 mg gum). Subjects were advised to use the gum whenever they felt an urge to smoke. Subjects set a target quit date but were encouraged to completely stop smoking on the first day of treatment. Individual counseling was also provided approximately 10-15 min at each visit. The treatment period was 12 weeks.  Control  Placebo gum group: Subjects received placebo gum that was identical in appearance to the nicotine gum and was minted, in a parallel manner to the intervention. Individual counseling was also provided approximately 10-15 min at each visit. The treatment period was 12 weeks.  Study Start/End Date  July 2006 to November 2007 (recruitment) | Study Population  Adult cigarette smokers  Sample Size  Total: n=43 subjects  Nicotine gum group: n=20 subjects  Placebo gum group: n=23 subjects  Subject Characteristics  *Mean age±SD*  Nicotine gum group: 45.05±11.83 yrs  Placebo gum group: 43.00±11.86 yrs  *Sex*  Nicotine gum group: male, 100% (n=20); female, 0% (n=0)  Placebo gum group: male, 95.7% (n=22); female, 4.3% (n=1)  *Race/ethnicity*  NR  *Mean yrs smoked±SD*  Nicotine gum group: 26.20±10.60  Placebo gum group: 23.09±9.30  *Mean cpd±SD*  Nicotine gum group: 19.70±5.25  Placebo gum group: 20.78±5.80  Abstinence Characteristics  *eCO-validated (≤10 ppm) total abstinence at end-of-treatment (%)*  Nicotine gum group: 50.0% (n=10)  Placebo gum group: 9.0% (n=2) | Arrhythmia  NR  Nonfatal Myocardial Infarction*  Nicotine gum group: n=0  Placebo gum group: n=0  Nonfatal Stroke*  Nicotine gum group: n=0  Placebo gum group: n=0  Cardiovascular Death*  Nicotine gum group: n=0  Placebo gum group: n=0  *No serious AEs were reported in the study. |
| 910 | Rungruanghiranya et al., 2012  Location: Thailand  Setting: Clinical  Trial Name: NR  Registration/Protocol: NR  Funding: Thai Health Professional Alliance Against Tobacco (ThaiPAT)  Design  Prospective, randomized clinical trial.  Eligibility  Inclusion: Age>18 yrs; smoking regularly ≥1 yr prior to study; desire to quit smoking; signed informed consent.  Exclusion: Current dental problems; active peptic ulcer disease; psychiatric disorders; citrus allergy; pregnancy; use of illicit drugs; participation in another clinical trial; using any first-line smoking cessation aids within 30 days.  Recruitment  Panyananthaphikkhu Chonprathan Medical Center.  Intervention  Nicotine gum group: Subjects received nicotine gum (brand NR) for 12 weeks. Subjects with FTND scores ≥4 received 4 mg nicotine gum and those with FTND scores <4 received 2 mg nicotine gum. Subjects were instructed to use the gum whenever they craved a cigarette but not to exceed 20 pieces per day.  Control  Fresh lime group: Subjects were instructed to use fresh lime whenever they craved cigarettes for a period of 12 weeks.  Study Start/End Date  March 2009 to September 2009 | Study Population  Adult cigarette smokers  Sample Size  Total: n=100 subjects  Nicotine gum group: n=53 subjects  Fresh lime group: n=47 subjects  Subject Characteristics  *Mean age±SD*  Nicotine gum group: 39.75±13.37 yrs  Fresh lime group: 47.23±17.93 yrs  *Sex*  Nicotine gum group: male, 92.5% (n=49); female, 7.5% (n=4)  Fresh lime group: male, 87.2% (n=41); female, 12.8% (n=6)  *Race/ethnicity*  NR  *Mean yrs smoked±SD*  Nicotine gum group: 23.04±12.0  Fresh lime group: 30.83±18.24  *Maximum cpd±SD*  Nicotine gum group: 17.8±9.1  Fresh lime group: 12.4±6.2  Abstinence Characteristics  *Continuous abstinence rate from week 9 to week 12 (eCO≤10 ppm) (%)*  Nicotine gum group: 66.0% (n=35)  Fresh lime group: 61.7% (n=29) | Arrhythmia  NR  Nonfatal Myocardial Infarction*  Nicotine gum group: n=0  Fresh lime group: n=0  Nonfatal Stroke*  Nicotine gum group: n=0  Fresh lime group: n=0  Cardiovascular Death*  Nicotine gum group: n=0  Fresh lime group: n=0  *No serious AEs occurred during the study. |
| 1910 | Sachs et al., 1993  Location: US  Setting: Physician’s office  Trial Name: NR  Registration/Protocol: NR  Funding: NIDA; Kabi Pharmacia AB; Parke-Davis  Design  Randomized, double-blind, placebo-controlled trial.  Eligibility  Inclusion: Age ≥18 yrs; smoking ≥10 cpd for ≥3 yrs; motivated to stop smoking; in good health.  Exclusion: Severe or symptomatic CVD; pregnancy or breastfeeding; current regular use of psychotropic medications; current or past alcohol or other drug abuse; current use of smokeless tobacco; chronic dermatological disorders (e.g., psoriasis, urticarial, or chronic dermatitis).  Recruitment  Newspaper and television notices.  Intervention  Nicotine patch group: Subjects received 30 cm^2^ nicotine patches releasing 15±3.5 mg of nicotine per 16 hrs for 12 weeks, followed by 20 cm^2^ patches for 3 weeks, then 10 cm^2^ patches for 3 weeks. Total treatment duration was 18 weeks. Note: Compliance was 62% at Week 12 (end of treatment) and 60% at Week 18 (end of tapering).  Control  Placebo patch group: Subjects received placebo patches, which were identical to the active patches in appearance, packaging, and labelling, but contained no nicotine. Note: Compliance was 54% at Week 12 (end of treatment) and 61% at Week 18 (end of tapering).  Study Start/End Date  January 1990 to April 1990 | Study Population  Adult cigarette smokers  Sample Size  Total: n=220 subjects  Nicotine patch group: n=113 subjects  Placebo patch group: n=107 subjects  Subject Characteristics  *Mean age (SD, range)*  Nicotine patch group: 47.5±10.7 (24-69) yrs  Placebo patch group: 47.8±10.8 (25-71) yrs  *Sex*  Nicotine patch group: male, 41%; female, 59%  Placebo patch group: male, 41%; female, 59%  *Race/ethnicity*  NR  *Mean yrs smoked±SD (range)*  Nicotine patch group: 28.7±10.5 (5-51)  Placebo patch group: 28.5±11.1 (6-50)  *Mean cpd±SD (range):*  Nicotine patch group: 27.3±9.5 (10-60)  Placebo patch group: 28.8±11.1 (10-70)  Abstinence Characteristics  *Continuous abstinence (eCO ≤9 ppm at each visit) Week 2‑Week 12 (%)*  Nicotine patch group: 45% (n=51)  Placebo patch group: 26% (n=28) | Arrhythmia  NR  Nonfatal Myocardial Infarction  Nicotine patch group: n=0  Placebo patch group: n=0  Nonfatal Stroke  NR  Cardiovascular Death  NR |
| 1820 | Sandborn et al., 1997  Location: US  Setting: Clinical  Trial Name: NR  Registration/Protocol: NR  Funding: Elan Pharmaceutical Research Corp  Design  Randomized, double-blind, placebo-controlled trial. Nicotine was used to treat active ulcerative colitis.  Eligibility  Inclusion: Nonsmokers or former smokers.  Exclusion: Used products containing nicotine in the last 3 months; receiving corticosteroids >20 mg per day; using cyclosporine, 6‑mercaptopurine, azathioprine, or methotrexate.  Recruitment  Subjects with mildly to moderately active ulcerative colitis were referred by colleagues at the Mayo Clinic for study enrollment.  Intervention  Nicotine patch group: Subjects received a small 11 mg nicotine patch (Elan Pharmaceutical Research Corp) per day for 7 days and a large 21 mg nicotine patch for the following 21 days.  Control  Placebo patch group: Subjects received non-nicotine patches (Elan Pharmaceutical Research Corp) identical in appearance to the nicotine patches.  Study Start/End Date  April 1993 to September 1995 | Study Population  Adult nonsmokers with mildly to moderately active ulcerative colitis  Sample Size  Total: n=64 subjects  Nicotine patch group: n=31 subjects  Placebo patch group: n=33 subjects  Subject Characteristics  *Median age (range)*  Nicotine patch group: 38 (17-77) yrs  Placebo patch group: 45 (22-70) yrs  *Sex*  Nicotine patch group: male, 39%; female, 61%  Placebo patch group: male, 39%; female, 61%  *Race/ethnicity*  NR  *Mean yrs smoked*  NA  *Mean cpd*  NA  Abstinence Characteristics  NA | Arrhythmia*  Nicotine patch group: n=3  Placebo patch group: n=0  *Reported as tachycardia  Nonfatal Myocardial Infarction  NR  Nonfatal Stroke  NR  Cardiovascular Death  NR |
| 1279 | Shiffman and Ferguson, 2008  Location: US  Setting: Community  Trial Name: NR  Registration/Protocol: NR  Funding: SmithKline Beecham, the corporate predecessor of GlaxoSmithKline Consumer Healthcare  Design  Secondary analysis of a double-blind, randomized, clinical trial. Note: This article reports on the results of two studies; however, only one study fits the inclusion criteria of this review and is therefore the only study summarized.  Eligibility  Inclusion: Age 18–65 yrs; smoking for ≥3 yrs; currently smoking ≥15 cpd; no use of tobacco products other than cigarettes; did not work at night or sleep during normal waking hrs; high motivation and efficacy for quitting smoking.  Exclusion: Not in good health; lactating; pregnant or likely to become pregnant; using psychoactive drugs; recent history of alcohol or drug abuse; history of severe mental illness; allergies to adhesives.  Recruitment  Subjects were recruited through advertising in local media, and were paid $150 for their participation.  Intervention  Nicotine patch group: Subjects received nicotine patch (21 mg per 24 hrs, GlaxoSmithKline) daily for a period of up to 2 weeks. Subjects replaced the patch every morning after waking. Subjects also received 10–15 min of behavioral counseling at each study visit.  Control  Placebo patch group: Subjects received placebo patch identical in appearance to the nicotine patch daily for up to 2 weeks. Subjects replaced the patch every morning after waking. Subjects also received 10–15 min of behavioral counseling at each study visit.  Study Start/End Date  1996 to 1997 | Study Population  Adult cigarette smokers  Sample Size  Total: n=102 subjects  Nicotine patch group: n=55 subjects  Placebo patch group: n=47 subjects  Subject Characteristics  *Mean age±SD*  Nicotine patch group: 38.8±7.9 yrs  Placebo patch group: 39.5±12.0 yrs  *Sex (% female)*  Nicotine patch group: 63.6%  Placebo patch group: 55.3%  *Race/ethnicity*  Nicotine patch group: Caucasian, 87.3%; African-American, 9.1%; Other, 3.6%  Placebo patch group: Caucasian, 93.6%; African-American, 4.3%; Other, 2.1%  *Mean yrs smoked±SD*  Nicotine patch group: 20.0±8.3  Placebo patch group: 19.6±11.9  *Mean cpd±SD*  Nicotine patch group: 22.6±7.6  Placebo patch group: 24.3±8.2  Abstinence Characteristics  NR | Arrhythmia  NR  Nonfatal Myocardial Infarction*  Nicotine patch group: n=0  Placebo patch group: n=0  Nonfatal Stroke*  Nicotine patch group: n=0  Placebo patch group: n=0  Cardiovascular Death*  Nicotine patch group: n=0  Placebo patch group: n=0  *No serious AEs were reported in the study. |
| 1247 | Shiffman et al., 2009  Location: US  Setting: Community  Trial Name: NR  Registration/Protocol: NR  Funding: SmithKline Beecham Consumer Healthcare (currently GalaxoSmithKline Consumer Healthcare)  Design  Multi-center, placebo-controlled, double-blind RCT. [Note: AE data only reported for initial 8-week phase of study, i.e. when attempting to achieve abstinence.]  Eligibility  Inclusion: Interest in quitting smoking using gradual reduction within 30 days; ≥18 yrs old; ability to understand English; capable of providing written informed consent.  Exclusion: Use of other nicotine-containing products, bupropion or NRT within 30 days of study entry; smoking any substance other than cigarettes within 30 days of study entry; history of heart disease; recent heart attack or irregular heartbeat; insulin-dependent diabetics; involvement in another clinical study; being in the same household as another subject or being related to study site staff; working during the night hrs; unable to attend visits before 2:00 PM; pregnancy or likelihood of becoming pregnant during the study.  Recruitment  Print and radio advertisements.  Intervention  Nicotine gum (2 mg) group: Subjects self-selected the study gum dose of 2 mg after reviewing the labels, which recommended that smokers of <25 cpd select the 2 mg dose. Subjects were instructed to start reducing smoking by not smoking for the first hr of the day and to instead use a single piece of gum during that hr. Abstinence was extended by an additional hr on each subsequent day, using one additional piece of gum. Once 24hr abstinence was achieved, subjects were instructed to use one piece of gum every 1-2 hrs for the first 6 weeks, followed by one piece every 2-4 hrs for the next 3 weeks, and one piece every 4-8 hrs for the final 3 weeks. Subjects were then permitted to use the gum as needed to stay smoke-free for an additional 12 weeks.  Nicotine gum (4 mg) group: Subjects self-selected the study gum dose of 4 mg after reviewing the labels, which recommended that smokers of ≥25 cpd select the 4 mg dose, along with the same instructions as described for the nicotine (2 mg) group.  Control  Placebo gum (2 mg) group: Subjects received placebo gum with same instructions as described for the intervention.  Placebo gum (4 mg) group: Subjects received placebo gum with same instructions as described for the intervention.  Study Start/End Date  NR | Study Population  General adult population  Sample Size  Total: n=3,297 subjects  Nicotine gum (2 mg) group: n=819 subjects  Placebo gum (2 mg) group: n=817 subjects  Nicotine gum (4 mg) group: n=830 subjects  Placebo gum (4 mg) group: n=831 subjects  Subject Characteristics  *Mean age±SD*  Nicotine gum (2 mg) group: 42.1±13.0 yrs  Placebo gum (2 mg) group: 42.2±13.3 yrs  Nicotine gum (4 mg) group: 46.1±11.3 yrs  Placebo gum (4 mg) group: 46.3±11.4 yrs  *Sex (% female)*  Nicotine gum (2 mg) group: 62.8%  Placebo gum (2 mg) group: 65.5%  Nicotine gum (4 mg) group: 46.1%  Placebo gum (4 mg) group: 46.3%  *Race/ethnicity*  Nicotine gum (2 mg) group: Caucasian, 84.5%; Black, 8.9%; Asian, 1.0%; Hispanic, 4.3%; Other, 1.3%  Placebo gum (2 mg) group: Caucasian, 83.2%; Black, 8.2%; Asian, 1.7%; Hispanic, 5.0%; Other, 1.8%  Nicotine gum (4 mg) group: Caucasian, 90.8%; Black, 5.7%; Asian, 0.7%; Hispanic, 1.7%; Other, 1.1%  Placebo gum (4 mg) group: Caucasian, 91.0%; Black, 4.8%; Asian, 0.5%; Hispanic, 2.5%; Other, 1.2%  *Mean yrs smoked±SD*  Nicotine gum (2 mg) group: 24.1±12.8  Placebo gum (2 mg) group: 24.3±13.6  Nicotine gum (4 mg) group: 29.2±11.3  Placebo gum (4 mg) group: 29.5±11.7  *Mean cpd±SD*  Nicotine gum (2 mg) group: 17.7±6.0  Placebo gum (2 mg) group: 17.6±6.1  Nicotine gum (4 mg) group: 32.0±9.6  Placebo gum (4 mg) group: 32.5±10.0  Abstinence Characteristics  *Abstinence at 28 days (%)*  Nicotine gum (2 mg and 4 mg combined) group: 10.3%  Control gum (2 mg and 4 mg placebo combined) group: 3.9% | Arrhythmia  Nicotine gum (2 mg and 4 mg combined) group: 0.4% (n=NR)  Placebo gum (2 mg and 4 mg combined) group: 0.4% (n=NR)  Reported as heart rate and rhythm disorders occurring during the first 8 weeks.  Nonfatal Myocardial Infarction  NR  Nonfatal Stroke  NR  Cardiovascular Death  NR |
| 1447 | Shiffman et al., 2006  Location: US  Setting: Community  Trial Name: NR  Registration/Protocol: NR  Funding: NIDA; nicotine and placebo patches were provided by GlaxoSmithKline Consumer Healthcare  Design  Randomized, double-blind, placebo-controlled clinical trial.  Eligibility  Inclusion: Age 21-65 yrs; smoking ≥15 cpd for 5 yrs; in good health; high motivation and confidence to quit smoking.  Exclusion: Regular use of non-cigarette tobacco products; weight <110 lbs; contraindications to nicotine patch use; serious medical illness; history of recent alcohol and/or drug abuse; history of mental illness; current participation in a smoking cessation clinic or study; recent participation in a clinical trial for smoking cessation; use of bupropion hydrochloride within the past 2 months; women planning to become pregnant or currently breastfeeding.  Recruitment  Subjects were recruited via TV, radio, print advertising, and fliers.  Intervention  Nicotine patch group: Subjects wore two nicotine patches daily, totaling 35 mg per day, for the first 3 weeks: a 22 cm^2,^ 21 mg nicotine patch (NicoDerm^®^ CQ^®^) and a 15cm^2^, 14 mg nicotine patch. Subjects wore two nicotine patches daily, totaling 21 mg per day, for the subsequent 2 weeks: a 22 cm^2^ 21 mg nicotine patch and a 15cm^2^ placebo patch. Subjects wore two patches totaling 0 mg for one final week: a 22 cm^2^ patch and a 15 cm^2^ patch, both inactive. Subjects also received cognitive behavioral treatment. Note: Patches were applied on 99.3% of days. There were no differences in compliance between subjects on active and placebo patch.  Control  Placebo patch group: Subjects wore two placebo patches (a 22 cm^2^ placebo patch and a 15cm^2^ placebo patch) daily, in a parallel manner as described in the intervention. Subjects also received cognitive behavioral treatment. Note: Patches were applied on 99.3% of days. There were no differences in compliance between subjects on active and placebo patch.  Study Start/End Date  October 1997 to February 2000 | Study Population  Adult cigarette smokers  Sample Size  Total: n=324 subjects  Nicotine patch group: n=188 subjects  Placebo patch group: n=136 subjects  Subject Characteristics  *Mean age±SE*  Nicotine patch group: 40.02±0.68 yrs  Placebo patch group: 38.90±0.78 yrs  *Sex*  Nicotine patch group: male, 47.0% (n=87); female, 53% (n=101)  Placebo patch group: male, 52.20% (n=71); female, 47.80% (n=65)  *Race/ethnicity*  Nicotine patch group (%): Caucasian, 84.50% (n=153); African American, 11.10% (n=20); Hispanic, 0.60% (n=1); Native American, 1.70% (n=3); Other, 2.20% (n=4)  Placebo patch group: Caucasian, 86.70% (n=117); African American, 11.10% (n=15); Hispanic, 0% (n=0); Native American, 1.50% (n=2); Other, 0.70% (n=1)  *Mean yrs smoked±SE*  Nicotine patch group: 22.77±0.71  Placebo patch group: 21.04±0.78  *Mean cpd±SE*  Nicotine patch group: 24.65±0.65  Placebo patch group: 23.79±0.76  Abstinence Characteristics  *7-day PPA rate at Week 5 (%):*  Nicotine patch group: 52% (n=98)  Placebo patch group: 26% (n=35) | Arrhythmia  NR  Nonfatal Myocardial Infarction*  Nicotine patch group: n=0  Placebo patch group: n=0  Nonfatal Stroke*  Nicotine patch group: n=0  Placebo patch group: n=0  Cardiovascular Death*  Nicotine patch group: n=0  Placebo patch group: n=0  *There were no serious AEs. |
| 1065 | Stapleton and Sutherland, 2011  Location: England  Setting: Clinical  Trial Name: Nicotine nasal spray for smoking cessation in primary care  Registration/Protocol: UK Clinical Trials (ISRCTN03150596)  Funding: NR  Design  Double-blind, placebo-controlled, multi-center (27 general practices) trial  Eligibility  Inclusion: Age 20-60 yrs; good general health; smoking ≥15 cpd for ≥3 yrs; well motivated to quit smoking (as determined by GP).  Exclusion: myocardial infarction within last 3 yrs; chronic nasal disorder; pregnancy; intending pregnancy; breastfeeding; currently under psychiatric care or using psychotropic medication.  Recruitment  General practice clinics (each clinic permitted to recruit a maximum of 60 subjects).  Intervention  Nicotine nasal spray group: Subjects received nicotine nasal spray (Pharmacia/McNeil AB, Stockholm, Sweden) for 12 weeks to use ad libitum. Each spray delivered 0.5 mg nicotine per 50 ml; a single 1 mg dose comprised two sprays, one to each nostril. Two bottles were provided per week, allowing a maximum average daily dose of 28 mg nicotine. For Weeks 11-12, subjects were provided with one bottle per week. Subjects were advised to use spray at each smoking urge, up to a maximum of three doses per hr or 32 doses per day. Subjects were also encouraged to use at least one dose per hr from the first day to speed the acquisition of tolerance to the irritant effects.  Control  Placebo nasal spray group: Subjects received placebo nasal spray containing black pepper oleo resin (piperine) to mimic the sensation of nicotine for 12 weeks to use ad libitum. Two bottles were provided per week; for Weeks 11-12, subjects were provided with one bottle per week. Subjects were advised to use spray whenever at each smoking urge, up to a maximum of three doses per hr or 32 doses per day. Subjects were also encouraged to use at least one dose per hr from the first day to speed the acquisition of tolerance to the irritant effects.  Study Start/End Date  February 1996 to February 1999 | Study Population  Healthy adult cigarette smokers  Sample Size  Total: n=761 subjects  Nicotine nasal spray group: n=506 subjects  Placebo nasal spray group: n=255 subjects  *Subject Characteristics*  *Mean age±SD*  Nicotine nasal spray group: 40.1±10.5 yrs  Placebo nasal spray group: 40.9±10.0 yrs  *Sex (% male)*  Nicotine nasal spray group: 45.3% (n=229)  Placebo nasal spray group: 37.5% (n=96)  Race/ethnicity  NR  *Yrs smoked*  NR  *Mean cpd±SD*  Nicotine nasal spray group: 22.7±8.2  Placebo nasal spray group: 24.0±8.1  Abstinence Characteristics  *eCO-verified abstinence from Weeks 3 through 12 (%)*  Nicotine nasal spray group: 15.4% (n=78)  Placebo nasal spray group: 6.7% (n=17) | Arrhythmia  NR  Nonfatal Myocardial Infarction  NR  Nonfatal Stroke  NR  Cardiovascular Death  Nicotine nasal spray group: n=0  Placebo nasal spray group: n=0 |
| 810 | Stein et al., 2013  Location: US  Setting: Clinical  Trial Name: Varenicline or Nicotine Patch and Nicotine Gum in Helping Smokers in a Methadone Treatment Program Stop Smoking  Registration/Protocol: NCT00790569  Funding: National Cancer Institute and the NIDA; sponsored by Butler Hospital, RI, US  Design  Randomized, placebo-controlled, double-blind clinical trial.  Eligibility  Inclusion: Age >18 yrs; smoking >10 cpd for the past 3 months; participated in a methadone maintained treatment program; interested in quitting smoking.  Exclusion: smoked<10 cpd, had been methadone-maintained for <4 weeks; pregnant or nursing; unwilling to set a smoking quit date in the next week.  Recruitment  Recruited at the methadone maintenance treatment program sites.  Intervention  NRT group: Subjects received Nicoderm patch (GlaxoSmithKline) at 4-week intervals for up to 24 weeks of therapy. For subjects who smoked >30 cpd the treatment began at 42 mg nicotine, and for subjects smoking <30 cpd the treatment began at 21 mg nicotine.  In addition to using a daily nicotine patch, subjects received a 4-week supply of 4 mg nicotine gum (Nicorette^®^) at the baseline visit. Subjects were instructed to chew gum when experiencing cravings, and felt that they were likely to restart smoking. Subjects were instructed to chew up to 1–2 pieces of gum per hour but no more than 24 pieces of gum per day. Subjects were provided refills of the nicotine gum at their request at any time during the treatment phase. Note: Adherence to treatment during 7 days immediately prior to the 6-month assessment was 48.8%.  Control  Varenicline group: Subjects received 0.5 mg varenicline once per day starting at the baseline visit for 3 days, followed by 0.5 mg twice per day for 4 days, followed by 1 mg twice daily after 1 week (as per the varenicline package insert from Pfizer, Inc.). Varenicline was dispensed at 4-week intervals for up to a 24-week course of therapy. Subjects were instructed to take varenicline for 1 week before attempting to quit smoking on day 8 of the study. Note: Adherence to treatment during 7 days immediately prior to the 6-month assessment was 34.2%.  Varenicline-placebo group: Subjects received 24 weeks of placebo tablets (identical in appearance to varenicline capsules) using an identical dosing and dispensing schedule as the active varenicline group. Note: Adherence to treatment during 7 days immediately prior to the 6-month assessment was 34.4%.  Study Start/End Date  December 2008 to October 2012 | Study Population  Cigarette smokers who are methadone-maintained  Sample Size  Total: n=315 subjects  NRT group: n=133 subjects  Varenicline group: n=137 subjects  Varenicline-placebo group: n=45 subjects  Subject Characteristics  *Mean age±SD*  NRT group: 40.3±9.3 yrs  Varenicline group: 39.2±9.7 yrs  Varenicline-placebo group: 40.6±10.6 yrs  *Sex (% male)*  NRT group: 48.9% (n=65)  Varenicline group: 46.0% (n=63)  Varenicline-placebo group: 62.2% (n=28)  *Race/ethnicity*  NRT group: Non-Hispanic White, 77.4% (n=103); African-American, 2.3% (n=3); Hispanic, 12.8% (n=17); Other, 7.5% (n=10)  Varenicline group: Non-Hispanic White, 82.5% (n=113); African-American, 1.5% (n=2); Hispanic, 12.4% (n=17); Other, 3.7% (n=5)  Varenicline-placebo group: Non-Hispanic White, 75.6% (n=34); African-American, 6.7% (n=3); Hispanic, 8.9% (n=4); Other, 8.9% (n=4)    *Mean yrs regular smoker±SD*  NRT group: 24.0±9.8  Varenicline group: 22.8±10.1  Varenicline-placebo group: 23.0±11.3  *Mean cpd±SD*  NRT group: 19.1±7.8  Varenicline group: 19.5±8.5  Varenicline-placebo group: 21.1±10.4  Abstinence Characteristics  *eCO verified (<8 ppm) 7-day PPA immediately prior to Month 6 assessment (%)*  NRT group: 8.3% (n=11)  Varenicline group: 3.7% (n=5)  Varenicline-placebo group: 2.2% (n=1)  *Continuous abstinence from Day 14 through the 6-month assessment (%)*  NRT group: 1.5% (n=2)  Varenicline group: 1.5% (n=2)  Varenicline-placebo group: 0.0% (n=0) | Arrhythmia  NR  Nonfatal Myocardial Infarction  NRT group: n=1  Varenicline group: n=0  Varenicline-placebo group: n=0  Nonfatal Stroke*  NRT group: n=0  Varenicline group: n=0  Varenicline-placebo group: n=0  *Three serious AEs were reported, none of which were nonfatal stroke.  Cardiovascular Death  NR |
| 1253 | Sun et al., 2009  Location: China  Setting: Community  Trial Name: NR  Registration/Protocol: NR  Funding: National High Technology Research and Development Program of China, China–Canada Joint Health Research Program, and NIH  Design  Randomized, double-blind, placebo-controlled clinical trial.  Eligibility  Inclusion: Motivated to stop smoking; age 18–65 yrs; smoking ≥10 cpd; tested positive for urine cotinine concentration; smoked for at least 1 yr.  Exclusion: A history of any psychiatric disorder listed in the DSM-IV alcohol abuse, and other drug abuse; allergies; pathological changes to the floor of the mouth; cardiovascular or cerebrovascular disease; use of any other NRT products during the last 6 months; pregnant or breast-feeding.  Recruitment  Recruited by newspaper advertisements.  Intervention  Nicotine tablet group: Subjects received one-on-one behavioral counseling and sublingual nicotine tablets (2 mg nicotine; Zhenke, Beijing Zesheng Biotechnology Institute). For the first 4 days of the first week, the highly dependent smokers (FNTD scores ≥7) used 2 tablets (4 mg of nicotine) per hr, and less dependent smokers (FNTD scores <7) used 1 tablet per hr, up to a maximum of 20 tablets per day. All subjects were advised to use the full recommended treatment dose for 1 month, and then gradually reduce the number of tablets used until they stopped using the tablets at the end of the second month. During the third month, they received no intervention. The total treatment period was 12 weeks.  Control  Placebo tablet group: Subjects received one-on-one behavioral counseling and placebo tablets identical in appearance to the active tablet but containing only capsaicin (3 μg). The total treatment period was 12 weeks.  Study Start/End Date  March 2004 to September 2004 | Study Population  Adult Han Chinese cigarette smokers  Sample Size  Total: n=211 subjects  Nicotine tablet group: n=101 subjects  Placebo tablet group: n=110 subjects  Subject Characteristics  *Mean age±SD*  Nicotine tablet group: 43.22±11.42 yrs  Placebo tablet group: 39.63±11.30 yrs  *Sex*  Nicotine tablet group: male, n=94; female, n=7  Placebo tablet group: male, n=106; female, n=4  *Race/ethnicity*  NA  *Mean yrs smoked±SD*  Nicotine tablet group: 23.49±10.58  Placebo tablet group: 20.66±11.23  *Mean cpd±SD*  Nicotine tablet group: 23.98.0±10.41  Placebo tablet group: 23.55±9.98  Abstinence Characteristics  *Self-reported abstinence at end-of-treatment (3 months) (%)*  Nicotine tablet group: 52.0%  Placebo tablet group: 19.0% | Arrhythmia  NR  Nonfatal Myocardial Infarction*  Nicotine tablet group: n=0  Placebo tablet group: n=0  Nonfatal Stroke*  Nicotine tablet group: n=0  Placebo tablet group: n=0  Cardiovascular Death*  Nicotine tablet group: n=0  Placebo tablet group: n=0  *No serious AEs were reported in the study. |
| 1869 | Thomas et al., 1995  Location: UK  Setting: Clinical  Trial Name: NR  Registration/Protocol: NR  Funding: Kabi Pharmacia  Design  Randomized, double-blind, placebo-controlled study.  Note: The objective of nicotine treatment was to maintain remission of ulcerative colitis after tapering treatment with mesalamine.  Eligibility  Inclusion: Age 18-70 yrs; diagnosis of ulcerative colitis; in clinical remission.  Exclusion: Evidence of active disease on sigmoidoscopy or recent symptoms; had taken medication other than mesalamine for the maintenance of remission in the previous 4 weeks; other medical problems, particularly of the cardiovascular system; pregnant; lactating; previously received nicotine therapy for colitis; current smokers; declined to participate.  Recruitment  Departmental registers.  Intervention  Nicotine patch group: Subjects received nicotine patches and were instructed to apply them in the morning and remove them at bedtime. The dose of nicotine was increased by 2.5 mg every 3 days until a maintenance dose of 15 mg daily was achieved. If subjects did not tolerate 15 mg nicotine daily, the maximal amount tolerated was used for the remainder of the treatment period. Total treatment period was 6 months.  Control  Placebo patch group: Subjects received placebo patches. The nicotine and placebo patches were identical in appearance, and the size of the patches was increased over a period of 18 days in an identical fashion in the 2 groups. Total treatment period was 6 months.  Study Start/End Date  NR | Study Population  Adult cigarette non-smokers with ulcerative colitis  Sample Size  Total: n=80 subjects  Nicotine patch group: n=40 subjects  Placebo patch group: n=40 subjects  Subject Characteristics  *Mean age±SD*  Nicotine patch group: 42±12 yrs  Placebo patch group: 40±9 yrs  *Sex*  Nicotine patch group: male, n=22; female, n=18  Placebo patch group: male, n=18; female, n=22  *Race/ethnicity*  NR  *Yrs smoked*  NA  *Average cpd*  NA  Abstinence Characteristics  NA | Arrhythmia  Nicotine patch group: n=1  Placebo patch group: n=0  Bradycardia occurred one subject in the nicotine patch group, a 40 yr old woman with influenza-like symptoms after 6 weeks of treatment. Subsequent investigation showed a nodal bradycardia for which no cause could be found. Because the bradycardia did not revert to sinus rhythm when treatment with nicotine was stopped, a permanent pacemaker was implanted, and the subject subsequently made a full recovery.  Nonfatal Myocardial Infarction*  Nicotine patch group: n=0  Placebo patch group: n=0  Nonfatal Stroke*  Nicotine patch group: n=0  Placebo patch group: n=0  Cardiovascular Death*  Nicotine patch group: n=0  Placebo patch group: n=0  *Serious AEs were listed and did not include nonfatal myocardial infarction, nonfatal stroke, or death. |
| 1104 | Thomsen et al., 2010  Location: Denmark  Setting: Clinical  Trial Name: Brief Smoking Intervention for Women Undergoing Breast Cancer Surgery  Registration/Protocol: NCT00299117  Funding: The Lundbeck Foundation; Doctor Fritz Karner and wife Edith Karner’s Foundation; Aase og Einar Danielsen’s Foundation; The Research Council at Herlev University Hospital; Civil Engi­neer Bent Bøgh and Wife Inge Bøgh’s Foundation; The Tryg Foundation NRT donated by McNeil Denmark ApS  Design  RCT  Eligibility  Inclusion: daily cigarette smokers; age ≥18 yrs; sufficient language proficiency.  Exclusion: Weekly alcohol intake >35 units; substance abuse; diagnosed psychiatric disease; dementia; American Society of Anesthesiologists’ physical status classification class >3; penetrating cancer; pregnancy and lactation; preoperative neoadjuvant chemotherapy.  Recruitment  Subjects scheduled for breast cancer surgery were recruited for the study.  Intervention  NRT group: Subjects received a 45-90 min counseling session 3 to 7 days before surgery. Personalized NRT (brand and dose NR) was offered free of charge for the recommended perioperative smoking cessation period (3 to 7 days preoperative and 10 days postoperative). NRT was administered in accordance with subject preferences and dependency assessed by the FTND score. Complete smoking cessation was recommended from 2 days before to 10 days after surgery.  Control  Control group: Subjects received routine preoperative information. This entailed inconsistent or no advice about the risks of smoking in relation to surgery.  Study Start/End Date  April 2006 to December 2008 | Study Population  Women with breast cancer who were cigarette smokers  Sample Size  Total: n=120 subjects  NRT group: n=58 subjects  Control group: n=62 subjects  Subject Characteristics  *Median age (range)*  NRT group: 57.5 (35-79) yrs  Control group: 56.5 (36-82) yrs  *Sex*  NA  *Race/ethnicity*  NR  *Median pack yrs (range)*  NRT group: 31.5 (8.8-64.5)  Control group: 27 (3.8-81)  *Average cpd*  NR  Abstinence Characteristics  *Continuous abstinence from 2 days before to 10 days after surgery (%)*  NRT group: 28.0% (n=16)  Control group: 11.0% (n=7) | Arrhythmia*  NR  Nonfatal Myocardial Infarction*  NR  Nonfatal Stroke*  NR  Cardiovascular Death  NRT group: n=0  Control group: n=0  *Authors reported “cardiovascular complications” without specifying what those complications were. |
| 1779 | Tønnesen et al., 1999  Location: Multinational (Austria; Belgium; Denmark; Finland; France; Germany; Greece; Ireland; Italy; Netherlands; Norway; Portugal; Spain; Sweden; Switzerland; UK)  Setting: Clinical  Trial Name: NR  Registration/Protocol: NR  Funding: NR  Design  Randomized, placebo-controlled, double-blind trial.  Eligibility  Inclusion: Smokers 20-70 yrs smoking >14 cpd for ≥3 yrs; motivated to stop smoking; made at least 1 prior quit attempt.  Exclusion: Myocardial infarction in the previous 3 months; unstable angina; severe cardiac arrhythmia; pregnant or lactating females; under psychiatric care or medication; alcohol or drug abuse; eczema; severe or malignant disease; existing use of nicotine substitution products and/or participation in formal smoking cessation programs in the last 6 months.  Recruitment  Advertisements in local newspapers, local radio, press conferences, wall posters in public areas of the hospital, or existing databases from previous recruitment to smoking cessation programs.  Intervention  Nicotine (25 mg long duration) group: Subjects received a 25 mg nicotine dose (15 mg plus 10 mg patches) for 22 weeks, followed by a 15 mg dose (15 mg plus placebo patches) for 2 weeks, and a 10 mg dose (10 mg plus placebo patch) for 2 weeks. Note: At Week 26, 52% (n=291) of subjects were using the patch every day.  Nicotine (25 mg short duration) group: Subjects received a 25 mg dose of nicotine (15 mg plus 10 mg patches) for 8 weeks, followed by a 15 mg dose (15 mg plus placebo patches) for 2 weeks, a 10 mg dose (10 mg plus placebo patch) for 2 weeks, and a 0 mg dose (two placebo patches) for 14 weeks. Note: At Week 26, 47% (n=290) of subjects were using the patch every day.  Nicotine (15 mg long duration) group: Subjects received a 15 mg nicotine dose (15 mg plus placebo patches) for 22 weeks, followed by a 10 mg dose (10 mg plus placebo patches) for 4 weeks. Note: At Week 26, 53% (n=274) of subjects were using the patch every day.  Nicotine (15 mg short duration) group: Subjects received a 15 mg nicotine dose (15 mg plus placebo patches) for 8 weeks, followed by a 10 mg dose (10 mg plus placebo patches) for 4 weeks, and 0 mg dose (two placebo patches) for 14 weeks. Note: At Week 26, 45% (n=222) of subjects were using the patch every day.  Control  Placebo group: Subjects received placebo patches, identical in appearance and packaging to nicotine patches for 26 weeks, and were instructed to wear two patches simultaneously for a total of 26 weeks. Note: At Week 26, 51% (n=186) of subjects were using the placebo patch every day.  Study Start/End Date  January 1994 to November 1995 | Study Population  General adult population  Sample Size  Total: n=3,575 subjects  Nicotine (25 mg long duration) group: n=715 subjects  Nicotine (25 mg short duration) group: n=715 subjects  Nicotine (15 mg long duration) group: n=715 subjects  Nicotine (15 mg short duration) group: n=716 subjects  Placebo group: n=714 subjects  Subject Characteristics  *Mean age±SD*  Nicotine (25 mg long duration) group: 40±10 yrs  Nicotine (25 mg short duration) group: 41±10 yrs  Nicotine (15 mg long duration) group: 40±10 yrs  Nicotine (15 mg short duration) group: 41±10 yrs  Placebo group: 41±10 yrs  *Sex (% male)*  Nicotine (25 mg long duration) group: 52%  Nicotine (25 mg short duration) group: 53%  Nicotine (15 mg long duration) group: 52%  Nicotine (15 mg short duration) group:51%  Placebo group: 52%  *Race/ethnicity*  NR  *Mean age at initiation of smoking±SD*  Nicotine (25 mg long duration) group: 17±4 yrs  Nicotine (25 mg short duration) group: 17±4 yrs  Nicotine (15 mg long duration) group: 17±4 yrs  Nicotine (15 mg short duration) group: 17±4 yrs  Placebo group: 17±4 yrs    *Mean cpd±SD*  Nicotine (25 mg long duration) group: 28±11  Nicotine (25 mg short duration) group: 26±9  Nicotine (15 mg long duration) group: 26±10  Nicotine (15 mg short duration) group: 27±10  Placebo group: 27±10  Abstinence Characteristics  *Continuous abstinence at Week 8 (%)*  Nicotine (25 mg long duration) group: 36.9% (n=264)  Nicotine (25 mg short duration) group: 40.8% (n=292)  Nicotine (15 mg long duration) group: 32.5% (n=232)  Nicotine (15 mg short duration) group: 29.2% (n=209)  Placebo group: 20.7% (n=148) | Arrhythmia  NR  The study reported tachycardia, but combined it with heart palpitations, and was therefore not relevant to this review.  Nonfatal Myocardial Infarction  Nicotine (25 mg) group: n=1  Nicotine (15 mg) group: n=2  Placebo group: n=1  One subject (a 59 yr old male) in the nicotine (25 mg) group experienced a myocardial infarction during treatment; two subjects (a 48 yr old male and a 50 yr old male) in the nicotine (15 mg) group) experienced myocardial infarctions after discontinuing treatment; one subject (a 66 yr old male) in the placebo group experienced a myocardial infarction.  Nonfatal Stroke  NR  Cardiovascular Death  NR |
| 521 | Tuisku et al., 2016  Location: Finland  Setting: Lapland Central Hospital in Rovaniemi.  Trial Name: Smoking Habits and Smoking Cessation in Young Adults  Registration/Protocol: NCT01531049  Funding: Ministry of Social Affairs and Health, Finnish Research Foundation of the Pulmonary Diseases, and the Finnish Medical Society Duodecim  Design  Randomized, placebo-controlled study. The study compared the nicotine patch to placebo in light smokers (Heaviness of Smoking Index: 0-2 points), and the nicotine patch to varenicline in heavy smokers (Heaviness of Smoking Index: 3-6 points).  Eligibility  Inclusion: Age 18-26 yrs; smoking daily for at least the past month; smoked ≥100 cigarettes in their life; motivated to quit smoking and to be volunteers in the study; willing to participate in the 52-week follow-up.  Exclusion: Current drug or alcohol abuse; known allergy to study medication; lactation; pregnancy or intention to become pregnant during the study period.  Recruitment  Community media, colleges, and the army in northern parts of Finland.  Intervention  10 mg nicotine patch group: Light smokers received a 10 mg per 16 hrs nicotine patch for 8 weeks (brand NR). 21.3% of subjects completed treatment. Note: 51.1% of subjects used nicotine patches over 2 weeks and 21.3% completed the treatment.  15 mg nicotine patch group: Heavy smokers received a 15 mg per 16 hrs nicotine patch for 8 weeks (brand NR). 9.8% of subjects completed treatment. Note: 37.3% of subjects used nicotine patches over 2 weeks and 9.8% completed the treatment.  Control  Placebo patch group: Light smokers received a placebo patch (Leukomed T^®^ (adhesive wound dressing), BSN Medical, Luxembourg) for 8 weeks. The placebo patch was not identical to the nicotine patch, but selected because it did resemble a medication-type patch. Note: 36.1% of subjects used placebo patches over 2 weeks and 10.5% completed the treatment.  Varenicline group: Heavy smokers received varenicline (0.5 daily for 3 days, 0.5 mg twice per day until the end of the first week, followed by 1 mg twice per day until the end of the study; Champix^®^, Pfizer, Sandwich, Kent, UK) for 12 weeks. 20% of subjects completed treatment. Note: 76.7% of subjects used varenicline over 2 weeks and 20.0% completed the treatment.  Study Start/End Date  Spring 2012 to Spring 2014 | Study Population  Adult cigarette smokers  Sample Size  Total: n=288 subjects  10 mg nicotine patch group: n=93 subjects  15 mg nicotine patch group: n=49 subjects  Placebo patch group: n=86 subjects  Varenicline group: n=60 subjects  Subject Characteristics  *Median age (IQR)*  Total: 21.0 (19.0-23.0) yrs  10 mg nicotine patch group: 21.0 (19.0-23.0) yrs  15 mg nicotine patch group: 22.0 (19.0-24.0) yrs  Placebo patch group: 20.0 (18.0‑23.3) yrs  Varenicline group: 21.0 (19.0‑23.8) yrs  *Sex (% female)*  Total: 50.2% (n=146)  10 mg nicotine patch group: 52.1% (n=49)  15 mg nicotine patch group: 45.1% (n=23)  Placebo patch group: 51.2% (n=44)  Varenicline group: 50.0% (n=30)  *Race/ethnicity*  NR  *Mean yrs smoked±SD*  Total: 6.4±3.0  10 mg nicotine patch group: 5.9±3.0  15 mg nicotine patch group: 7.0±2.7  Placebo patch group: 5.8±3.1  Varenicline group: 7.3±2.8  *Median cpd (IQR)*  Total: 14.0 (10.0-20.0)  10 mg nicotine patch group: 10.0 (7.0-14.3)  15 mg nicotine patch group: 18.0 (15.0-20.0)  Placebo group: 10.0 (8.0-15.0)  Varenicline group: 18.0 (15.0-20.0)  Abstinence Characteristics  *Self-reported abstinence at Week 4 (%)*  Total: 33.0% (n=96)  10 mg nicotine patch group: 26.6% (n=25)  15 mg nicotine patch group: 19.6% (n=10)  Placebo group: 19.8% (n=17)  Varenicline group: 73.3% (n=44)  *Self-reported abstinence at Week 12 (%)*  Total: 23.0% (n=67)  10 mg nicotine patch group: 23.4% (n=22)  15 mg nicotine patch group: 15.7% (n=8)  Placebo patch group: 17.4% (n=15)  Varenicline group: 36.7% (n=22) | Arrhythmia  NR  Nonfatal Myocardial Infarction*  10 mg nicotine patch group: n=0  15 mg nicotine patch group: n=0  Placebo patch group: n=0  Varenicline group: n=0  Nonfatal Stroke*  10 mg nicotine patch group: n=0  15 mg nicotine patch group: n=0  Placebo patch group: n=0  Varenicline group: n=0  Cardiovascular Death*  10 mg nicotine patch group: n=0  15 mg nicotine patch group: n=0  Placebo patch group: n=0  Varenicline group: n=0  *No serious AEs were observed in the study. |
| 1368 | Uyar et al., 2007  Location: Turkey  Setting: Smoking cessation clinic  Trial Name: NR  Registration/Protocol: NR  Funding: Patches were provided by Nicoinell TTS^®^  Design  RCT.  Eligibility  Inclusion: Age 18-75 yrs; smoking ≥10 cpd in the past yr  Exclusion: History of depression; pregnancy and lactation; symptomatic cardiac disease; regular psychotropic drug use; alcohol or drug abuse; chronic dermatological ailment (specific to nicotine patch group); history of head trauma or convulsion (specific to bupropion group).  Recruitment  Subjects were randomly selected from individuals who had been referred to a smoking cessation program.  Intervention  Nicotine patch group: Subjects wore a 30 cm^2^, 21 mg nicotine patch daily for the first 2 weeks; a 20 cm^2^, 14 mg nicotine patch for the following 2 weeks; a 10 cm^2^, and a 7 mg patch for the final 2 weeks. Note: compliance with nicotine patch was 22% on Week 6.  Control  Bupropion group: Bupropion tablets were provided by Zyban^®^ GlaxoSmithKlein; subjects were prescribed 150 mg for the first 3 days, followed by 150 mg twice per day for 6 weeks. Note: compliance with bupropion treatment was 40% on Week 6.  No treatment group: Subjects were advised to stop smoking and harmful effects of smoking and benefits of quitting were stressed.  Study Start/End Date  September 2002 to July 2003 | Study Population  Adult cigarette smokers  Sample Size  Total: n=131 subjects  Nicotine patch group: n=50 subjects  Bupropion group: n=50 subjects  No treatment group: n=31 subjects  Subject Characteristics  *Mean age±SD*  Nicotine patch group: 36.3±12.7 yrs  Bupropion group: 36.0±10.5 yrs  No treatment group: 36.0±10.6 yrs  *Sex*  Nicotine patch group: male, 80%; female, 20%  Bupropion group: male, 88%; female, 12%  No treatment group: male, 70%; female, 30%  *Race/ethnicity*  NR  *Mean age at smoking onset±SD*  Nicotine patch group: 17.6±5.5 yrs  Bupropion group: 20.1±5.5 yrs  No treatment group: 18.8±4.6 yrs  *Average cpd*  NR  Abstinence Characteristics  *Abstinence rate at 4 weeks (%)*  Nicotine patch group: 36.0%  Bupropion group:46.0%  No treatment group: 22.5%  *Abstinence rate at 8 weeks (%)*  Nicotine patch group: 28.0%  Bupropion group: 38.0%  No treatment group: 16.1% | Arrhythmia*  Nicotine patch group: n=0  Bupropion group: n=3  No treatment group: n=NR  *Reported as tachycardia  Only adverse drug reactions were reported, hence, values for the no treatment group were NR.  Nonfatal Myocardial Infarction  NR  Nonfatal Stroke  NR  Cardiovascular Death  NR |
| 1731 | Wallström et al., 2000  Location: Sweden  Setting: Community  Trial Name: NR  Registration/Protocol: NR  Funding: NR  Design  Double-blind, randomized, placebo-controlled study.  Eligibility  Inclusion: Male and female smokers ≥20 yrs motivated to stop smoking, smoking ≥10 cpd, have smoked for ≥3 yrs, eCO ≥10 ppm.  Exclusion: Pathological changes to the floor of the mouth, CVD, psychotropic medication use, use of other forms of tobacco, or any other NRT products during the last 6 months, pregnancy or breastfeeding.  Recruitment  Newspaper advertisement.  Intervention  Nicotine group: Subjects received nicotine sublingual tablets (Nicorette^®^ Microtab, Pharmacia & Upjohn). Highly dependent smokers (FTQ score ≥ 7) were instructed to use 2 tablets (4 mg nicotine) per hr, up to a maximum of 40 tablets per day. Subjects with an FTQ score <7 were instructed to use 1 tablet per hr, up to a maximum of 20 tablets per day. Subjects were advised to use the full treatment dose for 3 months. After 3 months, treatment could be tapered off up to the 6-month visit if necessary. Note: At Week 6, 83% of subjects in the high dependence group and 89% in the low dependence group used the nicotine tablets daily (mean daily consumption: high dependence group, 22±8 tablets; low dependence group, 11±4 tablets).  Control  Placebo group: Subjects received a placebo tablet identical in appearance to the nicotine tablet containing only 3 µg capsaicin, along with the same instructions as described for the intervention. Note: At Week 6, 77% of subjects in the high dependence group and 70% in the low dependence group used the placebo tablets daily (mean daily consumption: high dependence group, 22±9 tablets; low dependence group, 12±6 tablets).  Study Start/End Date  NR | Study Population  General adult population  Sample Size  Total: n=247 subjects  Nicotine group: n=123 subjects  Placebo group: n=124 subjects  Subject Characteristics  *Mean age±SD*  Nicotine group: 44.5±11.6 yrs  Placebo group: 44.7±11.4 yrs  *Sex*  Nicotine group: male, n=45; female, n=78  Placebo group: male, n=56; female, n=68  *Race/ethnicity*  NR  *Mean yrs smoked±SD*  Nicotine group: 26.1±10.3  Placebo group: 26.9±9.8  *Mean cpd±SD*  Nicotine group: 18.2±5.4  Placebo group: 20.6±6.8  Abstinence Characteristics  *Continuous abstinence from Week 2 to Month 6 (%):*  Nicotine group: 33%  Placebo group: 18% | Arrhythmia*  Nicotine group: n=1  Placebo group: n=0  *Described as atrial fibrillation  Nonfatal Myocardial Infarction**  Nicotine group: n=0  Placebo group: n=0  Nonfatal Stroke**  Nicotine group: n=0  Placebo group: n=0  Cardiovascular Death**  Nicotine group: n=0  Placebo group: n=0  **Reported serious AEs were listed, none of which were nonfatal myocardial infarction, nonfatal stroke, or cardiovascular death. |
| 167 | Xiao et al., 2020  Location: China  Setting: 10 academic or hospital-associated smoking cessation clinics in 6 cities  Trial Name: Efficacy and Safety Study of Nicotine Mint Lozenge (2 mg and 4 mg) in Smoking Cessation  Registration/Protocol: NCT00985985  Funding: Kaohsiung Veteran General Hospital  Design  Randomized, double-blind, placebo-controlled study.  Eligibility  Inclusion: Age ≥18 yrs; smoking daily ≥1 yr before study enrollment; strong motivation to quit smoking; capable of understanding instructions and providing written informed consent.  Exclusion: Using tobacco other than cigarettes or nicotine delivery systems other than lozenge; smoking illicit substances; used smoking cessation aids within 30 days before study entry; history of alcohol or drug use; currently involved in any other clinical trial or previous participation in this study; pregnant, breastfeeding, or of childbearing potential but refused use of medical contraception; unstable or uncontrolled systemic medical conditions, hyperthyroidism, or used insulin for diabetes mellitus; recent myocardial infarction or cerebral vascular accidents; allergy to aspartame or phenyl-pyruvic acid, diagnosis of phenylketonuria; or medical history endangering subject safety or study result validity.  Recruitment  Smoking cessation clinics and via advertisements published in the hospitals.  Intervention  2 mg nicotine lozenge group: Subjects with low nicotine dependence (i.e., first cigarette >30 min after waking) received 2 mg nicotine lozenge. Subjects were instructed to use one lozenge every 1-2 hrs (9-15 per day) during Weeks 1-6, one lozenge every 2-4 hrs (3‑6 per day) during Weeks 7-9, one lozenge every 4-8 hrs (3‑6 per day) during Weeks 10-12, and use occasionally (1-2 lozenges per day) after Week 12, and to discontinue use after Week 24.  4 mg nicotine lozenge group: Subjects with high nicotine dependence (i.e., first cigarette within 30 min of awakening) received the 4 mg nicotine lozenge. Subjects were instructed to use one lozenge every 1-2 hrs (9-15 per day) during Weeks 1-6, one lozenge every 2-4 hrs (3‑6 per day) during Weeks 7-9, one lozenge every 4-8 hrs (3‑6 per day) during Weeks 10-12, and use occasionally (1-2 lozenges per day) after Week 12, and to discontinue use after Week 24.  Note: Three subjects were excluded from safety analysis due to non-use of study drugs (treatment group NR). Safety analysis included subjects in good compliance with study protocol.  Control  2 mg placebo lozenge group: Subjects received placebo lozenges matching 2 mg nicotine lozenges  4 mg placebo lozenge group: Subjects received placebo lozenges matching 4 mg nicotine lozenges.  Study Start/End Date  May 2009 to February 2011 | Study Population  Adult cigarette smokers  Sample Size  Total: n=720 subjects  2 mg nicotine lozenge group: n=241 subjects  4 mg nicotine lozenge group: n=119 subjects  2 mg placebo lozenge group: n=242 subjects  4 mg placebo lozenge group: n=118 subjects  Subject Characteristics*  *Mean age±SD*  2 mg nicotine lozenge group: 38.4±11.8 yrs  4 mg nicotine lozenge group: 42.3±10.4 yrs  2 mg placebo lozenge group: 39.9±11.8 yrs  4 mg placebo lozenge group: 42.0±10.6 yrs  *Sex*  2 mg nicotine lozenge group: male, 95.4% (n=230); female, 4.6% (n=11)  4 mg nicotine lozenge group: male, 98.3% (n=118); female, 1.7% (n=2)  2 mg placebo lozenge group: male, 87.1% (n=235) male; female, 2.9% (n=7)  4 mg placebo lozenge group: male, 97.5% (n=116); female, 2.5% (n=2)  *Race/ethnicity*  NR  *Distribution of mean cpd*  2 mg nicotine lozenge group: ≤10 cpd, n=64 (26.6%); 11-20 cpd, n=144 (59.8%); 21-30 cpd, n=23 (9.5%); ≥31 cpd, n=10 (4.2%)  4 mg nicotine lozenge group: ≤10 cpd, n=8 (6.7%); 11-20 cpd, n=60 (50.0%); 21-30 cpd, n=37 (30.8%); ≥31 cpd, n=15 (12.5%)  2 mg placebo lozenge group: ≤10 cpd, n=69 (28.5%); 11-20 cpd, n=135 (55.8%); 21-30 cpd, n=28 (11.6%); ≥31 cpd, n=10 (4.1%)  4 mg placebo lozenge group: ≤10 cpd, n=8 (6.7%); 11-20 cpd, n=76 (63.9%); 21-30 cpd, n=15 (12.6%); ≥31 cpd, n=20 (16.8%)  *Subject characteristics are based on the sample at randomization (Total, n=723; 2 mg nicotine lozenge group, n=241; 4 mg nicotine lozenge group, n=120; 2 mg placebo lozenge group, n=242; 4 mg placebo lozenge group, n=120).  Abstinence Characteristics  *eCO-confirmed (≤10 ppm) 28-day continuous abstinence at Week 6 (%)*  2 mg nicotine lozenge group: 24.48% (n=59)  4 mg nicotine lozenge group: 30.83% (n=37)  2 mg placebo lozenge group: 21.49% (n=52)  4 mg placebo lozenge group: 20.17% (n=24)  *7-day PPA rate at Week 24 (%)*  2 mg nicotine lozenge group: 41.5%  4 mg nicotine lozenge group: 50.0%  2 mg placebo lozenge group: 43.1%  4 mg placebo lozenge group: 43.7% | Arrhythmia  NR  Nonfatal Myocardial Infarction*  2 mg nicotine lozenge group: n=0  4 mg nicotine lozenge group: n=0  2 mg placebo lozenge group: n=0  4 mg placebo lozenge group: n=0  Nonfatal Stroke*  2 mg nicotine lozenge group: n=0  4 mg nicotine lozenge group: n=0  2 mg placebo lozenge group: n=0  4 mg placebo lozenge group: n=0  Cardiovascular Death  2 mg nicotine lozenge group: n=0  4 mg nicotine lozenge group: n=0  2 mg placebo lozenge group: n=0  4 mg placebo lozenge group: n=0  *Reported serious AEs were listed, none of which were nonfatal myocardial infarction or nonfatal stroke. |

Abbreviations: μg=microgram(s); AEs=adverse events; BMI=body mass index; CAMH=Centre for Addiction and Mental Health; CI=confidence interval; cm^2^=square centimeter; cpd=cigarettes per day; CVD=cardiovascular disease; DSM=Diagnostic and Statistical Manual of Mental Disorders; ECG=electrocardiogram; eCO=exhaled/expired carbon monoxide; FTND=Fagerstrom Test for Nicotine Dependence; FTQ=Fagerstrom Tolerance Questionnaire; GP(s)=general practitioner(s); hr(s)=hour(s); ICU=intensive care unit; IQR=interquartile range; kg=kilograms; kg/m^2^=kilograms per square meter; mg=milligram; min = minutes; mmHg=millimeters of mercury; NA=not applicable; NCI=National Cancer Institute; ng/ml=nanograms per milliliter; NIAAA=National Institute on Alcohol Abuse and Alcoholism; NIDA=National Institute on Drug Abuse; NIH=National Institutes of Health; NR=not reported; NRT=nicotine replacement therapy; OR=odds ratio; PPA=point prevalence abstinence; ppm=parts per million; RCT=randomized control trial; SD=standard deviation; SE=standard error; UK=United Kingdom; US=United States; yr(s)=year(s).

## F. Risk of Bias Assessment

|  | **Random sequence generation (selection bias)** | **Allocation concealment (selection bias)** | **Blinding of participants and personnel (performance bias)** | **Blinding of outcome assessment (detection bias)** | **Incomplete outcome data (attrition bias)** | **Selective reporting (reporting bias)** | **Other bias** | **Overall Assessment^†^** |
| --- | --- | --- | --- | --- | --- | --- | --- | --- |
| Aubin et al., 2008^1^ |  |  |  |  |  |  | **-** |  |
| Benowitz et al., 2018 |  |  |  |  |  |  | **-** |  |
| Chen et al., 2020 |  |  |  |  |  |  | **-** |  |
| Covey et al., 2007^2^ |  |  |  |  |  |  | **-** |  |
| de Jong et al., 2018 |  |  |  |  |  |  | **-** |  |
| Ebbert et al., 2013^3^ |  |  |  |  |  |  | **-** |  |
| Ebbert et al., 2007^4^ |  |  |  |  |  |  | **-** |  |
| Etter et al., 2002^5^ |  |  |  |  |  |  | **-** |  |
| Foulds et al., 1993^6^ |  |  |  |  |  |  | **-** |  |
| Gilbert et al., 2020^7^ |  |  |  |  |  |  | **-** |  |
| Hays et al., 1999^8^ |  |  |  |  |  |  | **-** |  |
| Hughes et al., 2003^9^ |  |  |  |  |  |  | **-** |  |
| Joseph et al., 1996^10^ |  |  |  |  |  |  | **-** |  |
| Kavin and Shivey, 1995^11^ |  |  |  |  |  |  | **-** |  |
| Koychev et al., 2011^12^ |  |  |  |  |  |  | **-** |  |
| Kralikova et al., 2009 |  |  |  |  |  |  | **-** |  |
| Lambrichts et al., 2017^13^ |  |  |  |  |  |  | **-** |  |
| Lerman et al., 2015 |  |  |  |  |  |  | **-** |  |
| Lewis et al., 1998^14^ |  |  |  |  |  |  | **-** |  |
| Møller et al., 2002^15^ |  |  |  |  |  |  | **-** |  |
| Myung et al., 2007^16^ |  |  |  |  |  |  | **-** |  |
| Oncken et al., 2014^17^ |  |  |  |  |  |  | **-** |  |
| The Preloading Investigators, 2018^18^ |  |  |  |  |  |  | **-** |  |
| Ramon et al., 2014^19^ |  |  |  |  |  |  | **-** |  |
| Rohsenow et al., 2017^20^ |  |  |  |  |  |  | **-** |  |
| Rungruanghiranya et al., 2008^21^ |  |  |  |  |  |  | **-** |  |
| Rungruanghiranya et al., 2012^22^ |  |  |  |  |  |  | **-** |  |
| Sachs et al., 1993^23^ |  |  |  |  |  |  | **-** |  |
| Sandborn et al., 1997^24^ |  |  |  |  |  |  | **-** |  |
| Shiffman and Ferguson, 2008^25^ |  |  |  |  |  |  | **-** |  |
| Shiffman et al., 2009^26^ |  |  |  |  |  |  | **-** |  |
| Shiffman et al., 2006^27^ |  |  |  |  |  |  | **-** |  |
| Stapleton and Sutherland, 2011 |  |  |  |  |  |  | **-** |  |
| Stein et al., 2013^28^ |  |  |  |  |  |  | **-** |  |
| Sun et al., 2009^29^ |  |  |  |  |  |  | **-** |  |
| Thomas et al., 1995^30^ |  |  |  |  |  |  | **-** |  |
| Thomsen et al., 2010^31^ |  |  |  |  |  |  | **-** |  |
| Tønnesen et al., 1999^32^ |  |  |  |  |  |  | **-** |  |
| Tuisku et al., 2016^33^ |  |  |  |  |  |  | **-** |  |
| Uyar et al., 2007^34^ |  |  |  |  |  |  | **-** |  |
| Wallström et al., 2000^35^ |  |  |  |  |  |  | **-** |  |
| Xiao et al., 2020 |  |  |  |  |  |  | **-** |  |

Key: Low risk of bias; High risk of bias; Unclear risk of bias

**^†^**The overall assessment of bias was: “low” if all bias domains were assessed as “low” risk; “high” if at least one bias domain was assessed as “high” risk; and “unclear” if at least one bias domain was assessed as “unclear” risk, and no domains were assessed as “high” risk.

**Overall Summary of Risk of Bias Assessment:**

1. Open-label study design was used to evaluate NRT versus varenicline; dummy-placebos could have been used. Safety outcomes were not listed as outcomes of interest in the protocol and therefore they were not pre-specified outcomes of interest.
2. AEs were not listed in the study protocol and therefore they were not pre-specified outcomes of interest.
3. There was a >20% difference in attrition between groups (16% in the nicotine group, and 41% in the placebo group); AEs were not listed in the study protocol and therefore they were not pre-specified outcomes of interest.
4. Placebo-controlled, double-blind study design was used, however, it was not specified whether AE assessors were blinded. AEs were listed in methods, however, study protocol was not available and therefore it was unclear if AEs were pre-specified outcomes of interest.
5. The investigators were aware of the nature of products mailed to subjects and were therefore not blinded to treatment.
6. Overall attrition was >50% (72%) during treatment.
7. Method of allocation concealment was not specified. Details on who assessed AEs and how AEs were assessed were not provided.
8. Placebo-controlled, double-blind study design was used, however, it was not specified whether AE assessors were blinded. AEs were listed in methods, however, study protocol was not available and therefore it was unclear if AEs were pre-specified outcomes of interest.
9. Method of randomization and allocation concealment were not specified. Placebo-controlled, double-blind study design was used, however, it was not specified whether AE assessors were blinded. Attrition rate was not reported. AEs were not listed in methods and study protocol was not available, therefore it was unclear if AEs were pre-specified outcomes of interest.
10. The study did not specify if and how allocation concealment was done. Placebo-controlled, double-blind study design was used, however, it was not specified if AE assessors were blinded. Attrition rate was not reported.
11. Method of randomization and allocation concealment were not specified. It was not described how AEs were assessed. AEs were not listed in methods and study protocol was not available, therefore it was unclear if AEs were pre-specified outcomes of interest.
12. Method of randomization was not specified. AEs were listed in methods, however, study protocol was not available and therefore it was unclear if AEs were pre-specified outcomes of interest.
13. Both subjects and investigators were blinded to treatment allocation, however, allocated treatment was administered by the nursing staff, whose blinding status was unclear. It was not specified how AEs were reported. Study protocol was not available, therefore it was unclear if AEs were pre-specified outcomes of interest.
14. Number of subjects lost to follow-up was not reported. AEs were listed in methods, however, study protocol was not available and therefore it was unclear if AEs were pre-specified outcomes of interest.
15. AEs were listed in methods, however, study protocol was not available and therefore it was unclear if AEs were pre-specified outcomes of interest.
16. Placebo-controlled, double-blind study design was used, however, it was not specified who was blinded, to include outcome assessors. AEs were not listed in methods, and study protocol was not available, therefore it was unclear if AEs were pre-specified outcomes of interest.
17. Subjects and research personnel were not blinded to nicotine patch treatment, specifically. There was a >20% difference in attrition between groups: 42% of subjects in placebo group; 42% of subjects in topiramate group; and 16% of those in nicotine/topiramate group
18. Open-label study design was used; no placebo group.
19. Subjects and care providers were blinded, however, it was unclear if investigators were also blinded. It was not specified who assessed AEs and whether or not they were blinded to treatment.
20. AEs were not listed in the study protocol and therefore they were not pre-specified outcomes of interest.
21. Method of allocation concealment was not specified. Placebo-controlled, double-blind study design was used, however, it was not specified if AE assessors were blinded. AEs were listed in methods, however, study protocol was not available and therefore it was unclear if AEs were pre-specified outcomes of interest.
22. Subjects and investigators were not blinded to treatment and there was no placebo group.
23. Method of randomization and allocation concealment was not specified. Placebo-controlled, double-blind study design was used, however, it was not specified if AE assessors were blinded. AEs were listed in methods, however, study protocol was not available and therefore it was unclear if AEs were pre-specified outcomes of interest.
24. AEs were listed in methods, however, study protocol was not available and therefore it was unclear if AEs were pre-specified outcomes of interest.
25. Method of allocation concealment was not specified. Placebo-controlled, double-blind study design was used, however, it was not specified if AE assessors were blinded. AEs were not listed in methods, and study protocol was not available, therefore it was unclear if AEs were pre-specified outcomes of interest.
26. Method of allocation concealment was not specified. Placebo-controlled, double-blind study design was used, however, it was not specified if AE assessors were blinded. Attrition was reported before and after the end of the treatment period; however, it was unclear there was sufficient retention at the 8-week milestone (i.e., end of treatment). AEs were listed in methods, however, study protocol was not available and therefore it was unclear if AEs were pre-specified outcomes of interest.
27. Placebo-controlled, double-blind study design was used, however, it was not specified if outcome assessors were blinded. AEs were not listed in methods, and study protocol was not available, therefore it was unclear if AEs were pre-specified outcomes of interest.
28. AEs were not listed in the study protocol and therefore they were not pre-specified outcomes of interest
29. Method of allocation concealment was not specified. Placebo-controlled, double-blind study design was used, however, it was not specified if AE assessors were blinded. A total 250 subjects were enrolled in the study and 211 subjects completed the study (84%), however, it was unclear whether all enrolled subjects were randomized, nor did the authors specify how many subjects were randomized to each group. AEs were not listed in methods, and study protocol was not available, therefore it was unclear if AEs were pre-specified outcomes of interest.
30. Overall attrition was >50% (53%) during treatment.
31. Subjects could not be blinded to treatment due to behavioral intervention. Given the nature of the intervention compared to usual care, intervention group received more face-to-face time.
32. Overall attrition was >50% during treatment phase (end of treatment was Week 24): 58% at Week 22, and 64% at Week 26.
33. Single-blind study design was used. Placebo group was included, but the nicotine and placebo patches were not identical. AEs were assessed using open-ended questions, and there was no indication that the assessors were blinded to treatment. Safety outcomes were not listed in the protocol and therefore they were not pre-specified outcomes of interest. .
34. Method of randomization and allocation concealment were not specified. It was not specified if AE assessors were blinded. It was reported that four subjects discontinued bupropion treatment due to AEs, however, it was not specified if additional subjects were lost to follow-up for other reasons. AEs were not listed in methods, and study protocol was not available, therefore it was unclear if AEs were pre-specified outcomes of interest.
35. Method of allocation concealment was not specified. Double-blind study design was used, however, it was not specified if AE assessors were blinded. Attrition or loss to follow-up was not reported. AEs were listed in methods, however, study protocol was not available and therefore it was unclear if AEs were pre-specified outcomes of interest.

## G. Subgroup Meta-Analyses - Arrythmia

Subgroup analyses by arrhythmia subtype were performed. Pooled analysis of two studies showed similar rates of atrial fibrillation in subjects randomized to nicotine and non-nicotine control groups (RR 1.41; 95% CI 0.15-13.54; I^2^ 0%). One study reporting incidence of bradycardia found no statistically significant difference between the nicotine and non-nicotine control groups (RR 3.00; 95% CI 0.13-71.51; I^2^ NA). Pooled analysis of three studies that reported the incidence of tachycardia showed no statistically significant differences between the nicotine and non-nicotine control groups (RR 2.59; 95% CI 0.15-43.49; I^2^ 66%). Based on the analysis of one study that reported the incidence of serious cardiac arrhythmia (arrhythmia type not specified), there was no statistically significant difference between the nicotine and non-nicotine control groups (RR 2.99; 95% CI 0.60-14.78; I^2^ NA). Pooled analysis of four studies reporting the incidence of cardiac arrhythmia showed similar rates of unspecified cardiac arrhythmia in the nicotine and non-nicotine control groups (RR 0.76; 95% CI 0.32-1.78; I^2^ 72%). A test for subgroup differences between the pooled results by arrhythmia subtype found no significant differences between the five subgroups (p=0.56; I^2^ 0%).

In a second subgroup analysis by duration of nicotine treatment period, pooled analysis of nine studies that reported the incidence of arrhythmia during a nicotine treatment period of less than 12 weeks showed no statistically significant difference between the nicotine and non-nicotine control groups (RR 1.12; 95% CI 0.52-2.44; I^2^ 59%). Two studies reported the incidence of arrhythmia during a nicotine treatment period of 12 or more weeks. Pooled analysis of two studies reporting arrhythmia during a nicotine treatment period of 12 or more weeks showed no statistically significant difference in rates of arrhythmia between groups (RR 3.01; 95% CI 0.32-28.56; I^2^ 0%). A test for subgroup differences between the pooled results of studies less than 12 weeks in duration and studies 12 weeks or longer in duration found no significant difference between the two subgroups (p=0.42; I^2^ 0%).

### Subgroup Analysis by Arrhythmia Subtype

Subgroup analyses for each of the four arrhythmia subtypes reported among the included studies—atrial fibrillation, bradycardia, tachycardia, and serious cardiac arrhythmia—as well as subgroup of studies with unspecified arrhythmia subtype, are presented below (see **Figure 1**). A test for subgroup differences between the pooled results by arrhythmia subtype found no significant differences between the five subgroups (p=0.56), with no statistical heterogeneity observed (I^2^ 0%).

### Subgroup Analysis for Atrial Fibrillation

Pooled analysis of two studies with 1,493 subjects overall (541 nicotine subjects and 952 non-nicotine control subjects) showed similar rates of atrial fibrillation in subjects randomized to nicotine and non-nicotine control groups (RR 1.41; 95% CI 0.15-13.54 (see **Figure 1**)^52^^,^ ^111^, with no statistical heterogeneity observed between the two studies (I^2^ 0%). One study was graded as “low” risk of bias^41^, and the other as “unclear” risk of bias^100^.

Both studies were smoking cessation studies among current cigarette smokers—one among a healthy study population^111^ and the other among subjects of unspecified health status^52^. One of the studies reported low rates of continuous cigarette smoking abstinence (from Week 2 onwards) in both its nicotine (33%) and placebo (18%) groups^111^; the second study did not provide rates of abstinence but did note that the one event of atrial fibrillation which occurred in the study was in a subject receiving placebo who had been smoking 15 cigarettes per day at the time of the event^52^. Nicotine versus placebo was evaluated in both studies, with one of the studies also evaluating nicotine versus varenicline^52^. Treatment duration was 12 weeks in both studies. Treatment adherence was relatively high in one study, with daily use reported in at least 70 percent of subjects in each group^111^. The second study reported treatment adherence by product type—patches and pills—but not differentiated by active versus placebo for each product type: 63 percent of subjects used 80 percent or more of the recommended patches, and 62 percent of subjects used 80 percent or more of the recommended pills^52^.

### Subgroup Analysis for Bradycardia

One study with a total of 80 subjects (40 subjects in both the nicotine and non-nicotine groups), and graded as having a “high” risk of bias, reported the incidence of bradycardia^110^. Analysis showed that there was no statistically significant difference in the incidence of bradycardia between the nicotine and non-nicotine control groups (RR 3.00; 95% CI 0.13-71.51) (see **Figure 1**). Analysis of heterogeneity was not applicable, given that only one study was included in the analysis.

The study reported one case of bradycardia in the nicotine group (40 subjects who received a 15 mg nicotine patch), occurring 6 weeks into a 6-month treatment period^110^. Subsequent investigation showed a nodal bradycardia for which no cause could be found. Because the bradycardia did not revert to sinus rhythm when treatment with nicotine was stopped, a permanent pacemaker was implanted and the subject subsequently made a full recovery. No cases of bradycardia were reported in the placebo group. Treatment adherence was not reported. Given that this was a study of nonsmokers and former cigarette smokers, cigarette smoking abstinence was not applicable.

### Subgroup Analysis for Tachycardia

Pooled analysis of three studies that reported the incidence of tachycardia showed that there were no statistically significant differences between the nicotine and non-nicotine control groups (RR 2.59; 95% CI 0.15-43.49) (see **Figure 1**)^106^^,^ ^115^^,^ ^122^. The overall pooled sample size was 186 subjects, with 92 and 94 subjects in the nicotine and non-nicotine control groups, respectively. All three studies were graded as “unclear” risk of bias. Statistical heterogeneity observed by the model was moderate to substantial (I^2^ 66%).

Two of the studies were among nonsmokers or former cigarette smokers—one among a healthy study population^122^, and the other among subjects with ulcerative colitis^106^. The third study was among current cigarette smokers with no specification of the study population’s health status; in this study, cigarette smoking abstinence (measure not reported) at Week 4 of the 6-week study was low, ranging from 22 percent to 46 percent across the three study groups^115^. Two of the studies evaluated nicotine versus placebo^106^^,^ ^122^, while the third study compared three groups: nicotine, bupropion, and no treatment^115^. One study, a crossover trial in a laboratory setting, had a treatment duration of 1 hour^111^, suggesting that adherence was optimal. Treatment duration in the remaining two studies was 4 weeks in one study^106^ and 6 weeks in the other study^115^, with only the latter study reporting adherence (22% in the nicotine patch group and 40% in the bupropion group at the end of the treatment)^115^.

### Subgroup Analysis for Serious Cardiac Arrhythmia

Based on the analysis of one “low” risk of bias study that reported the incidence of serious cardiac arrhythmia (arrhythmia type not specified)^119^, there was no statistically significant difference between the nicotine and non-nicotine control groups (RR 2.99; 95% CI 0.60-14.78) (see **Figure 1**). Analysis of heterogeneity was not applicable, given that only one study was included in the analysis.

In this study, Benowitz et al. (2018) evaluated safety data for adult cigarette smokers treated for 12 weeks (11 weeks for nicotine patches) with either 21 mg nicotine patches (with tapering to 7 mg; n=2,022), varenicline (n=2,016), bupropion (n=2,006), or placebo (n=2,014)^119^. Treatment adherence, evaluated by the mean number of days of medication exposure, was relatively high (~74 days) and similar across groups. Exhaled CO-verified continuous cigarette smoking abstinence rates from Weeks 9 to 12 were 23 percent in the nicotine group, 34 percent in the varenicline group, 23 percent in the bupropion group, and 12 percent in the placebo group.

### Subgroup Analysis for Cardiac Arrhythmia (Type not Specified)

Four studies, one with “low” risk of bias and three with “unclear” risk of bias, reported the incidence of cardiac arrhythmia without specifying the type^84^^,^ ^94^^,^ ^99^^,^ ^114^. Pooled analysis of 4,052 subjects (2,026 subjects in both the nicotine and non-nicotine control groups) showed similar rates of unspecified cardiac arrhythmia in the nicotine and non-nicotine control groups (RR 0.76; 95% CI 0.32-1.78) (see **Figure 1**). Statistical heterogeneity observed by the model was moderate to substantial (I^2^ 72%).

All four studies were among current cigarette smokers^84^^,^ ^94^^,^ ^99^^,^ ^114^; two among hospitalized subjects^94^^,^ ^114^, one among subjects with a history of at least one CVD^99^, and one among subjects with unspecified health status^84^. Cigarette smoking abstinence rates, reported by three studies, were low and ranged from 10 percent (continuous, self-reported abstinence up to Day 28)^84^ to 31 percent (self-reported 7-day PPA at the end of the treatment period)^114^ in the nicotine group, and from 4 percent (continuous, self-reported abstinence up to Day 28)^84^ to 18 percent (self-reported 7-day PPA at the end of the treatment period) in the non-nicotine control group^114^. All four studies evaluated nicotine versus placebo treatment; one of the studies also evaluated nicotine versus minimal care^114^. Treatment duration varied from up to 30 days^94^ to 10 weeks^99^. Treatment adherence was reported by two studies: one study reported that 73 percent of subjects in the nicotine group adhered to treatment at Week 6^99^, while the second study reported an adherence rate of 70 percent in the nicotine group on Week 1 of 6^114^.

One of the four studies provided additional detail on each occurrence of arrhythmia in their study. The study, by Joseph et al. (1996)^87^, reported that six hospital admissions for cardiac arrhythmia occurred among five subjects in the nicotine patch group; four of these subjects were currently smoking cigarettes at the time of admission, while one subject was not. In the placebo group, six hospital admissions for cardiac arrhythmia occurred among three subjects, all of whom were currently smoking cigarettes at the time of admission for arrhythmia.

Figure 1: Forest Plot, Subgroup Analysis for Arrhythmia by Type of Arrhythmia

### Subgroup Analysis for Duration of Nicotine Treatment Period (Arrhythmia)

Pooled analysis of nine studies that reported the incidence of arrhythmia during a nicotine treatment period of less than 12 weeks showed no statistically significant difference between the nicotine and non-nicotine control groups (RR 1.12; 95% CI 0.52-2.44) (see **Figure 2**)^52^^,^ ^84^^,^ ^94^^,^ ^99^^,^ ^106^^,^ ^110^^,^ ^111^^,^ ^114^^,^ ^115^^,^ ^119^^,^ ^122^. The overall pooled sample size was 13,542 subjects, with 4,558 and 8,984 subjects in the nicotine and non-nicotine control groups, respectively. Three of the studies were graded as “low” risk of bias^52^^,^ ^94^^,^ ^119^, and six were graded as “unclear” risk of bias^84^^,^ ^99^^,^ ^106^^,^ ^114^^,^ ^115^^,^ ^122^. Statistical heterogeneity observed by the model was moderate to substantial (I^2^ 59%).

Two studies, one with “high” risk of bias^110^ and one with “unclear” risk of bias^111^, reported the incidence of arrhythmia during a nicotine treatment period of 12 or more weeks. Pooled analysis of 327 subjects (163 and 164 subjects in the nicotine and non-nicotine control groups, respectively) showed no statistically significant difference in rates of arrhythmia between groups (RR 3.01; 95% CI 0.32-28.56) (see **Figure 2**). No statistical heterogeneity was observed by the model (I^2^ 0%).

A test for subgroup differences between the pooled results of studies less than 12 weeks in duration and studies 12 weeks or longer in duration found no significant difference between the two subgroups (p=0.42), with no statistical heterogeneity observed (I^2^ 0%).

Figure 2: Forest plot, Subgroup Analysis for Duration of Nicotine Treatment Period (Arrhythmia)

### Sensitivity Analyses

Two sensitivity analyses were performed to detect whether the pooled result from the main arrhythmia analysis was sensitive to the removal of (1) studies judged to be at “high” risk of bias, and (2) studies that did not report systematically collecting AE data in their methodology or protocol. In the first sensitivity analysis, the study by Thomas et al. (1995) was excluded)^110^, while in the second sensitivity analysis, studies by Kavin and Shivey (1995) and Uyar et al. (2007) were excluded^115^^,^ ^122^. In both sensitivity analyses, results were consistent with the main analysis after excluding studies with a “high” risk of bias (RR 1.16; 95% CI 0.56-2.43; I^2^ 54%; see **Figure 3**), and after excluding studies that did not report systematic collection of AE data (RR 1.09; 95% CI 0.56-2.09; I^2^ 44%; see **Figure 4**).

Figure 3: Forest plot, Sensitivity Analysis for Arrhythmia—Exclusion of High Risk of Bias Studies

Figure 4: Forest plot, Sensitivity Analysis for Arrhythmia—Exclusion of Studies Not Reporting Systematic Collection of AE Data

### Publication Bias for Arrhythmia

**Figure 5** shows the funnel plot for the outcome of arrhythmia. The plot is largely symmetrical, suggesting an absence of publication bias.

Figure 5: Funnel Plot for Publication Bias, Arrhythmia

## H. Subgroup Analysis for Duration of Nicotine Treatment Period (Nonfatal Myocardial Infarction)

In a subgroup analysis by duration of nicotine treatment period, pooled analysis of 20 studies during a treatment period of less than 12 weeks showed no statistically significant difference between the nicotine and non-nicotine control groups (RR 0.71; 95% CI 0.22-2.23; I^2^ 0%). Pooled analysis of 12 studies with a nicotine treatment period of 12 or more weeks showed no statistically significant difference in rates of nonfatal myocardial infarction between groups (RR 1.18; 95% CI 0.28-4.92; I^2^ 0%). A test for subgroup differences between the pooled results of studies less than 12 weeks in duration and studies 12 weeks or longer in duration found no significant difference between the two subgroups (p=0.58; I^2^ 0%).

Pooled analysis of 20 studies that reported the incidence of nonfatal myocardial infarction during a nicotine treatment period of less than 12 weeks showed no statistically significant difference between the nicotine and non-nicotine control groups (RR 0.71; 95% CI 0.22-2.23) (see **Figure 6**)^51^^,^ ^52^^,^ ^86^^,^ ^87^^,^ ^89^^,^ ^91^^,^ ^93-95^^,^ ^97-101^^,^ ^107^^,^ ^108^^,^ ^114^^,^ ^117^^,^ ^119^^,^ ^120^. The overall pooled sample size was 14,657 subjects, with 5,082 and 9,575 subjects in the nicotine and non-nicotine control groups, respectively. Three of the studies were graded as “low” risk of bias^52^^,^ ^94^^,^ ^119^, five were graded as “high” risk of bias^51^^,^ ^89^^,^ ^93^^,^ ^95^^,^ ^117^, and 12 were graded as “unclear” risk of bias^86^^,^ ^87^^,^ ^91^^,^ ^97-101^^,^ ^107^^,^ ^108^^,^ ^114^^,^ ^120^. No statistical heterogeneity was observed by the model (I^2^ 0%).

Twelve studies^83^^,^ ^90^^,^ ^96^^,^ ^102-105^^,^ ^110^^,^ ^111^^,^ ^116^^,^ ^118^^,^ ^121^, seven with “high” risk of bias^90^^,^ ^96^^,^ ^102^^,^ ^104^^,^ ^110^^,^ ^116^^,^ ^121^, two with “low” risk of bias^83^^,^ ^118^, and three with “unclear” risk of bias^103^^,^ ^105^^,^ ^111^, reported the incidence of nonfatal myocardial infarction during a nicotine treatment period of 12 or more weeks. Pooled analysis of 6,288 subjects (4,241 and 2,047 subjects in the nicotine and non-nicotine control groups, respectively) showed no statistically significant difference in rates of nonfatal myocardial infarction between groups (RR 1.18; 95% CI 0.28-4.92) (see **Figure 6**). No statistical heterogeneity was observed by the model (I^2^ 0%).

A test for subgroup differences between the pooled results of studies less than 12 weeks in duration and studies 12 weeks or longer in duration found no significant difference between the two subgroups (p=0.58) with no statistical heterogeneity observed (I^2^ 0%).

Figure 6: Forest plot, Subgroup Analysis for Duration of Nicotine Treatment Period (Nonfatal Myocardial Infarction)

### Sensitivity Analyses

Two sensitivity analyses were performed to detect whether pooled results from the main analysis was sensitive to the removal of (1) studies judged to be at high risk of bias, and (2) studies that did not report systematically collecting AE data. Consequently, in the first sensitivity analysis, 12 studies were excluded^51^^,^ ^89^^,^ ^90^^,^ ^93^^,^ ^95^^,^ ^96^^,^ ^102^^,^ ^104^^,^ ^110^^,^ ^116^^,^ ^117^^,^ ^121^, while four studies were excluded from the second sensitivity analysis^90^^,^ ^91^^,^ ^100^^,^ ^117^.

In both sensitivity analyses, results were consistent with the main analysis after excluding studies with a high risk of bias (RR 0.37; 95% CI 0.10‑1.42; I^2^ 0%; see **Figure 7**), and after excluding studies that did not report systematic collection of AE data (RR 0.76; 95% CI 0.30-1.92; I^2^ 0%; see **Figure 8**).

Figure 7: Forest plot, Sensitivity Analysis for Nonfatal Myocardial Infarction—Exclusion of High Risk of Bias Studies

Figure 8: Forest plot, Sensitivity Analysis for Myocardial Infarction—Exclusion of Studies Not Reporting Systematic Collection of AE data

### Publication Bias for Nonfatal Myocardial Infarction

Due to the small number of studies whose effect measures could be estimated (less than 10 overall), an evaluation of publication bias was not possible.

## I. Subgroup Analysis for Duration of Nicotine Treatment Period (Nonfatal Stroke)

In a subgroup analysis by duration of nicotine treatment period, pooled analysis of 19 studies with a nicotine treatment period of less than 12 weeks showed no statistically significant difference between the nicotine and non-nicotine control groups (RR 0.40; 95% CI 0.02-8.22; I2 NA). Pooled analysis of 10 studies with a nicotine treatment period of 12 or more weeks showed no statistically significant difference in rates of nonfatal stroke between groups (RR 0.26; 95% CI 0.03-2.38; I2 0%). A test for subgroup differences between the pooled results of studies less than 12 weeks in duration and studies 12 weeks or longer in duration found no significant difference between the two subgroups (p=0.83; I2 0%).

Ten studies^83^^,^ ^90^^,^ ^102-104^^,^ ^110^^,^ ^111^^,^ ^113^^,^ ^116^^,^ ^118^, five with “high” risk of bias^90^^,^ ^102^^,^ ^104^^,^ ^110^^,^ ^116^, three with “low” risk of bias^83^^,^ ^113^^,^ ^118^, and two with “unclear” risk of bias^103^^,^ ^111^ reported the incidence of nonfatal stroke during a nicotine treatment period of 12 or more weeks. Pooled analysis of 3,067 subjects (1,418 and 1,649 subjects in the nicotine and non-nicotine control groups, respectively) showed no statistically significant difference in rates of nonfatal stroke between groups (RR 0.26; 95% CI 0.03-2.38) (see **Figure 9**). No statistical heterogeneity was observed by the model (I^2^ 0%).

A test for subgroup differences between the pooled results of studies less than 12 weeks in duration and studies 12 weeks or longer in duration found no significant difference between the two subgroups (p=0.83) with no statistical heterogeneity observed (I^2^ 0%).

Figure 9: Forest plot, Subgroup Analysis for Duration of Nicotine Treatment Period (Nonfatal Stroke)

### Sensitivity Analyses

Two sensitivity analyses were performed to detect whether pooled results from the main analysis were sensitive to the removal of (1) studies judged to be at high risk of bias, and (2) studies that did not report systematically collecting AE data. Consequently, 10 studies were excluded from the first sensitivity analysis^51^^,^ ^89^^,^ ^90^^,^ ^93^^,^ ^95^^,^ ^102^^,^ ^104^^,^ ^110^^,^ ^116^^,^ ^117^, while four studies were excluded from the second sensitivity analysis^90^^,^ ^91^^,^ ^100^^,^ ^117^.

Results in the sensitivity analyses were unchanged from the main meta-analysis, after excluding studies with a high risk of bias (RR 0.30; 95% CI 0.05-1.80; I^2^ 0%; see **Figure 10**), and after excluding studies that did not report systematic collection of AE data (RR 0.30; 95% CI 0.05-1.80; I^2^ 0%; see **Figure 11**).

Figure 10: Forest plot, Sensitivity Analysis for Nonfatal Stroke—Exclusion of High Risk of Bias Studies

Figure 11: Forest plot, Sensitivity Analysis for Nonfatal Stroke—Exclusion of Studies Not Reporting Systematic Collection of AE Data

### Publication Bias for Nonfatal Stroke

Due to the small number of studies whose effect measures could be estimated (less than 10 overall), an evaluation of publication bias was not possible.

## J. Subgroup Analysis for Duration of Nicotine Treatment Period (Cardiovascular Death)

In a subgroup analysis by duration of nicotine treatment period, pooled analysis of 21 studies that reported the incidence of cardiovascular death during a nicotine treatment period of less than 12 weeks showed no statistically significant difference between the nicotine and non-nicotine control groups (RR 1.29; 95% CI 0.14-11.62; I2 0%). Pooled analysis of 12 studies with a nicotine treatment period of 12 or more weeks showed no statistically significant difference in rates of cardiovascular death between groups (RR 3.50; 95% CI 0.43-28.36; I2 0%). A test for subgroup differences between the pooled results of studies less than 12 weeks in duration and studies 12 weeks or longer in duration found no significant difference between the two subgroups (p=0.52; I2 0%). Pooled analysis of 19 studies that reported the incidence of nonfatal stroke during a nicotine treatment period of less than 12 weeks showed no statistically significant difference between the nicotine and non-nicotine control groups (RR 0.40; 95% CI 0.02-8.22) (see **Figure 9**)^51^^,^ ^52^^,^ ^86^^,^ ^88^^,^ ^89^^,^ ^91^^,^ ^93-95^^,^ ^97^^,^ ^98^^,^ ^100^^,^ ^101^^,^ ^107^^,^ ^108^^,^ ^114^^,^ ^117^^,^ ^119^^,^ ^120^. The overall pooled sample size was 14,146 subjects, with 4,829 and 9,317 subjects in the nicotine and non-nicotine control groups, respectively. Three of the studies were graded as “low” risk of bias^52^^,^ ^94^^,^ ^119^, five of them were graded as “high” risk of bias^51^^,^ ^89^^,^ ^93^^,^ ^95^^,^ ^117^, and 11 were graded as “unclear” risk of bias^86^^,^ ^88^^,^ ^91^^,^ ^97^^,^ ^98^^,^ ^100^^,^ ^101^^,^ ^107^^,^ ^108^^,^ ^114^^,^ ^120^. Heterogeneity could not be computed for this analysis, as the results were estimated from only one study.

Pooled analysis of 21 studies that reported the incidence of cardiovascular death during a nicotine treatment period of less than 12 weeks showed no statistically significant difference between the nicotine and non-nicotine control groups (RR 1.29; 95% CI 0.14-11.62) (see **Figure 12**)^51^^,^ ^86-89^^,^ ^91-95^^,^ ^97^^,^ ^98^^,^ ^100^^,^ ^101^^,^ ^107^^,^ ^108^^,^ ^112^^,^ ^114^^,^ ^117^^,^ ^119^^,^ ^120^. The overall pooled sample size was 13,698 subjects, with 4,805 and 8,893 subjects in the nicotine and non-nicotine control groups, respectively. Six of the studies were graded as “high” risk of bias^51^^,^ ^89^^,^ ^92^^,^ ^93^^,^ ^95^^,^ ^117^, two were graded as “low” risk of bias^94^^,^ ^119^, and 13 were graded as “unclear” risk of bias^86-88^^,^ ^91^^,^ ^97^^,^ ^98^^,^ ^100^^,^ ^101^^,^ ^107^^,^ ^108^^,^ ^112^^,^ ^114^^,^ ^120^. No statistical heterogeneity was observed by the model (I^2^ 0%).

Twelve studies^83^^,^ ^85^^,^ ^96^^,^ ^102-104^^,^ ^109-111^^,^ ^113^^,^ ^116^^,^ ^118^, six with “high” risk of bias^85^^,^ ^96^^,^ ^102^^,^ ^104^^,^ ^110^^,^ ^116^, four with “low” risk of bias^83^^,^ ^109^^,^ ^113^^,^ ^118^, and two with “unclear” risk of bias^103^^,^ ^111^ reported the incidence of cardiovascular death during a nicotine treatment period of 12 or more weeks. Pooled analysis of 4,684 subjects (2,180 and 2,504 subjects in the nicotine and non-nicotine control groups, respectively) showed no statistically significant difference in rates of cardiovascular death between groups (RR 3.50; 95% CI 0.43-28.36) (see **Figure 12**). No statistical heterogeneity was observed by the model (I^2^ 0%).

A test for subgroup differences between the pooled results of studies less than 12 weeks in duration and studies 12 weeks or longer in duration found no significant difference between the two subgroups (p=0.52) with no statistical heterogeneity observed (I^2^ 0%).

Figure 12: Forest plot, Subgroup Analysis for Duration of Nicotine Treatment Period (Cardiovascular Death)

### Sensitivity Analyses

Two sensitivity analyses were performed to detect whether pooled results from the main analysis was sensitive to the removal of (1) studies judged to be at high risk of bias, and (2) studies that did not report systematically collecting AE data. In the first sensitivity analysis, the study by Etter et al. (2002) was excluded, while three studies were excluded from the second sensitivity analysis^91^^,^ ^100^^,^ ^117^. In both sensitivity analyses, results were consistent with the main analysis after excluding studies with a high risk of bias (RR 1.52; 95% CI 0.27-8.53; I^2^ 0%; see **Figure 13**), and after excluding studies that did not report systematic collection of AE data (RR 2.18; 95% CI 0.48-9.91; I^2^ 0%; see **Figure 14**).

Figure 13: Forest plot, Sensitivity Analysis for Cardiovascular Death—Exclusion of High Risk of Bias Studies

Figure 14: Forest plot, Sensitivity Analysis for Cardiovascular Death—Exclusion of Studies Not Reporting Systematic Collection of AE data

### Publication Bias for Cardiovascular Death

Due to the small number of studies whose effect measures could be estimated (less than 10 overall), an evaluation of publication bias was not possible.

# 2 REFERENCES

1.	Roth GA, Johnson C, Abajobir A, Abd-Allah F, Abera SF, Abyu G, et al. Global, Regional, and National Burden of Cardiovascular Diseases for 10 Causes, 1990 to 2015. Journal of the American College of Cardiology. 2017;70(1):1-25.

2.	Khan MA, Hashim MJ, Mustafa H, Baniyas MY, Al Suwaidi S, AlKatheeri R, et al. Global Epidemiology of Ischemic Heart Disease: Results from the Global Burden of Disease Study. Cureus. 2020;12(7):e9349.

3.	Mensah GA, Roth GA, Fuster V. The Global Burden of Cardiovascular Diseases and Risk Factors: 2020 and Beyond. Journal of the American College of Cardiology. 2019;74(20):2529-32.

4.	Roth GA, Mensah GA, Johnson CO, Addolorato G, Ammirati E, Baddour LM, et al. Global Burden of Cardiovascular Diseases and Risk Factors, 1990-2019: Update From the GBD 2019 Study. J Am Coll Cardiol. 2020;76(25):2982-3021.

5.	Global burden of 369 diseases and injuries in 204 countries and territories, 1990-2019: a systematic analysis for the Global Burden of Disease Study 2019. Lancet (London, England). 2020;396(10258):1204-22.

6.	Khoury H, Lavoie L, Welner S, Folkerts K. The Burden of Major Adverse Cardiac Events and Antiplatelet Prevention in Patients with Coronary or Peripheral Arterial Disease. Cardiovascular therapeutics. 2016;34(2):115-24.

7.	Brown JC, Gerhardt TE, Kwon E. Risk Factors For Coronary Artery Disease. StatPearls. Treasure Island (FL): StatPearls Publishing Copyright © 2021, StatPearls Publishing LLC.; 2021.

8.	Pencina MJ, Navar AM, Wojdyla D, Sanchez RJ, Khan I, Elassal J, et al. Quantifying Importance of Major Risk Factors for Coronary Heart Disease. Circulation. 2019;139(13):1603-11.

9.	Dugani SB, Hydoub YM, Ayala AP, Reka R, Nayfeh T, Ding JF, et al. Risk Factors for Premature Myocardial Infarction: A Systematic Review and Meta-analysis of 77 Studies. Mayo Clin Proc Innov Qual Outcomes. 2021;5(4):783-94.

10.	Duncan MS, Freiberg MS, Greevy RA, Jr., Kundu S, Vasan RS, Tindle HA. Association of Smoking Cessation With Subsequent Risk of Cardiovascular Disease. Jama. 2019;322(7):642-50.

11.	Benowitz NL, Burbank AD. Cardiovascular toxicity of nicotine: Implications for electronic cigarette use. Trends in cardiovascular medicine. 2016;26(6):515-23.

12.	Benowitz NL, Fraiman JB. Cardiovascular effects of electronic cigarettes. Nat Rev Cardiol. 2017;14(8):447-56.

13.	Price LR, Martinez J. Cardiovascular, carcinogenic and reproductive effects of nicotine exposure: A narrative review of the scientific literature. F1000Res. 2019;8:1586.

14.	Adamopoulos D, Argacha JF, Gujic M, Preumont N, Degaute JP, van de Borne P. Acute effects of nicotine on arterial stiffness and wave reflection in healthy young non-smokers. Clin Exp Pharmacol Physiol. 2009;36(8):784-9.

15.	Benowitz NL, Hansson A, Jacob P, 3rd. Cardiovascular effects of nasal and transdermal nicotine and cigarette smoking. Hypertension. 2002;39(6):1107-12.

16.	Najem B, Houssière A, Pathak A, Janssen C, Lemogoum D, Xhaët O, et al. Acute cardiovascular and sympathetic effects of nicotine replacement therapy. Hypertension (Dallas, Tex : 1979). 2006;47(6):1162-7.

17.	Sjoberg N, Saint DA. A single 4 mg dose of nicotine decreases heart rate variability in healthy nonsmokers: implications for smoking cessation programs. Nicotine Tob Res. 2011;13(5):369-72.

18.	Perkins KA, Lerman C, Keenan J, Fonte C, Coddington S. Rate of nicotine onset from nicotine replacement therapy and acute responses in smokers. Nicotine Tob Res. 2004;6(3):501-7.

19.	Benowitz NL, Porchet H, Sheiner L, Jacob P, 3rd. Nicotine absorption and cardiovascular effects with smokeless tobacco use: comparison with cigarettes and nicotine gum. Clin Pharmacol Ther. 1988;44(1):23-8.

20.	Lewis MJ, Balaji G, Dixon H, Syed Y, Lewis KE. Influence of smoking abstinence and nicotine replacement therapy on heart rate and QT time-series. Clinical physiology and functional imaging. 2010;30(1):43-50.

21.	Stein PK, Rottman JN, Kleiger RE. Effect of 21 mg transdermal nicotine patches and smoking cessation on heart rate variability. The American journal of cardiology. 1996;77(9):701-5.

22.	Lee PN, Fariss MW. A systematic review of possible serious adverse health effects of nicotine replacement therapy. Arch Toxicol. 2017;91(4):1565-94.

23.	Page MJ, McKenzie JE, Bossuyt PM, Boutron I, Hoffmann TC, Mulrow CD, et al. The PRISMA 2020 statement: an updated guideline for reporting systematic reviews. BMJ (Clinical research ed). 2021;372:n71.

24.	Shea BJ, Reeves BC, Wells G, Thuku M, Hamel C, Moran J, et al. AMSTAR 2: a critical appraisal tool for systematic reviews that include randomised or non-randomised studies of healthcare interventions, or both. BMJ (Clinical research ed). 2017;358:j4008.

25.	Higgins JPT, Altman DG, Sterne JAC. Section 13.5. Assessing risk of bias in non-randomized studies. In: Higgins JPT, Green S, editors. Cochrane Handbook for Systematic Reviews of Interventions. Version 5.1.0: The Cochrane Collaboration; 2011.

26.	Guyatt GH, Oxman AD, Vist GE, Kunz R, Falck-Ytter Y, Alonso-Coello P, et al. GRADE: an emerging consensus on rating quality of evidence and strength of recommendations. BMJ. 2008;336(7650):924-6.

27.	DerSimonian R, Laird N. Meta-analysis in clinical trials. Control Clin Trials 1986;7(3):177-88.

28.	The Cochrane Collaboration. Identifying and measuring heterogeneity. 2011. In: Cochrane Handbook for Systematic Reviews of Interventions [Internet]. Cochrane. 5.1.0. Available from: <https://handbook-5-1.cochrane.org/chapter_9/9_5_2_identifying_and_measuring_heterogeneity.htm>.

29.	The Cochrane Collaboration. Recommendations on testing for funnel plot asymmetry. 2011. In: Cochrane Handbook for Systematic Reviews of Interventions [Internet]. Cochrane. 5.1.0. Available from: <https://handbook-5-1.cochrane.org/chapter_10/10_4_3_1_recommendations_on_testing_for_funnel_plot_asymmetry.htm>.

30.	The Nordic Cochrane Centre. Review Manager (RevMan). In: Centre TNC, editor. Copenhagen: The Cochrane Collaboration; 2014.

31.	Greenland S, Satterfield MH, Lanes SF. A meta-analysis to assess the incidence of adverse effects associated with the transdermal nicotine patch. Drug Saf. 1998;18(4):297-308.

32.	Mills EJ, Thorlund K, Eapen S, Wu P, Prochaska JJ. Cardiovascular events associated with smoking cessation pharmacotherapies: a network meta-analysis. Circulation. 2014;129(1):28-41.

33.	Carney G, Bassett K, Maclure M, Taylor S, Dormuth CR. Cardiovascular and neuropsychiatric safety of smoking cessation pharmacotherapies in non-depressed adults: a retrospective cohort study. Addiction. 2020;115(8):1534-46.

34.	Rigotti NA, Thorndike AN, Regan S, McKool K, Pasternak RC, Chang Y, et al. Bupropion for smokers hospitalized with acute cardiovascular disease. The American journal of medicine. 2006;119(12):1080-7.

35.	Chelladurai Y, Singh S. Varenicline and cardiovascular adverse events: a perspective review. Ther Adv Drug Saf. 2014;5(4):167-72.

36.	Gershon AS, Campitelli MA, Hawken S, Victor C, Sproule BA, Kurdyak P, et al. Cardiovascular and Neuropsychiatric Events after Varenicline Use for Smoking Cessation. American journal of respiratory and critical care medicine. 2018;197(7):913-22.

37.	Pfizer Inc. CHANTIX- varenicline tartrate Prescribing Information 2019 [cited 2021 2021/09/24]. Available from: <http://labeling.pfizer.com/ShowLabeling.aspx?id=557>.

38.	Charrois TL. Systematic reviews: what do you need to know to get started? The Canadian journal of hospital pharmacy. 2015;68(2):144-8.

39.	Murad MH, Asi N, Alsawas M, Alahdab F. New evidence pyramid. Evidence-based medicine. 2016;21(4):125-7.

40.	Moran AE, Forouzanfar MH, Roth GA, Mensah GA, Ezzati M, Flaxman A, et al. The global burden of ischemic heart disease in 1990 and 2010: the Global Burden of Disease 2010 study. Circulation. 2014;129(14):1493-501.

41.	Lippi G, Sanchis-Gomar F, Cervellin G. Global epidemiology of atrial fibrillation: An increasing epidemic and public health challenge. Int J Stroke. 2021;16(2):217-21.

42.	Desai DS, Hajouli S. Arrhythmias. StatPearls. Treasure Island (FL)2021.

43.	Lindberg T, Wimo A, Elmstahl S, Qiu C, Bohman DM, Sanmartin Berglund J. Prevalence and Incidence of Atrial Fibrillation and Other Arrhythmias in the General Older Population: Findings From the Swedish National Study on Aging and Care. Gerontol Geriatr Med. 2019;5:2333721419859687.

44.	Pan B, Jin X, Jun L, Qiu S, Zheng Q, Pan M. The relationship between smoking and stroke: A meta-analysis. Medicine. 2019;98(12):e14872.

45.	Teo KK, Ounpuu S, Hawken S, Pandey MR, Valentin V, Hunt D, et al. Tobacco use and risk of myocardial infarction in 52 countries in the INTERHEART study: a case-control study. Lancet (London, England). 2006;368(9536):647-58.

46.	U.S. Food and Drug Administration. What is a Serious Adverse Event? Internet: U.S. Food and Drug Administration; 2016 [cited 2021 2021/09/21]. Available from: <https://www.fda.gov/safety/reporting-serious-problems-fda/what-serious-adverse-event>.

47.	Higgins JP, Altman DG, Gøtzsche PC, Jüni P, Moher D, Oxman AD, et al. The Cochrane Collaboration's tool for assessing risk of bias in randomised trials. BMJ (Clinical research ed). 2011;343:d5928.

48.	Higgins J, Thomas J, Chandler J, Cumpston M, Li T, Page MJ, et al., editors. Cochrane Handbook for Systematic Reviews of Interventions. 2nd ed: John Wiley & Sons; 2021.

49.	Guyatt G, Oxman AD, Akl EA, Kunz R, Vist G, Brozek J, et al. GRADE guidelines: 1. Introduction-GRADE evidence profiles and summary of findings tables. Journal of clinical epidemiology. 2011;64(4):383-94.

50.	Schünemann H, Brożek J, Guyatt G, Oxman A, editors. The GRADE Handbook: GRADE Working Group; 2013.

51.	Investigators TP. Effects on abstinence of nicotine patch treatment before quitting smoking: parallel, two arm, pragmatic randomised trial. BMJ (Clinical research ed). 2018;361:k2164.

52.	Lerman C, Schnoll RA, Hawk LW, Jr., Cinciripini P, George TP, Wileyto EP, et al. Use of the nicotine metabolite ratio as a genetically informed biomarker of response to nicotine patch or varenicline for smoking cessation: a randomised, double-blind placebo-controlled trial. The Lancet Respiratory medicine. 2015;3(2):131-8.

53.	Berlin I, Grangé G, Jacob N, Tanguy ML. Nicotine patches in pregnant smokers: randomised, placebo controlled, multicentre trial of efficacy. BMJ (Clinical research ed). 2014;348:g1622.

54.	Bolliger CT, Zellweger JP, Danielsson T, van Biljon X, Robidou A, Westin A, et al. Smoking reduction with oral nicotine inhalers: double blind, randomised clinical trial of efficacy and safety. BMJ (Clinical research ed). 2000;321(7257):329-33.

55.	Carpenter MJ, Hughes JR, Solomon LJ, Callas PW. Both smoking reduction with nicotine replacement therapy and motivational advice increase future cessation among smokers unmotivated to quit. Journal of consulting and clinical psychology. 2004;72(3):371-81.

56.	Nides M, Danielsson T, Saunders F, Perfekt R, Kapikian R, Solla J, et al. Efficacy and Safety of a Nicotine Mouth Spray for Smoking Cessation: A Randomized, Multicenter, Controlled Study in a Naturalistic Setting. Nicotine & tobacco research : official journal of the Society for Research on Nicotine and Tobacco. 2020;22(3):339-45.

57.	Rigotti NA, Gonzales D, Dale LC, Lawrence D, Chang Y. A randomized controlled trial of adding the nicotine patch to rimonabant for smoking cessation: efficacy, safety and weight gain. Addiction (Abingdon, England). 2009;104(2):266-76.

58.	Tulloch HE, Pipe AL, Els C, Clyde MJ, Reid RD. Flexible, dual-form nicotine replacement therapy or varenicline in comparison with nicotine patch for smoking cessation: a randomized controlled trial. BMC medicine. 2016;14:80.

59.	Shiffman S, Dresler CM, Hajek P, Gilburt SJ, Targett DA, Strahs KR. Efficacy of a nicotine lozenge for smoking cessation. Archives of internal medicine. 2002;162(11):1267-76.

60.	Coleman T, Cooper S, Thornton JG, Grainge MJ, Watts K, Britton J, et al. A randomized trial of nicotine-replacement therapy patches in pregnancy. The New England journal of medicine. 2012;366(9):808-18.

61.	Bullen C, Howe C, Laugesen M, McRobbie H, Parag V, Williman J, et al. Electronic cigarettes for smoking cessation: a randomised controlled trial. Lancet (London, England). 2013;382(9905):1629-37.

62.	Aung MN, Yuasa M, Moolphate S, Lorga T, Yokokawa H, Fukuda H, et al. Effectiveness of a new multi-component smoking cessation service package for patients with hypertension and diabetes in northern Thailand: a randomized controlled trial (ESCAPE study). Substance abuse treatment, prevention, and policy. 2019;14(1):10.

63.	Batra A, Klingler K, Landfeldt B, Friederich HM, Westin A, Danielsson T. Smoking reduction treatment with 4-mg nicotine gum: a double-blind, randomized, placebo-controlled study. Clinical pharmacology and therapeutics. 2005;78(6):689-96.

64.	Cook JW, Collins LM, Fiore MC, Smith SS, Fraser D, Bolt DM, et al. Comparative effectiveness of motivation phase intervention components for use with smokers unwilling to quit: a factorial screening experiment. Addiction (Abingdon, England). 2016;111(1):117-28.

65.	Ebbert JO, Severson HH, Croghan IT, Danaher BG, Schroeder DR. A randomized clinical trial of nicotine lozenge for smokeless tobacco use. Nicotine & tobacco research : official journal of the Society for Research on Nicotine and Tobacco. 2009;11(12):1415-23.

66.	Glover ED, Glover PN, Franzon M, Sullivan CR, Cerullo CC, Howell RM, et al. A comparison of a nicotine sublingual tablet and placebo for smoking cessation. Nicotine & tobacco research : official journal of the Society for Research on Nicotine and Tobacco. 2002;4(4):441-50.

67.	Goedert JJ, Scoppio BM, Pfeiffer R, Neve L, Federici AB, Long LR, et al. Treatment of classic Kaposi sarcoma with a nicotine dermal patch: a phase II clinical trial. Journal of the European Academy of Dermatology and Venereology : JEADV. 2008;22(9):1101-9.

68.	Herrera N, Franco R, Herrera L, Partidas A, Rolando R, Fagerström KO. Nicotine gum, 2 and 4 mg, for nicotine dependence. A double-blind placebo-controlled trial within a behavior modification support program. Chest. 1995;108(2):447-51.

69.	Hill RD, Rigdon M, Johnson S. Behavioral smoking cessation treatment for older chronic smokers. Behavior Therapy. 1993;24(2):321-9.

70.	Hughes JR, Lesmes GR, Hatsukami DK, Richmond RL, Lichtenstein E, Jorenby DE, et al. Are higher doses of nicotine replacement more effective for smoking cessation? Nicotine & tobacco research : official journal of the Society for Research on Nicotine and Tobacco. 1999;1(2):169-74.

71.	Ikonomidis I, Marinou M, Vlastos D, Kourea K, Andreadou I, Liarakos N, et al. Effects of varenicline and nicotine replacement therapy on arterial elasticity, endothelial glycocalyx and oxidative stress during a 3-month smoking cessation program. Atherosclerosis. 2017;262:123-30.

72.	Joseph AM, Hecht SS, Murphy SE, Lando H, Carmella SG, Gross M, et al. Smoking reduction fails to improve clinical and biological markers of cardiac disease: a randomized controlled trial. Nicotine & tobacco research : official journal of the Society for Research on Nicotine and Tobacco. 2008;10(3):471-81.

73.	Koegelenberg CF, Noor F, Bateman ED, van Zyl-Smit RN, Bruning A, O'Brien JA, et al. Efficacy of varenicline combined with nicotine replacement therapy vs varenicline alone for smoking cessation: a randomized clinical trial. Jama. 2014;312(2):155-61.

74.	Molyneux A, Lewis S, Leivers U, Anderton A, Antoniak M, Brackenridge A, et al. Clinical trial comparing nicotine replacement therapy (NRT) plus brief counselling, brief counselling alone, and minimal intervention on smoking cessation in hospital inpatients. Thorax. 2003;58(6):484-8.

75.	Piper ME, Smith SS, Schlam TR, Fiore MC, Jorenby DE, Fraser D, et al. A randomized placebo-controlled clinical trial of 5 smoking cessation pharmacotherapies. Archives of general psychiatry. 2009;66(11):1253-62.

76.	Rennard SI, Glover ED, Leischow S, Daughton DM, Glover PN, Muramoto M, et al. Efficacy of the nicotine inhaler in smoking reduction: A double-blind, randomized trial. Nicotine & tobacco research : official journal of the Society for Research on Nicotine and Tobacco. 2006;8(4):555-64.

77.	Richmond RL, Harris K, de Almeida Neto A. The transdermal nicotine patch: results of a randomised placebo-controlled trial. The Medical journal of Australia. 1994;161(2):130-5.

78.	Rose JE, Behm FM. Adapting smoking cessation treatment according to initial response to precessation nicotine patch. The American journal of psychiatry. 2013;170(8):860-7.

79.	Schuppers AS, de Jong B, Van Den Oever HLA, Arbouw MEL, Kruisdijk-Gerritsen A, Van Zanten ARH. Nicotine Replacement Therapy in the Intensive Care Unit: A Randomized Controlled Pilot Study. Intensive Care Medicine Experimental. 2017;5(2).

80.	Tønnesen P, Lauri H, Perfekt R, Mann K, Batra A. Efficacy of a nicotine mouth spray in smoking cessation: a randomised, double-blind trial. The European respiratory journal. 2012;40(3):548-54.

81.	Warner DO, Patten CA, Ames SC, Offord KP, Schroeder DR. Effect of nicotine replacement therapy on stress and smoking behavior in surgical patients. Anesthesiology. 2005;102(6):1138-46.

82.	Winhusen TM, Brigham GS, Kropp F, Lindblad R, Gardin JG, 2nd, Penn P, et al. A randomized trial of concurrent smoking-cessation and substance use disorder treatment in stimulant-dependent smokers. The Journal of clinical psychiatry. 2014;75(4):336-43.

83.	Kralikova E, Kozak JT, Rasmussen T, Gustavsson G, Le Houezec J. Smoking cessation or reduction with nicotine replacement therapy: a placebo-controlled double blind trial with nicotine gum and inhaler. BMC public health. 2009;9:433.

84.	Shiffman S, Ferguson SG, Strahs KR. Quitting by gradual smoking reduction using nicotine gum: a randomized controlled trial. American journal of preventive medicine. 2009;36(2):96-104.e1.

85.	Etter JF, Laszlo E, Zellweger JP, Perrot C, Perneger TV. Nicotine replacement to reduce cigarette consumption in smokers who are unwilling to quit: a randomized trial. Journal of clinical psychopharmacology. 2002;22(5):487-95.

86.	Koychev I, McMullen K, Lees J, Dadhiwala R, Grayson L, Perry C, et al. A validation of cognitive biomarkers for the early identification of cognitive enhancing agents in schizotypy: a three-center double-blind placebo-controlled study. European neuropsychopharmacology : the journal of the European College of Neuropsychopharmacology. 2012;22(7):469-81.

87.	Lambrichts DPV, Boersema GSA, Tas B, Wu Z, Vrijland WW, Kleinrensink GJ, et al. Nicotine chewing gum for the prevention of postoperative ileus after colorectal surgery: a multicenter, double-blind, randomised, controlled pilot study. International journal of colorectal disease. 2017;32(9):1267-75.

88.	Møller AM, Villebro N, Pedersen T, Tønnesen H. Effect of preoperative smoking intervention on postoperative complications: a randomised clinical trial. Lancet (London, England). 2002;359(9301):114-7.

89.	Oncken C, Arias AJ, Feinn R, Litt M, Covault J, Sofuoglu M, et al. Topiramate for smoking cessation: a randomized, placebo-controlled pilot study. Nicotine & tobacco research : official journal of the Society for Research on Nicotine and Tobacco. 2014;16(3):288-96.

90.	Stein MD, Caviness CM, Kurth ME, Audet D, Olson J, Anderson BJ. Varenicline for smoking cessation among methadone-maintained smokers: a randomized clinical trial. Drug and alcohol dependence. 2013;133(2):486-93.

91.	Sun HQ, Guo S, Chen DF, Jiang ZN, Liu Y, Di XL, et al. Family support and employment as predictors of smoking cessation success: a randomized, double-blind, placebo-controlled trial of nicotine sublingual tablets in chinese smokers. The American journal of drug and alcohol abuse. 2009;35(3):183-8.

92.	Thomsen T, Tønnesen H, Okholm M, Kroman N, Maibom A, Sauerberg ML, et al. Brief smoking cessation intervention in relation to breast cancer surgery: a randomized controlled trial. Nicotine & tobacco research : official journal of the Society for Research on Nicotine and Tobacco. 2010;12(11):1118-24.

93.	Aubin HJ, Bobak A, Britton JR, Oncken C, Billing CB, Jr., Gong J, et al. Varenicline versus transdermal nicotine patch for smoking cessation: results from a randomised open-label trial. Thorax. 2008;63(8):717-24.

94.	de Jong B, Schuppers AS, Kruisdijk-Gerritsen A, Arbouw MEL, van den Oever HLA, van Zanten ARH. The safety and efficacy of nicotine replacement therapy in the intensive care unit: a randomised controlled pilot study. Annals of intensive care. 2018;8(1):70.

95.	Ebbert JO, Croghan IT, Schroeder DR, Hurt RD. A randomized phase II clinical trial of high-dose nicotine patch therapy for smokeless tobacco users. Nicotine & tobacco research : official journal of the Society for Research on Nicotine and Tobacco. 2013;15(12):2037-44.

96.	Foulds J, Stapleton J, Hayward M, Russell MA, Feyerabend C, Fleming T, et al. Transdermal nicotine patches with low-intensity support to aid smoking cessation in outpatients in a general hospital. A placebo-controlled trial. Archives of family medicine. 1993;2(4):417-23.

97.	Gilbert DG, Rabinovich NE, McDaniel JT. Nicotine patch for cannabis withdrawal symptom relief: a randomized controlled trial. Psychopharmacology. 2020;237(5):1507-19.

98.	Hughes JR, Novy P, Hatsukami DK, Jensen J, Callas PW. Efficacy of nicotine patch in smokers with a history of alcoholism. Alcoholism, clinical and experimental research. 2003;27(6):946-54.

99.	Joseph AM, Norman SM, Ferry LH, Prochazka AV, Westman EC, Steele BG, et al. The safety of transdermal nicotine as an aid to smoking cessation in patients with cardiac disease. The New England journal of medicine. 1996;335(24):1792-8.

100.	Myung SK, Seo HG, Park S, Kim Y, Kim DJ, Lee DH, et al. Sociodemographic and smoking behavioral predictors associated with smoking cessation according to follow-up periods: a randomized, double-blind, placebo-controlled trial of transdermal nicotine patches. Journal of Korean medical science. 2007;22(6):1065-70.

101.	Ramon JM, Morchon S, Baena A, Masuet-Aumatell C. Combining varenicline and nicotine patches: a randomized controlled trial study in smoking cessation. BMC medicine. 2014;12:172.

102.	Rohsenow DJ, Tidey JW, Martin RA, Colby SM, Swift RM, Leggio L, et al. Varenicline versus nicotine patch with brief advice for smokers with substance use disorders with or without depression: effects on smoking, substance use and depressive symptoms. Addiction (Abingdon, England). 2017;112(10):1808-20.

103.	Rungruanghiranya S, Ekpanyaskul C, Hattapornsawan Y, Tundulawessa Y. Effect of nicotine polyestex gum on smoking cessation and quality of life. Journal of the Medical Association of Thailand = Chotmaihet thangphaet. 2008;91(11):1656-62.

104.	Rungruanghiranya S, Ekpanyaskul C, Sakulisariyaporn C, Watcharanat P, Akkalakulawas K. Efficacy of fresh lime for smoking cessation. Journal of the Medical Association of Thailand = Chotmaihet thangphaet. 2012;95 Suppl 12:S76-82.

105.	Sachs DP, Säwe U, Leischow SJ. Effectiveness of a 16-hour transdermal nicotine patch in a medical practice setting, without intensive group counseling. Archives of internal medicine. 1993;153(16):1881-90.

106.	Sandborn WJ, Tremaine WJ, Offord KP, Lawson GM, Petersen BT, Batts KP, et al. Transdermal nicotine for mildly to moderately active ulcerative colitis. A randomized, double-blind, placebo-controlled trial. Annals of internal medicine. 1997;126(5):364-71.

107.	Shiffman S, Ferguson SG. The effect of a nicotine patch on cigarette craving over the course of the day: results from two randomized clinical trials. Current medical research and opinion. 2008;24(10):2795-804.

108.	Shiffman S, Scharf DM, Shadel WG, Gwaltney CJ, Dang Q, Paton SM, et al. Analyzing milestones in smoking cessation: illustration in a nicotine patch trial in adult smokers. Journal of consulting and clinical psychology. 2006;74(2):276-85.

109.	Stapleton JA, Sutherland G. Treating heavy smokers in primary care with the nicotine nasal spray: randomized placebo-controlled trial. Addiction (Abingdon, England). 2011;106(4):824-32.

110.	Thomas GA, Rhodes J, Mani V, Williams GT, Newcombe RG, Russell MA, et al. Transdermal nicotine as maintenance therapy for ulcerative colitis. The New England journal of medicine. 1995;332(15):988-92.

111.	Wallström M, Nilsson F, Hirsch JM. A randomized, double-blind, placebo-controlled clinical evaluation of a nicotine sublingual tablet in smoking cessation. Addiction (Abingdon, England). 2000;95(8):1161-71.

112.	Hays JT, Croghan IT, Schroeder DR, Offord KP, Hurt RD, Wolter TD, et al. Over-the-counter nicotine patch therapy for smoking cessation: results from randomized, double-blind, placebo-controlled, and open label trials. American journal of public health. 1999;89(11):1701-7.

113.	Chen LS, Baker TB, Miller JP, Bray M, Smock N, Chen J, et al. Genetic Variant in CHRNA5 and Response to Varenicline and Combination Nicotine Replacement in a Randomized Placebo-Controlled Trial. Clinical pharmacology and therapeutics. 2020;108(6):1315-25.

114.	Lewis SF, Piasecki TM, Fiore MC, Anderson JE, Baker TB. Transdermal nicotine replacement for hospitalized patients: a randomized clinical trial. Preventive medicine. 1998;27(2):296-303.

115.	Uyar M, Filiz A, Bayram N, Elbek O, Herken H, Topcu A, et al. A randomized trial of smoking cessation. Medication versus motivation. Saudi medical journal. 2007;28(6):922-6.

116.	Covey LS, Glassman AH, Jiang H, Fried J, Masmela J, LoDuca C, et al. A randomized trial of bupropion and/or nicotine gum as maintenance treatment for preventing smoking relapse. Addiction (Abingdon, England). 2007;102(8):1292-302.

117.	Tuisku A, Salmela M, Nieminen P, Toljamo T. Varenicline and Nicotine Patch Therapies in Young Adults Motivated to Quit Smoking: A Randomized, Placebo-controlled, Prospective Study. Basic & clinical pharmacology & toxicology. 2016;119(1):78-84.

118.	Xiao D, Kotler M, Kang J, Wang C. A Multicenter, Randomized, Double-blind, Parallel, Placebo-controlled Clinical Study to Evaluate the Efficacy and Safety of a Nicotine Mint Lozenge (2 and 4 mg) in Smoking Cessation. Journal of addiction medicine. 2020;14(1):69-77.

119.	Benowitz NL, Pipe A, West R, Hays JT, Tonstad S, McRae T, et al. Cardiovascular Safety of Varenicline, Bupropion, and Nicotine Patch in Smokers: A Randomized Clinical Trial. JAMA internal medicine. 2018;178(5):622-31.

120.	Ebbert JO, Dale LC, Patten CA, Croghan IT, Schroeder DR, Moyer TP, et al. Effect of high-dose nicotine patch therapy on tobacco withdrawal symptoms among smokeless tobacco users. Nicotine & tobacco research : official journal of the Society for Research on Nicotine and Tobacco. 2007;9(1):43-52.

121.	Tønnesen P, Paoletti P, Gustavsson G, Russell MA, Saracci R, Gulsvik A, et al. Higher dosage nicotine patches increase one-year smoking cessation rates: results from the European CEASE trial. Collaborative European Anti-Smoking Evaluation. European Respiratory Society. The European respiratory journal. 1999;13(2):238-46.

122.	Kavin H, Shivley S. Absence of effect of nicotine on rectal sensation, rectal compliance, and anal sphincter pressures in healthy subjects. Digestive diseases and sciences. 1995;40(10):2239-43.

123.	Mills EJ, Wu P, Lockhart I, Wilson K, Ebbert JO. Adverse events associated with nicotine replacement therapy (NRT) for smoking cessation. A systematic review and meta-analysis of one hundred and twenty studies involving 177,390 individuals. Tobacco induced diseases. 2010;8(1):8.

124.	Hariton E, Locascio JJ. Randomised controlled trials - the gold standard for effectiveness research: Study design: randomised controlled trials. BJOG : an international journal of obstetrics and gynaecology. 2018;125(13):1716.

125.	Working Group for the Study of Transdermal Nicotine in Patients With Coronary Artery Disease. Nicotine replacement therapy for patients with coronary artery disease. Working Group for the Study of Transdermal Nicotine in Patients with Coronary artery disease. Archives of internal medicine. 1994;154(9):989-95.
